# Towards a global DNA barcode reference library for quarantine identifications of lepidopteran stemborers, with an emphasis on sugarcane pests

**DOI:** 10.1038/s41598-019-42995-0

**Published:** 2019-05-07

**Authors:** Timothy R. C. Lee, Stacey J. Anderson, Lucy T. T. Tran-Nguyen, Nader Sallam, Bruno P. Le Ru, Desmond Conlong, Kevin Powell, Andrew Ward, Andrew Mitchell

**Affiliations:** 1 Department of Entomology, Australian Museum Research Institute, 1 William St, Darlinghurst, NSW 2010 Australia; 2 Biosecurity Operations, NAQS, Department of Agriculture and Water Resources, 1 Pederson Road, Eaton, NT 0812 Australia; 3 Northern Territory Department of Primary Industry and Resources, GPO Box 3000, Darwin, NT 0801 Australia; 4 Department of Agriculture and Water Resources, 114 Catalina Crescent, Airport Business Park, Cairns Airport, Cairns, QLD 4870 Australia; 5 African Insect Science for Food and Health (ICIPE), PO Box 30772-00100 Nairobi, Kenya; 6 IRD/CNRS, UMR IRD 247 EGCE, Laboratoire Evolution Génomes Comportement et Ecologie, Avenue de la terrasse, BP1, 91198, Gif-sur-Yvette, France and Université Paris-Sud 11, 91405 Orsay, France; 7 Department of Conservation Ecology and Entomology, Faculty of AgriSciences, University of Stellenbosch, Private Bag X1, Matieland, Western Cape 7602 South Africa; 8 South African Sugarcane Research Institute, 170 Flanders Drive, Mount Edgecombe, KwaZulu-Natal 4300 South Africa; 9 Sugar Research Australia, 71378 Bruce Highway, Gordonvale, QLD 4865 Australia; 10 Sugar Research Australia, 50 Meiers Road, Indooroopilly, QLD 4068 Australia

**Keywords:** Entomology, Biodiversity, Phylogenetics

## Abstract

Lepidopteran stemborers are among the most damaging agricultural pests worldwide, able to reduce crop yields by up to 40%. Sugarcane is the world’s most prolific crop, and several stemborer species from the families Noctuidae, Tortricidae, Crambidae and Pyralidae attack sugarcane. Australia is currently free of the most damaging stemborers, but biosecurity efforts are hampered by the difficulty in morphologically distinguishing stemborer species. Here we assess the utility of DNA barcoding in identifying stemborer pest species. We review the current state of the COI barcode sequence library for sugarcane stemborers, assembling a dataset of 1297 sequences from 64 species. Sequences were from specimens collected and identified in this study, downloaded from BOLD or requested from other authors. We performed species delimitation analyses to assess species diversity and the effectiveness of barcoding in this group. Seven species exhibited <0.03 K2P interspecific diversity, indicating that diagnostic barcoding will work well in most of the studied taxa. We identified 24 instances of identification errors in the online database, which has hampered unambiguous stemborer identification using barcodes. Instances of very high within-species diversity indicate that nuclear markers (e.g. 18S, 28S) and additional morphological data (genitalia dissection of all lineages) are needed to confirm species boundaries.

## Introduction

Stemborers are a polyphyletic group of moths from the families Noctuidae, Tortricidae, Crambidae and Pyralidae, the larvae of which bore into the stems of grasses and eat them from the inside. The grasses (Poaceae) comprise the world’s most economically important plant family^1^ including cereals and sugarcane. Cereals provide more than 50% of the world’s daily food calories^2^ and sugarcane is the world’s most prolific crop, with agricultural production by weight in 2016 78% higher than the next largest crop, maize^3^. Globally, annual crop losses due to pests amount to 20–40%^4^ and lepidopteran stemborers are the most significant pests of graminaceous crops; many are polyphagous, feeding on multiple crop species and alternative host plants. This makes stemborers among the most significant insect pests in the world and of major quarantine concern.

A thorough understanding of the diversity of pest species, and robust taxonomy and associated diagnostic tools, underpin biosecurity and the global quarantine measures protecting agriculture^5^. The last two decades have seen immense progress towards documenting the diversity of stemborers, particularly for the Apameini, Sesamiina (Noctuidae) of Africa^6–10^. However, stemborers of the Asian and Australasian regions and pyraloid stemborers globally, despite recent work on *Diatraea*^11^, remain poorly characterised and reliable resources for identifying the species remain few and narrow in scope. A major difficulty confronting early warning detection lies in distinguishing the minutia of species.

Australia has no significant exotic stemborer pest species present, likely due to its geographical isolation and employment of stringent quarantine^12^. The native Australian sugarcane stemborer, *Bathytricha truncata*^13^ (Lepidoptera: Noctuidae: Acronictinae), does not cause significant damage, which could be due to control by natural enemies^14^. Australia is the seventh largest sugarcane producer globally; the Australian industry generated 1.75 billion AUD in revenue in 2017, with ~83% of sugar produced for export^3,15^. Exotic stemborers could arrive in Australia due to Australia’s close proximity to Papua New Guinea and Indonesia, where significant stemborer pest species are present^14^. Introductions are made more likely by possible changes in stemborer range due to climate change^16^ and increased trade. Australian biosecurity agencies therefore require the capacity for rapid identification of exotic stemborers. The establishment of these pests in Australia could have a devastating effect on industry by reducing sugarcane yields up to 40%^17–19^. Sallam^14^ listed the 36 most significant sugarcane stemborer moth species ranked in terms of the threat posed to Australia, with seven species from two families (Crambidae and Noctuidae) regarded as ‘high threat’: *Chilo terrenellus*^20^, *Chilo infuscatellus*^21^, *Chilo sacchariphagus*^22^, *Chilo auricilius*^23^, *Scirpophaga excerptalis*^24^ (Crambidae), *Sesamia grisescens*^25^ and *Sesamia inferens*^13^ (Noctuidae).

To improve the ability of biosecurity agencies to detect stemborer incursions, and to circumvent the difficulties of morphological identification, (including the need for rearing juveniles to adulthood, which relies on live material and greatly slows the identification process), DNA barcoding could be used to establish species-level identity. DNA barcoding is the practice of sequencing a fragment of one mitochondrial gene from a large number of accurately identified specimens to form a database, and comparing sequences of that gene from unidentified specimens to this database^26,27^. DNA barcoding is an increasingly useful tool for identifying arthropod plant pests^28^ and, in particular, moths of quarantine concern^29^. Few studies of stemborers to date have used the barcode-standard region of the cytochrome *c* oxidase I (COI) gene, e.g. Lange *et al*.^30^ sequenced the COII and 16S genes of 24 species, while Barrera *et al*.^31^ analysed the COII gene in the genus *Diatraea*. Assefa *et al*.^32^ was the first study to use DNA barcoding to identify stemborers, specifically *Busseola* spp. larvae in Ethiopia. That study and subsequent barcoding studies of stemborers have been limited in scope or have not conformed to community standards for vouchering of specimens and the deposition of sequences and associated data on the Barcode of Life Data System website, (BOLD)^33^. In some cases, the species identifications associated with such sequences are demonstrably incorrect. These factors formed part of the motivation for the current study.

An assessment of species-level diversity in a sequence dataset where some individuals are unidentified can be performed using molecular species delimitation techniques. Some methods are based on genetic distances, such as Automated Barcode Gap Discovery (ABGD)^34^ and Refined Single Linkage Analysis (RESL)^35^. Other methods are tree-based, including the Generalized Mixed Yule Coalescent method (GMYC)^36,37^, the Bayesian Poisson Tree Process (bPTP)^38^, and the multi-rate Poisson Tree Process (mPTP)^39^. Applying multiple methods to the same dataset can provide a more reliable picture of species-level clustering^40^. This can assist in the identification of species which may be in need of taxonomic revision, and also instances where the COI barcode does not align with species boundaries, which can be due to introgression, incomplete lineage sorting or selective sweeps^41^. Species delimitation methods have been applied previously in stemborers, with the bPTP method having been shown to successfully delimit species in the genus *Acrapex*^42^. Examination of mean and extreme intra- and inter-specific genetic distance is also useful in investigating species boundaries^43–46^.

Due to the threat that stemborer incursions pose to agricultural crops, particularly for sugarcane in Australia, there is a need for both a comprehensive and well-curated database of barcode sequences and a reliable species delimitation method to identify intercepted specimens. In this study, we extend the work of Lange *et al*.^30^ by applying the universal COI barcode to this group.

This paper has four aims:

1. Assemble a new dataset of stemborer COI sequences to serve as the core of a verified reference DNA barcode dataset for biosecurity identifications, including all species listed by Sallam^14^ as posing a high risk to Australia, and as many of the medium and low risk species as possible.
2. Evaluate the accuracy of existing DNA barcode resources (BOLD) for stemborer species identification.
3. Survey the diversity of stemborer species affecting sugarcane and cereal crops, particularly those species of biosecurity concern, largely through matching barcodes from larval specimens reared from crops to those of adults identified robustly using morphological techniques.
4. Apply and evaluate different species delimitation methods (GMYC, mPTP, bPTP, ABGD, RESL), to determine accuracy in delimiting in accordance with current taxonomy, and also in accordance with one another.

## Results

### Dataset

The initial COI barcode dataset contained 508 sequences generated by us in this study, 73 sequences from the study of Chinese stemborers by Wang *et al*.^47^, including only those of their sequences without gaps and excluding their outgroup sequence, and 716 sequences downloaded from BOLD, for a total of 1297 sequences. The most sampled species was *Chilo orichalcociliellus*, with 142 sequences; nine species were represented by one sequence. We found 24 individuals in our dataset which, based on their position in the FastTree tree, were highly likely to have been misidentified (Supplementary Table 1). This reduced the total number of specimens correctly identified to species under current taxonomy to 1064, across 64 species. In the haplotypes dataset, there were 18 such misidentified sequences.

### Phylogenetic analysis for gene tree reconstruction

**Figure 1:**
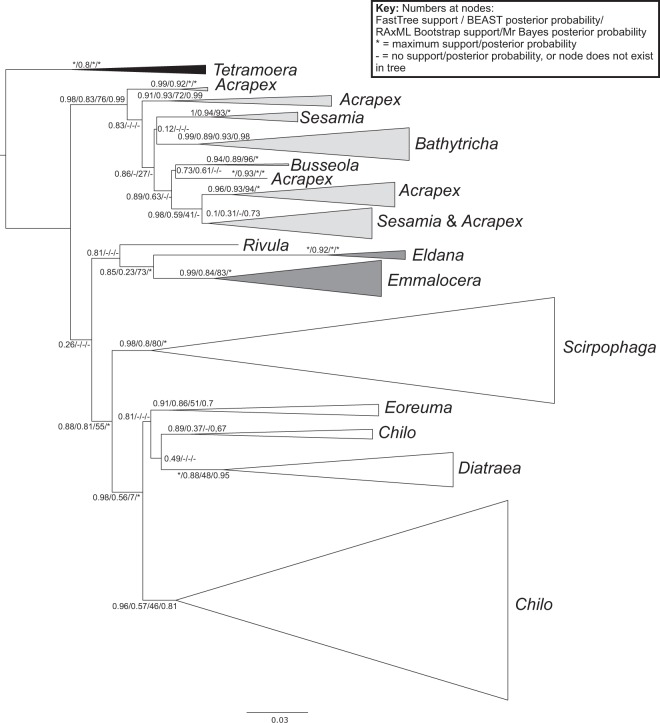
Phylogenetic tree estimated in FastTree from an alignment of 667 bp and 1297 sequences. Numbers at nodes indicate support values (in order), FastTree SH-like support, BEAST posterior probabilities, RAxML bootstrap values and MrBayes posterior probabilities. Asterisks represent maximum support; dashes represent no support. Shading on triangles indicates families: Black = Pyralidae, White = Tortricidae, Light grey = Noctuidae, Dark grey = Crambidae. Rivula is in the family Erebidae (not shaded on the diagram).

We estimated relationships among the haplotypes using FastTree, MrBayes, RAxML and BEAST. Trees were rooted using the Tortricidae as the outgroup, as the remaining families belong to the monophyletic Obtectomera^48^. Although the relationships among families and at deeper nodes within families were often poorly supported in all analyses, support values towards the tips were generally higher. Some genera were found to be paraphyletic in the analyses with high support, most notably *Acrapex* and *Sesamia*, which were divided into multiple clades in all haplotype dataset analyses (Fig. 1).

The majority of species were recovered as monophyletic*. Bathytricha truncata* was rendered paraphyletic through the insertion of a clade containing *B. monticola*, *B. leonina*, *B. phaeosticha* and *B. aethalion. Seasamia inferens* consisted of two separate clades within the *Sesamia* part of the tree. *Scirpophaga nivella* was found to be paraphyletic in all but the BEAST analysis, through the insertion of *Scirpophaga innotata*.

### Genetic distances and the barcoding gap

As genetic distance underpins some species delimitation analyses such as ABGD^34^ and RESL^35^, we investigated diversity in our haplotypes dataset by computed mean and maximal Kimura-2-Parameter genetic distances both within and between clades. Six comparisons between species had a minimum interspecific K2P distance of less than 0.03 (Table 1). Of the 49 non-singleton species, there were 27 with maximum within-species diversity above 0.02 (Table 2).

**Table 1:** Species pairs with minimum interspecific K2P distances between them of less than 0.03.

Species Pair	SpeciesIdentifier	MEGA7	
	Minimum K2P distance	Mean K2P Distance	Standard Error
*Bathytricha leonina* and *Bathytricha monticola*	0.0015	0.003	0.0017
*Bathytricha leonina* and *Bathytricha phaeosticha*	0.018	0.019	0.0056
*Bathytricha monticola* and *Bathytricha phaeosticha*	*0.020*	0.022	0.0059
*Chilo orichalcociliellus* and *Chilo thyrsis*	0.021	0.030	0.0064
*Scirpophaga innotata* and *Scirpophaga nivella*	0.022	0.042	0.0066
*Acrapex albivena* and *Acrapex minima*	0.028	0.029	0.0061

Number in italics indicates an instance where SpeciesIdentifier calculated no value, as *Bathytricha monticola* and *B. phaeosticha* are not nearest one another, as both are closer to *Bathytricha leonina*. This value was instead calculated in MEGA7.

**Table 2:** Species with maximum intraspecific K2P distances higher than 0.02.

Species	SpeciesIdentifier	MEGA7		Monophyletic on the FastTree tree?
	Maximum K2P distance	Mean K2P Distance	Standard Error	
*Sesamia inferens*	0.110	0.0523	0.0059	No
*Scirpophaga excerptalis*	0.107	0.0516	0.0054	Yes
*Bathytricha truncata*	0.098	0.0530	0.0061	No
*Chilo sacchariphagus*	0.081	0.0269	0.0037	Yes
*Emmalocera callirrhoda*	0.063	0.0394	0.0059	Yes
*Chilo crypsimetalla*	0.063	0.0158	0.0024	Yes
*Chilo infuscatellus*	0.062	0.0322	0.0041	Yes
*Eldana saccharina*	0.055	0.0235	0.0035	Yes
*Tetramoera gracilistra*	0.053	0.0319	0.0055	Yes
*Scirpophaga innotata*	0.053	0.0387	0.0069	Yes
*Chilo auricilius*	0.048	0.0224	0.0039	Yes
*Scirpophaga incertulas*	0.044	0.0201	0.0032	Yes
*Emmalocera latilimbella*	0.044	0.012	0.0022	Yes
*Eoreuma densella*	0.039	0.0203	0.0036	Yes
*Chilo orichalcociliellus*	0.038	0.0125	0.0024	Yes
*Chilo phragmitella*	0.034	0.0187	0.0036	Yes
*Diatraea saccharalis*	0.034	0.0166	0.0030	Yes
*Sesamia cretica*	0.033	0.0223	0.0052	Yes
*Eoreuma loftini*	0.031	0.0181	0.0037	Yes
*Sesamia grisescens*	0.030	0.0134	0.0029	Yes
*Diatraea grandiosella*	0.029	0.0287	0.0067	Yes
*Acrapex albicostata*	0.028	0.0157	0.0031	Yes
*Chilo quirimbellus*	0.027	0.0148	0.0037	Yes
*Chilo partellus*	0.026	0.0107	0.0024	Yes
*Scirpophaga nivella*	0.025	0.014	0.0029	No
*Chilo suppressalis*	0.025	0.0098	0.0021	Yes
*Acrapex exsanguis*	0.021	0.0101	0.0020	Yes

### Species delimitation

**Figure 2:**
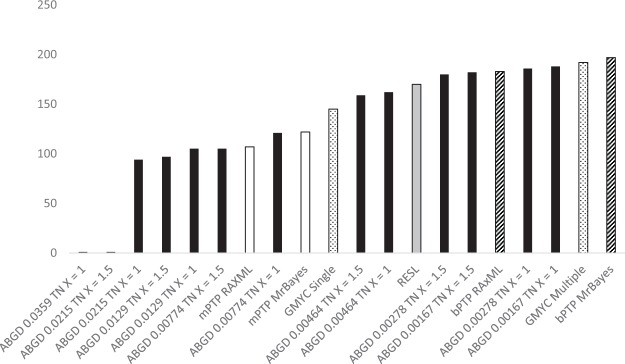
Number of taxa resulting from the species delimitation methods used in this study, based on the haplotypes dataset. Black = ABDG; Grey = RESL; White = mPTP; Spotted = GMYC; Striped = bPTP.

Species delimitation was performed on the haplotypes dataset and the genus-specific datasets using the GMYC, mPTP, bPTP, RESL and ABGD methods. Varying the relative gap width (X) or prior maximal intraspecific distance (PMID) values had a marked effect on the number of taxa estimated in the ABGD delimitations, ranging from 1 taxon (X = 1.5, PMID 0.0215 or X = 1, PMID = 0.0359) to 188 taxa (X = 1, PMID = 0.0017). The ABGD estimates most in line with the other delimitation methods’ estimates ranged from 94 taxa (X = 1, PMID = 0.0215) to 188 taxa (Fig. 2). The bPTP MrBayes method delimited the highest number of taxa, at 197. The GMYC single threshold method estimated 145 taxa, while the GMYC method with multiple thresholds delimited 192 species. The mPTP method delimited 107 and 122 species using the RAxML and MrBayes trees respectively, and the RESL method delimited 170 taxa.

**Figure 3:**
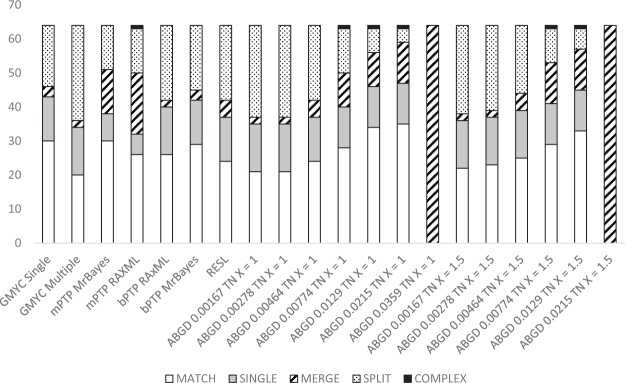
Composite bar graph showing the congruence of morphological identification with species delimitation method, for the haplotypes dataset.

The different delimitation methods applied had varying rates of success in matching current taxonomy (Fig. 3). Species were categorised as ‘matching’ (all individuals in one delimited group and no individuals identified as other species included), ‘merged’, (grouped with one or more other species), ‘split’ (two or more groupings containing the one identified species), or ‘complex’, (the species is split and at least one partition is merged with at least one other species), following Kekkonen *et al*.^49^, and we add a further category ‘single’, for taxa which are in the ‘match’ category but are represented by a single identified specimen in our dataset.

**Figure 4:**
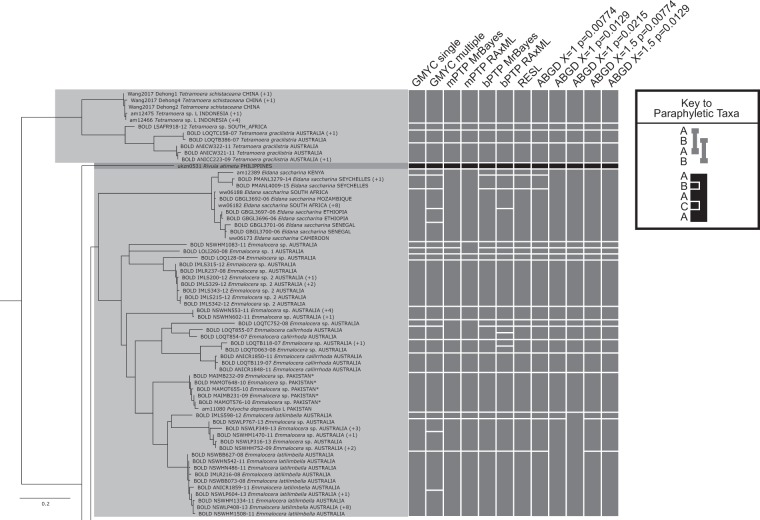
RAxML tree based on the haplotypes dataset (Part 1 of 5). Bars to the right of the tree indicate species delimitation groupings according to the 12 different species delimitation methods. The group of bars on the left are delimitations based on the whole haplotypes sequence dataset, bars on the right are based on the genus-specific analyses. Numbers in brackets indicate how many additional copies of this haplotype were present in the 1297 sequence dataset. Asterisks indicate sequences that we found to be misidentified, and which have now been corrected on BOLD (new species names appear in this figure). Names (or numbers in brackets) in bold indicate specimens where we performed genitalia dissections to confirm identity.

**Figure 5:**
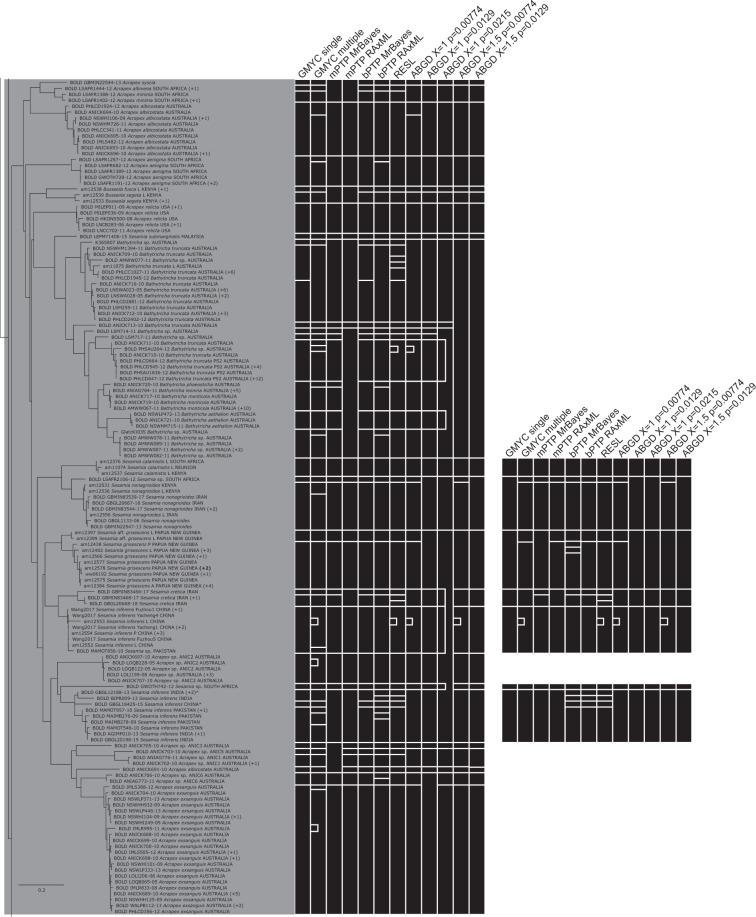
RAxML tree based on the haplotypes dataset (Part 2 of 5). Bars to the right of the tree indicate species delimitation groupings according to the 12 different species delimitation methods. The group of bars on the left are delimitations based on the whole haplotypes sequence dataset, bars on the right are based on the genus-specific analyses. Numbers in brackets indicate how many additional copies of this haplotype were present in the 1297 sequence dataset. Asterisks indicate sequences that we found to be misidentified, and which have now been corrected on BOLD (new species names appear in this figure). Names (or numbers in brackets) in bold indicate specimens where we performed genitalia dissections to confirm identity.

**Figure 6:**
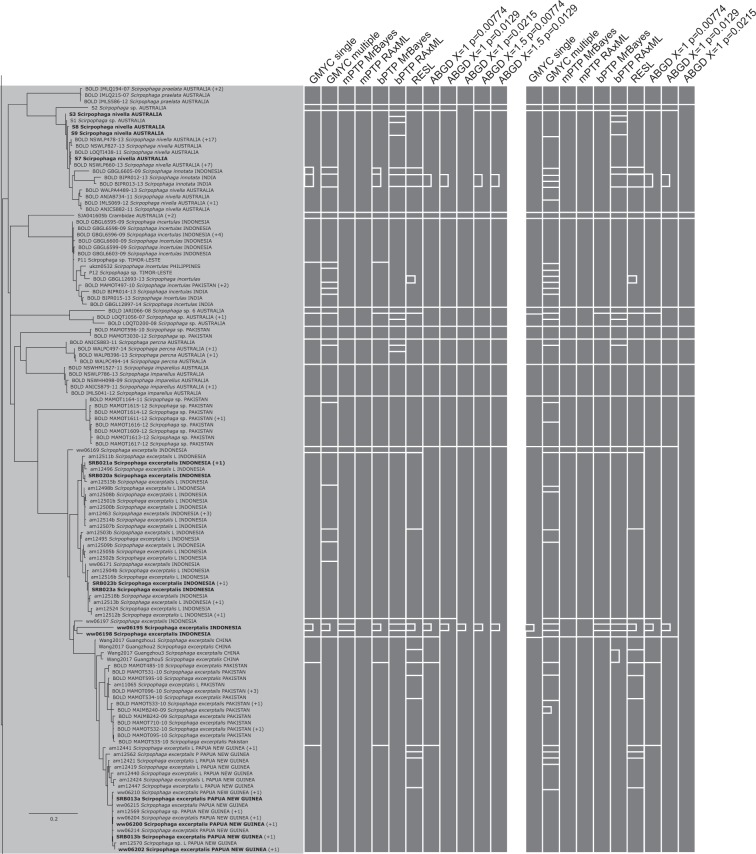
RAxML tree based on the haplotypes dataset (Part 3 of 5). Bars to the right of the tree indicate species delimitation groupings according to the 12 different species delimitation methods. The group of bars on the left are delimitations based on the whole haplotypes sequence dataset, bars on the right are based on the genus-specific analyses. Numbers in brackets indicate how many additional copies of this haplotype were present in the 1297 sequence dataset. Asterisks indicate sequences that we found to be misidentified, and which have now been corrected on BOLD (new species names appear in this figure). Names (or numbers in brackets) in bold indicate specimens where we performed genitalia dissections to confirm identity.

**Figure 7:**
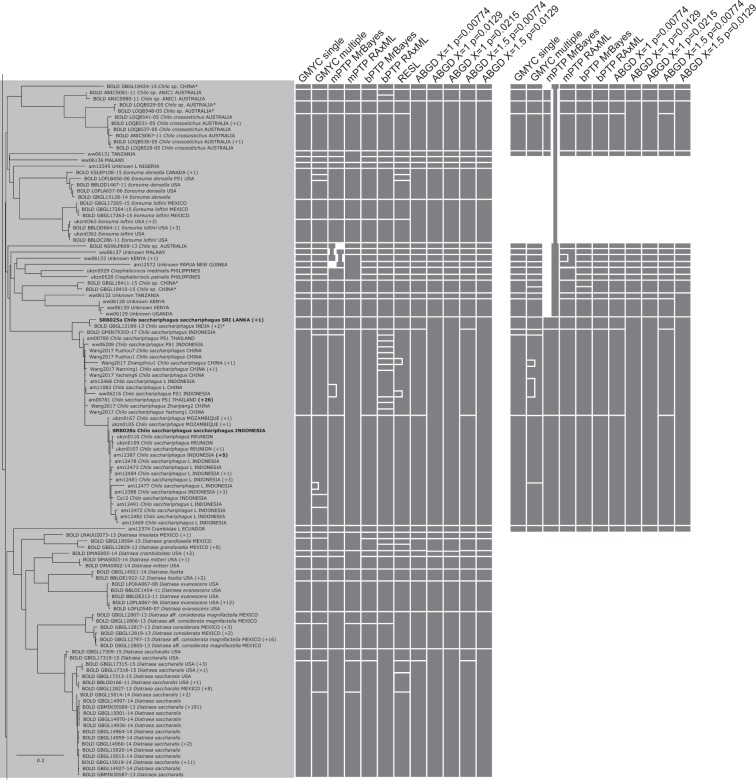
RAxML tree based on the haplotypes dataset (Part 4 of 5). Bars to the right of the tree indicate species delimitation groupings according to the 12 different species delimitation methods. The group of bars on the left are delimitations based on the whole haplotypes sequence dataset, bars on the right are based on the genus-specific analyses. Numbers in brackets indicate how many additional copies of this haplotype were present in the 1297 sequence dataset. Asterisks indicate sequences that we found to be misidentified, and which have now been corrected on BOLD (new species names appear in this figure). Names (or numbers in brackets) in bold indicate specimens where we performed genitalia dissections to confirm identity.

**Figure 8:**
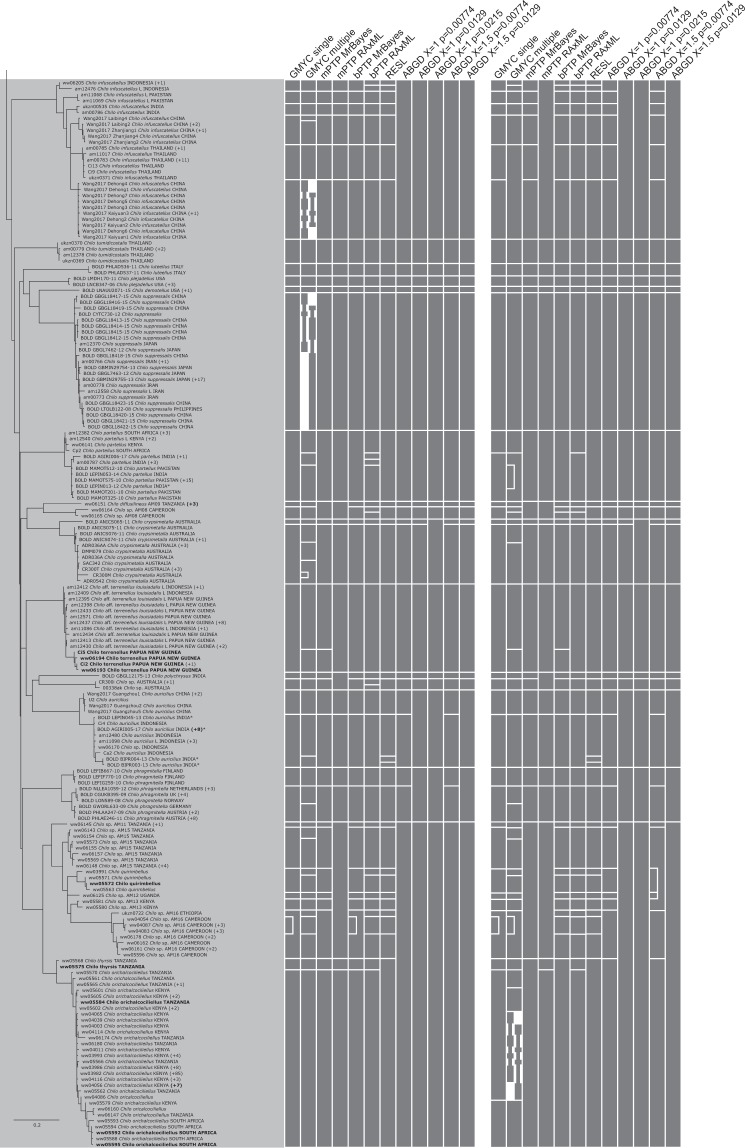
RAxML tree based on the haplotypes dataset (Part 5 of 5). Bars to the right of the tree indicate species delimitation groupings according to the 12 different species delimitation methods. The group of bars on the left are delimitations based on the whole haplotypes sequence dataset, bars on the right are based on the genus-specific analyses. Numbers in brackets indicate how many additional copies of this haplotype were present in the 1297 sequence dataset. Asterisks indicate sequences that we found to be misidentified, and which have now been corrected on BOLD (new species names appear in this figure). Names (or numbers in brackets) in bold indicate specimens where we performed genitalia dissections to confirm identity.

The multiple threshold GMYC method had the highest number of split taxa (28) of all methods (Fig 4–8). The GMYC single threshold method had the highest number of matches of the non-ABGD methods (30), while the ABGD methods exceeded this: PMID = 0.0129X = 1 (34) and X = 1.5 (33), and ABGD PMID = 0.0215X = 1 (35). The ABGD analyses were sensitive to changes in the PMID and X values, producing a range of delimitations ranging from entirely merged (ABGD PMID = 0.0359X = 1 and PMID = 0.0215X = 1.5, both delimiting one taxon across all specimens) to highly split (188 taxa).

Congruence among methods was high in many species. Based on the haplotypes dataset, in 29 out of the 64 species, at least 11 out of 12 methods agreed on whether the taxon was matching, single, split, merged or a complex (Table 3). Three of the seven high priority species were in this category: *Scirpophaga excerptalis* was split in all 12 delimitations, *Chilo terrenellus* matched in all 12 and *Sesamia inferens* was split in 11 out of 12 methods. Of the remaining four high priority species, *Sesamia grisescens* matched in 10 out of 12 methods, *Chilo sacchariphagus* and *Chilo auricilius* lent towards split (9 split/3 match), and *Chilo infuscatellus* was about even (7 split/5 match). Overall, the delimitations highlighted that diversity is likely to be underestimated among these high priority species, as most species either matched with current taxonomy or were split into multiple species. In the delimitations based on the *Chilo*, *Sesamia* and *Scirpophaga* subtrees, results were similar (Supplementary Table 1).

**Table 3:** Summary of agreement among the twelve species delimitation methods applied to the haplotypes dataset.

Genus	Species epithet	Match	Single	Merge	Split	Complex	High congruence
*Acrapex*	*aenigma*	10	0	0	2	0	
*Acrapex*	*albicostata*	10	0	0	2	0	
*Acrapex*	*albivena*	0	5	7	0	0	
*Acrapex*	*exsanguis*	10	0	0	2	0	
*Acrapex*	*minima*	5	0	7	0	0	
*Acrapex*	*relicta*	**12**	0	0	0	0	MATCH
*Acrapex*	*syscia*	0	10	2	0	0	
*Bathytricha*	*aethalion*	9	0	3	0	0	
*Bathytricha*	*leonina*	0	0	**12**	0	0	MERGE
*Bathytricha*	*monticola*	0	0	**12**	0	0	MERGE
*Bathytricha*	*phaeosticha*	0	3	9	0	0	
*Bathytricha*	*truncata*	0	0	2	9	1	
*Busseola*	*fusca*	0	10	2	0	0	
*Busseola*	*segeta*	10	0	2	0	0	
***Chilo***	***auricilius***	3	0	0	9	0	
*Chilo*	*crossostichus*	7	0	5	0	0	
*Chilo*	*crypsimetalla*	1	0	0	11	0	(Split)
*Chilo*	*demotellus*	0	**12**	0	0	0	SINGLE
*Chilo*	*diffusilineus*	0	**12**	0	0	0	SINGLE
***Chilo***	***infuscatellus***	5	0	0	7	0	
*Chilo*	*luteellus*	**12**	0	0	0	0	MATCH
*Chilo*	*orichalcociliellus*	6	0	6	0	0	
*Chilo*	*partellus*	10	0	0	2	0	
*Chilo*	*phragmitella*	3	0	0	9	0	
*Chilo*	*plejadellus*	**12**	0	0	0	0	MATCH
*Chilo*	*polychrysus*	0	**12**	0	0	0	SINGLE
*Chilo*	*quirimbellus*	3	0	6	3	0	
***Chilo***	***sacchariphagus***	3	0	0	9	0	
*Chilo*	*suppressalis*	11	0	0	1	0	(Match)
***Chilo***	***terrenellus***	**12**	0	0	0	0	MATCH
*Chilo*	*thyrsis*	6	0	6	0	0	
*Chilo*	*tumidicostalis*	**12**	0	0	0	0	MATCH
*Cnaphalocrocis*	*medinalis*	0	10	2	0	0	
*Cnaphalocrocis*	*patnalis*	0	10	2	0	0	
*Diatraea*	*considerata*	7	0	0	5	0	
*Diatraea*	*crambidoides*	0	11	1	0	0	(Single)
*Diatraea*	*evanescens*	**12**	0	0	0	0	MATCH
*Diatraea*	*grandiosella*	8	0	2	2	0	
*Diatraea*	*lineolata*	0	10	2	0	0	
*Diatraea*	*lisetta*	**12**	0	0	0	0	MATCH
*Diatraea*	*mitteri*	11	0	1	0	0	(Match)
*Diatraea*	*saccharalis*	5	0	0	7	0	
*Eldana*	*saccharina*	6	0	0	6	0	
*Emmalocera*	*callirrhoda*	0	0	0	**12**	0	SPLIT
*Emmalocera*	*latilimbella*	1	0	0	11	0	(Split)
*Eoreuma*	*densella*	10	0	0	2	0	
*Eoreuma*	*loftini*	5	0	0	7	0	
*Polyocha*	*depressellus*	0	**12**	0	0	0	SINGLE
*Rivula*	*atimeta*	0	**12**	0	0	0	SINGLE
***Scirpophaga***	***excerptalis***	0	0	0	**12**	0	SPLIT
*Scirpophaga*	*imparellus*	**12**	0	0	0	0	MATCH
*Scirpophaga*	*incertulas*	8	0	0	4	0	
*Scirpophaga*	*innotata*	0	0	3	5	4	
*Scirpophaga*	*nivella*	2	0	7	3	0	
*Scirpophaga*	*percna*	10	0	0	2	0	
*Scirpophaga*	*praelata*	**12**	0	0	0	0	MATCH
*Sesamia*	*calamistis*	**12**	0	0	0	0	MATCH
*Sesamia*	*cretica*	6	0	0	6	0	
***Sesamia***	***grisescens***	10	0	1	1	0	
***Sesamia***	***inferens***	0	0	0	11	1	(Split)
*Sesamia*	*nonagrioides*	11	0	0	1	0	(Match)
*Sesamia*	*submarginalis*	0	**12**	0	0	0	SINGLE
*Tetramoera*	*gracilistra*	0	0	0	**12**	0	SPLIT
*Tetramoera*	*schistaceana*	**12**	0	0	0	0	MATCH

MATCH = delimitation agrees with current taxonomy, MERGE = taxon groups with one or more other species, SPLIT = taxon split into multiple species, COMPLEX = taxon split and at least one partition merged with another species. High congruence lists the delimitation of taxa where all twelve delimitation methods agreed, or where all but one agreed (these appear in brackets). The seven species posing the highest level of threat to Australia according to Sallam^13^ are in bold and underlined; medium threat taxa are underlined but not in bold.

To test whether sampling bias influenced the number of species delimited in each taxon, we performed regression analyses on five of the whole haplotypes tree delimitations: GMYC single threshold, mPTP MrBayes, bPTP RAxML, RESL and ABGD 0.00774 TN X = 1. These five methods encompass the narrowest possible range of total number of species delimited (121–183, Fig. 2), while still including one delimitation from each method. In each case, a regression analysis was performed between the number of matching and split taxa delimited, and the number of specimens present of that species in the dataset. Singleton taxa were excluded to prevent biasing towards ‘matched’ taxa (as singletons cannot be split) and merged and complexed taxa were also excluded. Analyses were conducted using the Data Analysis package in Microsoft Excel. In all cases, there was a significant correlation between the number of taxa delimited and the number of samples included in the database (Table 4). However, r^2^ values in all cases were relatively low; although the value for RESL was high (0.75), this was due primarily to one outlier value (the large number of species delimited in *Scirpophaga excerptalis*), and without this species the  r^2^ value was 0.46.

**Table 4:** Regression analysis calculated on the relationship between number of taxa delimited and number of samples per species

Delimitation method	df	F	*p* value	r^2^	Mean no. of samples per species	Standard Error
GMYC single threshold	1,46	54.04	<0.05	0.54	9.60	1.58
bPTP RAxML	1,46	22.98	<0.05	0.33	9.60	1.58
mPTP MrBayes	1,41	47.88	<0.05	0.54	10.23	1.73
RESL	1,44	128.86	<0.05	0.75	9.35	1.59
ABGD 0.00774 TN X = 1	1,39	44.47	<0.05	0.53	9.83	1.76

## Discussion

High-threat species identified by Sallam^14^ were generally found to have high levels of intraspecific diversity. *Sesamia inferens* occurs in South, South-East and East Asia, New Guinea and the Solomon Islands, and is a pest of sugarcane and several cereals^50^. Although some studies have investigated its genetic diversity within parts of this range, particularly in China^47,51^, its overall genetic diversity is not well characterised. We found the species to be paraphyletic in all of the haplotypes dataset analyses, being split into two clades (Fig. 5). This species has the highest maximum intraspecific genetic distance in our dataset, at 11% K2P, and all delimitation methods applied split the taxon into at least two species, (e.g., RESL analysis split it into 6). This strongly indicate that our *S. inferens* specimens are actually from two different species. We include no sequences from the type locality (Sri Lanka^13^), but the clade from India and Pakistan is geographically closer to the type locality than the clade from China. It should be noted, however, that the India/Pakistan clade consisted only of sequences downloaded from BOLD, so we are unable to assess morphologically whether they might be a different species. More broadly, the Asian *Sesamia* includes 15 described species^52^, however its systematics is confused, and a revision combining morphological, ecological and molecular data is needed.

*Scirpophaga excerptalis* occurs throughout East, South-East and South Asia^53^. *S. excerptalis* formed a monophyletic clade in our haplotypes dataset analyses. *S. excerptalis* had a high maximum intraspecific divergence of 10.7% K2P, and was split into multiple species in all but one delimitation analysis. We identified ten of the *S. excerptalis* specimens in this study using genitalia dissections, including representatives from the three major clades. These results suggest either that *S. excerptalis* is a species complex, or that the mitochondrial gene tree does not match the species tree. Further work is required to test these possibilities.

*Chilo infuscatellus* occurs throughout Asia and parts of the Oceanian region^54^ and is the main pest of sugarcane in China^55^. In our dataset this species exhibited high intraspecific diversity, (maximum 6.2%). Species delimitation methods either matched current taxonomy (9 analyses) or split the taxon into at least six species (15 analyses). As we did not perform any genitalia dissections on our material for this species, we cannot discount the possibility that some of these specimens are misidentified.

*Chilo sacchariphagus* occurs in southern and south-eastern Asia, south-eastern Africa, Mauritius, Reunion and Madagascar^56^. Species delimitation analyses favoured splitting (18 analyses) over matching current taxonomy (6 analyses). *C. sacchariphagus* was divided into three groups in ten delimitation analyses, which our genitalia dissections indicate correspond to the three subspecies *C. sacchariphagus sacchariphagus*^22^, *C. sacchariphagus indicus*^57^ and *C. sacchariphagus stramineellus*^58^. These results have been confirmed by genitalia dissections for the first two subspecies. However, while *C. sacchariphagus stramineellus* can be differentiated by male genitalia, none of the dissected specimens of this subspecies have yielded COI sequences to date.

*Chilo auricilius* was recovered as monophyletic in all phylogenetic analyses. The distance to its closest non-conspecific neighbour, *Chilo orichalcociliellus*, is 6.93% K2P, which is sufficiently high to distinguish them when DNA barcoding. Species delimitation analysis favoured splitting (18 analyses) over matching current taxonomy (6 analyses).

Eight definitively identified *S. grisescens* sequences were included in the haplotypes dataset, all from Papua New Guinea. The closest distance from *S. grisescens* to its nearest congeneric, *S. inferens*, was 5.22% K2P distance. Twenty species delimitations matched current taxonomy, with one delimitation merging the species with *S. inferens* and three splitting it into multiple species. Two additional specimens (am12397 and am12399) clustered with *S. grisescens* in the tree, but as they were larval, without morphological identification and 2.94% divergent from the other specimens we considered them to be *Sesamia* aff. *grisescens*. Adult specimens would be useful in exploring whether these specimens are conspecific or not.

Four definitively identified *C. terrenellus* individuals occurred in our haplotypes dataset, all from Papua New Guinea. The closest distance from *C. terrenellus* to its nearest congeneric, *C. partellus*, was 7.91% K2P distance. All 24 species delimitation analyses matched the current taxonomy of *C. terrenellus*. Eleven sequences from Indonesian and Papua New Guinean specimens cluster very close to *C. terrenellus* and either represent this species or the morphologically similar species *C. louisiadalis*. Dissection of a larger series will be needed to confirm the identity of this clade.

Of the species of lesser biosecurity concern, 20 were also found to have maximum within-species divergences of more than 2% (Supplementary Material 1).

Some species were found to have low levels of inter-specific diversity. *Bathytricha* species are not well studied, with no taxonomic publications on the genus (other than a species checklist) since the species were described in the late 19^th^ and early 20^th^ century. Although a COI-only phylogeny is not definitive, high intraspecific divergence and paraphyly within *B. truncata* indicates it may represent at least two different species, while the high degree of similarity between *B. leonina* and *B. monticola* suggests that further investigation of the differentiation of these species is needed.

*Chilo thyrsis* is known only from Tanzania, while *Chilo orichalcociliellus* has a much broader distribution across south-east and central Africa^59^. In its original description, *C. thyrsis* was described as “Externally very similar to *Chilo argyrolepia*”^60^, and *C. argyrolepia* has been subsequently synonymized with *C. orichalcociliellus*^59^. *C. thyrsis* is merged with *C. orichalcociliellus* in 13 delimitations and is separate in 11, which does not indicate strong support for either the separation or merging of the taxa. As we only include two specimens of *C. thyrsis* in our dataset, we can only draw limited conclusions here, but the acknowledged close relationship between these two species may indicate they are recently diverged.

*Scirpophaga nivella* has a broad distribution across South and South-East Asia, southern and eastern China, Australia and the Western Pacific^53^. *Scirpophaga innotata* is known from Indonesia and the Philippines^61^, and also Malaysia and northern Australia^62^. In all but three of our phylogenetic analyses, *S. innotata* formed a clade inserted into *S. nivella*, rendering the latter paraphyletic. In the haplotypes BEAST tree, they formed sister clades, and in the FastTree and BEAST *Scirpophaga*-only analyses, *S. nivella* formed a clade inserted into *S. innotata*, rendering it paraphyletic. Fourteen of 24 delimitations grouped *S. nivella* and *S. innotata* as one species. A minimal K2P distance of 2.19% between the two species is lower than the level of intraspecific diversity we found in other species of the genus, like *S. excerptalis*.

*Acrapex minima* and *Acrapex albivena* are dealt with in Le Ru *et al*.^63^. In that study, a phylogenetic tree reconstructed based on four mitochondrial genes and two nuclear genes strongly differentiated the two species.

Given the high levels of intraspecific diversity found in several species in this study, delimitations matching current taxonomy may not be the most successful, but rather an underestimate of the true species number. A full assessment of the taxonomic status of these species will require nuclear and morphological data, as the COI barcode is comparatively short and susceptible to the skewed inheritance patterns resulting from *Wolbachia* infection^64,65^. Nevertheless, we can make some assessments of the relative merits of these methods as applied to this dataset. The ABGD method produced a broad range of delimitations depending on the prior maximal intraspecific distance (PMID) selected. Given that range, and our inability to independently assess which PMID is the most realistic, we exclude the ABGD method from the following comparisons. On this basis of matching current taxonomy, GMYC single threshold was the best method, with the highest combined number of matching and single taxa. If instead the criterion for successful delimitation is agreement with the consensus among the different methods we applied, the highest scoring method was again GMYC single threshold, with one disagreement with the consensus out of 64 taxa in the haplotypes dataset. The next best method was mPTP MrBayes, with two disagreements, then RESL with six. In the genus-level subtree analyses, the highest scoring methods are mPTP MrBayes and bPTP MrBayes, with two disagreements each out of 30 taxa, and GMYC single threshold and mPTP RAxML, with 4 disagreements each. Ultimately, congruence in delimitations across multiple methods remains the best method for assessing delimitation accuracy^40^, and we found this across all delimitations in several species (Table 3).

It is difficult to assess whether sampling was sufficient to delimit species accurately. When dealing with mitochondrial-only data, introgression and selective sweeps may make any amount of COI-only data insufficient to make an accurate assessment of species-level diversity. In the case of GMYC, Talavera *et al*.^66^ found that the most significant factor in sampling was capturing the extremities of each species’ diversity. Given the number of taxa included in our study with extreme within-species diversity above 5% (Table 2), we can be confident that at least for some species we have captured sufficient diversity. Future sampling efforts should be directed towards those species for which our sampling is poor, particularly *Sesamia grisescens* and *Chilo terrenellus*.

Our regression analyses indicate that there is a correlation between the number of individuals sampled with the number of taxa delimited in each species, although the r^2^ values in three out of four cases were under 0.6, and in the last case lowers to less than 0.6 when one outlier is removed. Such a correlation is not unexpected, given that no taxon can ever have more species delimited than it has samples. The low r^2^ values indicate that variables not in the model are having an effect on the relationship between number of sequences and number of taxa delimited; these other variables almost certainly include the actual absence or presence of cryptic diversity in these taxa.

Of the pairs of species with less than 3% minimum inter-specific diversity (Table 1), one species appears on the Sallam^14^ list: *Chilo orichalcociliellus* (Low threat, similar to *C. thyrsis*). Including only two *C. thyrsis* sequences in the dataset also does not allow us to properly explore the level of diversity in that group, and whether it is generally poorly differentiated from *Chilo orichalcociliellus*. In addition, our dataset lacks sequences from some species identified by Sallam^14^ as posing a low or medium threat to Australian sugarcane, which should be a high priority for future sequencing efforts. Apart from these caveats, the identity of all other stemborers of economic risk to Australia included in this study can be safely established through the use of the COI barcode.

When dealing with sequences downloaded from online public databases, one cannot verify the accuracy of specimen identifications (photographs, unless of genitalia dissections, are of little use in identifying stemborers). We found several instances of clear misidentification in our trees, where individuals identified as one species clustered closely with species in a different genus, or even a different family. These errors, which have now been corrected on BOLD (see Supplementary Table 2), are a cautionary tale for the uncritical use of public databases for quarantine identifications. Ideally, reference DNA barcode datasets should be established for quarantine pests and be validated through independent review processes to ensure the veracity of each specimen’s species identity. This is difficult in taxa such as *Chilo* which lack modern integrative revisionary taxonomic studies and associated identification resources. As for our own specimens, although we aimed to perform a genitalia dissection to confirm the identity of at least one individual from each cluster, we were unable to perform this in all clusters. We were also unable to verify the identity of juvenile specimens in most cases, although this study will help future researchers develop larval morphology keys through providing an improved barcode identification tool for juvenile specimens. This study has resolved instances of misidentification and indicated the possible need for taxonomic revision, both operational factors that must be resolved in robust barcoding systems^67^. Global analyses coupled with morphological taxonomic study are necessary, and incremental refinements to reference DNA barcode datasets should be performed as more data accumulates^68^.

The high levels of diversity that we find in this study, and the tendency in several cases for that diversity to be correlated with geography, indicate that barcoding could be used in this group to determine the source population of a specimen. This might be particularly important in situations where different populations require different biosecurity approaches. For example, *Eldana saccharina* populations in Africa are host to different parasitoid species, and are differentiated geographically and by their COI barcodes^69^, and whitefly (*Bemisia tabaci*) biotypes are known to have different pesticide resistance profiles^70^. Further work is required to determine whether divergent COI clusters in diverse species require different biosecurity responses.

Similar wide-ranging studies of North American and European Lepidoptera have tended to find considerably lower intraspecific diversity than we find here. In a study by Yang *et al*.^71^ on the North American Pyraustinae (Crambidae) including 1589 COI sequences from 103 species, maximum intraspecific distances (K2P) all were below 6%, and only three instances were found above 4%. Huemer *et al*.^72^ examined 1004 species (4925 sequences) of butterfly in Austria and Finland, finding the highest maximum intraspecific distance was 9.6% K2P, with only 3 instances above 8%; 12.3% of included species included more than 2% maximum intraspecific divergence. Hausmann *et al*.^73^ included 1395 sequences across 331 species of the Geometridae fauna of Bavaria, and found 9.2% maximum intraspecific divergence with seven species greater than 4%. In contrast, in our study of 1297 sequences across 64 species we find 27 species with maximum intraspecific K2P distances above 2%, 13 above 4%, and up to 11% in *S. inferens*. The unusually large intraspecific diversity in COI sequences observed for many species in this study needs to be resolved through the analysis of appropriate nuclear gene sequences and morphological work, to rigorously reassess species boundaries.

Overall, we find that COI DNA barcoding initiatives aimed at identifying stemborers of economic interest are likely to be successful. Four out of the seven species of greatest economic significance to Australia were found to have intraspecific distances >6%: *Chilo infuscatellus*, *C. sacchariphagus*, *Scirpophaga excerptalis* and *Sesamia inferens*. Species delimitation efforts in this large, unevenly sampled single-locus dataset were mixed, although several species exhibited congruent delimitations across methods. Non-monophyly within species was rare, encountered only three times, indicating that tree-based clustering may be a useful way to assign species identity to unknown individuals. Errors in identification found in online databases underline the importance of expertly identified voucher specimens and curation of sequence collections in establishing robust reference databases for accurate DNA barcode based identifications. This study is the first step in that direction for the lepidopteran stemborers of sugarcane and cereals.

## Methods

### Specimens

Specimens were collected by the authors or donated by colleagues from many countries. We attempted to sample as broadly as possible from cereal and sugarcane growing regions around the world, prioritising the “high risk” species of Sallam^14^. Two thirds of the specimens sequenced for this study were adults and one third were larvae. Adults are usually the only life stage reliably identified using current morphology techniques, but sampling adults usually precludes the collection of host plant information. In this case 68% of the adults sequenced for this study, including most of those from Africa, were collected from host plants as larvae and laboratory reared.

### Species identification

Where possible, adults and larvae were identified to species level in the field based on experience, morphological appearance and/or ecological and geographic distribution, and were gifted to us with this existing identification. After DNA sequencing and preliminary phylogenetic analysis (see below), adult specimens for which the morphological (field) identifications disagreed with the DNA barcode identifications were reassessed based on external morphological appearance. Our next step was to examine at least one specimen from each putative species or each DNA barcode cluster (whichever was the less inclusive group) and reassess its morphological identification (e.g. for *Scirpophaga excerptalis*, 11 specimens were examined). Genitalia dissections were conducted on adult specimens and compared with available images of type specimens (for certain *Chilo* species), original species descriptions, taxonomic revisions, and published resources for stemborer identification. The main literature referred to: Barrion^74^, Bleszynski^75^, Bojer^22^, Butani^76^, Common^77^, Chen *et al*.^78^, Dudgeon^23^, Holloway^79^, Kapur^57^, Lewvanich^80^, Maes^81^, Meijerman and Ulenberg^82^, Munroe and Solis^83^, Pagenstecher^20^, Polaszek^6^, Rao and Nagaraja^84^, Siddalingappa *et al*.^85^, Solis and Metz^11^, Snellen^21^, Swinhoe^86^, Tams and Bowden^87^, Walker^24^.

For sequences downloaded from BOLD, which includes data mined from GenBank, we used the species identification provided. However, samples which we had good reason to believe had been incorrectly identified were excluded from some analyses, as described below. We contacted BOLD about such samples and their identifications on the database were changed.

### DNA extraction

DNA was extracted from adult moths either from a single leg or from a whole abdomen if a genitalia dissection was required. For larvae, depending on the specimen size, either a proleg or a piece of abdominal integument and associated muscle, or in some cases the entire rear half of the specimen (up to 10–20 mg) was sampled. To avoid any cross contamination, dissection instruments and forceps were wiped with laboratory tissue, dipped in ethanol and flame-sterilized between samples. DNA was extracted using commercial silica-gel membrane-based kits, either Qiagen DNeasy (Qiagen, Chadstone, Australia) or Bioline Isolate II Genomic DNA Isolation Kit (Bioline, Eveleigh, Australia) following the manufacturers’ instructions, except for whole abdomen dissections we used two to three times the recommended volumes of tissue digestion buffer and proteinase-K, and stored the excess volume at −80 °C for potential later use.

### PCR amplification

PCR amplification used the protocols and primers described in Mitchell^88^, with some PCRs using the Folmer primers^89^. Samples were amplified using the primer pair AMbc0f1m and AMbc0r1m, and PCR products were visualised on a 1.5% agarose gel, stained with 1 drop of Biotium GelRed (Gene Target Solutions, Dural, Australia) per 50 mL of gel mix. Samples which did not show a band were re-amplified, using the primers M13F and AMbc0r2m, using 1 *μ*L of the PCR product from the first amplification as a template. If this re-amplification failed, then no further amplification attempts were made. PCR protocols for initial amplifications and re-amplifications were the same and used the following reaction mixture per well: 2.29 μL MilliQ water, 7.5 μL of 10% Trehalose solution, 1.5 μL 10x reaction buffer (no MgCl_2_), 0.75 μL MgCl_2_, 0.3 μL of dNTP mix, 0.3 μL forward and reverse primer at 5 μM each, 0.06 μL Platinum Taq and 2 μL template, (1 μL for reamplifications).

### Sequencing

Sequencing was carried out by Macrogen Inc. (Korea) and the Australian Genome Research Facility (Brisbane). Chromatograms were edited and consensus sequences generated using Geneious 10.2.2 (http://www.geneious.com)^90^.

### Barcode analysis

For this study we sequenced 508 specimens. Our target taxa included all genera containing species listed as sugarcane pests by Sallam^14^, i.e., *Tetramoera* Diakonoff, 1968 (Tortricidae), *Eldana* Walker, 1865 and *Emmalocera* Raganot, 1888 (Pyralidae), *Chilo* Zincken, 1817, *Diatraea* Guilding, 1828, *Eoreuma* (Ely, 1910) and *Scirpophaga* Treitschke, 1832 (Crambidae), and *Sesamia* Guenée, 1852 (Noctuidae). No samples or sequences could be obtained for *Acigona* Hübner (1825) (Erebidae) or *Maliarpha* Raganot (1888) (Pyralidae). The Australian native stemborer genus *Bathytricha* Turner, 1920 (Noctuidae) was included because *B. truncata*, despite being a minor pest, is the only native stemborer species recorded to infest cane in Australia^91^, and there is a need to distinguish it from exotic species. Similarly, *Acrapex* Hampson, 1894 (Noctuidae) was included as the Australian species currently placed in this genus appear closely related to Asian *Sesamia* species, while *Busseola* Thurau, 1904 (Noctuidae), *Rivula* Guenée, 1845 (Erebidae) and *Cnaphalocrocis* Lederer, 1863 (Crambidae) were added as they contain significant cereal pest species for which we had obtained specimens.

This dataset, including specimen collection data, sequences and sequence trace files, is available on the Barcode of Life Data System website (BOLD)^33^ as public project LSTEM (Lepidopteran Stemborers) (10.5883/DS-LSTEM) (Supplementary Material 2). We compiled a Supplementary Dataset on BOLD, consisting of all BOLD sequences for taxa identified as being congeneric with our sample of species. The BOLD public database sequences were downloaded on 11 October 2017.

We used a set of sequences from the study on Chinese sugarcane borers by Wang *et al*.^47^ supplied to us by the senior author. When the specimens we sequenced were added to those downloaded from BOLD and the sequences from Wang *et al*., this produced a final dataset of 1297 individuals, including representatives from all seven of the ‘high threat’ species and 11 of the 15 ‘medium threat’ species and two of the 14 ‘low threat’ species identified by Sallam^14^.

Sequences were aligned in Geneious using Multiple Alignment using Fast Fourier Transform (MAFFT)^92^. The resulting alignments were cropped to a length of 667 bp. Only sequences longer than 486 bp, the minimum barcode standard length^93^, were used, however exceptions were made to this rule for two *Chilo sacchariphagus* specimens: ww06216 and ukzn0269 (at 476 bp and 413 bp respectively). The first sequence was included because our preliminary analysis showed it to occupy a long branch and be of possible taxonomic interest, and the latter was included because it was the only *C. sacchariphagus* sequence in our dataset from the type locality, Mauritius. Of the 1297 individuals in this final dataset, 1089 were initially identified to species level (Supplementary Material 3).

GMYC^37,66,94^ and mPTP^39^, are known to encounter difficulties with datasets including identical sequences. As identical sequences are also often removed when performing delimitations to speed up the analysis (e.g., in bPTP^95^) we removed such duplicates using USEARCH 9.2.64^96^, removing the shortest of sequences with “maximum differences = 0”, “maximum substitutions = 0”, or “minimum match percentage identity = 100”. We then checked the resulting 623 sequence dataset in a Geneious distance matrix to determine whether any 100% identity sequences remained, and a further three sequences were removed after this. We verified that no species had been eliminated from the dataset through this procedure. The resulting 620 sequence dataset, henceforth the ‘haplotypes dataset’, was the main dataset used for species delimitation.

Preliminary analysis of the alignment was carried out using FastTree 2.1.5^97,98^ in Geneious, using default settings, to generate approximately maximum-likelihood trees. This analysis was the one used to identify likely misidentified sequences. Nucleotide substitution models were tested using PartitionFinder2^99^, using the greedy algorithm^100^ and the PhyML phylogeny estimator^101^, implemented on the CIPRES science gateway computing platform^102^. The best model was chosen based on the Bayesian Information Criterion. This was SYM + I + G for codon position 1, TRN + I + G for codon position 2 and GTR + G for codon position 3 in the full dataset, with a separate partition for each codon position.

In order to better investigate the effect of sample size and diversity on species delimitation, three further datasets were used: *Chilo* only, *Scirpophaga* only and *Sesamia* only subsets of the haplotypes tree. These datasets were formed by taking the smallest possible clades including all identified samples of those genera from the FastTree tree. This means that these datasets contain a mix of samples identified as being of that genus, and those that were not identified as being of that genus but grouped with them (i.e., putatively misidentified or unidentified sequences). A single BOLD sequence labelled as *Sesamia submarginalis* was excluded from the *Sesamia* analysis due to its deep divergence from other *Sesamia* samples. Model selection was also run on these datasets: for *Chilo*, this was TRN + G for codon position 1, F81 + G for codon position 2 and GTR + G for codon position 3; for *Scirpophaga* this was TRN + G for position 1, F81 for position 2 and TIM + I + G for position 3; for *Sesamia* this was TRN + I for position 1, F81 + I for position 2 and TIM for position 3.

Maximum-likelihood analyses were carried out using RAxML 8.2.10^103^ on the CIPRES science gateway. In each case, the tree was estimated using 100 random stating points, and levels of bootstrap support at nodes were calculated using a bootstrapping analysis with 1000 pseudoreplicates.

Bayesian analyses were carried out using MrBayes 3.2.6^104^ on the CIPRES science gateway. The analyses were terminated automatically when the standard deviation of split frequencies dropped below 0.01, so the number of generations was different in each analysis, (haplotypes dataset: 18,580,000, *Chilo*: 4,765,000, *Scirpophaga*: 2,495,000, *Sesamia*: 480,000). Samples were taken every 1000 steps, and the first 10% of samples were discarded as burnin. In each case two independent analyses were conducted, each consisting of one cold chain and seven heated chains.

BEAST analyses were carried out on the CIPRES portal, using the estimate for the rate of evolution in the insect COI gene from Papadopoulou *et al*.^105^ Analyses used an MCMC chain of 10,000,000 steps, with a burnin of 1000 steps.

Trees from the maximum-likelihood analyses and Bayesian analyses were visualized and Figures generated using FigTree 1.4.3 (http://tree.bio.ed.ac.uk/software/figtree/).

The haplotypes dataset alignment was exported to TaxonDNA/Species Identifier 1.8^106^, and all individuals with an aberrant position on the tree with species level identification, i.e., likely misidentifications, were removed prior to calculating the Kimura 2 parameter distance^107^ within and between species; 18 such sequences were found in the haplotypes dataset. Specimens not identified to species were also excluded from this analysis. Mean intraspecific and mean interspecific distances were calculated in the same dataset using MEGA7^108^, using K2P distances, uniform rates among sites, and the default 500 bootstrap replicates to calculate standard error.

### Species delimitation

Five different species delimitation methods were applied to each dataset to further investigate instances where barcode diversity was inconsistent with current taxonomy, and to help determine how many species there are among the unidentified specimens in the tree.

GMYC analysis, (single and multiple threshold), was carried out using the ‘splits’ package v 1.0-19^37,109^ in R v3.3.3^110^, using the BEAST trees as input.

ABGD was carried out using the online version of ABGD software^34^ (http://wwwabi.snv.jussieu.fr/public/abgd/abgdweb.html). Default settings were used, following the approach of Kekkonen and Hebert^111^, however distance matrices based on TrN distance calculated in MEGA7 were used as input, as the TrN model of evolution was more applicable to our dataset than JC or K2P, based on our Partitionfinder2 results. All analyses were run twice, using two different relative gap width (X) settings, X = 1.5 (the default) and X = 1. Only the recursive results were used as they allow for different gap thresholds among taxa^34^.

bPTP delimitation was carried out using the bPTP.py module v0.51^38^ in Python v2.7.14^112^, using both the MrBayes and RAxML trees in all cases.

mPTP delimitation was conducted using the mPTP webserver was used for this analysis (http://mptp.h-its.org/#/tree), using the MrBayes and RAxML trees as input. Trees that had any multifurcations first randomly resolved into 0-length bifurcating branches in Mesquite v3.5^113^.

RESL delimitation^35^ was carried out online at the BOLD website, using the default settings of the “cluster sequences” function.

## Supplementary information

Supplementary Materials

Supplementary Material 2

## Data Availability

DNA Sequences, raw sequence trace files and specimen collection data is available on BOLD as public project LSTEM. The 508 COI sequences produced in this study have been submitted to GenBank, accession numbers MK566231 – MK566738. The 508 sequence dataset is also available for direct download from BOLD using the 10.5883/DS-LSTEM. Full sequence alignment: Supplementary Material 3.

## References

[1] Vallée GC, Muñoz DS, Sankoff D (2016). Economic importance, taxonomic representation and scientific priority as drivers of genome sequencing projects. BMC Genomics.

[2] Awika, J. M. In *Advance*s in *ce*real *science: implications to food processing and health promotion ACS Symposium Series*. (eds Awika, J. M., Piironen, V. & Bean, S.) Ch. 1, 1–13 (American Chemical Society, 2011).

[3] FAO FAOSTAT. *Crop Statistics*, http://www.fao.org/faostat/en/#data/QC (2018).

[4] FAO. The future of food and agriculture - trends and challenges. (Rome, 2017).

[5] Boykin LM, Armstrong KF, Kubatko L, De Barro P (2012). Species delimitation and global biosecurity. Evolutionary bioinformatics online.

[6] Polaszek, A. *African cereal stemborers: economic importance, taxonomy, natural enemies and control*. (CAB International, 1998).

[7] Le Ru BP (2006). Geographic distribution and host plant ranges of East African noctuid stem borers. Annales de la Societe Entomologique de France (N.S.).

[8] Zilli, A., Varga, Z., Ronkay, G. & Ronkay, L. *Apameini I. A Taxonomic atlas of the Eurasian and North African Noctuoidea*. (Heterocera Press, 2009).

[9] Moyal P (2011). Morphological reinforcement, ancient introgressive hybridisation and species delimitation in African stem-borer species of the genus *Sesamia* Guenée (Lepidoptera: Noctuidae). Systematic Entomology.

[10] Kergoat GJ (2015). Integrative taxonomy reveals six new species related to the Mediterranean corn stalk borer *Sesamia nonagrioides* (Lefèbvre) (Lepidoptera, Noctuidae, Sesamiina). Zoological Journal of the Linnean Society.

[11] Solis M. Alma, Metz Mark (2016). An illustrated guide to the identification of the known species of Diatraea Guilding (Lepidoptera, Crambidae, Crambinae) based on genitalia. ZooKeys.

[12] Sallam, M. N. & Allsopp, P. G. *BSS249 Preparadness for a borer incursion*. *Chilo incursion management plan version 1*., http://www.planthealthaustralia.com.au/wp-content/uploads/2013/03/Chilo-species-CP-2002.pdf (2008).

[13] Walker, F. *Catalogue of Lepidoptera Heterocera List of the Specimens of Lepidopterous Insects in the Collection of the British**Museum*, Vol. Part 9 - Noctuidae (Edward Newman, 1856).

[14] Sallam MNS (2006). A review of sugarcane stem borers and their natural enemies in Asia and Indian Ocean Islands: an Australian perspective. Annales de la Societe Entomologique de France.

[15] Australia Sugar Milling Council. *Sugar Industry Summary Statistics*, https://asmc.com.au/industry-overview/fact-sheets/statistics/ (2018).

[16] Khadioli N (2014). Effect of temperature on the phenology of *Chilo partellus* (Swinhoe) (Lepidoptera, Crambidae); simulation and visualisation of the potential future distribution of *C. partellus* in Africa under warmer temperatures through the development of life-table parameters. Bulletin of entomological research.

[17] Vargas G, Gómez LA, Michaud JP (2015). Sugarcane stem borers of the Colombian Cauca River Valley: current pest status, biology and control. Florida Entomologist.

[18] Goebel F-R, Achadian E, Mcguire P (2014). The economic impact of sugarcane moth borers in Indonesia. Sugar Tech.

[19] White WH (2008). Re-evaluation of sugarcane borer (Lepidoptera: Crambidae) bioeconomics in Louisiana. Crop Protection.

[20] Pagenstecher A (1900). Die Lepidopteran fauna des Bismarck-Archipels II. Zoologica, Stuttgart.

[21] Snellen, P. C. T. Mededeelingen Van Het Proefstation Voor Suikerriet in West-Java. (1890).

[22] Böjer, W. *Report of the Committee on the ‘cane borer’*. (1856).

[23] Dudgeon GC (1905). Description of new species of moths from India and Burma. Journal of the Bombay Natural History Society.

[24] Walker, F. *List of the specimens of lepidopterous insects in the collection of the British**Museum. Part 27. Crambites and Tortricites*, (Edward Newman, 9 Devonshire St, Bishopsgate, 1863).

[25] Warren W (1911). Descriptions of some new Noctuidae in the Tring. Museum. Novitate Zoologicae.

[26] Hebert PDN, Cywinska A, Ball SL, deWaard JR (2003). Biological identifications through DNA barcodes. Proceedings of the Royal Society B: Biological Sciences.

[27] Mitchell A (2008). DNA barcoding demystified. Australian Journal of Entomology.

[28] Ashfaq M, Hebert PDN (2016). DNA barcodes for bio-surveillance: regulated and economically important arthropod plant pests. Genome.

[29] Mitchell A, Gopurenko D (2016). DNA barcoding the Heliothinae (Lepidoptera: Noctuidae) of Australia and utility of DNA barcodes for pest identiifcation in *Helicoverpa* and relatives. PLoS ONE.

[30] Lange CL, Scott KD, Graham GC, Sallam MN, Allsopp PG (2004). Sugarcane moth borers (Lepidoptera: Noctuidae and Pyraloidea): phylogenetics constructed using COII and 16S mitochondrial partial gene sequences. Bulletin of entomological research.

[31] Barrera GP (2017). Identification of *Diatraea* spp. (Lepidoptera: Crambidae) based on cytochrome oxidase II. PLOS ONE.

[32] Assefa Y, Mitchell A, Conlong DE, Moyal P (2007). DNA identification of *Busseola* (Lepidoptera: Noctuidae) larvae in Ethiopian sugarcane. African Entomology.

[33] RATNASINGHAM SUJEEVAN, HEBERT PAUL D. N. (2007). BARCODING: bold: The Barcode of Life Data System (http://www.barcodinglife.org). Molecular Ecology Notes.

[34] Puillandre N, Lambert A, Brouillet S, Achaz G (2012). ABGD, Automatic Barcode Gap Discovery for primary species delimitation. Molecular Ecology.

[35] Ratnasingham S, Hebert PDN (2013). A DNA-based registry for all animal species: The Barcode Index Number (BIN) system. PLOS ONE.

[36] Pons J (2006). Sequence-based species delimitation for the DNA taxonomy of undescribed insects. Systematic Biology.

[37] Fujisawa T, Barraclough TG (2013). Delimiting species using single-locus data and the Generalized Mixed Yule Coalescent approach: a revised method and evaluation on simulated data sets. Syst Biol.

[38] Zhang J, Kapli P, Pavlidis P, Stamatakis A (2013). A general species delimitation method with applications to phylogenetic placements. Bioinformatics.

[39] Kapli P (2017). Multi-rate Poisson tree process for single-locus species delimitation under maximum likelihood and Markov chain Monte Carlo. Bioinformatics.

[40] Dellicour S, Flot JF (2018). The hitchhiker’s guide to single-locus species delimitation. Mol Ecol Resour.

[41] Ballard JWO, Whitlock MC (2004). The incomplete natural history of mitochondria. Molecular Ecology.

[42] B. P. LeR (2014). Integrative taxonomy of *Acrapex* stem borers (Lepidoptera: Noctuidae: Apameini): combining morphology and Poissant Tree Process analyses. Invertebrate Systematics.

[43] Boehme P, Amendt J, Zehner R (2012). The use of COI barcodes for molecular identification of forensically important fly species in Germany. Parasitology research.

[44] Iftikhar, R., Ashfaq, M., Rasool, A. & Hebert, P. *DNA Barcode Analysis of Thrips (Thysanoptera) Diversity in Pakistan Reveals Cryptic Species Complexes*, Vol. 11 (2016). 10.1371/journal.pone.0146014 PMC4704811 26741134

[45] Lin X, Stur E, Ekrem T (2015). Exploring Genetic Divergence in a Species-Rich Insect Genus Using 2790 DNA Barcodes. PLOS ONE.

[46] Sun X, Bedos A, Deharveng L (2018). Unusually low genetic divergence at COI barcode locus between two species of intertidal Thalassaphorura (Collembola: Onychiuridae). PeerJ.

[47] Wang J-D (2018). DNA barcoding for identification of sugarcane borers in China. Neotropical Entomology.

[48] Mitter C, Davis DR, Cummings MP (2017). Phylogeny and evolution of Lepidoptera. Annual Review of Entomology.

[49] Kekkonen M, Mutanen M, Kaila L, Nieminen M, Hebert PDN (2015). Delineating species with DNA barcodes: A case of taxon dependant method performance in moths. PLOS ONE.

[50] Walker, K. *Asiatic pink stemborer (Sesamia inferens)*, PaDIL- http://www.padil.gov.au (2005).

[51] Tang X-T, Xu J, Sun M, Xie F-F, Du Y-Z (2014). First microsatellites from *Sesamia inferens* (Lepidoptera: Noctuidae). Annals of the Entomological Society of America.

[52] Poole, R. W. In *Lepidop*terum *C*atalo*gus (New Series) Fascicle 118* (ed. Eheppner, J. B.) 501–1013 (E. J. Brill/Flora & Fauna Publications, 1989).

[53] Chen F-Q, Wu C-S (2014). Taxonomic review of the subfamily Schoenobiinae (Lepidoptera: Pyraloidea: Crambidae) from China. Zoological Systematics.

[54] Anderson, S. & Tran-Nguyen, L. *Top Borer (Chilo infuscatellus)*, PaDIL- http://www.padil.gov.au (2012).

[55] Wang J, Wang W, Wang R, Zheng H, Gao S (2017). Molecular Detection of *Chilo infuscatellus*. Journal of Insect Science.

[56] Ganeshan, S. & Rajabalee, A. Parasitoids of the sugarcane spotted borer, *Chilo sacchariphagus* (Lepidoptera: Pyralidae), In Mauritius. *Proceedings of the South African Sugar Technologists Association* **71**, 87–90 (1997).

[57] Kapur AP (1950). The identity of some Crambinae associated with sugarcane in India and of certain species related to them (Lepidoptera: Pyralidae). Transactions of the Royal Entomological Society of London.

[58] Caradja A (1926). Noch einige Worte über ostasiatische Pyraliden und Microlepidopteren. Deutsche entomologische Zeitschrift “Iris”.

[59] De Prins, J. & De Prins, W. *Afromoths, online database of Afrotropical moth speices (Lepidoptera)*, www.afromoths.net (2018).

[60] Bleszynski S (1963). Studies on the Crambidae (Lepidoptera). Part 41. On some tropical Crambidae with descriptyions of new genera and species. Acta Zoologica Cracoviensia.

[61] Litsinger JA (2006). Rice white stemborer *Scirpophaga innotata* (Walker) in southern Mindanao, Philippines. II. Synchrony of planting and natural enemies. International Journal of Pest Management.

[62] Common, I. F. B. *Moths of Australia*. (Melbourne University Press, 1990).

[63] Le R B (2017). Molecular phylogenetics and definition of the *Acrapex minima* Janse group (Lepidoptera, Noctuidae, Apameini, Sesamiina) with the description of four new species from the Afrotropics. Annales de la Societe Entomologique de France (N.S.).

[64] Jiang W (2014). *Wolbachia* infection status and genetic structure in natural populations of *Polytremis nascens* (Lepidoptera: Hesperiidae). Infection, Genetics and Evolution.

[65] Stouthamer R, Breeuwer JAJ, Hurst GDD (1999). *Wolbachia pipientis*: microbial manipulator of arthropod reproduction. Annual Review of Microbiology.

[66] Talavera G, Dincă V, Vila R (2013). Factors affecting species delimtations with the GMYC model: insights from a butterfly survey. Methods in Ecology and Evolution.

[67] Mutanen M (2016). Species-Level Para- and Polyphyly in DNA Barcode Gene Trees: Strong Operational Bias in European Lepidoptera. Syst Biol.

[68] Boykin LM, Savill A, De Barro P (2017). Updated mtCOI reference dataset for the *Bemisia tabaci* species complex. F1000Research.

[69] Assefa Y, Mitchell A, Conlong DE (2006). Phylogeography of Eldana saccharina Walker (Lepidoptera: Pyralidae). Annales de la Societe Entomologique de France (N.S.).

[70] Naveen NC (2017). Insecticide resistance status in the whitefly, *Bemisia tabaci* genetic groups Asia-I, Asia-II-1 and Asia-II-7 on the Indian subcontinent. Scientific Reports.

[71] Yang Z, Landry J-F, Hebert PDN (2016). A DNA barcode library for North American Pyraustinae (Lepidoptera: Pyraloidea: Crambidae). PLOS ONE.

[72] Huemer P, Mutanen M, Sefc KM, Hebert PDN, Testing DNA (2014). barcode performance in 1000 species of European Lepidoptera: large geographic distances have small genetic impacts. PLOS ONE.

[73] Hausmann A, Haszprunar G, Hebert PDN (2011). DNA barcoding the Geometrid fauna of Bavaria (Lepidoptera): successes, surprises, and questions. PLOS ONE.

[74] Barrion AT, Catindig JLA, Litsinger JA (1990). Chilo auricilius Dudgeon (Lepidoptera: Pyralidae), the correct name for the dark-headed stem borer (SB) found in the Philippines. International Rice Research Newsletter.

[75] Bleszynski S (1970). A revision of the world species of *Chilo* Zincken (Lepidoptera: Pyralidae). Bulletin of the British Museum (Natural History), Entomology.

[76] Butani DK (1956). A key for the identification of sugarcane borers. Indian Journal of Entomology.

[77] Common IFB (1960). A revision of the Australian stem borers hitherto referred to *Schoenobius* and *Scirpophaga* (Lepidoptera: Pyralidae, Schoenobiinae). Australian Journal of Zoology.

[78] Chen F, Song S, Wu C (2006). A review of the genus *Scirpophaga* Treitschke, 1832 in China (Lepidoptera: Pyralidae). Zootaxa.

[79] Holloway, J. D. In *Afri*can cere*al* stem *borers: economic importance, taxonomy, natural enemies and control* (ed. Polaszek, A.) (CAB International, 1998).

[80] Lewvanich A (1981). A revision of the Old World species of *Scirpophaga* (Lepidoptera: Pyralidae). Bulletin of the British Museum (Natural History), Entomology.

[81] Maes, K. V. N. In *Afric*an c*er*eal stem *borers: economic importance, taxonomy, natural enemies and control*. (ed. Polaszek, A.) (CAB International, 1998).

[82] Meijerman L, Ulenberg SA (1996). Identification of African stemborer larvae (Lepidoptera: Noctuidae, Pyralidae) based on morphology. Bulletin of entomological research.

[83] Munroe, E. & Solis, M. A. In *Le*pi*dop*tera, *M*oths *and Butterflies, Vol. I. Arthropoda, Insecta, Vol. 4, Part 35. Handbook of Zo*olog*y*. (ed. Kristensen, N. P.) (Walter de Gruyter & Co., 1999).

[84] Rao VP, Nagaraja H (1966). A comparative study of the four species of paddy stem-borers belonging to the genera *Chilotraea* and *Chilo* in Asia (Lepidoptera: Pyralidae: Crambinae). Proceedings of the Indian Academy of Sciences - Section B.

[85] Siddalingappa CT, Hosamani V, Yalavar S (2010). Biology of maize stem borer Chilo partellus (Swinhoe) Crambidae: Lepidoptera. International Journal of Plant Protection.

[86] Swinhoe C (1884). On the Lepidoptera collected at Kurrachee. Proceedings of the Zoological Society of London.

[87] Tams WHT, Bowden J (1953). A revision of the African species of *Sesamia* Guenée and related genera (Agrotidae-Lepidoptera). Bulletin of entomological research.

[88] Mitchell A (2015). Collecting in collections: a PCR strategy and primer set for DNA barcoding of decades-old dried museum specimens. Molecular Ecology Resources.

[89] Folmer O, Black M, Hoeh W, Lutz R, Vrijenhoek R (1994). DNA primers for amplification of mitochondrial cytochrome c oxidase subunit I from diverse metazoan invertebrates. Molecular Marine Biology and Biotechnology.

[90] Kearse M (2012). Geneious Basic: an integrated and extendable desktop software platform for the organization and analysis of sequence data. Bioinformatics.

[91] Sallam, M. N., Allsopp, P. G., Chandler, K. J. & Samson, P. R. In *Pests of* Field Crops *and Pastures*. (ed. Bailey, P. T.) Ch. 11, 305–341 (CSIRO Publishing, 2007).

[92] Katoh K, Misawa K, Kuma KI, Miyata T (2002). MAFFT: a novel method for rapid multiple sequence alignment based on fast Fourier transform. Nucleic Acids Research.

[93] Hanner, R. *Proposed standards for BARCODE records in INSDC (BRIs)*, http://studentdnabarcoding.org/pdf/Barcode%20Data%20Standards.pdf (2009).

[94] Monaghan MT (2009). Accelerated species inventory on Madagascar using coalescent-based models of species delineation. Syst Biol.

[95] Malavasi V (2016). DNA-based taxonomy in ecologically versatile microalgae: a re-evaluation of the species concept within the coccoid green algal genus *Coccomyxa* (Trebouxiophyceae, Chlorophyta). PLOS ONE.

[96] Edgar RC (2010). Search and clustering orders of magnitude faster than BLAST. Bioinformatics.

[97] Price MN, Dehal PS, Arkin AP (2009). FastTree: Computing large minimum-evolution trees with profiles instead of a distance matrix. Molecular biology and evolution.

[98] Price MN, Dehal PS, Arkin AP (2010). FastTree 2 – Approximately maximum-likelihood trees for large alignments. PLOS ONE.

[99] Lanfear R, Frandsen PB, Wright AM, Senfeld T, Calcott B (2017). PartitionFinder 2: new methods for selecting partitioned models of evolution for molecular and morphological phylogenetic analysis. Molecular biology and evolution.

[100] Lanfear R, Calcott B, Ho SY, Guindon S (2012). PartitionFinder: combined selection of partitioning schemes and substitution models for phylogenetic analysis. Molecular biology and evolution.

[101] Guindon S (2010). New algorithms and methods to estimate maximum-likelihood phylogenies: assessing the performance of PhyML 3.0. Systematic Biology.

[102] Miller, M. A., Pfeiffer, W. & Schwartz, T. Creating the CIPRES Science Gateway for inference of large phylogenetic trees in *Gateway Computing Environments Workshop (GCE)*. 1–8 (IEEE).

[103] Stamatakis A (2014). RAxML version 8: a tool for phylogenetic analysis and post-analysis of large phylogenies. Bioinformatics.

[104] Ronquist F (2012). MrBayes 3.2: efficient Bayesian phylogenetic inference and model choice across a large model space. Syst Biol.

[105] Papadopoulou A, Anastasiou I, Vogler AP (2010). Revisiting the insect mitochondrial molecular clock: the mid-Aegean trench calibration. Molecular biology and evolution.

[106] Meier R, Shiyang K, Vaidya G, Ng PKL (2006). DNA barcoding and taxonomy in Diptera: a tale of high intraspecific variability and low identification success. Systematic Biology.

[107] Kimura M (1980). A simple method for estimating evolutionary rate of base substitutions through comparative studies of nucleotide sequences. Journal of Molecular Evolution.

[108] Kumar S, Stecher G, Tamura K (2016). MEGA7: Molecular Evolutionary Genetics Analysis version 7.0 for bigger datasets. Molecular biology and evolution.

[109] Ezard, T., Fujisawa, T. & Barraclough, T. G. Splits: species’ limits by threshold statistics. (R package, 2009).

[110] R Core Team. R: A language and environment for statistical computing (R Foundation for Statistical Computing, Vienna, Austria, 2017).

[111] Kekkonen M, Hebert PDN (2014). DNA barcode-based delineation of putative species: efficient start for taxonomic workflows. Molecular Ecology Resources.

[112] Python Software Foundation. *Python 2.7.14*, https://www.python.org/downloads/release/python-2714/ (2017).

[113] Maddison, W. P. & Maddison, D. R. Mesquite: a modular system for evolutionary analysis. (2018).

