# Supplementary material for: Towards a global DNA barcode reference library for quarantine identifications of lepidopteran stemborers, with an emphasis on sugarcane pests
Source: Sci Rep. 2019 May 7;9:7039. doi: 10.1038/s41598-019-42995-0 (PMC6504866; doi:10.1038/s41598-019-42995-0)
Supplement: Supplementary file 1 — Supplementary Materials [file 41598_2019_42995_MOESM1_ESM.pdf]

Towards a global DNA barcode reference library for quarantine identifications of lepidopteran stemborers, with an emphasis on sugarcane pests

Timothy R. C. Lee, Stacey J. Anderson, Lucy T. T. Tran-Nguyen, Nader Sallam, Bruno P. Le Ru, Desmond Conlong, Kevin Powell, Andrew Ward, Andrew Mitchell

Supplementary Material 1

Other highly diverse species

Bathytricha truncata: The genus *Bathytricha* appears to be in need of revision: there are deep divergences between two clades of *Bathytricha truncata* found in all of our tree reconstruction methods, no divergence between *B. monticola* and *B. leonina*, and one unidentified cluster. Nine delimitation methods split *B. truncata*, with two merging it with all other *Bathytricha* species and one complex delimitation.

Chilo crypsimetalla: This is the only *Chilo* species listed in the Checklist of the Lepidoptera of Australia[1] as *C. crossostichus* and *C. ochrileucalis* were synonymised with *C. crypsimetalla* in the checklist. There were 11 *C. crypsimetalla* in our dataset, and six *C. crossostichus*. All *C. crossostichus* were downloaded from BOLD, while four of the 11 *C. crypsimetalla* were downloaded from BOLD, with the other seven being identified and sequenced as part of this study. All the specimens from BOLD from both species were from adults and are vouchered with images, and we are confident they were identified correctly. The two taxa were monophyletic and strongly separated in our trees, therefore we consider *C. crypsimetalla* and *C. crossostichus* to be separate taxa. The 11 unique *C. crypsimetalla* haplotypes included in our study were all collected in the Northern Territory (NT) and Queensland (Australia). Despite the large distances among sites, spanning >1,700km, there is no clear geographical structuring among the samples. The two most genetically distant samples, at 6.25% K2P distance, were collected only ~40km apart in the NT. All but two species delimitation analyses split the taxon into more than one species, with the remaining two analyses matching current taxonomy. Further dissections from NT samples may help to establish whether *C. crypsimetalla* (*sensu stricto*) should be split into multiple species, and nuclear gene data and morphological studies will be required to properly characterise the relationship between *C. crypsimetalla* and *C. crossostichus*.

Chilo polychrysus: This species is a pest of rice, maize and occasionally sugarcane, distributed across much of Asia extending into Indonesia and Papua New Guinea. There are three BOLD sequences of *C. polychrysus* in our dataset, one from India (GBGL12175-13) and two from Queensland, Australia (ANICS080-11, ANICS081-11) and they form two unrelated groups in our analyses. Despite being recorded previously from rice in Australia[2] its presence in the country remains controversial[3] and it is still regarded as a "medium threat" quarantine risk to Australia[4]. Our analysis supports this latter view, that the Australian records likely refer to an unknown, perhaps undescribed species, since the

Indian and Australian sequences are split into two distant groups (12.7% K2P distance between them). In any case, given that our analysis only includes three individuals, none of which have satisfactory identifications based on genitalia comparison, we conclude that the dataset currently is not complete enough to allow for accurate barcoding identification of *C. polychrysus*, and the diversity of this species remains to be characterised.

Eldana saccharina: Eleven *E. saccharina* sequences are present in the haplotypes dataset, with individuals from Aldabra Atoll (Seychelles), South Africa, Mozambique, Kenya, Ethiopia, Senegal and Cameroon. The Aldabra Atoll records (from BOLD) are to the best of our knowledge the first record of this species away from the African mainland. Maximal intraspecific genetic distance was 5.5% K2P. Delimitation methods were split, with some agreeing with current taxonomy (6 methods) and others suggesting the taxon be split (6 methods). *Eldana saccharina* has previously been identified as consisting of three distinct populations, from Southern and Western Africa and from Ethiopia[5]. Although the COI sequences from that study used a different part of the gene and did not completely overlap with the barcode region, we ran a quick fast tree analysis (not presented) to determine the position of the Assefa *et al.* samples relative to those in this study. The same three clades were found, with the Kenyan sample included on a long branch in the Southern group, and a fourth clade, (sister to the others), consisted of the specimens from the Seychelles. One factor complicating COI delimitation in this species is that *E. saccharina* is known to be infected by *Wolbachia*, an endosymbiotic bacterium present in many arthropods[6]. *Wolbachia* can influence the reproductive success of its host, rendering it infertile when mating with non-infected individuals, influencing the sex ratio of offspring, and in some cases even feminising the host[7]. As *Wolbachia* are maternally inherited, these effects can change the pattern of inheritance of maternally inherited markers, such as COI, potentially causing divergence in the phylogenetic signal of nuclear and mitochondrial markers. Proper assessment of *Wolbachia* infected populations, therefore, requires the sequencing of nuclear markers in addition to mitochondrial ones to account for this effect.

Scirpophaga innotata: This species is a pest of rice found in tropical South-East Asia and Oceania[8] and also extending westwards into India, Pakistan and Iran[9,10]. Our study includes only three sequences from distant localities: Indonesia, China (Zhejiang province) and India (Orissa). Species delimitation methods were not in agreement on this taxon, with eight methods suggesting a merge with *Scirpophaga nivella*, eight suggesting it be split, and six suggesting a complex split with one clade merged with *S. nivella*.

Scirpophaga incertulas: Twelve identified specimens of this species were included in our haplotypes dataset, from the Philippines, Indonesia, India and Pakistan, but two further unidentified sequences clustered with them and were almost certainly also *S. incertulas*. This species was found to be monophyletic in all our analyses. Sixteen delimitation methods agreed with current taxonomy, while six suggested the taxon be split. With a minimum interspecific distance of 11% K2P, this species is likely to be easily separated from congenerics using the COI barcode.

Emmalocera/Polyocha species: *Polyocha depressellus* (*Emmalocera depressella* in much of the literature) is a pest of sugarcane on the Indian subcontinent. Our single sample of *P. depressellus* from Pakistan is a larva and therefore we cannot be certain of its identity. In our analyses its sequence grouped closely with five BOLD sequences, also from Pakistan material, however the BOLD sequences are identified as *Chilo infuscatellus*. The latter identification is clearly incorrect as *Emmalocera* and *Polyochra* sequences form a clade that is well separated from *Chilo* in all of our analyses. Two of the Australian species of *Emmalocera* included from BOLD data, *E. latilimbella* and *E. callirrhoda*, both show significant intraspecific diversity, but are easily distinguished from the *P. depressellus* sequence.

Tetramoera species: *Tetramoera schistaceana*, a species previously placed in *Eucosma* or *Argyroploce*, and known as the grey sugarcane stemborer in Asia, is another species represented only by larval samples in our dataset. However, it is easily distinguished from the only Australian species in the genus, *T. gracilistria*. Horak and Komai[11] found variation in genital morphology within *T. gracilistra* and our analysis similarly found two distinct clusters of this species, however with little geographical pattern to the variation.

The species described above in this section were all found to have maximum intraspecific divergence above 4%, but there were also several species with maximum divergence between 4% and 2%. Arranged alphabetically by genus, they were: *Acrapex albicostata*, *A. exsanguis*, *Chilo orichalcociliellus*, *C. partellus*, *C. phragmitella*, *C. quirimbellus*, *C. suppressalis*, *D. grandiosella*, *D. saccharalis*, *Eoreuma densella*, *E. loftini*, *Scirpophaga nivella* and *Sesamia cretica*. Some of these species appear on the Sallam[4] list: *Chilo partellus*, *D. saccharalis*, *Sesamia cretica* and *Eoreuma loftini*, (all medium threat), and *Chilo orichalcociliellus* (low threat).

Finally, although *Sesamia submarginalis* was not found to be highly diverse in this study, (as we included only one specimen), its position sister to *Bathytricha* and away from the rest of *Sesamia* is potentially indicative that it represents an undescribed genus. It was assigned to *Sesamia* by Holloway[12], although the Museum of Natural History, London, in its LepIndex lists it is assigned to *Busseola*[13]. We do not have reason to doubt the species identity of the one specimen in our dataset, (downloaded from BOLD), and we therefore persist in calling the species *Sesamia submarginalis*, while acknowledging that taxonomic revision of this species is needed.

References

1. Shaffer, M.; Nielsen, E. S. & Horak, M. (1996) Pyralidae. *In:* NIELSEN, E. S., EDWARDS, E. D. & RANGSI, T. V. (eds.) *Checklist of the Lepidoptera of Australia.* Collingwood, Vic, Australia: CSIRO Publishing.
2. Li, C. S. (1990) Status and control of *Chilo* spp., their distribution, host range and economic importance in Oceania. *Insect Science and its Application*, *11*(4-5), 535-539.
3. Sallam, M. N. & Allsopp, P. G. (2008) BSS249 Preparedness for borer incursion. *Chilo* incursion management plan version 1. http://www.planthealthaustralia.com.au/wp-content/uploads/2013/03/Chilo-species-CP-2002.pdf . Accessed 8 August 2018.
4. Sallam, M. N. S. (2006) A review of sugarcane stem borers and their natural enemies in Asia and Indian Ocean Islands: an Australian perspective. *Annales de la Societe Entomologique de France, 42*(3-4), 263-283.
5. Assefa, Y.; Mitchell, A. & Conlong, D. E. (2006) Phylogeography of *Eldana saccharina* Walker (Lepidoptera: Pyralidae). *Annales de la Société Entomologique de France (N.S.)*, *42*(3-4), 331-337.
6. Sweby, D. L.; Martin, L. A.; Govender, S.; Conlong, D. E. & Rutherford, R. S. (2010) The presence of *Wolbachia* in *Eldana saccharina* Walker (Lepidoptera: Pyralidae): implications for biological control. *Proceedings of the South African Sugar Technologists Association, 83*, 257-261.
7. Stouthamer, R.; Breeuwer, J. A. J. & Hurst, G. D. D. (1999) *Wolbachia pipientis*: microbial manipulator of arthropod reproduction. *Annual Review of Microbiology, 53*(1), 71-102.
8. Litsinger, J. A.; Alviola, A. L.; Dela Cruz, C. G.; Canapi, B. L.; Batay-An, E. H. & Barrion, A. T. (2006) Rice white stemborer *Scirpophaga innotata* (Walker) in southern Mindanao, Philippines. II. Synchrony of planting and natural enemies. *International Journal of Pest Management, 52*(1), 23-37.

9. Plantwise Knowledge Bank (2018) *Plantwise Technical Factsheet, White Rice Stem Borer (Scirpophaga innotata)* [Online]. Available: https://www.plantwise.org/knowledgebank/datasheet.aspx?dsid=55202 [Accessed 19th Feb 2018.
10. Leach, M. C. & Hobbs, S. L. A. (2013) Plantwise knowledge bank: delivering plant health information to developing country users. *Learned Publishing, 26*(3), 180-185.
11. Horak, M. & Komai, F. (2006) Australian Olethreutine genera: Tetramoera. *Olethreutine Moths of Australia.* Collingwood, Victoria, Australia: CSIRO Publishing.
12. Holloway, J. D. (1989) *The Moths of Borneo: Noctuidae, trifine subfamilies: Noctuinae, Heliothinae, Hadeninae, Acrocontinae, Amphipyrinae, Agaristinae,* Kuala Lumpur, Malaysia: Southdene Sdn. Bhd.
13. Beccaloni, G.; Scoble, M.; Kitching, I.; Simonsen, T.; Robinson, G.; Pitkin, B.; Hine, A. & Lyal, C. (2003) *The Global Lepidoptera Names Index (Lepindex)* [Online]. London: Natural History Museum. Available: http://www.nhm.ac.uk/our-science/data/lepindex/lepindex/ [Accessed 24th May 2018].

>AGIMP002-12|Chilo_sacchariphagus|AIN27002LKO|
AACTTTATATTTTATTTTTGGAATTTGAGCTGGAATAGTTGGAACATCACTTAGACTTTTAATTCGAGCTGAATTAGGAA
ATCCAGGTTCATTAATTGGAGATGATCAAATTTATAACACTATTGTTACAGCTCATGCATTTATTATAATTTTTTTTATA
GTAATACCAATTATAATTGGAGGATTTGGAAATTGATTAGTTCCATTAATATTAGGAGCTCCTGATATAGCTTTTCCCCG
TCTAAATAATATAAGATTTTGATTATTACCCCCCTCTTTAACCCTTTTAATTTCTAGAAGAATCGTTGAAAACGGAGCAG
GAACTGGATGAACAGTCTACCCCCCTTTATCTTCCAATATTTCACATGCTGGAAGTTCAGTAGATTTAGCCATCTTTTCC
CTCCATTTAGCTGGAATTTCTTCAATTTTAGGAGCTATTAATTTTATTACTACAATTATTAATATACGAATTAATGGACT
ATTATTTGATCAAATACCATTATTTGTTTGATCTGTTGGTATTACAGCATTACTTCTTCTCCTTTCTTTACCAGTATTAG
CAGGTGCTATTACTATATTACTAACTGATCGAAATTTAAATAC-------------------------------------
----
>AGIMP003-12|Sesamia_inferens|AIN27003LKO|
AACATTATATTTTATTTTTGGAATTTGAGCTGGTATAGTAGGAACATCATTAAGATTATTAATTCGAGCTGAATTAGGAA
CTCCTGGATCTTTAATTGGAGATGATCAAATTTATAATACTATTGTTACAGCTCATGCTTTTATTATAATTTTTTTTATA
GTTATACCAATTATAATTGGAGGATTTGGAAATTGACTTGTACCTTTAATATTAGGAGCTCCTGATATAGCTTTTCCACG
AATAAATAATATAAGATTTTGATTATTACCCCCCTCTTTAACTCTTTTAATTTCAAGCAGAATTGTAGAAAATGGAGCAG
GAACTGGGTGAACAGTGTACCCCCCACTTTCATCTAATATTGCCCATGGAGGAAGATCAGTAGATTTAGCTATTTTTTCC
CTTCATTTAGCTGGTATTTCATCTATTTTAGGAGCTATTAATTTTATTACAACAATTATCAATATACGATTAAATAGTTT
ATCTTTTGACCAAATACCTCTATTTATTTGAGCTGTTGGAATTACTGCATTTTTATTATTACTATCTTTACCTGTATTAG
CAGGGGCTATTACAATATTATTAACAGATCGAAATTTAAATAC-------------------------------------
----
>AGIMP005-12|Chilo_auricilius|AIN27005LKO|
AACTTTATACTTTATTTTTGGAATTTGAAGTGGAATAATTGGAACATCTCTAAGACTTTTAATTCGTGCTGAATTAGGAA
CTCCAGGGTCATTAATTGGAGATGATCAAATTTACAATACTATTGTTACAGCTCATGCATTTATTATAATTTTTTTTATA
GTTATACCAATTATAATTGGAGGCTTTGGTAATTGATTAGTACCATTAATACTAGGGGCTCCTGATATAGCCTTCCCTCG
AATAAATAATATAAGATTTTGATTATTGCCCCCATCATTAACATTATTAATTTCTAGAAGAATTGTAGAAAATGGAGCTG
GAACAGGATGAACGGTTTACCCCCCCCTTTCATCAAATATTGCCCATGGAGGAAGTTCTGTAGATTTAGCTATTTTTTCT
CTTCATTTAGCTGGTATTTCCTCAATTTTAGGAGCTATTAATTTTATTACAACAATTATTAATATACGAATTAATAAACT
ATCATTTGATCAATTACCATTATTTGTTTGATCTGTTGGTATTACAGCTTTATTATTACTACTTTCATTGCCGGTACTAG
CTGGAGCTATTACTATACTTTTAACTGATCGAAACTTAAATAC-------------------------------------
----
>AGIMP007-13|Chilo_partellus|AIN27003BLR|
AACTTTATATTTTATTTTTGGAATTTGAGCAGGAATAATTGGAACATCCCTTAGATTATTAATTCGTGCAGAATTAGGAA
CTCCTGGATCTTTAATTGGAGATGATCAAATTTATAATACTATTGTAACAGCACATGCATTTATTATAATTTTTTTTATA
GTTATACCAATTATAATTGGTGGATTTGGAAATTGATTAGTACCTTTAATATTGGGAGCCCCAGATATAGCTTTCCCACG
AATAAATAATATAAGATTTTGATTATTACCACCATCATTAACTTTACTAATTTCTAGAAGAATTGTTGAAAATGGAGCTG
GAACAGGATGAACAGTGTACCCCCCACTATCATCTAATATTGCTCATGCTGGAAGTTCAGTAGATTTAGCAATTTTTTCT
TTACATTTAGCTGGTATTTCATCAATTCTTGGTGCTATTAATTTTATTACAACCATCATTAATATACGAATTAATGGATT
ATTTTTTGATCAAATACCATTATTTGTTTGATCTGTAGGTATTACAGCTTTATTATTATTACTTTCTTTACCCGTTTTAG
CTGGGGCTATTACTATATTATTAACAGATCGAAATTTAAATACATCCTTTTTCGATCCTGATTGAGGGA-----------
----
>AGIRI006-17|Chilo_partellus|RO-NBAIR-6|
AACTTTATATTTTATTTTTGGAATTTGAGCAGGAATAATTGGAACATCCCTTAGATTATTAATTCGTGCAGAATTAGGAA
CTCCTGGATCTTTAATTGGAGATGATCAAATTTATAATACTATTGTAACAGCACATGCATTTATTATAATTTTTTTTATA
GTTATACCAATTATAATTGGTGGATTTGGAAATTGATTAGTACCTTTAATATTGGGAGCCCCAGATATAGCTTTCCCACG
AATAAATAATATAAGATTTTGATTATTACCACCATCATTAACTTTACTAATTTCTAGAAGAATTGTTGAAAATGGAGCTG
GAACAGGATGAACAGTGTACCCCCCACTATCATCTAATATTGCTCATGCTGGAAGTTCAGTAGATTTAGCAATTTTTTCT
TTACATTTAGCTGGTATTTCATCAATTCTTGGTGCTATTAATTTTATTACAACCATCATTAATATACGAATTAATGGATT
ATTTTTTGATCAAATACCATTATTTGTTTGATCTGTAGGTATTACAGCTTTATTATTATTACTTTCTTTACCCGTTTTAG
CTGGGGCTATTACTATATTATTAACAGATCGAAATTTAAATACATCCTTTTTCGATCCTGATTGAGGGA-----------
----
>AGIMP010-13|Sesamia_inferens|AIN27006BLR|
AACATTATATTTTATTTTTGGGATTTGAGCTGGTATAGTAGGAACATCATTAAGATTATTAATTCGAGCTGAATTAGGAA
CCCCAGGATCTTTAATTGGAGATGATCAAATTTATAATACTATTGTTACAGCTCATGCTTTTATTATAATTTTTTTTATA
GTTATACCAATTATAATTGGAGGATTTGGAAATTGACTTGTACCTTTAATATTAGGAGCTCCTGATATAGCATTTCCACG
AATAAATAATATAAGATTTTGATTATTACCCCCCTCTTTAACTCTTTTAATTTCAAGTAGAATTGTAGAAAATGGAGCAG
GAACTGGATGAACAGTGTACCCCCCACTTTCATCTAATATTGCTCATGGAGGAAGATCAGTAGATCTAGCTATTTTTTCT
CTTCATTTAGCTGGTATTTCATCTATTTTAGGAGCTATTAATTTTATTACAACAATTATCAATATACGACTAAATAGTTT

ATCTTTTGATCAAATACCTCTATTTATTTGAGCTGTTGGAATTACTGCATTTTTATTATTATTATCTTTACCTGTATTAG
CAGGAGCTATTACAATATTATTGACAGATCGAAATTTAAATACATCATTCTTTGACCCCGCAGGAGGGGGTG--------
----
>AGIRI009-17|Sesamia_inferens|RO-NBAIR-9|
AACATTATATTTTATTTTTGGGATTTGAGCTGGTATAGTAGGAACATCATTAAGATTATTAATTCGAGCTGAATTAGGAA
CCCCAGGATCTTTAATTGGAGATGATCAAATTTATAATACTATTGTTACAGCTCATGCTTTTATTATAATTTTTTTTATA
GTTATACCAATTATAATTGGAGGATTTGGAAATTGACTTGTACCTTTAATATTAGGAGCTCCTGATATAGCATTTCCACG
AATAAATAATATAAGATTTTGATTATTACCCCCCTCTTTAACTCTTTTAATTTCAAGTAGAATTGTAGAAAATGGAGCAG
GAACTGGATGAACAGTGTACCCCCCACTTTCATCTAATATTGCTCATGGAGGAAGATCAGTAGATCTAGCTATTTTTTCT
CTTCATTTAGCTGGTATTTCATCTATTTTAGGAGCTATTAATTTTATTACAACAATTATCAATATACGACTAAATAGTTT
ATCTTTTGATCAAATACCTCTATTTATTTGAGCTGTTGGAATTACTGCATTTTTATTATTATTATCTTTACCTGTATTAG
CAGGAGCTATTACAATATTATTGACAGATCGAAATTTAAATACATCATTCTTTGACCCCGCAGGAGGGGGTG--------
----
>AGIRI002-17|Chilo_sacchariphagus|RO-NBAIR-2|
AACTTTATATTTTATTTTTGGAATTTGAGCTGGAATAGTTGGAACATCACTTAGACTTTTAATTCGAGCTGAATTAGGAA
ATCCAGGTTCATTAATTGGAGATGATCAAATTTATAACACTATTGTTACAGCTCATGCATTTATTATAATTTTTTTTATA
GTAATACCAATTATAATTGGAGGATTTGGAAATTGATTAGTTCCATTAATATTAGGAGCTCCTGATATAGCTTTTCCCCG
TCTAAATAATATAAGATTTTGATTATTACCCCCCTCTTTAACCCTTTTAATTTCTAGAAGAATCGTTGAAAACGGAGCAG
GAACTGGATGAACAGTCTACCCCCCCTTTATCTTCCAATATTTCACATGCTGGAAGTTCAGTAGATTTAGCCATCTTTTCC
CTCCATTTAGCTGGAATTTCTTCAATTTTAGGAGCTATTAATTTTATTACTACAATTATTAATATACGAATTAATGGACT
ATTATTTGATCAAATACCATTATTTGTTTGATCTGTTGGTATTACAGCATTACTTCTTCTCCTTTCTTTACCAGTATTAG
CAGGTGCTATTACTATATTACTAACTGATCGAAATTTAAATAC-------------------------------------
----
>AGIRI003-17|Sesamia_inferens|RO-NBAIR-3|
AACATTATATTTTATTTTTGGAATTTGAGCTGGTATAGTAGGAACATCATTAAGATTATTAATTCGAGCTGAATTAGGAA
CTCCTGGATCTTTAATTGGAGATGATCAAATTTATAATACTATTGTTACAGCTCATGCTTTTATTATAATTTTTTTTATA
GTTATACCAATTATAATTGGAGGATTTGGAAATTGACTTGTACCTTTAATATTAGGAGCTCCTGATATAGCTTTTCCACG
AATAAATAATATAAGATTTTGATTATTACCCCCCTCTTTAACTCTTTTAATTTCAAGCAGAATTGTAGAAAATGGAGCAG
GAACTGGGTGAACAGTGTACCCCCCACTTTCATCTAATATTGCCCATGGAGGAAGATCAGTAGATTTAGCTATTTTTTCC
CTTCATTTAGCTGGTATTTCATCTATTTTAGGAGCTATTAATTTTATTACAACAATTATCAATATACGATTAAATAGTTT
ATCTTTTGACCAAATACCTCTATTTATTTGAGCTGTTGGAATTACTGCATTTTTATTATTACTATCTTTACCTGTATTAG
CAGGGGCTATTACAATATTATTAACAGATCGAAATTTAAATAC-------------------------------------
----
>AGIRI005-17|Chilo_auricilius|RO-NBAIR-5|
AACTTTATACTTTATTTTTGGAATTTGAAGTGGAATAATTGGAACATCTCTAAGACTTTTAATTCGTGCTGAATTAGGAA
CTCCAGGGTCATTAATTGGAGATGATCAAATTTACAATACTATTGTTACAGCTCATGCATTTATTATAATTTTTTTTATA
GTTATACCAATTATAATTGGAGGCTTTGGTAATTGATTAGTACCATTAATACTAGGGGCTCCTGATATAGCCTTCCCTCG
AATAAATAATATAAGATTTTGATTATTGCCCCCATCATTAACATTATTAATTTCTAGAAGAATTGTAGAAAATGGAGCTG
GAACAGGATGAACGGTTTACCCCCCCCTTTCATCAAATATTGCCCATGGAGGAAGTTCTGTAGATTTAGCTATTTTTTCT
CTTCATTTAGCTGGTATTTCCTCAATTTTAGGAGCTATTAATTTTATTACAACAATTATTAATATACGAATTAATAAACT
ATCATTTGATCAATTACCATTATTTGTTTGATCTGTTGGTATTACAGCTTTATTATTACTACTTTCATTGCCGGTACTAG
CTGGAGCTATTACTATACTTTTAACTGATCGAAACTTAAATAC-------------------------------------
----
>AMWW067-11|Bathytricha_monticola|K290807|
AACATTATATTTTATTTTTGGAATTTGAGCAGGAATAGTAGGAACCTCTTTAAGACTATTAATTCGAGCTGAATTAGGAA
CACCTGGATCTTTAATTGGAGATGATCAAATTTATAATACTATTGTAACAGCTCATGCTTTTATTATAATTTTTTTTATA
GTTATACCAATTATAATTGGAGGGTTTGGAAATTGACTTGTACCTTTAATATTAGGAGCACCTGATATAGCATTCCCACG
AATAAATAATATAAGTTTTTGATTACTTCCACCCTCTTTAACTCTCCTTATTTCAAGTAGAATTGTAGAAAATGGGGCAG
GAACTGGATGAACAGTTTACCCACCACTCTCATCTAATATTGCTCATGGAGGAAGATCTGTAGACTTAGCTATTTTTTCT
CTCCATTTGGCGGGAATCTCTTCTATTCTAGGAGCTATTAATTTTATTACAACTATTATTAATATACGATTAAATAGTTT
ATCTTTTGATCAAATACCTTTATTTATTTGAGCCGTAGGTATTACAGCATTTTTATTATTATTATCATTACCTGTATTAG
CTGGAGCTATTACTATACTATTAACAGATCGAAATTTAAATACATCATTTTTTGATCCTGCAGGAGGAGGAGATCCAATT
TTATATCAACATTTATTT---------
>AMWW077-11|Bathytricha|K290817|
AACATTATATTTCATCTTTGGAATTTGAGCAGGAATAGTAGGAACTTCTTTAAGACTATTAATTCGAGCTGAATTAGGAA
CTCCTGGATCCTTAATTGGAGATGATCAAATTTATAACACTATTGTAACAGCTCATGCCTTTATTATAATTTTTTTTATG
GTCATACCAATTATAATTGGAGGATTTGGAAATTGACTTGTACCTTTAATGTTAGGAGCACCTGATATAGCATTTCCACG

AATAAATAATATAAGTTTTTGATTACTACCCCCTTCTTTAACTCTACTTATTTCGAGAAGAGTCGTAGAAAATGGGGCAG
GAACTGGGTGAACAGTATATCCCCCACTTTCATCTAATATTGCCCATAGAGGAAGATCTGTAGATTTAGCTATTTTTTCC
CTTCATTTAGCTGGAATTTCTTCTATTCTAGGGGCTATTAATTTTATTACAACTATTATTAACATACGATTAAATAATTT
ATCTTTTGATCAAATACCTTTATTTATTTGAGCTGTAGGAATTACAGCATTTTTATTATTATTATCACTACCTGTATTAG
CTGGAGCTATTACTATGTTATTAACAGATCGAAATTTAAATACATCATTTTTTGACCCTGCGGGAGGAGGAGATCCAATC
TTATATCAACATTTATTT---------
>AMWW078-11|Bathytricha|K290818|
AACATTATATTTTATTTTTGGAATTTGAGCAGGAATAGTGGGAACCTCTTTAAGACTACTAATTCGTGCTGAATTAGGAA
CTCCCGGATCTCTAATTGGAGATGATCAAATTTATAATACTATTGTAACAGCTCATGCTTTTATTATAATTTTTTTCATA
GTTATACCAATCATAATTGGAGGATTTGGAAATTGACTTGTACCTTTAATATTAGGAGCACCTGATATAGCATTCCCACG
AATAAATAACATAAGTTTTTGATTACTTCCACCTTCTTTAACTCTTCTCATTTCAAGTAGAATTGTAGAAAATGGAGCAG
GAACTGGATGAACAGTTTACCCACCACTCTCATCTAATATTGCTCATAGTGGAAGATCCGTAGACTTAGCTATTTTTTCC
CTCCATTTAGCAGGAATCTCTTCCATCCTAGGAGCTATTAATTTTATTACAACTATTATTAATATACGATTAAATAGCTT
ATCTTTTGATCAAATACCTTTATTTATTTGAGCTGTAGGAATTACAGCATTTTTATTATTATTATCATTACCTGTATTAG
CTGGAGCTATTACTATATTACTAACAGATCGAAATTTAAATACATCATTTTTCGATCCCGCAGGAGGAGGGGATCCAATT
TTATATCAACATTTATTT---------
>AMWW081-11|Bathytricha_monticola|K290821|
AACATTATATTTTATTTTTGGAATTTGAGCAGGAATAGTAGGAACCTCTTTAAGACTATTAATTCGAGCTGAATTAGGAA
CACCTGGATCTTTAATTGGAGATGATCAAATTTATAATACTATTGTAACAGCTCATGCTTTTATTATAATTTTTTTTATA
GTTATACCAATTATAATTGGAGGGTTTGGAAATTGACTTGTACCTTTAATATTAGGAGCACCTGATATAGCATTCCCACG
AATAAATAATATAAGTTTTTGATTACTTCCACCCTCTTTAACTCTCCTTATTTCAAGTAGAATTGTAGAAAATGGGGCAG
GAACTGGATGAACAGTTTACCCACCACTCTCATCTAATATTGCTCATGGAGGAAGATCTGTAGACTTAGCTATTTTTTCT
CTCCATTTGGCGGGAATCTCTTCTATTCTAGGAGCTATTAATTTTATTACAACTATTATTAATATACGATTAAATAGTTT
ATCTTTTGATCAAATACCTTTATTTATTTGAGCCGTAGGTATTACAGCATTTTTATTATTATTATCATTACCTGTATTAG
CTGGAGCTATTACTATACTATTAACAGATCGAAATTTAAATACATCATTTTTTGATCCTGCAGGAGGAGGAGATCCAATT
TTATATCAACATTTATTT---------
>AMWW082-11|Bathytricha|K290822|
AACATTATATTTTATTTTTGGAATTTGAGCAGGAATAGTGGGAACCTCTTTAAGACTACTAATTCGCGCTGAATTAGGAA
CTCCCGGATCTCTAATTGGAGATGATCAAATTTATAATACTATTGTAACAGCTCATGCTTTTATTATAATTTTTTTCATA
GTTATACCAATCATAATTGGAGGATTTGGAAATTGACTTGTACCTTTAATATTAGGAGCACCTGATATAGCATTCCCACG
AATAAATAACATAAGTTTTTGATTACTTCCACCTTCTTTAACTCTTCTCATTTCAAGTAGAATTGTAGAAAATGGAGCAG
GAACTGGATGAACAGTTTACCCACCACTCTCATCTAATATTGCTCATAGTGGAAGATCCGTAGACTTAGCTATTTTTTCC
CTCCATTTAGCAGGAATCTCTTCCATCCTAGGAGCTATTAATTTTATTACAACTATTATTAATATACGATTAAATAACTT
ATCTTTTGATCAAATACCTTTATTTATTTGAGCTGTAGGAATTACAGCATTTTTATTATTATTATCATTACCTGTATTAG
CTGGAGCTATTACTATATTACTAACAGATCGAAATTTAAATACATCATTTTTCGATCCCGCAGGAGGAGGGGATCCAATT
TTATATCAACATTTATTT---------
>AMWW084-11|Bathytricha_monticola|K290824|
AACATTATATTTTATTTTTGGAATTTGAGCAGGAATAGTAGGAACCTCTTTAAGACTATTAATTCGAGCTGAATTAGGAA
CACCTGGATCTTTAATTGGAGATGATCAAATTTATAATACTATTGTAACAGCTCATGCTTTTATTATAATTTTTTTTATA
GTTATACCAATTATAATTGGAGGGTTTGGAAATTGACTTGTACCTTTAATATTAGGAGCACCTGATATAGCATTCCCACG
AATAAATAATATAAGTTTTTGATTACTTCCACCCTCTTTAACTCTCCTTATTTCAAGTAGAATTGTAGAAAATGGGGCAG
GAACTGGATGAACAGTTTACCCACCACTCTCATCTAATATTGCTCATGGAGGAAGATCTGTAGACTTAGCTATTTTTTCT
CTCCATTTGGCGGGAATCTCTTCTATTCTAGGAGCTATTAATTTTATTACAACTATTATTAATATACGATTAAATAGTTT
ATCTTTTGATCAAATACCTTTATTTATTTGAGCCGTAGGTATTACAGCATTTTTATTATTATTATCATTACCTGTATTAG
CTGGAGCTATTACTATACTATTAACAGATCGAAATTTAAATACATCATTTTTTGATCCTGCAGGAGGAGGAGATCCAATT
TTATATCAACATTTATTT---------
>AMWW087-11|Bathytricha|K290827|
AACATTATATTTTATTTTTGGAATTTGAGCAGGAATAGTGGGAACCTCTTTAAGACTACTAATTCGCGCTGAATTAGGAA
CTCCCGGATCTCTAATTGGAGATGATCAAATTTATAATACTATTGTAACAGCTCATGCTTTTATTATAATTTTTTTCATA
GTTATACCAATCATAATTGGAGGATTTGGAAATTGACTTGTACCTTTAATATTAGGAGCACCTGATATAGCATTCCCACG
AATAAATAACATAAGTTTTTGATTACTTCCACCTTCTTTAACTCTTCTCATTTCAAGTAGAATTGTAGAAAATGGAGCAG
GAACTGGATGAACAGTTTACCCACCACTCTCATCTAATATTGCTCATAGTGGAAGATCCGTAGACTTAGCTATTTTTTCC
CTCCATTTAGCAGGAATCTCTTCCATCCTAGGAGCTATTAATTTTATTACAACTATTATTAATATACGATTAAATAGCTT
ATCTTTTGATCAAATACCTTTATTTATTTGAGCTGTAGGAATTACAGCATTTTTATTATTATTATCATTACCTGTATTAG
CTGGAGCTATTACTATATTACTAACAGATCGAAATTTAAATACATCATTTTTCGATCCCGCAGGAGGAGGGGATCCAATT
TTATATCAACATTTATTT---------
>AMWW088-11|Bathytricha|K290828|

AACATTATATTTTATTTTTGGAATTTGAGCAGGAATAGTGGGAACCTCTTTAAGACTACTAATTCGCGCTGAATTAGGAA
CTCCCGGATCTCTAATTGGAGATGATCAAATTTATAATACTATTGTAACAGCTCATGCTTTTATTATAATTTTTTTCATA
GTTATACCAATCATAATTGGAGGATTTGGAAATTGACTTGTACCTTTAATATTAGGAGCACCTGATATAGCATTCCCACG
AATAAATAACATAAGTTTTTGATTACTTCCACCTTCTTTAACTCTTCTCATTTCAAGTAGAATTGTAGAAAATGGAGCAG
GAACTGGATGAACAGTTTACCCACCACTCTCATCTAATATTGCTCATAGTGGAAGATCCGTAGACTTAGCTATTTTTTCC
CTCCATTTAGCAGGAATCTCTTCCATCCTAGGAGCTATTAATTTTATTACAACTATTATTAATATACGATTAAATAGCTT
ATCTTTTGATCAAATACCTTTATTTATTTGAGCTGTAGGAATTACAGCATTTTTATTATTATTATCATTACCTGTATTAG
CTGGAGCTATTACTATATTACTAACAGATCGAAATTTAAATACATCATTTTTCGATCCCGCAGGAGGAGGGGATCCAATT
TTATATCAACATTTATTT---------
>AMWW089-11|Bathytricha|K290829|
AACATTATATTTTATTTTTGGAATTTGAGCAGGAATAGTGGGAACCTCTTTAAGACTACTAATTCGCGCTGAATTAGGAA
CTCCCGGATCTCTAATTGGAGATGATCAAATTTATAATACTATTGTAACAGCTCATGCTTTTATTATAATTTTTTTCATA
GTTATACCAATCATAATTGGAGGATTTGGAAATTGACTTGTACCTTTAATATTAGGAGCACCTGATATAGCATTCCCACG
AATAAATAACATAAGTTTTTGATTACTTCCACCTTCTTTAACTCTTCTCATTTCAAGTAGAATTGTAGAAAATGGAGTAG
GAACTGGATGAACAGTTTACCCACCACTCTCATCTAATATTGCTCATAGTGGAAGATCCGTAGACTTAGCTATTTTTTCC
CTCCATTTAGCAGGAATCTCTTCCATCCTAGGAGCTATTAATTTTATTACAACTATTATTAATATACGATTAAATAGCTT
ATCTTTTGATCAAATACCTTTATTTATTTGAGCTGTAGGAATTACAGCATTTTTATTATTATTATCATTACCTGTATTAG
CTGGAGCTATTACTATATTACTAACAGATCGAAATTTAAATACATCATTTTTCGATCCCGCAGGAGGAGGGGATCCAATT
TTATATCAACATTTATTT---------
>ANIAB734-11|Scirpophaga_nivella|CCDB-15750-G02|KF399959
AACTTTATATTTCATTTTTGGAATTTGAGCTGGTATAGTAGGAACTTCTTTAAGATTATTAATTCGAGCTGAATTAGGAA
CTCCAGGATCTTTAATTGGAGATGATCAAATTTATAATACCATTGTTACAGCTCATGCTTTTATTATAATTTTTTTTATA
GTAATACCAATTATAATTGGAGGATTTGGAAATTGACTTGTTCCTTTAATATTAGGAGCTCCTGATATAGCTTTCCCCCG
TATAAATAATATAAGATTTTGATTATTACCCCCCTCATTAACTCTACTAATTTCAAGAAGAATTGTAGAAAATGGTGCAG
GAACAGGATGAACAGTATACCCTCCCCTATCATCAAATATTGCTCACGGGGGAACTTCTGTAGATTTAGCTATTTTCTCT
TTACATCTTGCAGGAATTTCCTCTATTTTAGGAGCTATTAACTTTATTACCACTATTATTAATATACGAATTAATGGCTT
AACATTTGATCAAATACCCCTCTTTGTTTGAGCTGTTGGAATTACAGCCCTTCTTTTACTTCTCTCATTACCTGTATTAG
CTGGAGCTATTACTATATTATTAACTGATCGAAATTTAAATACCTCTTTTTTTGATCCAGCAGGAGGAGGAGATCCAATC
CTTTATCAACATTTATTT---------
>ANIAG766-11|Acrapex_sp._ANIC1|CCDB-15856-C12|KF390299
AACATTATATTTTATTTTTGGAATTTGAGCTGGTATAATAGGAACTTCTTTAAGTTTATTAATTCGAGCTGAACTAGGAA
CTCCTGGATCTTTAATTAGAGATGATCAAATTTATAATACTATTGTTACAGCACATGCTTTTATCATAATTTTTTTTATA
GTTATACCAATTATAATCGGAGGATTTGGAAATTGACTTATTCCATTAATATTAGGAGCCCCAGATATAGCATTCCCCCG
AATAAATAATATAAGATTTTGATTATTACCACCCTCCTTAATTTTATTAATTTCAAGTAGAATCGTAGAAAATGGAGTAG
GAACCGGATGAACAGTATATCCACCTCTTTCATCAAATATTGCTCACAACGGAAGATCAGTAGACTTAGCTATTTTTTCC
CTTCATTTAGCTGGAATTTCATCTATTTTAGGAGCCATTAATTTTATTACAACAATTATTAATATACGATTAAATAATTT
ATCTTTTGACCAATTACCATTATTTATTTGAGCTGTTGGAATTACTGCATTTCTATTATTACTTTCATTA----------
----

>ANIAG770-11|Acrapex_exsanguis|CCDB-15856-D04|KF389678
AACATTATATTTTATTTTTGGAATTTGAGCTGGTATACTAGGAACTTCTTTAAGTTTATTAATCCGAGCTGAATTAGGAA
CTCCAGAATCTTTAATTGGAGATGATCAAATTTATAATACTATTGTTACTGCTCACGCTTTTATTATAATTTTCTTTATA
GTTATACCAATTATAATTGGAGGATTTGGAAATTGACTTGTCCCATTAATACTAGGAGCTCCAGATATAGCATTTCCACG
TATAAATAATATAAGATTTTGATTATTACCTCCCTCTTTAAGTTTATTAATTTCAAGAAGAATTGTAGAAAATGGAGCAG
GAACTGGATGAACAGTATATCCTCCACTCTCATCTAATATTGCTCATAGAGGAAGATCAGTAGATTTAGCTATTTTTTCT
CTTCATTTAGCTGGTATTTCATCTATTTTAGGAGCTATTAATTTTATTACAACAATTATTAATATACGATTAAATAATTT
ATCTTTTGATCAAATACCTTTATTTGTTTGAGCTGTTGGAATTACTGCATTTTTACTATTACTCTCATTA----------
----

>ANIAG773-11|Acrapex_sp._ANIC6|CCDB-15856-D07|KF389783
AACACTATATTTTATTTTTGGAATTTGAGCTGGTATGTTAGGAACTTCTTTAAGTTTATTAATTCGAGCTGAATTAGGAA
CTCCTGGATCTTTAATTGGAGATGATCAAATCTATAATACTATTGTTACAGCTCATGCTTTTATTATAATTTTTTTTATA
GTTATACCAATTATAATTGGAGGATTTGGAAATTGACTTGTTCCATTAATATTAGGAGCTCCAGACATAGCATTTCCACG
TATAAATAATATAAGATTTTGATTATTACCCCCTTCTTTAACTCTATTAATTTCAAGTAGAATTGTAGAAAATGGGGCAG
GAACTGGATGAACAGTATATCCCCCACTTTCATCTAATATTGCTCATGGTGGAAGATCGGTAGATTTAGCTATTTTTTCT
CTTCATTTAGCTGGTATTTCATCTATTTTAGGAGCTATTAATTTTATTACAACAATTATTAATATACGATTAAATAATTT
ATCTTTTGATCAAATACCTTTATTTATTTGAGCTGTTGGAATTACTGCATTTTTACTATTACTTTCATTACCTGTATTAG

CTGGAGCTATTACAATATTATTAACTGATCGAAATTTAAATACATCATTTTTTGATCCCGCAGGAGGAGGAGATCCAATT
TTATATCAACATTTATTT---------
>ANIAG776-11|Acrapex_sp._ANIC1|CCDB-15856-D10|KF394482
AACATTATATTTTATTTTTGGAATTTGAGCTGGTATAATAGGAACTTCTTTTAGTTTATTAATTCGAGCTGAACTAGGAA
CTCCTGGATCTTTAATTAGAGATGATCAAATTTATAATACTATTGTTACAGCACATGCTTTTATCATAATTTTTTTTATA
GTTATACCAATTATAATTGGAGGATTTGGAAATTGACTTATTCCATTAATATTAGGAGCCCCAGATATAGCATTCCCCCG
AATAAATAATATAAGATTTTGATTATTACCACCCTCCTTAATTTTATTAATTTCAAGTAGAATCGTAGAAAATGGAGTAG
GAACCGGATGAACAGTATATCCACCTCTTTCATCAAATATTGCTCACAACGGAAGATCAGTAGACTTAGCTATTTTTTCC
CTTCATTTAGCTGGAATTTCATCTATTTTAGGAGCCATTAATTTTATTACAACAATTATTAATATACGATTAAAATAATTT
ATCTTTTGACCAATTACCATTATTTATTTGAGCTGTTGGAATTACTGCATTTCTATTATTACTTCATTA----------
----

>ANIAG784-11|Bathytricha_leonina|CCDB-15856-E06|KF389045
AACATTATATTTTATTTTTGGAATTTGAGCAGGAATAGTAGGAACCTCTTTAAGACTATTAATTCGAGCTGAATTAGGAA
CACCTGGATCTTTAATTGGAGATGATCAAATTTATAATACTATTGTAACAGCTCATGCTTTTATTATAATTTTTTTTATA
GTTATACCAATTATAATTGGAGGGTTTGGAAATTGACTTGTACCTTTAATATTAGGAGCACCTGATATAGCATTCCCACG
AATAAATAATATAAGTTTTTGATTACTTCCACCCTCTTTAACTCTCCTTATTTCAAGTAGAATTGTAGAAAATGGGGCAG
GAACTGGATGAACAGTTTACCCACCACTCTCATCTAATATTGCTCATGGAGGAAGATCTGTAGACTTAGCTATTTTTTCT
CTCCATTTGGCGGGAATCTCTTCTATTCTAGGAGCTATTAATTTTATTACAACTATTATTAATATACGATTAAATAGCTT
ATCTTTTGATCAAATACCTTTATTTATTTGAGCCGTAGGTATTACAGCATTTTTATTATTATTATCATTACCTGTATTAG
CTGGAGCTATTACTATACTATTAACAGATCGAAATTTAAATACATCATTTTTTGATCCTGCAGGAGGAGGAGATCCAATT
TTATATCAACATTTATTT---------
>ANIAG786-11|Bathytricha_leonina|CCDB-15856-E08|KF388729
AACATTATATTTTATTTTTGGAATTTGAGCAGGAATAGTAGGAACCTCTTTAAGACTATTAATTCGAGCTGAATTAGGAA
CACCTGGATCTTTAATTGGAGATGATCAAATTTATAATACTATTGTAACAGCTCATGCTTTTATTATAATTTTTTTTATA
GTTATACCAATTATAATTGGAGGGTTTGGAAATTGACTTGTACCTTTAATATTAGGAGCACCTGATATAGCATTCCCACG
AATAAATAATATAAGTTTTTGATTACTTCCACCCTCTTTAACTCTCCTTATTTCAAGTAGAATTGTAGAAAATGGGGCAG
GAACTGGATGAACAGTTTACCCACCACTCTCATCTAATATTGCTCATGGAGGAAGATCTGTAGACTTAGCTATTTTTTCT
CTCCATTTGGCGGGAATCTCTTCTATTCTAGGAGCTATTAATTTTATTACAACTATTATTAATATACGATTAAATAGCTT
ATCTTTTGATCAAATACCTTTATTTATTTGAGCCGTAGGTATTACAGCATTTTTATTATTATTATCATTACCTGTATTAG
CTGGAGCTATTACTATACTATTAACAGATCGAAATTTAAATACATCATTTTTTGATCCTGCAGGAGGAGGAGATCCAATT
TTATATCAACATTTATTT---------
>ANIAG788-11|Bathytricha_monticola|CCDB-15856-E10|KF388841
AACATTATATTTTATTTTTGGAATTTGAGCAGGAATAGTAGGAACCTCTTTAAGACTATTAATTCGAGCTGAATTAGGAA
CACCTGGATCTTTAATTGGAGATGATCAAATTTATAATACTATTGTAACAGCTCATGCTTTTATTATAATTTTTTTTATA
GTTATACCAATTATAATTGGAGGGTTTGGAAATTGACTTGTACCTTTAATATTAGGAGCACCTGATATAGCATTCCCACG
AATAAATAATATAAGTTTTTGATTACTTCCACCCTCTTTAACTCTCCTTATTTCAAGTAGAATTGTAGAAAATGGGGCAG
GAACTGGATGAACAGTTTACCCACCACTCTCATCTAATATTGCTCATGGAGGAAGATCTGTAGACTTAGCTATTTTTTCT
CTCCATTTGGCGGGAATCTCTTCTATTCTAGGAGCTATTAATTTTATTACAACTATTATTAATATACGATTAAATAGTTT
ATCTTTTGATCAAATACCTTTATTTATTTGAGCCGTAGGTATTACAGCATTTTTATTATTATTATCATTA----------
----

>ANIAG789-11|Bathytricha_monticola|CCDB-15856-E11|KF392527
AACATTATATTTTATTTTTGGAATTTGAGCAGGAATAGTAGGAACCTCTTTAAGACTATTAATTCGAGCTGAATTAGGAA
CACCTGGATCTTTAATTGGAGATGATCAAATTTATAATACTATTGTAACAGCTCATGCTTTTATTATAATTTTTTTTATA
GTTATACCAATTATAATTGGAGGGTTTGGAAATTGACTTGTACCTTTAATATTAGGAGCACCTGATATAGCATTCCCACG
AATAAATAATATAAGTTTTTGATTACTTCCACCCTCTTTAACTCTCCTTATTTCAAGTAGAATTGTAGAAAATGGGGCAG
GAACTGGATGAACAGTTTACCCACCACTCTCATCTAATATTGCTCATGGAGGAAGATCTGTAGACTTAGCTATTTTTTCT
CTCCATTTGGCGGGAATCTCTTCTATTCTAGGAGCTATTAATTTTATTACAACTATTATTAATATACGATTAAATAGTTT
ATCTTTTGATCAAATACCTTTATTTATTTGAGCCGTAGGTATTACAGCATTTTTATTATTATTATCATTA----------
----

>ANICC223-09|Tetramoera_gracilistria|ANIC_Gen_No._003385|HM372983
AACATTATATTTTATTTTTGGAATTTGATCAGGAATAATTGGTACATCTTTAAGATTATTAATTCGAGCTGAATTAGGAA
ATCCTGGATCTTTAATTGGAGATGATCAAATTTATAATACTATTGTAACCGCTCATGCTTTTATTATAATTTTTTTTATA
GTTATACCTATTATAATTGGAGGATTTGGAAATTGATTAGTTCCTTTAATATTAGGAGCTCCTGATATAGCTTTTCCTCG
TATAAATAATATAAGATTCTGATTACTTCCTCCTTCCATTATATTATTAATTTCAAGAAGAATTGTAGAAAATGGAGCAG

GAACTGGATGAACAGTTTACCCCCCACTATCATCCAATATTGCTCATAGAGGTAGTTCCGTAGATTTAGCTATTTTTTCT
TTACATCTAGCTGGAATTTCTTCCATTTTAGGAGCTGTAAACTTTATTACCACTATTATTAATATACGACCAAATAATAT
AAGACTAGATCAAATACCATTATTTGTTTGAGCTGTCGGTATTACAGCCTTATTATTACTTTTATCTTTACCAGTATTAG
CAGGTGCTATTACTATACTATTAACAGATCGTAATTTAAATACTTCATTTTTTGATCCTGCA------------------
----
>ANICK688-10|Acrapex_exsanguis|10ANIC-07685|HQ950760
AACATTATATTTTATTTTTGGAATTTGAGCTGGTATACTAGGAACTTCTTTAAGTTTATTAATCCGAGCTGAATTAGGAA
CTCCAGAATCTTTAATTGGAGATGATCAAATTTATAATACTATTGTTACTGCTCACGCTTTTATTATAATTTTCTTTATA
GTTATACCAATTATAATTGGAGGATTTGGAAATTGACTCGTCCCATTAATATTAGGAGCTCCAGATATAGCATTTCCACG
TATAAATAATATAAGATTTTGATTATTACCTCCCTCTTTAAGTTTATTAATTTCAAGAAGAATTGTAGAAAATGGAGCAG
GAACTGGATGAACAGTATATCCTCCACTCTCATCTAATATTGCTCATAGAGGAAGATCAGTAGATTTAGCTATTTTTTCT
CTTCATTTAGCTGGTATTTCATCTATTTTAGGAGCTATTAATTTTATTACAACAATTATTAATATACGATTAAATAATTT
ATCTTTTGATCAAATACCTTTATTTGTTTGAGCTGTTGGAATTACTGCATTTTTACTATTACTCTCATTACCTGTATTAG
CCGGAGCTATTACAATATTATTAACAGATCGAAATTTAAATACATCATTTTTTGATCCTGCAGGAGGAGGGGATCCAATT
TTATATCAACATTTATTT---------
>ANICK689-10|Acrapex_exsanguis|10ANIC-07686|HQ950761
AACATTATATTTTATTTTTGGAATTTGAGCTGGTATACTAGGAACTTCTTTAAGTTTATTAATCCGAGCTGAATTAGGAA
CTCCAGAATCTTTAATTGGAGATGATCAAATTTATAATACTATTGTTACTGCTCACGCTTTTATTATAATTTTCTTTATA
GTTATACCAATTATAATTGGAGGATTTGGAAATTGACTTGTCCCATTAATACTAGGAGCTCCAGATATAGCATTTCCACG
TATAAATAATATAAGATTTTGATTATTACCTCCCTCTTTAAGTTTATTAATTTCAAGAAGAATTGTAGAAAATGGAGCAG
GAACTGGATGAACAGTATATCCTCCACTCTCATCTAATATTGCTCATAGAGGAAGATCAGTAGATTTAGCTATTTTTTCT
CTTCATTTAGCTGGTATTTCATCTATTTTAGGAGCTATTAATTTTATTACAACAATTATTAATATACGATTAAATAATTT
ATCTTTTGATCAAATACCTTTATTTGTTTGAGCTGTTGGAATTACTGCATTTTTACTATTACTCTCATTACCTGTATTAG
CCGGAGCTATTACAATATTATTAACAGATCGAAATTTAAATACATCATTTTTTGATCCTGCAGGAGGAGGTGATCCAATT
TTATATCAACATTTATTT---------
>ANICK691-10|Acrapex_albicostata|10ANIC-07688|HQ950762
AACATTATATTTTATTTTTGGAATTTGAACCGGTATAATAGGAACTTCTTTAAGTCTATTAATTCGAGCTGAATTAGGAA
TACCTAGATCTTTAATTGGAGATGATCAAATTTATAATACTATTGTTACAGCTCATGCTTTTATTATAATTTTTTTTATA
GTTATACCAATTATAATTGGAGGATTTGGAAATTGACTTATCCCATTAATATTAGGAGCTCCAGATATAGCATTTCCTCG
TATAAATAATATAAGATTTTGATTATTACCTCCCTCTTTAACTTTATTAATTTCAAGTAGAATTGTAGAAAATGGAATAG
GAACAGGATGAACAGTATATCCACCTCTTTCATCTAATATTGCTCATAGAGGAAGATCAGTAGACTTAGCTATTTTTTCC
CTTCATTTAGCTGGAATTTCATCTATTTTAGGGGCTATTAATTTTATTACAACAATTATTAATATACGATTAAATAATTT
ATCTTTTGATCAAATACCTTTATTTATTTGAGCCATTGCCATTACTGCATTCTTACTATTACTTTCATTACCCGTATTAG
CTGGAGCAATTACAATATTATTAACAGATCGAAATTTAAATACATCATTTTTTGACCCTGCAGGAGGAGGTGATCCAATT
TTATATCAACATTTATTT---------
>ANICK693-10|Acrapex_albicostata|10ANIC-07690|HQ950763
TACATTATATTTTATTTTTGGAATTTGGGCAGGTATAGTAGGAACCTCTTTAAGATTATTAATTCGGGCTGAATTAGGAA
CCCCAGGATCTTTAATTGGAGATGATCAAATTTATAATACTATTGTTACAGCTCATGCTTTTATTATAATTTTCTTTATA
GTTATACCTATTATGATTGGGGGATTTGGAAATTGACTTGTCCCTTTAATATTAGGTGCTCCAGATATAGCATTTCCACG
AATAAATAATATAAGTTTTTGATTACTACCCCCCTCTTTAACCCTTCTTATTTCCAGTAGAATTGTAGAAAATGGAGCAG
GAACTGGATGAACTGTATACCCACCCCTTTCATCTAATATTGCCCATGGGGGAAGATCTGTAGATTTAGCTATTTTTTCT
TTACATTTAGCTGGAATTTCTTCTATTTTAGGAGCTATTAACTTTATTACTACAATTATTAATATACGATTAAATAGTTT
ATCCTTTGATCAAATACCTTTATTTATTTGAGCTGTAGGAATTACTGCATTTTTATTATTACTTTCTTTACCTGTATTAG
CTGGGGCTATTACAATACTATTAACAGATCGAAACTTAAATACCTCTTTTTTTGACCCTGCTGGAGGTGGAGATCCAATT
TTATATCAACATTTATTT---------
>ANICK694-10|Acrapex_albicostata|10ANIC-07691|HQ950764
TACATTATATTTTATTTTTGGAATTTGAGCAGGTATAATAGGAACCTCTTTAAGATTATTAATTCGGGCTGAATTAGGAA
CCCCAGGATCTTTAATTGGAGATGATCAAATTTATAACACTATTGTTACAGCTCATGCTTTTATTATAATTTTCTTTATG
GTCATACCTATTATAATTGGAGGATTTGGAAATTGACTTGTCCCTTTAATACTAGGTGCCCCAGATATAGCATTTCCACG
AATAAATAACATAAGTTTTTGGTTATTACCCCCCTCTTTAACCCTTCTTATTTCCAGTAGAATTGTAGAAAATGGAGCAG
GAACTGGATGAACTGTATACCCACCCCTTTCATCTAATATTGCCCATGGGGGAAGATCTGTAGATTTAGCTATTTTTTCT
TTACATTTAGCTGGGATTTCTTCTATTTTAGGAGCTATTAACTTTATTACTACAATTATTAATATACGATTAAATAGTTT
ATCCTTTGATCAAATACCTTTATTTATTTGAGCTGTAGGAATTACTGCATTTTTATTATTACTTTCTTTACCTGTATTAG
CTGGGGCTATTACAATATTATTAACAGATCGAAATTTAAATACCTCTTTTTTTGACCCTGCTGGAGGTGGAGATCCAATT
TTATATCAACATTTATTT---------
>ANICK695-10|Acrapex_albicostata|10ANIC-07692|HQ950765
TACATTATATTTTATTTTTGGAATTTGGGCAGGTATAGTAGGAACCTCTTTAAGATTATTAATTCGGGCTGAATTAGGAA

CCCCAGGATCTTTAATTGGAGATGATCAAATTTATAATACTATTGTTACAGCTCATGCTTTTATTATAATTTTCTTTATA
GTTATACCTATTATGATTGGAGGATTTGGAAATTGACTTGTCCCTTTAATATTAGGTGCTCCAGATATAGCATTTCCACG
AATAAATAATATAAGTTTTTGATTACTACCCCCCTCTTTAACCCTTCTTATTTCCAGTAGAATTGTAGAAAATGGAGCAG
GAACTGGATGAACTGTATACCCACCCCTTTCATCTAATATTGCCCATGGGGGAAGATCTGTAGATTTAGCTATTTTTTCT
TTACATTTAGCTGGAATTTCTTCTATTTTAGGAGCTATTAACTTTATTACTACAATTATTAATATACGATTAAATAGTTT
ATCCTTTGATCAAATACCTTTATTTATTTGAGCTGTAGGAATTACTGCATTTTTATTATTACTTTCTTTACCTGTATTAG
CTGGGGCTATTACAATACTATTAACAGATCGAAACTTAAATACCTCTTTTTTTGACCCTGCTGGAGGTGGAGATCCAATT
TTATATCAACATTTATTT---------
>ANICK696-10|Acrapex_albicostata|10ANIC-07693|HQ950766
TACATTATATTTTATTTTTGGAATTTGGGCAGGTATAGTAGGAACCTCTTTAAGATTATTAATTCGGGCTGAATTAGGAA
CCCCAGGATCTTTAATTGGAGATGATCAAATTTATAATACTATTGTTACAGCTCATGCTTTTATTATAATTTTCTTTATA
GTTATACCTATTATGATTGGGGGATTTGGAAATTGACTTGTCCCTTTAATATTAGGTGCTCCAGATATAGCATTTCCACG
AATAAATAATATAAGTTTTTGATTACTACCCCCCTCTTTAACTCTTCTTATTTCCAGTAGAATTGTAGAAAATGGAGCAG
GAACTGGATGAACTGTATACCCACCCCTTTCATCTAATATTGCCCATGGGGGAAGATCTGTAGATTTAGCTATTTTTTCT
TTACATTTAGCTGGAATTTCTTCTATTTTAGGAGCTATTAACTTTATTACTACAATTATTAATATACGATTAAATAGTTT
ATCCTTTGATCAAATACCTTTATTTATTTGAGCTGTAGGAATTACTGCATTTTTATTATTACTTTCTTTACCTGTATTAG
CTGGGGCTATTACAATACTATTAACAGATCGAAACTTAAATACCTCTTTTTTTGACCCTGCTGGAGGTGGAGATCCAATT
TTATATCAACATTTATTT---------
>ANICK697-10|Acrapex_sp._ANIC2|10ANIC-07694|HQ950767
AACATTATATTTTATTTTTGGAATTTGAGCTGGTATAGTAGGAACTTCATTAAGATTATTAATTCGAGCTGAATTAGGAA
CCCCTGGATCTTTAATTGGAGATGATCAAATTTATAATACTATTGTTACAGCTCATGCTTTTATTATAATTTTTTTCATG
GTTATACCAATTATAATTGGAGGATTTGGAAATTGACTTGTACCTTTAATATTAGGAGCTCCAGATATAGCATTTCCACG
AATAAATAATATAAGATTTTGATTATTACCTCCTTCTTTAACTTTATTAATTTCAAGTAGAATTGTAGAAAATGGAGCAG
GAACTGGATGAACAGTATATCCACCTCTCTCATCAAATATTGCACATGGAGGAAGATCTGTAGATTTAGCTATTTTTTCT
CTTCATTTAGCGGGTATTTCTTCTATTTTAGGAGCTATTAATTTTATCACAACAATTATCAATATACGATTAAATAATTT
ATCTTTTGATCAAATACCTCTATTTATTTGAGCTGTTGGAATTACCGCTTTTTTATTACTATTATCTTTACCTGTATTAG
CAGGAGCTATCACAATATTATTAACTGATCGAAATTTAAATACATCATT-------------------------------
----
>ANICK698-10|Acrapex_exsanguis|10ANIC-07695|HQ950768
AACATTATATTTTATTTTTGGAATTTGAGCTGGTATACTAGGAACTTCTTTAAGTTTATTAATCCGAGCTGAATTAGGAA
CTCCAGAATCTTTAATTGGAGATGATCAAATTTATAATACTATTGTTACTGCTCACGCTTTTATTATAATTTTCTTTATA
GTTATACCAATTATAATTGGAGGATTTGGAAATTGACTTGTCCCATTAATATTAGGAGCTCCAGATATAGCATTTCCACG
TATAAATAATATAAGATTTTGATTATTACCTCCCTCTTTAAGTTTATTAATTTCAAGAAGAATTGTAGAAAATGGAGCAG
GAACTGGGTGAACAGTATATCCTCCACTCTCATCTAATATTGCTCATAGAGGAAGATCAGTAGATTTAGCTATTTTTTCT
CTTCATTTAGCTGGTATTTCATCTATTTTAGGAGCTATTAATTTTATTACAACAATTATTAATATACGATTAAATAATTT
ATCTTTTGATCAAATACCTTTATTTGTTTGAGCTGTTGGAATTACTGCATTTTTACTATTACTCTCATTACCTGTATTAG
CCGGAGCTATTACAATATTATTAACAGATCGAAATTTAAATACATCATT-------------------------------
----
>ANICK699-10|Acrapex_exsanguis|10ANIC-07696|HQ950769
AACATTATATTTTATTTTTGGAATTTGAGCTGGTATACTAGGAACTTCTTTAAGTTTATTAATCCGAGCTGAATTAGGAA
CTCCAGAATCTTTAATTGGAGATGATCAAATTTATAATACTATTGTTACTGCTCACGCTTTTATTATAATTTTCTTTATA
GTTATACCAATTATAATTGGAGGATTTGGAAATTGACTTGTCCCATTAATATTAGGAGCTCCAGATATAGCATTTCCACG
TATAAATAATATAAGATTTTGATTATTACCTCCCTCTTTAAGTTTATTAATTTCAAGAAGAATTGTAGAAAATGGAGCAG
GAACTGGATGAACAGTATATCCTCCACTCTCATCTAATATTGCTCATAGAGGAAGATCAGTAGATTTAGCTATTTTTTCT
CTTCATTTAGCTGGTATTTCATCTATTTTAGGAGCTATTAATTTTATTACAACAATTATTAATATACGATTAAATAATTT
ATCTTTTGATCAAATACCTTTATTTGTTTGAGCTGTTGGAATTACTGCATTTTTACTATTACTCTCATTACCTGTATTAG
CCGGAGCTATTACAATATTATTAACAGATCGAAATTTAAATACATCATTTTTTGATCCTGCAGGAGGAGGTGATCCAATT
TTATATCAACATTTATTT---------
>ANICK700-10|Acrapex_exsanguis|10ANIC-07697|HQ950770
AACATTATATTTTATTTTTGGAATTTGAGCTGGTATACTAGGAACTTCTTTAAGTTTATTAATCCGAGCTGAATTAGGAA
CTCCAGAATCTTTAATTGGAGATGATCAAATTTATAATACTATTGTTACTGCTCACGCTTTTATTATAATTTTCTTTATA
GTTATACCAATTATAATTGGAGGATTTGGAAATTGACTTGTCCCATTAATATTAGGAGCTCCAGATATAGCATTTCCACG
TATAAATAATATAAGATTTTGATTATTACCTCCCTCTTTAAGTTTATTAATTTCAAGAAGAATTGTAGAAAATGGAGCAG
GAACTGGATGAACAGTATATCCTCCACTCTCATCTAATATTGCTCATAGAGGAAGATCAGTAGATTTAGCTATTTTTTCT
CTTCATTTAGCTGGTATTTCATCTATTTTAGGAGCTATTAATTTTATTACAACAATTATTAATATACGATTAAATAATTT
ATCTTTTGATCAAATACCTTTATTTGTTTGAGCTGTTGGAATTACTGCATTTTTACTATTACTTTCATTACCTGTATTAG
CCGGAGCTATTACAATATTATTAACAGATCGAAATTTAAATACATCATTTTTTGATCCTGCAGGAGGAGGTGATCCAATT

TTATACCAACATTTATTT---------
>ANICK701-10|Acrapex_exsanguis|10ANIC-07698|HQ950771
AACATTATATTTTATTTTTGGAATTTGAGCTGGTATACTAGGAACTTCTTTAAGTTTATTAATCCGAGCTGAATTAGGAA
CTCCAGAATCTTTAATTGGAGATGATCAAATTTATAATACTATTGTTACTGCTCACGCTTTTATTATAATTTTCTTTATA
GTTATACCAATTATAATTGGAGGATTTGGAAATTGACTTGTCCCATTAATACTAGGAGCTCCAGATATAGCATTTCCACG
TATAAATAATATAAGATTTTGATTATTACCTCCCTCTTTAAGTTTATTAATTTCAAGAAGAATTGTAGAAAATGGAGCAG
GAACTGGATGAACAGTATATCCTCCACTCTCATCTAATATTGCTCATAGAGGAAGATCAGTAGATTTAGCTATTTTTTCT
CTTCATTTAGCTGGTATTTCATCTATTTTAGGAGCTATTAATTTTATTACAACAATTATTAATATACGATTAAATAATTT
ATCTTTTGATCAAATACCTTTATTTGTTTGAGCTGTTGGAATTACTGCATTTTTACTATTACTCTCATTACCTGTATTAG
CCGGAGCTATTACAATATTATTAACAGATCGAAATTTAAATACATCATTTTTTGATCCTGCAGGAGGAGGTGATCCAATT
TTATATCAACATTTATTT---------
>ANICK702-10|Acrapex_sp._ANIC1|10ANIC-07699|HQ950772
AACATTATATTTTATTTTTGGAATTTGAGCTGGTATAATAGGAACTTCTTTAAGTTTATTAATTCGAGCTGAACTAGGAA
CTCCTGGATCTTTAATTAGAGATGATCAAATTTATAATACTATTGTTACAGCACATGCTTTTATCATAATTTTTTTTATA
GTTATACCAATTATAATCGGAGGATTTGGAAATTGACTTATTCCATTAATATTAGGAGCCCCAGATATAGCATTCCCCCG
AATAAATAATATAAGATTTTGATTATTACCACCCTCCTTAATTTTATTAATTTCAAGTAGAATCGTAGAAAATGGAGTAG
GAACCGGATGAACAGTATATCCACCTCTTTCATCAAATATTGCTCACAACGGAAGATCAGTAGACTTAGCTATTTTTTCC
CTTCATTTAGCTGGAATTTCATCTATTTTAGGAGCCATTAATTTTATTACAACAATTATTAATATACGATTAAATAATTT
ATCTTTTGACCAATTACCATTATTTATTTGAGCTGTTGGAATTACTGCATTTCTATTATTACTTTCATTACCTGTATTAG
CAGGAGCTATTACAATATTATTAACTGATCGAAATTTAAATACATCCTTTTTTGACCCAGCAGGAGGAGGTGATCCAATT
TTATATCAACATTTATTT---------
>ANICK703-10|Acrapex_sp._ANIC5|10ANIC-07700|HQ950773
AACATTATACTTTATTTTCGGAATTTGAGCTGGTATAATAGGAACTTCTTTAAGTTTATTAATTCGAGCTGAACTAGGAA
CTCCCGGATTTTTAATTAGAGATGATCAAATTTATAATACTATTGTTACAACTCATGCTTTTGTTATAATTTTTTTTATA
GTTATACCAATTATAATTGGCGGATTTGGGAATTGACTTATCCCATTAATATTAGGAGCCCCAGATATAGCATTTCCCCG
AATAAATAATATAAGATTTTGATTATTACCACCTTCCTTAATTTTATTAATTTCAAGCAGAATCGTAGAAAATGGGGCAG
GAACCGGATGAACAGTATACCCACCTCTTTCATCAAATATTGCTCACAACGGAAGATCAGTAGACTTAACTATTTTTTCC
CTTCATTTAGCCGGAATTTCATCTATTTTAGGAGCTATTAATTTTATTACAACAATTATTAATATACGATTAAATAATTT
ATCTTTTGACCAATTACCATTATTTGTTTGAGCTGTTGGAATTACTGCATTCTTATTATTACTTTCATTACCTGTATTAG
CAGGAGCTATCACAATATTATTAACTGATCGAAATTTAAATACATCATTTTTTGACCCAGCAGGAGGAGGTGATCCAATT
TTATACCAACATTTATTT---------
>ANICK704-10|Acrapex_exsanguis|10ANIC-07701|HQ950774
AACATTATATTTTATTTTTGGAATTTGAGCTGGTATACTAGGAACTTCTTTAAGTTTATTAATCCGAGCTGAATTAGGAA
CTCCAGAATCTTTAATTGGAGATGATCAAATTTATAATACTATTGTTACTGCTCACGCTTTTATTATAATTTTTTTTATA
GTTATACCAATTATAATTGGAGGATTTGGAAATTGACTTGTCCCATTAATATTAGGAGCTCCAGATATAGCATTTCCACG
TATAAATAATATAAGATTTTGATTATTACCTCCCTCTTTAAGTTTATTAATTTCAAGAAGAATTGTAGAAAATGGAGCAG
GAACTGGATGAACAGTATATCCCCCACTCTCATCTAATATTGCTCATAGAGGAAGATCAGTAGACTTAGCTATTTTTTCT
CTTCATTTAGCTGGTATTTCATCTATTTTAGGAGCTATTAATTTTATTACAACAATTATTAATATACGATTAAATAATTT
ATCTTTTGATCAAATACCTTTATTTGTTTGAGCTGTTGGAATTACTGCATTTTTACTATTACTCTCATTACCTGTATTAG
CCGGAGCTATTACAATATTATTAACAGATCGAAATTTAAATACATCATTTTTTGATCCTGCAGGGGGAGGTGATCCAAAT
TTATATCAACATTTATTT---------
>ANICK705-10|Acrapex_sp._ANIC3|10ANIC-07702|HQ950775
AACATTATATTTTATTTTTGGGATTTGAGCTGGTATAATAGGAACTTCTTTAAGTTTATTAATTCGAGCTGAATTAGGAA
CTCCTAGATTTTTAATTGGAGATGATCAAATTTATAATACTATTGTTACAGCTCATGCTTTCATTATAATTTTTTTTATA
GTTATACCTATTATAATTGGAGGATTTGGAAATTGACTTATTCCATTAATATTAGGAGCTCCAGATATAGCATTTCCGCG
AATAAATAATATAAGATTTTGATTATTACCTCCTTCTTTAATTTTATTAATTTCAAGTAGAATTGTAGAAAATGGAGCAG
GAACGGGATGAACAGTATACCCCCCACTTTCATCTAATATTAGTCATAGAGGAAGATCAGTAGATTTAACTATTTTTTCC
CTTCACTTAGCTGGTATTTCATCTATTTTAGGAGCCATTAATTTTATTACAACAATTATTAATATACGATTAAATAATTT
ATCTTTTGATCAAATACCTTTATTTATCTGAGCTGTTGGAATTACTGCATTTTTATTATTACTCTCACTACCTGTATTAG
CTGGAGCAATTACAATATTATTAACTGACCGAAATTTAAATACATCATTTTTCGATCCTGCAGGGGGAGGTGATCCAATT
TTATACCAACATTTATTT---------
>ANICK706-10|Acrapex_sp._ANIC6|10ANIC-07703|HQ950776
AACACTATATTTTATTTTTGGAATTTGAGCCGGTATGTTAGGAACTTCTTTAAGTTTATTAATTCGAGCTGAATTAGGAA
CTCCTGGATCTTTAATTGGAGATGATCAAATCTATAATACTATTGTTACAGCTCATGCTTTTATTATAATTTTTTTTATA
GTTATACCAATTATAATTGGAAGATTTGGAAATTGACTTGTTCCATTAATATTAGGAGCTCCAGACATAGCATTTCCACG
TATAAATAATATAAGATTTTGATTATTACCCCCTTCTTTAACTCTATTAATTTCAAGTAGAATTGTAGAAAATGGAGCAG
GAACTGGATGAACAGTATATCCCCCACTTTCATCTAATATTGCTCATGGTGGAAGATCAGTAGATTTAGCTATTTTTTCT

CTTCATTTAGCTGGTATTTCATCTATTTTAGGAGCTATTAATTTTATTACAACAATTATTAATATACGATTAAATAATTT
ATCTTTTGATCAAATACCTTTATTTATTTGAGCTGTTGGAATTACTGCATTTTTACTATTACTTTCATTACCTGTATTAG
CTGGAGCTATTACAATATTATTAACTGATCGAAATTTAAATACATCATTTTTTGATCCCGCAGGAGGGGGAGATCCAATT
TTATATCAACATTTATTT---------
>ANICK707-10|Acrapex_sp._ANIC2|10ANIC-07704|HQ950777
AACATTATATTTTATTTTTGGAATTTGAGCTGGTATAGTAGGAACTTCATTAAGATTATTAATTCGAGCTGAATTAGGAA
CCCCTGGATCTTTAATTGGAGATGATCAAATTTATAATACTATTGTTACAGCTCATGCTTTTATTATAATTTTTTTCATG
GTTATACCAATTATAATTGGAGGATTTGGAAATTGACTTGTACCTTTAATATTAGGAGCTCCAGATATAGCATTTCCACG
AATAAATAATATAAGATTTTGATTATTACCTCCTTCTTTAACTTTATTAATTTCAAGTAGAATTGTAGAAAATGGAACAG
GAACTGGATGAACAGTATATCCACCTCTCTCATCAAATATTGCACATGGAGGAAGATCTGTAGATTTAGCTATTTTTTCT
CTTCATTTAGCAGGTATTTCTTCTATTTTAGGAGCTATTAATTTTATCACAACAATTATCAATATACGATTAAATAATTT
ATCTTTTGATCAAATACCTCTATTTATTTGAGCTGTTGGAATTACCGCTTTTTTATTACTATTATCTTTACCTGTATTAG
CAGGAGCTATCACAATATTATTAACTGATCGAAATTTAAATACATCATTTTTTGATCCTGCAGGAGGAGGTGACCCAATT
TTATATCAACATTTATTT---------
>ANICK708-10|Acrapex_sp._ANIC2|10ANIC-07705|HQ950778
AACATTATATTTTATTTTTGGAATTTGAGCTGGTATAGTAGGAACTTCATTAAGATTATTAATTCGAGCTGAATTAGGAA
CCCCTGGATCTTTAATTGGAGATGATCAAATTTATAATACTATTGTTACAGCTCATGCTTTTATTATAATTTTTTTCATG
GTTATACCAATTATAATTGGAGGATTTGGAAATTGACTTGTACCTTTAATATTAGGAGCTCCAGATATAGCATTTCCACG
AATAAATAATATAAGATTTTGATTATTACCTCCTTCTTTAACTTTATTAATTTCAAGTAGAATTGTAGAAAATGGAGCAG
GAACTGGATGAACAGTATATCCACCTCTCTCATCAAATATTGCACATGGAGGAAGATCTGTAGATTTAGCTATTTTTTCT
CTTCATTTAGCAGGTATTTCTTCTATTTTAGGAGCTATTAATTTTATCACAACAATTATCAATATACGATTAAATAATTT
ATCTTTTGATCAAATACCTCTATTTATTTGAGCTGTTGGAATTACCGCTTTTTTATTACTATTATCTTTACCTGTATTAG
CAGGAGCTATCACAATATTATTAACTGATCGAAATTTAAATACATCATTTTTTGATCCTGCAGGAGGAGGTGACCCAATT
TTATATCAACATTTATTT---------
>ANICK709-10|Bathytricha_truncata|10ANIC-07706|HQ950779
AACATTATATTTCATCTTTGGAATTTGAGCAGGAATAGTGGGAACTTCTTTAAGACTATTAATTCGAGCTGAATTAGGAA
CTCCTGGATCCTTAATTGGAGATGATCAAATTTATAATACTATTGTAACAGCTCATGCCTTTATTATAATTTTTTTTATG
GTTATACCAATTATAATTGGAGGATTTGGAAATTGACTTGTACCTTTAATGTTAGGGGCACCTGATATAGCATTTCCACG
AATAAATAATATAAGTTTTTGATTACTACCCCCTTCTTTAACTCTACTTATTTCGAGAAGAGTTGTAGAAAATGGAGCAG
GAACTGGGTGAACAGTATACCCCCCACTTTCATCTAATATTGCCCATAGAGGAAGATCTGTGGACTTAGCTATTTTTTCC
CTTCATTTAGCTGGAATTTCTTCTATTTTAGGAGCTATTAATTTTATTACAACTATTATTAACATACGATTAAATAATTT
ATCTTTTGATCAAATACCTTTATTTATTTGAGCTGTAGGAATTACAGCATTTTTATTATTATTATCATTACCCGTATTAG
CTGGAGCTATTACCATATTATTAACAGATCGAAATTTAAATACATCATTTTTTGATCCTGCGGGAGGAGGAGATCCAATC
TTATATCAACATTTATTT---------
>ANICK710-10|Bathytricha_truncata|10ANIC-07707|HQ950780
AACATTATATTTCATTTTTGGGATTTGAGCAGGAATAGTAGGAACCTCTCTAAGACTATTAATTCGAGCTGAATTAGGGA
CTCCTGGATCTCTAATTGGAGATGACCAAATCTATAATACTATTGTAACAGCTCATGCCTTTATTATAATTTTTTTTATA
GTTATACCTATTATAATCGGAGGATTTGGGAATTGACTTGTACCTTTAATATTAGGAGCGCCTGATATGGCATTCCCACG
AATAAATAATATGAGTTTTTGGTTACTCCCACCCTCTTTAACTCTCCTTATTTCAAGTAGAGTTGTAGAAAATGGGGCGG
GAACTGGATGAACAGTTTACCCCCCACTCTCGTCTAATATTGCTCACGGGGGGGAGATCTGTAGACTTAGCTATTTTTTCC
CTCCACTTAGCTGGGATCTCTTCTATCCTGGGAGCTATTAATTTTATTACAACTATTATTAATATACGATTAAACAGTTT
ATCTTTTGATCAAATACCTTTATTTATTTGAGCTGTAGGAATTACAGCATTTTTATTATTACTATCATTACCTGTATTAG
CTGGGGCTATTACAATATTATTAACAGATCGTAATTTAAATACATCATTTTTTGATCCTGCGGGGGGAGGAGATCCAATT
TTATACCAACATTTATTT---------
>ANICK711-10|Bathytricha_truncata|10ANIC-07708|HQ950781
AACATTATATTTCATTTTTGGGATTTGAGCAGGAATAGTAGGAACCTCTCTAAGACTATTAATTCGAGCTGAATTAGGGA
CTCCTGGGTCTCTAATTGGAGATGATCAAATCTATAATACTATTGTAACAGCTCATGCCTTTATTATAATTTTTTTTATA
GTTATACCTATTATAATCGGAGGATTTGGGAATTGACTTGTACCTTTAATATTAGGAGCGCCTGATATGGCGTTCCCACG
AATAAATAATATGAGTTTTTGATTACTCCCACCCTCTTTAACTCTCCTTATTTCAAGTAGAATTGTAGAAAATGGGGCGG
GAACTGGATGAACAGTTTACCCCCCACTCTCATCTAATATTGCTCACGGGGGGGAGATCTGTAGACTTAGCTATTTTTTCC
CTCCACTTAGCTGGAATCTCTTCTATTCTGGGAGCTATTAATTTTATTACAACTATTATTAATATACGATTAAACAGTTT
ATCTTTTGATCAAATACCTTTATTTATTTGAGCTGTAGGAATTACAGCATTTTTATTATTACTATCATTACCTGTATTAG
CTGGGGCTATTACAATATTATTAACAGATCGTAATTTAAATACATCATT-------------------------------
----
>ANICK712-10|Bathytricha_truncata|10ANIC-07709|HQ950782
AACATTATATTTCATCTTTGGAATTTGAGCAGGAATAGTAGGAACTTCTTTAAGACTATTAATTCGAGCTGAATTAGGAA
CTCCTGGATCCTTAATTGGAGATGATCAAATTTATAATACTATTGTAACAGCTCATGCCTTTATTATAATTTTTTTTATA

GTTATACCAATTATAATCGGGGGATTTGGAAATTGACTTGTACCTTTAATGTTAGGAGCGCCTGATATGGCATTTCCACG
AATAAATAATATAAGTTTTTGATTACTACCCCCTTCTTTAACTCTACTTATTTCGAGAAGAGTTGTAGAAAATGGAGCAG
GAACTGGATGAACAGTATACCCTCCACTTTCATCTAATATTGCCCATAGAGGAAGATCTGTAGACTTAGCTATTTTTTCC
CTTCATTTAGCTGGAATTTCTTCTATTTTAGGAGCTATTAATTTTATTACAACTATTATTAACATACGATTAAATAATTT
ATCTTTTGATCAAATACCTTTATTTATTTGAGCTGTAGGAATTACAGCATTTTTATTATTATTATCATTACCTGTCTTAG
CTGGAGCTATTACTATATTATTAACAGATCGAAATTTAAATACATCATTTTTTGATCCTGCAGGAGGAGGAGATCCAATC
TTATATCAACATTTATTT---------
>ANICK713-10|Bathytricha_truncata|10ANIC-07710|HQ950783-SUPPRESSED
AACGTTATATTTCATTTTTGGAATTTGAGCGGGAATAGTAGGAACCTCTTTAAGACTATTAATTCGAGCTGAATTAAGAA
CCCCTGGATCTCTAATTGGAGATGATCAAATTTATAATACTATTGTAACAGCTCATGCCTTTATTGTAATTTTTTTTATA
GTTATACCAATTATGATTGGAGGATTTGGAAATTGACTTGTACCTTTGATATTAGGAGCCCCTGATATAGCATTTCCACG
AATAAATAATATAAGTTTTTGATTACTTCCACCCTCTTTAACTCTCCTCATTTCAAGAAGAGTTGTAGAAAATGGAGCAG
GAACTGGATGAACAGTATATCCCCNACTTTCATCTAATATTGCCCATGGAGGAAGATCTGTAGACTTAGCCATTTTTTCC
CTTCATTTAGCTGGAATCTCTTCTATTTTAGGAGCTATTAATTTTATTACAACTATTATTAATATACGATTAGATAGTTT
ATCTTTTGATCAAATACCTTTGTTTATTTGAGCTGTAGGAATTACAGCATTTTTATTATTATTATCATTACCTGTATTAG
CTGGAGCTATTACTATGTTATTAACAGACCGAAATTTAAATACATCATT------------------------------
----
>ANICK714-10|Bathytricha_leonina|10ANIC-07711|HQ950784
AACATTATATTTTATTTTTGGAATTTGAGCAGGAATAGTAGGAACCTCTTTAAGACTATTAATTCGAGCTGAATTAGGAA
CACCTGGATCTTTAATTGGAGATGATCAAATTTATAATACTATTGTAACAGCTCATGCTTTTATTATAATTTTTTTTATA
GTTATACCAATTATAATTGGAGGGTTTGGAAATTGACTTGTACCTTTAATATTAGGAGCACCTGATATAGCATTCCCACG
AATAAATAATATAAGTTTTTGATTACTTCCACCCTCTTTAACTCTCCTTATTTCAAGTAGAATTGTAGAAAATGGGGCAG
GAACTGGATGAACAGTTTACCCACCACTCTCATCTAATATTGCTCATGGAGGAAGATCTGTAGACTTAGCTATTTTTTCT
CTCCATTTGGCGGGAATCTCTTCTATTCTAGGAGCTATTAATTTTATTACAACTATTATTAATATACGATTAAATAGCTT
ATCTTTTGATCAAATACCTTTATTTATTTGAGCCGTAGGTATTACAGCATTTTTATTATTATTATCATTACCTGTATTAG
CTGGAGCTATTACTATACTATTAACAGATCGAAATTTAAATACATCATTTTTTGATCCTGCAGGAGGAGGAGATCCAATT
TTATATCAACATTTATTT---------
>ANICK715-10|Bathytricha_leonina|10ANIC-07712|KF387791
AACATTATATTTTATTTTTGGAATTTGAGCAGGAATAGTAGGAACCTCTTTAAGACTATTAATTCGAGCTGAATTAGGAA
CACCTGGATCTTTAATTGGAGATGATCAAATTTATAATACTATTGTAACAGCTCATGCTTTTATTATAATTTTTTTTATA
GTTATACCAATTATAATTGGAGGGTTTGGAAATTGACTTGTACCTTTAATATTAGGAGCACCTGATATAGCATTCCCACG
AATAAATAATATAAGTTTTTGATTACTTCCACCCTCTTTAACTCTCCTTATTTCAAGTAGAATTGTAGAAAATGGGGCAG
GAACTGGATGAACAGTTTACCCACCACTCTCATCTAATATTGCTCATGGAGGAAGATCTGTAGACTTAGCTATTTTTTCT
CTCCATTTGGCGGGAATCTCTTCTATTCTAGGAGCTATTAATTTTATTACAACTATTATTAATATACGATTAAATAGCTT
ATCTTTTGATCAAATACCTTTATTTATTTGAGCCGTAGGTATTACAGCATTTTTATTATTATTATCATTACCTGTATTAG
CTGGAGCTATTACTATACTATTAACAGATCGAAATTTAAATACATCATTTTTTGATCCTGCAGGAGGAGGAGATCCAATT
TTATATCAACATTTATTT---------
>ANICK716-10|Bathytricha_truncata|10ANIC-07713|HQ950785
AACATTATATTTCATCTTTGGAATTTGAGCAGGAATAGTAGGAACTTCTTTAAGACTATTAATTCGAGCTGAATTAGGAA
CTCCTGGATCCTTAATTGGAGATGATCAAATTTATAATACTATTGTAACAGCTCACGCCTTTATTATAATTTTTTTTATA
GTTATACCAATTATAATCGGGGGATTTGGAAATTGACTTGTACCTTTAATGTTAGGAGCACCTGATATAGCATTTCCACG
AATAAATAATATAAGTTTTTGATTACTACCCCCTTCTTTAACTCTACTTATTTCAAGAAGAGTTGTAGAAAATGGAGCAG
GAACTGGATGAACAGTATACCCCCCACTTTCATCTAATATTGCTCATAGAGGAAGATCTGTAGACTTAGCTATTTTTTCC
CTTCATTTAGCTGGAATTTCTTCTATTTTAGGAGCTATTAATTTTATTACAACTATTATTAACATACGATTAAATAATTT
ATCTTTTGATCAAATACCTTTATTTATTTGAGCTGTAGGAATTACAGCATTTTTATTATTATTATCATTACCTGTCTTAG
CTGGAGCTATTACTATATTATTAACAGATCGAAATTTAAATACATCATTTTTTGATCCTGCAGGAGGGGGAGATCCAATC
TTATACCAACATTTATTT---------
>ANICK717-10|Bathytricha_monticola|10ANIC-07714|HQ950786
AACATTATATTTTTTTTTTGGAATTTGAGCAGGAATAGTAGGAACCTCTTTAAGACTATTAATTCGAGCTGAATTAGGAA
CACCTGGATCTTTAATTAGAGATGATCAAATTTATAATACTATTGTAACAGCTCATGCTTTTATTATAATTTTTTTTATA
GTTATACCAATTATAATTGGAGGGTTTGGAAATTGACTTGTACCTTTAATATTAGGAGCACCTGATATAGCATTCCCACG
AATAAATAATATAAGTTTTTGATTACTTCCACCCTCTTTAACTCTCCTTATTTCAAGTAGAATTGTAGAAAATGGGGCAG
GAACTGGATGAACAGTTTACCCACCACTCTCATCTAATATTGCTCATGGAGGAAGATCTGTAGACTTAGCTATTTTTTCT
CTCCATTTGGCGGGAATCTCTTCTATTCTAGGAGCTATTAATTTTATTACAACTATTATTAATATACGATTAAATAGTTT
ATCTTTTGATCAAATACCTTTATTTATTTGAGCCGTAGGTATTACAGCATTTTTATTATTATTATCATTACCTGTATTAG
CTGGAGCTATTACTATACTATTAACAGATCGAAATTTAAATACATCATTTTTTGATCCTGCAGGAGGAGGAGATCCAATT
TTATATCAACATTTATTT---------

>ANICK718-10|Bathytricha_monticola|10ANIC-07715|HQ950787
AACATTATATTTTATTTTTGGAATTTGAGCAGGAATAGTAGGAACCTCTTTAAGACTATTAATTCGAGCTGAATTAGGAA
CACCTGGATCTTTAATTGGAGATGATCAAATTTATAATACTATTGTAACAGCTCATGCTTTTATTATAATTTTTTTTATA
GTTATACCAATTATAATTGGAGGGTTTGGAAATTGACTTGTACCTTTAATATTAGGAGCACCTGATATAGCATTCCCACG
AATAAATAATATAAGTTTTTGATTACTTCCACCCTCTTTAACTCTCCTTATTTCAAGTAGAATTGTAGAAAATGGGGCAG
GAACTGGATGAACAGTTTACCCACCACTCTCATCTAATATTGCTCATGGAGGAAGATCTGTAGACTTAGCTATTTTTTCT
CTCCATTTGGCGGGAATCTCTTCTATTCTAGGAGCTATTAATTTTATTACAACTATTATTAATATACGATTAAATAGTTT
ATCTTTTGATCAAATACCTTTATTTATTTGAGCCGTAGGTATTACAGCATTTTTATTATTATTATCATTACCTGTATTAG
CTGGAGCTATTACTATACTATTAACAGATCGAAATTTAAATACATCATTTTTTGATCCTGCAGGAGGAGGAGATCCAATT
TTATATCAACATTTATTT---------
>ANICK719-10|Bathytricha_monticola|10ANIC-07716|HQ950788
AACATTATATTTTATTTTTGGAATTTGAGCAGGAATAGTAGGAACCTCTTTAAGACTATTAATTCGAGCTGAATTAGGAA
CACCTGGATCTTTAATTGGAGATGATCAAATTTATAATACTATTGTAACAGCTCATGCTTTTATTATAATTTTTTTTATA
GTTATACCAATTATAATTGGAGGGTTTGGAAATTGACTTGTACCTTTAATATTAGGAGCACCTGATATAGCATTCCCACG
AATAAATAATATAAGTTTTTGATTACTTCCACCCTCTTTAACTCTCCTTATTTCAAGTAGAATTGTAGAAAATGGGGCAG
GAACTGGGTGAACAGTTTACCCACCACTCTCATCTAATATTGCTCATGGAGGAAGATCTGTAGACTTAGCTATTTTTTCT
CTCCATTTGGCGGGAATCTCTTCTATTCTAGGAGCTATTAATTTTATTACAACTATTATTAATATACGATTAAATAGTTT
ATCTTTTGATCAAATACCTTTATTTATTTGAGCCGTAGGTATTACAGCATTTTTATTATTATTATCATTACCTGTATTAG
CTGGAGCTATTACTATACTATTAACAGATCGAAATTTAAATACATCATTTTTTGATCCTGCAGGAGGAGGAGATCCAATT
TTATATCAACATTTATTT---------
>ANICK720-10|Bathytricha_phaeosticha|10ANIC-07717|HQ950789
AACATTATATTTCATTTTTGGAATTTGAGCAGGAATAGTAGGAACCTCTTTAAGACTATTAATTCGAGCTGAATTAGGAA
CTCCTGGATCTCTAATTGGAGATGACCAAATTTATAATACTATTGTAACAGCTCATGCTTTTATTATAATCTTTTTTATA
GTTATACCAATCATAATTGGAGGGTTTGGAAATTGACTTGTACCTTTAATATTAGNAGCACCTGATATAGCATTTCCACG
AATAAATAATATAAGTTTTTGATTACTTCCACCCTCTTTAACTCTCCTTATTTCAAGTAGAATTGTAGAAAATGGGGCAG
GAACTGGATGAACAGTTTATCCACCACTCTCATCTAATATTGCTCATGGAGGAAGATCTGTAGATTTAGCTATTTTTTCT
NTCCATTTGGCGGGNATCTCTTCTATTCTAGGAGCTATTAATTTTATTACAACTATTATTAATATACGATTAAATAGCTT
ATCTTTTGATCAAATACCTTTATTTATTTGAGCCGTAGGTATTACAGCATTTTTATTATTATTATCATTACCTGTATTAG
CTGGAGCTATTACTATATTATTAACAGATCGAAATTTAAACACATCATT-------------------------------
----
>ANICK721-10|Bathytricha_aethalion|10ANIC-07718|HQ950790
AACATTATATTTTATTTTTGGGATTTGAGCAGGGATAGTAGGAACTTCTTTAAGACTACTAATTCGAGCTGAACTAGGAA
CTCCTGGATCTCTGATTGGGGATGATCAAATTTATAATACTATTGTAACAGCTCATGCTTTTATTATAATTTTTTTTATG
GTTATACCAATCATAATTGGAGGATTTGGAAATTGACTTGTACCTTTAATATTAGGAGCGCCTGATATGGCATTCCCACG
AATAAATAACATAAGTTTTTGATTACTACCACCTTCTTTAACTCTTCTTATTTCAAGTAGAGTTGTAGAAAATGGAGCGG
GAACTGGATGAACAGTTTATCCACCACTCTCATCTAATATTGCTCATGGAGGAAGATCAGTGGACCTAGCTATTTTTTCC
CTCCATTTAGCTGGAATCTCTTCAATTCTAGGAGCTATTAATTTTATTACAACTATTATTAATATACGATTAAATAACTT
ATCTTTTGATCAAATACCTTTATTTATTTGAGCCGTAGGAATTACAGCTTTTTTATTATTATTATCATTACCTGTATTAG
CTGGAGCTATTACTATATTACTAACAGATCGAAATTTAAATACATCATTTTTCGATCCTGCAGGAGGGGGGGATCCAATT
TTATACCAACATTTATTT---------
>ANICR1848-11|Emmalocera_callirrhoda|11ANIC-03848|JN275875
AACTTTATATTTTATTTTTGGTATTTGATCTGGAATAATTGGTACTTCTTTAAGTCTTCTTATTCGTGCTGAATTAGGAA
CTTCAGGATCTTTAATTGGAGATGATCAAATTTATAATACTATTGTTACTAGTCATGCTTTTATTATAATTTTTTTTATA
GTTATACCTATTATAATTGGAGGGTTTGGAAATTGATTAGTTCCTTTAATATTAGGAGCTCCAGATATAGCTTTCCCCCG
AATAAATAATATAAGATTTTGACTTTTACCTCCATCCCTTACTTTACTACTTTCTAGTAGAATTGTAGAAAGTGGAGCAG
GAACAGGATGAACCGTTTATCCTCCTTTATCTTCTAATATTGCCCATAGAGGAAGTTCTGTAGACTTAGCTATTTTTTCT
TTACATTTAGCTGGAATTTCTTCTATTTTAGGAGCTATTAATTTTATTACTACTATTATTAATATAAAATTAAATGGTTT
ATCTTTTGATCAAATACCTTTATTTGTTTGAGCAGTTGGAATTACAGCCTTATTATTACTTTTATCTTTACCTGTATTAG
CAGGAGCTATTACTATATTATTAACAGATCGTAATTTAAATACTTCTTTTTTTGATCCTGCTGGAGGAGGAGATCCTATT
TTATATCAACATTTATTT---------
>ANICR1850-11|Emmalocera_callirrhoda|11ANIC-03850|JN275876
AACTTTATATTTTATTTTTGGTATTTGATCTGGAATAATTGGTACTTCTTTAAGTCTTCTTATTCGTGCTGAATTAGGAA
CTTCAGGATCTTTAATTGGGGATGATCAAATTTATAATACTATTGTTACTAGTCATGCTTTTATTATAATTTTTTTTATA
GTTATACCTATTATAATTGGAGGGTTTGGAAATTGATTAGTTCCTTTAATATTAGGAGCTCCAGATATAGCTTTCCCCCG
AATAAATAATATAAGATTTTGACTTTTACCTCCATCCCTTACTTTACTACTTTCTAGTAGAATTGTAGAAAGTGGAGCAG
GAACAGGATGAACCGTTTATCCTCCTTTATCTTCTAATATTGCCCATAGAGGAAGTTCTGTAGACTTAGCTATTTTTTCT
TTACATTTAGCTGGAATTTCTTCTATTTTAGGAGCTATTAATTTTATTACTACTATTATTAATATAAAATTAAATGGTTT

ATCTTTTGATCAAATACCTTTATTTGTTTGAGCAGTTGGAATTACAGCCTTATTATTACTTTTATCTTTACCTGTATTAG
CAGGAGCTATTACTATATTATTAACAGATCGTAATTTAAATACTTCTTTTTTGATCCTGCTGGAGGAGGAGATCCTATT
TTATATCAACATTTATTT---------
>ANICR1859-11|Emmalocera_latilimbella|11ANIC-03859|KF399599
AACTTTATATTTTATTTTTGGAATTTGATCAGGAATAGTAGGAACATCTATAAGTTTACTTATTCGAGCTGAATTAGGAA
CTCCTGGATCTTTAATTGGAGATGACCAAATTTATAATACTATTGTTACTGGTCATGCTTTTATTATAATTTTTTTTATA
GTTATACCTATTATAATTGGAGGATTTGGAAATTGATTAGTTCCTTTAATATTAGGAGCCCCAGATATAGCTTTTCCTCG
AATAAATAATATAAGATTCTGACTCTTACCCCCTTCCCTAAATTTATTAATTTTTAGAAGAATTGTAGAAAATGGAGCAG
GAACAGGATGAACAGTTTATCCCCCTTTATCCTCTAATATTGCCCATAGAGGTAGATCTGTTGATCTTGCTATTTTTTCT
TTACATTTAGCAGGAATTTCTTCTATTTTAGGAGCTATTAACTTTATTACTACTATTATTAATATAAAATTAAATGGATT
ATCATTTGATCAAATACCTTTATTTGTATGAGCTGTAGGAATTACAGCTTTATTATTACTTTTATCTTTACCAGTATTAG
CAGGAGCTATTACTATATTATTAACTGATCGAAATTTAAATACTTCTTTTTTGACCCTGCTGGAGGAGGGGATCCAATT
TTATATCAACATTTATTT---------
>ANICS064-11|Chilo_crypsimetalla|11ANIC-04064|KF391306
AACTTTATATTTTATTTTTGGAATCTGAGCAGGAATAATTGGAACATCTCTTAGTCTTTTAATTCGAGCTGAATTAGGTA
CCCCTGGATCTTTAATTGGAGATGATCAAATTTATAATACTATTGTCACTGCTCATGCCTTTATTATAATTTTTTTTATA
GTAATACCAATTATAATTGGAGGATTCGGAAATTGATTGGTTCCTTTAATATTAGGAGCTCCAGATATAGCTTTCCCACG
AATAAACAATATAAGATTTTGATTATTACCCCCCTCTTTAACTTTATTAATTTCTAGAAGAATTGTTGAAAATGGTGCTG
GAACAGGATGAACAGTATACCCCCCACTTTCATCTAATATTGCTCATGCTGGGAGATCAGTAGATTTAGCAATTTTTTCC
TTACACTTGGCAGGAATCTCTTCTATTTTAGGAGCTATTAATTTTATTACAACAATTATTAATATACGAATTAATGGATT
ATCATTTGATCAAATACCATTATTTGTGTGATCTGTCGGTATTACAGCATTATTATTATTACTTTCCTTA----------
----

>ANICS065-11|Chilo_crypsimetalla|11ANIC-04065|KF392101
AACTTTATATTTTATTTTTGGAATCTGAGCAGGAATAATTGGAACATCTCTTAGTCTTTTAATTCGAGCTGAATTAGGTA
CTCCTGGATCTTTAATTGGAGATGATCAAATTTATAATACTATTGTTACTGCTCATGCCTTTATTATAATTTTTTTTATA
GTTATACCAATTATAATTGGAGGATTTGGAAATTGATTAATCCCTTTAATATTAGGAGCTCCAGATATAGCTTTTCCACG
AATAAATAATATAAGATTTTGATTATTACCCCCCTCTTTAACTTTATTAATTTCTAGAAGAATCGTTGAAAATGGTGCTG
GAACAGGATGAACAGTATACCCCCCACTCTCATCTAATATTGCTCATGCTGGAAGATCAGTAGATTTAGCAATTTTTTCC
TTACACTTAGCTGGAATCTCCTCCATTTTAGGAGCCATTAATTTTATTACAACAATTATTAATATACGAATTAATAAATT
ATCATTTGATCAAATACCTTTATTTGTATGATCAGTTGGTATTACAGCATTATTATTATTACTTTCTTTACCTGTTTTAG
CTGGAGCTATTACTATATTATTAACTGATCGAAATTTAAATACATCTTTTTTGACCCTGCTGGAGGAGGAGATCCAATT
CTTTATCAACATTTATTT---------
>ANICS066-11|Chilo_crypsimetalla|11ANIC-04066|KF394087
AACTTTATATTTTATTTTTGGAATCTGAGCAGGAATAATTGGAACATCTCTTAGTCTTTTAATTCGAGCTGAATTAGGTA
CCCCTGGATCTTTAATTGGAGATGATCAAATTTATAATACTATTGTCACTGCTCATGCCTTTATTATAATTTTTTTTATA
GTAATACCAATTATAATTGGAGGATTCGGAAATTGATTGGTTCCTTTAATATTAGGAGCTCCAGATATAGCTTTCCCACG
AATAAACAATATAAGATTTTGATTATTACCCCCCTCTTTAACTTTATTAATTTCTAGAAGAATTGTTGAAAATGGTGCTG
GAACAGGATGAACAGTATACCCCCCACTTTCATCTAATATTGCTCATGCTGGGAGATCAGTAGATTTAGCAATTTTTTCC
TTACACTTAGCAGGAATCTCTTCTATTTTAGGAGCTATTAATTTTATTACAACAATTATTAATATACGAATTAATGGATT
ATCATTCGATCAAATACCATTATTTGTGTGATCTGTCGGTATTACAGCATTATTATTATTACTTTCCTTACCTGTTTTAG
CTGGAGCTATTACTATATTGTTAACTGATCGAAATTTAAATACATCTTTTTTGATCCTGCTGGAGGAGGTGATCCAATC
CTTTATCAACATTTATTT---------
>ANICS067-11|Chilo_crossostichus|11ANIC-04067|KF393339
AACTCTATATTTTATCTTTGGTATTTGAGCTGGAATAGTAGGAACTTCTTTAAGTTTATTAATCCGTGCTGAATTAGGAA
ATCCAGGATCATTAATTGGAGATGATCAAATTTATAATACTATTGTAACAGCTCATGCATTTATTATAATTTTTTTTATA
GTTATACCAATTATAATTGGAGGATTTGGAAATTGATTAGTTCCTTTAATATTAGGAGCTCCAGATATAGCTTTCCCACG
AATAAATAATATAAGATTTTGACTTCTTCCCCCTTCTTTAACACTTCTAATTTCTAGAAGAATTGTTGAAAATGGAGCTG
GAACTGGATGAACAGTGTACCCCCCACTTTCTTCTAATATTGCTCACGGAGGAAGCTCTGTAGATTTAGCAATTTTTTCT
CTTCATTTAGCTGGTATTTCATCTATTTTAGGTGCTATTAATTTTATTACAACTATTATCAATATACGAATTAACGGATT
ATCTTTTGATCAAATACCTTTATTTGTATGATCTGTTGGTATTACAGCTTTACTTCTTTTACTTTCTCTTCCAGTATTAG
CCGGAGCTATTACTATACTTCTAACAGATCGAAATTTAAATACTTCTTTCTTTGACCCTGCAGGAGGTGGAGACCCTATT
CTTTATCAACATTTATTT---------
>ANICS068-11|Chilo_crypsimetalla|11ANIC-04068|KF391786
AACTTTATATTTTATTTTTGGAATCTGAGCAGGAATAATTGGAACATCTCTTAGTCTTTTAATTCGAGCTGAATTAGGTA
CCCCTGGATCTTTAATTGGAGATGATCAAATTTATAATACTATTGTCACTGCTCATGCCTTTATTATAATTTTTTTTATA
GTAATACCAATTATAATTGGAGGATTCGGAAATTGATTGGTTCCTTTAATATTAGGAGCTCCAGATATAGCTTTCCCACG

AATAAACAATATAAGATTTTGATTATTACCCCCCTCTTTAACTTTATTAATTTCTAGAAGAATTGTTGAAAATGGTGCTG
GAACAGGATGAACAGTATACCCCCCACTTTCATCTAATATTGCTCATGCTGGGAGATCAGTAGATTTAGCAATTTTTTCC
TTACACTTAGCAGGAATCTCTTCTATTTTAGGAGCTATTAATTTTATTACAACAATTATTAATATACGAATTAATGGATT
ATCATTCGATCAAATACCATTATTTGTGTGATCTGTCGGTATTACAGCATTATTATTATTACTTTCCTTACCTGTTTTAG
CTGGAGCTATTACTATATTGTTAACTGATCGAAATTTAAATACATCTTTTTTTGATCCTGCTGGAGGAGGTGATCCAATC
CTTTATCAACATTTATTT---------
>ANICS074-11|Chilo_crypsimetalla|11ANIC-04074|KF392448
AACTTTATATTTTATTTTTGGAATCTGAGCAGGAATAATTGGAACATCTCTTAGTCTTTTAATTCGAGCTGAATTAGGTA
CCCCTGGATCTTTAATTGGAGATGATCAAATTTATAATACTATTGTCACTGCTCATGCCTTTATTATAATTTTTTTTATA
GTAATACCAATTATAATTGGAGGATTCGGAAATTGATTGGTTCCTTTAATATTAGGAGCTCCAGATATAGCTTTCCCACG
AATAAACAATATAAGATTTTGATTATTACCCCCCTCTTTAACTTTATTAATTTCTAGAAGAATTGTTGAAAATGGTGCTG
GAACAGGATGAACAGTATACCCCCCACTTTCATCTAATATTGCTCATGCTGGGAGATCAGTAGATTTAGCAATTTTTTCC
TTACACTTGGCAGGAATCTCTTCTATTTTAGGAGCTATTAATTTTATTACAACAATTATTAATATACGAATTAATGGATT
ATCATTTGATCAAATACCATTATTTGTGTGATCTGTCGGTATTACAGCATTATTATTATTACTTTCCTTACCTGTTTTAG
CTGGAGCTATTACTATATTGTTAACTGATCGAAATTTAAATACATCTTTTTTTGATCCCGCTGGAGGAGGTGATCCAATC
CTCTATCAACATTTATTT---------
>ANICS075-11|Chilo_crypsimetalla|11ANIC-04075|KF388910
AACTTTATATTTTATTTTTGGAATCTGAGCAGGAATAATTGGAACATCTCTTAGTCTTTTAATTCGAGCTGAATTAGGTA
CCCCTGGATCTTTAATTGGAGATGATCAAATTTATAATACTATTGTCACTGCTCATGCCTTTATTATAATTTTTTTTATA
GTAATACCAATTATAATTGGAGGATTTGGAAATTGATTGGTTCCTTTAATATTAGGAGCTCCAGATATAGCTTTCCCACG
AATAAACAATATAAGATTTTGATTATTACCCCCCTCTTTAACTTTATTAATTTCTAGAAGAATTGTTGAAAATGGTGCTG
GAACAGGATGAACAGTATACCCCCCACTTTCATCTAATATTGCTCATGCTGGGAGATCAGTAGATTTAGCAATTTTTTCC
TTACACTTGGCAGGAATCTCTTCTATTTTAGGAGCTATTAATTTTATTACAACAATTATTAATATACGAATTAATGGATT
ATCATTTGATCAAATACCATTATTTGTGTGATCTGTCGGTATTACAGCATTATTATTATTACTTTCCTTACCTGTTTTAG
CTGGAGCTATTACTATATTGTTAACTGATCGAAATTTAAATACATCTTTTTTTGATCCTGCTGGAGGAGGTGATCCAATC
CTCTATCAACATTTATTT---------
>ANICS076-11|Chilo_crypsimetalla|11ANIC-04076|KF391804
AACTTTATATTTTATTTTTGGAATCTGAGCAGGAATAATTGGAACATCTCTTAGTCTTTTAATTCGAGCTGAATTAGGTA
CCCCTGGATCTTTAATTGGAGATGATCAAATTTATAATACTATTGTCACTGCTCATGCCTTTATTATAATTTTTTTTATA
GTAATACCAATTATAATTGGAGGATTCGGAAATTGATTGGTTCCTTTAATATTAGGAGCTCCAGATATAGCTTTCCCACG
AATAAACAATATAAGATTTTGATTATTACCCCCCTCTTTAACTTTATTAATTTCTAGAAGAATTGTTGAAAATGGTGCTG
GAACAGGATGAACAGTATACCCCCCGCTTTCATCTAATATTGCTCATGCTGGGAGATCAGTAGATTTAGCAATTTTTTCC
TTACACTTGGCAGGAATCTCTTCTATTTTAGGAGCTATTAATTTTATTACAACAATTATTAATATACGAATTAATGGATT
ATCATTTGATCAAATACCATTATTTGTGTGATCTGTCGGTATTACAGCATTATTATTATTACTTTCCTTACCTGTTTTAG
CTGGAGCTATTACTATATTNTTAACTGATCGAAATTTAAATACATCTTTTTTTGATCCCGCTGGAGGAGGTGATCCAATC
CTCTATCAACATTTATTT---------
>ANICS080-11|Chilo_sp._ANIC1|11ANIC-04080|KF389933
AACTTTATATTTTATTTTTGGTATTTGAGCTGGAATAGTAGGAACTTCTTTAAGTTTATTAATTCGTGCCGAATTAGGAA
ATCCTGGGTCATTAATTGGTGATGATCAAATTTATAATACTATTGTAACAGCCCATGCATTTATTATAATTTTTTTTATA
GTTATACCAATTATAATTGGAGGATTTGGAAATTGATTAGTTCCCTTAATACTAGGGGCTCCTGATATAGCTTTCCCACG
AATAAATAATATAAGATTTTGACTTCTTCCCCCTTCTTTAACACTTCTAATTTCTAGAAGAATTGTTGAAAACGGAGCTG
GAACTGGATGAACAGTGTACCCCCCACTTTCTTCTAATATTGCTCACGGAGGAAGTTCTGTAGATTTAGCAATTTTTTCT
TTACATTTAGCTGGTATTTCATCTATTTTAGGTGCTATTAATTTTATTACTACTATTATTAATATACGAATTAATGGTCT
ATCATTTGATCAAATACCTTTATTTGTTTGATCAGTAGGAATTACTGCTTTACTTCTTTTACTTTCTTTACCTGTATTAG
CTGGAGCTATTACTATATTACTAACAGACCGAAATTTAAATACCTCTTTTTTCGATCCTGCAGGAGGTGGAGATCCTATC
CTCTATCAACACTTATTT---------
>ANICS081-11|Chilo_sp._ANIC1|11ANIC-04081|KF392703
AACTTTATATTTTATTTTTGGTATTTGAGCTGGAATAGTAGGAACTTCTTTAAGTTTATTAATTCGTGCCGAATTAGGAA
ATCCTGGGTCATTAATTGGTGATGATCAAATTTATAATACTATTGTAACAGCCCATGCATTTATTATAATTTTTTTTATA
GTTATACCAATTATAATTGGAGGATTTGGAAATTGATTAGTTCCCTTAATACTAGGGGCTCCTGATATAGCTTTCCCACG
AATAAATAATATAAGATTTTGACTTCTTCCCCCTTCTTTAACACTTCTAATTTCTAGAAGAATTGTTGAAAACGGAGCTG
GAACTGGATGAACAGTGTACCCCCCACTTTCTTCTAATATTGCTCACGGAGGAAGTTCTGTAGATTTAGCAATTTTTTCT
TTACATTTAGCTGGTATTTCATCTATTTTAGGTGCTATTAATTTTATTACTACTATTATTAATATACGAATTAATGGTCT
ATCATTTGATCAAATACCTTTATTTGTTTGATCAGTAGGAATTACTGCTTTACTTCTTTTACTTTCTTTACCTGTATTAG
CTGGAGCTATTACTATATTACTAACAGACCGAAATTTAAATACTTCTTTTTTTGATCCTGCGGGAGGTGGAGATCCTATT
CTCTATCAACACTTATTT---------
>ANICS877-11|Scirpophaga_imparellus|11ANIC-04877|JN278988

AACTTTATATTTTATTTTTGGTATTTGAGCTGGAATAGTGGGGACTTCTTTAAGTTTATTAATTCGAGCTGAATTAGGAA
CACCAGGATCATTAATTGGGGATGATCAAATTTATAATACTATTGTTACAGCCCATGCTTTTATTATAATTTTTTTTATA
GTAATACCTATTATAATTGGGGGATTCGGAAATTGACTAGTTCCCCTAATATTAGGAGCCCCTGATATAGCTTTTCCACG
TATAAATAATATAAGTTTTTGATTATTACCTCCCTCTCTTACTCTTTTAATTTCAAGAAGAATTGTTGAAAATGGAGCAG
GAACAGGATGAACTGTTTACCCCCCTTTATCCTCTAATATTGCCCATGGAGGAACATCTGTAGATCTAGCTATTTTTTCC
TTACATTTAGCGGGAATTTCATCTATTTTAGGAGCTATTAATTTTATTACAACTATTATTAATATACGAATTAATGGATT
ATCTTTTGATCAAATACCTTTATTTGTCTGAGCTGTAGGTATTACAGCACTTCTTTTACTTTTATCTTTACCTGTGTTAG
CTGGAGCTATTACTATACTACTAACAGATCGAAATTTAAATACATCTTTTTTCGACCCAGCAGGTGGGGGAGACCCAATT
CTTTATCAACATTTATTT---------
>ANICS879-11|Scirpophaga_imparellus|11ANIC-04879|JN278989
AACTTTATATTTTATTTTTGGTATTTGAGCTGGAATAGTGGGGACTTCTTTAAGTTTATTAATTCGAGCTGAATTAGGAA
CACCAGGATCATTAATTGGGGATGATCAAATTTATAATACTATTGTTACAGCCCATGCTTTTATTATAATTTTTTTTATA
GTAATACCTATTATAATTGGGGGATTCGGAAATTGACTAGTTCCCCTAATATTAGGAGCCCCTGATATAGCTTTTCCACG
TATAAATAATATAAGTTTTTGATTATTACCTCCCTCTCTTACTCTTTTAATTTCAAGAAGAATTGTTGAAAATGGAGCAG
GAACAGGATGAACTGTTTACCCCCCTTTATCCTCTAATATTGCCCATGGAGGAACATCTGTAGATCTAGCTATTTTTTCC
TTACATTTAGCGGGAATTTCATCTATTTTAGGAGCTATTAATTTTATTACAACTATTATTAATATACGAATTAATGGATT
ATCTTTTGATCAAATACCTTTATTTGTCTGAGCTGTAGGTATTACAGCACTTCTTTTACTTTTATCTTTACCTGTGTTAG
CTGGAGCTATTACTATACTACTAACAGATCGAAATTTAAATACATCTTTTTTCGACCCAGCAGGTGGGGGAGACCCAATT
CTTTATCAACATTTATTT---------
>ANICS882-11|Scirpophaga_nivella|11ANIC-04882|KF389935
AACTTTATATTTCATTTTTGGAATCTGAGCTGGTATAGTAGGAACTTCTTTAAGATTATTAATTCGAGCTGAATTAGGAA
CTCCAGGATCTTTAATTGGAGATGATCAAATTTATAATACCATTGTTACAGCTCATGCTTTTATTATAATTTTTTTTATA
GTAATACCAATTATAATTGGAGGATTTGGAAATTGACTTGTTCCTTTAATATTAGGAGCTCCTGATATAGCTTTCCCCCG
TATAAATAATATAAGATTTTGATTATTACCCCCCTCATTAACTCTACTAATTTCAAGAAGAATTGTAGAAAATGGTGCAG
GAACAGGATGAACAGTATACCCTCCCCTATCATCAAATATTGCCCATGGGGGAACTTCTGTAGATTTAGCTATTTTTTCT
TTACATCTTGCAGGAATTTCCTCTATTTTAGGAGCTATTAACTTTATTACCACTATTATTAATATACGAATTAATGGCTT
AACATTTGATCAAATACCCCTCTTTGTTTGAGCTGTTGGAATTACAGCCCTTCTTTTGCTCCTCTCATTA----------
----

>ANICS883-11|Scirpophaga_percna|11ANIC-04883|KF389043
AACTTTATATTTTATTTTTGGAATTTGAGCTGGAATAGTAGGAACTTCTTTAAGTTTATTAATTCGAGCTGAATTAGGAA
CCCCAGGATCATTAATTGGAGATGATCAAATTTATAACACTATTGTAACAGCCCATGCTTTTATTATAATTTTTTTTATA
GTTATACCTATTATAATTGGAGGTTTTGGAAATTGACTAGTTCCTCTAATATTAGGAGCTCCTGATATAGCTTTCCCCCG
ATTAAATAATATAAGTTTTTGATTATTACCCCCCTCTCTTACCCTTTTAATTTCAAGAAGAATTGTTGAAAATGGGGCAG
GAACAGGATGAACTGTTTACCCCCCACTATCCTCTAACATCGCCCATGGGGGTACATCAGTAGATTTAGCTATTTTTTCC
CTTCATTTAGCTGGAATCTCATCTATTCTAGGAGCTATTAATTTTATTACTACTATTATCAATATACGAATTAATGGACT
ATCTTTTGATCAAATACCTTTATTTGTTTGAGCTGTAGGAATTACAGCTCTTCTTTTACTCTTATCTTTA----------
----

>ANICW320-11|Tetramoera_gracilistria|11ANIC-12320|KF403414
AACACTATATTTTATTTTCGGAATTTGATCAGGAATAATTGGTACATCTTTAAGATTATTAATTCGAGCTGAATTAGGAA
ACCCCGGATCTTTAATTGNAGATGATCAAATTTATAATACTATTGTAACTGCTCATGCTTTTATTATAATTTTTTTTATA
GTAATGCCTATTATAATTGGAGGATTTGGAAATTGATTAGTTCCTTTAATATTAGGAGCTCCTGATATAGCTTTCCCTCG
TATAAATAACATAAGATTTTGATTACTTCCCCCCTCAATCATATTACTAATCTCAAGAAGAATTGTAGAAAATGGAGCAG
GAACTGGATGAACAGTTTACCCCCCACTATCATCCAATATTGCTCACAGAGGTAGTTCTGTAGATTTAGCTATTTTTTCT
TTACATTTAGCTGGAATTTCTTCTATTTTAGGAGCTGTAAATTTTATTACCACTATTATTAATATACGACCAAATAATAT
AAGATTAGATCAAATACCATTATTTGTTTGAGCTGTTGGTATTACAGCTCTATTATTACTTTTATCTTTA----------
----

>ANICW321-11|Tetramoera_gracilistria|11ANIC-12321|KF405514
AACATTATATTTTATTTTTGGAATTTGATCAGGAATAATTGGTACATCTTTAAGATTATTAATTCGAGCTGAATTAGGAA
ATCCTGGATCTTTAATTGGAGATGATCAAATTTATAATACTATTGTAACCGCTCATGCTTTTATTATAATTTTTTTTATA
GTTATACCTATTATAATTGGAGGATTTGGAAATTGATTAGTTCCTTTAATATTAGGAGCTCCTGATATAGCTTTTCCTCG
TATAAATAATATAAGATTCTGATTACTTCCTCCTTCCATTATATTATTAATTTCAAGAAGAATTGTAGAAAATGGAGCAG
GAACTGGATGAACAGTTTACCCCCCACTATCATCCAATATTGCTCATAGAGGTAGTTCCGTAGATTTAGCTATTTTTTCT
TTACATCTAGCTGGAATTTCCTCCATTTTAGGAGCTGTAAACTTTATTACCACTATTATTAATATACGACCAAATAATAT
AAGACTAGATCAAATACCATTATTTGTTTGAGCTGTCGGTATTACAGCATTATTATTACTTTTATCTTTA----------

----

>ANICW322-11|Tetramoera_gracilistria|11ANIC-12322|KF398638
AACATTATATTTTATTTTTGGAATTTGATCAGGAATAATTGGTACATCTTTAAGATTATTAATTCGAGCTGAATTAGGAA
ATCCTGGATCTTTAATTGGAGATGATCAAATTTATAATACTATTGTAACCGCTCATGCTTTTATTATAATTTTTTTTATA
GTTATACCTATTATAATTGGAGGATTTGGAAATTGATTAGTTCCTTTAATATTAGGAGCTCCTGATATAGCTTTTCCTCG
TATAAATAATATAAGATTCTGATTACTTCCTCCTTCAATTATATTATTAATTTCAAGAAGAATTGTAGAAAATGGAGCAG
GAACTGGATGAACAGTTTACCCCCCACTATCATCCAATATTGCTCATAGAGGTAGTTCCGTAGATTTAGCTATTTTTTCT
TTACATCTAGCTGGAATTTCTTCCATTTTAGGAGCTGTAAACTTTATTACCACTATTATTAATATACGACCAAATAATAT
AAGACTAGATCAAATACCATTATTTGTTTGAGCTGTCGGTATTACAGCCTTATTATTACTTTTATCTTTACCAGTATTAG
CAGGTGCTATTACTATACTATTAACAGATCGTAATTTAAATACTTCATTTTTTGATCCTGCAGGAGGAGGTGACCCTATT
TTATATCAACATTTATTT---------
>ANICW323-11|Tetramoera_gracilistria|11ANIC-12323|KF400771
AACATTATATTTTATTTTTGGAATTTGATCAGGAATAATTGGTACATCTTTAAGATTATTAATTCGAGCTGAATTAGGAA
ATCCTGGATCTTTAATTGGAGATGATCAAATTTATAATACTATTGTAACCGCTCATGCTTTTATTATAATTTTTTTTATA
GTTATACCTATTATAATTGGAGGATTTGGAAATTGATTAGTTCCTTTAATATTAGGAGCTCCTGATATAGCTTTTCCTCG
TATAAATAATATAAGATTCTGATTACTTCCTCCTTCCATTATATTATTAATTTCAAGAAGAATTGTAGAAAATGGAGCAG
GAACTGGATGAACAGTTTACCCCCCACTATCATCCAATATTGCTCATAGAGGTAGTTCCGTAGATTTAGCTATTTTTTCT
TTACATCTAGCTGGAATTTCTTCCATTTTAGGAGCTGTAAACTTTATTACCACTATTATTAATATACGACCAAATAATAT
AAGACTAGATCAAATACCATTATTTGTTTGAGCTGTCGGTATTACAGCCTTATTATTACTTTTATCTTTA----------
----

>BBLOB396-11|Diatraea_evanescens|BIOUG01396-B04|
AACTTTATATTTTATTTTTGGAATTTGAGCTGGAATACTGGGAACATCTTTAAGTTTATTAATTCGTGCAGAATTAGGTA
CAACTAATTCTTTAATTGGAGATGATCAAATCTACAATACAATTGTTACAGCTCATGCATTTATTATAATTTTTTTTATA
GTTATACCCATTATAATTGGAGGATTTGGAAACTGATTAGTACCATTAATATTAGGAGCTCCTGATATAGCTTTCCCTCG
AATAAATAATATAAGATTTTGATTACTACCCCCATCTTTAACTTTATTAATTTCTAGAAGAATTGTTGAAAATGGAGCTG
GAACAGGATGAACAGTTTACCCCCCACTTTCATCAAATATTGCTCATGGTGGTAGATCTGTAGACCTAGCAATTTTTTCA
TTACATTTAGCTGGAATCTCATCAATTTTAGGAGCTATTAATTTTATTACCACAATTATTAATATACGAATTAATGGATT
ATCATTCGATCAAATACCTCTATTTGTATGATCAGTAGGTATTACAGCCTTATTATTACTTTTATCTTTACCTGTTTTAG
CTGGAGCTATTACCATATTATTAACAGATCGAAACTTAAATACTTCCTTTTTCGACCCAGCTGGAGGGGGAGATCCAATT
TTATATCAACATTTATTT---------
>BBLOB420-11|Diatraea_evanescens|BIOUG01396-D04|
AACTTTATATTTTATTTTTGGAATTTGAGCTGGAATACTGGGAACATCTTTAAGTTTATTAATTCGTGCAGAATTAGGTA
CAACTAATTCTTTAATTGGAGATGATCAAATCTACAATACAATTGTTACAGCTCATGCATTTATTATAATTTTTTTTATA
GTTATACCCATTATAATTGGAGGATTTGGAAACTGATTAGTACCATTAATATTAGGAGCTCCTGATATAGCTTTCCCTCG
AATAAATAATATAAGATTTTGATTACTACCCCCATCTTTAACTTTATTAATTTCTAGAAGAATTGTTGAAAATGGAGCTG
GAACAGGATGAACAGTTTACCCCCCACTTTCATCAAATATTGCTCATGGTGGTAGATCTGTAGACCTAGCAATTTTTTCA
TTACATTTAGCTGGAATCTCATCAATTTTAGGAGCTATTAATTTTATTACCACAATTATTAATATACGAATTAATGGATT
ATCATTCGATCAAATACCTCTATTTGTATGATCAGTAGGTATTACAGCCTTATTATTACTTTTATCTTTACCTGTTTTAG
CTGGAGCTATTACCATATTATTAACAGATCGAAACTTAAATACTTCCTTTTTCGACCCAGCTGGAGGGGGAGATCCAATT
TTATATCAACATTTATTT---------
>BBLOC286-11|Eoreuma_loftini|BIOUG01455-A06|
AACTTTATATTTTATTTTTGGAATTTGAGCTGGAACAGTAGGAACATCTTTAAGTTTATTAATTCGAGCTGAATTAGGAA
ACCCAGGCTCTTTAATTGGAGATGATCAAATTTATAATACTATTGTTACAGCCCATGCATTTATTATAATTTTTTTTATA
GTAATACCTATTATAATTGGGGGATTTGGAAACTGATTAGTACCTTTAATATTAGGAGCTCCTGATATAGCATTCCCACG
AATAAATAATATAAGATTTTGATTATTACCTCCATCTTTAACACTACTAATTTCAAGAAGTATTGTTGAAAATGGAGCTG
GAACAGGATGAACAGTTTATCCCCCTCTTTCTTCTAATATTGCTCATGGAGGTAGATCTGTAGATCTAGCAATTTTTTCT
CTTCATTTAGCTGGAATTTCCTCAATTTTAGGAGCTATTAATTTTATTACAACAATTATTAATATACGAATTAATAATTT
ATCCTTTGATCAAATACCTTTATTTGTTTGATCAGTAGGTATTACAGCTTTACTTCTTCTTCTTTCTTTACCTGTATTAG
CTGGAGCTATTACTATATTATTAACAGATCGAAATTTAAATACTTCCTTTTTTGATCCTGCAGGGGGTGGAGATCCAATT
CTTTATCAACATTTATTT---------
>BBLOC1454-11|Diatraea_evanescens|BIOUG01545-A06|
AACTTTATATTTTATTTTTGGAATTTGAGCTGGAATACTGGGAACATCTTTAAGTTTATTAATTCGTGCAGAATTAGGTA
CAACTAATTCTTTAATTGGAGATGATCAAATTTACAATACAATTGTTACAGCTCATGCATTTATTATAATTTTTTTTATA
GTTATACCCATTATAATTGGGGGATTTGGAAACTGATTAGTACCATTAATATTAGGAGCTCCTGATATAGCTTTCCCTCG
AATAAATAATATAAGATTTTGATTACTACCCCCATCTTTAACTTTATTAATTTCTAGAAGAATTGTTGAAAATGGAGCTG

GAACAGGATGAACAGTTTACCCCCCACTTTCATCAAATATTGCTCATGGTGGTAGATCTGTAGACCTAGCAATTTTTTCA
TTACATTTAGCTGGAATCTCATCAATTTTAGGAGCTATTAATTTTATTACCACAATTATTAATATACGAATTAATGGATT
ATCATTCGATCAAATACCTCTATTTGTATGATCAGTAGGTATTACAGCCTTATTATTACTTTTATCTTTACCTGTTTTAG
CTGGAGCTATTACCATATTATTAACAGATCGAAACTTAAATACTTCCTTTTTCGACCCAGCTGGAGGGGGAGATCCAATT
TTATATCAACATTTATTT---------
>BBLOC1560-11|Diatraea_saccharalis|BIOUG01546-B05|
AACTTTATATTTTATTTTTGGAATTTGAGCAGGAATGTTAGGAACTTCCTTAAGCTTATTAATTCGAGCAGAATTAGGAA
CATCTAACTCTTTAATTGGAGATGATCAAATTTATAACACAATTGTAACAGCTCATGCTTTCATTATAATTTTTTTTATA
GTTATACCTATTATAATTGGAGGATTTGGAAATTGATTAGTACCTTTAATGTTAGGGGCCCCCGATATAGCTTTCCCACG
AATAAATAATATAAGATTTTGACTATTACCCCCATCTTTAACTCTACTAATTTCTAGAAGAATTGTAGAAAATGGAGCAG
GGACAGGATGAACAGTTTATCCCCCATTATCATCCAATATTGCCCATGGAGGAAGATCTGTGGATTTAGCAATTTTCTCT
TTACATTTAGCTGGAATTTCCTCAATTTTAGGGGCTATCAATTTCATCACTACCATTATTAATATACGAATTAATAATCT
ATCATTTGATCAAATACCTTTATTTATTTGATCAGTGGGTATTACAGCATTACTTTTATTACTTTCTTTACCGGTATTAG
CTGGAGCCATTACTATATTACTAACAGATCGAAATTTAAATACCTCCTTCTTTGATCCAGCAGGGGGGGGAGATCCTATT
CTATATCAACATTTATTT---------
>BBLOC1565-11|Diatraea_saccharalis|BIOUG01546-B10|
AACTTTATATTTTATTTTTGGAATTTGAGCAGGAATGTTAGGAACTTCCTTAAGCTTATTAATTCGAGCAGAATTAGGAA
CATCTAACTCTTTAATTGGAGATGATCAAATTTATAACACAATTGTAACAGCTCATGCTTTCATTATAATTTTTTTTATA
GTTATACCTATTATAATTGGAGGATTTGGAAATTGATTAGTACCTTTAATGTTAGGGGCCCCCGATATAGCTTTCCCACG
AATAAATAATATAAGATTTTGACTATTACCCCCATCTTTAACTCTACTAATTTCTAGAAGAATTGTAGAAAATGGAGCAG
GGACAGGATGAACAGTTTATCCCCCATTATCATCCAATATTGCCCATGGGGGAAGATCTGTGGATTTAGCAATTTTCTCT
TTACATTTAGCTGGAATTTCCTCAATTTTAGGGGCTATCAATTTCATCACTACCATTATTAATATACGAATTAATAATCT
ATCATTTGATCAAATACCTTTATTTATTTGATCAGTGGGTATTACAGCATTACTTTTATTACTTTCTTTACCGGTATTAG
CTGGAGCCATTACTATATTACTAACAGATCGAAATTTAAATACCTCCTTCTTTGATCCAGCAGGGGGGGGAGATCCTATT
CTATATCAACATTTATTT---------
>BBLOD166-11|Diatraea_saccharalis|BIOUG01552-E05|
AACTTTATATTTTATTTTTGGAATTTGAGCAGGAATGTTAGGAACTTCCTTAAGCTTATTAATTCGAGCAGAATTAGGAA
CATCTAACTCTTTAATTGGAGATGATCAAATTTATAACACAATTGTAACAGCTCATGCTTTCATTATAATTTTTTTTATA
GTTATACCTATTATAATTGGAGGATTTGGAAATTGATTAGTACCTTTAATGTTAGGGGCCCCCGATATAGCTTTCCCACG
AATAAATAATATAAGATTTTGACTATTACCCCCATCTTTAACTCTACTAATTTCTAGAAGAATTGTAGAAAATGGAGCAG
GGACAGGATGAACAGTTTATCCCCCATTATCATCCAATATTGCCCATGGAGGAAGATCTGTGGATTTAGCAATTTTCTCT
TTACATTTAGCTGGAATTTCCTCAATTTTAGGGGCTATCAATTTCATCACTACCATTATTAATATACGAATTAATAATCT
ATCATTTGATCAAATACCTTTATTTATTTGATCAGTGGGTATTACAGCATTACTTTTATTACTTTCTTTACCGGTATTAG
CTGGAGCCATTACTATATTACTAACAGATCGAAATTTAAATACCTCCTTCTTTGATCCAGCAGGGGGGGGAGATCCTATT
CTATATCAACATTTATTT---------
>BBLOD664-11|Eoreuma_loftini|BIOUG01566-G04|
AACTTTATATTTTATTTTTGGAATTTGAGCTGGAACAGTAGGAACATCTTTAAGTTTATTAATTCGAGCTGAATTAGGAA
ACCCAGGCTCTTTAATTGGAGATGATCAAATTTATAATACTATTGTTACAGCCCATGCATTTATTATAATTTTTTTTATA
GTAATACCTATTATAATTGGGGGATTTGGAAACTGATTAGTACCTTTAATATTAGGAGCTCCTGATATAGCATTCCCACG
AATAAATAATATAAGATTTTGATTATTACCCCCATCTTTAACACTACTAATTTCAAGAAGTATTGTTGAAAATGGAGCTG
GAACAGGATGAACAGTTTATCCCCCTCTTTCTTCTAATATTGCTCATGGAGGTAGATCTGTAGATCTAGCAATTTTTTCT
CTTCATTTAGCTGGAATTTCCTCAATTTTAGGAGCTATTAATTTTATTACAACAATTATTAATATACGAATTAATAATTT
ATCCTTTGATCAAATACCTTTATTTGTTTGATCAGTAGGTATTACAGCTTTACTTCTTCTTCTTTCTTTACCAGTATTAG
CTGGAGCTATTACTATATTATTAACAGATCGAAATTTAAATACTTCCTTTTTTGATCCTGCAGGGGGTGGAGATCCAATT
CTTTATCAACATTTATTT---------
>BBLOD1467-11|Eoreuma_densella|BIOUG01828-B12|
AACATTATATTTTATTTTTGGAATTTGAGCTGGAATAGTAGGAACATCTCTTAGTTTATTAATTCGAGCTGAATTAGGAA
ATCCTGGTTCCTTAATTGGAGATGATCAAATTTATAATACTATTGTCACAGCACATGCATTTATTATAATTTTTTTTATA
GTTATACCTATTATAATTGGAGGATTTGGAAATTGATTAGTTCCTTTAATATTAGGAGCCCCTGATATAGCATTCCCCCG
AATAAATAATATAAGATTTTGATTATTACCCCCCTCATTAACTTTATTAATTTCAAGTAGTATTGTCGAAAATGGTGCCG
GTACAGGATGAACTGTATACCCCCCTCTTTCCTCAAATATTGCCCATGGGGGGAGATCTGTTGACTTAGCAATTTTTTCT
TTACATTTAGCTGGAATTTCATCAATCTTAGGAGCTATCAACTTTATTACAACAATTATTAATATACGAATTAATAATTT
ATCATTTGATCAAATACCTTTATTTGTTTGATCAGTAGGAATTACAGCATTACTTCTCCTCCTTTCATTACCTGTTTTAG
CAGGAGCTATTACTATATTATTAACAGATCGAAATCTTAATACATCATTTTTTGACCCTGCTGGGGGAGGAGATCCAATT
CTCTACCAACATTTATTT---------
>BBLOE212-11|Diatraea_evanescens|BIOUG01903-A09|
AACTTTATATTTTATTTTTGGAATTTGAGCTGGAATACTGGGAACATCTTTAAGTTTATTAATTCGTGCAGAATTAGGTA

CAACTAATTCTTTAATTGGAGATGATCAAATTTACAATACAATTGTTACAGCTCATGCATTTATTATAATTTTTTTTATA
GTTATACCCATTATAATTGGGGGATTTGGAAACTGATTAGTACCATTAATATTAGGAGCTCCTGATATAGCTTTCCCTCG
AATAAATAATATAAGATTTTGATTACTACCCCCATCTTTAACTTTATTAATTTCTAGAAGAATTGTTGAAAATGGAGCTG
GAACAGGATGAACAGTTTACCCCCCACTTTCATCAAATATTGCTCATGGTGGTAGATCTGTAGACCTAGCAATTTTTTCA
TTACATTTAGCTGGAATCTCATCAATTTTAGGAGCTATTAATTTTATTACCACAATTATTAATATACGAATTAATGGATT
ATCATTCGATCAAATACCTCTATTTGTATGATCAGTAGGTATTACAGCCTTATTATTACTTTTATCTTTACCTGTTTTAG
CTGGAGCTATTACCATATTATTAACAGATCGAAACTTAAATACTTCCTTTTTCGACCCAGCTGGAGGAGGAGATCCAATT
TTATATCAACATTTATTT---------
>BBLOE1921-12|Diatraea_lisetta|BIOUG01994-D12|
AACTTTATATTTTATTTTTGGAATTTGAGCTGGAATATTAGGAACATCATTAAGTTTATTAATTCGTGCTGAATTAGGTA
CACCTAATTCTCTAATTGGAGATGATCAAATTTATAATACAATTGTTACAGCTCACGCATTTATTATAATTTTTTTTATA
GTTATACCTATTATAATTGGAGGATTTGGAAATTGATTAGTTCCTTTAATATTAGGAGCTCCTGATATAGCTTTCCCTCG
AATAAATAACATAAGATTTTGATTATTACCCCCATCTTTAACATTATTAATTTCTAGAAGAATTGTTGAAAATGGAGCTG
GAACAGGATGAACAGTATACCCCCCCCTTTCATCTAATATTGCTCATGGTGGAAGATCTGTAGATTTAGCAATTTTCTCC
CTTCATTTAGCTGGAATTTCATCAATTTTAGGAGCTATTAATTTTATTACTACAATTATTAATATACGAATTAATAGATT
ATCATTTGATCAAATACCACTTTTTATTTGATCAGTAGGTATTACAGCTTTATTATTATTACTTTCTTTACCTGTATTAG
CTGGAGCTATTACAATATTATTAACTGATCGAAATTTAAATACTTCTTTTTTTGATCCAGCTGGAGGAGGAGATCCTATT
TTATATCAACATCTTTTT---------
>BBLOE1922-12|Diatraea_lisetta|BIOUG01994-E01|
AACTTTATATTTTATTTTTGGAATTTGAGCTGGAATATTAGGAACATCATTAAGTTTATTAATTCGTGCTGAATTAGGTA
CACCTAATTCTCTAATTGGAGATGATCAAATTTATAATACAATTGTTACAGCTCACGCATTTATTATAATTTTTTTTATA
GTTATACCTATTATAATTGGAGGATTTGGAAATTGATTAGTTCCTTTAATATTAGGAGCTCCTGATATAGCTTTCCCTCG
AATAAATAACATAAGATTTTGATTATTACCCCCATCTTTAACATTATTAATTTCTAGAAGAATTGTTGAAAATGGAGCTG
GAACAGGATGAACAGTATACCCCCCCCTTTCATCTAATATTGCTCATGGTGGAAGATCTGTAGATTTAGCAATTTTCTCC
CTTCATTTAGCTGGAATTTCATCAATTTTAGGAGCTATTAATTTTATTACTACAATTATTAATATACGAATTAATAGATT
ATCATTTGATCAAATACCACTTTTTATTTGATCAGTAGGTATTACAGCTTTATTATTATTACTTTCTTTACCTGTATTAG
CTGGAGCTATTACAATATTATTAACTGATCGAAATTTAAATACTTCTTTTTTTGATCCAGCTGGAGGAGGAGATCCTATT
TTATATCAACATCTTTTT---------
>BIPR003-13|Chilo_auricilius|ChiloCT1|KF371520
-----------------T-------------------TGAGAACATCCCTAAGACTTTTAATTCGTGCTGAATTAGGAA
CTCCAGGATCATTAATTGGAGATGATCAAATTTACAATACTATTGTTACAGCTCATGCATTTATTATAATTTTTTTTATA
GTTATACCAATTATAATTGGAGGCTTTGGTAATTGATTGGTACCATTAATACTAGGGGCTCCTGATATAGCCTTCCCTCG
AATAAATAATATAAGATTTTGATTATTGCCCCCATCATTAACATTATTAATTTCTAGAAGAATTGTAGAAAATGGAGCTG
GAACAGGATGAACGGTTTACCCCCCCCTTTCATCAAATATTGCCCATGGAGGAAGTTCTGTAGATTTAGCTATTTTTTCC
CTTCATTTAGCTGGTATTTCCTCAATTTTAGGAGCTATTAATTTTATTACAACAATTATTAATATACGAATTAATAAACT
ATCATTTGATCAATTACCATTATTTGTTTGATCTGTTGGTATTACAGCCTTATTATTATTACTTTCATTACCAGTACTAG
CTGGAGCTATTACTATACTTTTAACTGATCGAAACTTAAATACATCTTTTTTTGACCCTGCCGGAGGGGGAGACCCAATT
CTTTATCAACATTTATTTTGATTTTTT
>BIPR004-13|Chilo_auricilius|ChiloCT2|KF371521

--------------TTGGAGATGATCAAATTTACAATACTATTGTTACAGCTCATGCATTTATTATAATTTTTTTTATA
GTTATACCAATTATAATTGGAGGCTTTGGTAATTGATTGGTACCATTAATACTAGGGGCTCCTGATATAGCCTTCCCTCG
AATAAATAATATAAGATTTTGATTATTGCCCCCATCATTAACATTATTAATTTCTAGAAGAATTGTAGAAAATGGAGCTG
GAACAGGATGAACGGTTTACCCCCCCCTTTCATCAAATATTGCCCATGGAGGAAGTTCTGTAGATTTAGCTATTTTTTCC
CTTCATTTAGCTGGTATTTCCTCAATTTTAGGAGCTATTAATTTTATTACAACAATTATTAATATACGAATTAATAAACT
ATCATTTGATCAATTACCATTATTTGTTTGATCTGTTGGTATTACAGCCTTATTATTATTACTTTCATTGCCAGTACTAG
CTGGAGCTATTACTATACTTTTAACTGATCGAAACTTAAATACATCTTTTTTTGACCCTGCCGGAGGGGGAGACCCAATT
CTTTATCAACATTTATTTTGATTTTTT
>BIPR009-13|Sesamia_inferens|PSBCT2|KF371530

------------------------------------ATTGTCACTACTCATGCTTTTATTATAATTTTTTTTATA
GTTTTCCCAATTTTTATTGTATGTTTTGCACATTGACTTGTACCTTTAATATTAGGAGCTCCTGATATAGCTTTTCCACG
AATAAATAATATAAGATTTTGATTATTACCCCCCTCTTTAACTCTTTTAATTTCAAGCAGAATTGTAGAAAATGGAGCAG
GAACTGGGTGAACAGTGTACCCCCCACTTTCATCTAATATTGCCCATGGAGGAAGATCAGTAGATTTAGCTATTTTTTCC
CTTCATTTAGCTGGTATTTCATCTATTTTAGGAGCTATTAATTTTATTACAACAATTATCAATATACGATTAAATAGTTT
ATCTTTTGACCAAATACCTCTATTTATTTGAGCTGTTGGAATTACTGCATTTTTATTATTACTATCTTTACCTGTATTAG
CAGGGGCTATTACAATATTATTAACAGATCGAAATTTAAATACATCATTCTTTGACCCCGCAGGGGGAGGTGACCCTATT

-TATATCAACATTTATTT-GATTTTTT
>BIPR012-13|Scirpophaga_innotata|WSBCT1|KF371528
AACTTTATATTTCATTTTTGGAATTTGAGCTGGTATAGTGGGAACTTCCTTAAGATTATTAATTCGGGCTGAATTAGGAA
CTCCAGGGTCTTTAATTGGAGATGATCAAATCTATAATACAATTGTTACAGCTCATGCTTTTATTATAATTTTTTTTATA
GTGATACCAATTATAATTGGAGGATTTGGAAATTGACTTGTTCCTTTAATATTAGGAGCTCCTGATATAGCTTTCCCCCG
TATAAATAATATAAGATTTTGATTATTACCCCCCTCATTAACTCTTTTAATTTCAAGAAGAATTGTAGAAAATGGTGCAG
GAACAGGATGAACAGTATATCCCCCCTTATCATCAAATATTGCTCATGGAGGTACTTCTGTAGATTTAGCTATTTTTTCT
TTACATCTTGCAGGAATTTCCTCTATTTTAGGAGCTATTAATTTTATCACCACTATTATTAACATACGAATTAATGGCTT
AACATTTGATCAAATACCACTTTTTGTTTGAGCCGTTGGAATTACAGCCCTTCTTTTACTCCTTTCATTACCTGTATTAG
CTGGAGCCATTACTATATTATTAACTGACCGAAATTTAAATACCTCTTTTTTTGATCCGACTGGAGGAGGAGATCCAATC
GGTTATCAAACATTTT-----------
>BIPR013-13|Scirpophaga_innotata|WSBCT2|KF371529
----------------------ATTTGAGCTGGTATATTGAGAACTTCCTTAAGATTATTAATTCGGGCTGAATTAGGAA
CTCCAGGGTCTTTAATTGGAGATGATCAAATCTATAATACAATTGTTACAGCTCATGCTTTTATTATAATTTTTTTTATA
GTGATACCAATTATAATTGGAGGATTTGGAAATTGACTTGTTCCTTTAATATTAGGAGCTCCTGATATAGCTTTCCCCCG
TATAAATAATATAAGATTTTGATTATTACCCCCCTCATTAACTCTTTTAATTTCAAGAAGAATTGTAGAAAATGGTGCAG
GAACAGGATGAACAGTATATCCCCCCTTATCATCAAATATTGCTCATGGAGGTACTTCTGTAGATTTAGCTATTTTTTCT
TTACATCTTGCAGGAATTTCCTCTATTTTAGGAGCTATTAATTTTATCACCACTATTATTAACATACGAATTAATGGCTT
AACATTTGATCAAATACCACTTTTTGTTTGAGCCGTTGGAATTACAGCCCTTCTTTTACTCCTTTCATTACCTGTATTAG
CTGGAGCCATTACTATATTATTAACTGACCGAAATTTAAATACCTCTTTTTTTGATCCAGCTGGAGGAGGAGATCCAATC
CTTTATCAACATTTATTT-GATTTTTT
>BIPR015-13|Scirpophaga_incertulas|YSBCT2|KF371526
TACTTTATATTTTATTTTTGGAATTTGAGCTGGTATAGTAGGAACTTCTTTAAGCTTACTTATTCGAGCTGAATTAGGAA
CTTCTGGATCCTTAATTGGAGATGATCAAATCTATAATACTATTGTCACAGCCCATGCCTTTATTATAATTTTTTTTATA
GTTATACCCATTATAATTGGAGGATTTGGAAATTGATTAGTCCCCCTAATATTAGGAGCCCCAGATATAGCTTTTCCCCG
ATTAAATAACATAAGATTCTGATTATTACCCCCCTCTTTAACACTCCTCATTTCTAGAAGAATTGTAGAAAATGGAGCTG
GAACAGGATGAACTGTTTACCCACCCCTATCATCCAATATTGCTCATGGGGGAACATCAGTAGATTTAGCTATTTTTTCA
CTACACCTAGCAGGAATCTCATCTATCTTAGGAGCTATTAATTTTATTACAACCATTATTAATATACGAATTAATGGATT
ATCATTTGACCAAATACCTCTATTTGTGTGAGCTGTTGGTATTACAGCCCTTCTTCTACTCCTCTCTCTCCCAGTTTTAG
CTGGAGCTATTACCATATTACTAACAGATCGAAATTTAAATACATCTTTTTTTGACCCAGCTGTAGGAGGAGATCCAATT
TTATATC--------------------
>BIPR014-13|Scirpophaga_incertulas|YSBCT1|KF371527
----------------------------------ATAGTAGGAAGTTCTTTAAGCTTACTTATTCGAGCTGAATTAGGAA
ATTCTGGATCCTTAATTGGAGATGATCAAATCTATAATACTATTGTCACAGCCCATGCCTTTGTTATAATTTTTTTTATA
GTTATACCCATTATAATTGGAGGATTTGGAAATTGACTAGTCCCCCTAATATTAGGAGCCCCAGATATAGCTTTCCCCCG
ATTAAATAACATAAGATTCTGATTATTACCTCCCTCTTTAACACTCCTCATTTCTAGAAGAATTGTAGAAAATGGAGCTG
GAACAGGATGAACTGTTTACCCACCCCTATCATCCAATATTGCTCATGGGGGAACATCAGTAGATTTAGCTATTTTTTCA
CTACACCTAGCAGGAATCTCATCTATCTTAGGAGCTATTAATTTTATTACAACCATTATTAATATACGAATTAATGGATT
ATCATTTGACCAAATACCTCTATTTGTGTGAGCTGTTGGTATTACAGCCCTTCTTGTACTCCTCTCTCTCCCAGTTTTAG
CTGGAGCTATTACCATATTACTAACAGATGGAAATTTAAATACATCTATTTTTGA--------------------------
----
>CGUKB395-09|Chilo_phragmitella|UKLB15G08|
AACTTTATATTTTATTTTTGGAATTTGAGCTGGAATAATTGGAACATCTCTTAGACTTTTAATTCGAGCTGAATTAGGAA
CTCCAGGATCACTAATTGGAGATGATCAAATTTATAATACTATTGTTACAGCTCATGCATTTATTATAATTTTTTTTATA
GTTATACCTATTATAATTGGTGGTTTTGGAAATTGATTAGTACCTTTAATATTAGGAGCCCCTGATATAGCTTTCCCACG
AATAAATAATATAAGATTTTGATTATTACCACCTTCATTAACCTTATTAATCTCTAGAAGAATTGTTGAAAATGGAGCTG
GAACAGGATGAACAGTGTACCCCCCACTCTCATCTAATATTGCTCATGCTGGAAGTTCAGTAGATTTAGCAATTTTTTCC
TTACATTTAGCTGGAATTTCATCAATTTTAGGTGCTATTAATTTTATTACAACAATTATTAATATACGAATTAATGGATT
ATCATTTGATCAAATACCCCTACTCATTTGAAGAATTGGCATTACAGCATTATTATTATTACTTTCTCTTCCAGTATTAG
CTGGTGCTATTACTATATTATTAACAGATCGAAATTTAAATACATCTTTTTTTGATCCAGCTGGAGGTGGAGATCCTATC
CTCTATCAACATTTATTT---------
>CGUKB587-09|Chilo_phragmitella|UKLB17G12|
AACTTTATATTTTATTTTTGGAATTTGAGCTGGAATAATTGGAACATCTCTTAGACTTTTAATTCGAGCTGAATTAGGAA
CTCCAGGATCACTAATTGGAGATGATCAAATTTATAATACTATTGTTACAGCTCATGCATTTATTATAATTTTTTTTATA
GTTATACCTATTATAATTGGTGGTTTTGGAAATTGATTAGTACCTTTAATATTAGGAGCCCCTGATATAGCTTTCCCACG
AATAAATAATATAAGATTTTGATTATTACCACCTTCATTAACCTTATTAATCTCTAGAAGAATTGTTGAAAATGGAGCTG
GAACAGGATGAACAGTGTACCCCCCACTCTCATCTAATATTGCTCATGCTGGAAGTTCAGTAGATTTAGCAATTTTTTCC

TTACATTTAGCTGGAATTTCATCAATTTTAGGTGCTATTAATTTTATTACAACAATTATTAATATACGAATTAATGGATT
ATCATTTGATCAAATACCCCTACTCATTTGAAGAATTGGCATTACAGCATTATTATTATTACTTTCTCTTCCAGTATTAG
CTGGTGCTATTACTATATTATTAACAGATCGAAATTTAAATACATCTTTTTTTGATCCAGCTGGAGGTGGAGATCCTATC
CTCTATCAACATTTATTT---------
>CGUKB589-09|Chilo_phragmitella|UKLB17H02|
AACTTTATATTTTATTTTTGGAATTTGAGCTGGAATAATTGGAACATCTCTTAGACTTTTAATTCGAGCTGAATTAGGAA
CTCCAGGATCACTAATTGGAGATGATCAAATTTATAATACTATTGTTACAGCTCATGCATTTATTATAATTTTTTTTATA
GTTATACCTATTATAATTGGTGGTTTTGGAAATTGATTAGTACCTTTAATATTAGGAGCCCCTGATATAGCTTTCCCACG
AATAAATAATATAAGATTTTGATTATTACCACCTTCATTAACCTTATTAATCTCTAGAAGAATTGTTGAAAATGGAGCTG
GAACAGGATGAACAGTGTACCCCCCACTCTCATCTAATATTGCTCATGCTGGAAGTTCAGTAGATTTAGCAATTTTTTCC
TTACATTTAGCTGGAATTTCATCAATTTTAGGTGCTATTAATTTTATTACAACAATTATTAATATACGAATTAATGGATT
ATCATTTGATCAAATACCCCTACTCATTTGAAGAATTGGCATTACAGCATTATTATTATTACTTTCTCTTCCAGTATTAG
CTGGTGCTATTACTATATTATTAACAGATCGAAATTTAAATACATCTTTTTTTGATCCAGCTGGAGGTGGAGATCCTATC
CTCTATCAACATTTATTT---------
>CGUKB590-09|Chilo_phragmitella|UKLB17H03|
AACTTTATATTTTATTTTTGGAATTTGAGCTGGAATAATTGGAACATCTCTTAGACTTTTAATTCGAGCTGAATTAGGAA
CTCCAGGATCACTAATTGGAGATGATCAAATTTATAATACTATTGTTACAGCTCATGCATTTATTATAATTTTTTTTATA
GTTATACCTATTATAATTGGTGGTTTTGGAAATTGATTAGTACCTTTAATATTAGGAGCCCCTGATATAGCTTTCCCACG
AATAAATAATATAAGATTTTGATTATTACCACCTTCATTAACCTTATTAATCTCTAGAAGAATTGTTGAAAATGGAGCTG
GAACAGGATGAACAGTGTACCCCCCACTCTCATCTAATATTGCTCATGCTGGAAGTTCAGTAGATTTAGCAATTTTTTCC
TTACATTTAGCTGGAATTTCATCAATTTTAGGTGCTATTAATTTTATTACAACAATTATTAATATACGAATTAATGGATT
ATCATTTGATCAAATACCCCTACTCATTTGAAGAATTGGCATTACAGCATTATTATTATTACTTTCTCTTCCAGTATTAG
CTGGTGCTATTACTATATTATTAACAGATCGAAATTTAAATACATCTTTTTTTGATCCAGCTGGAGGTGGAGATCCTATC
CTCTATCAACATTTATTT---------
>CGUKB624-09|Chilo_phragmitella|UKLB18C03|
AACTTTATATTTTATTTTTGGAATTTGAGCTGGAATAATTGGAACATCTCTTAGACTTTTAATTCGAGCTGAATTAGGAA
CTCCAGGATCACTAATTGGAGATGATCAAATTTATAATACTATTGTTACAGCTCATGCATTTATTATAATTTTTTTTATA
GTTATACCTATTATAATTGGTGGTTTTGGAAATTGATTAGTACCTTTAATATTAGGAGCCCCTGATATAGCTTTCCCACG
AATAAATAATATAAGATTTTGATTATTACCACCTTCATTAACCTTATTAATCTCTAGAAGAATTGTTGAAAATGGAGCTG
GAACAGGATGAACAGTGTACCCCCCACTCTCATCTAATATTGCTCATGCTGGAAGTTCAGTAGATTTAGCAATTTTTTCC
TTACATTTAGCTGGAATTTCATCAATTTTAGGTGCTATTAATTTTATTACAACAATTATTAATATACGAATTAATGGATT
ATCATTTGATCAAATACCCCTACTCATTTGAAGAATTGGCATTACAGCATTATTATTATTACTTTCTCTTCCAGTATTAG
CTGGTGCTATTACTATATTATTAACAGATCGAAATTTAAATACATCTTTTTTTGATCCAGCTGGAGGTGGAGATCCTATC
CTCTATCAACATTTATTT---------
>CYTC730-12|Chilo_suppressalis|HQ860290|HQ860290
AACTTTATATTTTATTTTTGGTATTTGAGCAGGTATAATTGGAACATCTCTTAGACTTTTAATTCGTGCTGAATTAGGAA
CTCCAGGATCTTTAATTGGAGATGATCAAATTTATAATACTATTGTTACAGCTCATGCATTTATTATAATTTTTTTTATA
GTTATACCAATTATAATTGGTGGATTTGGAAATTGATTAGTACCTTTAATATTAGGAGCTCCTGATATAGCTTTCCCACG
AATAAATAATATAAGATTTTGAATATTACCCCCCTCATTAACTTTATTAATTTCTAGAAGAATTGTTGAAAATGGAGCTG
GAACAGGATGAACAGTGTACCCCCCACTATCATCTAATATTGCTCACGCTGGAAGTTCAGTAGATTTAGCAATTTTCTCT
TTACATTTAGCTGGAATTTCTTCAATTCTAGGTGCTATTAATTTTATTACTACGATTATTAATATACGAATTAATGGTCT
TTCATTTGATCAAATACCTTTATTTGTTTGATCCGTAGGTATTACAGCTTTATTATTACTTCTATCTCTACCAGTATTAG
CTGGAGCAATTACAATATTATTAACCGATCGAAATTTAAATACATCTTTTTTTGATCCTGCTGGTGGTGGAGATCCAATT
CTTTATCAACATTTATTTTGATTTTTT
>DMAS001-14|Diatraea_crambidoides|MAS116|KR070995
AACTTTATACTTCATTTTTGGAATTTGAGCAGGAATATTAGGAACATCTTTAAGTTTATTAATTCGAGCAGAATTAGGTA
CACCTAACTCTCTTATTGGAGATGATCAAATTTATAATACAATTGTCACTGCTCATGCATTTATTATAATTTTTTTTATA
GTTATACCTATTATAATTGGGGGATTTGGAAATTGATTAGTTCCCTTAATATTAGGGGCTCCTGATATAGCTTTTCCTCG
AATAAATAACATAAGATTTTGATTATTACCCCCATCTTTAACTTTATTAATTTCTAGAAGAATCGTTGAAAACGGTGCTG
GAACAGGATGAACAGTATATCCCCCCTTATCATCTAATATTGCCCATGGGGGAAGATCTGTAGATTTAGCAATTTTTTCC
CTACATTTAGCAGGAATTTCCTCAATCTTAGGGGCTATTAATTTCATCACTACAATTATTAACATGCGAATTAATGGACT
ATCATTCGATCAAATACCTTTATTTGTTTGATCAGTGGGTATTACAGCATTATTATTATTACTTTCATTACCCGTTTTAG
CTGGAGCTATTACTATATTATTAACTGATCGAAATTTAAATACCTCTTTTTTTGATCCAGCGGGAGGGGGAGATCCCATC
CTATATCAACATTTATTT---------
>DMAS002-14|Diatraea_mitteri|MAS117|KR070998
AACTTTATATTTTATTTTTGGAATTTGAGCAGGAATATTAGGAACATCCTTAAGTTTATTAATTCGAGCAGAGTTAGGTA
CACCCAACTCTCTTATTGGAGATGATCAAATTTATAATACAATTGTCACTGCTCATGCATTTATCATAATTTTTTTTATA

GTTATACCTATTATAATTGGAGGATTTGGAAATTGATTAGTTCCTTTAATATTAGGAGCTCCTGATATAGCTTTCCCCCG
AATAAATAACATAAGATTTTGATTATTACCCCCATCTTTAACTTTATTGATTTCTAGAAGAATCGTTGAAAATGGAGCTG
GAACAGGATGAACAGTATATCCCCCTTTATCATCTAATATTGCTCACGGGGGAGGATCTGTAGATTTAGCAATTTTTTCT
TTACATTTAGCGGGAATTTCATCAATTTTAGGAGCTATTAATTTTATTACTACAATTATCAATATACGAATTAATGGATT
GTCATTTGATCAAATACCATTATTTGTATGATCAGTAGGTATTACAGCATTATTACTATTACTTTCATTACCTGTTTTAG
CTGGAGCTATTACTATATTATTAACTGATCGAAATTTAAATACCTCTTTTTTTGATCCAGCGGGGGGAGGAGATCCTATT
CTATATCAACACTTATTT---------
>DMAS003-14|Diatraea_mitteri|MAS118|KR070999
AACTTTATATTTTATTTTTGGAATTTGAGCAGGAATATTAGGAACATCCTTAAGTTTATTAATTCGAGCAGAGTTAGGTA
CACCCAACTCTCTTATTGGAGATGATCAAATTTATAATACAATTGTCACTGCTCATGCATTTATTATAATTTTTTTTATA
GTTATACCTATTATAATTGGAGGATTTGGAAATTGATTAGTTCCTTTAATATTAGGAGCTCCTGATATAGCTTTCCCCCG
AATAAATAACATAAGATTTTGATTATTACCCCCATCTTTAACTTTATTGATTTCTAGAAGAATCGTTGAAAATGGGGCTG
GAACAGGATGAACAGTATATCCCCCTTTATCATCTAATATTGCTCACGGGGGAGGATCTGTAGATTTAGCAATTTTTTCT
TTACATTTAGCGGGAATTTCATCAATTTTAGGAGCTATTAATTTTATTACTACAATTATCAATATACGAATTAATGGATT
GTCATTTGATCAAATACCATTATTTGTATGATCAGTAGGTATTACAGCATTATTACTATTACTTTCATTACCTGTTTTAG
CTGGAGCTATTACTATGTTATTAACTGATCGAAATTTAAATACCTCTTTTTTTGATCCAGCGGGGGGAGGAGATCCTATT
CTATATCAACACTTATTT---------
>DMAS004-14|Diatraea_mitteri|MAS119|KR071000
AACTTTATATTTTATTTTTGGAATTTGAGCAGGAATATTAGGAACATCCTTAAGTTTATTAATTCGAGCAGAGTTAGGTA
CACCCAACTCTCTTATTGGAGATGATCAAATTTATAATACAATTGTCACTGCTCATGCATTTATTATAATTTTTTTTATA
GTTATACCTATTATAATTGGAGGATTTGGAAATTGATTAGTTCCTTTAATATTAGGAGCTCCTGATATAGCTTTCCCCCG
AATAAATAACATAAGATTTTGATTATTACCCCCATCTTTAACTTTATTGATTTCTAGAAGAATCGTTGAAAATGGGGCTG
GAACAGGATGAACAGTATATCCCCCTTTATCATCTAATATTGCTCACGGGGGAGGATCTGTAGATTTAGCAATTTTTTCT
TTACATTTAGCGGGAATTTCATCAATTTTAGGAGCTATTAATTTTATTACTACAATTATCAATATACGAATTAATGGATT
GTCATTTGATCAAATACCATTATTTGTATGATCAGTAGGTATTACAGCATTATTACTATTACTTTCATTACCTGTTTTAG
CTGGAGCTATTACTATGTTATTAACTGATCGAAATTTAAATACCTCTTTTTTTGATCCAGCGGGGGGAGGAGATCCTATT
CTATATCAACACTTATTT---------
>DMAS005-14|Diatraea_crambidoides|MAS120|KR070996
AACTTTATACTTCATTTTTGGAATTTGAGCAGGAATATTAGGAACATCTTTAAGTTTATTAATTCGAGCAGAATTAGGTA
CACCTAACTCTCTTATTGGAGATGATCAAATTTATAATACAATTGTCACTGCTCATGCATTTATTATAATTTTTTTTATA
GTTATACCTATTATAATTGGGGGATTTGGAAATTGATTAGTTCCCTTAATATTAGGGGCTCCTGATATAGCTTTTCCTCG
AATAAATAACATAAGATTTTGATTATTACCCCCATCTTTAACTTTATTAATTTCTAGAAGAATCGTTGAAAACGGTGCTG
GAACAGGATGAACAGTATATCCCCCCTTATCATCTAATATTGCCCATGGGGGAAGATCTGTAGATTTAGCAATTTTTTCC
CTACATTTAGCAGGAATTTCCTCAATCTTAGGGGCTATTAATTTCATCACTACAATTATTAACATGCGAATTAATGGACT
ATCATTCGATCAAATACCTTTATTTGTTTGATCAGTGGGTATTACAGCATTATTATTATTACTTTCATTACCCGTTTTAG
CTGGAGCTATTACTATATTATTAACTGATCGAAATTTAAATACCTCTTTTTTTGATCCAGCGGGAGGGGGAGATCCCATC
CTATATCAACATTTATTT---------
>DMAS006-14|Diatraea_crambidoides|MAS121|KR070997
AACTTTATACTTCATTTTTGGAATTTGAGCAGGAATATTAGGAACATCTTTAAGTTTATTAATTCGAGCAGAATTAGGTA
CACCTAACTCTCTTATTGGAGATGATCAAATTTATAATACAATTGTCACTGCTCATGCATTTATTATAATTTTTTTTATA
GTTATACCTATTATAATTGGGGGATTTGGAAATTGATTAGTTCCCTTAATATTAGGGGCTCCTGATATAGCTTTTCCTCG
AATAAATAACATAAGATTTTGATTATTACCCCCATCTTTAACTTTATTAATTTCTAGAAGAATCGTTGAAAACGGTGCTG
GAACAGGATGAACAGTATATCCCCCCTTATCATCTAATATTGCCCATGGGGGAAGATCTGTAGATTTAGCAATTTTTTCC
CTACATTTAGCAGGAATTTCCTCAATCTTAGGGGCTATTAATTTCATCACTACAATTATTAACATGCGAATTAATGGACT
ATCATTCGATCAAATACCTTTATTTGTTTGATCAGTGGGTATTACAGCATTATTATTATTACTTTCATTACCCGTTTTAG
CTGGAGCTATTACTATATTATTAACTGATCGAAATTTAAATACCTCTTTTTTTGATCCAGCGGGAGGGGGAGATCCCATC
CTATATCAACATTTATTT---------
>FBLMS454-09|Chilo_phragmitella|BC_ZSM_Lep_23779|GU706443-SUPPRESSED
AACTTTATATTTTATTTTTGGAATTTGAGCTGGAATAATTGGAACATCTCTTAGACTTTTAATTCGAGCTGAATTAGGAA
CTCCAGGATCCCTAATTGGAGATGATCAAATTTATAATACTATTGTTACAGCTCATGCATTTATTATAATTTTTTTTATA
GTTATACCTATTATAATCGGTGGTTTTGGAAATTGATTAGTACCTTTAATATTAGGAGCCCCTGATATAGCTTTCCCACG
AATAAATAATATAAGATTTTGATTATTACCACCTTCATTAACCTTATTAATCTCTAGAAGAATTGTTGAAAATGGAGCTG
GAACAGGATGAACAGTGTACCCCCCACTTTCATCTAATATTGCTCATGCTGGAAGTTCAGTAGATTTAGCAATTTTTTCC
TTACATTTAGCTGGAATTTCATCAATTTTAGGTGCTATTAATTTTATTACAACAATTATTAATATACGAATTAATGGATT
ATCATTTGATCAAATACCCTTACTCATTTGAAGAATTGGTATTACAGCATTATTATTATTACTTTCTCTCCCAGTATTAG
CTGGTGCTATTACTATATTATTAACAGATCGAAATTTAAATACATCTTTTTTTGATCCAGCTGGAGGTGGAGATCCTATT
CTCTATCAACATTTATTT---------

>FBLMZ389-12|Chilo_phragmitella|BC_ZSM_Lep_61145|KX047506
AACTTTATATTTTATTTTTGGAATTTGAGCTGGAATAATTGGAACATCTCTTAGACTTTTAATTCGAGCTGAATTAGGAA
CTCCAGGATCCCTAATTGGAGATGATCAAATTTATAATACTATTGTTACAGCTCATGCATTTATTATAATTTTTTTTATA
GTTATACCTATTATAATCGGTGGTTTTGGAAATTGATTAGTACCTTTAATATTAGGAGCCCCTGATATAGCTTTCCCACG
AATAAATAATATAAGATTTTGATTATTACCACCTTCATTAACCTTATTAATCTCTAGAAGAATTGTTGAAAATGGAGCTG
GAACAGGATGAACAGTGTACCCCCCACTTTCATCTAATATTGCTCATGCTGGAAGTTCAGTAGATTTAGCAATTTTTTCC
TTACATTTAGCTGGAATTTCATCAATTTTAGGTGCTATTAATTTTATTACAACAATTATTAATATACGAATTAATGGATT
ATCATTTGATCAAATACCCTTACTCATTTGAAGAATTGGTATTACAGCATTATTATTATTACTTTCTCTCCCAGTATTAG
CTGGTGCTATTACTATATTATTAACAGATCGAAATTTAAATACATCTTTTTTTGATCCAGCTGGAGGTGGAGATCCTATT
CTCTATCAACATTTATTT---------
>GBGL1133-06|Sesamia_nonagrioides|AY649322|AY649322

------------------GGAGGATTTGGAAATTGATTAGTTCCTTTAATATTAGGAGCCCCAGATATAGCATTTCCACG
AATAAATAATATAAGATTTTGACTATTACCACCATCCTTAACCCTTTTAATTTCAAGTAGAATTGTAGAAAATGGGGCTG
GAACAGGATGAACAGTTTACCCCCCACTTTCATCTAACATCGCTCACGGAGGAAGATCTGTAGATTTAGCTATTTTTTCC
CTTCATTTAGCTGGAATTTCATCTATTCTAGGAGCTATTAATTTTATTACAACAATTATTAATATACGATTAAATAATTT
ATCATTTGATCAAATACCATTATTTATTTGAGCTGTTGGAATTACTGCTTTTTTATTACTATTATCATTACCCGTTTTAG
CAGGAGCTATTACTATACTACTTACGGATCGAAATTTAAATACATCATTTTTTGATCCTGCGGGAGGAGGTGATCCAATT
TTATACCAACACTTATTCTGATTTTTT
>GBGL3692-06|Eldana_saccharina|DQ486914|DQ486914
----TTATACTTTATTTTTGGTATTTGATCAGGAATAGTAGGAACTTCTCTTAGATTACTAATTCGAGCTGAATTAGGAA
ATCCAGGATCTTTAATTGGAGATGATCAAATTTATAATACTATTGTTACAGGTCATGCTTTTATTATAATTTTTTTTATA
GTTATACCTATTATAATTGGAGGATTTGGTAATTGACTCGTACCTCTAATACTTGGAGCCCCCGATATAGCTTTCCCCCG
TATAAATAATATAAGTTTTTGACTATTACCTCCTTCTCTTTCTCTATTAATTTTTAGAAGAATTGTTGAAAATGGAGCAG
GAACAGGATGAACAGTTTATCCCCCATTATCTTCAAATATCGCTCATAGAGGAAGTTCTGTAGATTTAGCTATTTTTTCT
CTTCATTTAGCTGGAATTTCATCAATTTTAGGGGCCATTAATTTTATTACAACAGTAATTAATATAAAATTAAATGGCCT
ATCATTTGATCAAATACCTTTATTTGTATGAGCTGTAAGAATTACAGCTCTTCTTTTACTTTTATCTTTACCAGTTTTAG
CAGGTGCAATTACTATACTATTAACAGATCGTAATCTAAATACATCTTTTTTTGACCCTGCCGGAGGAGGAGATCCTATT
CTTTACCAACATTTATTTTGATTTTTT
>GBGL3694-06|Eldana_saccharina|DQ486916|DQ486916
----TTATACTTTATTTTTGGTATTTGATCAGGAATAGTAGGAACTTCTCTTAGATTACTAATTCGAGCTGAATTAGGAA
ATCCAGGATCTTTAATTGGAGATGATCAAATTTATAATACTATTGTTACAGGTCATGCTTTTATTATAATTTTTTTTATA
GTTATACCTATTATAATTGGAGGATTTGGTAATTGACTCGTACCTCTAATACTCGGAGCCCCCGATATAGCTTTCCCCCG
TATAAATAATATAAGTTTTTGACTATTACCTCCTTCTCTTTCTCTATTAATTTTTAGAAGAATTGTTGAAAATGGAGCAG
GAACAGGATGAACAGTTTATCCCCCATTATCTTCAAATATCGCTCATAGAGGAAGTTCTGTAGATTTAGCTATTTTTTCT
CTTCATTTAGCTGGAATTTCATCAATTTTAGGGGCCATTAATTTTATTACAACAGTAATTAATATAAAATTAAATGGCCT
ATCATTTGATCAAATACCTTTATTTGTATGAGCTGTAAGAATTACAGCTCTTCTTTTACTTTTATCTTTACCAGTTTTAG
CAGGTGCAATTACTATACTATTAACAGATCGTAATCTAAATACATCTTTTTTTGACCCTGCCGGAGGAGGAGATCCTATT
CTTTACCAACATTTATTTTGATTTTTT
>GBGL3696-06|Eldana_saccharina|DQ486918|DQ486918
----TTATACTTTATTTTTGGTATTTGATCAGGAATAGTAGGAACTTCTCTTAGATTACTAATTCGAGCTGAATTAGGAA
ATCCAGGATCTTTAATTGGGGATGACCAAATTTATAATACTATTGTTACAGGTCATGCTTTTATTATAATTTTTTTTATA
GTTATACCTATTATAATTGGAGGATTTGGTAATTGACTCGTACCTCTAATACTCGGAGCCCCCGATATAGCTTTCCCCCG
TATAAATAATATAAGTTTTTGACTATTACCTCCTTCTCTTTCTCTATTAATTTTTAGAAGAATTGTTGAAAATGGGGCAG
GAACAGGATGAACAGTTTATCCCCCATTATCCTCAAATATCGCTCATAGAGGAAGTTCTGTAGATTTAGCTATTTTTTCT
CTTCATTTAGCTGGAATTTCATCAATTTTAGGGGCTATTAATTTTATTACAACAGTAATTAATATAAAATTAAATGGTCT
ATCATTTGATCAAATACCTTTATTCGTATGAGCTGTAAGAATTACAGCTCTTCTTTTACTTTTATCTTTACCAGTTTTAG
CAGGTGCAATTACTATACTATTAACAGATCGTAATCTAAATACATCTTTTTTTGACCCTGCCGGAGGGGGAGATCCTATT
CTTTACCAACATTTATTTTGATTTTTT
>GBGL3697-06|Eldana_saccharina|DQ486919|DQ486919
----TTATACTTTATTTTTGGTATTTGATCAGGAATAGTAGGAACTTCTCTTAGATTACTAATTCGAGCTGAATTAGGAA
ATCCAGGATCTTTAATTGGGGATGACCAAATTTATAATACTATTGTTACAGGTCATGCTTTTATTATAATTTTTTTTATA
GTTATACCTATTATAATTGGAGGATTTGGTAATTGACTCGTACCTCTAATACTCGGAGCCCCCGATATAGCTTTCCCCCG
TATAAATAATATAAGTTTTTGACTATTACCTCCTTCTCTTTCTCTATTAATTTTTAGAAGAATTGTTGAAAATGGGGCAG
GAACAGGATGAACAGTTTATCCCCCATTATCCTCAAATATCGCTCATAGAGGAAGTTCTGTAGATTTAGCTATTTTTTCT
CTTCATTTAGCTGGAATTTCATCAATTTTAGGGGCTATTAATTTTATTACAACAGTAATTAATATAAAATTAAATGGTCT

ATCATTTGATCAAATACCTTTATTCGTATGAGCTGTAAGAATTACAGCTCTTCTTTTACTTTTATCTTTACCAGTTTTAG
CAGGTGCAATTACTATACTATTAACAGATCGTAATCTAAATACATCTTTTTTTGACCCTGCCGGAGGAGGAGATCCTATT
CTTTACCAACATTTATTTTGATTTTTT
>GBGL3700-06|Eldana_saccharina|DQ486922|DQ486922
----------------------ATTTGATCAGGAATAGTAGGAACTTCTCTTAGATTACTAATTCGAGCTGAATTAGGAA
ATCCAGGATCTTTAATTGGAGATGACCAAATTTATAATACTATTGTTACAGGTCATGCTTTTATTATAATTTTTTTTATA
GTTATACCTATTATAATTGGAGGATTTGGTAATTGACTCGTACCTCTAATACTCGGAGCCCCCGATATAGCTTTCCCCCG
TATAAATAATATAAGTTTTTGACTACTACCTCCTTCTCTTTCTCTATTAATTTTTAGAAGAATTGTTGAAAACGGGGCAG
GAACAGGATGAACAGTCTATCCCCCATTATCTTCAAATATCGCTCATAGAGGAAGTTCTGTAGATTTAGCTATTTTTTCT
CTTCATTTAGCTGGAATTTCATCAATTTTAGGAGCTATTAATTTTATTACAACAGTAATTAATATAAAATTAAATGGTCT
ATCATTTGATCAAATACCTTTATTCGTATGAGCTGTAAGAATTACAGCTCTTCTTTTACTTTTATCTTTACCAGTTTTAG
CAGGTGCAATTACTATACTATTAACAGATCGTAATCTAAATACATCTTTTTTTGATCCTGCCGGAGGAGGAGATCCTATT
CTTTACCAACATTTATTTTGATTTTTT
>GBGL3701-06|Eldana_saccharina|DQ486923|DQ486923
--GGAA
ATCCAGGATCTTTAATTGGAGATGACCAAATTTATAATACTATTGTTACAGGTCATGCTTTTATTATAATTTTTTTTATA
GTTATACCTATTATAATTGGAGGATTTGGTAACTGACTCCTACCTCTAATACTCGGAGCCCCCGATATAGCTTTCCCCCG
TATAAATAATATAAGTTTAGACNATTACCTCCTTCTCTTTCTCTATTAATTTTTAGAAGAATTGTTGAAAACGGAGCAG
GAACAGGATGAACAGTCTATCCCCCATTATCTTCAAATATCGCTCATAGAGGAAGTTCTGTAGATTTAGCTATTTTTTCT
CTTCATTTAGCTGGAATTTCATCAATTTTAGGAGCTATTAATTTTATTACAACAGTAATTAATATAAAATTAAATGGTCT
ATCATTTGATCAAATACCTTTATTCGTATGAGCTGTAAGAATTACAGCTCTTCTTTTACTTTTATCTTTACCAGTTTTAG
CAGGTGCAATTACTATACTATTAACAGATCGTAATCTAAATACATCTTTTTTTGATCCTGCCGGAGGAGGAGATCCTATT
CTTTACCAACATTTATTTTGATTTTTT
>GBGL6595-09|Scirpophaga_incertulas|AB495274|AB495274
AACTTTATATTTTATTTTTGGAATTTGAGCTGGTATAGTAGGAACTTCTTTAAGCTTACTTATTCGAGCTGAATTAGGAA
CTTCTGGATCCTTAATTGGAGATGATCAAATCTATAACACTATTGTCACAGCCCATGCCTTTATTATAATTTTTTTTATA
GTTATACCCATTATAATTGGAGGATTTGGAAATTGATTAGTCCCCCTAATATTAGGAGCCCCAGATATAGCTTTCCCCCG
AATAAATAACANAAGATTCTGATTATTACCCCCCTCTTTAACACTCCTCATTTCTAGAAGAATTGTAGAAAATGGGGCTG
GAACAGGATGAACTGTTTACCCACCCCTATCATCCAATATTGCTCATGGGGGAACATCAGTAGATTTAGCTATTTTTTCT
CTACACCTAGCAGGAATTTCATCTATTTTAGGAGCTATTAATTTTATTACAACCATTATTAATATACGAATTAATGGATT
ATCATTTGACCAAATACCTCTATTTGTGTGAGCTGTTGGTATTACAGCCCTTCTTTTACTTCTCTCTCTCCCAGTTTTAG
CTGGAGCTATTACCATATTACTAACAGATCGAAATTTAAATACATCTTTTTTTGACCCAGCTGGAGGAGGAGATCCAATT
TTATATCAACAC---------------
>GBGL6596-09|Scirpophaga_incertulas|AB495273|AB495273
TACTTTATATTTTATTTTTGGAATTTGAGCTGGTATAGTAGGAACTTCTTTAAGCTTACTTATTCGAGCTGAATTAGGAA
CTTCTGGATCCTTAATTGGAGATGATCAAATCTATAACACTATTGTCACAGCCCATGCCTTTATTATAATTTTTTTTATA
GTTATACCCATTATAATTGGAGGATTTGGAAATTGATTAGTCCCCCTAATATTAGGAGCCCCAGATATAGCTTTCCCCCG
AATAAATAACATAAGATTCTGATTATTACCCCCCTCTTTAACACTCCTCATTTCTAGAAGAATTGTAGAAAATGGGGCTG
GAACAGGATGAACTGTTTACCCACCCCTATCATCCAATATTGCTCATGGGGGAACATCAGTAGATTTAGCTATTTTTTCT
CTACACCTAGCAGGAATTTCATCTATTTTAGGAGCTATTAATTTTATTACAACCATTATTAATATACGAATTAATGGATT
ATCATTTGACCAAATACCTCTATTTGTGTGAGCTGTTGGTATTACAGCCCTTCTTTTACTTCTCTCTCTCCCAGTTTTAG
CTGGAGCTATTACCATATTACTAACAGATCGAAATTTAAATACATCTTTTTTTGACCCAGCTGGAGGAGGAGATCCAATT
TTATATCAACAC---------------
>GBGL6597-09|Scirpophaga_incertulas|AB495272|AB495272
TACTTTATATTTTATTTTTGGAATTTGAGCTGGTATAGTAGGAACTTCTTTAAGCTTACTTATTCGAGCTGAATTAGGAA
CTTCTGGATCCTTAATTGGAGATGATCAAATCTATAACACTATTGTCACAGCCCATGCCTTTATTATAATTTTTTTTATA
GTTATACCCATTATAATTGGAGGATTTGGAAATTGATTAGTCCCCCTAATATTAGGAGCCCCAGATATAGCTTTCCCCCG
AATAAATAACATAAGATTCTGATTATTACCCCCCTCTTTAACACTCCTCATTTCTAGAAGAATTGTAGAAAATGGGGCTG
GAACAGGATGAACTGTTTACCCACCCCTATCATCCAATATTGCTCATGGGGGAACATCAGTAGATTTAGCTATTTTTTCT
CTACACCTAGCAGGAATTTCATCTATTTTAGGAGCTATTAATTTTATTACAACCATTATTAATATACGAATTAATGGATT
ATCATTTGACCAAATACCTCTATTTGTGTGAGCTGTTGGTATTACAGCCCTTCTTTTACTTCTCTCTCTCCCAGTTTTAG
CTGGAGCTATTACCATATTACTAACAGATCGAAATTTAAATACATCTTTTTTTGACCCAGCTGGAGGAGGAGATCCAATT
TTATATCAACAC---------------
>GBGL6598-09|Scirpophaga_incertulas|AB495271|AB495271
TACTTTATATTTTATTTTTGGAATTTGAGCTGGTATAGTAGGAACTTCTTTAAGCTTACTTATTCGAGCTGAATTAGGAA
CTTCTGGAGCCTTAATTGGAGATGATCAAATCTATAACACTATTGTCACAGCCCATGCCTTTATTATAATTTTTTTTATA
GTTATACCCATTATAATTGGAGGATTTGGAAATTGATTAGTCCCCCTAATATTAGGAGCCCCAGATATAGCTTTCCCCCG

AATAAATAACATAAGATTCTGATTATTACCCCCCTCTTTAACACTCCTCATTTCTAGAAGAATTGTAGAAAATGGGGCTG
GAACAGGATGAACTGTTTACCCACCCCTATCATCCAATATTGCTCATGGGGGAACATCAGTAGATTTAGCTATTTTTTCT
CTACACCTAGCAGGAATTTCATCTATTTTAGGAGCTATTAATTTTATTACAACCATTATTAATATACGAATTAATGGATT
ATCATTTGACCAAATACCTCTATTTGTGTGAGCTGTTGGTATTACAGCCCTTCTTTTACTTCTCTCTCTCCCAGTTTTAG
CTGGAGCTATTACCATATTACTAACAGATCGAAATTTAAATACATCTTTTTTTGACCCAGCTGGAGGAGGAGATCCAATT
TTATATCAACAC---------------
>GBGL6599-09|Scirpophaga_incertulas|AB495270|AB495270
TACTTTATATTTTATTTTTGGAATTTGAGCTGGTATAGTAGGAACTTCTTTAAGCTTACTTATTCGAGCTGAATTAGGAA
CTTCTGGATCCTTAATTGGAGATGATCAAATCTATAACACTATTGTCACAGCCCATGCCTTTATTATAATTTTTTTTATA
GTTATACCCATTATAATTGGAGGATTTGGAAATTGATTAGTCCCCCTAATATTAGGAGCCCCAGATATAGCTTTCCCCCG
AATAAATAACATAAGATTCTGATTATTACCCCCCTCTTTAACACTCCTCATTTCTAGAAGAATTGTAGAAAATGGGGCTG
GAACAGGATGAACTGTTTACCCACCCCTATCATCCAATATTGCTCATGGGGGAACATCAGTAGATTTAGCTATTTTTTCT
CTACACCTAGCAGGAATTTCATCTATTTTAGGAGCTATTAATTTTATTACAACCATTATTAATATACGAATTAATGGATT
ATCATTTGACCAAATACCTCTATTTGTATGAGCTGTTGGTATTACAGCCCTTCTTTTACTTCTCTCTCTCCCAGTTTTAG
CTGGAGCTATTACCATATTACTAACAGATCGAAATTTAAATACATCTTTTTTTGATCCAGCTGGAGGAGGAGATCCAATT
TTATATCAACAC---------------
>GBGL6600-09|Scirpophaga_incertulas|AB495269|AB495269
TACTTTATATTTTATTTTTGGAGTTTGAGCTGGTATAGTAGGAACTTCTTTAAGCTTACTTATTCGAGCTGAATTAGGAA
CTTCTGGATCCTTAATTGGAGATGATCAAATCTATAACACTATTGTCACAGCCCATGCCTTTATTATAATTTTTTTTATA
GTTATACCCATTATAATTGGAGGATTTGGAAATTGATTAGTCCCCCTAATATTAGGAGCCCCAGATATAGCTTTCCCCCG
AATAAATAACATAAGATTCTGATTATTACCCCCCTCTTTAACACTCCTCATTTCTAGAAGAATTGTAGAAAATGGGGCTG
GAACAGGATGAACTGTTTACCCACCCCTATCATCCAATATTGCTCATGGGGGAACATCAGTAGATTTAGCTATTTTTTCT
CTACACCTAGCAGGAATTTCATCTATTTTAGGAGCTATTAATTTTATTACAACCATTATTAATATACGAATTAATGGATT
ATCATTTGACCAAATACCTCTATTTGTGTGAGCTGTTGGTATTACAGCTCTTCTTTTACTTCTCTCTCTCCCAGTTTTAG
CTGGAGCTATTACCATATTACTAACAGATCGAAATTTAAATACATCTTTTTTTGACCCAGCTGGAGGAGGAGATCCAATT
TTATATCAACAC---------------
>GBGL6601-09|Scirpophaga_incertulas|AB495268|AB495268
TACTTTATATTTTATTTTTGGAATTTGAGCTGGTATAGTAGGAACTTCTTTAAGCTTACTTATTCGAGCTGAATTAGGAA
CTTCTGGATCCTTAATTGGAGATGATCAAATCTATAACACTATTGTCACAGCCCATGCCTTTATTATAATTTTTTTTATA
GTTATACCCATTATAATTGGAGGATTTGGAAATTGATTAGTCCCCCTAATATTAGGAGCCCCAGATATAGCTTTCCCCCG
AATAAATAACATAAGATTCTGATTATTACCCCCCTCTTTAACACTCCTCATTTCTAGAAGAATTGTAGAAAATGGGGCTG
GAACAGGATGAACTGTTTACCCACCCCTATCATCCAATATTGCTCATGGGGGAACATCAGTAGATTTAGCTATTTTTTCT
CTACACCTAGCAGGAATTTCATCTATTTTAGGAGCTATTAATTTTATTACAACCATTATTAATATACGAATTAATGGATT
ATCATTTGACCAAATACCTCTATTTGTGTGAGCTGTTGGTATTACAGCCCTTCTTTTACTTCTCTCTCTCCCAGTTTTAG
CTGGAGCTATTACCATATTACTAACAGATCGAAATTTAAATACATCTTTTTTTGACCCAGCTGGAGGAGGAGATCCAATT
TTATATCAACAC---------------
>GBGL6602-09|Scirpophaga_incertulas|AB495267|AB495267
TACTTTATATTTTATTTTTGGAATTTGAGCTGGTATAGTAGGAACTTCTTTAAGCTTACTTATTCGAGCTGAATTAGGAA
CTTCTGGATCCTTAATTGGAGATGATCAAATCTATAACACTATTGTCACAGCCCATGCCTTTATTATAATTTTTTTTATA
GTTATACCCATTATAATTGGAGGATTTGGAAATTGATTAGTCCCCCTAATATTAGGAGCCCCAGATATAGCTTTCCCCCG
AATAAATAACATAAGATTCTGATTATTACCCCCCTCTTTAACACTCCTCATTTCTAGAAGAATTGTAGAAAATGGGGCTG
GAACAGGATGAACTGTTTACCCACCCCTATCATCCAATATTGCTCATGGGGGAACATCAGTAGATTTAGCTATTTTTTCT
CTACACCTAGCAGGAATTTCATCTATTTTAGGAGCTATTAATTTTATTACAACCATTATTAATATACGAATTAATGGATT
ATCATTTGACCAAATACCTCTATTTGTGTGAGCTGTTGGTATTACAGCCCTTCTTTTACTTCTCTCTCTCCCAGTTTTAG
CTGGAGCTATTACCATATTACTAACAGATCGAAATTTAAATACATCTTTTTTTGACCCAGCTGGAGGAGGAGATCCAATT
TTATATCAACAC---------------
>GBGL6603-09|Scirpophaga_incertulas|AB495266|AB495266
TACTTTATATTTTATTTTTGGAATTTGAGCTGGTATAGTAGGAACTTCTTTAAGCTTACTTATTCGAGCTGAATTAGGAA
CTTCTGGATCCTTAATTGGAGATGATCAAATCTATAACACTATTGTCACAGCCCATGCCTTTATTATAATTTTTTTTATA
GTTATACCCATTATAATTGGAGGATTTGGAAATTGATTAGTCCCCCTAATATTAGGAGCCCCAGATATAGCTTTCCCCCG
AATAAATAACATAAGATTCTGATTATTACCCCCCTCTTTAACACTCCTCATTTCTAGAAGAATTGTAGAAAATGGGGCTG
GAACAGGATGAACTGTTTACCCACCCCTATCATCCAATATTGCTCATGGGGGAACATCAGTAGATTTAGCTATTTTTTCT
CTACACCTAGCAGGAATTTCATCTATTTTAGGAGCTATTAATTTTATTACAACCATTATTAATATACGAATTAATGGATT
ATCATTTGACCAAATACCTCTATTTGTGTGAGCTGTTGGTATTACAGCCCTTCTTTTACTTCTCTCTCTCCCAGTTTTAG
CTGGAGCTATTACCATATTACTAACAGATCGAAATTTAAATACATCTTTTTTTGATCCAGCTGGAGGAGGAGATCCAATT
TTATATCAACAC---------------
>GBGL6604-09|Scirpophaga_incertulas|AB495265|AB495265

TACTTTATATTTTATTTTTGGAATTTGAGCTGGTATAGTAGGAACTTCTTTAAGCTTACTTATTCGAGCTGAATTAGGAA
CTTCTGGATCCTTAATTGGAGATGATCAAATCTATAACACTATTGTCACAGCCCATGCCTTTATTATAATTTTTTTATA
GTTATACCCATTATAATTGGAGGATTTGGAAATTGATTAGTCCCCCTAATATTAGGAGCCCCAGATATAGCTTTCCCCCG
AATAAATAACATAAGATTCTGATTATTACCCCCCTCTTTAACACTCCTCATTTCTAGAAGAATTGTAGAAAATGGGGCTG
GAACAGGATGAACTGTTTACCCACCCCTATCATCCAATATTGCTCATGGGGGAACATCAGTAGATTTAGCTATTTTTTCT
CTACACCTAGCAGGAATTTCATCTATTTTAGGAGCTATTAATTTTATTACAACCATTATTAATATACGAATTAATGGATT
ATCATTTGACCAAATACCTCTATTTGTGTGAGCTGTTGGTATTACAGCCCTTCTTTTACTTCTCTCTCTCCCAGTTTTAG
CTGGAGCTATTACCATATTACTAACAGATCGAAATTTAAATACATCTTTTTTTGACCCAGCTGGAGGAGGAGATCCAATT
TTATATCAACAC---------------
>GBGL6605-09|Scirpophaga_innotata|AB495264|AB495264
AACTTTATATTTCATTTTTGGAATTTGAGCTGGTATAGTAGGAACTTCTTTAAGATTATTAATTCGAGCTGAATTAGGAA
CTCCAGGATCTTTAATTGGAGATGATCAAATTTATAATACCATTGTTACAGCTCATGCTTTTATTATAATTTTTTTATA
GTAATACCAATTATAATTGGAGGATTTGGAAATTGACTTGTTCCTTTAATATTAGGAGCTCCTGATATAGCTTTCCCCCG
TATAAATAATATAAGATTTTGATTATTACCCCCCTCATTAACTCTTCTAATTTCAAGTAGAATTGTAGAAAATGGTGCAG
GAACAGGATGAACAGTATACCCCCCTTTATCATCAAATATTGCTCATAGAGGAACTTCTGTAGATTTGGCTATTTTTTCT
TTACATCTTGCAGGAATTTCCTCTATTTTAGGAGCTATTAACTTTATTACCACTATTATTAATATACGAATTAATGGATT
AACATTTGATCAAATACCTCTATTTGTTTGAGCCGTTGGAATTACAGCCCTTCTTTTACTCCTCTCATTACCTGTATTAG
CTGGAGCCATTACTATATTATTAACTGATCGAAATTTAAATACTTCTTTTTTTGACCCCGCAGGAGGAGGAGATCCAATT
CTTTATCAACAT---------------
>GBGL7462-12|Chilo_suppressalis|AB238202|AB238202
AACTTTATATTTTATTTTTGGTATTTGAGCAGGTATAATTGGAACATCTCTTAGACTTTTAATTCGTGCTGAATTAGGAA
CTCCAGGATCTTTAATTGGAGATGATCAAATTTATAATACCATTGTTACAGCTCATGCATTTATTATAATTTTTTTATA
GTTATACCAATTATAATTGGTGGATTTGGAAATTGATTAGTACCTTTAATATTAGGAGCTCCTGATATAGCTTTCCCACG
AATAAATAATATAAGATTTTGAATATTACCCCCCTCATTAACTTTACTAATTTCTAGAAGAATTGTTGAAAATGGAGCTG
GAACAGGATGAACAGTGTACCCCCCACTATCATCTAATATTGCTCACGCTGGAAGTTCAGTAGATTTAGCAATTTTCTCT
TTACATTTAGCTGGAATTTCTTCAATTCTAGGTGCTATTAATTTTATTACTACGATTATTAATATACGAATTAATGGTCT
TTCATTTGATCAAATACCTTTATTTGTTTGATCCGTAGGTATTACAGCTTTATTATTACTTCTATCTCTACCAGTATTAG
CTGGAGCAATTACAATATTATTAACCGATCGAAATTTAAATACATCTTTTTTTGATCCTGCTGGTGGTGGAGATCCAATT
CTTTATCAACATTTATTTTGATTTTTT
>GBGL7463-12|Chilo_suppressalis|AB238204|AB238204
AACTTTATATTTTATTTTTGGTATTTGAGCAGGTATAATTGGAACATCTCTTAGACTTTTAATTCGTGCTGAATTAGGAA
CTCCAGGATCTTTAATTGGAGATGATCAAATTTATAATACCATTGTTACGGCTCATGCATTTATTATAATTTTTTTATA
GTTATACCAATTATAATTGGTGGATTTGGAAATTGATTAGTACCTTTAATATTAGGAGCTCCTGATATAGCTTTCCCACG
AATAAATAATATAAGATTTTGAATATTACCCCCCTCATTAACTTTACTAATTTCTAGAAGAATTGTTGAAAATGGAGCTG
GAACAGGTTGAACAGTGTACCCCCCACTATCATCTAATATTGCTCACGCTGGAAGTTCAGTAGATTTAGCAATTTTCTCT
TTACATTTAGCTGGAATTTCTTCAATTCTAGGTGCTATTAATTTTATTACTACAATTATTAATATACGAATTAATGGTCT
TTCATTTGACCAAATACCTTTATTTGTTTGATCCGTAGGTATTACAGCTTTATTATTACTTCTATCTCTACCAGTATTAG
CTGGAGCAATTACAATATTATTAACCGATCGAAATTTAAATACATCTTTTTTTGATCCTGCTGGTGGTGGAGATCCAATT
CTTTACCAACATTTATTTTGATTTTTT
>GBGL12175-13|Chilo_polychrysa|KC631647|KC631647

--CTATTGTTACAGCTCATGCATTTATTATAATTTTTTTATA
GTTATACCAATTATAATTGGTGGATTTGGTAATTGATTAATCCCCTTAATATTAGGAGCACCAGATATAGCTTTCCCACG
AATAAATAACATAAGATTTTGATTATTACCACCTTCATTAACACTTTTAATTTCAAGAAGAATTGTTGAAAATGGAGCAG
GAACAGGATGAACAGTATACCCCCCACTTTCATCTAATATTGCTCATGCTGGTAGTTCAGTTGATTTAGCAATTTTTTCT
CTTCATTTAGCTGGTATTTCTTCAATTTTAGGTGCTATTAATTTTATTACAACAATTATTAATATACGAATTAATAAATT
ATCTTTTGATCAATTACCATTATTTGTTTGATCTGTCGGTATCACAGCTTTATTATTATTACTTTCTTTACCTGTATTAG
CAGGTGCTATTACTATACTTTTAACTGATCGAAATTTAAATACATCTTTTTTTGATCCTGCTGGAGGAGGAAATCCAATT
CTTTATCAACATTTATTTTGATTTTTT
>GBGL12186-13|Chilo_auricilius|KC306951|KC306951
AACTTTATACTTTATTTTTGGAATTTGAAGTGGAATAATTGGAACATCTCTAAGACTTTTAATTCGTGCTGAATTAGGAA
CTCCAGGGTCATTAATTGGAGATGATCAAATTTACAATACTATTGTTACAGCTCATGCATTTATTATAATTTTTTTATA
GTTATACCAATTATAATTGGAGGCTTTGGTAATTGATTAGTACCATTAATACTAGGGGCTCCTGATATAGCCTTCCCTCG
AATAAATAATATAAGATTTTGATTATTGCCCCCATCATTAACATTATTAATTTCTAGAAGAATTGTAGAAAATGGAGCTG
GAACAGGATGAACGGTTTACCCCCCCCTTTCATCAAATATTGCCCATGGAGGAAGTTCTGTAGATTTAGCTATTTTTTCT
CTTCATTTAGCTGGTATTTCCTCAATTTTAGGAGCTATTAATTTTATTACAACAATTATTAATATACGAATTAATAAACT
ATCATTTGATCAATTACCATTATTTGTTTGATCTGTTGGTATTACAGCTTTATTATTACTACTTTCATTGCCGGTACTAG

CTGGAGCTATTACTATACTTTTAACTGATCGAAACTTAAATAC-------------------------------------
----
>GBGL12188-13|Sesamia_inferens|KC306949|KC306949
AACATTATATTTTATTTTTGGAATTTGAGCTGGTATAGTAGGAACATCATTAAGATTATTAATTCGAGCTGAATTAGGAA
CTCCTGGATCTTTAATTGGAGATGATCAAATTTATAATACTATTGTTACAGCTCATGCTTTTATTATAATTTTTTTTATA
GTTATACCAATTATAATTGGAGGATTTGGAAATTGACTTGTACCTTTAATATTAGGAGCTCCTGATATAGCTTTTCCACG
AATAAATAATATAAGATTTTGATTATTACCCCCCTCTTTAACTCTTTTAATTTCAAGCAGAATTGTAGAAAATGGAGCAG
GAACTGGGTGAACAGTGTACCCCCCACTTTCATCTAATATTGCCCATGGAGGAAGATCAGTAGATTTAGCTATTTTTTCC
CTTCATTTAGCTGGTATTTCATCTATTTTAGGAGCTATTAATTTTATTACAACAATTATCAATATACGATTAAATAGTTT
ATCTTTTGACCAAATACCTCTATTTATTTGAGCTGTTGGAATTACTGCATTTTTATTATTACTATCTTTACCTGTATTAG
CAGGGGCTATTACAATATTATTAACAGATCGAAATTTAAATAC-------------------------------------
----
>GBGL12189-13|Chilo_sacchariphagus|KC306948|KC306948
AACTTTATATTTTATTTTTGGAATTTGAGCTGGAATAGTTGGAACATCACTTAGACTTTTAATTCGAGCTGAATTAGGAA
ATCCAGGTTCATTAATTGGAGATGATCAAATTTATAACACTATTGTTACAGCTCATGCATTTATTATAATTTTTTTTATA
GTAATACCAATTATAATTGGAGGATTTGGAAATTGATTAGTTCCATTAATATTAGGAGCTCCTGATATAGCTTTTCCCCG
TCTAAATAATATAAGATTTTGATTATTACCCCCCTCTTTAACCCTTTTAATTTCTAGAAGAATCGTTGAAAACGGAGCAG
GAACTGGATGAACAGTCTACCCCCCTTTATCTTCCAATATTTCACATGCTGGAAGTTCAGTAGATTTAGCCATCTTTTCC
CTCCATTTAGCTGGAATTTCTTCAATTTTAGGAGCTATTAATTTTATTACTACAATTATTAATATACGAATTAATGGACT
ATTATTTGATCAAATACCATTATTTGTTTGATCTGTTGGTATTACAGCATTACTTCTTCTCCTTTCTTTACCAGTATTAG
CAGGTGCTATTACTATATTACTAACTGATCGAAATTTAAATAC-------------------------------------
----
>GBGL12693-13|Scirpophaga_incertulas|KC012450|KC012450
TACTTTATATTTTATTTTTGGAATTTGAGCTGGTATAGTAGGAACTTCTTTAAGCTTACTTATTCGAGCTGAATTAGGAA
CTTCTGGATCCTTAATTGGAGATGATCAAATCTATTATACTATTGTCACAGCCCATGCCTTTATTATAATTTTTTTTATA
GTTATACCCATTATAATTGGAGGATTTGGAAATTGATTAGTCCCCCTAATATTAGGAGCTCCAGATATAGCTTTTCCGCG
ACTAAATTATATAAGATTCTGATTATTACCCCCCTCTTTAACACTTCTCATTTCTTGAAGAATTGTAGAAAGTGGGGCTG
GAACAGGATGAACTGTTTACCCACCTTTATCATCCAATATTGCTCATGGAGGAACATCAGTAGATTTAGCTATTTTTTCT
CTACACCTAGCAGGAATTTCATCTATTTTAGGAGCTATTAATTTTATTACAACCATTATTAATATACGAATTAATGGATT
ATCATTTGACCAAATACCTCTATTTGTGTGAGCTGTTGGTATTACAGCCCTTCTTTTACTTCTCTCTCTCCCAGTTTTAG
CTGGAGCTATTACCATATTACTAACAGATCGAAATTTAAATACATCTTTTTTTGACCCAGCTGGGGGAGGAGATCCAATT
TTATACCAACATTTATATTGATTTTTT
>GBGL12796-13|Diatraea_aff._consideratamagnifactella|JQ888386|JQ888386
AACTTTATACTTTATTTTTGGTATTTGAGCAGGAATGTTAGGAACATCACTAAGTCTTTTAATTCGAGCAGAATTAGGAA
CTCCTAATTCTTTAATTGGAGATGATCAAATTTATAATACAATTGTTACAGCTCATGCTTTTATTATAATTTTCTTTATA
GTAATACCAATTATAATTGGAGGATTTGGAAATTGATTAGTACCCTTAATATTAGGAGCTCCTGATATAGCTTTTCCTCG
AATAAATAATATAAGATTTTGATTATTACCCCCATCTTTAACTCTATTAATTTCTAGAAGAATTGTAGAAAATGGAGCTG
GAACAGGATGAACAGTTTACCCTCCTCTATCATCTAATATTGCCCATGGGGGTAGATCAGTAGATTTAGCAATTTTTTCT
TTACATTTAGCTGGAATTTCTTCAATTTTAGGAGCTATTAATTTTATTACTACTATTATTAATATACGAATTAATGGTTT
ATCATTTGATCAAATACCTTTATTTATTTGATCAGTAGGTATTACAGCTTTACTTTTATTGCTTTCTTTACCAGTATTAG
CTGGAGCTATTACTATACTATTAACAGACCGAAATTTAAATACTTCTTTTTTTGATCCAGCTGGAGGAGGAGATCCAATT
CTATATCAACATTTATTTTGATTTTTT
>GBGL12797-13|Diatraea_aff._consideratamagnifactella|JQ888385|JQ888385
AACTTTATACTTTATTTTTGGTATTTGAGCAGGAATGTTAGGAACATCACTAAGTCTTTTAATTCGAGCAGAATTAGGAA
CTCCTAATTCTTTAATTGGAGATGATCAAATTTATAATACAATTGTTACAGCTCATGCTTTTATTATAATTTTCTTTATA
GTAATACCAATTATAATTGGAGGATTTGGAAATTGATTAGTACCCTTAATATTAGGAGCTCCTGATATAGCTTTTCCTCG
AATAAATAATATAAGATTTTGATTATTACCCCCATCTTTAACTCTATTAATTTCTAGAAGAATTGTAGAAAATGGAGCTG
GAACAGGATGAACAGTTTACCCTCCTCTATCATCTAATATTGCCCATGGGGGTAGATCAGTAGATTTAGCAATTTTTTCT
TTACATTTAGCTGGAATTTCTTCAATTTTAGGAGCTATTAATTTTATTACTACTATTATTAATATACGAATTAATGGTTT
ATCATTTGATCAAATACCTTTATTTATTTGATCAGTAGGTATTACAGCTTTACTTTTATTGCTTTCTTTACCAGTATTAG
CTGGAGCTATTACTATACTATTAACAGACCGAAATTTAAATACTTCTTTTTTTGATCCAGCTGGAGGAGGAGATCCAATT
CTATATCAACATTTATTTTGATTTTTT
>GBGL12798-13|Diatraea_aff._consideratamagnifactella|JQ888384|JQ888384
---------------TTTTGGTATTTGAGCAGGAATGTTAGGAACATCACTAAGTCTTTTAATTCGAGCAGAATTAGGAA
CTCCTAATTCTTTAATTGGAGATGATCAAATTTATAATACAATTGTTACAGCTCATGCTTTTATTATAATTTTCTTTATA
GTAATACCAATTATAATTGGAGGATTTGGAAATTGATTAGTACCYTTAATATTAGGAGCTCCTGATATAGCTTTTCCTCG
AATAAATAATATAAGATTTTGATTATTACCCCCATCTTTAACTCTATTAATTTCTAGAAGAATTGTAGAAAATGGAGCTG

GAACAGGATGAACAGTTTACCCTCCTCTATCATCTAATATTGCCCATGGGGGTAGATCAGTAGATTTAGCAATTTTTTCT
TTACATTTAGCTGGAATTTCTTCAATTTTAGGAGCTATTAATTTTATTACTACTATTATTAATATACGAATTAATGGTTT
ATCATTTGATCAAATACCTTTATTTATTTGATCAGTAGGTATTACAGCTTTACTTTTATTGCTTTCTTTACCAGTATTAG
CTGGAGCTATTACTATACTATTAACAGACCGAAATTTAAATACTTCTTTTTTTGATCCAGCTGGAGGAGGAGATCCAATT
CTATANCAACATTTATTTTGATTTTTT
>GBGL12799-13|Diatraea_aff._consideratamagnifactella|JQ888383|JQ888383
---------------TTTTGGTATTTGAGCAGGAATGTTAGGAACATCACTAAGTCTTTTAATTCGAGCAGAATTAGGAA
CTCCTAATTCTTTAATTGGAGATGATCAAATTTATAATACAATTGTTACAGCTCATGCTTTTATTATAATTTTCTTTATA
GTAATACCAATTATAATTGGAGGATTTGGAAATTGATTAGTACCCTTAATATTAGGAGCTCCTGATATAGCTTTTCCTCG
AATAAATAATATAAGATTTTGATTATTACCCCCATCTTTAACTCTATTAATTTCTAGAAGAATTGTAGAAAATGGAGCTG
GAACAGGATGAACAGTTTACCCYCCTCTATCATCTAATATTGCCCATGGGGGWAGATCAGTAGATTTAGCAATTTTTTCT
TTRCATTTAGCTGGAATTTCTTCAATTTTAGGAGCTATTAATTTTATTACTACTATTATTAATATACGAATTAATGGTTT
ATCATTTGATCAAATACCTTTATTTATTTGATCAGTAGGTATTACAGCTTTACTTTTATTGCTTTCTTTACCAGTATTAG
CTGGAGCTATTACTATACTATTAACAGACCGAAATTTAAATACTTCTTTTTTTGATCCAGCTGGAGGAGGAGATCCAATT
CTATATCAACATTTATTTTGATTTTTT
>GBGL12800-13|Diatraea_aff._consideratamagnifactella|JQ888382|JQ888382
---------------TTTTGGTATTTGAGCAGGAATGTTAGGAACATCACTAAGTCTTTTAATTCGAGCAGAATTAGGAA
CTCCTAATTCTTTAATTGGAGATGATCAAATTTATAATACAATTGTTACAGCTCATGCTTTTATTATAATTTTCTTTATA
GTAATACCAATTATAATTGGAGGATTTGGAAATTGATTAGTACCCTTAATATTAGGAGCTCCTGATATAGCTTTTCCTCG
AATAAATAATATAAGATTTTGATTATTACCCCCATCTTTAACTCTATTAATTTCTAGAAGAATTGTAGAAAATGGAGCTG
GAACAGGATGAACAGTTTACCCTCCTCTATCATCTAATATTGCCCATGGGGGTAGATCAGTAGATTTAGCAATTTTTTCT
TTACATTTAGCTGGAATTTCTTCAATTTTAGGAGCTATTAATTTTATTACTACTATTATTAATATACGAATTAATGGTTT
ATCATTTGATCAAATACCTTTATTTATTTGATCAGTAGGTATTACAGCTTTACTTTTATTGCTTTCTTTACCAGTATTAG
CTGGAGCTATTACTATACTATTAACAGACCGAAATTTAAATACTTCTTTTTTTGATCCAGCTGGAGGAGGAGATCCAATT
CTATATCAACATTTATTTTGATTTTTT
>GBGL12801-13|Diatraea_aff._consideratamagnifactella|JQ888381|JQ888381
---------------TTTTGGTATTTGAGCAGGAATGTTAGGAACATCACTAAGTCTTTTAATTCGAGCAGAATTAGGAA
CTCCTAATTCTTTAATTGGAGATGATCAAATTTATAATACAATTGTTACAGCTCATGCTTTTATTATAATTTTCTTTATA
GTAATACCAATTATAATTGGAGGATTTGGAAATTGATTAGTACCCTTAATATTAGGAGCTCCTGATATAGCTTTTCCTCG
AATAAATAATATAAGATTTTGATTATTACCCCCATCTTTAACTCTATTAATTTCTAGAAGAATTGTAGAAAATGGAGCTG
GAACAGGATGAACAGTTTACCCTCCTCTATCATCTAATATTGCCCATGGGGGTAGATCAGTAGATTTAGCAATTTTTTCT
TTACATTTAGCTGGAATTTCTTCAATTTTAGGAGCTATTAATTTTATTACTACTATTATTAATATACGAATTAATGGTTT
ATCATTTGATCAAATACCTTTATTTATTTGATCAGTAGGTATTACAGCTTTACTTTTATTGCTTTCTTTACCAGTATTAG
CTGGAGCTATTACTATACTATTAACAGACCGAAATTTAAATACTTCTTTTTTTGATCCAGCTGGAGGAGGAGATCCAATT
CTATATCAACATTTATTTTGATTTTTT
>GBGL12802-13|Diatraea_aff._consideratamagnifactella|JQ888380|JQ888380
AACTTTATACTTTATTTTTGGTATTTGAGCAGGAATGTTAGGAACATCACTAAGTCTTTTAATTCGAGCAGAATTAGGAA
CTCCTAATTCTTTAATTGGAGATGATCAAATTTATAATACAATTGTTACAGCTCATGCTTTTATTATAATTTTCTTTATA
GTAATACCAATTATAATTGGAGGATTTGGAAATTGATTAGTACCCTTAATATTAGGAGCTCCTGATATAGCTTTTCCTCG
AATAAATAATATAAGATTTTGATTATTACCCCCATCTTTAACTCTATTAATTTCTAGAAGAATTGTAGAAAATGGAGCTG
GAACAGGATGAACAGTTTACCCTCCTCTATCATCTAATATTGCCCATGGGGGTAGATCAGTAGATTTAGCAATTTTTTCT
TTACATTTAGCTGGAATTTCTTCAATTTTAGGAGCTATTAATTTTATTACTACTATTATTAATATACGAATTAATGGTTT
ATCATTTGATCAAATACCTTTATTTATTTGATCAGTAGGTATTACAGCTTTACTTTTATTGCTTTCTTTACCAGTATTAG
CTGGAGCTATTACTATACTATTAACAGACCGAAATTTAAATACTTCTTTTTTTGATCCAGCTGGAGGAGGAGATCCAATT
CTATATCAACATTTATTTTGATTTTTT
>GBGL12803-13|Diatraea_aff._consideratamagnifactella|JQ888379|JQ888379
---------------TTTTGGTATTTGAGCAGGAATGTTAGGAACATCACTAAGTCTTTTAATTCGAGCAGAATTAGGAA
CTCCTAATTCTTTAATTGGAGATGATCAAATTTATAATACAATTGTTACAGCTCATGCTTTTATTATAATTTTCTTTATA
GTAATACCAATTATAATTGGAGGATTTGGAAATTGATTAGTACCCTTAATATTAGGAGCTCCTGATATAGCTTTTCCTCG
AATAAATAATATAAGATTTTGATTATTACCCCCATCTTTAACTCTATTAATTTCTAGAAGAATTGTAGAAAATGGAGCTG
GAACAGGATGAACAGTTTACCCTCCTCTATCATCTAATATTGCCCATGGGGGTAGATCAGTAGATTTAGCAATTTTTTCT
TTACATTTAGCTGGAATTTCTTCAATTTTAGGAGCTATTAATTTTATTACTACTATTATTAATATACGAATTAATGGTTT
ATCATTTGATCAAATACCTTTATTTATTTGATCAGTAGGTATTACAGCTTTACTTTTATTGCTTTCTTTACCAGTATTAG
CTGGAGCTATTACTATACTATTAACAGACCGAAATTTAAATACTTCTTTTTTTGATCCAGCTGGAGGAGGAGATCCAATT
CTATATCAACATTTATTTTGATTTTTT
>GBGL12804-13|Diatraea_aff._consideratamagnifactella|JQ888378|JQ888378
---------------TTTTGGTATTTGAGCAGGAATGTTAGGAACATCACTAAGTCTTTTAATTCGAGCAGAATTAGGAA

CTCCTAATTCTTTAATTGGAGATGATCAAATTTATAATACAATTGTTACAGCTCATGCTTTTATTATAATTTTCTTTATA
GTAATACCAATTATAATTGGAGGATTTGGAAATTGATTAGTACCCTTAATATTAGGAGCTCCTGATATAGCTTTTCCTCG
AATAAATAATATAAGATTTTGATTATTACCCCCATCTTTAACTCTATTAATTTCTAGAAGAATTGTAGAAAATGGAGCTG
GAACAGGATGAACAGTTTACCCTCCTCTATCATCTAATATTGCCCATGGGGGTAGATCAGTAGATTTAGCAATTTTTTCT
TTACATTTAGCTGGAATTTCTTCAATTTTAGGAGCTATTAATTTTATTACTACTATTATTAATATACGAATTAATGGTTT
ATCATTTGATCAAATACCTTTATTTATTTGATCAGTAGGTATTACAGCTTTACTTTTATTGCTTTCTTTACCAGTATTAG
CTGGAGCTATTACTATACTATTAACAGACCGAAATTTAAATACTTCTTTTTTTGATCCAGCTGGAGGAGGAGATCCAATT
CTATATCAACATTTATTTTGATTTTTT
>GBGL12805-13|Diatraea_aff._consideratamagnifactella|JQ888377|JQ888377
--------------TTTTGGTATTTGAGCAGGAATGTTAGGAACATCNCTAAGTCTTTTAATTCGAGCAGAATTAGGAA
CTCCTAATTCTTTAATTGGAGATGATCAAATTTATAATACAATTGTTACAGCTCATGCTTTTATTATAATTTTCTTTATA
GTAATACCAATTATAATTGGAGGATTTGGAAATTGATTAGTACCYTTAATATTAGGAGCTCCTGATATAGCTTTTCCTCG
AATAAATAAYATAAGATTTTGATTATTACCCCCATCTTTAACTCTATTAATTTCTAGAAGAATTGTAGAAAATGGAGCTG
GAACAGGATGAACAGTTTACCCYCCTCTATCATCTAATATTGCCCATGGGGGWAGATCAGTAGATTTAGCAATTTTTTCT
TTRCATTTAGCTGGAATTTCTTCAATTTTAGGAGCTATTAATTTTATTACTACTATTATTAATATACGAATTAATGGTTT
ATCATTTGATCAAATACCTTTATTTATTTGATCAGTAGGTATTACAGCTTTACTTTTATTGCTTTCTTTACCGGTATTAG
CTGGAGCTATTACTATACTATTAACAGACCGAAATTTAAATACTTCTTTTTTTGATCCAGCTGGAGGAGGAGATCCAATT
CTATATCAACATTTATTTTGATTTTTT
>GBGL12806-13|Diatraea_aff._consideratamagnifactella|JQ888376|JQ888376
--------------TTTTGGTATTTGAGCAGGAATGTTAGGAACATCACTAAGTCTTTTAATTCGAGCAGAATTAGGAA
CTCCTAATTCTTTAATTGGAGATGATCAAATTTATAATACAATTGTTACAGCTCATGCTTTTATTATAATTTTCTTTATA
GTAATACCAATTATAATTGGAGGATTTGGAAATTGATTAGTACCTTTAATATTAGGGGCTCCTGATATAGCTTTCCCTCG
AATAAATAACATAAGATTTTGATTATTACCTCCATCCTTAACTCTATTAATTTCTAGAAGAATTGTAGAAAATGGAGCTG
GAACAGGATGAACAGTTTACCCCCCTTTATCATCTAATATTGCCCACGGAGGAAGATCAGTAGATTTAGCAATTTTTTCT
TTACATTTAGCTGGAATTTCTTCAATTTTAGGAGCTATTAATTTTATTACTACTATTATTAATATACGAATTAATGGTTT
ATCATTTGATCAAATACCTTTATTTATTTGATCAGTAGGTATTACAGCTTTACTTTTATTACTTTCTTTACCAGTATTAG
CTGGAGCTATTACTATACTATTAACAGATCGAAATTTAAATACTTCTTTTTTTGATCCAGCTGGAGGAGGAGATCCAATT
CTATACCAACATTTATTTTGATTTTTT
>GBGL12807-13|Diatraea_aff._consideratamagnifactella|JQ888375|JQ888375
--------------TTTTGGTATTTGAGCAGGAATGTTAGGAACATCACTAAGTCTTTTAATTCGAGCAGAATTAGGAA
CTCCTAATTCTTTAATTGGAGATGATCAAATTTATAATACAATTGTTACAGCTCATGCTTTTATTATAATTTTCTTTATA
GTAATACCAATTATAATTGGAGGATTTGGAAATTGATTAGTACCTTTAATATTAGGGGCTCCTGATATAGCTTTCCCTCG
AATAAATAACATAAGATTTTGATTATTACCTCCATCCTTAACTCTATTAATTTCTAGAAGAATTGTAGAAAATGGAGCTG
GAACAGGATGAACAGTTTACCCCCCTTTATCATCTAATATTGCCCACAGAGGAAGATCAGTAGATTTAGCAATTTTTTCT
TTACATTTAGCTGGAATTTCTTCAATTTTAGGAGCTATTAATTTTATTACTACTATTATTAATATACGAATTAATGGTTT
ATCATTTGATCAAATACCTTTATTTATTTGATCAGTAGGTATTACAGCTTTACTTTTATTACTTTCTTTACCAGTATTAG
CTGGAGCTATTACTATACTATTAACAGATCGAAATTTAAATACTTCTTTTTTTGATCCAGCTGGAGGAGGAGATCCAATT
CTATACCAACATTTATTTTGATTTTTT
>GBGL12808-13|Diatraea_aff._consideratamagnifactella|JQ888374|JQ888374
--------------TTTTGGTATTTGAGCAGGAATGTTAGGAACATCACTAAGTCTTTTAATTCGAGCAGAATTAGGAA
CTCCTAATTCTTTAATTGGAGATGATCAAATTTATAATACAATTGTTACAGCTCATGCTTTTATTATAATTTTCTTTATA
GTAATACCAATTATAATTGGAGGATTTGGAAATTGATTAGTACCCTTAATATTAGGAGCTCCTGATATAGCTTTTCCTCG
AATAAATAATATAAGATTTTGATTATTACCCCCATCTTTAACTCTATTAATTTCTAGAAGAATTGTAGAAAATGGAGCTG
GAACAGGATGAACAGTTTACCCTCCTCTATCATCTAATATTGCCCATGGGGGTAGATCAGTAGATTTAGCAATTTTTTCT
TTACATTTAGCTGGAATTTCTTCAATTTTAGGAGCTATTAATTTTATTACTACTATTATTAATATACGAATTAATGGTTT
ATCATTTGATCAAATACCTTTATTTATTTGATCAGTAGGTATTACAGCTTTACTTTTATTGCTTTCTTTACCAGTATTAG
CTGGAGCTATTACTATACTATTAACAGACCGAAATTTAAATACTTCTTTTTTTGATCCAGCTGGAGGAGGAGATCCAATT
CTATATCAACATTTATTTTGATTTTTT
>GBGL12809-13|Diatraea_aff._consideratamagnifactella|JQ888373|JQ888373
--------------TTTTGGTATTTGAGCAGGAATGTTAGGAACATCACTAAGTCTTTTAATTCGAGCAGAATTAGGAA
CTCCTAATTCTTTAATTGGAGATGATCAAATTTATAATACAATTGTTACAGCTCATGCTTTTATTATAATTTTCTTTATA
GTAATACCAATTATAATTGGAGGATTTGGAAATTGATTAGTACCCTTAATATTAGGAGCTCCTGATATAGCTTTTCCTCG
AATAAATAATATAAGATTTTGATTATTACCCCCATCTTTAACTCTATTAATTTCTAGAAGAATTGTAGAAAATGGAGCTG
GAACAGGATGAACAGTTTACCCTCCTCTATCATCTAATATTGCCCATGGGGGTAGATCAGTAGATTTAGCAATTTTTTCT
TTACATTTAGCTGGAATTTCTTCAATTTTAGGAGCTATTAATTTTATTACTACTATTATTAATATACGAATTAATGGTTT
ATCATTTGATCAAATACCTTTATTTATTTGATCAGTAGGTATTACAGCTTTACTTTTATTGCTTTCTTTACCAGTATTAG
CTGGAGCTATTACTATACTATTAACAGACCGAAATTTAAATACTTCTTTTTTTGATCCAGCTGGAGGAGGAGATCCAATT

CTATATCAACATTTATTTTGATTTTTT
>GBGL12810-13|Diatraea_aff._consideratamagnifactella|JQ888372|JQ888372
--------------TTTTGGTATTTGAGCAGGAATGTTAGGAACATCACTAAGTCTTTTAATTCGAGCAGAATTAGGAA
CTCCTAATTCTTTAATTGGAGATGATCAAATTTATAATACAATTGTTACAGCTCATGCTTTTATTATAATTTTCTTTATA
GTAATACCAATTATAATTGGAGGATTTGGAAATTGATTAGTACCCTTAATATTAGGAGCTCCTGATATAGCTTTTCCTCG
AATAAATAATATAAGATTTTGATTATTACCCCCATCTTTAACTCTATTAATTTCTAGAAGAATTGTAGAAAATGGAGCTG
GAACAGGATGAACAGTTTACCCTCCTCTATCATCTAATATTGCCCATGGGGGTAGATCAGTAGATTTAGCAATTTTTTCT
TTACATTTAGCTGGAATTTCTTCAATTTTAGGAGCTATTAATTTTATTACTACTATTATTAATATACGAATTAATGGTTT
ATCATTTGATCAAATACCTTTATTTATTTGATCAGTAGGTATTACAGCTTTACTTTTATTGCTTTCTTTACCAGTATTAG
CTGGAGCTATTACTATACTATTAACAGACCGAAATTTAAATACTTCTTTTTTTGATCCAGCTGGAGGAGGAGATCCAATT
CTATATCAACATTTATTTTGATTTTTT
>GBGL12811-13|Diatraea_aff._consideratamagnifactella|JQ888371|JQ888371
--------------TTTTGGTATTTGAGCAGGAATGTTAGGAACATCACTAAGTCTTTTAATTCGAGCAGAATTAGGAA
CTCCTAATTCTTTAATTGGAGATGATCAAATTTATAATACAATTGTTACAGCTCATGCTTTTATTATAATTTTCTTTATA
GTAATACCAATTATAATTGGAGGATTTGGAAATTGATTAGTACCCTTAATATTAGGAGCTCCTGATATAGCTTTTCCTCG
AATAAATAATATAAGATTTTGATTATTACCCCCATCTTTAACTCTATTAATTTCTAGAAGAATTGTAGAAAATGGAGCTG
GAACAGGATGAACAGTTTACCCTCCTCTATCATCTAATATTGCCCATGGGGGTAGATCAGTAGATTTAGCAATTTTTTCT
TTACATTTAGCTGGAATTTCTTCAATTTTAGGAGCTATTAATTTTATTACTACTATTATTAATATACGAATTAATGGTTT
ATCATTTGATCAAATACCTTTATTTATTTGATCAGTAGGTATTACAGCTTTACTTTTATTGCTTTCTTTACCAGTATTAG
CTGGAGCTATTACTATACTATTAACAGACCGAAATTTAAATACTTCTTTTTTTGATCCAGCTGGAGGAGGAGATCCAATT
CTATATCAACATTTATTTTGATTTTTT
>GBGL12812-13|Diatraea_aff._consideratamagnifactella|JQ888370|JQ888370
---ATCACTAAGTCTTTTAATTCGAGCAGAATTAGGAA
CTCCTAATTCTTTAATTGGAGATGATCAAATTTATAATACAATTGTTACAGCTCATGCTTTTATTATAATTTTCTTTATA
GTAATACCAATTATAATTGGAGGATTTGGAAATTGATTAGTACCCTTAATATTAGGAGCTCCTGATATAGCTTTTCCTCG
AATAAATAATATAAGATTTTGATTATTACCCCCATCTTTAACTCTATTAATTTCTAGAAGAATTGTAGAAAATGGAGCTG
GAACAGGATGAACAGTTTACCCTCCTCTATCATCTAATATTGCCCATGGGGGTAGATCAGTAGATTTAGCAATTTTTTCT
TTACATTTAGCTGGAATTTCTTCAATTTTAGGAGCTATTAATTTTATTACTACTATTATTAATATACGAATTAATGGTTT
ATCATTTGATCAAATACCTTTATTTATTTGATCAGTAGGTATTACAGCTTTACTTTTATTGCTTTCTTTACCAGTATTAG
CTGGAGCTATTACTATACTATTAACAGACCGAAATTTAAATACTTCTTTTTTTGATCCAGCTGGAGGAGGAGATCCAATT
CTATATCAACATTTATTTTGATTTTTT
>GBGL12813-13|Diatraea_aff._consideratamagnifactella|JQ888369|JQ888369
--------------TTTTGGTATTTGAGCAGGAATGTTAGGAACATCACTAAGTCTTTTAATTCGAGCAGAATTAGGAA
CTCCTAATTCTTTAATTGGAGATGATCAAATTTATAATACAATTGTTACAGCTCATGCTTTTATTATAATTTTCTTTATA
GTAATACCAATTATAATTGGAGGATTTGGAAATTGATTAGTACCCTTAATATTAGGAGCTCCTGATATAGCTTTTCCTCG
AATAAATAATATAAGATTTTGATTATTACCCCCATCTTTAACTCTATTAATTTCTAGAAGAATTGTAGAAAATGGAGCTG
GAACAGGATGAACAGTTTACCCTCCTCTATCATCTAATATTGCCCATGGGGGTAGATCAGTAGATTTAGCAATTTTTTCT
TTACATTTAGCTGGAATTTCTTCAATTTTAGGAGCTATTAATTTTATTACTACTATTATTAATATACGAATTAATGGTTT
ATCATTTGATCAAATACCTTTATTTATTTGATCAGTAGGTATTACAGCTTTACTTTTATTGCTTTCTTTACCAGTATTAG
CTGGAGCTATTACTATACTATTAACAGACCGAAATTTAAATACTTCTTTTTTTGATCCAGCTGGAGGAGGAGATCCAATT
CTATATCAACATTTATTTTGATTTTTT
>GBGL12814-13|Diatraea_aff._consideratamagnifactella|JQ888368|JQ888368
--------------TTTTGGTATTTGAGCAGGAATGTTAGGAACATCACTAAGTCTTTTAATTCGAGCAGAATTAGGAA
CTCCTAATTCTTTAATTGGAGATGATCAAATTTATAATACAATTGTTACAGCTCATGCTTTTATTATAATTTTCTTTATA
GTAATACCAATTATAATTGGAGGATTTGGAAATTGATTAGTACCCTTAATATTAGGAGCTCCTGATATAGCTTTTCCTCG
AATAAATAATATAAGATTTTGATTATTACCCCCATCTTTAACTCTATTAATTTCTAGAAGAATTGTAGAAAATGGAGCTG
GAACAGGATGAACAGTTTACCCTCCTCTATCATCTAATATTGCCCATGGGGGTAGATCAGTAGATTTAGCAATTTTTTCT
TTACATTTAGCTGGAATTTCTTCAATTTTAGGAGCTATTAATTTTATTACTACTATTATTAATATACGAATTAATGGTTT
ATCATTTGATCAAATACCTTTATTTATTTGATCAGTAGGTATTACAGCTTTACTTTTATTGCTTTCTTTACCAGTATTAG
CTGGAGCTATTACTATACTATTAACAGACCGAAATTTAAATACTTCTTTTTTTGATCCAGCTGGAGGAGGAGATCCAATT
CTATATCAACATTTATTTTGATTTTTT
>GBGL12815-13|Diatraea_aff._consideratamagnifactella|JQ888367|JQ888367
--------------TTTTGGTATTTGAGCAGGAATGTTAGGAACATCACTAAGTCTTTTAATTCGAGCAGAATTAGGAA
CTCCTAATTCTTTAATTGGAGATGATCAAATTTATAATACAATTGTTACAGCTCATGCTTTTATTATAATTTTCTTTATA
GTAATACCAATTATAATTGGAGGATTTGGAAATTGATTAGTACCCTTAATATTAGGAGCTCCTGATATAGCTTTTCCTCG
AATAAATAATATAAGATTTTGATTATTACCCCCATCTTTAACTCTATTAATTTCTAGAAGAATTGTAGAAAATGGAGCTG
GAACAGGATGAACAGTTTACCCTCCTCTATCATCTAATATTGCCCATGGGGGTAGATCAGTAGATTTAGCAATTTTTTCT

TTACATTTAGCTGGAATTTCTTCAATTTTAGGAGCTATTAATTTTATTACTACTATTATTAATATACGAATTAATGGTTT
ATCATTTGATCAAATACCTTTATTTATTTGATCAGTAGGTATTACAGCTTTACTTTTATTGCTTTCTTTACCAGTATTAG
CTGGAGCTATTACTATACTATTAACAGACCGAAATTTAAATACTTCTTTTTTTGATCCAGCTGGAGGAGGAGATCCAATT
CTATATCAACATTTATTTTGATTTTTT
>GBGL12816-13|Diatraea_considerata|JQ888366|JQ888366
AACTTTATACTTTATTTTTGGTATTTGAGCAGGAATGTTAGGAACATCATTAAGTCTTTTAATTCGAGCAGAATTAGGAA
CTCCTAATTCTTTAATTGGAGATGATCAAATTTATAATACAATTGTTACAGCTCATGCTTTTATTATAATTTTCTTTATA
GTAATACCAATTATAATTGGAGGATTTGGTAATTGATTAGTACCCTTAATATTAGGAGCTCCTGATATAGCTTTTCCTCG
AATAAATAACATAAGATTTTGATTATTACCCCCATCTTTAACTCTATTAATTTCTAGAAGAATTGTAGAAAATGGAGCTG
GAACAGGATGAACAGTTTACCCCCCTCTATCATCTAATATTGCCCATGGAGGTAGATCAGTAGATTTAGCAATTTTTTCT
TTACATTTAGCTGGAATTTCTTCAATTTTAGGAGCTATTAATTTTATTACTACTATTATTAATATACGAATTAATGGTTT
ATCATTTGATCAAATACCTTTATTTATTTGATCAGTAGGTATTACAGCTTTACTTTTATTGCTTTCTTTACCAGTATTAG
CTGGAGCTATTACTATACTATTAACAGACCGAAATTTAAATACTTCTTTTTTTGATCCAGCTGGAGGAGGAGATCCAATT
CTATATCAACATTTATTTTGATTTTTT
>GBGL12817-13|Diatraea_considerata|JQ888365|JQ888365
AACTTTATACTTTATTTTTGGTATTTGAGCAGGAATGTTAGGAACATCATTAAGTCTTTTAATTCGAGCAGAATTAGGAA
CTCCTAATTCTTTAATTGGAGATGATCAAATTTATAATACAATTGTTACAGCTCATGCTTTTATTATAATTTTCTTTATA
GTAATACCAATTATAATTGGAGGATTTGGTAATTGATTAGTACCCTTAATATTAGGAGCTCCTGATATAGCTTTTCCTCG
AATAAATAACATAAGATTTTGATTATTACCCCCATCTTTAACTCTATTAATTTCTAGAAGAATTGTAGAAAATGGAGCTG
GAACAGGATGAACAGTTTACCCCCCTCTATCATCTAATATTGCCCATGGAGGTAGATCAGTAGATTTAGCAATTTTTTCT
TTACATTTAGCTGGAATTTCTTCAATTTTAGGAGCTATTAATTTTATTACTACTATTATTAATATACGAATTAATGGTTT
ATCATTTGATCAAATACCTTTATTTATTTGATCAGTAGGTATTACAGCTTTACTTTTATTGCTTTCTTTACCAGTATTAG
CTGGAGCTATTACTATACTATTAACAGACCGAAATTTAAATACTTCTTTTTTTGATCCAGCTGGAGGAGGAGATCCAATT
CTATATCAACATTTATTTTGATTTTTT
>GBGL12818-13|Diatraea_considerata|JQ888364|JQ888364
AACTTTATACTTTATTTTTGGTATTTGAGCAGGAATATTAGGAACATCACTAAGTCTTTTAATTCGAGCAGAATTAGGAA
CTCCTAATTCTTTAATTGGAGATGATCAAATTTATAATACAATTGTTACAGCTCATGCTTTTATTATAATTTTCTTTATA
GTAATACCAATTATAATTGGAGGATTTGGAAATTGATTAGTACCTTTAATATTAGGAGCTCCTGATATAGCTTTTCCTCG
AATAAATAACATAAGATTTTGATTATTACCCCCATCTTTAACTCTATTAATTTCTAGAAGAATTGTAGAAAATGGAGCTG
GAACAGGATGAACAGTTTACCCTCCTCTATCATCTAATATTGCCCATGGGGGTAGATCAGTAGATTTAGCAATTTTTTCT
TTACATTTAGCTGGAATTTCTTCAATTTTAGGAGCTATTAATTTTATTACTACTATTATTAATATACGAATTAATGGTTT
ATCATTTGATCAAATACCTTTATTTATTTGATCAGTAGGTATTACAGCTTTACTTTTATTGCTTTCTTTACCAGTATTAG
CTGGAGCTATTACTATACTATTAACAGACCGAAATTTAAATACTTCTTTTTTTGATCCAGCTGGAGGAGGAGATCCAATT
CTATATCAACATTTATTTTGATTTTTT
>GBGL12819-13|Diatraea_considerata|JQ888363|JQ888363
AACTTTATACTTTATTTTTGGTATTTGAGCAGGAATATTAGGAACATCACTAAGTCTTTTAATTCGAGCAGAATTAGGAA
CTCCTAATTCTTTAATTGGAGATGATCAAATTTATAATACAATTGTTACAGCTCATGCTTTTATTATAATTTTCTTTATA
GTAATACCAATTATAATTGGAGGATTTGGAAATTGATTAGTACCTTTAATATTAGGAGCTCCTGATATAGCTTTTCCTCG
AATAAATAACATAAGATTTTGATTATTACCCCCATCTTTAACTCTATTAATTTCTAGAAGAATTGTAGAAAATGGAGCTG
GAACAGGATGAACAGTTTACCCTCCTCTATCATCTAATATTGCCCATGGGGGTAGATCAGTAGATTTAGCAATTTTTTCT
TTACATTTAGCTGGAATTTCTTCAATTTTAGGAGCTATTAATTTTATTACTACTATTATTAATATACGAATTAATGGTTT
ATCATTTGATCAAATACCTTTATTTATTTGATCAGTAGGTATTACAGCTTTACTTTTATTGCTTTCTTTACCAGTATTAG
CTGGAGCTATTACTATACTATTAACAGACCGAAATTTAAATACTTCTTTTTTTGATCCAGCTGGAGGAGGAGATCCAATT
CTATATCAACATTTATTTTGATTTTTT
>GBGL12820-13|Diatraea_considerata|JQ888362|JQ888362
AACTTTATACTTTATTTTTGGTATTTGAGCAGGAATATTAGGAACATCACTAAGTCTTTTAATTCGAGCAGAATTAGGAA
CTCCTAATTCTTTAATTGGAGATGATCAAATTTATAATACAATTGTTACAGCTCATGCTTTTATTATAATTTTCTTTATA
GTAATACCAATTATAATTGGAGGATTTGGAAATTGATTAGTACCTTTAATATTAGGAGCTCCTGATATAGCTTTTCCTCG
AATAAATAACATAAGATTTTGATTATTACCCCCATCTTTAACTCTATTAATTTCTAGAAGAATTGTAGAAAATGGAGCTG
GAACAGGATGAACAGTTTACCCTCCTCTATCATCTAATATTGCCCATGGGGGTAGATCAGTAGATTTAGCAATTTTTTCT
TTACATTTAGCTGGAATTTCTTCAATTTTAGGAGCTATTAATTTTATTACTACTATTATTAATATACGAATTAATGGTTT
ATCATTTGATCAAATACCTTTATTTATTTGATCAGTAGGTATTACAGCTTTACTTTTATTGCTTTCTTTACCAGTATTAG
CTGGAGCTATTACTATACTATTAACAGACCGAAATTTAAATACTTCTTTTTTTGATCCAGCTGGAGGAGGAGATCCAATT
CTATATCAACATTTATTTTGATTTTTT
>GBGL12821-13|Diatraea_considerata|JQ888361|JQ888361
AACTTTATACTTTATTTTTGGTATTTGAGCAGGAATGTTAGGAACATCATTAAGTCTTTTAATTCGAGCAGAATTAGGAA
CTCCTAATTCTTTAATTGGAGATGATCAAATTTATAATACAATTGTTACAGCTCATGCTTTTATTATAATTTTCTTTATA

GTAATACCAATTATAATTGGAGGATTTGGTAATTGATTAGTACCCTTAATATTAGGAGCTCCTGATATAGCTTTTCCTCG
AATAAATAACATAAGATTTTGATTATTACCCCCATCTTTAACTCTATTAATTTCTAGAAGAATTGTAGAAAATGGAGCTG
GAACAGGATGAACAGTTTACCCCCCTCTATCATCTAATATTGCCCATGGAGGTAGATCAGTAGATTTAGCAATTTTTTCT
TTACATTTAGCTGGAATTTCTTCAATTTTAGGAGCTATTAATTTTATTACTACTATTATTAATATACGAATTAATGGTTT
ATCATTTGATCAAATACCTTTATTTATTTGATCAGTAGGTATTACAGCTTTACTTTTATTGCTTCTTTACCAGTATTAG
CTGGAGCTATTACTATACTATTAACAGACCGAAATTTAAATACTTCTTTTTTTGATCCAGCTGGAGGAGGAGATCCAATT
CTATATCAACATTTATTTTGATTTTTT
>GBGL12822-13|Diatraea_saccharalis|JQ888360|JQ888360
-----------TTATTTTTGGAATTTGAGCAGGAATGTTAGGAACTTCCTTAAGCTTATTAATTCGAGCAGAATTAGGAA
CATCTAACTCTTTAATTGGAGATGATCAAATTTATAACACAATTGTAACAGCTCATGCTTTCATTATAATTTTTTTTATA
GTTATACCTATTATAATTGGAGGATTTGGAAATTGATTAGTACCTTTAATGTTAGGGGCCCCCGATATAGCTTTCCCACG
AATAAATAATATAAGATTTTGACTATTACCCCCATCTTTAACTCTACTAATTTCTAGAAGAATTGTAGAAAATGGAGCAG
GGACAGGATGAACAGTTTATCCCCCATTATCATCCAATATTGCCCATGGGGGAAGATCTGTGGATTTAGCAATTTTCTCT
TTACATTTAGCTGGAATTTCCTCAATTTTAGGGGCTATCAATTTCATCACTACCATTATTAATATACGAATTAATAATCT
ATCATTTGATCAAATACCTTTATTTATTTGATCAGTGGGTATTACAGCATTACTTTTATTACTTTCTTTACCGGTATTAG
CTGGAGCCATTACTATATTACTAACAGATCGAAATTTAAATACCTCCTTCTTTGATCCAGCAGGGGGGGGAGATCCTATT
CTATATCAACATTTATTTTGATTTTTT
>GBGL12823-13|Diatraea_saccharalis|JQ888359|JQ888359
----------TTTATTTTTGGAATTTGAGCAGGAATGTTAGGAACTTCCTTAAGCTTATTAATTCGAGCAGAATTAGGAA
CATCTAACTCTTTAATTGGAGATGATCAAATTTATAACACAATTGTAACAGCTCATGCTTTCATTATAATTTTTTTTATA
GTTATACCTATTATAATTGGAGGATTTGGAAATTGATTAGTACCTTTAATGTTAGGGGCCCCCGATATAGCTTTCCCACG
AATAAATAATATAAGATTTTGACTATTACCCCCATCTTTAACTCTACTAATTTCTAGAAGAATTGTAGAAAATGGAGCAG
GGACAGGATGAACAGTTTATCCCCCATTATCATCCAATATTGCCCATGGGGGAAGATCTGTGGATTTAGCAATTTTCTCT
TTACATTTAGCTGGAATTTCCTCAATTTTAGGGGCTATCAATTTCATCACTACCATTATTAATATACGAATTAATAATCT
ATCATTTGATCAAATACCTTTATTTATTTGATCAGTGGGTATTACAGCATTACTTTTATTACTTTCTTTACCGGTATTAG
CTGGAGCCATTACTATATTACTAACAGATCGAAATTTAAATACCTCCTTCTTTGATCCAGCAGGGGGGGGAGATCCTATT
CTATATCAACATTTATTTTGATTTTTT
>GBGL12824-13|Diatraea_saccharalis|JQ888358|JQ888358
-----------TTATTTTTGGAATTTGAGCAGGAATGTTAGGAACTTCCTTAAGCTTATTAATTCGAGCAGAATTAGGAA
CATCTAACTCTTTAATTGGAGATGATCAAATTTATAACACAATTGTAACAGCTCATGCTTTCATTATAATTTTTTTTATA
GTTATACCTATTATAATTGGAGGATTTGGAAATTGATTAGTACCTTTAATGTTAGGGGCCCCCGATATAGCTTTCCCACG
AATAAATAATATAAGATTTTGACTATTACCCCCATCTTTAACTCTACTAATTTCTAGAAGAATTGTAGAAAATGGAGCAG
GGACAGGATGAACAGTTTATCCCCCATTATCATCCAATATTGCCCATGGGGGAAGATCTGTGGATTTAGCAATTTTCTCT
TTACATTTAGCTGGAATTTCCTCAATTTTAGGGGCTATCAATTTCATCACTACCATTATTAATATACGAATTAATAATCT
ATCATTTGATCAAATACCTTTATTTATTTGATCAGTGGGTATTACAGCATTACTTTTATTACTTTCTTTACCGGTATTAG
CTGGAGCCATTACTATATTACTAACAGATCGAAATTTAAATACCTCCTTCTTTGATCCAGCAGGGGGGGGAGATCCTATT
CTATATCAACATTTATTTTGATTTTTT
>GBGL12825-13|Diatraea_saccharalis|JQ888357|JQ888357
-----------TTATTTTTGGAATTTGAGCAGGAATGTTAGGAACTTCCTTAAGCTTATTAATTCGAGCAGAATTAGGAA
CATCTAACTCTTTAATTGGAGATGATCAAATTTATAACACAATTGTAACAGCTCATGCTTTCATTATAATTTTTTTTATA
GTTATACCTATTATAATTGGAGGATTTGGAAATTGATTAGTACCTTTAATGTTAGGGGCCCCCGATATAGCTTTCCCACG
AATAAATAATATAAGATTTTGACTATTACCCCCATCTTTAACTCTACTAATTTCTAGAAGAATTGTAGAAAATGGAGCAG
GGACAGGATGAACAGTTTATCCCCCATTATCATCCAATATTGCCCATGGGGGAAGATCTGTGGATTTAGCAATTTTCTCT
TTACATTTAGCTGGAATTTCCTCAATTTTAGGGGCTATCAATTTCATCACTACCATTATTAATATACGAATTAATAATCT
ATCATTTGATCAAATACCTTTATTTATTTGATCAGTGGGTATTACAGCATTACTTTTATTACTTTCTTTACCGGTATTAG
CTGGAGCCATTACTATATTACTAACAGATCGAAATTTAAATACCTCCTTCTTTGATCCAGCAGGGGGGGGAGATCCTATT
CTATATCAACATTTATTTTGATTTTTT
>GBGL12826-13|Diatraea_saccharalis|JQ888356|JQ888356
AACTTTATATTTTATTTTTGGAATTTGAGCAGGAATGTTAGGAACTTCCTTAAGCTTATTAATTCGAGCAGAATTAGGAA
CATCTAACTCTTTAATTGGAGATGATCAAATTTATAACACAATTGTAACAGCTCATGCTTTCATTATAATTTTTTTTATA
GTTATACCTATTATAATTGGAGGATTTGGAAATTGATTAGTACCTTTAATGTTAGGGGCCCCCGATATAGCTTTCCCACG
AATAAATAATATAAGATTTTGACTATTACCCCCATCTTTAACTCTACTAATTTCTAGAAGAATTGTAGAAAATGGAGCAG
GGACAGGATGAACAGTTTATCCCCCATTATCATCCAATATTGCCCATGGGGGAAGATCTGTGGATTTAGCAATTTTCTCT
TTACATTTAGCTGGAATTTCCTCAATTTTAGGGGCTATCAATTTCATCACTACCATTATTAATATACGAATTAATAATCT
ATCATTTGATCAAATACCTTTATTTATTTGATCAGTGGGTATTACAGCATTACTTTTATTACTTTCTTTACCGGTATTAG
CTGGAGCCATTACTATATTACTAACAGATCGAAATTTAAATACCTCCTTCTTTGATCCAGCAGGGGGGGGAGATCCTATT
CTATATCAACATTTATTTTGATTTTTT

>GBGL12827-13|Diatraea_saccharalis|JQ888355|JQ888355
AACTTTATATTTTATTTTTGGAATTTGAGCAGGAATGTTAGGAACTTCCTTAAGCTTATTAATTCGAGCAGAATTAGGAA
CATCTAACTCTTTAATTGGAGATGATCAAATTTATAACACAATTGTAACAGCTCATGCTTTCATTATAATTTTTTTTATA
GTTATACCTATTATAATTGGAGGATTTGGAAATTGATTAGTACCTTTAATGTTAGGGGCCCCCGATATAGCTTTCCCACG
AATAAATAATATAAGATTTTGACTATTACCCCCATCTTTAACTCTACTAATTTCTAGAAGAATTGTAGAAAATGGAGCAG
GGACAGGATGAACAGTTTATCCCCCATTATCATCCAATATTGCCCATGGGGGAAGATCTGTGGATTTAGCAATTTTCTCT
TTACATTTAGCTGGAATTTCCTCAATTTTAGGGGCTATCAATTTCATCACTACCATTATTAATATACGAATTAATAATCT
ATCATTTGATCAAATACCTTTATTTATTTGATCAGTGGGTATTACAGCATTACTTTTATTACTTTCTTTACCGGTATTAG
CTGGAGCCATTACTATATTACTAACAGATCGAAATTTAAATACCTCCTTCTTTGATCCAGCAGGGGGGGGAGATCCTATT
CTATATCAACATTTATTTTGATTTTTT
>GBGL12828-13|Diatraea_saccharalis|JQ888354|JQ888354
AACTTTATATTTTATTTTTGGAATTTGAGCAGGAATGTTAGGAACTTCCTTAAGCTTATTAATTCGAGCAGAATTAGGAA
CATCTAACTCTTTAATTGGAGATGATCAAATTTATAACACAATTGTAACAGCTCATGCTTTCATTATAATTTTTTTTATA
GTTATACCTATTATAATTGGAGGATTTGGAAATTGATTAGTACCTTTAATGTTAGGGGCCCCCGATATAGCTTTCCCACG
AATAAATAATATAAGATTTTGACTATTACCCCCATCTTTAACTCTACTAATTTCTAGAAGAATTGTAGAAAATGGAGCAG
GGACAGGATGAACAGTTTATCCCCCATTATCATCCAATATTGCCCATGGGGGAAGATCTGTGGATTTAGCAATTTTCTCT
TTACATTTAGCTGGAATTTCCTCAATTTTAGGGGCTATCAATTTCATCACTACCATTATTAATATACGAATTAATAATCT
ATCATTTGATCAAATACCTTTATTTATTTGATCAGTGGGTATTACAGCATTACTTTTATTACTTTCTTTACCGGTATTAG
CTGGAGCCATTACTATATTACTAACAGATCGAAATTTAAATACCTCCTTCTTTGATCCAGCAGGGGGGGGAGATCCTATT
CTATATCAACATTTATTTTGATTTTTT
>GBGL12829-13|Diatraea_grandiosella|JQ888353|JQ888353
--------------TATTTGGAATTTGAGCAGGAATATTAGGAACATCTCTAAGTTTACTAATTCGTGCTGAACTCGGTA
CACCTAATTCTTTAATTGGTGATGATCAAATTTATAATACAATTGTTACAGCTCATGCATTTATTATAATTTTTTTTATA
GTTATACCTATTATAATTGGGGGATTTGGAAATTGATTAGTACCTCTTATATTAGGGGCTCCGGATATGGCTTTCCCACG
AATAAATAATATAAGATTTTGATTATTACCCCCATCTTTAACTTTATTAATTTCCAGAAGAATCGTTGAAAATGGAGCTG
GAACAGGATGAACAGTATACCCCCCATTATCATCAAATATTGCCCACGGAGGAAGATCCGTAGATTTAGCAATTTTTTCT
CTGCATCTAGCTGGAATTTCATCAATTTTAGGAGCCATTAATTTCATTACCACAATTATTAATATACGAATTAATGGCCT
ATCTTTCGATCAAATACCTTTATTCGTATGATCTGTTGGTATTACTGCATTACTATTACTTCTTTCTTTACCAGTATTAG
CTGGAGCTATTACTATATTATTAACTGATCGAAATTTAAATACTTCTTTTTTTGACCCAGCGGGAGGGGGAGACCCTATT
CTCTATCAACATCTTTTTTGATTTTTT
>GBGL12830-13|Diatraea_grandiosella|JQ888352|JQ888352
--------------TATTTGGAATTTGAGCAGGAATATTAGGAACATCTCTAAGTTTACTAATTCGTGCTGAACTCGGTA
CACCTAATTCTTTAATTGGTGATGATCAAATTTATAATACAATTGTTACAGCTCATGCATTTATTATAATTTTTTTTATA
GTTATACCTATTATAATTGGGGGATTTGGAAATTGATTAGTACCTCTTATATTAGGGGCTCCGGATATGGCTTTCCCACG
AATAAATAATATAAGATTTTGATTATTACCCCCATCTTTAACTTTATTAATTTCCAGAAGAATCGTTGAAAATGGAGCTG
GAACAGGATGAACAGTATACCCCCCATTATCATCAAATATTGCCCACGGAGGAAGATCCGTAGATTTAGCAATTTTTTCT
CTGCATCTAGCTGGAATTTCATCAATTTTAGGAGCCATTAATTTCATTACCACAATTATTAATATACGAATTAATGGCCT
ATCTTTCGATCAAATACCTTTATTCGTATGATCTGTTGGTATTACTGCATTACTATTACTTCTTTCTTTACCAGTATTAG
CTGGAGCTATTACTATATTATTAACTGATCGAAATTTAAATACTTCTTTTTTTGACCCAGCGGGAGGGGGAGACCCTATT
CTCTATCAACATCTTTTTTGATTTTTT
>GBGL12831-13|Diatraea_grandiosella|JQ888351|JQ888351
--------------TATTTGGAATTTGAGCAGGAATATTAGGAACATCTCTAAGTTTACTAATTCGTGCTGAACTCGGTA
CACCTAATTCTTTAATTGGTGATGATCAAATTTATAATACAATTGTTACAGCTCATGCATTTATTATAATTTTTTTTATA
GTTATACCTATTATAATTGGGGGATTTGGAAATTGATTAGTACCTCTTATATTAGGGGCTCCGGATATGGCTTTCCCACG
AATAAATAATATAAGATTTTGATTATTACCCCCATCTTTAACTTTATTAATTTCCAGAAGAATCGTTGAAAATGGAGCTG
GAACAGGATGAACAGTATACCCCCCATTATCATCAAATATTGCCCACGGAGGAAGATCCGTAGATTTAGCAATTTTTTCT
CTGCATCTAGCTGGAATTTCATCAATTTTAGGAGCCATTAATTTCATTACCACAATTATTAATATACGAATTAATGGCCT
ATCTTTCGATCAAATACCTTTATTCGTATGATCTGTTGGTATTACTGCATTACTATTACTTCTTTCTTTACCAGTATTAG
CTGGAGCTATTACTATATTATTAACTGATCGAAATTTAAATACTTCTTTTTTTGACCCAGCGGGAGGGGGAGACCCTATT
CTCTATCAACATCTTTTTTGATTTTTT
>GBGL12832-13|Diatraea_grandiosella|JQ888350|JQ888350
--------------TATTTGGAATTTGAGCAGGAATATTAGGAACATCTCTAAGTTTACTAATTCGTGCTGAACTCGGTA
CACCTAATTCTTTAATTGGTGATGATCAAATTTATAATACAATTGTTACAGCTCATGCATTTATTATAATTTTTTTTATA
GTTATACCTATTATAATTGGGGGATTTGGAAATTGATTAGTACCTCTTATATTAGGGGCTCCGGATATGGCTTTCCCACG
AATAAATAATATAAGATTTTGATTATTACCCCCATCTTTAACTTTATTAATTTCCAGAAGAATCGTTGAAAATGGAGCTG
GAACAGGATGAACAGTATACCCCCCATTATCATCAAATATTGCCCACGGAGGAAGATCCGTAGATTTAGCAATTTTTTCT
CTGCATCTAGCTGGAATTTCATCAATTTTAGGAGCCATTAATTTCATTACCACAATTATTAATATACGAATTAATGGCCT

ATCTTTCGATCAAATACCTTTATTCGTATGATCTGTTGGTATTACTGCATTACTATTACTTCTTTCTTTACCAGTATTAG
CTGGAGCTATTACTATATTATTAACTGATCGAAATTTAAATACTTCTTTTTTTGACCCAGCGGGAGGGGGAGACCCTATT
CTCTATCAACATCTTTTTTGATTTTTT
>GBGL12833-13|Diatraea_grandiosella|JQ888349|JQ888349
--------------TATTTGGAATTTGAGCAGGAATATTAGGAACATCTCTAAGTTTACTAATTCGTGCTGAACTCGGTA
CACCTAATTCTTTAATTGGTGATGATCAAATTTATAATACAATTGTTACAGCTCATGCATTTATTATAATTTTTTTTATA
GTTATACCTATTATAATTGGGGGATTTGGAAATTGATTAGTACCTCTTATATTAGGGGCTCCGGATATGGCTTTCCCACG
AATAAATAATATAAGATTTTGATTATTACCCCCATCTTTAACTTTATTAATTTCCAGAAGAATCGTTGAAAATGGAGCTG
GAACAGGATGAACAGTATACCCCCCATTATCATCAAATATTGCCCACGGAGGAAGATCCGTAGATTTAGCAATTTTTTCT
CTGCATCTAGCTGGAATTTCATCAATTTTAGGAGCCATTAATTTCATTACCACAATTATTAATATACGAATTAATGGCCT
ATCTTTCGATCAAATACCTTTATTCGTATGATCTGTTGGTATTACTGCATTACTATTACTTCTTTCTTTACCAGTATTAG
CTGGAGCTATTACTATATTATTAACTGATCGAAATTTAAATACTTCTTTTTTTGACCCAGCGGGAGGGGGAGACCCTATT
CTCTATCAACATCTTTTTTGATTTTTT
>GBGL12834-13|Diatraea_grandiosella|JQ888348|JQ888348
--------------TATTTGGAATTTGAGCAGGAATATTAGGAACATCTCTAAGTTTACTAATTCGTGCTGAACTCGGTA
CACCTAATTCTTTAATTGGTGATGATCAAATTTATAATACAATTGTTACAGCTCATGCATTTATTATAATTTTTTTTATA
GTTATACCTATTATAATTGGGGGATTTGGAAATTGATTAGTACCTCTTATATTAGGGGCTCCGGATATGGCTTTCCCACG
AATAAATAATATAAGATTTTGATTATTACCCCCATCTTTAACTTTATTAATTTCCAGAAGAATCGTTGAAAATGGAGCTG
GAACAGGATGAACAGTATACCCCCCATTATCATCAAATATTGCCCACGGAGGAAGATCCGTAGATTTAGCAATTTTTTCT
CTGCATCTAGCTGGAATTTCATCAATTTTAGGAGCCATTAATTTCATTACCACAATTATTAATATACGAATTAATGGCCT
ATCTTTCGATCAAATACCTTTATTCGTATGATCTGTTGGTATTACTGCATTACTATTACTTCTTTCTTTACCAGTATTAG
CTGGAGCTATTACTATATTATTAACTGATCGAAATTTAAATACTTCTTTTTTTGACCCAGCGGGAGGGGGAGACCCTATT
CTCTATCAACATCTTTTTTGATTTTTT
>GBGL12835-13|Diatraea_grandiosella|JQ888347|JQ888347
---GTTTACTAATTCGTGCTGAACTCGGTA
CACCTAATTCTTTAATTGGTGATGATCAAATTTATAATACAATTGTTACAGCTCATGCATTTATTATAATTTTTTTTATA
GTTATACCTATTATAATTGGGGGATTTGGAAATTGATTAGTACCTCTTATATTAGGGGCTCCGGATATGGCTTTCCCACG
AATAAATAATATAAGATTTTGATTATTACCCCCATCTTTAACTTTATTAATTTCCAGAAGAATCGTTGAAAATGGAGCTG
GAACAGGATGAACAGTATACCCCCCATTATCATCAAATATTGCCCACGGAGGAAGATCCGTAGATTTAGCAATTTTTTCT
CTGCATCTAGCTGGAATTTCATCAATTTTAGGAGCCATTAATTTCATTACCACAATTATTAATATACGAATTAATGGCCT
ATCTTTCGATCAAATACCTTTATTCGTATGATCTGTTGGTATTACTGCATTACTATTACTTCTTTCTTTACCAGTATTAG
CTGGAGCTATTACTATATTATTAACTGATCGAAATTTAAATACTTCTTTTTTTGACCCAGCGGGAGGGGGAGACCCTATT
CTCTATCAACATCTTTTTTGATTTTTT
>GBGL12836-13|Diatraea_grandiosella|JQ888346|JQ888346
AACCTTATATTTCATATTTGGAATTTGAGCAGGAATATTAGGAACATCTCTAAGTTTACTAATTCGTGCTGAACTCGGTA
CACCTAATTCTTTAATTGGTGATGATCAAATTTATAATACAATTGTTACAGCTCATGCATTTATTATAATTTTTTTTATA
GTTATACCTATTATAATTGGGGGATTTGGAAATTGATTAGTACCTCTTATATTAGGGGCTCCGGATATGGCTTTCCCACG
AATAAATAATATAAGATTTTGATTATTACCCCCATCTTTAACTTTATTAATTTCCAGAAGAATCGTTGAAAATGGAGCTG
GAACAGGATGAACAGTATACCCCCCATTATCATCAAATATTGCCCACGGAGGAAGATCCGTAGATTTAGCAATTTTTTCT
CTGCATCTAGCTGGAATTTCATCAATTTTAGGAGCCATTAATTTCATTACCACAATTATTAATATACGAATTAATGGCCT
ATCTTTCGATCAAATACCTTTATTCGTATGATCTGTTGGTATTACTGCATTACTATTACTTCTTTCTTTACCAGTATTAG
CTGGAGCTATTACTATATTATTAACTGATCGAAATTTAAATACTTCTTTTTTTGACCCAGCGGGAGGGGGAGACCCTATT
CTCTATCAACATCTTTTTTGATTTTTT
>GBGL12837-13|Diatraea_grandiosella|JQ888345|JQ888345
AACCTTATATTTCATATTTGGAATTTGAGCAGGAATATTAGGAACATCTCTAAGTTTACTAATTCGTGCTGAACTCGGTA
CACCTAATTCTTTAATTGGTGATGATCAAATTTATAATACAATTGTTACAGCTCATGCATTTATTATAATTTTTTTTATA
GTTATACCTATTATAATTGGGGGATTTGGAAATTGATTAGTACCTCTTATATTAGGGGCTCCGGATATGGCTTTCCCACG
AATAAATAATATAAGATTTTGATTATTACCCCCATCTTTAACTTTATTAATTTCCAGAAGAATCGTTGAAAATGGAGCTG
GAACAGGATGAACAGTATACCCCCCATTATCATCAAATATTGCCCACGGAGGAAGATCCGTAGATTTAGCAATTTTTTCT
CTGCATCTAGCTGGAATTTCATCAATTTTAGGAGCCATTAATTTCATTACCACAATTATTAATATACGAATTAATGGCCT
ATCTTTCGATCAAATACCTTTATTCGTATGATCTGTTGGTATTACTGCATTACTATTACTTCTTTCTTTACCAGTATTAG
CTGGAGCTATTACTATATTATTAACTGATCGAAATTTAAATACTTCTTTTTTTGACCCAGCGGGAGGGGGAGACCCTATT
CTCTATCAACATCTTTTTTGATTTTTT
>GBGL12897-14|Scirpophaga_incertulas|KF268959|KF268959

---ATTATAATTTTTTTTATA
GTTATACCCATTATAATTGGAGGATTTGGAAATTGATTAGTCCCCCTAAKATTAGGAGCCCCAGATATAGCTTTTCCCCG

ATTAAATAACATAAGATTCTGATTATTACCCCCCTCTTTAACACTCCTCATTTCTAGAAGAATTGTAGAAAATGGAGCTG
GAACAGGATGAACTGTTTACCCACCCCTATCATCCAATATTGCTCATGGAGGAACATCAGTAGATTTAGCTATTTTTTCA
CTACACCTAGCAGGAATCTCATCTATCTTAGGAGCTATTAATTTTATTACAACCATTATTAATATACGAATTAATGGGTT
ATCATTTGACCAAATACCCCTATTTGTGTGAGCTGTTGGTATTACAGCCCTTCTTCTACTCCTCTCTCTCCCAGTTTTAG
CTGGAGCTATTACCATATTACTAACAGATYKAAATTTAAATACATCTTTTTTTGACCCAGCTGGAGGAGGAGACCCAATT
TTATATCAA------------------
>GBGL14920-14|Diatraea_lisetta|KJ657593|KJ657593
-------------------------GAGCTGGAATATTAGGAACATCATTAAGTTTATTAATTCGTGCTGAATTAGGTA
CACCTAATTCTCTAATTGGAGATGATCAAATTTATAATACAATTGTTACAGCTCACGCATTTATTATAATTTTTTTTATA
GTTATACCTATTATAATTGGAGGATTTGGAAATTGATTAGTTCCTTTAATATTAGGAGCTCCTGATATAGCTTTCCCTCG
AATAAATAACATAAGATTTTGATTATTACCCCCATCTTTAACATTATTAATTTCTAGAAGAATTGTTGAAAATGGAGCTG
GAACAGGATGAACAGTATACCCCCCCCTTTCATCTAATATTGCTCATGGTGGAAGATCTGTAGATTTAGCAATTTTCTCC
CTTCATTTAGCTGGAATTTCATCAATTTTAGGAGCTATTAATTTTATTACTACAATTATTAATATACGAATTAATAGATT
ATCATTTGATCAAATACCACTTTTTATTTGATCAGTAGGTATTACAGCTTTATTATTATTACTTTCTTTACCTGTATTAG
CTGGAGCTATTACAATATTATTAACTGATCGAAATTTAAATACTTCTTTTTTTGATCCAGCTGGAGGAGGAGATCCTATT
TTATATCAACATCTTTTTTGATTTTT-
>GBGL14921-14|Diatraea_lisetta|KJ657595|KJ657595
-------------------------GAGCTGGAATATTAGGAACATCATTAAGTTTATTAATTCGTGCTGAATTAGGTA
CACCTAATTCTCTAATTGGAGATGATCAAATTTATAATACAATTGTTACAGCTCACGCATTTATTATAATTTTTTTTATA
GTTATACCTATTATAATTGGAGGATTTGGAAATTGATTAGTTCCTTTAATATTAGGAGCTCCTGATATAGCTTTCCCTCG
AATAAATAACATAAGATTTTGATTATTGCCCCCATCTTTAACATTATTAATTTCTAGAAGAATTGTTGAAAATGGAGCTG
GAACAGGATGAACAGTATACCCCCCCCTTTCATCTAATATTGCTCATGGTGGAAGATCTGTAGATTTAGCAATTTTCTCC
CTTCATTTAGCTGGAATTTCATCAATTTTAGGAGCTATTAATTTTATTACTACAATTATTAATATACGAATTAATAGATT
ATCATTTGATCAAATACCACTTTTTATTTGATCAGTAGGTATTACAGCTTTATTATTATTACTTTCTTTACCTGTATTAG
CTGGAGCTATTACAATATTATTAACTGATCGAAATTTAAATACTTCTTTTTTTGATCCAGCTGGAGGAGGAGATCCTATT
TTATATCAACATCTTTTTTGATTTTT-
>GBGL14922-14|Diatraea_saccharalis|JX442547|JX442547
------------------------TGAGCAGGAATATTAGGAACTTCATTAAGTTTATTAATTCGAGCAGAATTAGGAA
CATCTAACTCTTTAATTGGAGATGATCAAATTTATAACACAATTGTAACAGCTCATGCTTTCATTATAATTTTTTTTATA
GTTATACCTATTATAATTGGAGGATTTGGAAATTGATTGGTACCTTTAATATTAGGAGCCCCCGATATAGCTTTCCCACG
AATAAATAATATAAGATTTTGATTATTACCCCCATCTTTAACCCTACTAATTTCTAGAAGAATTGTAGAAAATGGAGCAG
GAACAGGATGAACAGTTTATCCCCCATTATCATCTAATATTGCCCATGGGGGAAGATCTGTGGACTTAGCAATTTTCTCT
TTACATTTAGCTGGGATTTCCTCAATTTTAGGGGCTATTAATTTCATCACTACCATTATTAATATACGAATTAATAATCT
ATCATTTGATCAAATACCTTTATTTATTTGATCAGTGGGCATTACAGCATTACTTTTATTACTTTCTTTACCAGTATTAG
CTGGAGCCATTACTATATTACTAACAGATCGAAATTTAAATACCTCCTTCTTTGATCCAGCGGGAGGGGGAGATCCTATT
CTATATCAACATTTATTTTGATTTTTT
>GBGL14923-14|Diatraea_saccharalis|JX442548|JX442548
------------------------TGAGCAGGAATATTAGGAACTTCATTAAGTTTATTAATTCGAGCAGAATTAGGAA
CATCTAACTCTTTAATTGGAGATGATCAAATTTATAACACAATTGTAACAGCTCATGCTTTCATTATAATTTTTTTTATA
GTTATACCTATTATAATTGGAGGATTTGGAAATTGATTGGTACCTTTAATATTAGGAGCCCCCGATATAGCTTTCCCACG
AATAAATAATATAAGATTTTGATTATTACCCCCATCTTTAACCCTACTAATTTCTAGAAGAATTGTAGAAAATGGAGCAG
GAACAGGATGAACAGTTTATCCCCCATTATCATCTAATATTGCCCATGGGGGAAGATCTGTGGACTTAGCAATTTTCTCT
TTACATTTAGCTGGGATTTCCTCAATTTTAGGGGCTATTAATTTCATCACTACCATTATTAATATACGAATTAATAATCT
ATCATTTGATCAAATACCTTTATTTATTTGATCAGTGGGCATTACAGCATTACTTTTATTACTTTCTTTACCAGTATTAG
CTGGAGCCATTACTATATTACTAACAGATCGAAATTTAAATACCTCCTTCTTTGATCCAGCGGGAGGGGGAGATCCTATT
CTATATCAACATTTATTTTGATTTTTT
>GBGL14924-14|Diatraea_saccharalis|JX442549|JX442549
------------------------TGAGCAGGAATATTAGGAACTTCATTAAGTTTATTAATTCGAGCAGAATTAGGAA
CATCTAACTCTTTAATTGGAGATGATCAAATTTATAACACAATTGTAACAGCTCATGCTTTCATTATAATTTTTTTTATA
GTTATACCTATTATAATTGGAGGATTTGGAAATTGATTGGTACCTTTAATATTAGGAGCCCCCGATATAGCTTTCCCACG
AATAAATAATATAAGATTTTGATTATTACCCCCATCTTTAACCCTACTAATTTCTAGAAGAATTGTAGAAAATGGAGCAG
GAACAGGATGAACAGTTTATCCCCCATTATCATCTAATATTGCCCATGGGGGAAGATCTGTGGACTTAGCAATTTTCTCT
TTACATTTAGCTGGGATTTCCTCAATTTTAGGGGCTATTAATTTCATCACTACCATTATTAATATACGAATTAATAATCT
ATCATTTGATCAAATACCTTTATTTATTTGATCAGTGGGCATTACAGCATTACTTTTATTACTTTCTTTACCAGTATTAG
CTGGAGCCATTACTATATTACTAACAGATCGAAATTTAAATACCTCCTTCTTTGATCCAGCGGGAGGGGGAGATCCTATT
CTATATCAACATTTATTTTGATTTTTT
>GBGL14925-14|Diatraea_saccharalis|JX442550|JX442550

-------------------------TGAGCAGGAATATTAGGAACTTCATTAAGTTTATTAATTCGAGCAGAATTAGGAA
CATCTAACTCTTTAATTGGAGATGATCAAATTTATAACACAATTGTAACAGCTCATGCTTTCATTATAATTTTTTTATA
GTTATACCTATTATAATTGGAGGATTTGGAAATTGATTGGTACCTTTAATATTAGGAGCCCCCGATATAGCTTTCCCACG
AATAAATAATATAAGATTTTGATTATTACCCCCATCTTTAACCCTACTAATTTCTAGAAGAATTGTAGAAAATGGAGCAG
GAACAGGATGAACAGTTTATCCCCCATTATCATCTAATATTGCCCATGGGGGAAGATCTGTGGACTTAGCAATTTTCTCT
TTACATTTAGCTGGGATTTCCTCAATTTTAGGGGCTATTAATTTCATCACTACCATTATTAATATACGAATTAATAATCT
ATCATTTGATCAAATACCTTTATTTATTTGATCAGTGGGCATTACAGCATTACTTTTATTACTTTCTTTACCAGTATTAG
CTGGAGCCATTACTATATTACTAACAGATCGAAATTTAAATACCTCCTTCTTTGATCCAGCGGGAGGGGGAGATCCTATT
CTATATCAACATTTATTTTGATTTTTT
>GBGL14926-14|Diatraea_saccharalis|JX442551|JX442551
-------------------------TGAGCAGGAATATTAGGAACTTCATTAAGTTTATTAATTCGAGCAGAATTAGGAA
CATCTAACTCTTTAATTGGAGATGATCAAATTTATAACACAATTGTAACAGCTCATGCTTTCATTATAATTTTTTTATA
GTTATACCTATTATAATTGGAGGATTTGGAAATTGATTGGTACCTTTAATATTAGGAGCCCCCGATATAGCTTTCCCACG
AATAAATAATATAAGATTTTGATTATTACCCCCATCTTTAACCCTACTAATTTCTAGAAGAATTGTAGAAAATGGAGCAG
GAACAGGATGAACAGTTTATCCCCCATTATCATCTAATATTGCCCATGGGGGAAGATCTGTGGACTTAGCAATTTTCTCT
TTACATTTAGCTGGGATTTCCTCAATTTTAGGGGCTATTAATTTCATCACTACCATTATTAATATACGAATTAATAATCT
ATCATTTGATCAAATACCTTTATTTATTTGATCAGTGGGCATTACAGCATTACTTTTATTACTTTCTTTACCAGTATTAG
CTGGAGCCATTACTATATTACTAACAGATCGAAATTTAAATACCTCCTTCTTTGATCCAGCGGGAGGGGGAGATCCTATT
CTATATCAACATTTATTTTGATTTTTT
>GBGL14927-14|Diatraea_saccharalis|JX442552|JX442552
-------------------------TGAGCAGGAATATTAGGAACTTCATTAAGTTTATTAATTCGAGCAGAATTAGGAA
CATCTAACTCTTTAATTGGAGATGATCAAATTTATAACACAATTGTAACAGCTCATGCTTTCATTATAATTTTTTTATA
GTTATACCTATTATAATTGGAGGATTTGGAAATTGATTGGTACCTTTAATATTAGGAGCCCCCGATATAGCTTTCCCACG
AATAAATAATATAAGATTTTGATTATTACCCCCATCTTTAACCCTACTAATTTCTAGAAGAATTGTAGAAAATGGAGCAG
GAACAGGATGAACAGTTTATCCCCCATTATCATCTAATATTGCCCATGGAGGAAGATCTGTGGACTTAGCAATTTTCTCT
TTACATTTAGCTGGGATTTCCTCAATTTTAGGGGCTATWAATTTCATCACTACCATTATTAATATACGAATTAATAATCT
ATCATTTGATCAAATACCTTTATTTATTTGATCAGTGGGWATTACAGCATTACTTTTATTACTTTCTTTACCAGTATTAG
CTGGAGCCATTACTATATTACTAACAGATCGAAATTTAAATACCTCCTTCTTTGATCCAGNGGGAGGGGGAGATCCTATT
CTATATCAACATTTATTTTGATTTTTT
>GBGL14928-14|Diatraea_saccharalis|JX442553|JX442553
-------------------------TGAGCAGGAATATTAGGAACTTCATTAAGTTTATTAATTCGAGCAGAATTAGGAA
CATCTAACTCTTTAATTGGAGATGATCAAATTTATAACACAATTGTAACAGCTCATGCTTTCATTATAATTTTTTTATA
GTTATACCTATTATAATTGGAGGATTTGGAAATTGATTGGTACCTTTAATATTAGGAGCCCCCGATATAGCTTTCCCACG
AATAAATAATATAAGATTTTGATTATTACCCCCATCTTTAACCCTACTAATTTCTAGAAGAATTGTAGAAAATGGAGCAG
GAACAGGATGAACAGTTTATCCCCCATTATCATCTAATATTGCCCATGGAGGAAGATCTGTGGACTTAGCAATTTTCTCT
TTACATTTAGCTGGGATTTCCTCAATTTTAGGGGCTATCAATTTCATCACTACCATTATTAATATACGAATTAATAATCT
ATCATTTGATCAAATACCTTTATTTATTTGATCAGTGGGTATTACAGCATTACTTTTATTACTTTCTTTACCAGTATTAG
CTGGAGCCATTACTATATTACTAACAGATCGAAATTTAAATACCTCCTTCTTTGATCCAGCGGGAGGGGGAGATCCTATT
CTATATCAACATTTATTTTGATTTTTT
>GBGL14929-14|Diatraea_saccharalis|JX442554|JX442554
-------------------------TGAGCAGGAATATTAGGAACTTCATTAAGTTTATTAATTCGAGCAGAATTAGGAA
CATCTAACTCTTTAATTGGAGATGATCAAATTTATAACACAATTGTAACAGCTCATGCTTTCATTATAATTTTTTTATA
GTTATACCTATTATAATTGGAGGATTTGGAAATTGATTGGTACCTTTAATATTAGGAGCCCCCGATATAGCTTTCCCACG
AATAAATAATATAAGATTTTGATTATTACCCCCATCTTTAACCCTACTAATTTCTAGAAGAATTGTAGAAAATGGAGCAG
GAACAGGATGAACAGTTTATCCCCCATTATCATCTAATATTGCCCATGGGGGAAGATCTGTGGACTTAGCAATTTTCTCT
TTACATTTAGCTGGGATTTCCTCAATTTTAGGGGCTATTAATTTCATCACTACCATTATTAATATACGAATTAATAATCT
ATCATTTGATCAAATACCTTTATTTATTTGATCAGTGGGCATTACAGCATTACTTTTATTACTTTCTTTACCAGTATTAG
CTGGAGCCATTACTATATTACTAACAGATCGAAATTTAAATACCTCCTTCTTTGATCCAGCGGGAGGGGGAGATCCTATT
CTATATCAACATTTATTTTGATTTTTT
>GBGL14930-14|Diatraea_saccharalis|JX442555|JX442555
-------------------------TGAGCAGGAATATTAGGAACTTCATTAAGTTTATTAATTCGAGCAGAATTAGGAA
CATCTAACTCTTTAATTGGAGATGATCAAATTTATAACACAATTGTAACAGCTCATGCTTTCATTATAATTTTTTTATA
GTTATACCTATTATAATTGGAGGATTTGGAAATTGATTGGTACCTTTAATATTAGGAGCCCCCGATATAGCTTTCCCACG
AATAAATAATATAAGATTTTGATTATTACCCCCATCTTTAACCCTACTAATTTCTAGAAGAATTGTAGAAAATGGAGCAG
GAACAGGATGAACAGTTTATCCCCCATTATCATCTAATATTGCCCATGGGGGAAGATCTGTGGACTTAGCAATTTTCTCT
TTACATTTAGCTGGGATTTCCTCAATTTTAGGGGCTATTAATTTCATCACTACCATTATTAATATACGAATTAATAATCT
ATCATTTGATCAAATACCTTTATTTATTTGATCAGTGGGCATTACAGCATTACTTTTATTACTTTCTTTACCAGTATTAG

CTGGAGCCATTACTATATTACTAACAGATCGAAATTTAAATACCTCCTTCTTTGATCCAGCGGGAGGGGGAGATCCTATT
CTATATCAACATTTATTTTGATTTTTT
>GBGL14931-14|Diatraea_saccharalis|JX442556|JX442556
-------------------------TGAGCAGGAATATTAGGAACTTCATTAAGTTTATTAATTCGAGCAGAATTAGGAA
CATCTAACTCTTTAATTGGAGATGATCAAATTTATAACACAATTGTAACAGCTCATGCTTTCATTATAATTTTTTTATA
GTTATACCTATTATAATTGGAGGATTTGGAAATTGATTGGTACCTTTAATATTAGGAGCCCCCGATATAGCTTTCCCACG
AATAAATAATATAAGATTTTGATTATTACCCCCATCTTTAACCCTACTAATTTCTAGAAGAATTGTAGAAAATGGAGCAG
GAACAGGATGAACAGTTTATCCCCCATTATCATCTAATATTGCCCATGGGGGAAGATCTGTGGACTTAGCAATTTTCTCT
TTACATTTAGCTGGGATTTCCTCAATTTTAGGGGCTATTAATTTCATCACTACCATTATTAATATACGAATTAATAATCT
ATCATTTGATCAAATACCTTTATTTATTTGATCAGTGGGCATTACAGCATTACTTTTATTACTTTCTTTACCAGTATTAG
CTGGAGCCATTACTATATTACTAACAGATCGAAATTTAAATACCTCCTTCTTTGATCCAGCGGGAGGGGGAGATCCTATT
CTATATCAACATTTATTTTGATTTTTT
>GBGL14932-14|Diatraea_saccharalis|JX442557|JX442557
-------------------------TGAGCAGGAATATTAGGAACTTCATTAAGTTTATTAATTCGAGCAGAATTAGGAA
CATCTAACTCTTTAATTGGAGATGATCAAATTTATAACACAATTGTAACAGCTCATGCTTTCATTATAATTTTTTTATA
GTTATACCTATTATAATTGGAGGATTTGGAAATTGATTGGTACCTTTAATATTAGGAGCCCCCGATATAGCTTTCCCACG
AATAAATAATATAAGATTTTGATTATTACCCCCATCTTTAACCCTACTAATTTCTAGAAGAATTGTAGAAAATGGAGCAG
GAACAGGATGAACAGTTTATCCCCCATTATCATCTAATATTGCCCATGGAGGAAGATCTGTGGACTTAGCAATTTTCTCT
TTACATTTAGCTGGGATTTCCTCAATTTTAGGGGCTATCAATTTCATCACTACCATTATTAATATACGAATTAATAATCT
ATCATTTGATCAAATACCTTTATTTATTTGATCAGTGGGTATTACAGCATTACTTTTATTACTTTCTTTACCAGTATTAG
CTGGAGCCATTACTATATTACTAACAGATCGAAATTTAAATACCTCCTTCTTTGATCCAGCGGGAGGGGGAGATCCTATT
CTATATCAACATTTATTTTGATTTTTT
>GBGL14933-14|Diatraea_saccharalis|JX442558|JX442558
-------------------------TGAGCAGGAATATTAGGAACTTCATTAAGTTTATTAATTCGAGCAGAATTAGGAA
CATCTAACTCTTTAATTGGAGATGATCAAATTTATAACACAATTGTAACAGCTCATGCTTTCATTATAATTTTTTTATA
GTTATACCTATTATAATTGGAGGATTTGGAAATTGATTGGTACCTTTAATATTAGGAGCCCCCGATATAGCTTTCCCACG
AATAAATAATATAAGATTTTGATTATTACCCCCATCTTTAACCCTACTAATTTCTAGAAGAATTGTAGAAAATGGAGCAG
GAACAGGATGAACAGTTTATCCCCCATTATCATCTAATATTGCCCATGGAGGAAGATCTGTGGACTTAGCAATTTTCTCT
TTACATTTAGCTGGGATTTCCTCAATTTTAGGGGCTATCAATTTCATCACTACCATTATTAATATACGAATTAATAATCT
ATCATTTGATCAAATACCTTTATTTATTTGATCAGTGGGTATTACAGCATTACTTTTATTACTTTCTTTACCAGTATTAG
CTGGAGCCATTACTATATTACTAACAGATCGAAATTTAAATACCTCCTTCTTTGATCCAGCGGGAGGGGGAGATCCTATT
CTATATCAACATTTATTTTGATTTTTT
>GBGL14934-14|Diatraea_saccharalis|JX442559|JX442559
-------------------------TGAGCAGGAATATTAGGAACTTCATTAAGTTTATTAATTCGAGCAGAATTAGGAA
CATCTAACTCTTTAATTGGAGATGATCAAATTTATAACACAATTGTAACAGCTCATGCTTTCATTATAATTTTTTTATA
GTTATACCTATTATAATTGGAGGATTTGGAAATTGATTGGTACCTTTAATATTAGGAGCCCCCGATATAGCTTTCCCACG
AATAAATAATATAAGATTTTGATTATTACCCCCATCTTTAACCCTACTAATTTCTAGAAGAATTGTAGAAAATGGAGCAG
GAACAGGATGAACAGTTTATCCCCCATTATCATCTAATATTGCCCATGGAGGAAGATCTGTGGACTTAGCAATTTTCTCT
TTACATTTAGCTGGGATTTCCTCAATTTTAGGGGCTATCAATTTCATCACTACCATTATTAATATACGAATTAATAATCT
ATCATTTGATCAAATACCTTTATTTATTTGATCAGTGGGTATTACAGCATTACTTTTATTACTTTCTTTACCAGTATTAG
CTGGAGCCATTACTATATTACTAACAGATCGAAATTTAAATACCTCCTTCTTTGATCCAGCGGGAGGGGGAGATCCTATT
CTATATCAACATTTATTTTGATTTTTT
>GBGL14935-14|Diatraea_saccharalis|JX442560|JX442560
-------------------------TGAGCAGGAATATTAGGAACTTCATTAAGTTTATTAATTCGAGCAGAATTAGGAA
CATCTAACTCTTTAATTGGAGATGATCAAATTTATAACACAATTGTAACAGCTCATGCTTTCATTATAATTTTTTTATA
GTTATACCTATTATAATTGGAGGATTTGGAAATTGATTGGTACCTTTAATATTAGGAGCCCCCGATATAGCTTTCCCACG
AATAAATAATATAAGATTTTGATTATTACCCCCATCTTTAACCCTACTAATTTCTAGAAGAATTGTAGAAAATGGAGCAG
GAACAGGATGAACAGTTTATCCCCCATTATCATCTAATATTGCCCATGGAGGAAGATCTGTGGACTTAGCAATTTTCTCT
TTACATTTAGCTGGGATTTCCTCAATTTTAGGGGCTATCAATTTCATCACTACCATTATTAATATACGAATTAATAATCT
ATCATTTGATCAAATACCTTTATTTATTTGATCAGTGGGTATTACAGCATTACTTTTATTACTTTCTTTACCAGTATTAG
CTGGAGCCATTACTATATTACTAACAGATCGAAATTTAAATACCTCCTTCTTTGATCCAGCGGGAGGGGGAGATCCTATT
CTATATCAACATTTATTTTGATTTTTT
>GBGL14936-14|Diatraea_saccharalis|JX442561|JX442561
-------------------------TGAGCAGGAATATTAGGAACTTCATTAAGTTTATTAATTCGAGCAGAATTAGGAA
CATCTAACTCTTTAATTGGAGATGATCAAATTTATAACACAATTGTAACAGCTCATGCTTTCATTATAATTTTTTTATA
GTTATACCTATTATAATTGGAGGATTTGGAAATTGATTGGTACCTTTAATATTAGGAGCCCCCGATATAGCTTTCCCACG
AATAAATAATATAAGATTTTGATTATTACCCCCATCTTTAACCCTACTAATTTCTAGAAGAATTGTAGAAAATGGAGCAG

GAACAGGATGAACAGTTTATCCCCCATTATCATCTAATATTGCCCATGGGGGAAGATCTGTGGACTTAGCAATTTTCTCT
TTACATTTAGCTGGAATTTCCTCAATTTTAGGGGCTATTAATTTCATCACTACCATTATTAATATACGAATTAATAATCT
ATCATTTGATCAAATACCTTTATTTATTTGATCAGTGGGCATTACAGCATTACTTTTATTACTTTCTTTACCAGTATTAG
CTGGAGCCATTACTATATTACTAACAGATCGAAATTTAAATACCTCCTTCTTTGATCCAGCGGGAGGGGGAGATCCTATT
CTATATCAACATTTATTTTGATTTTTT
>GBGL14937-14|Diatraea_saccharalis|JX442562|JX442562
------------------------TGAGCAGGAATATTAGGAACTTCATTAAGTTTATTAATTCGAGCAGAATTAGGAA
CATCTAACTCTTTAATTGGAGATGATCAAATTTATAACACAATTGTAACAGCTCATGCTTTCATTATAATTTTTTTTATA
GTTATACCTATTATAATTGGAGGATTTGGAAATTGATTGGTACCTTTAATATTAGGAGCCCCCGATATAGCTTTCCCACG
AATAAATAATATAAGATTTTGATTATTACCCCCATCTTTAACCCTACTAATTTCTAGAAGAATTGTAGAAAATGGAGCAG
GAACAGGATGAACAGTTTATCCCCCATTATCATCTAATATTGCCCATGGGGGAAGATCTGTGGACTTAGCAATTTTCTCT
TTACATTTAGCTGGGATTTCCTCAATTTTAGGGGCTATTAATTTCATCACTACCATTATTAATATACGAATTAATAATCT
ATCATTTGATCAAATACCTTTATTTATTTGATCAGTGGGCATTACAGCATTACTTTTATTACTTTCTTTACCAGTATTAG
CTGGAGCCATTACTATATTACTAACAGATCGAAATTTAAATACCTCCTTCTTTGATCCAGCGGGAGGGGGAGATCCTATT
CTATATCAACATTTATTTTGATTTTTT
>GBGL14938-14|Diatraea_saccharalis|JX442563|JX442563
------------------------TGAGCAGGAATATTAGGAACTTCATTAAGTTTATTAATTCGAGCAGAATTAGGAA
CATCTAACTCTTTAATTGGAGATGATCAAATTTATAACACAATTGTAACAGCTCATGCTTTCATTATAATTTTTTTTATA
GTTATACCTATTATAATTGGAGGATTTGGAAATTGATTGGTACCTTTAATATTAGGAGCCCCCGATATAGCTTTCCCACG
AATAAATAATATAAGATTTTGATTATTACCCCCATCTTTAACCCTACTAATTTCTAGAAGAATTGTAGAAAATGGAGCAG
GAACAGGATGAACAGTTTATCCCCCATTATCATCTAATATTGCCCATGGGGGAAGATCTGTGGACTTAGCAATTTTCTCT
TTACATTTAGCTGGGATTTCCTCAATTTTAGGGGCTATTAATTTCATCACTACCATTATTAATATACGAATTAATAATCT
ATCATTTGATCAAATACCTTTATTTATTTGATCAGTGGGCATTACAGCATTACTTTTATTACTTTCTTTACCAGTATTAG
CTGGAGCCATTACTATATTACTAACAGATCGAAATTTAAATACCTCCTTCTTTGATCCAGCGGGAGGGGGAGATCCTATT
CTATATCAACATTTATTTTGATTTTTT
>GBGL14939-14|Diatraea_saccharalis|JX442564|JX442564
------------------------TGAGCAGGAATATTAGGAACTTCATTAAGTTTATTAATTCGAGCAGAATTAGGAA
CATCTAACTCTTTAATTGGAGATGATCAAATTTATAACACAATTGTAACAGCTCATGCTTTCATTATAATTTTTTTTATA
GTTATACCTATTATAATTGGAGGATTTGGAAATTGATTGGTACCTTTAATATTAGGAGCCCCCGATATAGCTTTCCCACG
AATAAATAATATAAGATTTTGATTATTACCCCCATCTTTAACCCTACTAATTTCTAGAAGAATTGTAGAAAATGGAGCAG
GAACAGGATGAACAGTTTATCCCCCATTATCATCTAATATTGCCCATGGGGGAAGATCTGTGGACTTAGCAATTTTCTCT
TTACATTTAGCTGGGATTTCCTCAATTTTAGGGGCTATTAATTTCATCACTACCATTATTAATATACGAATTAATAATCT
ATCATTTGATCAAATACCTTTATTTATTTGATCAGTGGGCATTACAGCATTACTTTTATTACTTTCTTTACCAGTATTAG
CTGGAGCCATTACTATATTACTAACAGATCGAAATTTAAATACCTCCTTCTTTGATCCAGCGGGAGGGGGAGATCCTATT
CTATATCAACATTTATTTTGATTTTTT
>GBGL14940-14|Diatraea_saccharalis|JX442565|JX442565
------------------------TGAGCAGGAATATTAGGAACTTCATTAAGTTTATTAATTCGAGCAGAATTAGGAA
CATCTAACTCTTTAATTGGAGATGATCAAATTTATAACACAATTGTAACAGCTCATGCTTTCATTATAATTTTTTTTATA
GTTATACCTATTATAATTGGAGGATTTGGAAATTGATTGGTACCTTTAATATTAGGAGCCCCCGATATAGCTTTCCCACG
AATAAATAATATAAGATTTTGATTATTACCCCCATCTTTAACCCTACTAATTTCTAGAAGAATTGTAGAAAATGGAGCAG
GAACAGGATGAACAGTTTATCCCCCATTATCATCTAATATTGCCCATGGGGGAAGATCTGTGGACTTAGCAATTTTCTCT
TTACATTTAGCTGGGATTTCCTCAATTTTAGGGGCTATTAATTTCATCACTACCATTATTAATATACGAATTAATAATCT
ATCATTTGATCAAATACCTTTATTTATTTGATCAGTGGGCATTACAGCATTACTTTTATTACTTTCTTTACCAGTATTAG
CTGGAGCCATTACTATATTACTAACAGATCGAAATTTAAATACCTCCTTCTTTGATCCAGCGGGAGGGGGAGATCCTATT
CTATATCAACATTTATTTTGATTTTTT
>GBGL14941-14|Diatraea_saccharalis|JX442566|JX442566
------------------------TGAGCAGGAATATTAGGAACTTCATTAAGTTTATTAATTCGAGCAGAATTAGGAA
CATCTAACTCTTTAATTGGAGATGATCAAATTTATAACACAATTGTAACAGCTCATGCTTTCATTATAATTTTTTTTATA
GTTATACCTATTATAATTGGAGGATTTGGAAATTGATTGGTACCTTTAATATTAGGAGCCCCCGATATAGCTTTCCCACG
AATAAATAATATAAGATTTTGATTATTACCCCCATCTTTAACCCTACTAATTTCTAGAAGAATTGTAGAAAATGGAGCAG
GAACAGGATGAACAGTTTATCCCCCATTATCATCTAATATTGCCCATGGGGGAAGATCTGTGGACTTAGCAATTTTCTCT
TTACATTTAGCTGGGATTTCCTCAATTTTAGGGGCTATTAATTTCATCACTACCATTATTAATATACGAATTAATAATCT
ATCATTTGATCAAATACCTTTATTTATTTGATCAGTGGGCATTACAGCATTACTTTTATTACTTTCTTTACCAGTATTAG
CTGGAGCCATTACTATATTACTAACAGATCGAAATTTAAATACCTCCTTCTTTGATCCAGCGGGAGGGGGAGATCCTATT
CTATATCAACATTTATTTTGATTTTTT
>GBGL14942-14|Diatraea_saccharalis|JX442567|JX442567
------------------------TGAGCAGGAATATTAGGAACTTCATTAAGTTTATTAATTCGAGCAGAATTAGGAA

CATCTAACTCTTTAATTGGAGATGATCAAATTTATAACACAATTGTAACAGCTCATGCTTTCATTATAATTTTTTTATA
GTTATACCTATTATAATTGGAGGATTTGGAAATTGATTGGTACCTTTAATATTAGGAGCCCCCGATATAGCTTTCCCACG
AATAAATAATATAAGATTTTGATTATTACCCCCATCTTTAACCCTACTAATTTCTAGAAGAATTGTAGAAAATGGAGCAG
GAACAGGATGAACAGTTTATCCCCCATTATCATCTAATATTGCCCATGGGGGAAGATCTGTGGACTTAGCAATTTTCTCT
TTACATTTAGCTGGGATTTCCTCAATTTTAGGGGCTATTAATTTCATCACTACCATTATTAATATACGAATTAATAATCT
ATCATTTGATCAAATACCTTTATTTATTTGATCAGTGGGCATTACAGCATTACTTTTATTACTTTCTTTACCAGTATTAG
CTGGAGCCATTACTATATTACTAACAGATCGAAATTTAAATACCTCCTTCTTTGATCCAGCGGGAGGGGGAGATCCTATT
CTATATCAACATTTATTTTGATTTTTT
>GBGL14943-14|Diatraea_saccharalis|JX442568|JX442568
------------------------TGAGCAGGAATATTAGGAACTTCATTAAGTTTATTAATTCGAGCAGAATTAGGAA
CATCTAACTCTTTAATTGGAGATGATCAAATTTATAACACAATTGTAACAGCTCATGCTTTCATTATAATTTTTTTATA
GTTATACCTATTATAATTGGAGGATTTGGAAATTGATTGGTACCTTTAATATTAGGAGCCCCCGATATAGCTTTCCCACG
AATAAATAATATAAGATTTTGATTATTACCCCCATCTTTAACCCTACTAATTTCTAGAAGAATTGTAGAAAATGGAGCAG
GAACAGGATGAACAGTTTATCCCCCATTATCATCTAATATTGCCCATGGGGGAAGATCTGTGGACTTAGCAATTTTCTCT
TTACATTTAGCTGGGATTTCCTCAATTTTAGGGGCTATTAATTTCATCACTACCATTATTAATATACGAATTAATAATCT
ATCATTTGATCAAATACCTTTATTTATTTGATCAGTGGGCATTACAGCATTACTTTTATTACTTTCTTTACCAGTATTAG
CTGGAGCCATTACTATATTACTAACAGATCGAAATTTAAATACCTCCTTCTTTGATCCAGCGGGAGGGGGAGATCCTATT
CTATATCAACATTTATTTTGATTTTTT
>GBGL14944-14|Diatraea_saccharalis|JX442569|JX442569
------------------------TGAGCAGGAATATTAGGAACTTCATTAAGTTTATTAATTCGAGCAGAATTAGGAA
CATCTAACTCTTTAATTGGAGATGATCAAATTTATAACACAATTGTAACAGCTCATGCTTTCATTATAATTTTTTTATA
GTTATACCTATTATAATTGGAGGATTTGGAAATTGATTGGTACCTTTAATATTAGGAGCCCCCGATATAGCTTTCCCACG
AATAAATAATATAAGATTTTGATTATTACCCCCATCTTTAACCCTACTAATTTCTAGAAGAATTGTAGAAAATGGAGCAG
GAACAGGATGAACAGTTTATCCCCCATTATCATCTAATATTGCCCATGGGGGAAGATCTGTGGACTTAGCAATTTTCTCT
TTACATTTAGCTGGGATTTCCTCAATTTTAGGGGCTATTAATTTCATCACTACCATTATTAATATACGAATTAATAATCT
ATCATTTGATCAAATACCTTTATTTATTTGATCAGTGGGCATTACAGCATTACTTTTATTACTTTCTTTACCAGTATTAG
CTGGAGCCATTACTATATTACTAACAGATCGAAATTTAAATACCTCCTTCTTTGATCCAGCGGGAGGGGGAGATCCTATT
CTATATCAACATTTATTTTGATTTTTT
>GBGL14945-14|Diatraea_saccharalis|JX442570|JX442570
------------------------TGAGCAGGAATATTAGGAACTTCATTAAGTTTATTAATTCGAGCAGAATTAGGAA
CATCTAACTCTTTAATTGGAGATGATCAAATTTATAACACAATTGTAACAGCTCATGCTTTCATTATAATTTTTTTATA
GTTATACCTATTATAATTGGAGGATTTGGAAATTGATTGGTACCTTTAATATTAGGAGCCCCCGATATAGCTTTCCCACG
AATAAATAATATAAGATTTTGATTATTACCCCCATCTTTAACCCTACTAATTTCTAGAAGAATTGTAGAAAATGGAGCAG
GAACAGGATGAACAGTTTATCCCCCATTATCATCTAATATTGCCCATGGGGGAAGATCTGTGGACTTAGCAATTTTCTCT
TTACATTTAGCTGGGATTTCCTCAATTTTAGGGGCTATTAATTTCATCACTACCATTATTAATATACGAATTAATAATCT
ATCATTTGATCAAATACCTTTATTTATTTGATCAGTGGGCATTACAGCATTACTTTTATTACTTTCTTTACCAGTATTAG
CTGGAGCCATTACTATATTACTAACAGATCGAAATTTAAATACCTCCTTCTTTGATCCAGCGGGAGGGGGAGATCCTATT
CTATATCAACATTTATTTTGATTTTTT
>GBGL14946-14|Diatraea_saccharalis|JX442571|JX442571
------------------------TGAGCAGGAATATTAGGAACTTCATTAAGTTTATTAATTCGAGCAGAATTAGGAA
CATCTAACTCTTTAATTGGAGATGATCAAATTTATAACACAATTGTAACAGCTCATGCTTTCATTATAATTTTTTTATA
GTTATACCTATTATAATTGGAGGATTTGGAAATTGATTGGTACCTTTAATATTAGGAGCCCCCGATATAGCTTTCCCACG
AATAAATAATATAAGATTTTGATTATTACCCCCATCTTTAACCCTACTAATTTCTAGAAGAATTGTAGAAAATGGAGCAG
GAACAGGATGAACAGTTTATCCCCCATTATCATCTAATATTGCCCATGGGGGAAGATCTGTGGACTTAGCAATTTTCTCT
TTACATTTAGCTGGGATTTCCTCAATTTTAGGGGCTATTAATTTCATCACTACCATTATTAATATACGAATTAATAATCT
ATCATTTGATCAAATACCTTTATTTATTTGATCAGTGGGCATTACAGCATTACTTTTATTACTTTCTTTACCAGTATTAG
CTGGAGCCATTACTATATTACTAACAGATCGAAATTTAAATACCTCCTTCTTTGATCCAGCGGGAGGGGGAGATCCTATT
CTATATCAACATTTATTTTGATTTTTT
>GBGL14947-14|Diatraea_saccharalis|JX442572|JX442572
------------------------TGAGCAGGAATATTAGGAACTTCATTAAGTTTATTAATTCGAGCAGAATTAGGAA
CATCTAACTCTTTAATTGGAGATGATCAAATTTATAACACAATTGTAACAGCTCATGCTTTCATTATAATTTTTTTATA
GTTATACCTATTATAATTGGAGGATTTGGAAATTGATTGGTACCTTTAATATTAGGAGCCCCCGATATAGCTTTCCCACG
AATAAATAATATAAGATTTTGATTATTACCCCCATCTTTAACCCTACTAATTTCTAGAAGAATTGTAGAAAATGGAGCAG
GAACAGGATGAACAGTTTATCCCCCATTATCATCTAATATTGCCCATGGGGGAAGATCTGTGGACTTAGCAATTTTCTCT
TTACATTTAGCTGGGATTTCCTCAATTTTAGGGGCTATTAATTTCATCACTACCATTATTAATATACGAATTAATAATCT
ATCATTTGATCAAATACCTTTATTTATTTGATCAGTGGGCATTACAGCATTACTTTTATTACTTTCTTTACCAGTATTAG
CTGGAGCCATTACTATATTACTAACAGATCGAAATTTAAATACCTCCTTCTTTGATCCAGCGGGAGGGGGAGATCCTATT

CTATATCAACATTTATTTTGATTTTTT
>GBGL14948-14|Diatraea_saccharalis|JX442573|JX442573
-------------------------TGAGCAGGAATATTAGGAACTTCATTAAGTTTATTAATTCGAGCAGAATTAGGAA
CATCTAACTCTTTAATTGGAGATGATCAAATTTATAACACAATTGTAACAGCTCATGCTTTCATTATAATTTTTTTATA
GTTATACCTATTATAATTGGAGGATTTGGAAATTGATTGGTACCTTTAATATTAGGAGCCCCCGATATAGCTTTCCCACG
AATAAATAATATAAGATTTTGATTATTACCCCCATCTTTAACCCTACTAATTTCTAGAAGAATTGTAGAAAATGGAGCAG
GAACAGGATGAACAGTTTATCCCCCATTATCATCTAATATTGCCCATGGGGGAAGATCTGTGGACTTAGCAATTTTCTCT
TTACATTTAGCTGGGATTTCCTCAATTTTAGGGGCTATTAATTTCATCACTACCATTATTAATATACGAATTAATAATCT
ATCATTTGATCAAATACCTTTATTTATTTGATCAGTGGGCATTACAGCATTACTTTTATTACTTTCTTTACCAGTATTAG
CTGGAGCCATTACTATATTACTAACAGATCGAAATTTAAATACCTCCTTCTTTGATCCAGCGGGAGGGGGAGATCCTATT
CTATATCAACATTTATTTTGATTTTTT
>GBGL14949-14|Diatraea_saccharalis|JX442574|JX442574
-------------------------TGAGCAGGAATATTAGGAACTTCATTAAGTTTATTAATTCGAGCAGAATTAGGAA
CATCTAACTCTTTAATTGGAGATGATCAAATTTATAACACAATTGTAACAGCTCATGCTTTCATTATAATTTTTTTATA
GTTATACCTATTATAATTGGAGGATTTGGAAATTGATTGGTACCTTTAATATTAGGAGCCCCCGATATAGCTTTCCCACG
AATAAATAATATAAGATTTTGATTATTACCCCCATCTTTAACCCTACTAATTTCTAGAAGAATTGTAGAAAATGGAGCAG
GAACAGGATGAACAGTTTATCCCCCATTATCATCTAATATTGCCCATGGGGGAAGATCTGTGGACTTAGCAATTTTCTCT
TTACATTTAGCTGGGATTTCCTCAATTTTAGGGGCTATTAATTTCATCACTACCATTATTAATATACGAATTAATAATCT
ATCATTTGATCAAATACCTTTATTTATTTGATCAGTGGGCATTACAGCATTACTTTTATTACTTTCTTTACCAGTATTAG
CTGGAGCCATTACTATATTACTAACAGATCGAAATTTAAATACCTCCTTCTTTGATCCAGCGGGAGGGGGAGATCCTATT
CNATATCAACATTTATTTTGATTTTTT
>GBGL14950-14|Diatraea_saccharalis|JX442576|JX442576
-------------------------TGAGCAGGAATATTAGGAACTTCATTAAGTTTATTAATTCGAGCAGAATTAGGAA
CATCTAACTCTTTAATTGGAGATGATCAAATTTATAACACAATTGTAACAGCTCATGCTTTCATTATAATTTTTTTATA
GTTATACCTATTATAATTGGAGGATTTGGAAATTGATTGGTACCTTTAATATTAGGAGCCCCCGATATAGCTTTCCCACG
AATAAATAATATAAGATTTTGATTATTACCCCCATCTTTAACCCTACTAATTTCTAGAAGAATTGTAGAAAATGGAGCAG
GAACAGGATGAACAGTTTATCCCCCATTATCATCTAATATTGCCCATGGGGGAAGATCTGTGGACTTAGCAATTTTCTCT
TTACATTTAGCTGGGATTTCCTCAATTTTAGGGGCTATTAATTTCATCACTACCATTATTAATATACGAATTAATAATCT
ATCATTTGATCAAATACCTTTATTTATTTGATCAGTGGGCATTACAGCATTACTTTTATTACTTTCTTTACCAGTATTAG
CTGGAGCCATTACTATATTACTAACAGATCGAAATTTAAATACCTCCTTCTTTGATCCAGCGGGAGGGGGAGATCCTATT
CTATATCAACATTTATTTTGATTTTTT
>GBGL14951-14|Diatraea_saccharalis|JX442577|JX442577
-------------------------TGAGCAGGAATATTAGGAACTTCATTAAGTTTATTAATTCGAGCAGAATTAGGAA
CATCTAACTCTTTAATTGGAGATGATCAAATTTATAACACAATTGTAACAGCTCATGCTTTCATTATAATTTTTTTATA
GTTATACCTATTATAATTGGAGGATTTGGAAATTGATTGGTACCTTTAATATTAGGAGCCCCCGATATAGCTTTCCCACG
AATAAATAATATAAGATTTTGATTATTACCCCCATCTTTAACCCTACTAATTTCTAGAAGAATTGTAGAAAATGGAGCAG
GAACAGGATGAACAGTTTATCCCCCATTATCATCTAATATTGCCCATGGGGGAAGATCTGTGGACTTAGCAATTTTCTCT
TTACATTTAGCTGGGATTTCCTCAATTTTAGGGGCTATTAATTTCATCACTACCATTATTAATATACGAATTAATAATCT
ATCATTTGATCAAATACCTTTATTTATTTGATCAGTGGGCATTACAGCATTACTTTTATTACTTTCTTTACCAGTATTAG
CTGGAGCCATTACTATATTACTAACAGATCGAAATTTAAATACCTCCTTCTTTGATCCAGCGGGAGGGGGAGATCCTATT
CTATATCAACATTTATTTTGATTTTTT
>GBGL14952-14|Diatraea_saccharalis|JX442578|JX442578
-------------------------TGAGCAGGAATATTAGGAACTTCATTAAGTTTATTAATTCGAGCAGAATTAGGAA
CATCTAACTCTTTAATTGGAGATGATCAAATTTATAACACAATTGTAACAGCTCATGCTTTCATTATAATTTTTTTATA
GTTATACCTATTATAATTGGAGGATTTGGAAATTGATTGGTACCTTTAATATTAGGAGCCCCCGATATAGCTTTCCCACG
AATAAATAATATAAGATTTTGATTATTACCCCCATCTTTAACCCTACTAATTTCTAGAAGAATTGTAGAAAATGGAGCAG
GAACAGGATGAACAGTTTATCCCCCATTATCATCTAATATTGCCCATGGGGGAAGATCTGTGGACTTAGCAATTTTCTCT
TTACATTTAGCTGGGATTTCCTCAATTTTAGGGGCTATTAATTTCATCACTACCATTATTAATATACGAATTAATAATCT
ATCATTTGATCAAATACCTTTATTTATTTGATCAGTGGGCATTACAGCATTACTTTTATTACTTTCTTTACCAGTATTAG
CTGGAGCCATTACTATATTACTAACAGATCGAAATTTAAATACCTCCTTCTTTGATCCAGCGGGAGGGGGAGATCCTATT
CTATATCAACATTTATTTTGATTTTTT
>GBGL14953-14|Diatraea_saccharalis|JX442579|JX442579
-------------------------TGAGCAGGAATATTAGGAACTTCATTAAGTTTATTAATTCGAGCAGAATTAGGAA
CATCTAACTCTTTAATTGGAGATGATCAAATTTATAACACAATTGTAACAGCTCATGCTTTCATTATAATTTTTTTATA
GTTATACCTATTATAATTGGAGGATTTGGAAATTGATTGGTACCTTTAATATTAGGAGCCCCCGATATAGCTTTCCCACG
AATAAATAATATAAGATTTTGATTATTACCCCCATCTTTAACCCTACTAATTTCTAGAAGAATTGTAGAAAATGGAGCAG
GAACAGGATGAACAGTTTATCCCCCATTATCATCTAATATTGCCCATGGGGGAAGATCTGTGGACTTAGCAATTTTCTCT

TTACATTTAGCTGGGATTTCCTCAATTTTAGGGGCTATTAATTTCATCACTACCATTATTAATATACGAATTAATAATCT
ATCATTTGATCAAATACCTTTATTTATTTGATCAGTGGGCATTACAGCATTACTTTTATTACTTTCTTTACCAGTATTAG
CTGGAGCCATTACTATATTACTAACAGATCGAAATTTAAATACCTCCTTCTTTGATCCAGCGGGAGGGGGAGATCCTATT
CTATATCAACATTTATTTTGATTTTTT
>GBGL14954-14|Diatraea_saccharalis|JX442580|JX442580
------------------------TGAGCAGGAATATTAGGAACTTCATTAAGTTTATTAATTCGAGCAGAATTAGGAA
CATCTAACTCTTTAATTGGAGATGATCAAATTTATAACACAATTGTAACAGCTCATGCTTTCATTATAATTTTTTTATA
GTTATACCTATTATAATTGGAGGATTTGGAAATTGATTGGTACCTTTAATATTAGGAGCCCCCGATATAGCTTTCCCACG
AATAAATAATATAAGATTTTGATTATTACCCCCATCTTTAACCCTACTAATTTCTAGAAGAATTGTAGAAAATGGAGCAG
GAACAGGATGAACAGTTTATCCCCCATTATCATCTAATATTGCCCATGGGGGAAGATCTGTGGACTTAGCAATTTTCTCT
TTACATTTAGCTGGGATTTCCTCAATTTTAGGGGCTATTAATTTCATCACTACCATTATTAATATACGAATTAATAATCT
ATCATTTGATCAAATACCTTTATTTATTTGATCAGTGGGCATTACAGCATTACTTTTATTACTTTCTTTACCAGTATTAG
CTGGAGCCATTACTATATTACTAACAGATCGAAATTTAAATACCTCCTTCTTTGATCCAGCGGGAGGGGGAGATCCTATT
CTATATCAACATTTATTTTGATTTTTT
>GBGL14955-14|Diatraea_saccharalis|JX442581|JX442581
------------------------TGAGCAGGAATATTAGGAACTTCATTAAGTTTATTAATTCGAGCAGAATTAGGAA
CATCTAACTCTTTAATTGGAGATGATCAAATTTATAACACAATTGTAACAGCTCATGCTTTCATTATAATTTTTTTATA
GTTATACCTATTATAATTGGAGGATTTGGAAATTGATTGGTACCTTTAATATTAGGAGCCCCCGATATAGCTTTCCCACG
AATAAATAATATAAGATTTTGATTATTACCCCCATCTTTAACCCTACTAATTTCTAGAAGAATTGTAGAAAATGGAGCAG
GAACAGGATGAACAGTTTATCCCCCATTATCATCTAATATTGCCCATGGGGGAAGATCTGTGGACTTAGCAATTTTCTCT
TTACATTTAGCTGGGATTTCCTCAATTTTAGGGGCTATTAATTTCATCACTACCATTATTAATATACGAATTAATAATCT
ATCATTTGATCAAATACCTTTATTTATTTGATCAGTGGGCATTACAGCATTACTTTTATTACTTTCTTTACCAGTATTAG
CTGGAGCCATTACTATATTACTAACAGATCGAAATTTAAATACCTCCTTCTTTGATCCAGCGGGAGGGGGAGATCCTATT
CTATATCAACATTTATTTTGATTTTTT
>GBGL14956-14|Diatraea_saccharalis|JX442582|JX442582
------------------------TGAGCAGGAATATTAGGAACTTCATTAAGTTTATTAATTCGAGCAGAATTAGGAA
CATCTAACTCTTTAATTGGAGATGATCAAATTTATAACACAATTGTAACAGCTCATGCTTTCATTATAATTTTTTTATA
GTTATACCTATTATAATTGGAGGATTTGGAAATTGATTGGTACCTTTAATATTAGGAGCCCCCGATATAGCTTTCCCACG
AATAAATAATATAAGATTTTGATTATTACCCCCATCTTTAACCCTACTAATTTCTAGAAGAATTGTAGAAAATGGAGCAG
GAACAGGATGAACAGTTTATCCCCCATTATCATCTAATATTGCCCATGGGGGAAGATCTGTGGACTTAGCAATTTTCTCT
TTACATTTAGCTGGGATTTCCTCAATTTTAGGGGCTATTAATTTCATCACTACCATTATTAATATACGAATTAATAATCT
ATCATTTGATCAAATACCTTTATTTATTTGATCAGTGGGCATTACAGCATTACTTTTATTACTTTCTTTACCAGTATTAG
CTGGAGCCATTACTATATTACTAACAGATCGAAATTTAAATACCTCCTTCTTTGATCCAGCGGGAGGGGGAGATCCTATT
CTATATCAACATTTATTTTGATTTTTT
>GBGL14957-14|Diatraea_saccharalis|JX442583|JX442583
------------------------TGAGCAGGAATATTAGGAACTTCATTAAGTTTATTAATTCGAGCAGAATTAGGAA
CATCTAACTCTTTAATTGGAGATGATCAAATTTATAACACAATTGTAACAGCTCATGCTTTCATTATAATTTTTTTATA
GTTATACCTATTATAATTGGAGGATTTGGAAATTGATTGGTACCTTTAATATTAGGAGCCCCCGATATAGCTTTCCCACG
AATAAATAATATAAGATTTTGATTATTACCCCCATCTTTAACCCTACTAATTTCTAGAAGAATTGTAGAAAATGGAGCAG
GAACAGGATGAACAGTTTATCCCCCATTATCATCTAATATTGCCCATGGGGGAAGATCTGTGGACTTAGCAATTTTCTCT
TTACATTTAGCTGGGATTTCCTCAATTTTAGGGGCTATTAATTTCATCACTACCATTATTAATATACGAATTAATAATCT
ATCATTTGATCAAATACCTTTATTTATTTGATCAGTGGGCATTACAGCATTACTTTTATTACTTTCTTTACCAGTATTAG
CTGGAGCCATTACTATATTACTAACAGATCGAAATTTAAATACCTCCTTCTTTGATCCAGCGGGAGGGGGAGATCCTATT
CTATATCAACATTTATTTTGATTTTTT
>GBGL14958-14|Diatraea_saccharalis|JX442584|JX442584
------------------------TGAGCAGGAATATTAGGAACTTCATTAAGTTTATTAATTCGAGCAGAATTAGGAA
CATCTAACTCTTTAATTGGAGATGATCAAATTTATAACACAATTGTAACAGCTCATGCTTTCATTATAATTTTTTTATA
GTTATACCTATTATAATTGGAGGATTTGGAAATTGATTGGTACCTTTAATATTAGGAGCCCCCGATATAGCTTTCCCACG
AATAAATAATATAAGATTTTGATTATTACCCCCATCTTTAACCCTACTAATTTCTAGAAGAATTGTAGAAAATGGAGCAG
GAACAGGATGAACAGTTTATCCCCCATTATCATCTAATATTGCCCATGGGGGAAGATCTGTGGACTTAGCAATTTTCTCT
TTACATTTAGCTGGGATTTCCTCAATTTTAGGGGCTATTAATTTCATCACTACCATTATTAATATACGAATTAATAATCT
ATCATTTGATCAAATACCTTTATTTATTTGATCAGTGGGCATTACAGCATTACTTTTATTACTTTCTTTACCAGTATTAG
CTGGAGCCATTACTATATTACTAACAGATCGAAATTTAAATACCTCCTTCTTTGATCCAGCGGGAGGGGGAGATCCTATT
CTATATCAACATTTATTTTGATTTTTT
>GBGL14959-14|Diatraea_saccharalis|JX442585|JX442585
------------------------TGAGCAGGAATATTAGGAACTTCATTAAGTTTATTAATTCGAGCAGAATTAGGAA
CATCTAACTCTTTAATTGGAGATGATCAAATTTATAACACAATTGTAACAGCTCATGCTTTCATTATAATTTTTTTATA

GTTATACCTATTATAATTGGAGGATTTGGAAATTGATTGGTACCTTTAATATTAGGAGCCCCCGATATAGCTTTCCCACG
AATAAATAATATAAGATTTTGATTATTACCCCCATCTTTAACCCTACTAATTTCTAGAAGAATTGTAGAAAATGGAGCAG
GAACAGGATGAACAGTTTATCCCCCATTATCATCTAATATTGCCCATGGGGGAAGATCTGTGGACTTAGCAATTTTCTCT
TTACATTTAGCTGGGATTTCCTCAATTTTAGGGGCTATTAATTTCATCACTACCATTATTAATATACGAATTAATAATCT
ATCATTTGATCAAATACCTTTATTTATTTGATCAGTGGGTATTACAGCATTACTTTTATTACTTTCTTTACCAGTATTAG
CTGGAGCCATTACTATATTACTAACAGATCGAAATTTAAATACCTCCTTCTTTGATCCAGCAGGAGGGGGAGATCCTATT
CTATATCAACATTTATTTTGATTTTTT
>GBGL14960-14|Diatraea_saccharalis|JX442586|JX442586
-------------------------TGAGCAGGAATATTAGGAACTTCATTAAGTTTATTAATTCGAGCAGAATTAGGAA
CATCTAACTCTTTAATTGGAGATGATCAAATTTATAACACAATTGTAACAGCTCATGCTTTCATTATAATTTTTTTATA
GTTATACCTATTATAATTGGAGGATTTGGAAATTGATTGGTACCTTTAATATTAGGAGCCCCCGATATAGCTTTCCCACG
AATAAATAATATAAGATTTTGATTATTACCCCCATCTTTAACCCTACTAATTTCTAGAAGAATTGTAGAAAATGGAGCAG
GAACAGGATGAACAGTTTATCCCCCATTATCATCTAATATTGCCCATGGGGGAAGATCTGTGGACTTAGCAATTTTCTCT
TTACATTTAGCTGGGATTTCCTCAATTTTAGGGGCTATCAATTTCATCACTACCATTATTAATATACGAATTAATAATCT
ATCATTTGATCAAATACCTTTATTTATTTGATCAGTGGGTATTACAGCATTACTTTTATTACTTTCTTTACCAGTATTAG
CTGGAGCCATTACTATATTACTAACAGATCGAAATTTAAATACCTCCTTCTTTGATCCAGCAGGAGGGGGAGATCCTATT
CTATATCAACATTTATTTTGATTTTTT
>GBGL14961-14|Diatraea_saccharalis|JX442587|JX442587
-------------------------TGAGCAGGAATATTAGGAACTTCATTAAGTTTATTAATTCGAGCAGAATTAGGAA
CATCTAACTCTTTAATTGGAGATGATCAAATTTATAACACAATTGTAACAGCTCATGCTTTCATTATAATTTTTTTATA
GTTATACCTATTATAATTGGAGGATTTGGAAATTGATTGGTACCTTTAATATTAGGAGCCCCCGATATAGCTTTCCCACG
AATAAATAATATAAGATTTTGATTATTACCCCCATCTTTAACCCTACTAATTTCTAGAAGAATTGTAGAAAATGGAGCAG
GAACAGGATGAACAGTTTATCCCCCATTATCATCTAATATTGCCCATGGGGGAAGATCTGTGGACTTAGCAATTTTCTCT
TTACATTTAGCTGGGATTTCCTCAATTTTAGGGGCTATCAATTTCATCACTACCATTATTAATATACGAATTAATAATCT
ATCATTTGATCAAATACCTTTATTTATTTGATCAGTGGGTATTACAGCATTACTTTTATTACTTTCTTTACCAGTATTAG
CTGGAGCCATTACTATATTACTAACAGATCGAAATTTAAATACCTCCTTCTTTGATCCAGCAGGAGGGGGAGATCCTATT
CTATATCAACATTTATTTTGATTTTTT
>GBGL14962-14|Diatraea_saccharalis|JX442588|JX442588
-------------------------TGAGCAGGAATATTAGGAACTTCATTAAGTTTATTAATTCGAGCAGAATTAGGAA
CATCTAACTCTTTAATTGGAGATGATCAAATTTATAACACAATTGTAACAGCTCATGCTTTCATTATAATTTTTTTATA
GTTATACCTATTATAATTGGAGGATTTGGAAATTGATTGGTACCTTTAATATTAGGAGCCCCCGATATAGCTTTCCCACG
AATAAATAATATAAGATTTTGATTATTACCCCCATCTTTAACCCTACTAATTTCTAGAAGAATTGTAGAAAATGGAGCAG
GAACAGGATGAACAGTTTATCCCCCATTATCATCTAATATTGCCCATGGGGGAAGATCTGTGGACTTAGCAATTTTCTCT
TTACATTTAGCTGGGATTTCCTCAATTTTAGGGGCTATTAATTTCATCACTACCATTATTAATATACGAATTAATAATCT
ATCATTTGATCAAATACCTTTATTTATTTGATCAGTGGGCATTACAGCATTACTTTTATTACTTTCTTTACCAGTATTAG
CTGGAGCCATTACTATATTACTAACAGATCGAAATTTAAATACCTCCTTCTTTGATCCAGCGGGAGGGGGAGATCCTATT
CTATATCAACATTTATTTTGATTTTTT
>GBGL14963-14|Diatraea_saccharalis|JX442589|JX442589
-------------------------TGAGCAGGAATATTAGGAACTTCATTAAGTTTATTAATTCGAGCAGAATTAGGAA
CATCTAACTCTTTAATTGGAGATGATCAAATTTATAACACAATTGTAACAGCTCATGCTTTCATTATAATTTTTTTATA
GTTATACCTATTATAATTGGAGGATTTGGAAATTGATTGGTACCTTTAATATTAGGAGCCCCCGATATAGCTTTCCCACG
AATAAATAATATAAGATTTTGATTATTACCCCCATCTTTAACCCTACTAATTTCTAGAAGAATTGTAGAAAATGGAGCAG
GAACAGGATGAACAGTTTATCCCCCATTATCATCTAATATTGCCCATGGGGGAAGATCTGTGGACTTAGCAATTTTCTCT
TTACATTTAGCTGGGATTTCCTCAATTTTAGGGGCTATTAATTTCATCACTACCATTATTAATATACGAATTAATAATCT
ATCATTTGATCAAATACCTTTATTTATTTGATCAGTGGGCATTACAGCATTACTTTTATTACTTTCTTTACCAGTATTAG
CTGGAGCCATTACTATATTACTAACAGATCGAAATTTAAATACCTCCTTCTTTGATCCAGCGGGAGGGGGAGATCCTATT
CTATATCAACATTTATTTTGATTTTTT
>GBGL14964-14|Diatraea_saccharalis|JX442590|JX442590
-------------------------TGAGCAGGAATATTAGGAACTTCATTAAGTTTATTAATTCGAGCAGAATTAGGAA
CATCTAACTCTTTAATTGGAGATGATCAAATTTATAACACAATTGTAACAGCTCATGCTTTCATTATAATTTTTTTATA
GTTATACCTATTATAATTGGAGGATTTGGAAATTGATTGGTACCTTTAATATTAGGAGCCCCCGATATAGCTTTCCCACG
AATAAATAATATAAGATTTTGATTATTACCCCCATCTTTAACCCTACTAATTTCTAGAAGAATTGTAGAAAATGGAGCAG
GAACAGGATGAACAGTTTATCCCCCATTATCATCTAATATTGCCCATGGGGGAAGATCTGTGGACTTAGCAATTTTCTCT
TTACATTTAGCTGGAATTTCCTCAATTTTAGGGGCTATCAATTTCATCACTACCATTATTAATATACGAATTAATAATCT
ATCATTTGATCAAATACCTTTATTTATTTGATCAGTGGGTATTACAGCATTACTTTTATTACTTTCTTTACCAGTATTAG
CTGGAGCCATTACTATATTACTAACAGATCGAAATTTAAATACCTCCTTCTTTGATCCAGCAGGAGGGGGAGATCCTATT
CTATATCAACATTTATTTTGATTTTTT

>GBGL14965-14|Diatraea_saccharalis|JX442591|JX442591
------------------------TGAGCAGGAATATTAGGAACTTCATTAAGTTTATTAATTCGAGCAGAATTAGGAA
CATCTAACTCTTTAATTGGAGATGATCAAATTTATAACACAATTGTAACAGCTCATGCTTTCATTATAATTTTTTTATA
GTTATACCTATTATAATTGGAGGATTTGGAAATTGATTGGTACCTTTAATATTAGGAGCCCCCGATATAGCTTTCCCACG
AATAAATAATATAAGATTTTGATTATTACCCCCATCTTTAACCCTACTAATTTCTAGAAGAATTGTAGAAAATGGAGCAG
GAACAGGATGAACAGTTTATCCCCCATTATCATCTAATATTGCCCATGGGGGAAGATCTGTGGACTTAGCAATTTTCTCT
TTACATTTAGCTGGGATTTCCTCAATTTTAGGGGCTATTAATTTCATCACTACCATTATTAATATACGAATTAATAATCT
ATCATTTGATCAAATACCTTTATTTATTTGATCAGTGGGCATTACAGCATTACTTTTATTACTTTCTTTACCAGTATTAG
CTGGAGCCATTACTATATTACTAACAGATCGAAATTTAAATACCTCCTTCTTTGATCCAGCGGGAGGGGGAGATCCTATT
CTATATCAACATTTATTTTGATTTTTT
>GBGL14966-14|Diatraea_saccharalis|JX442592|JX442592
------------------------TGAGCAGGAATATTAGGAACTTCATTAAGTTTATTAATTCGAGCAGAATTAGGAA
CATCTAACTCTTTAATTGGAGATGATCAAATTTATAACACAATTGTAACAGCTCATGCTTTCATTATAATTTTTTTATA
GTTATACCTATTATAATTGGAGGATTTGGAAATTGATTGGTACCTTTAATATTAGGAGCCCCCGATATAGCTTTCCCACG
AATAAATAATATAAGATTTTGATTATTACCCCCATCTTTAACCCTACTAATTTCTAGAAGAATTGTAGAAAATGGAGCAG
GAACAGGATGAACAGTTTATCCCCCATTATCATCTAATATTGCCCATGGGGGAAGATCTGTGGACTTAGCAATTTTCTCT
TTACATTTAGCTGGGATTTCCTCAATTTTAGGGGCTATTAATTTCATCACTACCATTATTAATATACGAATTAATAATCT
ATCATTTGATCAAATACCTTTATTTATTTGATCAGTGGGCATTACAGCATTACTTTTATTACTTTCTTTACCAGTATTAG
CTGGAGCCATTACTATATTACTAACAGATCGAAATTTAAATACCTCCTTCTTTGATCCAGCGGGAGGGGGAGATCCTATT
CTATATCAACATTTATTTTGATTTTTT
>GBGL14967-14|Diatraea_saccharalis|JX442593|JX442593
------------------------TGAGCAGGAATATTAGGAACTTCATTAAGTTTATTAATTCGAGCAGAATTAGGAA
CATCTAACTCTTTAATTGGAGATGATCAAATTTATAACACAATTGTAACAGCTCATGCTTTCATTATAATTTTTTTATA
GTTATACCTATTATAATTGGAGGATTTGGAAATTGATTGGTACCTTTAATATTAGGAGCCCCCGATATAGCTTTCCCACG
AATAAATAATATAAGATTTTGATTATTACCCCCATCTTTAACCCTACTAATTTCTAGAAGAATTGTAGAAAATGGAGCAG
GAACAGGATGAACAGTTTATCCCCCATTATCATCTAATATTGCCCATGGGGGAAGATCTGTGGACTTAGCAATTTTCTCT
TTACATTTAGCTGGGATTTCCTCAATTTTAGGGGCTATTAATTTCATCACTACCATTATTAATATACGAATTAATAATCT
ATCATTTGATCAAATACCTTTATTTATTTGATCAGTGGGCATTACAGCATTACTTTTATTACTTTCTTTACCAGTATTAG
CTGGAGCCATTACTATATTACTAACAGATCGAAATTTAAATACCTCCTTCTTTGATCCAGCGGGAGGGGGAGATCCTATT
CTATATCAACATTTATTTTGATTTTTT
>GBGL14968-14|Diatraea_saccharalis|JX442594|JX442594
------------------------TGAGCAGGAATATTAGGAACTTCATTAAGTTTATTAATTCGAGCAGAATTAGGAA
CATCTAACTCTTTAATTGGAGATGATCAAATTTATAACACAATTGTAACAGCTCATGCTTTCATTATAATTTTTTTATA
GTTATACCTATTATAATTGGAGGATTTGGAAATTGATTGGTACCTTTAATATTAGGAGCCCCCGATATAGCTTTCCCACG
AATAAATAATATAAGATTTTGATTATTACCCCCATCTTTAACCCTACTAATTTCTAGAAGAATTGTAGAAAATGGAGCAG
GAACAGGATGAACAGTTTATCCCCCATTATCATCTAATATTGCCCATGGGGGAAGATCTGTGGACTTAGCAATTTTCTCT
TTACATTTAGCTGGGATTTCCTCAATTTTAGGGGCTATTAATTTCATCACTACCATTATTAATATACGAATTAATAATCT
ATCATTTGATCAAATACCTTTATTTATTTGATCAGTGGGCATTACAGCATTACTTTTATTACTTTCTTTACCAGTATTAG
CTGGAGCCATTACTATATTACTAACAGATCGAAATTTAAATACCTCCTTCTTTGATCCAGCGGGAGGGGGAGATCCTATT
CTATATCAACATTTATTTTGATTTTTT
>GBGL14969-14|Diatraea_saccharalis|JX442595|JX442595
------------------------TGAGCAGGAATATTAGGAACTTCATTAAGTTTATTAATTCGAGCAGAATTAGGAA
CATCTAACTCTTTAATTGGAGATGATCAAATTTATAACACAATTGTAACAGCTCATGCTTTCATTATAATTTTTTTATA
GTTATACCTATTATAATTGGAGGATTTGGAAATTGATTGGTACCTTTAATATTAGGAGCCCCCGATATAGCTTTCCCACG
AATAAATAATATAAGATTTTGATTATTACCCCCATCTTTAACCCTACTAATTTCTAGAAGAATTGTAGAAAATGGAGCAG
GAACAGGATGAACAGTTTATCCCCCATTATCATCTAATATTGCCCATGGGGGAAGATCTGTGGACTTAGCAATTTTCTCT
TTACATTTAGCTGGGATTTCCTCAATTTTAGGGGCTATTAATTTCATCACTACCATTATTAATATACGAATTAATAATCT
ATCATTTGATCAAATACCTTTATTTATTTGATCAGTGGGCATTACAGCATTACTTTTATTACTTTCTTTACCAGTATTAG
CTGGAGCCATTACTATATTACTAACAGATCGAAATTTAAATACCTCCTTCTTTGATCCAGCGGGAGGGGGAGATCCTATT
CTATATCAACATTTATTTTGATTTTTT
>GBGL14970-14|Diatraea_saccharalis|JX442596|JX442596
------------------------TGAGCAGGAATATTAGGAACTTCATTAAGTTTATTAATTCGAGCAGAATTAGGAA
CATCTAACTCTTTAATTGGAGATGATCAAATTTATAACACAATTGTAACAGCTCATGCTTTCATTATAATTTTTTTATA
GTTATACCTATTATAATTGGAGGATTTGGAAATTGATTGGTACCTTTAATATTAGGAGCCCCCGATATAGCTTTCCCACG
AATAAATAATATAAGATTTTGATTATTACCCCCATCTTTAACCCTACTAATTTCTAGAAGAATTGTAGAAAATGGAGCAG
GAACAGGATGAACAGTTTATCCCCCATTATCATCTAATATTGCCCATGGGGGAAGATCTGTGGACTTAGCAATTTTCTCT
TTACATTTAGCTGGGATTTCCTCAATTTTAGGGGCTATTAATTTCATCACTACCATTATTAATATACGAATTAATAATCT

ATCATTTGATCAAATACCTTTATTTATTTGATCAGTGGGCATTACAGCATTACTTTTATTACTTTCTTTACCAGTATTAG
CTGGAGCCATTACTATATTACTAACAGATCGAAATTTAAATACTTCCTTCTTTGATCCAGCGGGAGGGGGAGATCCTATT
CTATATCAACATTTATTTTGATTTTTT
>GBGL14971-14|Diatraea_saccharalis|JX442597|JX442597
------------------------TGAGCAGGAATATTAGGAACTTCATTAAGTTTATTAATTCGAGCAGAATTAGGAA
CATCTAACTCTTTAATTGGAGATGATCAAATTTATAACACAATTGTAACAGCTCATGCTTTCATTATAATTTTTTTTATA
GTTATACCTATTATAATTGGAGGATTTGGAAATTGATTGGTACCTTTAATATTAGGAGCCCCCGATATAGCTTTCCCACG
AATAAATAATATAAGATTTTGATTATTACCCCCATCTTTAACCCTACTAATTTCTAGAAGAATTGTAGAAAATGGAGCAG
GAACAGGATGAACAGTTTATCCCCCATTATCATCTAATATTGCCCATGGGGGAAGATCTGTGGACTTAGCAATTTTCTCT
TTACATTTAGCTGGGATTTCCTCAATTTTAGGGGCTATTAATTTCATCACTACCATTATTAATATACGAATTAATAATCT
ATCATTTGATCAAATACCTTTATTTATTTGATCAGTGGGCATTACAGCATTACTTTTATTACTTTCTTTACCAGTATTAG
CTGGAGCCATTACTATATTACTAACAGATCGAAATTTAAATACCTCCTTCTTTGATCCAGCGGGAGGGGGAGATCCTATT
CTATATCAACATTTATTTTGATTTTTT
>GBGL14972-14|Diatraea_saccharalis|JX442599|JX442599
------------------------TGAGCAGGAATATTAGGAACTTCATTAAGTTTATTAATTCGAGCAGAATTAGGAA
CATCTAACTCTTTAATTGGAGATGATCAAATTTATAACACAATTGTAACAGCTCATGCTTTCATTATAATTTTTTTTATA
GTTATACCTATTATAATTGGAGGATTTGGAAATTGATTGGTACCTTTAATATTAGGAGCCCCCGATATAGCTTTCCCACG
AATAAATAATATAAGATTTTGATTATTACCCCCATCTTTAACCCTACTAATTTCTAGAAGAATTGTAGAAAATGGAGCAG
GAACAGGATGAACAGTTTATCCCCCATTATCATCTAATATTGCCCATGGGGGAAGATCTGTGGACTTAGCAATTTTCTCT
TTACATTTAGCTGGGATTTCCTCAATTTTAGGGGCTATTAATTTCATCACTACCATTATTAATATACGAATTAATAATCT
ATCATTTGATCAAATACCTTTATTTATTTGATCAGTGGGCATTACAGCATTACTTTTATTACTTTCTTTACCAGTATTAG
CTGGAGCCATTACTATATTACTAACAGATCGAAATTTAAATACCTCCTTCTTTGATCCAGCGGGAGGGGGAGATCCTATT
CTATATCAACATTTATTTTGATTTTTT
>GBGL14973-14|Diatraea_saccharalis|JX442600|JX442600
------------------------TGAGCAGGAATATTAGGAACTTCATTAAGTTTATTAATTCGAGCAGAATTAGGAA
CATCTAACTCTTTAATTGGAGATGATCAAATTTATAACACAATTGTAACAGCTCATGCTTTCATTATAATTTTTTTTATA
GTTATACCTATTATAATTGGAGGATTTGGAAATTGATTGGTACCTTTAATATTAGGAGCCCCCGATATAGCTTTCCCACG
AATAAATAATATAAGATTTTGATTATTACCCCCATCTTTAACCCTACTAATTTCTAGAAGAATTGTAGAAAATGGAGCAG
GAACAGGATGAACAGTTTATCCCCCATTATCATCTAATATTGCCCATGGGGGAAGATCTGTGGACTTAGCAATTTTCTCT
TTACATTTAGCTGGGATTTCCTCAATTTTAGGGGCTATTAATTTCATCACTACCATTATTAATATACGAATTAATAATCT
ATCATTTGATCAAATACCTTTATTTATTTGATCAGTGGGCATTACAGCATTACTTTTATTACTTTCTTTACCAGTATTAG
CTGGAGCCATTACTATATTACTAACAGATCGAAATTTAAATACCTCCTTCTTTGATCCAGCGGGAGGGGGAGATCCTATT
CTATATCAACATTTATTTTGATTTTTT
>GBGL14974-14|Diatraea_saccharalis|JX442601|JX442601
------------------------TGAGCAGGAATATTAGGAACTTCATTAAGTTTATTAATTCGAGCAGAATTAGGAA
CATCTAACTCTTTAATTGGAGATGATCAAATTTATAACACAATTGTAACAGCTCATGCTTTCATTATAATTTTTTTTATA
GTTATACCTATTATAATTGGAGGATTTGGAAATTGATTGGTACCTTTAATATTAGGAGCCCCCGATATAGCTTTCCCACG
AATAAATAATATAAGATTTTGATTATTACCCCCATCTTTAACCCTACTAATTTCTAGAAGAATTGTAGAAAATGGAGCAG
GAACAGGATGAACAGTTTATCCCCCATTATCATCTAATATTGCCCATGGGGGAAGATCTGTGGACTTAGCAATTTTCTCT
TTACATTTAGCTGGGATTTCCTCAATTTTAGGGGCTATTAATTTCATCACTACCATTATTAATATACGAATTAATAATCT
ATCATTTGATCAAATACCTTTATTTATTTGATCAGTGGGCATTACAGCATTACTTTTATTACTTTCTTTACCAGTATTAG
CTGGAGCCATTACTATATTACTAACAGATCGAAATTTAAATACCTCCTTCTTTGATCCAGCGGGAGGGGGAGATCCTATT
CTATATCAACATTTATTTTGATTTTTT
>GBGL14975-14|Diatraea_saccharalis|JX442602|JX442602
------------------------TGAGCAGGAATATTAGGAACTTCATTAAGTTTATTAATTCGAGCAGAATTAGGAA
CATCTAACTCTTTAATTGGAGATGATCAAATTTATAACACAATTGTAACAGCTCATGCTTTCATTATAATTTTTTTTATA
GTTATACCTATTATAATTGGAGGATTTGGAAATTGATTGGTACCTTTAATATTAGGAGCCCCCGATATAGCTTTCCCACG
AATAAATAATATAAGATTTTGATTATTACCCCCATCTTTAACCCTACTAATTTCTAGAAGAATTGTAGAAAATGGAGCAG
GAACAGGATGAACAGTTTATCCCCCATTATCATCTAATATTGCCCATGGGGGAAGATCTGTGGACTTAGCAATTTTCTCT
TTACATTTAGCTGGGATTTCCTCAATTTTAGGGGCTATTAATTTCATCACTACCATTATTAATATACGAATTAATAATCT
ATCATTTGATCAAATACCTTTATTTATTTGATCAGTGGGCATTACAGCATTACTTTTATTACTTTCTTTACCAGTATTAG
CTGGAGCCATTACTATATTACTAACAGATCGAAATTTAAATACCTCCTTCTTTGATCCAGCGGGAGGGGGAGATCCTATT
CTATATCAACATTTATTTTGATTTTTT
>GBGL14976-14|Diatraea_saccharalis|JX442603|JX442603
------------------------TGAGCAGGAATATTAGGAACTTCATTAAGTTTATTAATTCGAGCAGAATTAGGAA
CATCTAACTCTTTAATTGGAGATGATCAAATTTATAACACAATTGTAACAGCTCATGCTTTCATTATAATTTTTTTTATA
GTTATACCTATTATAATTGGAGGATTTGGAAATTGATTGGTACCTTTAATATTAGGAGCCCCCGATATAGCTTTCCCACG

AATAAATAATATAAGATTTTGATTATTACCCCCATCTTTAACCCTACTAATTTCTAGAAGAATTGTAGAAAATGGAGCAG
GAACAGGATGAACAGTTTATCCCCCATTATCATCTAATATTGCCCATGGGGGAAGATCTGTGGACTTAGCAATTTTCTCT
TTACATTTAGCTGGGATTTCCTCAATTTTAGGGGCTATTAATTTCATCACTACCATTATTAATATACGAATTAATAATCT
ATCATTTGATCAAATACCTTTATTTATTTGATCAGTGGGCATTACAGCATTACTTTTATTACTTTCTTTACCAGTATTAG
CTGGAGCCATTACTATATTACTAACAGATCGAAATTTAAATACCTCCTTCTTTGATCCAGCGGGAGGGGGAGATCCTATT
CTATATCAACATTTATTTTGATTTTTT
>GBGL14977-14|Diatraea_saccharalis|JX442604|JX442604
-------------------------TGAGCAGGAATATTAGGAACTTCATTAAGTTTATTAATTCGAGCAGAATTAGGAA
CATCTAACTCTTTAATTGGAGATGATCAAATTTATAACACAATTGTAACAGCTCATGCTTTCATTATAATTTTTTTTATA
GTTATACCTATTATAATTGGAGGATTTGGAAATTGATTGGTACCTTTAATATTAGGAGCCCCCGATATAGCTTTCCCACG
AATAAATAATATAAGATTTTGATTATTACCCCCATCTTTAACCCTACTAATTTCTAGAAGAATTGTAGAAAATGGAGCAG
GAACAGGATGAACAGTTTATCCCCCATTATCATCTAATATTGCCCATGGGGGAAGATCTGTGGACTTAGCAATTTTCTCT
TTACATTTAGCTGGGATTTCCTCAATTTTAGGGGCTATTAATTTCATCACTACCATTATTAATATACGAATTAATAATCT
ATCATTTGATCAAATACCTTTATTTATTTGATCAGTGGGCATTACAGCATTACTTTTATTACTTTCTTTACCAGTATTAG
CTGGAGCCATTACTATATTACTAACAGATCGAAATTTAAATACCTCCTTCTTTGATCCAGCGGGAGGGGGAGATCCTATT
CTATATCAACATTTATTTTGATTTTTT
>GBGL14978-14|Diatraea_saccharalis|JX442605|JX442605
-------------------------TGAGCAGGAATATTAGGAACTTCATTAAGTTTATTAATTCGAGCAGAATTAGGAA
CATCTAACTCTTTAATTGGAGATGATCAAATTTATAACACAATTGTAACAGCTCATGCTTTCATTATAATTTTTTTTATA
GTTATACCTATTATAATTGGAGGATTTGGAAATTGATTGGTACCTTTAATATTAGGAGCCCCCGATATAGCTTTCCCACG
AATAAATAATATAAGATTTTGATTATTACCCCCATCTTTAACCCTACTAATTTCTAGAAGAATTGTAGAAAATGGAGCAG
GAACAGGATGAACAGTTTATCCCCCATTATCATCTAATATTGCCCATGGGGGAAGATCTGTGGACTTAGCAATTTTCTCT
TTACATTTAGCTGGGATTTCCTCAATTTTAGGGGCTATTAATTTCATCACTACCATTATTAATATACGAATTAATAATCT
ATCATTTGATCAAATACCTTTATTTATTTGATCAGTGGGCATTACAGCATTACTTTTATTACTTTCTTTACCAGTATTAG
CTGGAGCCATTACTATATTACTAACAGATCGAAATTTAAATACCTCCTTCTTTGATCCAGCGGGAGGGGGAGATCCTATT
CTATATCAACATTTATTTTGATTTTTT
>GBGL14979-14|Diatraea_saccharalis|JX442606|JX442606
-------------------------TGAGCAGGAATATTAGGAACTTCATTAAGTTTATTAATTCGAGCAGAATTAGGAA
CATCTAACTCTTTAATTGGAGATGATCAAATTTATAACACAATTGTAACAGCTCATGCTTTCATTATAATTTTTTTTATA
GTTATACCTATTATAATTGGAGGATTTGGAAATTGATTGGTACCTTTAATATTAGGAGCCCCCGATATAGCTTTCCCACG
AATAAATAATATAAGATTTTGATTATTACCCCCATCTTTAACCCTACTAATTTCTAGAAGAATTGTAGAAAATGGAGCAG
GAACAGGATGAACAGTTTATCCCCCATTATCATCTAATATTGCCCATGGAGGAAGATCTGTGGACTTAGCAATTTTCTCT
TTACATTTAGCTGGGATTTCCTCAATTTTAGGGGCTATCAATTTCATCACTACCATTATTAATATACGAATTAATAATCT
ATCATTTGATCAAATACCTTTATTTATTTGATCAGTGGGTATTACAGCATTACTTTTATTACTTTCTTTACCAGTATTAG
CTGGAGCCATTACTATATTACTAACAGATCGAAATTTAAATACCTCCTTCTTTGATCCAGCGGGAGGGGGAGATCCTATT
CTATATCAACATTTATTTTGATTTTTT
>GBGL14980-14|Diatraea_saccharalis|JX442607|JX442607
-------------------------TGAGCAGGAATATTAGGAACTTCATTAAGTTTATTAATTCGAGCAGAATTAGGAA
CATCTAACTCTTTAATTGGAGATGATCAAATTTATAACACAATTGTAACAGCTCATGCTTTCATTATAATTTTTTTTATA
GTTATACCTATTATAATTGGAGGATTTGGAAATTGATTGGTACCTTTAATATTAGGAGCCCCCGATATAGCTTTCCCACG
AATAAATAATATAAGATTTTGATTATTACCCCCATCTTTAACCCTACTAATTTCTAGAAGAATTGTAGAAAATGGAGCAG
GAACAGGATGAACAGTTTATCCCCCATTATCATCTAATATTGCCCATGGGGGAAGATCTGTGGACTTAGCAATTTTCTCT
TTACATTTAGCTGGGATTTCCTCAATTTTAGGGGCTATTAATTTCATCACTACCATTATTAATATACGAATTAATAATCT
ATCATTTGATCAAATACCTTTATTTATTTGATCAGTGGGCATTACAGCATTACTTTTATTACTTTCTTTACCAGTATTAG
CTGGAGCCATTACTATATTACTAACAGATCGAAATTTAAATACCTCCTTCTTTGATCCAGCGGGAGGGGGAGATCCTATT
CTATATCAACATTTATTTTGATTTTTT
>GBGL14981-14|Diatraea_saccharalis|JX442608|JX442608
-------------------------TGAGCAGGAATATTAGGAACTTCATTAAGTTTATTAATTCGAGCAGAATTAGGAA
CATCTAACTCTTTAATTGGAGATGATCAAATTTATAACACAATTGTAACAGCTCATGCTTTCATTATAATTTTTTTTATA
GTTATACCTATTATAATTGGAGGATTTGGAAATTGATTGGTACCTTTAATATTAGGAGCCCCCGATATAGCTTTCCCACG
AATAAATAATATAAGATTTTGATTATTACCCCCATCTTTAACCCTACTAATTTCTAGAAGAATTGTAGAAAATGGAGCAG
GAACAGGATGAACAGTTTATCCCCCATTATCATCTAATATTGCCCATGGGGGAAGATCTGTGGACTTAGCAATTTTCTCT
TTACATTTAGCTGGGATTTCCTCAATTTTAGGGGCTATTAATTTCATCACTACCATTATTAATATACGAATTAATAATCT
ATCATTTGATCAAATACCTTTATTTATTTGATCAGTGGGCATTACAGCATTACTTTTATTACTTTCTTTACCAGTATTAG
CTGGAGCCATTACTATATTACTAACAGATCGAAATTTAAATACCTCCTTCTTTGATCCAGCGGGAGGGGGAGATCCTATT
CTATATCAACATTTATTTTGATTTTTT
>GBGL14982-14|Diatraea_saccharalis|JX442609|JX442609

------------------------TGAGCAGGAATATTAGGAACTTCATTAAGTTTATTAATTCGAGCAGAATTAGGAA
CATCTAACTCTTTAATTGGAGATGATCAAATTTATAACACAATTGTAACAGCTCATGCTTTCATTATAATTTTTTTATA
GTTATACCTATTATAATTGGAGGATTTGGAAATTGATTGGTACCTTTAATATTAGGAGCCCCCGATATAGCTTTCCCACG
AATAAATAATATAAGATTTTGATTATTACCCCCATCTTTAACCCTACTAATTTCTAGAAGAATTGTAGAAAATGGAGCAG
GAACAGGATGAACAGTTTATCCCCCATTATCATCTAATATTGCCCATGGGGGAAGATCTGTGGACTTAGCAATTTTCTCT
TTACATTTAGCTGGGATTTCCTCAATTTTAGGGGCTATTAATTTCATCACTACCATTATTAATATACGAATTAATAATCT
ATCATTTGATCAAATACCTTTATTTATTTGATCAGTGGGCATTACAGCATTACTTTTATTACTTTCTTTACCAGTATTAG
CTGGAGCCATTACTATATTACTAACAGATCGAAATTTAAATACCTCCTTCTTTGATCCAGCGGGAGGGGGAGATCCTATT
CTATATCAACATTTATTTTGATTTTTT
>GBGL14983-14|Diatraea_saccharalis|JX442610|JX442610
------------------------TGAGCAGGAATATTAGGAACTTCATTAAGTTTATTAATTCGAGCAGAATTAGGAA
CATCTAACTCTTTAATTGGAGATGATCAAATTTATAACACAATTGTAACAGCTCATGCTTTCATTATAATTTTTTTATA
GTTATACCTATTATAATTGGAGGATTTGGAAATTGATTGGTACCTTTAATATTAGGAGCCCCCGATATAGCTTTCCCACG
AATAAATAATATAAGATTTTGATTATTACCCCCATCTTTAACCCTACTAATTTCTAGAAGAATTGTAGAAAATGGAGCAG
GAACAGGATGAACAGTTTATCCCCCATTATCATCTAATATTGCCCATGGGGGAAGATCTGTGGACTTAGCAATTTTCTCT
TTACATTTAGCTGGGATTTCCTCAATTTTAGGGGCTATTAATTTCATCACTACCATTATTAATATACGAATTAATAATCT
ATCATTTGATCAAATACCTTTATTTATTTGATCAGTGGGCATTACAGCATTACTTTTATTACTTTCTTTACCAGTATTAG
CTGGAGCCATTACTATATTACTAACAGATCGAAATTTAAATACCTCCTTCTTTGATCCAGCGGGAGGGGGAGATCCTATT
CTATATCAACATTTATTTTGATTTTTT
>GBGL14984-14|Diatraea_saccharalis|JX442611|JX442611
------------------------TGAGCAGGAATATTAGGAACTTCATTAAGTTTATTAATTCGAGCAGAATTAGGAA
CATCTAACTCTTTAATTGGAGATGATCAAATTTATAACACAATTGTAACAGCTCATGCTTTCATTATAATTTTTTTATA
GTTATACCTATTATAATTGGAGGATTTGGAAATTGATTGGTACCTTTAATATTAGGAGCCCCCGATATAGCTTTCCCACG
AATAAATAATATAAGATTTTGATTATTACCCCCATCTTTAACCCTACTAATTTCTAGAAGAATTGTAGAAAATGGAGCAG
GAACAGGATGAACAGTTTATCCCCCATTATCATCTAATATTGCCCATGGGGGAAGATCTGTGGACTTAGCAATTTTCTCT
TTACATTTAGCTGGGATTTCCTCAATTTTAGGGGCTATTAATTTCATCACTACCATTATTAATATACGAATTAATAATCT
ATCATTTGATCAAATACCTTTATTTATTTGATCAGTGGGCATTACAGCATTACTTTTATTACTTTCTTTACCAGTATTAG
CTGGAGCCATTACTATATTACTAACAGATCGAAATTTAAATACCTCCTTCTTTGATCCAGCGGGAGGGGGAGATCCTATT
CTATATCAACATTTATTTTGATTTTTT
>GBGL14985-14|Diatraea_saccharalis|JX442612|JX442612
------------------------TGAGCAGGAATATTAGGAACTTCATTAAGTTTATTAATTCGAGCAGAATTAGGAA
CATCTAACTCTTTAATTGGAGATGATCAAATTTATAACACAATTGTAACAGCTCATGCTTTCATTATAATTTTTTTATA
GTTATACCTATTATAATTGGAGGATTTGGAAATTGATTGGTACCTTTAATATTAGGAGCCCCCGATATAGCTTTCCCACG
AATAAATAATATAAGATTTTGATTATTACCCCCATCTTTAACCCTACTAATTTCTAGAAGAATTGTAGAAAATGGAGCAG
GAACAGGATGAACAGTTTATCCCCCATTATCATCTAATATTGCCCATGGGGGAAGATCTGTGGACTTAGCAATTTTCTCT
TTACATTTAGCTGGGATTTCCTCAATTTTAGGGGCTATTAATTTCATCACTACCATTATTAATATACGAATTAATAATCT
ATCATTTGATCAAATACCTTTATTTATTTGATCAGTGGGCATTACAGCATTACTTTTATTACTTTCTTTACCAGTATTAG
CTGGAGCCATTACTATATTACTAACAGATCGAAATTTAAATACCTCCTTCTTTGATCCAGCGGGAGGGGGAGATCCTATT
CTATATCAACATTTATTTTGATTTTTT
>GBGL14986-14|Diatraea_saccharalis|JX442613|JX442613
------------------------TGAGCAGGAATATTAGGAACTTCATTAAGTTTATTAATTCGAGCAGAATTAGGAA
CATCTAACTCTTTAATTGGAGATGATCAAATTTATAACACAATTGTAACAGCTCATGCTTTCATTATAATTTTTTTATA
GTTATACCTATTATAATTGGAGGATTTGGAAATTGATTGGTACCTTTAATATTAGGAGCCCCCGATATAGCTTTCCCACG
AATAAATAATATAAGATTTTGATTATTACCCCCATCTTTAACCCTACTAATTTCTAGAAGAATTGTAGAAAATGGAGCAG
GAACAGGATGAACAGTTTATCCCCCATTATCATCTAATATTGCCCATGGGGGAAGATCTGTGGACTTAGCAATTTTCTCT
TTACATTTAGCTGGGATTTCCTCAATTTTAGGGGCTATTAATTTCATCACTACCATTATTAATATACGAATTAATAATCT
ATCATTTGATCAAATACCTTTATTTATTTGATCAGTGGGCATTACAGCATTACTTTTATTACTTTCTTTACCAGTATTAG
CTGGAGCCATTACTATATTACTAACAGATCGAAATTTAAATACCTCCTTCTTTGATCCAGCGGGAGGGGGAGATCCTATT
CTATATCAACATTTATTTTGATTTTTT
>GBGL14987-14|Diatraea_saccharalis|JX442614|JX442614
------------------------TGAGCAGGAATATTAGGAACTTCATTAAGTTTATTAATTCGAGCAGAATTAGGAA
CATCTAACTCTTTAATTGGAGATGATCAAATTTATAACACAATTGTAACAGCTCATGCTTTCATTATAATTTTTTTATA
GTTATACCTATTATAATTGGAGGATTTGGAAATTGATTGGTACCTTTAATATTAGGAGCCCCCGATATAGCTTTCCCACG
AATAAATAATATAAGATTTTGATTATTACCCCCATCTTTAACCCTACTAATTTCTAGAAGAATTGTAGAAAATGGAGCAG
GAACAGGATGAACAGTTTATCCCCCATTATCATCTAATATTGCCCATGGGGGAAGATCTGTGGACTTAGCAATTTTCTCT
TTACATTTAGCTGGGATTTCCTCAATTTTAGGGGCTATTAATTTCATCACTACCATTATTAATATACGAATTAATAATCT
ATCATTTGATCAAATACCTTTATTTATTTGATCAGTGGGCATTACAGCATTACTTTTATTACTTTCTTTACCAGTATTAG

CTGGAGCCATTACTATATTACTAACAGATCGAAATTTAAATACCTCCTTCTTTGATCCAGCGGGAGGGGGAGATCCTATT
CTATATCAACATTTATTTTGATTTTTT
>GBGL14988-14|Diatraea_saccharalis|JX442615|JX442615
-------------------------TGAGCAGGAATATTAGGAACTTCATTAAGTTTATTAATTCGAGCAGAATTAGGAA
CATCTAACTCTTTAATTGGAGATGATCAAATTTATAACACAATTGTAACAGCTCATGCTTTCATTATAATTTTTTTATA
GTTATACCTATTATAATTGGAGGATTTGGAAATTGATTGGTACCTTTAATATTAGGAGCCCCCGATATAGCTTTCCCACG
AATAAATAATATAAGATTTTGATTATTACCCCCATCTTTAACCCTACTAATTTCTAGAAGAATTGTAGAAAATGGAGCAG
GAACAGGATGAACAGTTTATCCCCCATTATCATCTAATATTGCCCATGGGGGAAGATCTGTGGACTTAGCAATTTTCTCT
TTACATTTAGCTGGGATTTCCTCAATTTTAGGGGCTATTAATTTCATCACTACCATTATTAATATACGAATTAATAATCT
ATCATTTGATCAAATACCTTTATTTATTTGATCAGTGGGCATTACAGCATTACTTTTATTACTTTCTTTACCAGTATTAG
CTGGAGCCATTACTATATTACTAACAGATCGAAATTTAAATACCTCCTTCTTTGATCCAGCGGGAGGGGGAGATCCTATT
CTATATCAACATTTATTTTGATTTTTT
>GBGL14989-14|Diatraea_saccharalis|JX442616|JX442616
-------------------------TGAGCAGGAATATTAGGAACTTCATTAAGTTTATTAATTCGAGCAGAATTAGGAA
CATCTAACTCTTTAATTGGAGATGATCAAATTTATAACACAATTGTAACAGCTCATGCTTTCATTATAATTTTTTTATA
GTTATACCTATTATAATTGGAGGATTTGGAAATTGATTGGTACCTTTAATATTAGGAGCCCCCGATATAGCTTTCCCACG
AATAAATAATATAAGATTTTGATTATTACCCCCATCTTTAACCCTACTAATTTCTAGAAGAATTGTAGAAAATGGAGCAG
GAACAGGATGAACAGTTTATCCCCCATTATCATCTAATATTGCCCATGGGGGAAGATCTGTGGACTTAGCAATTTTCTCT
TTACATTTAGCTGGGATTTCCTCAATTTTAGGGGCTATTAATTTCATCACTACCATTATTAATATACGAATTAATAATCT
ATCATTTGATCAAATACCTTTATTTATTTGATCAGTGGGCATTACAGCATTACTTTTATTACTTTCTTTACCAGTATTAG
CTGGAGCCATTACTATATTACTAACAGATCGAAATTTAAATACCTCCTTCTTTGATCCAGCGGGAGGGGGAGATCCTATT
CTATATCAACATTTATTTTGATTTTTT
>GBGL14990-14|Diatraea_saccharalis|JX442617|JX442617
-------------------------TGAGCAGGAATATTAGGAACTTCATTAAGTTTATTAATTCGAGCAGAATTAGGAA
CATCTAACTCTTTAATTGGAGATGATCAAATTTATAACACAATTGTAACAGCTCATGCTTTCATTATAATTTTTTTATA
GTTATACCTATTATAATTGGAGGATTTGGAAATTGATTGGTACCTTTAATATTAGGAGCCCCCGATATAGCTTTCCCACG
AATAAATAATATAAGATTTTGATTATTACCCCCATCTTTAACCCTACTAATTTCTAGAAGAATTGTAGAAAATGGAGCAG
GAACAGGATGAACAGTTTATCCCCCATTATCATCTAATATTGCCCATGGGGGAAGATCTGTGGACTTAGCAATTTTCTCT
TTACATTTAGCTGGGATTTCCTCAATTTTAGGGGCTATTAATTTCATCACTACCATTATTAATATACGAATTAATAATCT
ATCATTTGATCAAATACCTTTATTTATTTGATCAGTGGGCATTACAGCATTACTTTTATTACTTTCTTTACCAGTATTAG
CTGGAGCCATTACTATATTACTAACAGATCGAAATTTAAATACCTCCTTCTTTGATCCAGCGGGAGGGGGAGATCCTATT
CTATATCAACATTTATTTTGATTTTTT
>GBGL14991-14|Diatraea_saccharalis|JX442618|JX442618
-------------------------TGAGCAGGAATATTAGGAACTTCATTAAGTTTATTAATTCGAGCAGAATTAGGAA
CATCTAACTCTTTAATTGGAGATGATCAAATTTATAACACAATTGTAACAGCTCATGCTTTCATTATAATTTTTTTATA
GTTATACCTATTATAATTGGAGGATTTGGAAATTGATTGGTACCTTTAATATTAGGAGCCCCCGATATAGCTTTCCCACG
AATAAATAATATAAGATTTTGATTATTACCCCCATCTTTAACCCTACTAATTTCTAGAAGAATTGTAGAAAATGGAGCAG
GAACAGGATGAACAGTTTATCCCCCATTATCATCTAATATTGCCCATGGGGGAAGATCTGTGGACTTAGCAATTTTCTCT
TTACATTTAGCTGGGATTTCCTCAATTTTAGGGGCTATTAATTTCATCACTACCATTATTAATATACGAATTAATAATCT
ATCATTTGATCAAATACCTTTATTTATTTGATCAGTGGGCATTACAGCATTACTTTTATTACTTTCTTTACCAGTATTAG
CTGGAGCCATTACTATATTACTAACAGATCGAAATTTAAATACCTCCTTCTTTGATCCAGCGGGAGGGGGAGATCCTATT
CTATATCAACATTTATTTTGATTTTTT
>GBGL14992-14|Diatraea_saccharalis|JX442619|JX442619
-------------------------TGAGCAGGAATATTAGGAACTTCATTAAGTTTATTAATTCGAGCAGAATTAGGAA
CATCTAACTCTTTAATTGGAGATGATCAAATTTATAACACAATTGTAACAGCTCATGCTTTCATTATAATTTTTTTATA
GTTATACCTATTATAATTGGAGGATTTGGAAATTGATTGGTACCTTTAATATTAGGAGCCCCCGATATAGCTTTCCCACG
AATAAATAATATAAGATTTTGATTATTACCCCCATCTTTAACCCTACTAATTTCTAGAAGAATTGTAGAAAATGGAGCAG
GAACAGGATGAACAGTTTATCCCCCATTATCATCTAATATTGCCCATGGGGGAAGATCTGTGGACTTAGCAATTTTCTCT
TTACATTTAGCTGGGATTTCCTCAATTTTAGGGGCTATTAATTTCATCACTACCATTATTAATATACGAATTAATAATCT
ATCATTTGATCAAATACCTTTATTTATTTGATCAGTGGGCATTACAGCATTACTTTTATTACTTTCTTTACCAGTATTAG
CTGGAGCCATTACTATATTACTAACAGATCGAAATTTAAATACCTCCTTCTTTGATCCAGCGGGAGGGGGAGATCCTATT
CTATATCAACATTTATTTTGATTTTTT
>GBGL14993-14|Diatraea_saccharalis|JX442620|JX442620
-------------------------TGAGCAGGAATATTAGGAACTTCATTAAGTTTATTAATTCGAGCAGAATTAGGAA
CATCTAACTCTTTAATTGGAGATGATCAAATTTATAACACAATTGTAACAGCTCATGCTTTCATTATAATTTTTTTATA
GTTATACCTATTATAATTGGAGGATTTGGAAATTGATTGGTACCTTTAATATTAGGAGCCCCCGATATAGCTTTCCCACG
AATAAATAATATAAGATTTTGATTATTACCCCCATCTTTAACCCTACTAATTTCTAGAAGAATTGTAGAAAATGGAGCAG

GAACAGGATGAACAGTTTATCCCCCATTATCATCTAATATTGCCCATGGGGGAAGATCTGTGGACTTAGCAATTTTCTCT
TTACATTTAGCTGGGATTTCCTCAATTTTAGGGGCTATCAATTTCATCACTACCATTATTAATATACGAATTAATAATCT
ATCATTTGATCAAATACCTTTATTTATTTGATCAGTGGGTATTACAGCATTACTTTTATTACTTTCTTTACCAGTATTAG
CTGGAGCCATTACTATATTACTAACAGATCGAAATTTAAATACCTCCTTCTTTGATCCAGCAGGAGGGGGAGATCCTATT
CTATATCAACATTTATTTTGATTTTTT
>GBGL14994-14|Diatraea_saccharalis|JX442621|JX442621
-------------------------TGAGCAGGAATATTAGGAACTTCATTAAGTTTATTAATTCGAGCAGAATTAGGAA
CATCTAACTCTTTAATTGGAGATGATCAAATTTATAACACAATTGTAACAGCTCATGCTTTCATTATAATTTTTTTTATA
GTTATACCTATTATAATTGGAGGATTTGGAAATTGATTGGTACCTTTAATATTAGGAGCCCCCGATATAGCTTTCCCACG
AATAAATAATATAAGATTTTGATTATTACCCCCATCTTTAACCCTACTAATTTCTAGAAGAATTGTAGAAAATGGAGCAG
GAACAGGATGAACAGTTTATCCCCCATTATCATCTAATATTGCCCATGGGGGAAGATCTGTGGACTTAGCAATTTTCTCT
TTACATTTAGCTGGGATTTCCTCAATTTTAGGGGCTATTAATTTCATCACTACCATTATTAATATACGAATTAATAATCT
ATCATTTGATCAAATACCTTTATTTATTTGATCAGTGGGCATTACAGCATTACTTTTATTACTTTCTTTACCAGTATTAG
CTGGAGCCATTACTATATTACTAACAGATCGAAATTTAAATACCTCCTTCTTTGATCCAGCGGGAGGGGGAGATCCTATT
CTATATCAACATTTATTTTGATTTTTT
>GBGL14995-14|Diatraea_saccharalis|JX442622|JX442622
-------------------------TGAGCAGGAATATTAGGAACTTCATTAAGTTTATTAATTCGAGCAGAATTAGGAA
CATCTAACTCTTTAATTGGAGATGATCAAATTTATAACACAATTGTAACAGCTCATGCTTTCATTATAATTTTTTTTATA
GTTATACCTATTATAATTGGAGGATTTGGAAATTGATTGGTACCTTTAATATTAGGAGCCCCCGATATAGCTTTCCCACG
AATAAATAATATAAGATTTTGATTATTACCCCCATCTTTAACCCTACTAATTTCTAGAAGAATTGTAGAAAATGGAGCAG
GAACAGGATGAACAGTTTATCCCCCATTATCATCTAATATTGCCCATGGGGGAAGATCTGTGGACTTAGCAATTTTCTCT
TTACATTTAGCTGGGATTTCCTCAATTTTAGGGGCTATTAATTTCATCACTACCATTATTAATATACGAATTAATAATCT
ATCATTTGATCAAATACCTTTATTTATTTGATCAGTGGGCATTACAGCATTACTTTTATTACTTTCTTTACCAGTATTAG
CTGGAGCCATTACTATATTACTAACAGATCGAAATTTAAATACCTCCTTCTTTGATCCAGCGGGAGGGGGAGATCCTATT
CTATATCAACATTTATTTTGATTTTTT
>GBGL14996-14|Diatraea_saccharalis|JX442623|JX442623
-------------------------TGAGCAGGAATATTAGGAACTTCATTAAGTTTATTAATTCGAGCAGAATTAGGAA
CATCTAACTCTTTAATTGGAGATGATCAAATTTATAACACAATTGTAACAGCTCATGCTTTCATTATAATTTTTTTTATA
GTTATACCTATTATAATTGGAGGATTTGGAAATTGATTGGTACCTTTAATATTAGGAGCCCCCGATATAGCTTTCCCACG
AATAAATAATATAAGATTTTGATTATTACCCCCATCTTTAACCCTACTAATTTCTAGAAGAATTGTAGAAAATGGAGCAG
GAACAGGATGAACAGTTTATCCCCCATTATCATCTAATATTGCCCATGGGGGAAGATCTGTGGACTTAGCAATTTTCTCT
TTACATTTAGCTGGGATTTCCTCAATTTTAGGGGCTATTAATTTCATCACTACCATTATTAATATACGAATTAATAATCT
ATCATTTGATCAAATACCTTTATTTATTTGATCAGTGGGCATTACAGCATTACTTTTATTACTTTCTTTACCAGTATTAG
CTGGAGCCATTACTATATTACTAACAGATCGAAATTTAAATACCTCCTTCTTTGATCCAGCGGGAGGGGGAGATCCTATT
CTATATCAACATTTATTTTGATTTTTT
>GBGL14997-14|Diatraea_saccharalis|JX442624|JX442624
-------------------------TGAGCAGGAATATTAGGAACTTCATTAAGTTTATTAATTCGAGCAGAATTAGGAA
CATCTAACTCTTTAATTGGAGATGATCAAATTTATAACACAATTGTAACAGCTCATGCTTTCATTATAATTTTTTTTATA
GTTATACCTATTATAATTGGAGGATTTGGAAATTGATTGGTACCTTTAATATTAGGAGCCCCCGATATAGCTTTCCCACG
AATAAATAATATAAGATTTTGATTATTACCCCCATCTTTAACCCTACTAATTTCTAGAAGAATTGTAGAAAATGGAGCAG
GAACAGGGTGAACAGTTTATCCCCCATTATCATCTAATATTGCCCATGGGGGAAGATCTGTGGACTTAGCAATTTTCTCT
TTACATTTAGCTGGGATTTCCTCAATTTTAGGGGCTATTAATTTCATCACTACCATTATTAATATACGAATTAATAATCT
ATCATTTGATCAAATACCTTTATTTATTTGATCAGTGGGCATTACAGCATTACTTTTATTACTTTCTTTACCAGTATTAG
CTGGAGCCATTACTATATTACTAACAGATCGAAATTTAAATACCTCCTTCTTTGATCCAGCGGGAGGGGGAGATCCTATT
CTATATCAACATTTATTTTGATTTTTT
>GBGL14998-14|Diatraea_saccharalis|JX442625|JX442625
-------------------------TGAGCAGGAATATTAGGAACTTCATTAAGTTTATTAATTCGAGCAGAATTAGGAA
CATCTAACTCTTTAATTGGAGATGATCAAATTTATAACACAATTGTAACAGCTCATGCTTTCATTATAATTTTTTTTATA
GTTATACCTATTATAATTGGAGGATTTGGAAATTGATTGGTACCTTTAATATTAGGAGCCCCCGATATAGCTTTCCCACG
AATAAATAATATAAGATTTTGATTATTACCCCCATCTTTAACCCTACTAATTTCTAGAAGAATTGTAGAAAATGGAGCAG
GAACAGGATGAACAGTTTATCCCCCATTATCATCTAATATTGCCCATGGGGGAAGATCTGTGGACTTAGCAATTTTCTCT
TTACATTTAGCTGGGATTTCCTCAATTTTAGGGGCTATTAATTTCATCACTACCATTATTAATATACGAATTAATAATCT
ATCATTTGATCAAATACCTTTATTTATTTGATCAGTGGGCATTACAGCATTACTTTTATTACTTTCTTTACCAGTATTAG
CTGGAGCCATTACTATATTACTAACAGATCGAAATTTAAATACCTCCTTCTTTGATCCAGCGGGAGGGGGAGATCCTATT
CTATATCAACATTTATTTTGATTTTTT
>GBGL14999-14|Diatraea_saccharalis|JX442626|JX442626
-------------------------TGAGCAGGAATATTAGGAACTTCATTAAGTTTATTAATTCGAGCAGAATTAGGAA

CATCTAACTCTTTAATTGGAGATGATCAAATTTATAACACAATTGTAACAGCTCATGCTTTCATTATAATTTTTTTATA
GTTATACCTATTATAATTGGAGGATTTGGAAATTGATTGGTACCTTTAATATTAGGAGCCCCCGATATAGCTTTCCCACG
AATAAATAATATAAGATTTTGATTATTACCCCCATCTTTAACCCTACTAATTTCTAGAAGAATTGTAGAAAATGGAGCAG
GAACAGGATGAACAGTTTATCCCCCATTATCATCTAATATTGCCCATGGGGGAAGATCTGTGGACTTAGCAATTTTCTCT
TTACATTTAGCTGGGATTTCCTCAATTTTAGGGGCTATTAATTTCATCACTACCATTATTAATATACGAATTAATAATCT
ATCATTTGATCAAATACCTTTATTTATTTGATCAGTGGGCATTACAGCATTACTTTTATTACTTTCTTTACCAGTATTAG
CTGGAGCCATTACTATATTACTAACAGATCGAAATTTAAATACCTCCTTCTTTGATCCAGCGGGAGGGGGAGATCCTATT
CTATATCAACATTTATTTTGATTTTTT
>GBGL15000-14|Diatraea_saccharalis|JX442627|JX442627
-------------------------TGAGCAGGAATATTAGGAACTTCATTAAGTTTATTAATTCGAGCAGAATTAGGAA
CATCTAACTCTTTAATTGGAGATGATCAAATTTATAACACAATTGTAACAGCTCATGCTTTCATTATAATTTTTTTATA
GTTATACCTATTATAATTGGAGGATTTGGAAATTGATTGGTACCTTTAATATTAGGAGCCCCCGATATAGCTTTCCCACG
AATAAATAATATAAGATTTTGATTATTACCCCCATCTTTAACCCTACTAATTTCTAGAAGAATTGTAGAAAATGGAGCAG
GAACAGGATGAACAGTTTATCCCCCATTATCATCTAATATTGCCCATGGGGGAAGATCTGTGGACTTAGCAATTTTCTCT
TTACATTTAGCTGGGATTTCCTCAATTTTAGGGGCTATTAATTTCATCACTACCATTATTAATATACGAATTAATAATCT
ATCATTTGATCAAATACCTTTATTTATTTGATCAGTGGGCATTACAGCATTACTTTTATTACTTTCTTTACCAGTATTAG
CTGGAGCCATTACTATATTACTAACAGATCGAAATTTAAATACCTCCTTCTTTGATCCAGCGGGAGGGGGAGATCCTATT
CTATATCAACATTTATTTTGATTTTTT
>GBGL15001-14|Diatraea_saccharalis|JX442628|JX442628
-------------------------TGAGCAGGAATATTAGGAACTTCATTAAGTTTATTAATTCGAGCAGAATTAGGAA
CATCTAACTCTTTAATTGGAGATGATCAAATTTATAACACAATTGTAACAGCTCATGCTTTCATTATAATTTTTTTATA
GTTATACCTATTATAATTGGAGGATTTGGAAATTGATTGGTACCTTTAATATTAGGAGCCCCCGATATAGCTTTCCCACG
AATAAATAATATAAGATTTTGATTATTACCCCCATCTTTAACCCTACTAATTTCTAGAAGAATTGTAGAAAATGGAGCAG
GAACAGGATGAACAGTTTATCCCCCATTATCATCTAATATTGCCCATGGGGGAAGATCTGTGGACTTAGCAATTTTCTCT
TTACATTTAGCTGGGATTTCCTCAATTTTAGGGGCTATTAATTTCATCACTACCATTATTAATATACGAATTAATAATCT
ATTATTTGATCAAATACCTTTATTTATTTGATCAGTGGGCATTACAGCATTACTTTTATTACTTTCTTTACCAGTATTAG
CTGGAGCCATTACTATATTACTAACAGATCGAAATTTAAATACCTCCTTCTTTGATCCAGCGGGAGGGGGAGATCCTATT
CTATATCAACATTTATTTTGATTTTTT
>GBGL15002-14|Diatraea_saccharalis|JX442629|JX442629
-------------------------TGAGCAGGAATATTAGGAACTTCATTAAGTTTATTAATTCGAGCAGAATTAGGAA
CATCTAACTCTTTAATTGGAGATGATCAAATTTATAACACAATTGTAACAGCTCATGCTTTCATTATAATTTTTTTATA
GTTATACCTATTATAATTGGAGGATTTGGAAATTGATTGGTACCTTTAATATTAGGAGCCCCCGATATAGCTTTCCCACG
AATAAATAATATAAGATTTTGATTATTACCCCCATCTTTAACCCTACTAATTTCTAGAAGAATTGTAGAAAATGGAGCAG
GAACAGGATGAACAGTTTATCCCCCATTATCATCTAATATTGCCCATGGGGGAAGATCTGTGGACTTAGCAATTTTCTCT
TTACATTTAGCTGGGATTTCCTCAATTTTAGGGGCTATTAATTTCATCACTACCATTATTAATATACGAATTAATAATCT
ATCATTTGATCAAATACCTTTATTTATTTGATCAGTGGGCATTACAGCATTACTTTTATTACTTTCTTTACCAGTATTAG
CTGGAGCCATTACTATATTACTAACAGATCGAAATTTAAATACCTCCTTCTTTGATCCAGCGGGAGGGGGAGATCCTATT
CTATATCAACATTTATTTTGATTTTTT
>GBGL15003-14|Diatraea_saccharalis|JX442630|JX442630
-------------------------TGAGCAGGAATATTAGGAACTTCATTAAGTTTATTAATTCGAGCAGAATTAGGAA
CATCTAACTCTTTAATTGGAGATGATCAAATTTATAACACAATTGTAACAGCTCATGCTTTCATTATAATTTTTTTATA
GTTATACCTATTATAATTGGAGGATTTGGAAATTGATTGGTACCTTTAATATTAGGAGCCCCCGATATAGCTTTCCCACG
AATAAATAATATAAGATTTTGATTATTACCCCCATCTTTAACCCTACTAATTTCTAGAAGAATTGTAGAAAATGGAGCAG
GAACAGGATGAACAGTTTATCCCCCATTATCATCTAATATTGCCCATGGGGGAAGATCTGTGGACTTAGCAATTTTCTCT
TTACATTTAGCTGGGATTTCCTCAATTTTAGGGGCTATTAATTTCATCACTACCATTATTAATATACGAATTAATAATCT
ATCATTTGATCAAATACCTTTATTTATTTGATCAGTGGGCATTACAGCATTACTTTTATTACTTTCTTTACCAGTATTAG
CTGGAGCCATTACTATATTACTAACAGATCGAAATTTAAATACCTCCTTCTTTGATCCAGCGGGAGGGGGAGATCCTATT
CTATATCAACATTTATTTTGATTTTTT
>GBGL15004-14|Diatraea_saccharalis|JX442631|JX442631
-------------------------TGAGCAGGAATATTAGGAACTTCATTAAGTTTATTAATTCGAGCAGAATTAGGAA
CATCTAACTCTTTAATTGGAGATGATCAAATTTATAACACAATTGTAACAGCTCATGCTTTCATTATAATTTTTTTATA
GTTATACCTATTATAATTGGAGGATTTGGAAATTGATTGGTACCTTTAATATTAGGAGCCCCCGATATAGCTTTCCCACG
AATAAATAATATAAGATTTTGATTATTACCCCCATCTTTAACCCTACTAATTTCTAGAAGAATTGTAGAAAATGGAGCAG
GAACAGGATGAACAGTTTATCCCCCATTATCATCTAATATTGCCCATGGGGGAAGATCTGTGGACTTAGCAATTTTCTCT
TTACATTTAGCTGGGATTTCCTCAATTTTAGGGGCTATTAATTTCATCACTACCATTATTAATATACGAATTAATAATCT
ATCATTTGATCAAATACCTTTATTTATTTGATCAGTGGGCATTACAGCATTACTTTTATTACTTTCTTTACCAGTATTAG
CTGGAGCCATTACTATATTACTAACAGATCGAAATTTAAATACCTCCTTCTTTGATCCAGCGGGAGGGGGAGATCCTATT

CTATATCAACATTTATTTTGATTTTTT
>GBGL15005-14|Diatraea_saccharalis|JX442632|JX442632
-------------------------TGAGCAGGAATATTAGGAACTTCATTAAGTTTATTAATTCGAGCAGAATTAGGAA
CATCTAACTCTTTAATTGGAGATGATCAAATTTATAACACAATTGTAACAGCTCATGCTTTCATTATAATTTTTTTATA
GTTATACCTATTATAATTGGAGGATTTGGAAATTGATTGGTACCTTTAATATTAGGAGCCCCCGATATAGCTTTCCCACG
AATAAATAATATAAGATTTTGATTATTACCCCCATCTTTAACCCTACTAATTTCTAGAAGAATTGTAGAAAATGGAGCAG
GAACAGGATGAACAGTTTATCCCCCATTATCATCTAATATTGCCCATGGGGGAAGATCTGTGGACTTAGCAATTTTCTCT
TTACATTTAGCTGGGATTTCCTCAATTTTAGGGGCTATTAATTTCATCACTACCATTATTAATATACGAATTAATAATCT
ATCATTTGATCAAATACCTTTATTTATTTGATCAGTGGGCATTACAGCATTACTTTTATTACTTTCTTTACCAGTATTAG
CTGGAGCCATTACTATATTACTAACAGATCGAAATTTAAATACCTCCTTCTTTGATCCAGCGGGAGGGGGAGATCCTATT
CTATATCAACATTTATTTTGATTTTTT
>GBGL15006-14|Diatraea_saccharalis|JX442633|JX442633
-------------------------TGAGCAGGAATATTAGGAACTTCATTAAGTTTATTAATTCGAGCAGAATTAGGAA
CATCTAACTCTTTAATTGGAGATGATCAAATTTATAACACAATTGTAACAGCTCATGCTTTCATTATAATTTTTTTATA
GTTATACCTATTATAATTGGAGGATTTGGAAATTGATTGGTACCTTTAATATTAGGAGCCCCCGATATAGCTTTCCCACG
AATAAATAATATAAGATTTTGATTATTACCCCCATCTTTAACCCTACTAATTTCTAGAAGAATTGTAGAAAATGGAGCAG
GAACAGGATGAACAGTTTATCCCCCATTATCATCTAATATTGCCCATGGGGGAAGATCTGTGGACTTAGCAATTTTCTCT
TTACATTTAGCTGGGATTTCCTCAATTTTAGGGGCTATTAATTTCATCACTACCATTATTAATATACGAATTAATAATCT
ATCATTTGATCAAATACCTTTATTTATTTGATCAGTGGGCATTACAGCATTACTTTTATTACTTTCTTTACCAGTATTAG
CTGGAGCCATTACTATATTACTAACAGATCGAAATTTAAATACCTCCTTCTTTGATCCAGCGGGAGGGGGAGATCCTATT
CTATATCAACATTTATTTTGATTTTTT
>GBGL15007-14|Diatraea_saccharalis|JX442634|JX442634
-------------------------TGAGCAGGAATATTAGGAACTTCATTAAGTTTATTAATTCGAGCAGAATTAGGAA
CATCTAACTCTTTAATTGGAGATGATCAAATTTATAACACAATTGTAACAGCTCATGCTTTCATTATAATTTTTTTATA
GTTATACCTATTATAATTGGAGGATTTGGAAATTGATTGGTACCTTTAATATTAGGAGCCCCCGATATAGCTTTCCCACG
AATAAATAATATAAGATTTTGATTATTACCCCCATCTTTAACCCTACTAATTTCTAGAAGAATTGTAGAAAATGGAGCAG
GAACAGGATGAACAGTTTATCCCCCATTATCATCTAATATTGCCCATGGGGGAAGATCTGTGGACTTAGCAATTTTCTCT
TTACATTTAGCTGGGATTTCCTCAATTTTAGGGGCTATTAATTTCATCACTACCATTATTAATATACGAATTAATAATCT
ATCATTTGATCAAATACCTTTATTTATTTGATCAGTGGGCATTACAGCATTACTTTTATTACTTTCTTTACCAGTATTAG
CTGGAGCCATTACTATATTACTAACAGATCGAAATTTAAATACCTCCTTCTTTGATCCAGCGGGAGGGGGAGATCCTATT
CTATATCAACATTTATTTTGATTTTTT
>GBGL15008-14|Diatraea_saccharalis|JX442635|JX442635
-------------------------TGAGCAGGAATATTAGGAACTTCATTAAGTTTATTAATTCGAGCAGAATTAGGAA
CATCTAACTCTTTAATTGGAGATGATCAAATTTATAACACAATTGTAACAGCTCATGCTTTCATTATAATTTTTTTATA
GTTATACCTATTATAATTGGAGGATTTGGAAATTGATTGGTACCTTTAATATTAGGAGCCCCCGATATAGCTTTCCCACG
AATAAATAATATAAGATTTTGATTATTACCCCCATCTTTAACCCTACTAATTTCTAGAAGAATTGTAGAAAATGGAGCAG
GAACAGGATGAACAGTTTATCCCCCATTATCATCTAATATTGCCCATGGGGGAAGATCTGTGGACTTAGCAATTTTCTCT
TTACATTTAGCTGGGATTTCCTCAATTTTAGGGGCTATTAATTTCATCACTACCATTATTAATATACGAATTAATAATCT
ATCATTTGATCAAATACCTTTATTTATTTGATCAGTGGGCATTACAGCATTACTTTTATTACTTTCTTTACCAGTATTAG
CTGGAGCCATTACTATATTACTAACAGATCGAAATTTAAATACCTCCTTCTTTGATCCAGCGGGAGGGGGAGATCCTATT
CTATATCAACATTTATTTTGATTTTTT
>GBGL15009-14|Diatraea_saccharalis|JX442636|JX442636
-------------------------TGAGCAGGAATATTAGGAACTTCATTAAGTTTATTAATTCGAGCAGAATTAGGAA
CATCTAACTCTTTAATTGGAGATGATCAAATTTATAACACAATTGTAACAGCTCATGCTTTCATTATAATTTTTTTATA
GTTATACCTATTATAATTGGAGGATTTGGAAATTGATTGGTACCTTTAATATTAGGAGCCCCCGATATAGCTTTCCCACG
AATAAATAATATAAGATTTTGATTATTACCCCCATCTTTAACCCTACTAATTTCTAGAAGAATTGTAGAAAATGGAGCAG
GAACAGGATGAACAGTTTATCCCCCATTATCATCTAATATTGCCCATGGGGGAAGATCTGTGGACTTAGCAATTTTCTCT
TTACATTTAGCTGGGATTTCCTCAATTTTAGGGGCTATTAATTTCATCACTACCATTATTAATATACGAATTAATAATCT
ATCATTTGATCAAATACCTTTATTTATTTGATCAGTGGGCATTACAGCATTACTTTTATTACTTTCTTTACCAGTATTAG
CTGGAGCCATTACTATATTACTAACAGATCGAAATTTAAATACCTCCTTCTTTGATCCAGCGGGAGGGGGAGATCCTATT
CTATATCAACATTTATTTTGATTTTTT
>GBGL15010-14|Diatraea_saccharalis|JX442637|JX442637
-------------------------TGAGCAGGAATATTAGGAACTTCATTAAGTTTATTAATTCGAGCAGAATTAGGAA
CATCTAACTCTTTAATTGGAGATGATCAAATTTATAACACAATTGTAACAGCTCATGCTTTCATTATAATTTTTTTATA
GTTATACCTATTATAATTGGAGGATTTGGAAATTGATTGGTACCTTTAATATTAGGAGCCCCCGATATAGCTTTCCCACG
AATAAATAATATAAGATTTTGATTATTACCCCCATCTTTAACCCTACTAATTTCTAGAAGAATTGTAGAAAATGGAGCAG
GAACAGGATGAACAGTTTATCCCCCATTATCATCTAATATTGCCCATGGGGGAAGATCTGTGGACTTAGCAATTTTCTCT

TTACATTTAGCTGGGATTTCCTCAATTTTAGGGGCTATTAATTTCATCACTACCATTATTAATATACGAATTAATAATCT
ATCATTTGATCAAATACCTTTATTTATTTGATCAGTGGGCATTACAGCATTACTTTTATTACTTTCTTTACCAGTATTAG
CTGGAGCCATTACTATATTACTAACAGATCGAAATTTAAATACCTCCTTCTTTGATCCAGCGGGAGGGGGAGATCCTATT
CTATATCAACATTTATTTTGATTTTTT
>GBGL15011-14|Diatraea_saccharalis|JX442638|JX442638
-------------------------TGAGCAGGAATATTAGGAACTTCATTAAGTTTATTAATTCGAGCAGAATTAGGAA
CATCTAACTCTTTAATTGGAGATGATCAAATTTATAACACAATTGTAACAGCTCATGCTTTCATTATAATTTTTTTTATA
GTTATACCTATTATAATTGGAGGATTTGGAAATTGATTGGTACCTTTAATATTAGGAGCCCCCGATATAGCTTTCCCACG
AATAAATAATATAAGATTTTGATTATTACCCCCATCTTTAACCCTACTAATTTCTAGAAGAATTGTAGAAAATGGAGCAG
GAACAGGATGAACAGTTTATCCCCCATTATCATCTAATATTGCCCATGGGGGAAGATCTGTGGACTTAGCAATTTTCTCT
TTACATTTAGCTGGGATTTCCTCAATTTTAGGGGCTATTAATTTCATCACTACCATTATTAATATACGAATTAATAATCT
ATCATTTGATCAAATACCTTTATTTATTTGATCAGTGGGCATTACAGCATTACTTTTATTACTTTCTTTACCAGTATTAG
CTGGAGCCATTACTATATTACTAACAGATCGAAATTTAAATACCTCCTTCTTTGATCCAGCGGGAGGGGGAGATCCTATT
CTATATCAACATTTATTTTGATTTTTT
>GBGL15012-14|Diatraea_saccharalis|JX442639|JX442639
-------------------------TGAGCAGGAATATTAGGAACTTCATTAAGTTTATTAATTCGAGCAGAATTAGGAA
CATCTAACTCTTTAATTGGAGATGATCAAATTTATAACACAATTGTAACAGCTCATGCTTTCATTATAATTTTTTTTATA
GTTATACCTATTATAATTGGAGGATTTGGAAATTGATTGGTACCTTTAATATTAGGAGCCCCCGATATAGCTTTCCCACG
AATAAATAATATAAGATTTTGATTATTACCCCCATCTTTAACCCTACTAATTTCTAGAAGAATTGTAGAAAATGGAGCAG
GAACAGGATGAACAGTTTATCCCCCATTATCATCTAATATTGCCCATGGGGGAAGATCTGTGGACTTAGCAATTTTCTCT
TTACATTTAGCTGGGATTTCCTCAATTTTAGGGGCTATTAATTTCATCACTACCATTATTAATATACGAATTAATAATCT
ATCATTTGATCAAATACCTTTATTTATTTGATCAGTGGGCATTACAGCATTACTTTTATTACTTTCTTTACCAGTATTAG
CTGGAGCCATTACTATATTACTAACAGATCGAAATTTAAATACCTCCTTCTTTGATCCAGCGGGAGGGGGAGATCCTATT
CTATATCAACATTTATTTTGATTTTTT
>GBGL15013-14|Diatraea_saccharalis|JX442640|JX442640
-------------------------TGAGCAGGAATATTAGGAACTTCATTAAGTTTATTAATTCGAGCAGAATTAGGAA
CATCTAACTCTTTAATTGGAGATGATCAAATTTATAACACAATTGTAACAGCTCATGCTTTCATTATAATTTTTTTTATA
GTTATACCTATTATAATTGGAGGATTTGGAAATTGATTGGTACCTTTAATATTAGGAGCCCCCGATATAGCTTTCCCACG
AATAAATAATATAAGATTTTGATTATTACCCCCATCTTTAACCCTACTAATTTCTAGAAGAATTGTAGAAAATGGAGCAG
GAACAGGATGAACAGTTTATCCCCCATTATCATCTAATATTGCCCATGGAGGAAGATCTGTGGACTTAGCAATTTTCTCT
TTACATTTAGCTGGGATTTCCTCAATTTTAGGGGCTATCAATTTCATCACTACCATTATTAATATACGAATTAATAATCT
ATCATTTGATCAAATACCTTTATTTATTTGATCAGTGGGTATTACAGCATTACTTTTATTACTTTCTTTACCAGTATTAG
CTGGAGCCATTACTATATTACTAACAGATCGAAATTTAAATACCTCCTTCTTTGATCCAGCGGGAGGGGGAGATCCTATT
CTATATCAACATTTATTTTGATTTTTT
>GBGL15014-14|Diatraea_saccharalis|JX442641|JX442641
-------------------------TGAGCAGGAATATTAGGAACTTCATTAAGTTTATTAATTCGAGCAGAATTAGGAA
CATCTAACTCTTTAATTGGAGATGATCAAATTTATAACACAATTGTAACAGCTCATGCTTTCATTATAATTTTTTTTATA
GTTATACCTATTATAATTGGAGGATTTGGAAATTGATTAGTACCTTTAATATTAGGAGCCCCCGATATAGCTTTCCCACG
AATAAATAATATAAGATTTTGATTATTACCCCCATCTTTAACCCTACTAATTTCTAGAAGAATTGTAGAAAATGGAGCAG
GAACAGGATGAACAGTTTATCCCCCATTATCATCTAATATTGCCCATGGGGGAAGATCTGTGGACTTAGCAATTTTCTCT
TTACATTTAGCTGGGATTTCCTCAATTTTAGGGGCTATCAATTTCATCACTACCATTATTAATATACGAATTAATAATCT
ATCATTTGATCAAATACCTTTATTTATTTGATCAGTGGGTATTACAGCATTACTTTTATTACTTTCTTTACCAGTATTAG
CTGGAGCCATTACTATATTACTAACAGATCGAAATTTAAATACCTCCTTCTTTGATCCAGCGGGAGGGGGAGATCCTATT
CTATATCAACATTTATTTTGATTTTTT
>GBGL15015-14|Diatraea_saccharalis|JX442642|JX442642
-------------------------TGAGCAGGAATATTAGGAACTTCATTAAGTTTATTAATTCGAGCAGAATTAGGAA
CATCTAACTCTTTAATTGGAGATGATCAAATTTATAACACAATTGTAACAGCTCATGCTTTCATTATAATTTTTTTTATA
GTTATACCTATTATAATTGGAGGATTTGGAAATTGATTGGTACCTTTAATATTAGGAGCCCCCGATATAGCTTTCCCACG
AATAAATAATATAAGATTTTGATTATTACCCCCATCTTTAACCCTACTAATTTCTAGAAGAATTGTAGAAAACGGAGCAG
GAACAGGATGAACAGTTTATCCCCCATTATCATCTAATATTGCCCATGGAGGAAGATCTGTGGACTTAGCAATTTTCTCT
TTACATTTAGCTGGGATTTCCTCAATTTTAGGGGCTATCAATTTCATCACTACCATTATTAATATACGAATTAATAATCT
ATCATTTGATCAAATACCTTTATTTATTTGATCAGTGGGTATTACAGCATTACTTTTATTACTTTCTTTACCAGTATTAG
CTGGAGCCATTACTATATTACTAACAGATCGAAATTTAAATACCTCCTTCTTTGATCCAGCGGGAGGGGGAGATCCTATT
CTATATCAACATTTATTTTGATTTTTT
>GBGL15016-14|Diatraea_saccharalis|JX442643|JX442643
-------------------------TGAGCAGGAATATTAGGAACTTCATTAAGTTTATTAATTCGAGCAGAATTAGGAA
CATCTAACTCTTTAATTGGAGATGATCAAATTTATAACACAATTGTAACAGCTCATGCTTTCATTATAATTTTTTTTATA

GTTATACCTATTATAATTGGAGGATTTGGAAATTGATTAGTACCTTTAATATTAGGAGCCCCCGATATAGCTTTCCCACG
AATAAATAATATAAGATTTTGATTATTACCCCCATCTTTAACCCTACTAATTTCTAGAAGAATTGTAGAAAATGGAGCAG
GAACAGGATGAACAGTTTATCCCCCATTATCATCTAATATTGCCCATGGGGGAAGATCTGTGGACTTAGCAATTTTCTCT
TTACATTTAGCTGGGATTTCCTCAATTTTAGGGGCTATCAATTTCATCACTACCATTATTAATATACGAATTAATAATCT
ATCATTTGATCAAATACCTTTATTTATTTGATCAGTGGGTATTACAGCATTACTTTTATTACTTTCTTTACCAGTATTAG
CTGGAGCCATTACTATATTACTAACAGATCGAAATTTAAATACCTCCTTCTTTGATCCAGCGGGAGGGGGAGATCCTATT
CTATATCAACATTTATTTTGATTTTTT
>GBGL15017-14|Diatraea_saccharalis|JX442644|JX442644
-------------------------TGAGCAGGAATATTAGGAACTTCATTAAGTTTATTAATTCGAGCAGAATTAGGAA
CATCTAACTCTTTAATTGGAGATGATCAAATTTATAACACAATTGTAACAGCTCATGCTTTCATTATAATTTTTTTTATA
GTTATACCTATTATAATTGGAGGATTTGGAAATTGATTAGTACCTTTAATATTAGGAGCCCCCGATATAGCTTTCCCACG
AATAAATAATATAAGATTTTGATTATTACCCCCATCTTTAACCCTACTAATTTCTAGAAGAATTGTAGAAAATGGAGCAG
GAACAGGATGAACAGTTTATCCCCCATTATCATCTAATATTGCCCATGGGGGAAGATCTGTGGACTTAGCAATTTTCTCT
TTACATTTAGCTGGGATTTCCTCAATTTTAGGGGCTATCAATTTCATCACTACCATTATTAATATACGAATTAATAATCT
ATCATTTGATCAAATACCTTTATTTATTTGATCAGTGGGTATTACAGCATTACTTTTATTACTTTCTTTACCAGTATTAG
CTGGAGCCATTACTATATTACTAACAGATCGAAATTTAAATACCTCCTTCTTTGATCCAGCGGGAGGGGGAGATCCTATT
CTATATCAACATTTATTTTGATTTTTT
>GBGL15018-14|Diatraea_saccharalis|JX442645|JX442645
-------------------------TGAGCAGGAATATTAGGAACTTCATTAAGTTTATTAATTCGAGCAGAATTAGGAA
CATCTAACTCTTTAATTGGAGATGATCAAATTTATAACACAATTGTAACAGCTCATGCTTTCATTATAATTTTTTTTATA
GTTATACCTATTATAATTGGAGGATTTGGAAATTGATTGGTACCTTTAATATTAGGAGCCCCCGATATAGCTTTCCCACG
AATAAATAATATAAGATTTTGATTATTACCCCCATCTTTAACCCTACTAATTTCTAGAAGAATTGTAGAAAATGGAGCAG
GAACAGGATGAACAGTTTATCCCCCATTATCATCTAATATTGCCCATGGAGGAAGATCTGTGGACTTAGCAATTTTCTCT
TTACATTTAGCTGGGATTTCCTCAATTTTAGGGGCTATCAATTTCATCACTACCATTATTAATATACGAATTAATAATCT
ATCATTTGATCAAATACCTTTATTTATTTGATCAGTGGGTATTACAGCATTACTTTTATTACTTTCTTTACCAGTATTAG
CTGGAGCCATTACTATATTACTAACAGATCGAAATTTAAATACCTCCTTCTTTGATCCAGCGGGAGGGGGAGATCCTATT
CTATATCAACATTTATTTTGATTTTTT
>GBGL15019-14|Diatraea_saccharalis|JX442646|JX442646
-------------------------TGAGCAGGAATATTAGGAACTTCATTAAGTTTATTAATTCGAGCAGAATTAGGAA
CATCTAACTCTTTAATTGGAGATGATCAAATTTATAACACAATTGTAACAGCTCATGCTTTCATTATAATTTTTTTTATA
GTTATACCTATTATAATTGGAGGATTTGGAAATTGATTGGTACCTTTAATATTAGGAGCCCCCGATATAGCTTTCCCACG
AATAAATAATATAAGATTTTGATTATTACCCCCATCTTTAACCCTACTAATTTCTAGAAGAATTGTAGAAAATGGAGCAG
GAACAGGATGAACAGTTTATCCCCCATTATCATCTAATATTGCCCATGGAGGAAGATCTGTGGACTTAGCAATTTTCTCT
TTACATTTAGCTGGGATTTCCTCAATTTTAGGGGCTATCAATTTCATCACTACCATTATTAATATACGAATTAATAATCT
ATCATTTGATCAAATACCTTTATTTATTTGATCAGTGGGTATTACAGCATTACTTTTATTACTTTCTTTACCAGTATTAG
CTGGAGCCATTACTATATTACTAACAGATCGAAATTTAAATACCTCCTTCTTTGATCCAGCGGGAGGGGGAGATCCTATT
CTATATCAACATTTATTTTGATTTTTT
>GBGL15020-14|Diatraea_saccharalis|JX442647|JX442647
-------------------------TGAGCAGGAATATTAGGAACTTCATTAAGTTTATTAATTCGAGCAGAATTAGGAA
CATCTAACTCTTTAATTGGAGATGATCAAATTTATAACACAATTGTAACAGCTCATGCTTTCATTATAATTTTTTTTATA
GTTATACCTATTATAATTGGAGGATTTGGAAATTGATTGGTACCTTTAATATTAGGAGCCCCCGATATAGCTTTCCCACG
AATAAATAATATAAGATTTTGATTATTACCCCCATCTTTAACCCTACTAATTTCTAGAAGAATTGTAGAAAATGGAGCAG
GAACAGGATGAACAGTTTATCCCCCATTATCATCTAATATTGCCCATGGAGGAAGATCTGTGGACTTAGCAATTTTCTCT
TTACATTTAGCTGGGATTTCCTCAATTTTAGGGGCTATCAATTTCATCACTACCATTATTAATATACGAATTAATAATCT
ATCATTTGATCAAATACCTTTATTTATTTGATCAGTGGGTATTACAGCATTACTTTTATTACTTTCTTTACCAGTATTAG
CTGGAGCCATTACTATATTACTAACAGATCGAAATTTAAATACCTCCTTCTTTGATCCAGCGGGGGGGGGAGATCCTATT
CTATATCAACATTTATTTTGATTTTTT
>GBGL15021-14|Diatraea_saccharalis|JX442648|JX442648
-------------------------TGAGCAGGAATATTAGGAACTTCATTAAGTTTATTAATTCGAGCAGAATTAGGAA
CATCTAACTCTTTAATTGGAGATGATCAAATTTATAACACAATTGTAACAGCTCATGCTTTCATTATAATTTTTTTTATA
GTTATACCTATTATAATTGGAGGATTTGGAAATTGATTGGTACCTTTAATATTAGGAGCCCCCGATATAGCTTTCCCACG
AATAAATAATATAAGATTTTGATTATTACCCCCATCTTTAACCCTACTAATTTCTAGAAGAATTGTAGAAAATGGAGCAG
GAACAGGATGAACAGTTTATCCCCCATTATCATCTAATATTGCCCATGGAGGAAGATCTGTGGACTTAGCAATTTTCTCT
TTACATTTAGCTGGGATTTCCTCAATTTTAGGGGCTATCAATTTCATCACTACCATTATTAATATACGAATTAATAATCT
ATCATTTGATCAAATACCTTTATTTATTTGATCAGTGGGTATTACAGCATTACTTTTATTACTTTCTTTACCAGTATTAG
CTGGAGCCATTACTATATTACTAACAGATCGAAATTTAAATACCTCCTTCTTTGATCCAGCGGGAGGGGGAGATCCTATT
CTATATCAACATTTATTTTGATTTTTT

>GBGL15128-14|Eoreuma_densella|KJ657612|KJ657612
-------------------------GAGCTGGTATAGTAGGAACATCTCTTAGTTTATTAATTCGAGCTGAATTAGGAA
ATCCTGGTTCCTTAATTGGAGATGATCAAATTTATAATACTATTGTCACAGCACATGCATTTATTATAATTTTTTTTATA
GTTATACCTATTATAATTGGAGGATTTGGAAATTGATTAGTTCCTTTAATATTAGGAGCTCCTGATATAGCATTCCCCCG
AATAAATAATATAAGATTTTGATTATTACCCCCCTCATTAACTTTATTAATTTCAAGTAGTATTGTCGAAAATGGTGCCG
GTACAGGATGAACTGTATACCCCCCTCTTTCCTCAAATATTGCCCATGGGGGAAGATCTGTTGACTTAGCAATTTTTTCT
TTACATTTAGCTGGAATTTCATCAATCTTAGGAGCTATCAACTTTATTACAACAATTATTAATATACGAATTAATAATTT
ATCATTTGATCAAATACCTTTATTTGTTTGATCAGTAGGAATTACAGCATTACTTCTCCTCCTTTCATTGCCTGTTTTAG
CAGGAGCTATTACTATATTATTAACAGATCGAAATCTTAATACATCATTTTTTGACCCTGCTGGAGGAGGAGATCCAATT
CTCTACCAACATTTATTTTGATTTTT-
>GBGL15129-14|Eoreuma_loftini|KJ657588|KJ657588
-------------------------GAGCTGGAACAGTAGGAACATCTTTAAGTTTATTAATTCGAGCTGAATTAGGAA
ACCCAGGCTCTTTAATTGGAGATGATCAAATTTATAATACTATTGTTACAGCCCATGCATTTATTATAATTTTTTTTATA
GTAATACCTATTATAATTGGGGGATTTGGAAACTGATTAGTACCTTTAATATTAGGAGCTCCTGATATAGCATTCCCACG
AATAAATAATATAAGATTTTGATTATTACCCCCATCTTTAACACTACTAATTTCAAGAAGTATTGTTGAAAATGGAGCTG
GAACAGGATGAACAGTTTATCCCCCTCTTTCTTCTAATATTGCTCATGGAGGTAGATCTGTAGATCTAGCAATTTTTTCT
CTTCATTTAGCTGGAATTTCCTCAATTTTAGGAGCTATTAATTTTATTACAACAATTATTAATATACGAATTAATAATTT
ATCCTTTGATCAAATACCTTTATTTGTTTGATCAGTAGGTATTACAGCTTTACTTCTTCTTCTTTCTTTACCAGTATTAG
CTGGAGCTATTACTATATTATTAACAGATCGAAATTTAAATACTTCCTTTTTTGATCCTGCAGGGGGTGGAGATCCAATT
CTTTATCAACATTTATTTTGATTTTT-
>GBGL15130-14|Eoreuma_loftini|KJ657614|KJ657614
-------------------------GAGCTGGAACAGTAGGAACATCTTTAAGTTTATTAATTCGAGCTGAATTAGGAA
ACCCAGGCTCTTTAATTGGAGATGATCAAATTTATAATACTATTGTTACAGCCCATGCATTTATTATAATTTTTTTTATA
GTAATACCTATTATAATTGGGGGATTTGGAAATTGATTAGTACCTTTAATATTAGGAGCTCCTGATATAGCATTCCCACG
AATAAATAATATAAGATTTTGATTATTACCCCCATCTTTAACACTACTAATTTCAAGAAGTATTGTTGAAAATGGAGCTG
GAACAGGATGAACAGTTTATCCCCCTCTTTCTTCTAATATTGCTCATGGAGGTAGATCTGTAGATCTAGCAATTTTTTCT
CTTCATTTAGCTGGAATTTCCTCAATTTTAGGAGCTATTAATTTTATTACAACAATTATTAATATACGAATTAATAATTT
ATCCTTTGATCAAATACCTTTATTTGTTTGATCAGTAGGTATTACAGCTTTACTTCTTCTTCTTTCTTTACCAGTATTAG
CTGGAGCTATTACTATATTATTAACAGATCGAAATTTAAATACTTCCTTTTTTGATCCTGCAGGGGGTGGAGATCCAATT
CTTTATCAACATTTATTTTGATTTTT-
>GBGL15131-14|Eoreuma_loftini|KJ657634|KJ657634
-------------------------GAGCTGGAACAGTAGGAACATCTTTAAGTTTATTAATTCGAGCTGAATTAGGAA
ACCCAGGCTCTTTAATTGGAGATGATCAAATTTATAATACTATTGTTACAGCCCATGCATTTATTATAATTTTTTTTATA
GTAATACCTATTATAATTGGGGGATTTGGAAACTGATTAGTACCTTTAATATTAGGAGCTCCTGATATAGCATTCCCACG
AATAAATAATATAAGATTTTGATTATTACCCCCATCTTTAACACTACTAATTTCAAGAAGTATTGTTGAAAATGGAGCTG
GAACAGGATGAACAGTTTATCCCCCTCTTTCTTCTAATATTGCTCATGGAGGTAGATCTGTAGATCTAGCAATTTTTTCT
CTTCATTTAGCTGGAATTTCCTCAATTTTAGGAGCTATTAATTTTATTACAACAATTATTAATATACGAATTAATAATTT
ATCCTTTGATCAAATACCTTTATTTGTTTGATCAGTAGGTATTACAGCTTTACTTCTTCTTCTTTCTTTACCAGTATTAG
CTGGAGCTATTACTATATTATTAACAGATCGAAATTTAAATACTTCCTTTTTTGATCCTGCAGGGGGTGGAGATCCAATT
CTTTATCAACATTTATTTTGATTTTT-
>GBGL15132-14|Eoreuma_loftini|KJ657686|KJ657686
-------------------------GAGCTGGAACAGTAGGAACATCTTTAAGTTTATTAATTCGAGCTGAATTAGGAA
ACCCAGGCTCTTTAATTGGAGATGATCAAATTTATAATACTATTGTTACAGCCCATGCATTTATTATAATTTTTTTTATA
GTAATACCTATTATAATTGGGGGATTTGGAAACTGATTAGTACCTTTAATATTAGGAGCTCCTGATATAGCATTCCCACG
AATAAATAATATAAGATTTTGATTATTACCCCCATCTTTAACACTACTAATTTCAAGAAGTATTGTTGAAAATGGAGCTG
GAACAGGATGAACAGTTTATCCCCCTCTTTCTTCTAATATTGCTCATGGAGGTAGATCTGTAGATCTAGCAATTTTTTCT
CTTCATTTAGCTGGAATTTCCTCAATTTTAGGAGCTATTAATTTTATTACAACAATTATTAATATACGAATTAATAATTT
ATCCTTTGATCAAATACCTTTATTTGTTTGATCAGTAGGTATTACAGCTTTACTTCTTCTTCTTTCTTTACCAGTATTAG
CTGGAGCTATTACTATATTATTAACAGATCGAAATTTAAATACTTCCTTTTTTGATCCTGCAGGGGGTGGAGATCCAATT
CTTTATCAACATTTATTTTGATTTT--
>GBGL17263-15|Eoreuma_loftini|KM068906|KM068906
AACTCTATATTTTATTTTGGGAATTTGAGCTGGAACAGTAGGAACATCTTTAAGTTTATTAATTCGAGCTGAATTAGGAA
ATCCAGGCTCTTTAATTGGAGATGATCAAATTTATAATACTATTGTTACAGCACATGCATTTATTATAATTTTTTTTATA
GTAATACCTATTATAATTGGAGGATTTGGAAACTGATTAGTACCTTTAATATTAGGGGCTCCTGATATAGCATTCCCGCG
AATAAATAATATAAGATTTTGATTATTACCCCCATCTTTAACACTACTAATTTCAAGAAGTATTGTTGAAAATGGAGCTG
GAACAGGATGAACAGTTTATCCTCCTCTTTCTTCTAATATTGCTCATGGAGGTAGATCTGTAGATCTAGCAATTTTTTCT
CTTCATTTAGCTGGAATTTCTTCAATTTTAGGAGCCATTAATTTTATTACAACAATCATTAATATACGAATTAATAACTT

ATCCTTTGATCAAATACCTTTATTTGTCTGATCAGTAGGTATTACAGCTTTACTTCTTCTTCTTTCTTTACCTGTATTAG
CTGGAGCTATTACTATATTATTAACAGATCGAAATTTAAACACTTCATTTTTTGATCCTGCGGGAGGTGGAGATCCAATT
CTTTATCAACATTTATTTTGATTTT--
>GBGL17264-15|Eoreuma_loftini|KM068905|KM068905
AACTCTATATTTTATTTTGGGAATTTGAGCTGGAACAGTAGGAACATCTTTAAGTTTATTAATTCGAGCTGAATTAGGAA
ATCCAGGCTCTTTAATTGGAGATGATCAAATTTATAATACTATTGTTACAGCACATGCATTTATTATAATTTTTTTTATA
GTAATACCTATTATAATTGGAGGATTTGGAAACTGATTAGTACCTTTAATATTAGGGGCTCCTGATATAGCATTCCCGCG
AATAAATAATATAAGATTTTGATTATTGCCCCCATCTTTAACACTACTAATTTCAAGAAGTATTGTTGAAAATGGAGCTG
GAACAGGATGAACAGTTTATCCTCCTCTTTCTTCTAATATTGCTCATGGAGGTAGATCTGTAGATCTAGCAATTTTTTCT
CTTCATTTAGCTGGAATTTCTTCAATTTTAGGAGCCATTAATTTTATTACAACAATCATTAATATACGAATTAATAACTT
ATCCTTTGATCAAATACCTTTATTTGTCTGATCAGTAGGTATTACAGCTTTACTTCTTCTTCTTTCTTTACCTGTATTAG
CTGGAGCTATTACTATATTATTAACAGATCGAAATTTAAACACTTCATTTTTTGATCCTGCGGGAGGTGGAGATCCAATT
CTTTATCAACATTTATTTTGATTTT--
>GBGL17265-15|Eoreuma_loftini|KM068904|KM068904
AACTCTATATTTTATTTTTGGAATTTGAGCTGGAACAGTAGGAACATCTTTAAGTTTATTAATTCGAGCTGAATTAGGAA
ATCCAGGCTCTTTAATTGGAGATGATCAAATTTATAATACTATTGTTACAGCACATGCATTTATTATAATTTTTTTTATA
GTAATACCTATTATAATTGGAGGATTTGGAAACTGATTAGTACCTTTAATATTAGGGGCTCCTGATATAGCATTCCCGCG
AATAAATAATATAAGATTTTGATTATTGCCCCCATCTTTAACACTACTAATTTCAAGAAGTATTGTTGAAAATGGAGCTG
GAACAGGATGAACAGTTTATCCTCCTCTTTCTTCTAATATTGCTCATGGGGGTAGATCTGTAGATCTAGCAATTTTTTCT
CTTCATTTAGCTGGAATTTCTTCAATTTTAGGAGCCATTAATTTTATTACAACAATCATTAATATACGAATTAATAACTT
ATCCTTTGATCAAATACCTTTATTTGTCTGATCAGTAGGTATTACAGCTTTACTTCTTCTTCTTTCTTTACCTGTATTAG
CTGGAGCTATTACTATATTATTAACAGATCGAAATTTAAACACTTCATTTTTTGATCCTGCGGGAGGTGGAGATCCAATT
CTTTATCAACATTTATTTTGATTTT--
>GBGL17309-15|Diatraea_saccharalis|KM289006|KM289006
AACTTTATATTTTATTTTTGGAATTTGAGCAGGAATGTTAGGAACTTCCTTAAGTTTATTAATTCGAGCAGAATTAGGAA
CATCTAACTCTTTAATTGGAGATGATCAAATTTATAATACAATTGTTACAGCTCATGCTTTCATTATAATTTTTTTTATA
GTTATACCTATTATAATTGGAGGATTTGGAAATTGATTAGTACCTTTAATATTAGGAGCCCCTGATATAGCTTTCCCACG
AATAAATAATATAAGATTTTGACTATTACCCCCATCTTTAACTCTACTAATTTCTAGAAGAATTGTAGAAAATGGAGCAG
GAACAGGATGAACGGTTTATCCCCCATTATCATCTAATATTGCCCATGGGGGAAGATCTGTGGACTTAGCAATTTTCTCT
TTGCATTTAGCTGGAATTTCCTCAATTTTAGGGGCTATCAATTTCATTACTACCATTATTAATATACGAATTAATAATCT
ATCATTTGATCAAATACCTTTATTTGTTTGATCAGTAGGTATTACAGCATTACTTTTATTACTTTCTTTACCAGTATTAG
CTGGAACCATTACTATATTACTAACAGATCGAAATTTAAATACCTCCTTTTTTGATCCAGCAGGAGGGGGAGATCCTATT
CTATATCAACATTTATTTTGATTTTTT
>GBGL17310-15|Diatraea_saccharalis|KM289005|KM289005
AACTTTATATTTTATTTTTGGAATTTGAGCAGGAATGTTAGGAACTTCCTTAAGTTTATTAATTCGAGCAGAATTAGGAA
CATCTAACTCTTTAATTGGAGATGATCAAATTTATAATACAATTGTTACAGCTCATGCTTTCATTATAATTTTTTTTATA
GTTATACCTATTATAATTGGAGGATTTGGAAATTGATTAGTACCTTTAATATTAGGAGCCCCTGATATAGCTTTCCCACG
AATAAATAATATAAGATTTTGACTATTACCCCCATCTTTAACTCTGCTAATTTCTAGAAGAATTGTAGAAAATGGAGCAG
GAACAGGATGAACGGTTTATCCCCCATTATCATCTAATATTGCCCATGGGGGAAGATCTGTGGACTTAGCAATTTTCTCT
TTGCATTTAGCTGGAATTTCCTCAATTTTAGGGGCTATCAATTTCATTACTACCATTATTAATATACGAATTAATAATCT
ATCATTTGATCAAATACCTTTATTTGTTTGATCAGTGGGTATTACAGCATTACTTTTATTACTTTCTTTACCAGTATTAG
CTGGAGCCATTACTATATTACTAACAGATCGAAATTTAAATACCTCCTTTTTTGATCCAGCAGGAGGGGGAGATCCTATT
CTATATCAACATTTATTTTGATT----
>GBGL17311-15|Diatraea_saccharalis|KM289004|KM289004
---TTCCTTAAGCTTATTAATTCGAGCAGAATTAGGAA
CATCTAACTCTTTAATTGGAGATGATCAAATTTATAACACAATTGTAACAGCTCATGCTTTCATTATAATTTTTTTTATA
GTTATACCTATTATAATTGGAGGATTTGGAAATTGATTAGTACCTTTAATGTTAGGGGCCCCCGATATAGCTTTCCCACG
AATAAATAATATAAGATTTTGACTATTACCCCCATCTTTAACTCTACTAATTTCTAGAAGAATTGTAGAAAATGGAGCAG
GGACAGGATGAACAGTTTATCCCCCATTATCATCCAATATTGCCCATGGGGGAAGATCTGTGGATTTAGCAATTTTCTCT
TTACATTTAGCTGGAATTTCCTCAATTTTAGGGGCTATCAATTTCATCACTACCATTATTAATATACGAATTAATAATCT
ATCATTTGATCAAATACCTTTATTTATTTGATCAGTGGGTATTACAGCATTACTTTTATTACTTTCTTTACCGGTATTAG
CTGGAGCCATTACTATATTACTAACAGATC--

>GBGL17312-15|Diatraea_saccharalis|KM289003|KM289003
AACTTTATATTTTATTTTTGGAATTTGAGCAGGAATGTTAGGAACTTCCTTAAGCTTATTAATTCGAGCAGAATTAGGAA
CATCTAACTCTTTAATTGGAGATGATCAAATTTATAACACAATTGTAACAGCTCATGCTTTCATTATAATTTTTTTTATA
GTTATACCTATTATAATTGGAGGATTTGGAAATTGATTAGTACCTTTAATATTAGGGGCCCCCGATATAGCTTTCCCACG

AATAAATAACATAAGATTTTGACTATTACCCCCATCTTTAACTCTACTAATTTCTAGAAGAATTGTAGAAAATGGAACAG
GGACAGGATGAACAGTTTATCCCCCATTATCATCCAATATTGCCCATGGGGGAAGATCTGTGGATTTAGCAATTTTCTCT
TTACATTTAGCTGGAATTTCCTCAATTTTAGGGGCTATCAATTTTATCACTACCATTATTAATATACGAATTAATAATCT
ATCATTTGATCAAATACCTTTATTTATTTGATCAGTGGGTATTACAGCATTACTTTTATTACTTTCTTTACCAGTATTAG
CTGGAGCCATTACTATATTACTAACAGATCGAAATTTAAATACCTCCTTCTTTGATCCAGCGGGGGGGGGAGATCCTATT
CTATATCAACATTTATTTTGATT----
>GBGL17313-15|Diatraea_saccharalis|KM289002|KM289002
AACTTTATATTTTATTTTTGGAATTTGAGCAGGAATATTAGGAACTTCCTTAAGCTTATTAATTCGAGCAGAATTAGGAA
CATCTAACTCTTTAATTGGAGATGATCAAATTTATAATACAATTGTAACAGCTCATGCTTTCATTATAATTTTTTTTATA
GTTATACCTATTATAATTGGAGGATTTGGAAATTGATTAGTACCTTTAATATTAGGAGCCCCCGATATAGCTTTCCCACG
AATAAATAATATAAGATTTTGACTATTACCCCCATCTTTAACTCTACTAATTTCTAGAAGAATTGTAGAAAATGGAGCAG
GGACAGGGTGAACAGTTTATCCCCCATTATCATCCAATATTGCCCATGGGGGAAGATCTGTAGATTTAGCAATTTTCTCC
TTACATTTAGCTGGAATTTCCTCAATTTTAGGGGCTATCAATTTCATCACTACCATTATTAATATACGAATTAATAATCT
ATCATTTGATCAAATACCCTTATTTATTTGATCAGTGGGTATTACAGCATTACTTTTATTACTTTCTTTACCAGTATTAG
CTGGAGCCATTACTATATTACTAACAGATCGAAATTTAAATACCTCCTTCTTTGATCCAGCGGGGGGAGGAGATCCTATT
CTATATCAACATTTATTTTGATTTT--
>GBGL17314-15|Diatraea_saccharalis|KM289001|KM289001
AACTTTATATTTTATTTTTGGAATTTGAGCAGGAATATTAGGAACTTCCTTAAGCTTATTAATTCGAGCAGAATTAGGAA
CATCTAACTCTTTAATTGGAGATGATCAAATTTATAATACAATTGTAACAGCTCATGCTTTCATTATAATTTTTTTTATA
GTTATACCTATTATAATTGGAGGATTTGGAAATTGATTAGTACCTTTAATATTAGGAGCCCCCGATATAGCTTTCCCACG
AATAAATAATATAAGATTTTGACTATTACCCCCATCTTTAACTCTACTAATTTCTAGAAGAATTGTAGAAAATGGAGCAG
GGACAGGGTGAACAGTTTATCCCCCATTATCATCCAATATTGCCCATGGGGGAAGATCTGTAGATTTAGCAATTTTCTCC
TTACATTTAGCTGGAATTTCCTCAATTTTAGGGGCTATCAATTTCATCACTACCATTATTAATATACGAATTAATAATCT
ATCATTTGATCAAATACCCTTATTTATTTGATCAGTGGGTATTACAGCATTACTTTTATTACTTTCTTTACCAGTATTAG
CTGGAGCCATTACTATATTACTAACAGATCGAAATTTAAATACCTCCTTCTTTGATCCAGCGGGGGGAGGAGATCCTATT
CTATATCAACATTTATTTTGATTTTTT
>GBGL17315-15|Diatraea_saccharalis|KM289000|KM289000
---TCGAGCAGAATTAGGAA
CATCTAACTCTTTAATTGGAGATGATCAAATTTATAATACAATTGTAACAGCTCATGCTTTCATTATAATTTTTTTTATA
GTTATACCTATTATAATTGGAGGATTTGGAAATTGATTAGTACCTTTAATATTAGGAGCCCCCGATATAGCTTTCCCACG
AATAAATAATATAAGATTTTGACTATTACCCCCATCTTTAACTCTACTAATTTCTAGAAGAATTGTAGAAAATGGAGCAG
GGACAGGGTGAACAGTTTATCCCCCATTATCATCCAATATTGCCCATGGGGGAAGATCTGTAGATTTAGCAATTTTCTCC
TTACATTTAGCTGGAATTTCCTCAATTTTAGGGGCTATCAATTTCATCACTACCATTATTAATATACGAATTAATAATCT
ATCATTTGATCAAATACCCTTATTTATTTGATCAGTGGGTATTACAGCATTACTTTTATTACTTTCTTTACCAGTATTAG
CTGGAGCCATTACTATATTACTAACAGATCGAAATTTAAATACCTCCTTCTTTGATCCAGCGGGGGGAGGAGATCCTATT
CTATATCAACATTTATTTTGATTTTTT
>GBGL17316-15|Diatraea_saccharalis|KM288999|KM288999
AACTTTATATTTTATTTTTGGAATTTGAGCAGGAATATTAGGAACTTCCTTAAGCTTATTAATTCGAGCAGAATTAGGAA
CATCTAACTCTTTAATTGGAGATGATCAAATTTATAATACAATTGTAACAGCTCATGCTTTCATTATAATTTTTTTTATA
GTTATACCTATTATAATTGGAGGATTTGGAAATTGATTAGTACCTTTAATATTAGGAGCCCCCGATATAGCTTTCCCACG
AATAAATAATATAAGATTTTGACTATTACCCCCATCTTTAACTCTACTAATTTCTAGAAGAATTGTAGAAAATGGAGCAG
GGACAGGGTGAACAGTTTATCCCCCACTATCATCCAATATTGCCCATGGGGGAAGATCTGTAGATTTAGCAATTTTCTCC
TTACATTTAGCTGGAATTTCCTCAATTTTAGGGGCTATCAATTTCATCACTACCATTATTAATATACGAATTAATAATCT
ATCATTTGATCAAATACCCTTATTTATTTGATCAGTGGGTATTACAGCATTACTTTTATTACTTTCTTTACCAGTATTAG
CTGGAGCCATTACTATATTACTAACAGATCGAAATTTAAATACCTCCTTCTTTGATCCAGCGGGGGGAGGAGATCCTATT
CTATATCAACATTTATTTTGATTTTTT
>GBGL17822-15|Sesamia_inferens|KJ500027|KJ500027
----------------------------------ATAGTAGGAACATCATTAAGATTATTAATTCGAGCTGAATTAGGAA
CCCCAGGATCTTTAATTGGAGATGATCAAATTTATAATACTATTGTTACAGCTCATGCTTTTATTATAATTTTTTTTATA
GTTATACCAATTATAATTGGAGGATTTGGAAATTGACTTGTACCTTTAATATTAGGAGCTCCTGATATAGCATTTCCACG
AATAAATAATATAAGATTTTGATTATTACCCCCCTCTTTAACTCTTTTAATTTCAAGTAGAATTGTAGAAAATGGAGCAG
GAACTGGGTGAACAGTGTACCCCCCACTTTCATCTAATATTGCCCATGGAGGAAGATCAGTAGATCTAGCTATTTTTTCC
CTTCATTTAGCTGGTATTTCATCTATTTTAGGAGCTATTAATTTTATTACAACAATTATCAATATACGATTAAATAGTTT
ATCTTTTGATCAAATACCTCTATTTATTTGAGCTGTTGGAATTACTGCATTTTTATTATTATTATCTTTACCTGTATTAG
CAGGAGCTATTACAATATTATTGACAGATCGAAATTTAAATACATCATTCTTTGACCCCGCAGGAGGAGGTGATCCTATT
TTATACCAACATTTATTTTGATTTTT-
>GBGL18407-15|Chilo_partellus|KP233794|KP233794

AACTTTATATTTTATTTTTGGAATTTGAGCAGGAATAATTGGAACATCCCTTAGATTATTAATTCGTGCAGAATTAGGAA
CTCCTGGATCTTTAATTGGAGATGATCAAATTTATAATACTATTGTAACAGCACATGCATTTATTATAATTTTTTTTATA
GTTATACCAATTATAATTGGTGGATTTGGAAATTGATTAGTACCTTTAATATTAGGAGCCCCAGATATAGCTTTCCCACG
AATAAATAATATAAGATTTTGATTATTACCACCATCATTAACTTTACTAATTTCTAGAAGAATTGTTGAAAATGGAGCTG
GAACAGGATGAACAGTGTACCCCCCACTATCATCTAATATTGCTCATGCTGGAAGTTCAGTAGATTTAGCAATTTTTTCT
TTACATTTAGCTGGTATTTCATCAATTCTTGGTGCTATTAATTTTATTACAACAATCATTAATATACGAATTAATGGATT
ATTTTTTGATCAAATACCATTATTTGTTTGATCTGTAGGTATTACAGCTTTATTATTATTACTTCTTTACCTGTTTTAG
CTGGAGCTATTACTATATTATTAACAGATCGAAATTTAAATACATCCTTTTTCGATCCTGCTGGAGGAGGAGATCCTATT
CTTTATCAACACTTA------------
>GBGL18408-15|Chilo_partellus|KP233795|KP233795
AACTTTATATTTTATTTTTGGAATTTGAGCAGGAATAATTGGAACATCCCTTAGATTATTAATTCGTGCAGAATTAGGAA
CTCCTGGATCTTTAATTGGAGATGATCAAATTTATAATACTATTGTAACAGCACATGCATTTATTATAATTTTTTTTATA
GTTATACCAATTATAATTGGTGGATTTGGAAATTGATTAGTACCTTTAATATTGGGAGCCCCAGATATAGCTTTCCCACG
AATAAATAATATAAGATTTTGATTATTACCACCATCATTAACTTTACTAATTTCTAGAAGAATTGTTGAAAATGGAGCTG
GAACAGGATGAACAGTGTACCCCCCACTATCATCTAATATTGCTCATGCTGGAAGTTCAGTAGATTTAGCAATTTTTTCT
TTACATTTAGCTGGTATTTCATCAATTCTTGGTGCTATTAATTTTATTACAACAATCATTAATATACGAATTAATGGATT
ATTTTTTGATCAAATACCATTATTTGTTTGATCTGTAGGTATTACAGCTTTATTATTATTACTTCTTTACCTGTTTTAG
CTGGGGCTATTACTATATTATTAACAGATCGAAATTTAAATACATCCTTTTTCGATCCTGCTGGAGGAGGAGATCCTATT
CTTTATCAACACTTA------------
>GBGL18409-15|Chilo_partellus|KP233796|KP233796
AACTTTATATTTTATTTTTGGAATTTGAGCAGGAATAATTGGAACATCCCTTAGATTATTAATTCGTGCAGAATTAGGAA
CTCCTGGATCTTTAATTGGAGATGATCAAATTTATAATACTATTGTAACAGCACATGCATTTATTATAATTTTTTTTATA
GTTATACCAATTATAATTGGTGGATTTGGAAATTGATTAGTACCTTTAATATTAGGAGCCCCAGATATAGCTTTCCCACG
AATAAATAATATAAGATTTTGATTATTACCACCATCATTAACTTTACTAATTTCTAGAAGAATTGTTGAAAATGGAGCTG
GAACAGGATGAACAGTGTACCCCCCACTATCATCTAATATTGCTCATGCTGGAAGTTCAGTAGATTTAGCAATTTTTTCT
TTACATTTAGCTGGTATTTCATCAATTCTTGGTGCTATTAATTTTATTACAACAATCATTAATATACGAATTAATGGATT
ATTTTTTGATCAAATACCATTATTTGTTTGATCTGTAGGTATTACAGCTTTATTATTATTACTTCTTTACCTGTTTTAG
CTGGAGCTATTACTATATTATTAACAGATCGAAATTTAAATACATCCTTTTTCGATCCTGCTGGAGGAGGAGATCCTATT
CTTTATCAACACTTA------------
>GBGL18410-15|Chilo|KM510319|KM510319
AACATTATATTTTATTTTTGGAATTTGAGCAGGAATAATTGGAACATCCTTAAGTTTATTAATTCGGGCAGAATTAGGAA
ATCCTGGTTCATTAATTGGAGATGATCAAATTTATAACACTATTGTTACAGCTCACGCTTTTATTATAATTTTTTTCATG
GTAATACCAATTATAATTGGTGGATTTGGGAATTGATTAGTTCCTTTAATATTAGGGGCCCCTGATATAGCATTTCCTCG
AATAAATAATATAAGTTTTTGATTATTACCCCCTTCTTTAACTCTTTTAATTTCAAGAAGAATCGTAGAAAATGGAGCTG
GGACAGGATGAACAGTATACCCCCCACTTTCATCTAATATTGCCCATGGTGGAAGTTCTGTTGATTTAGCTATTTTTTCC
TTACATTTAGCGGGAATCTCATCAATTTTAGGAGCTATCAATTTTATTACCACTATTATCAATATACGAATTAATGGTTT
ATCTTTTGATCAAATACCATTATTTGTTTGATCTGTAAGTATTACAGCTTTATTGTTACTCTTGTCATTACCTGTTTTAG
CTGGTGCTATTACTATATTATTAACTGACCGAAATTTAAATACATCTTTTTTTGATCCTGCCGGAGGGGGAGACCCTATT
TTATATCAACACTTA------------
>GBGL18411-15|Chilo|KM510322|KM510322
AACATTATATTTTATTTTTGGAATTTGAGCAGGAATAATTGGGACATCCTTAAGTTTATTAATTCGGGCAGAATTAGGAA
ATCCTGGTTCATTAATTGGAGACGACCAAATTTATAACACTATTGTTACAGCCCACGCTTTTATTATAATTTTTTTTATG
GTAATACCAATTATAATTGGTGGATTTGGAAATTGATTAGTTCCTTTAATATTAGGAGCCCCTGATATAGCATTTCCTCG
AATAAATAATATAAGTTTTTGATTATTACCCCCTTCTTTAACTCTTTTAATTTCAAGAAGAATCGTAGAAAATGGAGCTG
GAACAGGATGAACAGTATACCCCCCACTTTCATCTAATATTGCCCATGGCGGAAGTTCTGTTGATTTAGCTATTTTTTCC
TTACATTTAGCGGGAATCTCATCAATTTTAGGAGCTATCAATTTTATTACCACTATTATCAATATACGAATTAATGGTTT
ATCTTTTGATCAAATACCATTATTTGTTTGATCTGTAAGTATTACAGCTTTATTATTACTCTTATCATTACCTGTTTTAG
CTGGTGCTATTACTATATTATTAACTGACCGAAATTTAAATACATCTTTTTTTGATCCTGCCGGAGGGGGAGACCCCATT
TTATACCAACATTTA------------
>GBGL18412-15|Chilo_suppressalis|KM510324|KM510324
AACTTTATATTTTATTTTTGGTATTTGAGCAGGTATAATTGGAACATCTCTTAGACTTTTAATTCGTGCTGAATTAGGAA
CTCCAGGATCTTTAATTGGAGATGATCAAATTTATAATACCATTGTTACAGCTCATGCATTTATTATGATTTTTTTTATA
GTTATACCAATTATAATTGGTGGATTTGGAAATTGATTAGTACCTTTAATATTAGGGGCTCCTGATATAGCTTTCCCACG
AATAAATAATATAAGATTTTGAATATTACCCCCCTCATTAACTTTACTAATTTCTAGAAGAATTGTTGAAAATGGAGCTG
GAACAGGATGAACAGTGTACCCCCCACTATCATCTAATATTGCTCACGCTGGAAGTTCAGTAGATTTAGCAATTTTCTCT
TTACATTTAGCTGGAATTTCTTCAATTCTAGGTGCTATTAATTTTATTACTACGATTATTAATATACGAATTAATGGTCT
TTCATTTGATCAAATACCTTTATTTGTTTGATCCGTAGGTATTACAGCTTTATTATTACTTCTATCTCTACCAGTATTAG

CTGGAGCAATTACAATATTATTAACCGATCGAAATTTAAATACATCTTTTTTTGATCCTGCTGGTGGTGGAGATCCAATT
CTTTATCAACATTTA------------
>GBGL18413-15|Chilo_suppressalis|KM510325|KM510325
AACTTTATATTTTATTTTTGGTATTTGGGCAGGTATAATTGGAACATCTCTTAGACTTTTAATTCGTGCTGAATTAGGAA
CTCCAGGATCTTTAATTGGAGATGATCAAATTTATAATACCATTGTTACAGCTCATGCATTTATTATGATTTTTTTTATA
GTTATACCAATTATAATTGGTGGATTTGGAAATTGATTAGTACCTTTAATATTAGGGGCTCCTGATATAGCTTTCCCACG
AATAAATAATATAAGATTTTGAATATTACCCCCCTCATTAACTTTACTAATTTCTAGAAGAATTGTTGAAAATGGAGCTG
GAACAGGATGAACAGTGTACCCCCCACTATCATCTAATATTGCTCACGCTGGAAGTTCAGTAGATTTAGCAATTTTCTCT
TTACATTTAGCTGGAATTTCTTCAATTCTAGGTGCTATTAATTTTATTACTACGATTATTAATATACGAATTAATGGTCT
TTCATTTGATCAAATACCTTTATTTGTTTGATCCGTAGGTATTACAGCTTTATTATTACTTCTATCTCTACCAGTATTAG
CTGGAGCAATTACAATATTATTAACCGATCGAAATTTAAATACATCTTTTTTTGATCCTGCTGGTGGTGGAGATCCAATT
CTTTATCAACATTTA------------
>GBGL18414-15|Chilo_suppressalis|KM510326|KM510326
AACTTTATATTTTATTTTTGGTATTTGAGCAGGTATAATTGGAACATCTCTTAGACTTTTAATTCGTGCTGAATTAGGAA
CTCCAGGATCTTTAATTGGAGATGATCAAATTTATAATACCATTGTTACAGCTCATGCATTTATTATGATTTTTTTTATA
GTTATACCAATTATAATTGGTGGATTTGGAAATTGATTAGTACCTTTAATATTAGGGGCTCCTGATATAGCTTTCCCACG
AATAAATAATATAAGATTTTGAATATTACCCCCCTCATTAACTTTACTAATTTCTAGAAGAATTGTTGAAAATGGAGCTG
GAACAGGATGAACAGTGTACCCCCCACTATCATCTAATATTGCTCACGCTGGAAGTTCAGTAGATTTAGCAATTTTCTCT
TTACATTTAGCTGGAATTTCTTCAATTCTAGGTGCTATTAATTTTATTACTACGATTATTAATATACGAATTAATGGTCT
TTCATTTGATCAAATACCTTTATTTGTTTGATCCGTAGGTATTACAGCTTTATTATTACTTCTATCTCTACCAGTATTAG
CTGGAGCAATTACAATATTATTAACCGATCGAAATTTAAATACATCTTTTTTTGATCCTGCTGGTGGTGGAGATCCAATT
CTTTATCAACTTTTA------------
>GBGL18415-15|Chilo_suppressalis|KM510327|KM510327
AACTTTATATTTTATTTTTGGTATTTGAGCAGGTATAATTGGAACATCTCTTAGACTTTTAATTCGTGCTGAATTAGGAA
CTCCAGGATCTTTAATTGGAGATGATCAAATTTATAATACCATTGTTACAGCTCATGCATTTATTATGATTTTTTTTATA
GTTATACCAATTATAATTGGTGGATTTGGAAATTGATTAGTACCTTTAATATTAGGGGCTCCTGATATAGCTTTCCCACG
AATAAATAATATAAGATTTTGAATATTACCCCCCTCATTAACTTTACTAATTTCTAGAAGAATTGTTGAAAATGGAGCTG
GAACAGGATGAACAGTGTACCCCCCACTATCATCTAATATTGCTCACGCTGGAAGTTCAGTAGATTTAGCAATTTTCTCT
TTACATTTAGCTGGAATTTCTTCAATTCTAGGTGCTATTAATTTTATTACTACGATTATTAATATACGAATTAATGGTCT
TTCATTTGATCAAATACCTTTATTTGTTTGATCCGTAGGTATTACAGCTTTATTATTACTTCTATCTCTACCAGTATTAG
CTGGAGCAATTACAATATTATTAACCGATCGAAATTTAAATACATCTTTTTTTGATCCTGCTGGTGGTGGAGATCCAATT
CCTTATCAACATTTA------------
>GBGL18416-15|Chilo_suppressalis|KM510328|KM510328
AACTTTATATTTTATTTTTGGTATTTGATCAGGTATAATTGGAACATCTCTTAGACTTTTAATTCGTGCTGAATTAGGAA
CTCCAGGATCTTTAATTGGAGATGATCAAATTTATAACACTATTGTTACAGCTCATGCATTTATTATAATTTTTTTTATA
GTTATACCAATTATAATTGGTGGATTTGGAAATTGATTAGTACCTTTAATATTAGGAGCTCCTGATATAGCTTTCCCACG
AATAAATAATATAAGATTTTGAATATTACCCCCCTCATTAACTTTACTAATTTCTAGAAGAATTGTTGAAAATGGAGCTG
GAACAGGATGAACAGTGTACCCCCCACTATCATCTAATATTGCTCACGCTGGAAGTTCAGTAGATTTAGCAATTTTCTCT
TTACATTTAGCTGGAATTTCTTCAATTCTAGGTGCTATTAATTTTATTACTACGATTATTAATATACGAATTAATGGTCT
TTCATTTGATCAAATACCTTTATTTGTTTGATCCGTAGGTATTACAGCTTTATTATTACTTCTATCTCTACCAGTATTAG
CTGGAGCAATTACAATATTATTAACCGATCGAAATTTAAATACATCTTTTTTTGATCCTGCTGGTGGTGGAGATCCAATT
CTTTATCAACATTTA------------
>GBGL18417-15|Chilo_suppressalis|KM510329|KM510329
AACTTTATATTTTATTTTTGGTATTTGATCAGGTATAATTGGAACATCTCTTAGACTTTTAATTCGTGCTGAATTAGGAA
CTCCAGGATCTTTAATTGGAGATGATCAAATTTATAACACTATTGTTACAGCTCATGCATTTATTATAATTTTTTTTATA
GTTATACCAATTATAATTGGTGGATTTGGAAATTGATTAGTACCTTTAATATTAGGAGCTCCTGATATAGCTTTCCCACG
AATAAATAATATAAGATTTTGAATATTGCCCCCCTCATTAACTTTACTAATTTCTAGAAGAATTGTTGAAAATGGAGCTG
GAACAGGATGAACAGTGTACCCCCCACTATCATCTAATATTGCTCACGCTGGAAGTTCAGTAGATTTAGCAATTTTCTCT
TTACATTTAGCTGGAATTTCTTCAATTCTAGGTGCTATTAATTTTATTACTACGATTATTAATATACGAATTAATGGTCT
TTCATTTGATCAAATACCTTTATTTGTTTGATCCGTAGGTATTACAGCTTTATTATTACTTCTATCTCTACCAGTATTAG
CTGGAGCAATTACAATATTATTAACCGATCGAAATTTAAATACATCTTTTTTTGATCCTGCTGGTGGTGGAGATCCAATT
CTTTATCAACATTTA------------
>GBGL18418-15|Chilo_suppressalis|KM510330|KM510330
AACTTTATATTTTATTTTTGGTATTTGAGCAGGTATAATTGGAACATCTCTTAGACTTTTAATTCGTGCTGAATTAGGAA
CTCCAGGATCTTTAATTGGAGATGATCAAATTTATAATACCATTGTTACGGCCCATGCATTTATTATAATTTTTTTTATA
GTTATACCAATTATAATTGGTGGATTTGGAAATTGATTAGTACCTTTAATATTAGGAGCTCCTGATATAGCTTTCCCACG
AATAAATAATATAAGATTTTGAATATTACCCCCCTCATTAACTTTACTAATTTCTAGAAGAATTGTTGAAAATGGAGCTG

GAACAGGATGAACAGTGTACCCCCCACTATCATCTAATATTGCTCACGCTGGAAGTTCAGTAGATTTAGCAATTTTCTCT
TTACATTTAGCTGGAATTTCTTCAATTCTAGGTGCTATTAATTTTATTACTACGATTATTAATATACGAATTAATGGTCT
TTCATTTGATCAAATACCTTTATTTGTTTGATCCGTAGGTATTACAGCCTTATTATTACTTCTATCTCTACCAGTATTAG
CTGGAGCAATTACAATATTATTAACCGATCGAAATTTAAATACATCTTTTTTTGATCCTGCTGGTGGTGGAGATCCAATT
CTTTATCAACATCTA------------
>GBGL18419-15|Chilo_suppressalis|KM510331|KM510331
AACTTTATATTTTATTTTTGGTATTTGAGCAGATATAATTGGAACATCTCTTAGACTTTTAATTCGTGCTGAATTAGGAA
CTCCAGGATATTTAATTGGAGATGATCAAATTTATAATACTATTGTTACAGCTCATGCATTTATTATATTTTTTTTTATA
GTTATACCAATTATAATTGGTGGATTTGGAAATTGATTAGTACCTTTAATATTAGGGGCTCCTGATATAGCTTTCCCACG
AATAAATAATATAAGATTTTGAATATTACCCCCCTCATTAACTTTACTAATTTCTAGAAGAATTGTTGAAAATGGAGCTG
GAACAGGATGAACAGTGTACCCCCCACTATCATCTAATATTGCTCACGCTGGAAGTTCAGTAGATTTAGCAATTTTCTCT
TTACATTTAGCTGGAATTTCTTCAATTCTAGGTGCTATTAATTTTATTACTACGATTATTAATATACGAATTAATGGTCT
TTCATTTGATCAAATACCTCTATTTGTTTGATCCGTAGGTATTACAGCTTTATTGTTACTTCTATCTCTACCAGTATTAG
CTGGAGCAATTACAATATTATTAACCGATCGAAATTTAAATACATCTTTTTTTGATCCTGCTGGTGGTGGAGATCCAATT
CTTTATCAACATTTA------------
>GBGL18420-15|Chilo_suppressalis|KM510332|KM510332
AACTTTATATTTTATTTTTGGTATTTGAGCAGGTATAATTGGAACATCTCTTAGACTTTTAATTCGTGCTGAATTAGGAA
CTCCAGGATCTTTAATTGGGGATGATCAAATTTATAATACCATTGTTACAGCTCATGCATTTATTATAATTTTTTTTATA
GTTATACCAATTATAATTGGTGGATTTGGAAATTGATTAGTACCTTTAATATTAGGGGCTCCTGATATAGCTTTCCCACG
AATAAATAATATAAGATTTTGAATATTACCCCCCTCTTTAACTTTACTAATTTCTAGAAGAATTGTTGAAAATGGAGCTG
GAACAGGTTGAACAGTGTACCCCCCACTATCATCTAATATTGCTCACGCTGGAAGTTCAGTAGATTTAGCAATTTTCTCT
TTACATTTAGCTGGAATTTCTTCAATTCTAGGTGCTATTAATTTTATTACTACAATTATTAATATACGAATTAATGGTCT
TTCATTTGATCAAATACCTTTATTTGTTTGATCCGTAGGTATTACAGCTTTATTATTACTTCTATCTCTACCAGTATTAG
CTGGAGCAATTACAATATTATTAACCGATCGAAATTTAAATACATCTTTTTTTGATCCTGCTGGTGGTGGAGATCCAATT
CTTTACCAACATTTA------------
>GBGL18421-15|Chilo_suppressalis|KM510333|KM510333
AACTTTATATTTTATTTTTGGTATTTGAGCAGGTATAATTGGAACATCTCTTAGACTTTTAATTCGTGCTGAATTAGGAA
CTCCAGGATCTTTAATTGGGGATGATCAAATTTATAATACCATTGTTACAGCTCATGCATTTATTATAATTTTTTTTATA
GTTATACCAATTATAATTGGTGGATTTGGAAATTGATTAGTACCTTTAATATTAGGGGCTCCTGATATAGCTTTCCCACG
AATAAATAATATAAGATTTTGAATATTACCCCCCTCTTTAACTTTACTAATTTCTAGAAGAATTGTTGAAAATGGAGCTG
GAACAGGTTGAACAGTGTACCCCCCACTATCATCTAATATTGCTCACGCTGGAAGTTCAGTAGATTTAGCAATTTTCTCT
TTACATTTAGCTGGAATTTCTTCAATTCTAGGTGCTATTAATTTTATTACTACAATTATTAATATACGAATTAATGGTCT
TTCATTTGATCAAATACCTTTATTTGTTTGATCCGTAGGTATTACAGCTTTATTATTACTTCTATCTCTACCAGTATTAG
CTGGAGCAATTACAATATTATTAACCGATCGAAATTTAAATACATCTTTTTTTGATCCTGCTGGTGGTGGGGATCCAATT
CTTTACCAACATTTA------------
>GBGL18422-15|Chilo_suppressalis|KM510334|KM510334
AACTTTATATTTTATTTTTGGTATTTGAGCAGGTATAATTGGAACATCTCTTAGACTTTTAATTCGTGCTGAATTAGGAA
CTCCAGGATCTTTAATTGGGGATGATCAAATTTATAATACCATTGTTACAGCTCATGCATTTATTATATTTTTTTTTATA
GTTATACCAATTATAATTGGTGGATTTGGAAATTGATTAGTACCTTTAATATTAGGGGCTCCTGATATAGCTTTCCCACG
AATAAATAATATAAGATTTTGAATATTACCCCCCTCTTTAACTTTACTAATTTCTAGAAGAATTGTTGAAAATGGAGCTG
GAACAGGTTGAACAGTGTACCCCCCACTATCATCTAATATTGCTCACGCTGGAAGTTCAGTAGATTTAGCAATTTTCTCT
TTACATTTAGCTGGAATTTCTTCAATTCTAGGTGCTATTAATTTTATTACTACAATTATTAATATACGAATTAATGGTCT
TTCATTTGATCAAATACCTTTATTTGTTTGATCCGTAGGTATTACAGCTTTATTATTACTTCTATCTCTACCAGTATTAG
CTGGAGCAATTACAATATTATTAACCGATCGAAATTTAAATACATCTTTTTTTGATCCTGCTGGTGGTGGGGATCCAATT
CTTTACCAACATTTA------------
>GBGL18423-15|Chilo_suppressalis|KM510335|KM510335
AACTTTATATTTTATTTTTGGTATTTGAGCAGGTATAATTGGAACATCTCTTAGACTTTTAATTCGTGCTGAATTAGGAA
CTCCAGGATCTTTAATTGGAGATGATCAAATTTATAATACCATTGTTACAGCTCATGCATTTATTATAATTTTTTTTATA
GTTATACCAATTATAATTGGTGGATTTGGAAATTGATTAGTACCTTTAATATTAGGGGCTCCTGATATAGCTTTCCCACG
AATAAATAATATAAGATTTTGAATATTACCCCCCTCTTTAACTTTACTAATTTCTAGAAGAATTGTTGAAAATGGAGCTG
GAACAGGTTGAACAGTGTACCCCCCACTATCATCTAATATTGCTCACGCTGGAAGTTCAGTAGATTTAGCAATTTTCTCT
TTACATTTAGCTGGAATTTCTTCAATTCTAGGTGCTATTAATTTTATTACTACAATTATTAATATACGAATTAATGGTCT
TTCATTTGATCAAATACCTTTATTTGTTTGATCCGTAGGTATTACAGCTTTATTATTACTTCTATCTCTACCAGTATTAG
CTGGAGCAATTACAATATTATTAACCGATCGAAATTTAAATACATCTTTTTTTGATCCTGCTGGTGGTGGAGATCCAATT
CTTTACCAACATTTA------------
>GBGL18424-15|Chilo|KM510336|KM510336
AACTTTATATTTTATTTTTGGAATTTGAGCTGGAATAGTTGGTACTTCTTTAAGATTATTAATTCGTGCAGAATTAGGAA

ATCCTGGATCTTTAATTGGAGATGATCAAATTTATAATACTATTGTAACTGCACATGCATTTATTATAATTTTTTTATA
GTTATACCAATTATAATTGGAGGATTTGGAAATTGATTAGTACCTTTAATATTAGGTGCTCCAGATATAGCTTTCCCACG
AATAAATAACATAAGATTTTGATTATTACCCCCTTCTTTAACTTTATTAATTTCTAGAAGAATTGTAGAAAATGGAGCAG
GAACCGGATGAACAGTATATCCCCCACTTTCATCTAATATTGCTCATGGAGGTAGCTCAGTTGATTTAGCTATTTTTTCT
TTACATTTAGCTGGAATTTCTTCTATTTTAGGTGCTATCAATTTTATCACTACAATTATTAATATACGAATTAACGGTTT
ATCATTCGATCAAATACCACTATTTGTATGATCAGTAGGAATTACAGCATTACTTCTTCTTTTATCTTTACCAGTATTAG
CTGGAGCTATTACTATATTATTAACAGATCGAAATTTAAATACTTCTTTTTTTGATCCTGCAGGAGGAGGTGATCCTATT
TTATATCAACATTTA------------
>GBGL18425-15|Sesamia_inferens|KM510338|KM510338
AACATTATATTTTATTTTTGGAATTTGAGCTGGTATAGTAGGAACATCATTAAGATTATTAATTCGAGCTGAATTAGGAA
CTCCTGGGTCTTTAATTGGGGATGATCAAATTTATAATACTATTGTTACAGCTCATGCTTTTATTATAATTTTTTTTATA
GTTATACCAATTATAATTGGAGGATTTGGAAATTGACTTGTACCTTTAATATTAGGAGCTCCTGATATAGCATTCCCACG
AATAAATAATATAAGATTTTGATTGTTACCTCCTTCTTTAACTCTTTTAATTTCAAGCAGAATTGTAGAAAGTGGAGCAG
GTACTGGATGAACAGTGTACCCCCCTCTTTCATCCAATATTGCCCATGGAGGAAGATCAGTAGATTTAGCTATTTTCTCC
CTTCATTTAGCTGGTATTTCATCTATTTTAGGAGCTATTAATTTTATTACAACAATTATCAATATACGACTAAATAGTTT
ATCTTTTGATCAAATACCTCTATTTATTTGAGCTGTTGGAATTACTGCATTTTTATTATTACTATCTTTACCTGTATTAG
CAGGAGCTATCACAATATTATTAACAGATCGAAATTTAAATACATCATTCTTTGACCCCGCAGGGGGAGGTGACCCTATT
TTATACCAACATTTA------------
>GBGL18593-15|Diatraea_considerata|KP259614|KP259614
AACTTTATACTTTATTTTTGGTATTTGAGCAGGNATGTTAGGAACATCATTAAGTCTTTTAATTCGAGCAGAATTAGGAA
CTCCTAATTCTTTAATTGGAGATGATCAAATTTATAATACAATTGTTACAGCTCATGCTTTTATTATAATTTTCTTTATA
GTAATACCAATTATAATTGGAGGATTTGGTAATTGATTAGTACCCTTAATATTAGGAGCTCCTGATATAGCTTTTCCTCG
AATAAATAACATAAGATTTTGATTATTACCCCCATCTTTAACTCTATTAATTTCTAGAAGAATTGTAGAAAATGGAGCTG
GAACAGGATGAACAGTTTACCCCCCTCTATCATCTAATATTGCCCATGGAGGTAGATCAGTAGATTTAGCAATTTTTTCT
TTACATTTAGCTGGAATTTCTTCAATTTTAGGAGCTATTAATTTTATTACTACTATTATTAATATACGAATTAATGGTTT
ATCATTTGATCAAATACCTTTATTTATTTGATCAGTAGGTATTACAGCTTTACTTTTATTGCTTTCTTTACCAGTATTAG
CTGGAGCTATTACTATACTATTAACAGACCGAAATTTAAATACTTCTTTTTTTGATCCAGCTGGAGGAGGAGATCCAATT
CTATATCAACATTTATTTTGATTTTTT
>GBGL18594-15|Diatraea_grandiosella|KP259615|KP259615
----------------TTTGGANTTTGAGCAGGGATATTAGGAACATCCCTAAGTTTACTAATTCGTGCTGAACTCGGTA
CACCTAATTCTTTAATTGGTGATGATCAAATTTATAATACAATTGTTACAGCTCATGCATTTATTATAATTTTTTTTATA
GTTATACCTATTATAATTGGGGGATTTGGAAATTGATTAGTACCCCTTATATTAGGAGCTCCGGATATGGCTTTTCCACG
AATAAATAATATAAGATTTTGATTATTACCCCCATCTTTAACTTTATTAATTTCCAGAAGAATTGTTGAAAATGGAGCTG
GAACAGGATGAACAGTATACCCCCCATTATCATCAAATATTGCCCACGGGGGAAGATCAGTAGATTTAGCAATTTTTTCT
CTACATTTAGCTGGAATTTCATCAATTTTAGGAGCCATTAATTTCATTACCACAATTATTAACATACGAATTAATGGGCT
ATCTTTTGATCAAATACCTTTATTCGTATGATCTGTTGGTATTACTGCATTACTATTACTTCTCTCTTTACCAGTATTAG
CTGGTGCTATTACTATACTATTAACTGATCGAAATTTAAATACTTCTTTCTTTGACCCGGCGGGAGGGGGAGACCCTATT
CTCTATCAACATCTTTTTTG-------
>GBGL18632-15|Eoreuma_loftini|KP259616|KP259616
AACTTTATATTTTATTTTTGGANTTTGAGCTGGNACAGTAGGAACATCTTTAAGTTTATTAATTCGAGCTGAATTAGGAA
ACCCAGGCTCTTTAATTGGAGATGATCAAATTTATAATACTATTGTTACAGCCCATGCATTTATTATAATTTTTTTTATA
GTAATACCTATTATAATTGGGGGATTTGGAAATTGATTAGTACCTTTAATATTAGGAGCTCCTGATATAGCATTCCCACG
AATAAATAATATAAGATTTTGATTATTACCCCCATCTTTAACACTACTAATTTCAAGAAGTATTGTTGAAAATGGAGCTG
GAACAGGATGAACAGTTTATCCCCCTCTTTCTTCTAATATTGCTCATGGAGGTAGATCTGTAGATCTAGCAATTTTTTCT
CTTCATTTAGCTGGAATTTCCTCAATTTTAGGAGCTATTAATTTTATTACAACAATTATTAATATACGAATTAATAATTT
ATCCTTTGATCAAATACCTTTATTTGTTTGATCAGTAGGTATTACAGCTTTACTTCTTCTTCTTTCTTTACCAGTATTAG
CTGGAGCTATTACTATATTATTAACAGATCGAAATTTAAATACTTCCTTTTTTGATCCTGCAGGGGGTGGAGATCCAAT-
----
>GBGL20196-15|Scirpophaga_excerptalis|KJ013411|KJ013411
GACATTATATTTTATTTTTGGAATTTGAGCTGGTATAGTGGGAACTTCCCTTAGTTTACTAATTCGAGCCGAACTAGGTA
CTCCTGGATCACTAATTGGAGATGATCAAATCTATAATACTATTGTAACTGCTCACGCTTTTATTATAATTTTTTTTATA
GTTATACCTATTATAATTGGGGGATTCGGAAACTGATTAGTACCTTTAATATTAGGAGCTCCAGATATAGCTTTTCCCCG
AATAAACAACATAAGTTTTTGATTATTACCCCCTTCTTTAACCCTCTTAATCTCAAGAAGAATTGTTGAAAATGGAGCTG
GAACAGGATGAACTGTTTACCCGCCCCTATCCTCCAATATTGCCCACGGTGGGACTTCTGTAGATTTAGCTATTTTTTCA
TTACATTTAGCTGGAATTTCTTCTATTCTAGGGGCTATTAACTTCATTACAACTATTATTAATATACGAATTAATGGACT
ATCCTTTGATCAAATACCTTTATTCGTGTGAGCAGTTGGTATTACTGCCCTTCTTCTTCTTCTCTCACTACCTGTATTAG
CGGGAGCTATCACTATATTATTAACAGATCGAAACCTAAATACCTCTTTCTTTGACCCAGCAGGAGGGGGGGACCCAATT

CTTTATCAACATTTA------------
>GBGL20198-15|Sesamia_inferens|KJ013410|KJ013410
AACATTATATTTTATTTTTGGGATTTGAGCTGGTATAGTAGGAACATCATTAAGATTATTAATTCGAGCTGAATTAGGAA
CCCCAGGATCTTTAATTGGAGATGATCAAATTTATAATACTATTGTTACAGCTCATGCTTTTATTATAATTTTTTTTATA
GTTATACCAATTATAATTGGAGGATTTGGAAATTGACTTGTACCTTTAATATTAGGAGCTCCTGATATAGCATTTCCACG
AATAAATAATATAAGATTTTGATTATTACCCCCCTCTTTAACTCTTTTAATTTCAAGTAGAATTGTAGAAAATGGAGCAG
GAACTGGATGAACAGTGTACCCCCCACTTTCATCTAATATTGCTCATGGAGGAAGATCAGTAGATCTAGCTATTTTTTCC
CTTCATTTAGCTGGTATTTCATCTATTTTAGGAGCTATTAATTTTATTACAACAATTATCAATATACGACTAAATAGTTT
ATCTTTTGATCAAATACCTCTATTTATTTGAGCTGTTGGAATTACTGCATTTTTATTATTATTATCTTTACCTGTATTAG
CAGGAGCTATTACAATATTATTGACAGATCGAAATTTAAATACATCATTCTTTGACCCCGCAGGAGGGGGTGATCCTATT
TTATACCAACATTTA------------
>GBGL20667-18|Sesamia_nonagrioides|KU891969|KU891969
-----------------TGG-ATTTGAGCTGGAATAGTAGGAACTTCATTAAGACTATTAATTCGAGCTGAATTAGGAA
CTCCTGGATCTTTAATTGGAGATGATCAAATTTATAATACTATTGTTACAGCTCATGCTTTTATTATAATTTTTTTTATA
GTTATACCTATTATAATTGGAGGATTTGGAAATTGACTTGTACCTTTAATATTAGGAGCCCCAGATATAGCATTTCCACG
AATAAATAATATAAGATTTTGACTATTACCACCATCCTTAACCCTTTTAATTTCAAGTAGAATTGTAGAAAATGGGGGTG
GAACAGGATGAACAGTTTACCCCCCACTTTCATCTAACATCGCTCATGGAGGAAGATCTGTAGATTTAGCTATTTTTTCC
CTTCATTTAGCTGGAATTTCATCTATTCTAGGAGCTATTAATTTTATTACAACAATTATTAATATACGATTAAATAATTT
ATCATTTGATCAAATACCATTATTTATTTGAGCTGTTGGAATTACTGCTTTTTTATTACTATTATCATTACCCGTTTTAG
CAGGAGCTATTACTATACTACTTACGGATCGAAATTTAAATACATCATTTTTTGATCCTGCGGGAGGAGGTGATCCAATT
TTATACCAACACTTATTCTGATTTTT-
>GBGL20668-18|Sesamia_cretica|KU891973|KU891973_(reversed)
--AAATAGGGA
TTCCTGGATCTTTAATTGGAGATGATCAAATTTATAATACTATTGTTACAGCTCATGCTTTTATCATAATTTTTTTTATA
GTTATACCAATTATAATTGGAGGATTTGGTAATTGACTCGTACCTTTAATATTAGGAGCTCCAGATATAGCATTTCCACG
AATAAATAACATAAGATTTTGATTATTACCCCCTTCTTTAACTTTATTAATTTCAAGAAGAATTGTAGAAAATGGAGCAG
GTACCGGATGAACGGTATATCCCCCCCTCTCATCTAATATTGCTCATGGAGGAAGATCTGTAGATTTAGCTATTTTTTCC
CTTCATTTAGCGGGTATTTCATCTATTTTAGGAGCGATTAATTTTATTACAACAATTATTAATATACGATTAAATAACTT
ATCTTTTGATCAAATACCTTTATTTGTTTGAGCTGTTGGAATTACTGCATTCTTATTATTATTATCTTTACCTGTTTTAG
CAGGAGCTATTACAATATTATTAACAGATCGAAATTTAAATACATCATTCTTTGATCCTGCAGGAGGAGGTGATCCAATT
TTATATCAACATTTATTTTGATTTTT-
>GBGL20669-18|Sesamia_cretica|KU891975|KU891975_(reversed)
--AAATAGGGA
TTCCTGGATCTTTAATTGGAGATGATCAAATTTATAATACTATTGTTACAGCTCATGCTTTTATCATAATTTTTTTTATA
GTTATACCAATTATAATTGGAGGATTTGGTAATTGACTCGTACCTTTAATATTAGGAGCTCCAGATATAGCATTTCCACG
AATAAATAACATAAGATTTTGATTATTACCCCCTTCTTTAACTTTATTAATTTCAAGAAGAATTGTAGAAAATGGAGCAG
GTACAGGATGAACGGTATATCCCCCCCTCTCATCTAATATTGCTCATGGAGGAAGATCTGTAGATTTAGCTATTTTTTCC
CTTCATTTAGCGGGTATTTCATCTATTTTAGGAGCTATTAATTTTATTACAACAATTATTAATATACGATTAAATAACTT
ATCTTTTGATCAAATACCTTTATTTGTTTGAGCTGTTGGAATTACTGCATTCTTATTATTATTATCTTTACCTGTTTTAG
CAGGAGCTATTACAATATTATTAACAGATCGAAATTTAAATACATCATTCTTTGATCCTGCAGGAGGAGGTGATCCAATT
TTATATCAACATTTATTTTGATTTTT-
>GBMIN22544-13|Acrapex_syscia|JX282424|JX282424
-ACATTATATTTTATTTTTGGAATTTGGGCTGGTATAGTGGGAACTTCTTTGAGATTATTAATTCGAGCTGAATTAGGAA
CTCCTGGATCTTTAATTGGAGACGATCAAATTTATAATACTATTGTTACAGCCCATGCTTTTATTATAATTTTTTTTATA
GTTATACCAATTATAATTGGAGGATTTGGAAATTGACTTGTACCTTTAATATTAGGAGCCCCAGATATAGCATTCCCCCG
AATAAATAATATAAGTTTTTGATTATTACCTCCTTCCTTAACCCTTTTAATTTCCAGAAGAATCGTAGAAAATGGGGCTG
GAACTGGGTGAACTGTATATCCCCCCTTATCGTCTAATATTGCTCATGGAGGAAGTTCTGTAGATTTAGCCATTTTTTCC
CTTCATTTAGCTGGAATCTCCTCTATCTTAGGTGCTATTAATTTTATTACAACAATTATTAATATACGATTAAATAACCT
ATCTTTTGATCAAATACCTTTATTTATTTGAGCTGTAGGTATCACCGCATTCTTACTATTATTATCTTTACCTGTTTTAG
CTGGAGCTATTACTATACTATTAACAGATCGAAACTTAAACACTTCTTTTTTTGACCCTGCTGGAGGGGGCGATCCAATC
TTATACCAACATTTATTTTGATTTTTT
>GBMIN22547-13|Sesamia_nonagrioides|JX282463|JX282463
-ACATTATATTTTATTTTTGGAATTTGAGCTGGAATAGTAGGAACTTCATTAAGACTATTAATTCGAGCTGAATTAGGAA
CTCCTGGATCTTTAATTGGAGATGATCAAATTTATAATACTATTGTTACAGCTCATGCTTTTATTATAATTTTTTTTATA
GTTATACCTATTATAATTGGAGGATTTGGAAATTGACTTGTACCTTTAATATTAGGAGCCCCAGATATAGCATTTCCACG
AATAAATAATATAAGATTTTGACTATTACCACCATCCTTAACCCTTTTAATTTCAAGTAGAATTGTAGAAAATGGGGCTG
GAACAGGATGAACAGTTTACCCCCCACTTTCATCTAACATCGCTCACGGAGGAAGATCTGTAGATTTAGCTATTTTTTCC

CTTCATTTAGCTGGAATTTCATCTATTCTAGGAGCTATTAATTTTATTACAACAATTATTAATATACGATTAAATAATTT
ATCATTTGATCAAATACCATTATTTATTTGAGCTGTTGGAATTACTGCTTTTTTATTACTATTATCATTACCCGTTTTAG
CAGGAGCTATTACTATACTACTTACGGATCGAAATTTAAATACATCATTTTTTGATCCTGCGGGAGGAGGTGATCCAATT
TTATACCAACACTTATTCTGATTTTTT
>GBMIN29754-13|Chilo_suppressalis|AB238205|AB238205
AACTTTATATTTTATTTTTGGTATTTGAGCAGGTATAATTGGAACATCTCTTAGACTTTTAATTCGTGCTGAATTAGGAA
CTCCAGGATCTTTAATTGGGGATGATCAAATTTATAATACCATTGTTACGGCTCATGCATTTATTATAATTTTTTTTATA
GTTATACCAATTATAATTGGTGGATTTGGAAATTGATTAGTACCTTTAATATTAGGAGCTCCTGATATAGCTTTCCCACG
AATAAATAATATAAGATTTTGAATATTACCCCCCTCATTAACTTTACTAATTTCTAGAAGAATTGTTGAAAATGGAGCTG
GAACAGGTTGAACAGTGTACCCCCCACTATCATCTAATATTGCTCACGCTGGAAGTTCAGTAGATTTAGCAATTTTCTCT
TTACATTTAGCTGGAATTTCTTCAATTCTAGGTGCTATTAATTTTATTACTACGATTATTAATATACGAATTAATGGTCT
TTCATTTGACCAAATACCTTTATTTGTTTGATCCGTAGGTATTACAGCCTTATTATTACTTCTATCTCTACCAGTATTAG
CTGGAGCAATTACAATATTATTAACCGATCGAAATTTAAATACATCTTTTTTTGATCCTGCTGGTGGTGGAGATCCAATT
CTTTACCAACATTTATTTTGATTTTTT
>GBMIN29755-13|Chilo_suppressalis|AB238203|AB238203
AACTTTATATTTTATTTTTGGTATTTGAGCAGGTATAATTGGAACATCTCTTAGACTTTTAATTCGTGCTGAATTAGGAA
CTCCAGGATCTTTAATTGGAGATGATCAAATTTATAATACCATTGTTACGGCTCATGCATTTATTATAATTTTTTTTATA
GTTATACCAATTATAATTGGTGGATTTGGAAATTGATTAGTACCTTTAATATTAGGGGCTCCTGATATAGCTTTCCCACG
AATAAATAATATAAGATTTTGAATATTACCCCCCTCATTAACTTTACTAATTTCTAGAAGAATTGTTGAAAATGGAGCTG
GAACAGGTTGAACAGTGTACCCCCCACTATCATCTAATATTGCTCACGCTGGAAGTTCAGTAGATTTAGCAATTTTCTCT
TTACATTTAGCTGGAATTTCTTCAATTCTAGGTGCTATTAATTTTATTACTACGATTATTAATATACGAATTAATGGTCT
TTCATTTGATCAAATACCTTTATTTGTTTGATCCGTAGGTATTACAGCTTTATTATTACTTCTATCTCTACCAGTATTAG
CTGGAGCAATTACAATATTATTAACCGATCGAAATTTAAATACATCTTTTTTTGATCCTGCTGGTGGTGGAGATCCAATT
CTTTACCAACATTTATTTTGATTTTTT
>GBMIN30577-13|Diatraea_saccharalis|JN108985|JN108985
-------TATTTTATTTTTGGAATTTGAGCAGGAATATTAGGAACTTCATTAAGTTTATTAATTCGAGCAGAATTAGGAA
CATCTAACTCTTTAATTGGAGATGATCAAATTTATAACACAATTGTAACAGCTCATGCTTTCATTATAATTTTTTTTATA
GTTATACCTATTATAATTGGAGGATTTGGAAATTGATTGGTACCTTTAATATTAGGAGCCCCCGATATAGCTTTCCCACG
AATAAATAATATAAGATTTTGATTATTACCCCCATCTTTAACCCTACTAATTTCTAGAAGAATTGTAGAAAATGGAGCAG
GAACAGGATGAACAGTTTATCCCCCATTATCATCTAATATTGCCCATGGGGGAAGATCTGTGGACTTAGCAATTTTCTCT
TTACATTTAGCTGGGATTTCCTCAATTTTAGGGGCTATTAATTTCATCACTACCATTATTAATATACGAATTAATAATCT
ATCATTTGATCAAATACCTTTATTTATTTGATCAGTGGGCATTACAGCATTACTTTTATTACTTTCTTTACCAGTATTAG
CTGGAGCCATTACTATATTACTAACAGATCGAAATTTAAATACCTCCTTCTTTGATCCAGCGGGAGGGGGAGATCCTATT
CTATATCAACATTTATTTTGATTTTTT
>GBMIN30578-13|Diatraea_saccharalis|JN108983|JN108983
-------TATTTTATTTTTGGAATTTGAGCAGGAATATTAGGAACTTCATTAAGTTTATTAATTCGAGCAGAATTAGGAA
CATCTAACTCTTTAATTGGAGATGATCAAATTTATAACACAATTGTAACAGCTCATGCTTTCATTATAATTTTTTTTATA
GTTATACCTATTATAATTGGAGGATTTGGAAATTGATTGGTACCTTTAATATTAGGAGCCCCCGATATAGCTTTCCCACG
AATAAATAATATAAGATTTTGATTATTACCCCCATCTTTAACCCTACTAATTTCTAGAAGAATTGTAGAAAATGGAGCAG
GAACAGGATGAACAGTTTATCCCCCATTATCATCTAATATTGCCCATGGGGGAAGATCTGTGGACTTAGCAATTTTCTCT
TTACATTTAGCTGGGATTTCCTCAATTTTAGGGGCTATTAATTTCATCACTACCATTATTAATATACGAATTAATAATCT
ATCATTTGATCAAATACCTTTATTTATTTGATCAGTGGGCATTACAGCATTACTTTTATTACTTTCTTTACCAGTATTAG
CTGGAGCCATTACTATATTACTAACAGATCGAAATTTAAATACCTCCTTCTTTGATCCAGCGGGAGGGGGAGATCCTATT
CTATATCAACATTTATTTTGATTTTTT
>GBMIN30579-13|Diatraea_saccharalis|JN108981|JN108981
-------TATTTTATTTTTGGAATTTGAGCAGGAATATTAGGAACTTCATTAAGTTTATTAATTCGAGCAGAATTAGGAA
CATCTAACTCTTTAATTGGAGATGATCAAATTTATAACACAATTGTAACAGCTCATGCTTTCATTATAATTTTTTTTATA
GTTATACCTATTATAATTGGAGGATTTGGAAATTGATTGGTACCTTTAATATTAGGAGCCCCCGATATAGCTTTCCCACG
AATAAATAATATAAGATTTTGATTATTACCCCCATCTTTAACCCTACTAATTTCTAGAAGAATTGTAGAAAATGGAGCAG
GAACAGGATGAACAGTTTATCCCCCATTATCATCTAATATTGCCCATGGGGGAAGATCTGTGGACTTAGCAATTTTCTCT
TTACATTTAGCTGGGATTTCCTCAATTTTAGGGGCTATTAATTTCATCACTACCATTATTAATATACGAATTAATAATCT
ATCATTTGATCAAATACCTTTATTTATTTGATCAGTGGGCATTACAGCATTACTTTTATTACTTTCTTTACCAGTATTAG
CTGGAGCCATTACTATATTACTAACAGATCGAAATTTAAATACCTCCTTCTTTGATCCAGCGGGAGGGGGAGATCCTATT
CTATATCAACATTTATTTTGATTTTTT
>GBMIN30580-13|Diatraea_saccharalis|JN108979|JN108979
-------TATTTTATTTTTGGAATTTGAGCAGGAATATTAGGAACTTCATTAAGTTTATTAATTCGAGCAGAATTAGGAA
CATCTAACTCTTTAATTGGAGATGATCAAATTTATAACACAATTGTAACAGCTCATGCTTTCATTATAATTTTTTTTATA

GTTATACCTATTATAATTGGAGGATTTGGAAATTGATTGGTACCTTTAATATTAGGAGCCCCCGATATAGCTTTCCCACG
AATAAATAATATAAGATTTTGATTATTACCCCCATCTTTAACCCTACTAATTTCTAGAAGAATTGTAGAAAATGGAGCAG
GAACAGGATGAACAGTTTATCCCCCATTATCATCTAATATTGCCCATGGGGGAAGATCTGTGGACTTAGCAATTTTCTCT
TTACATTTAGCTGGGATTTCCTCAATTTTAGGGGCTATTAATTTCATCACTACCATTATTAATATACGAATTAATAATCT
ATCATTTGATCAAATACCTTTATTTATTTGATCAGTGGGCATTACAGCATTACTTTTATTACTTTCTTTACCAGTATTAG
CTGGAGCCATTACTATATTACTAACAGATCGAAATTTAAATACCTCCTTCTTTGATCCAGCGGGAGGGGGAGATCCTATT
CTATATCAACATTTATTTTGATTTTTT
>GBMIN30581-13|Diatraea_saccharalis|JN108977|JN108977
-------TATTTTATTTTTGGAATTTGAGCAGGAATATTAGGAACTTCATTAAGTTTATTAATTCGAGCAGAATTAGGAA
CATCTAACTCTTTAATTGGAGATGATCAAATTTATAACACAATTGTAACAGCTCATGCTTTCATTATAATTTTTTTTATA
GTTATACCTATTATAATTGGAGGATTTGGAAATTGATTGGTACCTTTAATATTAGGAGCCCCCGATATAGCTTTCCCACG
AATAAATAATATAAGATTTTGATTATTACCCCCATCTTTAACCCTACTAATTTCTAGAAGAATTGTAGAAAATGGAGCAG
GAACAGGATGAACAGTTTATCCCCCATTATCATCTAATATTGCCCATGGGGGAAGATCTGTGGACTTAGCAATTTTCTCT
TTACATTTAGCTGGGATTTCCTCAATTTTAGGGGCTATTAATTTCATCACTACCATTATTAATATACGAATTAATAATCT
ATCATTTGATCAAATACCTTTATTTATTTGATCAGTGGGCATTACAGCATTACTTTTATTACTTTCTTTACCAGTATTAG
CTGGAGCCATTACTATATTACTAACAGATCGAAATTTAAATACCTCCTTCTTTGATCCAGCGGGAGGGGGAGATCCTATT
CTATATCAACATTTATTTTGATTTTTT
>GBMIN30582-13|Diatraea_saccharalis|JN108975|JN108975
-------TATTTTATTTTTGGAATTTGAGCAGGAATATTAGGAACTTCATTAAGTTTATTAATTCGAGCAGAATTAGGAA
CATCTAACTCTTTAATTGGAGATGATCAAATTTATAACACAATTGTAACAGCTCATGCTTTCATTATAATTTTTTTTATA
GTTATACCTATTATAATTGGAGGATTTGGAAATTGATTGGTACCTTTAATATTAGGAGCCCCCGATATAGCTTTCCCACG
AATAAATAATATAAGATTTTGATTATTACCCCCATCTTTAACCCTACTAATTTCTAGAAGAATTGTAGAAAATGGAGCAG
GAACAGGATGAACAGTTTATCCCCCATTATCATCTAATATTGCCCATGGGGGAAGATCTGTGGACTTAGCAATTTTCTCT
TTACATTTAGCTGGGATTTCCTCAATTTTAGGGGCTATTAATTTCATCACTACCATTATTAATATACGAATTAATAATCT
ATCATTTGATCAAATACCTTTATTTATTTGATCAGTGGGCATTACAGCATTACTTTTATTACTTTCTTTACCAGTATTAG
CTGGAGCCATTACTATATTACTAACAGATCGAAATTTAAATACCTCCTTCTTTGATCCAGCGGGAGGGGGAGATCCTATT
CTATATCAACATTTATTTTGATTTTTT
>GBMIN30583-13|Diatraea_saccharalis|JN108973|JN108973
-------TATTTTATTTTTGGAATTTGAGCAGGAATATTAGGAACTTCATTAAGTTTATTAATTCGAGCAGAATTAGGAA
CATCTAACTCTTTAATTGGAGATGATCAAATTTATAACACAATTGTAACAGCTCATGCTTTCATTATAATTTTTTTTATA
GTTATACCTATTATAATTGGAGGATTTGGAAATTGATTGGTACCTTTAATATTAGGAGCCCCCGATATAGCTTTCCCACG
AATAAATAATATAAGATTTTGATTATTACCCCCATCTTTAACCCTACTAATTTCTAGAAGAATTGTAGAAAATGGAGCAG
GAACAGGATGAACAGTTTATCCCCCATTATCATCTAATATTGCCCATGGGGGAAGATCTGTGGACTTAGCAATTTTCTCT
TTACATTTAGCTGGGATTTCCTCAATTTTAGGGGCTATTAATTTCATCACTACCATTATTAATATACGAATTAATAATCT
ATCATTTGATCAAATACCTTTATTTATTTGATCAGTGGGCATTACAGCATTACTTTTATTACTTTCTTTACCAGTATTAG
CTGGAGCCATTACTATATTACTAACAGATCGAAATTTAAATACCTCCTTCTTTGATCCAGCGGGAGGGGGAGATCCTATT
CTATATCAACATTTATTTTGATTTTTT
>GBMIN30584-13|Diatraea_saccharalis|JN108971|JN108971
-------TATTTTATTTTTGGAATTTGAGCAGGAATATTAGGAACTTCATTAAGTTTATTAATTCGAGCAGAATTAGGAA
CATCTAACTCTTTAATTGGAGATGATCAAATTTATAACACAATTGTAACAGCTCATGCTTTCATTATAATTTTTTTTATA
GTTATACCTATTATAATTGGAGGATTTGGAAATTGATTGGTACCTTTAATATTAGGAGCCCCCGATATAGCTTTCCCACG
AATAAATAATATAAGATTTTGATTATTACCCCCATCTTTAACCCTACTAATTTCTAGAAGAATTGTAGAAAATGGAGCAG
GAACAGGATGAACAGTTTATCCCCCATTATCATCTAATATTGCCCATGGGGGAAGATCTGTGGACTTAGCAATTTTCTCT
TTACATTTAGCTGGGATTTCCTCAATTTTAGGGGCTATTAATTTCATCACTACCATTATTAATATACGAATTAATAATCT
ATCATTTGATCAAATACCTTTATTTATTTGATCAGTGGGCATTACAGCATTACTTTTATTACTTTCTTTACCAGTATTAG
CTGGAGCCATTACTATATTACTAACAGATCGAAATTTAAATACCTCCTTCTTTGATCCAGCGGGAGGGGGAGATCCTATT
CTATATCAACATTTATTTTGATTTTTT
>GBMIN30585-13|Diatraea_saccharalis|JN108969|JN108969
-------TATTTTATTTTTGGAATTTGAGCAGGAATATTAGGAACTTCATTAAGTTTATTAATTCGAGCAGAATTAGGAA
CATCTAACTCTTTAATTGGAGATGATCAAATTTATAACACAATTGTAACAGCTCATGCTTTCATTATAATTTTTTTTATA
GTTATACCTATTATAATTGGAGGATTTGGAAATTGATTGGTACCTTTAATATTAGGAGCCCCCGATATAGCTTTCCCACG
AATAAATAATATAAGATTTTGATTATTACCCCCATCTTTAACCCTACTAATTTCTAGAAGAATTGTAGAAAATGGAGCAG
GAACAGGATGAACAGTTTATCCCCCATTATCATCTAATATTGCCCATGGGGGAAGATCTGTGGACTTAGCAATTTTCTCT
TTACATTTAGCTGGGATTTCCTCAATTTTAGGGGCTATTAATTTCATCACTACCATTATTAATATACGAATTAATAATCT
ATCATTTGATCAAATACCTTTATTTATTTGATCAGTGGGCATTACAGCATTACTTTTATTACTTTCTTTACCAGTATTAG
CTGGAGCCATTACTATATTACTAACAGATCGAAATTTAAATACCTCCTTCTTTGATCCAGCGGGAGGGGGAGATCCTATT
CTATATCAACATTTATTTTGATTTTTT

>GBMIN30586-13|Diatraea_saccharalis|JN108967|JN108967
-------TATTTTATTTTTGGAATTTGAGCAGGAATATTAGGAACTTCATTAAGTTTATTAATTCGAGCAGAATTAGGAA
CATCTAACTCTTTAATTGGAGATGATCAAATTTATAACACAATTGTAACAGCTCATGCTTTCATTATAATTTTTTTTATA
GTTATACCTATTATAATTGGAGGATTTGGAAATTGATTGGTACCTTTAATATTAGGAGCCCCCGATATAGCTTTCCCACG
AATAAATAATATAAGATTTTGATTATTACCCCCATCTTTAACCCTACTAATTTCTAGAAGAATTGTAGAAAATGGAGCAG
GAACAGGATGAACAGTTTATCCCCCATTATCATCTAATATTGCCCATGGGGGAAGATCTGTGGACTTAGCAATTTTCTCT
TTACATTTAGCTGGGATTTCCTCAATTTTAGGGGCTATTAATTTCATCACTACCATTATTAATATACGAATTAATAATCT
ATCATTTGATCAAATACCTTTATTTATTTGATCAGTGGGCATTACAGCATTACTTTTATTACTTTCTTTACCAGTATTAG
CTGGAGCCATTACTATATTACTAACAGATCGAAATTTAAATACCTCCTTCTTTGATCCAGCGGGAGGGGGAGATCCTATT
CTATATCAACATTTATTTTGATTTTTT
>GBMIN30587-13|Diatraea_saccharalis|JN108965|JN108965
-------TATTTTATTTTTGGAATTTGAGCAGGAATATTAGGAACTTCATTAAGTTTATTAATTCGAGCAGAATTAGGAA
CATCTAACTCTTTAATTGGAGATGATCAAATTTATAACACAATTGTAACAGCTCATGCTTTCATTATAATTTTTTTTATA
GTTATACCTATTATAATTGGAGGATTTGGAAATTGATTGGTACCTTTAATATTAGGAGCCCCCGATATAGCTTTCCCACG
AATAAATAATATAAGATTTTGATTATTACCCCCATCTTTAACCCTACTAATTTCTAGAAGAATTGTAGAAAATGGAGCAG
GAACAGGATGAACAGTTTATCCCCCATTATCATCTAATATTGCCCATGGAGGAAGATCTGTGGACTTAGCAATTTTCTCT
TTACATTTAGCTGGGATTTCCTCAATTTTAGGGGCTATCAATTTCATCACTACCATTATTAATATACGAATTAATAATCT
ATCATTTGATCAAATACCTTTATTTATTTGATCAGTGGGWATTACAGCATTACTTTTATTACTTTCTTTACCAGTATTAG
CTGGAGCCATTACTATATTACTAACAGATCGAAATTTAAATACCTCCTTCTTTGATCCAGTGGGAGGGGGAGATCCTATT
CTATATCAACATTTATTTTGATTTTTT
>GBMIN30588-13|Diatraea_saccharalis|JN108963|JN108963
-------TATTTTATTTTTGGAATTTGAGCAGGAATATTAGGAACTTCATTAAGTTTATTAATTCGAGCAGAATTAGGAA
CATCTAACTCTTTAATTGGAGATGATCAAATTTATAACACAATTGTAACAGCTCATGCTTTCATTATAATTTTTTTTATA
GTTATACCTATTATAATTGGAGGATTTGGAAATTGATTGGTACCTTTAATATTAGGAGCCCCCGATATAGCTTTCCCACG
AATAAATAATATAAGATTTTGATTATTACCCCCATCTTTAACCCTACTAATTTCTAGAAGAATTGTAGAAAATGGAGCAG
GAACAGGATGAACAGTTTATCCCCCATTATCATCTAATATTGCCCATGGAGGAAGATCTGTGGACTTAGCAATTTTCTCT
TTACATTTAGCTGGGATTTCCTCAATTTTAGGGGCTATCAATTTCATCACTACCATTATTAATATACGAATTAATAATCT
ATCATTTGATCAAATACCTTTATTTATTTGATCAGTGGGTATTACAGCATTACTTTTATTACTTTCTTTACCAGTATTAG
CTGGAGCCATTACTATATTACTAACAGATCGAAATTTAAATACCTCCTTCTTTGATCCAGCGGGAGGGGGAGATCCTATT
CTATATCAACATTTATTTTGATTTTTT
>GBMIN30589-13|Diatraea_saccharalis|JN108961|JN108961
-------TATTTTATTTTTGGAATTTGAGCAGGAATATTAGGAACTTCATTAAGTTTATTAATTCGAGCAGAATTAGGAA
CATCTAACTCTTTAATTGGAGATGATCAAATTTATAACACAATTGTAACAGCTCATGCTTTCATTATAATTTTTTTTATA
GTTATACCTATTATAATTGGAGGATTTGGAAATTGATTGGTACCTTTAATATTAGGAGCCCCCGATATAGCTTTCCCACG
AATAAATAATATAAGATTTTGATTATTACCCCCATCTTTAACCCTACTAATTTCTAGAAGAATTGTAGAAAATGGAGCAG
GAACAGGATGAACAGTTTATCCCCCATTATCATCTAATATTGCCCATGGGGGAAGATCTGTGGACTTAGCAATTTTCTCT
TTACATTTAGCTGGGATTTCCTCAATTTTAGGGGCTATWAATTTCATCACTACCATTATTAATATACGAATTAATAATCT
ATCATTTGATCAAATACCTTTATTTATTTGATCAGTGGGCATTACAGCATTACTTTTATTACTTTCTTTACCAGTATTAG
CTGGAGCCATTACTATATTACTAACAGATCGAAATTTAAATACCTCCTTCTTTGATCCAGCGGGAGGGGGAGATCCTATT
CTATATCAACATTTATTTTGATTTTTT
>GBMIN30590-13|Diatraea_saccharalis|JN108959|JN108959
-------TATTTTATTTTTGGAATTTGAGCAGGAATATTAGGAACTTCATTAAGTTTATTAATTCGAGCAGAATTAGGAA
CATCTAACTCTTTAATTGGAGATGATCAAATTTATAACACAATTGTAACAGCTCATGCTTTCATTATAATTTTTTTTATA
GTTATACCTATTATAATTGGAGGATTTGGAAATTGATTGGTACCTTTAATATTAGGAGCCCCCGATATAGCTTTCCCACG
AATAAATAATATAAGATTTTGATTATTACCCCCATCTTTAACCCTACTAATTTCTAGAAGAATTGTAGAAAATGGAGCAG
GAACAGGATGAACAGTTTATCCCCCATTATCATCTAATATTGCCCATGGGGGAAGATCTGTGGACTTAGCAATTTTCTCT
TTACATTTAGCTGGGATTTCCTCAATTTTAGGGGCTATTAATTTCATCACTACCATTATTAATATACGAATTAATAATCT
ATCATTTGATCAAATACCTTTATTTATTTGATCAGTGGGCATTACAGCATTACTTTTATTACTTTCTTTACCAGTATTAG
CTGGAGCCATTACTATATTACTAACAGATCGAAATTTAAATACCTCCTTCTTTGATCCAGCGGGAGGGGGAGATCCTATT
CTATATCAACATTTATTTTGATTTTTT
>GBMIN30591-13|Diatraea_saccharalis|JN108957|JN108957
-------TATTTTATTTTTGGAATTTGAGCAGGAATATTAGGAACTTCATTAAGTTTATTAATTCGAGCAGAATTAGGAA
CATCTAACTCTTTAATTGGAGATGATCAAATTTATAACACAATTGTAACAGCTCATGCTTTCATTATAATTTTTTTTATA
GTTATACCTATTATAATTGGAGGATTTGGAAATTGATTGGTACCTTTAATATTAGGAGCCCCCGATATAGCTTTCCCACG
AATAAATAATATAAGATTTTGATTATTACCCCCATCTTTAACCCTACTAATTTCTAGAAGAATTGTAGAAAATGGAGCAG
GAACAGGATGAACAGTTTATCCCCCATTATCATCTAATATTGCCCATGGGGGAAGATCTGTGGACTTAGCAATTTTCTCT
TTACATTTAGCTGGGATTTCCTCAATTTTAGGGGCTATTAATTTCATCACTACCATTATTAATATACGAATTAATAATCT

ATCATTTGATCAAATACCTTTATTTATTTGATCAGTGGGCATTACAGCATTACTTTTATTACTTTCTTTACCAGTATTAG
CTGGAGCCATTACTATATTACTAACAGATCGAAATTTAAATACCTCCTTCTTTGATCCAGCGGGAGGGGGAGATCCTATT
CTATATCAACATTTATTTTGATTTTTT
>GBMIN30603-13|Diatraea_saccharalis|JN108986|JN108986
-------TATTTTATTTTTGGAATTTGAGCAGGAATATTAGGAACTTCATTAAGTTTATTAATTCGAGCAGAATTAGGAA
CATCTAACTCTTTAATTGGAGATGATCAAATTTATAACACAATTGTAACAGCTCATGCTTTCATTATAATTTTTTTATA
GTTATACCTATTATAATTGGAGGATTTGGAAATTGATTGGTACCTTTAATATTAGGAGCCCCCGATATAGCTTTCCCACG
AATAAATAATATAAGATTTTGATTATTACCCCCATCTTTAACCCTACTAATTTCTAGAAGAATTGTAGAAAATGGAGCAG
GAACAGGATGAACAGTTTATCCCCCATTATCATCTAATATTGCCCATGGGGGAAGATCTGTGGACTTAGCAATTTTCTCT
TTACATTTAGCTGGGATTTCCTCAATTTTAGGGGCTATTAATTTCATCACTACCATTATTAATATACGAATTAATAATCT
ATCATTTGATCAAATACCTTTATTTATTTGATCAGTGGGCATTACAGCATTACTTTTATTACTTTCTTTACCAGTATTAG
CTGGAGCCATTACTATATTACTAACAGATCGAAATTTAAATACCTCCTTCTTTGATCCAGCGGGAGGGGGAGATCCTATT
CTATATCAACATTTA------------
>GBMIN30604-13|Diatraea_saccharalis|JN108984|JN108984
-------TATTTTATTTTTGGAATTTGAGCAGGAATATTAGGAACTTCATTAAGTTTATTAATTCGAGCAGAATTAGGAA
CATCTAACTCTTTAATTGGAGATGATCAAATTTATAACACAATTGTAACAGCTCATGCTTTCATTATAATTTTTTTATA
GTTATACCTATTATAATTGGAGGATTTGGAAATTGATTGGTACCTTTAATATTAGGAGCCCCCGATATAGCTTTCCCACG
AATAAATAATATAAGATTTTGATTATTACCCCCATCTTTAACCCTACTAATTTCTAGAAGAATTGTAGAAAATGGAGCAG
GAACAGGATGAACAGTTTATCCCCCATTATCATCTAATATTGCCCATGGGGGAAGATCTGTGGACTTAGCAATTTTCTCT
TTACATTTAGCTGGGATTTCCTCAATTTTAGGGGCTATTAATTTCATCACTACCATTATTAATATACGAATTAATAATCT
ATCATTTGATCAAATACCTTTATTTATTTGATCAGTGGGCATTACAGCATTACTTTTATTACTTTCTTTACCAGTATTAG
CTGGAGCCATTACTATATTACTAACAGATCGAAATTTAAATACCTCCTTCTTTGATCCAGCGGGAGGGGGAGATCCTATT
CTATATCAACATTTATTTTGATTTTTT
>GBMIN30605-13|Diatraea_saccharalis|JN108982|JN108982
-------TATTTTATTTTTGGAATTTGAGCAGGAATATTAGGAACTTCATTAAGTTTATTAATTCGAGCAGAATTAGGAA
CATCTAACTCTTTAATTGGAGATGATCAAATTTATAACACAATTGTAACAGCTCATGCTTTCATTATAATTTTTTTATA
GTTATACCTATTATAATTGGAGGATTTGGAAATTGATTGGTACCTTTAATATTAGGAGCCCCCGATATAGCTTTCCCACG
AATAAATAATATAAGATTTTGATTATTACCCCCATCTTTAACCCTACTAATTTCTAGAAGAATTGTAGAAAATGGAGCAG
GAACAGGATGAACAGTTTATCCCCCATTATCATCTAATATTGCCCATGGGGGAAGATCTGTGGACTTAGCAATTTTCTCT
TTACATTTAGCTGGGATTTCCTCAATTTTAGGGGCTATTAATTTCATCACTACCATTATTAATATACGAATTAATAATCT
ATCATTTGATCAAATACCTTTATTTATTTGATCAGTGGGCATTACAGCATTACTTTTATTACTTTCTTTACCAGTATTAG
CTGGAGCCATTACTATATTACTAACAGATCGAAATTTAAATACCTCCTTCTTTGATCCAGCGGGAGGGGGAGATCCTATT
CTATATCAACATTTATTTTGATTTTTT
>GBMIN30606-13|Diatraea_saccharalis|JN108980|JN108980
-------TATTTTATTTTTGGAATTTGAGCAGGAATATTAGGAACTTCATTAAGTTTATTAATTCGAGCAGAATTAGGAA
CATCTAACTCTTTAATTGGAGATGATCAAATTTATAACACAATTGTAACAGCTCATGCTTTCATTATAATTTTTTTATA
GTTATACCTATTATAATTGGAGGATTTGGAAATTGATTGGTACCTTTAATATTAGGAGCCCCCGATATAGCTTTCCCACG
AATAAATAATATAAGATTTTGATTATTACCCCCATCTTTAACCCTACTAATTTCTAGAAGAATTGTAGAAAATGGAGCAG
GAACAGGATGAACAGTTTATCCCCCATTATCATCTAATATTGCCCATGGGGGAAGATCTGTGGACTTAGCAATTTTCTCT
TTACATTTAGCTGGGATTTCCTCAATTTTAGGGGCTATTAATTTCATCACTACCATTATTAATATACGAATTAATAATCT
ATCATTTGATCAAATACCTTTATTTATTTGATCAGTGGGCATTACAGCATTACTTTTATTACTTTCTTTACCAGTATTAG
CTGGAGCCATTACTATATTACTAACAGATCGAAATTTAAATACCTCCTTCTTTGATCCAGCGGGAGGGGGAGATCCTATT
CTATATCAACATTTATTTTGATTTTTT
>GBMIN30607-13|Diatraea_saccharalis|JN108978|JN108978
-------TATTTTATTTTTGGAATTTGAGCAGGAATATTAGGAACTTCATTAAGTTTATTAATTCGAGCAGAATTAGGAA
CATCTAACTCTTTAATTGGAGATGATCAAATTTATAACACAATTGTAACAGCTCATGCTTTCATTATAATTTTTTTATA
GTTATACCTATTATAATTGGAGGATTTGGAAATTGATTGGTACCTTTAATATTAGGAGCCCCCGATATAGCTTTCCCACG
AATAAATAATATAAGATTTTGATTATTACCCCCATCTTTAACCCTACTAATTTCTAGAAGAATTGTAGAAAATGGAGCAG
GAACAGGATGAACAGTTTATCCCCCATTATCATCTAATATTGCCCATGGGGGAAGATCTGTGGACTTAGCAATTTTCTCT
TTACATTTAGCTGGGATTTCCTCAATTTTAGGGGCTATTAATTTCATCACTACCATTATTAATATACGAATTAATAATCT
ATCATTTGATCAAATACCTTTATTTATTTGATCAGTGGGCATTACAGCATTACTTTTATTACTTTCTTTACCAGTATTAG
CTGGAGCCATTACTATATTACTAACAGATCGAAATTTAAATACCTCCTTCTTTGATCCAGCGGGAGGGGGAGATCCTATT
CTATATCAACATTTATTTTGATTTTTT
>GBMIN30608-13|Diatraea_saccharalis|JN108976|JN108976
-------TATTTTATTTTTGGAATTTGAGCAGGAATATTAGGAACTTCATTAAGTTTATTAATTCGAGCAGAATTAGGAA
CATCTAACTCTTTAATTGGAGATGATCAAATTTATAACACAATTGTAACAGCTCATGCTTTCATTATAATTTTTTTATA
GTTATACCTATTATAATTGGAGGATTTGGAAATTGATTGGTACCTTTAATATTAGGAGCCCCCGATATAGCTTTCCCACG

AATAAATAATATAAGATTTTGATTATTACCCCCATCTTTAACCCTACTAATTTCTAGAAGAATTGTAGAAAATGGAGCAG
GAACAGGATGAACAGTTTATCCCCCATTATCATCTAATATTGCCCATGGGGGAAGATCTGTGGACTTAGCAATTTTCTCT
TTACATTTAGCTGGGATTTCCTCAATTTTAGGGGCTATTAATTTCATCACTACCATTATTAATATACGAATTAATAATCT
ATCATTTGATCAAATACCTTTATTTATTTGATCAGTGGGCATTACAGCATTACTTTTATTACTTTCTTTACCAGTATTAG
CTGGAGCCATTACTATATTACTAACAGATCGAAATTTAAATACCTCCTTCTTTGATCCAGCGGGAGGGGGAGATCCTATT
CTATATCAACATTTATTTTGATTTTTT
>GBMIN30609-13|Diatraea_saccharalis|JN108974|JN108974
-------TATTTTATTTTTGGAATTTGAGCAGGAATATTAGGAACTTCATTAAGTTTATTAATTCGAGCAGAATTAGGAA
CATCTAACTCTTTAATTGGAGATGATCAAATTTATAACACAATTGTAACAGCTCATGCTTTCATTATAATTTTTTTTATA
GTTATACCTATTATAATTGGAGGATTTGGAAATTGATTGGTACCTTTAATATTAGGAGCCCCCGATATAGCTTTCCCACG
AATAAATAATATAAGATTTTGATTATTACCCCCATCTTTAACCCTACTAATTTCTAGAAGAATTGTAGAAAATGGAGCAG
GAACAGGATGAACAGTTTATCCCCCATTATCATCTAATATTGCCCATGGGGGAAGATCTGTGGACTTAGCAATTTTCTCT
TTACATTTAGCTGGGATTTCCTCAATTTTAGGGGCTATTAATTTCATCACTACCATTATTAATATACGAATTAATAATCT
ATCATTTGATCAAATACCTTTATTTATTTGATCAGTGGGCATTACAGCATTACTTTTATTACTTTCTTTACCAGTATTAG
CTGGAGCCATTACTATATTACTAACAGATCGAAATTTAAATACCTCCTTCTTTGATCCAGCGGGAGGGGGAGATCCTATT
CTATATCAACATTTATTTTGATTTTTT
>GBMIN30610-13|Diatraea_saccharalis|JN108972|JN108972
-------TATTTTATTTTTGGAATTTGAGCAGGAATATTAGGAACTTCATTAAGTTTATTAATTCGAGCAGAATTAGGAA
CATCTAACTCTTTAATTGGAGATGATCAAATTTATAACACAATTGTAACAGCTCATGCTTTCATTATAATTTTTTTTATA
GTTATACCTATTATAATTGGAGGATTTGGAAATTGATTGGTACCTTTAATATTAGGAGCCCCCGATATAGCTTTCCCACG
AATAAATAATATAAGATTTTGATTATTACCCCCATCTTTAACCCTACTAATTTCTAGAAGAATTGTAGAAAATGGAGCAG
GAACAGGATGAACAGTTTATCCCCCATTATCATCTAATATTGCCCATGGGGGAAGATCTGTGGACTTAGCAATTTTCTCT
TTACATTTAGCTGGGATTTCCTCAATTTTAGGGGCTATTAATTTCATCACTACCATTATTAATATACGAATTAATAATCT
ATCATTTGATCAAATACCTTTATTTATTTGATCAGTGGGCATTACAGCATTACTTTTATTACTTTCTTTACCAGTATTAG
CTGGAGCCATTACTATATTACTAACAGATCGAAATTTAAATACCTCCTTCTTTGATCCAGCGGGAGGGGGAGATCCTATT
CTATATCAACATTTATTTTGATTTTTT
>GBMIN30611-13|Diatraea_saccharalis|JN108970|JN108970
-------TATTTTATTTTTGGAATTTGAGCAGGAATATTAGGAACTTCATTAAGTTTATTAATTCGAGCAGAATTAGGAA
CATCTAACTCTTTAATTGGAGATGATCAAATTTATAACACAATTGTAACAGCTCATGCTTTCATTATAATTTTTTTTATA
GTTATACCTATTATAATTGGAGGATTTGGAAATTGATTGGTACCTTTAATATTAGGAGCCCCCGATATAGCTTTCCCACG
AATAAATAATATAAGATTTTGATTATTACCCCCATCTTTAACCCTACTAATTTCTAGAAGAATTGTAGAAAATGGAGCAG
GAACAGGATGAACAGTTTATCCCCCATTATCATCTAATATTGCCCATGGGGGAAGATCTGTGGACTTAGCAATTTTCTCT
TTACATTTAGCTGGGATTTCCTCAATTTTAGGGGCTATTAATTTCATCACTACCATTATTAATATACGAATTAATAATCT
ATCATTTGATCAAATACCTTTATTTATTTGATCAGTGGGCATTACAGCATTACTTTTATTACTTTCTTTACCAGTATTAG
CTGGAGCCATTACTATATTACTAACAGATCGAAATTTAAATACCTCCTTCTTTGATCCAGCGGGAGGGGGAGATCCTATT
CTATATCAACATTTATTTTGATTTTTT
>GBMIN30612-13|Diatraea_saccharalis|JN108968|JN108968
-------TATTTTATTTTTGGAATTTGAGCAGGAATATTAGGAACTTCATTAAGTTTATTAATTCGAGCAGAATTAGGAA
CATCTAACTCTTTAATTGGAGATGATCAAATTTATAACACAATTGTAACAGCTCATGCTTTCATTATAATTTTTTTTATA
GTTATACCTATTATAATTGGAGGATTTGGAAATTGATTGGTACCTTTAATATTAGGAGCCCCCGATATAGCTTTCCCACG
AATAAATAATATAAGATTTTGATTATTACCCCCATCTTTAACCCTACTAATTTCTAGAAGAATTGTAGAAAATGGAGCAG
GAACAGGATGAACAGTTTATCCCCCATTATCATCTAATATTGCCCATGGGGGAAGATCTGTGGACTTAGCAATTTTCTCT
TTACATTTAGCTGGGATTTCCTCAATTTTAGGGGCTATTAATTTCATCACTACCATTATTAATATACGAATTAATAATCT
ATCATTTGATCAAATACCTTTATTTATTTGATCAGTGGGCATTACAGCATTACTTTTATTACTTTCTTTACCAGTATTAG
CTGGAGCCATTACTATATTACTAACAGATCGAAATTTAAATACCTCCTTCTTTGATCCAGCGGGAGGGGGAGATCCTATT
CTATATCAACATTTATTTTGATTTTTT
>GBMIN30613-13|Diatraea_saccharalis|JN108966|JN108966
-------TATTTTATTTTTGGAATTTGAGCAGGAATATTAGGAACTTCATTAAGTTTATTAATTCGAGCAGAATTAGGAA
CATCTAACTCTTTAATTGGAGATGATCAAATTTATAACACAATTGTAACAGCTCATGCTTTCATTATAATTTTTTTTATA
GTTATACCTATTATAATTGGAGGATTTGGAAATTGATTGGTACCTTTAATATTAGGAGCCCCCGATATAGCTTTCCCACG
AATAAATAATATAAGATTTTGATTATTACCCCCATCTTTAACCCTACTAATTTCTAGAAGAATTGTAGAAAATGGAGCAG
GAACAGGATGAACAGTTTATCCCCCATTATCATCTAATATTGCCCATGGGGGAAGATCTGTGGACTTAGCAATTTTCTCT
TTACATTTAGCTGGGATTTCCTCAATTTTAGGGGCTATTAATTTCATCACTACCATTATTAATATACGAATTAATAATCT
ATCATTTGATCAAATACCTTTATTTATTTGATCAGTGGGCATTACAGCATTACTTTTATTACTTTCTTTACCAGTATTAG
CTGGAGCCATTACTATATTACTAACAGATCGAAATTTAAATACCTCCTTCTTTGATCCAGCGGGAGGGGGAGATCCTATT
CTATATCAACATTTATTTTGATTTTTT
>GBMIN30614-13|Diatraea_saccharalis|JN108964|JN108964

-------TATTTTATTTTTGGAATTTGAGCAGGAATATTAGGAACTTCATTAAGTTTATTAATTCGAGCAGAATTAGGAA
CATCTAACTCTTTAATTGGAGATGATCAAATTTATAACACAATTGTAACAGCTCATGCTTTCATTATAATTTTTTTTATA
GTTATACCTATTATAATTGGAGGATTTGGAAATTGATTGGTACCTTTAATATTAGGAGCCCCCGATATAGCTTTCCCACG
AATAAATAATATAAGATTTTGATTATTACCCCCATCTTTAACCCTACTAATTTCTAGAAGAATTGTAGAAAATGGAGCAG
GAACAGGATGAACAGTTTATCCCCCATTATCATCTAATATTGCCCATGGAGGAAGATCTGTGGACTTAGCAATTTTCTCT
TTACATTTAGCTGGGATTTCCTCAATTTTAGGGGCTATCAATTTCATCACTACCATTATTAATATACGAATTAATAATCT
ATCATTTGATCAAATACCTTTATTTATTTGATCAGTGGGTATTACAGCATTACTTTTATTACTTCTTTACCAGTATTAG
CTGGAGCCATTACTATATTACTAACAGATCGAAATTTAAATACCTCCTTCTTTGATCCAGCGGGAGGGGGAGATCCTATT
CTATATCAACATTTATTTTGATTTTTT
>GBMIN30615-13|Diatraea_saccharalis|JN108962|JN108962
-------TATTTTATTTTTGGAATTTGAGCAGGAATATTAGGAACTTCATTAAGTTTATTAATTCGAGCAGAATTAGGAA
CATCTAACTCTTTAATTGGAGATGATCAAATTTATAACACAATTGTAACAGCTCATGCTTTCATTATAATTTTTTTTATA
GTTATACCTATTATAATTGGAGGATTTGGAAATTGATTGGTACCTTTAATATTAGGAGCCCCCGATATAGCTTTCCCACG
AATAAATAATATAAGATTTTGATTATTACCCCCATCTTTAACCCTACTAATTTCTAGAAGAATTGTAGAAAATGGAGCAG
GAACAGGATGAACAGTTTATCCCCCATTATCATCTAATATTGCCCATGGGGGAAGATCTGTGGACTTAGCAATTTTCTCT
TTACATTTAGCTGGGATTTCCTCAATTTTAGGGGCTATTAATTTCATCACTACCATTATTAATATACGAATTAATAATCT
ATCATTTGATCAAATACCTTTATTTATTTGATCAGTGGGCATTACAGCATTACTTTTATTACTTCTTTACCAGTATTAG
CTGGAGCCATTACTATATTACTAACAGATCGAAATTTAAATACCTCCTTCTTTGATCCAGCGGGAGGGGGAGATCCTATT
CTATATCAACATTTATTTTGATTTTTT
>GBMIN30616-13|Diatraea_saccharalis|JN108960|JN108960
-------TATTTTATTTTTGGAATTTGAGCAGGAATATTAGGAACTTCATTAAGTTTATTAATTCGAGCAGAATTAGGAA
CATCTAACTCTTTAATTGGAGATGATCAAATTTATAACACAATTGTAACAGCTCATGCTTTCATTATAATTTTTTTTATA
GTTATACCTATTATAATTGGAGGATTTGGAAATTGATTGGTACCTTTAATATTAGGAGCCCCCGATATAGCTTTCCCACG
AATAAATAATATAAGATTTTGATTATTACCCCCATCTTTAACCCTACTAATTTCTAGAAGAATTGTAGAAAATGGAGCAG
GAACAGGATGAACAGTTTATCCCCCATTATCATCTAATATTGCCCATGGGGGAAGATCTGTGGACTTAGCAATTTTCTCT
TTACATTTAGCTGGGATTTCCTCAATTTTAGGGGCTATTAATTTCATCACTACCATTATTAATATACGAATTAATAATCT
ATCATTTGATCAAATACCTTTATTTATTTGATCAGTGGGCATTACAGCATTACTTTTATTACTTCTTTACCAGTATTAG
CTGGAGCCATTACTATATTACTAACAGATCGAAATTTAAATACCTCCTTCTTTGATCCAGCGGGAGGGGGAGATCCTATT
CTATATCAACATTTATTTTGATTTTTT
>GBMIN30617-13|Diatraea_saccharalis|JN108958|JN108958
-------TATTTTATTTTTGGAATTTGAGCAGGAATATTAGGAACTTCATTAAGTTTATTAATTCGAGCAGAATTAGGAA
CATCTAACTCTTTAATTGGAGATGATCAAATTTATAACACAATTGTAACAGCTCATGCTTTCATTATAATTTTTTTTATA
GTTATACCTATTATAATTGGAGGATTTGGAAATTGATTGGTACCTTTAATATTAGGAGCCCCCGATATAGCTTTCCCACG
AATAAATAATATAAGATTTTGATTATTACCCCCATCTTTAACCCTACTAATTTCTAGAAGAATTGTAGAAAATGGAGCAG
GAACAGGATGAACAGTTTATCCCCCATTATCATCTAATATTGCCCATGGGGGAAGATCTGTGGACTTAGCAATTTTCTCT
TTACATTTAGCTGGGATTTCCTCAATTTTAGGGGCTATTAATTTCATCACTACCATTATTAATATACGAATTAATAATCT
ATCATTTGATCAAATACCTTTATTTATTTGATCAGTGGGCATTACAGCATTACTTTTATTACTTCTTTACCAGTATTAG
CTGGAGCCATTACTATATTACTAACAGATCGAAATTTAAATACCTCCTTCTTTGATCCAGCGGGAGGGGGAGATCCTATT
CTATATCAACATTTATTTTGATTTTTT
>GBMIN79350-17|Chilo_sacchariphagus|KU525011|KU525011
AACTTTATATTTTATTTTTGGAATTTGAGCTGGTATAGTTGGAACATCCCTTAGACTTTTAATTCGAGCTGAATTAGGAA
ATCCAGGTTCATTAATCGGAGATGATCAAATTTATAATACTATTGTTACAGCCCATGCATTTATTATAATTTTTTTTATA
GTAATACCAATTATAATTGGAGGATTTGGAAATTGATTAGTTCCATTAATATTAGGAGCTCCTGATATAGCCTTCCCTCG
TCTAAATAATATAAGATTTTGATTATTACCTCCCTCTTTAACCCTCTTAATTTCTAGAAGAATCGTTGAAAATGGAGCAG
GAACTGGATGAACAGTCTACCCCCCCCTATCTTCCAATATTTCACATGCTGGAAGTTCAGTAGATTTAGCCATCTTCTCC
CTCCATTTAGCTGGAATTTCTTCAATTTTAGGAGCTATCAATTTCATTACTACAATTATTAATATACGAATTAATGGATT
ATTATTTGATCAAATACCATTATTTGTTTGATCTGTTGGTATTACAGCATTACTTCTCCTCCTTTCTTTGCCAGTATTAG
CAGGTGCTATTACTATACTATTAACTGATCGAAATTTAAATACATCTTTTTTTGACCCAGCTGGAGGGGGTGATCCAATT
TTATATCAACATTTATTT---------
>GBMIN83466-17|Sesamia_cretica|KU891974|KU891974
---AAATAGGTA
TTCCGGGATCTTTAATTGGAGATGATCAAATTTATAACACTATTGTTACAGCTCATGCTTTTATTATAATTTTTTTTATA
GTTATACCAATTATAATTGGAGGATTTGGTAATTGACTCGTACCTTTAATATTTGGAGCTCCAGATATTGCATTACCACG
AATAAATAATATAAGATTTTGATTATTACCCCCTTCTTTAACTTTATTAATTTCAAGAAGAATTGTAGAAAATGGGGCCG
GTACAGGATGAACTGTATATCCCCCCCTCTCATCTAATATTGCCCATGGAGGAAGATCTGTAGATTTAGCTATTTTTTCC
CTTCATTTAGCTGGTATTTCATCTATTTTAGGAGCCATTAATTTTATTACAACAATTATTAATATACGATTAAATACCTT
ATCCTTTGATCAAATACCTTTATTTGTTTGAGCTGTTGGAATTACTGCATTCTTATTCTTATTATCTTTACCTGTTTTAG

CAGGAGCTATTACAATATTATTAACAGATCGAAATTTAAATACATCATTCTTTGATCCTGCAGGAGGAGGTGATCCATTT
TTATATCAACATTTATTTTGATTTTT-
>GBMIN83468-17|Sesamia_cretica|KU891972|KU891972
---AAATAGGGA
TTCCTGGATCTTTAATTGGAGATGATCAAATTTATAATACTATTGTTACAGCTCATGCTTTTATCATAATTTTTTTATA
GTTATACCAATTATAATTGGAGGATTTGGTAATTGACTCGTACCTTTAATATTAGGAGCTCCAGATATAGCATTTCCACG
AATAAATAACATAAGATTTTGATTATTACCCCCTTCTTTAACTTTATTAATTTCAAGAAGAATTGTAGAAAATGGAGCAG
GTACAGGATGAACGGTATATCCCCCCCTCTCATCTAATATTGCTCATGGAGGAAGATCTGTAGATTTAGCTATTTTTTCC
CTTCATTTAGCGGGTATTTCATCTATTTTAGGAGCTATTAATTTTATTACAACAATTATTAATATACGATTAAATAACTT
ATCTTTTGATCAAATACCTTTATTTGTTTGAGCTGTTGGAATTACTGCATTCTTATTATTATTATCTTTACCTGTTTTAG
CAGGAGCTATTACAATATTATTAACAGATCGAAATTTAAATACATCATTCTTTGATCCTGCAGGAGGAGGTGATCCAATT
TTATATCAACATTTATTTTGATTTTT-
>GBMIN83499-17|Sesamia_nonagrioides|KU891968|KU891968
------------------TGG-ATTTGAGCTGGAATAGTAGGAACTTCATTAAGACTATTAATTCGAGCTGAATTAGGAA
CTCCTGGATCTTTAATTGGAGATGATCAAATTTATAATACTATTGTTACAGCTCATGCTTTTATTATAATTTTTTTATA
GTTATACCTATTATAATTGGAGGATTTGGAAATTGACTTGTACCTTTAATATTAGGAGCCCCAGATATAGCATTTCCACG
AATAAATAATATAAGATTTTGACTATTACCACCATCCTTAACCCTTTTAATTTCAAGTAGAATTGTAGAAAATGGGGCTG
GAACAGGATGAACAGTTTACCCCCCACTTTCATCTAACATCGCTCATGGAGGAAGATCTGTAGATTTAGCTATTTTTTCC
CTTCATTTAGCTGGAATTTCATCTATTCTAGGAGCTATTAATTTTATTACAACAATTATTAATATACGATTAAATAATTT
ATCATTTGATCAAATACCATTATTTATTTGAGCTGTTGGAATTACTGCTTTTTTATTACTATTATCATTACCCGTTTTAG
CAGGAGCTATTACTATACTACTTACGGATCGAAATTTAAATACATCATTTTTTGATCCTGCGGGAGGAGGTGATCCAATT
TTATACCAACACTTATTCTGATTTTT-
>GBMIN83513-17|Sesamia_nonagrioides|KU891971|KU891971
------------------TGG-ATTTGAGCTGGAATAGTAGGAACTTCATTAAGACTATTAATTCGAGCTGAATTAGGAA
CTCCTGGATCTTTAATTGGAGATGATCAAATTTATAATACTATTGTTACAGCTCATGCTTTTATTATAATTTTTTTATA
GTTATACCTATTATAATTGGAGGATTTGGAAATTGACTTGTACCTTTAATATTAGGAGCCCCAGATATAGCATTTCCACG
AATAAATAATATAAGATTTTGACTATTACCACCATCCTTAACCCTTTTAATTTCAAGTAGAATTGTAGAAAATGGGGCTG
GAACAGGATGAACAGTTTACCCCCCACTTTCATCTAACATCGCTCATGGAGGAAGATCTGTAGATTTAGCTATTTTTTCC
CTTCATTTAGCTGGAATTTCATCTATTCTAGGAGCTATTAATTTTATTACAACAATTATTAATATACGATTAAATAATTT
ATCATTTGATCAAATACCATTATTTATTTGAGCTGTTGGAATTACTGCTTTTTTATTACTATTATCATTACCCGTTTTAG
CAGGAGCTATTACTATACTACTTACGGATCGAAATTTAAATACATCATTTTTTGATCCTGCGGGAGGAGGTGATCCAATT
TTATACCAACACTTATTCTGATTTTT-
>GBMIN83539-17|Sesamia_nonagrioides|KU891970|KU891970
------------------TGG-ATTTGAGCTGGAATAGTAGGAACTTCATTAAGACTATTAATTCGAGCTGAATTAGGAA
CTCCTGGATCTTTAATTGGAGACGATCAAATTTATAATACTATTGTTACAGCTCATGCTTTTATTATAATTTTTTTATA
GTTATACCTATTATAATTGGAGGATTTGGAAATTGACTTGTACCTTTAATATTAGGAGCTCCAGATATAGCATTTCCACG
AATAAATAATATAAGATTTTGACTATTACCACCATCCTTAACCCTTTTAATTTCAAGTAGAATTGTAGAAAATGGGGCTG
GAACAGGATGAACAGTTTACCCCCCACTTTCATCTAACATCGCTCATGGAGGAAGATCTGTAGATTTAGCTATTTTTTCC
CTTCATTTAGCTGGAATTTCATCTATTCTAGGAGCTATTAATTTTATTACAACAATTATTAATATACGATTAAATAATTT
ATCATTTGATCAAATACCATTATTTATTTGAGCTGTTGGAATTACTGCTTTTTTATTACTATTATCATTACCCGTTTTAG
CAGGAGCTATTACTATACTACTTACGGATCGAAATTTAAATACATCATTTTTTGATCCTGCGGGAGGAGGTGATCCAATT
TTATACCAACACTTATTCTGATTTTT-
>GBMIN83544-17|Sesamia_nonagrioides|KU891967|KU891967
------------------TGG-ATTTGAGCTGGAATAGTAGGAACTTCATTAAGACTATTAATTCGAGCTGAATTAGGAA
CTCCTGGATCTTTAATTGGAGATGATCAAATTTATAATACTATTGTTACAGCTCATGCTTTTATTATAATTTTTTTATA
GTTATACCTATTATAATTGGAGGATTTGGAAATTGACTTGTACCTTTAATATTAGGAGCCCCAGATATAGCATTTCCACG
AATAAATAATATAAGATTTTGACTATTACCACCATCCTTAACCCTTTTAATTTCAAGTAGAATTGTAGAAAATGGGGCTG
GAACAGGATGAACAGTTTACCCCCCACTTTCATCTAACATCGCTCATGGAGGAAGATCTGTAGATTTAGCTATTTTTTCC
CTTCATTTAGCTGGAATTTCATCTATTCTAGGAGCTATTAATTTTATTACAACAATTATTAATATACGATTAAATAATTT
ATCATTTGATCAAATACCATTATTTATTTGAGCTGTTGGAATTACTGCTTTTTTATTACTATTATCATTACCCGTTTTAG
CAGGAGCTATTACTATACTACTTACGGATCGAAATTTAAATACATCATTTTTTGATCCTGCGGGAGGAGGTGATCCAATT
TTATACCAACACTTATTCTGATTTTT-
>GWORL633-09|Chilo_phragmitella|BC_ZSM_Lep_22915|GU686716-SUPPRESSED
AACTTTATATTTTATTTTTGGAATTTGAGCTGGAATAATTGGAACATCTCTTAGACTTTTAATTCGAGCTGAATTAGGAA
CTCCAGGATCCCTAATTGGAGATGATCAAATTTATAATACTATTGTTACAGCTCATGCATTTATTATAATTTTTTTATA
GTTATACCTATTATAATCGGTGGTTTTGGAAATTGATTAGTACCTTTAATATTAGGGGCCCCTGATATAGCTTTCCCACG
AATAAATAATATAAGATTTTGATTATTACCACCTTCATTAACCTTATTAATCTCTAGAAGAATTGTTGAAAATGGAGCTG

GAACAGGATGAACAGTGTACCCCCCACTTTCATCTAATATTGCTCATGCTGGAAGTTCAGTAGATTTAGCAATTTTTTCC
TTACATTTAGCTGGAATTTCATCAATTTTAGGTGCTATTAATTTTATTACAACAATTATTAATATACGAATTAATGGATT
ATCATTTGATCAAATACCCTTACTCATTTGAAGAATTGGTATTACAGCATTATTATTATTACTTTCTCTCCCAGTATTAG
CTGGTGCTATTACTATATTATTAACAGATCGAAATTTAAATACATCTTTTTTTGATCCAGCTGGAGGTGGAGATCCTATT
CTCTATCAACATTTATTT---------
>GWOTH542-12|Acrapex_albivena|BC_ZSM_Lep_66523|
TACATTATATTTTATTTTTGGGATTTGAGCCGGTATAGTAGGAACTTCTTTAAGATTATTAATTCGAGCTGAACTAGGAA
CTCCAGGATCTTTAATTGGAGATGATCAAATTTATAATACTATTGTTACAGCCCATGCTTTTATTATAATTTTTTTTATA
GTTATACCAATTATAATTGGGGGATTTGGAAATTGACTTGTACCTTTAATATTGGGGGCACCAGATATAGCATTTCCTCG
AATAAATAATATAAGTTTTTGATTATTACCCCCCTCTTTAACTCTTTTAATTTCCAGAAGAATCGTAGAAAATGGGGCAG
GAACTGGGTGAACTGTATACCCTCCTTTATCATCTAATATTGCCCATGGGGGAAGTTCTGTAGATTTAGCTATTTTTTCT
CTTCATTTAGCTGGGATCTCATCTATTTTAGGTGCTATTAATTTTATCACTACAATTATTAATATACGATTAAATAATTT
ATCTTTTGATCAAATACCTTTATTTATTTGAGCTGTAGGTATTACTGCATTCTTATTATTACTATCTTTACCTGTTTTAG
CAGGAGCTATTACTATGTTACTAACAGATCGAAATTTAAATACTTCTTTTTTTGACCCTGCTGGAGGGGGAGATCCTATT
TTATATCAACACTTATTT---------
>GWOTH728-12|Acrapex_aenigma|BC_ZSM_Lep_66044|
AACATTATATTTTATTTTTGGAATTTGAGCTGGTATAGTAGGAACTTCATTAAGTTTATTAATTCGAGCTGAATTAGGAA
CTCCAGGATCTTTAATTGGAGATGATCAAATTTATAATACTATTGTTACAGCTCATGCTTTTATCATAATTTTTTTTATA
GTTATACCTATTATAATTGGAGGATTTGGTAATTGACTTGTACCATTAATATTGGGAGCACCTGATATAGCATTCCCTCG
AATAAATAATATAAGTTTTTGACTACTTCCTCCTTCTTTAACCCTTTTAATTTCAAGAAGAATTGTAGAAAATGGAGCAG
GAACTGGATGAACAGTTTACCCCCCACTGTCATCTAATATCGCTCATGGAGGAAGTTCTGTAGATTTAGCTATTTTTTCT
CTTCATTTAGCTGGAATCTCTTCTATTTTAGGAGCTATTAATTTTATCACAACAATTATTAATATACGACTTAATAGTTT
ATCTTTTGATCAAATACCTTTATTTATTTGAGCTGTAGGAATCACTGCATTTTTATTATTATTATCTTTACCAGTTTTAG
CTGGAGCTATTACTATATTATTAACTGATCGAAATTTAAATACATCATTTTTTGATCCTGCTGGGGGAGGAGATCCAATT
TTATATCAACATTTATTT---------
>GWOTH742-12|Sesamia|BC_ZSM_Lep_66058|
AACATTATATTTTATTTTTGGAATTTGAGCAGGTATAGTAGGAACTTCTCTAAGATTATTAATTCGAGCTGAATTAGGAA
CCCCAGGATCTTTAATTGGAGATGATCAAATTTATAATACTATTGTCACAGCTCATGCTTTTATTATAATTTTTTTTATG
GTTATACCAATTATAATTGGAGGATTTGGAAATTGACTTGTACCCTTAATATTAGGTGCACCAGATATAGCATTTCCACG
AATAAATAATATAAGTTTTTGATTACTTCCTCCCTCTTTAACTTTACTAATTTCAAGTAGAATTGTAGAAAATGGAGCAG
GAACTGGATGAACAGTTTATCCCCCACTTTCATCTAATATTGCCCATGGAGGAAGATCAGTAGATTTAGCTATTTTTTCT
CTTCATTTAGCTGGTATTTCATCTATTTTAGGAGCTATTAACTTTATTACAACAATTATTAACATACGATTAAATAGATT
ATCTTTTGATCAAATACCTCTATTTATTTGAGCTGTTGGAATTACAGCATTTTTACTTCTTCTATCTTTACCTGTTTTAG
CAGGAGCTATTACAATATTATTAACAGATCGAAATTTAAACACATCATTTTTTGATCCAGCAGGAGGAGGTGATCCAATT
CTATATCAACACTTATTT---------
>HKONS500-08|Acrapex_relicta|3029-COI-08|
--AACTTCTTTGAGATTACTAATTCGAGCTGAATTAGGAA
ATCCTGGATCTTTAATTGGTGATGATCAAATTTATAACACTATTGTTACAGCTCATGCTTTTATTATAATTTTTTTTATA
GTTATACCTATTATAATTGGAGGATTTGGAAATTGACTTGTACCTCTAATATTAGGAGCCCCAGATATAGCATTCCCACG
AATAAATAATATAAGTTTTTGATTACTCCCTCCCTCATTAACTTTACTAATTTCAAGAAGAATTGTAGAAAATGGTGCAG
GAACAGGATGAACAGTGTACCCCCCACTTTCATCTAATATTGCTCATGGAGGAAGCTCCGTAGATTTAGCAATTTTTTCC
CTTCATTTAGCANGTATTTCTTCTATTTTAGGAGCTATTAATTTTATTACCACAATTATTAATATACGATTAAATAATTT
ATCTTTTGATCAAATACCTTTATTTATTTGAGCTGTAGGAATTACTGCATTTTTATTATTATTATCACTACCTGTTTTAG
CGGGAGCCATTACAATACTACTAACAGATCGAAATCTAAATACATCATTTTTTGAC------------------------
----
>IARI066-08|Scirpophaga_sp._6|08-IARI-0066|
AACTTTATATTTTATTTTTGGAATTTGAGCTGGTATAATTGGAACTTCTTTAAGTTTACTAATTCGAACTGAATTAGGAA
CTCCAGGTCCTTTAATTGAAGATGATCAAATTTATAATACTATTGTTACAGCTCATGCTTTTATTATAATTTTTTTTATA
GTTATACCTATTATAATTGGAGGATTTGGAAATTGATTAGTACCATTAATATTAGGAGCTCCTGATATAGCATTTCCCCG
TCTTAATAATATAAGATTTTGACTATTACCTCCATCTTTAACTATTTTAATTTCTAGAAGAATTGTAGAAAATGGGGCAG
GAACTGGATGAACTGTATACCCTCCTCTATCTTCTAATATTTCCCATAATGGAACATCTGTAGATTTAGCTATTTTTTCT
CTTCATTTAGCCGGAATCTCATCTATTTTAGGAGCTATTAATTTTATTACAACTATTATTAATATACGAATTAATGGATT
ATCATTTGATCAAATACCTTTATTTGTTTGAGCTGTAGGTATTACAGCATTACTTCTTTTATTATCTTTACCAGTTTTAG
CAGGAGCTATTACTATACTTTTAACTGATCGAAATTTAAATACCTCTTTTTTTGATCCAGCTGGAGGAGGAGATCCAATT
CTTTATCAACATTTATTT---------
>IMLQ194-07|Scirpophaga_praelata|IM07-0005|
AACTTTATATTTTATTTTTGGAATTTGAGCTGGTATAGTAGGAACCTCCTTAAGATTATTAATTCGAGCTGAATTAGGAA

CCCCAGGATCTTTAATTGGTGATGATCAAATTTATAATACTATTGTTACAGCTCATGCTTTTATTATAATTTTTTTTATG
GTAATACCAATCTTAATTGGAGGATTCGGTAACTGATTAATTCCACTAATATTAGGAGCTCCTGATATAGCCTTCCCCCG
TATAAATAATATAAGATTTTGATTACTACCCCCCTCATTAACCCTTTTAATTTCAAGAAGAGTTGTCGAAAATGGGGCAG
GAACGGGATGAACTGTATATCCCCCCCTATCTTCAAATATTGCTCATGGGGGGACTTCTGTAGATTTAGCGATTTTTTCA
CTACACCTTGCGGGAATCTCCTCTATTTTAGGAGCTATTAATTTTATCACTACAATTATTAACATACGAATTACCGGATT
ATCATTTGATCAGATACCTTTATTTGTGTGAGCTGTAGGTATTACAGCCCTTCTTTTACTCCTATCTTTACCCGTACTAG
CTGGAGCTATTACTATACTACTCACTGATCGAAACTTAAACACCTCTTTCTTCGACCCAGCAGGGGGAGGAGACCCAATC
CTCTACCAACATCTATTT---------
>IMLQ215-07|Scirpophaga_praelata|IM07-0081|
AACTTTATATTTTATTTTTGGAATTTGAGCTGGTATAGTAGGAACTTCCTTAAGATTATTAATTCGAGCTGAATTAGGAA
CCCCAGGATCTTTAATTGGTGATGATCAAATTTATAATACTATTGTTACAGCTCATGCTTTTATTATAATTTTTTTTATG
GTAATACCAATCTTAATTGGAGGATTCGGTAACTGATTAATTCCACTAATATTAGGAGCTCCTGATATAGCCTTCCCCCG
TATAAATAATATAAGATTTTGATTACTACCCCCCTCATTAACCCTTTTAATTTCAAGAAGAGTTGTCGAAAATGGGGCAG
GAACGGGATGAACTGTATATCCCCCCCTATCTTCAAATATTGCTCATGGAGGGACTTCTGTAGATTTAGCGATTTTTTCA
CTACACCTTGCGGGAATCTCCTCTATTTTAGGAGCTATTAATTTTATCACTACAATTATTAACATACGAATTACCGGATT
ATCATTTGATCAGATACCTTTATTTGTGTGAGCTGTAGGTATTACAGCCCTTCTTTTACTCCTATCTTTACCCGTACTAG
CTGGAGCTATTACTATACTACTCACTGATCGAAACTTAAACACCTCTTTCTTTGACCCAGCAGGGGGAGGAGACCCAATC
CTCTACCAACATCTATTT---------
>IMLQ814-08|Scirpophaga_praelata|IM08-0067|
AACTTTATATTTTATTTTTGGAATTTGAGCTGGTATAGTAGGAACCTCCTTAAGATTATTAATTCGAGCTGAATTAGGAA
CCCCAGGATCTTTAATTGGTGATGATCAAATTTATAATACTATTGTTACAGCTCATGCTTTTATTATAATTTTTTTTATG
GTAATACCAATCTTAATTGGAGGATTCGGTAACTGATTAATTCCACTAATATTAGGAGCTCCTGATATAGCCTTCCCCCG
TATAAATAATATAAGATTTTGATTACTACCCCCCTCATTAACCCTTTTAATTTCAAGAAGAGTTGTCGAAAATGGGGCAG
GAACGGGATGAACTGTATATCCCCCCCTATCTTCAAATATTGCTCATGGGGGGACTTCTGTAGATTTAGCGATTTTTTCA
CTACACCTTGCGGGAATCTCCTCTATTTTAGGAGCTATTAATTTTATCACTACAATTATTAACATACGAATTACCGGATT
ATCATTTGATCAGATACCTTTATTTGTGTGAGCTGTAGGTATTACAGCCCTTCTTTTACTCCTATCTTTACCCGTACTAG
CTGGAGCTATTACTATACTACTCACTGATCGAAACTTAAACACCTCTTTCTTCGACCCAGCAGGGGGAGGAGACCCAATC
CTCTACCAACATCTATTT---------
>IMLQ908-08|Scirpophaga_praelata|IM08-0260|
AACTTTATATTTTATTTTTGGAATTTGAGCTGGTATAGTAGGAACCTCCTTAAGATTATTAATTCGAGCTGAATTAGGAA
CCCCAGGATCTTTAATTGGTGATGATCAAATTTATAATACTATTGTTACAGCTCATGCTTTTATTATAATTTTTTTTATG
GTAATACCAATCTTAATTGGAGGATTCGGTAACTGATTAATTCCACTAATATTAGGAGCTCCTGATATAGCCTTCCCCCG
TATAAATAATATAAGATTTTGATTACTACCCCCCTCATTAACCCTTTTAATTTCAAGAAGAGTTGTCGAAAATGGGGCAG
GAACGGGATGAACTGTATATCCCCCCCTATCTTCAAATATTGCTCATGGGGGGACTTCTGTAGATTTAGCGATTTTTTCA
CTACACCTTGCGGGAATCTCCTCTATTTTAGGAGCTATTAATTTTATCACTACAATTATTAACATACGAATTACCGGATT
ATCATTTGATCAGATACCTTTATTTGTGTGAGCTGTAGGTATTACAGCCCTTCTTTTACTCCTATCTTTACCCGTACTAG
CTGGAGCTATTACTATACTACTCACTGATCGAAACTTAAACACCTCTTTCTTCGACCCAGCAGGGGGAGGAGACCCAATC
CTCTACCAACATCTATTT---------
>IMLR216-08|Emmalocera_latilimbella|IM07-0591|
AACTTTATATTTTATTTTTGGAATTTGATCAGGAATAGTAGGAACATCTATAAGTTTACTTATTCGAGCTGAATTAGGAA
CTCCTGGATCTTTAATTGGAGATGACCAAATTTATAATACTATTGTTACTGGTCATGCTTTTATTATAATTTTTTTTATA
GTTATACCTATTATAATTGGCGGATTTGGAAATTGATTAGTTCCTTTAATATTAGGGGCCCCAGATATAGCTTTTCCTCG
AATAAATAATATAAGATTCTGACTCTTACCCCCTTCCCTTAATTTATTAATTTTTAGAAGAATTGTAGAAAATGGAGCAG
GAACAGGATGAACAGTTTATCCCCCTTTATCCTCTAATATTGCCCATAGAGGTAGATCTGTTGATCTTGCTATTTTTTCT
TTACATTTAGCAGGAATTTCTTCTATTTTAGGAGCTATTAACTTTATTACTACTATTATTAATATAAAATTAAATGGATT
ATCATTTGATCAAATACCTTTACTTGTATGAGCTGTAGGAATTACAGCCTTATTATTACTTTTATCTTTACCAGTATTAG
CAGGAGCTATTACTATATTATTAACTGATCGAAATTTAAATACTTCTTTTTTTGACCCTGCTGGAGGAGGAGATCCAATT
TTATATCAACATTTATTT---------
>IMLR237-08|Emmalocera|IM08-0082|
AACTTTATATTTTATTTTTGGAATTTGAGCTGGAATAGTCGGAACATCTTTAAGTCTTCTTATTCGAGCTGAATTAGGAA
CCCCTGGGTCTTTAATTGGAGATGATCAAATTTATAATACTATTGTTACAGGACATGCTTTTATTATAATTTTTTTTATA
GTTATACCTATTATAATTGGAGGATTTGGAAATTGATTAGTTCCTCTTATATTAGGAGCCCCAGATATAGCTTTCCCTCG
AATAAATAATATAAGATTTTGACTTTTACCTCCCTCTCTTACTTTACTTATTTCTAGAAGAATTGTAGAAAATGGAGCAG
GAACAGGATGAACTGTCTACCCCCCTTTATCTTCTAATATTGCTCATGGAGGAAGTTCAGTAGATCTTGCTATTTTTTCC
CTACATTTAGCTGGTATTTCTTCCATTCTTGGAGCTATTAATTTTATTACTACTATTATTAATATAAAATTAAATGGCTT
ATCATTTGATCAAATACCATTATTTGTGTGAGCTGTAGGAATTACAGCTTTATTATTACTACTATCCTTACCTGTTCTTG
CTGGAGCTATTACCATATTATTAACTGATCGTAACTTAAATACTTCTTTTTTTGATCCTGCTGGAGGGGGAGACCCTATT

CTCTATCAACATTTATTT---------
>IMLR833-08|Acrapex_exsanguis|IM08-2395|
AACATTATATTTTATTTTTGGAATTTGAGCTGGTATACTAGGAACTTCTTTAAGTTTATTAATCCGAGCTGAATTAGGAA
CTCCAGAATCTTTAATTGGAGATGATCAAATTTATAATACTATTGTTACTGCTCACGCTTTTATTATAATTTTCTTTATA
GTTATACCAATTATAATTGGAGGATTTGGAAATTGACTTGTCCCATTAATACTAGGAGCTCCAGATATAGCATTTCCACG
TATAAATAATATAAGATTTTGATTATTACCTCCCTCTTTAAGTTTATTAATTTCAAGAAGAATCGTAGAAAATGGAGCAG
GAACTGGATGAACAGTATATCCTCCACTCTCATCTAATATTGCTCATAGAGGAAGATCAGTAGATTTAGCTATTTTTTCT
CTTCATTTAGCTGGTATCTCATCTATTTTAGGAGCTATTAATTTTATTACAACAATTATTAATATACGATTAAATAATTT
ATCTTTTGATCAAATACCTTTATTTGTTTGAGCTGTTGGAATTACTGCATTTTTACTATTACTCTCATTACCTGTATTAG
CCGGAGCTATTACAATATTATTAACAGATCGAAATTTAAATACATCATTTTTTGATCCTGCAGGAGGAGGTGATCCA---
----
>IMLR865-08|Acrapex_exsanguis|IM08-2476|
AACATTATATTTTATTTTTGGAATTTGAGCTGGTATACTAGGAACTTCTTTAAGTTTATTAATCCGAGCTGAATTAGGAA
CTCCAGAATCTTTAATTGGAGATGATCAAATTTATAATACTATTGTTACTGCTCACGCTTTTATTATAATTTTCTTTATA
GTTATACCAATTATAATTGGAGGATTTGGAAATTGACTTGTCCCATTAATATTAGGAGCTCCAGATATAGCATTTCCACG
TATAAATAATATAAGATTTTGATTATTACCTCCCTCTTTAAGTTTATTAATTTCAAGAAGAATTGTAGAAAATGGAGCAG
GAACTGGATGAACAGTATATCCTCCACTCTCATCTAATATTGCTCATAGAGGAAGATCAGTAGATTTAGCTATTTTTTCT
CTTCATTTAGCTGGTATTTCATCTATTTTAGGAGCTATTAATTTTATTACAACAATTATTAATATACGATTAAATAATTT
ATCTTTTGATCAAATACCTTTATTTGTTTGAGCTGTTGGAATTACTGCATTTTTACTATTACTCTCATTACCTGTATTAG
CCGGAGCTATTACAATACTATTAACAGATCGAAATTTAAATACATCATTTTTTGATCCTGCAGGAGGAGGTGATCCAATT
TTATACCAACATTTATTT---------
>IMLR877-08|Acrapex_exsanguis|IM08-2514|
AACATTATATTTTATTTTTGGAATTTGAGCTGGTATACTAGGAACTTCTTTAAGTTTATTAATCCGAGCTGAATTAGGAA
CTCCAGAATCTTTAATTGGAGATGATCAAATTTATAATACTATTGTTACTGCTCACGCTTTTATTATAATTTTCTTTATA
GTTATACCAATTATAATTGGAGGATTTGGAAATTGACTTGTCCCATTAATACTAGGAGCTCCAGATATAGCATTTCCACG
TATAAATAATATAAGATTTTGATTATTACCTCCCTCTTTAAGTTTATTAATTTCAAGAAGAATTGTAGAAAATGGAGCAG
GAACTGGATGAACAGTATATCCTCCACTCTCATCTAATATTGCTCATAGAGGAAGATCAGTAGATTTAGCTATTTTTTCT
CTTCATTTAGCTGGTATTTCATCTATTTTAGGAGCTATTAATTTTATTACAACAATTATTAATATACGATTAAATAATTT
ATCTTTTGATCAAATACCTTTATTTGTTTGAGCTGTTGGAATTACTGCATTTTTACTATTACTCTCATTACCTGTATTAG
CCGGAGCTATTACAATATTATTAACAGATCGAAATTTAAATACATCATTTTTTGATCCTGCAGGAGGAGGTGATCCAATT
TTATATCAACATTTATTT---------
>IMLR995-11|Acrapex_exsanguis|IM08-2164|
AACATTATATTTTATTTTTGGAATTTGAGCTGGTATACTAGGAACTTCTTTAAGTTTATTAATCCGAGCTGAATTAGGAA
CTCCAGAATCTTTAATTGGAGATAGTCAAATTTATAATACTATTGTTACTGCTCACGCTTTTATTATAATTTTTTTTATA
GTTATACCAATTATAATTGGAGGATTTGGGAATTGACTTGTCCCATTAATATTAGGAGCTCCAGATATAGCATTTCCACG
TATAAATAATATAAGATTTTGATTATTACCCCCCTCTTTAAGTTTATTAATTTCAAGAAGAATTGTAGAAAATGGAGTAG
GAACTGGATGAACAGTATATCCTCCACTCTCATCTAATATTGCTCATAGAGGAAGATCAGTAGACTTAGCTATTTTTTCT
CTTCATTTAGCTGGTATTTCATCTATTTTAGGAGCTATTAATTTTATTACAACAATTATTAATATACGATTAAATAATTT
ATCTTTTGATCAAATACCTTTATTTGTTTGAGCTGTTGGAATTACTGCATTTTTATTATTAATCTCATTACCTGTATTAG
CCGGAGCTATTACAATATTATTAACAGATCGAAATTTAAATACATCATTTTTTGATCCTGCAGGAGGAGGTGATCCAATT
TTATATCAACATTTATTT---------
>IMLS041-12|Scirpophaga_imparellus|IM11-0033|
AACTTTATATTTTATTTTTGGTATTTGAGCTGGAATAGTGGGGACTTCTTTAAGTTTATTAATTCGAGCTGAATTAGGAA
CACCAGGATCATTAATTGGGGATGATCAAATTTATAATACTATTGTTACAGCCCATGCTTTTATTATAATTTTTTTTATA
GTAATACCTATTATAATTGGGGGATTCGGAAATTGACTAGTTCCCCTAATATTAGGAGCCCCTGATATAGCTTTTCCACG
TATAAATAATATAAGTTTTTGATTATTACCTCCCTCTCTTACTCTTTTAATTTCAAGAAGAATTGTTGAAAATGGAGCAG
GAACAGGATGAACTGTTTACCCCCCTTTATCCTCTAATATTGCTCATGGAGGAACATCTGTAGATCTAGCTATTTTTTCC
TTACATTTAGCGGGAATTTCATCTATTTTAGGAGCTATTAATTTTATTACAACTATTATTAATATACGAATTAATGGATT
ATCTTTTGATCAAATACCTTTATTTGTCTGAGCTGTAGGTATTACAGCACTTCTTTTACTTTTATCTTTACCTGTGTTAG
CTGGAGCTATTACTATACTACTAACAGATCGAAATTTAAATACATCTTTTTTCGACCCAGCAGGTGGGGGAGACCCAATT
CTTTATCAACATTTATTT---------
>IMLS069-12|Scirpophaga_nivella|IM11-0066|
AACTTTATATTTCATTTTTGGAATCTGAGCTGGTATAGTAGGAACTTCTTTAAGATTATTAATTCGAGCTGAATTAGGAA
CTCCAGGATCTTTAATTGGAGATGATCAAATTTATAATACCATTGTTACAGCTCATGCTTTTATTATAATTTTTTTTATA
GTAATACCAATTATAATTGGAGGATTTGGAAATTGACTTGTTCCTTTAATATTAGGAGCTCCTGATATAGCTTTCCCCCG
TATAAATAATATAAGATTTTGATTATTACCCCCCTCATTAACTCTACTAATTTCAAGAAGAATTGTAGAAAATGGTGCAG
GAACAGGATGAACAGTATACCCTCCCCTATCATCAAATATTGCCCATGGGGGAACTTCTGTAGATTTAGCTATTTTTTCT

TTACATCTTGCAGGAATTTCCTCTATTTTAGGAGCTATTAACTTTATTACCACTATTATTAATATACGAATTAATGGCTT
AACATTTGATCAAATACCCCTCTTTGTTTGAGCTGTTGGAATTACAGCCCTTCTTTTACTCCTCTCATTACCTGTATTAG
CTGGAGCTATTACTATATTATTAACTGATCGAAATTTAAATACCTCTTTTTTTGATCCAGCAGGAGGAGGAGATCCAATC
CTTTATCAACATTTATTT---------
>IMLS194-12|Scirpophaga_nivella|IM11-0198|
AACTTTATATTTCATTTTTGGAATTTGAGCTGGTATAGTAGGAACTTCTTTAAGATTATTAATTCGAGCTGAATTAGGAA
CTCCAGGATCTTTAATTGGAGATGATCAAATTTATAATACCATTGTTACAGCTCATGCTTTTATTATAATTTTTTTTATA
GTAATACCAATTATAATTGGAGGGTTTGGAAATTGACTTGTTCCTTTAATATTAGGAGCTCCTGATATAGCTTTCCCTCG
TATAAATAATATAAGATTTTGATTATTACCCCCCTCATTAACTCTCCTAATTTCAAGAAGAATTGTAGAAAATGGTGCAG
GAACAGGATGAACAGTATACCCCCCCTTATCATCAAATATTGCTCATGGAGGAACTTCTGTAGATTTAGCTATTTTTTCT
CTACATCTTGCAGGAATTTCCTCTATTTTAGGAGCTATTAACTTTATTACCACTATTATTAATATACGAATTAATGGCTT
AACATTTGATCAAATACCTCTCTTTGTTTGAGCTGTTGGAATTACAGCCCTTCTTTTACTCCTCTCATTACCCGTATTAG
CTGGAGCTATTACTATATTATTAACTGATCGAAATTTAAATACCTCTTTTTTTGATCCAGCGGGAGGAGGAGATCCAATC
CTTTATCAACATTTATTT---------
>IMLS200-12|Emmalocera_sp._2|IM11-0205|
AACTTTATATTTTATTTTTGGAATTTGAGCTGGAATAGTCGGAACATCTTTAAGTCTTCTTATTCGAGCTGAATTAGGAA
CCCCTGGGTCTTTAATTGGAGATGATCAAATTTATAATACTATTGTTACAGGACATGCTTTTATTATAATTTTTTTTATA
GTTATACCTATTATAATTGGAGGATTTGGAAATTGATTAGTTCCTCTTATATTAGGAGCTCCAGATATAGCTTTCCCTCG
AATAAATAATATAAGATTTTGACTTTTACCTCCCTCTCTTACTTTACTTATTTCTAGAAGAATTGTAGAAAATGGAGCAG
GAACAGGATGAACTGTCTACCCCCCTTTATCTTCTAATATTGCTCATGGAGGAAGTTCAGTAGATCTTGCTATTTTTTCC
CTACATTTAGCTGGTATTTCTTCCATTCTTGGAGCTATTAATTTTATTACTACTATTATTAATATAAAATTAAATGGCTT
ATCATTTGATCAAATACCATTATTTGTGTGAGCTGTAGGAATTACAGCTTTATTATTACTACTATCCTTACCTGTTCTTG
CTGGAGCAATTACCATATTATTAACTGATCGTAACTTAAATACTTCTTTTTTTGATCCTGCTGGAGGGGGAGACCCTATT
CTCTATCAACATTTATTT---------
>IMLS215-12|Emmalocera_sp._2|IM11-0220|
AACTTTATATTTTATTTTTGGAATTTGAGCTGGAATAGTCGGAACATCTTTAAGTCTTCTTATTCGAGCTGAATTAGGAA
CCCCTGGATCTTTAATTGGAGATGATCAAATTTATAATACTATTGTTACAGGACATGCTTTTATTATAATTTTTTTTATA
GTTATACCTATTATAATTGGAGGATTTGGAAATTGATTAGTTCCTCTTATATTAGGAGCTCCAGATATAGCTTTCCCTCG
AATAAATAATATAAGATTTTGACTTTTACCTCCCTCTCTTACTTTACTTATTTCTAGAAGAATTGTAGAAAATGGAGCAG
GAACAGGATGAACTGTCTACCCCCCTTTATCTTCTAATATTGCTCATAGAGGAAGTTCAGTAGATCTTGCTATTTTTTCC
CTACATTTAGCTGGTATTTCTTCCATTCTTGGAGCTATTAATTTTATTACTACTATTATTAATATAAAATTAAATGGCTT
ATCATTTGATCAAATACCATTATTTGTGTGAGCTGTGGGAATTACAGCTTTATTATTACTACTATCCTTACCTGTTCTTG
CTGGAGCTATTACTATATTATTAACTGATCGTAACTTAAATACTTCTTTTTTTGATCCTGCTGGAGGGGGAGACCCTATT
CTCTATCAACATTTATTT---------
>IMLS291-12|Scirpophaga_nivella|IM11-0304|
AACTTTATATTTCATTTTTGGAATCTGAGCTGGTATAGTAGGAACTTCTTTAAGATTATTAATTCGAGCTGAATTAGGAA
CTCCAGGATCTTTAATTGGAGATGATCAAATTTATAATACCATTGTTACAGCTCATGCTTTTATTATAATTTTTTTTATA
GTAATACCAATTATAATTGGAGGATTTGGAAATTGACTTGTTCCTTTAATATTAGGAGCTCCTGATATAGCTTTCCCCCG
TATAAATAATATAAGATTTTGATTATTACCCCCCTCATTAACTCTACTAATTTCAAGAAGAATTGTAGAAAATGGTGCAG
GAACAGGATGAACAGTATACCCTCCCCTATCATCAAATATTGCCCATGGGGGAACTTCTGTAGATTTAGCTATTTTTTCT
TTACATCTTGCAGGAATTTCCTCTATTTTAGGAGCTATTAACTTTATTACCACTATTATTAATATACGAATTAATGGCTT
AACATTTGATCAAATACCCCTCTTTGTTTGAGCTGTTGGAATTACAGCCCTTCTTTTACTCCTCTCATTACCTGTATTAG
CTGGAGCTATTACTATATTATTAACTGATCGAAATTTAAATACCTCTTTTTTTGATCCAGCAGGAGGAGGAGATCCAATC
CTTTATCAACATTTATTT---------
>IMLS315-12|Emmalocera_sp._2|IM11-0328|
AACTTTATATTTTATTTTTGGAATTTGAGCTGGAATAGTCGGAACATCTTTAAGTCTTCTTATTCGAGCTGAATTAGGAA
CCCCTGGGTCTTTAATTGGAGATGATCAAATTTATAATACTATTGTTACAGGACATGCTTTTATTATAATTTTTTTTATA
GTTATACCTATTATAATTGGAGGATTTGGAAATTGATTAGTTCCTCTTATATTAGGAGCCCCAGATATAGCTTTCCCTCG
AATAAATAATATAAGATTTTGACTTTTACCCCCCTCTCTTACTTTACTTATTTCTAGAAGAATTGTAGAAAATGGAGCAG
GAACAGGATGAACTGTCTACCCCCCTTTATCTTCTAATATTGCTCATGGAGGAAGTTCAGTAGATCTTGCTATTTTTTCC
CTACATTTAGCTGGTATTTCTTCCATTCTTGGAGCTATTAATTTTATTACTACTATTATTAATATAAAATTAAATGGCTT
ATCATTTGATCAAATACCATTATTTGTGTGAGCTGTAGGAATTACAGCTTTATTATTACTACTATCCTTACCTGTTCTTG
CTGGAGCTATTACCATATTATTAACTGATCGTAACTTAAATACTTCTTTTTTTGATCCTGCTGGAGGGGGAGACCCTATT
CTCTATCAACATTTATTT---------
>IMLS329-12|Emmalocera_sp._2|IM11-0342|
AACTTTATATTTTATTTTTGGAATTTGAGCTGGAATAGTCGGAACATCTTTAAGTCTTCTTATTCGAGCTGAATTAGGAA
CCCCTGGGTCTTTAATTGGAGATGATCAAATTTATAATACTATTGTTACAGGACATGCTTTTATTATAATTTTTTTTATA

GTTATACCTATTATAATTGGAGGATTTGGAAATTGATTAGTTCCTCTTATATTAGGAGCTCCAGATATAGCTTTCCCTCG
AATAAATAATATAAGATTTTGACTTTTACCTCCCTCTCTTACTTTACTTATTTCTAGAAGAATTGTAGAAAATGGAGCAG
GAACAGGATGAACTGTCTACCCCCCTTTATCTTCTAATATTGCTCATGGAGGAAGTTCAGTAGATCTTGCTATTTTTTCC
CTACATTTAGCTGGTATTTCTTCCATTCTTGGAGCTATTAATTTTATTACTACTATTATTAATATAAAATTAAATGGCTT
ATCATTTGATCAAATACCATTATTTGTGTGAGCTGTAGGAATTACAGCTTTATTATTACTACTATCCTTACCTGTTCTTG
CTGGAGCTATTACCATATTATTAACTGATCGTAACTTAAATACTTCTTTTTTTGATCCTGCTGGAGGGGGAGACCCTATT
CTCTATCAACATTTATTT---------
>IMLS330-12|Emmalocera_sp._2|IM11-0343|
AACTTTATATTTTATTTTTGGAATTTGAGCTGGAATAGTCGGAACATCTTTAAGTCTTCTTATTCGAGCTGAATTAGGAA
CCCCTGGGTCTTTAATTGGAGATGATCAAATTTATAATACTATTGTTACAGGACATGCTTTTATTATAATTTTTTTTATA
GTTATACCTATTATAATTGGAGGATTTGGAAATTGATTAGTTCCTCTTATATTAGGAGCTCCAGATATAGCTTTCCCTCG
AATAAATAATATAAGATTTTGACTTTTACCTCCCTCTCTTACTTTACTTATTTCTAGAAGAATTGTAGAAAATGGAGCAG
GAACAGGATGAACTGTCTACCCCCCTTTATCTTCTAATATTGCTCATGGAGGAAGTTCAGTAGATCTTGCTATTTTTTCC
CTACATTTAGCTGGTATTTCTTCCATTCTTGGAGCTATTAATTTTATTACTACTATTATTAATATAAAATTAAATGGCTT
ATCATTTGATCAAATACCATTATTTGTGTGAGCTGTAGGAATTACAGCTTTATTATTACTACTATCCTTACCTGTTCTTG
CTGGAGCTATTACCATATTATTAACTGATCGTAACTTAAATACTTCTTTTTTTGATCCTGCTGGAGGGGGAGACCCTATT
CTCTATCAACATTTATTT---------
>IMLS342-12|Emmalocera_sp._2|IM11-0355|
AACTTTATATTTTATTTTTGGAATTTGAGCTGGAATAGTCGGAACATCTTTAAGTCTTCTTATTCGAGCTGAATTAGGAA
CCCCTGGATCTTTAATTGGAGATGATCAAATTTATAATACTATTGTTACAGGACATGCTTTTATTATAATTTTTTTTATA
GTTATACCTATTATAATTGGAGGATTTGGAAATTGATTAGTTCCTCTTATATTAGGAGCTCCAGATATAGCTTTCCCTCG
AATAAATAATATAAGATTTTGACTTTTACCTCCCTCTCTTACTTTACTTATTTCTAGAAGAATTGTAGAAAATGGAGCAG
GAACAGGATGAACTGTCTACCCCCCTTTATCTTCTAATATTGCTCATGGAGGAAGTTCAGTAGATCTTGCTATTTTTTCC
CTACATTTAGCTGGTATTTCTTCCATTCTTGGAGCTATTAATTTTATTACTACTATTATTAATATAAAATTAAATGGCTT
ATCATTTGATCAAATACCATTATTTGTGTGAGCTGTGGGAATTACAGCTTTATTATTACTACTATCCTTACCTGTTCTTG
CTGGAGCTATTACTATATTATTAACTGATCGTAACTTAAATACTTCTTTTTTTGATCCTGCTGGAGGGGGAGACCCTATT
CTCTATCAACATTTATTT---------
>IMLS343-12|Emmalocera_sp._2|IM11-0356|
AACTTTATATTTTATTTTTGGAATTTGAGCTGGAATAGTCGGAACATCTTTAAGTCTTCTTATTCGAGCTGAATTAGGAA
CCCCTGGGTCTTTAATTGGAGATGATCAAATTTATAATACTATTGTTACAGGACATGCTTTTATTATAATTTTTTTTATA
GTTATACCTATTATAATTGGAGGATTTGGAAATTGATTAGTTCCTCTTATATTAGGAGCTCCAGATATAGCTTTCCCTCG
AATAAATAATATAAGATTTTGACTTTTACCTCCCTCTCTTACTTTACTTATTTCTAGAAGAATTGTAGAAAATGGGGCAG
GAACAGGATGAACTGTCTACCCCCCTTTATCTTCTAATATTGCTCATAGAGGAAGTTCAGTAGATCTTGCTATTTTTTCC
CTACATTTAGCTGGTATTTCTTCCATTCTTGGAGCTATTAATTTTATTACTACTATTATTAATATAAAATTAAATGGCTT
ATCATTTGATCAAATACCATTATTTGTGTGAGCTGTAGGAATTACAGCTTTATTATTACTACTATCCTTACCTGTTCTTG
CTGGAGCTATTACCATATTATTAACTGATCGTAACTTAAATACTTCTTTTTTTGATCCTGCTGGAGGGGGAGACCCTATT
CTCTATCAACATTTATTT---------
>IMLS348-12|Scirpophaga_nivella|IM12-0001|
AACTTTATATTTCATTTTTGGAATTTGAGCTGGTATAGTAGGAACTTCTTTAAGATTATTAATTCGAGCTGAATTAGGAA
CTCCAGGATCTTTAATTGGAGATGATCAAATTTATAATACCATTGTTACAGCTCATGCTTTTATTATAATTTTTTTTATA
GTAATACCAATTATAATTGGAGGGTTTGGAAATTGACTTGTTCCTTTAATATTAGGAGCTCCTGATATAGCTTTCCCTCG
TATAAATAATATAAGATTTTGATTATTACCCCCCTCATTAACTCTCCTAATTTCAAGAAGAATTGTAGAAAATGGTGCAG
GAACAGGATGAACAGTATACCCCCCCTTATCATCAAATATTGCTCATGGAGGAACTTCTGTAGATTTAGCTATTTTTTCT
CTACATCTTGCAGGAATTTCCTCTATTTTAGGAGCTATTAACTTTATTACCACTATTATTAATATACGAATTAATGGCTT
AACATTTGATCAAATACCTCTCTTTGTTTGAGCTGTTGGAATTACAGCCCTTCTTTTACTCCTCTCATTACCCGTATTAG
CTGGAGCTATTACTATATTATTAACTGATCGAAATTTAAATACCTCTTTTTTTGATCCAGCGGGAGGAGGAGATCCAATC
CTTTATCAACATTTATTT---------
>IMLS388-12|Acrapex_exsanguis|IM12-0041|
AACATTATATTTTATTTTTGGAATTTGAGCTGGTATATTAGGAACTTCTTTAAGTTTATTAATCCGAGCTGAATTAGGAA
CTCCAGAATCTTTAATTGGAGATGATCAAATTTATAATACTATTGTTACTGCTCACGCTTTTATTATAATTTTTTTTATA
GTTATACCAATTATAATTGGAGGATTTGGAAATTGACTTGTCCCATTAATATTAGGAGCTCCAGATATAGCATTTCCACG
TATAAATAATATAAGATTTTGATTATTACCTCCCTCTTTAAGTTTATTAATTTCAAGAAGAATTGTAGAGAATGGAGCAG
GAACTGGATGAACAGTATATCCCCCACTCTCATCTAATATTGCTCATAGAGGAAGATCAGTGGACTTAGCTATTTTTTCT
CTTCATTTAGCTGGTATTTCATCCATTTTAGGAGCTATTAATTTTATTACAACAATTATTAATATACGATTAAATAATTT
ATCTTTTGATCAAATACCTTTATTTATTTGAGCTGTTGGAATTACTGCATTTTTATTATTACTCTCATTACCTGTATTAG
CCGGAGCTATTACAATATTATTAACAGATCGAAATTTAAATACATCATTTTTTGATCCTGCAGGAGGAGGTGATCCAATT
TTATATCAACATTTATTT---------

>IMLS449-12|Emmalocera_sp._2|IM12-0103|
AACTTTATATTTTATTTTTGGAATTTGAGCTGGAATAGTCGGAACATCTTTAAGTCTTCTTATTCGAGCTGAATTAGGAA
CCCCTGGGTCTTTAATTGGAGATGATCAAATTTATAATACTATTGTTACAGGACATGCTTTTATTATAATTTTTTTTATA
GTTATACCTATTATAATTGGAGGATTTGGAAATTGATTAGTTCCTCTTATATTAGGAGCTCCAGATATAGCTTTCCCTCG
AATAAATAATATAAGATTTTGACTTTTACCTCCCTCTCTTACTTTACTTATTTCTAGAAGAATTGTAGAAAATGGAGCAG
GAACAGGATGAACTGTCTACCCCCCTTTATCTTCTAATATTGCTCATGGAGGAAGTTCAGTAGATCTTGCTATTTTTTCC
CTACATTTAGCTGGTATTTCTTCCATTCTTGGAGCTATTAATTTTATTACTACTATTATTAATATAAAATTAAATGGCTT
ATCATTTGATCAAATACCATTATTTGTGTGAGCTGTAGGAATTACAGCTTTATTATTACTACTATCCTTACCTGTTCTTG
CTGGAGCAATTACCATATTATTAACTGATCGTAACTTAAATACTTCTTTTTTTGATCCTGCTGGAGGGGGAGACCCTATT
CTCTATCAACATTTATTT---------
>IMLS470-12|Acrapex_albicostata|IM12-0124|
TACATTATATTTTATTTTTGGAATTTGGGCAGGTATAGTAGGAACCTCTTTAAGATTATTAATTCGGGCTGAATTAGGAA
CCCCAGGATCTTTAATTGGAGATGATCAAATTTATAATACTATTGTTACAGCTCATGCTTTTATTATAATTTTCTTTATA
GTTATACCTATTATGATTGGGGGATTTGGAAATTGACTTGTCCCTTTAATATTAGGTGCTCCAGATATAGCATTTCCACG
AATAAATAATATAAGTTTTTGATTACTACCCCCCTCTTTAACTCTTCTTATTTCCAGTAGAATTGTAGAAAATGGAGCAG
GAACTGGATGAACTGTATACCCACCCCTTTCATCTAATATTGCCCATGGGGGAAGATCTGTAGATTTAGCTATTTTTTCT
TTACATTTAGCTGGAATTTCTTCTATTTTAGGAGCTATTAACTTTATTACTACAATTATTAATATACGATTAAATAGTTT
ATCCTTTGATCAAATACCTTTATTTATTTGAGCTGTAGGAATTACTGCATTTTTATTATTACTTTCTTTACCTGTATTAG
CTGGGGCTATTACAATACTATTAACAGATCGAAACTTAAATACCTCTTTTTTTGACCCTGCTGGAGGTGGAGATCCAATT
TTATATCAACATTTATTT---------
>IMLS482-12|Acrapex_albicostata|IM12-0136|
TACATTATATTTTATTTTTGGAATTTGGGCAGGTATAGTAGGAACCTCTTTAAGATTATTAATTCGGGCTGAATTAGGAA
CCCCAGGATCTTTAATTGGAGATGATCAAATTTATAATACTATTGTTACAGCTCATGCTTTTATTATAATTTTCTTTATA
GTTATACCTATTATGATTGGAGGATTTGGAAATTGACTTGTCCCTTTAATATTAGGTGCTCCAGATATAGCATTTCCACG
AATAAATAATATAAGTTTTTGATTACTACCCCCCTCTTTAACCCTTCTTATTTCCAGTAGAATTGTAGAAAATGGAGCAG
GAACTGGATGAACTGTATACCCACCCCTTTCATCTAATATTGCCCATGGGGGAAGATCTGTAGATTTAGCTATTTTTTCT
TTACATTTAGCTGGAATTTCTTCTATTTTAGGAGCTATTAACTTTATTACTACAATTATTAATATACGATTAAATAGTTT
ATCCTTTGATCAAATACCTTTATTTATTTGAGCTGTAGGAATTACTGCATTTTTATTATTACTTTCTTTACCTGTATTAG
CTGGGGCTATTACAATACTATTAACAGATCGAAACTTAAATACCTCTTTTTTTGACCCTGCTGGAGGTGGAGATCCAATC
TTATATCAACATTTATTT---------
>IMLS505-12|Acrapex_exsanguis|IM12-0160|
AACATTATATTTTATTTTTGGAATTTGAGCTGGTATACTAGGAACTTCTTTAAGTTTATTAATCCGAGCTGAATTAGGAA
CTCCAGAATCTTTAATTGGAGATGATCAAATTTATAATACTATTGTTACTGCTCACGCTTTTATTATAATTTTCTTTATA
GTTATACCAATTATAATTGGAGGATTTGGAAATTGACTTGTCCCATTAATATTAGGAGCTCCAGATATAGCATTTCCACG
TATAAATAATATAAGATTTTGATTATTACCTCCCTCTTTAAGTTTATTAATTTCAAGAAGAATTGTAGAAAATGGAGCAG
GAACTGGATGAACAGTATATCCTCCACTCTCATCTAATATTGCTCATAGAGGAAGATCAGTAGATTTAGCTATTTTTTCT
CTTCATTTAGCTGGTATTTCATCTATTTTAGGAGCTATTAATTTTATTACAACAATTATTAATATACGATTAAATAATTT
ATCTTTTGATCAAATACCTTTATTTGTTTGAGCTGTTGGAATTACTGCATTTTTACTATTACTCTCATTACCTGTATTAG
CCGGAGCTATTACAATACTATTAACAGATCGAAATTTAAATACATCATTTTTTGATCCTGCAGGAGGAGGTGATCCAATT
TTATACCAACATTTATTT---------
>IMLS529-12|Scirpophaga_nivella|IM12-0188|
AACTTTATATTTCATTTTTGGAATTTGAGCTGGTATAGTAGGAACTTCTTTAAGATTATTAATTCGAGCTGAATTAGGAA
CTCCAGGATCTTTAATTGGAGATGATCAAATTTATAATACCATTGTTACAGCTCATGCTTTTATTATAATTTTTTTTATA
GTAATACCAATTATAATTGGAGGGTTTGGAAATTGACTTGTTCCTTTAATATTAGGAGCTCCTGATATAGCTTTCCCTCG
TATAAATAATATAAGATTTTGATTATTACCCCCCTCATTAACTCTCCTAATTTCAAGAAGAATTGTAGAAAATGGTGCAG
GAACAGGATGAACAGTATACCCCCCCTTATCATCAAATATTGCTCATGGAGGAACTTCTGTAGATTTAGCTATTTTTTCT
CTACATCTTGCAGGAATTTCCTCTATTTTAGGAGCTATTAACTTTATTACCACTATTATTAATATACGAATTAATGGCTT
AACATTTGATCAAATACCTCTCTTTGTTTGAGCTGTTGGAATTACAGCCCTTCTTTTACTCCTCTCATTACCCGTATTAG
CTGGAGCTATTACTATATTATTAACTGATCGAAATTTAAATACCTCTTTTTTTGATCCAGCGGGAGGAGGAGATCCAATC
CTTTATCAACATTTATTT---------
>IMLS567-12|Emmalocera_sp._2|IM12-0229|
AACTTTATATTTTATTTTTGGAATTTGAGCTGGAATAGTCGGAACATCTTTAAGTCTTCTTATTCGAGCTGAATTAGGAA
CCCCTGGGTCTTTAATTGGAGATGATCAAATTTATAATACTATTGTTACAGGACATGCTTTTATTATAATTTTTTTTATA
GTTATACCTATTATAATTGGAGGATTTGGAAATTGATTAGTTCCTCTTATATTAGGAGCTCCAGATATAGCTTTCCCTCG
AATAAATAATATAAGATTTTGACTTTTACCTCCCTCTCTTACTTTACTTATTTCTAGAAGAATTGTAGAAAATGGAGCAG
GAACAGGATGAACTGTCTACCCCCCTTTATCTTCTAATATTGCTCATGGAGGAAGTTCAGTAGATCTTGCTATTTTTTCC
CTACATTTAGCTGGTATTTCTTCCATTCTTGGAGCTATTAATTTTATTACTACTATTATTAATATAAAATTAAATGGCTT

ATCATTTGATCAAATACCATTATTTGTGTGAGCTGTAGGAATTACAGCTTTATTATTACTACTATCCTTACCTGTTCTTG
CTGGAGCTATTACCATATTATTAACTGATCGTAACTTAAATACTTCTTTTTTTGATCCTGCTGGAGGGGGAGACCCTATT
CTCTATCAACATTTATTT---------
>IMLS586-12|Scirpophaga_praelata|IM12-0249|
AACTTTATATTTTATTTTTGGAATTTGAGCTGGTATAGTAGGAACTTCCTTAAGATTATTAATTCGAGCTGAATTAGGAA
CCCCAGGATCTTTAATTGGTGATGATCAAATTTATAATACTATTGTTACAGCTCATGCTTTTATTATAATTTTTTTTATG
GTAATACCAATCTTAATTGGAGGATTCGGTAACTGATTAATTCCACTAATATTAGGAGCTCCTGATATAGCCTTCCCCCG
TATAAATAATATAAGATTTTGATTACTACCCCCCTCATTAACCCTTTTAATTTCAAGAAGAGTTGTCGAAAATGGGGCAG
GAACGGGATGAACTGTATATCCCCCCCTATCTTCAAATATTGCTCATGGGGGGACTTCTGTAGATTTAGCGATTTTTTCA
CTACACCTTGCGGGAATCTCCTCTATTTTAGGAGCTATTAATTTTATCACTACAATTATTAACATACGAATTACCGGATT
ATCATTTGATCAGATACCTTTATTTGTGTGAGCTGTAGGTATTACAGCCCTTCTTTTACTCCTATCTTTACCCGTACTAG
CTGGAGCTATTACTATACTACTCACTGATCGAAACTTAAACACCTCTTTCTTTGACCCAGCAGGGGGAGGAGACCCAATC
CTCTACCAACATCTATTT---------
>IMLS598-12|Emmalocera_latilimbella|IM12-0261|
AACTTTATATTTTATTTTTGGAATTTGATCAGGAATAGTAGGAACATCTTTAAGTTTACTTATTCGAGCTGAATTAGGAA
CTCCTAGATCTTTAATTGGAGATGATCAAATTTATAATACTATTGTTACCGGTCATGCTTTCATTATAATTTTTTTTATA
GTTATACCTATTATAATTGGTGGATTTGGAAATTGATTAGTTCCTTTAATATTAGGAGCTCCAGATATAGCTTTCCCTCG
AATAAATAATATAAGATTTTGACTTTTACCTCCTTCTCTTAATTTATTAATTTCTAGAAGAATTGTAGAAAATGGAGCAG
GAACAGGATGAACGGTATATCCTCCTTTATCTTCTAATATTGCTCATAGAGGAAGATCTGTTGATCTTGCTATTTTTTCC
TTACATTTAGCTGGAATTTCTTCTATTTTAGGAGCTATTAATTTTATTACTACTATTATTAATATAAAACTAAATGGATT
ATCATTTGATCAAATACCTTTATTCGTATGAGCTGTAGGAATTACAGCTTTATTATTACTTTTATCTTTACCAGTATTAG
CAGGAGCTATTACTATATTATTAACTGATCGAAATTTAAATACTTCTTTTTTTGACCCTGCTGGAGGAGGAGATCCAATT
TTATATCAACATTTATTT---------
>KSLEP107-15|Eoreuma_densella|BIOUG20646-A12|KR940704
AACATTATATTTTATTTTTGGGATTTGAGCTGGAATAGTAGGAANNTCTCTTAGTTTATTAATTCGAGCTGAATTAGGAA
ATCCTGGTTCCTTAATTGGAGATGATCAAATTTATAATACTATTGTCACAGCACATGCATTTATTATAATTTTTTTTATA
GTTATACCTATTATAATTGGAGGATTTGGAAATTGATTAGTTCCTTTAATATTAGGGGCCCCTGATATGGCATTCCCCCG
AATAAATAATATAAGATTTTGATTATTGCCCCCCTCATTAACTTTATTGATTTCAAGTAGTATTGTCGAAAATGGTGCCG
GTACAGGATGAACTGTGTATCCCCCTCTTTCTTCAAATATTGCCCATGGGGGGAGATCTGTTGACTTAGCAATTTTTTCT
TTACATTTAGCTGGAATCTCATCAATTTTAGGAGCTATTAACTTTATTACAACAATTATTAATATACGAATTAATAATTT
ATCATTTGATCAGATACCTTTATTTGTTTGATCAGTAGGAATTACAGCATTACTTCTCCTCCTTTCATTGCCTGTTTTAG
CAGGAGCTATTACTATATTATTAACAGATCGAAATCTTAATACATCATTTT-----------------------------
----
>KSLEP108-15|Eoreuma_densella|BIOUG20646-B01|KR937553
AACATTATATTTTATTTTTGGGATTTGAGCTGGAATAGTAGGAACATCTCTTAGTTTATTAATTCGAGCTGAATTAGGAA
ATCCTGGTTCCTTAATTGGAGATGATCAAATTTATAATACTATTGTCACAGCACATGCATTTATTATAATTTTTTTTATA
GTTATACCTATTATAATTGGAGGATTTGGAAATTGATTAGTTCCTTTAATATTAGGGGCCCCTGATATGGCATTCCCCCG
AATAAATAATATAAGATTTTGATTATTGCCCCCCTCATTAACTTTATTGATTTCAAGTAGTATTGTCGAAAATGGTGCCG
GTACAGGATGAACTGTGTATCCCCCTCTTTCTTCAAATATTGCCCATGGGGGGAGATCTGTTGACTTAGCAATTTTTTCT
TTACATTTAGCTGGAATCTCATCAATTTTAGGAGCTATTAACTTTATTACAACAATTATTAATATACGAATTAATAATTT
ATCATTTGATCAGATACCTTTATTTGTTTGATCAGTAGGAATTACAGCATTACTTCTCCTCCTTTCATTGCCTGTTTTAG
CAGGAGCTATTACTATATTATTAACAGATCGAAATCTTAATACATCATTTTT----------------------------
----
>LEATB027-13|Chilo_phragmitella|TLMF_Lep_10204|
AACTTTATATTTTATTTTTGGAATTTGAGCTGGAATAATTGGAACATCTCTTAGACTTTTAATTCGAGCTGAATTAGGAA
CTCCAGGATCCCTAATTGGAGATGATCAAATTTATAATACTATTGTTACAGCTCATGCATTTATTATAATTTTTTTTATA
GTTATACCTATTATAATCGGTGGTTTTGGAAATTGATTAGTACCTTTAATATTAGGAGCCCCTGATATAGCTTTCCCACG
AATAAATAATATAAGATTTTGATTATTACCACCTTCATTAACCTTATTAATCTCTAGAAGAATTGTTGAAAATGGAGCTG
GAACAGGATGAACAGTGTACCCCCCACTTTCATCTAATATTGCTCATGCTGGAAGTTCAGTAGATTTAGCAATTTTTTCC
TTACATTTAGCTGGAATTTCATCAATTTTAGGTGCTATTAATTTTATTACAACAATTATTAATATACGAATTAATGGATT
ATCATTTGATCAAATACCCTTACTCATTTGAAGAATTGGTATTACAGCATTATTATTATTACTTTCTCTCCCAGTATTAG
CTGGTGCTATTACTATATTATTAACAGATCGAAATTTAAATACATCTTTTTTTGATCCAGCTGGAGGTGGAGATCCTATT
CTCTATCAACATTTATTT---------
>LEATH491-14|Chilo_phragmitella|TLMF_Lep_15703|
AACTTTATATTTTATTTTTGGAATTTGAGCTGGAATAATTGGAACATCTCTTAGACTTTTAATTCGAGCTGAATTAGGAA
CTCCAGGATCCCTAATTGGAGATGATCAAATTTATAATACTATTGTTACAGCTCATGCATTTATTATAATTTTTTTTATA
GTTATACCTATTATAATCGGTGGTTTTGGAAATTGATTAGTACCTTTAATATTAGGAGCCCCTGATATAGCTTTCCCACG

AATAAATAATATAAGATTTTGATTATTACCACCTTCATTAACCTTATTAATCTCTAGAAGAATTGTTGAAAATGGAGCTG
GAACAGGATGAACAGTGTACCCCCCACTTTCATCTAATATTGCTCATGCTGGAAGTTCAGTAGATTTAGCAATTTTTTCC
TTACATTTAGCTGGAATTTCATCAATTTTAGGTGCTATTAATTTTATTACAACAATTATTAATATACGAATTAATGGATT
ATCATTTGATCAAATACCCTTACTCATTTGAAGAATTGGTATTACAGCATTATTATTATTACTTTCTCTCCCAGTATTAG
CTGGTGCTATTACTATATTATTAACAGATCGAAATTTAAATACATCTTTTTTTGATCCAGCTGGAGGTGGAGATCCTATT
CTCTATCAACATTTATTT---------
>LEATH492-14|Chilo_phragmitella|TLMF_Lep_15704|
AACTTTATATTTTATTTTTGGAATTTGAGCTGGAATAATTGGAACATCTCTTAGACTTTTAATTCGAGCTGAATTAGGAA
CTCCAGGATCCCTAATTGGAGATGATCAAATTTATAATACTATTGTTACAGCTCATGCATTTATTATAATTTTTTTTATA
GTTATACCTATTATAATCGGTGGTTTTGGAAATTGATTAGTACCTTTAATATTAGGAGCCCCTGATATAGCTTTCCCACG
AATAAATAATATAAGATTTTGATTATTACCACCTTCATTAACCTTATTAATCTCTAGAAGAATTGTTGAAAATGGAGCTG
GAACAGGATGAACAGTGTACCCCCCACTTTCATCTAATATTGCTCATGCTGGAAGTTCAGTAGATTTAGCAATTTTTTCC
TTACATTTAGCTGGAATTTCATCAATTTTAGGTGCTATTAATTTTATTACAACAATTATTAATATACGAATTAATGGATT
ATCATTTGATCAAATACCCTTACTCATTTGAAGAATTGGTATTACAGCATTATTATTATTACTTTCTCTCCCAGTATTAG
CTGGTGCTATTACTATATTATTAACAGATCGAAATTTAAATACATCTTTTTTTGATCCAGCTGGAGGTGGAGATCCTATT
CTCTATCAACATTTATTT---------
>LEFIA873-10|Chilo_phragmitella|MM09750|HM387011-SUPPRESSED
AACTTTATATTTTATTTTTGGAATTTGAGCTGGAATAATTGGAACATCTCTTAGACTTTTAATTCGAGCTGAATTAGGAA
CTCCAGGATCACTAATTGGAGATGATCAAATTTATAATACTATTGTTACAGCTCATGCATTTATTATAATTTTTTTTATA
GTTATACCTATTATAATTGGTGGTTTTGGAAATTGATTAGTACCTTTAATATTAGGAGCCCCTGATATAGCTTTCCCACG
AATAAATAATATAAGATTTTGATTATTACCACCTTCATTAACCTTATTAATCTCTAGAAGAATTGTTGAAAATGGAGCTG
GAACAGGATGAACAGTGTACCCCCCACTCTCATCTAATATTGCTCATGCTGGAAGTTCAGTAGATTTAGCAATTTTTTCC
TTACATTTAGCTGGAATTTCATCAATTTTAGGTGCTATTAATTTTATTACAACAATTATTAATATACGAATTAATGGATT
ATCATTTGATCAAATACCCCTACTCATTTGAAGAATTGGCATTACAGCATTATTATTATTACTTTCTCTTCCAGTATTAG
CTGGTGCTATTACTATATTATTAACAGATCGAAATTTAAATACATCTTTTTTTGATCCAGCTGGAGGTGGAGATCCTATT
CTCTATCAACATTTATTT---------
>LEFIB667-10|Chilo_phragmitella|MM02448|HM871545-SUPPRESSED
AACTTTATATTTTATTTTTGGAATTTGAGCTGGAATAATTGGAACATCCCTTAGACTTTTAATTCGAGCTGAATTAGGAA
CTCCAGGATCACTAATTGGAGATGATCAAATTTATAATACTATTGTTACAGCTCATGCATTTATTATAATTTTTTTTATA
GTTATACCTATTATAATTGGTGGTTTTGGAAATTGATTAGTACCTTTAATATTAGGAGCTCCTGATATAGCTTTCCCACG
AATAAATAATATAAGATTTTGATTATTACCCCCTTCATTAACCTTACTAATTTCTAGAAGAATTGTTGAAAATGGAGCCG
GAACAGGATGAACAGTATACCCCCCACTTTCATCAAATATTGCTCATGCTGGAAGTTCAGTAGATTTAGCAATTTTTTCA
TTACATTTAGCTGGAATTTCATCAATTTTAGGTGCTATTAATTTTATTACAACAATTATTAATATACGAATTAATGGATT
ATCATTTGATCAAATACCATTACTTATTTGAAGAATTGGTATTACAGCCTTATTATTATTACTTTCCCTTCCAGTATTAG
CTGGTGCTATTACTATATTATTAACAGATCGAAATTTAAATACATCCTTTTTTGATCCAGCTGGAGGTGGAGATCCTATT
CTTTATCAACATTTATTT---------
>LEFIF770-10|Chilo_phragmitella|MM12991|HM875454-SUPPRESSED
AACTTTATATTTTATTTTTGGGATTTGAGCTGGAATAATTGGAACATCCCTTAGACTTTTAATTCGAGCTGAATTAGGAA
CTCCAGGATCACTAATTGGAGATGATCAAATTTATAATACTATTGTTACAGCTCATGCATTTATTATAATTTTTTTTATA
GTTATACCTATTATAATTGGTGGTTTTGGAAATTGATTAGTACCTTTAATATTAGGAGCTCCTGATATAGCTTTCCCACG
AATAAATAATATAAGATTTTGATTATTACCCCCTTCATTAACCTTACTAATTTCTAGAAGAATTGTTGAAAATGGAGCCG
GAACAGGATGAACAGTATACCCCCCACTTTCATCAAATATTGCTCATGCTGGAAGTTCAGTAGATTTAGCAATTTTTTCA
TTACATTTAGCTGGAATTTCATCAATTTTAGGTGCTATTAATTTTATTACAACAATTATTAATATACGAATTAATGGATT
ATCATTTGATCAAATACCATTACTTATTTGAAGAATTGGTATTACAGCCTTATTATTATTACTTTCCCTTCCAGTATTAG
CTGGTGCCATTACTATATTATTAACAGATCGAAATTTAAATACATCCTTTTTTGATCCAGCTGGAGGTGGAGATCCTATT
CTTTATCAACATTTATTT---------
>LEFIG259-10|Chilo_phragmitella|MM14190|HM875938-SUPPRESSED
AACTTTATATTTTATTTTTGGGATTTGAGCTGGAATAATTGGAACATCCCTTAGACTTTTAATTCGAGCTGAATTAGGAA
CTCCAGGATCACTAATTGGAGATGATCAAATTTATAATACTATTGTTACAGCTCATGCATTTATTATAATTTTTTTTATA
GTTATACCTATTATAATTGGTGGTTTTGGAAATTGATTAGTACCTTTAATATTAGGAGCTCCTGATATAGCTTTCCCACG
AATAAATAATATAAGATTTTGATTATTACCCCCTTCATTAACCTTACTAATTTCTAGAAGAATTGTTGAAAATGGAGCCG
GAACAGGATGAACAGTATACCCCCCACTTTCATCAAATATTGCTCATGCTGGAAGTTCAGTAGATTTAGCAATTTTTTCA
TTACATTTAGCTGGAATTTCATCAATTTTAGGTGCTATTAATTTTATTACAACAATTATTAATATACGAATTAATGGATT
ATCATTTGATCAAATACCATTACTTATTTGAAGAATTGGTATTACAGCCTTATTATTATTACTTTCCCTTCCAGTATTAG
CTGGTGCTATTACTATATTATTAACAGATCGAAATTTAAATACATCCTTTTTTGATCCAGCTGGAGGTGGAGATCCTATT
CTTTATCAACATTTATTT---------
>LEPIN013-12|Chilo_partellus|Cs_L1|

AACTTTATATTTTATTTTTGGAATTTGAGCAGGAATAATTGGAACATCCCTTAGATTATTAATTCGTGCAGAATTAGGAA
CTCCTGGATCTTTAATTGGAGATGATCAAATTTATAATACTATTGTAACAGCACATGCATTTATTATAATTTTTTTTATA
GTTATACCAATTATAATTGGTGGATTTGGAAATTGATTAGTACCTTTAATATTAGGAGCCCCAGATATAGCTTTCCCACG
AATAAATAATATAAGATTTTGATTATTACCACCATCATTAACTTTACTAATTTCTAGAAGAATTGTTGAAAATGGAGCTG
GAACAGGATGAACAGTGTACCCCCCACTATCATCTAATATTGCTCATGCTGGAAGTTCAGTAGATTTAGCAATTTTTTCT
TTACATTTAGCTGGTATTTCATCAATTCTTGGTGCTATTAATTTTATTACAACAATCATTAATATACGAATTAATGGATT
ATTTTTTGATCAAATACCATTATTTGTTTGATCTGTAGGTATTACAGCTTTATTATTATTACTTTCTTTACCTGTTTTAG
CTGGAGCTATTACTGTATTATTAACAGATCGAAATTTAAATACATCCTTTTTCGATCCTGCTGGAGGAGGAGATCCTATT
CTTTATCAACACTTATTT---------
>LEPIN045-13|Chilo_auricilius|Cs_L1-104|
AACTTTATACTTTATTTTTGGAATTTGAAGTGGAATAATTGGAACATCTCTAAGACTTTTAATTCGTGCTGAATTAGGAA
CTCCAGGGTCATTAATTGGAGATGATCAAATTTACAATACTATTGTTACAGCTCATGCATTTATTATAATTTTTTTTATA
GTTATACCAATTATAATTGGAGGCTTTGGTAATTGATTAGTACCATTAATACTAGGGGCTCCTGATATAGCCTTCCCTCG
AATAAATAATATAAGATTTTGATTATTGCCCCCATCATTAACATTATTAATTTCTAGAAGAATTGTAGAAAATGGAGCTG
GAACAGGATGAACGGTTTACCCCCCCCTTTCATCAAATATTGCCCATGGAGGAAGTTCTGTAGATTTAGCTATTTTTTCT
CTTCATTTAGCTGGTATTTCCTCAATTTTAGGAGCTATTAATTTTATTACAACAATTATTAATATACGAATTAATAAACT
ATCATTTGATCAATTACCATTATTTGTTTGATCTGTTGGTATTACAGCTTTATTATTACTACTTTCATTGCCGGTACTAG
CCGGAGCTATTACTATACTTTTAACTGATCGAAACTTAAATACATCTTTTTTTGACCCTGCTGGAGGAGGAGACCCAATT
CTTTATCAACATTTATTT---------
>LEPIN048-14|Chilo_partellus|Cp_L1-182|
AACTTTATATTTTATTTTTGGAATTTGAGCAGGAATAATTGGAACATCCCTTAGATTATTAATTCGTGCAGAATTAGGAA
CTCCTGGATCTTTAATTGGAGATGATCAAATTTATAATACTATTGTAACAGCACATGCATTTATTATAATTTTTTTTATA
GTTATACCAATTATAATTGGTGGATTTGGAAATTGATTAGTACCTTTAATATTAGGAGCCCCAGATATAGCTTTCCCACG
AATAAATAATATAAGATTTTGATTATTACCACCATCATTAACTTTACTAATTTCTAGAAGAATTGTTGAAAATGGAGCTG
GAACAGGATGAACAGTGTACCCCCCACTATCATCTAATATTGCTCATGCTGGAAGTTCAGTAGATTTAGCAATTTTTTCT
TTACATTTAGCTGGTATTTCATCAATTCTTGGTGCTATTAATTTTATTACAACAATCATTAATATACGAATTAATGGATT
ATTTTTTGATCAAATACCATTATTTGTTTGATCTGTAGGTATTACAGCTTTATTATTATTACTTTCTTTACCTGTTTTAG
CTGGAGCTATTACTATATTATTAACAGATCGAAATTTAAATACATCCTTTTTCGATCCTGCTGGAGGAGGAGATCCTATT
CTTTATCAACACTTATTT---------
>LEPIN053-14|Chilo_partellus|Cp_G1-189|
AACTTTATATTTTATTTTTGGAATTTGAGCAGGAATAATTGGAACATCCCTTAGATTATTAATTCGTGCAGAATTAGGAA
CTCCTGGATCTTTAATTGGAGATGATCAAATTTATAATACTATTGTAACAGCACATGCATTTATTATAATTTTTTTTATA
GTTATACCAATTATAATTGGTGGATTTGGAAATTGATTAGTACCTTTAATATTAGGAGCCCCAGATATAGCTTTCCCACG
AATAAATAATATAAGATTTTGATTATTACCACCATCATTAACTTTACTAATTTCTAGAAGAATTGTTGAAAATGGAGCTG
GAACAGGATGAACAGTGTACCCCCCACTATCATCTAATATTGCTCATGCTGGAAGTTCAGTAGATTTAGCAATTTTTTCT
TTACATTTAGCTGGTATTTCATCAATTCTTGGTGCTATTAATTTTATTACAACAATCATTAATATACGAATTAATGGATT
ATTTTTTGATCAAATACCATTATTTGTTTGATCTGTAGGTATTACAGCTTTATTATTATTACTTTCTTTACCTGTTTTAG
CTGGAGCTATTACTATATTATTAACAGATCGAAATTTAAATACATCCTTTTTCGATCCTGCTGGAGGAGGAGATCCTATT
CTTCATCAACACTTATTT---------
>LEPMY1406-15|Sesamia_submarginalis|BIOUG21481-B11|
AACATTATATTTTATTTTTGGAATTTGAGCCGGAATAGTTGGAACTTCTTTAAGATTATTAATTCGAGCTGAATTAGGAA
CTCCTGGATCTCTAATTGGTGATGATCAAATTTATAATACTATTGTAACAGCCCATGCCTTCATTATAATTTTTTTTATA
GTTATACCAATTATAATTGGGGGGTTTGGAAATTGACTTGTACCTTTAATATTGGGAGCCCCTGATATAGCTTTTCCACG
AATAAATAATATGAGTTTTTGATTACTTCCCCCCTCTTTAACTCTCCTTATCTCAAGAAGAGTTGTAGAAAATGGAGCAG
GAACTGGATGAACTGTTTATCCCCCACTCTCATCTAATATTGCCCATGGGGGGGGATCGGTAGATTTAGCCATCTTTTCC
CTTCATTTAGCTGGTATTTCTTCTATTTTAGGGGCTATTAATTTTATTACAACAATTATTAATATACGATTAAATAATTT
ATCTTTTGATCAAATACCTTTATTTATTTGAGCTGTAGGAATTACAGCATTTTTATTATTATTATCATTACCTGTGTTAG
CTGGAGCTATTACCATATTATTAACAGATCGAAATTTAAATACATCATTTTTTGATCCGGCGGGAGGAGGAGATCCAATT
TTATATCAACATTTATTT---------
>LGSMB084-04|Diatraea_evanescens|DNA-ATBI-0933|GU089417
AACTTTATATTTTATTTTTGGAATTTGAGCTGGAATACTGGGAACATCTTTAAGTTTATTAATTCGTGCAGAATTAGGTA
CAACTAATTCTTTAATTGGAGATGATCAAATCTACAATACAATTGTTACAGCTCATGCATTTATTATAATTTTTTTTATA
GTTATACCCATTATAATTGGAGGATTTGGAAACTGATTAGTACCATTAATATTAGGAGCTCCTGATATAGCTTTCCCTCG
AATAAATAATATAAGATTTTGATTACTACCCCCATCTTTAACTTTATTAATTTCTAGAAGAATTGTTGAAAATGGAGCTG
GAACAGGATGAACAGTTTACCCCCCACTTTCATCAAATATTGCTCATGGTGGTAGATCTGTAGACCTAGCAATTTTTTCA
TTACATTTAGCTGGAATCTCATCAATTTTAGGAGCTATTAATTTTATTACCACAATTATTAATATACGAATTAATGGATT
ATCATTCGATCAAATACCTCTATTTGTATGATCAGTAGGTATTACAGCCTTATTATTACTTTTATCTTTACCTGTTTTAG

CTGGAGCTATTACCATATTATTAACAGATCGAAACTTAAATACTTCCTTTTTCGACCCAGCTGGAGGGGGAGATCCAATT
TTATATCAACATTTATTT---------
>LGSMB085-04|Diatraea_evanescens|DNA-ATBI-0934|GU089416
AACTTTATATTTTATTTTTGGAATTTGAGCTGGAATACTGGGAACATCTTTAAGTTTATTAATTCGTGCAGAATTAGGTA
CAACTAATTCTTTAATTGGAGATGATCAAATCTACAATACAATTGTTACAGCTCATGCATTTATTATAATTTTTTTTATA
GTTATACCCATTATAATTGGAGGATTTGGAAACTGATTAGTACCATTAATATTAGGAGCTCCTGATATAGCTTTCCCTCG
AATAAATAATATAAGATTTTGATTACTACCCCCATCTTTAACTTTATTAATTTCTAGAAGAATTGTTGAAAATGGAGCTG
GAACAGGATGAACAGTTTACCCCCCACTTTCATCAAATATTGCTCATGGTGGTAGATCTGTAGACCTAGCAATTTTTTCA
TTACATTTAGCTGGAATCTCATCAATTTTAGGAGCTATTAATTTTATTACCACAATTATTAATATACGAATTAATGGATT
ATCATTCGATCAAATACCTCTATTTGTATGATCAGTAGGTATTACAGCCTTATTATTACTTTTATCTTTACCTGTTTTAG
CTGGAGCTATTACCATATTATTAACAGATCGAAACTTAAATACTTCCTTTTTCGACCCAGCTGGAGGGGGAGATCCAATT
TTATATCAACATTTATTT---------
>LMDH170-11|Chilo_plejadellus|BIOUG01047-E12|
AACCTTATATTTTATTTTTGGAATTTGAGCAGGAATAATTGGAACATCTCTTAGACTTTTAATCCGTGCTGAACTAGGAA
CTCCTGGATTTTTAATTGGAGATGATCAAATTTATAATACTATTGTAACAGCTCATGCATTTATTATAATTTTTTTTATA
GTTATACCTATCATAATTGGTGGATTTGGAAATTGATTAGTACCTTTAATATTAGGAGCTCCTGATATAGCTTTTCCACG
AATAAATAATATAAGATTCTGAATATTACCCCCCTCATTAACTTTACTAATTTCTAGTAGTATTGTTGAAAATGGAGCTG
GAACAGGATGAACGGTGTACCCCCCACTATCATCTAATATTGCCCATGCTGGAAGTTCAGTAGATTTAGCAATTTTTTCC
CTACATTTAGCTGGAATTTCTTCAATTTTAGGAGCTATTAATTTTATTACCACAATTATTAATATACGAATTAATGGATT
ATCATTTGATCAAATACCTTTATTTGTTTGATCTGTAGGTATTACAGCCTTATTACTCCTACTCTCACTACCGGTATTAG
CTGGTGCTATTACGATATTACTAACTGACCGAAATTTAAATACATCTTTCTTCGATCCAGCTGGTGGGGGGGATCCAATT
CTTTATCAACATTTATTT---------
>LNAUU2071-15|Chilo_demotellus|CCDB-28955-G04|
AACATTATATTTTATTTTTGGTATTTGAGCAGGAATAATTGGAACATCCCTTAGACTTTTAATTCGAGCAGAATTAGGAA
TGCCTGGATCTTTAATTGGAGATGATCAAATTTATAATACTATTGTTACAGCCCATGCATTTATTATAATTTTTTTCATA
GTTATACCAATTATAATTGGTGGATTTGGAAATTGATTAGTACCTTTAATATTAGGAGCACCAGATATAGCTTTCCCACG
AATAAATAATATAAGATTTTGAATATTACCCCCGTCATTAACTCTTCTAATTTCTAGAAGAATTGTTGAAAATGGAGCTG
GAACAGGATGAACAGTGTACCCCCCACTGTCATCTAATATTGCTCATGCTGGAAGTTCTGTAGATTTAGCAATTTTTTCT
TTACATTTAGCTGGAATTTCTTCTATTTTAGGTGCTATTAATTTTATTACTACAATTATTAATATACGAATTAATGGACT
TTCATTTGATCAAATACCATTATTTGTTTGATCTGTAGGTATTACAGCTTTATTATTATTACTTTCTTTACCTGTATTAG
CAGGTGCTATTACAATATTATTAACTGATCGAAATTTAAATACATCTTTTTTTGACCCAGCTGGAGGTGGAGATCCAATC
CTTTATCAACACTTATTT---------
>LNAUU2072-15|Chilo_demotellus|CCDB-28955-G05|
AACATTATATTTTATTTTTGGTATTTGAGCAGGAATAATTGGAACATCCCTTAGACTTTTAATTCGAGCAGAATTAGGAA
TGCCTGGATCTTTAATTGGAGATGATCAAATTTATAATACTATTGTTACAGCCCATGCATTTATTATAATTTTTTTCATA
GTTATACCAATTATAATTGGTGGATTTGGAAATTGATTAGTACCTTTAATATTAGGAGCACCAGATATAGCTTTCCCACG
AATAAATAATATAAGATTTTGAATATTACCCCCGTCATTAACTCTTCTAATTTCTAGAAGAATTGTTGAAAATGGAGCTG
GAACAGGATGAACAGTGTACCCCCCACTGTCATCTAATATTGCTCATGCTGGAAGTTCTGTAGATTTAGCAATTTTTTCT
TTACATTTAGCTGGAATTTCTTCTATTTTAGGTGCTATTAATTTTATTACTACAATTATTAATATACGAATTAATGGACT
TTCATTTGATCAAATACCATTATTTGTTTGATCTGTAGGTATTACAGCTTTATTATTATTACTTTCTTTACCTGTATTAG
CAGGTGCTATTACAATATTATTAACTGATCGAAATTTAAATACATCTTTTTTTGACCCAGCTGGAGGTGGAGATCCAATC
CTTTATCAACACTTATTT---------
>LNAUU2073-15|Diatraea_lineolata|CCDB-28955-G06|
AACTTTATATTTTATATTTGGAATTTGAGCAGGAATACTAGGAACATCTTTAAGTTTATTAATTCGAGCTGAACTTGGAA
CACCCAATTCTTTAATTGGCGATGATCAAATTTATAACACAATTGTTACAGCTCATGCATTTATTATAATTTTTTTTATG
GTTATACCTATTATAATTGGGGGATTTGGAAATTGATTAGTACCCCTTATATTAGGAGCCCCAGATATGGCTTTCCCCCG
AATAAATAACATAAGATTCTGATTATTACCCCCATCTTTAACCCTATTAATTTCTAGAAGAATTGTTGAAAATGGGGCTG
GAACAGGATGAACAGTATACCCCCCATTATCATCTAATATTGCTCATGGGGGAAGATCTGTGGATTTAGCTATTTTTTCA
CTGCATTTAGCTGGGATTTCATCAATCTTAGGGGCCATTAATTTTATTACTACAATTATCAATATGCGAATTAATGGTTT
ATCATTTGATCAAATACCCCTATTCGTGTGATCTGTTGGTATTACAGCACTATTATTATTACTTTCTTTACCAGTATTGG
CAGGGGCTATTACTATACTATTAACTGATCGAAATTTAAATACCTCTTTTTTCGACCCAGCGGGAGGGGGAGATCCCATT
CTTTACCAACATCTTTTC---------
>LNAUU2074-15|Diatraea_lineolata|CCDB-28955-G07|
AACTTTATATTTTATATTTGGAATTTGAGCAGGAATACTAGGAACATCTTTAAGTTTATTAATTCGAGCTGAACTTGGAA
CACCCAATTCTTTAATTGGCGATGATCAAATTTATAACACAATTGTTACAGCTCATGCATTTATTATAATTTTTTTTATG
GTTATACCTATTATAATTGGGGGATTTGGAAATTGATTAGTACCCCTTATATTAGGAGCCCCAGATATGGCTTTCCCCCG
AATAAATAACATAAGATTCTGATTATTACCCCCATCTTTAACCCTATTAATTTCTAGAAGAATTGTTGAAAATGGGGCTG

GAACAGGATGAACAGTATACCCCCCATTATCATCTAATATTGCTCATGGGGGAAGATCTGTGGATTTAGCTATTTTTTCA
CTGCATTTAGCTGGGATTTCATCAATCTTAGGGGCCATTAATTTTATTACTACAATTATCAATATGCGAATTAATGGTTT
ATCATTTGATCAAATACCCCTATTCGTGTGATCTGTTGGTATTACAGCACTATTATTATTACTTTCTTTACCAGTATTGG
CAGGGGCTATTACTATACTATTAACTGATCGAAATTTAAATACCTCTTTTTCGACCCAGCGGGAGGGGGAGATCCCATT
CTTTACCAACATCTTTTC---------
>LNCB283-06|Acrapex_relicta|06-NCCC-1239|
AACATTATATTTTATTTTTGGAATTTGAGCAGGTATGGTAGGAACTTCTTTGAGATTACTAATTCGAGCTGAATTAGGAA
ATCCTGGATCTTTAATTGGTGATGATCAAATTTATAATACTATTGTTACAGCCCATGCTTTTATTATAATTTTTTTTATA
GTTATACCTATTATAATTGGAGGATTTGGAAATTGACTTGTACCTCTAATATTAGGAGCCCCAGATATAGCATTCCCACG
AATAAATAATATAAGTTTTTGGTTACTCCCTCCCTCATTAACTTTACTAATTTCAAGAAGAATTGTAGAAAATGGTGCAG
GAACAGGATGAACAGTGTACCCCCCACTTTCATCTAATATTGCTCATGGAGGAAGCTCCGTAGATTTAGCAATTTTTTCC
CTTCATTTAGCAGGTATTTCTTCTATTTTAGGAGCTATTAATTTTATTACCACAATTATTAATATACGATTAAATAATTT
ATCTTTTGATCAAATACCTTTATTTATTTGAGCTGTAGGAATTACTGCATTTTTATTATTATTATCACTACCTGTTTTAG
CAGGAGCCATTACAATACTACTAACAGATCGAAATCTAAATACATCATTTTTTGACCCAGCAGGAGGGGGAGATCCAATT
TTATATCAACATTTATTT---------
>LNCB284-06|Acrapex_relicta|06-NCCC-1240|
AACATTATATTTTATTTTTGGAATTTGAGCAGGTATGGTAGGAACTTCTTTGAGATTACTAATTCGAGCTGAATTAGGAA
ATCCTGGATCTTTAATTGGTGATGATCAAATTTATAATACTATTGTTACAGCCCATGCTTTTATTATAATTTTTTTTATA
GTTATACCTATTATAATTGGAGGATTTGGAAATTGACTTGTACCTCTAATATTAGGAGCCCCAGATATAGCATTCCCACG
AATAAATAATATAAGTTTTTGGTTACTCCCTCCCTCATTAACTTTACTAATTTCAAGAAGAATTGTAGAAAATGGTGCAG
GAACAGGATGAACAGTGTACCCCCCACTTTCATCTAATATTGCTCATGGAGGAAGCTCCGTAGATTTAGCAATTTTTTCC
CTTCATTTAGCAGGTATTTCTTCTATTTTAGGAGCTATTAATTTTATTACCACAATTATTAATATACGATTAAATAATTT
ATCTTTTGATCAAATACCTTTATTTATTTGAGCTGTAGGAATTACTGCATTTTTATTATTATTATCACTACCTGTTTTAG
CAGGAGCCATTACAATACTACTAACAGATCGAAATCTAAATACATCATTTTTTGACCCAGCAGGAGGGGGAGATCCAATT
TTATATCAACATTTATTT---------
>LNCB285-06|Acrapex_relicta|06-NCCC-1241|
AACATTATATTTTATTTTTGGAATTTGAGCAGGTATAGTGGGAACTTCTTTGAGATTGCTAATTCGAGCTGAATTAGGAA
ATCCTGGATCTTTAATTGGTGATGATCAAATTTATAATACTATTGTTACAGCCCATGCTTTTATTATAATTTTTTTTATA
GTTATACCTATTATAATTGGAGGATTTGGAAATTGACTTGTACCTCTAATATTAGGAGCCCCAGATATAGCATTCCCACG
AATAAATAATATAAGTTTTTGGTTACTCCCTCCCTCATTAACTTTACTAATTTCAAGAAGAATTGTAGAAAATGGTGCAG
GAACAGGATGAACAGTGTACCCCCCACTTTCATCTAATATTGCTCATGGAGGAAGCTCCGTAGATTTAGCAATTTTTTCC
CTTCATTTAGCAGGTATTTCTTCTATTTTAGGAGCTATTAATTTTATTACCACAATTATTAATATACGATTAAATAATTT
ATCTTTTGATCAAATACCTTTATTTATTTGAGCTGTAGGAATTACTGCATTTTTATTATTATTATCACTACCTGTTTTAG
CAGGAGCCATTACAATATTACTAACAGATCGAAATCTAAATACATCATTTTTTGACCCAGCAGGAGGGGGAGATCCAATT
TTATATCAACATTTATTT---------
>LNCB347-06|Chilo_plejadellus|06-NCCC-1303|
AACCTTATATTTTATTTTTGGAATTTGAGCAGGAATAATTGGAACATCTCTTAGACTTTTAATCCGTGCTGAACTAGGAA
CTCCTGGATTTTTAATTGGAGATGATCAAATTTATAATACTATTGTAACAGCTCATGCATTTATTATAATTTTTTTTATA
GTTATACCTATTATAATTGGTGGATTTGGAAATTGATTAGTACCTTTAATATTAGGAGCTCCTGATATAGCTTTTCCACG
AATAAATAATATAAGATTCTGAATATTACCCCCCTCATTAACTTTACTAATTTCTAGTAGTATTGTTGAAAACGGAGCTG
GAACAGGATGAACGGTGTACCCCCCACTATCATCTAATATTGCCCATGCTGGAAGTTCAGTAGATTTAGCAATTTTTTCC
CTACATTTAGCTGGAATTTCTTCAATTTTAGGAGCTATTAATTTTATTACCACAATTATTAATATACGAATTAATGGATT
ATCATTTGATCAAATACCTTTATTTGTTTGATCTGTAGGTATTACAGCCTTATTACTCCTACTCTCACTACCGGTATTAG
CTGGTGCTATTACAATATTACTAACTGACCGAAATTTAAATACATCTTTCTTCGATCCAGCTGGTGGGGGGGATCCAATT
CTTTATCAACATTTATTT---------
>LNCB348-06|Chilo_plejadellus|06-NCCC-1304|
AACCTTATATTTTATTTTTGGAATTTGAGCAGGAATAATTGGAACATCTCTTAGACTTTTAATCCGTGCTGAACTAGGAA
CTCCTGGATTTTTAATTGGAGATGATCAAATTTATAATACTATTGTAACAGCTCATGCATTTATTATAATTTTTTTTATA
GTTATACCTATTATAATTGGTGGATTTGGAAATTGATTAGTACCTTTAATATTAGGAGCTCCTGATATAGCTTTTCCACG
AATAAATAATATAAGATTCTGAATATTACCCCCCTCATTAACTTTACTAATTTCTAGTAGTATTGTTGAAAACGGAGCTG
GAACAGGATGAACGGTGTACCCCCCACTATCATCTAATATTGCCCATGCTGGAAGTTCAGTAGATTTAGCAATTTTTTCC
CTACATTTAGCTGGAATTTCTTCAATTTTAGGAGCTATTAATTTTATTACCACAATTATTAATATACGAATTAATGGATT
ATCATTTGATCAAATACCTTTATTTGTTTGATCTGTAGGTATTACAGCCTTATTACTCCTACTCTCACTACCGGTATTAG
CTGGTGCTATTACAATATTACTAACTGACCGAAATTTAAATACATCTTTCTTCGATCCAGCTGGTGGGGGGGATCCAATT
CTTTATCAACATTTATTT---------
>LNCB349-06|Chilo_plejadellus|06-NCCC-1305|
AACCTTATATTTTATTTTTGGAATTTGAGCAGGAATAATTGGAACATCTCTTAGACTTTTAATCCGTGCTGAACTAGGAA

CTCCTGGATTTTTAATTGGAGATGATCAAATTTATAATACTATTGTAACAGCTCATGCATTTATTATAATTTTTTTTATA
GTTATACCTATTATAATTGGTGGATTTGGAAATTGATTAGTACCTTTAATATTAGGAGCTCCTGATATAGCTTTCCACG
AATAAATAATATAAGATTCTGAATATTACCCCCCTCATTAACTTTACTAATTTCTAGTAGTATTGTTGAAAACGGAGCTG
GAACAGGATGAACGGTGTACCCCCCACTATCATCTAATATTGCCCATGCTGGAAGTTCAGTAGATTTAGCAATTTTTTCC
CTACATTTAGCTGGAATTTCTTCAATTTTAGGAGCTATTAATTTTATTACCACAATTATTAATATACGAATTAATGGATT
ATCATTTGATCAAATACCTTTATTTGTTTGATCTGTAGGTATTACAGCCTTATTACTCCTACTCTCACTACCGGTATTAG
CTGGTGCTATTACAATATTACTAACTGACCGAAATTTAAATACATCTTTCTTCGATCCAGCTGGTGGGGGGGATCCAATT
CTTTATCAACATTTATTT---------
>LNCB350-06|Chilo_plejadellus|06-NCCC-1306|
AACCTTATATTTTATTTTTGGAATTTGAGCAGGAATAATTGGAACATCTCTTAGACTTTTAATCCGTGCTGAACTAGGAA
CTCCTGGATTTTTAATTGGAGATGATCAAATTTATAATACTATTGTAACAGCTCATGCATTTATTATAATTTTTTTTATA
GTTATACCTATTATAATTGGTGGATTTGGAAATTGATTAGTACCTTTAATATTAGGAGCTCCTGATATAGCTTTCCACG
AATAAATAATATAAGATTCTGAATATTACCCCCCTCATTAACTTTACTAATTTCTAGTAGTATTGTTGAAAACGGAGCTG
GAACAGGATGAACGGTGTACCCCCCACTATCATCTAATATTGCCCATGCTGGAAGTTCAGTAGATTTAGCAATTTTTTCC
CTACATTTAGCTGGAATTTCTTCAATTTTAGGAGCTATTAATTTTATTACCACAATTATTAATATACGAATTAATGGATT
ATCATTTGATCAAATACCTTTATTTGTTTGATCTGTAGGTATTACAGCCTTATTACTCCTACTCTCACTACCGGTATTAG
CTGGTGCTATTACAATATTACTAACTGACCGAAATTTAAATACATCTTTCTTCGATCCAGCTGGTGGGGGGGATCCAATT
CTTTATCAACATTTATTT---------
>LNCC702-11|Acrapex_relicta|11-NCCC-227|
AACATTATATTTTATTTTTGGAATTTGAGCAGGTATGGTAGGAACTTCTTTGAGATTACTAATTCGAGCTGAATTAGGAA
ATCCTGGATCTTTAATTGGTGATGATCAAATTTATAATACTATTGTTACAGCCCATGCTTTTATTATAATTTTTTTTATA
GTTATACCTATTATAATTGGAGGATTTGGAAATTGACTTGTACCTCTAATATTAGGAGCCCCAGATATAGCATTCCCACG
AATAAATAATATAAGTTTTTGGTTACTCCCTCCCTCATTAACTTTACTAATTTCAAGAAGAATTGTAGAAAATGGTGCAG
GAACAGGATGAACAGTGTACCCCCCACTTTCATCTAATATTGCTCATGGAGGAAGCTCTGTAGATTTAGCAATTTTTTCC
CTTCATTTAGCAGGTATTTCTTCTATTTTAGGAGCTATTAATTTTATTACCACAATTATTAATATACGATTAAATAATTT
ATCTTTTGATCAAATACCTTTATTTATTTGAGCTGTAGGAATTACTGCATTTTTATTATTATTATCACTACCTGTTTTAG
CAGGAGCCATTACAATACTACTAACAGATCGAAATCTAAATACATCATTTTTTGACCCAGCAGGAGGGGGAGATCCAATT
TTATATCAACATTTATTT---------
>LNSWA023-05|Bathytricha_truncata|05-NSW-00023|
AACATTATATTTCATCTTTGGAATTTGAGCAGGAATAGTAGGAACTTCTTTAAGACTATTAATTCGAGCTGAATTAGGAA
CTCCTGGATCCTTAATTGGAGATGATCAAATTTATAATACTATTGTAACAGCTCATGCCTTTATTATAATTTTTTTTATA
GTTATACCAATTATAATCGGGGGATTTGGAAATTGACTTGTACCTTTAATGTTAGGAGCGCCTGATATAGCATTTCCACG
AATAAATAATATAAGTTTTTGATTACTACCCCCTTCTTTAACTCTACTTATTTCGAGAAGAGTTGTAGAAAATGGAGCAG
GAACTGGATGAACAGTATACCCCCCACTTTCATCTAATATCGCCCATAGAGGAAGATCTGTAGACTTAGCTATTTTTTCC
CTTCATTTAGCTGGAATTTCTTCTATTTTAGGAGCTATTAATTTTATTACAACTATTATTAACATACGATTAAATAATTT
ATCTTTTGATCAAATACCTTTATTTATTTGAGCTGTAGGAATTACAGCATTTTTATTATTATTATCATTACCTGTCTTAG
CTGGAGCTATTACTATATTATTAACAGATCGAAATTTAAATACATCATTTTTTGATCCTGCAGGAGGGGGAGATCCAATC
TTATATCAACATTTATTT---------
>LNSWA024-05|Bathytricha_truncata|05-NSW-00024|
AACATTATATTTCATCTTTGGAATTTGAGCAGGAATAGTAGGAACTTCTTTAAGACTATTAATTCGAGCTGAATTAGGAA
CTCCTGGATCCTTAATTGGAGATGATCAAATTTATAATACTATTGTAACAGCTCATGCCTTTATTATAATTTTTTTTATA
GTTATACCAATTATAATCGGGGGATTTGGAAATTGACTTGTACCTTTAATGTTAGGAGCGCCTGATATAGCATTTCCACG
AATAAATAATATAAGTTTTTGATTACTACCCCCTTCTTTAACTCTACTTATTTCGAGAAGAGTTGTAGAAAATGGAGCAG
GAACTGGATGAACAGTATACCCCCCACTTTCATCTAATATCGCCCATAGAGGAAGATCTGTAGACTTAGCTATTTTTTCC
CTTCATTTAGCTGGAATTTCTTCTATTTTAGGAGCTATTAATTTTATTACAACTATTATTAACATACGATTAAATAATTT
ATCTTTTGATCAAATACCTTTATTTATTTGAGCTGTAGGAATTACAGCATTTTTATTATTATTATCATTACCTGTCTTAG
CTGGAGCTATTACTATATTATTAACAGATCGAAATTTAAATACATCATTTTTTGATCCTGCAGGAGGGGGAGATCCAATC
TTATATCAACATTTATTT---------
>LNSWA026-05|Bathytricha_truncata|05-NSW-00026|
AACATTATATTTCATCTTTGGAATTTGAGCAGGAATAGTAGGAACTTCTTTAAGACTATTAATTCGAGCTGAATTAGGAA
CTCCTGGATCCTTAATTGGAGATGATCAAATTTATAATACTATTGTAACAGCTCATGCCTTTATTATAATTTTTTTTATA
GTTATACCAATTATAATCGGGGGATTTGGAAATTGACTTGTACCTTTAATGTTAGGAGCGCCTGATATAGCATTTCCACG
AATAAATAATATAAGTTTTTGATTACTACCCCCTTCTTTAACTCTACTTATTTCGAGAAGAGTTGTAGAAAATGGAGCAG
GAACTGGATGAACAGTATACCCCCCACTTTCATCTAATATCGCCCATAGAGGAAGATCTGTAGACTTAGCTATTTTTTCC
CTTCATTTAGCTGGAATTTCTTCTATTTTAGGAGCTATTAATTTTATTACAACTATTATTAACATACGATTAAATAATTT
ATCTTTTGATCAAATACCTTTATTTATTTGAGCTGTAGGAATTACAGCATTTTTATTATTATTATCATTACCTGTCTTAG
CTGGAGCTATTACTATATTATTAACAGATCGAAATTTAAATACATCATTTTTTGATCCTGCAGGAGGGGGAGATCCAATC

TTATATCAACATTTATTT---------
>LNSWA027-05|Bathytricha_truncata|05-NSW-00027|
AACATTATATTTCATCTTTGGAATTTGAGCAGGAATAGTAGGAACTTCTTTAAGACTATTAATTCGAGCTGAATTAGGAA
CTCCTGGATCCTTAATTGGAGATGATCAAATTTATAATACTATTGTAACAGCTCATGCCTTTATTATAATTTTTTTTATA
GTTATACCAATTATAATCGGGGGATTTGGAAATTGACTTGTACCTTTAATGTTAGGAGCGCCTGATATAGCATTTCCACG
AATAAATAATATAAGTTTTTGATTACTACCCCCTTCTTTAACTCTACTTATTTCGAGAAGAGTTGTAGAAAATGGAGCAG
GAACTGGATGAACAGTATACCCCCCACTTTCATCTAATATCGCCCATAGAGGAAGATCTGTAGACTTAGCTATTTTTTCC
CTTCATTTAGCTGGAATTTCTTCTATTTTAGGAGCTATTAATTTTATTACAACTATTATTAACATACGATTAAATAATTT
ATCTTTTGATCAAATACCTTTATTTATTTGAGCTGTAGGAATTACAGCATTTTTATTATTATTATCATTACCTGTCTTAG
CTGGAGCTATTACTATATTATTAACAGATCGAAATTTAAATACATCATTTTTTGATCCTGCAGGAGGGGGAGATCCAATC
TTATATCAACATTTATTT---------
>LNSWA028-05|Bathytricha_truncata|05-NSW-00028|
AACATTATATTTCATCTTTGGAATTTGAGCAGGAATAGTAGGAACTTCTTTAAGACTATTAATTCGAGCTGAATTAGGAA
CTCCTGGATCCTTAATTGGAGATGATCAAATTTATAATACTATTGTAACAGCTCATGCCTTTATTATAATTTTTTTTATA
GTTATACCAATTATAATCGGGGGATTTGGAAATTGACTTGTACCTTTAATGTTAGGAGCGCCTGATATAGCATTTCCACG
AATAAATAATATAAGTTTTTGATTACTACCTCCTTCTTTAACTCTACTTATTTCGAGAAGAGTTGTAGAAAATGGAGCAG
GAACTGGATGAACAGTATACCCCCCACTTTCATCTAATATCGCCCATAGAGGAAGATCTGTAGACTTAGCTATTTTTTCC
CTTCATTTAGCTGGAATTTCTTCTATTTTAGGAGCTATTAATTTTATTACAACTATTATTAACATACGATTAAATAATTT
ATCTTTTGATCAAATACCTTTATTTATTTGAGCTGTAGGAATTACAGCATTTTTATTATTATTATCATTACCTGTCTTAG
CTGGAGCTATTACTATATTATTAACAGATCGAAATTTAAATACATCATTTTTTGATCCTGCAGGAGGGGGAGATCCAATC
TTATATCAACATTTATTT---------
>LNSWA029-05|Bathytricha_truncata|05-NSW-00029|
AACATTATATTTCATCTTTGGAATTTGAGCAGGAATAGTAGGAACTTCTTTAAGACTATTAATTCGAGCTGAATTAGGAA
CTCCTGGATCCTTAATTGGAGATGATCAAATTTATAATACTATTGTAACAGCTCATGCCTTTATTATAATTTTTTTTATA
GTTATACCAATTATAATCGGGGGATTTGGAAATTGACTTGTACCTTTAATGTTAGGAGCGCCTGATATAGCATTTCCACG
AATAAATAATATAAGTTTTTGATTACTACCTCCTTCTTTAACTCTACTTATTTCGAGAAGAGTTGTAGAAAATGGAGCAG
GAACTGGATGAACAGTATACCCCCCACTTTCATCTAATATCGCCCATAGAGGAAGATCTGTAGACTTAGCTATTTTTTCC
CTTCATTTAGCTGGAATTTCTTCTATTTTAGGAGCTATTAATTTTATTACAACTATTATTAACATACGATTAAATAATTT
ATCTTTTGATCAAATACCTTTATTTATTTGAGCTGTAGGAATTACAGCATTTTTATTATTATTATCATTACCTGTCTTAG
CTGGAGCTATTACTATATTATTAACAGATCGAAATTTAAATACATCATTTTTTGATCCTGCAGGAGGGGGAGATCCAATC
TTATATCAACATTTATTT---------
>LNSWA030-05|Bathytricha_truncata|05-NSW-00030|
AACATTATATTTCATCTTTGGAATTTGAGCAGGAATAGTAGGAACTTCTTTAAGACTATTAATTCGAGCTGAATTAGGAA
CTCCTGGATCCTTAATTGGAGATGATCAAATTTATAATACTATTGTAACAGCTCATGCCTTTATTATAATTTTTTTTATA
GTTATACCAATTATAATCGGGGGATTTGGAAATTGACTTGTACCTTTAATGTTAGGAGCGCCTGATATAGCATTTCCACG
AATAAATAATATAAGTTTTTGATTACTACCCCCTTCTTTAACTCTACTTATTTCGAGAAGAGTTGTAGAAAATGGAGCAG
GAACTGGATGAACAGTATACCCCCCACTTTCATCTAATATCGCCCATAGAGGAAGATCTGTAGACTTAGCTATTTTTTCC
CTTCATTTAGCTGGAATTTCTTCTATTTTAGGAGCTATTAATTTTATTACAACTATTATTAACATACGATTAAATAATTT
ATCTTTTGATCAAATACCTTTATTTATTTGAGCTGTAGGAATTACAGCATTTTTATTATTATTATCATTACCTGTCTTAG
CTGGAGCTATTACTATATTATTAACAGATCGAAATTTAAATACATCATTTTTTGATCCTGCAGGAGGGGGAGATCCAATC
TTATATCAACATTTATTT---------
>LNSWA031-05|Bathytricha_truncata|05-NSW-00031|
AACATTATATTTCATCTTTGGAATTTGAGCAGGAATAGTAGGAACTTCTTTAAGACTATTAATTCGAGCTGAATTAGGAA
CTCCTGGATCCTTAATTGGAGATGATCAAATTTATAATACTATTGTAACAGCTCATGCCTTTATTATAATTTTTTTTATA
GTTATACCAATTATAATCGGGGGATTTGGAAATTGACTTGTACCTTTAATGTTAGGAGCGCCTGATATAGCATTTCCACG
AATAAATAATATAAGTTTTTGATTACTACCCCCTTCTTTAACTCTACTTATTTCGAGAAGAGTTGTAGAAAATGGAGCAG
GAACTGGATGAACAGTATACCCCCCACTTTCATCTAATATCGCCCATAGAGGAAGATCTGTAGACTTAGCTATTTTTTCC
CTTCATTTAGCTGGAATTTCTTCTATTTTAGGAGCTATTAATTTTATTACAACTATTATTAACATACGATTAAATAATTT
ATCTTTTGATCAAATACCTTTATTTATTTGAGCTGTAGGAATTACAGCATTTTTATTATTATTATCATTACCTGTCTTAG
CTGGAGCTATTACTATATTATTAACAGATCGAAATTTAAATACATCATTTTTTGATCCTGCAGGAGGGGGAGATCCAATC
TTATATCAACATTTATTT---------
>LNSWA032-05|Bathytricha_truncata|05-NSW-00032|
AACATTATATTTCATCTTTGGAATTTGAGCAGGAATAGTAGGAACTTCTTTAAGACTATTAATTCGAGCTGAATTAGGAA
CTCCTGGATCCTTAATTGGAGATGATCAAATTTATAATACTATTGTAACAGCTCATGCCTTTATTATAATTTTTTTTATA
GTTATACCAATTATAATCGGGGGATTTGGAAATTGACTTGTACCTTTAATGTTAGGAGCGCCTGATATAGCATTTCCACG
AATAAATAATATAAGTTTTTGATTACTACCCCCTTCTTTAACTCTACTTATTTCGAGAAGAGTTGTAGAAAATGGAGCAG
GAACTGGATGAACAGTATACCCCCCACTTTCATCTAATATCGCCCATAGAGGAAGATCTGTAGACTTAGCTATTTTTTCC

CTTCATTTAGCTGGAATTTCTTCTATTTTAGGAGCTATTAATTTTATTACAACTATTATTAACATACGATTAAATAATTT
ATCTTTTGATCAAATACCTTTATTTATTTGAGCTGTAGGAATTACAGCATTTTTATTATTATTATCATTACCTGTCTTAG
CTGGAGCTATTACTATATTATTAACAGATCGAAATTTAAATACATCATTTTTTGATCCTGCAGGAGGGGGAGATCCAATC
TTATATCAACATTTATTT---------
>LNSWA033-05|Bathytricha_truncata|05-NSW-00033|
AACATTATATTTCATCTTTGGAATTTGAGCAGGAATAGTAGGAACTTCTTTAAGACTATTAATTCGAGCTGAATTAGGAA
CTCCTGGATCCTTAATTGGAGATGATCAAATTTATAATACTATTGTAACAGCTCATGCCTTTATTATAATTTTTTTTATA
GTTATACCAATTATAATCGGGGGATTTGGAAATTGACTTGTACCTTTAATGTTAGGAGCGCCTGATATAGCATTTCCACG
AATAAATAATATAAGTTTTTGATTACTACCTCCTTCTTTAACTCTACTTATTTCGAGAAGAGTTGTAGAAAATGGAGCAG
GAACTGGATGAACAGTATACCCCCCACTTTCATCTAATATCGCCCATAGAGGAAGATCTGTAGACTTAGCTATTTTTTCC
CTTCATTTAGCTGGAATTTCTTCTATTTTAGGAGCTATTAATTTTATTACAACTATTATTAACATACGATTAAATAATTT
ATCTTTTGATCAAATACCTTTATTTATTTGAGCTGTAGGAATTACAGCATTTTTATTATTATTATCATTACCTGTCTTAG
CTGGAGCTATTACTATATTATTAACAGATCGAAATTTAAATACATCATTTTTTGATCCTGCAGGAGGGGGAGATCCAATC
TTATATCAACATTTATTT---------
>LOFLA060-06|Diatraea_evanescens|06-FLOR-0060|
NNCTTTATATTTTATTTTTGGAATTTGAGCTGGAATACTGGGAACATCTTTAAGTTTATTAATTCGTGCAGAATTAGGTA
CAACTAATTCTTTAATTGGAGATGATCAAATCTACAATACAATTGTTACAGCTCATGCATTTATTATAATTTTTTTTATA
GTTATACCCATTATAATTGGAGGATTTGGAAACTGATTAGTACCATTAATATTAGGAGCTCCTGATATAGCTTTCCCTCG
AATAAATAATATAAGATTTTGATTACTACCCCCATCTTTAACTTTATTAATTTCTAGAAGAATTGTTGAAAATGGAGCTG
GAACAGGATGAACAGTTTACCCCCCACTTTCATCAAATATTGCTCATGGTGGTAGATCTGTAGACCTAGCAATTTTTTCA
TTACATTTAGCTGGAATCTCATCAATTTTAGGAGCTATTAATTTTATTACCACAATTATTAATATACGAATTAATGGATT
ATCATTCGATCAAATACCTCTATTTGTATGATCAGTAGGTATTACAGCCTTATTATTACTTTTATCTTTACCTGTTTTAG
CTGGAGCTATTACCATATTATTAACAGATCGAAACTTAAATACTTCCTTTTTCGACCCAGCTGGAGGGGGAGATCCAATT
TTATATCAACATTTATTT---------
>LOFLA067-06|Diatraea_evanescens|06-FLOR-0067|
AACTTTATATTTTATTTTTGGAATTTGAGCTGGAATACTGGGAACATCTTTAAGTTTATTAATTCGTGCAGAATTAGGTA
CAACTAATTCTTTAATTGGAGATGATCAAATCTACAATACAATTGTTACAGCTCATGCATTTATTATAATTTTTTTTATA
GTTATACCCATTATAATTGGAGGATTTGGAAACTGATTAGTACCATTAATATTAGGAGCTCCTGATATAGCTTTCCCTCG
AATAAATAATATAAGATTTTGATTACTACCCCCATCTTTAACTTTATTAATTTCTAGAAGAATTGTTGAAAATGGAGCTG
GAACAGGATGAACAGTTTACCCCCCACTTTCATCAAATATTGCTCATGGTGGTAGATCTGTAGACCTAGCAATTTTTTCA
TTACATTTAGCTGGAATCTCATCAATTTTAGGAGCTATTAATTTTATTACCACAATTATTAATATACGAATTAATGGATT
ATCATTCGATCAAATACCTCTATTTGTATGATCAGTAGGTATTACAGCCTTATTATTACTTTTATCTTTACCTGTTTTAG
CTGGAGCTATTACCATATTATTAACAGATCGAAACTTAAATACTTCCTTTTTCGACCCAGCTGGAGGGGGAGATCCAATT
TTATATCAACATTTATTTTGATTTTTT
>LOFLA068-06|Diatraea_evanescens|06-FLOR-0068|
AACTTTATATTTTATTTTTGGAATTTGAGCTGGAATACTGGGAACATCTTTAAGTTTATTAATTCGTGCAGAATTAGGTA
CAACTAATTCTTTAATTGGAGATGATCAAATCTACAATACAATTGTTACAGCTCATGCATTTATTATAATTTTTTTTATA
GTTATACCCATTATAATTGGAGGATTTGGAAACTGATTAGTACCATTAATATTAGGAGCTCCTGATATAGCTTTCCCTCG
AATAAATAATATAAGATTTTGATTACTACCCCCATCTTTAACTTTATTAATTTCTAGAAGAATTGTTGAAAATGGAGCTG
GAACAGGATGAACAGTTTACCCCCCACTTTCATCAAATATTGCTCATGGTGGTAGATCTGTAGACCTAGCAATTTTTTCA
TTACATTTAGCTGGAATCTCATCAATTTTAGGAGCTATTAATTTTATTACCACAATTATTAATATACGAATTAATGGATT
ATCATTCGATCAAATACCTCTATTTGTATGATCAGTAGGTATTACAGCCTTATTATTACTTTTATCTTTACCTGTTTTAG
CTGGAGCTATTACCATATTATTAACAGATCGAAACTTAAATACTTCCTTTTTCGACCCAGCTGGAGGGGGAGATCCAATT
TTATATCAACATTTATTT---------
>LOFLA266-06|Diatraea_evanescens|06-FLOR-0266|
AACTTTATATTTTATTTTTGGAATTTGAGCTGGAATACTGGGAACATCTTTAAGTTTATTAATTCGTGCAGAATTAGGTA
CAACTAATTCTTTAATTGGAGATGATCAAATCTACAATACAATTGTTACAGCTCATGCATTTATTATAATTTTTTTTATA
GTTATACCCATTATAATTGGAGGATTTGGAAACTGATTAGTACCATTAATATTAGGAGCTCCTGATATAGCTTTCCCTCG
AATAAATAATATAAGATTTTGATTACTACCCCCATCTTTAACTTTATTAATTTCTAGAAGAATTGTTGAAAATGGAGCTG
GAACAGGATGAACAGTTTACCCCCCACTTTCATCAAATATTGCTCATGGTGGTAGATCTGTAGACCTAGCAATTTTTTCA
TTACATTTAGCTGGAATCTCATCAATTTTAGGAGCTATTAATTTTATTACCACAATTATTAATATACGAATTAATGGATT
ATCATTCGATCAAATACCTCTATTTGTATGATCAGTAGGTATTACAGCCTTATTATTACTTTTATCTTTACCTGTTTTAG
CTGGAGCTATTACCATATTATTAACAGATCGAAACTTAAATACTTCCTTTTTCGACCCAGCTGGAGGGGGAGATCCAATT
TTATATCAACATTTATTT---------
>LOFLA657-06|Eoreuma_densella|06-FLOR-0657|
AACATTATATTTTATTTTTGGAATTTGAGCTGGAATAGTAGGAACATCTCTTAGTTTATTAATTCGAGCTGAATTAGGAA
ATCCTGGTTCCTTAATTGGAGATGATCAAATTTATAATACTATTGTCACAGCACATGCATTTATTATAATTTTTTTTATA

GTTATACCTATTATAATTGGAGGATTTGGAAATTGATTAGTTCCTTTAATATTAGGAGCCCCTGATATAGCATTCCCCCG
AATAAATAATATAAGATTTTGATTATTACCCCCCTCATTAACTTTATTAATTTCAAGTAGTATTGTCGAAAATGGTGCCG
GTACAGGATGAACTGTATACCCCCCTCTTTCCTCAAATATTGCCCATGGGGGAAGATCTGTTGACTTAGCAATTTTTTCT
TTACATTTAGCTGGAATTTCATCAATCTTAGGAGCTATCAACTTTATTACAACAATTATTAATATACGAATTAATAATTT
ATCATTTGATCAAATACCTTTATTTGTTTGATCAGTAGGAATTACAGCATTACTTCTCCTCCTTTCATTGCCTGTTTTAG
CAGGAGCTATTACTATATTATTAACAGATCGAAATCTTAATACATCATTTTTTGATCCTGCTGGAGGAGGAGATCCAATT
CTCTACCAACATTTATTT---------
>LOFLB456-06|Eoreuma_densella_PS1|06-FLOR-1396|
AACATTATATTTTATCTTTGGAATTTGAGCTGGAATAGTAGGAACATCTCTTAGTTTATTAATTCGAGCTGAATTAGGAA
ATCCTGGTTCTTTAATTGGAGATGATCAAATTTATAATACTATTGTCACAGCACATGCATTTATTATAATTTTTTTTATA
GTTATACCTATTATAATTGGAGGATTTGGAAATTGATTAGTTCCTTTAATATTAGGAGCTCCTGATATAGCATTCCCCCG
AATAAATAATATAAGATTTTGATTATTACCCCCCTCATTAACTTTGTTAATTTCAAGTAGTATTGTCGAAAATGGTGCCG
GTACAGGATGAACTGTATACCCCCCCCTTTCCTCAAATATTGCCCATGGGGGGAGATCTGTTGACTTGGCAATTTTTTCT
TTACATTTAGCTGGGATCTCATCAATCTTAGGAGCCATTAATTTTATTACAACAATTATTAATATGCGAATTAATAATTT
ATCATTTGATCAAATACCTTTATTTGTTTGATCGGTAGGAATTACAGCATTACTTCTCCTTCTTTCATTGCCTGTTTTAG
CAGGAGCTATTACTATATTATTAACAGATCGAAATCTTAACACATCATTTTTTGACCCTGCTGGAGGAGGAGATCCAATT
CTCTACCAACATTTATTT---------
>LOFLC426-06|Diatraea_evanescens|06-FLOR-2306|
AACTTTATATTTTATTTTTGGAATTTGAGCTGGAATACTGGGAACATCTTTAAGTTTATTAATTCGTGCAGAATTAGGTA
CAACTAATTCTTTAATTGGAGATGATCAAATCTACAATACAATTGTTACAGCTCATGCATTTATTATAATTTTTTTTATA
GTTATACCCATTATAATTGGAGGATTTGGAAACTGATTAGTACCATTAATATTAGGAGCTCCTGATATAGCTTTCCCTCG
AATAAATAATATAAGATTTTGATTACTACCCCCATCTTTAACTTTATTAATTTCTAGAAGAATTGTTGAAAATGGAGCTG
GAACAGGATGAACAGTTTACCCCCCACTTTCATCAAATATTGCTCATGGTGGTAGATCTGTAGACCTAGCAATTTTTTCA
TTACATTTAGCTGGAATCTCATCAATTTTAGGAGCTATTAATTTTATTACCACAATTATTAATATACGAATTAATGGATT
ATCATTCGATCAAATACCTCTATTTGTATGATCAGTAGGTATTACAGCCTTATTATTACTTTTATCTTTACCTGTTTTAG
CTGGAGCTATTACCATATTATTAACAGATCGAAACTTAAATACTTCCTTTTTCGACCCAGCTGGAGGGGGAGATCCAATT
TTATATCAACATTTATTT---------
>LOFLD150-07|Diatraea_evanescens|HLC-16724|
AACTTTATATTTTATTTTTGGAATTTGAGCTGGAATACTGGGAACATCTTTAAGTTTATTAATTCGTGCAGAATTAGGTA
CAACTAATTCTTTAATTGGAGATGATCAAATCTACAATACAATTGTTACAGCTCATGCATTTATTATAATTTTTTTTATA
GTTATACCCATTATAATTGGAGGATTTGGAAACTGATTAGTACCATTAATATTAGGAGCTCCTGATATAGCTTTCCCTCG
AATAAATAATATAAGATTTTGATTACTACCCCCATCTTTAACTTTATTAATTTCTAGAAGAATTGTTGAAAATGGAGCTG
GAACAGGATGAACAGTTTACCCCCCACTTTCATCAAATATTGCTCATGGTGGTAGATCTGTAGACCTAGCAATTTTTTCA
TTACATTTAGCTGGAATCTCATCAATTTTAGGAGCTATTAATTTTATTACCACAATTATTAATATACGAATTAATGGATT
ATCATTCGATCAAATACCTCTATTTGTATGATCAGTAGGTATTACAGCCTTATTATTACTTTTATCTTTACCTGTTTTAG
CTGGAGCTATTACCATATTATTAACAGATCGAAACTTAAATACTTCCTTTTTCGACCCAGCTGGAGGGGGAGATCCAATT
TTATATCAACATTTATTT---------
>LOFLD273-07|Diatraea_evanescens|HLC-16848|
AACTTTATATTTTATTTTTGGAATTTGAGCTGGAATACTGGGAACATCTTTAAGTTTATTAATTCGTGCAGAATTAGGTA
CAACTAATTCTTTAATTGGAGATGATCAAATCTACAATACAATTGTTACAGCTCATGCATTTATTATAATTTTTTTTATA
GTTATACCCATTATAATTGGAGGATTTGGAAACTGATTAGTACCATTAATATTAGGAGCTCCTGATATAGCTTTCCCTCG
AATAAATAATATAAGATTTTGATTACTACCCCCATCTTTAACTTTATTAATTTCTAGAAGAATTGTTGAAAATGGAGCTG
GAACAGGATGAACAGTTTACCCCCCACTTTCATCAAATATTGCTCATGGTGGTAGATCTGTAGACCTAGCAATTTTTTCA
TTACATTTAGCTGGAATCTCATCAATTTTAGGAGCTATTAATTTTATTACCACAATTATTAATATACGAATTAATGGATT
ATCATTCGATCAAATACCTCTATTTGTATGATCAGTAGGTATTACAGCCTTATTATTACTTTTATCTTTACCTGTTTTAG
CTGGAGCTATTACCATATTATTAACAGATCGAAACTTAAATACTTCCTTTTTCGACCCAGCTGGAGGGGGAGATCCAATT
TTATATCAACATTTATTT---------
>LOFLD320-07|Diatraea_evanescens|HLC-16895|
AACTTTATATTTTATTTTTGGAATTTGAGCTGGAATACTGGGAACATCTTTAAGTTTATTAATTCGTGCAGAATTAGGTA
CAACTAATTCTTTAATTGGAGATGATCAAATCTACAATACAATTGTTACAGCTCATGCATTTATTATAATTTTTTTTATA
GTTATACCCATTATAATTGGAGGATTTGGAAACTGATTAGTACCATTAATATTAGGAGCTCCTGATATAGCTTTCCCTCG
AATAAATAATATAAGATTTTGATTACTACCCCCATCTTTAACTTTATTAATTTCTAGAAGAATTGTTGAAAATGGAGCTG
GAACAGGATGAACAGTTTACCCCCCACTTTCATCAAATATTGCTCATGGTGGTAGATCTGTAGACCTAGCAATTTTTTCA
TTACATTTAGCTGGAATCTCATCAATTTTAGGAGCTATTAATTTTATTACCACAATTATTAATATACGAATTAATGGATT
ATCATTCGATCAAATACCTCTATTTGTATGATCAGTAGGTATTACAGCCTTATTATTACTTTTATCTTTACCTGTTTTAG
CTGGAGCTATTACCATATTATTAACAGATCGAAACTTAAATACTTCCTTTTTCGACCCAGCTGGAGGGGGAGATCCAATT
TTATATCAACATTTATTT---------

>LOFLD483-07|Diatraea_evanescens|HLC-17099|
AACTTTATATTTTATTTTTGGAATTTGAGCTGGAATACTGGGAACATCTTTAAGTTTATTAATTCGTGCAGAATTAGGTA
CAACTAATTCTTTAATTGGAGATGATCAAATCTACAATACAATTGTTACAGCTCATGCATTTATTATAATTTTTTTTATA
GTTATACCCATTATAATTGGAGGATTTGGAAACTGATTAGTACCATTAATATTAGGAGCTCCTGATATAGCTTTCCCTCG
AATAAATAATATAAGATTTTGATTACTACCCCCATCTTTAACTTTATTAATTTCTAGAAGAATTGTTGAAAATGGAGCTG
GAACAGGATGAACAGTTTACCCCCCACTTTCATCAAATATTGCTCATGGTGGTAGATCTGTAGACCTAGCAATTTTTTCA
TTACATTTAGCTGGAATCTCATCAATTTTAGGAGCTATTAATTTTATTACCACAATTATTAATATACGAATTAATGGATT
ATCATTCGATCAAATACCTCTATTTGTATGATCAGTAGGTATTACAGCCTTATTATTACTTTTATCTTTACCTGTTTTAG
CTGGAGCTATTACCATATTATTAACAGATCGAAACTTAAATACTTCCTTTTTCGACCCAGCTGGAGGGGGAGATCCAATT
TTATATCAACATTTATTT---------
>LOFLD540-07|Diatraea_evanescens|HLC-17156|
AACTTTATATTTTATTTTTGGAATTTGAGCTGGAATACTGGGAACATCTTTAAGTTTATTAATTCGTGCAGAATTAGGTA
CAACTAATTCTTTAATTGGAGATGATCAAATCTACAATACAATTGTTACAGCTCATGCATTTATTATAATTTTTTTTATA
GTTATACCCATTATAATTGGAGGATTTGGAAACTGATTAGTACCATTAATATTAGGAGCTCCTGATATAGCTTTCCCTCG
AATAAATAATATAAGATTTTGATTACTACCCCCATCTTTAACTTTATTAATTTCTAGAAGAATTATTGAAAATGGAGCTG
GAACAGGATGAACAGTTTACCCCCCACTTTCATCAAATATTGCTCATGGTGGTAGATCTGTAGACCTAGCAATTTTTTCA
TTACATTTAGCTGGAATCTCATCAATTTTAGGAGCTATTAATTTTATTACCACAATTATTAATATACGAATTAATGGATT
ATCATTCGATCAAATACCTCTATTTGTATGATCAGTAGGTATTACAGCCTTATTATTACTTTTATCTTTACCTGTTTTAG
CTGGAGCTATTACCATATTATTAACAGATCGAAACTTAAATACTTCCTTTTTCGACCCAGCTGGAGGGGGAG--------
----
>LOLI199-08|Acrapex|08-QLDLI-199|
AACATTATATTTTATTTTTGGAATTTGAGCTGGTATAGTAGGAACTTCATTAAGATTATTAATTCGAGCTGAATTAGGAA
CCCCTGGATCTTTAATTGGAGATGATCAAATTTATAATACTATTGTTACAGCTCATGCTTTTATTATAATTTTTTTCATG
GTTATACCAATTATAATTGGAGGATTTGGAAATTGACTTGTACCTTTAATATTAGGAGCTCCAGATATAGCATTTCCACG
AATAAATAATATAAGATTTTGATTATTACCTCCTTCTTTAACTTTATTAATTTCAAGTAGAATTGTAGAAAATGGAGCAG
GAACTGGATGAACAGTATATCCACCTCTCTCATCAAATATTGCACATGGAGGAAGATCTGTAGATTTAGCTATTTTTTCT
CTTCATTTAGCAGGTATTTCTTCTATTTTAGGAGCTATTAATTTTATCACAACAATTATCAATATACGATTAAATAATTT
ATCTTTTGATCAAATACCTCTATTTATTTGAGCTGTTGGAATTACCGCTTTTTTATTACTATTATCTTTACCTGTATTAG
CAGGAGCTATCACAATATTATTAACTGATCGAAATTTAAATACATCATTTTTTGATCCTGCAGGAGGAG-----------
----
>LOLI206-08|Acrapex_exsanguis|08-QLDLI-206|
AACATTATATTTTATTTTTGGAATTTGAGCTGGTATACTAGGAACTTCTTTAAGTTTATTGATCCGAGCTGAATTAGGAA
CTCCAGAATCTTTAATTGGAGATGATCAAATTTATAATACTATTGTTACTGCTCACGCTTTTATTATAATTTTCTTTATA
GTTATACCAATTATAATTGGAGGATTTGGAAATTGACTTGTCCCATTAATACTAGGAGCTCCAGATATAGCATTTCCACG
TATAAATAATATAAGATTTTGATTATTACCTCCCTCTTTAAGTTTATTAATTTCAAGAAGAATTGTAGAAAATGGAGCAG
GAACTGGATGAACAGTATATCCTCCACTCTCATCTAATATTGCTCATAGAGGAAGATCAGTAGATTTAGCTATTTTTTCT
CTTCATTTAGCTGGTATTTCATCTATTTTAGGAGCTATTAATTTTATTACAACAATTATTAATATACGATTAAATAATTT
ATCTTTTGATCAAATACCTTTATTTGTTTGAGCTGTTGGAATTACTGCATTTTTACTATTACTCTCATTACCTGTATTAG
CCGGAGCTATTACAATATTATTAACAGATCGAAATTTAAATACATCATTTTTTGATCCTGCAGGAGGAGGTGATCCAATT
TTATATCAACATTTATTT---------
>LOLI260-08|Emmalocera|08-QLDLI-260|
AACTTTATATTTTATTTTTGGAATTTGAGCTGGAATAGTAGGAACATCTTTAAGTCTTTTAATTCGAGCTGAATTAGGAA
CCCCAGGATCTTTAATTGGAGATGATCAAATTTATAATACTATTGTTACAGGTCATGCTTTTATTATAATTTTTTTCATA
GTAATACCTATTATAATTGGAGGATTTGGAAATTGATTAGTACCTTTAATATTAGGAGCACCAGATATAGCTTTTCCTCG
GATAAATAATATAAGATTTTGATTCTTACCCCCCTCTCTTACTTTACTAATTTTTAGAAGAATTGTAGAGAATGGAGCAG
GAACTGGTTGAACTGTTTACCCCCCTTTATCTTCTAACATTGCCCATGGAGGAGGCTCTGTTGATCTTGCTATTTTTTCT
CTTCATTTAGCCGGAATTTCTTCTATTCTAGGAGCTATTAATTTTATTACTACTATTATTAATATAAAATTAAATGGATT
ATCATTTGACCAAATACCTTTATTTGTATGAGCTGTAGGAATTACTACTTTATTATTACTTTTATCTTTACCTGTTCTTG
CGGGAGCTATTACTATATTATTAACAGATCGAAATTTAAATACTTCTTTTTTTGATCCTGCTGGAGGAGGAGATCCTATT
CTTTACCAACATTTATTT---------
>LON589-08|Chilo_phragmitella|NHMO-08241|KX047952
-ACTTTATATTTTATTTTTGGAATTTGAGCTGGAATAATTGGAACATCTCTTAGACTTTTAATTCGAGCTGAATTAGGAA
CTCCAGGATCACTAATTGGAGATGATCAAATTTATAATACTATTGTTACAGCTCATGCATTTATTATAATTTTTTTTATA
GTTATACCTATTATAATTGGTGGTTTTGGAAATTGATTAGTACCTTTAATATTAGGAGCCCCTGATATAGCTTTCCCACG
AATAAATAATATAAGATTTTGATTATTACCACCTTCATTAACCTTATTAATCTCTAGAAGAATTGTTGAAAATGGAGCTG
GAACAGGATGAACAGTGTACCCCCCACTCTCATCTAATATTGCTCATGCTGGAAGTTCAGTAGATTTAGCAATTTTTTCC
TTACATTTAGCTGGAATTTCATCAATTTTAGGTGCTATTAATTTTATTACAACAATTATTAATATACGAATTAATGGATT

ATCATTTGATCAAATACCCCTACTCATTTGAAGAATTGGCATTTCAGCATTATTATTATTACTTTCTCTTCCAGTATTAG
CTGGTGCTATTACTATATTATTAACAGATCGAAATTTAAATACATCTTTTTTTGATCCAGCTGGAGGTGGAGATCCTATC
CTCTATCAACATTTATTT---------
>LOQ128-04|Emmalocera_sp._1|04HBL004128|
AACTTTATATTTTATTTTTGGAATTTGAGCTGGAATAGTAGGTACATCTTTAAGTCTTCTTATTCGAGCTGAATTAGGAA
CTCCTGGATCTTTAATTGGTGATGATCAAATTTATAATACTATTGTTACAGGACATGCTTTTATTATAATTTTTTTTATA
GTTATACCTATTATAATTGGGGGATTTGGAAATTGATTAGTCCCTCTTATATTAGGAGCTCCAGATATGGCTTTCCCTCG
AATAAATAATATAAGATTTTGACTTTTACCCCCTTCCCTTACTTTACTTATTTCTAGAAGAATTGTAGAAAATGGAGCAG
GGACAGGATGAACTGTTTATCCCCCTTTATCTTCTAATATTGCTCACGGAGGAAGTTCAGTAGATCTTGCTATTTTTTCT
CTTCATTTAGCTGGTATTTCTTCTATTCTAGGAGCTATTAACTTTATCACTACTATTATTAATATAAAATTAAATAATTT
ATCATTTGATCAAATACCATTATTTGTATGAGCTGTAGGAATTACAGCCTTATTATTATTATTATCTTTACCTGTTCTTG
CTGGAGCTATTACCATATTATTAACTGATCGTAATTTAAATACTTCTTTTTTTGACCCTGCTGGAGGAGGAGACCCTATT
CTTTATCAACATTTATTT---------
>LOQ493-04|Acrapex_sp._ANIC2|04HBL004493|
--ACTTCATTAAGATTATTAATTCGAGCTGAATTAGGAA
CCCCTGGATCTTTAATTGGAGATGATCAAATTTATAATACTATTGTTACAGCTCATGCTTTTATTATAATTTTTTTCATG
GTTATACCAATTATAATTGGAGGATTTGGAAATTGACTTGTACCTTTAATATTAGGAGCTCCAGATATAGCATTTCCACG
AATAAATAATATAAGATTTTGATTATTACCTCCTTCTTTAACTTTATTAATTTCAAGTAGAATTGTAGAAAATGGAGCAG
GAACTGGATGAACAGTATATCCACCTCTCTCATCAAATATTGCACATGGAGGAAGATCTGTAGATTTAGCTATTTTTTCT
CTTCATTTAGCAGGTATTTCTTCTATTTTAGGAGCTATTAATTTTATCACAACAATTATCAATATACGATTAAATAATTT
ATCTTTTGATCAAATACCTCTATTTATTTGAGCTGTTGGAATTACCGCTTTTTTATTACTATTATCTTTACCTGTATTAG
CAGGAGCTATCACAATATTATTAACTGATCGAAATTTAAATACATCA---------------------------------
----
>LOQB055-05|Acrapex_sp._ANIC2|Moth_055.03LZ|
--GAACTTCATTAAGATTATTAATTCGAGCTGAATTAGGAA
CCCCTGGATCTTTAATTGGAGATGATCAAATTTATAATACTATTGTTACAGCTCATGCTTTTATTATAATTTTTTTCATG
GTTATACCAATTATAATTGGAGGATTTGGAAATTGACTTGTACCTTTAATATTAGGAGCTCCAGATATAGCATTTCCACG
AATAAATAATATAAGATTTTGATTATTACCTCCTTCTTTAACTTTATTAATTTCAAGTAGAATTGTAGAAAATGGAGCAG
GAACTGGATGAACAGTATATCCACCTCTCTCATCAAATATTGCACATGGAGGAAGATCTGTAGATTTAGCTATTTTTTCT
CTTCATTTAGCAGGTATTTCTTCTATTTTAGGAGCTATTAATTTTATCACAACAATTATCAATATACGATTAAATAATTT
ATCTTTTGATCAAATACCTCTATTTATTTGAGCTGTTGGAATTACCGCTTTTTTATTACTATTATCTTTACCTGTATTAG
CAGGAGCTATCACAATATTATTAACTGATCGAAATTTAAATACATCATTTTTTGA-------------------------
----
>LOQB065-05|Acrapex_exsanguis|Moth_065.03LZ|
-------------------------------------CTANGAACTTCTTTAAGTTTATTAATCCGAGCTGAATTAGGAA
CTCCAGAATCTTTAATTGGAGATGATCAAATTTATAATACTATTGTTACTGCTCACGCTTTTATTATAATTTTCTTTATA
GTTATACCAATTATAATTGGAAGATTTGGAAATTGACTTGTCCCATTAATACTAGGAGCTCCAGATATAGCATTTCCACG
TATAAATAATATAAGATTTTGATTATTACCTCCCTCTTTAAGTTTATTAATTTCAAGAAGAATTGTAGAAAATGGAGCAG
GAACTGGATGAACAGTATATCCTCCACTCTCATCTAATATTGCTCATAGAGGAAGATCAGTAGATTTAGCTATTTTTTCT
CTTCATTTAGCTGGTATTTCATCTATTTTAGGAGCTATTAATTTTATTACAACAATTATTAATATACGATTAAATAATTT
ATCTTTTGATCAAATACCTTTATTTGTTTGAGCTGTTGGAATTACTGCATTTTTACTATTACTCTCATTACCTGTATTAG
CCGGAGCTATTACAATATTATTAACAGATCGAAATTTAAATACATCATTTTTTG--------------------------
----
>LOQB122-05|Acrapex_sp._ANIC2|Moth_122.03LZ|
AACATTATATTTTATTTTTGGAATTTGAGCTGGTATAGTAGGAACTTCATTAAGATTATTAATTCGAGCTGAATTAGGAA
CCTCTGGATCTTTAATTGGAGATGATCAAATTTATAATACTATTGTTACAGCTCATGCTTTTATTATAATTTTTTTCATG
GTTATACCAATTATAATTGGAGGATTTGGAAATTGACTTGTACCTTTAATATTAGGAGCTCCAGATATAGCATTTCCACG
AATAAATAATATAAGATTTTGATTATTACCTCCTTCTTTAACTTTATTAATTTCAAGTAGAATTGTAGAAAATGGAGCAG
GAACTGGATGAACAGTATATCCACCTCTCTCATCAAATATTGCACATGGAGGAAGATCTGTAGATTTAGCTATTTTTTCT
CTTCATTTAGCAGGTATTTCTTCTATTTTAGGAGCTATTAATTTTATCACAACAATTATCAATATACGATTAAATAATTT
ATCTTTTGATCAAATACCTCTATTTATTTGAGCTGTTGGAATTACCGCTTTTTTATTACTATTATCTTTACCTGTATTAG
CAGGAGCTATCACAATATTATTAACTGATCGAAATTTAAATACATCATTTTTTGATCCTGCAGGAGGAGGTGACCCAATT
TTANATCAACATTT-------------
>LOQB228-05|Acrapex_sp._ANIC2|Moth_228.01LZ|
--------------------------------------TAGGACTTCATTAAGATTATTAATTCGAGCTGAATTAGGAA
CCCCTGGATCTTTAATTGGAGATGATCAAATTTATAATACTATTGTTACAGCTCATGCTTTTATTATAATTTTTTTCATA
GTTATACCAATTATAATTGGAGGATTTGGAAATTGACTTGTACCTTTAATATTAGGAGCTCCAGATATAGCATTTCCACG

AATAAATAATATAAGATTTTGATTATTACCTCCTTCTTTAACTTTATTAATTTCAAGTAGAATTGTAGAAAATGGAGCAG
GAACTGGATGAACAGTATATCCACCTCTCTCATCAAATATTGCACATGGAGGAAGATCTGTAGATTTAGCTATTTTTTCT
CTTCATTTAGCAGGTATTTCTTCTATTTTAGGAGCTATTAATTTTATCACAACAATTATCAATATACGATTAAATAATTT
ATCTTTTGATCAAATACCTCTATTTATTTGAGCTGTTGGAATTACCGCTTTTTTATTACTATTATCTTTACCTGTATTAG
CAGGAGCTATCACAATATTATTAACTGATCGAAATTTAAATACATCATTTTTTGAT------------------------
----
>LOQB528-05|Chilo_crossostichus|Moth_007.03CL|
--GAACTTCTTTAAGTTTATTAATCCGTGCTGAATTAGGAA
ATCCAGGATCATTAATTGGAGATGATCAAATTTATAATACTATTGTAACAGCTCATGCATTTATTATAATTTTTTTTATA
GTTATACCAATTATAATTGGAGGATTTGGAAATTGATTAGTTCCTTTAATATTAGGAGCTCCAGATATAGCTTTTCCACG
AATAAATAATATAAGATTTTGACTTCTTCCCCCTTCTTTAACACTTCTAATTTCTAGAAGAATTGTTGAAAATGGAGCTG
GAACTGGATGAACAGTGTACCCCCCACTTTCTTCTAATATTGCTCACGGAGGAAGCTCTGTAGATTTAGCAATTTTTTCT
CTTCATTTAGCTGGTATTTCATCTATTTTAGGTGCTATTAATTTTATTACAACTATTATTAATATACGAATTAACGGATT
ATCTTTTGATCAAATACCTTTATTTGTATGATCTGTTGGTATTACAGCTTTACTTCTTTTACTTTCTCTTCCAGTATTAG
CTGGAGCTATTACTATACTTCTAACAGATCGAAATTTAAATACTTCTTCTTTTGA-------------------------
----
>LOQB529-05|Chilo|Moth_008.03CL|
AACTTTATACTTTATTTTTGGTATTTGAGCTGGAATAGTAGGAACTTCTTTAAGTTTACTAATTCGTGCAGAATTGGGTA
ATCCTGGATCATTAATTGGAGATGATCAAATTTATAATACTATTGTAACAGCCCATGCATTTATTATAATTTTTTTTATA
GTTATACCAATTATAATTGGAGGATTTGGAAATTGATTAGTTCCTTTAATATTAGGAGCTCCAGATATAGCTTTTCCACG
AATAAATAATATAAGATTTTGACTTCTTCCCCCTTCTTTAACTCTTCTAATTTCTAGAAGAATTGTTGAAAATGGAGCAG
GAACTGGATGAACAGTGTACCCCCCACTTTCTTCTAATATCGCTCACGGAGGAAGATCAGTAGATTTAGCAATTTTTTCA
CTTCACTTAGCTGGTATTTCATCTATTTTAGGTGCTATTAATTTTATTACAACTATTATTAATATACGAATTAATGGATT
ATCTTTTGATCAAATACCTTTATTTGTTTGATCAGTTGGAATTACAGCCTTACTTCTTTTACTTTCTCTTCCAGTATTAG
CTGGAGCTATTACTATACTACTTACAGATCGAAATTTAAATACTTCTTTTTTTGATCCAGCAGGAGGTGGTGATCCTATT
CTTTATCAACATTTATTT---------
>LOQB530-05|Chilo_crossostichus|Moth_009.03CL|
AACTCTATATTTTATCTTTGGTATTTGAGCTGGAATAGTAGGAACTTCTTTAAGTTTATTAATCCGTGCTGAATTAGGAA
ATCCAGGATCATTAATTGGAGATGATCAAATTTATAATACTATTGTAACAGCTCATGCATTTATTATAATTTTTTTTATA
GTTATACCAATTATAATTGGAGGATTTGGAAATTGATTAGTTCCTTTAATATTAGGAGCTCCAGATATAGCTTTTCCACG
AATAAATAATATAAGATTTTGACTTCTTCCCCCTTCTTTAACACTTCTAATTTCTAGAAGAATTGTTGAAAATGGAGCTG
GAACTGGATGAACAGTGTACCCCCCACTTTCTTCTAATATTGCTCACGGAGGAAGCTCTGTAGATTTAGCAATTTTTTCT
CTTCATTTAGCTGGTATTTCATCTATTTTAGGTGCTATTAATTTTATTACAACTATTATTAATATACGAATTAACGGATT
ATCTTTTGATCAAATACCTTTATTTGTATGATCTGTTGGTATTACAGCTTTACTTCTTTTACTTTCTCTTCCAGTATTAG
CTGGAGCTATTACTATACTTCTAACAGATCGAAATTTAAATACTTCTTTCTTTGACCCTGCAGGAGGTGGAGACCCTATT
CTTTACCAACATTTATTT---------
>LOQB531-05|Chilo_crossostichus|Moth_010.03CL|
AACTCTATATTTTATCTTTGGTATTTGAGCTGGAATAGTAGGAACTTCTTTAAGTTTATTAATCCGTGCTGAATTAGGAA
ATCCGGGATCATTAATTGGAGATGATCAAATTTATAATACTATTGTAACAGCTCATGCATTTATTATAATTTTTTTTATA
GTTATACCAATTATAATTGGAGGATTTGGAAATTGATTAGTTCCTTTAATATTAGGAGCTCCAGATATAGCTTTTCCACG
AATAAATAATATAAGATTTTGACTTCTTCCCCCTTCTTTAACACTTCTAATTTCTAGAAGAATTGTTGAAAATGGAGCTG
GAACTGGATGAACAGTGTACCCCCCACTTTCTTCTAATATTGCTCACGGAGGAAGCTCTGTAGATTTAGCAATTTTTTCT
CTTCATTTAGCTGGTATTTCATCTATTTTAGGTGCTATTAATTTTATTACAACTATTATTAATATACGAATTAACGGATT
ATCTTTTGATCAAATACCTTTATTTGTATGATCTGTTGGTATTACAGCTTTACTTCTTTTACTTTCTCTTCCAGTATTAG
CTGGAGCTATTACTATACTTCTAACAGATCGAAATTTAAATACTTCTTTCTTTGACCCTGCAGGAGGTGGAGACCCTATT
CTTTATCAACATTTATTT---------
>LOQB537-05|Chilo_crossostichus|Moth_016.03CL|
AACTCTATATTTTATCTTTGGTATTTGAGCTGGAATAGTAGGAACTTCTTTAAGTTTATTAATCCGTGCTGAATTAGGAA
ATCCAGGATCATTAATTGGAGATGATCAAATTTATAATACTATTGTAACAGCTCATGCATTTATTATAATTTTTTTTATA
GTTATACCAATTATAATTGGAGGATTTGGAAATTGATTAGTTCCTTTAATATTAGGAGCTCCAGATATAGCTTTTCCACG
AATAAATAATATAAGATTTTGACTTCTTCCCCCTTCTTTAACACTTCTAATTTCTAGAAGAATTGTTGAAAATGGAGCTG
GAACTGGATGAACAGTGTACCCCCCACTTTCTTCTAATATTGCTCACGGAGGAAGCTCTGTAGATTTAGCAATTTTTTCT
CTTCATTTAGCTGGTATTTCATCTATTTTAGGTGCTATTAATTTTATTACAACTATTATTAATATGCGAATTAACGGATT
ATCTTTTGATCAAATACCTTTATTTGTATGATCTGTTGGTATTACAGCTTTACTTCTTTTACTTTCTCTTCCAGTATTAG
CTGGAGCTATTACTATACTTCTAACAGATCGAAATTTAAATACTTCTTTCTTTGACCCTGCAGGAGGTGGAGACCCTATT
CTTTATCAACATTTATTT---------
>LOQB541-05|Chilo_crossostichus|Moth_020.03CL|

--GACTTCTCTAAGTTTATTAATCCGTGCTGAATTAGGAA
ATCCGGGATCATTAATTGGAGATGATCAAATTTATAATACTATTGTAACAGCTCATGCATTTATTATAATTTTTTTTATG
GTTATACCAATTATAATTGGAGGATTTGGAAATTGATTAGTTCCTTTAATATTAGGAGCTCCAGATATAGCTTTTCCACG
AATAAATAATATAAGATTTTGACTTCTTCCCCCTTCTTTAACACTTCTAATTTCTAGAAGAATTGTTGAAAATGGAGCTG
GAACTGGATGAACAGTGTACCCCCCACTTTCTTCTAATATTGCTCACGGAGGAAGCTCTGTAGATTTAGCAATTTTTTCT
CTTCATTTAGCTGGTATTTCATCTATTTTAGGTGCTATTAATTTTATTACAACTATTATTAATATACGAATTAACGGATT
ATCTTTTGATCAAATACCTTTATTTGTATGATCTGTTGGTATTACAGCTTTACTTCTTTTACTTTCTCTTCCAGTATTAG
CTGGAGCTATTACTATACTTCTAACAGATCGAAATTTAAATACTTC----------------------------------
----
>LOQB542-05|Chilo_crossostichus|Moth_021.03CL|
--CTGAATTAGGAA
ATCCAGGATCATTAATTGGAGATGATCAAATTTATAATACTATTGTAACAGCTCATGCATTTATTATAATTTTTTTTATA
GTTATACCAATTATAATTGGAGGATTTGGAAATTGATTAGTTCCTTTAATATTAGGAGCTCCAGATATAGCTTTTCCACG
AATAAATAATATAAGATTTTGACTTCTTCCCCCTTCTTTAACACTTCTAATTTCTAGAAGAATTGTTGAAAATGGAGCTG
GAACTGGATGAACAGTGTACCCCCCACTTTCTTCTAATATTGCTCACGGAGGAAGCTCTGTAGATTTAGCAATTTTTTCT
CTTCATTTAGCTGGTATTTCATCTATTTTAGGTGCTATTAATTTTATTACAACTATTATTAATATACGAATTAACGGATT
ATCTTTTGATCAAATACCTTTATTTGTATGATCTGTTGGTATTACAGCTTTACTTCTTTTACTTTCTCTTCCAGTATTAG
CTGGAGCTATTA--

>LOQB547-05|Chilo_crossostichus|Moth_026.03CL|
AACTCTATATTTTATCTTTGGTATTTGAGCTGGAATAGTAGGAACTTCTTTAAGTTTATTAATCCGTGCTGAATTAGGAA
ATCCGGGATCATTAATTGGAGATGATCAAATTTATAATACTATTGTAACAGCTCATGCATTTATTATAATTTTTTTTATA
GTTATACCAATTATAATTGGAGGATTTGGAAATTGATTAGTTCCTTTAATATTAGGAGCTCCAGATATAGCTTTTCCACG
AATAAATAATATAAGATTTTGACTTCTTCCCCCTTCTTTAACACTTCTAATTTCTAGAAGAATTGTTGAAAATGGAGCTG
GAACTGGATGAACAGTGTACCCCCCACTTTCTTCTAATATTGCTCACGGAGGAAGCTCTGTAGATTTAGCAATTTTTTCT
CTTCATTTAGCTGGTATTTCATCTATTTTAGGTGCTATTAATTTTATTACAACTATTATTAATATACGAATTAACGGATT
ATCTTTTGATCAAATACCTTTATTTGTATGATCTGTTGGTATTACAGCTTTACTTCTTTTACTTTCTCTTCCAGTATTAG
CTGGAGCTATTACTATACTTCTAACAGATCGAAATTTAAATACTTCTTTCTTTGACCCTGCAGG----------------
----
>LOQB548-05|Chilo|Moth_027.03CL|
------------------------------------AGTAGGAACTTCTTTAAGTTTACTAATNCGTGCAGAATTAGGTA
ATCCTGGATCATTAATTGGAGATGATCAAATTTATAATACTATTGTAACAGCCCATGCATTTATTATAATTTTTTTTATA
GTTATACCAATTATAATTGGAGGATTTGGAAATTGATTAGTTCCTTTAATATTAGGAGCTCCAGATATAGCTTTTCCACG
AATAAATAATATAAGATTTTGACTTCTTCCCCCTTCTTTAACTCTTCTAATTTCTAGAAGAATTGTTGAAAATGGAGCAG
GAACTGGATGAACAGTGTACCCCCCACTTTCTTCTAATATCGCTCACGGAGGAAGATCAGTAGATTTAGCAATTTTTTCA
CTTCACTTAGCTGGTATTTCATCTATTTTAGGTGCTATTAATTTTATTACAACTATTATTAATATACGAATTAATGGATT
ATCTTTTGATCAAATACCTTTATTTGTTTGATCAGTTGGAATTACAGCCTTACTTCTTTTACTTTCTCTTCCAGTATTAG
CTGGAGCTATTACTATACTAC---

>LOQT854-07|Emmalocera_callirrhoda|gvc6570-1L|
-ACTTTATATTTTATTTTTGGAATTTGATCTGGAATAGTCGGAACATCTTTAAGTCTTCTTATTCGAGCTGAATTAGGAA
CTTCAGGATCTTTAATTGGAGATGATCAAATTTATAATACTATTGTTACTAGTCATGCTTTCATTATAATTTTTTTTATA
GTTATACCAATTATAATTGGAGGATTTGGAAATTGATTAGTTCCTTTAATATTAGGAGCTCCAGATATAGCTTTTCCTCG
AATAAATAATATAAGATTTTGACTTTTACCTCCCTCTCTTACTCTACTTCTTTCTAGAAGAATTGTGGAAAATGGAGCAG
GGACAGGTTGAACTGTTTATCCTCCTTTATCATCTAATATTGCTCACGGGGGAAGTTCTGTAGATTTAGCTATTTTTTCT
CTACATTTAGCTGGAATTTCTTCTATTTTAGGAGCTATTAATTTTATTACCACTATTATTAATATAAAATTAAATGGTTT
ATCTTTTGATCAAATACCTTTATTTGTTTGAGCTGTAGGAATTACAGCTTTATTATTACTTTTATCTTTACCTGTTTTAG
CAGGAGCTATTACTATATTATTAACTGATCGAAATTTAAATACCTCTTTTTTTGACCCTGCTGGAGGAGG----------
----
>LOQT855-07|Emmalocera_callirrhoda|gvc6571-1L|
TACTTTATATTTTATTTTTGGAATTTGATCTGGTATAGTCGGAACATCTTTAAGTCTTCTTATTCGAGCTGAATTAGGAA
CTTCAGGATCTTTAATTGGAGATGATCAAATTTATAATACTATTGTTACTAGTCATGCTTTTATTATAATTTTTTTTATA
GTTATACCAATTATAATTGGAGGATTTGGAAATTGATTAGTTCCTTTAATATTAGGAGCCCCAGATATAGCTTTTCCTCG
AATAAACAATATAAGATTTTGACTTTTACCTCCTTCTCTTACTCTACTTCTCTCTAGAAGAATTGTAGAAAATGGAGCAG
GAACAGGTTGAACTGTTTATCCCCCTTTATCATCTAATATTGCTCACGGAGGAAGTTCTGTAGATTTAGCTATTTTTTCT
CTACATTTAGCTGGAATTTCTTCTATTTTAGGAGCTATTAATTTTATTACTACTATTATTAATATAAAATTAAATGGTTT
ATCTTTTGATCAAATACCTTTATTTGTTTGAGCTGTAGGAATTACAGCTTTATTATTACTTTTATCTTTACCTGTTTTAG

CAGGAGCTATTACTATATTATTAACTGATCGAAATTTAAATACTTCTTTTTTTGATCCTGCTGGAGGAGGAGATCCTATT
TTATATCAACACTTATTT---------
>LOQT1056-07|Scirpophaga|gvc6758-1L|
AACCTTATATTTTATTTTTGGAATTTGAGCTGGTATAATTGGAACTTCTTTAAGTTTATTAATTCGAACTGAATTAGGAA
CTTCAGGTTCTTTAATTGGAGATGATCAAATTTATAACACTATTGTTACAGCTCATGCTTTTATTATAATTTTTTTTATA
GTTATACCTATTATAATTGGAGGATTTGGGAACTGATTAGTACCATTAATATTAGGAGCTCCAGATATAGCATTCCCCCG
TTTAAATAATATAAGATTTTGATTATTACCCCCATCCTTAACTATTTTAATTTCAAGAAGAATTGTAGAAAATGGAGCAG
GAACTGGATGAACTGTATATCCTCCTCTATCTTCTAATATTTCTCATAGAGGAACATCTGTAGATTTAGCTATTTTTTCA
CTTCATTTAGCTGGAATTTCATCTATTTTAGGAGCTATTAATTTTATTACCACTATTATTAATATACGAATTAATGGATT
ATCATTTGATCAAATACCTTTATTTGTTTGAGCTGTTGGTATTACAGCACTTCTTCTTTTATTATCTTTACCAGTTTTAG
CAGGAGCTATTACTATACTTTTAACTGATCGAAATTTAAATACATCTTTTTTTGATCCTGCTGGAGGAGGAGATCCAATT
CTTTATCAACATTTATTT---------
>LOQTB118-07|Emmalocera|gvc6878-1L|
AACTTTATATTTTATTTTTGGTATTTGGTCTGGAATAATTGGTACTTCTTTAAGTCTTCTTATTCGTGCTGAATTAGGAA
CTTCAGGATCTTTAATTGGAGATGATCAAATTTATAATACCATTGTCACTAGTCATGCTTTTATTATAATTTTTTTTATA
GTTATACCTATTATAATTGGAGGATTTGGAAATTGATTAATTCCTTTAATATTAGGAGCTCCAGATATAGCTTTCCCTCG
AATAAATAATATAAGATTTTGACTTTTACCTCCATCTCTTATTTTACTACTTTCTAGTAGAATTGTAGAAAGTGGAGCAG
GAACAGGATGAACTGTTTATCCCCCCTTATCTTCTAATATTGCTCATAGAGGAAGTTCTGTAGATTTAGCTATTTTTTCT
TTACATTTAGCTGGAATTTCCTCTATTTTAGGAGCTATTAATTTTATTACTACTATTATTAATATAAAATTAAATGGTTT
ATCTTTTGATCAAATACCTTTATTTGTTTGAGCAGTTGGAGTTACAACTTTATTACTACTTTTATCTTTACCTGTATTAG
CAGGAGCTATTACCATATTATTAACAGATCGAAATTTAAATACTTCTTTCTTTGACCCAGCTGGAGGAGGAGATCCTATT
TTATACCAACATCTATTT---------
>LOQTB119-07|Emmalocera_callirrhoda|gvc6879-1L|
AACTTTATATTTTATTTTTGGTATTTGATCTGGAATAATTGGTACTTCTTTAAGTCTTCTTATTCGTGCTGAATTAGGAA
CTTCAGGATCTTTAATTGGAGATGATCAAATTTATAATACTATTGTTACTAGTCATGCTTTTATTATAATTTTTTTTATA
GTTATACCTATTATAATTGGAGGATTTGGAAATTGATTAGTTCCTTTAATATTAGGAGCTCCAGATATAGCTTTCCCCCG
AATAAATAATATAAGATTTTGACTTTTACCTCCATCCCTTACTTTACTACTTTCTAGTAGAATTGTAGAAAGTGGGGCAG
GAACAGGATGAACCGTTTATCCTCCTTTATCTTCTAATATTGCCCATAGAGGAAGTTCTGTAGACTTAGCTATTTTTTCT
TTACATTTAGCTGGAATTTCTTCTATTTTAGGAGCTATTAATTTTATTACTACTATTATTAATATAAAATTAAATGGTTT
ATCTTTTGATCAAATACCTTTATTTGTTTGAGCAGTTGGAATTACAGCCTTATTATTACTTTTATCTTTACCTGTATTAG
CAGGAGCTATTACTATATTATTAACAGATCGTAATTTAAATACTTCTTTTTTTGATCCTGCTGGAGGAGGAGATCCTATT
TTATATCAACATTTATTT---------
>LOQTB188-07|Emmalocera|gvc6949-1L|
-------------------------------------TTGGTACTTCTTTAAGTCTTCTTATTCGTGCTGAATTAGGAA
CTTCAGGATCTTTAATTGGAGATGATCAAATTTATAATACCATTGTCACTAGTCATGCTTTTATTATAATTTTTTTTATA
GTTATACCTATTATAATTGGAGGATTTGGAAATTGATTAATTCCTTTAATATTAGGAGCTCCAGATATAGCTTTCCCTCG
AATAAATAATATAAGATTTTGACTTTTACCTCCATCTCTTATTTTACTACTTTCTAGTAGAATTGTAGAAAGTGGAGCAG
GAACAGGATGAACTGTTTATCCCCCCTTATCTTCTAATATTGCTCATAGAGGAAGTTCTGTAGATTTAGCTATTTTTTCT
TTACATTTAGCTGGAATTTCCTCTATTTTAGGAGCTATTAATTTTATTACTACTATTATTAATATAAAATTAAATGGTTT
ATCTTTTGATCAAATACCTTTATTTGTTTGAGCAGTTGGAGTTACAACTTTATTACTACTTTTATCTTTACCTGTATTAG
CAGGAGCTATTACCATATTATTAACAGATCGAAATTTAAATACTTCTTTCTTTGACCCAGCTGGAGGAGGAG--------
----
>LOQTB386-07|Tetramoera_gracilistria|gvc7129-1L|
AACACTATATTTTATTTTCGGAATTTGATCAGGAATAATTGGTACATCTTTAAGATTATTAATTCGAGCTGAATTAGGAA
ACCCCGGATCTTTAATTGGAAATGATCAAATTTATAATACTATTGTAACTGCTCATGCTTTTATTATAATTTTTTTTATA
GTAATGCCTATTATAATTGGAGGATTTGGAAATTGATTAGTTCCTTTAATATTAGGAGCTCCTGATATAGCTTTCCCTCG
TATAAATAACATAAGATTTTGATTACTTCCCCCCTCAATCATATTACTAATCTCAAGAAGAATTGTAGAAAATGGAGCAG
GAACTGGATGAACAGTTTACCCCCCACTATCATCCAATATTGCTCACAGAGGTAGTTCTGTAGATTTAGCTATTTTTTCT
TTACATTTAGCTGGAATTTCTTCTATTTTAGGAGCTGTAAATTTTATTACCACTATTATTAATATACGACCAAATAATAT
AAGATTAGATCAAATACCATTATTTGTTTGAGCTGTTGGTATTACAGCTCTATTATTACTTTTATCTTTACCGGTATTAG
CTGGTGCTATTACTATATTATTAACAGATCGTAATCTGAATACTTCATTTTTTGATCCTGCAGGAGGAGGTGATCCTATT
CTGTATCAACATTTATTT---------
>LOQTC158-07|Tetramoera_gracilistria|gvc7804-1L|
AACACTATATTTTATTTTCGGAATTTGATCAGGAATAATTGGTACATCTTTAAGATTATTAATTCGAGCTGAATTAGGAA
ACCCCGGATCTTTAATTGGAGATGATCAAATTTATAATACTATTGTAACTGCTCATGCTTTTATTATAATTTTTTTTATA
GTAATGCCTATTATAATTGGAGGATTTGGAAATTGATTAGTTCCTTTAATATTAGGAGCTCCTGATATAGCTTTCCCTCG
TATAAATAACATAAGATTTTGATTACTTCCCCCCTCAATCATATTACTAATCTCAAGAAGAATTGTAGAAAATGGAGCAG

GAACTGGATGAACAGTTTACCCCCCACTATCATCCAATATTGCTCACAGAGGTAGTTCTGTAGATTTAGCTATTTTTTCT
TTACATTTAGCTGGAATTTCTTCTATTTTAGGAGCTGTAAATTTTATTACCACTATTATTAATATACGACCAAATAATAT
AAGATTAGATCAAATACCATTATTTGTTTGAGCTGTTGGTATTACAGCTCTATTATTACTTTTATCTTTACCGGTATTAG
CTGGTGCTATTACTATATTATTAACAGATCGTAATCTGAATACTTCATTTTTTGATCCTGCAGGAGGAGGTGATCCTATT
CTATATCAACATTTATTT---------
>LOQTC752-08|Emmalocera|gvc8396-1L|
TACTTTATATTTTATTTTTGGAATTTGATCTGGAATAATTGGTACATCATTAAGACTCCTTATTCGTGCTGAATTAGGAA
CTTCAGGTTCTTTAATTGGAGATGATCAAATTTATAATACTATTGTTACTAGACATGCTTTTATTATAATTTTTTTTATA
GTTATACCAATTATAATTGGAGGATTTGGTAATTGATTGGTTCCTTTAATATTAGGAGCTCCAGATATAGCTTTTCCTCG
AATAAATAATATAAGATTTTGACTTTTACCTCCTTCTCTTACTTTATTACTTTCTAGAAGAATTGTAGAAAATGGAGCTG
GAACAGGTTGAACTGTTTACCCACCTTTATCTTCTAATATTGCTCATGGAGGTAGATCTGTAGATTTAGCTATTTTTTCT
CTTCATTTAGCAGGAATTTCTTCTATTTTAGGAGCTATTAATTTTATTACAACTATTATTAATATAAAATTAAATGGATT
ATCTTTTGATCAAATACCTTTATTTGTTTGAGCTGTAGGAATTACAGCTTTATTATTATTATTATCTTTACCTGTATTAG
CAGGAGCTATTACTATATTATTAACTGATCGAAATTTAAATACTTCTTTTTTTGATCCTGCTGGAGGAGGAGATCCTATT
TTATACCAGCATTTATTT---------
>LOQTC884-08|Scirpophaga|gvc8531-1L|
AACCTTATATTTTATTTTTGGAATTTGAGCTGGTATAATTGGAACTTCTTTAAGTTTATTAATTCGAACTGAATTAGGAA
CTTCAGGTTCTTTAATTGGAGATGATCAAATTTATAACACTATTGTTACAGCTCATGCTTTTATTATAATTTTTTTTATA
GTTATACCTATTATAATTGGAGGATTTGGGAACTGATTAGTACCATTAATATTAGGAGCTCCAGATATAGCATTCCCCCG
TTTAAATAATATAAGATTTTGATTATTACCCCCATCCTTAACTATTTTAATTTCAAGAAGAATTGTAGAAAATGGAGCAG
GAACTGGATGAACTGTATATCCTCCTCTATCTTCTAATATTTCTCATAGAGGAACATCTGTAGATTTAGCTATTTTTTCA
CTTCATTTAGCTGGAATTTCATCTATTTTAGGAGCTATTAATTTTATTACCACTATTATTAATATACGAATTAATGGATT
ATCATTTGATCAAATACCTTTATTTGTTTGAGCTGTTGGTATTACAGCACTTCTTCTTTTATTATCTTTACCAGTTTTAG
CAGGAGCTATTACTATACTTTTAACTGATCGAAATTTAAATACATCTTTTTTTGATCCTGCTGGAGGAGGAGATCCAATT
CTTTATCAACATTTATTT---------
>LOQTD063-08|Emmalocera|gvc8662-1L|
AACTTTATATTTTATTTTTGGTATTTGGTCTGGAATAATTGGTACTTCTTTAAGTCTTCTTATTCGTGCTGAATTAGGAA
CTTCAGGATCTTTAATTGGAGATGATCAAATTTATAATACCATTGTCACTAGTCATGCTTTTATTATAATTTTTTTTATA
GTTATACCTATTATAATTGGAGGATTTGGAAATTGATTAGTTCCTTTAATATTAGGGGCTCCAGATATAGCTTTCCCTCG
AATAAATAATATAAGATTTTGACTTTTACCTCCATCTCTTACTTTACTACTTTCTAGTAGAATTGTAGAAAGTGGAGCAG
GAACAGGATGAACTGTTTATCCCCCCTTATCTTCTAATATTGCTCATAGAGGAAGTTCTGTAGATTTAGCTATTTTTTCT
TTACATTTAGCTGGAATTTCCTCTATTTTAGGGGCTATTAATTTTATTACTACTATTATTAATATAAAATTAAATGGTTT
ATCTTTTGATCAAATACCTTTATTTGTTTGAGCAGTTGGAATTACAACTTTATTACTACTTTTATCTTTACCTGTATTAG
CAGGAGCTATTACCATATTATTAACAGATCGAAATTTAAATACTTCTTTCTTTGACCCAGCTGGAGGAGGAGATCCTATT
TTATACCAACATCTATTT---------
>LOQTD200-08|Scirpophaga|gvc8803-1L|
AACTTTATATTTTATTTTTGGAATTTGAGCTGGTATAATTGGAACTTCTTTAAGTTTATTAATTCGAACTGAATTAGGAA
CTTCAGGTTCTTTAATCGGAGATGATCAAATTTATAATACTATTGTTACAGCTCATGCTTTTATTATAATTTTTTTCATA
GTTATACCTATTATAATTGGAGGATTTGGAAACTGATTAGTACCATTAATATTAGGAGCTCCAGATATAGCATTCCCCCG
TTTAAATAATATAAGATTTTGATTATTACCNCCATCNTTAACTATTTTAATTTCAAGAAGAATTGTAGAAAATGGAGCAG
GAACTGGATGAACTGTATATCCTCCTCTATCTTCTAATATATCTCATAGAGGAACATCTGTAGATTTAGCTATTTTTTCA
CTTCATTTAGCTGGAATTTCATCTATCTTAGGAGCTATTAATTTTATTACNACTATTATTAATATACGAATTAATGGATT
ATCATTTGATCAAATACCTTTATTTGTTTGAGCTGTTGGTATTACAGCACTTCTTCTTTTATTATCTTTACCAGTTTTAG
CAGGAGCCATTACTATACTTTTAACTGATCGAAATTTAAATACATCTTTTTTTGATCCTGCTGGAGGAGGAGATCCAATT
CTTTATCAACATTTATTT---------
>LOQTI438-11|Scirpophaga_nivella|gvc16526-1L|
AACTTTATATTTCATTTTTGGAATTTGAGCTGGTATAGTAGGAACTTCTTTAAGATTATTAATTCGAGCTGAATTAGGAA
CTCCAGGATCTTTAATTGGAGATGATCAAATTTATAATACCATTGTTACAGCTCATGCTTTTATTATAATTTTTTTTATA
GTAATACCAATTATAATTGGAGGGTTTGGAAATTGACTTGTTCCTTTAATATTAGGAGCTCCTGATATAGCTTTCCCTCG
TATAAATAATATAAGATTTTGATTATTACCCCCCTCATTAACTCTCCTAATTTCAAGAAGAATTGTAGAAAATGGTGCAG
GAACAGGATGAACAGTATACCCCCCCTTATCATCAAATATTGCTCATGGAGGAACTTCTGTAGATTTAGCTATTTTTTCT
CTACATCTTGCAGGAATTTCCTCTATTTTAGGAGCTATTAACTTTATTACCACTATTATTAATATACGAATTAATGGCTT
AACATTTGATCAAATACCTCTCTTTATTTGAGCTGTTGGAATTACAGCCCTTCTTTTACTCCTCTCATTACCCGTATTAG
CTGGAGCTATTACTATATTATTAACTGATCGAAATTTAAATACCTCTTTTTTTGATCCAGCGGGAGGAGGAGATCCAATC
CTTTATCAACATTTATTT---------
>LPOKA067-08|Diatraea_evanescens|MDOK-0067|
AACTTTATATTTTATTTTTGGAATTTGAGCTGGAATACTGGGAACATCTTTAAGTTTATTAATTCGTGCAGAATTAGGTA

CAACTAATTCTTTAATTGGAGATGATCAAATTTACAATACAATTGTTACAGCTCATGCATTTATTATAATTTTTTTATA
GTTATACCCATTATAATTGGGGGATTTGGAAACTGATTAGTACCATTAATATTAGGAGCTCCTGATATAGCTTTCCCTCG
AATAAATAATATAAGATTTTGATTACTACCCCCATCTTTAACTTTATTAATTTCTAGAAGAATTGTTGAAAATGGAGCTG
GAACAGGATGAACAGTTTACCCCCCACTTTCATCAAATATTGCTCATGGTGGTAGATCTGTAGACCTAGCAATTTTTTCA
TTACATTTAGCTGGAATCTCATCAATTTTAGGAGCTATTAATTTTATTACCACAATTATTAATATACGAATTAATGGATT
ATCATTCGATCAAATACCTCTATTTGTATGATCAGTAGGTATTACAGCCTTATTATTACTTTTATCTTTACCTGTTTTAG
CTGGAGCTATTACCATATTATTAACAGATCGAAACTTAAATACTTCCTTTTTCGACCCAGCTGGAGGGGGGG--------
----
>LSAFA608-13|Acrapex_aenigma|BIOUG06232-H03|
AACATTATATTTTATTTTTGGAATTTGAGCTGGTATAGTAGGAACTTCATTAAGTTTATTAATTCGAGCTGAATTAGGAA
CTCCAGGATCTTTAATTGGAGATGATCAAATTTATAATACTATTGTTACAGCTCATGCTTTTATTATAATTTTTTTATA
GTTATACCTATTATAATTGGAGGATTTGGTAATTGACTTGTACCATTAATATTGGGAGCACCTGATATAGCATTCCCTCG
AATAAATAATATAAGTTTTTGACTACTTCCTCCTTCTTTAACCCTTTTAATTTCAAGAAGAATTGTAGAAAATGGAGCAG
GAACTGGATGAACAGTTTACCCCCCACTGTCATCTAATATCGCTCATGGAGGAAGTTCTGTAGATTTAGCTATTTTTTCT
CTTCATTTAGCTGGAATCTCTTCTATTTTAGGAGCTATTAATTTTATCACAACAATTATTAATATACGACTTAATAGTTT
ATCTTTTGATCAAATACCTTTATTTATTTGAGCT---

>LSAFR682-12|Acrapex_aenigma|BIOUG02070-B05|
AACATTATATTTTATTTTTGGAATTTGAGCTGGTATAGTAGGAACTTCATTAAGTTTATTAATTCGAGCTGAATTAGGAA
CTCCAGGATCTTTAATTGGAGATGATCAAATTTATAATACTATTGTTACAGCTCATGCTTTTATTATAATTTTTTTATA
GTTATACCTATTATAATTGGAGGATTTGGTAATTGACTTGTGCCATTAATATTGGGAGCACCTGATATAGCATTCCCTCG
AATAAATAATATAAGTTTTTGACTACTTCCTCCTTCTTTAACCCTTTTAATTTCAAGAAGAATTGTAGAAAATGGAGCAG
GAACTGGATGAACAGTTTACCCCCCACTGTCATCTAATATCGCTCATGGAGGAAGTTCTGTAGATTTAGCTATTTTTTCT
CTTCATTTAGCTGGAATCTCTTCTATTTTAGGAGCTATTAATTTTATCACAACAATTATTAATATACGACTTAATAGTTT
ATCTTTTGATCAAATACCTTTATTTATTTGAGCTGTAGGAATCACTGCATTTTTATTATTATTATCTTTACCAGTTTTAG
CTGGAGCTATTACTATATTATTAACTGATCGAAATTTAAATACATCATTTTTTGATCCTGCTGGGGGAGGAGATCCAATT
TTATATCAACATTTATTT---------
>LSAFR918-12|Tetramoera|BIOUG02073-F03|
AACATTATATTTTATTTTTGGAATTTGATCAGGAATAATTGGTACATCTTTAAGATTATTAATTCGAGCTGAATTAGGAA
ATCCTGGATCTTTAATTGGAGATGACCAAATTTATAATACTATTGTAACTGCTCATGCTTTTATTATAATTTTTTTATA
GTTATACCTATTATAATTGGAGGATTTGGAAATTGATTAGTTCCTTTAATATTAGGAGCTCCTGATATAGCTTTTCCTCG
TATAAATAATATAAGATTTTGATTACTTCCCCCCTCTATTATATTATTAATCTCAAGAAGAATTGTAGAAAATGGAGCAG
GAACTGGATGAACAGTTTACCCCCCACTATCATCTAATATTGCTCATAGAGGTAGATCTGTAGATTTAGCTATTTTCTCC
CTGCATTTAGCTGGAATTTCTTCTATTTTAGGAGCTGTAAACTTTATTACTACTATTATCAATATACGACCAAATAATAT
AAGATTAGACCAAATACCTTTATTTGTTTGAGCTGTTGGTATTACAGCCCTTTTATTACTTTTATCTTTACCAGTATTAG
CTGGTGCTATTACTATACTTTTAACAGACCGTAATCTAAATACATCATTTTTCGATCCTGCTGGAGGAGGTGACCCTATT
TTATATCAACACTTATTT---------
>LSAFR1191-12|Acrapex_aenigma|BIOUG02076-E03|
AACATTATATTTTATTTTTGGAATTTGAGCTGGTATAGTAGGAACTTCATTAAGTTTATTAATTCGAGCTGAATTAGGAA
CTCCAGGATCTTTAATTGGAGATGATCAAATTTATAATACTATTGTTACAGCTCATGCTTTTATTATAATTTTTTTATA
GTTATACCTATTATAATTGGAGGATTTGGTAATTGACTTGTACCATTAATATTGGGAGCACCTGATATAGCATTCCCTCG
AATAAATAATATAAGTTTTTGACTACTTCCTCCTTCTTTAACCCTTTTAATTTCAAGAAGAATTGTAGAAAATGGAGCAG
GAACTGGATGAACAGTTTACCCCCCACTGTCATCTAATATCGCTCATGGAGGAAGTTCTGTAGATTTAGCTATTTTTTCT
CTTCATTTAGCTGGAATCTCTTCTATTTTAGGAGCTATTAATTTTATCACAACAATTATTAATATACGACTTAATAGTTT
ATCTTTTGATCAAATACCTTTATTTATTTGAGCTGTAGGAATCACTGCATTTTTATTATTATTATCTTTACCAGTTTTAG
CTGGAGCTATTACTATATTATTAACTGATCGAAATTTAAATACATCATTTTTTGATCCTGCTGGGGGAGGAGATCCAATT
TTATATCAACATTTATTT---------
>LSAFR1257-12|Acrapex_aenigma|BIOUG02077-B10|
AACATTATATTTTATTTTTGGAATCTGAGCTGGTATAGTAGGAACTTCATTAAGTTTATTAATTCGAGCTGAATTAGGAA
CCCCGGGATCTTTAATTGGAGATGATCAAATTTATAATACTATTGTTACAGCTCATGCTTTTATTATAATTTTTTTATA
GTTATACCTATTATAATCGGAGGATTTGGTAATTGACTTGTACCTTTAATATTAGGAGCACCTGATATAGCATTTCCTCG
AATAAATAATATAAGTTTTTGACTACTTCCCCCTTCTTTAACCCTTTTAATTTCAAGAAGAATTGTAGAAAATGGAGCAG
GAACTGGATGAACAGTTTACCCCCCACTGTCATCTAATATCGCTCATGGAGGAAGTTCTGTAGATTTAGCTATTTTTTCT
CTTCATTTAGCTGGAATTTCTTCTATTTTAGGAGCTATTAATTTTATCACAACAATTATTAATATACGACTTAATAGTTT
ATCTTTTGATCAAATACCTTTATTTATTTGAGCTGTAGGAATTACTGCATTTTTATTATTATTATCTTTACCAGTTTTAG
CTGGAGCTATTACTATATTATTAACTGATCGAAATTTAAATACATCATTTTTTGATCCTGCTGGAGGAGGAGATCCAATT

TTATATCAACATTTATTT---------
>LSAFR1388-12|Acrapex_minima|BIOUG02078-E10|
TACATTATATTTTATTTTTGGAATTTGAGCCGGTATAGTAGGAACTTCTTTAAGATTATTAATTCGAGCTGAGCTAGGAA
CTCCAGGATCTTTAATTGGAGATGATCAAATTTATAATACTATTGTTACAGCTCATGCTTTTATTATAATTTTTTTATA
GTTATACCAATTATAATTGGAGGATTTGGAAATTGACTTGTACCTTTAATATTAGGGGCACCAGACATAGCATTCCCTCG
AATAAATAATATAAGTTTTTGATTATTACCCCCCTCTTTAACTCTTTTAATTTCCAGAAGAATCGTAGAAAATGGGGCAG
GAACTGGGTGAACTGTATACCCCCCTTTATCATCTAATATTGCCCATGGAGGAAGTTCTGTAGATTTAGCTATTTTTTCT
CTTCATTTAGCTGGAATCTCATCTATTTTAGGAGCTATTAATTTTATCACTACAATTATTAATATACGATTAAATAACTT
ATCTTTTGATCAAATACCTTTATTTATTTGAGCTGTAGGTATTACTGCATTTTTATTATTATTATCTTTACCTGTTTTAG
CAGGAGCTATTACTATATTATTAACAGATCGAAATTTAAATACTTCTTTTTTTGACCCTGCTGGAGGAGGAGATCCTATT
TTATATCAACATTTATTT---------
>LSAFR1389-12|Acrapex_aenigma|BIOUG02078-E11|
AACATTATATTTTATTTTTGGAATTTGAGCTGGTATAGTAGGAACTTCATTAAGTTTATTAATTCGAGCTGAATTAGGAA
CTCCAGGATCTTTAATTGGAGATGATCAAATTTATAATACTATTGTTACAGCTCATGCTTTTATTATAATTTTTTTATA
GTTATACCTATTATAATTGGAGGATTTGGTAATTGACTTGTACCATTAATATTGGGAGCACCTGATATAGCATTCCCTCG
AATAAATAATATAAGTTTTTGACTACTTCCTCCTTCTTTAACCCTTTTAATTTCAAGAAGAATTGTAGAAAATGGAGCAG
GAACTGGATGAACAGTTTACCCCCCACTGTCATCTAATATCGCTCATGGAGGAAGTTCTGTAGATTTAGCTATTTTTTCT
CTTCATTTAGCTGGAATCTCTTCTATCTTAGGAGCTATTAATTTTATCACAACAATTATTAATATACGACTTAATAGTTT
ATCTTTTGATCAAATACCTTTATTTATTTGAGCTGTAGGAATCACTGCATTTTTATTATTATTATCTTTACCAGTTTTAG
CTGGAGCTATTACTATATTATTAACTGATCGAAATTTAAATACATCATTTTTTGATCCTGCTGGGGGAGGAGATCCAATT
TTATATCAACATTTATTT---------
>LSAFR1402-12|Acrapex_minima|BIOUG02078-F12|
TACATTATATTTTATTTTTGGAATTTGAGCCGGTATGGTAGGAACTTCTTTAAGATTATTAATTCGAGCTGAATTAGGAA
CTCCAAGATCTTTAATTGGAGATGATCAAATTTATAATACTATTGTTACAGCTCATGCTTTTATTATAATTTTTTTATA
GTTATACCAATTATAATTGGAGGATTTGGAAATTGACTTGTACCTTTAATATTAGGGGCACCAGACATAGCATTCCCTCG
AATAAATAATATAAGTTTTTGATTATTACCCCCCTCTTTAACTCTTTTAATTTCCAGAAGAATCGTAGAAAATGGGGCAG
GAACTGGGTGAACTGTATACCCCCCTTTATCATCTAATATTGCCCATGGAGGAAGTTCTGTAGATTTAGCTATTTTTTCT
CTTCATTTAGCTGGAATCTCATCTATTTTAGGCGCTATTAATTTTATCACTACAATTATTAATATACGATTAAATAACTT
ATCTTTTGATCAAATACCTTTATTTATTTGAGCTGTAGGTATTACTGCATTTTTATTATTACTATCTTTACCTGTTTTAG
CAGGAGCTATTACTATATTATTAACAGATCGAAATTTAAATACTTCTTTTTTTGACCCTGCTGGAGGAGGAGATCCTATT
TTATATCAACATTTATTT---------
>LSAFR1444-12|Acrapex_albivena|BIOUG02079-B07|
TACATTATATTTTATTTTTGGGATTTGAGCCGGTATAGTAGGAACTTCTTTAAGATTATTAATTCGAGCTGAACTAGGAA
CTCCAGGATCTTTAATTGGAGATGATCAAATTTATAATACTATTGTTACAGCCCATGCTTTTATTATAATTTTTTTATA
GTTATACCAATTATAATTGGGGGATTTGGAAATTGACTTGTACCTTTAATATTGGGGGCACCAGATATAGCATTTCCTCG
AATAAATAATATAAGTTTTTGATTATTACCCCCCTCTTTAACTCTTTTAATTTCCAGAAGAATCGTAGAAAATGGGGCAG
GAACTGGGTGAACTGTATACCCTCCTTTATCATCTAATATTGCCCATGGGGGAAGTTCTGTAGATTTAGCTATTTTTTCT
CTTCATTTAGCTGGGATCTCATCTATTTTAGGTGCTATTAATTTTATCACTACAATTATTAATATACGATTAAATAATTT
ATCTTTTGATCAAATACCTTTATTTATTTGAGCTGTAGGTATTACTGCATTCTTATTATTACTATCTTTACCTGTTTTAG
CAGGAGCTATTACTATGTTACTAACAGATCGAAATTTAAATACTTCTTTTTTTGACCCTGCTGGAGGGGGAGATCCTATT
TTATATCAACACTTATTT---------
>LSAFR1458-12|Acrapex_minima|BIOUG02079-C09|
TACATTATATTTTATTTTTGGAATTTGAGCCGGTATGGTAGGAACTTCTTTAAGATTATTAATTCGAGCTGAATTAGGAA
CTCCAAGATCTTTAATTGGAGATGATCAAATTTATAATACTATTGTTACAGCTCATGCTTTTATTATAATTTTTTTATA
GTTATACCAATTATAATTGGAGGATTTGGAAATTGACTTGTACCTTTAATATTAGGGGCACCAGACATAGCATTCCCTCG
AATAAATAATATAAGTTTTTGATTATTACCCCCCTCTTTAACTCTTTTAATTTCCAGAAGAATCGTAGAAAATGGGGCAG
GAACTGGGTGAACTGTATACCCCCCTTTATCATCTAATATTGCCCATGGAGGAAGTTCTGTAGATTTAGCTATTTTTTCT
CTTCATTTAGCTGGAATCTCATCTATTTTAGGCGCTATTAATTTTATCACTACAATTATTAATATACGATTAAATAACTT
ATCTTTTGATCAAATACCTTTATTTATTTGAGCTGTAGGTATTACTGCATTTTTATTATTACTATCTTTACCTGTTTTAG
CAGGAGCTATTACTATATTATTAACAGATCGAAATTTAAATACTTCTTTTTTTGACCCTGCTGGAGGAGGAGATCCTATT
TTATATCAACATTTA------------
>LSAFR1492-12|Acrapex_aenigma|BIOUG02079-F07|
AACATTATATTTTATTTTTGGAATTTGAGCTGGTATAGTAGGAACTTCATTAAGTTTATTAATTCGAGCTGAATTAGGAA
CTCCAGGATCTTTAATTGGAGATGATCAAATTTATAATACTATTGTTACAGCTCATGCTTTTATTATAATTTTTTTATA
GTTATACCTATTATAATTGGAGGATTTGGTAATTGACTTGTACCATTAATATTGGGAGCACCTGATATAGCATTCCCTCG
AATAAATAATATAAGTTTTTGACTACTTCCTCCTTCTTTAACCCTTTTAATTTCAAGAAGAATTGTAGAAAATGGAGCAG
GAACTGGATGAACAGTTTACCCCCCACTGTCATCTAATATCGCTCATGGAGGAAGTTCTGTAGATTTAGCTATTTTTTCT

CTTCATTTAGCTGGAATCTCTTCTATTTTAGGAGCTATTAATTTTATCACAACAATTATTAATATACGACTTAATAGTTT
ATCTTTTGATCAAATACCTTTATTTATTTGAGCTGTAGGAATCACTGCATTTTTATTATTATTATCTTTACCAGTTTTAG
CTGGAGCTATTACTATATTATTAACTGATCGAAATTTAAATACATCATTTTTTGATCCTGCTGGGGGAGGAGATCCAATT
TTATATCAACATTTATTT---------
>LSAFR2106-12|Sesamia|BIOUG02086-B04|
AACATTATATTTTATTTTTGGAATTTGAGCCGGAATAGTAGGAACTTCATTAAGACTATTAATTCGAGCTGAATTAGGAA
CTCCTGGATCTTTAATTGGAGATGATCAAATTTACAATACTATTGTTACAGCTCATGCTTTTATTATAATTTTTTTTATA
GTAATACCTATTATAATTGGAGGATTTGGAAATTGACTTGTACCTTTAATATTAGGAGCCCCAGATATAGCATTTCCACG
AATAAATAATATAAGATTTTGATTATTACCACCATCTTTAACTCTTTTAATTTCAAGTAGAATTGTAGAAAATGGAGCAG
GAACAGGATGAACAGTTTACCCTCCACTTTCATCTAATATTGCTCATGGGGGGAGATCTGTAGATTTAGCCATTTTTTCC
CTTCATTTAGCTGGAATTTCATCTATTCTAGGAGCTATTAATTTTATTACAACAATTATTAATATACGATTAAATAATTT
ATCATTTGATCAAATACCATTATTTATTTGAGCTGTTGGAATTACTGCTTTTTTATTACTACTATCATTACCCGTTTTAG
CAGGAGCTATTACTATATTACTTACAGATCGAAATTTAAATACATCATTTTTTGACCCCGCAGGAGGAGGGGATCCAATT
TTATATCAACACTTATTT---------
>LSM259-11|Bathytricha_truncata|K290835|
AACATTATATTTCATCTTTGGAATTTGAGCAGGAATAGTAGGAACTTCTTTAAGACTATTAATTCGAGCTGAATTAGGAA
CTCCTGGATCCTTAATTGGAGATGATCAAATTTATAATACTATTGTAACAGCTCATGCCTTTATTATAATTTTTTTTATA
GTTATACCAATTATAATCGGGGGATTTGGAAATTGACTTGTACCTTTAATGTTAGGAGCGCCTGATATGGCATTTCCACG
AATAAATAATATAAGTTTTTGATTACTACCCCCTTCTTTAACTCTACTTATTTCGAGAAGAGTTGTAGAAAATGGAGCAG
GAACTGGATGAACAGTATACCCTCCACTTTCATCTAATATTGCCCATAGAGGAAGATCTGTAGACTTAGCTATTTTTTCC
CTTCATTTAGCTGGAATTTCTTCTATTTTAGGAGCTATTAATTTTATTACAACTATTATTAACATACGATTAAATAATTT
ATCTTTTGATCAAATACCTTTATTTATTTGAGCTGTAGGAATTACAGCATTTTTATTATTATTATCATTACCTGTCTTAG
CTGGAGCTATTACCATATTATTAACAGATCGAAATTTAAATACATCATTTTTTGATCCTGCAGGAGGAGGAGATCCAATC
TTATATCAACATTTATTT---------
>LSM710-11|Bathytricha_monticola|K287609|
AACATTATATTTTATTTTTGGAATTTGAGCAGGAATAGTAGGAACCTCTTTAAGACTATTAATTCGAGCTGAATTAGGAA
CACCTGGATCTTTAATTGGAGATGATCAAATTTATAATACTATTGTAACAGCTCATGCTTTTATTATAATTTTTTTTATA
GTTATACCAATTATAATTGGAGGGTTTGGAAATTGACTTGTACCTTTAATATTAGGAGCACCTGATATAGCATTCCCACG
AATAAATAATATAAGTTTTTGATTACTTCCACCCTCTTTAACTCTCCTTATTTCAAGTAGAATTGTAGAAAATGGGGCAG
GAACTGGATGAACAGTTTACCCACCACTCTCATCTAATATTGCTCATGGAGGAAGATCTGTAGACTTAGCTATTTTTTCT
CTCCATTTGGCGGGAATCTCTTCTATTCTAGGAGCTATTAATTTTATTACAACTATTATTAATATACGATTAAATAGTTT
ATCTTTTGATCAAATACCTTTATTTATTTGAGCCGTAGGTATTACAGCATTTTTATTATTATTATCATTACCTGTATTAG
CTGGAGCTATTACTATACTATTAACAGATCGAAATTTAAATACATCATTTTTTGATCCTGCAGGAGGAGGAGATCCAATT
TTATATCAACATTTATTT---------
>LSM711-11|Bathytricha_monticola|K287610|
AACATTATATTTTATTTTTGGAATTTGAGCAGGAATAGTAGGAACCTCTTTAAGACTATTAATTCGAGCTGAATTAGGAA
CACCTGGATCTTTAATTGGAGATGATCAAATTTATAATACTATTGTAACAGCTCATGCTTTTATTATAATTTTTTTTATA
GTTATACCAATTATAATTGGAGGGTTTGGAAATTGACTTGTACCTTTAATATTAGGAGCACCTGATATAGCATTCCCACG
AATAAATAATATAAGTTTTTGATTACTTCCACCCTCTTTAACTCTCCTTATTTCAAGTAGAATTGTAGAAAATGGGGCAG
GAACTGGATGAACAGTTTACCCACCACTCTCATCTAATATTGCTCATGGAGGAAGATCTGTAGACTTAGCTATTTTTTCT
CTCCATTTGGCGGGAATCTCTTCTATTCTAGGAGCTATTAATTTTATTACAACTATTATTAATATACGATTAAATAGTTT
ATCTTTTGATCAAATACCTTTATTTATTTGAGCCGTAGGTATTACAGCATTTTTATTATTATTATCATTACCTGTATTAG
CTGGAGCTATTACTATACTATTAACAGATCGAAATTTAAATACATCATTTTTTGATCCTGCAGGAGGAGGAGATCCAATT
TTATATCAACATTTATTT---------
>LSM712-11|Bathytricha_monticola|K287611|
AACATTATATTTTATTTTTGGAATTTGAGCAGGAATAGTAGGAACCTCTTTAAGACTATTAATTCGAGCTGAATTAGGAA
CACCTGGATCTTTAATTGGAGATGATCAAATTTATAATACTATTGTAACAGCTCATGCTTTTATTATAATTTTTTTTATA
GTTATACCAATTATAATTGGAGGGTTTGGAAATTGACTTGTACCTTTAATATTAGGAGCACCTGATATAGCATTCCCACG
AATAAATAATATAAGTTTTTGATTACTTCCACCCTCTTTAACTCTCCTTATTTCAAGTAGAATTGTAGAAAATGGGGCAG
GAACTGGATGAACAGTTTACCCACCACTCTCATCTAATATTGCTCATGGAGGAAGATCTGTAGACTTAGCTATTTTTTCT
CTCCATTTGGCGGGAATCTCTTCTATTCTAGGAGCTATTAATTTTATTACAACTATTATTAATATACGATTAAATAGTTT
ATCTTTTGATCAAATACCTTTATTTATTTGAGCCGTAGGTATTACAGCATTTTTATTATTATTATCATTACCTGTATTAG
CTGGAGCTATTACTATACTATTAACAGATCGAAATTTAAATACATCATTTTTTGATCCTGCAGGAGGAGGAGATCCAATT
TTATATCAACATTTATTT---------
>LSM713-11|Bathytricha_monticola|K287612|
AACATTATATTTTATTTTTGGAATTTGAGCAGGAATAGTAGGAACCTCTTTAAGACTATTAATTCGAGCTGAATTAGGAA
CACCTGGATCTTTAATTGGAGATGATCAAATTTATAATACTATTGTAACAGCTCATGCTTTTATTATAATTTTTTTTATA

GTTATACCAATTATAATTGGAGGGTTTGGAAATTGACTTGTACCTTTAATATTAGGAGCACCTGATATAGCATTCCCACG
AATAAATAATATAAGTTTTTGATTACTTCCACCCTCTTTAACTCTCCTTATTTCAAGTAGAATTGTAGAAAATGGGGCAG
GAACTGGATGAACAGTTTACCCACCACTCTCATCTAATATTGCTCATGGAGGAAGATCTGTAGACTTAGCTATTTTTTCT
CTCCATTTGGCGGGAATCTCTTCTATTCTAGGAGCTATTAATTTTATTACAACTATTATTAATATACGATTAAATAGTTT
ATCTTTTGATCAAATACCTTTATTTATTTGAGCCGTAGGTATTACAGCATTTTTATTATTATTATCATTACCTGTATTAG
CTGGAGCTATTACTATACTATTAACAGATCGAAATTTAAATACATCATTTTTTGATCCTGCAGGAGGAGGAGATCCAATT
TTATATCAACATTTATTT---------
>LSM714-11|Bathytricha|K287613|
AACATTATATTTCATTTTTGGAATTTGAGCAGGAATAGTAGGAACTTCTTTAAGATTATTAATTCGAGCTGAATTAGGAA
CTCCTGGATCTTTAATTGGAGATGACCAAATTTATAATACTATTGTAACGGCTCATGCCTTTATTATAATTTTTTTTATA
GTTATACCAATTATAATTGGAGGATTTGGAAATTGACTTGTACCTTTAATATTAGGAGCACCTGATATAGCATTCCCACG
AATAAATAATATGAGTTTTTGATTACTTCCACCCTCTTTAACTCTCCTTATTTCAAGTAGAGTTGTAGAAAATGGAGCAG
GAACTGGATGAACAGTTTACCCCCCACTCTCATCTAATATTGCTCATGGAGGAAGATCTGTAGACTTAGCTATTTTTTCC
CTTCATTTAGCTGGAATCTCTTCTATTCTAGGAGCTATTAATTTTATTACAACTATTATTAATATACGATTAAATAGTTT
ATCTTTTGATCAAATACCTTTATTTATTTGAGCTGTAGGAATTACAGCATTTTTATTATTATTATCATTACCTGTATTAG
CTGGAGCTATTACTATATTATTAACTGATCGAAATTTAAACACATCATTTTTCGATCCTGCAGGAGGGGGGGATCCAATT
TTATATCAACATTTATTT---------
>LSM715-11|Bathytricha_monticola|K287614|
AACATTATATTTTATTTTTGGAATTTGAGCAGGAATAGTAGGAACCTCTTTAAGACTATTAATTCGAGCTGAATTAGGAA
CACCTGGATCTTTAATTGGAGATGATCAAATTTATAATACTATTGTAACAGCTCATGCTTTTATTATAATTTTTTTTATA
GTTATACCAATTATAATTGGAGGGTTTGGAAATTGACTTGTACCTTTAATATTAGGAGCACCTGATATAGCATTCCCACG
AATAAATAATATAAGTTTTTGATTACTTCCACCCTCTTTAACTCTCCTTATTTCAAGTAGAATTGTAGAAAATGGGGCAG
GAACTGGATGAACAGTTTACCCACCACTCTCATCTAATATTGCTCATGGAGGAAGATCTGTAGACTTAGCTATTTTTTCT
CTCCATTTGGCGGGAATCTCTTCTATTCTAGGAGCTATTAATTTTATTACAACTATTATTAATATACGATTAAATAGTTT
ATCTTTTGATCAAATACCTTTATTTATTTGAGCCGTAGGTATTACAGCATTTTTATTATTATTATCATTACCTGTATTAG
CTGGAGCTATTACTATACTATTAACAGATCGAAATTTAAATACATCATTTTTTGATCCTGCAGGAGGAGGAGATCCAATT
TTATATCAACATTTATTT---------
>LSM716-11|Bathytricha_leonina|K287615|
AACATTATATTTTATTTTTGGAATTTGAGCAGGAATAGTAGGAACCTCTTTAAGACTATTAATTCGAGCTGAATTAGGAA
CACCTGGATCTTTAATTGGAGATGATCAAATTTATAATACTATTGTAACAGCTCATGCTTTTATTATAATTTTTTTTATA
GTTATACCAATTATAATTGGAGGGTTTGGAAATTGACTTGTACCTTTAATATTAGGAGCACCTGATATAGCATTCCCACG
AATAAATAATATAAGTTTTTGATTACTTCCACCCTCTTTAACTCTCCTTATTTCAAGTAGAATTGTAGAAAATGGGGCAG
GAACTGGATGAACAGTTTACCCACCACTCTCATCTAATATTGCTCATGGAGGAAGATCTGTAGACTTAGCTATTTTTTCT
CTCCATTTGGCGGGAATCTCTTCTATTCTAGGAGCTATTAATTTTATTACAACTATTATTAATATACGATTAAATAGCTT
ATCTTTTGATCAAATACCTTTATTTATTTGAGCCGTAGGTATTACAGCATTTTTATTATTATTATCATTACCTGTATTAG
CTGGAGCTATTACTATACTATTAACAGATCGAAATTTAAATACATCATTTTTTGATCCTGCAGGAGGAGGAGATCCAATT
TTATATCAACATTTATTT---------
>LSM717-11|Bathytricha|K287616|
AACATTATATTTTATTTTTGGAATTTGAGCAGGAATAGTAGGAACCTCTCTAAGACTATTAATTCGAGCTGAATTAGGGA
CTCCTGGATCTTTAATTGGAGATGATCAAATTTATAATACTATTGTAACGGCTCATGCCTTTATTATAATTTTTTTTATA
GTTATACCAATTATAATCGGAGGATTTGGAAATTGACTTGTGCCTTTAATGTTAGGAGCACCTGATATAGCATTTCCACG
AATAAATAATATAAGTTTTTGATTACTCCCACCCTCTTTAACTCTCCTTATTTCAAGTAGAATTGTAGAAAATGGGGCGG
GAACTGGATGAACAGTTTACCCTCCACTCTCATCTAATATTGCTCATAGAGGAAGATCTGTAGACTTAGCTATTTTTTCC
CTCCACTTAGCTGGAATCTCTTCTATTTTAGGAGCTATTAATTTTATTACAACTATTATTAATATACGATTAAATAATTT
ATCTTTTGATCAAATACCTTTATTTATTTGAGCTGTAGGAATTACAGCATTTTTATTATTATTATCATTACCTGTATTAG
CTGGAGCTATTACTATATTATTAACAGACCGAAATTTAAATACATCATTTTTCGATCCTGCAGGAGGAGGAGACCCAATT
TTATATCAACATTTATTT---------
>LSTEM001-18|Chilo_crypsimetalla|ADR036a|
AACTTTATATTTTATTTTTGGAATCTGAGCAGGAATAATTGGAACATCTCTTAGTCTTTTAATTCGAGCTGAATTAGGTA
TCCCTGGATCTTTAATTGGAGATGATCAAATTTATAATACTATTGTCACTGCTCATGCCTTTATTATAATTTTTTTTATA
GTAATACCAATTATAATTGGAGGATTCGGAAATTGATTGGTTCCTTTAATATTAGGAGCTCCAGATATAGCTTTCCCACG
AATAAACAATATAAGATTTTGATTATTACCCCCCTCTTTAACTTTATTAATTTCTAGAAGAATTGTTGAAAATGGTGCTG
GAACAGGATGAACAGTATACCCCCCACTTTCATCTAATATTGCTCATGCTGGGAGATCAGTAGATTTAGCAATTTTTTCC
TTACACTTAGCAGGAATCTCTTCTATTTTAGGAGCTATTAATTTTATTACAACAATTATTAATATACGAATTAATGGATT
ATCATTCGATCAAATACCATTATTTGTGTGATCTGTCGGTATTACAGCATTATTATTATTACTTTCCTTACCTGTTTTAG
CTGGAGCTATTACTATATTGTTAACTGATCGAAATTTAAATACATCTTTTTTTGATCCTGCTGGAGGAGGTGATCCAATC
CTTTATCAACATTTATTTTGATTTTTT

>LSTEM002-18|Chilo_crypsimetalla|ADR036aa|
AACTTTATATTTTATTTTTGGAATCTGAGCAGGAATAATTGGAACATCTCTTAGTCTTTTAATTCGAGCTGAATTAGGTA
CCCCTGGATCTTTAATTGGAGATGATCAAATTTATAATACTATTGTCACTGCTCATGCCTTTATTATAATTTTTTTTATA
GTAATACCAATTATAATTGGAGGATTCGGAAATTGATTGGTTCCTTTAATATTAGGAGCTCCAGATATAGCTTTCCCACG
AATAAACAATATAAGATTTTGATTATTACCCCCCTCTTTAACTTTATTAATTTCTAGAAGAATTGTTGAAAATGGTGCTG
GAACAGGATGAACAGTATACCCCCCACTTTCATCTAATATTGCTCATGCTGGGAGATCAGTAGATTTAGCAATTTTTTCC
TTACACTTAGCAGGAATCTCTTCTATTTTAGGAGCTATTAATTTTATTACAACAATTATTAATATACGAATTAATGGATT
ATCATTCGATCAAATACCATTATTTGTGTGATCTGTCGGTATTACAGCATTATTATTATTACTTTCCTTACCTGTTTTAG
CTGGAGCTATTACTATATTGTTAACTGATCGAAATTTAAATACATCTTTTTTTGATCCTGCTGGAGGAGGTGATCCAATC
CTTTATCAACATTTATTTTGATTTTTG
>LSTEM003-18|Chilo_crypsimetalla|ADR0542|
----------TTTTTTTTTCGGATCTGAGCAGGAATAATTGGAACATCTCTTAGTCTTTTAATTCGAGCTGAATTAGGTA
CCCCTGGATCTTTAATTGGAGATGATCAAATTTATAATACTATTGTCACTGCTCATGCCTTTATTATAATTTTTTTTATA
GTAATACCAATTATAATTGGAGGATTCGGAAATTGATTGGTTCCTTTAATATTAGGAGCTCCAGATATAGCTTTCCCACG
AATAAACAATATAAGATTTTGATTATTACCCCCCTCTTTAACTTTATTAATTTCTAGAAGAATTGTTGAAAATGGTGCTG
GAACAGGATGAACAGTATACCCCCCACTTTCATCTAATATTGCTCATGCTGGGAGATCAGTAGATTTAGCAATTTTTTCC
TTACACTTAGCAGGAATCTCTTCTATTTTAGGAGCTATTAATTTTATTACAACAATTATTAATATACGAATTAATGGATT
ATCATTCGATCAAATACCATTATTTGTGTGATCTGTCGGTATTACAGCATTATTATTATTACTTTCCTTACCTGTTTTAG
CTGGAGCTATTACTATATTGTTAACTGATCGAAATTTAAATACATCTTTTTTTGATCCTGCTGGAGGAGGTGATCCAATC
CTTTATCAACATTTATTTTGATTTTTT
>LSTEM004-18|Chilo_suppressalis|am00759|
AACTTTATATTTTATTTTTGGTATTTGAGCAGGTATAATTGGAACATCTCTTAGACTTTTAATTCGTGCTGAATTAGGAA
CTCCAGGATCTTTAATTGGAGATGATCAAATTTATAATACCATTGTTACGGCTCATGCATTTATTATAATTTTTTTTATA
GTTATACCAATTATAATTGGTGGATTTGGAAATTGATTAGTACCTTTAATATTAGGGGCTCCTGATATAGCTTTCCCACG
AATAAATAATATAAGATTTTGAATATTACCCCCCTCATTAACTTTACTAATTTCTAGAAGAATTGTTGAAAATGGAGCTG
GAACAGGTTGAACAGTGTACCCCCCACTATCATCTAATATTGCTCACGCTGGAAGTTCAGTAGATTTAGCAATTTTCTCT
TTACATTTAGCTGGAATTTCTTCAATTCTAGGTGCTATTAATTTTATTACTACGATTATTAATATACGAATTAATGGTCT
TTCATTTGATCAAATACCTTTATTTGTTTGATCCGTAGGTATTACAGCTTTATTATTACTTCTATCTCTACCAGTATTAG
CTGGAGCAATTACAATATTATTAACCGATCGAAATTTAAATACATCTTTTTTTGATCCTGCTGGTGGTGGAGATCCAATT
CTTTACCAACATTTATTTTGATTTTTT
>LSTEM005-18|Chilo_suppressalis|am00760|
AACTTTATATTTTATTTTTGGTATTTGAGCAGGTATAATTGGAACATCTCTTAGACTTTTAATTCGTGCTGAATTAGGAA
CTCCAGGATCTTTAATTGGAGATGATCAAATTTATAATACCATTGTTACGGCTCATGCATTTATTATAATTTTTTTTATA
GTTATACCAATTATAATTGGTGGATTTGGAAATTGATTAGTACCTTTAATATTAGGGGCTCCTGATATAGCTTTCCCACG
AATAAATAATATAAGATTTTGAATATTACCCCCCTCATTAACTTTACTAATTTCTAGAAGAATTGTTGAAAATGGAGCTG
GAACAGGTTGAACAGTGTACCCCCCACTATCATCTAATATTGCTCACGCTGGAAGTTCAGTAGATTTAGCAATTTTCTCT
TTACATTTAGCTGGAATTTCTTCAATTCTAGGTGCTATTAATTTTATTACTACGATTATTAATATACGAATTAATGGTCT
TTCATTTGATCAAATACCTTTATTTGTTTGATCCGTAGGTATTACAGCTTTATTATTACTTCTATCTCTACCAGTATTAG
CTGGAGCAATTACAATATTATTAACCGATCGAAATTTAAATACATCTTTTTTTGATCCTGCTGGTGGTGGAGATCCAATT
CTTTACCAACATTTATTTTGATTTTTT
>LSTEM006-18|Chilo_suppressalis|am00761|
AACTTTATATTTTATTTTTGGTATTTGAGCAGGTATAATTGGAACATCTCTTAGACTTTTAATTCGTGCTGAATTAGGAA
CTCCAGGATCTTTAATTGGAGATGATCAAATTTATAATACCATTGTTACGGCTCATGCATTTATTATAATTTTTTTTATA
GTTATACCAATTATAATTGGTGGATTTGGAAATTGATTAGTACCTTTAATATTAGGGGCTCCTGATATAGCTTTCCCACG
AATAAATAATATAAGATTTTGAATATTACCCCCCTCATTAACTTTACTAATTTCTAGAAGAATTGTTGAAAATGGAGCTG
GAACAGGTTGAACAGTGTACCCCCCACTATCATCTAATATTGCTCACGCTGGAAGTTCAGTAGATTTAGCAATTTTCTCT
TTACATTTAGCTGGAATTTCTTCAATTCTAGGTGCTATTAATTTTATTACTACGATTATTAATATACGAATTAATGGTCT
TTCATTTGATCAAATACCTTTATTTGTTTGATCCGTAGGTATTACAGCTTTATTATTACTTCTATCTCTACCAGTATTAG
CTGGAGCAATTACAATATTATTAACCGATCGAAATTTAAATACATCTTTTTTTGATCCTGCTGGTGGTGGAGATCCAATT
CTTTACCAACATTTATTTTGATTTTTT
>LSTEM007-18|Chilo_suppressalis|am00762|
AACTTTATATTTTATTTTTGGTATTTGAGCAGGTATAATTGGAACATCTCTTAGACTTTTAATTCGTGCTGAATTAGGAA
CTCCAGGATCTTTAATTGGAGATGATCAAATTTATAATACCATTGTTACGGCTCATGCATTTATTATAATTTTTTTTATA
GTTATACCAATTATAATTGGTGGATTTGGAAATTGATTAGTACCTTTAATATTAGGGGCTCCTGATATAGCTTTCCCACG
AATAAATAATATAAGATTTTGAATATTACCCCCCTCATTAACTTTACTAATTTCTAGAAGAATTGTTGAAAATGGAGCTG
GAACAGGTTGAACAGTGTACCCCCCACTATCATCTAATATTGCTCACGCTGGAAGTTCAGTAGATTTAGCAATTTTCTCT
TTACATTTAGCTGGAATTTCTTCAATTCTAGGTGCTATTAATTTTATTACTACGATTATTAATATACGAATTAATGGTCT

TTCATTTGATCAAATACCTTTATTTGTTTGATCCGTAGGTATTACAGCTTTATTATTACTTCTATCTCTACCAGTATTAG
CTGGAGCAATTACAATATTATTAACCGATCGAAATTTAAATACATCTTTTTTTGATCCTGCTGGTGGTGGAGATCCAATT
CTTTACCAACATTTATTTTGATTTTTT
>LSTEM008-18|Chilo_suppressalis|am00763|
AACTTTATATTTTATTTTTGGTATTTGAGCAGGTATAATTGGAACATCTCTTAGACTTTTAATTCGTGCTGAATTAGGAA
CTCCAGGATCTTTAATTGGAGATGATCAAATTTATAATACCATTGTTACGGCTCATGCATTTATTATAATTTTTTTTATA
GTTATACCAATTATAATTGGTGGATTTGGAAATTGATTAGTACCTTTAATATTAGGGGCTCCTGATATAGCTTTCCCACG
AATAAATAATATAAGATTTTGAATATTACCCCCCTCATTAACTTTACTAATTTCTAGAAGAATTGTTGAAAATGGAGCTG
GAACAGGTTGAACAGTGTACCCCCCACTATCATCTAATATTGCTCACGCTGGAAGTTCAGTAGATTTAGCAATTTTCTCT
TTACATTTAGCTGGAATTTCTTCAATTCTAGGTGCTATTAATTTTATTACTACGATTATTAATATACGAATTAATGGTCT
TTCATTTGATCAAATACCTTTATTTGTTTGATCCGTAGGTATTACAGCTTTATTATTACTTCTATCTCTACCAGTATTAG
CTGGAGCAATTACAATATTATTAACCGATCGAAATTTAAATACATCTTTTTTTGATCCTGCTGGTGGTGGAGATCCAATT
CTTTACCAACATTTATTTTGATTTTTT
>LSTEM009-18|Chilo_suppressalis|am00764|
AACTTTATATTTTATTTTTGGTATTTGAGCAGGTATAATTGGAACATCTCTTAGACTTTTAATTCGTGCTGAATTAGGAA
CTCCAGGATCTTTAATTGGAGATGATCAAATTTATAATACCATTGTTACGGCTCATGCATTTATTATAATTTTTTTTATA
GTTATACCAATTATAATTGGTGGATTTGGAAATTGATTAGTACCTTTAATATTAGGGGCTCCTGATATAGCTTTCCCACG
AATAAATAATATAAGATTTTGAATATTACCCCCCTCATTAACTTTACTAATTTCTAGAAGAATTGTTGAAAATGGAGCTG
GAACAGGTTGAACAGTGTACCCCCCACTATCATCTAATATTGCTCACGCTGGAAGTTCAGTAGATTTAGCAATTTTCTCT
TTACATTTAGCTGGAATTTCTTCAATTCTAGGTGCTATTAATTTTATTACTACGATTATTAATATACGAATTAATGGTCT
TTCATTTGATCAAATACCTTTATTTGTTTGATCCGTAGGTATTACAGCTTTATTATTACTTCTATCTCTACCAGTATTAG
CTGGAGCAATTACAATATTATTAACCGATCGAAATTTAAATACATCTTTTTTTGATCCTGCTGGTGGTGGAGATCCAATT
CTTTACCAACATTTATTTTGATTTTTT
>LSTEM010-18|Chilo_suppressalis|am00765|
AACTTTATATTTTATTTTTGGTATTTGAGCAGGTATAATTGGAACATCTCTTAGACTTTTAATTCGTGCTGAATTAGGAA
CTCCAGGATCTTTAATTGGAGATGATCAAATTTATAATACCATTGTTACGGCTCATGCATTTATTATAATTTTTTTTATA
GTTATACCAATTATAATTGGTGGATTTGGAAATTGATTAGTACCTTTAATATTAGGGGCTCCTGATATAGCTTTCCCACG
AATAAATAATATAAGATTTTGAATATTACCCCCCTCATTAACTTTACTAATTTCTAGAAGAATTGTTGAAAATGGAGCTG
GAACAGGTTGAACAGTGTACCCCCCACTATCATCTAATATTGCTCACGCTGGAAGTTCAGTAGATTTAGCAATTTTCTCT
TTACATTTAGCTGGAATTTCTTCAATTCTAGGTGCTATTAATTTTATTACTACGATTATTAATATACGAATTAATGGTCT
TTCATTTGATCAAATACCTTTATTTGTTTGATCCGTAGGTATTACAGCTTTATTATTACTTCTATCTCTACCAGTATTAG
CTGGAGCAATTACAATATTATTAACCGATCGAAATTTAAATACATCTTTTTTTGATCCTGCTGGTGGTGGAGATCCAATT
CTTTACCAACATTTATTTTGATTTTTT
>LSTEM011-18|Chilo_suppressalis|am00766|
AACTTTATATTTTATTTTTGGTATTTGAGCAGGTATAATTGGAACATCTCTTAGACTTTTAATTCGTGCTGAATTAGGAA
CTCCAGGATCTTTAATTGGAGATGATCAAATTTATAATACCATTGTTACGGCTCATGCATTTATTATAATTTTTTTTATA
GTTATACCAATTATAATTGGTGGATTTGGAAATTGATTAGTACCTTTAATATTAGGAGCTCCTGATATAGCTTTCCCACG
AATAAATAATATAAGATTTTGAATATTACCCCCCTCATTAACTTTACTAATTTCTAGAAGAATTGTTGAAAATGGAGCTG
GAACAGGTTGAACAGTGTACCCCCCACTATCATCTAATATTGCTCACGCTGGAAGTTCAGTAGATTTAGCAATTTTCTCT
TTACATTTAGCTGGAATTTCTTCAATTCTAGGTGCTATTAATTTTATTACTACGATTATTAATATACGAATTAATGGTCT
TTCATTTGATCAAATACCTTTATTTGTTTGATCCGTAGGTATTACAGCTTTATTATTACTTCTATCTCTACCAGTATTAG
CTGGAGCAATTACAATATTATTAACCGATCGAAATTTAAATACATCTTTTTTTGATCCTGCTGGTGGTGGAGATCCAATT
CTTTACCAACATTTATTTTGATTTTTT
>LSTEM012-18|Chilo_suppressalis|am00767|
AACTTTATATTTTATTTTTGGTATTTGAGCAGGTATAATTGGAACATCTCTTAGACTTTTAATTCGTGCTGAATTAGGAA
CTCCAGGATCTTTAATTGGAGATGATCAAATTTATAATACCATTGTTACGGCTCATGCATTTATTATAATTTTTTTTATA
GTTATACCAATTATAATTGGTGGATTTGGAAATTGATTAGTACCTTTAATATTAGGGGCTCCTGATATAGCTTTCCCACG
AATAAATAATATAAGATTTTGAATATTACCCCCCTCATTAACTTTACTAATTTCTAGAAGAATTGTTGAAAATGGAGCTG
GAACAGGTTGAACAGTGTACCCCCCACTATCATCTAATATTGCTCACGCTGGAAGTTCAGTAGATTTAGCAATTTTCTCT
TTACATTTAGCTGGAATTTCTTCAATTCTAGGTGCTATTAATTTTATTACTACGATTATTAATATACGAATTAATGGTCT
TTCATTTGATCAAATACCTTTATTTGTTTGATCCGTAGGTATTACAGCTTTATTATTACTTCTATCTCTACCAGTATTAG
CTGGAGCAATTACAATATTATTAACCGATCGAAATTTAAATACATCTTTTTTTGATCCTGCTGGTGGTGGAGATCCAATT
CTTTACCAACATTTATTTTGATTTTTT
>LSTEM013-18|Chilo_suppressalis|am00768|
AACTTTATATTTTATTTTTGGTATTTGAGCAGGTATAATTGGAACATCTCTTAGACTTTTAATTCGTGCTGAATTAGGAA
CTCCAGGATCTTTAATTGGAGATGATCAAATTTATAATACCATTGTTACGGCTCATGCATTTATTATAATTTTTTTTATA
GTTATACCAATTATAATTGGTGGATTTGGAAATTGATTAGTACCTTTAATATTAGGGGCTCCTGATATAGCTTTCCCACG

AATAAATAATATAAGATTTTGAATATTACCCCCCTCATTAACTTTACTAATTTCTAGAAGAATTGTTGAAAATGGAGCTG
GAACAGGTTGAACAGTGTACCCCCCACTATCATCTAATATTGCTCACGCTGGAAGTTCAGTAGATTTAGCAATTTTCTCT
TTACATTTAGCTGGAATTTCTTCAATTCTAGGTGCTATTAATTTTATTACTACGATTATTAATATACGAATTAATGGTCT
TTCATTTGATCAAATACCTTTATTTGTTTGATCCGTAGGTATTACAGCTTTATTATTACTTCTATCTCTACCAGTATTAG
CTGGAGCAATTACAATATTATTAACCGATCGAAATTTAAATACATCTTTTTTGATCCTGCTGGTGGTGGAGATCCAATT
CTTTACCAACATTTATTTTGATTTTTT
>LSTEM014-18|Chilo_suppressalis|am00769|
AACTTTATATTTTATTTTTGGTATTTGAGCAGGTATAATTGGAACATCTCTTAGACTTTTAATTCGTGCTGAATTAGGAA
CTCCAGGATCTTTAATTGGAGATGATCAAATTTATAATACCATTGTTACGGCTCATGCATTTATTATAATTTTTTTTATA
GTTATACCAATTATAATTGGTGGATTTGGAAATTGATTAGTACCTTTAATATTAGGGGCTCCTGATATAGCTTTCCCACG
AATAAATAATATAAGATTTTGAATATTACCCCCCTCATTAACTTTACTAATTTCTAGAAGAATTGTTGAAAATGGAGCTG
GAACAGGTTGAACAGTGTACCCCCCACTATCATCTAATATTGCTCACGCTGGAAGTTCAGTAGATTTAGCAATTTTCTCT
TTACATTTAGCTGGAATTTCTTCAATTCTAGGTGCTATTAATTTTATTACTACGATTATTAATATACGAATTAATGGTCT
TTCATTTGATCAAATACCTTTATTTGTTTGATCCGTAGGTATTACAGCTTTATTATTACTTCTATCTCTACCAGTATTAG
CTGGAGCAATTACAATATTATTAACCGATCGAAATTTAAATACATCTTTTTTGATCCTGCTGGTGGTG-----------
----
>LSTEM015-18|Chilo_suppressalis|am00770|
AACTTTATATTTTATTTTTGGTATTTGAGCAGGTATAATTGGAACATCTCTTAGACTTTTAATTCGTGCTGAATTAGGAA
CTCCAGGATCTTTAATTGGAGATGATCAAATTTATAATACCATTGTTACGGCTCATGCATTTATTATAATTTTTTTTATA
GTTATACCAATTATAATTGGTGGATTTGGAAATTGATTAGTACCTTTAATATTAGGGGCTCCTGATATAGCTTTCCCACG
AATAAATAATATAAGATTTTGAATATTACCCCCCTCATTAACTTTACTAATTTCTAGAAGAATTGTTGAAAATGGAGCTG
GAACAGGTTGAACAGTGTACCCCCCACTATCATCTAATATTGCTCACGCTGGAAGTTCAGTAGATTTAGCAATTTTCTCT
TTACATTTAGCTGGAATTTCTTCAATTCTAGGTGCTATTAATTTTATTACTACGATTATTAATATACGAATTAATGGTCT
TTCATTTGATCAAATACCTTTATTTGTTTGATCCGTAGGTATTACAGCTTTATTATTACTTCTATCTCTACCAGTATTAG
CTGGAGCAATTACAATATTATTAACCGATCGAAATTTAAATACATCTTTTTTGATCCTGCTGGTGGTGGAGATCCAATT
CTTTACCAACATTTATTTTGATTTTTT
>LSTEM016-18|Chilo_suppressalis|am00771|
AACTTTATATTTTATTTTTGGTATTTGAGCAGGTATAATTGGAACATCTCTTAGACTTTTAATTCGTGCTGAATTAGGAA
CTCCAGGATCTTTAATTGGAGATGATCAAATTTATAATACCATTGTTACGGCTCATGCATTTATTATAATTTTTTTTATA
GTTATACCAATTATAATTGGTGGATTTGGAAATTGATTAGTACCTTTAATATTAGGGGCTCCTGATATAGCTTTCCCACG
AATAAATAATATAAGATTTTGAATATTACCCCCCTCATTAACTTTACTAATTTCTAGAAGAATTGTTGAAAATGGAGCTG
GAACAGGTTGAACAGTGTACCCCCCACTATCATCTAATATTGCTCACGCTGGAAGTTCAGTAGATTTAGCAATTTTCTCT
TTACATTTAGCTGGAATTTCTTCAATTCTAGGTGCTATTAATTTTATTACTACGATTATTAATATACGAATTAATGGTCT
TTCATTTGATCAAATACCTTTATTTGTTTGATCCGTAGGTATTACAGCTTTATTATTACTTCTATCTCTACCAGTATTAG
CTGGAGCAATTACAATATTATTAACCGATCGAAATTTAAATACATCTTTTTTGATCCTGCTGGTGGTGGAGATCCAATT
CTTTACCAACATTTATTTTGATTTTTT
>LSTEM017-18|Chilo_suppressalis|am00773|
AACTTTATATTTTATTTTTGGTATTTGAGCAGGTATAATTGGAACATCTCTTAGACTTTTAATTCGTGCTGAATTAGGAA
CTCCAGGATCTTTAATTGGAGATGATCAAATTTATAATACCATTGTTACGGCTCATGCATTTATTATAATTTTTTTTATA
GTTATACCAATTATAATTGGTGGATTTGGAAATTGATTAGTACCTTTAATATTAGGGGCTCCTGATATAGCTTTCCCACG
AATAAATAATATAAGATTTTGAATATTACCCCCCTCATTAACTTTACTAATTTCTAGAAGAATTGTTGAAAATGGAGCTG
GAACAGGTTGAACAGTGTACCCCCCACTATCATCTAATATTGCTCACGCTGGAAGTTCAGTAGATTTAGCAATTTTCTCT
TTACATTTAGCTGGAATTTCTTCAATTCTAGGTGCTATTAATTTTATTACTACAATTATTAATATACGAATTAATGGTCT
TTCATTTGATCAAATACCTTTATTTGTTTGATCCGTAGGTATTACAGCTTTATTATTACTTCTATCTCTACCAGTATTAG
CTGGAGCAATTACAATATTATTAACCGATCGAAATTTAAATACATCTTTTTTGATCCTGCTGGTGGTGGAGATCCAATT
CTTTACCAACATTTATTTTGATTTTTT
>LSTEM018-18|Chilo_suppressalis|am00774|
AACTTTATATTTTATTTTTGGTATTTGAGCAGGTATAATTGGAACATCTCTTAGACTTTTAATTCGTGCTGAATTAGGAA
CTCCAGGATCTTTAATTGGAGATGATCAAATTTATAATACCATTGTTACGGCTCATGCATTTATTATAATTTTTTTTATA
GTTATACCAATTATAATTGGTGGATTTGGAAATTGATTAGTACCTTTAATATTAGGGGCTCCTGATATAGCTTTCCCACG
AATAAATAATATAAGATTTTGAATATTACCCCCCTCATTAACTTTACTAATTTCTAGAAGAATTGTTGAAAATGGAGCTG
GAACAGGTTGAACAGTGTACCCCCCACTATCATCTAATATTGCTCACGCTGGAAGTTCAGTAGATTTAGCAATTTTCTCT
TTACATTTAGCTGGAATTTCTTCAATTCTAGGTGCTATTAATTTTATTACTACGATTATTAATATACGAATTAATGGTCT
TTCATTTGATCAAATACCTTTATTTGTTTGATCCGTAGGTATTACAGCTTTATTATTACTTCTATCTCTACCAGTATTAG
CTGGAGCAATTACAATATTATTAACCGATCGAAATTTAAATACATCTTTTTTGATCCTGCTGGTGGTGGAGATCCAATT
CTTTACCAACATTTATTTTGATTTTTT
>LSTEM019-18|Chilo_suppressalis|am00775|

AACTTTATATTTTATTTTTGGTATTTGAGCAGGTATAATTGGAACATCTCTTAGACTTTTAATTCGTGCTGAATTAGGAA
CTCCAGGATCTTTAATTGGAGATGATCAAATTTATAATACCATTGTTACGGCTCATGCATTTATTATAATTTTTTTTATA
GTTATACCAATTATAATTGGTGGATTTGGAAATTGATTAGTACCTTTAATATTAGGGGCTCCTGATATAGCTTTCCCACG
AATAAATAATATAAGATTTTGAATATTACCCCCCTCATTAACTTTACTAATTTCTAGAAGAATTGTTGAAAATGGAGCTG
GAACAGGTTGAACAGTGTACCCCCCACTATCATCTAATATTGCTCACGCTGGAAGTTCAGTAGATTTAGCAATTTTCTCT
TTACATTTAGCTGGAATTTCTTCAATTCTAGGTGCTATTAATTTTATTACTACGATTATTAATATACGAATTAATGGTCT
TTCATTTGATCAAATACCTTTATTTGTTTGATCCGTAGGTATTACAGCTTTATTATTACTTCTATCTCTACCAGTATTAG
CTGGAGCAATTACAATATTATTAACCGATCGAAATTTAAATACATCTTTTTTTGATCCTGCTGGTGGTGGAGATCCAATT
CTTTACCAACATTTATTTTGATTTTTT
>LSTEM020-18|Chilo_suppressalis|am00776|
AACTTTATATTTTATTTTTGGTATTTGAGCAGGTATAATTGGAACATCTCTTAGACTTTTAATTCGTGCTGAATTAGGAA
CTCCAGGATCTTTAATTGGAGATGATCAAATTTATAATACCATTGTTACGGCTCATGCATTTATTATAATTTTTTTTATA
GTTATACCAATTATAATTGGTGGATTTGGAAATTGATTAGTACCTTTAATATTAGGGGCTCCTGATATAGCTTTCCCACG
AATAAATAATATAAGATTTTGAATATTACCCCCCTCATTAACTTTACTAATTTCTAGAAGAATTGTTGAAAATGGAGCTG
GAACAGGTTGAACAGTGTACCCCCCACTATCATCTAATATTGCTCACGCTGGAAGTTCAGTAGATTTAGCAATTTTCTCT
TTACATTTAGCTGGAATTTCTTCAATTCTAGGTGCTATTAATTTTATTACTACGATTATTAATATACGAATTAATGGTCT
TTCATTTGATCAAATACCTTTATTTGTTTGATCCGTAGGTATTACAGCTTTATTATTACTTCTATCTCTACCAGTATTAG
CTGGAGCAATTACAATATTATTAACCGATCGAAATTTAAATACATCTTTTTTTGATCCTGCTGGTGGTGGAGATCCAATT
CTTTACCAACATTTATTTTGATTTTTT
>LSTEM021-18|Chilo_suppressalis|am00777|
AACTTTATATTTTATTTTTGGTATTTGAGCAGGTATAATTGGAACATCTCTTAGACTTTTAATTCGTGCTGAATTAGGAA
CTCCAGGATCTTTAATTGGAGATGATCAAATTTATAATACCATTGTTACGGCTCATGCATTTATTATAATTTTTTTTATA
GTTATACCAATTATAATTGGTGGATTTGGAAATTGATTAGTACCTTTAATATTAGGAGCTCCTGATATAGCTTTCCCACG
AATAAATAATATAAGATTTTGAATATTACCCCCCTCATTAACTTTACTAATTTCTAGAAGAATTGTTGAAAATGGAGCTG
GAACAGGTTGAACAGTGTACCCCCCACTATCATCTAATATTGCTCACGCTGGAAGTTCAGTAGATTTAGCAATTTTCTCT
TTACATTTAGCTGGAATTTCTTCAATTCTAGGTGCTATTAATTTTATTACTACGATTATTAATATACGAATTAATGGTCT
TTCATTTGATCAAATACCTTTATTTGTTTGATCCGTAGGTATTACAGCTTTATTATTACTTCTATCTCTACCAGTATTAG
CTGGAGCAATTACAATATTATTAACCGATCGAAATTTAAATACATCTTTTTTTGATCCTGCTGGTGGTGGAGATCCAATT
CTTTACCAACATTTATTTTGATTTTTT
>LSTEM022-18|Chilo_suppressalis|am00778|
AACTTTATATTTTATTTTTGGTATTTGAGCAGGTATAATTGGAACATCTCTTAGACTTTTAATTCGCGCTGAATTAGGAA
CTCCAGGATCTTTAATTGGAGATGATCAAATTTATAATACCATTGTTACGGCTCATGCATTTATTATAATTTTTTTTATA
GTTATACCAATTATAATTGGTGGATTTGGAAATTGATTAGTACCTTTAATATTAGGGGCTCCTGATATAGCTTTCCCACG
AATAAATAATATAAGATTTTGAATATTACCCCCCTCATTAACTTTACTAATTTCTAGAAGAATTGTTGAAAATGGAGCTG
GAACAGGTTGAACAGTGTACCCCCCACTATCATCTAATATTGCTCACGCTGGAAGTTCAGTAGATTTAGCAATTTTCTCT
TTACATTTAGCTGGAATTTCTTCAATTCTAGGTGCTATTAATTTTATTACTACGATTATTAATATACGAATTAATGGTCT
TTCATTTGATCAAATACCTTTATTTGTTTGATCCGTAGGTATTACAGCTTTATTATTACTTCTATCTCTACCAGTATTAG
CTGGAGCAATTACAATATTATTAACCGATCGAAATTTAAATACATCTTTTTTTGATCCTGCTGGTGGTGGAGATCCAATT
CTTTACCAACATTTATTTTGATTTTTT
>LSTEM023-18|Chilo_tumidicostalis|am00779|
AACTTTATATTTTATTTTTGGAATTTGAGCTGGAATAATTGGTACATCTTTAAGACTCTTAATTCGAGCTGAATTAGGAA
CCCCAGGATCTTTAATTGGAGATGATCAAATTTATAATACTATTGTCACAGCTCATGCATTTATTATAATTTTTTTCATA
GTAATACCAATTATAATTGGTGGATTTGGAAATTGATTGGTACCTTTAATATTAGGAGCTCCAGATATAGCTTTCCCACG
AATAAATAATATAAGATTTTGATTATTACCCCCCTCATTAACACTATTAATTTCAAGTAGAATTGTAGAAAATGGAGCAG
GAACAGGATGAACAGTGTACCCCCCACTATCATCTAATATTGCTCATGCTGGAAGCTCTGTTGATTTAGCAATTTTCTCA
TTACATCTAGCTGGTATTTCCTCTATTTTAGGAGCTATTAATTTTATTACAACAATTATTAATATACGAATTAATGGGTT
ATCATTTGATCAGATACCTTTATTTGTATGATCTGTAGGAATTACAGCTTTATTACTATTACTTTCATTACCAGTTTTAG
CAGGAGCTATTACTATATTATTAACAGATCGAAATTTAAATACTTCCTTTTTTGACCCTGCTGGAGGGGGAGATCCTATT
CTCTACCAACATTTATTTTGATTTTTT
>LSTEM024-18|Chilo_sacchariphagus|am00780|
AACTTTATATTTTATTTTTGGAATTTGAGCTGGAATAGTTGGAACATCCCTTAGACTTTTAATTCGAGCTGAATTAGGAA
ATCCAGGTTCATTAATCGGAGATGATCAAATTTATAATACTATTGTTACAGCCCATGCATTTATTATAATTTTTTTTATA
GTAATACCAATTATAATTGGAGGATTTGGAAATTGATTAGTTCCATTAATATTAGGGGCTCCTGATATAGCCTTCCCTCG
TCTAAATAATATAAGATTTTGATTATTACCCCCTTCTTTAACCCTTCTAATTTCTAGAAGAATCGTTGAAAATGGAGCAG
GAACTGGATGAACAGTCTACCCCCCCCTATCTTCCAATATTTCACATGCTGGAAGTTCAGTAGATTTAGCCATCTTCTCC
CTCCATTTAGCTGRAATTTCTTCAATTTTAGGAGCTATCAATTTCATTACTACAATTATTAATATACGAATTAATGGATT
ATTATTTGATCAAATACCATTATTTGTTTGATCTGTTGGTATTACAGCATTACTTCTCCTCCTTTCTTTACCAGTATTAG

CAGGTGCTATTACTATACTATTAACTGAYCGAAATTTAAATACATCTTTTTTTGACCCAGCTGGAGGAGGTGATCCAATT
TTATATCAACATTTATTTTGATTTTTT
>LSTEM025-18|Chilo_sacchariphagus|am00781|
AACTTTATATTTTATTTTTGGAATTTGAGCTGGAATAGTTGGAACATCCCTTAGACTTTTAATTCGAGCTGAATTAGGAA
ATCCAGGTTCATTAATCGGAGATGATCAAATTTATAATACTATTGTTACAGCCCATGCATTTATTATAATTTTTTTTATA
GTAATACCAATTATAATTGGAGGATTTGGAAATTGATTAGTTCCATTAATATTAGGGGCTCCTGATATAGCCTTCCCTCG
TCTAAATAATATAAGATTTTGATTATTACCCCCTTCTTTAACCCTTCTAATTTCTAGAAGAATCGTTGAAAATGGAGCAG
GAACTGGATGAACAGTCTACCCCCCCCTATCTTCCAATATTTCACATGCTGGAAGTTCAGTAGATTTAGCCATCTTCTCC
CTTCATTTAGCTGGAATTTCTTCAATTTTAGGAGCTATCAATTTCATTACTACAATTATTAATATACGAATTAATGGATT
ATTATTTGATCAAATACCATTATTTGTTTGATCTGTTGGTATTACAGCATTACTTCTCCTCCTTTCTTTACCAGTATTAG
CAGGTGCTATTACTATACTATTAACTGACCGAAATTTAAATACATCTTTTTTTGACCCAGCTGGAGGAGGTGATCCAATT
TTATATCAACATTTATTTTGATTTTTT
>LSTEM026-18|Chilo_sacchariphagus|am00782|
AACTTTATATTTTATTTTTGGAATTTGAGCTGGAATAGTTGGAACATCCCTTAGACTTTTAATTCGAGCTGAATTAGGAA
ATCCAGGTTCATTAATCGGAGATGATCAAATTTATAATACTATTGTTACAGCCCATGCATTTATTATAATTTTTTTTATA
GTAATACCAATTATAATTGGAGGATTTGGAAATTGATTAGTTCCATTAATATTAGGGGCTCCTGATATAGCCTTCCCTCG
TCTAAATAATATAAGATTTTGATTATTACCCCCTTCTTTAACCCTTCTAATTTCTAGAAGAATCGTTGAAAATGGAGCAG
GAACTGGATGAACAGTCTACCCCCCCCTATCTTCCAATATTTCACATGCTGGAAGTTCAGTAGATTTAGCCATCTTCTCC
CTTCATTTAGCTGGAATTTCTTCAATTTTAGGAGCTATCAATTTCATTACTACAATTATTAATATACGAATTAATGGATT
ATTATTTGATCAAATACCATTATTTGTTTGATCTGTTGGTATTACAGCATTACTTCTCCTCCTTTCTTTACCAGTATTAG
CAGGTGCTATTACTATACTATTAACTGACCGAAATTTAAATACATCTTTTTTTGACCCAGCTGGAGGAGGTGATCCAATT
TTATATCAACATTTATTTTGATTTTTT
>LSTEM027-18|Chilo_infuscatellus|am00783|
AACTTTATATTTTATTTTTGGAATTTGAGCAGGAATAATTGGAACCTCTCTTAGACTTTTAATTCGAGCTGAATTAGGAA
CTCCAGGATCTTTAATTGGAGATGATCAAATTTATAATACTATTGTTACAGCTCATGCATTTATTATAATTTTTTTTATA
GTAATACCAATTATAATCGGAGGATTTGGAAATTGATTAGTTCCTTTAATACTAGGAGCACCTGATATAGCTTTCCCACG
GATAAATAATATAAGTTTTTGATTATTACCACCATCACTAACATTATTGATTTCTAGAAGAATTGTTGAAAATGGAGCAG
GAACTGGTTGAACTGTTTATCCTCCTTTATCTTCAAATATTGCTCATGGGGGAAGCTCTGTAGATTTAGCAATTTTTTCC
CTTCATTTAGCTGGTATTTCATCAATTTTAGGAGCTATTAATTTTATTACAACAATTATTAATATACGAGTTAATGGTCT
ATCATTTGATCAAATACCTTTATTTGTCTGATCTGTAGGAATTACAGCATTATTATTATTACTTTCTCTACCAGTATTAG
CAGGTGCTATTACTATACTACTAACTGATCGAAATTTAAATACATCTTTTTTTGATCCTGCAGGAGGGGGAGATCCAATC
CTCTACCAACATTTATTTTGATTTTTT
>LSTEM028-18|Chilo_infuscatellus|am00784|
AACTTTATATTTTATTTTTGGAATTTGAGCAGGAATAATTGGAACCTCTCTTAGACTTTTAATTCGAGCTGAATTAGGAA
CTCCAGGATCTTTAATTGGAGATGATCAAATTTATAATACTATTGTTACAGCTCATGCATTTATTATAATTTTTTTTATA
GTAATACCAATTATAATCGGAGGATTTGGAAATTGATTAGTTCCTTTAATACTAGGAGCACCTGATATAGCTTTCCCACG
GATAAATAATATAAGTTTTTGATTATTACCACCATCACTAACATTATTGATTTCTAGAAGAATTGTTGAAAATGGAGCAG
GAACTGGTTGAACTGTTTATCCTCCTTTATCTTCAAATATTGCTCATGGGGGAAGCTCTGTAGATTTAGCAATTTTTTCC
CTTCATTTAGCTGGTATTTCATCAATTTTAGGAGCTATTAATTTTATTACAACAATTATTAATATACGAGTTAATGGTCT
ATCATTTGATCAAATACCTTTATTTGTCTGATCTGTAGGAATTACAGCATTATTATTATTACTTTCTCTACCAGTATTAG
CAGGTGCTATTACTATACTACTAACTGATCGAAATTTAAATACATCTTTTTTTGATCCTGCAGGAGGGGGAGATCCAATC
CTCTACCAACATTTATTTTGATTTTTT
>LSTEM029-18|Chilo_infuscatellus|am00785|
AACTTTATATTTTATTTTTGGAATTTGAGCAGGAATAATTGGAACCTCTCTTAGACTTTTAATTCGAGCTGAATTAGGAA
CTCCAGGATCTTTAATTGGAGATGATCAAATTTATAATACTATTGTTACAGCTCATGCATTTATTATAATTTTTTTTATA
GTAATACCAATTATAATCGGAGGATTTGGAAATTGATTAGTTCCTTTAATACTAGGAGCACCTGATATAGCTTTCCCACG
GATAAATAATATAAGTTTTTGATTATTACCACCATCACTAACATTATTGATTTCTAGAAGAATTGTTGAAAATGGAGCAG
GAACTGGTTGAACTGTTTATCCTCCTTTATCTTCAAATATTGCTCATGGGGGAAGCTCTGTAGATTTAGCAATTTTTTCC
CTTCATTTAGCWGGTATTTCATCAATTTTAGGGGCTATTAMTTTTAYTACAACAATTATTAATATACGAGTTAATGGTCT
ATCATTTGATCAAATACCTTTATTTGTCTGATCTGTAGGAATTACAGCATTATTATTATTACTTTCTCTACCAGTATTAG
CAGGTGCTATTACTATACTACTAACTGATCGAAATTTAAATACATCTTTTTTTGATCCTGCAGGAGGGGGAGATCCAATC
CTCTACCAACATTTATTTTGATTTTTT
>LSTEM030-18|Chilo_infuscatellus|am00786|
AACTTTATATTTTATTTTTGGAATTTGAGCAGGAATAATTGGAACTTCCCTTAGACTTTTAATTCGAGCTGAATTAGGGA
CTCCAGGATCTTTAATTGGAGATGATCAAATTTATAATACTATTGTTACAGCTCATGCATTTATTATAATTTTTTTTATA
GTAATACCAATTATAATTGGGGGATTTGGAAATTGATTAGTTCCTTTAATACTAGGGGCACCTGATATAGCTTTCCCACG
AATAAATAATATAAGCTTTTGATTATTACCACCATCATTAACATTATTAATTTCTAGAAGAATTGTTGAAAATGGGGCAG

GGACTGGTTGAACTGTTTATCCCCCTTTATCCTCAAATATTGCTCATGGTGGAAGCTCTGTAGATTTAGCAATTTTTTCC
CTTCACTTAGCGGGTATTTCATCAATTTTAGGAGCTATTAATTTTATTACAACAATTATTAATATACGAGTTAATGGCCT
ATCATTTGATCAAATACCTTTATTTGTTTGATCTGTAGGTATTACAGCACTTTTATTATTACTTTCTCTACCAGTATTAG
CAGGTGCTATTACTATACTACTAACTGATCGAAATTTAAATACATCTTTTTTTGACCCTGCTGGAGGGGGGGATCCAATC
CTTTATCAACATTTATTTTGATTTTTT
>LSTEM031-18|Chilo_partellus|am00787|
AACTTTATATTTTATTTTTGGAATTTGAGCAGGAATAATTGGAACATCCCTTAGATTATTAATTCGTGCAGAATTAGGAA
CTCCTGGATCTTTAATTGGAGATGATCAAATTTATAATACTATTGTAACAGCACATGCATTTATTATAATTTTTTTTATA
GTTATACCAATTATAATTGGTGGATTTGGAAATTGATTAGTACCTTTAATATTGGGAGCCCCAGATATAGCTTTCCCACG
AATAAATAATATAAGATTTTGATTATTACCACCATCATTAACTTTACTAATTTCTAGAAGAATTGTTGAAAATGGAGCTG
GAACAGGATGAACAGTGTACCCCCCACTATCATCTAATATTGCTCATGCTGGAAGTTCAGTAGATTTAGCAATTTTTTCT
TTACATTTAGCTGGTATTTCATCAATTCTTGGTGCTATTAATTTTATTACAACAATCATTAATATACGAATTAATGGATT
ATTTTTTGATCAAATACCATTATTTGTTTGATCTGTAGGTATTACAGCTTTATTATTATTACTTTCTTTACCTGTTTTAG
CTGGGGCTATTACTATATTATTAACAGATCGAAATTTAAATACATCCTTTTTCGATCCTGCTGGAGGAGGAGATCCTATT
CTTTATCAACACTTATTTTGATTTTTT
>LSTEM032-18|Chilo_partellus|am00788|
AACTTTATATTTTATTTTTGGAATTTGAGCAGGAATAATTGGAACATCCCTTAGATTATTAATTCGTGCAGAATTAGGAA
CTCCTGGATCTTTAATTGGAGATGATCAAATTTATAATACTATTGTAACAGCACATGCATTTATTATAATTTTTTTTATA
GTTATACCAATTATAATTGGTGGATTTGGAAATTGATTAGTACCTTTAATATTGGGAGCCCCAGATATAGCTTTCCCACG
AATAAATAATATAAGATTTTGATTATTACCACCATCATTAACTTTACTAATTTCTAGAAGAATTGTTGAAAATGGAGCTG
GAACAGGATGAACAGTGTACCCCCCACTATCATCTAATATTGCTCATGCTGGAAGTTCAGTAGATTTAGCAATTTTTTCT
TTACATTTAGCTGGTATTTCATCAATTCTTGGTGCTATTAATTTTATTACAACAATCATTAATATACGAATTAATGGATT
ATTTTTTGATCAAATACCATTATTTGTTTGATCTGTAGGTATTACAGCTTTATTATTATTACTTTCTTTACCTGTTTTAG
CTGGGGCTATTACTATATTATTAACAGATCGAAATTTAAATACATCCTTTTTCGATCCTGCTGGAGGAGGAGATCCTATT
CTTTATCAACACTTATTTTGATTTTTT
>LSTEM033-18|Chilo_infuscatellus|am11008|
AACTTTATATTTTATTTTTGGAATTTGAGCAGGAATAATTGGAACCTCTCTTAGACTTTTAATTCGAGCTGAATTAGGAA
CTCCAGGATCTTTAATTGGAGATGATCAAATTTATAATACTATTGTTACAGCTCATGCATTTATTATAATTTTTTTTATA
GTAATACCAATTATAATCGGAGGATTTGGAAATTGATTAGTTCCTTTAATACTAGGAGCACCTGATATAGCTTTCCCACG
GATAAATAATATAAGTTTTTGATTATTACCACCATCACTAACATTATTGATTTCTAGAAGAATTGTTGAAAATGGAGCAG
GAACTGGTTGAACTGTTTATCCTCCTTTATCTTCAAATATTGCTCATGGGGGAAGCTCTGTAGATTTAGCAATTTTTTCC
CTTCATTTAGCTGGTATTTCATCAATTTTAGGGGCTATTAATTTTATTACAACAATTATTAATATACGAGTTAATGGTCT
ATCATTTGATCAAATACCTTTATTTGTCTGATCTGTAGGAATTACAGCATTATTATTATTACTTTCTCTACCAGTATTA-
----

>LSTEM034-18|Chilo_infuscatellus|am11009|
AACTTTATATTTTATTTTTGGAATTTGAGCAGGAATAATTGGAACCTCTCTTAGACTTTTAATTCGAGCTGAATTAGGAA
CTCCAGGATCTTTAATTGGAGATGATCAAATTTATAATACTATTGTTACAGCTCATGCATTTATTATAATTTTTTTTATA
GTAATACCAATTATAATCGGAGGATTTGGAAATTGATTAGTTCCTTTAATACTAGGAGCACCTGATATAGCTTYCCCACG
GATAAATAATATAAGTTTTTGATTATTACCACCATCACTAACATTATTGATTTCTAGAAGAATTGTTGAAAATGGAGCAG
GAACTGGTTGAACTGTTTATCCTCCTTTATCTTCAAATATTGCTCATGGGGGAAGCTCTGTAGATTTAGCAATTTTTTCC
CTTCATTTAGCTGGTATTTCATCAATTTTAGGAGCTATTAATTTTATTACAACAATTATTAATATACGAGTTAATGGTCT
ATCATTTGATCAAATACCTTTATTTGTCTGATCTGTAGGAATTACAGCATTATTATTATTACTTTCTCTACCAGTATTA-
----

>LSTEM035-18|Chilo_infuscatellus|am11010|
AACTTTATATTTTATTTTTGGAATTTGAGCAGGAATAATTGGAACCTCTCTTAGACTTTTAATTCGAGCTGAATTAGGAA
CTCCAGGATCTTTAATTGGAGATGATCAAATTTATAATACTATTGTTACAGCTCATGCATTTATTATAATTTTTTTTATA
GTAATACCAATTATAATCGGAGGATTTGGAAATTGATTAGTTCCTTTAATACTAGGAGCACCTGATATAGCTTTCCCACG
GATAAATAATATAAGTTTTTGATTATTACCACCATCACTAACATTATTGATTTCTAGAAGAATTGTTGAAAATGGAGCAG
GAACTGGTTGAACTGTTTATCCTCCTTTATCTTCAAATATTGCTCATGGGGGAAGCTCTGTAGATTTAGCAATTTTTTCC
CTTCATTTAGCTGGTATTTCATCAATTTTAGGAGCTATTAATTTTATTACAACAATTATTAATATACGAGTTAATGGTCT
ATCATTTGATCAAATACCTTTATTTGTCTGATCTGTAGGAATTACAGCATTATTATTATTACTTTCTCTACCAGTATTA-
----

>LSTEM036-18|Chilo_infuscatellus|am11011|
AACTTTATATTTTATTTTTGGAATTTGAGCAGGAATAATTGGAACCTCTCTTAGACTTTTAATTCGAGCTGAATTAGGAA

CTCCAGGATCTTTAATTGGAGATGATCAAATTTATAATACTATTGTTACAGCTCATGCATTTATTATAATTTTTTTATA
GTAATACCAATTATAATCGGAGGATTTGGAAATTGATTAGTTCCTTTAATACTAGGAGCACCTGATATAGCTTTCCCACG
GATAAATAATATAAGTTTTTGATTATTACCACCATCACTAACATTATTGATTTCTAGAAGAATTGTTGAAAATGGAGCAG
GAACTGGTTGAACTGTTTATCCTCCTTTATCTTCAAATATTGCTCATGGGGGAAGCTCTGTAGATTTAGCAATTTTTTCC
CTTCATTTAGCTGGTATTTCATCAATTTTAGGAGCTATTAATTTTATTACAACAATTATTAATATACGAGTTAATGGTCT
ATCATTTGATCAAATACCTTTATTTGTCTGATCTGTAGGAATTACAGCATTATTATTATTACTTTCTCTACCAGTATTA-
----

>LSTEM037-18|Chilo_infuscatellus|am11013|
AACTTTATATTTTATTTTTGGAATTTGAGCAGGAATAATTGGAACCTCTCTTAGACTTTTAATTCGAGCTGAATTAGGAA
CTCCAGGATCTTTAATTGGAGATGATCAAATTTATAATACTATTGTTACAGCTCATGCATTTATTATAATTTTTTTATA
GTAATACCAATTATAATCGGAGGATTTGGAAATTGATTAGTTCCTTTAATACTAGGAGCACCTGATATAGCTTTCCCACG
GATAAATAATATAAGTTTTTGATTATTACCACCATCACTAACATTATTGATTTCTAGAAGAATTGTTGAAAATGGAGCAG
GAACTGGTTGAACTGTTTATCCTCCTTTATCTTCAAATATTGCTCATGGGGGAAGCTCTGTAGATTTAGCAATTTTTTCC
CTTCATTTAGCTGGTATTTCATCAATTTTAGGAGCTATTAATTTTATTACAACAATTATTAATATACGAGTTAATGGTCT
ATCATTTGATCAAATACCTTTATTTGTCTGATCTGTAGGAATTACAGCATTATTATTATTACTTTCTCTACCAGTATTA-
----

>LSTEM038-18|Chilo_infuscatellus|am11015|
AACTTTATATTTTATTTTTGGAATTTGAGCAGGAATAATTGGAACCTCTCTTAGACTTTTAATTCGAGCTGAATTAGGAA
CTCCAGGATCTTTAATTGGAGATGATCAAATTTATAATACTATTGTTACAGCTCATGCATTTATTATAATTTTTTTATA
GTAATACCAATTATAATCGGAGGATTTGGAAATTGATTAGTTCCTTTAATACTAGGAGCACCTGATATAGCTTTCCCACG
GATAAATAATATAAGTTTTTGATTATTACCACCATCACTAACATTATTGATTTCTAGAAGAATTGTTGAAAATGGAGCAG
GAACTGGTTGAACTGTTTATCCTCCTTTATCTTCAAATATTGCTCATGGGGGAAGCTCTGTAGATTTAGCAATTTTTTCC
CTTCATTTAGCTGGTATTTCATCAATTTTAGGAGCTATTAATTTTATTACAACAATTATTAATATACGAGTTAATGGTCT
ATCATTTGATCAAATACCTTTATTTGTCTGATCTGTAGGAATTACAGCATTATTATTATTACTTTCTCTACCAGTATTA-
----

>LSTEM039-18|Chilo_infuscatellus|am11016|
AACTTTATATTTTATTTTTGGAATTTGAGCAGGAATAATTGGAACCTCTCTTAGACTTTTAATTCGAGCTGAATTAGGAA
CTCCAGGATCTTTAATTGGAGATGATCAAATTTATAATACTATTGTTACAGCTCATGCATTTATTATAATTTTTTTATA
GTAATACCAATTATAATCGGAGGATTTGGAAATTGATTAGTTCCTTTAATACTAGGAGCACCTGATATAGCTTTCCCACG
GATAAATAATATAAGTTTTTGATTATTACCACCATCACTAACATTATTGATTTCTAGAAGAATTGTTGAAAATGGAGCAG
GAACTGGTTGAACTGTTTATCCTCCTTTATCTTCAAATATTGCTCATGGGGGAAGCTCTGTAGATTTAGCAATTTTTTCC
CTTCATTTAGCTGGTATTTCATCAATTTTAGGAGCTATTAATTTTATTACAACAATTATTAATATACGAGTTAATGGTCT
ATCATTTGATCAAATACCTTTATTTGTCTGATCTGTAGGAATTACAGCATTATTATTATTACTTTCTCTACCAGTATTA-
----

>LSTEM040-18|Chilo_infuscatellus|am11017|
AACTTTATATTTTATTTTTGGAATTTGAGCAGGAATAATTGGAACCTCTCTCAGACTTTTAATTCGAGTTGAATTAGGAA
CTCCAGGATCTTTAATTGGAGATGATCAAATTTATAATACTATTGTTACAGCTCATGCATTTATTATAATTTTTTTATA
GTAATACCAATTATAATCGGAGGATTTGGAAATTGATTAGTTCCTTTAATACTAGGAGCACCTGATATAGCTTTCCCACG
GATAAATAATATAAGTTTTTGATTATTACCACCATCACTAACATTATTGATTTCTAGAAGAATTGTTGAAAATGGAGCAG
GAACTGGTTGAACTGTTTATCCTCCTTTATCTTCAAATATTGCTCATGGGGGAAGCTCTGTAGATTTAGCAATTTTTTCC
CTTCATTTAGCTGGTATTTCATCAATTTTAGGAGCTATTAATTTTATTACAACAATTATTAATATACGAGTTAATGGTCT
ATCATTTGATCAAATACCTTTATTTGTCTGATCTGTAGGAATTACAGCATTATTATTATTACTTTCTCTACCAGTATTA-
----

>LSTEM041-18|Chilo_infuscatellus|am11020|
AACTTTATATTTTATTTTTGGAATTTGAGCAGGAATAATTGGAACCTCTCTTAGACTTTTAATTCGAGCTGAATTAGGAA
CTCCAGGATCTTTAATTGGAGATGATCAAATTTATAATACTATTGTTACAGCTCATGCATTTATTATAATTTTTTTATA
GTAATACCAATTATAATCGGAGGATTTGGAAATTGATTAGTTCCTTTAATACTAGGAGCACCTGATATAGCTTTCCCACG
GATAAATAATATAAGTTTTTGATTATTACCACCATCACTAACATTATTGATTTCTAGAAGAATTGTTGAAAATGGAGCAG
GAACTGGTTGAACTGTTTATCCTCCTTTATCTTCAAATATTGCTCATGGGGGAAGCTCTGTAGATTTAGCAATTTTTTCC
CTTCATTTAGCTGGTATTTCATCAATTTTAGGAGCTATTAATTTTATTACAACAATTATTAATATACGAGTTAATGGTCT
ATCATTTGATCAAATACCTTTATTTGTCTGATCTGTAGGAATTACAGCATTATTATTATTACTTTCTCTACCAGTATTA-
----

>LSTEM042-18|Chilo_sacchariphagus|am11023|
AACTTTATATTTTATTTTTGGAATTTGAGCTGGAATAGTTGGAACATCCCTTAGACTTTTAATTCGAGCTGAATTAGGAA
ATCCAGGTTCATTAATCGGAGATGATCAAATTTATAATACTATTGTTACAGCCCATGCATTTATTATAATTTTTTTTATA
GTAATACCAATTATAATTGGAGGATTTGGAAATTGATTAGTTCCATTAATATTAGGGGCTCCTGATATAGCCTTCCCTCG
TCTAAATAATATAAGATTTTGATTATTACCCCCTTCTTTAACCCTTCTAATTTCTAGAAGAATCGTTGAAAATGGAGCAG
GAACTGGATGAACAGTCTACCCCCCCCTATCTTCCAATATTTCACATGCTGGAAGTTCAGTAGATTTAGCCATCTTCTCC
CTTCATTTAGCTGGAATTTCTTCAATTTTAGGAGCTATCAATTTCATTACTACAATTATTAATATACGAATTAATGGATT
ATTATTTGATCAAATACCATTATTTGTTTGATCTGTTGGTATTACAGCATTACTTCTCCTCCTTTCTTTACCAGTATTAG
CAGGTGCTATTACTATACTATTAACTGACCGAAATTTAAATACATCTTTTTTTGACCCAGCTGGAGGAGGTGATCCAATT
TTATATCAACATTTATTTTGATTTTTT
>LSTEM043-18|Scirpophaga_excerptalis|am11065|
GACATTATATTTTATTTTTGGAATTTGAGCTGGTATAGTAGGAACTTCACTTAGTTTACTAATTCGAGCCGAATTAGGTA
CTCCTGGATCACTAATTGGAGATGATCAAATCTATAATACTATTGTAACTGCTCACGCTTTTATTATAATTTTTTTTATA
GTTATACCTATTATAATTGGGGGATTCGGAAACTGATTAGTACCTTTAATATTAGGAGCTCCAGATATGGCTTTCCCCCG
AATAAATAATATAAGTTTTTGATTATTACCCCCTTCTTTAACCCTCCTAATCTCAAGAAGAATTGTTGAAAATGGGGCTG
GAACAGGATGAACTGTTTACCCGCCCCTATCCTCCAATATTGCCCACGGTGGGACTTCTGTAGATTTAGCTATTTTTTCA
TTACATTTAGCTGGAATTTCTTCTATTCTAGGGGCTATTAACTTCATTACAACTATTATTAATATGCGAATTAATGGACT
ATCTTTTGATCAAATACCTTTATTCGTATGAGCAGTTGGTATTACTGCCCTTCTTCTTCTTCTCTCACTACCTGTATTAG
CAGGAGCTATCACTATATTATTAACAGATCGAAACTTAAATACCTCTTTCTTTGACCCAGCAGGAGGTGGGGACCCAATT
CTTTAT---------------------
>LSTEM044-18|Chilo_infuscatellus|am11068|
AACTTTATATTTTATTTTTGGAATTTGAGCAGGAATAATCGGAACTTCTCTTAGACTTTTAATTCGAGCTGAATTAGGAA
CTCCAGGATCTTTAATTGGGGATGATCAAATTTATAATACTATTGTTACAGCTCATGCATTTATTATAATTTTTTTTATA
GTTATACCAATTATAATCGGAGGATTTGGAAATTGATTAGTTCCTCTAATACTAGGAGCACCTGATATAGCTTTCCCACG
AATAAATAATATAAGTTTTTGATTATTACCACCATCATTAACATTATTGATTTCTAGAAGAATTGTTGAAAATGGAGCAG
GAACTGGTTGAACTGTTTATCCTCCTTTATCCTCAAATATTGCCCATGGTGGGAGATCTGTAGATTTAGCAATTTTTTCT
CTTCATTTAGCAGGTATTTCATCAATTTTAGGGGCTATTAATTTTATTACAACAATTATTAATATACGAGTTAATGGTCT
ATCATTTGATCAAATACCCCTATTTGTTTGATCTGTAGGTATTACAGCACTATTATTATTACTTTCTTTACCAGTATTAG
CAGGTGCTATTACTATACTACTAACTGATCGAAATTTAAATACATCTTTTTTTGATCCTGCTGGAGGGGGAGATCCAATC
CTATAC---------------------
>LSTEM045-18|Chilo_infuscatellus|am11069|
AACTTTATATTTTATTTTTGGAATTTGAGCAGGAATAATCGGAACTTCTCTTAGACTTTTAATTCGAGCTGAATTAGGAA
CTCCAGGATCTTTAATTGGGGATGATCAAATTTATAATACTATTGTTACAGCTCATGCATTTATTATAATTTTTTTTATA
GTTATACCAATTATAATCGGAGGATTTGGAAATTGATTAGTTCCTCTAATACTAGGAGCACCTGATATAGCTTTCCCACG
AATAAATAATATAAGTTTTTGATTATTACCACCATCATTAACATTATTGATTTCTAGAAGAATTGTTGAAAACGGAGCAG
GAACTGGTTGAACTGTTTATCCTCCTTTATCCTCAAATATTGCCCATGGTGGGAGATCTGTAGATTTAGCAATTTTTTCT
CTTCATTTAGCAGGTATTTCATCAATTTTAGGAGCTATTAATTTTATTACAACAATTATTAATATACGAGTTAATGGTCT
ATCATTTGATCAAATACCCCTATTTGTTTGATCTGTAGGTATTACAGCACTATTATTATTACTTTCTTTACCAGTATTAG
CAGGTGCTATTACTATACTACTAACTGATCGAAATTTAAATACATCTTTTTTTGATCCTGCTGGAGGGGGAGATCCAATC
CTATAC---------------------
>LSTEM046-18|Sesamia_calamistis|am11074|
AACCTTATATTTTATTTTTGGAATTTGAGCAGGAATAGTAGGAACTTCATTAAGTTTATTAATTCGAGCTGAATTAGGAA
CTCCTGGCTCTTTAATYGGAGATGATCAAATTTATAATACTATTGTCACAGCTCATGCTTTTATTATRATTTTTTTTATA
GTTATACCTATCATAATTGGAGGATTTGGAAACTGACTTGTACCTTTYATATTAGGAGCACCAGATATAGCATTTCCACG
AATAAATAATATAAGATTTTGATTATTACCCCCATCCTTAACCCTGTTAATTTCAAGTAGAATCGTAGAAAACGGAGCAG
GAACAGGATGAACAGTATATCCCCCACTTTCATCTAATATTGCTCATGGGGGAAGATCAGTAGATTTAGCTATTTTTTCT
CTTCATTTAGCTGGGATTTCATCTATTTTAGGAGCAATTAATTTTATTACAACAATTATTAATATACGATTAAATAGTCT
ATCATTCGATCAAATACCCCTATTCATTTGGGCTGTTGGAATTACTGCCTTTTTATTACTATTATCTTTACCTGTTTTAG
CGGGAGCTATTACTATATTACTTACAGATCGAAATTTAAATACTTCATTTTTTGATCCTGCAGGAGGAGGAGATCCAATT
TTATAT---------------------
>LSTEM047-18|Bathytricha_truncata|am11075|
AACATTATATTTCATCTTTGGAATTTGAGCAGGAATAGTAGGAACTTCTTTAAGACTATTAATTCGAGCTGAATTAGGAA
CTCCTGGATCTTTAATTGGGGATGATCAAATTTATAATACTATTGTAACAGCTCATGCCTTTATTATAATTTTTTTTATG
GTTATGCCAATTATAATTGGAGGATTTGGAAATTGACTTGTACCTTTAATGTTAGGGGCACCTGATATGGCATTCCCACG
AATAAATAATATAAGTTTTTGATTACTACCCCCTTCTTTAACTCTACTTATTTCGAGAAGAGTTGTAGAAAATGGAGCAG
GAACTGGGTGAACAGTATACCCCCCACTCTCATCTAATATTGCCCATAGAGGAAGATCTGTAGATTTAGCTATTTTTTCC

CTTCATTTAGCTGGAATTTCTTCTATTTTAGGAGCTATTAATTTTATTACAACTATTATTAACATACGATTAAATAATTT
ATCTTTTGATCAAATACCTTTATTTATTTGAGCTGTAGGAATTACAGCATTTTTATTATTATTATCATTACCCGTATTGG
CTGGGGCTATTACTATATTATTAACAGATCGAAATTTAAATACATCATTTTTTGATCCTGCGGGAGGAGGAGACCCAATC
TTATAT---------------------
>LSTEM048-18|Polyocha_depressellus|am11080|
ACTTTTATATTTTATTTTTGGAATTTGATCAGGTATAGTAGGTACTTCTTTAAGTCTTCTTATTCGAGCTGAATTAGGAA
CTCCTAGATCTTTAATTGGAGATGACCAAATTTATAATACTATTGTTACTGGGCATGCTTTTATTATAATTTTTTTTATA
GTTATACCTATTATAATTGGAGGATTTGGAAATTGATTAGTTCCTTTAATATTAGGAGCCCCAGATATAGCTTTCCCTCG
AATAAATAATATAAGATTTTGACTTTTACCTCCCTCTCTTAATTTATTAATTTCTAGAAGAATTGTAGAAAATGGAGCTG
GAACAGGTTGAACAGTTTATCCCCCTTTATCTTCTAATATTGCTCATAGTGGAAGTTCTGTTGATCTTGCTATTTTTTCT
TTACATCTAGCAGGAATTTCTTCTATTTTAGGTGCTATTAATTTTATTACTACTATTATTAATATAAAATTAAATGGTTT
ATCTTTTGATCAAATACCTTTATTTGTTTGAGCTGTAGGAATTACAGCTTTATTATTACTTTTATCCTTGCCAGTATTAG
CAGGAGCTATTACTATATTATTAACTGACCGAAATTTAAATACTTCTTTCTTTGACCCTGCAGGAGGAGGAGACCCAATT
TTATAT---------------------
>LSTEM049-18|Chilo_sacchariphagus|am11083|

--ATGATTTTTTTATA
GTAATACCAATYATAATTGGAGGATTTGGAAATTGATTAGTTCCMTTAATATTAGGGGCTCCTGATATAGCCTTCCCTCG
TCTAAATAATATAAGATTTTGATTATTACCCCCTTCTTTAACCCTTCTAATTTCTAGAAGAATCGTTGAAAATGGAGCAG
GAACTGGATGAACAGTCTACCCCCCCCTATCTTCCAATATTTCWCATGCTGGAAGTTCAGTAGATTTAGCCATCTTCTCC
CTTCATTTAGCTGGAATTTCTTCAATTTTAGGAGCTATCAATTTCATTACTACAATTATTAATATACGAATTAATGGATT
ATTATTTGATCAAATACCATTATTTGTTTGATCTGTTGGTATTACAGCATTACTTCTCCTCCTTTCTTTACCAGTATTAG
CAGGTGCTATTACTATACTATTAACTGACCGAAATTTAAATACATCTTTTTTTGACCCAGCTGGAGGAGGTGATCCAATT
TTATAT---------------------
>LSTEM050-18|Chilo_sacchariphagus|am11085|
AACTTTATATTTTATTTTTGGAATTTGAGCTGGAATAGTTGGAACATCCCTTAGACTTTTAATTCGAGCTGAATTAGGAA
ATCCAGGTTCATTAATCGGRGATGATCAAATTTATAATACTATTGTTACAGCCCATGCATTTATTATAATTTTTTTTATA
GTAATACCAATTATAATTGGAGGATTTGGAAATTGATTAGTTCCATTAATATTAGGGGCTCCTGATATAGCCTTCCCTCG
TCTAAATAATATAAGATTTTGATTATTACCCCCTTCTTTAACCCTTCTAATTTCTAGAAGAATCGTTGAAAATGGAGCAG
GAACTGGATGAACAGTCTACCCCCCCCTATCTTCCAATATTTCACATGCTGGAAGTTCAGTAGATTTAGCCATCTTCTCC
CTTCATTTAGCTGGAATTTCTTCAATTTTAGGAGCTATCAATTTCATTACTACAATTATTAATATACGAATTAATGGATT
ATTATTTGATCAAATACCATTATTTGTTTGATCTGTTGGTATTACAGCATTACTTCTCCTCCTTTCTTTACCAGTATTAG
CAGGTGCTATTACTATACTATTAACTGACCGAAATTTAAATACATCTTTTTTTGACCCAGCTGGAGGAGGTGATCCAATT
TTATAT---------------------
>LSTEM051-18|Chilo_aff._terrenellus_louisiadalis|am11086|
AACTTTATATTTTATTTTTGGTATTTGAGCTGGAATAATTGGAACATCCCTTAGAATTTTAATTCGTGCTGAATTAGGTA
CTCCAGGATCCTTAATTGGTGACGATCAAATTTATAATACCATTGTAACAGCTCATGCATTTATTATAATTTTTTTTATA
GTAATACCAATTATAATTGGAGGATTTGGAAATTGATTAGTGCCATTAATATTAGGTGCCCCAGATATGGCTTTCCCCCG
AATAAATAACATAAGATTTTGAATATTACCCCCTTCATTAACCTTATTAATTTCTAGAAGAATTGTTGAAAATGGAGCTG
GAACAGGATGAACAGTATACCCCCCACTTTCATCTAATATTGCTCATGCTGGAAGTTCAGTAGATTTAGCAATTTTTTCA
CTACATTTAGCCGGTATTTCTTCAATTTTAGGAGCTATTAATTTTATTACAACAATTATTAATATACGTATTAATAAATT
ATCATTTGATCAAATACCTTTATTTGTTTGATCTGTAGGTATTACAGCATTACTTTTATTACTTTCATTACCTGTTTTAG
CTGGAGCTATTACTATACTACTAACGGATCGAAATTTAAATACATCTTTTTTTGACCCTGCTGGAGGAGGGGATCCTATT
CTTTAC---------------------
>LSTEM052-18|Chilo_aff._terrenellus_louisiadalis|am11091|
AACTTTATATTTTATTTTTGGTATTTGAGCTGGAATAATTGGAACATCCCTTAGAATTTTAATTCGTGCTGAATTAGGTA
CTCCAGGATCCTTAATTGGTGACGATCAAATTTATAATACCATTGTAACAGCTCATGCATTTATTATAATTTTTTTTATA
GTAATACCAATTATAATTGGAGGATTTGGAAATTGATTAGTGCCATTAATATTAGGTGCCCCAGATATGGCTTTCCCCCG
AATAAATAACATAAGATTTTGAATATTACCCCCTTCATTAACCTTATTAATTTCTAGAAGAATTGTTGAAAATGGAGCTG
GAACAGGATGAACAGTATACCCCCCACTTTCATCTAATATTGCTCATGCTGGAAGTTCAGTAGATTTAGCAATTTTTTCA
CTACATTTAGCCGGTATTTCTTCAATTTTAGGAGCTATTAATTTTATTACAACAATTATTAATATACGTATTAATAAATT
ATCATTTGATCAAATACCTTTATTTGTTTGATCTGTAGGTATTACAGCATTACTTTTATTACTTTCATTACCTGTTTTAG
CTGGAGCTATTACTATACTACTAACGGATCGAAATTTAAATACATCTTTTTTTGACCCTGCTGGAGGAGGGGATCCTATT
CTTTAC---------------------
>LSTEM053-18|Chilo_auricilius|am11097|
AACTTTATACTTTATTTTTGGAATTTGAAGTGGAATAATTGGAACATCTCTAAGACTTTTAATTCGTGCTGAATTAGGAA
CTCCAGGGTCATTAATTGGAGATGATCAAATTTACAATACTATTGTTACAGCTCATGCATTTATTATAATTTTTTTTATA

GTTATACCAATTATAATTGGAGGCTTTGGTAATTGATTAGTACCATTAATACTAGGGGCTCCTGATATAGCCTTCCCTCG
AATAAATAATATAAGATTTTGATTATTGCCCCCATCATTAACATTATTAATTTCTAGAAGAATTGTAGAAAATGGAGCTG
GAACAGGATGAACGGTTTACCCCCCCCTTTCATCAAATATTGCCCATGGAGGAAGTTCTGTAGATTTAGCTATTTTTTCT
CTTCATTTAGCTGGTATTTCCTCAATTTTAGGAGCTATTAATTTTATTACAACAATTATTAATATACGAATTAATAAACT
ATCATTTGATCAATTACCATTATTTGTTTGATCTGTTGGTATTACAGCTTTATTATTACTACTTTCATTGCCGGTACTAG
CTGGAGCTATTACTATACTTTTAACTGATCGAAACTTAAATACATCTTTTTTTGACCCTGCTGGAGGAGGAGACCCAATT
CTTTAT---------------------
>LSTEM054-18|Chilo_auricilius|am11098|
-ACTTTATACTTTATTTTTGGAATTTGGAGTGGAATAATTGGAACATCTCTAAGACTTTTAATTCGTGCTGAATTAGGAA
CTCCAGGGTCATTAATTGGAGATGATCAAATTTACAATACTATTGTTACAGCTCATGCATTTATTATAATTTTTTTTATA
GTTATACCAATTATAATCGGAGGCTTTGGTAATTGATTAGTACCATTAATACTAGGGGCTCCTGATATAGCCTTCCCTCG
AATAAATAATATAAGATTTTGATTATTGCCCCCATCATTAACATTATTAATTTCTAGAAGAATTGTAGAAAATGGAGCTG
GAACAGGATGAACGGTTTACCCCCCCCTTTCATCAAATATTGCCCATGGAGGAAGTTCTGTAGATTTAGCTATTTTTTCT
CTTCATTTAGCTGGTATTTCCTCAATTTTAGGAGCTATTAATTTTATTACAACAATTATTAATATACGAATTAATAAACT
ATCATTTGATCAATTACCATTATTTGTTTGATCTGTTGGTATTACAGCTTTATTATTACTACTTTCATTGCCGGTACTAG
CTGGAGCTATTACTATACTTTTAACTGATCGAAACTTAAATACATCTTTTTTTGACCCTGCTGGAG--------------
----
>LSTEM055-18|Tetramoera_sp.|am11099|
AACATTATATTTTATTTTTGGAATTTGAGCCGGAATAATTGGAACATCTCTAAGATTATTAATTCGAGCAGAATTAGGAA
ATCCTGGCTCTTTAATTGGAGATGATCAAATTTATAATACTATTGTAACTGCTCATGCTTTTATTATAATTTTTTTCATA
GTTATACCTATCATAATTGGAGGATTTGGAAATTGATTAGTACCATTAATATTAGGAGCCCCTGATATAGCTTTTCCTCG
TATAAATAATATAAGATTTTGATTACTCCCTCCTTCTATTATATTATTAATTTCAAGAAGAATTGTAGAAAATGGAGCAG
GAACAGGATGAACAGTTTATCCCCCCCTTTCATCTAATATTGCCCATAGAGGTAGATCAGTAGATCTAGCTATTTTTTCT
TTACATTTAGCTGGAATTTCTTCTATTTTAGGAGCTGTAAACTTTATTACAACTATTATTAATATACGACCAAATAATAT
AAGATTAGATCAAATACCCCTATTTGTTTGAGCTGTTGGCATTACAGCTCTTCTTTTATTATTATCTTTACCAGTATTAG
CAGGAGCTATTACTATACTCTTAACAGACCGTAATTTAAATACTTCATTTTTTGATCCTGCTGGTGGAGGAGATCCAATT
TTATAC---------------------
>LSTEM056-18|Chilo_terrenellus|am12368|
AACTTTATATTTTATTTTTGGTATTTGAGCTGGAATAATTGGAACATCTCTTAGAATTTTAATTCGCGCTGAATTAGGTA
CTCCAGGATCCTTAATTGGTGATGATCAAATTTATAATACTATTGTAACAGCTCATGCATTTATTATAATTTTTTTTATA
GTAATACCAATTATAATTGGAGGATTTGGAAATTGATTAGTGCCATTAATWTTAGGTGCCCCAGATATGGCCTTCCCCCG
AATAAATAATATAAGATTTTGAATATTACCCCCCTCATTAACTTTATTAATTTCTAGAARAATTGTTGAAAATGGAGCTG
GAACAGGATGAACAGTATACCCCCCACTTTCATCTAAWATTGCTCATGCTGGAAGTTCAGTARATTTAGCAATTTTTTCA
CTACATTTAGCCGGTATTTCTTCAATTTTAGGAGCTATTAATTTTATTACAACAATTATTAATATACGTATTAATAAATT
ATCATTTGATCAAATACCTTTATTTGTTTGATCTGTAGGTATTACAGCATTACTTTTATTACTTTCATTACCTGTTTTAG
CTGGAGCTATTACTATACTACTAACAGATCGGAATTTAAATACATCTTTTTTTGACCCTGCCGGAGGAGGAGATCCTATT
CTTTAC---------------------
>LSTEM057-18|Chilo_suppressalis|am12370|
AACTTTATATTTTATTTTTGGTATTTGAGCAGGTATAATTGGAACATCTCTTAGACTTTTAATTCGTGCTGAATTAGGAA
CTCCAGGATCTTTAATTGGAGATGATCAAATTTATAATACCATTGTTACAGCTCATGCATTTATTATAATTTTTTTTATA
GTTATACCAATTATAATTGGTGGATTTGGAAATTGATTAGTACCTTTAATATTAGGAGCTCCTGATATAGCTTTCCCACG
AATAAATAATATAAGATTTTGAATATTACCCCCCTCATTAACTTTACTAATTTCTAGAAGAATTGTTGAAAATGGAGCTG
GAACAGGATGAACAGTGTACCCCCCACTATCATCTAATATTGCTCACGCTGGAAGTTCAGTAGATTTAGCAATTTTCTCT
TTACATTTAGCTGGAATTTCTTCAATTCTAGGTGCTATTAATTTTATTACTACGATTATTAATATACGAATTAATGGTCT
TTCATTTGATCAAATACCTTTATTTGTTTGATCCGTAGGTATTACAGCTTTATTATTACTTCTATCTCTACCAGTATTAG
CTGGAGCAATTACAATATTATTAACCGATCGAAATTTAAATACATCTTTTTTTGATCCTGCTGGTGGTGGAAATCCAATT
CTTTAT---------------------
>LSTEM058-18|Busseola_fusca|am12373|
AACATTATATTTTATTTTTGGAATTTGAGCTGGTATAGTAGGAACCTCTTTGAGATTATTAATTCGAGCTGAATTAGGAA
CTCCTGGTTCTTTAATTGGGGATGATCAAATCTATAATACTATTGTTACAGCTCATGCTTTTATTATAATTTTTTTTATA
GTTATACCAATTATAATTGGGGGATTTGGAAATTGACTTGTACCCCTAATATTAGGAGCCCCAGATATAGCATTCCCACG
AATAAATAATATAAGTTTTTGATTATTACCTCCTTCTTTAACTCTATTAATTTCAAGAAGAATTGTAGAAAATGGAGCGG
GAACAGGATGAACAGTATATCCCCCACTTTCATCTAATATTGCTCATGGAGGAAGATCTGTAGATTTAGCTATTTTTTCA
TTACATTTAGCTGGTATTTCTTCTATTTTAGGAGCTATTAATTTTATTACAACAATTATTAATATGCGTTTAAATAGTCT
TTCTTTCGATCAAATACCTTTATTTATCTGAGCTGTAGGAATTACCGCATTTTTATTATTACTATCATTACCAGTTTTAG
CAGGAGCTATTACAATATTATTAACCGATCGAAACTTAAATACATCATTCTTTGATCCTGCGGGAGGAGGTGATCCGATT
TTATAT---------------------

>LSTEM059-18|Crambidae|am12374|
AACTTTATATTTTATATTTGGAATTTGAGCTGGAATATTAGGAACATCTTTAAGTCTTTTAATTCGTGCTGAATTAGGAA
CTTCTAACTCTTTAATTGGAAATGATCAAATTTATAATACTATTGTAACAGCACATGCTTTTATTATAATTTTTTTATA
GTTATACCTATTATAATTGGAGGATTCGGAAATTGATTAGTTCCTTTAATATTAGGAGCTCCTGATATAGCTTTTCCACG
AATAAATAATATAAGATTTTGATTATTACCCCCTTCTTTAACTCTTTTAATTTCTAGAAGAATTGTTGAAAATGGAGCTG
GAACAGGATGAACAGTTTATCCCCCACTTTCTTCTAATATTGCCCATAGTGGAAGATCTGTTGATTTAGCTATTTTTTCT
CTTCATTTAGCTGGAATTTCTTCTATTTTAGGAGCTATTAACTTTATTACAACTATTATTAATATACGAATTAATAGACT
TTCATTTGATCAAATACCTCTTTTTGTCTGATCTGTTGGTATTACTGCTTTACTTCTTCTTCTTTCTCTACCAGTTTTAG
CTGGAGCTATTACTATACTTTTAACTGACCGTAATTTAAATACATCTTTTTTTGATCCTGCAGGAGGTGGAGATCCTATT
CTTTAT---------------------
>LSTEM060-18|Sesamia_calamistis|am12376|
AACATTATATTTTATTTTTGGAATTTGAGCAGGAATAGTAGGAACTTCATTAAGTTTATTAATTCGAGCTGAATTAGGAA
CTCCTGGATCTTTAATTGGAGATGATCAAATTTATAATACTATTGTTACAGCTCATGCTTTTATTATAATTTTTTTATA
GTTATACCTATTATAATTGGAGGATTTGGAAACTGACTTGTACCTTTAATATTAGGAGCACCAGATATAGCATTTCCACG
AATAAATAATATAAGATTTTGATTATTACCCCCATCCTTAACCCTTTTAATTTCAAGTAGAATCGTAGAAAACGGAGCAG
GAACAGGATGAACAGTATATCCCCCACTTTCATCTAATATTGCTCATGGGGGAAGATCAGTAGATTTAGCTATTTTTTCT
CTTCATTTAGCTGGGATTTCATCTATTTTAGGAGCAATTAATTTTATTACAACAATTATTAATATACGATTAAATAGTCT
ATCATTCGATCAAATACCCCTATTCATTTGGGCTGTTGGAATTACTGCCTTTTTATTACTATTATCTTTACCTGTTTTAG
CGGGAGCTATTACTATATTACTTACAGATCGAAATTTAAATACTTCATTTTTTGATCCTGCAGGAGGAGGAGATCCAATT
TTATAT---------------------
>LSTEM061-18|Chilo_tumidicostalis|am12378|
AACTTTATATTTTATTTTTGGAATTTGAGCTGGAATAATTGGTACATCTTTAAGACTCTTAATTCGAGCTGAATTAGGAA
CCCCAGGATCTTTAATTGGAGATGATCAAATTTATAATACTATTGTCACAGCTCATGCATTTATYATAATTTTTTTCATA
GTAATACCAATTATAATTGGTGGATTTGGAAATTGATTGGTACCTTTAATATTAGGAGCTCCAGATATAGCTTTCCCACG
AATAAATAATATAAGATTTTGATTATTACCCCCCTCATTAACACTATTAATTTCAAGTAGAATTGTAGAAAATGGAGCAG
GAACAGGATGAACAGTGTACCCCCCACTATCATCTAATATTGCTCATGCTGGAAGCTCTGTTGATTTAGCAATTTTCTCA
TTACATCKAGCTGGTATTTCCTCTATTTTAGGAGCTATTAATTTTATTACAACAATTATKAATATACGAATTAATGGATT
ATCATTTGATCAGATACCTTTATTGGTATGATCTGTAGGAATTACAGCTTTATTACTATTACTTTCATTACCAGTTTTAG
CRGGAGCTATKACTATATTATTAACAGATCGAAATTTAAATACTTCCTTTTTTGACCCTGCTGGAGGGGGAGATCCTATT
CTCTAC---------------------
>LSTEM062-18|Busseola_segeta|am12379|
AACATTATATTTTATTTTTGGAATTTGAGCTGGTATAGTGGGAACCTCATTAAGATTATTAATTCGAGCTGAATTAGGAA
CTCCTGGTTCTTTAATTGGAGATGATCAAATTTACAATACTATTGTTACAGCACATGCTTTTATTATAATTTTTTTATA
GTTATACCTATTATAATTGGAGGGTTTGGAAATTGACTTGTACCTTTAATATTAGGAGCCCCAGATATAGCATTTCCACG
AATAAATAATATAAGTTTTTGATTATTACCTCCTTCTTTAACATTACTAATTTCGAGAAGAATTGTAGAAAATGGAGCAG
GAACAGGATGAACTGTATACCCCCCACTTTCATCTAATATTGCTCATGGAGGTAGATCTGTGGATTTAGCTATTTTTTCC
TTGCACTTAGCTGGTATTTCTTCTATTTTAGGTGCTATTAATTTCATTACAACAATTATTAATATACGTTTAAATAGTCT
TTCTTTTGATCAAATACCTTTATTTATTTGAGCTGTAGGAATTACTGCATTTTTATTATTATTATCATTACCTGTTTTAG
CAGGGGCTATTACAATATTATTAACTGATCGAAATTTAAACACATCATTTTTTGATCCTGCTGGAGGAGGAGATCCAATT
TTATAT---------------------
>LSTEM063-18|Eoreuma_loftini|am12380|
AACTTTATATTTTATTTTTGGAATTTGAGCTGGAACAGTAGGAACATCTTTAAGTTTATTAATTCGAGCTGAATTAGGAA
ACCCAGGCTCTTTAATTGGAGATGATCAAATTTATAATACTATTGTTACAGCCCATGCATTTATTATAATTTTTTTATA
GTAATACCTATTATAATTGGGGGATTTGGAAATTGATTAGTACCTTTAATATTAGGAGCTCCTGATATAGCATTCCCACG
AATAAATAATATAAGATTTTGATTATTACCCCCATCTTTAACACTACTAATTTCAAGAAGTATTGTTGAAAATGGAGCTG
GAACAGGATGAACAGTTTATCCCCCTCTTTCTTCTAATATTGCTCATGGAGGTAGATCTGTAGATCTAGCAATTTTTTCT
CTTCATTTAGCTGGAATTTCCTCAATTTTAGGAGCTATTAATTTTATTACAACAATTATTAATATACRAATTAATAATTT
ATCCTTTGATCAAATACCTTTATTTGTTTGATCAGTAGGTATTACAGCTTTACTTCTTCTTCTTTCTTTACCAGTATTAG
CTGGAGCTATTACTATATTATTAACAGATCGAAATTTAAATACTTCCTTTTTTGATCCTGCAGGGGGTGGAGATCCAATT
CTTTAT---------------------
>LSTEM064-18|Chilo_partellus|am12382|
AACTTTATATTTTATTTTTGGAATTTGAGCAGGAATAATTGGGACATCCCTTAGATTATTAATTCGTGCAGAATTAGGAA
CTCCTGGATCTTTAATTGGAGATGATCAAATTTATAATACTATTGTAACAGCACACGCATTTATTATAATTTTTTTATA
GTTATACCAATTATAATTGGTGGATTTGGAAATTGATTAGTACCTTTAATATTAGGAGCCCCAGATATAGCTTTCCCACG
AATAAATAATATAAGATTTTGATTATTACCACCATCATTAACTTTATTAATTTCTAGAAGAATTGTTGAAAATGGAGCTG
GAACAGGATGAACAGTGTACCCCCCACTATCATCTAATATTGCTCATGCTGGAAGTTCAGTAGATTTAGCAATTTTTTCT
TTACATTTAGCTGGTATTTCATCAATTCTCGGTGCTATTAATTTTATTACAACAATTATTAATATACGAATTAATGGATT

ATCTTTTGATCAAATACCATTATTTGTTTGATCTGTAGGTATTACAGCTTTATTATTATTACTTTCTTTACCTGTTTTAG
CTGGAGCTATTACTATATTATTAACAGATCGAAATTTAAATACATCCTTTTTCGATCCTGCTGGAGGAGGAGATCCTATT
CTTTAT---------------------
>LSTEM065-18|Sesamia_grisescens|am12384|
AACATTATATTTTATTTTCGGAATTTGAGCTGGTATAGTTGGAACATCACTAAGATTATTAATTCGAGCCGAACTAGGAA
CCCCTGGTTCTTTAATTGGAGACGATCAAATTTATAATACTATTGTTACAGCTCATGCTTTCATTATAATTTTCTTTATA
GTTATACCAATTATAATTGGTGGATTTGGGAATTGACTCGTACCTTTAATATTAGGAGCCCCTGATATAGCATTTCCACG
AATAAATAATATAAGATTTTGATTATTACCCCCCTCTTTAACTTTACTAATTTCAAGTAGAATTGTAGAAAATGGGGCAG
GAACTGGGTGAACAGTGTACCCCCCACTTTCATCTAATATTGCCCATGGAGGAAGATCAGTGGACTTAGCTATTTTTTCC
CTTCATTTAGCGGGTATTTCATCTATTTTAGGAGCTATTAATTTTATCACAACAATTATTAATATACGATTAAATAACTT
ATCCTTTGATCAAATACCTTTATTTATTTGAGCTGTTGGAATTACTGCATTTTTATTATTATTATCTTTACCTGTTTTAG
CAGGAGCTATTACAATATTATTAACCGATCGAAACTTAAATACATCATTTTTCGATCCGGCAGGAGGAGGCGATCCTATT
TTATAC---------------------
>LSTEM066-18|Chilo_sacchariphagus|am12387|
AACTTTATATTTTATTTTTGGAATTTGAGCTGGAATAATTGGAACATCCCTTAGACTTTTAATTCGAGCTGAATTAGGAA
ATCCAGGTTCATTAATTGGAGATGATCAAATTTATAATACTATTGTTACAGCTCATGCATTTATTATAATTTTTTTTATA
GTAATACCAATTATAATCGGAGGATTTGGAAATTGATTAGTTCCATTAATATTAGGAGCTCCTGATATAGCCTTTCCTCG
TTTAAATAAYATAAGATTTTGATTATTACCCCCTTCTTTAACTCTTTTAATTTCTAGAAGAATTGTTGAAAATGGAGCAG
GAACTGGATGAACAGTCTACCCCCCCCTATCTTCCAATATTTCACATGCTGGAAGTTCAGTAGATTTAGCCATCTTTTCT
CTTCATTTAGCAGGAATTTCTTCAATTTTAGGAGCTATTAATTTTATTACTACAATTATTAATATACGAATTAATGGATT
ATTATTTGATCAAATACCATTATTTGTTTGATCTGTTGGTATTACAGCATTACTTCTTCTTCTCTCTTTACCAGTATTAG
CAGGTGCTATTACCATACTATTAACTGACCGAAATTTAAATACATCTTTTTTTGATCCAGCTGGAGGAGGTGATCCAATT
TTATAT---------------------
>LSTEM067-18|Chilo_sacchariphagus|am12388|
AACTTTATATTTTATTTTTGGAATTTGAGCTGGAATAATTGGAACATCCCTTAGACTTTTAATTCGAGCTGAATTAGGAA
ATCCAGGTTCATTAATTGGAGATGATCAAATTTATAATACTATTGTTACAGCTCATGCATTTATTATAATTTTTTTTATA
GTAATACCAATTATAATCGGAGGATTTGGAAATTGATTAGTTCCATTAATATTAGGAGCTCCTGATATAGCCTTTCCTCG
TTTAAATAATATAAGATTTTGATTATTGCCCCCTTCTTTAACTCTTTTAATTTCTAGAAGAATTGTTGAAAATGGAGCAG
GAACTGGATGAACAGTCTACCCCCCCCTATCTTCCAATATTTCACATGCTGGAAGTTCAGTAGATTTAGCCATCTTTTCT
CTTCATTTAGCAGGAATTTCTTCAATTTTAGGAGCTATTAATTTTATTACTACAATTATTAATATACGAATTAATGGATT
ATTATTTGATCAAATACCATTATTTGTTTGATCTGTTGGTATTACAGCATTACTTCTTCTTCTCTCTTTACCAGTATTAG
CAGGTGCTATTACCATACTATTAACTGACCGAAATTTAAATACATCTTTTTTTGATCCAGCTGGAGGAGGTGATCCAATT
TTATAT---------------------
>LSTEM068-18|Eldana_saccharina|am12389|
GACATTATACTTTATTTTTGGTATTTGATCAGGGATAGTAGGAACTTCTCTTAGATTACTAATTCGAGCTGAATTAGGAA
ACCCAGGATCTTTAATTGGAGATGATCAAATTTATAATACTATTGTTACAGGCCATGCCTTTATTATAATTTTTTTTATA
GTTATACCTATTATAATTGGAGGATTTGGTAATTGACTCGTACCCCTAATACTCGGAGCCCCCGATATAGCTTTCCCTCG
TATAAATAATATAAGTTTTTGATTATTGCCTCCTTCTCTTTCCCTCCTAATTTTTAGAAGAATTGTTGAAAATGGAGCAG
GAACAGGATGAACAGTTTACCCCCCATTATCCTCAAATATCGCCCATAGAGGAAGTTCTGTAGATTTAGCTATTTTTTCT
CTTCATCTAGCCGGAATCTCATCAATTTTAGGGGCCATTAATTTTATTACAACAGTAATTAATATAAAATTAAATGGCCT
ATCATTTGATCAAATACCTTTATTTGTATGAGCTGTAAGAATTACAGCTCTTCTTTTACTTCTATCTTTACCAGTTTTAG
CAGGTGCAATTACTATACTTCTAACAGATCGTAATTTAAATACATCTTTTTTTGATCCTGCCGGAGGAGGAGATCCTATT
CTTTAC---------------------
>LSTEM069-18|Chilo_aff._terrenellus_louisiadalis|am12394|
AACTTTATATTTTATYTTTGGTATYWKAGCTGGAATAATTGGAACATCCCTTAGAATTTTAATTCGTGCTGAATTAGGTA
CTCCAGGATCCTTAATTGGTGACGATCAAATTTATAATACCATTGTAACAGCTCATGCATTTATTATAATTTTTTTTATA
GTAATACCAATTATAATTGGAGGATTTGGAAATTGATTAGTGCCATTAATATTAGGTGCCCCAGATATGGCTTTCCCCCG
AATAAATAATATAAGATTTTGAATATTACCCCCCTCATTAACCTTATTAATTTCTAGAAGAATTGTTGAAAATGGAGCTG
GAACAGGATGAACAGTATACCCCCCACTTTCATCTAATATTGCTCATGCTGGAAGTTCAGTAGATTTAGCAATTTTTTCA
CTACATTTAGCCGGTATTTCTTCAATTTTAGGAGCTATTAATTTTATTACAACAATTATTAATATACGTATTAATAAATT
ATCATTTGATCAAATACCTTTATTTGTTTGATCTGTAGGTATTACAGCATTACTTTTATTACTTTCATTACCTGTTTTAG
CTGGAGCTATTACTATACTACTAACGGATCGAAATTTAAATACATCTTTTTTTGACCCTGCTGGAGGAGGGGATCCTATT
CTTTACCAACATTTATTTTGATTTTTT
>LSTEM070-18|Chilo_aff._terrenellus_louisiadalis|am12395|
AACTTTATATTTTATTTTTGGTATTTGAGCTGGAATAATTGGAACATCCCTTAGAATTTTAATTCGTGCTGAATTAGGTA
CTCCAGGATCCTTAATTGGTGACGATCAAATTTATAATACCATTGTAACAGCTCATGCATTTATTATAATTTTTTTTATA
GTAATACCGATTATAATTGGAGGATTTGGAAATTGATTAGTACCATTAATATTAGGTGCCCCAGATATGGCTTTCCCTCG

AATAAATAATATAAGATTTTGAATATTACCCCCCTCATTAACCTTATTAATTTCTAGAAGAATTGTTGAAAATGGAGCTG
GAACAGGATGAACAGTATACCCCCCACTTTCATCTAATATTGCTCATGCTGGAAGTTCAGTAGATTTAGCAATTTTTTCA
CTACATTTAGCCGGTATTTCTTCAATTTTAGGAGCTATTAATTTTATTACAACAATTATTAATATACGTATTAATAAATT
ATCATTTGATCAAATACCTTTATTTGTTTGATCTGTAGGTATTACAGCATTACTTTTATTACTTTCATTACCTGTTTTAG
CTGGAGCTATTACTATACTACTAACGGATCGAAATTTAAATACATCTTTTTTTGACCCTGCTGGAGGAGGGGATCCTATT
CTTTACCAACATTTATTTTGATTTTTT
>LSTEM071-18|Sesamia_aff._grisescens|am12397|
AACATTATATTTTATTTTTGGAATTTGAGCTGGTATAGTTGGAACATCACTAAGATTATTAATTCGAGCTGAATTAGGAA
CTCCTGGTTCTTTAATTGGAGACGATCAAATTTATAATACTATTGTTACAGCTCATGCTTTTATTATAATTTTCTTTATA
GTTATACCAATTATAATTGGAGGATTTGGAAATTGACTTGTACCTTTAATATTAGGAGCCCCTGATATAGCATTCCCACG
AATAAATAATATAAGATTTTGATTATTGCCCCCCTCTTTAACTTTACTAATTTCAAGTAGAATTGTAGAAAATGGAGCAG
GAACTGGATGAACGGTGTACCCCCCACTTTCATCTAATATTGCTCATGGGGGAAGATCAGTAGACTTAGCTATTTTTTCC
CTTCATTTAGCAGGTATTTCATCTATTTTAGGAGCTATTAATTTTATCACAACAATTATTAATATACGATTAAATAACTT
ATCCTTTGATCAAATACCTTTATTTATTTGAGCTGTTGGGATTACTGCATTTTTATTATTATTATCTTTACCTGTTTTAG
CAGGAGCTATTACAATATTATTAACTGATCGAAACTTAAATACATCATTTTTTGATCCAGCAGRARSGRCGGAYCCTATT
TTATATCAACATTTATTTTGATTTTTT
>LSTEM072-18|Chilo_aff._terrenellus_louisiadalis|am12398|

--ATTGTAACAGCTCATGCATTTATTATAATTTTTTTTATA
GTAATACCAATTATAATTGGAGGATTTGGAAATTGATTAGTGCCATTAATATTAGGTGCCCCAGATATGGCTTTCCCCCG
AATAAATAATATAAGATTTTGAATATTACCCCCCTCATTAACCTTATTAATTTCTAGAAGAATTGTTGAAAATGGAGCTG
GAACAGGATGAACAGTATACCCCCCACTTTCATCTAATATTGCTCATGCTGGAAGTTCAGTAGATTTAGCAATTTTTTCA
CTACATTTAGCCGGYATTTCTTCAATTTTAGGAGCTATTAATTTTATTACAACAATTATTAATATACGTATTAATAAATT
ATCATTTGATCAAATACCTTTATTTGTTTGATCTGTAGGTATTACAGCATTACTTTTATTACTTTCATTACCTGTTTTAG
CTGGAGCTATTACTATACTACTAACGGATCGAAATTTAAATACATCTTTTTTTGACCCTGCTGTAGGAGGGGATCCTATT
CTTTACCAACATTTATTTTGATTTTTT
>LSTEM073-18|Sesamia_aff._grisescens|am12399|
AACATTATATTTTATTTTTGGAATTTGAGCTGGTATAGTTGGAACATCACTAAGATTATTAATTCGAGCTGAATTAGGAA
CTCCTGGTTCTTTAATTGGAGACGATCAAATTTATAATACTATTGTTACAGCTCATGCTTTTATTATAATTTTCTTTATA
GTTATACCAATTATAATTGGAGGATTTGGAAATTGACTTGTACCTTTAATATTAGGAGCCCCTGATATAGCATTCCCACG
AATAAATAATATAAGATTTTGATTATTGCCCCCCTCTTTAACTTTACTAATTTCAAGTAGAATTGTAGAAAATGGAGCAG
GAACTGGATGAACGGTGTACCCCCCACTTTCATCTAATATTGCTCATGGGGGAAGATCAGTAGACTTAGCTATTTTTTCC
CTTCATTTAGCAGGTATTTCATCTATTTTAGGAGCTATTAATTTTATCACAACAATTATTAATATACGATTAAATAACTT
ATCCTTTGATCAAATACCTTTATTTATTYGAGCTGTTGGGATTACTGCATTTTTATTATTATTATCTTYWCCTGSSTTAG
CAGGAGCTATTACAATATTATTAACTGAT--

>LSTEM074-18|Chilo_aff._terrenellus_louisiadalis|am12400|
AACKTTATATNTTATNTTTGTTATNTGAGCTGGAATAATTGGAACATCCCTTAGAATTTTAATTCGTGCTGAATTAGGTA
CTCCAGGATCCTTAATTGGTGACGATCAAATTTATAATACCATTGTAACAGCTCATGCATTTATTATAATTTTTTTTATA
GTAATACCAATTATAATTGGAGGATTTGGAAATTGATTAGTGCCATTAATATTAGGTGCCCCAGATATGGCTTTCCCCCG
AATAAATAATATAAGATTTTGAATATTACCCCCCTCATTAACCTTATTAATTTCTAGAAGAATTGTTGAAAATGGAGCTG
GAACAGGATGAACAGTATACCCCCCACTTTCATCTAATATTGCTCATGCTGGAAGTTCAGTAGATTTAGCAATTTTTTCA
CTACATTTAGCCGGTATTTCTTCAATTTTAGGAGCTATTAATTTTATTACAACAATTATTAATATACGTATTAATAAATT
ATCATTTGATCAAATACCTTTATTTGTTTGATCTGTAGGTATTACAGCATTACTTTTATTACTTTCATTACCTGNTTTAG
CTGGAGCTATTACTATACTACTAACGGATCKAAATTTAAATACATCTTTTTTTGACCCTGCTGKAGGAGKGRATCSTATT
CTTTACCAACATTTATTTTGATTTTTT
>LSTEM075-18|Chilo_aff._terrenellus_louisiadalis|am12401|
AACTTTATATTTTATTTTTGGTATTTGAGCTGGAATAATTGGAACATCCCTTAGAATTTTAATTCGTGCTGAATTAGGTA
CTCCAGGATCCTTAATTGGTGACGATCAAATTTATAATACCATTGTAACAGCTCATGCATTTATTATAATTTTTTTTATA
GTAATACCAATTATAATTGGAGGATTTGGAAATTGATTAGTGCCATTAATATTAGGTGCCCCAGATATGGCTTTCCCCCG
AATAAATAATATAAGATTTTGAATATTACCCCCCTCATTAACCTTATTAATTTCTAGAAGAATTGTTGAAAATGGAGCTG
GAACAGGATGAACAGTATACCCCCCACTTTCATCTAATATTGCTCATGCTGGAAGTTCAGTAGATTTAGCAATTTTTTCA
CTACATTTAGCCGGTATTTCTTCAATTTTAGGAGCTATTAATTTTATTACAACAATTATTAATATACGTATTAATAAATT
ATCATTTGATCAAATACCTTTATTTGTTTGATCTGTAGGTATTACAGCATTACTTTTATTACTTT---------------
----

>LSTEM076-18|Sesamia_grisescens|am12402|

AACATTATATTTTATTTTCGGAATTTGAGCTGGTATAGTTGGAACCTCACTAAGATTATTAATTCGAGCCGAACTAGGAA
CCCCTGGTTCTTTAATTGGAGACGATCAAATTTATAATACTATTGTTACAGCTCATGCTTTTATTATAATTTTCTTTATA
GTTATACCAATTATAATTGGCGGATTTGGAAATTGACTCGTACCTTTAATATTAGGAGCCCCTGATATAGCATTCCCACG
AATAAATAATATAAGATTTTGATTATTACCCCCCTCTTTAACTTTACTAATTTCAAGTAGAATTGTAGAAAATGGGGCAG
GAACTGGATGAACAGTGTACCCCCCACTTTCATCTAATATTGCCCATGGAGGAAGATCAGTGGACTTAGCTATTTTTTCC
CTTCATTTAGCAGGTATTTCATCTATTTTAGGGGCTATTAATTTTATCACAACAATTATCAATATACGATTAAATAGCTT
ATCCTTTGATCAAATACCTTTATTTATTTGAGCTGTTGGAATTACTGCATTTTTATTATTATTATCTTTACCTGTTTTAG
CAGGAGCTATTACAATATTATTAACCGATCGAAACTTAAATACATCATTTTTCGACCCAGCAGGAGGAGGTGATCCTATT
TTATACCAACATTTATTTTGATTTTTT
>LSTEM077-18|Sesamia_grisescens|am12404|
AACATTATATTTTATTTTCGGAATTTGAGCTGGTATAGTTGGAACCTCACTAAGATTATTAATTCGAGCCGAACTAGGAA
CCCCTGGTTCTTTAATTGGAGACGATCAAATTTATAATACTATTGTTACAGCTCATGCTTTTATTATAATTTTCTTTATA
GTTATACCAATTATAATTGGCGGATTTGGAAATTGACTCGTACCTTTAATATTAGGAGCCCCTGATATAGCATTCCCACG
AATAAATAATATAAGATTTTGATTATTACCCCCCTCTTTAACTTTACTAATTTCAAGTAGAATTGTAGAAAATGGGGCAG
GAACTGGATGAACAGTGTACCCCCCACTTTCATCTAATATTGCCCATGGAGGAAGATCAGTGGACTTAGCTATTTTTTCC
CTTCATTTAGCAGGTATTTCATCTATTTTAGGGGCTATTAATTTTATCACAACAATTATCAATATACGATTAAATAGCTT
ATCCTTTGATCAAATACCTTTATTTATTTGAGCTGTTGGAATTACTGCATTTTTATTATTATTATCTTTACCTGTTTTAG
CAGGAGCTATTACAATATTATTAACCGATCGAAACTTAAATACATCATTTTTCGACCCAGCAGGAGGAGGTGATCCTATT
TTATACCAACATTTATTTTGATTTTTT
>LSTEM078-18|Chilo_aff._terrenellus_louisiadalis|am12406|
AACTTTATATTTTATTTTTGGTATTTGAGCTGGAATAATTGGAACATCCCTTAGAATTTTAATTCGTGCTGAATTAGGTA
CTCCAGGATCCTTAATTGGTGACGATCAAATTTATAATACCATTGTAACAGCTCATGCATTTATTATAATTTTTTTTATA
GTAATACCAATTATAATTGGAGGATTTGGAAATTGATTAGTGCCATTAATATTAGGTGCCCCAGATATGGCTTTCCCCCG
AATAAATAATATAAGATTTTGAATATTACCCCCCTCATTAACCTTATTAATTTCTAGAAGAATTGTTGAAAATGGAGCTG
GAACAGGATGAACAGTATACCCCCCACTTTCATCTAATATTGCTCATGCTGGAAGTTCAGTAGATTTAGCAATTTTTTCA
CTACATTTAGCCGGTATTTCTTCAATTTTAGGAGCTATTAATTTTATTACAACAATTATTAATATACGTATTAATAAATT
ATCATTTGATCAAATACCTTTATTTGTTTGATCTGTAGGTATTACAGCATTACTTTTATTACTTTCATTACCTGTTTTAG
CTGGAGCTATTACTATACTACTAACGGATCGAAATTTAAATACATCTTTTTTGACCCTGCTGGAGGAGGGGATCCTATT
CTTTACCAACATTTATTTTGATTTTTT
>LSTEM079-18|Chilo_aff._terrenellus_louisiadalis|am12409|
AACTTTATATTTTATTTTTGGTATTTGAGCTGGAATAATTGGAACATCCCTTAGAATTTTAATTCGTGCTGAATTAGGTA
CTCCAGGATCCTTAATTGGTGACGATCAAATTTATAATACCATTGTAACAGCTCATGCATTTATTATAATTTTTTTTATA
GTAATACCGATTATAATTGGAGGATTTGGAAATTGATTAGTGCCATTAATATTAGGTGCCCCAGATATGGCTTTCCCTCG
AATAAATAATATAAGATTTTGAATATTACCCCCCTCATTAACCTTATTAATTTCTAGAAGAATTGTTGAAAATGGAGCTG
GAACAGGATGAACAGTATACCCCCCACTTTCATCTAATATTGCTCATGCTGGAAGTTCAGTAGATTTAGCAATTTTTTCA
CTACATTTAGCCGGTATTTCTTCAATTTTAGGAGCTATTAATTTTATTACAACAATTATTAATATACGTATTAATAAATT
ATCATTTGATCAAATACCTTTATTTGTTTGATCTGTAGGTATTACAGCATTACTTTTATTACTTTCATTACCTGTTTTAG
CTGGAGCTATTACTATACTACTAACGGATCGAAATTTAAATACATCTTTTTTGACCCTGCTGGAGGAGGGGATCCTATT
CTTTACCAACATTTATTTTGATTTTTT
>LSTEM080-18|Chilo_aff._terrenellus_louisiadalis|am12410|
AACTTTATATTTTATTTTTGGTATTTGAGCTGGAATAATTGGAACATCCCTTAGAATTTTAATTCGTGCTGAATTAGGTA
CTCCAGGATCCTTAATTGGTGATGATCAAATTTATAATACCATTGTAACAGCTCATGCATTTATTATAATTTTTTTTATA
GTAATACCGATTATAATTGGAGGATTTGGAAATTGATTAGTGCCATTAATATTAGGTGCCCCAGATATGGCTTTCCCTCG
AATAAATAATATAAGATTTTGAATATTACCCCCCTCATTAACCTTATTAATTTCTAGAAGAATTGTTGAAAATGGAGCTG
GAACAGGATGAACAGTATACCCCCCACTTTCATCTAATATTGCTCATGCTGGAAGTTCAGTAGATTTAGCAATTTTTTCA
CTACATTTAGCCGGTATTTCTTCAATTTTAGGAGCTATTAATTTTATTACAACAATTATTAATATACGTATTAATAAATT
ATCATTTGATCAAATACCTTTATTTGTTTGATCTGTAGGTATTACAGCATTACTTTTATTACTTTCATTACCTGTTTTAG
CTGGAGCTATTACTATACTACTAACGGATCGAAATTTAAATACATCTTTTTTGACCCTGCTGGAGGAGGGGATCCTATT
CTTTACCAACATTTATTTTGATTTTTT
>LSTEM081-18|Chilo_aff._terrenellus_louisiadalis|am12412|
AACTTTATATTTTATTTTTGGTATTTGAGCTGGAATAATTGGAACATCCCTTAGAATTTTAATTCGTGCTGAATTAGGTA
CTCCAGGATCCTTAATTGGTGATGATCAAATTTATAATACCATTGTAACAGCTCATGCATTTATTATAATTTTTTTTATA
GTAATACCGATTATAATTGGAGGATTTGGAAATTGATTAGTGCCATTAATATTAGGTGCCCCAGATATGGCTTTCCCTCG
AATAAATAATATAAGATTTTGAATATTACCCCCCTCATTAACCTTATTAATTTCTAGAAGAATTGTTGAAAATGGAGCTG
GAACAGGATGAACAGTATACCCCCCACTTTCATCTAATATTGCTCATGCTGGAAGTTCAGTAGATTTAGCAATTTTTTCA
CTACATTTAGCCGGTATTTCTTCAATTTTAGGAGCTATTAATTTTATTACAACAATTATTAATATACGTATTAATAAATT
ATCATTTGATCAAATACCTTTATTTGTTTGATCTGTAGGTATTACAGCATTACTTTTATTACTTTCATTACCTGNTTTAG

CTGGAGCTATTACTATACTACTAACGGATCGAAATTTAAATACATCTTTTTTGACCCTGCTGGAGGAGGGGATCCTATT
CTTTACCAACATTTATTTTGATTTTTT
>LSTEM082-18|Chilo_aff._terrenellus_louisiadalis|am12413|
AACTTTATATTTTATTTTTGGTATTTGAGCTGGAATAATTGGAACATCCCTTAGAATTTTAATTCGTGCTGAATTAGGTA
CTCCAGGATCCTTAATTGGTGACGATCAAATTTATAATACCATTGTAACAGCTCATGCATTTATTATAATTTTTTTTATA
GTAATACCAATTATAATTGGAGGATTTGGAAATTGATTAGTGCCATTAATATTAGGTGCCCCAGATATGGCTTTCCCCCG
AATAAATAATATAAGATTTTGAATATTACCCCCCTCATTAACCTTATTAATTTCTAGAAGAATTGTTGAAAATGGAGCTG
GAACAGGATGAACAGTATACCCCCCACTTTCATCTAATATTGCTCATGCTGGAAGTTCAGTAGATTTAGCAATTTTTTCA
CTACATTTAGCCGGTATTTCTTCAATTTTAGGAGCTATTAATTTTATTACAACAATTATTAATATACGTATTAATAAATT
ATCATTTGATCAAATACCTTTATTTGTWWGATCTGTAGGTATTACAGCATTACTTTTATTACTTTCATTACCTGNNTTAG
CTGGAGCTATTACCATACTACTAACGGATCNAAATTTAAATACATCTTTTTTGACCCTGCTGGAGGAGGGGATCCTATT
CTTTACCAACATTTATTTTGATTTTTT
>LSTEM083-18|Chilo_aff._terrenellus_louisiadalis|am12414|
AACTTTATATTTTATTTTTGGTATTTGAGCTGGAATAATTGGAACATCCCTTAGAATTTTAATTCGTGCTGAATTAGGTA
CTCCAGGATCCTTAATTGGTGACGATCAAATTTATAATACCATTGTAACAGCTCATGCATTTATTATAATTTTTTTTATA
GTAATACCAATTATAATTGGAGGATTTGGAAATTGATTAGTGCCATTAATATTAGGTGCCCCAGATATGGCTTTCCCCCG
AATAAATAATATAAGATTTTGAATATTACCCCCCTCATTAACCTTATTAATTTCTAGAAGAATTGTTGAAAATGGAGCTG
GAACAGGATGAACAGTATACCCCCCACTTTCATCTAATATTGCTCATGCTGGAAGTTCAGTAGATTTAGCAATTTTTTCA
CTACATTTAGCCGGTATTTCTTCAATTTTAGGAGCTATTAATTTTATTACAACAATTATTAATATACGTATTAATAAATT
ATCATTTGATCAAATACCTTTATTTGTTTGATCTGTAGGTATTACAGCATTACTTTTATTACT-----------------
----

>LSTEM084-18|Chilo_aff._terrenellus_louisiadalis|am12416|
AACTTTATATTTTATTTTTGGTATTTGAGCTGGAATAATTGGAACATCCCTTAGAATTTTAATTCGTGCTGAATTAGGTA
CTCCAGGATCCTTAATTGGTGACGATCAAATTTATAATACCATTGTAACAGCTCATGCATTTATTATAATTTTTTTTATA
GTAATACCAATTATAATTGGAGGATTTGGAAATTGATTAGTGCCATTAATATTAGGTGCCCCAGATATGGCTTTCCCCCG
AATAAATAATATAAGATTTTGAATATTACCCCCCTCATTAACCTTATTAATTTCTAGAAGAATTGTTGAAAATGGAGCTG
GAACAGGATGAACAGTATACCCCCCACTTTCATCTAATATTGCTCATGCTGGAAGTTCAGTAGATTTAGCAATTTTTTCA
CTACATTTAGCCGGTATTTCTTCAATTTTAGGAGCTATTAATTTTATTACAACAATTATTAATATACGTATTAATAAATT
ATCATTTGATCAAATACCTTTATTTGTTTGATCTGTAGGTATTACAGCATTACTTTTATTACTTTCATTACCTGTTTTAG
CTGGAGCTATTACTATACTACTAACGGATCGAAATTTAAATACATCTTTTTTGACCCTGCTGGAGGAGGGGATCCTATT
CTTTACCAACATTTATTTTGATTTTTT
>LSTEM085-18|Scirpophaga_excerptalis|am12419|
---CTTCACTTAGTTTACTAATTCGAGCCGAACTAGGTA
CTCCTGGATCAYTAATCGGAGATGATCAAATCTATAATACTATTGTAACTGCTCACGCTTTTATTATAATTTTTTTTATG
GTTATACCTATTATAATTGGGGGATTCGGAAACTGATTAGTGCCTTTAATATTDGGAGCCCCAGATATAGCHTTTCCCSG
AATAAATAATATAAGTTTTTGATTATTACCCCCTTCTTTAACCCTCTTAATCTCAAGAAGAGTGGTTGAAAATGGAGCTG
GAACAGGATGAACTGTTTACCCGCCCTTATCCTCCAATATTGCWCATGGTGGGASTTCTGTAGATTTAGCCATTTTTTCA
TTACATTTAGCTGGAATTTCTTCTATTCTAGGGGCTATTAACTTCATTACAACTATTATTAATATACGAATTAATGGRCT
ATCTTTTGATCAAATACCTTTATTCGTATGAGCAGTTGGTATTACTGCCCTTCTTCTTCTTCTCTCACTACCTGTATTAG
CGGGAGCTATTACTATATTAYTAACAGATCGAAACTTAAATACCTCTTTCTTTGACCCAGCAGGAGGTGGAGACCCAATT
CTTTATCAACACYTATTT---------
>LSTEM086-18|Chilo_aff._terrenellus_louisiadalis|am12420|

--ATTGTAACAGCTCATGCATTTATTATAATTTTTTTTATA
GTAATACCAATTATAATTGGAGGATTTGGAAATTGATTAGTGCCATTAATATTAGGTGCCCCAGATATGGCTTTCCCCCG
AATAAATAATATAAGATTTTGAATATTACCCCCCTCATTAACCTTATTAATTTCTAGAAGAATTGTTGAAAATGGAGCTG
GAACAGGATGAACAGTATACCCCCCACTTTCATCTAATATTGCTCATGCTGGAAGTTCAGTAGATTTAGCAATTTTTTCA
CTACATTTAGCCGGNATTTCTTCAATTTTAGGAGCTATTAATTTTATTACAACAATTATTAATATACGTATTAATAAATT
ATCATTTGATCAAATACCTTTATTTGTTTGATCTGTAGGTATTACAGCATTACTTTTATTACTTTCATTACCTGTTTTAG
CTGGAGCTATTACTATACTACTAACGGATCGAAATTTAAATACATCTTTTTTGACCCTGCTGGAGGAGGGGATCCTATT
CTTTACCAACATTTATTTTGATTTTTT
>LSTEM087-18|Scirpophaga_excerptalis|am12421|
GACATTATATTTTATTTTTGGAATTTGAGCTGGTATAGTNGGGACTTCACTTAGTTTACTAATTCGNGCTGAACTAGGTA
CTCCTGGATCATTAATCGGAGATGATCAAATCTATAATACTATTGTAACTGCTCACGCTTTTATTATAATTTTTTTTATG
GTTATACCTATTATAATTGGGGGATTCGGGAACTGATTAGTGCCTTTAATATTGGGAGCCCCAGATATAGCCTTYCCCCG
AATAAATAATATAAGTTTTTGATTRTTACCCCCTTCTTTAACCCTCTTAATCTCAAGAAGARTCGTTGAAAATGGAGCTG

GAACAGGATGAACTGTTTACCCGCCCTTATCCTCCAATATTGCYCATRGTGGGACTTCTGTAGATTTAGCCATTTTTTCA
TTACATTTAGCTGGAATTTCTTCTATTCTAGGGGCTATTAACTTCATTACAACTATTATTAATATACGAATTAATGGACT
ATCTTTTGATCAAATACCTTTATTCGTRTGAGCAGTTGGTATTACTGCCCTTCTTCTTCTTCTCTCNCTWCCKGTATTAG
CGGGRGCTATTACTATATTATTAACAGATCGAW--

>LSTEM088-18|Chilo_aff._terrenellus_louisiadalis|am12422|
AACTTTATATTTTATTTTTGGTATTTGAGCTGGAATAATTGGAACATCCCTTAGAATTTTAATTCGTGCTGAATTAGGTA
CTCCAGGATCCTTAATTGGTGACGATCAAATTTATAATACCATTGTAACAGCTCATGCATTTATTATAATTTTTTTTATA
GTAATACCAATTATAATTGGAGGATTTGGAAATTGATTAGTGCCATTAATATTAGGTGCCCCAGATATGGCTTTCCCCCG
AATAAATAATATAAGATTTTGAATATTACCCCCCTCATTAACCTTATTAATTTCTAGAAGAATTGTTGAAAATGGAGCTG
GAACAGGATGAACAGTATACCCCCCACTTTCATCTAATATTGCTCATGCTGGAAGTTCAGTAGATTTAGCAATTTTTTCA
CTACATTTAGCCGGTATTTCTTCAATTTTAGGAGCTATTAATTTTATTACAACAATTATTAATATACGTATTAATAAATT
ATCATTTGATCAAATACCTTTATTTGTTTGATCTGTAGGTATTACAGCATTACTTTTATTACTTTCATTACCTGTTTTAG
CTGGAGCTATTACTATACTACTAACGGATCGAAATTTAAATACATCTTTTTTTGACCCTGCTGGAGGAGGGGATCCTATT
CTTTACCAACATTTATTTTGATTTTTT
>LSTEM089-18|Scirpophaga_excerptalis|am12424|
AACATTATATTTTATTTTTGGAATTTGAGCTGGTATAGTGGGGACTTCACTTAGTTTACTAATTCGAGCCGAACTAGGTA
CTCCTGGATCACTAATTGGAGATGATCAAATCTATAATACTATTGTAACTGCTCACGCTTTTATTATAATTTTTTTTATG
GTTATACCTATTATAATTGGAGGATTCGGTAACTGATTAGTACCTTTAATATTGGGAGCCCCAGATATAGCCTTTCCCCG
AATAAATAATATAAGTTTTTGATTATTACCCCCTTCTTTAACTCTCTTAATCTCAAGAAGAGTCGTTGAAAATGGAGCTG
GGACAGGATGAACTGTTTACCCGCCCTTATCCTCCAATATTGCTCATGGTGGGACTTCTGTAGATTTAGCCATTTTTTCA
TTACATTTAGCTGGAATTTCTTCTATTCTAGGGGCTATTAACTTCATTACAACTATTATTAATATACGAATTAATGGACT
ATCTTTTGATCAAATACCCTTATTCGTATGAGCAGTTGGTATCACTGCCCTTCTTCTTCTTCTCTCACTACCTGTATTAG
CGGGAGCTATTACTATATTACTAACAGATCGAAACTTAAATACCTCTTTCTTTGACCCAGCAGGAGGTGGAGACCCAATT
CTTTATCAACACTTATTTTGATTTTTT
>LSTEM090-18|Chilo_aff._terrenellus_louisiadalis|am12430|
AACTTTATATTTTATTTTTGGTATTTGAGCTGGAATAATTGGAACATCCCTTAGAATTTTAATTCGTGCTGAATTAGGTA
CTCCAGGATCCTTAATTGGTGACGATCAAATTTATAATACCATTGTAACAGCTCATGCATTTATTATAATTTTTTTTATA
GTAATACCAATTATAATTGGAGGATTTGGAAATTGATTAGTGCCATTAATATTAGGTGCCCCAGATATGGCTTTCCCCCG
AATAAATAATATAAGATTTTGAATATTACCCCCCTCATTAACCTTATTAATTTCTAGAAGAATTGTTGAAAATGGAGCTG
GAACAGGATGAACAGTATACCCCCCACTTTCATCTAATATTGCTCATGCTGGAAGTTCAGTAGATTTAGCAATTTTTTCA
CTACATTTAGCCGGTATTTCTTCAATTTTAGGAGCTATTAATTTTATTACAACAATTATTAATATACGTATTAATAAATT
ATCATTTGATCAAATACCTTTATTTGTNTGATCTGTAGGTATTACAGCATTACTTTTATTACTTTCNTTACCNGNNTTAG
CTGGAGCTATTACCATACTACTAACGGA---

>LSTEM091-18|Chilo_aff._terrenellus_louisiadalis|am12431|
AACTTTATATTTTATTTTTGGTATTTGAGCTGGAATAATTGGAACATCCCTTAGAATTTTAATTCGTGCTGAATTAGGTA
CTCCAGGATCCTTAATTGGTGACGATCAAATTTATAATACCATTGTAACAGCTCATGCATTTATTATAATTTTTTTTATA
GTAATACCAATTATAATTGGAGGATTTGGAAATTGATTAGTGCCATTAATATTAGGTGCCCCAGATATGGCTTTCCCCCG
AATAAATAATATAAGATTTTGAATATTACCCCCCTCATTAACCTTATTAATTTCTAGAAGAATTGTTGAAAATGGAGCTG
GAACAGGATGAACAGTATACCCCCCACTTTCATCTAATATTGCTCATGCTGGAAGTTCAGTAGATTTAGCAATTTTTTCA
CTACATTTAGCCGGTATTTCTTCAATTTTAGGAGCTATTAATTTTATTACAACAATTATTAATATACGTATTAATAAATT
ATCATTTGATCAAATACCTTTATTTGTTTGATCTGTAGGTATTACAGCATTACTTTTATTACTTTCATTACCTGTTTTAG
CTGGAGCTATTACTATACTACTAACGGATCGAAATTTARRNACATCTTTTTTTGACCCTGCTG-----------------
----
>LSTEM092-18|Chilo_aff._terrenellus_louisiadalis|am12432|
AACTTTATATTTTATTTTTGGTATTTGAGCTGGAATAATTGGAACATCCCTTAGAATTTTAATTCGTGCTGAATTAGGTA
CTCCAGGATCCTTAATTGGTGACGATCAAATTTATAATACCATTGTAACAGCTCATGCATTTATTATAATTTTTTTTATA
GTAATACCAATTATAATTGGAGGATTTGGAAATTGATTAGTGCCATTAATATTAGGTGCCCCAGATATGGCTTTCCCCCG
AATAAATAATATAAGATTTTGAATATTACCCCCCTCATTAACCTTATTAATTTCTAGAAGAATTGTTGAAAATGGAGCTG
GAACAGGATGAACAGTATACCCCCCACTTTCATCTAATATTGCTCATGCTGGAAGTTCAGTAGATTTAGCAATTTTTTCA
CTACATTTAGCCGGTATTTCTTCAATTTTAGGAGCTATTAATTTTATTACAACAATTATTAATATACGTATTAATAAATT
ATCATTTGATCAAATACCTTTATTTGTTTGATCTGTAGGTATTACAGCATTACTTTTATTACTTTC--------------
----

>LSTEM093-18|Chilo_aff._terrenellus_louisiadalis|am12433|
AACTTTATATTTTATTTTTGGTATTTGAGCTGGAATAATTGGAACATCCCTTAGAATTTTAATTCGTGCTGAATTAGGTA

CTCCAGGATCCTTAATTGGTGACGATCAAATTTATAATACCATTGTAACAGCTCATGCATTTATTATAATTTTTTTATA
GTAATACCAATTATAATTGGAGGATTTGGAAATTGATTAGTGCCATTAATATTAGGTGCCCCAGATATGGCTTTCCCCCG
AATAAATAATATAAGATTTTGAATATTACCCCCCTCATTAACCTTATTAATTTCTAGAAGAATTGTTGAAAATGGAGCTG
GAACAGGATGAACAGTATACCCCCCACTTTCATCTAATATTGCTCATGCTGGAAGTTCAGTAGATTTAGCAATTTTTTCA
CTACATTTAGCCGGTATTTCTTCAATTTTAGGAGCTATTAATTTTATTACAACAATTATTAATATACGTATTAATAAATT
ATCATTTGATCAAATACCTTTATTTGTTTGATCTGTAGGTATTACAGCATTACTTTTATTACTTTCATTACCTGTTTTAG
CTGGAGCTATTACTATACTACTAACGGATCGAAATTTAAATACATCTTTTTTGACCCTGCTGC----------------
----
>LSTEM094-18|Chilo_aff._terrenellus_louisiadalis|am12434|
ARCTTTATATTTTATTTTTGGTANTNGAGCTGGAATAATTGGAACATCCCTTAGAATTTTAATTCGTGCTGAATTAGGTA
CCCCAGGATCCTTAATTGGNGACGATCAAATTTATAATACCATTGTAACAGCTCATGCATTTATTATGATTTTTTTATA
GTAATACCAATTATAATTGGAGGATTTGGAAATTGATTAGTGCCATTAATATTAGGTGCCCCAGATATGGCTTTCCCCCG
AATAAATAATATAAGATTTTGAATATTACCCCCCTCATTAACCTTATTAATTTCTAGAAGAATTGTTGAAAATGGAGCTG
GAACAGGATGAACAGTATACCCCCCACTTTCATCTAATATTGCTCATGCTGGAAGTTCAGTAGATTTAGCAATTTTTTCA
CTACATTTAGCCGGTATTTCTTCAATTTTAGGAGCTATTAATTTTATTACAACAATTATTAATATACGTATTAATAAATT
ATCATTTGATCAAATACCTTTATTTGTTTGATCTGAAGGTATTACAGCATTACTTTTATTACTTTCATTACCTGTTTTAG
CTGGAGCTATTACTATACTACTAACGGATCGAAATTTAAATACATCTTTTTTGACCCTGCTGGAGGAGGGGATCCTATT
CTTTACCAACATTTATTTAGATTTTTT
>LSTEM095-18|Chilo_aff._terrenellus_louisiadalis|am12437|
--GGAACATCCCTTAGAATTTTAATTCGTGCTGAATTAGGTA
CTCCAGGATCCTTAATTGGTGACGATCAAATTTATAATACCATTGTAACAGCTCATGCATTTATTATAATTTTTTTATA
GTAATACCAATTATAATTGGAGGATTTGGAAATTGATTAGTGCCATTAATATTAGGTGCCCCAGATATGGCTTTCCCCCG
AATAAATAATATAAGATTTTGAATATTACCCCCCTCATTAACCTTATTAATTTCTAGAAGAATTGTTGAAAATGGAGCTG
GAACAGGATGAACAGTATACCCCCCACTTTCATCTAATATTGCTCATGCTGGAAGTTCAGTAGATTTAGCAATTTTTTCA
CTACATTTAGCCGGTATTTCTTCAATTTTAGGAGCTATTAATTTTATTACAACAATTATTAATATACGTATTAATAAATT
ATCATTTGATCAAATACCTTTATTTGTTTGATCTGTAGGTATTACAGCATTACTTTTATTACTTTCATTACCTGTTTTAG
CTGGAGCTATTACTATACTACTAACGGATCGAAATTTAAATACATCTTTTTTGACCCTGCTGGAGGAGGGGATCCTATT
CTTTACCAACA----------------
>LSTEM096-18|Sesamia_grisescens|am12438|
---GAACATCASTAAGATTATTAATTCGAGCCGAACTAGGAA
CCCCTGGCTCTTTAATTGGAGACGATCAAATTTATAATACTATTGTTACAGCTCATGCTTTTATTATAATTTTCTTTATA
GTTATACCAATTATAATTGGTGGATTTGGGAATTGACTCGTACCTTTAATATTAGGGGCCCCTGATATAGCATTCCCACG
AATAAATAATATAAGATTTTGATTATTACCCCCCTCTTTAACTTTACTAATTTCAAGTAGAATTGTAGAAAATGGGGCAG
GAACTGGATGGACAGTGTACCCCCCACTTTCATCTAATATTGCCCATGGGGGAAGATCAGTGGACTTAGCTATTTTTTCC
CTTCATTTAGCAGGTATTTCATCTATTTTAGGGGCTATTAATTTTATCACAACAATTATCAATATACGATTAAATAACTT
ATCCTTTGATCAAATACCTTTATTTATTTGAGCTGTTGGAATTACTGCATTTTTATTATTATTATCTTTACCTGTTTTAG
CGGGAGCTATTACAATATTATTAACCGATCGAAACTTAAATACATCATTTTTCGACCCGGCAGGGGGAGGTGATCCTATT
TTATACCAACATYTGTTTTGATTCTTT
>LSTEM097-18|Scirpophaga_excerptalis|am12440|
GACATTATATTTTATTTTTGGAATTTGAGCTGGTATAGTGGGGACTTCACTTAGTTTACTAATTCGAGCCGAACTAGGTA
CTCCTGGATCACTAATCGGAGATGATCAGATCTATAATACTATTGTAACTGCTCACGCTTTTATTATAATTTTTTTTATG
GTTATACCTATTATAATTGGGGGATTCGGAAACTGATTAGTGCCTTTAATATTGGGAGCCCCAGATATAGCCTTTCCCCG
AATAAATAATATAAGTTTTTGATTATTACCCCCCTCTTTAACCCTCTTAATCTCAAGAAGAGTCGTTGAAAATGGAGCTG
GGACAGGATGAACTGTTTACCCGCCCTTATCCTCCAATATTGCTCATGGTGGGACTTCTGTAGATTTAGCCATTTTTTCA
TTACATTTAGCTGGAATTTCTTCTATTCTAGGGGCTATTAACTTCATTACAACTATTATTAATATACGAATTAATGGGCT
ATCTTTTGATCAAATACCTTTATTCGTATGAGCAGTTGGTATCACTGCCCTTCTTCTTCTTCTCTCACTACCTGTATTAG
CGGGAGCTATTACTATATTACTAACAGATCGAAACTTAAATACCTCTTTCTTTGACCCAGCAGGAGGTGGAGACCCAATT
CTTTATCAACACTTATTTTGATTTTTT
>LSTEM098-18|Scirpophaga_excerptalis|am12441|
GACATTATATTTTATTTTTGGAATTTGAGCTGGTATAGTAGGGACTTCACTTAGTTTACTAATTCGAGCCGAACTAGGTA
CTCCTGGATCATTAATCGGAGATGATCAAATCTATAATACTATTGTAACTGCTCACGCTTTTATTATAATTTTTTTCATA
GTTATACCTATTATAATTGGGGGATTCGGGAACTGATTAGTGCCTTTAATATTAGGGGCCCCAGATATAGCCTTCCCCCG
AATAAATAATATAAGTTTTTGATTATTACCCCCCTCTTTAACCCTCTTAATCTCAAGAAGAATCGTTGAAAATGGGGCTG
GAACAGGATGAACTGTTTACCCGCCCTTATCCTCCAATATTGCCCACGGTGGGACTTCTGTAGATTTAGCCATTTTTTCA
CTACATTTAGCTGGAATTTCTTCTATTCTAGGGGCTATTAACTTCATTACAACTATTATTAATATACGAATTAATGGACT
ATCTTTTGATCAAATACCTTTATTCGTATGAGCAGTTGGTATTACTGCCCTTCTTCTTCTTCTCTCACTACCTGTATTAG
CGGGAGCTATTACTATATTATTAACAGATCGAAACTTAAATACCTCTTTCTTTGACCCAGCAGGAGGTGGAGACCCAATT

CTTTATCAACACTTATTTTGATTTTTT
>LSTEM099-18|Scirpophaga_excerptalis|am12442|
GACATTATATTTTATTTTTGGAATTTGAGCTGGTATAGTAGGGACTTCACTTAGTTTACTAATTCGAGCCGAACTAGGTA
CTCCTGGATCATTAATCGGAGATGATCAAATCTATAATACTATTGTAACTGCTCACGCTTTTATTATAATTTTTTTCATA
GTTATACCTATTATAATTGGGGGATTCGGGAACTGATTAGTGCCTTTAATATTAGGGGCCCCAGATATAGCCTTCCCCCG
AATAAATAATATAAGTTTTTGATTATTACCCCCCTCTTTAACCCTCTTAATCTCAAGAAGAATCGTTGAAAATGGGGCTG
GAACAGGATGAACTGTTTACCCGCCCTTATCCTCCAATATTGCCCACGGTGGGACTTCTGTAGATTTAGCCATTTTTTCA
CTACATTTAGCTGGAATTTCTTCTATTCTAGGGGCTATTAACTTCATTACAACTATTATTAATATACGAATTAATGGACT
ATCTTTTGATCAAATACCTTTATTCGTATGAGCAGTTGGTATTACTGCCCTTCTTCTTCTTCTCTCACTACCTGTATTAG
CGGGAGCTATTACTATATTATTAACAGATCGAAACTTAAATACCTCTTTCTTTGACCCAGCAGGAGGTGGAGACCCAATT
CTTTATCAACACTTATTTTGATTTTTT
>LSTEM100-18|Sesamia_grisescens|am12446|
AACATTATATTTTATTTTCGGAATTTGAGCTGGTATAGTTGGAACCTCACTAAGATTATTAATTCGAGCCGAACTAGGAA
CCCCTGGTTCTTTAATTGGAGACGATCAAATTTATAATACTATTGTTACAGCTCATGCTTTTATTATAATTTTCTTTATA
GTTATACCAATTATAATTGGCGGATTTGGAAATTGACTCGTACCTTTAATATTAGGAGCCCCTGATATAGCATTCCCACG
AATAAATAATATAAGATTTTGATTATTACCCCCCTCTTTAACTTTACTAATTTCAAGTAGAATTGTAGAAAATGGGGCAG
GAACTGGATGAACAGTGTACCCCCCACTTTCATCTAATATTGCCCATGGAGGAAGATCAGTGGACTTAGCTATTTTTTCC
CTTCATTTAGCAGGTATTTCATCTATTTTAGGGGCTATTAATTTTATCACAACAATTATCAATATACGATTAAATAGCTT
ATCCTTTGATCAAATACCTTTATTTATTTGAGCTGTTGGAATTACTGCATTTTTATTATTATTATCTTTACCTGTTTTAG
CAGGAGCTATTACAATATTATTAACCGATCGAAACTTAAATACATCATTTTTCGACCCAGCAGGAGGAGGTGATCCTATT
TTATACCAACATTTATTTTGATTTTTT
>LSTEM101-18|Scirpophaga_excerptalis|am12447|
GACATTATATTTTATTTTTGGAATTTGAGCTGGTATAGTGGGGACTTCACTTAGTTTACTAATTCGAGCCGAACTAGGTA
CTCCTGGATCACTAATCGGAGATGATCAAATCTATAATACTATTGTAACTGCTCACGCTTTTATTATAATTTTTTTTATG
GTTATACCTATTATAATTGGAGGATTCGGTAACTGATTAGTACCTTTAATATTGGGAGCCCCAGATATAGCCTTTCCCCG
AATAAATAATATAAGTTTTTGATTATTACCCCCTTCTTTAACTCTCTTAATCTCAAGAAGAGTCGTTGAAAATGGAGCTG
GAACAGGATGAACTGTTTACCCGCCCTTATCCTCCAATATTGCTCATGGTGGGACTTCTGTAGATTTAGCCATTTTTTCA
TTACATTTAGCTGGAATTTCTTCTATTCTAGGGGCTATTAACTTCATTACAACTATTATTAATATACGAATTAATGGACT
ATCTTTTGATCAAATACCTTTATTCGTATGAGCAGTTGGTATCACTGCCCTTCTTCTTCTTCTCTCACTACCTGTATTAG
CGGGAGCTATTACTATATTACTAACAGATCGAAACTTAAATACCTCTTTCTTTGACCCAGCAGGAGGTGGAGACCCAATT
CTTTATCAACACTTATTTTGATTTTTT
>LSTEM102-18|Sesamia_grisescens|am12449|
AACATTATATTTTATTTTCGGAATTTGAGCTGGTATAGTTGGAACCTCACTAAGATTATTAATTCGAGCCGAACTAGGAA
CCCCTGGTTCTTTAATTGGAGACGATCAAATTTATAATACTATTGTTACAGCTCATGCTTTTATTATAATTTTCTTTATA
GTTATACCAATTATAATTGGCGGATTTGGAAATTGACTCGTACCTTTAATATTAGGAGCCCCTGATATAGCATTCCCACG
AATAAATAATATAAGATTTTGATTATTACCCCCCTCTTTAACTTTACTAATTTCAAGTAGAATTGTAGAAAATGGGGCAG
GAACTGGATGAACAGTGTACCCCCCACTTTCATCTAATATTGCCCATGGAGGAAGATCAGTGGACTTAGCTATTTTTTCC
CTTCATTTAGCAGGTATTTCATCTATTTTAGGGGCTATTAATTTTATCACAACAATTATCAATATACGATTAAATAGCTT
ATCCTTTGATCAAATACCTTTATTTATTTGAGCTGTTGGAATTACTGCATTTTTATTATTATTATCTTTACCTGTTTTAG
CAGGAGCTATTACAATATTATTAACCGATCGAAACTTAAATACATCATTTTTCGACCCAGCAGGAGGAGGTGATCCTATT
TTATACCAACATTTATTTTGATTTTTT
>LSTEM103-18|Scirpophaga_excerptalis|am12463|
AACATTATATTTTATTTTTGGAATTTGAGCTGGTATAGTAGGAACTTCACTTAGTTTATTAATTCGAGCTGAATTAGGTA
CCCCTGGGTCACTAATTGGAGATGATCAAATCTATAATACTATCGTAACTGCTCATGCTTTTATTATAATTTTTTTTATA
GTTATACCTATTATAATTGGGGGATTTGGAAACTGATTAGTACCTTTAATGTTAGGAGCTCCAGATATAGCTTTTCCTCG
AATAAATAATATAAGTTTTTGATTATTACCTCCTTCTTTAACCCTCTTAATCTCGAGAAGAATTGTTGAAAATGGAGCTG
GAACAGGATGAACTGTATACCCCCCTCTATCCTCTAATATTGCTCATGGTGGAACTTCTGTAGATCTAGCCATTTTTTCA
TTACATTTAGCTGGAATTTCCTCTATTCTAGGAGCTATTAACTTTATCACAACTATTATTAATATACGAATTAATGGATT
ATCTTTTGATCAAATACCTTTATTTGTATGAGCAGTTGGTATTACTGCTCTTCTCCTTCTTCTCTCACTACCTGTATTAG
CGGGAGCTATTACTATATTATTAACAGATCGAAACTTAAATACTTCTTTCTTTGACCCAGCAGGAGGTGGAGATCCAATC
CTTTATCAACATTTATTTTGATTTTTT
>LSTEM104-18|Tetramoera_sp.|am12465|
AACATTATATTTTATTTTTGGAATTTGAGCCGGAATAATTGGAACATCTCTAAGATTATTAATTCGAGCAGAATTAGGAA
ATCCTGGCTCTTTAATTGGAGATGATCAAATTTATAATACTATTGTAACTGCTCATGCTTTTATTATAATTTTTTTCATA
GTTATACCTATCATAATTGGAGGATTTGGAAATTGATTAGTACCATTAATATTAGGAGCCCCTGATATAGCTTTTCCTCG
TATAAATAATATAAGATTTTGATTACTCCCTCCTTCTATTATATTATTAATTTCAAGAAGAATTGTAGAAAATGGAGCAG
GAACAGGATGAACAGTTTATCCCCCCCTTTCATCTAATATTGCCCATAGAGGTAGATCAGTAGATCTAGCTATTTTTTCT

TTACATTTAGCTGGAATTTCTTCTATTTTAGGAGCTGTAAACTTTATTACAACTATTATTAATATACGACCAAATAATAT
AAGATTAGATCAAATACCCCTATTTGTTTGAGCTGTTGGCATTACAGCTCTTCTTTTATTATTATCTTTACCAGTATTAG
CAGGAGCTATTACTATACTCTTAACAGACCGTAATTTAAATACTTCATTTTTTGATCCTGCTGGTGGAGGAGATCCAATT
TTATACCA-------------------
>LSTEM105-18|Tetramoera_sp.|am12466|
AACATTATATTTTATTTTTGGAATTTGAGCCGGAATAATTGGWRCATCTCTAAGATTATTAATTCGAGCAGAATTAGGAA
ATCCTGGCTCTTTAATTGGAGATGATCAAATTTATAATACTATTGTAACTGCTCATGCTTTTATTATAATTTTTTTCATA
GTTATACCTATCATAATTGGAGGATTTGGAAATTGATTAGTACCATTAATATTAGGAGCCCCTGATATAGCTTTTCCTCG
TATAAATAATATAAGATTTTGATTACTCCCTCCTTCTATTATATTATTAATTTCAAGAAGAATTGTAGAAAATGGAGCAG
GAACAGGATGAACAGTTTATCCCCCCCTTTCATCTAATATTGCCCATAGAGGTAGATCAGTAGATCTAGCTATTTTTTCT
TTACATTTAGCTGGAATTTCTTCTATTTTAGGAGCTGTAAACTTTATTACAACTATTATTAATATACGACCAAATAATAT
AAGATTAGATCAAATACCCCTATTTGTTTGAGCTGTTGGCATTACAGCTCTTCTTTTATTATTATCTTTACCAGTATTAG
CAGGAGCTATTACTATACTCTTAACAGACCGTAATTTAAATACTTCATTTTTTGATCCTGCTGGTGGAGGAGATCCAATT
YYATACCAACACTTATTTTGATTTTTT
>LSTEM106-18|Chilo_sacchariphagus|am12468|
ARCTTTATATTTTATTTTGGAAATTTGAGCTGGAATAGTTGGAACATCCCTTAGACTTTTAATTCGAGCTGAATTAGGAA
ATCCAGGTTCATTAATCGGAGATGATCAAATTTATAATACTATTGTTACAGCCCATGCATTTATTATAATTTTTTTTATA
GTAATACCAATTATAATTGGAGGATTTGGAAATTGATTAGTTCCATTAATATTAGGGGCTCCTGATATAGCCTTCCCTCG
TCTAAATAATATAAGATTTTGATTATTACCCCCTTCTTTAACCCTTCTAATTTCTAGAAGAATCGTTGAAAATGGAGCAG
GAACTGGATGAACAGTCTACCCCCCCCTATCTTCCAATATTTCACATGCTGGAAGTTCAGTAGATTTAGCCATCTTCTCC
CTTCATTTAGCTGGAATTTCTTCAATTTTAGGAGCTATCAATTTCATTACTACAATTATTAATATACGAATTAATGGATT
ATTATTTGATCAAATACCATTATTTGTTTGATCTGTTGGTATTACAGCATTACTTCTCCTCCTTTCTTTACCAGTATTAG
CAGGNGCTATTACTATACTATTAACTGACCGAAATTTAAATACATCTTTTTTTGACCCAGCTGGAGGAGGNGATCCAANT
TTATATCAACATTTATTTTGATTTTTT
>LSTEM107-18|Chilo_sacchariphagus|am12469|
--CTTTATATTTATTTATTGGAATNTGAGCTGGAATAATTGGAACATCCCTTAGACTTTTAATTCGAGCTGAATTAGGAA
ATCCAGGTTCATTAATTGGAGATGATCAAATTTATAATACTATTGTTACAGCTCATGCATTTATTATAATTTTTTTTATA
GTAATACCAATTATAATCGGAGGATTTGGAAATTGATTAGTTCCATTAATATTAGGAGCTCCTGATATAGCCTTTCCTCG
TTTAAATAATATAAGATTTTGATTATTACCCCCTTCTTTAACTCTTTTAATTTCTAGAAGAATTGTTGAAAATGGAGCAG
GAACTGGATGAACAGTCTACCCCCCCCTATCTTCCAATATTTCACATGCTGGAAGTTCAGTAGATTTAGCCATCTTTTCT
CTTCATTTAGCAGGAATTTCTTCAATTTTAGGAGCTATTAATTTTATTACTACAATTATTAATATACGAATTAATGGATT
ATTATTTGATCAAATACCATTA--

>LSTEM108-18|Chilo_sacchariphagus|am12470|
AACTTTATATTTTATTTTTGGAATTTGAGCTGGAATAATTGGAACATCCCTTAGACTTTTAATTCGAGCTGAATTAGGAA
ATCCAGGTTCATTAATTGGAGATGATCAAATTTATAATACTATTGTTACAGCTCATGCATTTATTATAATTTTTTTTATA
GTAATACCAATTATAATCGGAGGATTTGGAAATTGATTAGTTCCATTAATATTAGGAGCTCCTGATATAGCCTTTCCTCG
TTTAAATAATATAAGATTTTGATTATTACCCCCTTCTTTAACTCTTTTAATTTCTAGAAGAATTGTTGAAAATGGAGCAG
GAACTGGATGAACAGTCTACCCCCCCCTATCTTCCAATATTTCACATGCTGGAAGTTCAGTAGATTTAGCCATCTTTTCT
CTTCATTTAGCAGGAATTTCTTCAATTTTAGGAGCTATTAATTTTATTACTACAATTATTAATATACGAATTAATGGATT
ATTATTTGATCAAATACCATTATTTGTTTGATCTGTTGGTATTACAGCATTACTTCTTCTTCTCTCTTTACCAGTATTAG
CAGGTGCTATTACCATACTATTAACTGACCGAAATTTAAATACATCTTTTTTTGATCCAGCTGGAGGAGGTGATCCAATT
TTATATCAACATTTATTTTGATTTTTT
>LSTEM109-18|Chilo_sacchariphagus|am12471|
AACTTTATATTTTATTTTTGGAATTTGAGCTGGAATAATTGGAACATCCCTTAGACTTTTAATTCGAGCTGAATTAGGAA
ATCCAGGTTCATTAATTGGAGATGATCAAATTTATAATACTATTGTTACAGCTCATGCATTTATTATAATTTTTTTTATA
GTAATACCAATTATAATCGGAGGATTTGGAAATTGATTAGTTCCATTAATATTAGGAGCTCCTGATATAGCCTTTCCTCG
TTTAAATAATATAAGATTTTGATTATTACCCCCTTCTTTAACTCTTTTAATTTCTAGAAGAATTGTTGAAAATGGAGCAG
GAACTGGATGAACAGTCTACCCCCCCCTATCTTCCAATATTTCACATGCTGGAAGTTCAGTAGATTTAGCCATCTTTTCT
CTTCATTTAGCAGGAATTTCTTCAATTTTAGGAGCTATTAATTTTATTACTACAATTATTAATATACGAATTAATGGATT
ATTATTTGATCAAATACCATTATTTGTTTGA---

>LSTEM110-18|Chilo_sacchariphagus|am12472|
AACTTTATATYTATTCNTTGGAATTNGAGCTGGAATAATTGGAACATCCCTTAGACTTTTAATTCGAGCTGAATTAGGAA
ATCCAGGTTCATTAATTGGAGATGATCAAATTTATAATNCTATTGTTACAGCTCATGCATTTATTATAATTTTTTTTATA

GTAATACCAATTATAATCGGAGGATTTGGAAATTGATTAGKTCCATTAATATTAGGAGCTCCTGATATAGCCTTTCCTCG
TTTAAATAATATAAGATTTTGATTATTGCCCCCTTCTTTAACTCTTTTAATTTCTAGAAGAATTGTTGAAAATGGAGCAG
GAACTGGATGAACAGTCTACCCCCCCCTATCTTCCAATATTTCACATGCTGGAAGTTCAGTAGATTTAGCCATCTTTTCT
CTTCATTTAGCAGGAATTTCTTCAATTTTAGGAGCTATTAATTTTATTACTACAATTATTAATATACGAATTAATGGATT
ATTATTTGATCAAATACCATTATTTGNNNGATCTGNNGGTATTACAGCATTACTTCTTCTTCTCTCTTTACCAGNATTAG
CAGGRGCTATTACCATACTATTAACTGACCGAAATTTAAATACATCTTTTTTGATCCNNMTHTANNNGGNGATCCAATT
TTATATCAACATTTATTTTGATTTTTT
>LSTEM111-18|Chilo_sacchariphagus|am12473|
AACTTTATATTTTATTTTTGGAATTTGAGCTGGAATAATTGGAACATCCCTTAGACTTTTAATTCGAGCTGAATTAGGAA
ATCCAGGTTCATTAATTGGAGATGATCAAATTTATAATACTATTGTTACAGCTCATGCATTTATTATAATTTTTTTTATA
GTAATACCAATTATAATCGGAGGATTTGGAAATTGATTAATTCCATTAATATTAGGAGCTCCTGATATAGCCTTTCCTCG
TTTAAATAATATAAGATTTTGATTATTACCCCCTTCTTTAACTCTTTTAATTTCTAGAAGAATTGTTGAAAATGGAGCAG
GAACTGGATGAACAGTCTACCCCCCCCTATCTTCCAATATTTCACATGCTGGAAGTTCAGTAGATTTAGCCATCTTTTCT
CTTCATTTAGCAGGAATTTCTTCAATTTTAGGAGCTATTAATTTTATTACTACAATTATTAATATACGAATTAATGGATT
ATTATTCGATCAAATACCATTATTTGNTTGATCTGNNGGTATTACAGCATTACTTCTTCTTCTCTCTTTACCAGTATTAG
CWGKRGCTATTACCATACTATTAACTGACCGAAATTTAAATACATCTTTTTTGATCCAGCTGGAGGAGGNGATCCAATT
TTATATCAACATTNATTTTGATTTTTT
>LSTEM112-18|Chilo_sacchariphagus|am12474|

---ATTGTTRCAGCCCATGCATTTATTATRATTTTTTTTATA
GTAATACCAATTATAATTGGAGGATTTGGAAATTGATTAGTTCCATTAATATTAGGGGCTCCTGATATAGCCTTCCCTCG
TCTAAATAATATAAGATTTTGATTATTACCCCCTTCTTTAACCCTTCTAATTTCTAGAAGAATCGTTGAAAATGGAGCAG
GAACTGGATGAACAGTCTACCCCCCCCTATCTTCCAATATTTCACATGCTGGAAGTTCAGTAGATTTAGCCATCTTCTCC
CTTCATTTAGCTGGAATTTCTTCAATTTTAGGAGCTATCAATTTCATTACTACAATTATTAATATACGAATTAATGGATT
ATTATTTGATCAAATACCATTATTTGTTTGATCTGTTGGTATTACAGCATTACTTCTCCTCCTTTCTTTACCAGTATTAG
CAGGTGCTATTACTATACTATTAACTGAYCGAAATTTAAATACATCTTTTTTGACCCAGCTGRAGGAGGNGATCCAATT
TTATATCAACATTWATTTKGATTTTTT
>LSTEM113-18|Tetramoera_sp.|am12475|
AACATTATATTTTATTTTTGGAATTTGAGCCGGAATAATTGGAACATCTCTAAGATTATTAATTCGAGCAGAATTAGGAA
ATCCTGGCTCTTTAATTGGAGATGATCAAATTTATAATACTATTGTAACTGCTCATGCTTTTATTATAATTTTTTTCATA
GTTATACCTATCATAATTGGAGGATTTGGAAATTGATTAGTACCATTAATATTAGGAGCCCCTGATATAGCTTTTCCTCG
TATAAATAATATAAGATTTTGATTACTCCCTCCTTCTATTATATTATTAATTTCAAGAAGAATTGTAGAAAATGGAGCAG
GAACAGGATGAACAGTTTATCCCCCCCTTTCATCTAATATTGCCCATAGAGGTAGATCAGTAGATCTAGCTATTTTTTCT
TTACATTTAGCTGGAATTTCTTCTATTTTAGGAGCTGTAAACTTTATTACAACTATTATTAATATACGACCAAATAATAT
AAGATTAGATCAAATACCCCTATTTGTTTGAGCTGTTGGCATTACAGCTCTTCTTTTATTATTATCTTTACCAGTATTAG
CAGGAGCTATTACTATACTCTTAACAGACCGTAATTTAAATACTTCATTTTTTGATCCTGCTGGTGGAGGAGATCCAATT
TTTTACCAACACTTATTTTGATTTTTT
>LSTEM114-18|Chilo_infuscatellus|am12476|
AACTTTATATTTTATTTTTGGAATTTGGGCGGGAATAATTGGGACTTCTCTTAGACTTCTAATTCGAGCTGAATTAGGAA
CTCCAGGATCTTTAATTGGAGATGATCAAATTTATAATACTATTGTTACAGCCCATGCATTTATTATAATTTTTTTTATA
GTAATACCAATTATAATTGGAGGATTTGGAAATTGATTAGTTCCTTTAATATTAGGAGCACCTGATATAGCTTTCCCACG
AATAAATAATATAAGTTTTTGATTATTACCACCATCATTAACATTATTGATTTCTAGAAGAATTGTTGAAAATGGAGCAG
GAACTGGTTGAACTGTTTATCCCCCCTTATCTTCAAATATTGCCCATGGGGGTAGTTCTGTAGATTTAGCAATTTTTTCC
CTTCATTTGGCGGGTATTTCATCAATTTTAGGGGCTATTAATTTTATTACAACAATTATTAATATACGAGTTAATGGTTT
ATCATTTGATCAAATACCTCTATTTGTTTGATCCGTAGGTATTACAGCACTATTATTATTACTTTCTTTACCAGTATTAG
CAGGTGCTATTACTATATTACTAACTGATCGGAATTTAAATACATCTTTTTTTGATCCTGCTGGAKGTGTAGATCGAATT
CTCTATCAACATTTATTTTGATTTTTT
>LSTEM115-18|Chilo_sacchariphagus|am12477|
-------------TTTGGGAATTTTGAGCTGGAATAATTGGAACATCCCTTAGACTTTTAATTCGAGCTGAATTAGGAA
ATCCAGGTTCATTAATTGGAGATGATCAAATTTATAATACTATTGTTACAGCTCATGCATTTATTATAATTTTTTTTATA
GTAATACCAATTATAATCGGAGGATTTGGAAATTGATTAGTTCCATTAATATTAGGAGCTCCTGATATAGCCTTTCCTCG
TTTAAATAATATAAGATTTTGATTATTGCCCCCTTCTTTAACTCTTTTAATTTCTAGAAGAATTGTTGAAAATGGAGCAG
GAACTGGATGAACAGTCTACCCCCCCCTATCTTCCAATATTTCACATGCTGGAAGTTCAGTAGATTTAGCCATCTTTTCT
CTTCATTTAGCAGGAATTTCTTCAATTTTAGGAGCTATTAATTTTATTACTACAATTATTAATATACGAATTAATGGATT
ATTATTTGATCAAATACCATTATTTGTTTGATCTGTTGGTATTACAGCATTACTTCTTCTTCTCTCTTTACCAGTATTAG
CAGGTGCTATTACCATACTATYAACTGACCGAAATTTAAATACATCTTTTTTGATCCAGCTGGAGGAGGTGATCCAATT
TTATATCAACATCTAGTGATGATTCTT

>LSTEM116-18|Chilo_sacchariphagus|am12478|
AACTTTATATTTTATTTTTGGAATTTGAGCTGGAATAATTGGAACATCCCTTAGACTTTTAATTCGAGCTGAATTAGGAA
ATCCAGGTTCATTAATTGGAGATGATCAAATTTATAATACTATTGTTACAGCTCATGCATTTATTATAATTTTTTTTATA
GTAATACCAATTATAATCGGAGGATTTGGAAATTGATTAGTTCCATTAATATTAGGAGCTCCTGATATAGCCTTTCCTCG
TTTAAATAATATAAGATTTTGATTATTACCTCCTTCTTTAACTCTTTTAATTTCTAGAAGAATTGTTGAAAATGGAGCAG
GAACTGGATGAACAGTCTACCCCCCCCTATCTTCCAATATTTCACATGCTGGAAGTTCAGTAGATTTAGCCATCTTTTCT
CTTCATTTAGCAGGAATTTCTTCAATTTTAGGAGCTATTAATTTTATTACTACAATTATTAATATACGAATTAATGGATT
ATTATTTGATCAAATACCATTATTTGTTTGATCTGTTGGTATTACAGCATTACTTCTTCTTCTCTCTTTACCAGTATTAG
CAGGTGCTATTACCATACTATTAACTGACCGAAATTTAAATACATCTTTTTTTGATCCAGCTGGAGGAGGTGATCCAATT
TTATATCAACATTTATTTTGATTTTTT
>LSTEM117-18|Tetramoera_sp.|am12479|
AACATTATATTTTATTTTTGGAATTTGAGCCGGAATAATTGGAACATCTCTAAGATTATTAATTCGAGCAGAATTAGGAA
ATCCTGGCTCTTTAATTGGAGATGATCAAATTTATAATACTATTGTAACTGCTCATGCTTTTATTATAATTTTTTTCATA
GTTATACCTATCATAATTGGAGGATTTGGAAATTGATTAGTACCATTAATATTAGGAGCCCCTGATATAGCTTTTCCTCG
TATAAATAATATAAGATTTTGATTACTCCCTCCTTCTATTATATTATTAATTTCAAGAAGAATTGTAGAAAATGGAGCAG
GAACAGGATGAACAGTTTATCCCCCCCTTTCATCTAATATTGCCCATAGAGGTAGATCAGTAGATCTAGCTATTTTTTCT
TTACATTTAGCTGGAATTTCTTCTATTTTAGGAGCTGTAAACTTTATTACAACTATTATTAATATACGACCAAATAATAT
AAGATTAGATCAAATACCCCTATTTGTTTGAGCTGTTGGCATTACAGCTCTTCTTTTATTATTATCTTTACCAGTATTAG
CAGGAGCTATTACTATACTCTTAACAGACCGTAATTTAAATACTTCATTTTTTGATCCTGCTGGTG-------------
----
>LSTEM118-18|Chilo_auricilius|am12480|
AACTTTATACTTTATTTTTGGAATTTGAAGTGGAATAATTGGAACATCTCTAAGACTTTTAATTCGTGCTGAATTAGGAA
CTCCAGGGTCATTAATTGGAGATGATCAAATTTACAATACTATTGTTACAGCTCATGCATTTATTATAATTTTTTTTATA
GTTATACCAATTATAATTGGAGGCTTTGGTAATTGATTAGTACCATTAATACTAGGGGCTCCTGATATAGCCTTCCCTCG
AATAAATAATATAAGATTTTGATTATTGCCCCCATCATTAACATTATTAATTTCTAGAAGAATTGTAGAAAATGGAGCTG
GAACAGGATGAACGGTTTACCCCCCCCTTTCATCAAATATTGCCCATGGAGGAAGTTCTGTAGATTTAGCTATTTTTTCT
CTTCATTTAGCTGGTATTTCCTCAATTTTAGGAGCTATTAATTTTATTACAACAATTATTAATATACGAATTAATAAACT
ATCATTTGATCAATTACCATTATTTGTTTGATCTGTTGGTATTACAGCTTTATTATTACTACTTTTWTTGNAGGTACTAG
CTGGAGCTATTACTATACTTTTAACTGATCGAAACTTAAATACATCTTTTTTTGACCCTGCTGGAGGA-----------
----
>LSTEM119-18|Chilo_sacchariphagus|am12481|
AACTTTATATTTTATTTTTGGAATTTGAGCTGGAATAATTGGAACATCCCTTAGACTTTTAATTCGAGCTGAATTAGGAA
ATCCAGGTTCATTAATTGGAGATGATCAAATTTATAATACTATTGTTACAGCTCATGCATTTATTATAATTTTTTTTATA
GTAATACCAATTATAATCGGAGGATTTGGAAATTGATTAGTTCCATTAATATTAGGAGCTCCTGATATAGCCTTTCCTCG
TTTAAATAATATAAGATTTTGATTATTACCCCCTTCTTTAACTCTTTTAATTTCTAGAAGAATTGTTGAAAATGGAGCAG
GAACTGGATGAACAGTCTACCCCCCCCTATCTTCCAATATTTCACATGCTGGAAGTTCAGTAGATTTAGCCATCTTTTCT
CTTCATTTAGCAGGAATTTCTTCAATTTTAGGAGCTATTAATTTTATTACTACAATTATTAATATACGAATTAATGGATT
ATTATTTGATCAAATACCATTATTTGTTTGATCTGTTGGTATTACAGCATTACTTCTTCTTCTCTCTTTACCAGTATTAG
CAGGTGCTATTACCATACTATTAACTGACCGAAATTTAAATACATCTTTTTTTGATCCAGCTGKAGGAGKRAATCSAMKT
YYATATCAACATTTATTTTGATTTTTT
>LSTEM120-18|Chilo_sacchariphagus|am12482|
AACTTTATATTTATNTTTTGGAATTTGAGCTGGAATAATTGGAACATCCCTTAGACTTTTAATTCGAGCTGAATTAGGAA
ATCCAGGTTCATTAATTGGAGATGATCAAATTTATAATACTATTGTTACAGCTCATGCATTTATTATAATTTTTTTTATA
GTAATACCAATTATAATCGGAGGATTTGGAAATTGATTAGTTCCATTAATATTAGGAGCTCCTGATATAGCCTTTCCTCG
TTTAAATAATATAAGATTTTGATTATTACCCCCTTCTTTAACTCTTTTAATTTCTAGAAGAATTGTTGAAAATGGAGCAG
GAACTGGATGAACAGTCTACCCCCCCCTATCTTCCAATATTTCACATGCTGGAAGTTCAGTAGATTTAGCCATCTTTTCT
CTTCATTTAGCAGGAATTTCTTCAATTTTAGGAGCTATTAATTTTATTACTACAATTATTMATATMCGAATTAATGGATT
ATTATTTGATCAAATACCATT---

>LSTEM121-18|Chilo_sacchariphagus|am12484|
AACTTTATATTTTATTTTTGGAATTTGAGCTGGAATAATTGGAACATCCCTTAGACTTTTAATTCGAGCTGAATTAGGAA
ATCCAGGTTCATTAATTGGAGATGATCAAATTTATAATACTATTGTTACAGCTCATGCATTTATTATAATTTTTTTTATA
GTAATACCAATTATAATCGGAGGATTTGGAAATTGATTAGTTCCATTAATATTAGGAGCTCCTGATATAGCCTTTCCTCG
TTTAAATAATATAAGATTTTGATTATTACCCCCTTCTTTAACTCTTTTAATTTCTAGAAGAATTGTTGAAAATGGAGCAG
GAACTGGATGAACAGTCTACCCCCCCCTATCTTCCAATATTTCACATGCTGGAAGTTCAGTAGATTTAGCCATCTTTTCT
CTTCATTTAGCAGGAATTTCTTCAATTTTAGGAGCTATTAATTTTATTACTACAATTATTAATATACGAATTAATGGATT

ATTATTCGATCAAATACCATTATTTGTTTGATCTGTTGGTATTACAGCATTACTTCTTCTTCTCTCTTTACCAGTATTAG
CAGGTGCTATTACCATACTATTAACTGACCGAAATTTAAATACATCTTTTTTTGATCCAGCTGGAGGAGGTGATCCAATT
TTATATCAACATTTATTTTGATTTTTT
>LSTEM122-18|Chilo_auricilius|am12485|
AACTTTATACTTTATTTTTGGAATTTGAAGTGGAATAATTGGAACATCTCTAAGACTTTTAATTCGTGCTGAATTAGGAA
CTCCAGGGTCATTAATTGGAGATGATCAAATTTACAATACTATTGTTACAGCTCATGCATTTATTATAATTTTTTTTATA
GTTATACCAATTATAATTGGAGGCTTTGGTAATTGATTAGTACCATTAATACTAGGGGCTCCTGATATAGCCTTCCCTCG
AATAAATAATATAAGATTTTGATTATTGCCCCCATCATTAACATTATTAATTTCTAGAAGAATTGTAGAAAATGGAGCTG
GAACAGGATGAACGGTTTACCCCCCCCTTTCATCAAATATTGCCCATGGAGGAAGTTCTGTAGATTTAGCTATTTTTTCT
CTTCATTTAGCTGGTATTTCCTCAATTTTAGGAGCTATTAATTTTATTACAACAATTATTAATATACGAATTAATAAACT
ATCATTTGATCAATTACCATTATTTGTTTGATCTGTTGGTATTACAGCTTTATTATTACTACTTTCATTGCCGGTACTAG
CTGGAGCTATTACTATACTTTTAACTGATCGAAACTTAAATACATCTTTTTTGACCCTGCTGGAGGAGGAGACCCAATT
CTTTATCAACATTTATTTTGATTTTTT
>LSTEM123-18|Chilo_sacchariphagus|am12486|
AACTTTATATTTTATTTTTGGAATTTGAGCTGGAATAATTGGAACATCCCTTAGACTTTTAATTCGAGCTGAATTAGGAA
ATCCAGGTTCATTAATTGGAGATGATCAAATTTATAATACTATTGTTACAGCTCATGCATTTATTATAATTTTTTTTATA
GTAATACCAATTATAATCGGAGGATTTGGAAATTGATTAGTTCCATTAATATTAGGAGCTCCTGATATAGCCTTTCCTCG
TTTAAATAATATAAGATTTTGATTATTGCCCCCTTCTTTAACTCTTTTAATTTCTAGAAGAATTGTTGAAAATGGAGCAG
GAACTGGATGAACAGTCTACCCCCCCCTATCTTCCAATATTTCACATGCTGGAAGTTCAGTAGATTTAGCCATCTTTTCT
CTTCATTTAGCAGGAATTTCTTCAATTTTAGGAGCTATTAATTTTATTACTACAATTATTAATATACGAATTAATGGATT
ATTATTTGATCAAATACCATTATTTGTTTGATCTGTTGGTATTACAGCATTACTTCTTCTTCTCTCTTTACCAGTATTAN
CAGGNGCTATTACCATACTATTAACTGACCGAAATTTAAATACATCTTTTTTTGATCCAGCTGGAGGAGGTGATCCAATT
TTATATCAACATTTATTTTGATTTTTT
>LSTEM124-18|Tetramoera_sp.|am12487|
AACATTATATTTTATTTTTGGAATTTGAGCCGGAATAATTGGAACATCTCTAAGATTATTAATTCGAGCAGAATTAGGAA
ATCCTGGCTCTTTAATTGGAGATGATCAAATTTATAATACTATTGTAACTGCTCATGCTTTTATTATAATTTTTTTCATA
GTTATACCTATCATAATTGGAGGATTTGGAAATTGATTAGTACCATTAATATTAGGAGCCCCTGATATAGCTTTTCCTCG
TATAAATAATATAAGATTTTGATTACTCCCTCCTTCTATTATATTATTAATTTCAAGAAGAATTGTAGAAAATGGAGCAG
GAACAGGATGAACAGTTTATCCCCCCCTTTCATCTAATATTGCCCATAGAGGTAGATCAGTAGATCTAGCTATTTTTTCT
TTACATTTAGCTGGAATTTCTTCTATTTTAGGAGCTGTAAACTTTATTACAACTATTATTAATATACGACCAAATAATAT
AAGATTAGATCAAATACCCCTATTTGTTTGAGCTGTTGGCATTACAGCTCTTCTTTTATTATTATCTTTACCAGTATTAG
CAGGAGCTATTACTATACTCTTAACAGACCGNAATTTAAATACTTCATTTTTTGATCCTGCTGGTGGAGGAGATCCAATT
TTATACCAACACTTATTTTGATTTTTT
>LSTEM125-18|Chilo_auricilius|am12489|
AACTTTATACTTTATTTTTGGAATTTGGAGTGGAATAATTGGAACATCTCTAAGACTTTTAATTCGTGCTGAATTAGGAA
CTCCAGGGTCATTAATTGGAGATGATCAAATTTACAATACTATTGTTACAGCTCATGCATTTATTATAATTTTTTTTATA
GTTATACCAATTATAATCGGAGGCTTTGGTAATTGATTAGTACCATTAATACTAGGGGCTCCTGATATAGCCTTCCCTCG
AATAAATAATATAAGATTTTGATTATTGCCCCCATCATTAACATTATTAATTTCTAGAAGAATTGTAGAAAATGGAGCTG
GAACAGGATGAACGGTTTACCCCCCCCTTTCATCAAATATTGCCCATGGAGGAAGTTCTGTAGATTTAGCTATTTTTTCT
CTTCATTTAGCTGGTATTTCCTCAATTTTAGGAGCTATTAATTTTATTACAACAATTATTAATATACGAATTAATAAACT
ATCATTTGATCAATTACCATTATTTGTTTGATCTGTTGGTATTACAGCTTTATTATTACTACTTTCATTGCCGGTACTAG
CTGGAGCTATTACTATACTTTTAACTGATCGAAACTTAAATACATCTTTTTTGACCCTGCTGGAGGAGGAGACCCAATT
CTTTATCAGCATTTATTTTGATTTTTT
>LSTEM126-18|Chilo_sacchariphagus|am12491|

--TTGTTACAGCTCATGCATTTATTATRAATTTTTTTATA
GTARTACCAATTATAATCGGAGGATTTGGAAATTGATTARTTCCWTTAATATTAGGAGCTCCTGATATAGCCTTTCCTCG
WWTAAATAATATAAGATTTTGATTATTGCCCCCTTCYTTAACTCTTTTAATTTYTAGAATAATTDTTGAARATGGAGCAG
GAACTGGATGAACAGTNTACCCCCCCCTATCTTCCAATATTTCACATGCTGGAAGTTCAGTAGATTTAGCCATCTTTTCT
CTTCATTTAGCAGGAATTTCTTCAATTTTAGGAGCTATTAATTTTATTACTACAATTATTAATATACGAATTAATGGATT
ATTATTTGATCAAATACCATTATTTGTTTGATCTGTTGGTATTACAGCATTACTTCTTCTTCTCTCTTTACCAGKATTAG
CAGGTGCTATTACCATACTATTAACTGACCGAAATTTAAATACATCTTTTTTTGATCCAGCTGGAGGAGGTGATCCAATT
TTATATCAACATTTATTTTGATTTTTT
>LSTEM127-18|Chilo_sacchariphagus|am12492|
AACTTTATATTTTATTTTTGGAATTTGAGCTGGAATAATTGGAACATCCCTTAGACTTTTAATTCGAGCTGAATTAGGAA
ATCCAGGTTCATTAATTGGAGATGATCAAATTTATAATACTATTGTTACAGCTCATGCATTTATTATAATTTTTTTTATA
GTAATACCAATTATAATCGGAGGATTTGGAAATTGATTAGTTCCATTAATATTAGGAGCTCCTGATATAGCCTTTCCTCG

TTTAAATAATATAAGATTTTGATTATTACCCCCTTCTTTAACTCTTTTAATTTCTAGAAGAATTGTTGAAAATGGAGCAG
GAACTGGATGAACAGTCTACCCCCCCCTATCTTCCAATATTTCACATGCTGGAAGTTCAGTAGATTTAGCCATCTTTTCT
CTTCATTTAGCAGGAATTTCTTCAATTTTAGGAGCTATTAATTTTATTACTACAATTATTAATATACGAATTAATGGATT
ATTATTTGATCAAATACCATTATTTGKTTGATCTGTTGGTATTACAGCATTACTTCTTCTTCTCTCTTTACCAGTATTAG
CAGGKGCTATTACCATACTATTAACTGACCGAAATTTAAATACATCTTTTTTTGATCC----------------------
----
>LSTEM128-18|Chilo_auricilius|am12493|
AACTTTATACTTTATTTTTGGAATTTGGAGTGGAATAATTGGAACATCTCTAAGACTTTTAATTCGTGCTGAATTAGGAA
CTCCAGGGTCATTAATTGGAGATGATCAAATTTACAATACTATTGTTACAGCTCATGCATTTATTATAATTTTTTTTATA
GTTATACCAATTATAATCGGAGGCTTTGGTAATTGATTAGTACCATTAATACTAGGGGCTCCTGATATAGCCTTCCCTCG
AATAAATAATATAAGATTTTGATTATTGCCCCCATCATTAACATTATTAATTTCTAGAAGAATTGTAGAAAATGGAGCTG
GAACAGGATGAACGGTTTACCCCCCCCTTTCATCAAATATTGCCCATGGAGGAAGTTCTGTAGATTTAGCTATTTTTTCT
CTTCATTTAGCTGGTATTTCCTCAATTTTAGGAGCTATTAATTTTATTACAACAATTATTAATATACGAATTAATAAACT
ATCATTTGATCAATTACCATTATTTGTTTGATCTGTTGGTATTACAGCTTTATTATTACTACTTTCATTGCCGGTACTAG
CTGGAGCTATTACTATACTTTTAACTGATCGAAACTTAAATACATCTTTTTTTGACCCTGCTGGAGGAGGAGACCCAATT
CTTTATCAGCATTTATTTTGATTTTTT
>LSTEM129-18|Scirpophaga_excerptalis|am12494|
AACATTATATTTTATTTTTGGAATTTGAGCTGGTATAGTAGGAACTTCACTTAGTTTATTAATTCGAGCTGAATTAGGTA
CCCCTGGGTCACTAATTGGAGATGATCAAATCTATAATACTATCGTAACTGCTCATGCTTTTATTATAATTTTTTTTATA
GTTATACCTATTATAATTGGGGGATTTGGAAACTGATTAGTACCTTTAATGTTAGGAGCTCCAGATATAGCTTTTCCTCG
AATAAATAATATAAGTTTTTGATTATTACCTCCTTCTTTAACCCTCTTAATCTCGAGAAGAATTGTTGAAAATGGAGCTG
GAACAGGATGAACTGTATACCCCCCTCTATCCTCTAATATTGCTCATGGTGGAACTTCTGTAGATCTAGCCATTTTTTCA
TTACATTTAGCTGGAATTTCCTCTATTCTAGGAGCTATTAACTTTATCACAACTATTATTAATATACGAATTAATGGATT
ATCTTTTGATCAAATACCTTTATTTGTATGAGCAGTTGGTATTACTGCTCTTCTCCTTCTTCTCTCACTACCTGTATTAG
CGGGAGCTATTACTATATTATTAACAGATCGAAACTTAAATACTTCTTTCTTTGACCCAGCAGGAGGTGGAGATCCAATC
CTTTATCAACATTTATTTTGATTTTTT
>LSTEM130-18|Scirpophaga_excerptalis|am12495|
AACATTATATTTTATTTTTGGAATTTGAGCTGGTATAGTAGGAACTTCACTTAGTTTATTAATTCGAGCTGAATTAGGTA
CTCCTGGATCACTAATTGGAGATGATCAAATCTATAATACTATCGTAACTGCTCATGCTTTTATTATAATTTTTTTTATA
GTTATACCTATTATAATTGGGGGATTTGGAAACTGATTAGTACCTTTAATGTTAGGAGCCCCAGATATAGCTTTCCCTCG
AATAAATAATATAAGTTTTTGATTATTACCCCCTTCTTTAACTCTCTTAATCTCGAGAAGAATTGTTGAAAATGGAGCTG
GAACAGGATGAACTGTATACCCTCCCCTATCCTCTAATATTGCTCACGGTGGAACTTCTGTAGATCTAGCCATTTTTTCA
TTACATTTAGCTGGAATTTCCTCTATTCTAGGAGCTATTAACTTCATCACAACTATTATTAATATACGAATTAATGGATT
ATCTTTTGATCAAATACCTTTATTTGTGTGAGCAGTTGGTATTACCGCTCTTCTCCTTCTTCTCTCACTACCTGTATTAG
CGGGAGCTATTACTATATTATTAACAGATCGAAACTTAAATACTTCTTTCTTTGATCCAGCAGGAGGTGGAGATCCAATC
CTTTATCAACATTTATTTTGATTTTTT
>LSTEM131-18|Scirpophaga_excerptalis|am12496|
AACATTATATTTTATTTTTGGAATTTGAGCTGGTATAGTAGGAACTTCACTTAGTTTATTAATTCGAGCTGAATTAGGTA
CTCCTGGRTCACTAATTGGAGATGATCAAATCTATAATACTATCGTAACTGCTCATGCTTTTATTATAATTTTTTTTATA
GTTATACCTATTATAATTGGAGGATTTGGAAACTGATTAGTACCTTTAATGTTAGGAGCCCCAGATATAGCTTTTCCTCG
AATAAATAATATAAGTTTTTGATTATTACCTCCTTCTTTAACCCTCTTAATCTCGAGAAGAATTGTTGAAAATGGAGCTG
GAACAGGATGAACTGTATACCCCCCYCTATCCTCTAATATTGCTCATGGTGGAACTTCTGTAGATCTAGCCATTTTTTCA
TTACATTTAGCTGGAATTTCCTCTATTCTAGGAGCTATTAACTTCATCACAACTATTATTAATATACGAATTAATGGATT
ATCTTTTGATCAAATACCTTTATTTGTATGAGCAGTTGGTATTACCGCCCTTCTCCTTCTTCTCTCACTACCTGTATTAG
CAGGAGCTATTACTATATTATTAACAGATCGAAACTTAAATACTTCTTTCTTTGACCCAGCAGGAGGTGGAGATCCAATT
CTTTATCAACATTTATTTTGATTTTTT
>LSTEM132-18|Scirpophaga_excerptalis|am12497|
AACATTATATTTTATTTTTGGAATTTGAGCTGGTATAGTAGGAACTTCACTTAGTTTATTAATTCGAGCTGAATTAGGTA
CCCCTGGGTCACTAATTGGAGATGATCAAATCTATAATACTATCGTAACTGCTCATGCTTTTATTATAATTTTTTTTATA
GTTATACCTATTATAATTGGGGGATTTGGAAACTGATTAGTACCTTTAATGTTAGGAGCTCCAGATATAGCTTTTCCTCG
AATAAATAATATAAGTTTTTGATTATTACCTCCTTCTTTAACCCTCTTAATCTCGAGAAGAATTGTTGAAAATGGAGCTG
GAACAGGATGAACTGTATACCCCCCTCTATCCTCTAATATTGCTCATGGTGGAACTTCTGTAGATCTAGCCATTTTTTCA
TTACATTTAGCTGGAATTTCCTCTATTCTAGGAGCTATTAACTTTATCACAACTATTATTAATATACGAATTAATGGATT
ATCTTTTGATCAAATACCTTTATTTGTATGAGCAGTTGGTATTACTGCTCTTCTCCTTCTTCTCTCACTACCTGTATTAG
CGGGAGCTATTACTATATTATTAACAGATCGAAACTTAAATACTTCTTTCTTTGACCCAGCAGGAGGTGGAGATCCAATC
CTTTATCAACATTTATTTTGATTTTTT
>LSTEM133-18|Scirpophaga_excerptalis|am12498|

AACATTATATTTTATTTTTGGAATTTGAGCTGGTATAGTAGGAACTTCACTTAGTTTATTAATTCGAGCTGAATTAGGTA
CCCCTGGGTCACTAATTGGAGATGATCAAATCTATAATACTATCGTAACTGCTCATGCTTTTATTATAATTTTTTTTATA
GTTATACCTATTATAATTGGGGGATTTGGAAACTGATTAGTACCTTTAATGTTAGGAGCTCCAGATATAGCTTTTCCTCG
AATAAATAATATAAGTTTTTGATTATTACCTCCTTCTTTAACCCTCTTAATCTCGAGAAGAATTGTTGAAAATGGAGCTG
GAACAGGATGAACTGTATACCCCCCTCTATCCTCTAATATTGCTCATGGTGGAACTTCTGTAGATCTAGCCATTTTTTCA
TTACATTTAGCTGGAATTTCCTCTATTCTAGGAGCTATTAACTTTATCACAACTATTATTAATATACGAATTAATGGATT
ATCTTTTGATCAAATACCTTTATTTGTGTGAGCAGTTGGTATTACTGCTCTTCTCCTTCTTCTCTCACTACCTGTATTAG
CGGGAGCTATTACTATATTATTAACAGATCGAAACTTAAATACTTCTTTCTTTGACCCAGCAGGAGGTGGAGATCCAATC
CTTTATCAACATTTATTTTGATTTTTT
>LSTEM134-18|Scirpophaga_excerptalis|am12499|
AACATTATATTTTATTTTTGGAATTTGAGCTGGTATAGTAGGAACTTCACTTAGTTTATTAATTCGAGCTGAATTAGGTA
CTCCTGGGTCACTAATTGGAGATGATCAAATCTATAATACTATCGTAACTGCTCATGCTTTTATTATAATTTTTTTTATA
GTTATACCTATTATAATTGGAGGATTTGGAAACTGATTAGTACCTTTAATATTAGGAGCCCCAGATATAGCTTTCCCTCG
AATAAATAATATAAGTTTTTGATTACTACCCCCTTCTTTAACTCTCTTAATCTCGAGAAGAATTGTTGAAAATGGAGCTG
GGACAGGATGAACTGTATACCCTCCTCTATCCTCTAATATTGCTCACGGTGGAACTTCTGTAGATCTAGCCATTTTTTCA
TTACATTTAGCTGGAATTTCCTCTATTCTAGGAGCTATTAACTTCATCACAACTATTATTAATATACGAATTAATGGGTT
ATCTTTTGATCAAATACCTTTATTTGTATGAGCAGTTGGTATTACCGCTCTTCTCCTTCTTCTCTCACTACCTGTATTAG
CAGGAGCTATTACTATATTATTAACAGATCGAAACTTAAATACTTCTTTCTTTGATCCAGCAGGAGGTGGAGATCCAATC
CTTTATCAACATTTATTTTGATTTTTT
>LSTEM135-18|Scirpophaga_excerptalis|am12500|
AACATTATATTTTATTTTTGGAATTTGAGCTGGTATAGTAGGAACTTCACTTAGTTTATTAATTCGAGCTGAATTAGGTA
CCCCTGGATCACTAATTGGAGATGATCAAATCTATAATACTATCGTAACTGCTCATGCTTTTATTATAATTTTTTTTATA
GTTATACCTATTATAATTGGGGGATTTGGAAACTGATTAGTACCTTTAATGTTAGGAGCTCCAGATATAGCTTTTCCTCG
AATAAATAATATAAGTTTTTGATTATTACCTCCTTCTTTAACCCTCTTAATCTCGAGAAGAATTGTTGAAAATGGAGCTG
GAACAGGATGAACTGTATACCCCCCTCTATCCTCTAATATTGCTCATGGTGGAACTTCTGTAGATCTAGCCATTTTTTCA
TTACATTTAGCTGGAATTTCCTCTATTCTAGGAGCTATTAACTTTATCACAACTATTATTAATATACGAATTAATGGATT
ATCTTTTGATCAAATACCTTTATTTGTATGAGCAGTTGGTATTACTGCTCTTCTCCTTCTTCTCTCACTACCTGTATTAG
CGGGAGCTATTACTATATTATTAACAGATCGAAACTTAAATACTTCTTTCTTTGACCCAGCAGGAGGTGGAGATCCAATC
CTTTATCAACATTTATTTTGATTTTTT
>LSTEM136-18|Scirpophaga_excerptalis|am12501|
---ATTATATTTTATTTTTGGAATTTGAGCTGGTATAGTGGGAACTTCACTTAGTTTATTAATTCGAGCTGAATTAGGTA
CCCCTGGGTCACTAATTGGAGATGATCAAATCTATAATACTATCGTAACTGCTCATGCTTTTATTATAATTTTTTTTATA
GTTATACCTATTATAATTGGGGGATTTGGAAACTGATTAGTACCTTTAATGTTAGGAGCTCCAGATATAGCTTTTCCTCG
AATAAATAATATAAGTTTTTGATTATTACCTCCTTCTTTAACCCTCTTAATCTCGAGAAGAATTGTTGAAAATGGAGCTG
GAACAGGATGAACTGTATACCCCCCTCTATCCTCTAATATTGCTCATGGTGGAACTTCTGTAGATCTAGCCATTTTTTCA
TTACATTTAGCTGGAATTTCCTCTATTCTAGGAGCTATTAACTTTATCACAACTATTATTAATATACGAATTAATGGATT
ATCTTTTGATCAAATACCTTTATTTGTATGAGCAGTTGGTATTACTGCTCTTCTCCTTCTTCTCTCACTACCTGTATTAG
CAGGAGCTATTACTATATTATTAACAGATCGAAACTTAAATACTTCTTTCTTTGACCCAGCAGGAGGTGGAGATCCAATC
CTTTATCAACATTTATTTTGATTTTTT
>LSTEM137-18|Scirpophaga_excerptalis|am12502|
AACATTATATTTTATTTTTGGAATTTGAGCTGGTATAGTAGGAACTTCACTTAGTTTATTAATTCGAGCTGAATTAGGTA
CTCCTGGGTCACTAATTGGAGATGATCAAATCTATAATACTATCGTAACTGCTCATGCTTTTATTATAATTTTTTTTATA
GTTATACCTATTATAATTGGGGGATTTGGAAACTGATTAGTACCTTTAATGTTAGGAGCCCCAGATATAGCTTTCCCTCG
AATAAATAATATAAGTTTTTGATTATTACCCCCTTCTTTAACTCTCTTAATCTCGAGAAGAATTGTTGAAAATGGAGCTG
GAACAGGATGAACTGTATACCCTCCTTTATCCTCTAATATTGCTCACGGTGGAACTTCTGTAGATCTAGCCATTTTTTCA
TTACATTTAGCTGGAATTTCCTCTATTCTGGGAGCTATTAACTTCATCACAACTATTATTAATATACGAATTAATGGATT
ATCTTTTGATCAAATACCTTTATTTGTATGAGCAGTTGGTATTACCGCTCTTCTTCTTCTTCTCTCACTACCTGTATTAG
CAGGAGCTATTACTATATTATTAACAGATCGAAACTTAAATACTTCTTTCTTTGATCCAGCAGGAGGTGGAGATCCAATC
CTTTATCAACATTTATTTTGATTTTTT
>LSTEM138-18|Scirpophaga_excerptalis|am12503|
AACATTATATTTTATTTTTGGAATTTGAGCTGGTATAGTAGGAACTTCACTTAGTTTATTAATTCGAGCTGAATTAGGTA
CTCCTGGATCACTAATTGGAGATGATCAAATCTATAATACTATCGTAACTGCTCATGCTTTTATTATAATTTTTTTTATA
GTTATACCTATTATAATTGGGGGATTTGGAAACTGATTAGTACCTTTAATGTTAGGAGCCCCAGATATAGCTTTCCCTCG
AATAAATAATATAAGTTTTTGATTATTACCCCCTTCTTTAACTCTCTTAATCTCGAGAAGAATTGTTGAAAATGGAGCTG
GAACAGGATGAACTGTATACCCTCCCCTATCCTCTAATATTGCTCACGGTGGAACTTCTGTAGATCTAGCCATTTTTTCG
TTACATTTAGCTGGAATTTCCTCTATTCTAGGAGCTATTAACTTCATCACAACTATTATTAATATACGAATTAATGGATT
ATCTTTTGATCAAATACCTTTATTTGTGTGAGCAGTTGGTATTACCGCTCTTCTCCTTCTTCTCTCACTACCTGTATTAG

CAGGAGCTATTACTATATTATTAACAGATCGAAACTTAAATACTTCTTTCTTTGATCCAGCAGGAGGTGGAGATCCAATC
CTTTATCAACATTTATTTTGATTTTTT
>LSTEM139-18|Scirpophaga_excerptalis|am12504|
---TAGTTTATTAATTCNAGCTGAATTAGGTA
CTCCTGGATCACTAATTGGAGATGATCAAATCTATAATACTATCGTAACTGCTCATGCTTTTATTATAATTTTTTTATA
GTTATACCTATTATAATTGGAGGATTTGGAAACTGATTAGTACCTTTAATATTAGGAGCCCCAGATATAGCTTTCCCTCG
AATAAATAATATAAGTTTTTGATTACTACCCCCTTCTTTAACTCTCTTAATCTCGAGAAGAATTGTTGAAAATGGAGCTG
GAACAGGATGAACTGTATACCCTCCTCTATCCTCTAATATTGCTCACGGTGGAACTTCTGTAGATCTAGCCATTTTTTCA
TTACATTTAGCTGGAATTTCCTTTATTCTAGGAGCTATTAACTTCATCACAACTATTATTAATATACGAATTAATGGGTT
ATCTTTTGATCAAATACCTTTATTTGTATGAGCAGKNGGTAKTACCGCTCTTCTCCTTCTTCTCTCACTACCTGTATTAG
CAGGAGCTATT--

>LSTEM140-18|Scirpophaga_excerptalis|am12505|
AACATTATATTTTATTTTTGGAATTTGAGCTGGTATAGTAGGAACTTCACTTAGTTTATTAATTCGAGCTGAATTAGGTA
CTCCTGGGTCACTAATTGGAGATGATCAAATCTATAATACTATCGTAACTGCTCATGCTTTTATTATAATTTTTTTATA
GTTATACCTATTATAATTGGAGGATTTGGAAACTGATTAGTACCTTTAATGTTAGGAGCCCCAGATATAGCTTTCCCTCG
AATAAATAATATAAGTTTTTGATTATTACCCCCTTCTTTAACTCTCTTAATCTCGAGAAGAATTGTTGAAAATGGAGCTG
GAACAGGATGAACTGTATACCCTCCTTTATCCTCTAATATTGCTCACGGTGGAACTTCTGTAGATCTAGCCATTTTTTCA
TTACATTTAGCTGGAATTTCCTCTATTCTAGGAGCTATTAACTTCATCACAACTATTATTAATATACGAATTAATGGATT
ATCTTTTGATCAAATACCTTTATTTGTATGAGCAGTTGGTATTACCGCTCTTCTTCTTCTTCTCTCACTACCTGTATTAG
CAGGAGCTATTACTATATTATTAACAGATCGAAACTTAAATACTTCTTTCTTTGATCCAGCAGGAGGTGGAGATCCAATC
CTTTATCAACATTTATTTTGATTTTTT
>LSTEM141-18|Scirpophaga_excerptalis|am12506|
AACATTATATTTTATTTTTGGAATTTGAGCTGGTATAGTAGGAACTTCACTTAGTTTATTAATTCGAGCTGAATTAGGTA
CTCCTGGGTCACTAATCGGRGATGATCAAATCTATAATACTATCGTAACTGCTCATGCTTTTATTATAATTTTTTTATA
GTTATACCTATTATAATTGGAGGATTTGGAAACTGATTAGTACCTTTAATATTAGGAGCCCCAGATATAGCTTTCCCTCG
AATAAATAATATAAGTTTTTGATTACTACCCCCTTCTTTAACTCTCTTAATCTCGAGAAGAATTGTTGAAAATGGAGCTG
GAACAGGATGAACTGTATACCCCCCTCTATCCTCTAATATTGCTCACGGTGGAACTTCTGTAGATYTAGCCATTTTTTCA
TTACATTTAGCTGGAATTTCCTCTATTCTAGGAGCTATTAACTTCATCACAACTATTATTAATATACGAATTAATGGRTT
ATCTTTTGATCAAATACCTTTATTTGTATGAGCAGTTGGTATTACCGCTCTTCTCCTTCTTCTCTCACTACCTGTATTAG
CGGGAGCTATTACTATATTATTAACAGATCGAAACTTAAATACTTCTTTCTTTGATCCAGCAGGAGGTGG----------
----
>LSTEM142-18|Scirpophaga_excerptalis|am12507|
AACATTATATTTTATTTTTGGAATTTGAGCTGGTATAGTAGGAACTTCACTTAGTTTATTAATTCGAGCTGAATTAGGTA
CCCCTGGGTCACTAATTGGAGATGATCAAATCTATAATACTATCGTAACTGCTCATGCTTTTATTATAATTTTTTTATA
GTTATACCTATTATAATTGGGGGATTTGGAAACTGATTAGTACCTTTAATGTTAGGAGCTCCAGATATAGCTTTTCCTCG
AATAAATAATATAAGTTTTTGATTATTACCTCCTTCTTTAACCCTCTTAATCTCGAGAAGAATTGTTGAAAATGGAGCTG
GAACAGGATGAACTGTATACCCCCCTCTATCCTCTAATATTGCTCATGGTGGAACTTCTGTAGATCTAGCCATTTTTTCA
TTACATTTAGCTGGAATTTCCTCTATTCTAGGAGCTATTAACTTTATCACAACTATTATTAATATACGAATTAATGGATT
ATCTTTTGATCAAATACCTTTATTTGTATGAGCAGTTGGTATTACTGCTCTTCTCCTTCTTCTCTCACTACCTGTATTAG
CGGGAGCTATTACTATATTATTAACAGATCGAAACTTAAATACTTCTTTCTTTGACCCAGCAGGAGGTGGAGATCCCATC
CTTTATCAACATTTATTTTGATTTTTT
>LSTEM143-18|Scirpophaga_excerptalis|am12508|
AACATTATATTTTATTTTTGGAATTTGAGCTGGTATAGTGGGAACTTCACTTAGTTTATTAATTCGAGCTGAATTAGGTA
CCCCTGGGTCACTAATTGGAGATGATCAAATCTATAATACTATCGTAACTGCTCATGCTTTTATTATAATTTTTTTATA
GTTATACCTATTATAATTGGGGGATTTGGAAACTGATTAGTACCTTTAATGTTAGGAGCTCCAGATATAGCTTTTCCTCG
AATAAATAATATAAGTTTTTGATTATTACCTCCTTCTTTAACCCTCTTAATCTCGAGAAGAATTGTTGAAAATGGAGCTG
GAACAGGATGAACTGTATACCCCCCTCTATCCTCTAATATTGCTCATGGTGGAACTTCTGTAGATCTAGCCATTTTTTCA
TTACATTTAGCTGGAATTTCCTCTATTCTAGGAGCTATTAACTTTATCACAACTATTATTAATATACGAATTAATGGATT
ATCTTTTGATCAAATACCTTTATTTGTATGAGCAGTTGGTATTACTGCTCTTCTCCTTCTTCTCTCACTACCTGTATTAG
CGGGAGCTATTACTATATTATTAACAGATCGAAACTTAAATACTTCTTTCTTTGACCCAGCAGGAGGTGGAGATCCAATC
CTTTATCAACATTTATTTTGATTTTTT
>LSTEM144-18|Scirpophaga_excerptalis|am12509|
AACATTATATTTTATTTTTGGAATTTGAGCTGGTATAGTAGGAACTTCACTTAGTTTATTAATTCGAGCTGAATTAGGTA
CTCCTGGGTCACTAATTGGAGATGATCAAATCTATAATACTATCGTAACTGCTCATGCTTTTATTATAATTTTTTTATA
GTTATACCTATTATAATTGGGGGATTTGGAAACTGATTAGTACCTTTAATGTTAGGAGCCCCAGATATAGCTTTCCCTCG
AATAAATAATATAAGTTTTTGATTATTACCCCCTTCTTTAACTCTCTTAATCTCGAGAAGAATTGTTGAAAATGGAGCTG

GAACAGGATGAACTGTATACCCTCCTCTATCCTCTAATATTGCTCACGGTGGAACTTCTGTAGATCTAGCCATTTTTTCA
TTACATTTAGCTGGAATTTCCTCTATTCTAGGAGCTATTAACTTCATCACAACTATTATTAATATACGAATTAATGGATT
ATCTTTTGATCAAATACCTTTATTTGTATGAGCAGTTGGTATTACCGCTCTTCTTCTTCTTCTCTCACTACCTGTATTAG
CAGGAGCTATTACTATATTATTAACAGATCGAAACTTAAATACTTCTTTCTTTGATCCAGCAGGAGGTGGAGATCCAATC
CTTTATCAACATTTATTTTGATTTTTT
>LSTEM145-18|Scirpophaga_excerptalis|am12510|
AACATTATATTTTATTTTTGGAATTTGAGCTGGTATAGTAGGAACTTCACTTAGTTTATTAATTCGAGCTGAATTAGGTA
CCCCTGGGTCACTAATTGGAGATGATCAAATCTATAATACTATCGTAACTGCTCATGCTTTTATTATAATTTTTTTTATA
GTTATACCTATTATAATTGGGGGATTTGGAAACTGATTAGTACCTTTAATGTTAGGAGCTCCAGATATAGCTTTTCCTCG
AATAAATAATATAAGTTTTTGATTATTACCTCCTTCTTTAACCCTCTTAATCTCGAGAAGAATTGTTGAAAATGGAGCTG
GAACAGGATGAACTGTATACCCCCCTCTATCCTCTAATATTGCTCATGGTGGAACTTCTGTAGATCTAGCCATTTTTTCA
TTACATTTAGCTGGAATTTCCTCTATTCTAGGAGCTATTAACTTTATCACAACTATTATTAATATACGAATTAATGGATT
ATCTTTTGATCAAATACCTTTATTTGTATGAGCAGTTGGTATTACTGCTCTTCTCCTTCTTCTCTCACTACCTGTATTAG
CGGGAGCTATTACTATATTATTAACAGATCGAAACTTAAATACTTCTTTCTTTGACCCAGCAGGAGGTGGAGATCCAATC
CTTTATCAACATTTATTTTGATTTTTT
>LSTEM146-18|Scirpophaga_excerptalis|am12511|
AACATTATATTTTATTTTTGGAATTTGAGCTGGTATAGTAGGAACTTCACTTAGTTTATTAATTCGAGCTGAATTAGGTA
CTCCTGGGTCACTAATTGGAGATGATCAAATCTATAATACTATCGTAACTGCTCATGCTTTTATTATAATTTTTTTTATA
GTTATACCTATTATAATTGGGGGATTTGGAAACTGATTAGTACCTTTAATGTTAGGAGCCCCAGATATAGCTTTTCCTCG
AATAAATAATATAAGTTTTTGATTATTACCTCCTTCTTTAACCCTCTTAATCTCGAGAAGAATTGTCGAAAATGGGGCTG
GAACAGGATGAACTGTATACCCCCCTCTATCCTCTAATATTGCTCATGGTGGAACTTCTGTAGATCTAGCCATTTTTTCA
TTACATTTAGCTGGAATTTCCTCTATTCTAGGAGCTATTAACTTTATCACAACTATTATTAATATACGAATTAATGGATT
ATCTTTTGATCAAATACCTTTATTTGTGTGAGCAGTTGGTATTACCGCTCTTCTCCTTCTTCTCTCACTACCTGTATTAG
CAGGAGCTATTACTATATTATTAACAGATCGAAACTTAAATACTTCTTTCTTTGACCCAGCAGGAGGTGGAGATCCAATT
CTTTATCAACATTTATTTTGATTTTTT
>LSTEM147-18|Scirpophaga_excerptalis|am12512|
AACATTATATTTTATTTTTGGAATTTGAGCTGGTATAGTAGGAACTTCACTTAGTTTATTAATTCGAGCTGAATTAGGTA
CTCCTGGGTCACTAATCGGGGATGATCAAATCTATAATACTATCGTAACTGCTCATGCTTTTATTATAATTTTTTTTATA
GTTATACCTATTATAATTGGAGGATTTGGAAACTGATTAGTACCTTTAATATTAGGAGCCCCAGATATAGCTTTCCCTCG
AATAAATAATATAAGTTTTTGATTACTACCCCCTTCTTTAACTCTCTTAATCTCGAGAAGAATTGTTGAAAATGGAGCTG
GAACAGGATGAACTGTATACCCCCCTCTATCCTCTAATATTGCTCACGGTGGAACTTCTGTAGATCTAGCCATTTTTTCA
TTACATTTAGCTGGAATTTCCTCTATTCTAGGAGCTATTAACTTCATCACAACTATTATTAATATACGAATTAATGGGTT
ATCTTTTGATCAAATACCTTTATTTGTATGAGCAGTTGGTATTACCGCTCTTCTCCTTCTTCTCTCACTACCTGTATTAG
CGGGAGCTATTACTATATTATTAACAGATCGAAACTTAAATACTTCTTTCTTTGATCCAGCAGGAGGTGGAGATCCAATC
CTTTATCAACATTTATTTTGATTTTTT
>LSTEM148-18|Scirpophaga_excerptalis|am12513|
AACATTATATTTTATTTTTGGAATTTGAGCTGGTATAGTAGGAACTTCACTTAGTTTATTAATTCGAGCTGAATTAGGTA
CTCCTGGGTCACTAATTGGAGATGATCAAATCTATAATACTATCGTAACTGCTCATGCTTTTATTATAATTTTTTTTATG
GTTATACCTATTATAATTGGAGGATTTGGAAACTGATTAGTACCTTTAATATTAGGAGCCCCAGATATAGCTTTCCCTCG
AATAAATAATATAAGTTTTTGATTACTACCCCCTTCTTTAACTCTCTTAATCTCGAGAAGAATTGTTGAAAATGGAGCTG
GAACAGGATGAACTGTATACCCTCCTCTATCCTCTAATATTGCTCACGGTGGAACTTCTGTAGATCTAGCCATTTTTTCA
TTACATTTAGCTGGAATTTCCTCTATTCTAGGAGCTATTAACTTCATCACAACTATTATTAATATACGAATTAATGGGTT
ATCTTTTGATCAAATACCTTTATTTGTATGAGCAGTTGGTATTACTGCTCTTCTCCTTCTTCTCTCACTACCTGTATTAG
CAGGAGCTATTACTATATTATTAACAGATCGAAACTTAAATACTTCTTTCTTTGATCCAGCAGGAGGTGGAGATCCAATC
CTTTATCAACATTTATTTTGATTTTTT
>LSTEM149-18|Scirpophaga_excerptalis|am12514|
AACATTATATTTTATTTTTGGAATTTGAGCTGGTATAGTAGGAACTTCACTTAGTTTATTAATTCGAGCTGAATTAGGTA
CCCCTGGGTCACTAATTGGAGATGATCAAATCTATAATACTATCGTAACTGCTCATGCTTTTATTATAATTTTTTTTATA
GTTATACCTATTATAATTGGGGGATTTGGAAACTGATTAGTACCTTTAATGTTAGGAGCTCCAGATATAGCTTTTCCTCG
AATAAATAATATAAGTTTTTGATTATTACCTCCTTCTTTAACCCTCTTAATCTCGAGAAGAATTGTTGAAAATGGAGCTG
GAACAGGATGAACTGTATACCCCCCTCTATCCTCTAATATTGCTCATGGTGGAACTTCTGTAGATCTAGCCATTTTTTCA
TTACATTTAGCTGGAATTTCCTCTATTCTAGGAGCTATTAACTTYATCACAACTATTATTAATATACGAATTAATGGATT
ATCTTTTGATCAAATACCTTTATTTGTATGAGCAGTTGGTATTACTGCTCTTCTCCTTCTTCTCTCACTACCTGTATTAG
CGGGAGCTATTACTATATTATTAACAGATCGAAACTTAAATACTTCTTTCTTTGATCCAGCAGGAGGTGGAGATCCAATC
CTTTATCAACATTTATTTTGATTTTTT
>LSTEM150-18|Scirpophaga_excerptalis|am12515|
--TTAGTTTATTAATTCGAGCTGAATTAGGTA

CTCCYGGATCACTAATTGGAGATGATCAAATCTATAATACTATCGTAACTGCTCATGCTTTTATTATAATTTTTTTTATA
GTTATACCTATTATAATTGGGGGATTTGGAAACTGATTAGTACCTTTAATGTTAGGAGCCCCAGATATAGCTTTTCCTCG
AATAAATAATATAAGTTTTTGATTATTACCTCCTTCTTTAACCCTCTTAATCTCGAGAAGAATTGTTGAAAATGGAGCTG
GAACAGGATGAACTGTATACCCCCCTCTATCCTCTAATATTGCTCATGGTGGAACTTCTGTAGATCTAGCCATTTTTTCA
TTACATTTAGCTGGAATTTCCTCTATTCTAGGAGCTATTAACTTTATCACAACTATTATTAATATACGAATTAATGGATT
ATCTTTTGATCAAATACCTTTATTTGTCTGAGCAGTTGGTATTACCGCTCTTCTCCTTCTTCTCTCACTACCTGTATTAG
CAGGAGCTATTACTATATTATTAACAGATCGAAACTTAAATACTTCTTTCTTTGACCCAGCAGGAGGTGGAGATCCAATT
CTTTATCAACATTTATTTTGATTTTTT
>LSTEM151-18|Scirpophaga_excerptalis|am12516|
AACATTATATTTTATTTTTGGAATTTGAGCTGGTATAGTAGGAACTTCACTTAGTTTATTAATTCGAGCTGAATTAGGTA
CTCCTGGATCACTAATTGGAGATGATCAAATCTATAATACTATCGTAACTGCTCATGCTTTTATTATAATTTTTTTTATA
GTTATACCTATTATAATTGGAGGATTTGGAAACTGATTAGTACCTTTAATATTAGGAGCCCCAGATATAGCTTTCCCTCG
AATAAATAATATAAGTTTTTGATTACTACCCCCTTCTTTAACTCTCTTAATCTCGAGAAGAATTGTTGAAAATGGAGCTG
GAACAGGATGAACTGTATACCCTCCTCTATCCTCTAATATTGCTCACGGTGGAACTTCTGTAGATCTAGCCATTTTTTCA
TTACATTTAGCTGGAATTTCCTCTATTCTAGGAGCTATTAACTTCATCACAACTATTATTAATATACGAATTAATGGGTT
ATCTTTTGATCAAATACCTTTATTTGTATGAGCAGTTGGTATTACCGCTCTTCTCCTTCTTCTCTCACTACCTGTATTAG
CAGGAGCTATTACTATATTATTAACAGATCGAAACTTAAATACTTCTTTCTTTGATCCAGCAGGAGGTGGAGATCCAATC
CTTTATCAACATTTATTTTGATTTTTT
>LSTEM152-18|Scirpophaga_excerptalis|am12518|
AACATTATATTTTATTTTTGGAATTTGAGCTGGTATAGTAGGAACTTCACTTAGTTTATTAATTCGAGCTGAATTAGGTA
CTCCTGGGTCACTAATTGGAGATGATCAAATCTATAATACTATCGTAACTGCTCATGCTTTTATTATAATTTTTTTTATG
GTTATACCTATTATAATTGGAGGATTTGGAAACTGATTAGTACCTTTAATATTAGGAGCCCCAGATATAGCTTTCCCTCG
AATAAATAATATAAGTTTTTGATTACTACCCCCTTCTTCAACTCTCTTAATCTCGAGAAGAATTGTTGAAAATGGAGCTG
GAACAGGATGAACTGTATACCCTCCTCTATCCTCTAATATTGCTCACGGTGGAACTTCTGTAGATCTAGCCATTTTTTCA
TTACATTTAGCTGGAATTTCCTCTATTCTAGGAGCTATTAACTTCATCACAACTATTATTAATATACGAATTAATGGGTT
ATCTTTTGATCAAATACCTTTATTTGTATGAGCAGTTGGTATTACTGCTCTTCTCCTTCTTCTCTCACTACCTGTATTAG
CAGGAGCTATTACTATATTATTAACAGATCGAAACTTAAATACTTCTTTCTTTGATC-----------------------
----
>LSTEM153-18|Scirpophaga_excerptalis|am12519|
AACATTATATTTTATTTTTGGAATTTGAGCTGGTATAGTAGGAACTTCACTTAGTTTATTAATTCGAGCTGAATTAGGTA
CTCCTGGGTCACTAATTGGAGATGATCAAATCTATAATACTATCGTAACTGCTCATGCTTTTATTATAATTTTTTTTATG
GTTATACCTATTATAATTGGAGGATTTGGAAACTGATTAGTACCTTTAATATTAGGAGCCCCAGATATAGCTTTCCCTCG
AATAAATAATATAAGTTTTTGATTACTACCCCCTTCTTTAACTCTCTTAATCTCGAGAAGAATTGTTGAAAATGGAGCTG
GAACAGGATGAACTGTATACCCTCCTCTATCCTCTAATATTGCTCACGGTGGAACTTCTGTAGATCTAGCCATTTTTTCA
TTACATTTAGCTGGAATTTCCTCTATTCTAGGAGCTATTAACTTCATCACAACTATTATTAATATACGAATTAATGGGTT
ATCTTTTGATCAAATACCTTTATTTGTATGAGCAGTTGGTATTACTGCTCTTCTCCTTCTTCTCTCACTACCTGTATTAG
CAGGAGCTATTACTATATTATTAACAGATCGAAACTTAAATACTTCTTTCTTTGATCCAGCAGGAGGTGGAGATCCAATC
CTTTATCAACATTTATTTTGATTTTTT
>LSTEM154-18|Chilo_auricilius|am12520|
AACTTTATACTTTATTTTTGGAATTTGGAGTGGAATAATTGGAACATCTCTAAGACTTTTAATTCGTGCTGAATTAGGAA
CTCCAGGGTCATTAATTGGAGATGATCAAATTTACAATACTATTGTTACAGCTCATGCATTTATTATAATTTTTTTTATA
GTTATACCAATTATAATCGGAGGCTTTGGTAATTGATTAGTACCATTAATACTAGGGGCTCCTGATATAGCCTTCCCTCG
AATAAATAATATAAGATTTTGATTATTGCCCCCATCATTAACATTATTAATTTCTAGAAGAATTGTAGAAAATGGAGCTG
GAACAGGATGAACGGTTTACCCCCCCCTTTCATCAAATATTGCCCATGGAGGAAGTTCTGTAGATTTAGCTATTTTTTCT
CTTCATTTAGCTGGTATTTCCTCAATTTTAGGAGCTATTAATTTTATTACAACAATTATTAATATACGAATTAATAAACT
ATCATTTGATCAATTACCATTATTTGTTTGATCTGTTGGTATTACAGCTTTATTATTACTACTTTCATTGCCGGTACTAG
CTGGAGCTATTACTATACTTTTAACTGATCGAAACTTAAATACATCTTTTTTTGACCCTGCTGGAGGAGGAGACCCAATT
CTTTATCAGCATTTATTTTGATTTTTT
>LSTEM155-18|Chilo_sacchariphagus|am12521|
AACTTTATATTTTATTTTTGGAATTTGAGCTGGAATAATTGGAACATCCCTTAGACTTTTAATTCGAGCTGAATTAGGAA
ATCCAGGTTCATTAATTGGAGATGATCAAATTTATAATACTATTGTTACAGCTCATGCATTTATTATAATTTTTTTTATA
GTAATACCAATTATAATCGGAGGATTTGGAAATTGATTAGTTCCATTAATATTAGGAGCTCCTGATATAGCCTTTCCTCG
TTTAAATAATATAAGATTTTGATTATTGCCCCCTTCTTTAACTCTTTTAATTTCTAGAAGAATTGTTGAAAATGGAGCAG
GAACTGGATGAACAGTCTACCCCCCCCTATCTTCCAATATTTCACATGCTGGAAGTTCAGTAGATTTAGCCATCTTTTCT
CTTCATTTAGCAGGAATTTCTTCAATTTTAGGAGCTATTAATTTTATTACTACAATTATTAATATACGAATTAATGGATT
ATTATTTGATCAAATACCATTATTTGTTTGATCTGTTGGTATTACAGCATTACTTCTTCTTCTCTCTTTACCAGTATTAG
CAGGTGCTATTACCATACTATTAACTGACCGAAATTTAAATACATCTTTTTTTGATCCAGCTGGAGGAGGTGATCCAATT

TTATATCAACATTTATTTTGATTTTTT
>LSTEM156-18|Chilo_sacchariphagus|am12522|
AACTTTATATTTTATTTTTGGAATTTGAGCTGGAATAATTGGAACATCCCTTAGACTTTTAATTCGAGCTGAATTAGGAA
ATCCAGGTTCATTAATTGGAGATGATCAAATTTATAATACTATTGTTACAGCTCATGCATTTATTATAATTTTTTTTATA
GTAATACCAATTATAATCGGAGGATTTGGAAATTGATTAGTTCCATTAATATTAGGAGCTCCTGATATAGCCTTTCCTCG
TTTAAATAATATAAGATTTTGATTATTACCCCCTTCTTTAACTCTTTTAATTTCTAGAAGAATTGTTGAAAATGGAGCAG
GAACTGGATGAACAGTCTACCCCCCCCTATCTTCCAATATTTCACATGCTGGAAGTTCAGTAGATTTAGCCATCTTTTCT
CTTCATTTAGCAGGAATTTCTTCAATTTTAGGAGCTATTAATTTTATTACTACAATTATTAATATACGAATTAATGGATT
ATTATTTGATCAAATACCATTRTWWGTYTGAT--

>LSTEM157-18|Tetramoera_schistaceana|am12523|
AACATTATATTTTATTTTTGGAATTTGAGCCGGAATAATTGGAACATCTCTAAGATTATTAATTCGAGCAGAATTAGGAA
ATCCTGGCTCTTTAATTGGAGATGATCAAATTTATAATACTATTGTAACTGCTCATGCTTTTATTATAATTTTTTTCATA
GTTATACCTATCATAATTGGAGGATTTGGAAATTGATTAGTACCATTAATATTAGGAGCCCCTGATATAGCTTTTCCTCG
TATAAATAATATAAGATTTTGATTACTCCCTCCTTCTATTATATTATTAATTTCAAGAAGAATTGTAGAAAATGGAGCAG
GAACAGGATGAACAGTTTATCCCCCCCTTTCATCTAATATTGCCCATAGAGGTAGATCAGTAGATCTAGCTATTTTTTCT
TTACATTTAGCTGGAATTTCTTCTATTTTAGGAGCTGTAAACTTTATTACAACTATTATTAATATACGACCAAATAATAT
AAGATTAGATCAAATACCCCTATTTGTTTGAGCTGTTGGCATTACAGCTCTTCTTTTATTATTATCTTTACCAGTATTAG
CAGGAGCTATTACTATACTCTTAACAGACCGTAATTTAAATACTTCATTTTTTGATCCTGCTGGTGGAGGAGATCCAATT
TTATACCAACACTTATTTTGATTTTTT
>LSTEM158-18|Scirpophaga_excerptalis|am12524|
AACATTATATTTTATTTTTGGAATTTGAGCTGGTATAGTAGGAACTTCACTTAGTTTATTAATTCGAGCTGAATTAGGTA
CTCCTGGGTCACTAATCGGGAATGATCAAATCTATAATACTATCGTAACTGCTCATGCTTTTATTATAATTTTTTTTATA
GTTATACCTATTATAATTGGAGGATTTGGAAACTGATTAGTACCTTTAATATTAGGAGCCCCAGATATAGCTTTCCCTCG
AATAAATAATATAAGTTTTTGATTACTACCCCCTTCTTTAACTCTCTTAATCTCGAGAAGAATTGTTGAAAATGGAGCTG
GAACAGGATGAACTGTATACCCCCCTCTATCCTCTAATATTGCTCACGGTGGAACTTCTGTAGATCTAGCCATTTTTTCA
TTACATTTAGCTGGAATTTCCTCTATTCTAGGAGCTATTAACTTCATCACAACTATTATTAATATACGAATTAATGGGTT
ATCTTTTGATCAAATACCTTTATTTGTATGAGCAGTTGGTATTACCGCTCTTCTCCTTCTTCTCTCACTACCTGTATTAG
CGGGAGCTATTACTATATTATTAACAGATCGAAACTTAAATACTTCTTTCTTTGATCCAGCAGGAGGTGGAGATCCAATC
CTTTATCAACATTTATTTTGATTTTTT
>LSTEM159-18|Sesamia_nonagrioides|am12531|
AACATTATATNNNATTTTNGGWATTTGAGCTGGAATAGTAGGAACTTCATTAAGNCTATTAATTCGAGCTGAATTAGGAA
CTCCTGGATCTTTAATTGGAGATGATCAAATTTATAATAATATTGTTACAGCTCATGCTTTTATTATAATTTTTTTTATA
GTTATACCTATTATAATTGGAGGATTTGGAAATTGACTTGTACCTTTAATATTAGGAGCCCCAGATATAGCATTTCCACG
AATAAATAATATAAGATTTTGACTATTACCACCATCCTTAACCCTTTTAATTTCAAGTAGAATTGTAGAAAATGGAGCTG
GAACAGGATGAACAGTTTACCCCCCACTTTCATCTAACATCGCTCATGGGGGAAGATCTGTAGATTTAGCTATTTTTTCC
CTTCATTTGGCTGGGATTTCATCTATTCTAGGAGCTATTAATTTTATTACAACAATTATTAATATACGATTAAATAATTT
ATCATTTGATCAAATACCATTATTTATTTGAGCTGTTGGAATTACTGCTTTTTTATTACTATTATCATTACCCGTTTTAG
CAGGAGCTATTACTATACTACTTACGGATCGAAATTTAAATACATCATTTTTTGATCCTGCGGGAGGAGGNGATCCAATT
TTATACCAACACTTATTCTGATTTTTT
>LSTEM160-18|Busseola_segeta|am12533|
AACATTATATTTTATTTTTGGAATTTGAGCTGGTATAGTGGGAACCTCATTAAGATTATTAATTCGAGCTGAATTAGGAA
CTCCTGGTTCTTTAATTGGAGATGATCAAATTTACAATACTATTGTTACAGCACATGCTTTTATTATAATTTTTTTTATA
GTTATACCTATTATAATTGGAGGGTTTGGAAATTGACTTGTACCTTTAATATTAGGAGCCCCAGATATAGCATTTCCACG
AATAAATAATATAAGTTTTTGATTATTACCTCCTTCTTTAACATTACTAATTTCGAGAAGAATTGTAGAAAATGGAGCAG
GAACAGGATGAACTGTATACCCCCCACTTTCATCTAATATTGCTCATGGAGGTAGATCTGTGGATTTAGCTATTTTTTCC
TTGCACTTAGCTGGTATTTCTTCTATTTTAGGTGCTATTAATTTCATTACAACAATTATTAATATACGTTTAAATAGTCT
TTCTTTTGATCAAATACCTTTATTTATTTGAGCTGTAGGAATTACTGCATTTTTATTATTATTATCATTACCTGTTTTAG
CAGGGGCTATTACAATATTATTAACTGATCGAAATTTAAACACATCATTTTTTGATCCTGCTGGAGGAGGAGATCCAATT
TTATATCAACATTTGTTTTGATTTTTT
>LSTEM161-18|Sesamia_nonagrioides|am12536|
AACATTATATTTTATTTTTGGAATTTGAGCTGGAATAGTAGGAACTTCATTAAGACTATTAATTCGAGCTGAATTAGGAA
CCCCTGGATCTTTAATTGGAGATGATCAAATTTATAATACTATTGTTACAGCTCATGCTTTTATTATAATTTTTTTTATA
GTTATACCTATTATAATTGGAGGATTTGGAAATTGACTTGTACCTTTAATATTAGGAGCCCCAGATATAGCATTTCCACG
AATAAATAATATAAGATTTTGACTATTACCACCATCCTTAACCCTTTTAATTTCAAGTAGAATTGTAGAAAATGGAGCTG
GAACAGGATGAACAGTTTACCCCCCACTTTCATCTAACATCGCTCATGGGGGAAGATCTGTAGATTTAGCTATTTTTTCC

CTTCATTTGGCTGGGATTTCATCTATTCTAGGAGCTATTAATTTTATTACAACAATTATTAATATACGATTAAATAATTT
ATCATTTGATCAAATACCATTATTTATTTGAGCTGTTGGAATTACTGCTTTTTATTACTATTATCATTACCCGTTTTAG
CAGGAGCTATTACTATACTACTTACGGATCGAAATTTAAATACATCATTTTTTGATCCTGCGGGAGGAGGTGATCCAATT
TTATACCAACACTTATTCTGATTTTTT
>LSTEM162-18|Sesamia_calamistis|am12537|
AACATTATATTTTATTTTTGGAATTTGAGCAGGAATAGTAGGAACTTCATTAAGTTTATTAATTCGAGCTGAATTAGGAA
CTCCTGGCTCTTTAATTGGAGATGATCAAATTTATAATACTATTGTCACAGCTCATGCTTTTATTATAATTTTTTTTATA
GTTATACCTATTATAATTGGAGGATTTGGAAACTGACTTGTACCTTTAATATTAGGAGCACCAGATATAGCATTTCCACG
AATAAATAATATAAGATTTTGATTATTACCCCCATCCTTAACCCTTTTAATTTCAAGTAGAATCGTAGAAAACGGAGCAG
GAACAGGATGAACAGTATATCCCCCACTTTCATCTAATATTGCTCATGGGGGAAGATCAGTAGATTTAGCTATTTTTTCT
CTTCATTTAGCTGGGATTTCATCTATTTTAGGAGCAATTAATTTTATTACAACAATTATTAATATACGATTAAATAGTCT
ATCATTCGATCAAATACCCCTATTCATTTGGGCTGTTGGAATTACTGCCTTTTTATTACTATTATCTTTACCTGTTTTAG
CGGGAGCTATTACTATATTACTTACAGATCGAAATTTAAATACTTCATTTTTTGATCCTGCAGGAGGAGGAGATCCAATT
TTATATCAACACTTATTTTGATTTTTT
>LSTEM163-18|Busseola_fusca|am12538|
AACATTATATTTTATTTTTGGAATTTGAGCTGGTATAGTAGGAACCTCTTTGAGATTATTAATTCGAGCTGAATTAGGAA
CTCCTGGTTCTTTAATTGGGGATGATCAAATCTATAATACTATTGTTACAGCTCATGCTTTTATTATAATTTTTTTTATA
GTTATACCAATTATAATTGGGGGATTTGGAAATTGACTTGTACCCCTAATATTAGGAGCCCCAGATATAGCATTCCCACG
AATAAATAATATAAGTTTTTGATTATTACCTCCTTCTTTAACTCTATTAATTTCAAGAAGAATTGTAGAAAATGGAGCGG
GAACAGGATGAACAGTATATCCCCCACTTTCATCTAATATTGCTCATGGAGGAAGATCTGTAGATTTAGCTATTTTTTCA
TTACATTTAGCTGGTATTTCTTCTATTTTAGGAGCTATTAATTTTATTACAACAATTATTAATATGCGTTTAAATAGTCT
TTCTTTCGATCAAATACCTTTATTTATCTGAGCTGTAGGAATTACCGCATTTTTATTATTACTATCATTACCAGTTTTAG
CAGGAGCTATTACAATATTATTAACCGATCGAAACTTAAATACATCATTCTTTGATCCTGCGGGAGGAGGTGATCCGATT
TTATATCAACACTTATTTTGATTTTTT
>LSTEM164-18|Busseola_segeta|am12539|
AACATTATATTTTATTTTTGGAATTTGAGCTGGTATAGTAGGAACCTCATTAAGATTATTAATTCGAGCTGAATTAGGAA
CTCCTGGTTCTTTAATTGGAGATGATCAAATTTACAATACTATTGTTACAGCACATGCTTTTATTATAATTTTTTTTATA
GTTATACCTATTATAATTGGAGGATTTGGGAATTGACTTGTACCTTTAATATTAGGAGCCCCAGATATGGCATTTCCACG
AATAAATAATATAAGTTTTTGATTATTACCTCCTTCTTTAACATTACTAATTTCGAGAAGAATTGTAGAAAATGGAGCAG
GAACAGGATGAACTGTATACCCTCCACTTTCATCTAATATTGCTCATGGGGGTAGATCTGTAGATTTAGCTATTTTTTCC
CTGCACTTAGCTGGTATTTCTTCTATTTTAGGTGCTATTAATTTCATTACAACAATTATTAATATACGTTTAAATAGTCT
TTCTTTTGATCAAATACCTTTATTTATTTGAGCTGTAGGAATTACTGCATTTTTGTTATTATTGTCATTACCTGTTTTAG
CAGGGGCTATTACAATATTATTAACTGATCGAAATTTAAACACATCATTTTTTGATCCTGCTGGAGGAGGAGATCCAATT
TTATATCAACATTTGTTTTGATTTTTT
>LSTEM165-18|Chilo_partellus|am12540|
AACTTTATATTTTATTTTTGGAATTTGAGCAGGAATAATTGGGACATCCCTTAGATTATTAATTCGTGCAGAATTAGGAA
CTCCTGGATCTTTAATTGGAGATGATCAAATTTATAATACTATTGTAACAGCACACGCATTTATTATAATTTTTTTTATA
GTTATACCAATTATAATTGGTGGATTTGGAAATTGATTAGTACCTTTAATATTAGGAGCCCCAGATATAGCTTTCCCACG
AATAAATAATATAAGATTTTGATTATTACCACCATCATTAACTTTATTAATTTCTAGAAGAATTGTTGAAAATGGAGCTG
GAACAGGATGAACAGTGTACCCCCCACTATCATCTAATATTGCTCATGCCGGAAGTTCAGTAGATTTAGCAATTTTTTCT
TTACATTTAGCTGGTATTTCATCAATTCTCGGTGCTATTAATTTTATTACAACAATTATTAATATACGAATTAATGGATT
ATCTTTTGATCAAATACCATTATTTGTTTGATCTGTAGGTATTACAGCTTTATTATTATTACTTTCTTTACCTGTTTTAG
CTGGAGCTATTACTATATTATTAACAGATCGAAATTTAAATACATCCTTTTTCGATCCTGCTGGAGGAGGAGATCCTATT
CTTTATCAACACTTATTTTGATTTTTT
>LSTEM166-18|Pyralidae|am12545|
AACTTTATATTTTATTTTTGGTATTTGAGCTGGAATAGTTGGAACTTCTTTAAGTTTACTAATTCGAGCAGAATTAGGAA
ATCCTGGATCTTTAATTGGAGATGATCAAATTTATAATACTATTGTAACAGCTCATGCATTTATTATAATTTTTTTTATA
GTTATACCTATTATAATTGGAGGATTTGGAAATTGATTAGTTCCTCTAATATTAGGAGCACCTGATATAGCATTCCCTCG
AATAAATAATATAAGTTTTTGACTTTTACCCCCTTCTTTAACTTTATTAATTTCAAGAAGAATTGTTGAAAATGGAGCAG
GAACAGGTTGAACAGTATATCCCCCTCTTTCTTCTAATATTGCTCATGGTGGAAGTTCTGTAGATTTAGCAATTTTTTCC
TTACATTTAGCTGGAATTTCTTCAATTTTAGGAGCTATTAATTTTATTACAACTATTATTAATATACGAATTAATAGTTT
ATCTTTTGATCAAATACCTTTATTTGTTTGATCAGTAGGAATTACTGCTTTACTTTTACTTCTTTCTTTACCAGTATTAG
CTGGAGCAATTACAATATTATTAACTGATCGAAATTTAAATACATCATTTTTTGATCCTGCAGGAGGAGGAGATCCAATT
TTATATCAACATTTATTTTGATTTTTT
>LSTEM167-18|Sesamia_inferens|am12552|
AACATTATATTTTATTTTTGGAATTTGAGCTGGTATAGTAGGAACTTCATTAAGATTATTAATTCGAGCTGAATTAGGAA
TTCCTGGATCTTTAATTGGGGATGATCAAATTTATAACACTATTGTTACAGCTCATGCTTTTATTATAATTTTTTTTATA

GTTATACCAATTATAATTGGGGGATTTGGTAATTGACTTGTACCTTTAATATTAGGAGCTCCAGATATAGCATTCCCACG
AATAAATAATATAAGATTTTGATTATTACCCCCCTCCTTAACTTTATTAATTTCAAGTAGAATTGTAGAAAATGGGGCAG
GTACAGGATGAACAGTATATCCACCTCTCTCATCTAATATTGCCCATGGGGGAAGATCAGTAGACTTAGCTATTTTTTCT
CTTCATTTAGCGGGTATTTCATCTATTTTAGGAGCTATTAATTTTATTACAACAATTATTAATATACGATTAAATAGATT
ATCCTTTGATCAAATACCTTTATTTGTTTGAGCTGTTGGGATTACTGCATTTTTATTATTATTATCTTTACCTGTTTTAG
CGGGGGCTATTACAATGTTATTAACAGATCGAAACTTAAATACATCCTTCTTTGACCCTGCGGGAGGGGGTGATCCAATT
TTATATCAACAYYTRTTYTGATTCTTT
>LSTEM168-18|Sesamia_inferens|am12553|
AA-------------TTTTGGAGTCTGGTATTTAGTTAGGGAACTTTCATTAAGATTATTAATTCGAGCTGAATTAGGAA
TTCCTGGATCTTTAATTGGGGATGATCAAATTTATAACACTATTGTTACAGCTCATGCTTTTATTATAATTTTTTTTATA
GTTATACCAATTATAATTGGGGGATTTGGTAATTGACTTGTACCTTTAATATTAGGAGCTCCAGATATAGCATTCCCACG
AATAAATAATATAAGATTTTGATTATTACCCCCCTCCTTAACTTTATTAATTTCAAGTAGAATTGTAGAAAATGGGGCAG
GTACAGGATGAACAGTATATCCACCTCTCTCATCTAATATTGCCCATGGGGGAAGATCAGTAGACTTAGCTATTTTTTCT
CTTCATTTAGCGGGTATTTCATCTATTTTAGGAGCTATTAATTTTATTACAACAATTATTAATATACGATTAAATAGATT
ATCCTTTGATCAAATACCTTTATTTGTTTGAGCTGTTGGGATTACTGCATTTTTATTATTATTATCTTTACCTGTTTTAG
CGGGGGCTATTACAATGTTATTAACAGATCGAAACTTAAATACATCCTTCTTTGACCCTGCGGGAGGGGGTGATCCAATT
TTATATCAACATTTATTTTGATTTTTT
>LSTEM169-18|Sesamia_inferens|am12554|
AACATTATATTTTATTTTTGGAATTTGAGCTGGTATAGTAGGAACTTCATTAAGATTATTAATTCGAGCTGAATTAGGAA
TTCCTGGATCTTTAATTGGGGATGATCAAATTTATAACACTATTGTTACAGCTCATGCTTTTATTATAATTTTTTTTATA
GTTATACCAATTATAATTGGGGGATTTGGTAATTGACTTGTACCTTTAATATTAGGAGCTCCAGATATAGCATTCCCACG
AATAAATAATATAAGATTTTGATTATTACCCCCCTCCTTAACTTTATTAATTTCAAGTAGAATTGTAGAAAATGGGGCAG
GTACAGGATGAACAGTATATCCACCTCTCTCATCTAATATTGCCCATGGGGGAAGATCAGTAGACTTAGCTATTTTTTCT
CTTCATTTAGCGGGTATTTCATCTATTTTAGGAGCTATTAATTTTATTACAACAATTATTAATATACGATTAAATAGATT
ATCCTTTGATCAAATACCTTTATTTGTTTGAGCTGTTGGGATTACTGCATTTTTATTATTATTATCTTTACCTGTTTTAG
CGGGGGCTATTACAATGTTATTAACAGATCGAAACTTAAATACATCCTTCTTTGACCCTGCGGGAGGGGGTGATCCAATT
TTATATCAACATTTATTTTGATTTTTT
>LSTEM170-18|Chilo_sacchariphagus|am12555|
AACYTTATATTTTATTTTTGGAATNTGAGCTGGAATAGTTGGAACATCCCTTAGACTTTTAATTCGAGCTGAATTAGGAA
ATCCAGGTTCATTAATCGGAGATGATCAAATTTATAATACTATTGTTACAGCCCATGCATTTATTATAATTTTTTTTATA
GTAATACCAATYATAATTGGAGGATTTGGAAATTGATTAGTTCCATTAATATTAGGGGCTCCTGATATAGCCTTCCCTCG
TCTAAATAATATAAGATTTTGATTATTACCCCCTTCTTTAACCCTTCTAATTTCTAGAAGAATCGTTGAAAATGGAGCAG
GAACTGGATGAACAGTCTACCCCCCCCTATCTTCCAATATTTCACATGCTGGAAGTTCAGTAGATTTAGCCATCTTCTCC
CTTCATTTAGCTGGAATTTCTTCAATTTTAGGAGCTATCAATTTCATTACTACAATTATTAATATACGAATTAATGGATT
ATTATTTGATCAAATACCATTATTTGTTTGATCTGTTGGTATTACAGCATTACTTCTCCTCCTTTCTTTACCAGTATTAG
CAGGTGCTATTACTATACTATTAACTGACCGAAATTTAAATACATCTTTTTTTGACCCAGCTGGAGGAGGTGATCCAATT
TTATATCAACATTTATTTTGATTTTTT
>LSTEM171-18|Sesamia_nonagrioides|am12556|
AACATTATATTTTATTTTTGGAATTTGAGCTGGAATAGTAGGAACTTCATTAAGACTATTAATTCGAGCTGAATTAGGAA
CTCCTGGATCTTTAATTGGAGATGATCAAATTTATAATACTATTGTTACAGCTCATGCTTTTATTATAATTTTTTTTATA
GTTATACCTATTATAATTGGAGGATTTGGAAATTGACTTGTACCTTTAATATTAGGAGCCCCAGATATAGCATTTCCACG
AATAAATAATATAAGATTTTGACTATTACCACCATCCTTAACCCTTTTAATTTCAAGTAGAATTGTAGAAAATGGGGCTG
GAACAGGATGAACAGTTTACCCCCCACTTTCATCTAACATCGCTCATGGAGGAAGATCTGTAGATTTAGCTATTTTTTCC
CTTCATTTAGCTGGAATTTCATCTATTCTAGGAGCTATTAATTTTATTACAACAATTATTAATATACGATTAAATAATTT
ATCATTTGATCAAATACCATTATTTATTTGAGCTGTTGGAATTACTGCTTTTTTATTACTATTATCATTACCCGTTTTAG
CAGGAGCTATTACTATACTACTTACGGATCGAAATTTAAATACATCATTTTTTGATCCTGCGGGAGGAGGTGATCCAATT
TTATACCAACACTTATTCTGATTTTTT
>LSTEM172-18|Chilo_suppressalis|am12558|
AACTTTATATTTTATTTTTGGTATTTGAGCAGGTATAATTGGAACATCTCTTAGACTTTTAATTCGTGCTGTTTCCGGAA
CTCSAGGATCTTTAATTGGAGATGATCAAATTTATAATACCATTGTTACGGCTCATGCATTTATTATAATTTTTTTTATA
GTTATACCAATTATAATTGGTGGATTTGGAAATTGATTAGTACCTTTAATATTAGGGGCTCCTGATATAGCTTTCCCACG
AATAAATAATATAAGATTTTGAATATTACCCCCCTCATTAACTTTACTAATTTCTAGAAGAATTGTTGAAAATGGAGCTG
GAACAGGTTGAACAGTGTACCCCCCACTATCATCTAATATTGCTCACGCTGGAAGTTCAGTAGATTTAGCAATTTTCTCT
TTACATTTAGCTGGAATTTCTTCAATTCTAGGTGCTATTAATTTTATTACTACGATTATTAATATACGAATTAATGGTCT
TTCATTTGATCAAATACCTTTATTTGTTTGATCCGTAGGTATTACAGCTTTATTATTACTTCTATCTCTACCAGTATTAG
CTGGAGCAATTACAATATTATTAACCGATCGAAATTTAAATACATCTTTTTTTGATCCTGCTGGTGGTGGAGATCCAATT
CTTTACCAACAYYTRTTCTGATTCTTT

>LSTEM173-18|Scirpophaga_excerptalis|am12561|
AACATTATATTTTATTTTTGGAATTTGAGCTGGTATAGTGGGGACTTCACTTAGTTTACTAATTCGAGCCGAACTAGGTA
CTCCGGGATCATTAATCGGAGATGATCAAATCTATAATACTATTGTAACTGCTCACGCTTTTATTATAATTTTTTTTATG
GTTATGCCCATTATAATTGGGGGATTCGGAAACTGATTAGTGCCTTTAATATTGGGAGCCCCAGATATGGCCTTCCCCCG
AATAAATAATATAAGTTTTTGATTATTACCCCCTTCTTTAACCCTCTTAATCTCAAGAAGAGTCGTTGAAAATGGAGCTG
GAACAGGATGAACTGTTTATCCGCCCTTATCCTCCAATATTGCTCATAGTGGGACTTCTGTAGATTTAGCCATTTTTTCA
TTACATTTAGCTGGAATTTCTTCTATTCTAGGGGCTATTAACTTCATTACAACTATTATTAATATACGAATTAATGGACT
ATCTTTTGATCAAATACCTTTATTTGTATGAGCAGTTGGTATTACTGCCCTTCTTCTTCTTCTCTCACTACCTGTATTAG
CGGGAGCTATTACTATATTATTAACAGATCGAAACTTAAATACTTCTTTCTTTGACCCAGCAGGAGGTGGAGACCCAATT
CTTTATCAACACTTATTTTGATTCTTT
>LSTEM174-18|Scirpophaga_excerptalis|am12562|
AACATTATATTTTATTTTTGGAATTTGAGCTGGTATAGTAGGGACTTCACTTAGTTTACTAATTCGAGCCGAACTAGGTA
CTCCTGGATCATTAATCGGAGATGATCAAATCTATAATACTATTGTAACTGCTCACGCTTTTATTATAATTTTTTTTATG
GTTATACCTATTATAATTGGGGGATTCGGGAACTGATTAGTGCCTTTAATATTGGGAGCCCCAGATATGGCCTTCCCCCG
AATAAATAATATAAGTTTTTGATTATTACCCCCTTCTTTAACCCTCTTAATCTCAAGAAGAATCGTTGAAAATGGGGCTG
GAACAGGATGAACTGTTTACCCGCCCTTATCCTCCAATATTGCTCATGGTGGAACTTCTGTAGATTTAGCCATTTTTTCA
TTGCATTTAGCTGGAATTTCTTCTATTCTAGGGGCTATTAACTTCATTACAACTATTATCAATATACGAATTAATGGACT
ATCTTTTGATCAAATACCTTTATTCGTATGAGCAGTTGGTATTACTGCCCTTCTTCTTCTCCTCTCACTACCTGTATTAG
CAGGAGCTATTACTATATTATTAACAGATCGAAACTTAAATACCTCTTTCTTTGACCCAGCAGGAGGTGGAGACCCAATT
CTTTATCAACACTTATTTTGATTTTTT
>LSTEM175-18|Sesamia_grisescens|am12564|
AACATTATATTTTATTTTCGGAATTTGAGCTGGTATAGTTGGAACATCACTAAGATTATTAATTCGAGCCGAACTAGGAA
CCCCTGGTTCTTTAATTGGAGACGATCAAATTTATAATACTATTGTTACAGCTCATGCTTTCATTATAATTTTCTTTATA
GTTATACCAATTATAATTGGTGGATTTGGGAATTGACTCGTACCTTTAATATTAGGAGCCCCTGATATAGCATTTCCACG
AATAAATAATATAAGATTTTGATTATTACCCCCCTCTTTAACTTTACTAATTTCAAGTAGAATTGTAGAAAATGGGGCAG
GAACTGGGTGAACAGTGTACCCCCCACTTTCATCTAATATTGCCCATGGAGGAAGATCAGTGGACTTAGCTATTTTTTCC
CTTCATTTAGCGGGTATTTCATCTATTTTAGGAGCTATTAATTTTATCACAACAATTATTAATATACGATTAAATAACTT
ATCCTTTGATCAAATACCTTTATTTATTTGAGCTGTTGGAATTACTGCATTTTTATTATTATTATCTTTACCTGTTTTAG
CAGGAGCTATTACAATATTATTAACCGATCGAAACTTAAATACATCATTTTTCGATCCGGCAGGAGGAGGCGATCCTATT
TTATACCAACATTTATTTTGATTTTTT
>LSTEM176-18|Sesamia_grisescens|am12565|
AACATTATATTTTATTTTCGGAATTTGAGCTGGTATAGTTGGAACATCACTAAGATTATTAATTCGAGCCGAACTAGGAA
CCCCTGGTTCTTTAATTGGAGACGATCAAATTTATAATACTATTGTTACAGCTCATGCTTTCATTATAATTTTCTTTATA
GTTATACCAATTATAATTGGTGGATTTGGGAATTGACTCGTACCTTTAATATTAGGAGCCCCTGATATAGCATTTCCACG
AATAAATAATATAAGATTTTGATTATTACCCCCCTCTTTAACTTTACTAATTTCAAGTAGAATTGTAGAAAATGGGGCAG
GAACTGGGTGAACAGTGTACCCCCCACTTTCATCTAATATTGCCCATGGAGGAAGATCAGTGGACTTAGCTATTTTTTCC
CTTCATTTAGCGGGTATTTCATCTATTTTAGGAGCTATTAATTTTATCACAACAATTATTAATATACGATTAAATAACTT
ATCCTTTGATCAAATACCTTTATTTATTTGAGCTGTTGGAATTACTGCATTTTTATTATTATTATCTTTACCTGTTTTAG
CAGGAGCTATTACAATATTATTAACCGATCGAAACTTAAATACATCATTTTTCGATCCGGCAGGAGGAGGCGATCCTATT
TTATACCAACATTTATTTTGATTTTTT
>LSTEM177-18|Sesamia_grisescens|am12566|
AACATTATATTTTATTTTCGGAATTTGAGCTGGTATAGTTGGAACATCACTAAGATTATTAATTCGAGCCGAACTAGGAA
CCCCTGGTTCTTTAATTGGAGACGATCAAATTTATAATACTATTGTTACAGCTCATGCTTTCATTATAATTTTCTTTATA
GTTATACCAATTATAATTGGTGGATTTGGGAATTGACTCGTACCTTTAATATTAGGAGCCCCTGATATAGCATTCCCACG
AATAAATAATATAAGATTTTGATTATTACCCCCCTCTTTAACTTTACTAATTTCAAGTAGAATTGTAGAAAATGGGGCAG
GAACTGGATGAACAGTGTACCCCCCACTTTCATCTAATATTGCCCATGGAGGAAGATCAGTGGACTTAGCTATTTTTTCT
CTTCATTTAGCAGGTATTTCATCTATTTTAGGGGCTATTAATTTTATCACAACAATTATTAATATACGATTAAATAACTT
ATCCTTTGATCAAATACCTTTATTTATTTGAGCTGTTGGAATTACTGCATTTTTATTATTATTATCTTTACCTGTTTTAG
CAGGAGCTATTACAATATTATTAACCGATCGAAACTTAAATACATCATTTTTCGATCCGGCAGGAGGAGGCGATCCTATT
TTATACCAACATTTATTTTGATTTTTT
>LSTEM178-18|Sesamia_grisescens|am12568|
AACATTATATTTTATTTTCGGAATTTGAGCTGGTATAGTTGGAACATCACTAAGATTATTAATTCGAGCCGAACTAGGAA
CCCCTGGTTCTTTAATTGGAGACGATCAAATTTATAATACTATTGTTACAGCTCATGCTTTCATTATAATTTTCTTTATA
GTTATACCAATTATAATTGGTGGATTTGGGAATTGACTCGTACCTTTAATATTAGGAGCCCCTGATATAGCATTCCCACG
AATAAATAATATAAGATTTTGATTATTACCCCCCTCTTTAACTTTACTAATTTCAAGTAGAATTGTAGAAAATGGGGCAG
GAACTGGATGAACAGTGTACCCCCCACTTTCATCTAATATTGCCCATGGAGGAAGATCAGTGGACTTAGCTATTTTTTCT
CTTCATTTAGCAGGTATTTCATCTATTTTAGGGGCTATTAATTTTATCACAACAATTATTAATATACGATTAAATAACTT

ATCCTTTGATCAAATACCTTTATTTATTTGAGCTGTTGGAATTACTGCATTTTTATTATTATTATCTTTACCTGTTTTAG
CAGGAGCTATTACAATATTATTAACCGATCGAAACTTAAATACATCATTTTTCGATCCGGCAGGAGGAGGCGATCCTATT
TTATACCAACATTTATTTTGATTTTTT
>LSTEM179-18|Scirpophaga_excerptalis|am12569|
AACATTATATTTTATTTTTGGAATTTGAGCTGGTATAGTGGGGACTTCACTTAGTTTACTAATTCGAGCCGAACTAGGTA
CTCCGGGATCATTAATCGGAGATGATCAAATCTATAATACTATTGTAACTGCTCACGCTTTTATTATAATTTTTTTTATG
GTTATGCCCATTATAATTGGGGGATTCGGAAACTGATTAGTGCCTTTAATATTGGGAGCCCCAGATATGGCCTTCCCCCG
AATAAATAATATAAGTTTTTGATTATTACCCCCTTCTTTAACCCTCTTAATCTCAAGAAGAGTCGTTGAAAATGGAGCTG
GAACAGGATGAACTGTTTATCCGCCCTTATCCTCCAATATTGCTCATAGTGGGACTTCTGTAGATTTAGCCATTTTTTCA
TTACATTTAGCTGGAATTTCTTCTATTCTAGGGGCTATTAACTTCATTACAACTATTATTAATATACGAATTAATGGACT
ATCTTTTGATCAAATACCTTTATTTGTATGAGCAGTTGGTATTACTGCCCTTCTTCTTCTTCTCTCACTACCTGTATTAG
CGGGAGCTATTACTATATTATTAACAGATCGAAACTTAAATACTTCTTTCTTTGACCCAGCAGGAGGTGGAGACCCAATT
CTTTATCAACACTTATTTTGATTCTTT
>LSTEM180-18|Scirpophaga|am12570|
GACATTATATTTTATTTTTGGAATTTGAGCTGGTATAGTGGGGACTTCACTTAGTTTACTAATTCGAGCCGAACTAGGTA
CTCCAGGATCATTAATCGGAGATGATCAAATCTATAATACTATTGTAACTGCTCACGCTTTTATTATAATTTTTTTTATG
GTTATACCCATTATAATTGGGGGATTCGGAAACTGATTAGTGCCTTTAATGTTGGGGGCCCCAGATATGGCCTTCCCCCG
AATAAATAATATAAGTTTTTGATTATTACCCCCCTCTTTAACCCTCTTAATCTCAAGAAGAGTCGTTGAAAATGGAGCTG
GAACAGGATGAACTGTTTATCCGCCCTTATCCTCCAATATTGCTCATGGTGGGACTTCTGTAGATTTAGCCATTTTTTCA
TTACATTTAGCTGGAATTTCTTCTATTCTAGGGGCTATTAACTTCATTACAACTATTATTAATATACGAATTAATGGACT
ATCTTTTGATCAAATACCTTTATTTGTATGAGCAGTTGGTATTACTGCCCTTCTTCTTCTTCTCTCACTACCTGTATTAG
CAGGAGCTATTACTATATTATTAACAGATCGAAACTTAAATACTTCTTTCTTTGACCCAGCAGGAGGTGGAGATCCAATT
CTTTATCAACACTTATTTTGATTCTTT
>LSTEM181-18|Chilo_aff._terrenellus_louisiadalis|am12571|
AACTTTATATTTTATTTTTGGTATTTGAGCTGGAATAATTGGAACATCCCTTAGAATTTTAATTCGTGCTGAATTAGGTA
CTCCAGGATCCTTAATTGGTGACGATCAAATTTATAATACCATTGTAACAGCTCATGCATTTATTATAATTTTTTTTATA
GTAATACCAATTATAATTGGAGGATTTGGAAATTGATTAGTGCCATTAATATTAGGTGCCCCAGATATGGCTTTCCCCCG
AATAAATAATATAAGATTTTGAATATTACCCCCCTCATTAACCTTATTAATTTCTAGAAGAATTGTTGAAAATGGAGCTG
GAACAGGATGAACAGTATACCCCCCACTTTCATCTAATATTGCTCATGCTGGAAGTTCAGTAGATTTAGCAATTTTTTCA
CTACATTTAGCCGGTATTTCTTCAATTTTAGGAGCTATTAATTTTATTACAACAATTATTAATATACGTATTAATAAATT
ATCATTTGATCAAATACCTTTATTTGTTTGATCTGTAGGTATTACTGCATTACTTTTATTACTTTCATTACCTGTTTTAG
CTGGAGCTATTACTATACTACTAACGGATCGAAATTTAAATACATCTTTTTTTGACCCTGCTGGAGGAGGGGATCCTATT
CTTTACCAACATTTATTTTGATTTTTT
>LSTEM182-18|Crambidae|am12572|
AACTTTATATTTTATTTTTGGAATTTGAAGAGGAATAGTAGGAACTTCTCTAAGTTTATTAATTCGAGCTGAATTAGGTA
ATCCTGGATCATTAATTGGAGATGATCAAATTTATAATACAATTGTAACAGCTCATGCATTTATTATAATTTTTTTTATA
GTAATACCTATTATAATTGGTGGTTTTGGAAATTGATTAGTACCTCTAATATTAGGAGCTCCTGATATAGCATTCCCACG
AATAAATAATATAAGATTTTGATTATTACCCCCATCATTAACCCTTTTAATTTCAAGAAGAATCGTTGAAAATGGAGCAG
GAACTGGTTGAACTGTTTACCCCCCACTTTCATCTAATATTGCTCATGGAGGTAGATCTGTAGATCTAGCTATTTTTTCA
TTACATTTAGCTGGTATTTCTTCAATTTTAGGAGCAATTAACTTCATTACAACAATTATCAATATACGAATTAATGGAAT
ATCTTTTGATCAAATACCATTATTTGTATGATCTGTAGGAATTACAGCATTATTATTATTATTATCACTTCCTGTTTTAG
CTGGGGCTATCACTATACTATTAACAGATCGAAATTTAAATACATCATTTTTTGATCCTGCTGGAGGAGGAGACCCTATT
TTATATCAACATTTATTTTGATTTTTT
>LSTEM183-18|Sesamia_grisescens|am12575|
AACATTATATTTTATTTTCGGAATTTGAGCTGGTATAGTTGGAACATCACTAAGATTATTAATTCGAGCCGAACTAGGAA
CCCCTGGTTCTTTAATTGGAGACGATCAAATTTATAATACTATTGTTACAGCTCATGCTTTCATTATAATTTTCTTTATA
GTTATACCAATTATAATTGGTGGATTTGGGAATTGACTCGTACCTTTAATATTAGGAGCCCCTGATATAGCATTTCCACG
AATAAATAATATAAGATTTTGATTATTACCCCCCTCTTTAACTTTACTAATTTCAAGTAGAATTGTAGAAAATGGGGCAG
GAACTGGGTGAACAGTGTACCCCCCACTTTCATCTAATATTGCCCATGGAGGAAGATCAGTGGACTTAGCTATTTTTTCC
CTTCATTTAGCGGGTATTTCATCTATTTTAGGAGCTATTAATTTTATCACAACAATTATTAATATACGATTAAATAACTT
ATCCTTTGATCAAATACCTTTATTTATTTGAGCTGTTGGAATTACTGCATTTTTATTATTATTATCTTTACCTGTTTTAG
CGGGAGCTATTACAATATTATTAACCGATCGAAACTTAAATACATCATTTTTCGATCCGGCAGGAGGAGGCGATCCTATT
TTATACCAACATTTATTTTGATTTTTT
>LSTEM184-18|Sesamia_grisescens|am12576|
AACATTATATTTTATTTTCGGAATTTGAGCTGGTATAGTTGGAACATCACTAAGATTATTAATTCGAGCCGAACTAGGAA
CCCCTGGTTCTTTAATTGGAGACGATCAAATTTATAATACTATTGTTACAGCTCATGCTTTCATTATAATTTTCTTTATA
GTTATACCAATTATAATTGGTGGATTTGGGAATTGACTCGTACCTTTAATATTAGGAGCCCCTGATATAGCATTTCCACG

AATAAATAATATAAGATTTTGATTATTACCCCCCTCTTTAACTTTACTAATTTCAAGTAGAATTGTAGAAAATGGGGCAG
GAACTGGGTGAACAGTGTACCCCCCACTTTCATCTAATATTGCCCATGGAGGAAGATCAGTGGACTTAGCTATTTTTTCC
CTTCATTTAGCGGGTATTTCATCTATTTTAGGAGCTATTAATTTTATCACAACAATTATTAATATACGATTAAATAACTT
ATCCTTTGATCAAATACCTTTATTTATTTGAGCTGTTGGAATTACTGCATTTTTATTATTATTATCTTTACCTGTTTTAG
CAGGAGCTATTACAATATTATTAACCGATCGAAACTTAAATACATCATTTTTCGATCCGGCAGGAGGAGGCGATCCTATT
TTATACCAACATTTATTTTGATTTTTT
>LSTEM185-18|Sesamia_grisescens|am12577|
AACATTATATTTTATTTTTGGAATTTGAGCTGGTATAGTTGGAACATCACTAAGATTATTAATTCGAGCCGAACTAGGAA
CCCCTGGTTCTTTAATTGGAGACGATCAAATTTATAATACTATTGTTACAGCTCATGCTTTCATTATAATTTTCTTTATA
GTTATACCAATTATAATTGGTGGATTTGGGAATTGACTCGTACCTTTAATATTAGGAGCCCCTGATATAGCATTTCCACG
AATAAATAATATAAGATTTTGATTATTACCCCCCTCTTTAACTTTACTAATTTCAAGTAGAATTGTAGAAAATGGGGCAG
GAACTGGGTGAACAGTGTACCCCCCACTTTCATCTAATATTGCCCATGGAGGAAGATCAGTGGACTTAGCTATTTTTTCC
CTTCATTTAGCAGGTATTTCATCTATTTTAGGAGCTATTAATTTTATCACAACAATTATTAATATACGATTAAATAACTT
ATCCTTTGATCAAATACCTTTATTTATTTGAGCTGTTGGAATTACTGCATTTTTATTATTATTATCTTTACCTGTTTTAG
CAGGAGCTATTACAATATTATTAACCGATCGAAACTTAAATACATCATTTTTCGATCCGGCAGGAGGAGGCGATCCTATT
TTATACCAACATTTATTTTGATTTTTT
>LSTEM186-18|Sesamia_grisescens|am12578|
AACATTATATTTTATTTTCGGAATTTGAGCTGGTATAGTTGGAACATCACTAAGATTATTAATTCGAGCCGAACTAGGGA
CCCCTGGTTCTTTAATTGGAGACGATCAAATTTATAATACTATTGTTACAGCTCATGCTTTCATTATAATTTTCTTTATA
GTTATACCAATTATAATTGGTGGATTTGGAAATTGACTCGTACCTTTAATATTAGGAGCCCCTGATATAGCATTTCCACG
AATAAATAATATAAGATTTTGATTATTACCCCCCTCTTTAACTTTACTAATTTCAAGTAGAATTGTAGAAAATGGGGCAG
GAACTGGGTGAACAGTGTACCCCCCACTTTCATCTAATATTGCCCATGGAGGAAGATCAGTGGACTTAGCTATTTTTTCC
CTTCATTTAGCAGGTATTTCATCTATTTTAGGAGCTATTAATTTTATCACAACAATTATTAATATACGATTAAATAACTT
ATCCTTTGATCAAATACCTTTATTTATTTGAGCTGTTGGAATTACTGCATTTTTATTATTATTATCTTTACCTGTTTTAG
CAGGAGCTATTACAATATTATTAACCGATCGAAACTTAAATACATCATTTTTCGATCCGGCAGGAGGAGGCGATCCTATT
TTATACCAACATTTATTTTGATTTTT-
>LSTEM187-18|Sesamia_grisescens|am12579|
AACATTATATTTTATTTTCGGAATTTGAGCTGGTATAGTTGGAACATCACTAAGATTATTAATTCGAGCCGAACTAGGAA
CCCCTGGTTCTTTAATTGGAGACGATCAAATTTATAATACTATTGTTACAGCTCATGCTTTCATTATAATTTTCTTTATA
GTTATACCAATTATAATTGGTGGATTTGGGAATTGACTCGTACCTTTAATATTAGGAGCCCCTGATATAGCATTTCCACG
AATAAATAATATAAGATTTTGATTATTACCCCCCTCTTTAACTTTACTAATTTCAAGTAGAATTGTAGAAAATGGGGCAG
GAACTGGGTGAACAGTGTACCCCCCACTTTCATCTAATATTGCCCATGGAGGAAGATCAGTGGACTTAGCTATTTTTTCC
CTTCATTTAGCGGGTATTTCATCTATTTTAGGAGCTATTAATTTTATCACAACAATTATTAATATACGATTAAATAACTT
ATCCTTTGATCAAATACCTTTATTTATTTGAGCTGTTGGAATTACTGCATTTTTATTATTATTATCTTTACCTGTTTTAG
CAGGAGCTATTACAATATTATTAACCGATCGAAACTTAAATACATCATTTTTC---------------------------
----
>LSTEM188-18|Chilo_crypsimetalla|DMM079|
AACTTTATATTTTATTTTTGGAATCTGAGCAGGAATAATTGGAACATCTCTTAGTCTTTTAATTCGAGCTGAATTAGGTA
CCCCTGGATCTTTAATTGGAGATGATCAAATTTATAATACTATTGTCACTGCTCATGCCTTTATTATAATTTTTTTTATA
GTAATACCAATTATAATTGGAGGATTCGGAAATTGATTGGTTCCTTTAATATTAGGAGCTCCAGATATAGCTTTCCCACG
AATAAACAATATAAGATTTTGATTATTACCCCCCTCTTTAACTTTATTAATTTCTAGAAGAATTGTTGAAAATGGTGCTG
GAACAGGATGAACAGTATACCCCCCACTTTCATCTAATATTGCTCATGCTGGGAGATCAGTAGATTTAGCAATTTTTTCC
TTACACTTAGCAGGAATCTCTTCTATTTTAGGAGCTATTAATTTTATTACAACAATTATTAATATACGAATTAATGGATT
ATCATTCGATCAAATACCATTATTTGTGTGATCTGTCGGTATTACAGCATTATTATTATTACTTTCCTTACCTGTTTTAG
CTGGAGCTATTACTATATTGTTAACTGATCGAAATTTAAATACATCTTTTTTTGATCCTGCTGGAGGTG-----------
----
>LSTEM189-18|Bathytricha_sp.|GlatzKI035|
-ACATTATATTTTATTTTTGGAATTTGAGCTGGAATAGTAGGAACCTCTTTAAGACTACTAATTCGAGCTGAATTAGGAA
CTCCCGGATCTCTAATTGGAGATGATCAAATTTATAATACTATTGTAACAGCTCATGCTTTTATTATAATTTTTTTCATA
GTTATACCAATCATAATTGGAGGATTTGGAAATTGACTTGTACCTTTAATATTAGGAGCACCTGATATAGCATTCCCACG
AATAAATAACATAAGTTTTTGACTACTTCCACCCTCTTTAACTCTTCTCATTTCAAGTAGAATTGTAGAAAATGGAGCAG
GAACTGGATGAACAGTTTACCCACCACTCTCATCTAATATTGCTCATGGTGGAAGATCTGTAGACTTAGCTATTTTTTCC
CTCCATTTAGCGGGAATCTCTTCCATTCTAGGAGCTATTAATTTTATTACAACTATTATTAATATACGATTAAATAGATT
ATCTTTTGATCAAATACCTTTATTTATTTGAGCTGTAGGAATTACAGCATTTTTATTATTATTATCATTACCTGTATTAG
CTGGAGCTATTACTATATTACTAACAGATCGAAATTTAAATACATCATTTTTCGATCCCGCAGGAGGAGGAGATCCAATT
TTATATCAACATTTATTTTGATTTTTT
>LSTEM190-18|Chilo_crypsimetalla|JW023|

AACTTTATATTTTATTTTTGGAATCTGAGCAGGAATAATTGGAACATCTCTTAGTCTTTTAATTCGAGCTGAATTAGGTA
CCCCTGGATCTTTAATTGGAGATGATCAAATTTATAATACTATTGTCACTGCTCATGCCTTTATTATAATTTTTTTTATA
GTAATACCAATTATAATTGGAGGATTCGGAAATTGATTGGTTCCTTTAATATTAGGAGCTCCAGATATAGCTTTCCCACG
AATAAACAATATAAGATTTTGATTATTACCCCCCTCTTTAACTTTATTAATTTCTAGAAGAATTGTTGAAAATGGTGCTG
GAACAGGATGAACAGTATACCCCCCACTTTCATCTAATATTGCTCATGCTGGAAGATCAGTAGATTTAGCAATTTTTTCC
TTACACTTAGCAGGAATCTCTTCTATTTTAGGAGCTATTAATTTTATTACAACAATTATTAATATACGAATTAATGGATT
ATCATTCGATCAAATACCATTATTTGTGTGATCTGTCGGTATTACAGCATTATTATTATTACTTTCCTTACCTGTTTTAG
CTGGAGCTATTACTATATTGTTAACTGATCGAAATTTAAATACATCTTTTTTTGATCCTGCTGGAGGAGGTGATCCAATC
CTTTATCAACATTTATTTTGATTTTT-
>LSTEM191-18|Bathytricha_sp.|K365807|
AACATTATATTTCATCTTTGGAATTTGAGCAGGAATAGTTGGAACTTCTTTAAGACTATTAATTCGAGCTGAATTAGGAA
CTCCTGGATCCTTAATTGGGGATGATCAAATTTATAATACTATTGTAACAGCTCATGCCTTTATCATAATTTTTTTTATG
GTTATACCAATTATAATTGGGGGATTTGGAAATTGACTTGTACCTTTAATATTAGGAGCACCTGATATGGCATTTCCACG
AATAAATAATATAAGTTTTTGATTACTACCCCCTTCTTTAACTCTACTTATTTCAAGAAGAGTTGTAGAAAATGGAGCAG
GAACTGGATGAACAGTATACCCCCCACTTTCATCTAATATTGCTCATGGAGGAAGATCTGTAGACTTAGCTATTTTTTCC
CTTCATTTAGCTGGAATTTCTTCTATTTTAGGAGCTATTAATTTTATTACAACTATTATTAATATACGATTAAATAGTTT
ATCTTTTGATCAAATACCTTTATTTATTTGAGCTGTAGGAATTACAGCATTTTTATTATTATTATCATTACCTGTATTAG
CTGGAGCTATTACTATATTATTAACAGATCGAAATTTAAATACATCATTTTTTGATCCTGCAGGAGGAGGAGACCCAATC
TTATACCAACATTTATTTTGATTTTTT
>LSTEM192-18|Scirpophaga|LTN-P11|
TACGTTATATTTTATTTTTGGAATTTGAGCTGGTATAGTAGGAACTTCTTTAAGCTTACTTATTCGAGCTGAATTAGGAA
CTTCTGGATCCTTAATTGGAGATGATCAAATCTATAACACTATTGTCACAGCCCATGCCTTTATTATAATTTTTTTTATA
GTTATACCCATTATAATTGGAGGATTTGGAAATTGATTAGTCCCCCTAATATTAGGAGCCCCAGATATAGCTTTCCCCCG
AATAAATAACATAAGATTCTGATTATTACCCCCCTCTTTAACACTCCTCATTTCTAGAAGAATTGTAGAAAATGGGGCTG
GAACAGGRTGAACTGTTTACCCACCCCTATCATCCAATATTGCTCATGGGGGAACATCAGTAGATTTAGCTATTTTTTCT
CTACACCTAGCAGGAATTTCATCTATTTTAGGAGCTATTAATTTTATTACAACCATTATTAATATACGAATTAATGGATT
ATCATTTGACCAAATACCTCTATTTGTGTGAGCTGTTGGTATTACAGCCCTTCTTTTACTTCTCTCTCTCCCAGTTTTAG
CTGGAGCTATTACCATATTACTAACAGATCGAAATTTAAATACATCTTTTTTTGACCCAGCTGGAGGAGGAGATCCAATT
TTATAT---------------------
>LSTEM193-18|Scirpophaga|LTN-P12|
TACTTTATATTTTATTTTTGGAATTTGAGCTGGTATAGTAGGAACTTCTTTAAGCTTACTTATTCGAGCTGAATTAGGAA
CTTCTGGATCCTTAATTGGAGATGATCAAATCTATAATACTATTGTCACAGCCCATGCCTTTATTATAATTTTTTTTATA
GTTATACCCATTATAATTGGAGGATTTGGAAATTGATTAGTCCCACTAATATTAGGAGCTCCAGATATAGCTTTTCCCCG
ACTAAATAATATAAGATTCTGATTATTACCCCCCTCTTTAACACTTCTCATTTCTAGAAGAATTGTAGAAAGTGGAGCTG
GAACAGGATGAACTGTTTACCCACCTCTATCATCCAATATTGCTCATGGAGGAACATCAGTAGATTTAGCTATTTTTTCT
CTACACCTAGCAGGAATTTCATCTATTTTAGGAGCTATTAATTTTATTACAACCATTATTAATATACGAATTAATGGATT
ATCATTTGACCAAATACCTCTATTTGTGTGAGCTGTTGGTATTACAGCCCTTCTTTTACTTCTCTCTCTCCCAGTTTTAG
CTGGAGCTATTACCATATTACTAACAGATCGAAATTTAAATACATCTTTTTTTGACCCAGCTGGGGGAGGAGATCCAATT
TTATAC---------------------
>LSTEM194-18|Scirpophaga|LTN-S1|
AACTTTATATTTTATTTTTGGAATTTGAGCTGGTATAGTAGGAACTTCTTTAAGATTATTAATTCGAGCTGAATTAGGAA
CTCCAGGATCTTTAATTGGAGATGATCAAATTTATAATACCATTGTTACAGCTCATGCTTTTATTATAATTTTTTTTATA
GTAATACCAATTATAATTGGAGGGTTTGGAAATTGACTTGTTCCTTTAATATTAGGAGCTCCTGATATAGCTTTCCCTCG
TATAAATAATATAAGATTTTGATTATTACCCCCCTCATTAACTCTCCTAATTTCAAGAAGAATTGTAGAAAATGGTGCAG
GAACAGGATGAACAGTATACCCCCCTTTATCATCAAATATTGCTCATGGAGGAACTTCTGTAGATTTAGCTATTTTTTCT
TTACATCTTGCAGGAATTTCCTCTATTTTAGGAGCTATTAACTTTATTACCACTATTATTAATATACGAATTAATGGCTT
AACATTTGATCAAATACCTCTCTTTGTTTGAGCTGTTGGAATTACAGCCCTTCTTTTACTCCTCTCATTACCCGTATTAG
CTGGGGCTATTACTATATTATTAACTGATCGAAATTTAAATACCTCTTTTTTTGATCCAGCGGGAGGAGGAGATCCAATC
CTTTAT---------------------
>LSTEM195-18|Scirpophaga|LTN-S2|
AACTTTATATTTTATTTTTGGAATTTGAGCTGGTATAGTAGGAACTTCTTTAAGTTTATTAATTCGAGCTGAATTAGGAA
CTCCAGGATCTTTAATTGGAGATGATCAAATTTATAAYACCATTGTTACAGCTCATGCTTTYATTATAATTTTTTTTATA
GTAATACCAATTATAATTGGAGGGTTTGGAAATTGACTTGTTCCTTTCATATTAGGAGCTCCTGATATAGCTTTCCCTCG
TATAAATAATATAAGATTTTGATTTCTACCCCCCTCATTAACTCTCCTAATTTCAAGAAGAATTGTAGAAAATGAGGCAG
GAACAGGATGAACAGTTTTCCCCCCTTATTCATCAAATATGGTTCATGGAGGAACTTCAGTAGATTTAGCTATTTTTTCT
TTACATCTAGCRGGAATTTCCTCTATTTTAGGAGCTATTAAYTTYATTACCACTATTATTAATATAARAWTWAATGGATT
AWCATTTGATCAAATACCTTTMTTTGTTTGAGCTGTNGGAATTACAGCCTTWTTATTTCTCCTATCWTTACCAGTTTTAG

CTGGGGCTATTACTATATTATTAACAGATCGAAACTTAAATACCT-----------------------------------
----
>LSTEM196-18|Scirpophaga_nivella|LTN-S3|
AACTTTATATTTTATTTTTGGAATTTGAGCTGGTATAGTAGGAACTTCTTTAAGATTATTAATTCGAGCTGAATTAGGAA
CTCCAGGATCTTTAATTGGAGATGATCAAATTTATAATACCATTGTTACAGCTCATGCTTTTATTATAATTTTTTTTATA
GTAATACCAATTATAATTGGAGGGTTTGGAAATTGACTTGTTCCTTTAATATTAGGAGCTCCTGATATAGCTTTCCCTCG
TATAAATAATATAAGATTTTGATTATTACCCCCCTCATTAACTCTCCTAATTTCAAGAAGAATTGTAGAAAATGGTGCAG
GAACAGGATGAACAGTATACCCCCCTTTATCATCAAATATTGCTCATGGAGGAACTTCTGTAGATTTAGCTATTTTTTCT
TTACATCTTGCAGGAATTTCCTCTATTTTAGGAGCTATTAACTTTATTACCACTATTATTAATATACGAATTAATGGATT
AACATTTGATCAAATACCTCTCTTTGTTTGAGCTGTTGGAATTACAGCCCTTCTTTTACTCCTCTCATTACCCGTATTAG
CTGGGGCTATTACTATATTATTAACTGATCGAAATTTAAATACC-----------------------------------
----
>LSTEM197-18|Scirpophaga_nivella|LTN-S7|
AACTTTATATTTCATTTTTGGAATTTGAGCTGGTATAGTAGGAACTTCTTTAAGATTATTAATTCGAGCTGAATTAGGAA
CTCCAGGATCTTTAATTGGAGATGATCAAATTTATAATACCATTGTTACAGCTCATGCTTTTATTATAATTTTTTTTATA
GTAATACCAATTATAATTGGAGGGTTTGGAAATTGACTTGTTCCTTTAATATTAGGAGCTCCTGATATAGCTTTCCCTCG
TATAAATAATATAAGATTTTGATTATTACCCCCCTCATTAACTCTCCTAATTTCAAGAAGAATTGTAGAAAATGGTGCAG
GAACAGGATGAACAGTATACCCCCCCTTATCATCAAATATTGCTCATGGAGGAACTTCTGTAGATTTAGCTATTTTTTCT
CTACATCTTGCAGGAATTTCCTCTATTTTAGGAGCTATTAACTTTATTACCACTATTATTAATATACGAATTAATGGCTT
AACATTTGATCAAATACCTCTCTTTGTTTGAGCTGTTGGAATTACAGCCCTTCTTTTACTCCTCTCATTACCCGTATTAG
CTGGAGCTATTACTATATTATTAACTGATCGAAATTTAAATACCTCTTTTTTTGATCCAGCGGGAGGAGGGGATCCAATC
CTTTAT---------------------
>LSTEM198-18|Scirpophaga_nivella|LTN-S8|
AACTTTATATTTCATTTTTGGAATTTGAGCTGGTATAGTAGGAACTTCTTTAAGATTATTAATTCGAGCTGAATTAGGAA
CTCCAGGATCTTTAATTGGAGATGATCAAATTTATAATACCATTGTTACAGCTCATGCTTTTATTATAATTTTTTTTATA
GTAATACCAATTATAATTGGAGGGTTTGGAAATTGACTTGTTCCTTTAATATTAGGAGCTCCTGATATAGCTTTCCCTCG
TATAAATAATATAAGATTTTGATTATTACCCCCCTCATTAACTCTCCTAATTTCAAGAAGAATTGTAGAAAATGGTGCAG
GAACAGGATGAACAGTATACCCCCCTTTATCATCAAATATTGCTCATGGAGGAACTTCTGTAGATTTAGCTATTTTTTCT
TTACATCTTGCAGGAATTTCCTCTATTTTAGGAGCTATTAACTTTATTACCACTATTATTAATATACGAATTAATGGCTT
AACATTTGATCAAATACCTCTCTTTGTTTGAGCTGTTGGAATTACAGCCCTTCTTTTACTCCTCTCATTACCCGTATTAG
CTGGGGCTATTACTATATTATTAACTGATCGAAATTTAAATACCTCTTTTTTTGATCCAGGGGGAGGAGGAGATCCAATC
CTTTAT---------------------
>LSTEM199-18|Scirpophaga_nivella|LTN-S9|
AACTTTATATTTCATTTTTGGAATTTGAGCTGGTATAGTAGGAACTTCTTTAAGATTATTAATTCGAGCTGAATTAGGAA
CTCCAGGATCTTTAATTGGAGATGATCAAATTTATAATACCATTGTTACAGCTCATGCTTTTATTATAATTTTTTTTATA
GTAATACCAATTATAATTGGAGGGTTTGGAAATTGACTTGTTCCTTTAATATTAGGAGCTCCTGATATAGCTTTCCCTCG
TATAAATAATATAAGATTTTGATTATTACCCCCCTCATTAACTCTCCTAATTTCAAGAAGAATTGTAGAAAATGGTGCAG
GAACAGGATGAACAGTATACCCCCCTTTATCATCAAATATTGCTCATGGAGGAACTTCTGTAGATTTAGCTATTTTTTCT
TTACATCTTGCAGGAATTTCCTCTATTTTAGGAGCTATTAACTTTATTACCACTATTATTAATATACGAATTAATGGCTT
AACATTTGATCAAATACCTCTCTTTGTTTGAGCTGTTGGAATTACAGCCCTTCTTTTACTCCTCTCATTACCCGTATTAG
CTGGGGCTATTACTATATTATTAACTGATCGAAATTTAAATACCTCTTTTTTTGATCCAGCGGGAGGAGGAGATCCAATC
CTTTAT---------------------
>LSTEM200-18|Chilo_crypsimetalla|SAC342|
AACTTTATATTTTATTTTTGGAATCTGAGCAGGAATAATTGGAACATCTCTTAGTCTTTTAATTCGAGCTGAATTAGGTA
CCCCTGGATCTTTAATTGGAGATGATCAAATTTATAATACTATTGTCACTGCTCATGCCTTTATTATAATTTTTTTTATA
GTAATACCAATTATAATTGGAGGATTCGGAAATTGATTGGTTCCTTTAATATTAGGAGCTCCAGATATAGCTTTCCCACG
AATAAACAATATAAGATTTTGATTATTACCCCCCTCTTTAACTTTATTAATTTCTAGAAGAATTGTTGAAAATGGTGCTG
GAACAGGATGAACAGTATACCCCCCACTTTCATCTAATATTGCTCATGCTGGAAGATCAGTAGATTTAGCAATTTTTTCC
TTACACTTAGCAGGAATCTCTTCTATTTTAGGAGCTATTAATTTTATTACAACAATTATTAATATACGAATTAATGGATT
ATCATTCGATCAAATACCATTATTTGTATGATCTGTCGGTATTACAGCATTATTATTATTACTTTCCTTACCTGTTTTAG
CTGGAGCTATTACTATATTGTTAACTGATCGAAATTTAAATACATCTTTTTTTGATCCTGCTGGAGGAGGTGATCCAATC
CTTTATCAACATTTATTTTGATTTTT-
>LSTEM201-18|Chilo_aff._crypisimetalla|SJA00338ak|
-------TACCTTTATTTTGGAGTTTGAGCAGGTATAATCGGAACATCTCTTAGTCTTTTAATTCGTGCTGAATTAGGAA
CACCAGGATCATTAATTGGAGATGATCAAATTTATAATACTATTGTAACAGCTCATGCATTTATTATAATTTTTTTTATA
GTTATACCAATCATAATTGGAGGATTCGGAAATTGATTAGTTCCTTTAATATTAGGAGCCCCAGATATAGCATTTCCTCG
AATAAATAATATAAGATTTTGATTACTACCTCCTTCATTAACATTATTAATTTCAAGAAGAATTGTTGAAAATGGAGCCG

GAACAGGATGAACAGTATACCCCCCACTTTCATCCAATATTGCTCATGCTGGAAGTTCAGTAGATCTAGCAATTTTTTCC
TTGCATTTAGCTGGAATTTCCTCAATTTTAGGTGCAATTAACTTTATTACAACAATTATTAATATACGAATTAATAAACT
ATCATTTGACCAATTACCATTATTTGTTTGATCTGTAGGCATTACAGCCTTATTATTACTTCTATCTCTTCCTGTATTAG
CTGGAGCTATTACTATACTTCTAACTGATCGAAATTTAAATACATCTTTTTTTGATCCTGCCGGAGGAGGAGATCCAATT
CTTTACCAACATTTATTTTGATTTTTT
>LSTEM202-18|Crambinae|SJA041605b|
TACCTTATATTTTATTTTTGGAATTTGAGCTGGTATAGTGGGAACTTCCTTAAGTCTTCTTATTCGAGCTGAATTAGGAA
CTCCCGGATCTTTAATTGGAGATGATCAAATTTATAATACTATTGTTACTGCTCATGCTTTTATTATAATTTTCTTTATA
GTTATACCTATTATAATTGGGGGATTTGGAAATTGATTGGTTCCTTTAATATTAGGGGCACCTGATATAGCCTTCCCTCG
AATAAATAATATAAGATTTTGATTATTACCCCCCTCTTTAACTCTCCTTATTTCAAGAAGAATTGTAGAAAATGGGGCTG
GAACAGGGTGAACTGTTTATCCCCCCTTATCCTCTAATATTGCTCACGGAGGAACATCTGTTGATTTAGCTATTTTTTCT
TTACATTTAGCTGGAATTTCTTCAATCTTAGGGGCTATTAATTTTATTACAACTATTATTAATATACGAGTTAATGGATT
ATCATTTGATCAAATACCTTTATTTGTGTGAGCTGTAGGTATTACAGCTCTTTTATTACTTCTATCTCTCCCAGTATTAG
CTGGAGCTATTACTATATTATTAACTGATCGAAATCTAAATACATCTTTTTTCGATCCAGCTGGAGGGGGGGACCCAATT
TTATAT---------------------
>LSTEM203-18|Crambidae|SJA041607|
TACCTTATATTTTATTTTTGGAATTTGAGCTGGTATAGTGGGAACTTCCTTAAGTCTTCTTATTCGAGCTGAATTAGGAA
CTCCCGGATCTTTAATTGGAGATGATCAAATTTATAATACTATTGTTACTGCTCATGCTTTTATTATAATTTTCTTTATA
GTTATACCTATTATAATTGGGGGATTTGGAAATTGATTGGTTCCTTTAATATTAGGGGCACCTGATATAGCCTTCCCTCG
AATAAATAATATAAGATTTTGATTATTACCCCCCTCTTTAACTCTCCTTATTTCAAGAAGAATTGTAGAAAATGGGGCTG
GAACAGGGTGAACTGTTTATCCCCCCTTATCCTCTAATATTGCTCACGGAGGAACATCTGTTGATTTAGCTATTTTTTCT
TTACATTTAGCTGGAATTTCTTCAATCTTAGGGGCTATTAATTTTATTACAACTATTATTAATATACGAGTTAATGGATT
ATCATTTGATCAAATACCTTTATTTGTGTGAGCTGTAGGTATTACAGCTCTTTTATTACTTCTATCTCTCCCAGTATTAG
CTGGAGCTATTACTATATTATTAACTGATCGAAATCTAAATACATCTTTTTTCGATCCAGCTGGAGGGGGGGACCCAATT
TTATAT---------------------
>LSTEM204-18|Crambinae|SJA041626a|
TACCTTATATTTTATTTTTGGAATTTGAGCTGGTATAGTGGGAACTTCCTTAAGTCTTCTTATTCGAGCTGAATTAGGAA
CTCCCGGATCTTTAATTGGAGATGATCAAATTTATAATACTATTGTTACTGCTCATGCTTTTATTATAATTTTCTTTATA
GTTATACCTATTATAATTGGGGGATTTGGAAATTGATTGGTTCCTTTAATATTAGGGGCACCTGATATAGCCTTCCCTCG
AATAAATAATATAAGATTTTGATTATTACCCCCCTCTTTAACTCTCCTTATTTCAAGAAGAATTGTAGAAAATGGGGCTG
GAACAGGGTGAACTGTTTATCCCCCCTTATCCTCTAATATTGCTCACGGAGGAACATCTGTTGATTTAGCTATTTTTTCT
TTACATTTAGCTGGAATTTCTTCAATCTTAGGGGCTATTAATTTTATTACAACTATTATTAATATACGAGTTAATGGATT
ATCATTTGATCAAATACCTTTATTTGTGTGAGCTGTAGGTATTACAGCTCTTTTATTACTTCTATCTCTCCCAGTATTAG
CTGGAGCTATTACTATATTATTAACTGATCGAAATCTAAATACATCTTTTTTCGATCCAGCTGGAGGGGGGGACCCAATT
TTATAT---------------------
>LSTEM205-18|Chilo_auricilius|SJA-Ca1|
---ACATCTCTAAGACTTTTAATTCGTGCTGAATTAGGAA
CTCCAGGGTCATTAATTGGAGATGATCAAATTTACAATACTATTGTTACAGCTCATGCATTTATTATAATTTTTTTTATA
GTTATACCAATTATAATTGGAGGCTTTGGTAATTGATTAGTACCATTAATACTAGGGGCTCCTGATATAGCCTTCCCTCG
AATAAATAATATAAGATTTTGATTATTGCCCCCATCATTAACATTATTAATTTCTAGAAGAATTGTAGAAAATGGAGCTG
GAACAGGATGAACGGTTTACCCCCCCCTTTCATCAAATATTGCCCATGGAGGAAGTTCTGTAGATTTAGCTATTTTTTCT
CTTCATTTAGCTGGTATTTCCTCAATTTTAGGAGCTATTAATTTTATTACAACAATTATTAATATACGAATTAATAAACT
ATCATTTGATCAATTACCATTATTTGTTTGATCTGTTGGTATTACAGCTTTATTATTACTACTTTCATTGCCGGTACTAG
CTGGAGCTATTACTATACTTTTAACTGATCGAAACTTAAATACATCTTTTTTTGACCCTGCTGGACGAGGAGACCCAATT
CTTTATCAACATTTATTTTGATTTTTT
>LSTEM206-18|Chilo_auricilius|SJA-Ca2|
-------------------------------------ATAAGAACATCTCTAAGACTTTTAATTCGTGCTGAATTAGGAA
CTCCAGGGTCATTAATTGGAGATGATCAAATTTACAATACTATTGTTACAGCTCATGCATTTATTATAATTTTTTTTATA
GTTATACCAATTATAATTGGAGGCTTTGGTAATTGATTAGTACCATTAATACTAGGGGCTCCTGATATAGCCTTCCCTCG
AATAAATAATATAAGATTTTGATTATTGCCCCCATCATTAACATTATTAATTTCTAAAAAAATTGTAAAAAATGGAGCTG
GAACAGGATGAACGGTTTACCCCCCCCTTTCATCAAATATTGCCCATGGAGGAAGTTCTGTAAATTTAGCTATTTTTTCT
CTTCATTTAGCTGGTATTTCCTCAATTTTAGGAGCTATTAATTTTATTACAACAATTATTAATATACGAATTAATAAACT
ATCATTTGATCAATTACCATTATTTGTTTGATCTGTTGGTATTACAGCTTTATTATTACTACTTTCATTGCCGGTACTAG
CTGGAGCTATTACTATACTTTTAACTGATCGAAACTTAAATACATCTTTTTTTGACCCTGCTGGAGGAGGAAACCCAATT
CTTTATCAACATTTATTTTGATTTTTT
>LSTEM207-18|Chilo_infuscatellus|SJA-Ci13|
AACTTTATATTTTATTTTTGGAATTTGAGCAGGAATAATTGGAACCTCTCTTAGACTTTTAATTCGAGCTGAATTAGGAA

CTCCAGGATCTTTAATTGGAGATGATCAAATTTATAATACTATTGTTACAGCTCATGCATTTATTATAATTTTTTTTATA
GTAATACCAATTATAATCGGAGGATTTGGAAATTGATTAGTTCCTTTAATACTAGGAGCACCTGATATAGCTTTCCCACG
GATAAATAATATAAGTTTTTGATTATTACCACCATCACTAACATTATTGATTTCTAGAAGAATTGTTGAAAATGGAGCAG
GAACTGGTTGAACTGTTTATCCTCCTTTATCTTCAAATATTGCTCATGGGGGAAGCTCTGTAGATTTAGCARTTTTTTCC
CTTCATTTAGCTGGTATTTCATCAATTTTAGGAGCTATTAATTTTATTACAACAATTATTAATATACGAGTTAATGGTCT
ATCAKTTGATCAAATACCTTTATTTGTTTGATCTGTAGGAATTACAGCATTATTATTATTACTTTCTCTACCAGTATTAG
CAGGTGCTATTACTATACTACTAACTGATCGAAATTTAAATACATCTTTTTTTGATCCTGCAGGAGGGGGAGATCCAATC
CTCTAC---------------------
>LSTEM208-18|Chilo_infuscatellus|SJA-Ci16|
AACTTTATATTTTATTTTTGGAATTTGAGCAGGAATAATTGGAACCTCTCTTAGACTTTTAATTCGAGCTGAATTAGGAA
CTCCAGGATCTTTAATTGGAGATGATCAAATTTATAATACTATTGTTACAGCTCATGCATTTATTATAATTTTTTTTATA
GTAATACCAATTATAATCGGAGGATTTGGAAATTGATTAGTTCCTTTAATACTAGGAGCACCTGATATAGCTTTCCCACG
GATAAATAATATAAGTTTTTGATTATTACCACCATCACTAACATTATTGATTTCTAGAAGAATTGTTGAAAATGGAGCAG
GAACTGGTTGAACTGTTTATCCTCCTTTATCTTCAAATATTGCTCATGGGGGAAGCTCTGTAGATTTAGCAATTTTTTCC
CTTCATTTAGCTGGTATTTCATCAATTTTAGGAGCTATTAATTTTATTACAACAATTATTAATATACGAGTTAATGGTCT
ATCATTTGATCAAATACCTTTATTTGTCTGATCTGTAGGAATTACAGCATTATTATTATTACTTTCTCTACCAGTATTAG
CAGGTGCTATTACTATACTACTAACTGATCGAAATTTAAATACATCTTTTTTTGATCCTGCAGGAGGGGGAGATCCAATC
CTCTAC---------------------
>LSTEM209-18|Chilo_infuscatellus|SJA-Ci19|
AACTTTATATTTTATTTTTGGAATTTGAGCAGGAATAATTGGAACCTCTCTTAGACTTTTAATTCGAGCTGAATTAGGAA
CTCCAGGATCTTTAATTGGAGATGATCAAATTTATAATACTATTGTTACAGCTCATGCATTTATTATAATTTTTTTTATA
GTAATACCAATTATAATCGGAGGATTTGGAAATTGATTAGTTCCTTTAATACTAGGAGCACCTGATATAGCTTTCCCACG
GATAAATAATATAAGTTTTTGATTATTACCACCATCACTAACATTATTGATTTCTAGAAGAATTGTTGAAAATGGAGCAG
GAACTGGTTGAACTGTTTATCCTCCTTTATCTTCAAATATTGCTCATGGGGGAAGCTCTGTAGATTTAGCAATTTTTTCC
CTTCATTTAGCTGGTATTTCATCAATTTTAGGAGCTATTAATTTTATTACAACAATTATTAATATACGAGTTAATGGTCT
ATCATTTGATCAAATACCTTTATTTGTCTGATCTGTAGGAATTACAGCATTATTATTATTACTTTCTCTACCAGTATTAG
CAGGTGCTATTACTATACTACTAACTGATCGAAATTTAAATACATCTTTTTTTGATCCTGCAGGAGGGGGAGATCCAATC
CTCTAC---------------------
>LSTEM210-18|Chilo_terrenellus|SJA-Ci2|
AACTTTATATTTTATTTTTGGTATTTGAGCTGGAATAATTGGAACATCTCTTAGAATTTTAATTCGCGCTGAATTAGGTA
CTCCAGGATCCTTAATTGGTGATGATCAAATTTATAATACTATTGTAACAGCTCATGCATTTATTATAATTTTTTTTATA
GTAATACCAATTATAATTGGAGGATTTGGAAATTGATTAGTGCCATTAATATTAGGTGCCCCAGATATGGCCTTCCCCCG
AATAAATAATATAAGATTTTGAATATTACCCCCCTCATTAACTTTATTAATTTCTAGAAGAATTGTTGAAAATGGAGCTG
GAACAGGATGAACAGTATACCCCCCACTTTCATCTAATATTGCTCATGCTGGAAGTTCAGTAGATTTAGCAATTTTTTCA
CTACATTTAGCCGGTATTTCTTCAATTTTAGGAGCTATTAATTTTATTACAACAATTATTAATATACGTATTAATAAATT
ATCATTTGATCAAATACCTTTATTTGTTTGATCTGTAGGTATTACAGCATTACTTTTATTACTTTCATTACCTGTTTTAG
CTGGAGCTATTACTATACTACTAACAGATCGGAATTTAAATACATCTTTTTTTGACCCTGCCGGAGGAGGAGATCCTATT
CTTTACCAACATTTATTTTGATTCTAT
>LSTEM211-18|Chilo_infuscatellus|SJA-Ci21|
AACTTTATATTTTATTTTTGGAATTTGAGCAGGAATAATTGGAACCTCTCTTAGACTTTTAATTCGAGCTGAATTAGGAA
CTCCAGGATCTTTAATTGGAGATGATCAAATTTATAATACTATTGTTACAGCTCATGCATTTATTATAATTTTTTTTATA
GTAATACCAATTATAATCGGAGGATTTGGAAATTGATTAGTTCCTTTAATACTAGGAGCACCTGATATAGCTTTCCCACG
GATAAATAATATAAGTTTTTGATTATTACCACCATCACTAACATTATTGATTTCTAGAAGAATTGTTGAAAATGGAGCAG
GAACTGGTTGAACTGTTTATCCTCCTTTATCTTCAAATATTGCTCATGGGGGAAGCTCTGTAGATTTAGCAATTTTTTCC
CTTCATTTAGCTGGTATTTCATCAATTTTAGGAGCTATTAATTTTATTACAACAATTATTAATATACGAGTTAATGGTCT
ATCATTTGATCAAATACCTTTATTTGTCTGATCTGTAGGAATTACAGCATTATTATTATTACTTTCTCTACCAGTATTAG
CAGGTGCTATTACTATACTACTAACTGATCGAAATTTAAATACATCTTTTTTTGATCCTGCAGGAGGGGGAGATCCAATC
CTCTAC---------------------
>LSTEM212-18|Chilo_auricilius|SJA-Ci4|
AACTTTATACTTTATTTTTGGAATTTGAAGTGGAATAATTGGAACATCTCTAAGACTTTTAATTCGWGCTGAATTAGGAA
CTCCAGGGTCATTAATTGGAGATGATCAAATTTACAATACTATTGTTACAGCTCATGCATTTATTATAATTTTTTTTATA
GTTATACCAATTATAATTGGAGGCTTTGGTAATTGATTAGTACCATTAATACTAGGRGCTCCTGATATAGCCTTCCCTCG
AATAAATAATATAAGATTTTGATTATTGCCCCCATCATTAACATTATTAATTTCTAGAAGAATTGTAGAAAATGGAGCTG
GAACAGGATGAACGGTTTACCCCCCCCTTTCATCAAATATTGCCCATGGAGGAAGTTCTGTAGATTTAGCTAATTTTTCT
CTTCATTTAGCTGGTATTTCCTCAATTTTAGGAGCTATTAATTTTATTACAACAATTATTAATATACGAATTAATAAACT
ATCATTTGATCAATTACCATTATTTGTTTGATCTGTTGGTATTACAGCTTTATTATTACTACTTTCATTGCCGGTACTAG
CTGGAGCTATTACTATACTTTTAACTGATCGAAACTTAAATACATCTTTTTTTGACCCTGCTGGAGGAGGAGACCCAATT

CTTTAT---------------------
>LSTEM213-18|Chilo_terrenellus|SJA-Ci5|
AACTTTATATTTTATTTTTGGTATTTGAGCTGGAATAATTGGAACATCTCTTAGAATTTTAATTCGCGCTGAATTAGGTA
CTCCAGGATCCTTAATTGGTGATGATCAAATTTATAATACCATTGTAACAGCTCATGCATTTATTATAATTTTTTTTATA
GTAATACCAATTATAATTGGAGGATTTGGAAATTGATTAGTGCCATTAATATTAGGTGCCCCAGATATGGCCTTCCCCCG
AATAAATAATATAAGATTTTGAATATTACCCCCCTCATTAACTTTATTAATTTCTAGAAGAATTGTTGAAAATGGAGCTG
GAACAGGATGAACAGTATACCCCCCACTTTCATCTAATATTGCTCATGCTGGAAGTTCAGTAGATTTAGCAATTTTTTCA
CTACATTTAGCCGGTATTTCTTCAATTTTAGGAGCTATTAATTTTATTACAACAATTATTAATATACGTATTAATAAATT
ATCATTTGATCAAATACCTTTATTTGTTTGATCTGTAGGTATTACAGCATTACTTTTATTACTTTCATTACCTGTTTTAG
CTGGAGCTATTACTATACTACTAACAGATCGGAATTTAAATACATCTTTTTTGACCCTGCCGGAGGAGGAGATCCTATT
CTTTACCAACATTTATTTTGATTTTTT
>LSTEM214-18|Chilo_infuscatellus|SJA-Ci9|
AACTTTATATTTTATTTTTGGAATTTGAGCAGGAATAATTGGAACCTCTCTTAGACTTTTAATTCGAGCTGAATTAGGAA
CTCCAGGATCTTTAATTGGAGATGATCAAATTTATAATACTATTGTTACAGCTCATGCATTTATTATAATTTTTTTTATA
GTAATACCAATTATAATCGGAGGATTTGGAAATTGATTAGTTCCTTTAATACTAGGAGCACCTGATATAGCTTTCCCACG
GATAAATAATATAAGTTTTTGATTATTACCACCATCACTAACATTATTGATTTCTAGAAGAATTGTTGAAAATGGAGCAG
GAACTGGTTGAACTGTTTATCCTCCTTTATCTTCAAATATTGCTCATGGAGGAAGCTCTGTAGATTTAGCAATTTTTTCC
CTTCATTTAGCTGGTATTTCATCAATTTTAGGAGCTATTAATTTTATTACAACAATTATTAATATACGAGTTAATGGTCT
ATCATTTGATCAAATACCTTTATTTGTCTGATCTGTAGGAATTACAGCATTATTATTATTACTTTCTCTACCAGTATTAG
CAGGTGCTATTACTATACTACTAACTGATCGAAATTTAAATACATCTTTTTTGATCCTGCAGGAGGGGGAGATCCAATC
CTCTAC---------------------
>LSTEM215-18|Chilo_partellus|SJA-Cp1|
AACTTTATATTTTATTTTTGGAATTTGAGCAGGAATAATTGGGACATCCCTTAGATTATTAATTCGTGCAGAATTAGGAA
CTCCTGGATCTTTAATTGGAGATGATCAAATTTATAATACTATTGTAACAGCACACGCATTTATTATAATTTTTTTTATA
GTTATACCAATTATAATTGGTGGATTTGGAAATTGATTAGTACCTTTAATATTAGGAGCCCCAGATATAGCTTTCCCACG
AATAAATAATATAAGATTTTGATTATTACCACCATCATTAACTTTATTAATTTCTAGAAGAATTGTTGAAAATGGAGCTG
GAACAGGATGAACAGTGTACCCCCCACTATCATCTAATATTGCTCATGCCGGAAGTTCAGTAGATTTAGCAATTTTTTCT
TTACATTTAGCTGGTATTTCATCAATTCTCGGTGCTATTAATTTTATTACAACAATTATTAATATACGAATTAATGGATT
ATCTTTTGATCAAATACCATTATTTGTTTGATCTGTAGGTATTACAGCTTTATTATTATTACTTTCTTTACCTGTTTTAG
CTGGAGCTATTACTATATTATTAACAGATCGAAATTTAAATACATCCTTTTTCGATCCTGCTGGAGGAGGAGATCCTATT
CTTTATCAACACTTATTTTGATTTTTT
>LSTEM216-18|Chilo_partellus|SJA-Cp2|
AACTTTATATTTTATTTTGWGAATTTGAGCAGGAATAATTGGGACATCCCTTAGATTATTAATTCGTGCAGAATTAGGAA
CTCCTGGATCTTTAATTGGAGATGATCAAATTTATAATACTATTGTAACAGCACACGCATTTATTATAATTTTTTTTATA
GTTATACCAATTATAATTGGTGGATTTGGAAATTGATTAGTACCTTTAATATTAGGAGCCCCAGATATAGCTTTCCCACG
AATAAATAATATAAGATTTTGATTATTACCACCATCATTAACTTTATTAATTTCTAGAAGAATTGTTGAAAATGGAGCTG
GAACAGGATGAACAGTGTACCCCCCACTATCATCTAATATTGCTCATGCCGGAAGTTCAGTAGATTTAGCAATTTTTTCT
TTACATTTAGCTGGTATTTCATCAATTCTCGGTGCTATTAATTTTATTACAACAATTATTAATATACGAATTAATGGATT
ATCTTTTGATCAAATACCATTATTTGTTTGATCTGTAGGTATTACAGCTTTATTATTATTACTTTCTTTACCTGTTTTAG
CTGGAGCTATTACTATATTATTAACAGATCGAAATTTAAATACATCCTTTTTCGATCCTGCTGGAGGAGGAGATCCTATT
CTTTATCAACACTTATTTTGATTTTTG
>LSTEM217-18|Chilo|SJA-CR300e|
AACATTATATTTTATTTTTGGAGTTTGAGCAGGTATAATCGGAACATCTCTTAGTCTTTTAATTCGTGCTGAATTAGGAA
CACCAGGATCATTAATTGGAGATGATCAAATTTATAATACTATTGTAACAGCTCATGCATTTATTATAATTTTTTTTATA
GTTATACCAATCATAATTGGAGGATTCGGAAATTGATTAGTTCCTTTAATATTAGGAGCCCCAGATATAGCATTTCCTCG
AATAAATAATATAAGATTTTGATTACTACCTCCTTCATTAACATTATTAATTTCAAGAAGAATTGTTGAAAATGGAGCCG
GAACAGGATGAACAGTATACCCCCCACTTTCATCCAATATTGCTCATGCTGGAAGTTCAGTAGATCTAGCAATTTTTTCC
TTGCATTTAGCTGGAATTTCCTCAATTTTAGGTGCAATTAACTTTATTACAACAATTATTAATATACGAATTAATAAACT
ATCATTTGACCAATTACCATTATTTGTTTGATCTGTAGGCATTACAGCCTTATTATTACTTCTATCTCTTCCTGTATTAG
CTGGAGCTATTACTATACTTCTAACTGATCGAAATTTAAATACATCTTTTTTTGATCCTGCCGGAGGAGGAGATCCAAT-
----
>LSTEM218-18|Chilo_aff._crypisimetalla|SJA-CR300i|
AACATTATATTTTATTTTTGGAGTTTGAGCAGGTATAATCGGAACATCTCTTAGTCTTTTAATTCGTGCTGAATTAGGAA
CACCAGGATCATTAATTGGAGATGATCAAATTTATAATACTATTGTAACAGCTCATGCATTTATTATAATTTTTTTTATA
GTTATACCAATCATAATTGGAGGATTCGGAAATTGATTAGTTCCTTTAATATTAGGAGCCCCAGATATAGCATTTCCTCG
AATAAATAATATAAGATTTTGATTACTACCTCCTTCATTAACATTATTAATTTCAAGAAGAATTGTTGAAAATGGAGCCG
GAACAGGATGAACAGTATACCCCCCACTTTCATCCAATATTGCTCATGCTGGAAGTTCAGTAGATCTAGCAATTTTTTCC

TTGCATTTAGCTGGAATTTCCTCAATTTTAGGTGCAATTAACTTTATTACAACAATTATTAATATACGAATTAATAAACT
ATCATTTGACCAATTACCATTATTTGTTTGATCTGTAGGCATTACAGCCTTATTATTACTTCTATCTCTTCCTGTATTAG
CTGGAGCTATTACTATACTTCTAACTGATCGAAATTTAAATACATCTTTTTTTGATCCTGCCGGAGGAGGAGATCCAAT-
----
>LSTEM219-18|Chilo_crypsimetalla|SJA-CR300m|
----------------------ATCTGAGCAGGAATAATTGGAACATCTCTTAGTCTTTTAATTCGAGCTGAATTAGGTA
CCCCTGGATCTTTAATTGGAGATGATCAAATTTATAATACTATTGTCACTGCTCATGCCTTTATTATAATTTTTTTTATA
GTAATACCAATTATAATTGGAGGATTCGGAAATTGATTGGTTCCTTTAATATTAGGAGCTCCAGATATAGCTTTCCCACG
AATAAACAATATAAGATTTTGATTATTACCCCCCTCTTTAACTTTATTACTTTCTAGAAGAATTGTTGAAAATGGTGCTG
GAACAGGATGAACAGTATACCCCCCACTTTCATCTAATATTGCTCATGCTGGGAGATCAGTAGATTTAGCAATTTTTTCC
TTACACTTAGCAGGAATCTCTTCTATTTTAGGAGCTATTAATTTTATTACAACAATTATTAATATACGAATTAATGGATT
ATCATTCGATCAAATACCATTATTTGTGTGATCTGTCGGTATTACCGCATTATTATTATTACTTTCCTTACCTGTTTTAG
CTGGAGCTAGTACTATATTGTTAACTGATCGAAATTGAAATACATCTTTTTTTGATCCTGCTGGAGGAGGTGATCCAATC
GTTCATCTTCTTTCATTTCGATTTTTT
>LSTEM220-18|Chilo_crypsimetalla|SJA-CR300p|
AACTTTATATTTTATTTTTGGAATCTGAGCAGGAATAATTGGAACATCTCTTAGTCTTTTAATTCGAGCTGAATTAGGTA
CCCCTGGATCTTTAATTGGAGATGATCAAATTTATAATACTATTGTCACTGCTCATGCCTTTATTATAATTTTTTTTATA
GTAATACCAATTATAATTGGAGGATTCGGAAATTGATTGGTTCCTTTAATATTAGGAGCTCCAGATATAGCTTTCCCACG
AATAAACAATATAAGATTTTGATTATTACCCCCCTCTTTAACTTTATTAATTTCTAGAAGAATTGTTGAAAATGGTGCTG
GAACAGGATGAACAGTATACCCCCCACTTTCATCTAATATTGCTCATGCTGGGAGATCAGTAGATTTAGCAATTTTTTCC
TTACACTTAGCAGGAATCTCTTCTATTTTAGGAGCTATTAATTTTATTACAACAATTATTAATATACGAATTAATGGATT
ATCATTCGATCAAATACCATTATTTGTGTGATCTGTCGGTATTACAGCATTATTATTATTACTTTCCTTACCTGTTTTAG
CTGGAGCTATTACTATATTGTTAACTGATCGAAATTTAAATACATCTTTTTTTGATCCTGCTGGAGGAGGTGATCCAATC
CTTTATCAACATTTATTTTGATTTTT-
>LSTEM221-18|Chilo|SJA-CR300s|
AACTTTATATTTTATTTTTGGAATCTGAGCAGGAATAATTGGAACATCTCTTAGTCTTTTAATTCGAGCTGAATTAGGTA
CCCCTGGATCTTTAATTGGAGATGATCAAATTTATAATACTATTGTCACTGCTCATGCCTTTATTATAATTTTTTTTATA
GTAATACCAATTATAATTGGAGGATTCGGAAATTGATTGGTTCCTTTAATATTAGGAGCTCCAGATATAGCTTTCCCACG
AATAAACAATATAAGATTTTGATTATTACCCCCCTCTTTAACTTTATTAATTTCTAGAAGAATTGTTGAAAATGGTGCTG
GAACAGGATGAACAGTATACCCCCCACTTTCATCTAATATTGCTCATGCTGGAAGATCAGTAGATTTAGCAATTTTTTCC
TTACACTTAGCAGGAATCTCTTCTATTTTAGGAGCTATTAATTTTATTACAACAATTATTAATATACGAATTAATGGATT
ATCATTCGATCAAATACCATTATTTGTGTGATCTGTCGGTATTACAGCATTATTATTATTACTTTCCTTACCTGTTTTAG
CTGGAGCTATTACTATATTGTTAACTGATCGAAATTTAAATACATCTTTTTTTGATCCTGCTGGAGGAGGTGATCCAATC
CTTTATCAACATTTATTTTGATTTTTT
>LSTEM222-18|Chilo_crypsimetalla|SJA-CR300t|
AACTTTATATTTTATTTTTGGAATCTGAGCAGGAATAATTGGAACATCTCTTAGTCTTTTAATTCGAGCTGAATTAGGTA
CCCCTGGATCTTTAATTGGAGATGATCAAATTTATAATACTATTGTCACTGCTCATGCCTTTATTATAATTTTTTTTATA
GTAATACCAATTATAATTGGAGGATTCGGAAATTGATTGGTTCCTTTAATATTAGGAGCTCCAGATATAGCTTTCCCACG
AATAAACAATATAAGATTTTGATTATTACCCCCCTCTTTAACTTTATTAATTTCTAGAAGAATTGTTGAAAATGGTGCTG
GAACAGGATGAACAGTATACCCCCCACTTTCATCTAATATTGCTCATGCTGGAAGATCAGTAGATTTAGCAATTTTTTCC
TTACACTTAGCAGGAATCTCTTCTATTTTAGGAGCTATTAATTTTATTACAACAATTATTAATATACGAATTAATGGATT
ATCATTCGATCAAATACCATTATTTGTGTGATCTGTCGGTATTACAGCATTATTATTATTACTTTCCTTACCTGTTTTAG
CTGGAGCTATTACTATATTGTTAACTGATCGAAATTTAAATACATCTTTTTTTGATCCTGCTGGAGGAGGTGATCCAATC
CTTTATCAACATTTATTTTGATTTTTT
>LSTEM223-18|Chilo_crypsimetalla|SJA-CR300v|
AACTTTATATTTTATTTTTGGAATCTGAGCAGGAATAATTGGAACATCTCTTAGTCTTTTAATTCGAGCTGAATTAGGTA
CCCCTGGATCTTTAATTGGAGATGATCAAATTTATAATACTATTGTCACTGCTCATGCCTTTATTATAATTTTTTTTATA
GTAATACCAATTATAATTGGAGGATTCGGAAATTGATTGGTTCCTTTAATATTAGGAGCTCCAGATATAGCTTTCCCACG
AATAAACAATATAAGATTTTGATTATTACCCCCCTCTTTAACTTTATTAATTTCTAGAAGAATTGTTGAAAATGGTGCTG
GAACAGGATGAACAGTATACCCCCCACTTTCATCTAATATTGCTCATGCTGGAAGATCAGTAGATTTAGCAATTTTTTCC
TTACACTTAGCAGGAATCTCTTCTATTTTAGGAGCTATTAATTTTATTACAACAATTATTAATATACGAATTAATGGATT
ATCATTCGATCAAATACCATTATTTGTGTGATCTGTCGGTATTACAGCATTATTATTATTACTTTCCTTACCTGTTTTAG
CTGGAGCTATTACTATATTGTTAACTGATCGAAATTTAAATACATCTTTTTTTGATCCTGCTGGAGGAGGTGATCCAATC
CTTTATCAACATTTATTTTGATTTTT-
>LSTEM224-18|Chilo_sacchariphagus|SJA-Cs11|
---TCCCTTAGACTTTTAATTCGAGCTGAATTAGGAA
ATCCAGGTTCATTAATTGGAGATGATCAAATTTATAATACTATTGTTACAGCTCATGCATTTATTATAATTTTTTTTATA

GTAATACCAATTATAATCGGAGGATTTGGAAATTGATTAGTTCCATTAATATTAGGAGCTCCTGATATAGCCTTTCCTCG
TTTAAATAATATAAGATTTTGATTATTGCCCCCTTCTTTAACTCTTTTAATTTCTAGAAGAATTGTTGAAAATGGAGCAG
GAACTGGATGAACAGTCTACCCCCCCCTATCTTCCAATATTTCACATGCTGGAAGTTCAGTAGATTTAGCCATCTTTTCT
CTTCATTTAGCAGGAATTTCTTCAATTTTAGGAGCTATTAATTTTATTACTACAATTATTAATATACGAATTAATGGATT
ATTATTTGATCAAATACCATTATTTGTTTGATCTGTTGGTATTACAGCATTACTTCTTCTTCTCTCTTTACCAGTATTAG
CAGGTGCTATTACCATACTATTAACTGACCGAAATTTAAATACATCTTTTTTTGATCCAGCTGGAGGAGGTGATCCAATT
TTATATCAACATTTATTTTGATTTTTT
>LSTEM225-18|Chilo_sacchariphagus|SJA-Cs12|

--AAAGGTTCATTAATTGGAGATGATCAAATTTATAATACTATTGTTACAGCTCATGCATTTATTATAATTTTTTTTATA
GTAATACCAATTATAATCGGAGGATTTGGAAATTGATTAGTTCCATTAATATTAGGAGCTCCTGATATAGCCTTTCCTCG
TTTAAATAATATAAGATTTTGATTATTGCCCCCTTCTTTAACTCTTTTAATTTCTAGAAGAATTGTTGAAAATGGAGCAG
GAACTGGATGAACAGTCTACCCCCCCCTATCTTCCAATATTTCACATGCTGGAAGTTCAGTAGATTTAGCCATCTTTTCT
CTTCATTTAGCAGGAATTTCTTCAATTTTAGGAGCTATTAATTTTATTACTACAATTATTAATATACGAATTAATGGATT
ATTATTTGATCAAATACCATTATTTGTTTGATCTGTTGGTATTACAGCATTACTTCTTCTTCTCTCTTTACCAGTATTAG
CAGGTGCTATTACCATACTATTAACTGACCGAAATTTAAATACATCTTTTTTTGATCCAGCTGGAGGAGGTGATCCAATT
TTATATCAACATTTATTTTGATTTTTT
>LSTEM226-18|Chilo_auricilius|SJA-U2|
AACTTTATACTTTATTTTTGGAATTTGAAGTGGAATAATTGGGACATCTCTAAGACTTTTAATTCGTGCTGAATTAGGAA
CCCCGGGATCATTAATTGGAGATGATCAAATTTATAATACTATTGTTACAGCTCATGCATTTATTATAATTTTTTTTATA
GTTATACCAATTATAATTGGAGGCTTTGGTAACTGATTAGTACCATTAATGCTAGGAGCTCCTGATATAGCCTTCCCTCG
AATAAATAATATAAGATTTTGATTATTACCCCCATCATTAACATTATTAATTTCTAGAAGAATTGTAGAAAATGGAGCTG
GAACAGGATGAACGGTATACCCCCCCCTTTCATCAAATATTGCCCATGGTGGAAGTTCTGTAGATTTAGCCATTTTTTCT
CTTCATTTAGCTGGTATTTCCTCAATTTTAGGAGCTATTAATTTTATTACAACAATTATTAATATACGAATTAATAAATT
ATCATTTGATCAACTACCATTATTTGTTTGATCTGTTGGTATTACAGCCTTATTATTATTACTTTCATTACCAGTACTAG
CTGGAGCTATTACTATACTTTTAACTGATCGAAACTTAAATACATCTTTTTTTGACCCTGCTGGGGGAGGAGACCCTATT
CTTTAT---------------------
>LSTEM227-18|Scirpophaga_excerptalis|SRB013a|
GACATTATATTTTATTTTTGGAATTTGAGCTGGTATAGTGGGGACTTCACTTAGTTTACTAATTCGAGCCGAACTAGGTA
CTCCGGGATCATTAATCGGAGATGATCAAATCTATAATACTATTGTAACTGCTCACGCTTTTATTATAATTTTTTTTATG
GTTATGCCCATTATAATTGGGGGATTCGGAAACTGATTAGTGCCTTTAATATTGGGAGCCCCAGATATGGCCTTCCCCCG
AATAAATAATATAAGTTTTTGATTATTACCCCCTTCTTTAACCCTCTTAATCTCAAGAAGAGTCGTTGAAAATGGAGCTG
GAACAGGATGAACTGTTTATCCGCCCTTATCCTCCAATATTGCTCATGGTGGGACTTCTGTAGATTTAGCCATTTTTTCA
TTACATTTAGCTGGAATTTCTTCTATTCTAGGGGCTATTAACTTCATTACAACTATTATTAATATACGAATTAATGGACT
ATCTTTTGATCAAATACCTTTATTTGTATGAGCAGTTGGTATTACTGCCCTTCTTCTTCTTCTCTCACTACCTGTATTAG
CGGGAGCTATTACTATATTATTAACAGATCGAAACTTAAATACTTCTTTCTTTGACCCAGCAGGAGGTGGAGACCCCATT
CTTTAT---------------------
>LSTEM228-18|Scirpophaga_excerptalis|SRB013b|
GACATTATATTTTATTTTTGGAATTTGAGCTGGTATAGTGGGGACTTCACTTAGTTTACTAATTCGAGCCGAACTAGGTA
CTCCGGGATCATTAATYGGAGATGATCAAATCTATAATACTATTGTAACTGCTCACGCTTTTATTATAATTTTTTTTATG
GTTATGCCCATTATAATTGGGGGATTCGGAAACTGATTAGTGCCTTTAATATTGGGAGCCCCAGATATGGCCTTCCCCCG
AATAAATAATATAAGTTTTTGATTATTACCCCCTTCTTTAACCCTCTTAATCTCAAGAAGAGTCGTTGAAAATGGAGCTG
GAACAGGATGAACTGTTTATCCGCCCTTATCCTCCAATATTGCTCATGGTGGGACTTCTGTAGATTTAGCCATTTTTTCA
TTACATTTAGCTGGAATTTCTTCTATTCTAGGGGCTATTAACTTCATTACAACTATTATTAATATACGAATTAATGGACT
ATCTTTTGATCAAATACCTTTATTTGTATGAGCAGTTGGTATTACTGCCCTTCTTCTTCTTCTCTCACTACCTGTATTAG
CAGGAGCTATTACTATATTATTAACAGATCGAAACTTAAATACTTCTTTCTTTGACCCAGCAGGAGGTGGAGACCCAATT
CTTTAT---------------------
>LSTEM229-18|Sesamia_grisescens|SRB016a|
AACATTATATTTTATTTTCGGAATTTGAGCTGGTATAGTTGGAACATCACTAAGATTATTAATTCGAGCCGAACTAGGGA
CCCCTGGTTCTTTAATTGGAGACGATCAAATTTATAATACTATTGTTACAGCTCATGCTTTCATTATAATTTTCTTTATA
GTTATACCAATTATAATTGGTGGATTTGGAAATTGACTCGTACCTTTAATATTAGGAGCCCCTGATATAGCATTTCCACG
AATAAATAATATAAGATTTTGATTATTACCCCCCTCTTTAACTTTACTAATTTCAAGTAGAATTGTAGAAAATGGGGCAG
GAACTGGGTGAACAGTGTACCCCCCACTTTCATCTAATATTGCCCATGGAGGAAGATCAGTGGACTTAGCTATTTTTTCC
CTTCATTTAGCAGGTATTTCATCTATTTTAGGAGCTATTAATTTTATCACAACAATTATTAATATACGATTAAATAACTT
ATCCTTTGATCAAATACCTTTATTTATTTGAGCTGTTGGAATTACTGCATTTTTATTATTATTATCTTTACCTGTTTTAG
CAGGAGCTATTACAATATTATTAACCGATCGAAACTTAAATACATCATTTTTCGATCCGGCAGGAGGAGGCGATCCTATT
TTATAC---------------------

>LSTEM230-18|Sesamia_grisescens|SRB016b|
AACATTATATTTTATTTTCGGAATTTGAGCTGGTATAGTTGGAACATCACTAAGATTATTAATTCGAGCCGAACTAGGGA
CCCCTGGTTCTTTAATTGGAGACGATCAAATTTATAATACTATTGTTACAGCTCATGCTTTCATTATAATTTTCTTTATA
GTTATACCAATTATAATTGGTGGATTTGGAAATTGACTCGTACCTTTAATATTAGGAGCCCCTGATATAGCATTTCCACG
AATAAATAATATAAGATTTTGATTATTACCCCCCTCTTTAACTTTACTAATTTCAAGTAGAATTGTAGAAAATGGGGCAG
GAACTGGGTGAACAGTGTACCCCCCACTTTCATCTAATATTGCCCATGGAGGAAGATCAGTGGACTTAGCTATTTTTTCC
CTTCATTTAGCAGGTATTTCATCTATTTTAGGAGCTATTAATTTTATCACAACAATTATTAATATACGATTAAATAACTT
ATCCTTTGATCAAATACCTTTATTTATTTGAGCTGTTGGAATTACTGCATTTTTATTATTATTATCTTTACCTGTTTTAG
CAGGAGCTATTACAATATTATTAACCGATCGAAACTTAAATACATCATTTTTCGATCCGGCAGGAGGAGGCGATCCTATT
TTATAC---------------------
>LSTEM231-18|Chilo_auricilius|SRB017a|
AACTTTATACTTTATTTTTGGAATTTGAAGTGGAATAATTGGAACATCTCTAAGACTTTTAATTCGTGCTGAATTAGGAA
CTCCAGGGTCATTAATTGGAGATGATCAAATTTACAATACTATTGTTACAGCTCATGCATTTATTATAATTTTTTTTATA
GTTATACCAATTATAATTGGAGGCTTTGGTAATTGATTAGTACCATTAATACTAGGGGCTCCTGATATAGCCTTCCCTCG
AATAAATAATATAAGATTTTGATTATTGCCCCCATCATTAACATTATTAATTTCTAGAAGAATTGTAGAAAATGGAGCTG
GAACAGGATGAACGGTTTACCCCCCCCTTTCATCAAATATTGCCCATGGAGGAAGTTCTGTAGATTTAGCTATTTTTTCT
CTTCATTTAGCTGGTATTTCCTCAATTTTAGGAGCTATTAATTTTATTACAACAATTATTAATATACGAATTAATAAACT
ATCATTTGATCAATTACCATTATTTGTTTGATCTGTTGGTATTACAGCTTTATTATTACTACTTTCATTGCCGGTACTAG
CTGGAGCTATTACTATACTTTTAACTGATCGAAACTTAAATACATCTTTTTTTGACCCTGCTGGAGGAGGAGACCCAATT
CTTTAT---------------------
>LSTEM232-18|Chilo_auricilius|SRB017b|
AACTTTATACTTTATTTTTGGAATTTGAAGTGGAATAATTGGAACATCTCTAAGACTTTTAATTCGTGCTGAATTAGGAA
CTCCAGGGTCATTAATTGGAGATGATCAAATTTACAATACTATTGTTACAGCTCATGCATTTATTATAATTTTTTTTATA
GTTATACCAATTATAATTGGAGGCTTTGGTAATTGATTAGTACCATTAATACTAGGGGCTCCTGATATAGCCTTCCCTCG
AATAAATAATATAAGATTTTGATTATTGCCCCCATCATTAACATTATTAATTTCTAGAAGAATTGTAGAAAATGGAGCTG
GAACAGGATGAACGGTTTACCCCCCCCTTTCATCAAATATTGCCCATGGAGGAAGTTCTGTAGATTTAGCTATTTTTTCT
CTTCATTTAGCTGGTATTTCCTCAATTTTAGGAGCTATTAATTTTATTACAACAATTATTAATATACGAATTAATAAACT
ATCATTTGATCAATTACCATTATTTGTTTGATCTGTTGGTATTACAGCTTTATTATTACTACTTTCATTGCCGGTACTAG
CTGGAGCTATTACTATACTTTTAACTGATCGAAACTTAAATACATCTTTTTTTGACCCTGCTGGAGGAGGAAACCCAATT
CTTTAT---------------------
>LSTEM233-18|Chilo_sacchariphagus_sacchariphagus|SRB019a|
AACTTTATATTTTATTTTTGGAATTTGAGCTGGAATAATTGGAACATCCCTTAGACTTTTAATTCGAGCTGAATTAGGAA
ATCCAGGTTCATTAATTGGAGATGATCAAATTTATAATACTATTGTTACAGCTCATGCATTTATTATAATTTTTTTTATA
GTAATACCAATTATAATCGGAGGATTTGGAAATTGATTAGTTCCATTAATATTAGGAGCTCCTGATATAGCCTTTCCTCG
TTTAAATAATATAAGATTTTGATTATTACCCCCTTCTTTAACTCTTTTAATTTCTAGAAGAATTGTTGAAAATGGAGCAG
GAACTGGATGAACAGTCTACCCCCCCCTATCTTCCAATATTTCACATGCTGGAAGTTCAGTAGATTTAGCCATCTTTTCT
CTTCATTTAGCAGGAATTTCTTCAATTTTAGGAGCTATTAATTTTATTACTACAATTATTAATATACGAATTAATGGATT
ATTATTTGATCAAATACCATTATTTGTTTGATCTGTTGGTATTACAGCATTACTTCTTCTTCTCTCTTTACCAGTATTAG
CAGGTGCTATTACCATACTATTAACTGACCGAAATTTAAATACATCTTTTTTTGATCCAGCTGGAGGAGGTGATCCAATT
TTATAT---------------------
>LSTEM234-18|Chilo_sacchariphagus_sacchariphagus|SRB019b|
AACTTTATATTTTATTTTTGGAATTTGAGCTGGAATAATTGGAACATCCCTTAGACTTTTAATTCGAGCTGAATTAGGAA
ATCCAGGTTCATTAATTGGAGATGATCAAATTTATAATACTATTGTTACAGCTCAYGCATTTATTATAATTTTTTTTATA
GTAATACCAATTATAATCGGAGGATTTGGAAATTGATTAGTTCCATTAATATTAGGAGCTCCTGATATAGCCTTTCCTCG
TTTAAATAATATAAGATTTTGATTATTACCCCCTTCTTTAACTCTTTTAATTTCTAGAAGAATTGTTGAAAATGGAGCAG
GAACTGGATGAACAGTCTACCCCCCCCTATCTTCCAATATTTCACATGCTGGAAGTTCAGTAGATTTAGCCATCTTTTCT
CTTCATTTAGCAGGAATTTCTTCAATTTTAGGAGCTATTAATTTTATTACTACAATTATTAATATACGAATTAATGGATT
ATTATTTGATCAAATACCATTATTTGTTTGATCTGTTGGTATTACAGCATTACTTCTTCTTCTCTCTTTACCAGTATTAG
CAGGTGCTATTACCATACTATTAACTGACCGAAATTTAAATACATCTTTTTTTGATCCAGCTGGAGGAGGTGATCCAATT
TTATAT---------------------
>LSTEM235-18|Scirpophaga_excerptalis|SRB020a|
AACATTATATTTTATTTTTGGAATTTGAGCTGGTATAGTAGGAACTTCACTTAGTTTATTAATTCGAGCTGAATTAGGTA
CTCCCGGGTCACTAATTGGAGATGATCAAATCTATAATACTATCGTAACTGCTCATGCTTTTATTATAATTTTTTTTATA
GTTATACCTATTATAATTGGGGGATTTGGAAACTGATTAGTACCTTTAATGTTAGGAGCCCCAGATATAGCTTTTCCTCG
AATAAATAATATAAGTTTTTGATTATTACCTCCTTCTTTAACCCTCTTAATCTCGAGAAGAATTGTTGAAAATGGAGCTG
GAACAGGATGAACTGTATACCCCCCTCTATCCTCTAATATTGCTCATGGTGGAACTTCTGTAGATCTAGCCATTTTTTCA
TTACATTTAGCTGGAATTTCCTCTATTCTAGGAGCTATTAACTTTATCACAACTATTATTAATATACGAATTAATGGATT

ATCTTTTGATCAAATACCTTTATTTGTATGAGCAGTTGGTATTACCGCTCTTCTCCTTCTTCTCTCACTACCTGTATTAG
CAGGAGCTATTACTATATTATTAACAGATCGAAACTTAAATACTTCTTTCTTTGACCCAGCAGGAGGTGGAGATCCAATT
CTTTAT---------------------
>LSTEM236-18|Scirpophaga_excerptalis|SRB020b|
AACATTATATTTTATTTTTGGAATTTGAGCTGGTATAGTAGGAACTTCACTTAGTTTATTAATTCGAGCTGAATTAGGTA
CTCCTGGGTCACTAATTGGAGATGATCAAATCTATAATACTATYGTAACTGCTCATGCTTTTATTATAATTTTTTTTATA
GTTATACCTATTATAATTGGAGGATTTGGAAACTGATTAGTACCTTTAATGTTAGGAGCCCCAGATATAGCTTTTCCTCG
AATAAATAATATAAGTTTTTGATTATTACCTCCTTCTTTAACCCTCTTAATCTCGAGAAGAATTGTTGAAAATGGAGCTG
GAACAGGATGAACTGTATACCCCCCTCTATCCTCTAATATTGCTCATGGTGGAACTTCTGTAGATCTAGCCWTTTTTTCA
TTACATTTAGCTGGAATTTCCTCTATTCTAGGAGCTATTAACTTTATCACAACTATTATTAATATACRAATTAATGGATT
ATCTTTTGATCAAATACCTTTATTTGTATGAGCAGTTGGTATTACCGCCCTTCTCCTTCTTCTCTCACTACCTGTATTAG
CGGGAGCTATTACTATATTATTAACAGATCGAAACTTAAATACTTCTTTCTTTGACCCAGCAGGAGGTGGAGATCCAATT
CTTTAT---------------------
>LSTEM237-18|Scirpophaga_excerptalis|SRB021a|
AACATTATATTTTATTTTTGGAATTTGAGCTGGTATAGTAGGAACTTCACTTAGTTTATTAATTCGAGCTGAATTAGGTA
CTCCTGGGTCACTAATTGGAGATGATCAAATCTATAATACTATCGTAACTGCTCATGCTTTTATTATAATTTTTTTTATA
GTTATACCTATTATAATTGGAGGATTTGGAAACTGATTAGTACCTTTAATGTTAGGAGCCCCAGATATAGCTTTTCCTCG
AATAAATAATATAAGTTTTTGATTATTACCTCCTTCTTTAACCCTCTTAATCTCGAGAAGAATTGTTGAAAATGGAGCTG
GAACAGGATGAACTGTATACCCCCCTCTATCCTCTAATATTGCTCATGGTGGAACTTCTGTAGATYTAGCCWTTTTTTCA
TTACATTTAGCTGGAATTTCCTCTATTCTAGGAGCTATTAACTTTATCACAACTATTATTAATATACGAATTAATGGATT
ATCTTTTGATCAAATACCTTTATTTGTATGAGCAGTTGGTATTACCGCCCTTCTCCTTCTTCTCTCACTACCTGTATTAG
CGGGAGCTATTACTATATTATTAACAGATCGAAACTTAAATACTTCTTTCTTTGACCCAGCAGGAGGTGGAGATCCAATT
CTTTAT---------------------
>LSTEM238-18|Scirpophaga_excerptalis|SRB023a|
AACATTATATTTTATTTTTGGAATTTGAGCTGGTATAGTAGGAACTTCACTTAGTTTATTAATTCGAGCTGAATTAGGTA
CTCCTGGGTCACTAATTGGAGATGATCAAATCTATAATACTATYGTAACTGCTCATGCTTTTATTATAATTTTTTTTATA
GTTATACCTATTATAATTGGAGGATTTGGAAACTGATTAGTACCTTTAATATTAGGAGCCCCAGATATAGCTTTCCCTCG
AATAAATAATATAAGTTTTTGATTACTACCCCCTTCTTTAACTCTCTTAATCTCGAGAAGAATTGTTGAAAATGGAGCTG
GAACAGGATGAACTGTATACCCTCCTCTATCCTCTAATATTGCTCACGGTGGAACTTCTGTAGATCTAGCCATTTTTTCA
TTACATTTAGCTGGAATTTCCTCTATTCTAGGAGCTATTAACTTCATCACAACTATTATTAATATACGAATTAATGGGTT
ATCTTTTGATCAAATACCTTTATTTGTATGAGCAGTTGGTATTACCGCTCTTCTCCTTCTTCTCTCACTACCTGTATTAG
CAGGAGCTATTACTATATTATTAACAGATCGAAACTTAAATACTTCTTTCTTTGATCCAGCAGGAGGTGGAGATCCAATC
CTTTAT---------------------
>LSTEM239-18|Scirpophaga_excerptalis|SRB023b|
AACATTATATTTTATTTTTGGAATTTGAGCTGGTATAGTAGGAACTTCACTTAGTTTATTAATTCGAGCTGAATTAGGTA
CTCCTGGGTCACTAATTGGAGATGATCAAATCTATAATACTATCGTAACTGCTCATGCTTTTATTATAATTTTTTTTATA
GTTATACCTATTATAATTGGAGGATTTGGAAACTGATTAGTACCTTTAATATTAGGAGCCCCAGATATAGCTTTCCCTCG
AATAAATAATATAAGTTTTTGATTACTACCCCCTTCTTTAACTCTCTTAATCTCGAGAAGAATTGTTGAAAATGGAGCTG
GGACAGGATGAACTGTATACCCTCCTCTATCCTCTAATATTGCTCACGGTGGAACTTCTGTAGATCTAGCCATTTTTTCA
TTACATTTAGCTGGAATTTCCTCTATTCTAGGAGCTATTAACTTCATCACAACTATTATTAATATACGAATTAATGGGTT
ATCTTTTGATCAAATACCTTTATTTGTATGAGCAGTTGGTATTACCGCTCTTCTCCTTCTTCTCTCACTACCTGTATTAG
CAGGAGCTATTACTATATTATTAACAGATCGAAACTTAAATACTTCTTTCTTTGATCCAGCAGGAGGTGGAGATCCAATC
CTTTAT---------------------
>LSTEM240-18|Chilo_sacchariphagus_sacchariphagus|SRB025a|
AACTTTATATTTTATTTTTGGAATTTGGGCTGGAATAGTTGGAACATCACTTAGACTTTTAATTCGAGCTGAATTAGGAA
ATCCAGGTTCATTAATTGGAGATGATCAAATTTATAACACTATTGTTACAGCTCATGCATTTATTATAATTTTTTTCATG
GTAATACCAATTATAATTGGAGGATTTGGAAATTGATTAGTTCCATTAATATTAGGAGCTCCTGATATAGCCTTTCCCCG
TCTAAATAATATAAGATTTTGATTATTACCCCCCTCTTTAACCCTTTTAATTTCTAGAAGAATCGTTGAAAACGGAGCAG
GAACTGGATGAACAGTCTACCCCCCTTTATCTTCCAATATTTCACATGCTGGAAGTTCAGTAGATTTAGCCATCTTTTCC
CTCCATTTAGCTGGAATTTCTTCAATTTTAGGAGCTATTAATTTTATTACTACAATTATTAACATACGAATTAATGGACT
ATTATTTGATCAAATACCATTATTTGTTTGATCTGTTGGTATTACAGCATTACTTCTTCTCCTTTCCTTACCAGTATTAG
CAGGTGCTATTACTATATTACTAACTGATCGAAATTTAAATACATCTTTTTTTGATCCTGCTGGAGGAGGTGACCCAATT
TTATAC---------------------
>LSTEM241-18|Chilo_sacchariphagus|SRB025b|
AACTTTATATTTTATTTTTGGAATTTGGGCTGGAATAGTTGGAACATCACTTAGACTTTTAATTCGAGCTGAATTAGGAA
ATCCAGGTTCATTAATTGGAGATGATCAAATTTATAACACTATTGTTACAGCTCATGCATTTATTATAATTTTTTTCATG
GTAATACCAATTATAATTGGAGGATTTGGAAATTGATTAGTTCCATTAATATTAGGAGCTCCTGATATAGCCTTTCCCCG

TCTAAATAATATAAGATTTTGATTATTACCCCCCTCTTTAACCCTTTTAATTTCTAGAAGAATCGTTGAAAACGGAGCAG
GAACTGGATGAACAGTCTACCCCCCTTTATCTTCCAATATTTCACATGCTGGAAGTTCAGTAGATTTAGCCATCTTTTCC
CTCCATTTAGCTGGAATTTCTTCAATTTTAGGAGCTATTAATTTTATTACTACAATTATTAACATACGAATTAATGGACT
ATTATTTGATCAAATACCATTATTTGTTTGATCTGTTGGTATTACAGCATTACTTCTTCTCCTTTCCTTACCAGTATTAG
CAGGTGCTATTACTATATTACTAACTGATCGAAATTTAAATACATCTTTTTTGATCCTGCTGGAGGAGGTGACCCAATT
TTATAC---------------------
>LSTEM242-18|Chilo_auricilius|SRB026a|
AACTTTATACTTTATTTTTGGAATTTGAAGTGGAATAATTGGAACATCTCTAAGACTTTTAATTCGTGCTGAATTAGGAA
CTCCAGGGTCATTAATTGGAGATGATCAAATTTACAATACTATTGTTACAGCTCATGCATTTATTATAATTTTTTTTATA
GTTATACCAATTATAATTGGAGGCTTTGGTAATTGATTAGTACCATTAATACTAGGGGCTCCTGATATAGCCTTCCCTCG
AATAAATAATATAAGATTTTGATTATTGCCCCCATCATTAACATTATTAATTTCTAGAARAATTGTAGAAAATGGAGCTG
GAACAGGATGAACGGTTTACCCCCCCCTTTCATCAAATATTGCCCATGGAGGAAGTTCTGTAGATTTAGCTATTTTTTCT
CTTCATTTAGCTGGTATTTCCTCAATTTTAGGAGCTATTAATTTTATTACAACAATTATTAATATACGAATTAATAAACT
ATCATTTGATCAATTACCATTATTTGTTTGATCTGTTGGTATTACAGCTTTATTATTACTACTTTCATTGCCGGTACTAG
CTGGAGCTATTACTATACTTTTAACTGATCGAAACTTAAATACATCTTTTTTGACCCTGCTGGAGGAGGAGACCCAATT
CTTTAT---------------------
>LSTEM243-18|Chilo_sacchariphagus_sacchariphagus|SRB027a|
AACTTTATATTTTATTTTTGGAATTTGAGCTGGAATAATTGGAACATCCCTTAGACTTTTAATTCGAGCTGAATTAGGAA
ATCCAGGTTCATTAATTGGAGATGATCAAATTTATAATACTATTGTTACAGCTCATGCATTTATTATAATTTTTTTTATA
GTAATACCAATTATAATCGGAGGATTTGGAAATTGATTAGTTCCATTAATATTAGGAGCTCCTGATATAGCCTTTCCTCG
TTTAAATAATATAAGATTTTGATTATTACCCCCTTCTTTAACTCTTTTAATTTCTAGAAGAATTGTTGAAAATGGAGCAG
GAACTGGATGAACAGTCTACCCCCCCCTATCTTCCAATATTTCACATGCTGGAAGTTCAGTAGATTTAGCCATCTTTTCT
CTTCATTTAGCAGGAATTTCTTCAATTTTAGGAGCTATTAATTTTATTACTACAATTATTAATATACGAATTAATGGATT
ATTATTTGATCAAATACCATTATTTGTTTGATCTGTTGGTATTACAGCATTACTTCTTCTTCTCTCTTTACCAGTATTAG
CAGGTGCTATTACCATACTATTAACTGACCGAAATTTAAATACATCTTTTTTGATCCAGCTGGAGGAGGTGATCCAATT
TTATAT---------------------
>LSTEM244-18|Chilo_sacchariphagus_sacchariphagus|SRB028a|
AACTTTATATTTTATTTTTGGAATTTGAGCTGGAATAATTGGAACATCCCTTAGACTTTTAATTCGAGCTGAATTAGGAA
ATCCAGGTTCATTAATTGGAGATGATCAAATTTATAATACTATTGTTACAGCTCATGCATTTATTATAATTTTTTTTATA
GTAATACCAATTATAATYGGAGGATTTGGAAATTGATTAGTTCCATTAATATTAGGAGCTCCTGATATAGCCTTTCCTCG
TTTAAATAATATAAGATTTTGATTATTACCCCCTTCTTTAACTCTTTTAATTTYTAGAAGAATTGTTGAAAATGGAGCAG
GAACTGGATGAACAGTCTACCCCCCCCTATCTTCCAATATTTCACATGCTGGAAGTTCAGTAGATTTAGCCATCTTTTCT
CTTCATTTAGCAGGAATTTCTTCAATTTTAGGAGCTATTAATTTTATTACTACAATTATTAATATGCGAATTAATGGATT
ATTATTTGATCAAATACCATTATTTGTTTGATCTGTTGGTATTACAGCATTACTTCTTCTTCTCTCTTTACCAGTATTAG
CAGGTGCTATTACCATACTATTAACTGACCGAAATTTAAATACATCTTTTTTGATCCAGCTGGAGGAGGTGATCCAATT
TTATAT---------------------
>LSTEM245-18|Chilo_orichalcociliellus|SRB029a|
AACTTTATATTTTATTTTTGGAATTTGAGCAGGAATAATTGGAACATCACTTAGACTCCTAATTCGAGCTGAATTGGGAA
CCCCTGGATCTTTAATTGGTGATGATCAAATTTATAATACTATTGTTACAGCTCATGCATTTATTATAATTTTTTTTATA
GTTATACCAATTATAATTGGAGGATTTGGAAATTGATTAGTACCTTTAATGTTAGGAGCTCCTGATATAGCCTTCCCACG
AATAAATAATATAAGATTTTGATTACTACCCCCATCACTAACTTTATTAATTTCTAGAAGAATTGTTGAAAATGGAGCTG
GAACTGGGTGAACAGTTTATCCCCCACTTTCATCCAATATTGCCCATGGTGGGAGTTCAGTAGATCTAGCAATTTTTTCT
CTTCATTTAGCTGGAATTTCCTCAATTTTAGGTGCTATTAATTTTATTACAACAATCATTAATATACGAATTAATGGATT
ATCATTTGATCAAATACCTTTATTTGTTTGATCTGTTGGTATTACAGCCTTATTATTATTACTTTCATTACCTGTTTTAG
CAGGAGCTATTACTATATTATTAACTGATCGAAATTTAAATACATCATTTTTTGATCCTGCTGGTGGAGGAGATCCAATT
CTTTAT---------------------
>LSTEM246-18|Chilo_sacchariphagus|ukzn0105|
---------TTTTATTTTTGGAATTTGAGCTGGAATAATTGGAACATCCCTTAGACTTTTAATTCGAGCTGAATTAGGAA
ATCCAGGTTCATTAATTGGAGATGATCAAATTTATAATACTATTGTTACAGCCCATGCATTTATTATAATTTTTTTTATA
GTAATACCAATTATAATCGGAGGATTTGGAAATTGATTAGTTCCATTAATATTAGGAGCTCCTGATATAGCCTTTCCTCG
TTTAAATAATATAAGATTTTGATTATTACCCCCTTCTTTAACTCTTTTAATTTCTAGAAGAATTGTTGAAAATGGAGCAG
GAACTGGATGAACAGTCTACCCCCCCCTATCTTCCAATATTTCACATGCTGGAAGTTCAGTAGATTTAGCCATCTTTTCT
CTTCATTTAGCAGGAATTTCTTCAATTTTAGGAGCTATTAATTTTATTACTACAATTATTAATATGCGAATTAATGGATT
ATTATTTGATCAAATACCATTATTTGTTTGATCTGTTGGTATTACAGCATTACTTCTTCTTCTCTCTTTACCAGTATTAG
CAGGTGCTATTACCATACTATTAACTGACCGAAATTTAAATACATCTTTTTTGATCCAGCTGGAGGAGGTGATCCAATT
TTATATCAACATTTATTT---------
>LSTEM247-18|Chilo_sacchariphagus|ukzn0106|

---------TTTTATTTTTGGAATTTGAGCTGGAATAATTGGAACATCCCTTAGACTTTTAATTCGAGCTGAATTAGGAA
ATCCAGGTTCATTAATTGGAGATGATCAAATTTATAATACTATTGTTACAGCCCATGCATTTATTATAATTTTTTTATA
GTAATACCAATTATAATCGGAGGATTTGGAAATTGATTAGTTCCATTAATATTAGGAGCTCCTGATATAGCCTTTCCTCG
TTTAAATAATATAAGATTTTGATTATTACCCCCTTCTTTAACTCTTTTAATTTCTAGAAGAATTGTTGAAAATGGAGCAG
GAACTGGATGAACAGTCTACCCCCCCCTATCTTCCAATATTTCACATGCTGGAAGTTCAGTAGATTTAGCCATCTTTTCT
CTTCATTTAGCAGGAATTTCTTCAATTTTAGGAGCTATTAATTTTATTACTACAATTATTAATATGCGAATTAATGGATT
ATTATTTGATCAAATACCATTATTTGTTTGATCTGTTGGTATTACAGCATTACTTCTTCTTCTCTCTTTACCAGTATTAG
CAGGTGCTATTACCATACTATTAACTGACCGAAATTTAAATACATCTTTTTTTGATCCAGCTGGAGGAGGTGATCCAATT
TTATATCAACATTTATTT---------
>LSTEM248-18|Chilo_sacchariphagus|ukzn0107|
---------TTTTATTTTTGGAATTTGAGCTGGAATAATTGGAACATCCCTTAGACTTTTAATTCGAGCTGAATTAGGAA
ATCCAGGTTCATTAATTGGAGATGATCAAATTTATAATACTATTGTTACAGCTCATGCATTTATTATAATTTTTTTATA
GTAATACCAATTATAATCGGAGGATTTGGAAATTGATTAGTTCCATTAATATTAGGAGCTCCTGATATAGCCTTTCCTCG
TTTAAATAATATAAGATTTTGATTATTACCCCCTTCTTTAACTCTTTTAATTTCTAGAAGAATTGTTGAAAACGGAGCAG
GAACTGGATGAACAGTCTACCCCCCCCTATCTTCCAATATTTCACATGCTGGAAGTTCAGTAGATTTAGCCATCTTTTCT
CTTCATTTAGCAGGAATTTCTTCAATTTTAGGAGCTATTAATTTTATTACTACAATTATTAATATACGAATTAATGGATT
ATTATTTGATCAAATACCATTATTTGTTTGATCTGTTGGTATTACAGCATTACTTCTTCTTCTCTCTTTACCAGTATTAG
CAGGTGCTATTACCATATTATTAACTGACCGAAATTTAAATACATCTTTTTTTGATCCAGCTGGAGGAGGTGATCCAATT
TTATATCAACATTTATTT---------
>LSTEM249-18|Chilo_sacchariphagus|ukzn0108|
---------TTTTATTTTTGGAATTTGAGCTGGAATAATTGGAACATCCCTTAGACTTTTAATTCGAGCTGAATTAGGAA
ATCCAGGTTCATTAATTGGAGATGATCAAATTTATAATACTATTGTTACAGCTCATGCATTTATTATAATTTTTTTATA
GTAATACCAATTATAATCGGAGGATTTGGAAATTGATTAGTTCCATTAATATTAGGAGCTCCTGATATAGCCTTTCCTCG
TTTAAATAATATAAGATTTTGATTATTACCCCCTTCTTTAACTCTTTTAATTTCTAGAAGAATTGTTGAAAACGGAGCAG
GAACTGGATGAACAGTCTACCCCCCCCTATCTTCCAATATTTCACATGCTGGAAGTTCAGTAGATTTAGCCATCTTTTCT
CTTCATTTAGCAGGAATTTCTTCAATTTTAGGAGCTATTAATTTTATTACTACAATTATTAATATACGAATTAATGGATT
ATTATTTGATCAAATACCATTATTTGTTTGATCTGTTGGTATTACAGCATTACTTCTTCTTCTCTCTTTACCAGTATTAG
CAGGTGCTATTACCATATTATTAACTGACCGAAATTTAAATACATCTTTTTTTGATCCAGCTGGAGGAGGTGATCCAATT
TTATATCAACATTTATTT---------
>LSTEM250-18|Chilo_sacchariphagus|ukzn0109|
---------TTTTATTTTTGGAATTTGAGCTGGAATAATTGGAACATCCCTTAGACTTTTAATTCGAGCTGAATTAGGAA
ATCCAGGTTCATTAATTGGAGATGATCAAATTTATAATACTATTGTTACAGCTCATGCATTTATTATAATTTTTTTATA
GTAATACCAATTATAATCGGAGGATTTGGAAATTGATTAGTTCCATTAATATTAGGAGCTCCTGATATAGCCTTTCCTCG
TTTAAATAATATAAGATTTTGATTGTTACCCCCTTCTTTAACTCTTTTAATTTCTAGAAGAATTGTTGAAAACGGAGCAG
GAACTGGATGAACAGTCTACCCCCCCCTATCTTCCAATATTTCACATGCTGGAAGTTCAGTAGATTTAGCCATCTTTTCT
CTTCATTTAGCAGGAATTTCTTCAATTTTAGGAGCTATTAATTTTATTACTACAATTATTAATATACGAATTAATGGATT
ATTATTTGATCAAATACCATTATTTGTTTGATCTGTTGGTATTACAGCATTACTTCTTCTTCTCTCTTTACCAGTATTAG
CAGGTGCTATTACCATATTATTAACTGACCGAAATTTAAATACATCTTTTTTTGATCCAGCTGGAGGAGGTGATCCAATT
TTATATCAACATTTATTT---------
>LSTEM251-18|Chilo_sacchariphagus|ukzn0110|
--------------------------------GAATAATTGGAACATCCCTTAGACTTTTAATTCGAGCTGAATTAGGAA
ATCCAGGTTCATTAATTGGAGATGATCAAATTTATAATACTATTGTTACAGCTCATGCATTTATTATAATTTTTTTATA
GTAATACCAATTATAATCGGAGGATTTGGAAATTGATTAGTTCCATTAATATTAGGAGCTCCTGATATAGCCTTTCCTCG
TTTAAATAATATAAGATTTTGATTATTACCCCCTTCTTTAACTCTTTTAATTTCTAGAAGAATTGTTGAAAACGGAGCAG
GAACTGGATGAACAGTCTACCCCCCCCTATCTTCCAATATTTCACATGCTGGAAGTTCAGTAGATTTAGCCATCTTTTCT
CTTCATTTAACAGGAATTTCTTCAATTTTAGGAGCTATTAATTTTATTACTACAATTATTAATATACGAATTAATGGATT
ATTATTTGATCAAATACCATTATTTGTTTGATCTGTTGGTATTACAGCATTACTTCTTCTTCTCTCTTTACCAGTATTAG
CAGGTGCTATTACCATATTATTAACTGACCGAAATTTAAATACATCTTTTTTTGATCCAGCTGGAGGAGGTGATCCAATT
TTATATCAACATTTATTT---------
>LSTEM252-18|Chilo_sacchariphagus|ukzn0167|
-----------TTATTTTTGGAATTTGAGCTGGAATAATTGGAACATCCCTTAGACTTTTAATTCGAGCTGAATTAGGAA
ATCCAGGTTCATTAATTGGAGATGATCAAATTTATAATACTATTGTTACAGCCCATGCATTTATTATAATTTTTTTATA
GTAATACCAATTATAATCGGAGGATTTGGAAATTGATTAATTCCATTAATATTAGGAGCTCCTGATATAGCCTTTCCTCG
TTTAAATAATATAAGATTTTGATTATTACCCCCTTCTTTAACTCTTTTAATTTCTAGAAGAATTGTTGAAAATGGAGCAG
GAACTGGATGAACAGTCTACCCCCCCCTATCTTCCAATATTTCACATGCTGGAAGTTCAGTAGATTTAGCCATCTTTTCT
CTTCATTTAGCAGGAATTTCTTCAATTTTAGGAGCTATTAATTTTATTACTACAATTATTAATATGCGAATTAATGGATT
ATTATTTGATCAAATACCATTATTTGTTTGATCTGTTGGTATTACAGCATTACTTCTTCTTCTCTCTTTACCAGTATTAG

CAGGTGCTATTACCATACTATTAACTGACCGAAATTTAAATACATCTTTTTTGATCCAGCTGGAGGAGGTGATCCAATT
TTATATCAACATTTATTT---------
>LSTEM253-18|Chilo_sacchariphagus|ukzn0269|

--------------GATTTTGATTATTACCCCCTTCTTTAACTCTTTTAATTTCTAGAAGAAYTGTTGAAAATGGAGCAG
GAACTGGATGAACAGTCTACCCCCCCCTATCTTCCAATATTTCACATGCTGGAAGTTCAGTAGATTTAGCCATCTTTTCT
CTTCATTTAGCAGGAATTTCTTCAATTTTAGGAGCTATTAATTTTATTACTACAATTATTAATATGCGAATTAATGGATT
ATTATTTGATCAAATACCATTATTTGTTTGATCTGTTGGTATTACAGCATTACTTCTTCTTCTCTCTTTACCAGTATTAG
CAGGTGCTATTACCATACTATTAACTGACCGAAATTTAAATACATCTTTTTTGATCCAGCTGGAGGAGGTGATCCAATT
TTATATCAACATTTATTTTGATTTTTT
>LSTEM254-18|Chilo_suppressalis|ukzn0332|
-----------TTATTTTTGGTATTTGAGCAGGTATAATTGGAACATCTCTTAGACTTTTAATTCGTGCTGAATTAGGAA
CTCCAGGATCTTTAATTGGAGATGATCAAATTTATAATACCATTGTTACGGCTCATGCATTTATTATAATTTTTTTATA
GTTATACCAATTATAATTGGTGGATTTGGAAATTGATTAGTACCTTTAATATTAGGGGCTCCTGATATAGCTTTCCCACG
AATAAATAATATAAGATTTTGAATATTACCCCCCTCATTAACTTTACTAATTTCTAGAAGAATTGTTGAAAATGGAGCTG
GAACAGGTTGAACAGTGTACCCCCCACTATCATCTAATATTGCTCACGCTGGAAGTTCAGTAGATTTAGCAATTTTCTCT
TTACATTTAGCTGGAATTTCTTCAATTCTAGGTGCTATTAATTTTATTACTACGATTATTAATATACGAATTAATGGTCT
TTCATTTGATCAAATACCTTTATTTGTTTGATCCGTAGGTATTACAGCTTTATTATTACTTCTATCTCTACCAGTATTAG
CTGGAGCAATTACAATATTATTAACCGATCGAAATTTAAATACATCTTTTTTGATCCTGCTGGTGGTGGAGATCCAATT
CTTTACCAACATTTATTT---------
>LSTEM255-18|Eoreuma_loftini|ukzn0362|
AACTTTATATTTTATTTTTGGAATTTGAGCTGGAACAGTAGGAACATCTTTAAGTTTATTAATTCGAGCTGAATTAGGAA
ACCCAGGCTCTTTAATTGGAGATGATCAAATTTATAATACTATTGTTACAGCCCATGCATTTATTATAATTTTTTTATA
GTAATACCTATTATAATTGGAGGATTTGGAAACTGATTAGTACCTTTAATATTAGGAGCTCCTGATATAGCATTCCCACG
AATAAATAATATAAGATTTTGATTATTACCTCCATCTTTAACACTACTAATTTCAAGAAGTATTGTTGAAAATGGAGCTG
GAACAGGATGAACAGTTTATCCCCCTCTTTCTTCTAATATTGCTCATGGAGGTAGATCTGTAGATCTAGCAATTTTTTCT
CTTCATTTAGCTGGAATTTCCTCAATTTTAGGAGCTATTAATTTTATTACAACAATTATTAATATACGAATTAATAATTT
ATCCTTTGATCAAATACCTTTATTTGTTTGATCAGTAGGTATTACAGCTTTACTTCTTCTTCTTTCTTTACCTGTATTAG
CTGGAGCTATTACTATATTATTAACAGATCGAAATTTAAATACTTCCTTTTTTGATCCTGCAGGGGGTGGAGATCCAATT
CTTTATCAACATTTATTT---------
>LSTEM256-18|Eoreuma_loftini|ukzn0363|
AACTTTATATTTTATTTTTGGAATTTGAGCTGGAACAGTAGGAACATCTTTAAGTTTATTAATTCGAGCTGAATTAGGAA
ACCCAGGCTCTTTAATTGGAGATGATCAAATTTATAATACTATTGTTACAGCCCATGCATTTATTATAATTTTTTTATA
GTAATACCTATTATAATTGGGGGATTTGGAAATTGATTAGTACCTTTAATATTAGGAGCTCCTGATATAGCATTCCCACG
AATAAATAATATAAGATTTTGATTATTACCCCCATCTTTAACACTACTAATTTCAAGAAGTATTGTTGAAAATGGAGCTG
GAACAGGATGAACAGTTTATCCCCCTCTTTCTTCTAATATTGCTCATGGAGGTAGATCTGTAGATCTAGCAATTTTTTCT
CTTCATTTAGCTGGAATTTCCTCAATTTTAGGAGCTATTAATTTTATTACAACAATTATTAATATACGAATTAATAATTT
ATCCTTTGATCAAATACCTTTATTTGTTTGATCAGTAGGTATTACAGCTTTACTTCTTCTTCTTTCTTTACCAGTATTAG
CTGGAGCTATTACTATATTATTAACAGATCGAAATTTAAATACTTCCTTTTTTGATCCTGCAGGGGGTGGAGATCCAATT
CTTTATCAACATTTATTT---------
>LSTEM257-18|Diatraea_saccharalis|ukzn0364|
AACTTTATATTTTATTTTTGGAATTTGAGCAGGAATATTAGGAACTTCCTTAAGCTTATTAATTCGAGCAGAATTAGGAA
CATCTAACTCTTTAATTGGAGATGATCAAATTTATAATACAATTGTAACAGCTCATGCTTTCATTATAATTTTTTTATA
GTTATACCTATTATAATTGGAGGATTTGGAAATTGATTAGTACCTTTAATATTAGGAGCCCCCGATATAGCTTTCCCACG
AATAAATAATATAAGATTTTGACTATTACCCCCATCTTTAACTCTACTAATTTCTAGAAGAATTGTAGAAAATGGAGCAG
GGACAGGGTGAACAGTTTATCCCCCACTATCATCCAATATTGCCCATGGGGGAAGATCTGTAGATTTAGCAATTTTCTCC
TTACATTTAGCTGGAATTTCCTCAATTTTAGGGGCTATCAATTTCATCACTACCATTATTAATATACGAATTAATAATCT
ATCATTTGATCAAATACCCTTATTTATTTGATCAGTGGGTATTACAGCATTACTTTTATTACTTTCTTTACCAGTATTAG
CTGGAGCCATTACTATATTACTAACAGATCGAAATTTAAATACCTCCTTCTTTGATCCAGCGGGGGGAGGAGATCCTATT
CTATATCAACATTTATTT---------
>LSTEM258-18|Diatraea_saccharalis|ukzn0365|
AACTTTATATTTTATTTTTGGAATTTGAGCAGGAATATTAGGAACTTCCTTAAGCTTATTAATTCGAGCAGAATTAGGAA
CATCTAACTCTTTAATTGGAGATGATCAAATTTATAATACAATTGTAACAGCTCATGCTTTCATTATAATTTTTTTATA
GTTATACCTATTATAATTGGAGGATTTGGAAATTGATTAGTACCTTTAATATTAGGAGCCCCCGATATAGCTTTCCCACG
AATAAATAATATAAGATTTTGACTATTACCCCCATCTTTAACTCTACTAATTTCTAGAAGAATTGTAGAAAATGGAGCAG

GGACAGGGTGAACAGTTTATCCCCCATTATCATCCAATATTGCCCATGGGGGAAGATCTGTAGATTTAGCAATTTTCTCC
TTACATTTAGCTGGAATTTCCTCAATTTTAGGGGCTATCAATTTCATCACTACCATTATTAATATACGAATTAATAATCT
ATCATTTGATCAAATACCCTTATTTATTTGATCAGTGGGTATTACAGCATTACTTTTATTACTTTCTTTACCAGTATTAG
CTGGAGCCATTACTATATTACTAACAGATCGAAATTTAAATACCTCCTTCTTTGATCCAGCGGGGGGAGGAGATCCTATT
CTATATCAACATTTATTT---------
>LSTEM259-18|Chilo_suppressalis|ukzn0366|
AACTTTATATTTTATTTTTGGTATTTGAGCAGGTATAATTGGAACATCTCTTAGACTTTTAATTCGTGCTGAATTAGGAA
CTCCAGGATCTTTAATTGGAGATGATCAAATTTATAATACCATTGTTACGGCTCATGCATTTATTATAATTTTTTTTATA
GTTATACCAATTATAATTGGTGGATTTGGAAATTGATTAGTACCTTTAATATTAGGGGCTCCTGATATAGCTTTCCCACG
AATAAATAATATAAGATTTTGAATATTACCCCCCTCATTAACTTTACTAATTTCTAGAAGAATTGTTGAAAATGGAGCTG
GAACAGGTTGAACAGTGTACCCCCCACTATCATCTAATATTGCTCACGCTGGAAGTTCAGTAGATTTAGCAATTTTCTCT
TTACATTTAGCTGGAATTTCTTCAATTCTAGGTGCTATTAATTTTATTACTACGATTATTAATATACGAATTAATGGTCT
TTCATTTGATCAAATACCTTTATTTGTTTGATCCGTAGGTATTACAGCTTTATTATTACTTCTATCTCTACCAGTATTAG
CTGGAGCAATTACAATATTATTAACCGATCGAAATTTAAATACATCTTTTTTTGATCCTGCTGGTGGTGGAGATCCAATT
CTTTACCAACATTTATTT---------
>LSTEM260-18|Chilo_tumidicostalis|ukzn0367|
AACTTTATATTTTATTTTTGGAATTTGAGCTGGAATAATTGGTACATCTTTAAGACTCTTAATTCGAGCTGAATTAGGAA
CCCCAGGATCTTTAATTGGAGATGATCAAATTTATAATACTATTGTCACAGCTCATGCATTTATTATAATTTTTTTCATA
GTAATACCAATTATAATTGGTGGATTTGGAAATTGATTGGTACCTTTAATATTAGGAGCTCCAGATATAGCTTTCCCACG
AATAAATAATATAAGATTTTGATTATTACCCCCCTCATTAACACTATTAATTTCAAGTAGAATTGTAGAAAATGGAGCAG
GAACAGGATGAACAGTGTACCCCCCACTATCATCTAATATTGCTCATGCTGGAAGCTCTGTTGATTTAGCAATTTTCTCA
TTACATCTAGCTGGTATTTCCTCTATTTTAGGAGCTATTAATTTTATTACAACAATTATTAATATACGAATTAATGGGTT
ATCATTTGATCAGATACCTTTATTTGTATGATCTGTAGGAATTACAGCTTTATTACTATTACTTTCATTACCAGTTTTAG
CAGGAGCTATTACTATATTATTAACAGATCGAAATTTAAATACTTCCTTTTTTGACCCTGCTGGAGGGGGAGATCCTATT
CTCTACCAACATTTATTT---------
>LSTEM261-18|Chilo_tumidicostalis|ukzn0368|
AACTTTATATTTTATTTTTGGAATTTGAGCTGGAATAATTGGTACATCTTTAAGACTCTTAATTCGAGCTGAATTAGGAA
CCCCAGGATCTTTAATTGGAGATGATCAAATTTATAATACTATTGTCACAGCTCATGCATTTATTATAATTTTTTTCATA
GTAATACCAATTATAATTGGTGGATTTGGAAATTGATTGGTACCTTTAATATTAGGAGCTCCAGATATAGCTTTCCCACG
AATAAATAATATAAGATTTTGATTATTACCCCCCTCATTAACACTATTAATTTCAAGTAGAATTGTAGAAAATGGAGCAG
GAACAGGATGAACAGTGTACCCCCCACTATCATCTAATATTGCTCATGCTGGAAGCTCTGTTGATTTAGCAATTTTCTCA
TTACATCTAGCTGGTATTTCCTCTATTTTAGGAGCTATTAATTTTATTACAACAATTATTAATATACGAATTAATGGGTT
ATCATTTGATCAGATACCTTTATTTGTATGATCTGTAGGAATTACAGCTTTATTACTATTACTTTCATTACCAGTTTTAG
CAGGAGCTATTACTATATTATTAACAGATCGAAATTTAAATACTTCCTTTTTTGACCCTGCTGGAGGGGGAGATCCTATT
CTCTACCAACATTTATTT---------
>LSTEM262-18|Chilo_tumidicostalis|ukzn0369|
AACTTTATATTTTATTTTTGGAATTTGAGCTGGAATAATTGGTACATCTTTAAGACTCTTAATTCGAGCTGAATTAGGAA
CCCCAGGATCTTTAATTGGAGATGATCAAATTTATAATACTATTGTCACAGCTCATGCATTTATTATAATTTTTTTCATA
GTAATACCAATTATAATTGGTGGATTTGGAAATTGATTGGTACCTTTAATATTAGGAGCTCCAGATATAGCTTTCCCACG
AATAAATAATATAAGATTTTGATTATTACCCCCCTCATTAACACTATTAATTTCAAGTAGAATTGTAGAAAATGGAGCAG
GAACAGGATGAACAGTGTACCCCCCACTATCATCTAATATTGCTCATGCTGGAAGCTCTGTTGATTTAGCAATTTTCTCA
TTACATCTAGCTGGTATTTCCTCTATTTTAGGAGCTATTAATTTTATTACAACAATTATTAATATACGAATTAATGGATT
ATCATTTGATCAGATACCTTTATTTGTATGATCTGTAGGAATTACAGCTTTATTACTATTACTTTCATTACCAGTTTTAG
CAGGAGCTATTACTATATTATTAACAGATCGAAATTTAAATACTTCCTTTTTTGACCCTGCTGGAGGGGGAGATCCTATT
CTCTACCAACATTTATTT---------
>LSTEM263-18|Chilo_tumidicostalis|ukzn0370|
AACTTTATATTTTATTTTTGGAATTTGAGCTGGAATAATTGGTACATCTTTAAGACTCTTAATTCGAGCTGAATTAGGAA
CCCCAGGATCTTTAATTGGAGATGATCAAATTTATAATACTATTGTCACAGCTCATGCATTTATTATAATTTTTTTCATA
GTAATACCAATTATAATTGGTGGATTTGGAAATTGATTGGTACCTTTAATATTAGGAGCTCCAGATATAGCTTTCCCACG
AATAAATAATATAAGATTTTGATTATTACCCCCCTCATTAACACTATTAATTTCAAGTAGAATTGTAGAAAATGGAGCAG
GAACAGGATGAACAGTGTACCCCCCACTATCATCTAATATTGCTCATGCTGGAAGCTCTGTTGATTTAGCGATTTTCTCA
TTACATCTAGCTGGTATTTCCTCTATTTTAGGAGCTATTAATTTTATTACAACAATTATTAATATACGAATTAATGGATT
ATCATTTGATCAGATACCTTTATTTGTGTGATCTGTAGGAATTACAGCTTTATTACTATTACTTTCATTACCAGTTTTAG
CAGGAGCTATTACTATATTATTAACAGATCGAAATTTAAATACTTCCTTTTTTGACCCTGCTGGAGGGGGAGATCCTATT
CTCTACCAACATTTATTT---------
>LSTEM264-18|Chilo_infuscatellus|ukzn0371|
AACTTTATATTTTATTTTTGGAATTTGAGCAGGAATAATTGGAACCTCTCTTAGACTTTTAATTCGAGCTGAATTAGGAA

CTCCAGGATCTTTAATTGGGGATGATCAAATTTATAATACTATTGTTACAGCTCATGCATTTATTATAATTTTTTTATA
GTAATACCAATTATAATCGGAGGATTTGGAAATTGATTAGTTCCTTTAATATTAGGAGCACCTGATATAGCTTTCCCACG
GATAAATAATATAAGTTTTGATTATTACCACCATCACTAACATTATTGATTTCTAGAAGAATTGTTGAAAATGGAGCAG
GAACTGGTTGAACTGTTTATCCTCCTTTATCTTCAAATATTGCTCATGGGGGAAGCTCTGTAGATTTAGCAATTTTTTCC
CTTCATTTAGCTGGTATTTCATCAATTTTAGGAGCTATTAATTTTATTACAACAATTATTAATATACGAGTTAATGGTCT
ATCATTTGATCAAATACCTTTATTTGTCTGATCTGTAGGAATTACAGCATTATTATTATTACTTTCTCTACCAGTATTAG
CAGGTGCTATTACTATACTACTAACTGATCGAAATTTAAATACATCTTTTTTTGATCCTGCAGGAGGGGGAGATCCAATC
CTCTACCAACATTTATTT---------
>LSTEM265-18|Chilo_partellus|ukzn0372|
AACTTTATATTTTATTTTTGGAATTTGAGCAGGAATAATTGGGACATCCCTTAGATTATTAATTCGTGCAGAATTAGGAA
CTCCTGGATCTTTAATTGGAGATGATCAAATTTATAATACTATTGTAACAGCACACGCATTTATTATAATTTTTTTTATA
GTTATACCAATTATAATTGGTGGATTTGGAAATTGATTAGTACCTTTAATATTAGGAGCCCCAGATATAGCTTTCCCACG
AATAAATAATATAAGATTTTGATTATTACCACCATCATTAACTTTATTAATTTCTAGAAGAATTGTTGAAAATGGAGCTG
GAACAGGATGAACAGTGTACCCCCCACTATCATCTAATATTGCTCATGCTGGAAGTTCAGTAGATTTAGCAATTTTTTCT
TTACATTTAGCTGGTATTTCATCAATTCTCGGTGCTATTAATTTTATTACAACAATTATTAATATACGAATTAATGGATT
ATCTTTTGATCAAATACCATTATTTGTTTGATCTGTAGGTATTACAGCTTTATTATTATTACTTTCTTTACCTGTTTTAG
CTGGAGCTATTACTATATTATTAACAGATCGAAATTTAAATACATCCTTTTTCGATCCTGCTGGAGGAGGAGATCCTATT
CTTTATCAACACTTATTT---------
>LSTEM266-18|Chilo_partellus|ukzn0373|
AACTTTATATTTTATTTTTGGAATTTGAGCAGGAATAATTGGGACATCCCTTAGATTATTAATTCGTGCAGAATTAGGAA
CTCCTGGATCTTTAATTGGAGATGATCAAATTTATAATACTATTGTAACAGCACACGCATTTATTATAATTTTTTTTATA
GTTATACCAATTATAATTGGTGGATTTGGAAATTGATTAGTACCTTTAATATTAGGAGCCCCAGATATAGCTTTCCCACG
AATAAATAATATAAGATTTTGATTATTACCACCATCATTAACTTTATTAATTTCTAGAAGAATTGTTGAAAATGGAGCTG
GAACAGGATGAACAGTGTACCCCCCACTATCATCTAATATTGCTCATGCTGGAAGTTCAGTAGATTTAGCAATTTTTTCT
TTACATTTAGCTGGTATTTCATCAATTCTCGGTGCTATTAATTTTATTACAACAATTATTAATATACGAATTAATGGATT
ATCTTTTGATCAAATACCATTATTTGTTTGATCTGTAGGTATTACAGCTTTATTATTATTACTTTCTTTACCTGTTTTAG
CTGGAGCTATTACTATATTATTAACAGATCGAAATTTAAATACATCCTTTTTCGATCCTGCTGGAGGAGGAGATCCTATT
CTTTATCAACACTTATTT---------
>LSTEM267-18|Cnaphalocrocis_patnalis|ukzn0528|
TACTTTATATTTTATTTTTGGAATTTGAGCAGGAATAGTTGGAACATCATTAAGTTTACTAATTCGAGCTGAATTAGGAA
ATCCTGGTTCATTAATTGGAGATGATCAAATTTATAATACCATTGTAACAGCTCATGCATTTATTATAATTTTTTTTATA
GTAATACCAATTATAATTGGAAGATTCGGTAATTGATTAGTACCTTTAATATTAGGAGCTCCAGATATAGCTTTTCCACG
AATAAATAACATAAGATTTTGATTATTACCCCCATCTTTAACTTTATTAATTTCAAGAAGAATTGTAGAAAATGGAGCAG
GAACAGGATGAACAGTTTACCCCCCACTTTCATCTAATATTGCTCATGGAGGAAGATCTGTTGATTTAGCTATTTTTTCC
TTACATTTAGCTGGTATTTCATCTATTTTAGGAGCAATTAACTTTATTACAACAATTATTAATATACGAATTAATGGTTT
ATCTTTTGATCAAATACCTTTATTTGTATGAGCTGTAGGAATTACAGCTTTATTATTACTTCTTTCACTACCAGTTTTAG
CAGGTGCTATTACTATATTATTAACTGATCGTAATTTAAATACATCATTTTTTGATCCTGCTGGAGGAGGAGATCCTATT
TTATATCAACATTTATTT---------
>LSTEM268-18|Cnaphalocrocis_medinalis|ukzn0529|
TACTTTATATTTTATTTTTGGAATTTGAGCAGGAATAGTTGGAACATCATTAAGTTTATTAATTCGAGCTGAATTGGGAA
ATCCAGGATCATTAATTGGAGATGATCAAATTTATAATACTATTGTAACAGCTCATGCATTCATTATAATTTTTTTTATA
GTAATACCTATTATAATTGGAGGATTTGGAAATTGATTAGTGCCTTTAATATTAGGAGCCCCTGATATAGCTTTTCCACG
TATAAATAATATAAGATTTTGATTACTCCCCCCTTCATTAACTTTATTAATTTCAAGAAGAATCGTAGAAAATGGAGCAG
GAACAGGATGAACAGTTTACCCCCCACTTTCATCTAATATTGCTCATGGTGGAAGTTCTGTTGATTTAGCTATTTTTTCC
CTACATTTAGCAGGAATTTCATCAATTTTAGGAGCAATTAATTTTATTACAACAATTATTAATATACGAATTAATGGTTT
ATCTTTTGATCAAATACCTCTTTTTGTATGAGCTGTTGGAATTACAGCTTTATTACTTCTTCTTTCTTTACCAGTTTTAG
CAGGTGCTATTACTATATTATTAACTGATCGAAATTTAAATACATCATTTTTTGACCCTGCTGGAGGTGGAGATCCAATT
TTATATCAACATTTATTT---------
>LSTEM269-18|Rivula_atimeta|ukzn0531|
AACTTTATATTTTATTTTTGGAATTTGAGCTGGAATAGTAGGAACTTCATTAAGACTTCTAATTCGAGCAGAATTAGGAA
CCCCTGGGTCTTTAATTGGAGATGATCAAATTTACAATACTATTGTTACAGCACATGCTTTTATTATAATTTTTTTTATA
GTTATACCTATTATAATTGGAGGATTTGGTAATTGATTAGTTCCTCTAATATTAGGAGCTCCTGATATAGCTTTTCCTCG
TATAAATAATATAAGTTTTTGACTTTTACCTCCTTCACTTACTCTCTTAATTTCAAGAAGAATTGTAGAAAACGGAGCAG
GAACAGGATGAACAGTTTACCCCCCACTTTCATCTAATATTGCTCATGGAGGAAGATCTGTTGATTTAGCTATTTTTTCT
CTTCATTTAGCAGGAATTTCTTCTATTTTAGGAGCAATTAATTTTATTACAACAATCATTAATATACGATTAAATAATTT
ATCTTTTGATCAAATACCTTTATTTATTTGAGCTGTTGGAATTACTGCTTTTCTTTTACTTCTCTCATTACCGGTATTAG
CTGGAGCAATTACAATATTATTAACAGATCGAAATTTAAATACATCTTTTTTTGATCCCGCTGGAGGAGGAGATCCTATT

CTTTATCAACATTTATTT---------
>LSTEM270-18|Scirpophaga_incertulas|ukzn0532|
TACTTTATATTTTATTTTTGGAATTTGAGCTGGAATAGTAGGAACTTCTTTAAGCTTACTTATTCGAGCTGAATTAGGAA
CTTCTGGATCCTTAATTGGAGATGATCAAATCTATAATACTATTGTCACAGCCCATGCCTTTATTATAATTTTTTTATA
GTTATACCCATTATAATTGGAGGATTTGGAAATTGATTAGTCCCCCTAATATTAGGAGCTCCAGATATAGCTTTCCCCCG
ACTAAATAATATAAGATTCTGATTATTACCCCCCTCTTTAACACTCCTCATTTCTAGAAGAATTGTAGAAAATGGAGCTG
GAACAGGATGAACTGTTTACCCACCCCTATCATCCAATATTGCTCATGGAGGAACATCAGTAGATTTAGCTATTTTTTCT
CTGCACCTAGCAGGAATTTCATCTATTTTAGGAGCTATTAATTTTATTACAACTATTATTAATATACGAATTAATGGATT
ATCATTTGACCAAATACCTCTATTTGTGTGAGCTGTTGGTATTACAGCTCTTCTTTTACTTCTCTCTCTCCCAGTTTTAG
CCGGAGCTATTACCATATTACTAACAGATCGAAACTTAAATACATCTTTTTTGACCCAGCTGGGGGAGGAGATCCAATT
TTATACCAACATTTATTT---------
>LSTEM271-18|Chilo_infuscatellus|ukzn0535|
AACTTTATATTTTATTTTTGGAATTTGAGCAGGAATAATTGGAACTTCCCTTAGACTTTTAATTCGAGCTGAATTAGGGA
CTCCAGGATCTTTAATTGGAGATGATCAAATTTATAATACTATTGTTACAGCTCATGCATTTATTATAATTTTTTTTATA
GTAATACCAATTATAATTGGAGGATTTGGAAATTGATTAGTTCCTTTAATACTAGGGGCACCTGATATAGCTTTTCCACG
AATAAATAATATAAGCTTTTGATTATTACCACCATCATTAACATTATTAATTTCTAGAAGAATTGTTGAAAATGGGGCAG
GGACTGGTTGAACTGTTTATCCCCCTTTATCCTCAAATATTGCTCATGGGGGAAGCTCTGTAGATTTAGCAATTTTTTCC
CTTCACTTAGCGGGTATTTCATCAATTTTAGGAGCTATTAATTTTATTACAACAATTATTAATATACGAGTTAATGGTCT
ATCATTTGATCAAATACCTTTATTTGTTTGATCTGTAGGTATTACAGCACTTTTATTATTACTTTCTCTACCAGTATTAG
CAGGTGCTATTACTATACTACTAACTGATCGAAATTTAAATACATCTTTTTTGACCCTGCTGGAGGGGGGGATCCAATC
CTTTATCAACATTTATTTTGATTTTTG
>LSTEM272-18|Chilo_sp_AM16|ukzn0722|
----------TTTATTTTTGGAATTTGAGCAGGAATAATTGGAACATCACTTAGACTTTTAATTCGAGCTGAATTAGGAA
CTCCAGGATCTTTAATTGGTGATGATCAAATTTACAATACTATTGTTACAGCTCACGCATTTATTATAATTTTTTTTATA
GTCATACCAATTATAATTGGAGGATTTGGAAATTGATTAGTACCTCTAATATTAGGAGCTCCTGATATAGCTTTTCCACG
AATAAATAATATAAGATTTTGATTACTCCCCCCATCATTAACTTTACTAATTTTTAGAAGAATTGTAGAAACTGGAGCCG
GAACAGGATGAACAGTTTACCCCCCACTATCATCTAATATCGCACATGCTGGAAGTTCAGTAGATTTAGCAATTTTCTCC
CTCCATTTAGCTGGAATTTCTTCTATTTTAGGAGCTATTAACTTTATTACAACAATTATTAATATACGAATTAATGGATT
ATCATTTGATCAAATACCATTATTTGGTTGATCAGTTGGTATTACAGCTTTA----------------------------
----

>LSTEM273-18|Chilo_orichalcociliellus|ww03981|
AACTTTATATTTTATTTTTGGAATTTGAGCAGGAATAATTGGAACATCACTTAGACTCCTAATTCGAGCTGAATTAGGAA
CCCCTGGATCTTTAATTGGTGATGATCAAATTTATAATACTATTGTTACAGCTCATGCATTTATTATAATTTTTTTTATA
GTTATACCAATTATAATTGGAGGATTTGGAAATTGATTAGTACCTTTAATGTTAGGAGCTCCTGATATAGCCTTCCCACG
AATAAATAATATAAGATTTTGATTACTACCCCCATCACTAACTTTATTAATTTCTAGAAGAATTGTTGAAAATGGAGCTG
GAACTGGGTGAACAGTTTATCCCCCACTTTCATCCAATATTGCCCATGGTGGAAGTTCAGTAGATCTAGCAATTTTTTCT
CTTCATTTAGCTGGAATTTCCTCAATTTTAGGTGCTATTAATTTTATTACAACAATCATTAATATACGAATTAATGGATT
ATCATTTGATCAAATACCTTTATTTGTTTGATCTGTTGGTATTACAGCCTTATTATTATTACTTTCATTACCTGTTTTAG
CAGGAGCTATTACTATATTATTAACTGATCGAAATTTAAATACATCATTTTTTGATCCTGCTGGTGGAGGAGATCCAATT
CTTTATCAACATTTATTTTGATTTTTT
>LSTEM274-18|Chilo_orichalcociliellus|ww03982|
AACTTTATATTTTATTTTTGGAATTTGAGCAGGAATAATTGGAACATCACTTAGACTCCTAATTCGAGCTGAATTAGGAA
CCCCTGGATCTTTAATTGGTGATGATCAAATTTATAATACTATTGTTACAGCTCATGCATTTATTATAATTTTTTTTATA
GTTATACCAATTATAATTGGAGGATTTGGAAATTGATTAGTACCTTTAATGTTAGGAGCTCCTGATATAGCCTTCCCACG
AATAAATAATATAAGATTTTGATTACTACCCCCATCACTAACTTTATTAATTTCTAGAAGAATTGTTGAAAATGGAGCTG
GAACTGGGTGAACAGTTTATCCCCCACTTTCATCCAATATTGCCCATGGTGGGAGTTCAGTAGATCTAGCAATTTTTTCT
CTTCATTTAGCTGGAATTTCCTCAATTTTAGGTGCTATTAATTTTATTACAACAATCATTAATATACGAATTAATGGATT
ATCATTTGATCAAATACCTTTATTTGTTTGATCTGTTGGTATTACAGCCTTATTATTATTACTTTCATTACCTGTTTTAG
CAGGAGCTATTACTATATTATTAACTGATCGAAATTTAAATACATCATTTTTTGATCCTGCTGGTGGAGGAGATCCAATT
CTTTATCAACATTTATTTTGATTTTTT
>LSTEM275-18|Chilo_orichalcociliellus|ww03983|
AACTTTATATTTTATTTTTGGAATTTGAGCAGGAATAATTGGAACATCACTTAGACTCCTAATTCGAGCTGAATTGGGAA
CCCCTGGATCTTTAATTGGTGATGATCAAATTTATAATACTATTGTTACAGCTCATGCATTTATTATAATTTTTTTTATA
GTTATACCAATTATAATTGGAGGATTTGGAAATTGATTAGTACCTTTAATGTTAGGAGCTCCTGATATAGCCTTCCCACG
AATAAATAATATAAGATTTTGATTACTACCCCCATCACTAACTTTATTAATTTCTAGAAGAATTGTTGAAAATGGAGCTG
GAACTGGGTGAACAGTTTATCCCCCACTTTCATCCAATATTGCCCATGGTGGGAGTTCAGTAGATCTAGCAATTTTTTCT

CTTCATTTAGCTGGAATTTCCTCAATTTTAGGTGCTATTAATTTTATTACAACAATCATTAATATACGAATTAATGGATT
ATCATTTGATCAAATACCTTTATTTGTTTGATCTGTTGGTATTACAGCCTTATTATTATTACTTTCATTACCTGTTTTAG
CAGGAGCTATTACTATATTATTAACTGATCGAAATTTAAATACATCATTTTTTGATCCTGCTGGTGGAGGAGATCCAATT
CTTTATCAACATTTATTTTGATTTTTT
>LSTEM276-18|Chilo_orichalcociliellus|ww03985|
AACTTTATATTTTATTTTTGGAATTTGAGCAGGAATAATTGGAACATCACTTAGACTCCTAATTCGAGCTGAATTAGGAA
CCCCTGGATCTTTAATTGGTGATGATCAAATTTATAATACTATTGTTACAGCTCATGCATTTATTATAATTTTTTTTATA
GTTATACCAATTATAATTGGAGGATTTGGAAATTGATTAGTACCTTTAATGTTAGGAGCTCCTGATATAGCCTTCCCACG
AATAAATAATATAAGATTTTGATTACTACCCCCATCACTAACTTTATTAATTTCTAGAAGAATTGTTGAAAATGGAGCTG
GAACTGGGTGAACAGTTTATCCCCCACTTTCATCCAATATTGCCCATGGTGGGAGTTCAGTAGATCTAGCAATTTTTTCT
CTTCATTTAGCTGGAATTTCCTCAATTTTAGGTGCTATTAATTTTATTACAACAATCATTAATATACGAATTAATGGATT
ATCATTTGATCAAATACCTTTATTTGTTTGATCTGTTGGTATTACAGCCTTATTATTATTACTTTCATTACCTGTTTTAG
CAGGAGCTATTACTATATTATTAACTGATCGAAATTTAAATACATCATTTTTTGATCCTGCTGGTGGAGGAGATCCAATT
CTTTATCAACATTTATTTTGATTTTTT
>LSTEM277-18|Chilo_orichalcociliellus|ww03986|
AACTTTATATTTTATTTTTGGAATTTGAGCAGGAATAATTGGAACATCACTTAGACTCCTAATTCGAGCTGAATTAGGAA
CCCCTGGATCTTTAATTGGTGATGATCAAATTTATAATACTATTGTTACAGCTCATGCATTTATTATAATTTTTTTTATA
GTTATACCAATTATAATTGGAGGATTTGGAAATTGATTAGTACCTTTAATGTTAGGAGCTCCTGATATAGCCTTCCCACG
AATAAATAATATAAGATTTTGATTACTACCCCCATCACTAACTTTATTAATTTCTAGAAGAATTGTTGAAAATGGAGCTG
GAACTGGGTGAACAGTTTATCCCCCACTTTCATCCAATATTGCCCATGGTGGAAGTTCAGTAGATCTAGCAATTTTTTCT
CTTCATTTAGCTGGAATTTCCTCAATTTTAGGTGCTATTAATTTTATTACAACAATCATTAATATACGAATTAATGGATT
ATCATTTGATCAAATACCTTTATTTGTTTGATCTGTTGGTATTACAGCCTTATTATTATTACTTTCATTACCTGTTTTAG
CAGGAGCTATTACTATATTATTAACTGATCGAAATTTAAATACATCATTTTTTGATCCTGCTGGTGGAGGAGATCCAATT
CTTTATCAACATTWATTTTGATTTTTT
>LSTEM278-18|Chilo_orichalcociliellus|ww03987|
AACTTTATATTTTATTTTTGGAATTTGAGCAGGAATAATTGGAACATCACTTAGACTCCTAATTCGAGCTGAATTAGGAA
CCCCTGGATCTTTAATTGGTGATGATCAAATTTATAATACTATTGTTACAGCTCATGCATTTATTATAATTTTTTTTATA
GTTATACCAATTATAATTGGAGGATTTGGAAATTGATTAGTACCTTTAATGTTAGGAGCTCCTGATATAGCCTTCCCACG
AATAAATAATATAAGATTTTGATTACTACCCCCATCACTAACTTTATTAATTTCTAGAAGAATTGTTGAAAATGGAGCTG
GAACTGGGTGAACAGTTTATCCCCCACTTTCATCCAATATTGCCCATGGTGGGAGTTCAGTAGATCTAGCAATTTTTTCT
CTTCATTTAGCTGGAATTTCCTCAATTTTAGGTGCTATTAATTTTATTACAACAATCATTAATATACGAATTAATGGATT
ATCATTTGATCAAATACCTTTATTTGTTTGATCTGTTGGTATTACAGCCTTATTATTATTACTTTCATTACCTGTTTTAG
CAGGAGCTATTACTATATTATTAACTGATCGAAATTTAAATACATCATTTTTTGATCCTGCTGGTGGAGGAGATCCAATT
CTTTATCAACATTTATTTTGATTTTTT
>LSTEM279-18|Chilo_orichalcociliellus|ww03988|
AACTTTATATTTTATTTTTGGAATTTGAGCAGGAATAATTGGAACATCACTTAGACTCCTAATTCGAGCTGAATTAGGAA
CCCCTGGATCTTTAATTGGTGATGATCAAATTTATAATACTATTGTTACAGCTCATGCATTTATTATAATTTTTTTTATA
GTTATACCAATTATAATTGGAGGATTTGGAAATTGATTAGTACCTTTAATGTTAGGAGCTCCTGATATAGCCTTCCCACG
AATAAATAATATAAGATTTTGATTACTACCCCCATCACTAACTTTATTAATTTCTAGAAGAATTGTTGAAAATGGAGCTG
GAACTGGGTGAACAGTTTATCCCCCACTTTCATCCAATATTGCCCATGGTGGGAGTTCAGTAGATCTAGCAATTTTTTCT
CTTCATTTAGCTGGAATTTCCTCAATTTTAGGTGCTATTAATTTTATTACAACAATCATTAATATACGAATTAATGGATT
ATCATTTGATCAAATACCTTTATTTGTTTGATCTGTTGGTATTACAGCCTTATTATTATTACTTTCATTACCTGTTTTAG
CAGGAGCTATTACTATATTATTAACTGATCGAAATTTAAATACATCATTTTTTGATCCTGCTGGTGGAGGAGATCCAATT
CTTTATCAACATTTATTTTGATTTTTT
>LSTEM280-18|Chilo_orichalcociliellus|ww03989|
AACTTTATATTTTATTTTTGGAATTTGAGCAGGAATAATTGGAACATCACTTAGACTCCTAATTCGAGCTGAATTAGGAA
CCCCTGGATCTTTAATTGGTGATGATCAAATTTATAATACTATTGTTACAGCTCATGCATTTATTATAATTTTTTTTATA
GTTATACCAATTATAATTGGAGGATTTGGAAATTGATTAGTACCTTTAATGTTAGGAGCTCCTGATATAGCCTTCCCACG
AATAAATAATATAAGATTTTGATTACTACCCCCATCACTAACTTTATTAATTTCTAGAAGAATTGTTGAAAATGGAGCTG
GAACTGGGTGAACAGTTTATCCCCCACTTTCATCCAATATTGCCCATGGTGGGAGTTCAGTAGATCTAGCAATTTTTTCT
CTTCATTTAGCTGGAATTTCCTCAATTTTAGGTGCTATTAATTTTATTACAACAATCATTAATATACGAATTAATGGATT
ATCATTTGATCAAATACCTTTATTTGTTTGATCTGTTGGTATTACAGCCTTATTATTATTACTTTCATTACCTGTTTTAG
CAGGAGCTATTACTATATTATTAACTGATCGAAATTTAAATACATCATTTTTTGATCCTGCTGGTGGAGGAGATCCAATT
CTTTATCAACATTTATTTTGATTTTTT
>LSTEM281-18|Chilo_orichalcociliellus|ww03990|
----------------TTTGGAATTTGAGCAGGAATAATTGGAACATCACTTAGACTCCTAATTCGAGCTGAATTAGGAA
CCCCTGGATCTTTAATTGGTGATGATCAAATTTATAATACTATTGTTACAGCTCATGCATTTATTATAATTTTTTTTATA

GTTATACCAATTATAATTGGAGGATTTGGAAATTGATTAGTACCTTTAATGTTAGGAGCTCCTGATATAGCCTTCCCACG
AATAAATAATATAAGATTTTGATTACTACCCCCATCACTAACTTTATTAATTTCTAGAAGAATTGTTGAAAATGGAGCTG
GAACTGGGTGAACAGTTTATCCCCCACTTTCATCCAATATTGCCCATGGTGGGAGTTCAGTAGATCTAGCAATTTTTTCT
CTTCATTTAGCTGGAATTTCCTCAATTTTAGGTGCTATTAATTTTATTACAACAATCATTAATATACGAATTAATGGATT
ATCATTTGATCAAATACCTTTATTTGTTTGATCTGTTGGTATTACAGCCTTATTATTATTACTTTCATTACCTGTTTTAG
CAGGAGCTATTACTATATTATTAACTGATCGAAATTTAAATACATCATTTTTTGATCCTGCTGGTGGAGGAGATCCAATT
CTTTATCAACATTTATTTTGATTTTTT
>LSTEM282-18|Chilo_quirimbellus|ww03991|
AACTTTATATTTTATTTTTGGAATTTGAGCAGGAATAATTGGAACATCACTTAGACTTTTAATTCGAGCTGAATTAGGAA
CTCCAGGATCTTTAATTGGTGATGATCAAATTTATAATACTATTGTTACAGCTCATGCATTTATTATAATTTTTTTTATA
GTTATACCAATTATAATTGGTGGATTTGGAAATTGATTAGTACCTTTAATATTAGGAGCTCCCGATATGGCTTTTCCACG
AATAAATAATATAAGATTTTGATTACTTCCCCCATCATTAACTTTATTAATTTCTAGTAGAATCGTAGAAAACGGAGCCG
GAACAGGATGAACAGTTTACCCCCCACTCTCATCCAATATTGCACATGCTGGAAGTTCAGTAGATTTAGCAATTTTTTCT
CTCCACTTAGCTGGAATTTCTTCTATCTTAGGTGCAATTAACTTTATTACAACAATTATTAATATGCGAATTAATGGATT
ATCATTTGATCAAATACCATTATTTGTTTGATCCGTTGGTATTACAGCTTTATTATTATTACTTTCATTACCGGTTTTAG
CTGGTGCTATTACCATATTATTAACAGATCGAAATTTAAATACATCATTTTTTGATCCTGCTGGTGGGGGTGACCCAATT
CTTTATCAACACTTATTTTGATTTTTT
>LSTEM283-18|Chilo_orichalcociliellus|ww03992|
AACTTTATATTTTATTTTTGGAATTTGAGCAGGAATAATTGGAACATCACTTAGACTCCTAATTCGAGCTGAATTAGGAA
CCCCTGGATCTTTAATTGGTGATGATCAAATTTATAATACTATTGTTACAGCTCATGCATTTATTATAATTTTTTTTATA
GTTATACCAATTATAATTGGAGGATTTGGAAATTGATTAGTACCTTTAATGTTAGGAGCTCCTGATATAGCCTTCCCACG
AATAAATAATATAAGATTTTGATTACTACCCCCATCACTAACTTTATTAATTTCTAGAAGAATTGTTGAAAATGGAGCTG
GAACTGGGTGAACAGTTTATCCCCCACTTTCATCCAATATTGCCCATGGTGGAAGTTCAGTAGATCTAGCAATTTTTTCT
CTTCATTTAGCTGGAATTTCCTCAATTTTAGGTGCTATTAATTTTATTACAACAATCATTAATATACGAATTAATGGATT
ATCATTTGATCAAATACCTTTATTTGTTTGATCTGTTGGTATTACAGCCTTATTATTATTACTTTCATTACCTGTTTTAG
CAGGAGCTATTACTATATTATTAACTGATCGAAATTTAAATACATCATTTTTTGATCCTGCTGGTGGAGGAGATCCAATT
CTTTATCAACATTTATTTTGATTTTTT
>LSTEM284-18|Chilo_orichalcociliellus|ww03993|
AACTTTATATTTTATTTTTGGAATTTGAGCAGGAATAATTGGAACATCACTTAGACTCCTAATTCGAGCTGAATTAGGAA
CCCCTGGATCTTTAATTGGTGATGATCAAATTTATAATACTATTGTTACAGCTCATGCATTTATTATAATTTTTTTTATA
GTTATACCAATTATAATTGGAGGATTTGGAAATTGATTAGTACCTTTAATGTTAGGAGCTCCTGATATAGCCTTCCCACG
AATAAATAATATAAGATTTTGATTACTACCCCCATCACTAACTTTATTAATTTCTAGAAGAATTGTTGAAAATGGAGCTG
GAACTGGGTGAACAGTTTATCCCCCACTTTCATCCAATATTGCCCATGGTGGGAGTTCAGTAGATCTAGCAATTTTTTCT
CTTCATTTAGCTGGAATTTCCTCAATTTTAGGTGCTATTAATTTTATTACAACAATCATTAATATACGAATTAATGGGTT
ATCATTTGATCAAATACCTTTATTTGTTTGATCTGTTGGTATTACAGCCTTATTATTATTACTTTCATTACCTGTTTTAG
CAGGAGCTATTACTATATTATTAACTGATCGAAATTTAAATACATCATTTTTTGATCCTGCTGGTGGAGGAGATCCAATT
CTTTATCAACATTTATTTTGATTTTTT
>LSTEM285-18|Chilo_orichalcociliellus|ww03994|
AACTTTATATTTTATTTTTGGAATTTGAGCAGGAATAATTGGAACATCACTTAGACTCCTAATTCGAGCTGAATTAGGAA
CCCCTGGATCTTTAATTGGTGATGATCAAATTTATAATACTATTGTTACAGCTCATGCATTTATTATAATTTTTTTTATA
GTTATACCAATTATAATTGGAGGATTTGGAAATTGATTAGTACCTTTAATGTTAGGAGCTCCTGATATAGCCTTCCCACG
AATAAATAATATAAGATTTTGATTACTACCCCCATCACTAACTTTATTAATTTCTAGAAGAATTGTTGAAAATGGAGCTG
GAACTGGGTGAACAGTTTATCCCCCACTTTCATCCAATATTGCCCATGGTGGAAGTTCAGTAGATCTAGCAATTTTTTCT
CTTCATTTAGCTGGAATTTCCTCAATTTTAGGTGCTATTAATTTTATTACAACAATCATTAATATACGAATTAATGGATT
ATCATTTGATCAAATACCTTTATTTGTTTGATCTGTTGGTATTACAGCCTTATTATTATTACTTTCATTACCTGTTTTAG
CAGGAGCTATTACTATATTATTAACTGATCGAAATTTAAATACATCATTTTTTGATCCTGCTGGTGGAGGAGATCCAATT
CTTTATCAACATTTATTTTGATTTTTT
>LSTEM286-18|Chilo_orichalcociliellus|ww03995|
AACTTTATATTTTATTTTTGGAATTTGAGCAGGAATAATTGGAACATCACTTAGACTCCTAATTCGAGCTGAATTAGGAA
CCCCTGGATCTTTAATTGGTGATGATCAAATTTATAATACTATTGTTACAGCTCATGCATTTATTATAATTTTTTTTATA
GTTATACCAATTATAATTGGAGGATTTGGAAATTGATTAGTACCTTTAATGTTAGGAGCTCCTGATATAGCCTTCCCACG
AATAAATAATATAAGATTTTGATTACTACCCCCATCACTAACTTTATTAATTTCTAGAAGAATTGTTGAAAATGGAGCTG
GAACTGGGTGAACAGTTTATCCCCCACTTTCATCCAATATTGCCCATGGTGGGAGTTCAGTAGATCTAGCAATTTTTTCT
CTTCATTTAGCTGGAATTTCCTCAATTTTAGGTGCTATTAATTTTATTACAACAATCATTAATATACGAATTAATGGATT
ATCATTTGATCAAATACCTTTATTTGTTTGATCTGTTGGTATTACAGCCTTATTATTATTACTTTCATTACCTGTTTTAG
CAGGAGCTATTACTATATTATTAACTGATCGAAATTTAAATACATCATTTTTTGATCCTGCTGGTGGAGGAGATCCAATT
CTTTATCAACATTTATTTTGATTTTTT

>LSTEM287-18|Chilo_orichalcociliellus|ww03996|
-----------TTATTTTTGGAATTTGAGCAGGAATAATTGGAACATCACTTAGACTCCTAATTCGAGCTGAATTAGGAA
CCCCTGGATCTTTAATTGGTGATGATCAAATTTATAATACTATTGTTACAGCTCATGCATTTATTATAATTTTTTTTATA
GTTATACCAATTATAATTGGAGGATTTGGAAATTGATTAGTACCTTTAATGTTAGGAGCTCCTGATATAGCCTTCCCACG
AATAAATAATATAAGATTTTGATTACTACCCCCATCACTAACTTTATTAATTTCTAGAAGAATTGTTGAAAATGGAGCTG
GAACTGGGTGAACAGTTTATCCCCCACTTTCATCCAATATTGCCCATGGTGGGAGTTCAGTAGATCTAGCAATTTTTTCT
CTTCATTTAGCTGGAATTTCCTCAATTTTAGGTGCTATTAATTTTATTACAACAATCATTAATATACGAATTAATGGATT
ATCATTTGATCAAATACCTTTATTTGTTTGATCTGTTGGTATTACAGCCTTATTATTATTACTTTCATTACCTGTTTTAG
CAGGAGCTATTACTATATTATTAACTGATCGAAATTTAAATACATCATTTTTTGATCCTGCTGGTGGAGGAGATCCAATT
CTTTATCAACATTTATTTTGATTTTTT
>LSTEM288-18|Chilo_orichalcociliellus|ww03998|
AACTTTATATTTTATTTTTGGAATTTGAGCAGGAATAATTGGAACATCACTTAGACTCCTAATTCGAGCTGAATTAGGAA
CCCCTGGATCTTTAATTGGTGATGATCAAATTTATAATACTATTGTTACAGCTCATGCATTTATTATAATTTTTTTTATA
GTTATACCAATTATAATTGGAGGATTTGGAAATTGATTAGTACCTTTAATGTTAGGAGCTCCTGATATAGCCTTCCCACG
AATAAATAATATAAGATTTTGATTACTACCCCCATCACTAACTTTATTAATTTCTAGAAGAATTGTTGAAAATGGAGCTG
GAACTGGGTGAACAGTTTATCCCCCACTTTCATCCAATATTGCCCATGGTGGGAGTTCAGTAGATCTAGCAATTTTTTCT
CTTCATTTAGCTGGAATTTCCTCAATTTTAGGTGCTATTAATTTTATTACAACAATCATTAATATACGAATTAATGGATT
ATCATTTGATCAAATACCTTTATTTGTTTGATCTGTTGGTATTACAGCCTTATTATTATTACTTTCATTACCTGTTTTAG
CAGGAGCTATTACTATATTATTAACTGATCGAAATTTAAATACATCATTTTTTGATCCTGCTGGTGGAGGAGATCCAATT
CTTTATCAACATTTATTTTGATTTTTT
>LSTEM289-18|Chilo_orichalcociliellus|ww04000|
AACTTTATATTTTATTTTTGGAATTTGAGCAGGAATAATTGGAACATCACTTAGACTCCTAATTCGAGCTGAATTAGGAA
CCCCTGGATCTTTAATTGGTGATGATCAAATTTATAATACTATTGTTACAGCTCATGCATTTATTATAATTTTTTTTATA
GTTATACCAATTATAATTGGAGGATTTGGAAATTGATTAGTACCTTTAATGTTAGGAGCTCCTGATATAGCCTTCCCACG
AATAAATAATATAAGATTTTGATTACTACCCCCATCACTAACTTTATTAATTTCTAGAAGAATTGTTGAAAATGGAGCTG
GAACTGGGTGAACAGTTTATCCCCCACTTTCATCCAATATTGCCCATGGTGGGAGTTCAGTAGATCTAGCAATTTTTTCT
CTTCATTTAGCTGGAATTTCCTCAATTTTAGGTGCTATTAATTTTATTACAACAATCATTAATATACGAATTAATGGATT
ATCATTTGATCAAATACCTTTATTTGTTTGATCTGTTGGTATTACAGCCTTATTATTATTACTTTCATTACCTGTTTTAG
CAGGAGCTATTACTATATTATTAACTGATCGAAATTTAAATACATCATTTTTTGATCCTGCTGGTGGAGGAGATCCAATT
CTTTATCAACATTTATTTTGATTTTTT
>LSTEM290-18|Chilo_orichalcociliellus|ww04001|
AACTTTATATTTTATTTTTGGAATTTGAGCAGGAATAATTGGAACATCACTTAGACTCCTAATTCGAGCTGAATTAGGAA
CCCCTGGATCTTTAATTGGTGATGATCAAATTTATAATACTATTGTTACAGCTCATGCATTTATTATAATTTTTTTTATA
GTTATACCAATTATAATTGGAGGATTTGGAAATTGATTAGTACCTTTAATGTTAGGAGCTCCTGATATAGCCTTCCCACG
AATAAATAATATAAGATTTTGATTACTACCCCCATCACTAACTTTATTAATTTCTAGAAGAATTGTTGAAAATGGAGCTG
GAACTGGGTGAACAGTTTATCCCCCACTTTCATCCAATATTGCCCATGGTGGGAGTTCAGTAGATCTAGCAATTTTTTCT
CTTCATTTAGCTGGAATTTCCTCAATTTTAGGTGCTATTAATTTTATTACAACAATCATTAATATACGAATTAATGGATT
ATCATTTGATCAAATACCTTTATTTGTTTGATCTGTTGGTATTACAGCCTTATTATTATTACTTTCATTACCTGTTTTAG
CAGGAGCTATTACTATATTATTAACTGATCGAAATTTAAATACATCATTTTTTGATCCTGCTGGTGGAGGAGATCCAATT
CTTTATCAACATTTATTTTGATTTTTT
>LSTEM291-18|Chilo_orichalcociliellus|ww04002|
AACTTTATATTTTATTTTTGGAATTTGAGCAGGAATAATTGGAACATCACTTAGACTCCTAATTCGAGCTGAATTAGGAA
CCCCTGGATCTTTAATTGGTGATGATCAAATTTATAATACTATTGTTACAGCTCATGCATTTATTATAATTTTTTTTATA
GTTATACCAATTATAATTGGAGGATTTGGAAATTGATTAGTACCTTTAATGTTAGGAGCTCCTGATATAGCCTTCCCACG
AATAAATAATATAAGATTTTGATTACTACCCCCATCACTAACTTTATTAATTTCTAGAAGAATTGTTGAAAATGGAGCTG
GAACTGGGTGAACAGTTTATCCCCCACTTTCATCCAATATTGCCCATGGTGGGAGTTCAGTAGATCTAGCAATTTTTTCT
CTTCATTTAGCTGGAATTTCCTCAATTTTAGGTGCTATTAATTTTATTACAACAATCATTAATATACGAATTAATGGATT
ATCATTTGATCAAATACCTTTATTTGTTTGATCTGTTGGTATTACAGCCTTATTATTATTACTTTCATTACCTGTTTTAG
CAGGAGCTATTACTATATTATTAACTGATCGAAATTTAAATACATCATTTTTTGATCCTGCTGGTGGAGGAGATCCAATT
CTTTATCAACATTTATTTTGATTTTTT
>LSTEM292-18|Chilo_orichalcociliellus|ww04003|
AACTTTATATTTTATTTTTGGAATTTGAGCAGGAATAATTGGAACATCACTTAGACTCCTAATTCGAGCTGAATTAGGAA
CCCCTGGATCTTTAATTGGTGATGATCAAATTTATAATACTATTGTTACAGCTCATGCATTTATTATAATTTTTTTTATA
GTTATACCAATTATAATTGGAGGATTTGGAAATTGATTAGTACCTTTAATGTTAGGGGCTCCTGATATAGCCTTCCCACG
AATAAATAATATAAGATTTTGATTACTACCCCCATCACTAACTTTATTAATTTCTAGAAGAATTGTTGAAAATGGAGCTG
GAACTGGGTGAACAGTTTATCCCCCACTTTCATCCAATATTGCCCATGGTGGGAGTTCAGTAGATCTAGCAATTTTTTCT
CTTCATTTAGCTGGAATTTCCTCAATTTTAGGTGCTATTAATTTTATTACAACAATCATTAATATACGAATTAATGGATT

ATCATTTGATCAAATACCTTTATTTGTTTGATCTGTTGGTATTACAGCCTTATTATTATTACTTTCATTACCTGTTTTAG
CAGGAGCTATTACTATATTATTAACTGATCGAAATTTAAATACATCATTTTTTGATCCTGCTGGTGGAGGAGATCCAATT
CTTTATCAACATTTATTTTGATTTTTT
>LSTEM293-18|Chilo_orichalcociliellus|ww04004|
AACTTTATATTTTATTTTTGGAATTTGAGCAGGAATAATTGGAACATCACTTAGACTCCTAATTCGAGCTGAATTAGGAA
CCCCTGGATCTTTAATTGGTGATGATCAAATTTATAATACTATTGTTACAGCTCATGCATTTATTATAATTTTTTTTATA
GTTATACCAATTATAATTGGAGGATTTGGAAATTGATTAGTACCTTTAATGTTAGGAGCTCCTGATATAGCCTTCCCACG
AATAAATAATATAAGATTTTGATTACTACCCCCATCACTAACTTTATTAATTTCTAGAAGAATTGTTGAAAATGGAGCTG
GAACTGGGTGAACAGTTTATCCCCCACTTTCATCCAATATTGCCCATGGTGGGAGTTCAGTAGATCTAGCAATTTTTTCT
CTTCATTTAGCTGGAATTTCCTCAATTTTAGGTGCTATTAATTTTATTACAACAATCATTAATATACGAATTAATGGATT
ATCATTTGATCAAATACCTTTATTTGTTTGATCTGTTGGTATTACAGCCTTATTATTATTACTTTCATTACCTGTTTTAG
CAGGAGCTATTACTATATTATTAACTGATCGAAATTTAAATACATCATTTTTTGATCCTGCTGGTGGAGGAGATCCAATT
CTTTATCAACATTTATTTTGATTTTTT
>LSTEM294-18|Chilo_orichalcociliellus|ww04005|
AACTTTATATTTTATTTTTGGAATTTGAGCAGGAATAATTGGAACATCACTTAGACTCCTAATTCGAGCTGAATTAGGAA
CCCCTGGATCTTTAATTGGTGATGATCAAATTTATAATACTATTGTTACAGCTCATGCATTTATTATAATTTTTTTTATA
GTTATACCAATTATAATTGGAGGATTTGGAAATTGATTAGTACCTTTAATGTTAGGAGCTCCTGATATAGCCTTCCCACG
AATAAATAATATAAGATTTTGATTACTACCCCCATCACTAACTTTATTAATTTCTAGAAGAATTGTTGAAAATGGAGCTG
GAACTGGGTGAACAGTTTATCCCCCACTTTCATCCAATATTGCCCATGGTGGGAGTTCAGTAGATCTAGCAATTTTTTCT
CTTCATTTAGCTGGAATTTCCTCAATTTTAGGTGCTATTAATTTTATTACAACAATCATTAATATACGAATTAATGGATT
ATCATTTGATCAAATACCTTTATTTGTTTGATCTGTTGGTATTACAGCCTTATTATTATTACTTTCATTACCTGTTTTAG
CAGGAGCTATTACTATATTATTAACTGATCGAAATTTAAATACATCATTTTTTGATCCTGCTGGTGGAGGAGATCCAATT
CTTTATCAACATTWATTTTGATTTTTT
>LSTEM295-18|Chilo_orichalcociliellus|ww04006|
--CTAATTCGAGCTGAATTAGGAA
CCCCTGGATCTTTAATTGGTGATGATCAAATTTATAATACTATTGTTACAGCTCATGCATTTATTATAATTTTTTTTATA
GTTATACCAATTATAATTGGAGGATTTGGAAATTGATTAGTACCTTTAATGTTAGGAGCTCCTGATATAGCCTTCCCACG
AATAAATAATATAAGATTTTGATTACTACCCCCATCACTAACTTTATTAATTTCTAGAAGAATTGTTGAAAATGGAGCTG
GAACTGGGTGAACAGTTTATCCCCCACTTTCATCCAATATTGCCCATGGTGGGAGTTCAGTAGATCTAGCAATTTTTTCT
CTTCATTTAGCTGGAATTTCCTCAATTTTAGGTGCTATTAATTTTATTACAACAATCATTAATATACGAATTAATGGATT
ATCATTTGATCAAATACCTTTATTTGTTTGATCTGTTGGTATTACAGCCTTATTATTATTACTTTCATTACCTGTTTTAG
CAGGAGCTATTACTATATTATTAACTGATCGAAATTTAAATACATCATTTTTTGATCCTGCTGGTGGAGGAGATCCAATT
CTTTATCAACATTTATTTTGATTTTTT
>LSTEM296-18|Chilo_orichalcociliellus|ww04007|
AACTTTATATTTTATTTTTGGAATTTGAGCAGGAATAATTGGAACATCACTTAGACTCCTAATTCGAGCTGAATTAGGAA
CCCCTGGATCTTTAATTGGTGATGATCAAATTTATAATACTATTGTTACAGCTCATGCATTTATTATAATTTTTTTTATA
GTTATACCAATTATAATTGGAGGATTTGGAAATTGATTAGTACCTTTAATGTTAGGAGCTCCTGATATAGCCTTCCCACG
AATAAATAATATAAGATTTTGATTACTACCCCCATCACTAACTTTATTAATTTCTAGAAGAATTGTTGAAAATGGAGCTG
GAACTGGGTGAACAGTTTATCCCCCACTTTCATCCAATATTGCCCATGGTGGGAGTTCAGTAGATCTAGCAATTTTTTCT
CTTCATTTAGCTGGAATTTCCTCAATTTTAGGTGCTATTAATTTTATTACAACAATCATTAATATACGAATTAATGGATT
ATCATTTGATCAAATACCTTTATTTGTTTGATCTGTTGGTATTACAGCCTTATTATTATTACTTTCATTACCTGTTTTAG
CAGGAGCTATTACTATATTATTAACTGATCGAAATTTAAATACATCATTTTTTGATCCTGCTGGTGGAGGAGATCCAATT
CTTTATCAACATTTATTTTGATTTTTT
>LSTEM297-18|Chilo_orichalcociliellus|ww04008|
----TTATATTTTATTTTTGGRATTTGAGCAGGAATAATTGGAACATCACTTAGACTCCTAATTCGAGCTGAATTAGGAA
CCCCTGGATCTTTAATTGGTGATGATCAAATTTATAATACTATTGTTACAGCTCATGCATTTATTATAATTTTTTTTATA
GTTATACCAATTATAATTGGAGGATTTGGAAATTGATTAGTACCTTTAATGTTAGGAGCTCCTGATATAGCCTTCCCACG
AATAAATAATATAAGATTTTGATTACTACCCCCATCACTAACTTTATTAATTTCTAGAAGAATTGTTGAAAATGGAGCTG
GAACTGGGTGAACAGTTTATCCCCCACTTTCATCCAATATTGCCCATGGTGGGAGTTCAGTAGATCTAGCAATTTTTTCT
CTTCATTTAGCTGGAATTTCCTCAATTTTAGGTGCTATTAATTTTATTACAACAATCATTAATATACGAATTAATGGATT
ATCATTTGATCAAATACCTTTATTTGTTTGATCTGTTGGTATTACAGCCTTATTATTATTACTTTCATTACCTGTTTTAG
CAGGAGCTATTACTATATTATTAACTGATCGAAATTTAAATACATCATTTTTTGATCCTGCTGGTGGAGGAGATCCAATT
CTTTATCAACATTTATTTTGATTTTTT
>LSTEM298-18|Chilo_orichalcociliellus|ww04009|
AACTTTATATTTTATTTTTGGNATTTGAGCAGGAATAATTGGAACATCACTTAGACTCCTAATTCGAGCTGAATTAGGAA
CCCCTGGATCTTTAATTGGTGATGATCAAATTTATAATACTATTGTTACAGCTCATGCATTTATTATAATTTTTTTTATA
GTTATACCAATTATAATTGGAGGATTTGGAAATTGATTAGTACCTTTAATGTTAGGAGCTCCTGATATAGCCTTCCCACG

AATAAATAATATAAGATTTTGATTACTACCCCCATCACTAACTTTATTAATTTCTAGAAGAATTGTTGAAAATGGAGCTG
GAACTGGGTGAACAGTTTATCCCCCACTTTCATCCAATATTGCCCATGGTGGGAGTTCAGTAGATCTAGCAATTTTTTCT
CTTCATTTAGCTGGAATTTCCTCAATTTTAGGTGCTATTAATTTTATTACAACAATCATTAATATACGAATTAATGGATT
ATCATTTGATCAAATACCTTTATTTGTTTGATCTGTTGGTATTACAGCCTTATTATTATTACTTTCATTACCTGTTTTAG
CAGGAGCTATTACTATATTATTAACTGATCGAAATTTAAATACATCATTTTTTGATCCTGCTGGTGGAGGAGATCCAATT
CTTTATCAACATTTATTTTGATTTTTT
>LSTEM299-18|Chilo_orichalcociliellus|ww04010|
AACTTTATATTTTATTTTTGGAATTTGAGCAGGAATAATTGGAACATCACTTAGACTCCTAATTCGAGCTGAATTAGGAA
CCCCTGGATCTTTAATTGGTGATGATCAAATTTATAATACTATTGTTACAGCTCATGCATTTATTATAATTTTTTTTATA
GTTATACCAATTATAATTGGAGGATTTGGAAATTGATTAGTACCTTTAATGTTAGGAGCTCCTGATATAGCCTTCCCACG
AATAAATAATATAAGATTTTGATTACTACCCCCATCACTAACTTTATTAATTTCTAGAAGAATTGTTGAAAATGGAGCTG
GAACTGGGTGAACAGTTTATCCCCCACTTTCATCCAATATTGCCCATGGTGGAAGTTCAGTAGATCTAGCAATTTTTTCT
CTTCATTTAGCTGGAATTTCCTCAATTTTAGGTGCTATTAATTTTATTACAACAATCATTAATATACGAATTAATGGATT
ATCATTTGATCAAATACCTTTATTTGTTTGATCTGTTGGTATTACAGCCTTATTATTATTACTTTCATTACCTGTTTTAG
CAGGAGCTATTACTATATTATTAACTGATCGAAATTTAAATACATCATTTTTTGATCCTGCTGGTGGAGGAGATCCAATT
CTTTATCAACATTWATTTTGATTTTTT
>LSTEM300-18|Chilo_orichalcociliellus|ww04011|
AACTTTATATTTTATTTTTGGAATTTGAGCAGGAATAATTGGAACATCACTTAGACTCCTAATTCGAGCTGAATTAGGAA
CCCCTGGATCTTTAATTGGTGATGATCAAATTTATAATACTATTGTTACAGGTCATGCATGTATTATAATTTTTTTTATA
GTTATACCAATTATAATTGGAGGATTTGGAAATTGATTAGTACCTTTAATGTTAGGAGCTCCTGATATAGCCTTCCCACG
AATAAATAATATAAGATTTTGATTACTACCCCCATCACTAACTTTATTAATTTCTAGAAGAATTGTTGAAAATGGAGCTG
GAACTGGGTGAACAGTTTATCCCCCACTTTCATCCAATATTGCCCATGGTGGGAGTTCAGTAGATCTAGCAATTTTTTCT
CTTCATTTAGCTGGAATTTCCTCAATTTTAGGTGCTATTAATTTTATTACAACAATCATTAATATACGAATTAATGGATT
ATCATTTGATCAAATACCTTTATTTGTTTGATCTGTTGGTATTACAGCCTTATTATTATTACTTTCATTACCTGTTTTAG
CAGGAGCTATTACTATATTATTAACTGATCGAAATTTAAATACATCA---------------------------------
----
>LSTEM301-18|Chilo_orichalcociliellus|ww04012|
--------------TTTTTGGAATTTGAGCAGGAATAATTGGAACATCACTTAGACTCCTAATTCGAGCTGAATTAGGAA
CCCCTGGATCTTTAATTGGTGATGATCAAATTTATAATACTATTGTTACAGCTCATGCATTTATTATAATTTTTTTTATA
GTTATACCAATTATAATTGGAGGATTTGGAAATTGATTAGTACCTTTAATGTTAGGAGCTCCTGATATAGCCTTCCCACG
AATAAATAATATAAGATTTTGATTACTACCCCCATCACTAACTTTATTAATTTCTAGAAGAATTGTTGAAAATGGAGCTG
GAACTGGGTGAACAGTTTATCCCCCACTTTCATCCAATATTGCCCATGGTGGGAGTTCAGTAGATCTAGCAATTTTTTCT
CTTCATTTAGCTGGAATTTCCTCAATTTTAGGTGCTATTAATTTTATTACAACAATCATTAATATACGAATTAATGGATT
ATCATTTGATCAAATACCTTTATTTGTTTGATCTGTTGGTATTACAGCCTTATTATTATTACTTTCATTACCTGTTTTAG
CAGGAGCTATTACTATATTATTAACTGATCGAAATTTAAATACATCATTTTTTGATCCTGCTGGTGGAGGAGATCCAATT
CTTTATCAACATTTATTTTGATTTTTT
>LSTEM302-18|Chilo_orichalcociliellus|ww04013|
----TTATATTTTATTTTTGGAATTTGAGCAGGAATAATTGGAACATCACTTAGACTCCTAATTCGAGCTGAATTAGGAA
CCCCTGGATCTTTAATTGGTGATGATCAAATTTATAATACTATTGTTACAGCTCATGCATTTATTATAATTTTTTTTATA
GTTATACCAATTATAATTGGAGGATTTGGAAATTGATTAGTACCTTTAATGTTAGGAGCTCCTGATATAGCCTTCCCACG
AATAAATAATATAAGATTTTGATTACTACCCCCATCACTAACTTTATTAATTTCTAGAAGAATTGTTGAAAATGGAGCTG
GAACTGGGTGAACAGTTTATCCCCCACTTTCATCCAATATTGCCCATGGTGGGAGTTCAGTAGATCTAGCAATTTTTTCT
CTTCATTTAGCTGGAATTTCCTCAATTTTAGGTGCTATTAATTTTATTACAACAATCATTAATATACGAATTAATGGGTT
ATCATTTGATCAAATACCTTTATTTGTTTGATCTGTTGGTATTACAGCCTTATTATTATTACTTTCATTACCTGTTTTAG
CAGGAGCTATTACTATATTATTAACTGATCGAAATTTAAATACATCATTTTTTGATCCTGCTGGTGGAGGAGATCCAATT
CTTTATCAACATTTATTTTGATTTTTT
>LSTEM303-18|Chilo_orichalcociliellus|ww04014|
AACTTTATATTTTATTTTTGGAATTTGAGCAGGAATAATTGGAACATCACTTAGACTCCTAATTCGAGCTGAATTAGGAA
CCCCTGGATCTTTAATTGGTGATGATCAAATTTATAATACTATTGTTACAGCTCATGCATTTATTATAATTTTTTTTATA
GTTATACCAATTATAATTGGAGGATTTGGAAATTGATTAGTACCTTTAATGTTAGGAGCTCCTGATATAGCCTTCCCACG
AATAAATAATATAAGATTTTGATTACTACCCCCATCACTAACTTTATTAATTTCTAGAAGAATTGTTGAAAATGGAGCTG
GAACTGGGTGAACAGTTTATCCCCCACTTTCATCCAATATTGCCCATGGTGGGAGTTCAGTAGATCTAGCAATTTTTTCT
CTTCATTTAGCTGGAATTTCCTCAATTTTAGGTGCTATTAATTTTATTACAACAATCATTAATATACGAATTAATGGATT
ATCATTTGATCAAATACCTTTATTTGTTTGATCTGTTGGTATTACAGCCTTATTATTATTACTTTCATTACCTGTTTTAG
CAGGAGCTATTACTATATTATTAACTGATCGAAATTTAAATACATCATTTTTTGATCCTGCTGGTGGAGGAGATCCAATT
CTTTATCAACATTTATTTTGATTTTTT
>LSTEM304-18|Chilo_orichalcociliellus|ww04015|

------ATATTTTATTTTTGGAATTTGAGCAGGAATAATTGGAACATCACTTAGACTCCTAATTCGAGCTGAATTAGGAA
CCCCTGGATCTTTAATTGGTGATGATCAAATTTATAATACTATTGTTACAGCTCATGCATTTATTATAATTTTTTTTATA
GTTATACCAATTATAATTGGAGGATTTGGAAATTGATTAGTACCTTTAATGTTAGGAGCTCCTGATATAGCCTTCCCACG
AATAAATAATATAAGATTTTGATTACTACCCCCATCACTAACTTTATTAATTTCTAGAAGAATTGTTGAAAATGGAGCTG
GAACTGGGTGAACAGTTTATCCCCCACTTTCATCCAATATTGCCCATGGTGGGAGTTCAGTAGATCTAGCAATTTTTTCT
CTTCATTTAGCTGGAATTTCCTCAATTTTAGGTGCTATTAATTTTATTACAACAATCATTAATATACGAATTAATGGATT
ATCATTTGATCAAATACCTTTATTTGTTTGATCTGTTGGTATTACAGCCTTATTATTATTACTTTCATTACCTGTTTTAG
CAGGAGCTATTACTATATTATTAACTGATCGAAATTTAAATACATCATTTTTTGATCCTGCTGGTGGAGGAGATCCAATT
CTTTATCAACATTTATTTTGATTTTTT
>LSTEM305-18|Chilo_orichalcociliellus|ww04017|
AACTTTATATTTTATTTTTGGAATTTGAGCAGGAATAATTGGAACATCACTTAGACTCCTAATTCGAGCTGAATTAGGAA
CCCCTGGATCTTTAATTGGTGATGATCAAATTTATAATACTATTGTTACAGCTCATGCATTTATTATAATTTTTTTTATA
GTTATACCAATTATAATTGGAGGATTTGGAAATTGATTAGTACCTTTAATGTTAGGAGCTCCTGATATAGCCTTCCCACG
AATAAATAATATAAGATTTTGATTACTACCCCCATCACTAACTTTATTAATTTCTAGAAGAATTGTTGAAAATGGAGCTG
GAACTGGGTGAACAGTTTATCCCCCACTTTCATCCAATATTGCCCATGGTGGGAGTTCAGTAGATCTAGCAATTTTTTCT
CTTCATTTAGCTGGAATTTCCTCAATTTTAGGTGCTATTAATTTTATTACAACAATCATTAATATACGAATTAATGGATT
ATCATTTGATCAAATACCTTTATTTGTTTGATCTGTTGGTATTACAGCCTTATTATTATTACTTTCATTACCTGTTTTAG
CAGGAGCTATTACTATATTATTAACTGATCGAAATTTAAATACATCATTTTTTGATCCTGCTGGTGGAGGAGATCCAATT
CTTTATCAACATTTATTTTGATTTTTT
>LSTEM306-18|Chilo_orichalcociliellus|ww04018|
AACTTTATATTTTATTTTTGGAATTTGAGCAGGAATAATTGGAACATCACTTAGACTCCTAATTCGAGCTGAATTAGGAA
CCCCTGGATCTTTAATTGGTGATGATCAAATTTATAATACTATTGTTACAGCTCATGCATTTATTATAATTTTTTTTATA
GTTATACCAATTATAATTGGAGGATTTGGAAATTGATTAGTACCTTTAATGTTAGGAGCTCCTGATATAGCCTTCCCACG
AATAAATAATATAAGATTTTGATTACTACCCCCATCACTAACTTTATTAATTTCTAGAAGAATTGTTGAAAATGGAGCTG
GAACTGGGTGAACAGTTTATCCCCCACTTTCATCCAATATTGCCCATGGTGGGAGTTCAGTAGATCTAGCAATTTTTTCT
CTTCATTTAGCTGGAATTTCCTCAATTTTAGGTGCTATTAATTTTATTACAACAATCATTAATATACGAATTAATGGATT
ATCATTTGATCAAATACCTTTATTTGTTTGATCTGTTGGTATTACAGCCTTATTATTATTACTTTCATTACCTGTTTTAG
CAGGAGCTATTACTATATTATTAACTGATCGAAATTTAAATACATCATTTTTTGATCCTGCTGGTGGAGGAGATCCAATT
CTTTATCAACATTTATTTTGATTTTTT
>LSTEM307-18|Chilo_orichalcociliellus|ww04019|
AACTTTATATTTTATTTTTGGNATTTGAGCAGGAATAATTGGAACATCACTTAGACTCCTAATTCGAGCTGAATTAGGAA
CCCCTGGATCTTTAATTGGTGATGATCAAATTTATAATACTATTGTTACAGCTCATGCATTTATTATAATTTTTTTTATA
GTTATACCAATTATAATTGGAGGATTTGGAAATTGATTAGTACCTTTAATGTTAGGAGCTCCTGATATAGCCTTCCCACG
AATAAATAATATAAGATTTTGATTACTACCCCCATCACTAACTTTATTAATTTCTAGAAGAATTGTTGAAAATGGAGCTG
GAACTGGGTGAACAGTTTATCCCCCACTTTCATCCAATATTGCCCATGGTGGGAGTTCAGTAGATCTAGCAATTTTTTCT
CTTCATTTAGCTGGAATTTCCTCAATTTTAGGTGCTATTAATTTTATTACAACAATCATTAATATACGAATTAATGGATT
ATCATTTGATCAAATACCTTTATTTGTTTGATCTGTTGGTATTACAGCCTTATTATTATTACTTTCATTACCTGTTTTAG
CAGGAGCTATTACTATATTATTAACTGATCGAAATTTAAATACATCATTTTTTGATCCTGCTGGTGGAGGAGATCCAATT
CTTTATCAACATTTATTTTGATTTTTT
>LSTEM308-18|Chilo_orichalcociliellus|ww04020|
AACTTTATATTTTATTTTTGGAATTTGAGCAGGAATAATTGGAACATCACTTAGACTCCTAATTCGAGCTGAATTAGGAA
CCCCTGGATCTTTAATTGGTGATGATCAAATTTATAATACTATTGTTACAGCTCATGCATTTATTATAATTTTTTTTATA
GTTATACCAATTATAATTGGAGGATTTGGAAATTGATTAGTACCTTTAATGTTAGGAGCTCCTGATATAGCCTTCCCACG
AATAAATAATATAAGATTTTGATTACTACCCCCATCACTAACTTTATTAATTTCTAGAAGAATTGTTGAAAATGGAGCTG
GAACTGGGTGAACAGTTTATCCCCCACTTTCATCCAATATTGCCCATGGTGGGAGTTCAGTAGATCTAGCAATTTTTTCT
CTTCATTTAGCTGGAATTTCCTCAATTTTAGGTGCTATTAATTTTATTACAACAATCATTAATATACGAATTAATGGATT
ATCATTTGATCAAATACCTTTATTTGTTTGATCTGTTGGTATTACAGCCTTATTATTATTACTTTCATTACCTGTTTTAG
CAGGAGCTATTACTATATTATTAACTGATCGAAATTTAAATACATCATTTTTTGATCCTGCTGGTGGAGGAGATCCAATT
CTTTATCAACATTTATTTTGATTTTTT
>LSTEM309-18|Chilo_orichalcociliellus|ww04021|
AACTTTATATTTTATTTTTGGAATTTGAGCAGGAATAATTGGAACATCACTTAGACTCCTAATTCGAGCTGAATTAGGAA
CCCCTGGATCTTTAATTGGTGATGATCAAATTTATAATACTATTGTTACAGCTCATGCATTTATTATAATTTTTTTTATA
GTTATACCAATTATAATTGGAGGATTTGGAAATTGATTAGTACCTTTAATGTTAGGAGCTCCTGATATAGCCTTCCCACG
AATAAATAATATAAGATTTTGATTACTACCCCCATCACTAACTTTATTAATTTCTAGAAGAATTGTTGAAAATGGAGCTG
GAACTGGGTGAACAGTTTATCCCCCACTTTCATCCAATATTGCCCATGGTGGGAGTTCAGTAGATCTAGCAATTTTTTCT
CTTCATTTAGCTGGAATTTCCTCAATTTTAGGTGCTATTAATTTTATTACAACAATCATTAATATACGAATTAATGGATT
ATCATTTGATCAAATACCTTTATTTGTTTGATCTGTTGGTATTACAGCCTTATTATTATTACTTTCATTACCTGTTTTAG

CAGGAGCTATTACTATATTATTAACTGATCGAAATTTAAATACATCATTTTTTGATCCTGCTGGTGGAGGAGATCCAATT
CTTTATCAACATTTATTTTGATTTTTT
>LSTEM310-18|Chilo_orichalcociliellus|ww04022|
AACTTTATATTTTATTTTTGGAATTTGAGCAGGAATAATTGGAACATCACTTAGACTCCTAATTCGAGCTGAATTAGGAA
CCCCTGGATCTTTAATTGGTGATGATCAAATTTATAATACTATTGTTACAGCTCATGCATTTATTATAATTTTTTTTATA
GTTATACCAATTATAATTGGAGGATTTGGAAATTGATTAGTACCTTTAATGTTAGGAGCTCCTGATATAGCCTTCCCACG
AATAAATAATATAAGATTTTGATTACTACCCCCATCACTAACTTTATTAATTTCTAGAAGAATTGTTGAAAATGGAGCTG
GAACTGGGTGAACAGTTTATCCCCCACTTTCATCCAATATTGCCCATGGTGGGAGTTCAGTAGATCTAGCAATTTTTTCT
CTTCATTTAGCTGGAATTTCCTCAATTTTAGGTGCTATTAATTTTATTACAACAATCATTAATATACGAATTAATGGATT
ATCATTTGATCAAATACCTTTATTTGTTTGATCTGTTGGTATTACAGCCTTATTATTATTACTTTCATTACCTGTTTTAG
CAGGAGCTATTACTATATTATTAACTGATCGAAATTTAAATACATCATTTTTTGATCCTGCTGGTGGAGGAGATCCAATT
CTTTATCAACATTWATTTTGATTTTTT
>LSTEM311-18|Chilo_orichalcociliellus|ww04023|
AACTTTATATTTTATTTTTGGAATTTGAGCAGGAATAATTGGAACATCACTTAGACTCCTAATTCGAGCTGAATTAGGAA
CCCCTGGATCTTTAATTGGTGATGATCAAATTTATAATACTATTGTTACAGCTCATGCATTTATTATAATTTTTTTTATA
GTTATACCAATTATAATTGGAGGATTTGGAAATTGATTAGTACCTTTAATGTTAGGAGCTCCTGATATAGCCTTCCCACG
AATAAATAATATAAGATTTTGATTACTACCCCCATCACTAACTTTATTAATTTCTAGAAGAATTGTTGAAAATGGAGCTG
GAACTGGGTGAACAGTTTATCCCCCACTTTCATCCAATATTGCCCATGGTGGGAGTTCAGTAGATCTAGCAATTTTTTCT
CTTCATTTAGCTGGAATTTCCTCAATTTTAGGTGCTATTAATTTTATTACAACAATCATTAATATACGAATTAATGGATT
ATCATTTGATCAAATACCTTTATTTGTTTGATCTGTTGGTATTACAGCCTTATTATTATTACTTTCATTACCTGTTTTAG
CAGGAGCTATTACTATATTATTAACTGATCGAAATTTAAATACATCATTTTTTGATCCTGCTGGTGGAGGAGATCCAATT
CTTTATCAACATTTATTTTGATTTTTT
>LSTEM312-18|Chilo_orichalcociliellus|ww04024|
AACTTTATATTTTATTTTTGGNATTTGAGCAGGAATAATTGGAACATCACTTAGACTCCTAATTCGAGCTGAATTAGGAA
CCCCTGGATCTTTAATTGGTGATGATCAAATTTATAATACTATTGTTACAGCTCATGCATTTATTATAATTTTTTTTATA
GTTATACCAATTATAATTGGAGGATTTGGAAATTGATTAGTACCTTTAATGTTAGGAGCTCCTGATATAGCCTTCCCACG
AATAAATAATATAAGATTTTGATTACTACCCCCATCACTAACTTTATTAATTTCTAGAAGAATTGTTGAAAATGGAGCTG
GAACTGGGTGAACAGTTTATCCCCCACTTTCATCCAATATTGCCCATGGTGGGAGTTCAGTAGATCTAGCAATTTTTTCT
CTTCATTTAGCTGGAATTTCCTCAATTTTAGGTGCTATTAATTTTATTACAACAATCATTAATATACGAATTAATGGATT
ATCATTTGATCAAATACCTTTATTTGTTTGATCTGTTGGTATTACAGCCTTATTATTATTACTTTCATTACCTGTTTTAG
CAGGAGCTATTACTATATTATTAACTGATCGAAATTTAAATACATCATTTTTTGATCCTGCTGGTGGAGGAGATCCAATT
CTTTATCAACATTTATTTTGATTTTTT
>LSTEM313-18|Chilo_orichalcociliellus|ww04025|
AACTTTATATTTTATTTTTGGAATTTGAGCAGGAATAATTGGAACATCACTTAGACTCCTAATTCGAGCTGAATTAGGAA
CCCCTGGATCTTTAATTGGTGATGATCAAATTTATAATACTATTGTTACAGCTCATGCATTTATTATAATTTTTTTTATA
GTTATACCAATTATAATTGGAGGATTTGGAAATTGATTAGTACCTTTAATGTTAGGAGCTCCTGATATAGCCTTCCCACG
AATAAATAATATAAGATTTTGATTACTACCCCCATCACTAACTTTATTAATTTCTAGAAGAATTGTTGAAAATGGAGCTG
GAACTGGGTGAACAGTTTATCCCCCACTTTCATCCAATATTGCCCATGGTGGGAGTTCAGTAGATCTAGCAATTTTTTCT
CTTCATTTAGCTGGAATTTCCTCAATTTTAGGTGCTATTAATTTTATTACAACAATCATTAATATACGAATTAATGGATT
ATCATTTGATCAAATACCTTTATTTGTTTGATCTGTTGGTATTACAGCCTTATTATTATTACTTTCATTACCTGTTTTAG
CAGGAGCTATTACTATATTATTAACTGATCGAAATTTAAATACATCATTTTTTGATCCTGCTGGTGGAGGAGATCCAATT
CTTTATCAACATTTATTTTGATTTTTT
>LSTEM314-18|Chilo_orichalcociliellus|ww04026|
AACTTTATATTTTATTTTTGGAATTTGAGCAGGAATAATTGGAACATCACTTAGACTCCTAATTCGAGCTGAATTAGGAA
CCCCTGGATCTTTAATTGGTGATGATCAAATTTATAATACTATTGTTACAGCTCATGCATTTATTATAATTTTTTTTATA
GTTATACCAATTATAATTGGAGGATTTGGAAATTGATTAGTACCTTTAATGTTAGGAGCTCCTGATATAGCCTTCCCACG
AATAAATAATATAAGATTTTGATTACTACCCCCATCACTAACTTTATTAATTTCTAGAAGAATTGTTGAAAATGGAGCTG
GAACTGGGTGAACAGTTTATCCCCCACTTTCATCCAATATTGCCCATGGTGGGAGTTCAGTAGATCTAGCAATTTTTTCT
CTTCATTTAGCTGGAATTTCCTCAATTTTAGGTGCTATTAATTTTATTACAACAATCATTAATATACGAATTAATGGATT
ATCATTTGATCAAATACCTTTATTTGTTTGATCTGTTGGTATTACAGCCTTATTATTATTACTTTCATTACCTGTTTTAG
CAGGAGCTATTACTATATTATTAACTGATCGAAATTTAAATACATCATTTTTTGATCCTGCTGGTGGAGGAGATCCAATT
CTTTATCAACATTTATTTTGATTTTTT
>LSTEM315-18|Chilo_orichalcociliellus|ww04027|
AACTTTATATTTTATTTTTGGAATTTGAGCAGGAATAATTGGAACATCACTTAGACTCCTAATTCGAGCTGAATTAGGAA
CCCCTGGATCTTTAATTGGTGATGATCAAATTTATAATACTATTGTTACAGCTCATGCATTTATTATAATTTTTTTTATA
GTTATACCAATTATAATTGGAGGATTTGGAAATTGATTAGTACCTTTAATGTTAGGAGCTCCTGATATAGCCTTCCCACG
AATAAATAATATAAGATTTTGATTACTACCCCCATCACTAACTTTATTAATTTCTAGAAGAATTGTTGAAAATGGAGCTG

GAACTGGGTGAACAGTTTATCCCCCACTTTCATCCAATATTGCCCATGGTGGGAGTTCAGTAGATCTAGCAATTTTTTCT
CTTCATTTAGCTGGAATTTCCTCAATTTTAGGTGCTATTAATTTTATTACAACAATCATTAATATACGAATTAATGGATT
ATCATTTGATCAAATACCTTTATTTGTTTGATCTGTTGGTATTACAGCCTTATTATTATTACTTTCATTACCTGTTTTAG
CAGGAGCTATTACTATATTATTAACTGATCGAAATTTAAATACATCATTTTTTGATCCTGCTGGTGGAGGAGATCCAATT
CTTTATCAACATTTATTTTGATTTTTT
>LSTEM316-18|Chilo_orichalcociliellus|ww04028|
AACTTTATATTTTATTTTTGGNATTTGAGCAGGAATAATTGGAACATCACTTAGACTCCTAATTCGAGCTGAATTAGGAA
CCCCTGGATCTTTAATTGGTGATGATCAAATTTATAATACTATTGTTACAGCTCATGCATTTATTATAATTTTTTTTATA
GTTATACCAATTATAATTGGAGGATTTGGAAATTGATTAGTACCTTTAATGTTAGGAGCTCCTGATATAGCCTTCCCACG
AATAAATAATATAAGATTTTGATTACTACCCCCATCACTAACTTTATTAATTTCTAGAAGAATTGTTGAAAATGGAGCTG
GAACTGGGTGAACAGTTTATCCCCCACTTTCATCCAATATTGCCCATGGTGGGAGTTCAGTAGATCTAGCAATTTTTTCT
CTTCATTTAGCTGGAATTTCCTCAATTTTAGGTGCTATTAATTTTATTACAACAATCATTAATATACGAATTAATGGATT
ATCATTTGATCAAATACCTTTATTTGTTTGATCTGTTGGTATTACAGCCTTATTATTATTACTTTCATTACCTGTTTTAG
CAGGAGCTATTACTATATTATTAACTGATCGAAATTTAAATACATCATTTTTTGATCCTGCTGGTGGAGGAGATCCAATT
CTTTATCAACATTTATTTTGATTTTTT
>LSTEM317-18|Chilo_orichalcociliellus|ww04030|
AACTTTATATTTTATTTTTGGAATTTGAGCAGGAATAATTGGAACATCACTTAGACTCCTAATTCGAGCTGAATTAGGAA
CCCCTGGATCTTTAATTGGTGATGATCAAATTTATAATACTATTGTTACAGCTCATGCATTTATTATAATTTTTTTTATA
GTTATACCAATTATAATTGGAGGATTTGGAAATTGATTAGTACCTTTAATGTTAGGAGCTCCTGATATAGCCTTCCCACG
AATAAATAATATAAGATTTTGATTACTACCCCCATCACTAACTTTATTAATTTCTAGAAGAATTGTTGAAAATGGAGCTG
GAACTGGGTGAACAGTTTATCCCCCACTTTCATCCAATATTGCCCATGGTGGGAGTTCAGTAGATCTAGCAATTTTTTCT
CTTCATTTAGCTGGAATTTCCTCAATTTTAGGTGCTATTAATTTTATTACAACAATCATTAATATACGAATTAATGGATT
ATCATTTGATCAAATACCTTTATTTGTTTGATCTGTTGGTATTACAGCCTTATTATTATTACTTTCATTACCTGTTTTAG
CAGGAGCTATTACTATATTATTAACTGATCGAAATTTAAATACATCATTTTTTGATCCTGCTGGTGGAGGAGATCCAATT
CTTTATCAACATTTATTTTGATTTTTT
>LSTEM318-18|Chilo_orichalcociliellus|ww04031|
AACTTTATATTTTATTTTTGGAATTTGAGCAGGAATAATTGGAACATCACTTAGACTCCTAATTCGAGCTGAATTAGGAA
CCCCTGGATCTTTAATTGGTGATGATCAAATTTATAATACTATTGTTACAGCTCATGCATTTATTATAATTTTTTTTATA
GTTATACCAATTATAATTGGAGGATTTGGAAATTGATTAGTACCTTTAATGTTAGGAGCTCCTGATATAGCCTTCCCACG
AATAAATAATATAAGATTTTGATTACTACCCCCATCACTAACTTTATTAATTTCTAGAAGAATTGTTGAAAATGGAGCTG
GAACTGGGTGAACAGTTTATCCCCCACTTTCATCCAATATTGCCCATGGTGGGAGTTCAGTAGATCTAGCAATTTTTTCT
CTTCATTTAGCTGGAATTTCCTCAATTTTAGGTGCTATTAATTTTATTACAACAATCATTAATATACGAATTAATGGATT
ATCATTTGATCAAATACCTTTATTTGTTTGATCTGTTGGTATTACAGCCTTATTATTATTACTTTCATTACCTGTTTTAG
CAGGAGCTATTACTATATTATTAACTGATCGAAATTTAAATACATCATTTTTTGATCCTGCTGGTGGAGGAGATCCAATT
CTTTATCAACATTTATTTTGATTTTTT
>LSTEM319-18|Chilo_orichalcociliellus|ww04032|
AACTTTATATTTTATTTTTGGAATTTGAGCAGGAATAATTGGAACATCACTTAGACTCCTAATTCGAGCTGAATTAGGAA
CCCCTGGATCTTTAATTGGTGATGATCAAATTTATAATACTATTGTTACAGCTCATGCATTTATTATAATTTTTTTTATA
GTTATACCAATTATAATTGGAGGATTTGGAAATTGATTAGTACCTTTAATGTTAGGAGCTCCTGATATAGCCTTCCCACG
AATAAATAATATAAGATTTTGATTACTACCCCCATCACTAACTTTATTAATTTCTAGAAGAATTGTTGAAAATGGAGCTG
GAACTGGGTGAACAGTTTATCCCCCACTTTCATCCAATATTGCCCATGGTGGGAGTTCAGTAGATCTAGCAATTTTTTCT
CTTCATTTAGCTGGAATTTCCTCAATTTTAGGTGCTATTAATTTTATTACAACAATCATTAATATACGAATTAATGGATT
ATCATTTGATCAAATACCTTTATTTGTTTGATCTGTTGGTATTACAGCCTTATTATTATTACTTTCATTACCTGTTTTAG
CAGGAGCTATTACTATATTATTAACTGATCGAAATTTAAATACATCATTTTTTGATCCTGCTGGTGGAGGAGATCCAATT
CTTTATCAACATTTATTTTGATTTTTT
>LSTEM320-18|Chilo_orichalcociliellus|ww04033|
AACTTTATATTTTATTTTTGGAATTTGAGCAGGAATAATTGGAACATCACTTAGACTCCTAATTCGAGCTGAATTAGGAA
CCCCTGGATCTTTAATTGGTGATGATCAAATTTATAATACTATTGTTACAGCTCATGCATTTATTATAATTTTTTTTATA
GTTATACCAATTATAATTGGAGGATTTGGAAATTGATTAGTACCTTTAATGTTAGGAGCTCCTGATATAGCCTTCCCACG
AATAAATAATATAAGATTTTGATTACTACCCCCATCACTAACTTTATTAATTTCTAGAAGAATTGTTGAAAATGGAGCTG
GAACTGGGTGAACAGTTTATCCCCCACTTTCATCCAATATTGCCCATGGTGGGAGTTCAGTAGATCTAGCAATTTTTTCT
CTTCATTTAGCTGGAATTTCCTCAATTTTAGGTGCTATTAATTTTATTACAACAATCATTAATATACGAATTAATGGATT
ATCATTTGATCAAATACCTTTATTTGTTTGATCTGTTGGTATTACAGCCTTATTATTATTACTTTCATTACCTGTTTTAG
CAGGAGCTATTACTATATTATTAACTGATCGAAATTTAAATACATCATTTTTTGATCCTGCTGGTGGAGGAGATCCAATT
CTTTATCAACATTTATTTTGATTTTTT
>LSTEM321-18|Chilo_orichalcociliellus|ww04034|
AACTTTATATTTTATTTTTGGAATTTGAGCAGGAATAATTGGAACATCACTTAGACTCCTAATTCGAGCTGAATTAGGAA

CCCCTGGATCTTTAATTGGTGATGATCAAATTTATAATACTATTGTTACAGCTCATGCATTTATTATAATTTTTTTATA
GTTATACCAATTATAATTGGAGGATTTGGAAATTGATTAGTACCTTTAATGTTAGGAGCTCCTGATATAGCCTTCCCACG
AATAAATAATATAAGATTTTGATTACTACCCCCATCACTAACTTTATTAATTTCTAGAAGAATTGTTGAAAATGGAGCTG
GAACTGGGTGAACAGTTTATCCCCCACTTTCATCCAATATTGCCCATGGTGGGAGTTCAGTAGATCTAGCAATTTTTTCT
CTTCATTTAGCTGGAATTTCCTCAATTTTAGGTGCTATTAATTTTATTACAACAATCATTAATATACGAATTAATGGATT
ATCATTTGATCAAATACCTTTATTTGTTTGATCTGTTGGTATTACAGCCTTATTATTATTACTTTCATTACCTGTTTTAG
CAGGAGCTATTACTATATTATTAACTGATCGAAATTTAAATACATCATTTTTTGATCCTGCTGGTGGAGGAGATCCAATT
CTTTATCAACATTTATTTTGATTTTTT
>LSTEM322-18|Chilo_orichalcociliellus|ww04035|
AACTTTATATTTTATTTTTGGAATTTGAGCAGGAATAATTGGAACATCACTTAGACTCCTAATTCGAGCTGAATTAGGAA
CCCCTGGATCTTTAATTGGTGATGATCAAATTTATAATACTATTGTTACAGCTCATGCATTTATTATAATTTTTTTATA
GTTATACCAATTATAATTGGAGGATTTGGAAATTGATTAGTACCTTTAATGTTAGGAGCTCCTGATATAGCCTTCCCACG
AATAAATAATATAAGATTTTGATTACTACCCCCATCACTAACTTTATTAATTTCTAGAAGAATTGTTGAAAATGGAGCTG
GAACTGGGTGAACAGTTTATCCCCCACTTTCATCCAATATTGCCCATGGTGGGAGTTCAGTAGATCTAGCAATTTTTTCT
CTTCATTTAGCTGGAATTTCCTCAATTTTAGGTGCTATTAATTTTATTACAACAATCATTAATATACGAATTAATGGATT
ATCATTTGATCAAATACCTTTATTTGTTTGATCTGTTGGTATTACAGCCTTATTATTATTACTTTCATTACCTGTTTTAG
CAGGAGCTATTACTATATTATTAACTGATCGAAATTTAAATACATCATTTTTTGATCCTGCTGGTGGAGGAGATCCAATT
CTTTATCAACATTTATTTTGATTTTTT
>LSTEM323-18|Chilo_orichalcociliellus|ww04036|
AACTTTATATTTTATTTTTGGAATTTGAGCAGGAATAATTGGAACATCACTTAGACTCCTAATTCGAGCTGAATTAGGAA
CCCCTGGATCTTTAATTGGTGATGATCAAATTTATAATACTATTGTTACAGCTCATGCATTTATTATAATTTTTTTATA
GTTATACCAATTATAATTGGAGGATTTGGAAATTGATTAGTACCTTTAATGTTAGGAGCTCCTGATATAGCCTTCCCACG
AATAAATAATATAAGATTTTGATTACTACCCCCATCACTAACTTTATTAATTTCTAGAAGAATTGTTGAAAATGGAGCTG
GAACTGGGTGAACAGTTTATCCCCCACTTTCATCCAATATTGCCCATGGTGGGAGTTCAGTAGATCTAGCAATTTTTTCT
CTTCATTTAGCTGGAATTTCCTCAATTTTAGGTGCTATTAATTTTATTACAACAATCATTAATATACGAATTAATGGATT
ATCATTTGATCAAATACCTTTATTTGTTTGATCTGTTGGTATTACAGCCTTATTATTATTACTTTCATTACCTGTTTTAG
CAGGAGCTATTACTATATTATTAACTGATCGAAATTTAAATACATCATTTTTTGATCCTGCTGGTGGAGGAGATCCAATT
CTTTATCAACATTTATTTTGATTTTTT
>LSTEM324-18|Chilo_orichalcociliellus|ww04037|
AACTTTATATTTTATTTTTGGAATTTGAGCAGGAATAATTGGAACATCACTTAGACTCCTAATTCGAGCTGAATTAGGAA
CCCCTGGATCTTTAATTGGTGATGATCAAATTTATAATACTATTGTTACAGCTCATGCATTTATTATAATTTTTTTATA
GTTATACCAATTATAATTGGAGGATTTGGAAATTGATTAGTACCTTTAATGTTAGGAGCTCCTGATATAGCCTTCCCACG
AATAAATAATATAAGATTTTGATTACTACCCCCATCACTAACTTTATTAATTTCTAGAAGAATTGTTGAAAATGGAGCTG
GAACTGGGTGAACAGTTTATCCCCCACTTTCATCCAATATTGCCCATGGTGGGAGTTCAGTAGATCTAGCAATTTTTTCT
CTTCATTTAGCTGGAATTTCCTCAATTTTAGGTGCTATTAATTTTATTACAACAATCATTAATATACGAATTAATGGATT
ATCATTTGATCAAATACCTTTATTTGTTTGATCTGTTGGTATTACAGCCTTATTATTATTACTTTCATTACCTGTTTTAG
CAGGAGCTATTACTATATTATTAACTGATCGAAATTTAAATACATCATTTTTTGATCCTGCTGGTGGAGGAGATCCAATT
CTTTATCAACATTTATTTTGATTTTTT
>LSTEM325-18|Chilo_orichalcociliellus|ww04038|
AACTTTATATTTTATTTTTGGAATTTGAGCAGGAATAATTGGAACATCACTTAGACTCCTAATTCGAGCTGAATTAGGAA
CCCCTGGATCTTTAATTGGTGATGATCAAATTTATAATACTATTGTTACAGCTCATGCATTTATTATAATTTTTTTATA
GTTATACCAATTATAATTGGAGGATTTGGAAATTGATTAGTACCTTTAATGTTAGGAGCTCCTGATATAGCCTTCCCACG
AATAAATAATATAAGATTTTGATTACTACCCCCATCACTAACTTTATTAATTTCTAGAAGAATTGTTGAAAATGGAGCTG
GAACTGGGTGAACAGTTTATCCCCCACTTTCATCCAATATTGCCCATGGTGGGAGTTCAGTAGATCTAGCAATTTTTTCT
CTTCATTTAGCTGGAATTTCCTCAATTTTAGGTGCTATTAATTTTATTACAACAATCATTAATATACGAATTAATGGATT
ATCATTTGATCAAATACCTTTATTTGTTTGATCTGTTGGTATTACAGCCTTATTATTATTACTTTCATTACCTGTTTTAG
CAGGAGCTATTACTATATTATTAACTGATCGAAATTTAAATACATCATTTTTTGATCCTGCTGGTGGAGGAGATCCAATT
CTTTATCAACATTTATTTTGATTTTTT
>LSTEM326-18|Chilo_orichalcociliellus|ww04039|
AACTTTATATTTTATTTTTGGAATTTGAGCAGGAATAATTGGAACATCACTTAGACTCCTAATTCGAGCTGAATTAGGAA
CCCCTGGATCTTTAATTGGTGATGATCAAATTTATAATACTATTGTTACAGCTCATGCATTTATTATAATTTTTTTATA
GTTATACCAATTATAATTGGAGGATTTGGAAATTGATTAGTACCTTTAATGTTAGGAGCTCCTGATATAGCCTTCCCACG
AATAAATAATATAAGATTTTGATTACTACCCCCATCACTAACTTTATTAATTTCTAGAAGAATTGTTGAAAATGGAGCTG
GAACTGGGTGAACAGTTTATCCCCCACTTTCATCCAATATTGCCCATGGTGGGAGTTCAGTAGATCTAACAATTTTTTCT
CTTCATTTAGCTGGAATTTCCTCAATTTTAGGTGCTATTAATTTTATTACAACAATCATTAATATACGAATTAATGGATT
ATCATTTGATCAAATACCTTTATTTGTTTGATCTGTTGGTATTACAGCCTTATTATTATTACTTTCATTACCTGTTTTAG
CAGGAGCTATTACTATATTATTAACTGATCGAAATTTAAATACATCATTTTTTGATCCTGCTGGTGGAGGAGATCCAATT

CTTTATCAACATTTATTTTGATTTTTT
>LSTEM327-18|Chilo_orichalcociliellus|ww04040|
AACTTTATATTTTATTTTTGGAATTTGAGCAGGAATAATTGGAACATCACTTAGACTCCTAATTCGAGCTGAATTAGGAA
CCCCTGGATCTTTAATTGGTGATGATCAAATTTATAATACTATTGTTACAGCTCATGCATTTATTATAATTTTTTTTATA
GTTATACCAATTATAATTGGAGGATTTGGAAATTGATTAGTACCTTTAATGTTAGGAGCTCCTGATATAGCCTTCCCACG
AATAAATAATATAAGATTTTGATTACTACCCCCATCACTAACTTTATTAATTTCTAGAAGAATTGTTGAAAATGGAGCTG
GAACTGGGTGAACAGTTTATCCCCCACTTTCATCCAATATTGCCCATGGTGGGAGTTCAGTAGATCTAGCAATTTTTTCT
CTTCATTTAGCTGGAATTTCCTCAATTTTAGGTGCTATTAATTTTATTACAACAATCATTAATATACGAATTAATGGATT
ATCATTTGATCAAATACCTTTATTTGTTTGATCTGTTGGTATTACAGCCTTATTATTATTACTTTCATTACCTGTTTTAG
CAGGAGCTATTACTATATTATTAACTGATCGAAATTTAAATACATCATTTTTTGATCCTGCTGGTGGAGGAGATCCAATT
CTTTATCAACATTTATTTTGATTTTTT
>LSTEM328-18|Chilo_orichalcociliellus|ww04041|
AACTTTATATTTTATTTTTGGAATTTGAGCAGGAATAATTGGAACATCACTTAGACTCCTAATTCGAGCTGAATTAGGAA
CCCCTGGATCTTTAATTGGTGATGATCAAATTTATAATACTATTGTTACAGCTCATGCATTTATTATAATTTTTTTTATA
GTTATACCAATTATAATTGGAGGATTTGGAAATTGATTAGTACCTTTAATGTTAGGAGCTCCTGATATAGCCTTCCCACG
AATAAATAATATAAGATTTTGATTACTACCCCCATCACTAACTTTATTAATTTCTAGAAGAATTGTTGAAAATGGAGCTG
GAACTGGGTGAACAGTTTATCCCCCACTTTCATCCAATATTGCCCATGGTGGGAGTTCAGTAGATCTAGCAATTTTTTCT
CTTCATTTAGCTGGAATTTCCTCAATTTTAGGTGCTATTAATTTTATTACAACAATCATTAATATACGAATTAATGGATT
ATCATTTGATCAAATACCTTTATTTGTTTGATCTGTTGGTATTACAGCCTTATTATTATTACTTTCATTACCTGTTTTAG
CAGGAGCTATTACTATATTATTAACTGATCGAAATTTAAATACATCATTTTTTGATCCTGCTGGTGGAGGAGATCCAATT
CTTTATCAACATTTATTTTGATTTTTT
>LSTEM329-18|Chilo_orichalcociliellus|ww04042|
AACTTTATATTTTATTTTTGGAATTTGAGCAGGAATAATTGGAACATCACTTAGACTCCTAATTCGAGCTGAATTAGGAA
CCCCTGGATCTTTAATTGGTGATGATCAAATTTATAATACTATTGTTACAGCTCATGCATTTATTATAATTTTTTTTATA
GTTATACCAATTATAATTGGAGGATTTGGAAATTGATTAGTACCTTTAATGTTAGGAGCTCCTGATATAGCCTTCCCACG
AATAAATAATATAAGATTTTGATTACTACCCCCATCACTAACTTTATTAATTTCTAGAAGAATTGTTGAAAATGGAGCTG
GAACTGGGTGAACAGTTTATCCCCCACTTTCATCCAATATTGCCCATGGTGGGAGTTCAGTAGATCTAGCAATTTTTTCT
CTTCATTTAGCTGGAATTTCCTCAATTTTAGGTGCTATTAATTTTATTACAACAATCATTAATATACGAATTAATGGATT
ATCATTTGATCAAATACCTTTATTTGTTTGATCTGTTGGTATTACAGCCTTATTATTATTACTTTCATTACCTGTTTTAG
CAGGAGCTATTACTATATTATTAACTGATCGAAATTTAAATACATCATTTTTTGATCCTGCTGGTGGAGGAGATCCAATT
CTTTATCAACATTTATTTTGATTTTTT
>LSTEM330-18|Chilo_orichalcociliellus|ww04043|
AACTTTATATTTTATTTTTGGAATTTGAGCAGGAATAATTGGAACATCACTTAGACTCCTAATTCGAGCTGAATTAGGAA
CCCCTGGATCTTTAATTGGTGATGATCAAATTTATAATACTATTGTTACAGCTCATGCATTTATTATAATTTTTTTTATA
GTTATACCAATTATAATTGGAGGATTTGGAAATTGATTAGTACCTTTAATGTTAGGAGCTCCTGATATAGCCTTCCCACG
AATAAATAATATAAGATTTTGATTACTACCCCCATCACTAACTTTATTAATTTCTAGAAGAATTGTTGAAAATGGAGCTG
GAACTGGGTGAACAGTTTATCCCCCACTTTCATCCAATATTGCCCATGGTGGGAGTTCAGTAGATCTAGCAATTTTTTCT
CTTCATTTAGCTGGAATTTCCTCAATTTTAGGTGCTATTAATTTTATTACAACAATCATTAATATACGAATTAATGGATT
ATCATTTGATCAAATACCTTTATTTGTTTGATCTGTTGGTATTACAGCCTTATTATTATTACTTTCATTACCTGTTTTAG
CAGGAGCTATTACTATATTATTAACTGATCGAAATTTAAATACATCATTTTTTGATCCTGCTGGTGGAGGAGATCCAATT
CTTTATCAACATTTATTTTGATTTTTT
>LSTEM331-18|Chilo_orichalcociliellus|ww04045|
AACTTTATATTTTATTTTTGGAATTTGAGCAGGAATAATTGGAACATCACTTAGACTCCTAATTCGAGCTGAATTAGGAA
CCCCTGGATCTTTAATTGGTGATGATCAAATTTATAATACTATTGTTACAGCTCATGCATTTATTATAATTTTTTTTATA
GTTATACCAATTATAATTGGAGGATTTGGAAATTGATTAGTACCTTTAATGTTAGGAGCTCCTGATATAGCCTTCCCACG
AATAAATAATATAAGATTTTGATTACTACCCCCATCACTAACTTTATTAATTTCTAGAAGAATTGTTGAAAATGGAGCTG
GAACTGGGTGAACAGTTTATCCCCCACTTTCATCCAATATTGCCCATGGTGGGAGTTCAGTAGATCTAGCAATTTTTTCT
CTTCATTTAGCTGGAATTTCCTCAATTTTAGGTGCTATTAATTTTATTACAACAATCATTAATATACGAATTAATGGATT
ATCATTTGATCAAATACCTTTATTTGTTTGATCTGTTGGTATTACAGCCTTATTATTATTACTTTCATTACCTGTTTTAG
CAGGAGCTATTACTATATTATTAACTGATCGAAATTTAAATACATCATTTTTTGATCCTGCTGGTGGAGGAGATCCAATT
CTTTATCAACATTTATTTTGATTTTTT
>LSTEM332-18|Chilo_orichalcociliellus|ww04046|
AACTTTATATTTTATTTTTGGAATTTGAGCAGGAATAATTGGAACATCACTTAGACTCCTAATTCGAGCTGAATTAGGAA
CCCCTGGATCTTTAATTGGTGATGATCAAATTTATAATACTATTGTTACAGCTCATGCATTTATTATAATTTTTTTTATA
GTTATACCAATTATAATTGGAGGATTTGGAAATTGATTAGTACCTTTAATGTTAGGAGCTCCTGATATAGCCTTCCCACG
AATAAATAATATAAGATTTTGATTACTACCCCCATCACTAACTTTATTAATTTCTAGAAGAATTGTTGAAAATGGAGCTG
GAACTGGGTGAACAGTTTATCCCCCACTTTCATCCAATATTGCCCATGGTGGGAGTTCAGTAGATCTAGCAATTTTTTCT

CTTCATTTAGCTGGAATTTCCTCAATTTTAGGTGCTATTAATTTTATTACAACAATCATTAATATACGAATTAATGGATT
ATCATTTGATCAAATACCTTTATTTGTTTGATCTGTTGGTATTACAGCCTTATTATTATTACTTTCATTACCTGTTTTAG
CAGGAGCTATTACTATATTATTAACTGATCGAAATTTAAATACATCATTTTTTGATCCTGCTGGTGGAGGAGATCCAATT
CTTTATCAACATTTATTTTGATTTTTT
>LSTEM333-18|Chilo_orichalcociliellus|ww04047|
AACTTTATATTTTATTTTTGGAATTTGAGCAGGAATAATTGGAACATCACTTAGACTCCTAATTCGAGCTGAATTAGGAA
CCCCTGGATCTTTAATTGGTGATGATCAAATTTATAATACTATTGTTACAGCTCATGCATTTATTATAATTTTTTTTATA
GTTATACCAATTATAATTGGAGGATTTGGAAATTGATTAGTACCTTTAATGTTAGGAGCTCCTGATATAGCCTTCCCACG
AATAAATAATATAAGATTTTGATTACTACCCCCATCACTAACTTTATTAATTTCTAGAAGAATTGTTGAAAATGGAGCTG
GAACTGGGTGAACAGTTTATCCCCCACTTTCATCCAATATTGCCCATGGTGGGAGTTCAGTAGATCTAGCAATTTTTTCT
CTTCATTTAGCTGGAATTTCCTCAATTTTAGGTGCTATTAATTTTATTACAACAATCATTAATATACGAATTAATGGATT
ATCATTTGATCAAATACCTTTATTTGTTTGATCTGTTGGTATTACAGCCTTATTATTATTACTTTCATTACCTGTTTTAG
CAGGAGCTATTACTATATTATTAACTGATCGAAATTTAAATACATCATTTTTTGATCCTGCTGGTGGAGGAGATCCAATT
CTTTATCAACATTTATTTTGATTTTTT
>LSTEM334-18|Chilo_orichalcociliellus|ww04048|
AACTTTATATTTTATTTTTGGAATTTGAGCAGGAATAATTGGAACATCACTTAGACTCCTAATTCGAGCTGAATTAGGAA
CCCCTGGATCTTTAATTGGTGATGATCAAATTTATAATACTATTGTTACAGCTCATGCATTTATTATAATTTTTTTTATA
GTTATACCAATTATAATTGGAGGATTTGGAAATTGATTAGTACCTTTAATGTTAGGAGCTCCTGATATAGCCTTCCCACG
AATAAATAATATAAGATTTTGATTACTACCCCCATCACTAACTTTATTAATTTCTAGAAGAATTGTTGAAAATGGAGCTG
GAACTGGGTGAACAGTTTATCCCCCACTTTCATCCAATATTGCCCATGGTGGGAGTTCAGTAGATCTAGCAATTTTTTCT
CTTCATTTAGCTGGAATTTCCTCAATTTTAGGTGCTATTAATTTTATTACAACAATCATTAATATACGAATTAATGGATT
ATCATTTGATCAAATACCTTTATTTGTTTGATCTGTTGGTATTACAGCCTTATTATTATTACTTTCATTACCTGTTTTAG
CAGGAGCTATTACTATATTATTAACTGATCGAAATTTAAATACATCATTTTTTGATCCTGCTGGTGGAGGAGATCCAATT
CTTTATCAACATTTATTTTGATTTTTT
>LSTEM335-18|Chilo_orichalcociliellus|ww04049|
AACTTTATATTTTATTTTTGGAATTTGAGCAGGAATAATTGGAACATCACTTAGACTCCTAATTCGAGCTGAATTAGGAA
CCCCTGGATCTTTAATTGGTGATGATCAAATTTATAATACTATTGTTACAGCTCATGCATTTATTATAATTTTTTTTATA
GTTATACCAATTATAATTGGAGGATTTGGAAATTGATTAGTACCTTTAATGTTAGGAGCTCCTGATATAGCCTTCCCACG
AATAAATAATATAAGATTTTGATTACTACCCCCATCACTAACTTTATTAATTTCTAGAAGAATTGTTGAAAATGGAGCTG
GAACTGGGTGAACAGTTTATCCCCCACTTTCATCCAATATTGCCCATGGTGGGAGTTCAGTAGATCTAGCAATTTTTTCT
CTTCATTTAGCTGGAATTTCCTCAATTTTAGGTGCTATTAATTTTATTACAACAATCATTAATATACGAATTAATGGATT
ATCATTTGATCAAATACCTTTATTTGTTTGATCTGTTGGTATTACAGCCTTATTATTATTACTTTCATTACCTGTTTTAG
CAGGAGCTATTACTATATTATTAACTGATCGAAATTTAAATACATCATTTTTTGATCCTGCTGGTGGAGGAGATCCAATT
CTTTATCAACATTTATTTTGATTTTTT
>LSTEM336-18|Chilo_orichalcociliellus|ww04050|
AACTTTATATTTTATTTTTGGAATTTGAGCAGGAATAATTGGAACATCACTTAGACTCCTAATTCGAGCTGAATTAGGAA
CCCCTGGATCTTTAATTGGTGATGATCAAATTTATAATACTATTGTTACAGCTCATGCATTTATTATAATTTTTTTTATA
GTTATACCAATTATAATTGGAGGATTTGGAAATTGATTAGTACCTTTAATGTTAGGAGCTCCTGATATAGCCTTCCCACG
AATAAATAATATAAGATTTTGATTACTACCCCCATCACTAACTTTATTAATTTCTAGAAGAATTGTTGAAAATGGAGCTG
GAACTGGGTGAACAGTTTATCCCCCACTTTCATCCAATATTGCCCATGGTGGGAGTTCAGTAGATCTAGCAATTTTTTCT
CTTCATTTAGCTGGAATTTCCTCAATTTTAGGTGCTATTAATTTTATTACAACAATCATTAATATACGAATTAATGGATT
ATCATTTGATCAAATACCTTTATTTGTTTGATCTGTTGGTATTACAGCCTTATTATTATTACTTTCATTACCTGTTTTAG
CAGGAGCTATTACTATATTATTAACTGATCGAAATTTAAATACATCATTTTTTGATCCTGCTGGTGGAGGAGATCCAATT
CTTTATCAACATTTATTTTGATTTTTT
>LSTEM337-18|Chilo_orichalcociliellus|ww04051|
AACTTTATATTTTATTTTTGGAATTTGAGCAGGAATAATTGGAACATCACTTAGACTCCTAATTCGAGCTGAATTAGGAA
CCCCTGGATCTTTAATTGGTGATGATCAAATTTATAATACTATTGTTACAGCTCATGCATTTATTATAATTTTTTTTATA
GTTATACCAATTATAATTGGAGGATTTGGAAATTGATTAGTACCTTTAATGTTAGGAGCTCCTGATATAGCCTTCCCACG
AATAAATAATATAAGATTTTGATTACTACCCCCATCACTAACTTTATTAATTTCTAGAAGAATTGTTGAAAATGGAGCTG
GAACTGGGTGAACAGTTTATCCCCCACTTTCATCCAATATTGCCCATGGTGGGAGTTCAGTAGATCTAGCAATTTTTTCT
CTTCATTTAGCTGGAATTTCCTCAATTTTAGGTGCTATTAATTTTATTACAACAATCATTAATATACGAATTAATGGATT
ATCATTTGATCAAATACCTTTATTTGTTTGATCTGTTGGTATTACAGCCTTATTATTATTACTTTCATTACCTGTTTTAG
CAGGAGCTATTACTATATTATTAACTGATCGAAATTTAAATACATCATTTTTTGATCCTGCTGGTGGAGGAGATCCAATT
CTTTATCAACATTTATTTTGATTTTTT
>LSTEM338-18|Chilo_orichalcociliellus|ww04053|
AACTTTATATTTTATTTTTGGAATTTGAGCAGGAATAATTGGAACATCACTTAGACTCCTAATTCGAGCTGAATTAGGAA
CCCCTGGATCTTTAATTGGTGATGATCAAATTTATAATACTATTGTTACAGCTCATGCATTTATTATAATTTTTTTTATA

GTTATACCAATTATAATTGGAGGATTTGGAAATTGATTAGTACCTTTAATGTTAGGAGCTCCTGATATAGCCTTCCCACG
AATAAATAATATAAGATTTTGATTACTACCCCCATCACTAACTTTATTAATTTCTAGAAGAATTGTTGAAAATGGAGCTG
GAACTGGGTGAACAGTTTATCCCCCACTTTCATCCAATATTGCCCATGGTGGGAGTTCAGTAGATCTAGCAATTTTTTCT
CTTCATTTAGCTGGAATTTCCTCAATTTTAGGTGCTATTAATTTTATTACAACAATCATTAATATACGAATTAATGGATT
ATCATTTGATCAAATACCTTTATTTGTTTGATCTGTTGGTATTACAGCCTTATTATTATTACTTTCATTACCTGTTTTAG
CAGGAGCTATTACTATATTATTAACTGATCGAAATTTAAATACATCATTTTTTGATCCTGCTGGTGGAGGAGATCCAATT
CTTTATCAACATTTATTTTGATTTTTT
>LSTEM339-18|Chilo_sp_AM16|ww04054|
AACTTTATACTTTATTTTTGGAGTTTGAGCAGGAATAATTGGAACATCACTTAGACTTTTAATTCGAGCTGAATTAGGAA
CTCCAGGATCTTTAATTGGTGATGATCAAATCTACAATACTATTGTTACAGCTCACGCATTTATTATAATTTTTTTTATA
GTTATACCAATTATAATTGGAGGATTTGGAAATTGATTAGTACCTCTAATATTAGGAGCTCCTGATATAGCTTTTCCACG
AATAAATAATATAAGATTTTGATTATTACCCCCATCACTAACTTTACTAATTTCTAGAAGAATTGTAGAAACCGGAGCCG
GAACAGGATGAACAGTTTACCCCCCACTATCATCTAATATTGCACATGCTGGAAGTTCAGTAGATTTAGCAATTTTTTCC
CTCCATTTAGCTGGAATTTCTTCTATTTTAGGAGCTATTAACTTTATTACAACAATTATTAATATACGAATTAATGGATT
ATCATTTGATCAAATACCATTACTTGTTTGATCAGTTGGTATTACAGCTTTATTATTATTACTTTCTCTACCTGTTTTAG
CAGGTGCTATTACTATATTATTAACAGATCGAAATCTAAATACATCATTTTTTGATCCAGCTGGAGGGGGTGATCCAATT
CTTTATCAACACTTATTTTGATTTTTT
>LSTEM340-18|Chilo_orichalcociliellus|ww04056|
AACTTTATATTTTATTTTTGGAATTTGAGCAGGAATAATTGGAACATCACTTAGACTCCTAATTCGAGCTGAATTGGGAA
CCCCTGGATCTTTAATTGGTGATGATCAAATTTATAATACTATTGTTACAGCTCATGCATTTATTATAATTTTTTTTATA
GTTATACCAATTATAATTGGAGGATTTGGAAATTGATTAGTACCTTTAATGTTAGGAGCTCCTGATATAGCCTTCCCACG
AATAAATAATATAAGATTTTGATTACTACCCCCATCACTAACTTTATTAATTTCTAGAAGAATTGTTGAAAATGGAGCTG
GAACTGGGTGAACAGTTTATCCCCCACTTTCATCCAATATTGCCCATGGTGGGAGTTCAGTAGATCTAGCAATTTTTTCT
CTTCATTTAGCTGGAATTTCCTCAATTTTAGGTGCTATTAATTTTATTACAACAATCATTAATATACGAATTAATGGATT
ATCATTTGATCAAATACCTTTATTTGTTTGATCTGTTGGTATTACAGCCTTATTATTATTACTTTCATTACCTGTTTTAG
CAGGAGCTATTACTATATTATTAACTGATCGAAATTTAAATACATCATTTTTTGATCCTGCTGGTGGAGGAGATCCAATN
CTTTATCAACATTTATTTTGATTTTTT
>LSTEM341-18|Chilo_orichalcociliellus|ww04057|
AACTTTATATTTTATTTTTGGAATTTGAGCAGGAATAATTGGAACATCACTTAGACTCCTAATTCGAGCTGAATTGGGAA
CCCCTGGATCTTTAATTGGTGATGATCAAATTTATAATACTATTGTTACAGCTCATGCATTTATTATAATTTTTTTTATA
GTTATACCAATTATAATTGGAGGATTTGGAAATTGATTAGTACCTTTAATGTTAGGAGCTCCTGATATAGCCTTCCCACG
AATAAATAATATAAGATTTTGATTACTACCCCCATCACTAACTTTATTAATTTCTAGAAGAATTGTTGAAAATGGAGCTG
GAACTGGGTGAACAGTTTATCCCCCACTTTCATCCAATATTGCCCATGGTGGGAGTTCAGTAGATCTAGCAATTTTTTCT
CTTCATTTAGCTGGAATTTCCTCAATTTTAGGTGCTATTAATTTTATTACAACAATCATTAATATACGAATTAATGGATT
ATCATTTGATCAAATACCTTTATTTGTTTGATCTGTTGGTATTACAGCCTTATTATTATTACTTTCATTACCTGTTTTAG
CAGGAGCTATTACTATATTATTAACTGATCGAAATTTAAATACATCATTTTTTGATCCTGCTGGTGGAGGAGATCCAATT
CTTTATCAACATTWATTTTGATTTTTT
>LSTEM342-18|Chilo_orichalcociliellus|ww04058|
AACTTTATATTTTATTTTTGGNATTTGAGCAGGAATAATTGGAACATCACTTAGACTCCTAATTCGAGCTGAATTAGGAA
CCCCTGGATCTTTAATTGGTGATGATCAAATTTATAATACTATTGTTACAGCTCATGCATTTATTATAATTTTTTTTATA
GTTATACCAATTATAATTGGAGGATTTGGAAATTGATTAGTACCTTTAATGTTAGGAGCTCCTGATATAGCCTTCCCACG
AATAAATAATATAAGATTTTGATTACTACCCCCATCACTAACTTTATTAATTTCTAGAAGAATTGTTGAAAATGGAGCTG
GAACTGGGTGAACAGTTTATCCCCCACTTTCATCCAATATTGCCCATGGTGGGAGTTCAGTAGATCTAGCAATTTTTTCT
CTTCATTTAGCTGGAATTTCCTCAATTTTAGGTGCTATTAATTTTATTACAACAATCATTAATATACGAATTAATGGATT
ATCATTTGATCAAATACCTTTATTTGTTTGATCTGTTGGTATTACAGCCTTATTATTATTACTTTCATTACCTGTTTTAG
CAGGAGCTATTACTATATTATTAACTGATCGAAATTTAAATACATCATTTTTTGATCCTGCTGGTGGAGGAGATCCAATT
CTTTATCAACATTTATTTTGATTTTTT
>LSTEM343-18|Chilo_orichalcociliellus|ww04059|
AACTTTATATTTTATTTTTGGAATTTGAGCAGGAATAATTGGAACATCACTTAGACTCCTAATTCGAGCTGAATTAGGAA
CCCCTGGATCTTTAATTGGTGATGATCAAATTTATAATACTATTGTTACAGCTCATGCATTTATTATAATTTTTTTTATA
GTTATACCAATTATAATTGGAGGATTTGGAAATTGATTAGTACCTTTAATGTTAGGAGCTCCTGATATAGCCTTCCCACG
AATAAATAATATAAGATTTTGATTACTACCCCCATCACTAACTTTATTAATTTCTAGAAGAATTGTTGAAAATGGAGCTG
GAACTGGGTGAACAGTTTATCCCCCACTTTCATCCAATATTGCCCATGGTGGGAGTTCAGTAGATCTAGCAATTTTTTCT
CTTCATTTAGCTGGAATTTCCTCAATTTTAGGTGCTATTAATTTTATTACAACAATCATTAATATACGAATTAATGGATT
ATCATTTGATCAAATACCTTTATTTGTTTGATCTGTTGGTATTACAGCCTTATTATTATTACTTTCATTACCTGTTTTAG
CAGGAGCTATTACTATATTATTAACTGATCGAAATTTAAATACATCATTTTTTGATCCTGCTGGTGGAGGAGATCCAATT
CTTTATCAACATTTATTTTGATTTTTT

>LSTEM344-18|Chilo_orichalcociliellus|ww04060|
AACTTTATATTTTATTTTTGGAATTTGAGCAGGAATAATTGGAACATCACTTAGACTCCTAATTCGAGCTGAATTAGGAA
CCCCTGGATCTTTAATTGGTGATGATCAAATTTATAATACTATTGTTACAGCTCATGCATTTATTATAATTTTTTTTATA
GTTATACCAATTATAATTGGAGGATTTGGAAATTGATTAGTACCTTTAATGTTAGGAGCTCCTGATATAGCCTTCCCACG
AATAAATAATATAAGATTTTGATTACTACCCCCATCACTAACTTTATTAATTTCTAGAAGAATTGTTGAAAATGGAGCTG
GAACTGGGTGAACAGTTTATCCCCCACTTTCATCCAATATTGCCCATGGTGGGAGTTCAGTAGATCTAGCAATTTTTTCT
CTTCATTTAGCTGGAATTTCCTCAATTTTAGGTGCTATTAATTTTATTACAACAATCATTAATATACGAATTAATGGATT
ATCATTTGATCAAATACCTTTATTTGTTTGATCTGTTGGTATTACAGCCTTATTATTATTACTTTCATTACCTGTTTTAG
CAGGAGCTATTACTATATTATTAACTGATCGAAATTTAAATACATCATTTTTTGATCCTGCTGGTGGAGGAGATCCAATT
CTTTATCAACATTTATTTTGATTTTTT
>LSTEM345-18|Chilo_orichalcociliellus|ww04061|
AACTTTATATTTTATTTTTGGAATTTGAGCAGGAATAATTGGAACATCACTTAGACTCCTAATTCGAGCTGAATTAGGAA
CCCCTGGATCTTTAATTGGTGATGATCAAATTTATAATACTATTGTTACAGCTCATGCATTTATTATAATTTTTTTTATA
GTTATACCAATTATAATTGGAGGATTTGGAAATTGATTAGTACCTTTAATGTTAGGAGCTCCTGATATAGCCTTCCCACG
AATAAATAATATAAGATTTTGATTACTACCCCCATCACTAACTTTATTAATTTCTAGAAGAATTGTTGAAAATGGAGCTG
GAACTGGGTGAACAGTTTATCCCCCACTTTCATCCAATATTGCCCATGGTGGGAGTTCAGTAGATCTAGCAATTTTTTCT
CTTCATTTAGCTGGAATTTCCTCAATTTTAGGTGCTATTAATTTTATTACAACAATCATTAATATACGAATTAATGGATT
ATCATTTGATCAAATACCTTTATTTGTTTGATCTGTTGGTATTACAGCCTTATTATTATTACTTTCATTACCTGTTTTAG
CAGGAGCTATTACTATATTATTAACTGATCGAAATTTAAATACATCATTTTTTGATCCTGCTGGTGGAGGAGATCCAATT
CTTTATCAACATTTATTTTGATTTTTT
>LSTEM346-18|Chilo_orichalcociliellus|ww04062|
AACTTTATATTTTATTTTTGGAATTTGAGCAGGAATAATTGGAACATCACTTAGACTCCTAATTCGAGCTGAATTAGGAA
CCCCTGGATCTTTAATTGGTGATGATCAAATTTATAATACTATTGTTACAGCTCATGCATTTATTATAATTTTTTTTATA
GTTATACCAATTATAATTGGAGGATTTGGAAATTGATTAGTACCTTTAATGTTAGGAGCTCCTGATATAGCCTTCCCACG
AATAAATAATATAAGATTTTGATTACTACCCCCATCACTAACTTTATTAATTTCTAGAAGAATTGTTGAAAATGGAGCTG
GAACTGGGTGAACAGTTTATCCCCCACTTTCATCCAATATTGCCCATGGTGGGAGTTCAGTAGATCTAGCAATTTTTTCT
CTTCATTTAGCTGGAATTTCCTCAATTTTAGGTGCTATTAATTTTATTACAACAATCATTAATATACGAATTAATGGATT
ATCATTTGATCAAATACCTTTATTTGTTTGATCTGTTGGTATTACAGCCTTATTATTATTACTTTCATTACCTGTTTTAG
CAGGAGCTATTACTATATTATTAACTGATCGAAATTTAAATACATCATTTTTTGATCCTGCTGGTGGAGGAGATCCAATT
CTTTATCAACATTTATTTTGATTTTTT
>LSTEM347-18|Chilo_orichalcociliellus|ww04063|
AACTTTATATTTTATTTTTGGAATTTGAGCAGGAATAATTGGAACATCACTTAGACTCCTAATTCGAGCTGAATTAGGAA
CCCCTGGATCTTTAATTGGTGATGATCAAATTTATAATACTATTGTTACAGCTCATGCATTTATTATAATTTTTTTTATA
GTTATACCAATTATAATTGGAGGATTTGGAAATTGATTAGTACCTTTAATGTTAGGAGCTCCTGATATAGCCTTCCCACG
AATAAATAATATAAGATTTTGATTACTACCCCCATCACTAACTTTATTAATTTCTAGAAGAATTGTTGAAAATGGAGCTG
GAACTGGGTGAACAGTTTATCCCCCACTTTCATCCAATATTGCCCATGGTGGGAGTTCAGTAGATCTAGCAATTTTTTCT
CTTCATTTAGCTGGAATTTCCTCAATTTTAGGTGCTATTAATTTTATTACAACAATCATTAATATACGAATTAATGGATT
ATCATTTGATCAAATACCTTTATTTGTTTGATCTGTTGGTATTACAGCCTTATTATTATTACTTTCATTACCTGTTTTAG
CAGGAGCTATTACTATATTATTAACTGATCGAAATTTAAATACATCATTTTTTGATCCTGCTGGTGGAGGAGATCCAATT
CTTTATCAACATTTATTTTGATTTTTT
>LSTEM348-18|Chilo_orichalcociliellus|ww04064|
AACTTTATATTTTATTTTTGGAATTTGAGCAGGAATAATTGGAACATCACTTAGACTCCTAATTCGAGCTGAATTAGGAA
CCCCTGGATCTTTAATTGGTGATGATCAAATTTATAATACTATTGTTACAGCTCATGCATTTATTATAATTTTTTTTATA
GTTATACCAATTATAATTGGAGGATTTGGAAATTGATTAGTACCTTTAATGTTAGGAGCTCCTGATATAGCCTTCCCACG
AATAAATAATATAAGATTTTGATTACTACCCCCATCACTAACTTTATTAATTTCTAGAAGAATTGTTGAAAATGGAGCTG
GAACTGGGTGAACAGTTTATCCCCCACTTTCATCCAATATTGCCCATGGTGGGAGTTCAGTAGATCTAGCAATTTTTTCT
CTTCATTTAGCTGGAATTTCCTCAATTTTAGGTGCTATTAATTTTATTACAACAATCATTAATATACGAATTAATGGATT
ATCATTTGATCAAATACCTTTATTTGTTTGATCTGTTGGTATTACAGCCTTATTATTATTACTTTCATTACCTGTTTTAG
CAGGAGCTATTACTATATTATTAACTGATCGAAATTTAAATACATCATTTTTTGATCCTGCTGGTGGAGGAGATCCAATT
CTTTATCAACATTTATTTTGATTTTTT
>LSTEM349-18|Chilo_orichalcociliellus|ww04065|
AACTTTATATTTTATTTTTGGAATTTGAGCAGGAATAATTGGCACATCACTTAGACTCCTAATTCGAGCTGAATTAGGAA
CCCCTGGATCTTTAATTGGTGATGATCAAATTTATAATACTATTGTTACAGCTCATGCATTTATTATAATTTTTTTTATA
GTTATACCAATTATAATTGGAGGATTTGGAAATTGATTAGTACCTTTAATGTTAGGAGCTCCTGATATAGCCTTCCCACG
AATAAATAATATAAGATTTTGATTACTACCCCCATCACTAACTTTATTAATTTCTAGAAGAATTGTTGAAAATGGAGCTG
GAACTGGGTGAACAGTTTATCCCCCACTTTCATCCAATATTGCCCATGGTGGGAGTTCAGTAGATCTAGCAATTTTTTCT
CTTCATTTAGCTGGAATTTCCTCAATTTTAGGTGCTATTAATTTTATTACAACAATCATTAATATACGAATTAATGGATT

ATCATTTGATCAAATACCTTTATTTGTTTGATCTGTTGGTATTACAGCCTTATTATTATTACTTTCATTACCTGTTTTAG
CAGGAGCTATTACTATATTATTAACTGATCGAAATTTAAATACATCATTTTTTGATCCTGCTGGTGGAGGAGATCCAATT
CTTTATCAACATT-------------
>LSTEM350-18|Chilo_orichalcociliellus|ww04066|
AACTTTATATTTTATTTTTGGAATTTGAGCAGGAATAATTGGAACATCACTTAGACTCCTAATTCGAGCTGAATTAGGAA
CCCCTGGATCTTTAATTGGTGATGATCAAATTTATAATACTATTGTTACAGCTCATGCATTTATTATAATTTTTTTTATA
GTTATACCAATTATAATTGGAGGATTTGGAAATTGATTAGTACCTTTAATGTTAGGAGCTCCTGATATAGCCTTCCCACG
AATAAATAATATAAGATTTTGATTACTACCCCCATCACTAACTTTATTAATTTCTAGAAGAATTGTTGAAAATGGAGCTG
GAACTGGGTGAACAGTTTATCCCCCACTTTCATCCAATATTGCCCATGGTGGGAGTTCAGTAGATCTAGCAATTTTTTCT
CTTCATTTAGCTGGAATTTCCTCAATTTTAGGTGCTATTAATTTTATTACAACAATCATTAATATACGAATTAATGGATT
ATCATTTGATCAAATACCTTTATTTGTTTGATCTGTTGGTATTACAGCCTTATTATTATTACTTTCATTACCTGTTTTAG
CAGGAGCTATTACTATATTATTAACTGATCGAAATTTAAATACATCATTTTTTGATCCTGCTGGTGGAGGAGATCCAATT
CTTTATCAACATTTATTTTGATTTTTT
>LSTEM351-18|Chilo_orichalcociliellus|ww04067|
AACTTTATATTTTATTTTTGGNATTTGAGCAGGAATAATTGGAACATCACTTAGACTCCTAATTCGAGCTGAATTAGGAA
CCCCTGGATCTTTAATTGGTGATGATCAAATTTATAATACTATTGTTACAGCTCATGCATTTATTATAATTTTTTTTATA
GTTATACCAATTATAATTGGAGGATTTGGAAATTGATTAGTACCTTTAATGTTAGGAGCTCCTGATATAGCCTTCCCACG
AATAAATAATATAAGATTTTGATTACTACCCCCATCACTAACTTTATTAATTTCTAGAAGAATTGTTGAAAATGGAGCTG
GAACTGGGTGAACAGTTTATCCCCCACTTTCATCCAATATTGCCCATGGTGGGAGTTCAGTAGATCTAGCAATTTTTTCT
CTTCATTTAGCTGGAATTTCCTCAATTTTAGGTGCTATTAATTTTATTACAACAATCATTAATATACGAATTAATGGGTT
ATCATTTGATCAAATACCTTTATTTGTTTGATCTGTTGGTATTACAGCCTTATTATTATTACTTTCATTACCTGTTTTAG
CAGGAGCTATTACTATATTATTAACTGATCGAAATTTAAATACATCATTTTTTGATCCTGCTGGTGGAGGAGATCCAATT
CTTTATCAACATTTATTTTGATTTTTT
>LSTEM352-18|Chilo_orichalcociliellus|ww04068|
AACTTTATATTTTATTTTTGGAATTTGAGCAGGAATAATTGGAACATCACTTAGACTCCTAATTCGAGCTGAATTAGGAA
CCCCTGGATCTTTAATTGGTGATGATCAAATTTATAATACTATTGTTACAGCTCATGCATTTATTATAATTTTTTTTATA
GTTATACCAATTATAATTGGAGGATTTGGAAATTGATTAGTACCTTTAATGTTAGGAGCTCCTGATATAGCCTTCCCACG
AATAAATAATATAAGATTTTGATTACTACCCCCATCACTAACTTTATTAATTTCTAGAAGAATTGTTGAAAATGGAGCTG
GAACTGGGTGAACAGTTTATCCCCCACTTTCATCCAATATTGCCCATGGTGGGAGTTCAGTAGATCTAGCAATTTTTTCT
CTTCATTTAGCTGGAATTTCCTCAATTTTAGGTGCTATTAATTTTATTACAACAATCATTAATATACGAATTAATGGGTT
ATCATTTGATCAAATACCTTTATTTGTTTGATCTGTTGGTATTACAGCCTTATTATTATTACTTTCATTACCTGTTTTAG
CAGGAGCTATTACTATATTATTAACTGATCGAAATTTAAATACATCATTTTTTGATCCTGCTGGTGGAGGAGATCCAATT
CTTTATCAACATTTATTTTGATTTTTT
>LSTEM353-18|Chilo_orichalcociliellus|ww04069|
AACTTTATATTTTATTTTTGGAATTTGAGCAGGAATAATTGGAACATCACTTAGACTCCTAATTCGAGCTGAATTAGGAA
CCCCTGGATCTTTAATTGGTGATGATCAAATTTATAATACTATTGTTACAGCTCATGCATTTATTATAATTTTTTTTATA
GTTATACCAATTATAATTGGAGGATTTGGAAATTGATTAGTACCTTTAATGTTAGGAGCTCCTGATATAGCCTTCCCACG
AATAAATAATATAAGATTTTGATTACTACCCCCATCACTAACTTTATTAATTTCTAGAAGAATTGTTGAAAATGGAGCTG
GAACTGGGTGAACAGTTTATCCCCCACTTTCATCCAATATTGCCCATGGTGGGAGTTCAGTAGATCTAGCAATTTTTTCT
CTTCATTTAGCTGGAATTTCCTCAATTTTAGGTGCTATTAATTTTATTACAACAATCATTAATATACGAATTAATGGATT
ATCATTTGATCAAATACCTTTATTTGTTTGATCTGTTGGTATTACAGCCTTATTATTATTACTTTCATTACCTGTTTTAG
CAGGAGCTATTACTATATTATTAACTGATCGAAATTTAAATACATCATTTTTTGATCCTGCTGGTGGAGGAGATCCAATT
CTTTATCAACATTTATTTTGATTTTTT
>LSTEM354-18|Chilo_orichalcociliellus|ww04070|
AACTTTATATTTTATTTTTGGAATTTGAGCAGGAATAATTGGAACATCACTTAGACTCCTAATTCGAGCTGAATTAGGAA
CCCCTGGATCTTTAATTGGTGATGATCAAATTTATAATACTATTGTTACAGCTCATGCATTTATTATAATTTTTTTTATA
GTTATACCAATTATAATTGGAGGATTTGGAAATTGATTAGTACCTTTAATGTTAGGAGCTCCTGATATAGCCTTCCCACG
AATAAATAATATAAGATTTTGATTACTACCCCCATCACTAACTTTATTAATTTCTAGAAGAATTGTTGAAAATGGAGCTG
GAACTGGGTGAACAGTTTATCCCCCACTTTCATCCAATATTGCCCATGGTGGGAGTTCAGTAGATCTAGCAATTTTTTCT
CTTCATTTAGCTGGAATTTCCTCAATTTTAGGTGCTATTAATTTTATTACAACAATCATTAATATACGAATTAATGGATT
ATCATTTGATCAAATACCTTTATTTGTTTGATCTGTTGGTATTACAGCCTTATTATTATTACTTTC--------------
----

>LSTEM355-18|Chilo_orichalcociliellus|ww04071|
AACTTTATATTTTATTTTTGGAATTTGAGCAGGAATAATTGGAACATCACTTAGACTCCTAATTCGAGCTGAATTAGGAA
CCCCTGGATCTTTAATTGGTGATGATCAAATTTATAATACTATTGTTACAGCTCATGCATTTATTATAATTTTTTTTATA
GTTATACCAATTATAATTGGAGGATTTGGAAATTGATTAGTACCTTTAATGTTAGGAGCTCCTGATATAGCCTTCCCACG

AATAAATAATATAAGATTTTGATTACTACCCCCATCACTAACTTTATTAATTTCTAGAAGAATTGTTGAAAATGGAGCTG
GAACTGGGTGAACAGTTTATCCCCCACTTTCATCCAATATTGCCCATGGTGGGAGTTCAGTAGATCTAGCAATTTTTTCT
CTTCATTTAGCTGGAATTTCCTCAATTTTAGGTGCTATTAATTTTATTACAACAATCATTAATATACGAATTAATGGATT
ATCATTTGATCAAATACCTTTATTTGTTTGATCTGTTGGTATTACAGCCTTATTATTATTACTTTCATTACCTGTTTTAG
CAGGAGCTATTACTATATTATTAACTGATCGAAATTTAAATACATCATTTTTTGATCCTGCTGGTGGAGGAGATCCAATT
CTTTATCAACATTTATTTTGATTTTTT
>LSTEM356-18|Chilo_orichalcociliellus|ww04072|
AACTTTATATTTTATTTTTGGAATTTGAGCAGGAATAATTGGAACATCACTTAGACTCCTAATTCGAGCTGAATTAGGAA
CCCCTGGATCTTTAATTGGTGATGATCAAATTTATAATACTATTGTTACAGCTCATGCATTTATTATAATTTTTTTTATA
GTTATACCAATTATAATTGGAGGATTTGGAAATTGATTAGTACCTTTAATGTTAGGAGCTCCTGATATAGCCTTCCCACG
AATAAATAATATAAGATTTTGATTACTACCCCCATCACTAACTTTATTAATTTCTAGAAGAATTGTTGAAAATGGAGCTG
GAACTGGGTGAACAGTTTATCCCCCACTTTCATCCAATATTGCCCATGGTGGGAGTTCAGTAGATCTAGCAATTTTTTCT
CTTCATTTAGCTGGAATTTCCTCAATTTTAGGTGCTATTAATTTTATTACAACAATCATTAATATACGAATTAATGGATT
ATCATTTGATCAAATACCTTTATTTGTTTGATCTGTTGGTATTACAGCCTTATTATTATTACTTTCATTACCTGTTTTAG
CAGGAGCTATTACTATATTATTAACTGATCGAAATTTAAATACATCATTTTTTGATCCTGCTGGTGGAGGAGATCCAATT
CTTTATCAACATTTATTTTGATTTTTT
>LSTEM357-18|Chilo_orichalcociliellus|ww04073|
AACTTTATATTTTATTTTTGGAATTTGAGCAGGAATAATTGGAACATCACTTAGACTCCTAATTCGAGCTGAATTGGGAA
CCCCTGGATCTTTAATTGGTGATGATCAAATTTATAATACTATTGTTACAGCTCATGCATTTATTATAATTTTTTTTATA
GTTATACCAATTATAATTGGAGGATTTGGAAATTGATTAGTACCTTTAATGTTAGGAGCTCCTGATATAGCCTTCCCACG
AATAAATAATATAAGATTTTGATTACTACCCCCATCACTAACTTTATTAATTTCTAGAAGAATTGTTGAAAATGGAGCTG
GAACTGGGTGAACAGTTTATCCCCCACTTTCATCCAATATTGCCCATGGTGGGAGTTCAGTAGATCTAGCAATTTTTTCT
CTTCATTTAGCTGGAATTTCCTCAATTTTAGGTGCTATTAATTTTATTACAACAATCATTAATATACGAATTAATGGATT
ATCATTTGATCAAATACCTTTATTTGTTTGATCTGTTGGTATTACAGCCTTATTATTATTACTTTCATTACCTGTTTTAG
CAGGAGCTATTACTATATTATTAACTGATCGAAATTTAAATACATCATTTTTTGATCCTGCTGGTGGAGGAGATCCAATT
CTTTATCAACATTTATTTTGATTTTTT
>LSTEM358-18|Chilo_orichalcociliellus|ww04074|
AACTTTATATTTTATTTTTGGAATTTGAGCAGGAATAATTGGAACATCACTTAGACTCCTAATTCGAGCTGAATTAGGAA
CCCCTGGATCTTTAATTGGTGATGATCAAATTTATAATACTATTGTTACAGCTCATGCATTTATTATAATTTTTTTTATA
GTTATACCAATTATAATTGGAGGATTTGGAAATTGATTAGTACCTTTAATGTTAGGAGCTCCTGATATAGCCTTCCCACG
AATAAATAATATAAGATTTTGATTACTACCCCCATCACTAACTTTATTAATTTCTAGAAGAATTGTTGAAAATGGAGCTG
GAACTGGGTGAACAGTTTATCCCCCACTTTCATCCAATATTGCCCATGGTGGGAGTTCAGTAGATCTAGCAATTTTTTCT
CTTCATTTAGCTGGAATTTCCTCAATTTTAGGTGCTATTAATTTTATTACAACAATCATTAATATACGAATTAATGGATT
ATCATTTGATCAAATACCTTTATTTGTTTGATCTGTTGGTATTACAGCCTTATTATTATTACTTTCATTACCTGTTTTAG
CAGGAGCTATTACTATATTATTAACTGATCGAAATTTAAATACATCATTTTTTGATCCTGCTGGTGGAGGAGATCCAATT
CTTTATCAACATTTATTTTGATTTTTT
>LSTEM359-18|Chilo_orichalcociliellus|ww04075|
AACTTTATATTTTATTTTTGGAATTTGAGCAGGAATAATTGGAACATCACTTAGACTCCTAATTCGAGCTGAATTGGGAA
CCCCTGGATCTTTAATTGGTGATGATCAAATTTATAATACTATTGTTACAGCTCATGCATTTATTATAATTTTTTTTATA
GTTATACCAATTATAATTGGAGGATTTGGAAATTGATTAGTACCTTTAATGTTAGGAGCTCCTGATATAGCCTTCCCACG
AATAAATAATATAAGATTTTGATTACTACCCCCATCACTAACTTTATTAATTTCTAGAAGAATTGTTGAAAATGGAGCTG
GAACTGGGTGAACAGTTTATCCCCCACTTTCATCCAATATTGCCCATGGTGGGAGTTCAGTAGATCTAGCAATTTTTTCT
CTTCATTTAGCTGGAATTTCCTCAATTTTAGGTGCTATTAATTTTATTACAACAATCATTAATATACGAATTAATGGATT
ATCATTTGATCAAATACCTTTATTTGTTTGATCTGTTGGTATTACAGCCTTATTATTATTACTTTCATTACCTGTTTTAG
CAGGAGCTATTACTATATTATTAACTGATCGAAATTTAAATACATCATTTTTTGATCCTGCTGGTGGAGGAGATCCAATT
CTTTATCAACATTTATTTTGATTTTTT
>LSTEM360-18|Chilo_orichalcociliellus|ww04076|
AACTTTATATTTTATTTTTGGAATTTGAGCAGGAATAATTGGAACATCACTTAGACTCCTAATTCGAGCTGAATTAGGAA
CCCCTGGATCTTTAATTGGTGATGATCAAATTTATAATACTATTGTTACAGCTCATGCATTTATTATAATTTTTTTTATA
GTTATACCAATTATAATTGGAGGATTTGGAAATTGATTAGTACCTTTAATGTTAGGAGCTCCTGATATAGCCTTCCCACG
AATAAATAATATAAGATTTTGATTACTACCCCCATCACTAACTTTATTAATTTCTAGAAGAATTGTTGAAAATGGAGCTG
GAACTGGGTGAACAGTTTATCCCCCACTTTCATCCAATATTGCCCATGGTGGAAGTTCAGTAGATCTAGCAATTTTTTCT
CTTCATTTAGCTGGAATTTCCTCAATTTTAGGTGCTATTAATTTTATTACAACAATCATTAATATACGAATTAATGGATT
ATCATTTGATCAAATACCTTTATTTGTTTGATCTGTTGGTATTACAGCCTTATTATTATTACTTTCATTACCTGTTTTAG
CAGGAGCTATTACTATATTATTAACTGATCGAAATTTAAATACATCATTTTTTGATCCTGCTGGTGGAGGAGATCCAATT
CTTTATCAACATTTATTTTGATTTTTT
>LSTEM361-18|Chilo_orichalcociliellus|ww04077|

AACTTTATATTTTATTTTTGGAATTTGAGCAGGAATAATTGGAACATCACTTAGACTCCTAATTCGAGCTGAATTAGGAA
CCCCTGGATCTTTAATTGGTGATGATCAAATTTATAATACTATTGTTACAGCTCATGCATTTATTATAATTTTTTTTATA
GTTATACCAATTATAATTGGAGGATTTGGAAATTGATTAGTACCTTTAATGTTAGGAGCTCCTGATATAGCCTTCCCACG
AATAAATAATATAAGATTTTGATTACTACCCCCATCACTAACTTTATTAATTTCTAGAAGAATTGTTGAAAATGGAGCTG
GAACTGGGTGAACAGTTTATCCCCCACTTTCATCCAATATTGCCCATGGTGGAAGTTCAGTAGATCTAGCAATTTTTTCT
CTTCATTTAGCTGGAATTTCCTCAATTTTAGGTGCTATTAATTTTATTACAACAATCATTAATATACGAATTAATGGATT
ATCATTTGATCAAATACCTTTATTTGTTTGATCTGTTGGTATTACAGCCTTATTATTATTACTTTCATTACCTGTTTTAG
CAGGAGCTATTACTATATTATTAACTGATCGAAATTTAAATACATCATTTTTTGATCCTGCTGGTGGAGGAGATCCAATT
CTTTATCAACATTTATTTTGATTTTTT
>LSTEM362-18|Chilo_orichalcociliellus|ww04078|
AACTTTATATTTTATTTTTGGAATTTGAGCAGGAATAATTGGAACATCACTTAGACTCCTAATTCGAGCTGAATTAGGAA
CCCCTGGATCTTTAATTGGTGATGATCAAATTTATAATACTATTGTTACAGCTCATGCATTTATTATAATTTTTTTTATA
GTTATACCAATTATAATTGGAGGATTTGGAAATTGATTAGTACCTTTAATGTTAGGAGCTCCTGATATAGCCTTCCCACG
AATAAATAATATAAGATTTTGATTACTACCCCCATCACTAACTTTATTAATTTCTAGAAGAATTGTTGAAAATGGAGCTG
GAACTGGGTGAACAGTTTATCCCCCACTTTCATCCAATATTGCCCATGGTGGAAGTTCAGTAGATCTAGCAATTTTTTCT
CTTCATTTAGCTGGAATTTCCTCAATTTTAGGTGCTATTAATTTTATTACAACAATCATTAATATACGAATTAATGGATT
ATCATTTGATCAAATACCTTTATTTGTTTGATCTGTTGGTATTACAGCCTTATTATTATTACTTTCATTACCTGTTTTAG
CAGGAGCTATTACTATATTATTAACTGATCGAAATTTAAATACATCATTTTTTGATCCTGCTGGTGGAGGAGATCCAATT
CTTTATCAACATTTATTTTGATTTT--
>LSTEM363-18|Chilo_orichalcociliellus|ww04080|
AACTTTATATTTTATTTTTGGAATTTGAGCAGGAATAATTGGAACATCACTTAGACTCCTAATTCGAGCTGAATTAGGAA
CCCCTGGATCTTTAATTGGTGATGATCAAATTTATAATACTATTGTTACAGCTCATGCATTTATTATAATTTTTTTTATA
GTTATACCAATTATAATTGGAGGATTTGGAAATTGATTAGTACCTTTAATGTTAGGAGCTCCTGATATAGCCTTCCCACG
AATAAATAATATAAGATTTTGATTACTACCCCCATCACTAACTTTATTAATTTCTAGAAGAATTGTTGAAAATGGAGCTG
GAACTGGGTGAACAGTTTATCCCCCACTTTCATCCAATATTGCCCATGGTGGGAGTTCAGTAGATCTAGCAATTTTTTCT
CTTCATTTAGCTGGAATTTCCTCAATTTTAGGTGCTATTAATTTTATTACAACAATCATTAATATACGAATTAATGGATT
ATCATTTGATCAAATACCTTTATTTGTTTGATCTGTTGGTATTACAGCCTTATTATTATTACTTTCATTACCTGTTTTAG
CAGGAGCTATTACTATATTATTAACTGATCGAAATTTAAATACATCATTTTTTGATCCTGCTGGTGGAGGAGATCCAATT
CTTTATCAACATTTATTTTGATTTTTT
>LSTEM364-18|Chilo_orichalcociliellus|ww04081|
AACTTTATATTTTATTTTTGGAATTTGAGCAGGAATAATTGGAACATCACTTAGACTCCTAATTCGAGCTGAATTAGGAA
CCCCTGGATCTTTAATTGGTGATGATCAAATTTATAATACTATTGTTACAGCTCATGCATTTATTATAATTTTTTTTATA
GTTATACCAATTATAATTGGAGGATTTGGAAATTGATTAGTACCTTTAATGTTAGGAGCTCCTGATATAGCCTTCCCACG
AATAAATAATATAAGATTTTGATTACTACCCCCATCACTAACTTTATTAATTTCTAGAAGAATTGTTGAAAATGGAGCTG
GAACTGGGTGAACAGTTTATCCCCCACTTTCATCCAATATTGCCCATGGTGGGAGTTCAGTAGATCTAGCAATTTTTTCT
CTTCATTTAGCTGGAATTTCCTCAATTTTAGGTGCTATTAATTTTATTACAACAATCATTAATATACGAATTAATGGATT
ATCATTTGATCAAATACCTTTATTTGTTTGATCTGTTGGTATTACAGCCTTATTATTATTACTTTCATTACCTGTTTTAG
CAGGAGCTATTACTATATTATTAACTGATCGAAATTTAAATACATCATTTTTTGATCCTGCTGGTGGAGGAGATCCAATT
CTTTATCAACATTTATTTTGATTTTTT
>LSTEM365-18|Chilo_sp_AM16|ww04082|
AACTTTATATTTTATTTTTGGAGTTTGAGCAGGAATAATTGGAACATCACTTAGACTTTTAATTCGAGCTGAATTAGGAA
CTCCAGGATCTTTAATTGGTGATGATCAAATCTACAATACTATTGTTACAGCTCACGCATTTATTATAATTTTTTTTATA
GTTATACCAATTATAATTGGAGGATTTGGAAATTGATTAGTACCTCTAATATTAGGAGCTCCTGATATAGCTTTTCCACG
AATAAATAATATAAGATTTTGATTATTACCCCCATCACTAACTTTACTAATTTCTAGAAGAATCGTAGAAACCGGAGCCG
GAACAGGATGAACAGTTTACCCCCCACTATCATCTAATATTGCACATGCTGGAAGTTCAGTAGATTTAGCAATTTTTTCC
CTCCATTTAGCTGGAATTTCTTCTATTTTAGGAGCTATTAACTTTATTACAACAATTATTAATATACGAATTAATGGATT
ATCATTTGATCAAATACCATTACTTGTTTGATCAGTTGGTATTACAGCTTTATTATTATTACTTTCTCTACCTGTTTTAG
CAGGTGCTATTACTATATTATTAACAGATCGAAATCTAAATACATCATTTTTTGATCCAGCTGGAGGGGGTGATCCAATT
CTTTATCAACACTTATTTTGATTTTTT
>LSTEM366-18|Chilo_sp_AM16|ww04083|
AACTTTATACTTTATTTTTGGAGTTTGAGCAGGAATAATTGGAACATCACTTAGACTTTTAATTCGAGCTGAATTAGGAA
CTCCAGGATCTTTAATTGGTGATGATCAAATCTACAATACTATTGTTACAGCTCACGCATTTATTATAATTTTTTTTATA
GTTATACCAATTATAATTGGAGGATTTGGAAATTGATTAGTACCTCTAATATTAGGAGCTCCTGATATAGCTTTTCCACG
AATAAATAATATAAGATTTTGATTATTACCCCCATCACTAACTTTACTAATTTCTAGAAGAATCGTAGAAACCGGAGCCG
GAACAGGATGAACAGTTTACCCCCCACTATCATCTAATATTGCACATGCTGGAAGTTCAGTAGATTTAGCAATTTTTTCC
CTCCATTTAGCTGGAATTTCTTCTATTTTAGGAGCTATTAACTTTATTACAACAATTATTAATATACGAATTAATGGATT
ATCATTTGATCAAATACCATTACTTGTTTGATCAGTTGGTATTACAGCTTTATTATTATTACTTTCTCTACCTGTTTTAG

CAGGTGCTATTACTATATTATTAACAGATCGAAATCTAAATACATCATTTTTTGATCCAGCTGGAGGGGGTGATCCAATT
CTTTATCAACACTTATTTTGATTTTTT
>LSTEM367-18|Chilo_sp_AM16|ww04084|
AACTTTATATTTTATTTTTGGAGTTTGAGCAGGAATAATTGGAACATCACTTAGACTTTTAATTCGAGCTGAATTAGGAA
CTCCAGGATCTTTAATTGGTGATGATCAAATCTACAATACTATTGTTACAGCTCACGCATTTATTATAATTTTTTTTATA
GTTATACCAATTATAATTGGAGGATTTGGAAATTGATTAGTACCTCTAATATTAGGAGCTCCTGATATAGCTTTTCCACG
AATAAATAATATAAGATTTTGATTATTACCCCCATCACTAACTTTACTAATTTCTAGAAGAATCGTAGAAACCGGAGCCG
GAACAGGATGAACAGTTTACCCCCCACTATCATCTAATATTGCACATGCTGGAAGTTCAGTAGATTTAGCAATTTTTTCC
CTCCATTTAGCTGGAATTTCTTCTATTTTAGGAGCTATTAACTTTATTACAACAATTATTAATATACGAATTAATGGATT
ATCATTTGATCAAATACCATTACTTGTTTGATCAGTTGGTATTACAGCTTTATTATTATTACTTTCTCTACCTGTTTTAG
CAGGTGCTATTACTATATTATTAACAGATCGAAATCTAAATACATCATTTTTTGATCCAGCTGGAGGGGGTGATCCAATT
CTTTATCAACACTTATTTTGATTTTTT
>LSTEM368-18|Chilo_sp_AM16|ww04085|
AACTTTATATTTTATTTTTGGAGTTTGAGCAGGAATAATTGGAACATCACTTAGACTTTTAATTCGAGCTGAATTAGGAA
CTCCAGGATCTTTAATTGGTGATGATCAAATCTACAATACTATTGTTACAGCTCACGCATTTATTATAATTTTTTTTATA
GTTATACCAATTATAATTGGAGGATTTGGAAATTGATTAGTACCTCTAATATTAGGAGCTCCTGATATAGCTTTTCCACG
AATAAATAATATAAGATTTTGATTATTACCCCCATCACTAACTTTACTAATTTCTAGAAGAATCGTAGAAACCGGAGCCG
GAACAGGATGAACAGTTTACCCCCCACTATCATCTAATATTGCACATGCTGGAAGTTCAGTAGATTTAGCAATTTTTTCC
CTCCATTTAGCTGGAATTTCTTCTATTTTAGGAGCTATTAACTTTATTACAACAATTATTAATATACGAATTAATGGATT
ATCATTTGATCAAATACCATTACTTGTTTGATCAGTTGGTATTACAGCTTTATTATTATTACTTTCTCTACCTGTTTTAG
CAGGTGCTATTACTATATTATTAACAGATCGAAATCTAAATACAT----------------------------------
----
>LSTEM369-18|Chilo_orichalcociliellus|ww04086|
---TAATTCGAGCTGAATTGGGAA
CCCCTGGATCTTTAATTGGTGATGATCAAATTTATAATACTATTGTTACAGCTCATGCATTTATTATAATTTTTTTTATA
GTTATACCAATTATAATTGGAGGATTTGGAAATTGATTAGTACCTTTAATATTAGGAGCTCCTGATATAGCCTTTCCACG
AATAAATAATATAAGATTTTGATTACTACCCCCATCACTAACTTTATTAATTTCTAGAAGAATTGTTGAAAATGGAGCTG
GAACTGGATGAACAGTTTATCCCCCACTTTCATCCAATATTGCCCATGGTGGGAGTTCAGTAGATCTAGCAATTTTTTCT
CTTCATTTAGCTGGAATTTCCTCAATTTTAGGTGCTATTAATTTTATTACAACAATCATTAATATACGAATTAATGGATT
ATCATTTGATCAAATACCTTTATTTGTTTGATCTGTTGGTATTACAGCCTTATTATTATTACTTTCATTACCTGTTTTAG
CAGGAGCTATTACTATATTATTAACTGATCGAAATTTAAATACAT----------------------------------
----
>LSTEM370-18|Chilo_sp_AM16|ww04087|
AACTTTATATTTTATTTTTGGAGTTTGAGCAGGAATAATTGGAACATCACTTAGACTTTTAATTCGAGCTGAATTAGGAA
CTCCAGGATCTTTAATTGGTGATGATCAAATCTACAATACTATTGTTACAGCTCACGCATTTATTATAATTTTTTTTATA
GTTATACCAATTATAATTGGAGGATTTGGAAATTGATTAGTACCTCTAATATTAGGAGCTCCTGATATAGCTTTTCCACG
AATAAATAATATAAGATTTTGATTATTACCCCCATCACTAACTTTACTAATTTCTAGAAGAATCGTAGAAACCGGAGCCG
GAACAGGATGAACAGTTTACCCCCCACTATCATCTAATATTGCACATGCTGGAAGTTCAGTAGATTTAGCAATTTTTTCC
CTCCATTTAGCTGGAATTTCTTCTATTTTAGGAGCTATTAACTTTATTACAACAATTATTAATATACGAATTAATGGATT
ATCATTTGATCAAATACCATTACTTGTTTGATCAGTTGGTATTACAGCTTTATTATTATTACTTTCTCTACCTGTTTTAG
CAGGTGCTATTACTATATTATTAACAGATCGAAATCTAAATACATCATTTTTTGATCCAGCTGGAGGG------------
----
>LSTEM371-18|Chilo_orichalcociliellus|ww04089|
AACTTTATATTTTATTTTTGGAATTTGAGCAGGAATAATTGGAACATCACTTAGACTCCTAATTCGAGCTGAATTGGGAA
CCCCTGGATCTTTAATTGGTGATGATCAAATTTATAATACTATTGTTACAGCTCATGCATTTATTATAATTTTTTTTATA
GTTATACCAATTATAATTGGAGGATTTGGAAATTGATTAGTACCTTTAATGTTAGGAGCTCCTGATATAGCCTTCCCACG
AATAAATAATATAAGATTTTGATTACTACCCCCATCACTAACTTTATTAATTTCTAGAAGAATTGTTGAAAATGGAGCTG
GAACTGGGTGAACAGTTTATCCCCCACTTTCATCCAATATTGCCCATGGTGGGAGTTCAGTAGATCTAGCAATTTTTTCT
CTTCATTTAGCTGGAATTTCCTCAATTTTAGGTGCTATTAATTTTATTACAACAATCATTAATATACGAATTAATGGATT
ATCATTTGATCAAATACCTTTATTTGTTTGATCTGTTGGTATTACAGCCTTATTATTATTACTTTCATTACCTGTTTTAG
CAGGAGCTATTACTATATTATTAACTGATCGAAATTTAAATACATC---------------------------------
----
>LSTEM372-18|Chilo_orichalcociliellus|ww04090|
AACTTTATATTTTATTTTTGGAATTTGAGCAGGAATAATTGGAACATCACTTAGACTCCTAATTCGAGCTGAATTAGGAA
CCCCTGGATCTTTAATTGGTGATGATCAAATTTATAATACTATTGTTACAGCTCATGCATTTATTATAATTTTTTTTATA
GTTATACCAATTATAATTGGAGGATTTGGAAATTGATTAGTACCTTTAATGTTAGGAGCTCCTGATATAGCCTTCCCACG
AATAAATAATATAAGATTTTGATTACTACCCCCATCACTAACTTTATTAATTTCTAGAAGAATTGTTGAAAATGGAGCTG

GAACTGGGTGAACAGTTTATCCCCCACTTTCATCCAATATTGCCCATGGTGGGAGTTCAGTAGATCTAGCAATTTTTTCT
CTTCATTTAGCTGGAATTTCCTCAATTTTAGGTGCTATTAATTTTATTACAACAATCATTAATATACGAATTAATGGATT
ATCATTTGATCAAATACCTTTATTTGTTTGATCTGTTGGTATTACAGCCTTATTATTATTACTTTCATTACCTGTTTTAG
CAGGAGCTATTACTATATTATTAACTGATCGAAATTTAAATACATCATTTTTTGATCCTGCTGGTGGAGGAGATCCAATT
CTTTATCAACATTTATTTTGATTTTTT
>LSTEM373-18|Chilo_orichalcociliellus|ww04091|
AACTTTATATTTTATTTTTGGAATTTGAGCAGGAATAATTGGAACATCACTTAGACTCCTAATTCGAGCTGAATTAGGAA
CCCCTGGATCTTTAATTGGTGATGATCAAATTTATAATACTATTGTTACAGCTCATGCATTTATTATAATTTTTTTTATA
GTTATACCAATTATAATTGGAGGATTTGGAAATTGATTAGTACCTTTAATGTTAGGAGCTCCTGATATAGCCTTCCCACG
AATAAATAATATAAGATTTTGATTACTACCCCCATCACTAACTTTATTAATTTCTAGAAGAATTGTTGAAAATGGAGCTG
GAACTGGGTGAACAGTTTATCCCCCACTTTCATCCAATATTGCCCATGGTGGGAGTTCAGTAGATCTAGCAATTTTTTCT
CTTCATTTAGCTGGAATTTCCTCAATTTTAGGTGCTATTAATTTTATTACAACAATCATTAATATACGAATTAATGGATT
ATCATTTGATCAAATACCTTTATTTGTTTGATCTGTTGGTATTACAGCCTTATTATTATTACTTTCATTACCTGTTTTAG
CAGGAGCTATTACTATATTATTAACTGATCGAAATTTAAATACATCATTTTTTGATCCTGCTGGTGGAGGAGATCCAATT
CTTTATCAACATTTATTTTGATTTTTT
>LSTEM374-18|Chilo_orichalcociliellus|ww04092|
AACTTTATATTTTATTTTTGGAATTTGAGCAGGAATAATTGGAACATCACTTAGACTCCTAATTCGAGCTGAATTAGGAA
CCCCTGGATCTTTAATTGGTGATGATCAAATTTATAATACTATTGTTACAGCTCATGCATTTATTATAATTTTTTTTATA
GTTATACCAATTATAATTGGAGGATTTGGAAATTGATTAGTACCTTTAATGTTAGGAGCTCCTGATATAGCCTTCCCACG
AATAAATAATATAAGATTTTGATTACTACCCCCATCACTAACTTTATTAATTTCTAGAAGAATTGTTGAAAATGGAGCTG
GAACTGGGTGAACAGTTTATCCCCCACTTTCATCCAATATTGCCCATGGTGGGAGTTCAGTAGATCTAGCAATTTTTTCT
CTTCATTTAGCTGGAATTTCCTCAATTTTAGGTGCTATTAATTTTATTACAACAATCATTAATATACGAATTAATGGATT
ATCATTTGATCAAATACCTTTATTTGTTTGATCTGTTGGTATTACAGCCTTATTATTATTACTTTCATTACCTGTTTTAG
CAGGAGCTATTACTATATTATTAACTGATCGAAATTTAAATACATCATTTTTTGATCCTGCTGGTGGAGGAGATCCAATT
CTTTATCAACATTTATTTTGATTTTTT
>LSTEM375-18|Chilo_orichalcociliellus|ww04093|
AACTTTATATTTTATTTTTGGAATTTGAGCAGGAATAATTGGAACATCACTTAGACTCCTAATTCGAGCTGAATTAGGAA
CCCCTGGATCTTTAATTGGTGATGATCAAATTTATAATACTATTGTTACAGCTCATGCATTTATTATAATTTTTTTTATA
GTTATACCAATTATAATTGGAGGATTTGGAAATTGATTAGTACCTTTAATGTTAGGAGCTCCTGATATAGCCTTCCCACG
AATAAATAATATAAGATTTTGATTACTACCCCCATCACTAACTTTATTAATTTCTAGAAGAATTGTTGAAAATGGAGCTG
GAACTGGGTGAACAGTTTATCCCCCACTTTCATCCAATATTGCCCATGGTGGGAGTTCAGTAGATCTAGCAATTTTTTCT
CTTCATTTAGCTGGAATTTCCTCAATTTTAGGTGCTATTAATTTTATTACAACAATCATTAATATACGAATTAATGGATT
ATCATTTGATCAAATACCTTTATTTGTTTGATCTGTTGGTATTACAGCCTTATTATTATTACTTTCATTACCTGTTTTAG
CAGGAGCTATTACTATATTATTAACTGATCGAAATTTAAATACATCATTTTTTGATCCTGCTGGTGGAGGAGATCCAATT
CTTTATCAACATTTATTTTGATTTTTT
>LSTEM376-18|Chilo_orichalcociliellus|ww04094|
AACTTTATATTTTATTTTTGGAATTTGAGCAGGAATAATTGGAACATCACTTAGACTCCTAATTCGAGCTGAATTAGGAA
CCCCTGGATCTTTAATTGGTGATGATCAAATTTATAATACTATTGTTACAGCTCATGCATTTATTATAATTTTTTTTATA
GTTATACCAATTATAATTGGAGGATTTGGAAATTGATTAGTACCTTTAATGTTAGGAGCTCCTGATATAGCCTTCCCACG
AATAAATAATATAAGATTTTGATTACTACCCCCATCACTAACTTTATTAATTTCTAGAAGAATTGTTGAAAATGGAGCTG
GAACTGGGTGAACAGTTTATCCCCCACTTTCATCCAATATTGCCCATGGTGGGAGTTCAGTAGATCTAGCAATTTTTTCT
CTTCATTTAGCTGGAATTTCCTCAATTTTAGGTGCTATTAATTTTATTACAACAATCATTAATATACGAATTAATGGATT
ATCATTTGATCAAATACCTTTATTTGTTTGATCTGTTGGTATTACAGCCTTATTATTATTACTTTCATTACCTGTTTTAG
CAGGAGCTATTACTATATTATTAACTGATCGAAATTTAAATACATCATTTTTTGATCCTGCTGGTGGAGGAGATCCAATT
CTTTATCA-------------------
>LSTEM377-18|Chilo_orichalcociliellus|ww04096|
AACTTTATATTTTATTTTTGGAATTTGAGCAGGAATAATTGGAACATCACTTAGACTCCTAATTCGAGCTGAATTAGGAA
CCCCTGGATCTTTAATTGGTGATGATCAAATTTATAATACTATTGTTACAGCTCATGCATTTATTATAATTTTTTTTATA
GTTATACCAATTATAATTGGAGGATTTGGAAATTGATTAGTACCTTTAATGTTAGGAGCTCCTGATATAGCCTTCCCACG
AATAAATAATATAAGATTTTGATTACTACCCCCATCACTAACTTTATTAATTTCTAGAAGAATTGTTGAAAATGGAGCTG
GAACTGGGTGAACAGTTTATCCCCCACTTTCATCCAATATTGCCCATGGTGGGAGTTCAGTAGATCTAGCAATTTTTTCT
CTTCATTTAGCTGGAATTTCCTCAATTTTAGGTGCTATTAATTTTATTACAACAATCATTAATATACGAATTAATGGATT
ATCATTTGATCAAATACCTTTATTTGTTTGATCTGTTGGTATTACAGCCTTATTATTATTACTTTCATTACCTGTTTTAG
CAGGAGCTATTACTATATTATTAACTGATCGAAATTTAAATACATCATTTTTTGATCCTGCTGGTGGAGGAGATCCAATT
CTTTATCAACATTTATTTTGATTTTTT
>LSTEM378-18|Chilo_orichalcociliellus|ww04097|
AACTTTATATTTTATTTTTGGAATTTGAGCAGGAATAATTGGAACATCACTTAGACTCCTAATTCGAGCTGAATTAGGAA

CCCCTGGATCTTTAATTGGTGATGATCAAATTTATAATACTATTGTTACAGCTCATGCATTTATTATAATTTTTTTTATA
GTTATACCAATTATAATTGGAGGATTTGGAAATTGATTAGTACCTTTAATGTTAGGAGCTCCTGATATAGCCTTCCCACG
AATAAATAATATAAGATTTTGATTACTACCCCCATCACTAACTTTATTAATTTCTAGAAGAATTGTTGAAAATGGAGCTG
GAACTGGGTGAACAGTTTATCCCCCACTTTCATCCAATATTGCCCATGGTGGGAGTTCAGTAGATCTAGCAATTTTTTCT
CTTCATTTAGCTGGAATTTCCTCAATTTTAGGTGCTATTAATTTTATTACAACAATCATTAATATACGAATTAATGGATT
ATCATTTGATCAAATACCTTTATTTGTTTGATCTGTTGGTATTACAGCCTTATTATTATTACTTTCATTACCTGTTTTAG
CAGGAGCTATTACTATATTATTAACTGATCGAAATTTAAATACATCATTTTTTGATCCTGCTGGTGGAGGAGATCCAATT
CTTTATCAACATTTATTTTGATTTTTT
>LSTEM379-18|Chilo_orichalcociliellus|ww04098|
AACTTTATATTTTATTTTTGGAATTTGAGCAGGAATAATTGGAACATCACTTAGACTCCTAATTCGAGCTGAATTAGGAA
CCCCTGGATCTTTAATTGGTGATGATCAAATTTATAATACTATTGTTACAGCTCATGCATTTATTATAATTTTTTTTATA
GTTATACCAATTATAATTGGAGGATTTGGAAATTGATTAGTACCTTTAATGTTAGGAGCTCCTGATATAGCCTTCCCACG
AATAAATAATATAAGATTTTGATTACTACCCCCATCACTAACTTTATTAATTTCTAGAAGAATTGTTGAAAATGGAGCTG
GAACTGGGTGAACAGTTTATCCCCCACTTTCATCCAATATTGCCCATGGTGGGAGTTCAGTAGATCTAGCAATTTTTTCT
CTTCATTTAGCTGGAATTTCCTCAATTTTAGGTGCTATTAATTTTATTACAACAATCATTAATATACGAATTAATGGATT
ATCATTTGATCAAATACCTTTATTTGTTTGATCTGTTGGTATTACAGCCTTATTATTATTACTTTCATTACCTGTTTTAG
CAGGAGCTATTACTATATTATTAACTGATCGAAATTTAAATACATCATTTTTTGATCCTGCTGGTGGAGGAGATCCAATT
CTTTATCAACATTTATTTTGATTTTTT
>LSTEM380-18|Chilo_orichalcociliellus|ww04099|
AACTTTATATTTTATTTTTGGAATTTGAGCAGGAATAATTGGAACATCACTTAGACTCCTAATTCGAGCTGAATTAGGAA
CCCCTGGATCTTTAATTGGTGATGATCAAATTTATAATACTATTGTTACAGCTCATGCATTTATTATAATTTTTTTTATA
GTTATACCAATTATAATTGGAGGATTTGGAAATTGATTAGTACCTTTAATGTTAGGAGCTCCTGATATAGCCTTCCCACG
AATAAATAATATAAGATTTTGATTACTACCCCCATCACTAACTTTATTAATTTCTAGAAGAATTGTTGAAAATGGAGCTG
GAACTGGGTGAACAGTTTATCCCCCACTTTCATCCAATATTGCCCATGGTGGGAGTTCAGTAGATCTAGCAATTTTTTCT
CTTCATTTAGCTGGAATTTCCTCAATTTTAGGTGCTATTAATTTTATTACAACAATCATTAATATACGAATTAATGGATT
ATCATTTGATCAAATACCTTTATTTGTTTGATCTGTTGGTATTACAGCCTTATTATTATTACTTTCATTACCTGTTTTAG
CAGGAGCTATTACTATATTATTAACTGATCGAAATTTAAATACATCATTTTTTGATCCTGCTGGTGGAGGAGATCCAATT
CTTTATCAACATTTATTTTGATTTTTT
>LSTEM381-18|Chilo_orichalcociliellus|ww04100|
AACTTTATATTTTATTTTTGGAATTTGAGCAGGAATAATTGGAACATCACTTAGACTCCTAATTCGAGCTGAATTAGGAA
CCCCTGGATCTTTAATTGGTGATGATCAAATTTATAATACTATTGTTACAGCTCATGCATTTATTATAATTTTTTTTATA
GTTATACCAATTATAATTGGAGGATTTGGAAATTGATTAGTACCTTTAATGTTAGGAGCTCCTGATATAGCCTTCCCACG
AATAAATAATATAAGATTTTGATTACTACCCCCATCACTAACTTTATTAATTTCTAGAAGAATTGTTGAAAATGGAGCTG
GAACTGGGTGAACAGTTTATCCCCCACTTTCATCCAATATTGCCCATGGTGGGAGTTCAGTAGATCTAGCAATTTTTTCT
CTTCATTTAGCTGGAATTTCCTCAATTTTAGGTGCTATTAATTTTATTACAACAATCATTAATATACGAATTAATGGATT
ATCATTTGATCAAATACCTTTATTTGTTTGATCTGTTGGTATTACAGCCTTATTATTATTACTTTCATTACCTGTTTTAG
CAGGAGCTATTACTATATTATTAACTGATCGAAATTTAAATACATCATTTTTTGATCCTGCTGGTGGAGGAGATCCAATT
CTTTATCAACATTTATTTTGATTTTTT
>LSTEM382-18|Chilo_sp_AM16|ww04101|
AACTTTATACTTTATTTTTGGAGTTTGAGCAGGAATAATTGGAACATCACTTAGACTTTTAATTCGAGCTGAATTAGGAA
CTCCAGGATCTTTAATTGGTGATGATCAAATCTACAATACTATTGTTACAGCTCACGCATTTATTATAATTTTTTTTATA
GTTATACCAATTATAATTGGAGGATTTGGAAATTGATTAGTACCTCTAATATTAGGAGCTCCTGATATAGCTTTTCCACG
AATAAATAATATAAGATTTTGATTATTACCCCCATCACTAACTTTACTAATTTCTAGAAGAATCGTAGAAACCGGAGCCG
GAACAGGATGAACAGTTTACCCCCCACTATCATCTAATATTGCACATGCTGGAAGTTCAGTAGATTTAGCAATTTTTTCC
CTCCATTTAGCTGGAATTTCTTCTATTTTAGGAGCTATTAACTTTATTACAACAATTATTAATATACGAATTAATGGATT
ATCATTTGATCAAATACCATTACTTGTTTGATCAGTTGGTATTACAGCTTTATTATTATTACTTTCTCTACCTGTTTTAG
CAGGTGCTATTACTATATTATTAACAGATCGAAATCTAAATACATCATTTTTTGATCCAGCTGGAGGGGGTGATCCAATT
CTTTATCAACACTTATTTTGATTTTTT
>LSTEM383-18|Chilo_sp_AM16|ww04103|
AACTTTATACTTTATTTTTGGAGTTTGAGCAGGAATAATTGGAACATCACTTAGACTTTTAATTCGAGCTGAATTAGGAA
CTCCAGGATCTTTAATTGGTGATGATCAAATCTACAATACTATTGTTACAGCTCACGCATTTATTATAATTTTTTTTATA
GTTATACCAATTATAATTGGAGGATTTGGAAATTGATTAGTACCTCTAATATTAGGAGCTCCTGATATAGCTTTTCCACG
AATAAATAATATAAGATTTTGATTATTACCCCCATCACTAACTTTACTAATTTCTAGAAGAATCGTAGAAACCGGAGCCG
GAACAGGATGAACAGTTTACCCCCCACTATCATCTAATATTGCACATGCTGGAAGTTCAGTAGATTTAGCAATTTTTTCC
CTCCATTTAGCTGGAATTTCTTCTATTTTAGGAGCTATTAACTTTATTACAACAATTATTAATATACGAATTAATGGATT
ATCATTTGATCAAATACCATTACTTGTTTGATCAGTTGGTATTACAGCTTTATTATTATTACTTTCTCTACCTGTTTTAG
CAGGTGCTATTACTATATTATTAACAGATCGAAATCTAAATACATCATTTTTTGATCCAGCTGGAGGGGGTGATCCAATT

CTTTATCAACACTTATTTTGATTTTTT
>LSTEM384-18|Chilo_sp_AM16|ww04104|
AACTTTATACTTTATTTTTGGAGTTTGAGCAGGAATAATTGGAACATCACTTAGACTTTTAATTCGAGCTGAATTAGGAA
CTCCAGGATCTTTAATTGGTGATGATCAAATCTACAATACTATTGTTACAGCTCACGCATTTATTATAATTTTTTTATA
GTTATACCAATTATAATTGGAGGATTTGGAAATTGATTAGTACCTCTAATATTAGGAGCTCCTGATATAGCTTTTCCACG
AATAAATAATATAAGATTTTGATTATTACCCCCATCACTAACTTTACTAATTTCTAGAAGAATCGTAGAAACCGGAGCCG
GAACAGGATGAACAGTTTACCCCCCACTATCATCTAATATTGCACATGCTGGAAGTTCAGTAGATTTAGCAATTTTTTCC
CTCCATTTAGCTGGAATTTCTTCTATTTTAGGAGCTATTAACTTTATTACAACAATTATTAATATACGAATTAATGGATT
ATCATTTGATCAAATACCATTACTTGTTTGATCAGTTGGTATTACAGCTTTATTATTATTACTTTCTCTACCTGTTTTAG
CAGGTGCTATTACTATATTATTAACAGATCGAAATCTAAATACATCATTTTTTGATCCAGCTGGAGGGGGTGATCCAATT
CTTTATCAACACTTATTTTGATTTTTT
>LSTEM385-18|Chilo_orichalcociliellus|ww04111|
AACTTTATATTTTATTTTTGGAATTTGAGCAGGAATAATTGGAACATCACTTAGACTCCTAATTCGAGCTGAATTAGGAA
CCCCTGGATCTTTAATTGGTGATGATCAAATTTATAATACTATTGTTACAGCTCATGCATTTATTATAATTTTTTTATA
GTTATACCAATTATAATTGGAGGATTTGGAAATTGATTAGTACCTTTAATGTTAGGAGCTCCTGATATAGCCTTCCCACG
AATAAATAATATAAGATTTTGATTACTACCCCCATCACTAACTTTATTAATTTCTAGAAGAATTGTTGAAAATGGAGCTG
GAACTGGGTGAACAGTTTATCCCCCACTTTCATCCAATATTGCCCATGGTGGGAGTTCAGTAGATCTAGCAATTTTTTCT
CTTCATTTAGCTGGAATTTCCTCAATTTTAGGTGCTATTAATTTTATTACAACAATCATTAATATACGAATTAATGGATT
ATCATTTGATCAAATACCTTTATTTGTTTGATCTGTTGGTATTACAGCCTTATTATTATTACTTTCATTACCTGTTTTAG
CAGGAGCTATTACTATATTATTAACTGATCGAAATTTAAATACATCATTTTTTGATCCTGCTGGTGGAGGAGATCCAATT
CTTTATCAACATT--------------
>LSTEM386-18|Chilo_orichalcociliellus|ww04113|
AACTTTATATTTTATTTTTGGAATTTGAGCAGGAATAATTGGAACATCACTTAGACTCCTAATTCGAGCTGAATTAGGAA
CCCCTGGATCTTTAATTGGTGATGATCAAATTTATAATACTATTGTTACAGCTCATGCATTTATTATAATTTTTTTATA
GTTATACCAATTATAATTGGAGGATTTGGAAATTGATTAGTACCTTTAATGTTAGGAGCTCCTGATATAGCCTTCCCACG
AATAAATAATATAAGATTTTGATTACTACCCCCATCACTAACTTTATTAATTTCTAGAAGAATTGTTGAAAATGGAGCTG
GAACTGGGTGAACAGTTTATCCCCCACTTTCATCCAATATTGCCCATGGTGGGAGTTCAGTAGATCTAGCAATTTTTTCT
CTTCATTTAGCTGGAATTTCCTCAATTTTAGGTGCTATTAATTTTATTACAACAATCATTAATATACGAATTAATGGGTT
ATCATTTGATCAAATACCTTTATTTGTTTGATCTGTTGGTATTACAGCCTTATTATTATTACTTTCATTACCTGTTTTAG
CAGGAGCTATTACTATATTATTAACTGATCGAAATTTAAATACATCATTTTTTGATCCTGCTGGTGGAGGAGATCCAATT
CTTTATCAACATTTATTTTGATTTTTT
>LSTEM387-18|Chilo_orichalcociliellus|ww04114|
--AATTAGGAA
CCCCTGGATCTTTAATTGGWGATGATCAAATTTATAATACTATTGTTACAGCTCATGCATTTATTATAATTTTTTTATA
GTTATACCAATTATAATTGGAGGATTTGGAAATTGATTAGTACCTTTAATGTTAGGAGCTCCTGATATAGCCTTCCCACG
AATAAATAATATAAGATTTTGATTACTACCCCCATCACTAACTTTATTAATTTCTAGAAGAATTGTTGAAAATGGAGCTG
GAACTGGGTGAACAGTTTATCCCCCACTTTCATCCAATATTGCCCATGGTGGGAGTTCAGTAGATCTAGCAATTTTTTCT
CTTCATTTAGCTGGAATTTCCTCAATTTTAGGTGCTATTAATTTTATTACAACAATCATTAATATACGAATTAATGGATT
ATCATTTGATCAAATACCTTTATTTGTTTGATCTGTTGGTATTACAGCCTTATTATTATTACTTTCATTACCTGTTTTAG
CAGGAGCTATTACTATATTATTAACTGATCGAAATTTAAATACATCATTTTTTGATCCTGCTGGGGGAGGAGATCCAATT
CTTTATCAACATTTATTTTGATTTTTT
>LSTEM388-18|Chilo_orichalcociliellus|ww04115|
AACTTTATATTTTATTTTTGGAATTTGAGCAGGAATAATTGGAACATCACTTAGACTCCTAATTCGAGCTGAATTAGGAA
CCCCTGGATCTTTAATTGGTGATGATCAAATTTATAATACTATTGTTACAGCTCATGCATTTATTATAATTTTTTTATA
GTTATACCAATTATAATTGGAGGATTTGGAAATTGATTAGTACCTTTAATGTTAGGAGCTCCTGATATAGCCTTCCCACG
AATAAATAATATAAGATTTTGATTACTACCCCCATCACTAACTTTATTAATTTCTAGAAGAATTGTTGAAAATGGAGCTG
GAACTGGGTGAACAGTTTATCCCCCACTTTCATCCAATATTGCCCATGGTGGGAGTTCAGTAGATCTAGCAATTTTTTCT
CTTCATTTAGCTGGAATTTCCTCAATTTTAGGTGCTATTAATTTTATTACAACAATCATTAATATACGAATTAATGGATT
ATCATTTGATCAAATACCTTTATTTGTTTGATCTGTTGGTATTACAGCCTTATTATTATTACTTTCATTACCTGTTTTAG
CAGGAGCTATTACTATATTATTAACTGATCGAAATTTAAATACATCATTTTTTGATCCTGCTGGTGGAGGAGATCCAATT
CTTTATCAACATTTATTTTGATTTTTT
>LSTEM389-18|Chilo_orichalcociliellus|ww04116|
AACTTTATATTTTATTTTTGGAATTTGAGCAGGAATAATTGGAACATCACTTAGACTCCTAATTCGAGCTGAATTAGGAA
CCCCTGGATCTTTAATTGGTGATGATCAAATTTATAATACTATTGTTACAGCTCATGCATTTATTATAATTTTTTTATA
GTTATACCAATTATAATTGGAGGATTTGGAAATTGATTAGTACCTTTAATGTTAGGAGCTCCTGATATAGCCTTCCCACG
AATAAATAATATAAGATTTTGATTACTACCCCCATCACTAACTTTATTAATTTCTAGAAGAATTGTTGAAAATGGAGCTG
GAACTGGATGAACAGTTTATCCCCCACTTTCATCCAATATTGCCCATGGTGGGAGTTCAGTAGATCTAGCAATTTTTTCT

CTTCATTTAGCTGGAATTTCCTCAATTTTAGGTGCTATTAATTTTATTACAACAATCATTAATATACGAATTAATGGATT
ATCATTTGATCAAATACCTTTATTTGTTTGATCTGTTGGTATTACAGCCTTATTATTATTACTTTCATTACCTGTTTTAG
CAGGAGCTATTACTATATTATTAACTGATCGAAATTTAAATACATCATTTTTTGATCCTGCTGGTGGAGGAGATCCAATT
CTTTATCAACATTTATTTTGATTTTTT
>LSTEM390-18|Chilo_orichalcociliellus|ww04117|
AACTTTATATTTTATTTTTGGAATTTGAGCAGGAATAATTGGAACATCACTTAGACTCCTAATTCGAGCTGAATTAGGAA
CCCCTGGATCTTTAATTGGTGATGATCAAATTTATAATACTATTGTTACAGCTCATGCATTTATTATAATTTTTTTTATA
GTTATACCAATTATAATTGGAGGATTTGGAAATTGATTAGTACCTTTAATGTTAGGAGCTCCTGATATAGCCTTCCCACG
AATAAATAATATAAGATTTTGATTACTACCCCCATCACTAACTTTATTAATTTCTAGAAGAATTGTTGAAAATGGAGCTG
GAACTGGATGAACAGTTTATCCCCCACTTTCATCCAATATTGCCCATGGTGGGAGTTCAGTAGATCTAGCAATTTTTTCT
CTTCATTTAGCTGGAATTTCCTCAATTTTAGGTGCTATTAATTTTATTACAACAATCATTAATATACGAATTAATGGATT
ATCATTTGATCAAATACCTTTATTTGTTTGATCTGTTGGTATTACAGCCTTATTATTATTACTTTCATTACCTGTTTTAG
CAGGAGCTATTACTATATTATTAACTGATCGAAATTTAAATACATCATTTTTTGATCCTGCTGGTGGAGGAGATCCAATT
CTTTATCAACATTTATTTTGATTTTTT
>LSTEM391-18|Chilo_orichalcociliellus|ww04118|
AACTTTATATTTTATTTTTGGAATTTGAGCAGGAATAATTGGAACATCACTTAGACTCCTAATTCGAGCTGAATTAGGAA
CCCCTGGATCTTTAATTGGKGATGATCAAATTTATAATACTATTGTTACAGCTCATGCATTTATTATAATTTTTTTTATA
GTTATACCAATTATAATTGGAGGATTTGGAAATTGATTAGTACCTTTAATGTTAGGAGCTCCTGATATAGCCTTCCCACG
AATAAATAATATAAGATTTTGATTACTACCCCCATCACTAACTTTATTAATTTCTAGAAGAATTGTTGAAAATGGAGCTG
GAACTGGGTGAACAGTTTATCCCCCACTTTCATCCAATATTGCCCATGGTGGGAGTTCAGTAGATCTAGCAATTTTTTCT
CTTCATTTAGCTGGAATTTCCTCAATTTTAGGTGCTATTAATTTTATTACAACAATCATTAATATACGAATTAATGGATT
ATCATTTGATCAAATACCTTTATTTGTTTGATCTGTTGGTATTACAGCCTTATTATTATTACTTTCATTACCTGTTTTAG
CAGGAGCTATTACTATATTATTAACTGATCGAAATTTAAATACATCATTTTTTGATCCTGCTGGTGGAGGAGATCCAATT
CTTTATCAACATTTATTTTGATTTTTT
>LSTEM392-18|Chilo_orichalcociliellus|ww04119|
AACTTTATATTTTATTTTTGGAATTTGAGCAGGAATAATTGGAACATCACTTAGACTCCTAATTCGAGCTGAATTAGGAA
CCCCTGGATCTTTAATTGGTGATGATCAAATTTATAATACTATTGTTACAGCTCATGCATTTATTATAATTTTTTTTATA
GTTATACCAATTATAATTGGAGGATTTGGAAATTGATTAGTACCTTTAATGTTAGGAGCTCCTGATATAGCCTTCCCACG
AATAAATAATATAAGATTTTGATTACTACCCCCATCACTAACTTTATTAATTTCTAGAAGAATTGTTGAAAATGGAGCTG
GAACTGGGTGAACAGTTTATCCCCCACTTTCATCCAATATTGCCCATGGTGGGAGTTCAGTAGATCTAGCAATTTTTTCT
CTTCATTTAGCTGGAATTTCCTCAATTTTAGGTGCTATTAATTTTATTACAACAATCATTAATATACGAATTAATGGATT
ATCATTTGATCAAATACCTTTATTTGTTTGATCTGTTGGTATTACAGCCTTATTATTATTACTTTCATTACCTGTTTTAG
CAGGAGCTATTACTATATTATTAACTGATCGAAATTTAAATACATCATTTTTTGATCCTGCTGGTGGAGGAGATCCAATT
CTTTATCAACATTTATTTTGATTTTTT
>LSTEM393-18|Bathytricha_sp.|ww05532|
----TTATATTTTATTTTTGGAATTTGAGCAGGAATAGTGGGAACCTCTTTAAGACTACTAATTCGCGCTGAATTAGGAA
CTCCCGGATCTCTAATTGGAGATGATCAAATTTATAATACTATTGTAACAGCTCATGCTTTTATTATAATTTTTTTCATA
GTTATACCAATCATAATTGGAGGATTTGGAAATTGACTTGTACCTTTAATATTAGGAGCACCTGATATAGCATTCCCACG
AATAAATAACATAAGTTTTTGATTACTTCCACCTTCTTTAACTCTTCTCATTTCAAGTAGAATTGTAGAAAATGGAGCAG
GAACTGGATGAACAGTTTACCCACCACTCTCATCTAATATTGCTCATAGTGGAAGATCCGTAGACTTAGCTATTTTTTCC
CTCCATTTAGCAGGAATCTCTTCCATCCTAGGAGCTATTAATTTTATTACAACTATTATTAATATACGATTAAATAGCTT
ATCTTTTGATCAAATACCTTTATTTATTTGAGCTGTAGGAATTACAGCATTTTTATTATTATTATCATTACCTGTATTAG
CTGGAGCTATTACTATATTACTAACAGATCGAAATTTAAATACATCATTTTTCGATCCCGCAGGAGGAGGGGATCCAATT
TTATATCAACATTTATT----------
>LSTEM394-18|Chilo_orichalcociliellus|ww05561|
--AGACTCCTAATTCGAGCTGAATTAGGCA
CCCCTGGATCTTTAATTGGTGATGATCAAATTTATAATACTATTGTTACAGCTCATGCATTTATTATAATTTTTTTTATA
GTTATACCAATTATAATTGGAGGATTTGGAAATTGATTAGTACCTTTAATGTTAGGAGCTCCTGATATAGCCTTCCCACG
AATAAATAATATAAGATTTTGATTACTACCCCCATCACTAACTTTATTAATTTCTAGAAGAATTGTTGAAAATGGAGCTG
GAACTGGGTGAACAGTTTACCCCCCACTTTCATCCAATATTGCCCATGGTGGAAGTTCAGTAGATCTAGCAATTTTTTCT
CTTCATTTAGCTGGAATTTCCTCAATTTTAGGTGCTATTAATTTTATTACAACAATCATTAATATACGAATTAATGGATT
ATCATTTGATCAAATACCTTTATTTGTTTGATCTGTTGGTATTACAGCCTTATTATTACTACTTTCATTACCTGTTTTAG
CAGGAGCTATTACTATATTATTAACTGATCGAAATTTAAATACATCATTTTTTGTT-------------------------
----
>LSTEM395-18|Chilo_orichalcociliellus|ww05562|
--AACATCACTTAGACTCCTAATTCGAGCTGAATTGGGAA
CCCCTGGGTCTTTAATTGGTGATGATCAAATTTATAATACTATTGTTACAGCTCATGCATTTATTATAATTTTTTTTATA

GTTATACCAATTATAATTGGAGGATTTGGAAATTGATTAGTACCTTTAATGTTAGGAGCTCCTGATATAGCCTTCCCACG
AATAAATAATATAAGATTTTGATTACTACCCCCATCACTAACTTTATTAATTTCTAGAAGAATTGTTGAAAATGGAGCTG
GAACTGGATGAACAGTTTATCCCCCACTTTCATCCAATATTGCCCATGGTGGAAGTTCAGTAGATCTAGCAATTTTTTCT
CTTCATTTAGCTGGAATTTCCTCAATTTTAGGTGCTATTAATTTTATTACAACAATCATTAATATACGAATTAATGGATT
ATCATTTGATCAAATACCTTTATTTGTTTGATCTGTTGGTATTACAGCCTTATTATTATTACTTTCATTACCTGTTTTAG
CAGGAGCTATTACTATATTATTAACTGATCGAAATTTAAATACATCATTTTTTGATCCTGCTGGTGGAGGAGATCCAAT-
----
>LSTEM396-18|Chilo_quirimbellus|ww05563|
----TTATATTTTATTTTTGGAATTTGAGCAGGAATAATTGGAACATCACTTAGACTTTTAATTCGAGCTGAATTAGGAA
CTCCAGGATCTTTAATTGGTGATGATCAAATTTATAATACTATTGTTACAGCTCATGCATTTATTATAATTTTTTTTATA
GTTATACCAATTATAATTGGTGGATTTGGAAATTGATTAGTACCTTTAATATTGGGAGCTCCCGATATAGCTTTTCCACG
AATAAATAATATAAGATTTTGATTACTTCCCCCATCATTAACTTTATTAATTTCTAGTAGAATCGTAGAAAATGGAGCTG
GAACAGGATGAACAGTTTACCCCCCACTTTCATCTAACATTGCACATGCTGGAAGTTCAGTAGATTTAGCAATTTTTTCT
CTCCATTTAGCTGGAATTTCTTCCATCTTAGGTGCAATTAACTTTATTACAACAATCATTAATATACGAATTAATGGATT
ATCATTTGATCAAATACCATTATTTGTTTGATCTGTTGGTATCACAGCTTTATTATTACTACTTTCATTGCCAGTTTTAG
CTGGTGCTATTACCATATTATTAACAGATCGAAATTTAAATACATCATTTTTTGATCCTGCTGGTGGTGGTGACCCAAT-
----
>LSTEM397-18|Chilo_orichalcociliellus|ww05565|
--AACATCACTTAGACTCCTAATTCGAGCTGAATTAGGAA
CCCCTGGATCTTTAATTGGTGATGATCAAATTTATAATACTATTGTTACAGCTCATGCATTTATTATAATTTTTTTTATA
GTTATACCAATTATAATTGGAGGATTTGGAAATTGATTAGTACCTTTAATGTTAGGAGCTCCTGATATAGCCTTCCCACG
AATAAATAATATAAGATTTTGATTACTACCCCCATCACTAACTTTATTAATTTCTAGAAGAATTGTTGAAAATGGAGCTG
GAACTGGGTGAACAGTTTACCCCCCACTTTCATCCAATATTGCCCATGGTGGAAGTTCAGTAGATCTAGCAATTTTTTCT
CTTCATTTAGCTGGAATTTCCTCAATTTTAGGTGCTATTAATTTTATTACAACAATCATTAATATACGAATTAATGGATT
ATCATTTGATCAAATACCTTTATTTGTTTGATCTGTTGGTATTACAGCCTTATTATTACTACTTTCATTACCTGTTTTAG
CAGGAGCTATTACTATATTATTAACTGATCGAAATTTAAATACATCATTTTTTGATCCTGCTGGTGGT------------
----
>LSTEM398-18|Chilo_orichalcociliellus|ww05566|
--AACATCACTTAGACTCCTAATTCGAGCTGAATTAGGAA
CCCCTGGATCTTTAATTGGTGATGATCAAATTTATAATACTATTGTTACAGCTCATGCATTTATTATAATTTTTTTTATA
GTTATACCAATTATAATTGGAGGATTTGGAAATTGATTAGTACCTTTAATGTTAGGAGCTCCTGATATAGCCTTCCCACG
AATAAATAATATAAGATTTTGATTACTACCCCCATCACTAACTTTATTAATTTCTAGAAGAATTGTTGAAAATGGAGCTG
GAACTGGGTGAACAGTTTATCCCCCACTTTCATCCAATATTGCCCATAGTGGAAGTTCAGTAGATCTAGCAATTTTTTCT
CTTCATTTAGCTGGAATTTCCTCAATTTTAGGTGCTATTAATTTTATTACAACAATCATTAATATACGAATTAATGGATT
ATCATTTGATCAAATACCTTTATTTGTTTGATCTGTTGGTATTACAGCCTTATTATTATTACTTTCATTACCTGTTTTA-
----

>LSTEM399-18|Chilo_orichalcociliellus|ww05567|
--AACATCACTTAGACTCCTAATTCGAGCTGAATTAGGAA
CCCCTGGATCTTTAATTGGTGATGATCAAATTTATAATACTATTGTTACAGCTCATGCATTTATTATAATTTTTTTTATA
GTTATACCAATTATAATTGGAGGATTTGGAAATTGATTAGTACCTTTAATGTTAGGAGCTCCTGATATAGCCTTCCCACG
AATAAATAATATAAGATTTTGATTACTACCCCCATCACTAACTTTATTAATTTCTAGAAGAATTGTTGAAAATGGAGCTG
GAACTGGGTGAACAGTTTACCCCCCACTTTCATCCAATATTGCCCATGGTGGAAGTTCAGTAGATCTAGCAATTTTTTCT
CTTCATTTAGCTGGAATTTCCTCAATTTTAGGTGCTATTAATTTTATTACAACAATCATTAATATACGAATTAATGGATT
ATCATTTGATCAAATACCTTTATTTGTTTGATCTGTTGGTATTACAGCCTTATTATTACTACTTTCATTACCTGTTTTA-
----

>LSTEM400-18|Chilo_thyrsis|ww05568|
--AACATCACTTAGACTTTTAATTCGAGCTGAATTAGGGA
CTCCAGGATCTTTAATTGGTGATGATCAAATTTATAATACTATTGTTACAGCTCATGCATTTATTATAATTTTTTTTATA
GTTATACCAATTATAATTGGTGGATTTGGAAATTGATTAGTGCCTTTAATATTGGGAGCTCCTGATATAGCTTTTCCACG
AATAAATAATATAAGATTTTGATTACTCCCCCCGTCATTAACTTTATTAATTTCTAGWAGAATTGTTGAAAATGGAGCTG
GAACTGGGTGAACAGTTTACCCCCCACTTTCATCCAATATTGCCCATGGTGGAAGTTCAGTAGATCTAGCAATTTTTTCT
CTTCATTTAGCTGGAATTTCCTCAATTTTAGGTGCTATTAATTTTATTACAACAATCATTAATATATGAATTAATGGATT
ATCATTTGATCAAATACCTTTATTTGTTTGATCTGTTGGTATTACAGCCTTATTATTACTACTTTCATTACCTGTTTTA-
----

>LSTEM401-18|Chilo_sp_AM15|ww05569|
--ACATCACTTAGACTTTTAATTCGAGCTGAATTAGGGA
CTCCAGGATCTTTAATTGGTGATGATCAAATTTATAATACTATTGTTACAGCTCATGCATTTATTATAATTTTTTTATA
GTTATACCAATTATAATTGGTGGATTTGGAAATTGATTAGTGCCTTTAATATTGGGAGCTCCTGATATAGCTTTTCCACG
AATAAATAATATAAGATTTTGATTACTCCCCCCGTCATTAACTTTATTAATTTCTAGTAGAATTGTAGAAAATGGAGCTG
GAACAGGATGAACAGTTTACCCCCCACTCTCATCTAATATTGCCCATGCTGGAAGCTCAGTAGATTTGGCAATTTTTTCC
CTTCACTTAGCGGGTATTTCTTCTATCTTAGGTGCAATTAACTTTATTACAACAATCATTAATATACGAATTAATGGGTT
ATCATTTGATCAAATACCATTATTTGTTTGATCTGTTGGTATTACAGCTTTATTATTATTACTTTCATTACCAGTTTTAG
CAGGTGCTATTACCATGTTATTAACAGATCGAAATTTAAATACATCATTTTTTGATCCTGCTGGTGGGGGTGACCCCATT
CTATAC--------------------
>LSTEM402-18|Chilo_orichalcociliellus|ww05570|
---AGACTCTTAATTCGAGCGGAATTAGGAM
CNCCTGGATCTTTAATTGGTGATGATCAAATTTATAATACTATTGTTACAGCTCATGCATTTATTATAATTTTTTTATA
GTTATACCAATTATAATTGGAGGATTTGGAAATTGATTAGTACCTTTAATGTTAGGAGCTCCTGATATAGCCTTCCCACG
AATAAATAATATAAGATTTTGATTACTACCCCCATCACTAACTTTATTAATTTCTAGAAGAATTGTTGAAAATGGAGCTG
GAACTGGGTGAACAGTTTACCCCCCACTTTCATCCAATATTGCCCATGGTGGAAGTTCAGTAGATCTAGCAATTTTTTCT
CTTCATTTAGCTGGAATTTCCTCAATTTTAGGTGCTATTAATTTTATTACAACAATCATTAATATACGAATTAATGGATT
ATCATTTGATCAAATACCTTTATTTGTTTGATCTGTTGGTATTACAGCCTTATTATTACTACTTTCATTACC--------
----

>LSTEM403-18|Chilo_quirimbellus|ww05571|
---AACATCACTTAGACTTTTAATTCGAGCTGAATTAGGAA
CTCCAGGATCTTTAATTGGTGATGATCAAATTTATAATACTATTGTTACAGCTCATGCATTTATTATAATTTTTTTATA
GTTATACCAATTATAATTGGTGGATTTGGAAATTGATTAGTACCTTTAATATTGGGAGCTCCCGATATAGCTTTTCCACG
AATAAATAATATAAGATTTTGATTACTTCCCCCATCATTAACTTTATTAATTTCTAGTAGAATCGTAGAAAATGGAGCTG
GAACAGGATGAACAGTTTACCCCCCACTCTCATCCAACATTGCACATGCWGGAAGTTCAGTAGATTTAGCAATTTTTTCT
CTCCATTTAGCTGGAATTTCTTCCATCTTAGGTGCAATTAACTTTATTACAACAATCATTAATATGCGAATTAATGGATT
ATCATTTGATCAAATACCATTATTTGTTTGATCTGTTGGTATCACAGCTTTATTATTACTACTTTCATTACCAGTTTTA-
----

>LSTEM404-18|Chilo_quirimbellus|ww05572|
--ACATCACTTAGACTTTTAATTCGAGCTGAATTAGGAA
CTCCAGGATCTTTAATTGGTGATGATCAAATTTATAATACTATTGTTACAGCTCATGCATTTATTATAATTTTTTTATA
GTTATACCAATTATAATTGGTGGATTTGGAAATTGATTAGTACCTTTAATATTGGGAGCTCCCGATATAGCTTTTCCACG
AATAAATAATATAAGATTTTGATTACTTCCCCCATCATTAACTTTATTAATTTCTAGTAGAATCGTAGAAAATGGAGCTG
GAACAGGATGAACAGTTTACCCCCCACTCTCATCCAACATTGCACATGCTGGAAGTTCAGTAGATTTAGCAATTTTTTCT
CTCCATTTAGCTGGAATTTCTTCCATCTTAGGTGCAATTAACTTTATTACAACAATCATTAATATGCGAATTAATGGATT
ATCATTTGATCAAATACCATTATTTGTTTGATCTGTTGGTATCACAGCTTTATTATTACTACTTTCATTGCCAGTTTTAG
CTGGTGCTATTACCATATTATTAACAGATCGAAATTTAAATACATCATTTTTTGATCCTGCTGGTG--------------
----
>LSTEM405-18|Chilo_sp_AM15|ww05573|
---AGACTTTTAATTCGAGCTGAATTAGGGA
CTCCAGGATCTTTAATTGGTGATGATCAAATTTATAATACTATTGTTACAGCTCATGCATTTATTATAATTTTTTTATA
GTTATACCAATTATAATTGGTGGATTTGGAAATTGATTAGTGCCTTTAATATTGGGAGCTCCTGATATAGCTTTTCCACG
AATAAATAATATAAGATTTTGATTACTCCCCCCGTCATTAACTTTATTAATTTCTAGTAGAATTGTAGAAAATGGAGCTG
GAACAGGATGAACAGTTTACCCCCCACTCTCATCTAATATTGCCCATGCTGGAAGTTCAGTAGATTTGGCAATTTTTTCC
CTTCACTTAGCGGGTATTTCTTCTATCTTAGGTGCAATTAACTTTATTACAACAATCATTAATATACGAATTAATGGATT
ATCATTTGATCAAATACCATTATTTGTTTGATCTGTTGGTATTACAGCTTTATTGTTATTACTTTCATTGCCAGTTTTAG
CAGGTGCTATTACTATGTTATTAACAGATCGAAATTTAAATACATCATTTTTTGATCCT--------------------
----
>LSTEM406-18|Chilo_thyrsis|ww05575|
---AACATCACTTAGACTTTTAATTCGAGCTGAATTAGGGA
CTCCAGGATCTTTAATTGGTGATGATCAAATTTATAATACTATTGTTACAGCTCATGCATTTATTATAATTTTTTTATA
GTTATACCAATTATAATTGGWGGATTTGGAAATTGATTAGTGCCTTTAATATTGGGAGCTCCTGATATAGCTTTYCCACG
AATAAATAATATAAGATTTTGATTACTMCCCCCRTCAYTAACTTTATTAATTTCTAGAAGAATTGTTGAAAATGGAGCTG
GAACTGGGTGAACAGTTTACCCCCCACTTTCATCCAATATTGCCCATGGTGGAAGTTCAGTAGATCTAGCAATTTTTTCT
CTTCATTTAGCTGGAATTTCCTCAATTTTAGGTGCTATTAATTTTATTACAACAATCATTAATATACGAATTAATGGATT

ATCATTTGATCAAATACCTTTATTTGTTTGATCTGTTGGTATTACAGCCTTATTATTACTACTTTCATTACCTGTTTTA-
----

>LSTEM407-18|Chilo_sp_AM15|ww05577|
----------------------------------TAATTGGAACATCACTTAGACTTTTAATTCGAGCTGAATTAGGGA
CTCCAGGATCTTTAATTGGTGATGATCAAATTTATAATACTATTGTTACAGCTCATGCATTTATTATAATTTTTTTATA
GTTATACCAATTATAATTGGTGGATTTGGAAATTGATTAGTGCCTTTAATATTGGGAGCTCCTGATATARCTTTTCCACG
AATAAATAATATAAGATTTTGATTACTCCCCCCGTCATTAACTTTATTAATTTCTAGTAGAATTGTAGAAAATGGAGCTG
GAACAGGATGAACAGTTTACCCCCCACTCTCATCTAATATTGCCCATGCTGGAAGCTCAGTAGATTTGGCAATTTTTTCC
CTTCACTTAGCGGGTATTTCTTCTATCTTAGGTGCAATTAACTTTATTACAACAATCATTAATATACGAATTAATGGGTT
ATCATTTGATCAAATACCATTATTTGTTTGATCTGTTGGTATTACAGCTTTATTATTATTACTTTCATTACCAGTTTTAG
CAGGTGCTATTACCATGTTATTAACAGATCGAAATTTAAATACATCATTTTTTGATCCTGCTGGTGGG------------
----
>LSTEM408-18|Chilo_orichalcociliellus|ww05579|
------------------------TTGAGCAGGAATAATTGGAACATCACTTAGACTCTTAATTCGAGCTGAATTAGGAA
CCCCTGGATCTTTAATTGGTGATGATCAAATTTATAATACTATTGTTACAGCTCATGCATTTATTATAATTTTTTTATA
GTTATACCGATTATAATTGGAGGATTTGGAAATTGATTAGTACCTTTAATATTAGGAGCTCCTGATATAGCCTTTCCACG
AATAAATAATATAAGATTTTGATTACTACCCCCATCACTAACTTTATTAATTTCTAGAAGAATTGTTGAAAATGGAGCTG
GAACTGGATGAACAGTTTATCCCCCACTTTCATCCAATATTGCCCATGGTGGAAGTTCAGTAGATCTAGCAATTTTTTCT
CTTCATTTAGCTGGAATTTCCTCAATTTTAGGTGCTATTAATTTTATTACAACAATTATTAATATACGAATTAATGGATT
ATCATTTGATCAAATACCTTTATTTGTTTGATCTGTTGGTATTACAGCCTTATTATTATTACTTTCATTACCTGTTTTAG
CAGGAGCTATTACTATATTATTAACTGATCGAAATTTAAATACATCATTTTTTGATCCTGCTGGTGGAGGAGATCCAATT
CTT------------------------
>LSTEM409-18|Chilo_sp_AM13|ww05580|
----------TTTATTTTTGGAATTTGAGCAGGAATAATCGGAACATCACTTAGACTTTTAATTCGAGCTGAATTAGGAA
CTCCAGGATCTCTAATTGGTGATGATCAAATTTATAATACTATTGTTACAGCTCATGCATTTATTATAATTTTTTTATA
GTTATACCAATTATAATTGGTGGATTTGGAAATTGATTAGTACCTTTAATATTGGGAGCTCCTGATATAGCTTTTCCACG
AATAAATAATATAAGATTTTGATTACTTCCCCCATCATTAACTTTATTAATTTCTAGAAGAATTGTAGAAAATGGAGCTG
GAACAGGATGAACAGTTTACCCCCCACTCTCATCTAATATTGCACATGCTGGAAGTTCAGTAGATTTAGCAATTTTTTCC
CTTCACTTAGCTGGAATTTCTTCTATCTTAGGTGCAATTAACTTTATTACAACAATCATTAATATACGAATTAATGGATT
ATCATTTGATCAAATACCATTGTTTGTTTGATCAGTTGGTATTACAGCCTTATTATTACTACTCTCATTACCAGTTTTAG
CAGGTGCTATTACTATATTATTAACAGATCGAAATTTAAATACATCATTTTTTGATCCCGCTGGTGGGGGTGACCCAATT
CTCTACCA-------------------
>LSTEM410-18|Chilo_sp_AM13|ww05581|
----------TTTATTTTTGGAATTTGAGCAGGAATAATCGGAACATCACTTAGACTTTTAATTCGAGCTGAATTAGGAA
CTCCAGGATCTCTAATTGGTGATGATCAAATTTATAATACTATTGTTACAGCTCATGCATTTATTATAATTTTTTTATA
GTTATACCAATTATAATTGGTGGATTTGGAAATTGATTAGTACCTTTAATATTGGGAGCTCCCGATATAGCTTTTCCACG
AATAAATAATATAAGATTTTGATTACTTCCCCCATCATTAACTTTATTAATTTCTAGAAGAATTGTAGAAAATGGAGCTG
GAACAGGATGAACAGTTTACCCCCCACTCTCATCTAATATTGCACATGCTGGAAGTTCAGTAGATTTAGCAATTTTTTCC
CTTCACTTAGCTGGAATTTCTTCTATCTTAGGTGCAATTAACTTTATTACAACAATCATTAATATACGAATTAATGGGTT
ATCATTTGATCAAATACCATTATTTGTTTGATCAGTTGGTATTACAGCCTTATTATTACTACTCTCATTACCAGTTTTAG
CGGGTGCTATTACTATATTATTAACAGATCGAAATTTAAATACATCATTTTTTGATCCTGCTGGTGGGGGTGACCCCAT-
----
>LSTEM411-18|Chilo_orichalcociliellus|ww05584|

--------------------------------ATAATACTATTGTTACAGCTCATGCATTTATTATAATTTTTTTATA
GTTATACCAATTATAATTGGAGGATTTGGAAATTGATTAGTACCTTTAATATTAGGAGCTCCTGATATAGCCTTCCCACG
AATAAATAATATAAGATTTTGATTACTACCCCCATCACTAACTTTATTAATTTCTAGAAGAATTGTTGAAAATGGAGCTG
GAACTGGGTGAACAGTTTATCCCCCACTTTCATCCAATATTGCCCATGGTGGGAGTTCAGTAGATCTAGCAATTTTTTCT
CTTCATTTAGCTGGAATTTCCTCAATTTTAGGTGCTATTAATTTTATTACAACAATCATTAATATACGAATTAATGGATT
ATCATTTGATCAAATACCTTTATTTGTTTGATCTGTTGGTATTACAGCCTTATTATTATTACTTTCATTACCTGTTTTAG
CAGGAGCTATTACTATATTATTAACTGATCGAAATTTAAATACATCATTTTTTGATCCTGCTGGTGGT------------
----
>LSTEM412-18|Chilo_orichalcociliellus|ww05588|
--AACATCACTTAGACTCTTAATTCGAGCTGAATTAGGAA
CCCCTGGATCTTTAATTGGTGATGATCAAATTTATAATACTATTGTTACAGCTCATGCATTTATTATAATTTTTTTATG
GTTATACCAATTATAATTGGAGGATTTGGAAATTGATTAGTACCTTTAATATTAGGAGCTCCTGATATAGCCTTTCCACG

AATAAATAATATAAGATTTTGATTACTACCCCCATCACTAACTTTATTAATTTCTAGAAGAATTGTTGAAAATGGAGCTG
GAACTGGATGAACAGTTTATCCCCCACTTTCATCTAATATTGCCCATGGTGGAAGTTCTGTAGATCTAGCAATTTTTTCT
CTTCATTTAGCTGGAATTTCCTCAATTTTAGGTGCTATTAATTTTATTACAACAATTATTAATATACGAGTTAATGGATT
ATCATTTGATCAAATACCTTTATTTGTTTGATCTGTTGGTATTACAGCCTTATTATTATTACTTTCATTACCTGTTTTAG
CAGGAGCTATTACTATATTATTAACTGATCGAAATTTAAATACATCATTTTTTGATCCTGCTGGTGGTGGAGATC-----
----
>LSTEM413-18|Chilo_orichalcociliellus|ww05592|
---AACATCACTTAGACTTTTAATTCGAGCTGAATTAGGAA
CCCCTGGATCTTTAATTGGTGATGATCAAATTTATAATACTATTGTTACAGCTCATGCATTTATTATAATTTTTTTTATG
GTTATACCAATTATAATTGGAGGATTTGGAAATTGATTAGTACCTTTAATATTAGGAGCTCCTGATATAGCCTTTCCACG
AATAAATAATATAAGATTTTGATTACTACCCCCATCACTAACTTTATTAATTTCTAGAAGAATTGTTGAAAATGGAGCTG
GAACTGGATGAACAGTTTATCCCCCACTTTCATCTAATATTGCCCATGGTGGAAGTTCTGTAGATCTAGCAATTTTTTCT
CTTCATTTAGCTGGAATTTCCTCAATTTTAGGTGCTATTAATTTTATTACAACAATTATTAATATACGAGTTAATGGATT
ATCATTTGATCAAATACCTTTATTTGTTTGATCTGTTGGTATTACAGCCTTATTATTATTACTTTCATTACCTGTTTTA-
----

>LSTEM414-18|Chilo_orichalcociliellus|ww05593|
---AACATCACTTAGACTTTTAATTCGAGCTGAATTAGGAA
CCCCTGGATCTTTAATTGGTGATGATCAAATTTATAATACTATTGTTACAGCTCATGCATTTATTATAATTTTTTTTATG
GTTATACCAATTATAATTGGAGGATTTGGAAATTGATTAGTACCTTTAATATTAGGAGCTCCTGATATAGCCTTTCCACG
AATAAATAATATAAGATTTTGATTACTACCCCCATCACTAACTTTATTAATTTCTAGAAGAATTGTTGAAAATGGAGCTG
GAACTGGATGAACAGTTTATCCCCCACTTTCATCCAATATTGCCCATGGTGGAAGTTCAGTAGATCTAGCAATTTTTTCC
CTTCATTTAGCTGGAATTTCCTCAATTTTAGGTGCTATCAATTTTATTACAACAATTATTAATATACGAGTTAATGGATT
ATCATTTGATCAAATACCTTTATTTGTTTGATCTGTTGGTATTACAGCCTTATTATTACTACTTTCATTACCTGTTTTAG
CAGGAGCTATTACTATATTATTAACTGATCGAAATTTAAATACATCATTTTTTGATCCTGCTGGTGGAGGAGATCCAAT-
----
>LSTEM415-18|Chilo_orichalcociliellus|ww05594|
---AACATCACTTAGACTCTTAATTCGAGCTGAATTAGGAA
CCCCTGGATCTTTAATTGGTGATGATCAAATTTATAATACTATTGTTACAGCTCATGCATTTATTATAATTTTTTTTATG
GTTATACCAATTATAATTGGAGGATTTGGAAATTGATTAGTACCTTTAATATTAGGAGCTCCTGATATAGCCTTTCCACG
AATAAATAATATAAGATTTTGATTACTACCCCCATCACTAACTTTATTAATTTCTAGAAGAATTGTTGAAAATGGAGCTG
GAACTGGATGAACAGTTTATCCCCCACTTTCATCTAATATTGCCCATGGTGGAAGTTCTGTAGATCTAGCAATTTTTTCT
CTTCATTTAGCTGGAATTTCCTCAATTTTAGGTGCTATTAATTTTATTACAACAATTATTAATATACGAGTTAATGGATT
ATCATTTGATCAAATACCTTTATTTGTTTGATCTGTTGGTATTACAGCCTTATTATTATTACTTTCATTACCTGTTTTAG
CAGGAGCTATTACTATATTATTAACTGATCGAAATTTAAATACATCATTTTTTGATCCTGCTGGTGGAGGAGATCCAATT
CTT------------------------
>LSTEM416-18|Chilo_orichalcociliellus|ww05595|
--ACATCACTTAGACTCTTAATTCGAGCTGAATTAGGAA
CCCCTGGATCTTTAATTGGTGATGATCAAATTTATAATACTATTGTTACAGCTCATGCATTTATTATAATTTTTTTTATA
GTTATACCAATTATAATTGGAGGATTTGGAAATTGATTAGTACCTTTAATATTAGGAGCTCCTGATATAGCCTTTCCACG
AATAAATAATATAAGATTTTGATTACTACCCCCATCACTAACTTTATTAATTTCTAGAAGAATTGTTGAAAATGGAGCTG
GAACTGGATGAACAGTTTATCCCCCACTTTCATCTAATATTGCCCATGGTGGAAGTTCTGTAGATCTAGCAATTTTTTCT
CTTCATTTAGCTGGAATTTCCTCAATTTTAGGTGCTATTAATTTTATTACAACAATTATTAATATACGAGTTAATGGATT
ATCATTTGATCAAATACCTTTATTTGTTTGATCTGTTGGTATTACAGCCTTATTATTATTACTTTCATTACCTGTTTTAG
CAGGAGCTATTACTATATTATTAACTGATCGAAATTTAAATACATCATTTTTTGATCCTGCTGGTGG-------------
----
>LSTEM417-18|Chilo_sp_AM16|ww05596|
--ACATCAGTTAGACTTTTAATTCGAGCTGAATTAGGAA
CTCCAGGATCTTTAATTTGTGATGATCAAATTTACAATACTATTGTTACAGCTCACGCATTTATTATAATTTTTTTTATA
GTTATACCAATTATAATTGGAGGATTTGGAAATTGATTGGTACCTCTAATGTTAGGAGCTCCTGATATAGCTTTTCCACG
AATAAATAATATAAGATTTTGATTACTCCCCCCATCACTAACTTTACTAATTTCTAGAAGAATTGTAGAAACTGGAGCCG
GAACAGGATGAACAGTTTACCCCCCACTATCATCTAATATCGCACATGCTGGAAGTTCAGTAGATTTAGCAATTTTTTCC
CTCCATTTAGCTGGGATTTCTTCTATTTTAGGAGCTATTAATTTTATTACAACAATTATTAATATACGAATTAATGGATT
ATCATTTGATCAAATACCATTATTTGTTTGATCAGTTGGTATTACAGCTTTATTATTATTACTTTCTTTACCTGTTTTAG
CAGGTGCTATTACTATATTATTAACAGATCGAAATCTAAATACATCATTTTT----------------------------
----
>LSTEM418-18|Chilo_orichalcociliellus|ww05597|

--ATCACTTAGACTCCTAATTCGAGCTGAATTAGGGA
CCCCTGGATCTTTAATTGGTGATGATCAAATTTATAATACTATTGTTACAGCTCATGCATTTATTATAATTTTTTTTATA
GTTATACCAATTATAATTGGAGGATTTGGAAATTGATTAGTACCTTTAATGTTAGGAGCTCCTGATATAGCCTTCCCACG
AATAAATAATATAAGATTTTGATTACTACCCCCATCACTAACTTTATTAATTTCTAGAAGAATTGTTGAAAATGGAGCTG
GAACTGGGTGAACAGTTTATCCCCCACTTTCATCCAATATTGCCCATGGTGGGAGTTCAGTAGATCTAGCAATTTTTTCT
CTTCATTTAGCTGGAATTTCCTCAATTTTAGGTGCTATTAATTTTATTACAACAATCATTAATATACGAATTAATGGATT
ATCATTTGATCAAATACCTTTATTTGTTTGATCTGTTGGTATTACAGCCTTATTATTATTACTTTCATTACCTGTTTTAG
CAGGAGCTATTACTATATTATTAACTGATCGAAATTTAAATACATCATTTTTT-------------------------
----
>LSTEM419-18|Chilo_orichalcociliellus|ww05598|
------------------------GAGCAGGAATAATTGGAACATCACTTAGACTCCTAATTCGAGCTGAATTAGGGA
CCCCTGGATCTTTAATTGGTGATGATCAAATTTATAATACTATTGTTACAGCTCATGCATTTATTATAATTTTTTTTATA
GTTATACCAATTATAATTGGAGGATTTGGAAATTGATTAGTACCTTTAATGTTAGGAGCTCCTGATATAGCCTTCCCACG
AATAAATAATATAAGATTTTGATTACTACCCCCATCACTAACTTTATTAATTTCTAGAAGAATTGTTGAAAATGGAGCTG
GAACTGGGTGAACAGTTTATCCCCCACTTTCATCCAATATTGCCCATGGTGGGAGTTCAGTAGATCTAGCAATTTTTTCT
CTTCATTTAGCTGGAATTTCCTCAATTTTAGGTGCTATTAATTTTATTACAACAATCATTAATATACGAATTAATGGATT
ATCATTTGATCAAATACCTTTATTTGTTTGATCTGTTG---------------------------------------
----

>LSTEM420-18|Chilo_orichalcociliellus|ww05599|
--ATCACTTAGACTCCTAATTCGAGCTGAATTAGGAA
CCCCTGGATCTTTAATTGGTGATGATCAAATTTATAATACTATTGTTACAGCTCATGCATTTATTATAATTTTTTTTATA
GTTATACCAATTATAATTGGAGGATTTGGAAATTGATTAGTACCTTTAATGTTAGGAGCTCCTGATATAGCCTTCCCACG
AATAAATAATATAAGATTTTGATTACTACCCCCATCACTAACTTTATTAATTTCTAGAAGAATTGTTGAAAATGGAGCTG
GAACTGGGTGAACAGTTTATCCCCCACTTTCATCCAATATTGCCCATGGTGGGAGTTCAGTAGATCTAGCAATTTTTTCT
CTTCATTTAGCTGGAATTTCCTCAATTTTAGGTGCTATTAATTTTATTACAACAATCATTAATATACGAATTAATGGATT
ATCATTTGATCAAATACCTTTATTTGTTTGATCTGTTGGTATTACAGCCTTATTATTATTACTTTCATTACCTGTTTTAG
CAGGAGCTATTACTATATTATTAACTGATCGAAATTTAAATACATCATTTTTTGATCCTGCTGGTGGT------------
----
>LSTEM421-18|Chilo_orichalcociliellus|ww05601|
--ACATCACTTAGACTCCTAATTCGAGCTGAATTAGTGA
CCCCTGGATCTTTAATTGGTGATGATCAAATTTATAATACTATTGTTACAGCTCATGCATTTATTATAATTTTTTTTATA
GTTATACCAATTATAATTGGAGGATTTGGAAATTGATTAGTACCTTTAATGTTAGGAGCTCCTGATATAGCCTTCCCACG
AATAAATAATATAAGATTTTGATTACTACCCCCATCACTAACTTTATTAATTTCTAGAAGAATTGTTGAAAATGGAGCTG
GAACTGGGTGAACAGTTTATCCCCCACTTTCATCCAATATTGCCCATGGTGGAAGTTCAGTAGATCTAGCAATTTTTTCT
CTTCATTTAGCTGGAATTTCCTCAATTTTAGGTGCTATTAATTTTATTACAACAATCATTAATATACGAATTAATGGATT
ATCATTTGATCAAATACCTTTATTTGTTTGATCTGTTGGTATTACAGCCTTATTATTATTACTTTCATTACCTGTTTTAG
CAGGAGCTATTACTATATTATTAACTGATCGAAATTTAAATACATCATTTTTTGATCCTGCTGGTGGT------------
----
>LSTEM422-18|Chilo_orichalcociliellus|ww05602|
------------------------GAGCAGGAATAATTGGAACATCACTTAGACTCCTAATTCGAGCTGAATTAGGAA
CCCCTGGATCTTTAATTGGTGATGATCAAATTTATAATACTATTGTTACAGCTCATGCATTTATTATAATTTTTTTTATA
GTTATACCAATTATAATTGGAGGATTTGGAAATTGATTAGTACCTTTAATGTTAGGAGCTCCTGATATAGCCTTCCCACG
AATAAATAATATAAGATTTTGATTACTACCCCCATCACTAACTTTATTAATTTCTAGAAGAATTGTTGAAAATGGAGCTG
GAACTGGGTGAACAGTTTATCCCCCACTTTCATCCAATATTGCCCATGGTGGGAGTTCAGTAGATCTAGCAATTTTTTCT
CTTCATTTAGCTGGAATTTCCTCAATTTTAGGTGCTATTAATTTTATTACAACAATCATTAATATACGAATTAATGGATT
ATCATTTGATCAAATACCTTTATTTGTTTGATCTGTTGGTATTACAGCCTTATTATTATTACTTTCATTACCTGTTTTAG
CAGGAGCTATTACTATATTATTAACTGATCGAAATTTAAATACATCATTTTTTGATCCTGCTGGTGGT------------
----
>LSTEM423-18|Chilo_orichalcociliellus|ww05603|
---CATCACTTAGACTCCTAATTCGAGCTGAATTAGGAA
CCCCTGGATCTTTAATTGGTGATGATCAAATTTATAATACTATTGTTACAGCTCATGCATTTATTATAATTTTTTTTATA
GTTATACCAATTATAATTGGAGGATTTGGAAATTGATTAGTACCTTTAATGTTAGGAGCTCCTGATATAGCCTTCCCACG
AATAAATAATATAAGATTTTGATTACTACCCCCATCACTAACTTTATTAATTTCTAGAAGAATTGTTGAAAATGGAGCTG
GAACTGGGTGAACAGTTTATCCCCCACTTTCATCCAATATTGCCCATGGTGGGAGTTCAGTAGATCTAGCAATTTTTTCT
CTTCATTTAGCTGGAATTTCCTCAATTTTAGGTGCTATTAATTTTATTACAACAATCATTAATATACGAATTAATGGATT
ATCATTTGATCAAATACCTTTATTTGTTTGATCTGTTGGTATTACAGCCTTATTATTATTACTTTCATTACCTGTTTTAG

CAGGAGCTATTACTATATTATTAACTGATCGAAATTTAAATACATCATTTTTTGATCCTGCTGGTGGT------------
----
>LSTEM424-18|Chilo_orichalcociliellus|ww05604|
------------TATTTTTGGAATTTGAGCAGGAATAATTGGAACATCACTTAGACTCCTAATTCGAGCTGAATTAGGAA
CCCCTGGATCTTTAATTGGTGATGATCAAATTTATAATACTATTGTTACAGCTCATGCATTTATTATAATTTTTTTTATA
GTTATACCAATTATAATTGGAGGATTTGGAAATTGATTAGTACCTTTAATGTTAGGAGCTCCTGATATAGCCTTCCCACG
AATAAATAATATAAGATTTTGATTACTACCCCCATCACTAACTTTATTAATTTCTAGAAGAATTGTTGAAAATGGAGCTG
GAACTGGGTGAACAGTTTATCCCCCACTTTCATCCAATATTGCCCATGGTGGGAGTTCAGTAGATCTAGCAATTTTTTCT
CTTCATTTAGCTGGAATTTCCTCAATTTTAGGTGCTATTAATTTTATTACAACAATCATTAATATACGAATTAATGGATT
ATCATTTGATCAAATACCTTTATTTGTTTGATCTGTTGGTATTACAGCCTTATTATTATTACTTTCATTACCTGTTTTAG
CAGGAGCTATTACTATATTATTAACTGATCGAAATTTAAATACATCATTTTTTGATCCTGCTGGTGGAGGAGATCCAAT-
----
>LSTEM425-18|Chilo_orichalcociliellus|ww05605|
---ATCACTTAGACTCCTAATTCGAGCTGAATTAGGGA
CCCCTGGATCTTTAATTGGTGATGATCAAATTTATAATACTATTGTTACAGCTCATGCATTTATTATAATTTTTTTTATA
GTTATACCAATTATAATTGGAGGATTTGGAAATTGATTAGTACCTTTAATGTTAGGAGCTCCTGATATAGCCTTCCCACG
AATAAATAATATAAGATTTTGATTACTACCCCCATCACTAACTTTATTAATTTCTAGAAGAATTGTTGAAAATGGAGCTG
GAACTGGGTGAACAGTTTATCCCCCACTTTCATCCAATATTGCCCATGGTGGGAGTTCAGTAGATCTAGCAATTTTTTCT
CTTCATTTAGCTGGAATTTCCTCAATTTTAGGTGCTATTAATTTTATTACAACAATCATTAATATACGAATTAATGGATT
ATCATTTGATCAAATACCTTTATTTGTTTGATCTGTTGGTATTACAGCCTTATTATTATTACTTTCATTACCTGTTTTAG
CAGGAGCTATTACTATATTATTAACTGATCGAAATTTAAATACATCATTTTTTG---------------------------
----
>LSTEM426-18|Chilo_sp_AM12|AMSww06125|
AACTTTATATTTTATTTTTGGAATTTGAGCAGGAATAATTGGAACATCACTTAGACTTTTAATTCGAGCTGAATTAGGAA
CTCCAGGATCTTTAATTGGTGATGATCAAATTTATAATACTATTGTTACAGCTCATGCATTTATTATAATTTTTTTTATA
GTTATACCAATTATAATTGGTGGATTTGGAAATTGATTAGTACCTTTAATGTTAGGAGCTCCCGATATAGCTTTTCCACG
AATAAATAATATAAGATTTTGATTACTCCCCCCATCATTAACTTTATTAATTTCTAGTAGAATTGTAGAAAATGGAGCTG
GAACAGGATGAACAGTTTACCCCCCACTCTCATCTAATATTGCACATGCTGGAAGTTCAGTAGATTTAGCAATTTTTTCC
CTTCATTTAGCTGGAATTTCCTCTATTTTAGGTGCAATTAACTTTATTACAACTATCATTAATATACGAATTAATGGATT
ATCGTTTGATCAAATACCATTATTTGTTTGATCCGTCGGTATTACAGCTTTATTATTATTACTTTCATTACCAGTTTTA-
----

>LSTEM427-18|Chilo_orichalcociliellus|AMSww06126|
AACTTTATATTTTATTTTTGGAATTTGAGCAGGAATAATTGGAACATCACTTAGACTCCTAATTCGAGCTGAATTGGGAA
CCCCTGGATCTTTAATTGGTGATGATCAAATTTATAATACTATTGTTACAGCTCATGCATTTATTATAATTTTTTTTATA
GTTATACCAATTATAATTGGAGGATTTGGAAATTGATTAGTACCTTTAATGTTAGGAGCTCCTGATATAGCCTTCCCACG
AATAAATAATATAAGATTTTGATTACTACCCCCATCACTAACTTTATTAATTTCTAGAAGAATTGTTGAAAATGGAGCTG
GAACTGGGTGAACAGTTTATCCCCCACTTTCATCCAATATTGCCCATGGTGGGAGTTCAGTAGATCTAGCAATTTTTTCT
CTTCATTTAGCTGGAATTTCCTCAATTTTAGGTGCTATTAATTTTATTACAACAATCATTAATATACGAATTAATGGATT
ATCATTTGATCAAATACCTTTATTTGTTTGATCTGTTGGTATTACAGCCTTATTATTATT--------------------
----

>LSTEM428-18|Chilo_partellus|AMSww06127|
AACTTTATATTTTATTTTTGGAATTTGAGCAGGAATAATTGGGACATCCCTTAGATTATTAATTCGTGCAGAATTAGGAA
CTCCTGGATCTTTAATTGGAGATGATCAAATTTATAATACTATTGTAACAGCACACGCATTTATTATAATTTTTTTTATA
GTTATACCAATTATAATTGGTGGATTTGGAAATTGATTAGTACCTTTAATATTAGGAGCCCCAGATATAGCTTTCCCACG
AATAAATAATATAAGATTTTGATTATTACCACCATCATTAACTTTATTAATTTCTAGAAGAATTGTTGAAAATGGAGCTG
GAACAGGATGAACAGTGTACCCCCCACTATCATCTAATATTGCTCATGCTGGAAGTTCAGTAGATTTAGCAATTTTTTCT
TTACATTTAGCTGGTATTTCATCAATTCTCGGTGCTATTAATTTTATTACAACAATTATTAATATACGAATTAATGGATT
ATCTTTTGATCAAATACCATTATTTGTTTGATCTGTAGGTATTACAGCTTTATTATTATTACTTTCTTTACCTGTTTTAG
CTGGAGCTATTACTATATTATTAAC---

>LSTEM429-18|Lepidoptera|AMSww06128|
AACATTATATTTTATTTTTGGAATTTGAGCAGGAATAGTTGGAACATCTCTAAGATTATTAATTCGAGCTGAATTAGGAA
ATCCTGGATCATTAATTGGAGATGATCAAATTTATAATACTATTGTTACAGCTCATGCATTTATTATAATTTTTTTTATA
GTTATACCAATTATAATTGGAGGATTTGGAAATTGATTAGTACCTTTAATATTAGGAGCTCCTGATATAGCTTTTCCACG
AATAAATAATATAAGTTTCTGATTACTCCCTCCATCTTTAACACTTTTAATTTCAAGAAGAATTGTTGAAAACGGAGCAG

GAACTGGATGAACAGTATACCCCCCACTTTCATCTAATATTGCTCATGGAGGAAGTTCTGTTGATCTAGCTATTTTTTCA
TTACATTTAGCTGGAATTTCCTCAATTTTAGGAGCTATTAATTTTATTACTACAATTATTAATATACGTATTAATGGTTT
ATCATTTGATCAAATACCTTTATTTGTTTGATCTGTAGGTATTACAGCTTTATTACTTTTATTATCTTTACCTGTATTA-
----

>LSTEM430-18|Lepidoptera|AMSww06129|
AACATTATATTTTATTTTTGGAATTTGAGCAGGAATAGTTGGAACATCTCTAAGATTATTAATTCGAGCTGAATTAGGAA
ATCCTGGATCATTAATTGGAGATGATCAAATTTATAATACTATTGTTACAGCTCATGCATTTATTATAATTTTTTTTATA
GTTATACCAATTATAATTGGAGGATTTGGAAATTGATTAGTACCTTTAATATTAGGAGCTCCTGATATAGCTTTTCCACG
AATAAATAATATAAGTTTCTGATTACTCCCTCCATCTTTAACACTTTTAATTTCAAGAAGAATTGTTGAAAACGGAGCAG
GAACTGGATGAACAGTATACCCCCCACTTTCATCTAATATTGCTCATGGAGGAAGTTCTGTTGATCTAGCTATTTTTTCA
CTGCATTTAGCTGGAATTTCCTCAATTTTAGGAGCTATTAATTTTATTACTACAATTATTAATATACGTATTAATGGTTT
ATCATTTGATCAAATACCTTTATTTGTTTGATCTGTAGGTATTACAGCTTTATTACTTTTATTATCTTTACCTGTATTA-
----

>LSTEM431-18|Lepidoptera|AMSww06131|
AACATTATATTTTATCTTTGGAATTTGAGCGGGTATAGTGGGAACCTCCCTCAGACTCTTAATTCGTGCGGAGTTAGGAA
ATCCTGGATCATTAATTGGTGATGATCAAATTTATAATACTATTGTTACTGCTCATGCATTTATTATAATTTTTTTTATG
GTTATACCTATTATAATTGGTGGTTTTGGAAATTGGTTAGTTCCTTTAATATTGGGGGCCCCAGATATGGCATTCCCTCG
AATAAATAATATGAGATTTTGATTATTACCCCCCTCTCTTACTTTATTAATTTCAAGAAGAATTGTAGAAAATGGGGCTG
GAACTGGATGAACGGTCTATCCCCCCCTATCTTCTAATATTGCTCATGGTGGAAGTTCAGTTGATCTAGCTATTTTTTCA
TTACACTTAGCGGGAATTTCATCAATTTTAGGAGCTATTAATTTTATTACCACAATTATTAATATGCGAATTAATGGACT
ATCATTTGATCAAATACCATTATTTGTTTGGTCAGTGGGTATTACAGCTTTACTTCTCCTTCTTTCTCTTCCAGTATTAG
CAGGAGCTATTACCATATTATTAACTGATCGAAATTTAAATACATCATTTTTTGACCCTGCTGGGGGAGGGGATCCTATT
CTTTACCAACATTTATTTTGATTTTTT
>LSTEM432-18|Lepidoptera|AMSww06132|
AACATTATATTTTATTTTTGGAATTTGAGCAGGAATAATTGGAACATCTTTAAGATTATTAATTCGAGCTGAATTAGGAA
ATCCTGGATCTTTAATTGGAGATGATCAAATTTATAATACTATTGTTACAGCTCACGCATTTATCATAATTTTTTTTATA
GTTATACCAATTATAATTGGAGGGTTTGGAAATTGATTAGTACCATTAATATTAGGAGCTCCAGATATAGCTTTCCCCCG
AATAAATAATATAAGATTTTGATTATTACCCCCATCTTTAACACTTTTAATTTCAAGTAGAATTGTTGAAAATGGAGCAG
GAACTGGATGAACAGTATACCCCCCACTTTCATCTAATATTGCTCATGGTGGAAGATCTGTTGATTTAGCTATCTTTTCT
TTACATTTAGCAGGAATTTCTTCAATTTTAGGAGCTATTAATTTTATTACTACAATTATTAATATACGAATTAATGGATT
ATCTTTTGATCAAATACCTTTATTTGTTTGATCTGTAGGAATTACAGCTTTATTACTTTTATTATCATTACCTGTCTTAG
CAGGAGCTATTACTATATTATTAACTGATCGAAACTTAAA--

>LSTEM433-18|Lepidoptera|AMSww06133|
AACTTTATATTTTATTTTTGGTATTTGAGCAGGAATATTAGGAACATCTTTAAGACTTTTAATTCGAGCAGAATTAGGTA
ACCCAGGATCATTAATTGGAGATGATCAAATTTATAATACTATTGTAACAGCTCATGCATTCATTATAATTTTCTTTATA
GTAATACCAATTATAATTGGTGGATTTGGAAATTGATTAGTTCCTTTAATATTAGGAGCTCCTGATATAGCTTTCCCACG
AATAAATAATATAAGATTCTGATTATTACCCCCCTCTTTAACTCTATTAATTTCTAGAAGAATTGTTGAAAATGGAGCAG
GAACAGGATGAACAGTGTACCCCCCACTTTCATCTAATATTGCTCATGGAGGAGGATCAGTTGATTTAGCCATCTTTTCC
TTACATCTCGCTGGAATTTCTTCTATTTTAGGAGCTATTAATTTTATTACAACAATTATTAATATACGAATTAATAATTT
ATCTTTTGATCAAATACCTTTATTTGTTTGATCTGTAGGTATTACAGCTTTATTATTACTTCTTTCCCTTCCAGTATTA-
----

>LSTEM434-18|Lepidoptera|AMSww06134|
AACTTTATATTTTATTTTTGGTATTTGAGCAGGAATATTAGGAACATCTTTAAGACTTTTAATTCGAGCAGAATTAGGTA
ACCCAGGATCATTAATTGGAGATGATCAAATTTATAATACTATTGTAACAGCTCATGCATTCATTATAATTTTCTTTATA
GTAATACCAATTATAATTGGTGGATTTGGAAATTGATTAGTTCCTTTAATATTAGGAGCTCCTGATATAGCTTTCCCACG
AATAAATAATATAAGATTCTGATTATTACCCCCCTCTTTAACTCTATTAATTTCTAGAAGAATTGTTGAAAATGGAGCAG
GAACAGGATGAACAGTGTACCCCCCACTTTCATCTAATATTGCTCATGGAGGAGGATCAGTTGATTTAGCCATCTTTTCC
TTACATCTCGCTGGAATTTCTTCTATTTTAGGAGCTATTAATTTTATTACAACAATTATTAATATACGAATTAATAATTT
ATCTTTTGATCAAATACCTTTATTTGTTTGATCTGTAGGTATTACAGCTTTATTATTACTTCTTTCCCTTCCAGTATTA-
----

>LSTEM435-18|Chilo_orichalcociliellus|AMSww06135|
AACTTTATATTTTATTTTTGGAATTTGAGCAGGAATAATTGGAACATCACTTAGACTCCTAATTCGAGCTGAATTAGGAA

CCCCTGGATCTTTAATTGGTGATGATCAAATTTATAATACTATTGTTACAGCTCATGCATTTATTATAATTTTTTTTATA
GTTATACCAATTATAATTGGAGGATTTGGAAATTGATTAGTACCTTTAATGTTAGGAGCTCCTGATATAGCCTTCCCACG
AATAAATAATATAAGATTTTGATTACTACCCCCATCACTAACTTTATTAATTTCTAGAAGAATTGTTGAAAATGGAGCTG
GAACTGGGTGAACAGTTTATCCCCCACTTTCATCCAATATTGCCCATGGTGGAAGTTCAGTAGATCTAGCAATTTTTTCT
CTTCATTTAGCTGGAATTTCCTCAATTTTAGGTGCTATTAATTTTATTACAACAATCATTAATATACGAATTAATGGATT
ATCATTTGATCAAATACCTTTATTTGTTTGATCTGTTGGTATTACAGCCTTATTATTATTACTTTCATTACCTGTTTTA-
----

>LSTEM436-18|Lepidoptera|AMSww06136|
AACATTATATTTTATTTTTGGAATTTGAGCAGGAATAGTAGGAACATCTCTTAGATTACTGATTCGAGCTGAATTAGGAA
ATCCAGGCTCTTTAATTGGGGATGATCAAATTTATAATACTATTGTTACTGCTCATGCATTCATTATAATTTTTTTTATA
GTTATACCTATCATAATTGGAGGATTTGGAAATTGATTAGTACCTTTAATATTAGGAGCTCCTGATATAGCATTCCCCCG
AATAAACAATATAAGTTTTTGATTGTTACCCCCCTCTTTAACTTTATTAATTTCAAGAAGAATCGTAGAAAATGGAGCAG
GAACAGGATGAACAGTTTACCCTCCTTTATCATCTAATATTGCTCATGGGGGAAGATCTGTAGATTTAGCTATTTTTTCC
CTTCATTTAGCTGGAATCTCTTCTATTCTAGGAGCTATTAATTTTATTACTACAATCATTAATATAAAAATTAATGGTTT
ATCTTTTGATCAATTATCATTATTTGTTTGATCAGTAAGTATCACTGCACTACTTCTACTTCTTTCTTTACCTGTTTTAG
CTGGAGCTATTACTATATTATTAACTGATCGAAATTTAAATACATCATTTTTTGATCCTGCAGGAGGGGGAGATCCAATT
CTTTATCAACATTTATTTTGATTTTTT
>LSTEM437-18|Lepidoptera|AMSww06137|
AACTTTATATTTTATTTTTGGAATTTGAGCAGGTATAGTAGGAACATCTCTAAGTTTATTAATTCGAGCTGAATTAGGGA
ATCCTGGATCATTAATTGGAGATGATCAAATTTATAACACTATTGTTACTGCTCATGCATTTATTATAATTTTTTTTATG
GTTATACCTATTATAATTGGGGGGTTTGGAAATTGATTAGTACCTCTCATATTAGGAGCCCCTGATATAGCTTTTCCTCG
AATAAATAATATAAGTTTTTGATTATTACCCCCCTCATTGATATTATTAATTTCAAGAAGAATTGTAGAAAATGGAGCTG
GAACTGGTTGAACTGTGTATCCCCCTCTATCCTCTAATATTGCTCACGGGGGTGGTTCTGTAGATTTAGCTATCTTTTCA
TTACACCTAGCCGGTATTTCATCAATTTTAGGGGCTATTAATTTTATTACTACAATTATTAATATAAAAATCAATGGATT
ATCATTTGATCAAATACCTCTATTTGTTTGATCCGTAGGAATTACCGCTCTTTTATTACTTTTATCATTACCAGTATTAG
CAGGTGCTATTACTATACTTCTAACTGATCGAAATTTAAATACATCATTTTTCGATCCTGCTGGAGGGGGAGATCCTATT
TTATATCAACATTTATTTTGATTTTTT
>LSTEM438-18|Lepidoptera|AMSww06139|
AACATTATATTTTATTTTTGGAATTTGAGCAGGAATAGTTGGAACATCTCTAAGATTATTAATTCGAGCTGAATTAGGAA
ATCCTGGATCATTAATTGGAGATGATCAAATTTATAATACTATTGTTACAGCTCATGCATTTATTATAATTTTTTTTATA
GTTATACCAATTATAATTGGAGGATTTGGAAATTGATTAGTACCTTTAATATTAGGAGCTCCTGATATAGCTTTTCCACG
AATAAATAATATAAGTTTCTGATTACTCCCTCCATCTTTAACACTTTTAATTTCAAGAAGAATTGTTGAAAACGGAGCAG
GAACTGGATGAACAGTATACCCCCCACTTTCATCTAATATTGCTCATGGAGGAAGTTCTGTTGATCTAGCTATTTTTTCA
TTGCATTTAGCTGGAATTTCTTCAATTTTAGGAGCTATTAATTTTATTACTACAATTATTAATATGCGTATTAATGGTTT
ATCATTTGATCAAATACCTTTATTTGTTTGATCTGTAGGTATTACAGCTTTATTACTTTTATTATCTTTACCTGTATTAG
CTGGAGCTATTACAATATTACTAACTGATCGAAATCTAAATACATCTTTCTTTGATCCAGCTGGAGGAGGAGATCCTATC
TTATACCAACATTTATTTTGATTTTTT
>LSTEM439-18|Chilo_partellus|AMSww06140|
AACTTTATATTTTATTTTTGGAATTTGAGCAGGAATAATTGGGACATCCCTTAGATTATTAATTCGTGCAGAATTAGGAA
CTCCTGGATCTTTAATTGGAGATGATCAAATTTATAATACTATTGTAACAGCACACGCATTTATTATAATTTTTTTTATA
GTTATACCAATTATAATTGGTGGATTTGGAAATTGATTAGTACCTTTAATATTAGGAGCCCCAGATATAGCTTTCCCACG
AATAAATAATATAAGATTTTGATTATTACCACCATCATTAACTTTATTAATTTCTAGAAGAATTGTTGAAAATGGAGCTG
GAACAGGATGAACAGTGTACCCCCCACTATCATCTAATATTGCTCATGCCGGAAGTTCAGTAGATTTAGCAATTTTTTCT
TTACATTTAGCTGGTATTTCATCAATTCTCGGTGCTATTAATTTTATTACAACAATTATTAATATACGAATTAATGGATT
ATCTTTTGATCAAATACCATTATTTGTTTGATCTGTAGGTATTACAGCTTTATTATTATTACTTTCTTTACCTGTTTTAG
CTGGAGCTATTACTATATTATTAACAGATCGAAATTTAAATACATCCTTTTTCGATCCTGCTGGAGGAGGAGATCCTATT
CTTTATCAACACTTATTTTGATTTTTT
>LSTEM440-18|Chilo_partellus|AMSww06141|
AACTTTATATTTTATTTTTGGAATTTGAGCAGGAATAATTGGGACATCCCTTAGATTATTAATTCGTGCAGAATTAGGAA
CTCCTGGATCTTTAATTGGAGATGATCAAATTTATAATACTATTGTAACAGCACACGCATTTATTATAATTTTTTTTATA
GTTATACCAATTATAATTGGTGGATTTGGAAATTGATTAGTACCTTTAATATTAGGAGCCCCAGATATAGCTTTCCCACG
AATAAATAATATAAGATTTTGATTATTACCACCATCATTAACTTTATTAATTTCTAGAAGAATTGTTGAAAATGGAGCTG
GAACAGGATGAACAGTGTACCCCCCACTATCATCTAATATTGCTCATGCCGGAAGTTCAGTAGATTTAGCAATTTTTTCT
TTACATTTAGCTGGTATTTCATCAATTCTCGGTGCTATTAATTTTATTACAACAATTATTAATATACGAATTAATGGATT
ATCTTTTGATCAAATACCATTATTTGTTTGATCTGTAGGTATTACAGCTTTATTATTATTACTTTCTTTACCTGTTTTAG
CTGGAGCTATTACTATATTATTAACAGATAGAAATTTAAATACATCCTTTTTCGATCCTGCCGGAGG-------------

----
>LSTEM441-18|Chilo_orichalcociliellus|AMSww06142|
AACTTTATATTTTATTTTTGGAATTTGAGCAGGAATAATTGGAACATCACTTAGACTCCTAATTCGAGCTGAATTAGGAA
CCCCTGGATCTTTAATTGGTGATGATCAAATTTATAATACTATTGTTACAGCTCATGCATTTATTATAATTTTTTTTATA
GTTATACCAATTATAATTGGAGGATTTGGAAATTGATTAGTACCTTTAATGTTAGGAGCTCCTGATATAGCCTTCCCACG
AATAAATAATATAAGATTTTGATTACTACCCCCATCACTAACTTTATTAATTTCTAGAAGAATTGTTGAAAATGGAGCTG
GAACTGGGTGAACAGTTTATCCCCCACTTTCATCCAATATTGCCCATGGTGGGAGTTCAGTAGATCTAGCAATTTTTTCT
CTTCATTTAGCTGGAATTTCCTCAATTTTAGGTGCTATTAATTTTATTACAACAATCATTAATATACGAATTAATGGATT
ATCATTTGATCAAATACCTTTATTTGTTTGATCTGTTGGTATTACAGCCTTATTATTATTACTTTCATTACCTGTTTTAG
CAGGAGCTATTACTATATTATTAACTGATCGAAATTTAAATACATCATTTTTTGATCCTGCTGGTGGAGGAGATCCAATT
CTTTATCAACATTTATTTTGATTTTTT
>LSTEM442-18|Chilo_sp_AM15|AMSww06143|
AACTTTATATTTTATTTTTGGAATTTGGGCAGGAATAATTGGAACATCACTTAGACTTTTAATTCGAGCTGAATTAGGAA
CTCCAGGATCTTTAATTGGTGATGATCAAATTTATAATACTATTGTTACAGCTCATGCATTTATTATAATTTTTTTTATA
GTTATACCAATTATAATTGGTGGATTTGGAAATTGATTAGTGCCTTTAATATTAGGAGCTCCTGATATAGCTTTTCCACG
AATAAATAATATAAGATTTTGATTACTCCCCCCATCATTAACTTTATTAATTTCTAGTAGAATTGTAGAAAATGGAGCTG
GAACAGGATGAACAGTTTACCCCCCACTCTCATCTAATATTGCCCATGCTGGAAGTTCAGTAGATTTGGCAATTTTTTCC
CTTCACTTAGCAGGTATTTCTTCTATCTTAGGTGCAATTAACTTTATTACAACAATCATTAATATACGAATTAATGGATT
ATCATTTGATCAAATACCATTATTTGTTTGATCTGTTGGTATTACAGCTTTATTATTATTACTTTCATTACCAGTTTTAG
CGGGTGCTATTACTATATTATTAACAGATCGAAATTTAAATACATCATTTTTTGATCCTGCTGGTGGGGGTGACCCCATT
CTTTATCAACACTTATTTTGATTTTTT
>LSTEM443-18|Chilo_orichalcociliellus|AMSww06144|
AACTTTATATTTTATTTTTGGAATTTGAGCAGGAATAATTGGAACATCACTTAGACTCCTAATTCGAGCTGAATTAGGAA
CCCCTGGATCTTTAATTGGTGATGATCAAATTTATAATACTATTGTTACAGCTCATGCATTTATTATAATTTTTTTTATA
GTTATACCAATTATAATTGGAGGATTTGGAAATTGATTAGTACCTTTAATGTTAGGAGCTCCTGATATAGCCTTCCCACG
AATAAATAATATAAGATTTTGATTACTACCCCCATCACTAACTTTATTAATTTCTAGAAGAATTGTTGAAAATGGAGCTG
GAACTGGATGAACAGTTTATCCCCCACTTTCATCCAATATTGCCCATGGTGGGAGTTCAGTAGATCTAGCAATTTTTTCT
CTTCATTTAGCTGGAATTTCCTCAATTTTAGGTGCTATTAATTTTATTACAACAATCATTAATATACGAATTAATGGATT
ATCATTTGATCAAATACCTTTATTTGTTTGATCTGTTGGTATTACAGCCTTATTATTATTACTTTCATTACCTGTTTTA-
----

>LSTEM444-18|Chilo_sp_AM11|AMSww06145|
------ATATTTTATTTTTGGKATTTGAGCAGGAATAATTGGGACATCACTCAGACTTTTAATTCGAGCTGAATTAGGGA
CTCCTGGATCCTTAATTGGTGATGATCAAATTTATAATACTATTGTCACAGCCCATGCATTTATTATAATTTTTTTTATA
GTTATACCAATTATAATTGGTGGATTTGGAAATTGATTAGTACCTCTAATATTGGGAGCTCCTGATATAGCTTTCCCACG
AATAAATAATATAAGATTTTGATTACTCCCCCCATCATTAACTTTATTAATTTCTAGTAGAATTGTAGAAAATGGAGCTG
GAACAGGATGAACAGTTTACCCCCCACTTTCATCTAATATTGCTCATGCTGGAAGTTCAGTAGATTTAGCAATTTTTTCC
CTTCATTTAGCTGGAATTTCTTCTATTTTAGGTGCAATTAATTTTATTACAACAATCATTAATATACGAATTAATAGATT
ATTATTTGATCAAATACCATTATTTATTTGATCTGTTGGTATTACAGCCTTATTATTATTACTTTCATTACCAGTTTTAG
CGGGTGCTATTACTATATTATTAACAGATCGAAATTTAAATACATCATTTTTTGATCCTGCTGGTGGAGGAGATCCAATT
CTTTACCAACATTTATTTTGATTTTTT
>LSTEM445-18|Chilo_orichalcociliellus|AMSww06147|
AACTTTATATTTTATTTTTGGAATTTGAGCAGGAATAATTGGAACATCACTTAGACTTCTAATTCGAGCTGAATTAGGAA
CCCCTGGATCCCTAATTGGTGACGATCAAATTTATAATACTATTGTTACAGCTCATGCATTTATTATAATTTTTTTTATA
GTTATACCGATTATAATTGGAGGATTTGGAAATTGATTAGTACCTTTAATATTAGGAGCTCCTGATATAGCCTTTCCACG
AATAAATAATATAAGATTTTGATTACTACCCCCATCACTAACTTTATTAATTTCTAGAAGAATTGTTGAAAATGGAGCTG
GAACTGGATGAACAGTTTATCCCCCACTTTCATCCAATATTGCCCATGGTGGAAGTTCAGTAGATCTAGCAATTTTTTCT
CTTCATTTAGCTGGAATTTCCTCAATTTTAGGTGCTATTAATTTTATTACAACAATTATTAATATACGAGTTAATGGATT
ATCATTTGATCAAATACCTTTATTTGTTTGATCTGTTGGTATTACAGCCCTATTATTATTACTTTCATTACCTGTTTTAG
CAGGAGCTATTACTATATTATTAACTGATCGAAATTTAAATACATCATTTTTTGATCCTGCTGGTGGAGGGGATCCAATT
CTTTATCAACATTTATTTTGATTTTTT
>LSTEM446-18|Chilo_sp_AM15|AMSww06148|
AACTTTATATTTTATTTTTGGAATTTGGGCAGGAATAATTGGAACATCACTTAGACTTTTAATTCGAGCTGAATTAGGGA
CTCCAGGATCTTTAATTGGTGATGATCAAATTTATAATACTATTGTTACAGCTCATGCATTTATTATAATTTTTTTTATA
GTTATACCAATTATAATTGGTGGATTTGGAAATTGATTAGTGCCTTTAATATTGGGAGCTCCTGATATAGCTTTTCCACG
AATAAATAATATAAGATTTTGATTACTCCCCCCGTCATTAACTTTATTAATTTCTAGTAGAATTGTAGAAAATGGAGCTG
GAACAGGATGAACAGTTTACCCCCCACTCTCATCTAATATTGCCCATGCTGGAAGCTCAGTAGATTTGGCAATTTTTTCC

CTTCACTTAGCGGGTATTTCTTCTATCTTAGGTGCAATTAACTTTATTACAACAATCATTAATATACGAATTAATGGGTT
ATCATTTGATCAAATACCATTATTTGTTTGATCTGTTGGTATTACAGCTTTATTATTATTACTTTCATTACCAGTTTTAG
CAGGTGCTATTACCATGTTATTAACAGATCGAAATTTAAATACATCATTTTTTGATCCTGCTGGTGGGGGTGACCCCATT
CTTTATCAACACTTATTTTGATTTTTT
>LSTEM447-18|Chilo_sp_AM15|AMSww06149|
AACTTTATATTTTATTTTTGGAATTTGGGCAGGAATAATTGGAACATCACTTAGACTTTTAATTCGAGCTGAATTAGGGA
CTCCAGGATCTTTAATTGGTGATGATCAAATTTATAATACTATTGTTACAGCTCATGCATTTATTATAATTTTTTTTATA
GTTATACCAATTATAATTGGTGGATTTGGAAATTGATTAGTGCCTTTAATATTGGGAGCTCCTGATATAGCTTTTCCACG
AATAAATAATATAAGATTTTGATTACTCCCCCCGTCATTAACTTTATTAATTTCTAGTAGAATTGTAGAAAATGGAGCTG
GAACAGGATGAACAGTTTACCCCCCACTCTCATCTAATATTGCCCATGCTGGAAGCTCAGTAGATTTGGCAATTTTTTCC
CTTCACTTAGCGGGTATTTCTTCTATCTTAGGTGCAATTAACTTTATTACAACAATCATTAATATACGAATTAATGGGTT
ATCATTTGATCAAATACCATTATTTGTTTGATCTGTTGGTATTACAGCTTTATTATTATTACTTTCATTACCAGTTTTAG
CAGGTGCTATTACCATGTTATTAACAGATCGAAATTTAAATACATCATTTTTTGATCCTGCTGGTGGGGGTGACCCCATT
CTTTATCAACACTTATTTTGATTTTTT
>LSTEM448-18|Chilo_sp_AM15|AMSww06150|
AACTTTATATTTTATTTTTGGAATTTGGGCAGGAATAATTGGAACATCACTTAGACTTTTAATTCGAGCTGAATTAGGGA
CTCCAGGATCTTTAATTGGTGATGATCAAATTTATAATACTATTGTTACAGCTCATGCATTTATTATAATTTTTTTTATA
GTTATACCAATTATAATTGGTGGATTTGGAAATTGATTAGTGCCTTTAATATTGGGAGCTCCTGATATAGCTTTTCCACG
AATAAATAATATAAGATTTTGATTACTCCCCCCGTCATTAACTTTATTAATTTCTAGTAGAATTGTAGAAAATGGAGCTG
GAACAGGATGAACAGTTTACCCCCCACTCTCATCTAATATTGCCCATGCTGGAAGCTCAGTAGATTTGGCAATTTTTTCC
CTTCACTTAGCGGGTATTTCTTCTATCTTAGGTGCAATTAACTTTATTACAACAATCATTAATATACGAATTAATGGGTT
ATCATTTGATCAAATACCATTATTTGTTTGATCTGTTGGTATTACAGCTTTATTATTATTACTTTCATTACCAGTTTTAG
CAGGTGCTATTACCATGTTATTAACAGATCGAAATTTAAATACATCATTTTTTGATCCTGCTGGTG--------------
----
>LSTEM449-18|Chilo_diffusilineus|AMSww06151|
AACATTATATTTTATTTTTGGTATTTGAGCTGGAATAATTGGAACTTCCCTTAGTCTCTTAATTCGTGCTGAATTAGGTA
CACCTGGATCATTAATTGGAGATGATCAAATTTATAATACTATTGTTACAGCACATGCCTTTATTATAATTTTCTTTATA
GTTATACCTATTATAATTGGTGGATTTGGAAATTGATTAGTTCCACTAATATTAGGAGCCCCAGATATAGCTTTTCCACG
AATAAATAATATAAGATTTTGATTATTACCTCCTTCATTAACTTTATTAATTTCTAGAAGAATTGTTGAAAATGGAGCAG
GAACAGGATGAACAGTATACCCCCCACTTTCATCTAATATTGCACATGCTGGAAGTTCAGTAGATTTAGCAATTTTTTCC
TTACATTTAGCAGGTATTTCATCAATTTTAGGAGCTATTAACTTTATTACAACTATTATTAATATACGAATTAATGGATT
ATCCTTTGATCAAATACCATTATTTGTTTGATCTGTTGGTATTACAGCTTTATTATTATTACTTTCTTTACCTGTTTTAG
CTGGTGCTATTACTATACTATTAACAGATCGAAATTTAAATACATCTTTCTTTGATCCCGCTGGAGGAGGGGATCCAATT
CTTTATCAACATTTATTTTGATTTTTT
>LSTEM450-18|Chilo_diffusilineus|AMSww06152|
AACATTATATTTTATTTTTGGTATTTGAGCTGGAATAATTGGAACTTCCCTTAGTCTCTTAATTCGTGCTGAATTAGGTA
CACCTGGATCATTAATTGGAGATGATCAAATTTATAATACTATTGTTACAGCACATGCCTTTATTATAATTTTCTTTATA
GTTATACCTATTATAATTGGTGGATTTGGAAATTGATTAGTTCCACTAATATTAGGAGCCCCAGATATAGCTTTTCCACG
AATAAATAATATAAGATTTTGATTATTACCTCCTTCATTAACTTTATTAATTTCTAGAAGAATTGTTGAAAATGGAGCAG
GAACAGGATGAACAGTATACCCCCCACTTTCATCTAATATTGCACATGCTGGAAGTTCAGTAGATTTAGCAATTTTTTCC
TTACATTTAGCAGGTATTTCATCAATTTTAGGAGCTATTAACTTTATTACAACTATTATTAATATACGAATTAATGGATT
ATCCTTTGATCAAATACCATTATTTGTTTGATCTGTTGGTATTACAGCTTTATTATTATTACTTTCTTTACCTGTTTTAG
CTGGTGCTATTACTATACTATTAACAGATCGAAATTTAAATACATCTTTCTTTGATCCCGCTGGAGGAGGGGATCCAATT
CTTTATCAACATTTATTTTGATTTTTT
>LSTEM451-18|Chilo_sp_AM15|AMSww06154|
---------------------------------------TGGAACATCASTTAGACTTTTAATTCGAGCTGAATTAGGAA
CTCCAGGATCTTTAATTGGTGATGATCAAATTTATAATACTATTGTTACAGCTCATGCATTTATTATAATTTTTTTTATA
GTTATACCAATTATAATTGGTGGATTTGGAAATTGATTAGTGCCTTTAATATTGGGAGCTCCTGATATAGCTTTTCCACG
AATAAATAATATAAGATTTTGATTACTCCCCCCATCATTAACTTTATTAATTTCTAGTAGAATTGTAGAAAATGGAGCTG
GAACAGGATGAACAGTTTACCCCCCACTCTCATCTAATATTGCCCATGCTGGAAGTTCAGTAGATTTGGCAATTTTTTCC
CTTCACTTAGCAGGTATTTCTTCTATCTTAGGTGCAATTAACTTTATTACAACAATCATTAATATACGAATTAATGGATT
ATCATTTGATCAAATACCATTATTTGTTTGATCTGTTGGTATTACAGCTTTATTATTATTACTTTCATTACCAGTTTTAG
CGGGTGCTATTACTATATTATTAACAGATCGAAATTTAAATACATCATTTTTTGATCCTGCTGGTGGGGGTGACCCCATT
CTTTATCAACACTTATTTTGATTTTTT
>LSTEM452-18|Chilo_sp_AM15|AMSww06155|
AACTTTATATTTTATTTTTGGAATTTGAGCAGGAATAATTGGAACATCACTTAGACTTTTAATTCGAGCTGAATTAGGGA
CTCCAGGATCTTTAATTGGTGATGATCAAATTTATAATACTATTGTTACAGCTCATGCATTTATTATAATTTTTTTTATA

GTTATACCAATTATAATTGGTGGATTTGGAAATTGATTAGTGCCTTTAATATTGGGAGCTCCTGATATAGCTTTTCCACG
AATAAATAATATAAGATTTTGATTACTCCCCCCGTCATTAACTTTATTAATTTCTAGTAGAATTGTAGAAAATGGAGCTG
GAACAGGATGAACAGTTTACCCCCCACTCTCATCTAATATTGCCCATGCTGGAAGTTCAGTAGATTTGGCAATTTTTTCC
CTTCACTTAGCGGGTATTTCTTCTATCTTAGGTGCAATTAACTTTATTACAACAATCATTAATATACGAATTAATGGATT
ATCATTTGATCAAATACCATTATTTGTTTGATCTGTTGGTATTACAGCTTTATTGTTATTACTTTCATTACCAGTTTTAG
CAGGTGCTATTACTATGTTATTAACAGATCGAAATTTAAATACATCATTTTTTGATCCTGCTGGTGGGGGTGACCCCATT
CTTTATCAACACTTATTTTGATTTTTT
>LSTEM453-18|Chilo_diffusilineus|AMSww06156|
AACATTATATTTTATTTTTGGTATTTGAGCTGGAATAATTGGAACTTCCCTTAGTCTCTTAATTCGTGCTGAATTAGGTA
CACCTGGATCATTAATTGGAGATGATCAAATTTATAATACTATTGTTACAGCACATGCCTTTATTATAATTTTCTTTATA
GTTATACCTATTATAATTGGTGGATTTGGAAATTGATTAGTTCCACTAATATTAGGAGCCCCAGATATAGCTTTTCCACG
AATAAATAATATAAGATTTTGATTATTACCTCCTTCATTAACTTTATTAATTTCTAGAAGAATTGTTGAAAATGGAGCAG
GAACAGGATGAACAGTATACCCCCCACTTTCATCTAATATTGCACATGCTGGAAGTTCAGTAGATTTAGCAATTTTTTCC
TTACATTTAGCAGGTATTTCATCAATTTTAGGAGCTATTAACTTTATTACAACTATTATTAATATACGAATTAATGGATT
ATCCTTTGATCAAATACCATTATTTGTTTGATCTGTTGGTATTACAGCTTTATTATTATTACTTTCTTTACCTGTTTTAG
CTGGTGCTATTACTATACTATTAACAGATCGAAATTTAAATACATCTTTCTTTGATCCCGCTGGAGGAGGGGATCCAATT
CTTTATCAACATTTATTTTGATTTTTT
>LSTEM454-18|Chilo_sp_AM15|AMSww06157|
AACATTATATTTTATTTTTGGAATTTGAGCAGGAATAATTGGAACATCACTTAGACTTTTAATTCGAGCTGAATTAGGGA
CTCCAGGATCTTTAATTGGTGATGATCAAATTTATAATACTATTGTTACAGCTCATGCATTTATTATAATTTTTTTTATA
GTTATACCAATTATAATTGGTGGATTTGGAAATTGATTAGTGCCTTTAATATTGGGAGCTCCTGATATAGCTTTTCCACG
AATAAATAATATAAGATTTTGATTACTCCCCCCGTCATTAACTTTATTAATTTCTAGWAGAATTGTAGAAAATGGAGCTG
GAACAGGATGAACAGTTTACCCCCCACTCTCATCTAATATTGCCCATGCTGGAAGCTCAGTAGATTTGGCAATTTTTTCC
CTTCACTTAGCGGGTATTTCTTCTATCTTAGGAGCAATTAACTTTATTACAACAATCATTAATATACGAATTAATGGGTT
ATCATTTGATCAAATACCATTATTTGTTTGATCTGTTGGTATTACAGCTTTATTATTATTACTTTCATTACCAGTTTTAG
CAGGTGCTATTACCATGTTATTAACAGATCGAAATTTAAATACATCATTCTTTGATCCTGCTGGAGGAGGAGACCCCATT
CTTTATCAACACTTATTTTGATTTTTT
>LSTEM455-18|Chilo_diffusilineus|AMSww06158|
AACATTATATTTTATTTTTGGTATTTGAGCTGGAATAATTGGAACTTCCCTTAGTCTCTTAATTCGTGCTGAATTAGGTA
CACCTGGATCATTAATTGGAGATGATCAAATTTATAATACTATTGTTACAGCACATGCCTTTATTATAATTTTCTTTATA
GTTATACCTATTATAATTGGTGGATTTGGAAATTGATTAGTTCCACTAATATTAGGAGCCCCAGATATAGCTTTTCCACG
AATAAATAATATAAGATTTTGATTATTACCTCCTTCATTAACTTTATTAATTTCTAGAAGAATTGTTGAAAATGGAGCAG
GAACAGGATGAACAGTATACCCCCCACTTTCATCTAATATTGCACATGCTGGAAGTTCAGTAGATTTAGCAATTTTTTCC
TTACATTTAGCAGGTATTTCATCAATTTTAGGAGCTATTAACTTTATTACAACTATTATTAATATACGAATTAATGGATT
ATCCTTTGATCAAATACCATTATTTGTTTGATCTGTTGGTATTACAGCTTTATTATTATTACTTTCTTTACCTGTTTTAG
CTGGTGCTATTACTATACTATTAACAGATCGAAATTTAAATACATCTTTCTTTGATCCCGCTGGAGGAGGGGATCCAATT
CTTTATCAACATTTATTTTGATTTTTT
>LSTEM456-18|Chilo_orichalcociliellus|AMSww06159|
AACTTTATATTTTATTTTTGGAATTTGAGCAGGAATAATTGGAACATCACTTAGACTCCTAATTCGAGCTGAATTAGGAA
CCCCTGGATCTTTAATTGGTGATGATCAAATTTATAATACTATTGTTACAGCTCATGCATTTATTATAATTTTTTTTATA
GTTATACCAATTATAATTGGAGGATTTGGAAATTGATTAGTACCTTTAATGTTAGGAGCTCCTGATATAGCCTTCCCACG
AATAAATAATATAAGATTTTGATTACTACCCCCATCACTAACTTTATTAATTTCTAGAAGAATTGTTGAAAATGGAGCTG
GAACTGGGTGAACAGTTTATCCCCCACTTTCATCCAATATTGCCCATGGTGGGAGTTCAGTAGATCTAGCAATTTTTTCT
CTTCATTTAGCTGGAATTTCCTCAATTTTAGGTGCTATTAATTTTATTACAACAATCATTAATATACGAATTAATGGATT
ATCATTTGATCAAATACCTTTATTTGTTTGATCTGTTGGTATTACAGCCTTATTATTATTACTTTCATTACCTGTTTTAG
CAGGAGCTATTACTATATTATTAACTGATCGAAATTTAAATACATCATTTTTTGATCCTGCTGGTGG-------------
----
>LSTEM457-18|Chilo_orichalcociliellus|AMSww06160|
AACTTTATATTTTATTTTTGGAATTTGAGCAGGAATAATTGGAACATCACTTAGACTTCTAATTCGAGCTGAATTAGGAA
CCCCTGGATCCCTAATTGGTGACGATCAAATTTATAATACTATTGTTACAGCTCATGCATTTATTATAATTTTTTTTATA
GTTATACCGATTATAATTGGAGGATTTGGAAATTGATTAGTACCTTTAATATTAGGAGCTCCTGATATAGCCTTTCCACG
AATAAATAATATAAGATTTTGATTACTACCCCCATCATTAACTTTATTAATTTCTAGAAGAATTGTTGAAAATGGAGCTG
GAACTGGATGAACAGTTTATCCCCCACTTTCATCCAATATTGCCCATGGTGGAAGTTCAGTAGATCTAGCAATTTTTTCT
CTTCATTTAGCTGGAATTTCCTCAATTTTAGGTGCTATTAATTTTATTACAACAATTATTAATATACGAGTTAATGGATT
ATCATTTGATCAAATACCTTTATTTGTTTGATCTGTTGGTATTACAGCCTTATTATTATTACTTTCATTACCTGTTTTAG
CAGGAGCTATTACTATATTATTAACTGATCGAAATTTAAATACATCATTTTTTGATCCTGCTGGTGGAGGGGATCCAATT
CTTTATCAACATTTATTTTGATTTTTT

>LSTEM458-18|Chilo_sp_AM16|AMSww06161|
AACTTTATACTTTATTTTTGGAATTTGAGCAGGAATAATTGGAACATCACTTAGACTTTTAATTCGAGCTGAATTAGGAA
CTCCAGGATCTTTAATTGGTGATGATCAAATTTACAATACTATTGTTACAGCTCACGCATTTATTATAATTTTTTTTATA
GTTATACCAATTATAATTGGAGGATTTGGAAATTGATTGGTACCTCTAATGTTAGGAGCTCCTGATATAGCTTTTCCACG
AATAAATAATATAAGATTTTGATTACTCCCCCCATCACTAACTTTACTAATTTCTAGAAGAATTGTAGAAACTGGAGCCG
GAACAGGATGAACAGTTTACCCCCCACTATCATCTAATATCGCACATGCTGGAAGTTCAGTAGATTTAGCAATTTTTTCC
CTCCATTTAGCTGGGATTTCTTCTATTTTAGGAGCTATTAATTTTATTACAACAATTATTAATATACGAATTAATGGATT
ATCATTTGATCAAATACCATTATTTGTTTGATCAGTTGGTATTACAGCTTTATTATTATTACTTTCTTTACCTGTTTTAG
CAGGTGCTATTACTATATTATTAACAGATCGAAATCTAAATACATCATTTTTTGATCCAGCTGGAGGAGGTGATCCAATT
CTTTATCAACACTTATTTTGATTTTTT
>LSTEM459-18|Chilo_sp_AM16|AMSww06162|
--GAACTTCTCTTAGACTTTTAATTCGAGCTGAATTAGGAA
CTCCAGGATCTTTAATTGGTGATGATCAAATTTACAATACTATTGTTACAGCTCACGCATTTATTATAATTTTTTTTATA
GTTATACCAATTATAATTGGAGGATTTGGAAATTGATTGGTACCTCTAATGTTAGGGGCTCCTGATATAGCTTTTCCACG
AATAAATAATATAAGATTTTGATTACTCCCCCCATCACTAACTCTACTAATTTCTAGAAGAATTGTAGAAACTGGAGCCG
GAACAGGATGAACAGTTTACCCCCCACTATCATCTAATATCGCACATGCTGGAAGTTCAGTAGATTTAGCAATTTTTTCC
CTCCATTTAGCTGGAATTTCTTCTATTTTAGGAGCTATTAATTTTATTACAACAATTATTAATATACGAATTAATGGATT
ATCATTTGATCAAATACCATTATTTGTTTGATCAGTTGGTATTACAGCTTTATTATTATTACTTTCTTTACCTGTTTTAG
CAGGTGCTATTACTATATTATTAACAGATCGAAATCTAAATACATCATTTTTTGATCCAGCTGGAGGAGGTGATCCAATT
CTTTATCAACACTTATTTTGATTTTTT
>LSTEM460-18|Chilo_sp_AM16|AMSww06163|
AACTTTATACTTTATTTTTGGAATTTGAGCAGGAATAATTGGAACATCACTTAGACTTTTAATTCGAGCTGAATTAGGAA
CTCCAGGATCTTTAATTGGTGATGATCAAATTTACAATACTATTGTTACAGCTCACGCATTTATTATAATTTTTTTTATA
GTTATACCAATTATAATTGGAGGATTTGGAAATTGATTGGTACCTCTAATGTTAGGAGCTCCTGATATAGCTTTTCCACG
AATAAATAATATAAGATTTTGATTACTCCCCCCATCACTAACTTTACTAATTTCTAGAAGAATTGTAGAAACTGGAGCCG
GAACAGGATGAACAGTTTACCCCCCACTATCATCTAATATCGCACATGCTGGAAGTTCAGTAGATTTAGCAATTTTTTCC
CTCCATTTAGCTGGGATTTCTTCTATTTTAGGAGCTATTAATTTTATTACAACAATTATTAATATACGAATTAATGGATT
ATCATTTGATCAAATACCATTATTTGTTTGATCAGTTGGTATTACAGCTTTATTATTATTACTTTCTTTACCTGTTTTAG
CAGGTGCTATTACTATATTATTAACAGATCGAAATCTAAATACATCATTTTTTGATCCAGCTGGAGGAGGTGATCCAATT
CTTTATCAACACTTATTTTGATTTTTT
>LSTEM461-18|Chilo_sp_AM08|AMSww06164|
AACATTATATTTTATTTTTGGAATTTGAGCAGGAATAGTTGGAACTTCACTTAGTCTTTTAATTCGTGCTGAATTAGGAA
CACCAGGATCTTTAATTGGGGATGATCAAATTTATAACACTATTGTTACAGCACATGCTTTTATTATAATTTTTTTTATA
GTAATACCCATTATAATTGGTGGATTTGGTAATTGATTAGTTCCATTAATATTGGGTGCACCAGATATAGCTTTTCCACG
AATAAATAATATAAGATTTTGATTATTACCCCCCTCATTAACTTTACTAATTTCTAGAAGAATTGTTGAAAACGGAGCAG
GAACAGGATGAACAGTATACCCCCCACTTTCATCAAATATTGCCCATGCTGGAAGTTCAGTAGATTTAGCAATTTTTTCT
TTACATTTAGCGGGAATTTCTTCAATTTTAGGAGCTATTAATTTTATTACAACTATTATTAATATGCGAATTAATGGATT
ATCTTTTGATCAAATACCATTATTTGTATGATCTGTTGGTATTACAGCTTTATTATTATTACTTTCTTTACCTGTTTTAG
CAGGAGCTATTACTATATTATTAACAGATCGAAATTTAAATACATCTTTTTTTGATCCCGCAGGAGGTGGT---------
----
>LSTEM462-18|Chilo_sp_AM08|AMSww06165|
AACATTATATTTTATTTTTGGAATTTGAGCAGGAATAGTTGGAACTTCACTTAGTCTTTTAATTCGTGCTGAATTAGGAA
CACCAGGATCTTTAATTGGAGATGATCAAATTTATAACACTATTGTTACAGCACATGCTTTTATTATAATTTTTTTTATA
GTAATACCCATTATAATTGGTGGATTTGGTAATTGATTAGTTCCATTAATATTAGGTGCACCAGATATAGCTTTCCCACG
AATAAATAATATAAGATTTTGATTATTACCACCCTCATTAACTTTACTAATTTCTAGAAGAATTGTTGAAAACGGAGCAG
GAACAGGATGAACAGTGTACCCCCCACTTTCATCAAATATTGCCCATGCTGGAAGTTCAGTAGATTTAGCAATTTTTTCT
TTACATTTAGCAGGAATTTCTTCAATTTTAGGAGCTATTAATTTTATTACAACTATTATTAATATACGAATTAATGGATT
ATCTTTTGATCAAATACCATTATTTGTGTGATCTGTTGGTATTACAGCTTTATTATTATTACTTTCTTTACCTGTTTTAG
CAGGAGCTATTACTATATTATTAACAGATCGAAATTTAAATACATCTTTTTTTGACCCTGCAGGAGGGGGAGACCCTATT
CTTTACCAACATTTATTTTGATTTTTT
>LSTEM463-18|Chilo_sacchariphagus|AMSww06168|
AACTTTATATTTTATTTTTGGAATTTGAGCTGGAATAATTGGAACATCCCTTAGACTTTTAATTCGAGCTGAATTAGGAA
ATCCAGGTTCATTAATTGGAGATGATCAAATTTATAATACTATTGTTACAGCTCATGCATTTATTATAATTTTTTTTATA
GTAATACCAATTATAATCGGAGGATTTGGAAATTGATTAGTTCCATTAATATTAGGAGCTCCTGATATAGCCTTTCCTCG
TTTAAATAATATAAGATTTTGATTATTACCCCCTTCTTTAACTCTTTTAATTTCTAGAAGAATTGTTGAAAATGGAGCAG
GAACTGGATGAACAGTCTACCCCCCCCTATCTTCCAATATTTCACATGCTGGAAGTTCAGTAGATTTAGCCATCTTTTCT
CTTCATTTAGCAGGAATTTCTTCAATTTTAGGAGCTATTAATTTTATTACTACAATTATTAATATACGAATTAATGGATT

ATTATTTGATCAAATACCATTATTTGTTTGATCTGTTGGTATTACAGCATTACTTCTTCTTCTCTCTTTACCAGTATTAG
CAGGTGCTATTACCATACTATTAACTGACCGAAATTTAAATACATCTTTTTTGATCCAGCTGGAGGAGGTGATCCAATT
TTATATCAACATTTATTTTGATTTTTT
>LSTEM464-18|Scirpophaga_excerptalis|AMSww06169|
AACATTATATTTTATTTTTGGAATTTGAGCTGGTATAGTGGGAACTTCACTTAGTTTATTAATTCGAGCTGAATTAGGTA
CTCCCGGATCATTAATCGGAGATGATCAAATCTATAATACTATTGTAACTGCTCATGCTTTTATTATAATTTTTTTTATA
GTTATACCTATTATAATTGGGGGATTTGGAAACTGATTGGTACCTTTAATATTAGGAGCTCCAGATATAGCTTTCCCCCG
AATAAATAATATAAGTTTTTGATTATTACCCCCYTCTTTAACCCTCTTAATCTCGAGAAGAATTGTTGAAAATGGAGCTG
GAACAGGATGAACTGTCTACCCTCCCCTATCCTCTAATATTGCCCAKGGTGGAACTTCTGTAGATTTAGCCATTTTTTCA
TTACATTTAGCTGGAATTTCCTCTATTCTAGGAGCTATTAACTTCATCACAACTATTATTAATATACGAATTAATGGATT
ATCTTTTGATCAAATACCTTTATTCGTGTGAGCAGTTGGTATTACCGCTCTTCTCCTTCTTCTCTCATTACCTGTATTAG
CGGGAGCTATTACTATATTATTAACAGATCGAAACTTAAATACCTCTTTCTTTGATCCAGCAGGAGG-------------
----
>LSTEM465-18|Chilo|AMSww06170|

----------TTAATTGGAGATGATCAGATTTACAATACTATTGTTACAGCTCATGCATTTATTATAATTTTTTTTATA
GTTATACCAATTATAATTGGAGGCTTTGGTAATTGATTAGTACCATTAATACTAGGGGCTCCTGATATAGCCTTCCCTCG
AATAAATAATATAAGATTTTGATTATTGCCCCCATCATTAACATTATTAATTTCTAGAAGAATTGTAGAAAATGGAGCTG
GAACAGGATGAACGGTTTACCCCCCCCTTTCATCAAATATTGCCCATGGAGGAAGTTCTGTAGATTTAGCTATTTTTTCT
CTTCATTTAGCTGGTATTTCCTCAATTTTAGGAGCTATTAATTTTATTACAACAATTATTAATATACGAATTAATAAACT
ATCATTTGATCAATTACCATTATTTGTTTGATCTGTTGGTATTACAGCTTTATTATTACTACTTTCATTGCCGGTACTAG
CTGGAGCTATTACTATACTTTTAACTGATCGAAACTTAAATACATCTTTTTTGACCCTGCTGGAGGAGGAGACCCAATT
CTTTATCAACATTTATTTTGATTTTTT
>LSTEM466-18|Scirpophaga_excerptalis|AMSww06171|
AACATTATATTTTATTTTTGGAATTTGAGCTGGTATAGTAGGAACTTCACTTAGTTTATTAATTCGAGCTGAATTAGGTA
CTCCTGGATCACTAATTGGAGATGATCAAATCTATAATACTATCGTAACTGCTCATGCTTTTATTATAATTTTTTTTATA
GTTATACCTATTATAATTGGAGGATTTGGAAACTGATTAGTACCTTTAATATTAGGAGCCCCAGATATAGCTTTCCCTCG
AATAAATAATATAAGTTTTTGATTATTACCCCCTTCTTTAACTCTCTTAATCTCGAGAAGAATTGTTGAAAATGGAGCTG
GAACAGGATGAACTGTATACCCTCCTCTATCCTCTAATATTGCTCACGGTGGAACTTCTGTAGATYTAGCCATTTTTTCA
TTACATTTAGCTGGAATTTCCTCTATTCTAGGAGCTATTAACTTCATCACAACTATTATTAATATACGAATTAATGGATT
ATCTTTTGATCAAATACCTTTATTTGTATGAGCAGTTGGTATTACCGCTCTTCTCCTTCTTCTCTCACTACCTGTATTAG
CAGGAGCTATTACTATATTATTAACAGATCGAAACTTAAATACTTCTTTCTTTGATCCAGCAGGAGGTGGAGATCCAATC
CTTTATCAACATTTATTTTGATTTTTT
>LSTEM467-18|Chilo_orichalcociliellus|AMSww06172|
AACTTTATATTTTATTTTTGGAATTTGAGCAGGAATAATTGGAACATCACTTAGACTCCTAATTCGAGCTGAATTAGGAA
CCCCTGGATCTTTAATTGGTGATGATCAAATTTATAATACTATTGTTACAGCTCATGCATTTATTATAATTTTTTTTATA
GTTATACCAATTATAATTGGAGGATTTGGAAATTGATTAGTACCTTTAATGTTAGGAGCTCCTGATATAGCCTTCCCACG
AATAAATAATATAAGATTTTGATTACTACCCCCATCACTAACTTTATTAATTTCTAGAAGAATTGTTGAAAATGGAGCTG
GAACTGGATGAACAGTTTATCCCCCACTTTCATCCAATATTGCCCATGGTGGGAGTTCAGTAGATCTAGCAATTTTTTCT
CTTCATTTAGCTGGAATTTCCTCAATTTTAGGTGCTATTAATTTTATTACAACAATCATTAATATACGAATTAATGGATT
ATCATTTGATCAAATACCTTTATTTGTTTGATCTGTTGGTATTACAGCCTTATTATTATTACTTTCATTACCTGTTTTAG
CAGGAGCTATTACTATATTATTAACTGATCGAAATTTAAATACATCATTTTTTGATCCTGCTGG----------------
----
>LSTEM468-18|Eldana_saccharina|AMSww06173|
GACATTATACTTTATTTTTGGTATTTGATCAGGAATAGTAGGAACTTCTCTTAGATTACTAATTCGAGCTGAATTAGGAA
ATCCAGGATCTTTAATTGGAGATGACCAAATTTATAATACTATTGTTACAGGTCATGCTTTTATTATAATTTTTTTTATA
GTTATACCTATTATAATTGGAGGATTTGGTAATTGACTCGTACCTCTAATACTCGGAGCCCCCGATATAGCTTTCCCCCG
TATAAATAATATAAGTTTTTGACTATTACCTCCTTCTCTTTCTCTATTAATTTTTAGAAGAATTGTTGAAAACGGGGCAG
GAACAGGATGAACAGTCTATCCCCCATTATCTTCAAATATCGCTCATAGAGGAAGTTCTGTAGATTTAGCTATTTTTTCT
CTTCATTTAGCTGGAATTTCATCAATTTTAGGAGCTATTAATTTTATTACAACAGTAATTAATATAAAATTAAATGGTCT
ATCATTTGATCAAATACCTTTATTCGTATGAGCTGTAAGAATTACAGCTCTTCTTTTACTTTTATCTTTACCAGTTTTAG
CAGGTGCAATTACTATACTATTAACAGATCGTAATCTAAATACATCTTTTTTGATCCTGCCGGAGGAGGAGATCCTATT
CTTTACCAACATTTATTTTGATTTTTT
>LSTEM469-18|Chilo_orichalcociliellus|AMSww06174|
AACTTTATATTTTATTTTTGGAATGTGAGCAGGAATAACAGGAACATCASTTAGACTCCTAATTCGAGCTGAATTAGGAA
CCCCTGGATCTTTAATTGGTGATGATCAAATTTATAATACTATTGTTACAGCTCMTGCATTTATTATAATTTTTTTTATA
GTTATACCAATTATAAKTGGAGGATTTGGAAATTGATTAGTACCTTTAATGTYAGGAGCTCCTGATATAGCCTTCCCMCG

AATAAATAATATAASATTTTRATTACTACCCCCWTCMCTAACTTTATTAATTTCTAGAAGAATTGKTGAAAATGRASCTG
GAACTGGGTGAACAGTTTATCCCCCACTTTCATCCAATATTGCCCATGGKGGGAGTTCAGTAGATCTAGCAATTTTTTCT
CTTCATTTAGCTGGAATTTCCTCAATTTTAGGTGCTATTAATTTTATTACAACAATCATTAATATACGAATTAATGGATT
ATCATTTGATCAAATACCTTTATTTGTKWGATCTGTTGGTATTACMGCCTTATTATTATTACTTTCATTMCCTGTTWTAG
CAGGAGCTATTACTATATTATTAAGTGATCGAAATCTAAATACCTCTTTT------------------------------
----
>LSTEM470-18|Chilo_sp_AM16|AMSww06175|
AACTTTATACTTTATTTTTGGAATTTGAGCAGGAATAATTGGAACATCACTTAGACTTTTAATTCGAGCTGAATTAGGAA
CTCCAGGATCTTTAATTGGTGATGATCAAATTTACAATACTATTGTTACAGCTCACGCATTTATTATAATTTTTTTTATA
GTTATACCAATTATAATTGGAGGATTTGGAAATTGATTGGTACCTCTAATGTTAGGAGCTCCTGATATAGCTTTTCCACG
AATAAATAATATAAGATTTTGATTACTCCCCCCATCACTAACTTTACTAATTTCTAGAAGAATTGTAGAAACTGGAGCCG
GAACAGGATGAACAGTTTACCCCCCACTATCATCTAATATCGCACATGCTGGAAGTTCAGTAGATTTAGCAATTTTTTCC
CTCCATTTAGCTGGGATTTCTTCTATTTTAGGAGCTATTAATTTTATTACAACAATTATTAATATACGAATTAATGGATT
ATCATTTGATCAAATACCATTATTTGTTTGATCAGTTGGTATTACAGCTTTATTATTATTACTTTCTTTACCTGTTTTA-
----

>LSTEM471-18|Chilo_sp_AM16|AMSww06176|
AACTTTATACTTTATTTTTGGAATTTGAGCAGGAATAATTGGAACATCACTTAGACTTTTAATTCGAGCTGAATTAGGAA
CTCCAGGATCTTTAATTGGTGATGATCAAATTTACAATACTATTGTTACAGCTCACGCATTTATTATAATTTTTTTTATA
GTTATACCAATTATAATTGGAGGATTTGGAAATTGATTGGTACCTCTAATATTAGGAGCTCCTGATATAGCTTTTCCACG
AATAAATAATATAAGATTTTGATTACTCCCCCCATCACTAACTTTACTAATTTCTAGAAGAATTGTAGAAACTGGAGCCG
GAACAGGATGAACAGTTTACCCCCCACTATCATCTAATATCGCACATGCTGGAAGTTCAGTAGATTTAGCAATTTTTTCC
CTCCATTTAGCTGGGATTTCTTCTATTTTAGGAGCTATTAATTTTATTACAACAATTATTAATATACGAATTAATGGATT
ATCATTTGATCAAATACCATTATTTGTTTGATCAGTTGGTATTACAGCTTTATTATTATTACTTTCTTTACCTGTTTTA-
----

>LSTEM472-18|Chilo_sp_AM16|AMSww06177|
AACTTTATACTTTATTTTTGGAATTTGAGCAGGAATAATTGGAACATCACTTAGACTTTTAATTCGAGCTGAATTAGGAA
CTCCAGGATCTTTAATTGGTGATGATCAAATTTACAATACTATTGTTACAGCTCACGCATTTATTATAATTTTTTTTATA
GTTATACCAATTATAATTGGAGGATTTGGAAATTGATTGGTACCTCTAATATTAGGAGCTCCTGATATAGCTTTTCCACG
AATAAATAATATAAGATTTTGATTACTCCCCCCATCACTAACTTTACTAATTTCTAGAAGAATTGTAGAAACTGGAGCCG
GAACAGGATGAACAGTTTACCCCCCACTATCATCTAATATCGCACATGCTGGAAGTTCAGTAGATTTAGCAATTTTTTCC
CTCCATTTAGCTGGGATTTCTTCTATTTTAGGAGCTATTAATTTTATTACAACAATTATTAATATACGAATTAATGGATT
ATCATTTGATCAAATACCATTATTTGTTTGATCAGTTGGTATTACAGCTTTATTATTATTACTTTCTTTACCTGTTTTA-
----

>LSTEM473-18|Chilo_sp_AM16|AMSww06178|
AACTTTATACTTTATTTTTGGAATTTGAGCAGGAATAATTGGAACATCACTTAGACTTTTAATTCGAGCTGAATTAGGAA
CTCCAGGATCTTTAATTGGTGATGATCAAATTTACAATACTATTGTTACAGCTCACGCATTTATTATAATTTTTTTTATA
GTTATACCAATTATAATTGGAGGATTTGGAAATTGATTGGTACCTCTAATATTAGGAGCTCCTGATATAGCTTTTCCACG
AATAAATAATATAAGATTTTGATTACTCCCCCCATCACTAACTTTACTAATTTCTAGAAGAATTGTAGAAACTGGAGCCG
GAACAGGATGAACAGTTTACCCCCCACTATCATCTAATATCGCACATGCTGGAAGTTCAGTAGATTTAGCAATTTTTTCC
CTCCATTTAGCTGGGATTTCTTCTATTTTAGGAGCTATTAATTTTATTACAACAATTATTAATATACGAATTAATGGATT
ATCATTTGATCAAATACCATTATTTGTTTGATCAGTTGGTATTACAGCTTTATTATTATTACTTTCTTTACCTGTTTTAG
CAGGTGCTATTACTATATTATTAACAGATCGAAATCTAAATACATCATTTTTTGATCCAGCTGGAGG-------------
----
>LSTEM474-18|Chilo_sp_AM15|AMSww06179|
AACTTTATATTTTATTTTTGGAATTTGGGCAGGAATAATTGGAACATCACTTAGACTTTTAATTCGAGCTGAATTAGGGA
CTCCAGGATCTTTAATTGGTGATGATCAAATTTATAATACTATTGTTACAGCTCATGCATTTATTATAATTTTTTTTATA
GTTATACCAATTATAATTGGTGGATTTGGAAATTGATTAGTGCCTTTAATATTGGGAGCTCCTGATATAGCTTTTCCACG
AATAAATAATATAAGATTTTGATTACTCCCCCCGTCATTAACTTTATTAATTTCTAGTAGAATTGTAGAAAATGGAGCTG
GAACAGGATGAACAGTTTACCCCCCACTCTCATCTAATATTGCCCATGCTGGAAGCTCAGTAGATTTGGCAATTTTTTCC
CTTCACTTAGCGGGTATTTCTTCTATCTTAGGTGCAATTAACTTTATTACAACAATCATTAATATACGAATTAATGGGTT
ATCATTTGATCAAATACCATTATTTGTTTGATCTGTTGGTATTACAGCTTTATTATTATTACTTTCATTACCAGTTTTAG
CAGGTGCTATTACCATGTTATTAACAGATCGAAATTTAAATACATCATTTTTTGATCCTGCTGGTGGGGGTGACCCCATT
CTTTATCAACACTTATTTTGATTTTTT
>LSTEM475-18|Chilo|AMSww06180|

AACTTTATATTTTATTTTTGGAATTTGAGCAGGAATAATTGGAACATCACTTAGACTCCTAATTCGAGCTGAATTAGGAA
CCCCTGGATCTTTAATTGGTGATGATCAAATTTATAATACTATTGTTACAGCTCATGCATTTATTATAATTTTTTTTATA
GTTATACCAATTATAATTGGAGGATTTGGAAATTGATTAGTACCTTTAATGTTAGGAGCTCCTGATATAGCCTTCCCACG
AATAAATAATATAAGATTTTGATTACTACCCCCATCACTAACTTTATTAATTTCTAGAAGAATTGTTGAAAATGGAGCTG
GAACTGGGTGAACAGTTTATCCCCCACTTTCATCCAATATTGCCCATGGTGGGAGTTCAGTAGATCTAGCAATTTTTTCT
CTTCATTTAGCTGGAATTTCCTCAATTTTAGGTGCTATTAATTTTATTACAACAATCATTAATATACGAATTAATGGATT
ATCATTTGATCAAATACCTTTATTTGTTTGATCTGTTGGTATTACAGCCTTATTATTATTACTTTCATTACCTGTTTTAG
CAGGAGCTATTACTATATTATTAACTGATCGAAATTTAAATACATCATTTTTTGATCCTGGTGGTGGAGGAGATCCAATT
CTTTATCAACATTTATTTTGATTTTTT
>LSTEM476-18|Chilo_sp_AM11|AMSww06181|
---GGGACATCACTCAGACTTTAATTCGAGCTGAATTAGGGA
CTCCTGGATCCTTAATTGGTGATGATCAAATTTATAATACTATTGTCACAGCCCATGCATTTATTATAATTTTTTTTATA
GTTATACCAATTATAATTGGTGGATTTGGAAATTGATTAGTACCTCTAATATTGGGAGCTCCTGATATAGCTTTCCCACG
AATAAATAATATAAGATTTTGATTACTCCCCCCATCATTAACTTTATTAATTTCTAGTAGAATTGTAGAAAATGGAGCTG
GAACAGGATGAACAGTTTACCCCCCACTTTCATCTAATATTGCTCATGCTGGAAGTTCAGTAGATTTAGCAATTTTTTCC
CTTCATTTAGCTGGAATTTCTTCTATTTTAGGTGCAATTAATTTTATTACAACAATCATTAATATACGAATTAATAGATT
ATTATTTGATCAAATACCATTATTTATTTGATCTGTTGGTATTACAGCCTTATTATTATTACTTTCATTACCAGTTTTAG
CGGGTGCTATTACTATATTATTAACAGATCGAAATTTAAATACATCATTTTTTGATCCTGCTGGTGGAGGAGATCCAATT
CTTTACCAACATTTATTTTGATTTTTT
>LSTEM477-18|Eldana_saccharina|AMSww06182|
GACATTATACTTTATTTTTGGTATTTGATCAGGAATAGTAGGAACTTCTCTTAGATTACTAATTCGAGCTGAATTAGGAA
ATCCAGGATCTTTAATTGGAGATGATCAAATTTATAATACTATTGTTACAGGTCATGCTTTTATTATAATTTTTTTTATA
GTTATACCTATTATAATTGGAGGATTTGGTAATTGACTCGTACCTCTAATACTCGGAGCCCCCGATATAGCTTTCCCCCG
TATAAATAATATAAGTTTTTGACTATTACCTCCTTCTCTTTCTCTATTAATTTTTAGAAGAATTGTTGAAAATGGAGCAG
GAACAGGATGAACAGTTTATCCCCCATTATCTTCAAATATCGCTCATAGAGGAAGTTCTGTAGATTTAGCTATTTTTTCT
CTTCATTTAGCTGGAATTTCATCAATTTTAGGGGCCATTAATTTTATTACAACAGTAATTAATATAAAATTAAATGGCCT
ATCATTTGATCAAATACCTTTATTTGTATGAGCTGTAAGAATTACAGCTCTTCTTTTACTTTTATCTTTACCAGTTTTAG
CAGGTGCAATTACTATACTATTAACAGATCGTAATCTAAATACATCTTTTTTTGACCCTGCCGGAGGAGGAGATCCTATT
CTTTACCAACATTTATTTTGATTTTTT
>LSTEM478-18|Eldana_saccharina|AMSww06183|
GACATTATACTTTATTTTTGGTATTTGATCAGGAATAGTAGGAACTTCTCTTAGATTACTAATTCGAGCTGAATTAGGAA
ATCCAGGATCTTTAATTGGAGATGATCAAATTTATAATACTATTGTTACAGGTCATGCTTTTATTATAATTTTTTTTATA
GTTATACCTATTATAATTGGAGGATTTGGTAATTGACTCGTACCTCTAATACTCGGAGCCCCCGATATAGCTTTCCCCCG
TATAAATAATATAAGTTTTTGACTATTACCTCCTTCTCTTTCTCTATTAATTTTTAGAAGAATTGTTGAAAATGGAGCAG
GAACAGGATGAACAGTTTATCCCCCATTATCTTCAAATATCGCTCATAGAGGAAGTTCTGTAGATTTAGCTATTTTTTCT
CTTCATTTAGCTGGAATTTCATCAATTTTAGGGGCCATTAATTTTATTACAACAGTAATTAATATAAAATTAAATGGCCT
ATCATTTGATCAAATACCTTTATTTGTATGAGCTGTAAGAATTACAGCTCTTCTTTTACTTTTATCTTTACCAGTTTTAG
CAGGTGCAATTACTATACTATTAACAGATCGTAATCTAAATACATCTTTTTTTGACCCTGCCGGAGGAGGAGATCCTATT
CTTTACCAACATTTATTT---------
>LSTEM479-18|Eldana_saccharina|AMSww06184|
GACATTATACTTTATTTTTGGTATTTGATCAGGAATAGTAGGAACTTCTCTTAGATTACTAATTCGAGCTGAATTAGGAA
ATCCAGGATCTTTAATTGGAGATGATCAAATTTATAATACTATTGTTACAGGTCATGCTTTTATTATAATTTTTTTTATA
GTTATACCTATTATAATTGGAGGATTTGGTAATTGACTCGTACCTCTAATACTCGGAGCCCCCGATATAGCTTTCCCCCG
TATAAATAATATAAGTTTTTGACTATTACCTCCTTCTCTTTCTCTATTAATTTTTAGAAGAATTGTTGAAAATGGAGCAG
GAACAGGATGAACAGTTTATCCCCCATTATCTTCAAATATCGCTCATAGAGGAAGTTCTGTAGATTTAGCTATTTTTTCT
CTTCATTTAGCTGGAATTTCATCAATTTTAGGGGCCATTAATTTTATTACAACAGTAATTAATATAAAATTAAATGGCCT
ATCATTTGATCAAATACCTTTATTTGTATGAGCTGTAAGAATTACAGCTCTTCTTTTACTTTTATCTTTACCAGTTTTAG
CAGGTGCAATTACTATACTATTAACAGATCGTAATCTAAATACATCTTTTTTTGACCCTGCCGGAGGAGGAGATCCTATT
CTTTACCAACATTTATTTTGATTTTTT
>LSTEM480-18|Eldana_saccharina|AMSww06185|
----TTATACTTTATTTTTGGTATTTGATCAGGAATAGTAGGAACTTCTCTTAGATTACTAATTCGAGCTGAATTAGGAA
ATCCAGGATCTTTAATTGGAGATGATCAAATTTATAATACTATTGTTACAGGTCATGCTTTTATTATAATTTTTTTTATA
GTTATACCTATTATAATTGGAGGATTTGGTAATTGACTCGTACCTCTAATACTCGGAGCCCCCGATATAGCTTTCCCCCG
TATAAATAATATAAGTTTTTGACTATTACCTCCTTCTCTTTCTCTATTAATTTTTAGAAGAATTGTTGAAAATGGAGCAG
GAACAGGATGAACAGTTTATCCCCCATTATCTTCAAATATCGCTCATAGAGGAAGTTCTGTAGATTTAGCTATTTTTTCT
CTTCATTTAGCTGGAATTTCATCAATTTTAGGGGCCATTAATTTTATTACAACAGTAATTAATATAAAATTAAATGGCCT
ATCATTTGATCAAATACCTTTATTTGTATGAGCTGTAAGAATTACAGCTCTTCTTTTACTTTTATCTTTACCAGTTTTAG

CAGGTGCAATTACTATACTATTAACAGATCGTAATCTAAATACATCTTTTTTGACCCTGCCGGAGGAGGAGATCCTATT
CTTTACCAACATTTATTTTGATTTTT-
>LSTEM481-18|Eldana_saccharina|AMSww06186|
GACATTATACTTTATTTTTGGTATTTGATCAGGAATAGTAGGAACTTCTCTTAGATTACTAATTCGAGCTGAATTAGGAA
ATCCAGGATCTTTAATTGGAGATGATCAAATTTATAATACTATTGTTACAGGTCATGCTTTTATTATAATTTTTTTTATA
GTTATACCTATTATAATTGGAGGATTTGGTAATTGACTCGTACCTCTAATACTCGGAGCCCCCGATATAGCTTTCCCCCG
TATAAATAATATAAGTTTTTGACTATTACCTCCTTCTCTTTCTCTATTAATTTTTAGAAGAATTGTTGAAAATGGAGCAG
GAACAGGATGAACAGTTTATCCCCCATTATCTTCAAATATCGCTCATAGAGGAAGTTCTGTAGATTTAGCTATTTTTTCT
CTTCATTTAGCTGGAATTTCATCAATTTTAGGGGCCATTAATTTTATTACAACAGTAATTAATATAAAATTAAATGGCCT
ATCATTTGATCAAATACCTTTATTTGTATGAGCTGTAAGAATTACAGCTCTTCTTTTACTTTTATCTTTACCAGTTTTAG
CAGGTGCAATTACTATACTATTAACAGATCGTAATCTAAATACATCTTTTTTGACCCTGCCGGAGGWGGAGATCCTATT
CTTTACCAACATTTATTT---------
>LSTEM482-18|Eldana_saccharina|AMSww06187|
GACATTATACTTTATTTTTGGTATTTGATCAGGAATAGTAGGAACTTCTCTTAGATTACTAATTCGAGCTGAATTAGGAA
ATCCAGGATCTTTAATTGGAGATGATCAAATTTATAATACTATTGTTACAGGTCATGCTTTTATTATAATTTTTTTTATA
GTTATACCTATTATAATTGGAGGATTTGGTAATTGACTCGTACCTCTAATACTCGGAGCCCCCGATATAGCTTTCCCCCG
TATAAATAATATAAGTTTTTGACTATTACCTCCTTCTCTTTCTCTATTAATTTTTAGAAGAATTGTTGAAAATGGAGCAG
GAACAGGATGAACAGTTTATCCCCCATTATCTTCAAATATCGCTCATAGAGGAAGTTCTGTAGATTTAGCTATTTTTTCT
CTTCATTTAGCTGGAATTTCATCAATTTTAGGGGCCATTAATTTTATTACAACAGTAATTAATATAAAATTAAATGGCCT
ATCATTTGATCAAATACCTTTATTTGTATGAGCTGTAAGAATTACAGCTCTTCTTTTACTTTTATCTTTACCAGTTTTAG
CAGGTGCAATTACTATACTATTAACAGATCGTAATCTAAATACATCTTTTTTGACCCTGCCGGAGGAGGAGATCCTATT
CTTTACCAACATTTATTTTGATTTTTT
>LSTEM483-18|Eldana_saccharina|AMSww06188|
GACATTATACTTTATTTTTGGTATTTGATCAGGAATAGTAGGAACTTCTCTTAGATTACTAATTCGAGCTGAATTAGGAA
ATCCAGGATCTTTAATTGGAGATGATCAAATTTATAATACTATTGTTACAGGTCATGCTTTTATTATAATTTTTTTTATA
GTTATACCTATTATAATTGGAGGATTTGGTAATTGACTCGTACCTCTAATACTCGGAGCCCCCGATATAGCTTTCCCCCG
TATAAATAATATAAGTTTTTGACTATTACCTCCTTCTCTTTCTCTATTAATTTTTAGAAGAATTGTTGAAAATGGAGCAG
GAACAGGATGAACAGTTTATCCCCCATTATCTTCAAATATCGCTCATAGAGGAAGTTCTGTAGATTTAGCTATTTTTTCT
CTTCATTTAGCTGGAATTTCATCAATTTTAGGGGCCATTAATTTTATTACAACAGTAATTAATATAAAATTAAATGGCCT
ATCATTTGATCAAATACCTTTATTTGTATGAGCTGTAAGAATTACAGCTCTTCTTTTACTTTTATCTTTGCCAGTTTTAG
CAGGTGCAATTACTATACTATTAACAGATCGTAATCTAAATACATCTTTTTTGACCCTGCCGGAGGAGGAGATCCTATT
CTTTACCAACATTTATTTTGATTTTTT
>LSTEM484-18|Eldana_saccharina|AMSww06189|
-----TATACTTTATTTTTGGTATTTGATCAGGAATAGTAGGAACTTCTCTTAGATTACTAATTCGAGCTGAATTAGGAA
ATCCAGGATCTTTAATTGGAGATGATCAAATTTATAATACTATTGTTACAGGTCATGCTTTTATTATAATTTTTTTTATA
GTTATACCTATTATAATTGGAGGATTTGGTAATTGACTCGTACCTCTAATACTCGGAGCCCCCGATATAGCTTTCCCCCG
TATAAATAATATAAGTTTTTGACTATTACCTCCTTCTCTTTCTCTATTAATTTTTAGAAGAATTGTTGAAAATGGAGCAG
GAACAGGATGAACAGTTTATCCCCCATTATCTTCAAATATCGCTCATAGAGGAAGTTCTGTAGATTTAGCTATTTTTTCT
CTTCATTTAGCTGGAATTTCATCAATTTTAGGGGCCATTAATTTTATTACAACAGTAATTAATATAAAATTAAATGGCCT
ATCATTTGATCAAATACCTTTATTTGTATGAGCTGTAAGAATTACAGCTCTTCTTTTACTTTTATCTTTACCAGTTTTAG
CAGGTGCAATTACTATACTATTAACAGATCGTAATCTAAATACATCTTTTTTGACCCTGCCGGAGGAGGAGATCCTATT
CTTTACCAACATTTATTTTGATTTTTT
>LSTEM485-18|Eldana_saccharina|AMSww06190|
GACATTATACTTTATTTTTGGTATTTGATCAGGAATAGTAGGAACTTCTCTTAGATTACTAATTCGAGCTGAATTAGGAA
ATCCAGGATCTTTAATTGGAGATGATCAAATTTATAATACTATTGTTACAGGTCATGCTTTTATTATAATTTTTTTTATA
GTTATACCTATTATAATTGGAGGATTTGGTAATTGACTCGTACCTCTAATACTCGGAGCCCCCGATATAGCTTTCCCCCG
TATAAATAATATAAGTTTTTGACTATTACCTCCTTCTCTTTCTCTATTAATTTTTAGAAGAATTGTTGAAAATGGAGCAG
GAACAGGATGAACAGTTTATCCCCCATTATCTTCAAATATCGCTCATAGAGGAAGTTCTGTAGATTTAGCTATTTTTTCT
CTTCATTTAGCTGGAATTTCATCAATTTTAGGGGCCATTAATTTTATTACAACAGTAATTAATATAAAATTAAATGGCCT
ATCATTTGATCAAATACCTTTATTTGTATGAGCTGTAAGAATTACAGCTCTTCTTTTACTTTTATCTTTACCAGTTTTAG
CAGGTGCAATTACTATACTATTAACAGATCGTAATCTAAATACATCTTTTTTGACCCTGCCGGAGGAGGAGATCCTATT
CTTTACCAACATTTATTTTGATTTTTT
>LSTEM486-18|Sesamia_grisescens|AMSww06191|
AACATTATATTTTATTTTCGGAATTTGAGCTGGTATAGTTGGAACATCACTAAGATTATTAATTCGAGCCGAATTAGGGA
CCCCTGGTTCTTTAATTGGAGACGATCAAATTTATAATACTATTGTTACAGCTCATGCTTTCATTATAATTTTCTTTATA
GTTATACCAATTATAATTGGTGGATTTGGAAATTGACTCGTACCTTTAATATTAGGAGCCCCTGATATAGCATTTCCACG
AATAAATAATATAAGATTTTGATTATTACCCCCCTCTTTAACTTTACTAATTTCAAGTAGAATTGTAGAAAATGGGGCAG

GAACTGGGTGAACAGTGTACCCCCCACTTTCATCTAATATTGCCCATGGAGGAAGATCAGTGGACTTAGCTATTTTTTCC
CTTCATTTAGCAGGTATTTCATCTATTTTAGGAGCTATTAATTTTATCACAACAATTATTAATATACGATTAAATAACTT
ATCCTTTGATCAAATACCTTTATTTATTTGAGCTGTTGGAATTACTGCATTTTTATTATTATTATCTTTACCTGTTTTAG
CAGGAGCTATTACAATATTATTAACCGATCGAAACTTAAATACATCATTTTTCGATCCGGCAGGAG--------------
----
>LSTEM487-18|Sesamia_grisescens|AMSww06192|
AACATTATATTTTATTTTCGGAATTTGAGCTGGTATAGTTGGAACATCACTAAGATTATTAATTCGAGCCGAATTAGGGA
CCCCTGGTTCTTTAATTGGAGACGATCAAATTTATAATACTATTGTTACAGCTCATGCTTTCATTATAATTTTCTTTATA
GTTATACCAATTATAATTGGTGGATTTGGAAATTGACTCGTACCTTTAATATTAGGAGCCCCTGATATAGCATTTCCACG
AATAAATAATATAAGATTTTGATTATTACCCCCCTCTTTAACTTTACTAATTTCAAGTAGAATTGTAGAAAATGGGGCAG
GAACTGGGTGAACAGTGTACCCCCCACTTTCATCTAATATTGCCCATGGAGGAAGATCAGTGGACTTAGCTATTTTTTCC
CTTCATTTAGCAGGTATTTCATCTATTTTAGGAGCTATTAATTTTATCACAACAATTATTAATATACGATTAAATAACTT
ATCCTTTGATCAAATACCTTTATTTATTTGAGCTGTTGGAATTACTGCATTTTTATTATTATTATCTTTACCTGTTTTA-
----

>LSTEM488-18|Chilo_terrenellus|AMSww06193|
AACTTTATATTTTATTTTTGGTATTTGAGCTGGAATAATTGGAACATCTCTTAGAATTTTAATTCGCGCTGAATTAGGTA
CTCCAGGATCCTTAATTGGTGATGATCAAATTTATAATACTATTGTAACAGCTCATGCATTTATTATAATTTTTTTTATA
GTAATACCAATTATAATTGGAGGATTTGGAAATTGATTAGTGCCATTAATATTAGGTGCCCCAGATATAGCCTTCCCCCG
AATAAATAATATAAGATTTTGAATATTACCCCCCTCATTAACTTTATTAATTTCTAGAAGAATTGTTGAAAATGGAGCTG
GAACAGGATGAACAGTATACCCCCCACTTTCATCTAATATTGCTCATGCTGGAAGTTCAGTAGATTTAGCAATTTTTTCA
CTACATTTAGCCGGTATTTCTTCAATTTTAGGAGCTATTAATTTTATTACAACAATTATTAATATACGTATTAATAAATT
ATCATTTGATCAAATACCTTTATTTGTTTGATCTGTAGGTATTACAGCATTACTTTTATTACTTTCATTACCTGTTTTA-
----

>LSTEM489-18|Chilo_terrenellus|AMSww06194|
AACTTTATATTTTATTTTTGGTATTTGAGCTGGAATAATTGGAACATCTCTTAGAATTTTAATTCGCGCTGAATTAGGTA
CTCCAGGATCCTTAATTGGTGATGATCAAATTTATAATACTATTGTAACAGCTCATGCATTTATTATAATTTTTTTTATA
GTAATACCAATTATAATTGGAGGATTTGGAAATTGATTAGTGCCATTAATATTAGGTGCCCCAGATATGGCCTTCCCCCG
AATAAATAATATAAGATTTTGAATATTACCCCCCTCATTAACTTTATTAATTTCTAGAAGAATTGTTGAAAATGGAGCTG
GAACAGGATGAACAGTATACCCCCCACTTTCATCTAATATTGCTCATGCTGGAAGTTCAGTAGATTTAGCAATTTTTTCA
CTACATTTAGCCGGTATTTCTTCAATTTTAGGAGCTATTAATTTTATTACAACAATTATTAATATACGTATTAATAAATT
ATCATTTGATCAAATACCTTTATTTGTTTGATCTGTAGGTATTACAGCATTACTTTTATTACTTTCATTACCTGTTTTAG
CTGGAGCTATTACTATACTACTAACAGATCGGAATTTAAATACATCTTTTTTTGATCCTGCTGGAGGAGGAGATCCTATT
CTTTACCAACATTTATTTTGATTTTTT
>LSTEM490-18|Scirpophaga_excerptalis|AMSww06195|
-------------------------GAGCWGGTATAGTAGGAACTTCTCTTAGTTTACTAATTCGAGCTGAATTAGGAA
ATCCCGGATCTTTAATTGGAGATGATCAAATTTATAATACTATTGTTACTGGTCATGCTTTTATTATAATTTTTTTTATA
GTTATACCTATTATAATTGGAGGATTTGGAAATTGATTAGTACCTTTAATATTAGGAGCTCCHGATATAGCTTTCCCTCG
AATAAATAATATAAGWTTTTGATTATTACCTCCTTCTTTAACCCTACTAATYAGAAGAAGAATTGTTGAAAATGGAGCTG
GAACAGGATGAACTGTTTACCCACCCTTATCCTCCAATATTGCTCATGGTGGAACTTCTGTGGATTTAGCCATTTTTTCA
TTACATTTAGCTGGAATTTCCTCTATTCTAGGAGCTATTAATTTCATTACAACTATTGTTAATATACGAATTAATGGATT
ATCTTTTGATCAAATACCTTTATTCGTGTGAGCAGTTGGTATTACCGCT-------------------------------
----

>LSTEM491-18|Scirpophaga_excerptalis|AMSww06197|
AACATTATATTTTATTTTTGGAATTTGAGCTGGTATAGTAGGAACTTCACTTAGTTTATTAATTCGAGCTGAATTAGGTA
CTCCTGGGTCATTAATTGGAGATGATCAAATTTATAATACTATCGTAACTGCTCATGCTTTTATTATAATTTTTTTTATA
GTTATACCTATTATAATTGGAGGATTTGGAAACTGATTAGTACCTTTAATATTAGGAGCCCCAGATATAGCTTTCCCTCG
AATAAATAATATAAGTTTTTGATTAYTACCCCCYTCTTTAACYCTCTTAATCTCGAGAAWAATAGTWGAAAAAGGAGCTG
GAACAGGATGAACGGTCTACCCRCCYYYWTCYTCCAATATTGACCATGGTGGAACTTCTGTGGATTTAGCCATTTTTTCA
TTACATTTAGCTGGAATTTCCTCTATTCTAGGAGCTATTAACTTCATTACAACTATTATTAATATACGAATTAATGGATT
ATCTTTAGATCAAATACCTTTATTCGTGTGAGCAGTTGGTATTACCGCTCTTCTCCTTCTTCTCTCATTACCTGTATT--
----

>LSTEM492-18|Scirpophaga_excerptalis|AMSww06198|
AACATTATATTTTATTTTTGGAATTTGAGCTGGTATAGTRGGAACTTCACTTAGTTTATTAATTCGAGCTGAATTAGGTA

CTCCTGGGTCAYTAATTGGAGATGATCAAATCTATAATACTATCGTAACTGCTCATGCTTTTATTATAATTTTTTTTATA
GTTATACCTATTATAATTGGAGGATTTGGAAACTGATTAGTACCTTTAATATTAGGAGCCCCAGATATAGCTTTCCCTCG
AATAAATAATATAAGTTTTTGATTACTACCCCCCTCTTTAACTCTCTTAATCTCGAGAAGAATTGTTGAAAATGGAGCTG
GAACAGGATGAACTGTCTACCCACCCCTATCCTCCAATATTGCACATGGTGGAACTTCTGTGGATTTAGCCATCTTTTCA
TTACATTTAGCTGGAATTTCCTCTATTCTAGGAGCTATTAATTTCATTACAACTATTGTTAATATACGAATTAATGGATT
ATCTTTTGATCAAATACCTTTATTCGTGTGAGCAGTTGGTATTACCGCTCTCCTCCTTCTTCTCTCATTACCTGTATT--
----

>LSTEM493-18|Scirpophaga_excerptalis|AMSww06200|
GACATTATATTTTATTTTTGGAATTTGAGCTGGTATAGTGGGGACTTCACTTAGTTTACTAATCCGAGCCGAACTAGGTA
CTCCGGGATCATTAATCGGAGATGATCAAATCTATAATACTATTGTAACTGCTCACGCTTTTATTATAATTTTTTTTATG
GTTATGCCCATTATAATTGGGGGATTCGGAAACTGATTAGTGCCTTTAATATTGGGAGCCCCAGATATGGCCTTCCCCCG
AATAAATAATATAAGTTTTTGATTATTACCCCCTTCTTTAACCCTCTTAATCTCAAGAAGAGTCGTTGAAAATGGAGCTG
GAACAGGATGAACTGTTTATCCGCCCTTATCCTCCAATATTGCTCATGGTGGGACTTCTGTAGATTTAGCCATTTTTTCA
TTACATTTAGCTGGAATTTCTTCTATTCTAGGGGCTATTAACTTCATTACAACTATTATTAATATACGAATTAATGGACT
ATCTTTTGATCAAATACCTTTATTTGTATGAGCAGTTGGTATTACTGCCCTTCTTCTTCTTCTCTCACTACCTGTATTA-
----

>LSTEM494-18|Scirpophaga_excerptalis|AMSww06201|
GACATTATATTTTATTTTTGGAATTTGAGCTGGTATAGTGGGGACTTCACTTAGTTTACTAATTCGAGCCGAACTAGGTA
CTCCGGGATCATTAATCGGAGATGATCAAATCTATAATACTATTGTAACTGCTCACGCTTTTATTATAATTTTTTTTATG
GTTATACCCATTATAATTGGGGGATTCGGAAACTGATTAGTGCCTTTAATATTGGGGGCCCCAGATATGGCCTTCCCCCG
AATAAATAATATAAGTTTTTGATTATTACCCCCCTCTTTAACCCTCTTAATCTCAAGAAGAGTCGTTGAAAATGGAGCTG
GAACAGGATGAACTGTTTATCCGCCCTTATCCTCCAATATTGCTCATGGTGGGACTTCTGTAGATTTAGCCATTTTTTCA
TTACATTTAGCTGGAATTTCTTCTATTCTAGGGGCTATTAACTTCATTACAACTATTATTAATATACGAATTAATGGACT
ATCTTTTGATCAAATACCTTTATTTGTATGAGCAGTTGGTATTACTGCCCTTCTTCTTCTTCTCTCACTACCTGTATTAG
CGGGAGCTATTACTATTTTATTAACAGATCGAAATTTAAA--

>LSTEM495-18|Scirpophaga_excerptalis|AMSww06202|
GACATTATATTTTATTTTTGGAATTTGAGCTGGTATAGTGGGGACTTCACTTAGTTTACTAATTCGAGCCGAACTAGGTA
CTCCGGGATCATTAATCGGAGATGATCAAATCTATAATACTATTGTAACTGCTCACGCTTTTATTATAATTTTTTTTATG
GTTATACCCATTATAATTGGGGGATTCGGAAACTGATTAGTGCCTTTAATATTGGGGGCCCCAGATATGGCCTTCCCCCG
AATAAATAATATAAGTTTTTGATTATTACCCCCCTCTTTAACCCTCTTAATCTCAAGAAGAGTCGTTGAAAATGGAGCTG
GAACAGGATGAACTGTTTATCCGCCCTTATCCTCCAATATTGCTCATGGTGGGACTTCTGTAGATTTAGCCATTTTTTCA
TTACATTTAGCTGGAATTTCTTCTATTCTAGGGGCTATTAACTTCATTACAACTATTATTAATATACGAATTAATGGACT
ATCTTTTGATCAAATACCTTTATTTGTATGAGCAGTTGGTATTACTGCCCTTCTTCTTCTTCTCTCACTACCTGTATT--
----

>LSTEM496-18|Scirpophaga_excerptalis|AMSww06203|
GACATTATATTTTATTTTTGGAATTTGAGCTGGTATAGTGGGGACTTCACTTAGTTTACTAATCCGAGCCGAACTAGGTA
CTCCGGGATCATTAATCGGAGATGATCAAATCTATAATACTATTGTAACTGCTCACGCTTTTATTATAATTTTTTTTATG
GTTATGCCCATTATAATTGGGGGATTCGGAAACTGATTAGTGCCTTTAATGTTGGGAGCCCCAGATATGGCCTTCCCCCG
AATAAATAATATAAGTTTTTGATTATTACCCCCTTCTTTAACCCTCTTAATCTCAAGAAGAGTCGTTGAAAATGGAGCTG
GAACAGGATGAACTGTTTATCCGCCCTTATCCTCCAATATTGCTCATGGTGGGACTTCTGTAGATTTAGCCATTTTTTCA
TTACATTTAGCTGGAATTTCTTCTATTCTAGGGGCTATTAACTTCATTACAACTATTATTAATATACGAATTAATGGACT
ATCTTTTGATCAAATACCTTTATTTGTATGAGCAGTTGGTATTACTGCCCTTCTTCTTCTTCTCTCACTACCTGTATTAG
CGGGAGCTATTACTATATTATTAACAGATCGAAACTTAAATAC-------------------------------------
----
>LSTEM497-18|Scirpophaga_excerptalis|AMSww06204|
GACATTATATTTTATTTTTGGAATTTGAGCTGGTATAGTGGGGACTTCACTTAGTTTACTAATCCGAGCCGAACTAGGTA
CTCCGGGATCATTAATCGGAGATGATCAAATCTATAATACTATTGTAACTGCTCACGCTTTTATTATAATTTTTTTTATG
GTTATGCCCATTATAATTGGGGGATTCGGAAACTGATTAGTGCCTTTAATGTTGGGAGCCCCAGATATGGCCTTCCCCCG
AATAAATAATATAAGTTTTTGATTATTACCCCCTTCTTTAACCCTCTTAATCTCAAGAAGAGTCGTTGAAAATGGAGCTG
GAACAGGATGAACTGTTTATCCGCCCTTATCCTCCAATATTGCTCATGGTGGGACTTCTGTAGATTTAGCCATTTTTTCA
TTACATTTAGCTGGAATTTCTTCTATTCTAGGGGCTATTAACTTCATTACAACTATTATTAATATACGAATTAATGGACT
ATCTTTTGATCAAATACCTTTATTTGTATGAGCAGTTGGTATTACTGCCCTTCTTCTTCTTCTCTCACTACCTGTATTAG
CGGGAGCTATTACTATATTATTAACAGATCGAAACTTAAATACTTCTTTCTTTGACCCAGCAGGAGGTGGAGACCCAATT

CTTTATCAACACTTATTTTGATTCTTT
>LSTEM498-18|Chilo_infuscatellus|AMSww06205|
---TTTATATTTTATTTTTGGAATTTGGGCAGGAATAATTGGAACTTCTCTTAGACTTCTAATTCGAGCTGAATTAGGAA
CTCCAGGATCTTTAATTGGGGATGATCAAATTTATAATACTATTGTTACAGCTCATGCATTTATTATAATTTTTTTTATA
GTAATACCAATTATAATTGGGGGATTTGGAAATTGATTAGTTCCTTTAATATTAGGGGCACCTGATATAGCTTTCCCACG
AATAAATAATATAAGTTTTTGATTATTACCACCATCATTAACATTATTGATTTCTAGAAGAATTGTTGAAAATGGAGCAG
GAACTGGTTGAACTGTTTATCCCCCTCTATCCTCAAATATTGCCCATGGGGGTAGTTCTGTAGATTTAGCAATTTTTTCC
CTTCATTTAGCGGGTATTTCATCAATTTTAGGGGCTATTAATTTTATTACAACAATTATTAATATACGAGTTAATGGTTT
ATCATTTGATCAAATACCTCTATTTGTTTGATCTGTAGGTATTACAGCACTATTATTATTACTTTCTTTACCAGTATTAG
CAGGTGCTATTACTATATTACTAACTGATCGGAATTTAAATACATCTTTTTTTGACCCTGCTGGAGGGGGAGATCCAATT
CTCTATCAACATTTATTTTGATTTTTT
>LSTEM499-18|Chilo_sacchariphagus|AMSww06206|
AACTTTATATTTTATTTTTGGAATTTGAGCTGGAATAATTGGAACATCCCTTAGACTTTTAATTCGAGCTGAATTAGGAA
ATCCAGGTTCATTAATTGGAGATGATCAAATTTATAATACTATTGTTACAGCTCATGCATTTATTATAATTTTTTTTATA
GTAATACCAATTATAATCGGAGGATTTGGAAATTGATTAGTTCCATTAATATTAGGAGCTCCTGATATAGCCTTTCCTCG
TTTAAATAATATAAGATTTTGATTATTACCCCCTTCTTTAACTCTTTTAATTTCTAGAAGAATTGTTGAAAATGGAGCAG
GAACTGGATGAACAGTCTACCCCCCCCTATCTTCCAATATTTCACATGCTGGAAGTTCAGTAGATTTAGCCATCTTTTCT
CTTCATTTAGCAGGAATTTCTTCAATTTTAGGAGCTATTAATTTTATTACTACAATTATTAATATACGAATTAATGGATT
ATTATTCGATCAAATACCATTATTTGTTTGATCTGTTGGTATTACAGCATTACTTCTTCTTCTCTCTTTACCAGTATTAG
CAGGTGCTATTACCATACTATTAACTGACCGAAATTTAAATACATCTTTTTTTGATCCAGCTGGAGGAGGTGATCCAATT
TTATATCAACATTTATTTTGATTTTTT
>LSTEM500-18|Chilo_infuscatellus|AMSww06207|
AACTTTATATTTTATTTTTGGAATTTGGGCAGGAATAATTGGAACTTCTCTTAGACTTCTAATTCGAGCTGAATTAGGAA
CTCCAGGATCTTTAATTGGGGATGATCAAATTTATAATACTATTGTTACAGCTCATGCATTTATTATAATTTTTTTTATA
GTAATACCAATTATAATTGGGGGATTTGGAAATTGATTAGTTCCTTTAATATTAGGGGCACCTGATATAGCTTTCCCACG
AATAAATAATATAAGTTTTTGATTATTACCACCATCATTAACATTATTGATTTCTAGAAGAATTGTTGAAAATGGAGCAG
GAACTGGTTGAACTGTTTATCCCCCTCTATCCTCAAATATTGCCCATGGGGGTAGTTCTGTAGATTTAGCAATTTTTTCC
CTTCATTTAGCGGGTATTTCATCAATTTTAGGGGCTATTAATTTTATTACAACAATTATTAATATACGAGTTAATGGTTT
ATCATTTGATCAAATACCTCTATTTGTTTGATCTGTAGGTATTACAGCACTATTATTATTACTTTCTTTACCAGTATTAG
CAGGTGCTATTACTATATTACTAACTGATCGGAATTTAAATACATCTTTTTTTGACCCTGCTGGAGGGGGAGATCCAATT
CTCTATCAACATTTATTTTG-------
>LSTEM501-18|Chilo_sacchariphagus|AMSww06208|
---TTTATATTTTATTTTTGGGATTTGAGCTGGAATAGTTGGAACATCCCTTAGACTTTTAATTCGAGCTGAATTAGGAA
ATCCAGGTTCATTAATCGGAGATGATCAAATTTATAATACTATTGTTACAGCCCATGCATTTATTATAATTTTTTTTATA
GTAATACCAATTATAATTGGAGGATTTGGAAATTGATTAGTTCCATTAATATTAGGAGCTCCTGATATAGCCTTCCCTCG
TCTAAATAATATAAGATTTTGATTATTACCCCCTTCTTTAACCCTTCTAATTTCTAGAAGAATCGTTGAAAATGGAGCAG
GAACTGGATGAACAGTCTACCCCCCCCTATCTTCCAATATTTCACATGCTGGAAGTTCAGTAGATTTAGCCATCTTTTCC
CTTCATTTAGCTGGAATTTCTTCAATTTTAGGAGCTATCAATTTCATTACTACAATTATTAATATACGAATTAATGGATT
ATTATTTGATCAAATACCATTATTTGTTTGATCTGTTGGTATTACAGCATTACTTCTCCTCCTTTCTTTACCAGTATTAG
CATGTGCTATTACTATACTATTAACTGATCGAAATTTAAATACATCTTTTTT---------------------------
----
>LSTEM502-18|Scirpophaga_excerptalis|AMSww06210|
GACATTATATTTTATTTTTGGAATTTGAGCTGGTATAGTAGGGACTTCACTTAGTTTACTAATTCGAGCCGAACTAGGTA
CTCCGGGATCATTAATCGGAGATGATCAAATCTATAATACTATTGTAACTGCTCACGCTTTTATTATAATTTTTTTTATG
GTTATGCCCATTATAATTGGGGGATTCGGAAACTGATTAGTGCCTTTAATATTGGGAGCCCCAGATATGGCCTTCCCCCG
AATAAATAATATAAGTTTTTGATTATTACCCCCTTCTTTAACCCTCTTAATCTCAAGAAGAGTCGTTGAAAATGGAGCTG
GAACAGGATGAACTGTTTATCCGCCCTTATCCTCCAATATTGCTCATGGTGGGACTTCTGTAGATTTAGCCATTTTTTCA
TTACATTTAGCTGGAATTTCTTCTATTCTAGGGGCTATTAACTTCATTACAACTATTATTAATATACGAATTAATGGACT
ATCTTTTGATCAAATACCTTTATTTGTATGAGCAGTTGGTATTACTGCCCTTCTTCTTCTTCTCTCACTACCTGTATTAG
CGGGAGCCATTACTATATTATTAACAGATCGAAACTTAAATACTTCTTTCTTTGACCCAGCAGGAGGTGGAGA-------
----
>LSTEM503-18|Scirpophaga_excerptalis|AMSww06211|
GACATTATATTTTATTTTTGGAATTTGAGCTGGTATAGTGGGGACTTCACTTAGTTTACTAATTCGAGCCGAACTAGGTA
CTCCGGGATCATTAATCGGAGATGATCAAATCTATAATACTATTGTAACTGCTCACGCTTTTATTATAATTTTTTTTATG
GTTATGCCCATTATAATTGGGGGATTCGGAAACTGATTAGTGCCTTTAATATTGGGAGCCCCAGATATGGCCTTCCCCCG
AATAAATAATATAAGTTTTTGATTATTACCCCCTTCTTTAACCCTCTTAATCTCAAGAAGAGTCGTTGAAAATGGAGCTG
GAACAGGATGAACTGTTTATCCGCCCTTATCCTCCAATATTGCTCATGGTGGGACTTCTGTAGATTTAGCCATTTTTTCA

TTACATTTAGCTGGAATTTCTTCTATTCTAGGGGCTATTAACTTCATTACAACTATTATTAATATACGAATTAATGGACT
ATCTTTTGATCAAATACCTTTATTTGTATGAGCAGTTGGTATTACTGCCCTTCTTCTTCTTCTCTCACTACCTGTATTAG
CAGGAGCTATTACTATATTATTAACAGATCGAAACTTAAATACTTCTTTCTTTGACCCAGCAGGAGGTGGAGACCCAATT
CTTTATCAACACTTATTTTGATTCTTT
>LSTEM504-18|Scirpophaga_excerptalis|AMSww06212|
GACATTATATTTTATTTTTGGAATTTGAGCTGGTATAGTAGGGACTTCACTTAGTTTACTAATTCGAGCCGAACTAGGTA
CTCCGGGATCATTAATCGGAGATGATCAAATCTATAATACTATTGTAACTGCTCACGCTTTTATTATAATTTTTTTTATG
GTTATGCCCATTATAATTGGGGGATTCGGAAACTGATTAGTGCCTTTAATATTGGGAGCCCCAGATATGGCCTTCCCCCG
AATAAATAATATAAGTTTTTGATTATTACCCCCTTCTTTAACCCTCTTAATCTCAAGAAGAGTCGTTGAAAATGGAGCTG
GAACAGGATGAACTGTTTATCCGCCCTTATCCTCCAATATTGCTCATGGTGGGACTTCTGTAGATTTAGCCATTTTTTCA
TTACATTTAGCTGGAATTTCTTCTATTCTAGGGGCTATTAACTTCATTACAACTATTATTAATATACGAATTAATGGACT
ATCTTTTGATCAAATACCTTTATTTGTATGAGCAGTTGGTATTACTGCCCTTCTTCTTCTTCTCTCACTACCTGTATTAG
CGGGAGCCATTACTATATTATTAACAGATCGAAACTTAAATACTTCTTTCTTTGACCCAGCAGGAGGTGGAGACCCAATT
CTTTATCAACACTTATTTTGATTCTTT
>LSTEM505-18|Scirpophaga_excerptalis|AMSww06213|
----TTATATTTTATTTTTGGAATTTGAGCTGGTATAGTGGGGACTTCACTTAGTTTACTAATCCGAGCCGAACTAGGTA
CTCCGGGATCATTAATCGGAGATGATCAAATCTATAATACTATTGTAACTGCTCACGCTTTTATTATAATTTTTTTTATG
GTTATGCCCATTATAATTGGGGGATTCGGAAACTGATTAGTGCCTTTAATATTGGGAGCCCCAGATATGGCCTTCCCCCG
AATAAATAATATAAGTTTTTGATTATTACCCCCTTCTTTAACCCTCTTAATCTCAAGAAGAGTCGTTGAAAATGGAGCTG
GAACAGGATGAACTGTTTATCCGCCCTTATCCTCCAATATTGCTCATGGTGGGACTTCTGTAGATTTAGCCATTTTTTCA
TTACATTTAGCTGGAATTTCTTCTATTCTAGGGGCTATTAACTTCATTACAACTATTATTAATATACGAATTAATGGACT
ATCTTTTGATCAAATACCTTTATTTGTATGAGCAGTTGGTATTACTGCCCTTCTTCTTCTTCTCTCACTACCTGTATTAG
CGGGAGCTATTACTATATTATTAACAGATCGAAACTTAAATACTTCTTTCTTTGACCCAGCAGGAGGTGGAGACCCAATT
CTTTATCAACACTTATTTTGATTCTTT
>LSTEM506-18|Scirpophaga_excerptalis|AMSww06214|
GACATTATATTTTATTTTTGGAATTTGAGCTGGTATAGTGGGGACTTCACTTAGTTTACTAATTCGAGCCGAACTAGGTA
CTCCGGGATCATTAATTGGAGATGATCAAATCTATAATACTATTGTAACTGCTCACGCTTTTATTATAATTTTTTTTATG
GTTATGCCCATTATAATTGGGGGATTCGGAAACTGATTAGTGCCTTTAATATTGGGAGCCCCAGATATGGCCTTCCCCCG
AATAAATAATATAAGTTTTTGATTATTACCCCCTTCTTTAACCCTCTTAATCTCAAGAAGAGTCGTTGAAAATGGAGCTG
GAACAGGATGAACTGTTTATCCGCCCTTATCCTCCAATATTGCTCATGGTGGGACTTCTGTAGATTTAGCCATTTTTTCA
TTACATTTAGCTGGAATTTCTTCTATTCTAGGAGCTATTAACTTCATTACAACTATTATTAATATACGAATTAATGGACT
ATCTTTTGATCAAATACCTTTATTTGTATGAGCAGTTGGTATTACTGCCCTTCTTCTTCTTCTCTCACTACCTGTATTAG
CGGGAGCTATTACTATATTATTAACAGATCGAAACTTAAATACTTCTTTCTTTGACCCAGCAGGAGGTGGAGACCCAATT
CTTTATCAACACTTATTTTGATTCTTT
>LSTEM507-18|Scirpophaga_excerptalis|AMSww06215|
GACATTATATTTTATTTTTGGAATTTGAGCTGGTATAGTGGGGACTTCACTTAGTTTACTAATTCGAGCCGAACTAGGTA
CTCCGGGATCATTAATCGGAGATGATCAAATCTATAATACTATTGTAACTGCTCACGCTTTTATTATAATTTTTTTTATA
GTTATGCCCATTATAATTGGGGGATTCGGAAACTGATTAGTGCCTTTAATATTGGGAGCCCCAGATATGGCCTTCCCCCG
AATAAATAATATAAGTTTTTGATTATTACCCCCTTCTTTAACCCTCTTAATCTCAAGAAGAGTCGTTGAAAATGGAGCTG
GAACAGGATGAACTGTTTATCCGCCCTTATCCTCCAATATTGCTCATGGTGGGACTTCTGTAGATTTAGCCATTTTTTCA
TTACATTTAGCTGGAATTTCTTCTATTCTAGGGGCTATTAACTTCATTACAACTATTATTAATATACGAATTAATGGACT
ATCTTTTGATCAAATACCTTTATTTGTATGAGCAGTTGGTATTACTGCCCTTCTTCTTCTTCTCTCACTACCTGTATTAG
CGGGAGCTATTACTATATTATTAACAGATCGAAACTTAAATACTTCTTTCTTTGACCCAGCAGGAGGTGGAGACCCAATT
C--------------------------
>LSTEM508-18|Chilo_sacchariphagus|AMSww06216|

---ACAGSCCATGCATTTATTATGATKTTTTTTATR
ATAATACCAATTATAATKGGAGGATTTGGAAATTGATTAGTWCCWTTAATAWTAGGRGCTCCTGATATRGCCTTCCCTCG
TYTAAATAATWTAAGATTTTGATTATTACCCCCTTCTTTAACCCTTCTAATTTCYACAANAATCGYTGAAAAWGGAGCAG
GAACTGGATGAACWKTCTACCCCCCCCTATCTTCCAATATTTCACATGCTGGARGTTCAGTAGATTTAGCCATCTTCTCC
CTTCATTTAGCTGGAATTTCTTCAATTTTAGGAGCTATCAATTTCATTACTACAATTATTAATATACGAATTAATGGATT
ATTATTTGATCAAAAACCATTATTTGTTTGATCTGTTGGTATTACAGCATTACTTCTCCTCCTTTCTTTACCAGTATTAT
GAGGTGCTATTACTATACTAGTAMCTGAACGAAATTTAAATAC-------------------------------------
----
>LTOLB122-08|Chilo_suppressalis|MAS-92-1001-1|KF491628
AACTTTATATTTTATTTTTGGTATTTGAGCAGGTATAATTGGAACATCTCTTAGACTTTTAATTCGTGCTGAATTAGGAA
CTCCAGGATCTTTAATTGGGGATGATCAAATTTATAATACCATTGTTACAGCTCATGCATTTATTATAATTTTTTTTATA

GTTATACCAATTATAATTGGTGGATTTGGAAATTGATTAGTACCTTTAATATTAGGGGCTCCTGATATAGCTTTCCCACG
AATAAATAATATAAGATTTTGAATATTACCCCCCTCTTTAACTTTACTAATTTCTAGAAGAATTGTTGAAAATGGAGCTG
GAACAGGTTGAACAGTGTACCCCCCACTATCATCTAATATTGCTCACGCTGGAAGTTCAGTAGATTTAGCAATTTTCTCT
TTACATTTAGCTGGAATTTCTTCAATTCTAGGTGCTATTAATTTTATTACTACAATTATTAATATACGAATTAATGGTCT
TTCATTTGATCAAATACCTTTATTTGTTTGATCCGTAGGTATTACAGCTTTATTATTACTTCTATCTCTACCAGTATTAA
CTGGAGCAATTACAATATTATTAACCGATCGAAATTTAAATACATCTTTTTTTGATCCTGCTGGTGGTGGAGATCCAATT
CTTTACCAACATTTATTT---------
>MAIMB231-09|Emmalocera_sp.|NIBGE_IMB-00231|GU682015-SUPPRESSED
AACTTTATATTTTATTTTTGGAATTTGATCAGGTATAGTAGGTACTTCTTTAAGTCTTCTTATTCGAGCTGAATTAGGAA
CTCCTAGATCTTTAATTGGAGATGACCAAATTTATAATACTATTGTTACTGGACATGCTTTTATTATAATTTTTTTTATA
GTTATACCTATTATAATTGGAGGATTTGGAAATTGATTAGTTCCTTTAATATTAGGAGCCCCAGATATAGCTTTCCCTCG
AATAAATAATATAAGATTTTGACTTTTACCTCCCTCTCTTAATTTATTAATTTCTAGAAGAATTGTAGAAAATGGAGCTG
GAACAGGTTGAACAGTTTATCCCCCTTTATCTTCTAATATTGCTCATAGTGGAAGTTCTGTTGATCTTGCTATTTTTTCT
TTACATCTAGCAGGAATTTCTTCTATTTTAGGTGCTATTAATTTTATTACTACTATTATTAATATAAAATTAAATGGTTT
ATCTTTTGATCAAATACCTTTATTTGTTTGAGCTGTAGGAATTACAGCTTTATTATTACTTTTATCCTTGCCAGTATTAG
CAGGAGCTATTACTATATTATTAACTGACCGAAATTTAAATACTTCTTTCTTTGACCCTGCAGGAGGAGGAGACCCAATT
TTATACCAACATTTATTT---------
>MAIMB232-09|Emmalocera_sp.|NIBGE_IMB-00232|GU682013-SUPPRESSED
AACTTTATATTTTATTTTTGGAATTTGATCAGGTATAGTAGGTACTTCTTTAAGTCTTCTTATTCGAGCTGAATTAGGAA
CTCCTAGATCTTTAATTGGAGATGATCAAATTTATAATACTATTGTTACTGGACATGCTTTTATTATAATTTTTTTTATA
GTTATACCTATTATAATTGGAGGATTTGGAAATTGATTAGTTCCTTTAATATTAGGAGCCCCAGATATAGCTTTCCCTCG
AATAAATAATATAAGATTTTGACTTTTACCTCCCTCTCTTAATTTATTAATTTCTAGAAGAATTGTAGAAAATGGAGCTG
GAACAGGTTGAACAGTTTATCCCCCTTTATCTTCTAATATTGCCCATAGTGGGAGTTCTGTTGATCTTGCTATTTTTTCT
TTACATCTAGCAGGAATTTCTTCTATTTTAGGTGCTATTAATTTTATTACTACTATTATTAATATAAAATTAAATGGTTT
ATCTTTTGATCAAATACCTTTATTTGTTTGAGCTGTAGGAATTACAGCTTTATTATTACTTTTATCCTTGCCAGTATTAG
CAGGAGCTATTACTATATTATTAACTGACCGAAATTTAAATACTTCTTTCTTTGACCCTGCAGGAGGAGGAGACCCAATT
TTATATCAACATTTATTT---------
>MAIMB239-09|Scirpophaga_excerptalis|NIBGE_IMB-00239|GU682009-SUPPRESSED
GACATTATATTTTATTTTTGGAATTTGAGCTGGTATAGTAGGAACTTCACTTAGTTTACTAATTCGAGCCGAATTAGGTA
CTCCTGGATCACTAATTGGAGATGATCAAATCTATAATACTATTGTAACTGCTCACGCTTTTATTATAATTTTTTTTATA
GTTATACCTATTATAATTGGGGGATTCGGAAACTGATTAGTACCTTTAATATTAGGAGCTCCAGATATGGCTTTCCCCCG
AATAAATAACATAAGTTTTTGATTATTACCCCCTTCTTTAACCCTCTTAATCTCAAGAAGAATTGTTGAAAATGGGGCTG
GAACAGGATGAACTGTTTACCCGCCCCTATCCTCCAATATTGCCCACGGTGGGACTTCTGTAGATTTAGCTATTTTTTCA
TTACATTTAGCTGGAATTTCTTCTATTCTAGGGGCTATTAACTTCATTACAACTATTATTAATATGCGAATTAATGGACT
ATCTTTTGATCAAATACCTTTATTCGTGTGAGCAGTTGGTATTACTGCCCTTCTTCTTCTTCTCTCACTACCTGTATTAG
CAGGAGCTATCACTATATTATTAACAGATCGAAACTTAAATACCTCTTTCTTTGACCCAGCAGGAGGTGGGGACCCAATT
CTTTATCAACATTTATTT---------
>MAIMB240-09|Scirpophaga_excerptalis|NIBGE_IMB-00240|KX861751
AACATTATATTTTATTTTTGGAATTTGAGCTGGTATAGTAGGAACTTCCCTTAGTTTACTAATTCGAGCCGAACTAGGTA
CTCCTGGATCACTAATTGGAGATGATCAAATCTATAATACTATTGTAACTGCTCACGCTTTTATTATAATTTTTTTTATA
GTTATACCTATTATAATTGGGGGATTCGGAAACTGATTAGTACCTTTAATATTAGGAGCTCCAGATATAGCTTTTCCCCG
AATAAATAACATAAGTTTTTGATTACTACCCCCTTCTTTAACCCTCTTAATCTCAAGAAGAATTGTTGAAAATGGAGCTG
GAACAGGATGAACTGTTTACCCGCCTCTATCCTCCAATATTGCCCACGGTGGGACTTCTGTAGATTTAGCTATTTTTTCA
TTACATTTAGCTGGAATTTCTTCTATTCTAGGGGCTATCAACTTCATTACAACTATTATTAATATGCGAATTAATGGACT
ATCCTTTGATCAAATACCTTTATTCGTGTGAGCAGTTGGTATTACTGCCCTTCTTCTTCTTCTCTCACTACCTGTATTAG
CGGGAGCTATCACTATATTATTAACAGATCGAAACTTAAATACCTCTTTCTTTGACCCAGCAGGAGGGGGGGACCCAATT
CTTTATCAACATTTATTT---------
>MAIMB242-09|Scirpophaga_excerptalis|NIBGE_IMB-00242|GU682007-SUPPRESSED
GACATTATATTTTATTTTTGGAATTTGAGCTGGTATAGTGGGAACTTCCCTTAGTTTATTAATCCGAGCTGAACTAGGTA
CTCCTGGATCACTAATTGGAGATGATCAAATCTATAATACTATTGTAACTGCTCACGCTTTTATTATAATTTTTTTTATA
GTTATACCTATTATAATTGGGGGATTCGGAAACTGATTAGTACCTTTAATATTAGGAGCTCCAGATATAGCTTTTCCCCG
AATAAACAACATAAGTTTTTGATTATTACCCCCTTCTTTAACCCTCTTAATCTCAAGAAGAATTGTTGAAAATGGAGCTG
GAACAGGATGAACTGTTTACCCGCCCCTATCCTCCAATATTGCCCACGGTGGGACTTCTGTAGATTTAGCTATTTTTTCA
TTACATTTAGCTGGAATTTCTTCTATTCTAGGGGCTATTAACTTCATTACAACTATTATTAATATGCGAATTAATGGACT
ATCCTTTGATCAAATACCTTTATTCGTGTGAGCAGTTGGTATTACTGCCCTTCTTCTTCTTCTCTCACTACCTGTATTAG
CGGGAGCTATCACTATATTATTAACAGATCGAAACCTAAATACCTCTTTCTTTGACCCAGCAGGAGGGGGGGACCCAATT
CTTTATCAACATTTATTT---------

>MAIMB276-09|Sesamia_inferens|NIBGE_IMB-00276|GU681998-SUPPRESSED
AACATTATATTTTATTTTTGGAATTTGAGCTGGTATAGTAGGAACATCATTAAGATTATTAATTCGAGCTGAATTAGGAA
CCCCAGGATCTTTAATTGGAGATGATCAAATTTATAATACTATTGTTACAGCTCATGCTTTTATTATAATTTTTTTTATA
GTTATACCAATTATAATTGGAGGATTTGGAAATTGACTTGTACCTTTAATATTAGGAGCTCCTGATATAGCATTTCCACG
AATAAATAATATAAGATTTTGATTATTACCCCCCTCTTTAACTCTTTTAATTTCAAGTAGAATTGTAGAAAATGGAGCAG
GAACTGGATGAACAGTGTACCCCCCACTTTCATCTAATATTGCTCATGGAGGAAGATCAGTAGATCTAGCTATTTTTTCC
CTTCATTTAGCTGGTATTTCATCTATTTTAGGAGCTATTAATTTTATTACAACAATTATCAATATACGACTAAATAGCTT
ATCTTTTGATCAAATACCCCTATTTATTTGAGCTGTTGGAATTACTGCATTTTTATTATTATTATCTTTACCTGTGTTAG
CAGGAGCTATTACAATATTATTGACAGATCGAAATTTAAATACATCATTCTTTGACCCCGCAGGGGGAGGTGATCCTATT
TTATACCAACATTTATTT---------
>MAIMB278-09|Sesamia_inferens|NIBGE_IMB-00278|GU681997-SUPPRESSED
AACATTATATTTTATTTTTGGAATTTGAGCTGGTATAGTAGGAACATCATTAAGATTATTAATTCGAGCTGAATTAGGAA
CCCCAGGATCTTTAATTGGAGATGATCAAATTTATAATACTATTGTTACAGCTCATGCTTTTATTATAATTTTTTTTATA
GTTATACCAATTATAATCGGAGGATTTGGAAATTGACTTGTACCTTTAATATTAGGAGCTCCTGATATAGCATTTCCACG
AATAAATAATATAAGATTTTGATTATTACCCCCCTCTTTAACTCTTTTAATTTCAAGTAGAATTGTAGAAAATGGAGCAG
GAACTGGATGAACAGTGTACCCCCCACTTTCATCTAATATTGCTCATGGAGGAAGATCAGTAGATCTAGCTATTTTTTCC
CTTCATTTAGCTGGTATTTCATCTATTTTAGGAGCTATTAATTTTATTACAACAATTATCAATATACGACTAAATAGTTT
ATCTTTTGATCAAATACCTCTATTTATTTGAGCTGTTGGAATTACTGCATTTTTATTATTATTATCTTTACCTGTATTAG
CAGGAGCTATTACAATATTATTAACAGATCGAAATTTAAATACATCATTCTTTGACCCCGCAGGAGGAGGTGATCCTATT
TTATACCAACATTTATTT---------
>MAMOT094-10|Scirpophaga_excerptalis|NIBGE_MOT-00094|HQ990814-SUPPRESSED
GACATTATATTTTATTTTTGGAATTTGAGCTGGTATAGTAGGAACTTCACTTAGTTTACTAATTCGAGCCGAATTAGGTA
CTCCTGGATCACTAATTGGAGATGATCAAATCTATAATACTATTGTAACTGCTCACGCTTTTATTATAATTTTTTTTATA
GTTATACCTATTATAATTGGGGGATTCGGAAACTGATTAGTACCTTTAATATTAGGAGCTCCAGATATGGCTTTCCCCCG
AATAAATAACATAAGTTTTTGATTATTACCCCCTTCTTTAACCCTCTTAATCTCAAGAAGAATTGTTGAAAATGGGGCTG
GAACAGGATGAACTGTTTACCCGCCCCTATCCTCCAATATTGCCCACGGTGGGACTTCTGTAGATTTAGCTATTTTTTCA
TTACATTTAGCTGGAATTTCTTCTATTCTAGGGGCTATTAACTTCATTACAACTATTATTAATATGCGAATTAATGGACT
ATCTTTTGATCAAATACCTTTATTCGTGTGAGCAGTTGGTATTACTGCCCTTCTTCTTCTTCTCTCACTACCTGTATTAG
CAGGAGCTATCACTATATTATTAACAGATCGAAACTTAAATACCTCTTTCTTTGACCCAGCAGGAGGTGGGGACCCAATT
CTTTATCAACATTTATTT---------
>MAMOT095-10|Scirpophaga_excerptalis|NIBGE_MOT-00095|HQ990815-SUPPRESSED
GACATTATATTTTATTTTTGGAATTTGAGCTGGTATAGTGGGAACTTCCCTTAGTTTACTAATTCGAGCCGAACTAGGTA
CTCCTGGATCACTAATTGGAGATGATCAAATCTATAATACTATTGTAACTGCTCACGCTTTTATTATAATTTTTTTTATA
GTTATACCTATTATAATTGGGGGATTCGGAAACTGATTAGTACCTTTAATATTAGGAGCTCCAGATATAGCTTTTCCCCG
AATAAACAACATAAGTTTTTGATTATTACCCCCTTCTTTAACCCTCTTAATCTCAAGAAGAATTGTTGAAAATGGAGCTG
GAACAGGATGAACTGTTTACCCGCCTCTATCCTCCAATATTGCCCACGGTGGGACTTCTGTAGATTTAGCTATTTTTTCA
TTACATTTAGCTGGAATTTCTTCTATTCTAGGGGCTATTAACTTCATTACAACTATTATTAATATGCGAATTAATGGACT
ATCCTTTGATCAAATACCTTTATTCGTGTGAGCAGTTGGTATTACTGCCCTTCTTCTTCTTCTCTCACTACCTGTATTAG
CGGGAGCTATCACTATATTATTAACAGATCGAAACCTAAATACCTCTTTCTTTGACCCAGCAGGAGGGGGGGACCCAATT
CTTTATCAACATTTATTT---------
>MAMOT096-10|Scirpophaga_excerptalis|NIBGE_MOT-00096|HQ990816-SUPPRESSED
GACATTATATTTTATTTTTGGAATTTGAGCTGGTATAGTAGGAACTTCACTTAGTTTACTAATTCGAGCCGAATTAGGTA
CTCCTGGATCACTAATTGGAGATGATCAAATCTATAATACTATTGTAACTGCTCACGCTTTTATTATAATTTTTTTTATA
GTTATACCTATTATAATTGGGGGATTCGGAAACTGATTAGTACCTTTAATATTAGGAGCTCCAGATATGGCTTTCCCCCG
AATAAATAACATAAGTTTTTGATTATTACCCCCTTCTTTAACCCTCTTAATCTCAAGAAGAATTGTTGAAAATGGGGCTG
GAACAGGATGAACTGTTTACCCGCCCCTATCCTCCAATATTGCCCACGGTGGGACTTCTGTAGATTTAGCTATTTTTTCA
TTACATTTAGCTGGAATTTCTTCTATTCTAGGGGCTATTAACTTCATTACAACTATTATTAATATGCGAATTAATGGACT
ATCTTTTGATCAAATACCTTTATTCGTGTGAGCAGTTGGTATTACTGCCCTTCTTCTTCTTCTCTCACTACCTGTATTAG
CAGGAGCTATCACTATATTATTAACAGATCGAAACTTAAATACCTCTTTCTTTGACCCAGCAGGAGGTGGGGACCCAATT
CTTTATCAACATTTATTT---------
>MAMOT198-10|Chilo_partellus|NIBGE_MOT-00198|HQ990904-SUPPRESSED
AACTTTATATTTTATTTTTGGAATTTGAGCAGGAATAATTGGAACATCCCTTAGATTATTAATTCGTGCAGAATTAGGAA
CTCCTGGATCTTTAATTGGAGATGATCAAATTTATAATACTATTGTAACAGCACATGCATTTATTATAATTTTTTTTATA
GTTATACCAATTATAATTGGTGGATTTGGAAATTGATTAGTACCTTTAATATTAGGAGCCCCAGATATAGCTTTCCCACG
AATAAATAATATAAGATTTTGATTATTACCACCATCATTAACTTTACTAATTTCTAGAAGAATTGTTGAAAATGGAGCTG
GAACAGGATGAACAGTGTACCCCCCACTATCATCTAATATTGCTCATGCTGGAAGTTCAGTAGATTTAGCAATTTTTTCT
TTACATTTAGCTGGTATTTCATCAATTCTTGGTGCTATTAATTTTATTACAACAATCATTAATATACGAATTAATGGATT

ATTTTTTGATCAAATACCATTATTTGTTTGATCTGTAGGTATTACAGCTTTATTATTATTACTTTCTTTACCTGTTTTAG
CTGGAGCTATTACTATATTATTAACAGATCGAAATTTAAATACATCCTTTTTCGATCCTGCTGGAGGAGGAGATCCTATT
CTTTATCAACACTTATTT---------
>MAMOT199-10|Chilo_partellus|NIBGE_MOT-00199|HQ990905-SUPPRESSED
AACTTTATATTTTATTTTTGGAATTTGAGCAGGAATAATTGGAACATCCCTTAGATTATTAATTCGTGCAGAATTAGGAA
CTCCTGGATCTTTAATTGGAGATGATCAAATTTATAATACTATTGTAACAGCACATGCATTTATTATAATTTTTTTTATA
GTTATACCAATTATAATTGGTGGATTTGGAAATTGATTAGTACCTTTAATATTAGGAGCCCCAGATATAGCTTTCCCACG
AATAAATAATATAAGATTTTGATTATTACCACCATCATTAACTTTACTAATTTCTAGAAGAATTGTTGAAAATGGAGCTG
GAACAGGATGAACAGTGTACCCCCCACTATCATCTAATATTGCTCATGCTGGAAGTTCAGTAGATTTAGCAATTTTTTCT
TTACATTTAGCTGGTATTTCATCAATTCTTGGTGCTATTAATTTTATTACAACAATCATTAATATACGAATTAATGGATT
ATTTTTTGATCAAATACCATTATTTGTTTGATCTGTAGGTATTACAGCTTTATTATTATTACTTTCTTTACCTGTTTTAG
CTGGAGCTATTACTATATTATTAACAGATCGAAATTTAAATACATCCTTTTTCGATCCTGCTGGAGGAGGAGATCCTATT
CTTTATCAACACTTATTT---------
>MAMOT201-10|Chilo_partellus|NIBGE_MOT-00201|HQ990907-SUPPRESSED
AACTTTATATTTTATTTTTGGAATTTGAGCAGGAATAATTGGAACATCCCTTAGATTATTAATTCGTGCAGAATTAGGAA
CTCCTGGATCTTTAATTGGAGATGATCAAATTTATAATACTATTGTAACAGCACATGCATTTATTATAATTTTTTTTATA
GTTATACCAATTATAATTGGTGGATTTGGAAATTGATTAGTACCTTTAATATTAGGAGCCCCAGATATAGCTTTCCCACG
AATAAATAATATAAGATTTTGATTATTACCACCATCATTAACTTTACTAATTTCTAGAAGAATTGTTGAAAATGGAGCTG
GAACAGGATGAACAGTGTACCCCCCACTATCATCTAATATTGCTCATGCTGGAAGTTCAGTAGATTTAGCAATTTTTTCT
TTACATTTAGCTGGTATTTCATCAATTCTTGGTGCTATTAATTTTATTACAACAATCATCAATATACGAATTAATGGGTT
ATTTTTTGATCAAATACCATTATTTGTTTGATCTGTAGGTATTACAGCTTTATTATTATTACTTTCTTTACCTGTTTTAG
CTGGAGCTATTACTATATTATTAACAGATCGAAATTTAAATACATCCTTTTTCGATCCTGCTGGAGGAGGAGATCCTATT
CTTTATCAACACTTATTT---------
>MAMOT202-10|Chilo_partellus|NIBGE_MOT-00202|HQ990908-SUPPRESSED
AACTTTATATTTTATTTTTGGAATTTGAGCAGGAATAATTGGAACATCCCTTAGATTATTAATTCGTGCAGAATTAGGAA
CTCCTGGATCTTTAATTGGAGATGATCAAATTTATAATACTATTGTAACAGCACATGCATTTATTATAATTTTTTTTATA
GTTATACCAATTATAATTGGTGGATTTGGAAATTGATTAGTACCTTTAATATTAGGAGCCCCAGATATAGCTTTCCCACG
AATAAATAATATAAGATTTTGATTATTACCACCATCATTAACTTTACTAATTTCTAGAAGAATTGTTGAAAATGGAGCTG
GAACAGGATGAACAGTGTACCCCCCACTATCATCTAATATTGCTCATGCTGGAAGTTCAGTAGATTTAGCAATTTTTTCT
TTACATTTAGCTGGTATTTCATCAATTCTTGGTGCTATTAATTTTATTACAACAATCATTAATATACGAATTAATGGATT
ATTTTTTGATCAAATACCATTATTTGTTTGATCTGTAGGTATTACAGCTTTATTATTATTACTTTCTTTACCTGTTTTAG
CTGGAGCTATTACTATATTATTAACAGATCGAAATTTAAATACATCCTTTTTCGATCCTGCTGGAGGAGGAGATCCTATT
CTTTATCAACACTTATTT---------
>MAMOT221-10|Chilo_partellus|NIBGE_MOT-00221|HQ990926-SUPPRESSED
AACTTTATATTTTATTTTTGGAATTTGAGCAGGAATAATTGGAACATCCCTTAGATTATTAATTCGTGCAGAATTAGGAA
CTCCTGGATCTTTAATTGGAGATGATCAAATTTATAATACTATTGTAACAGCACATGCATTTATTATAATTTTTTTTATA
GTTATACCAATTATAATTGGTGGATTTGGAAATTGATTAGTACCTTTAATATTAGGAGCCCCAGATATAGCTTTCCCACG
AATAAATAATATAAGATTTTGATTATTACCACCATCATTAACTTTACTAATTTCTAGAAGAATTGTTGAAAATGGAGCTG
GAACAGGATGAACAGTGTACCCCCCACTATCATCTAATATTGCTCATGCTGGAAGTTCAGTAGATTTAGCAATTTTTTCT
TTACATTTAGCTGGTATTTCATCAATTCTTGGTGCTATTAATTTTATTACAACAATCATTAATATACGAATTAATGGATT
ATTTTTTGATCAAATACCATTATTTGTTTGATCTGTAGGTATTACAGCTTTATTATTATTACTTTCTTTACCTGTTTTAG
CTGGAGCTATTACTATATTATTAACAGATCGAAATTTAAATACATCCTTTTTCGATCCTGCTGGAGGAGGAGATCCTATT
CTTTATCAACACTTATTT---------
>MAMOT222-10|Chilo_partellus|NIBGE_MOT-00222|HQ990927-SUPPRESSED
AACTTTATATTTTATTTTTGGAATTTGAGCAGGAATAATTGGAACATCCCTTAGATTATTAATTCGTGCAGAATTAGGAA
CTCCTGGATCTTTAATTGGAGATGATCAAATTTATAATACTATTGTAACAGCACATGCATTTATTATAATTTTTTTTATA
GTTATACCAATTATAATTGGTGGATTTGGAAATTGATTAGTACCTTTAATATTAGGAGCCCCAGATATAGCTTTCCCACG
AATAAATAATATAAGATTTTGATTATTACCACCATCATTAACTTTACTAATTTCTAGAAGAATTGTTGAAAATGGAGCTG
GAACAGGATGAACAGTGTACCCCCCACTATCATCTAATATTGCTCATGCTGGAAGTTCAGTAGATTTAGCAATTTTTTCT
TTACATTTAGCTGGTATTTCATCAATTCTTGGTGCTATTAATTTTATTACAACAATCATTAATATACGAATTAATGGATT
ATTTTTTGATCAAATACCATTATTTGTTTGATCTGTAGGTATTACAGCTTTATTATTATTACTTTCTTTACCTGTTTTAG
CTGGAGCTATTACTATATTATTAACAGATCGAAATTTAAATACATCCTTTTTCGATCCTGCTGGAGGAGGAGATCCTATT
CTTTATCAACACTTATTT---------
>MAMOT325-10|Chilo_partellus|NIBGE_MOT-00325|HQ991020-SUPPRESSED
AACTTTATATTTTATTTTTGGAATTTGAGCAGGAATAATTGGAACATCCCTTAGATTATTAATTCGTGCAGAATTAGGAA
CTCCTGGATCTTTAATTGGAGATGATCAAATTTATAATACTATTGTAACAGCACATGCATTTATTATAATTTTTTTTATA
GTTATACCAATTATAATTGGTGGATTTGGAAATTGATTAGTACCTTTAATATTAGGAGCCCCAGATATAGCTTTCCCACG

AATAAATAATATAAGATTTTGATTATTACCACCATCATTAACTTTACTAATTTCTAGAAGAATTGTTGAAAATGGAGCTG
GAACAGGATGAACAGTGTACCCCCCACTATCATCTAATATTGCTCATGCTGGAAGTTCAGTAGATTTAGCAATTTTTTCT
TTACATTTAGCTGGTATTTCATCAATTCTTGGTGCTATTAATTTTATTACAACAATCATCAATATACGAATTAATGGATT
ATTTTTTGATCAAATACCATTATTTGTTTGATCTGTAGGTATTACAGCTTTATTATTATTACTTTCTTTACCTGTTTTAG
CTGGAGCTATTACTATACTATTAACAGATCGAAATTTAAATACATCCTTTTTCGATCCTGCTGGAGGAGGAGATCCTATT
CTTTATCAACACTTATTT---------
>MAMOT326-10|Chilo_partellus|NIBGE_MOT-00326|HQ991021-SUPPRESSED
AACTTTATATTTTATTTTTGGAATTTGAGCAGGAATAATTGGAACATCCCTTAGATTATTAATTCGTGCAGAATTAGGAA
CTCCTGGATCTTTAATTGGAGATGATCAAATTTATAATACTATTGTAACAGCACATGCATTTATTATAATTTTTTTTATA
GTTATACCAATTATAATTGGTGGATTTGGAAATTGATTAGTACCTTTAATATTGGGAGCCCCAGATATAGCTTTCCCACG
AATAAATAATATAAGATTTTGATTATTACCACCATCATTAACTTTACTAATTTCTAGAAGAATTGTTGAAAATGGAGCTG
GAACAGGATGAACAGTGTACCCCCCACTATCATCTAATATTGCTCATGCTGGAAGTTCAGTAGATTTAGCAATTTTTTCT
TTACATTTAGCTGGTATTTCATCAATTCTTGGTGCTATTAATTTTATTACAACAATCATTAATATACGAATTAATGGATT
ATTTTTTGATCAAATACCATTATTTGTTTGATCTGTAGGTATTACAGCTTTATTATTATTACTTTCTTTACCTGTTTTAG
CTGGGGCTATTACTATATTATTAACAGATCGAAATTTAAATACATCCTTTTTCGATCCTGCTGGAGGAGGAGATCCTATT
CTTTATCAACACTTATTT---------
>MAMOT482-10|Chilo_partellus|NIBGE_MOT-00482|HQ991174-SUPPRESSED
AACTTTATATTTTATTTTTGGAATTTGAGCAGGAATAATTGGAACATCCCTTAGATTATTAATTCGTGCAGAATTAGGAA
CTCCTGGATCTTTAATTGGAGATGATCAAATTTATAATACTATTGTAACAGCACATGCATTTATTATAATTTTTTTTATA
GTTATACCAATTATAATTGGTGGATTTGGAAATTGATTAGTACCTTTAATATTAGGAGCCCCAGATATAGCTTTCCCACG
AATAAATAATATAAGATTTTGATTATTACCACCATCATTAACTTTACTAATTTCTAGAAGAATTGTTGAAAATGGAGCTG
GAACAGGATGAACAGTGTACCCCCCACTATCATCTAATATTGCTCATGCTGGAAGTTCAGTAGATTTAGCAATTTTTTCT
TTACATTTAGCTGGTATTTCATCAATTCTTGGTGCTATTAATTTTATTACAACAATCATTAATATACGAATTAATGGATT
ATTTTTTGATCAAATACCATTATTTGTTTGATCTGTAGGTATTACAGCTTTATTATTATTACTTTCTTTACCTGTTTTAG
CTGGAGCTATTACTATATTATTAACAGATCGAAATTTAAATACATCCTTTTTCGATCCTGCTGGAGGAGGAGATCCTATT
CTTTATCAACACTTATTT---------
>MAMOT485-10|Scirpophaga_excerptalis|NIBGE_MOT-00485|HQ991177-SUPPRESSED
GACATTATATTTTATTTTTGGAATTTGAGCTGGTATAGTAGGAACTTCACTTAGTTTACTAATTCGAGCCGAACTAGGTA
CTCCTGGATCACTAATTGGAGATGATCAAATCTATAATACTATTGTAACTGCTCACGCTTTTATTATAATTTTTTTTATA
GTTATACCTATTATAATTGGAGGATTCGGAAACTGATTAGTACCTNTAATATTAGGAGCCCCAGATATAGCTTTCCCCCG
AATAAATAACATAAGTTTTTGATTATTACCCCCCTCTTTAACCCTCTTAATCTCAAGAAGAATTGTTGAAAATGGGGCTG
GAACAGGATGAACTGTTTACCCGCCCTTATCCTCCAATATTGCCCACGGTGGGACTTCTGTAGATTTAGCTATTTTTTCA
CTACATTTAGCTGGAATTTCTTCTATTCTAGGGGCTATTAACTTTATTACAACTATTATTAATATACGAATTAATGGACT
ATCTTTTGATCAAATACCTTTATTCGTATGAGCAGTTGGTATTACTGCCCTTCTTCTTCTTCTCTCACTACCTGTATTAG
CAGGAGCTATCACTATATTATTAACAGATCGAAACTTAAATACCTCTTTCTTTGACCCAGCAGGAGGTGGAGACCCAATT
CTTTATCAACATTTATTT---------
>MAMOT496-10|Scirpophaga_incertulas|NIBGE_MOT-00496|HQ991188-SUPPRESSED
TACTTTATATTTTATTTTTGGAATTTGAGCTGGTATAGTAGGAACTTCTTTAAGCTTACTTATTCGAGCTGAATTAGGAA
CTTCTGGATCCTTAATTGGAGATGATCAAATCTATAACACTATTGTCACAGCCCATGCCTTTATTATAATTTTTTTTATA
GTTATACCCATTATAATTGGAGGATTTGGAAATTGATTAGTCCCCCTAATATTAGGAGCCCCAGATATAGCTTTCCCCCG
ATTAAATAACATAAGATTCTGATTATTACCCCCCTCTTTAACACTCCTCATTTCTAGAAGAATTGTAGAAAATGGAGCTG
GAACAGGATGAACTGTTTACCCACCCCTATCATCCAATATTGCTCATGGGGGAACATCAGTAGATTTAGCTATTTTTTCA
CTACACCTAGCAGGAATTTCATCTATTTTAGGAGCTATTAATTTTATTACAACCATTATTAATATACGAATTAATGGATT
ATCATTTGACCAAATACCTCTATTTGTGTGAGCTGTTGGTATTACAGCCCTTCTTCTACTCCTCTCTCTCCCAGTTTTAG
CTGGAGCTATTACTATGTTATTAACAGATCGAAATTTAAATACATCTTTTTTTGACCCAGCTGGGGGAGGAGATCCAATT
TTATATCAACATTTATTT---------
>MAMOT497-10|Scirpophaga_incertulas|NIBGE_MOT-00497|HQ991189-SUPPRESSED
TACTTTATATTTTATTTTTGGAATTTGAGCTGGTATAGTAGGAACTTCTTTAAGCTTACTTATTCGAGCTGAATTAGGAA
CTTCTGGATCCTTAATTGGAGATGATCAAATCTATAACACTATTGTCACAGCCCATGCCTTTATTATAATTTTTTTTATA
GTTATACCCATTATAATTGGAGGATTTGGAAATTGATTAGTCCCCCTAATATTAGGAGCCCCAGATATAGCTTTCCCCCG
ATTAAATAACATAAGATTCTGATTATTACCCCCCTCTTTAACACTCCTCATTTCTAGAAGAATTGTAGAAAATGGAGCTG
GAACAGGATGAACTGTTTACCCACCCCTATCATCCAATATTGCTCATGGGGGAACATCAGTAGATTTAGCTATTTTTTCA
CTACACCTAGCAGGAATTTCATCTATTTTAGGAGCTATTAATTTTATTACAACCATTATTAATATACGAATTAATGGATT
ATCATTTGACCAAATACCTCTATTTGTGTGAGCTGTTGGTATTACAGCCCTTCTTCTACTCCTCTCTCTCCCAGTTTTAG
CTGGAGCTATTACTATGTTATTAACAGATCGAAATTTAAATACATCTTTTTTTGACCCAGCTGGGGGAGGAGATCCAATT
TTATATCAACATTTATTT---------
>MAMOT512-10|Chilo_partellus|NIBGE_MOT-00512|HQ991202-SUPPRESSED

AACTTTATATTTTATTTTTGGAATTTGAGCAGGAATAATTGGAACATCCCTTAGATTATTAATTCGTGCAGAATTAGGAA
CTCCTGGATCTTTAATTGGAGATGATCAAATTTATAATACTATTGTAACAGCACATGCTTTTATTATAATTTTTTTTATA
GTTATACCAATTATAATTGGTGGATTTGGAAATTGATTAGTACCTTTAATATTAGGAGCCCCAGATATAGCTTTCCCACG
AATAAATAATATAAGATTTTGATTATTACCACCATCATTAACTTTACTAATTTCTAGAAGAATTGTTGAAAATGGAGCTG
GAACAGGATGAACAGTGTACCCCCCACTATCATCTAATATTGCTCATGCTGGAAGTTCAGTAGATTTAGCAATTTTTTCT
TTACATTTAGCTGGTATTTCATCAATTCTTGGTGCTATTAATTTTATTACAACAATCATTAATATACGAATTAATGGATT
ATTTTTTGATCAAATACCATTATTTGTTTGATCTGTAGGTATTACAGCTTTATTATTATTACTTTCTTTACCTGTTTTAG
CTGGAGCTATTACTATATTATTAACAGATCGAAATTTAAATACATCCTTTTTCGATCCTGCTGGAGGAGGAGATCCTATT
CTTTATCAACACTTATTT---------
>MAMOT528-10|Chilo_partellus|NIBGE_MOT-00528|HQ991218-SUPPRESSED
AACTTTATATTTTATTTTTGGAATTTGAGCAGGAATAATTGGAACATCCCTTAGATTATTAATTCGTGCAGAATTAGGAA
CTCCTGGATCTTTAATTGGAGATGATCAAATTTATAATACTATTGTAACAGCACATGCATTTATTATAATTTTTTTTATA
GTTATACCAATTATAATTGGTGGATTTGGAAATTGATTAGTACCTTTAATATTAGGAGCCCCAGATATAGCTTTCCCACG
AATAAATAATATAAGATTTTGATTATTACCACCATCATTAACTTTACTAATTTCTAGAAGAATTGTTGAAAATGGAGCTG
GAACAGGATGAACAGTGTACCCCCCACTATCATCTAATATTGCTCATGCTGGAAGTTCAGTAGATTTAGCAATTTTTTCT
TTACATTTAGCTGGTATTTCATCAATTCTTGGTGCTATTAATTTTATTACAACAATCATTAATATACGAATTAATGGATT
ATTTTTTGATCAAATACCATTATTTGTTTGATCTGTAGGTATTACAGCTTTATTATTATTACTTTCTTTACCTGTTTTAG
CTGGAGCTATTACTATATTATTAACAGATCGAAATTTAAATACATCCTTTTTCGATCCTGCTGGAGGAGGAGATCCTATT
CTTTATCAACACTTATTT---------
>MAMOT529-10|Chilo_partellus|NIBGE_MOT-00529|HQ991219-SUPPRESSED
AACTTTATATTTTATTTTTGGAATTTGAGCAGGAATAATTGGAACATCCCTTAGATTATTAATTCGTGCAGAATTAGGAA
CTCCTGGATCTTTAATTGGAGATGATCAAATTTATAATACTATTGTAACAGCACATGCATTTATTATAATTTTTTTTATA
GTTATACCAATTATAATTGGTGGATTTGGAAATTGATTAGTACCTTTAATATTAGGAGCCCCAGATATAGCTTTCCCACG
AATAAATAATATAAGATTTTGATTATTACCACCATCATTAACTTTACTAATTTCTAGAAGAATTGTTGAAAATGGAGCTG
GAACAGGATGAACAGTGTACCCCCCACTATCATCTAATATTGCTCATGCTGGAAGTTCAGTAGATTTAGCAATTTTTTCT
TTACATTTAGCTGGTATTTCATCAATTCTTGGTGCTATTAATTTTATTACAACAATCATTAATATACGAATTAATGGATT
ATTTTTTGATCAAATACCATTATTTGTTTGATCTGTAGGTATTACAGCTTTATTATTATTACTTTCTTTACCTGTTTTAG
CTGGAGCTATTACTATATTATTAACAGATCGAAATTTAAATACATCCTTTTTCGATCCTGCTGGAGGAGGAGATCCTATT
CTTTATCAACACTTATTT---------
>MAMOT530-10|Scirpophaga_excerptalis|NIBGE_MOT-00530|HQ991220-SUPPRESSED
GACATTATATTTTATTTTTGGAATTTGAGCTGGTATAGTAGGAACTTCACTTAGTTTACTAATTCGAGCCGAATTAGGTA
CTCCTGGATCACTAATTGGAGATGATCAAATCTATAATACTATTGTAACTGCTCACGCTTTTATTATAATTTTTTTTATA
GTTATACCTATTATAATTGGGGGATTCGGAAACTGATTAGTACCTTTAATATTAGGAGCTCCAGATATGGCTTTCCCCCG
AATAAATAACATAAGTTTTTGATTATTACCCCCTTCTTTAACCCTCTTAATCTCAAGAAGAATTGTTGAAAATGGGGCTG
GAACAGGATGAACTGTTTACCCGCCCCTATCCTCCAATATTGCCCACGGTGGGACTTCTGTAGATTTAGCTATTTTTTCA
TTACATTTAGCTGGAATTTCTTCTATTCTAGGGGCTATTAACTTCATTACAACTATTATTAATATGCGAATTAATGGACT
ATCTTTTGATCAAATACCTTTATTCGTGTGAGCAGTTGGTATTACTGCCCTTCTTCTTCTTCTCTCACTACCTGTATTAG
CAGGAGCTATCACTATATTATTAACAGATCGAAACTTAAATACCTCTTTCTTTGACCCAGCAGGAGGTGGGGACCCAATT
CTTTATCAACATTTATTT---------
>MAMOT531-10|Scirpophaga_excerptalis|NIBGE_MOT-00531|HQ991221-SUPPRESSED
------------------T---------------ATAGTAGGAACTTCACTTAGTTTACTAATTCGAGCCGAACTAGGTA
CTCCTGGATCACTAATTGGAGATGATCAAATCTATAATACTATTGTAACTGCTCACGCTTTTATTATAATTTTTTTTATA
GTTATACCTATTATAATTGGGGGATTCGGAAACTGATTAGTACCTTTAATATTAGGAGCCCCAGATATAGCTTTCCCCCG
AATAAATAACATAAGTTTTTGATTATTACCCCCTTCTTTAACCCTCTTAATCTCAAGAAGAATTGTTGAAAATGGGGCTG
GAACAGGATGAACTGTTTACCCGCCCTTATCCTCCAATATTGCCCACGGTGGGACTTCTGTAGATTTAGCTATTTTTTCA
CTACATTTAGCTGGAATTTCTTCTATTCTAGGGGCTATTAACTTTATTACAACTATTATTAATATACGAATTAATGGACT
ATCTTTTGATCAAATACCTTTATTCGTATGAGCAGTTGGTATTACTGCCCTTCTTCTTCTTCTCTCACTACCTGTATTAG
CAGGAGCTATCACTATATTATTAACAGATCGAAACTTAAATACCTCTTTCTTTGACCCAGCAGGA---------------
----
>MAMOT532-10|Scirpophaga_excerptalis|NIBGE_MOT-00532|HQ991222-SUPPRESSED
GACATTATATTTTATTTTTGGAATTTGAGCTGGTATAGTGGGAACTTCCCTTAGTTTACTAATTCGAGCCGAACTAGGTA
CTCCTGGATCACTAATTGGAGATGATCAAATCTATAATACTATTGTAACTGCTCACGCTTTTATTATAATTTTTTTTATA
GTTATACCTATTATAATTGGGGGATTCGGAAACTGATTAGTACCTTTAATATTAGGAGCTCCAGATATAGCTTTTCCCCG
AATAAACAACATAAGTTTTTGATTATTACCCCCTTCTTTAACCCTCTTAATCTCAAGAAGAATTGTTGAAAATGGAGCTG
GAACAGGATGAACTGTTTACCCGCCCCTATCCTCCAATATTGCCCACGGTGGGACTTCTGTAGATTTAGCTATTTTTTCA
TTACATTTAGCTGGAATTTCTTCTATTCTAGGGGCTATTAACTTCATTACAACTATTATTAATATACGAATTAATGGACT
ATCCTTTGATCAAATACCTTTATTCGTGTGAGCAGTTGGTATTACTGCCCTTCTTCTTCTTCTCTCACTACCTGTATTAG

CGGGAGCTATCACTATATTATTAACAGATCGAAACCTAAATACCTCTTTCTTTGACCCAGCAGGAGGGGGGGA-------
----
>MAMOT533-10|Scirpophaga_excerptalis|NIBGE_MOT-00533|HQ991223-SUPPRESSED
GACATTATATTTTATTTTTGGAATTTGAGCTGGTATAGTAGGAACTTCCCTTAGTTTACTAATTCGAGCCGAACTAGGTA
CTCCTGGATCACTAATTGGAGATGATCAAATCTATAATACTATTGTAACTGCTCACGCTTTTATTATAATTTTTTTATA
GTTATACCTATTATAATTGGAGGATTCGGAAACTGATTAGTACCTTTAATATTAGGAGCTCCAGATATAGCTTTTCCCCG
AATAAATAACATAAGTTTTTGATTATTGCCCCCTTCTTTAACCCTCTTAATCTCAAGAAGAATTGTTGAAAATGGAGCTG
GAACAGGATGAACTGTTTACCCGCCCCTATCCTCCAATATTGCCCACGGTGGGACTTCTGTAGATTTAGCTATTTTTTCA
TTACATTTAGCTGGAATTTCTTCTATTCTAGGGGCTATTAACTTCATTACAACTATTATTAATATGCGAATTAATGGACT
ATCCTTTGATCAAATACCTTTATTCGTGTGAGCAGTTGGTATTACTGCCCTTCTTCTTCTTCTCTCACTACCTGTATTAG
CGGGAGCTATCACTATATTATTAACAGATCGAAACTTAAATACCTCTTTCTTTGACCCAGCAGGAGGGGGGGACCCAATT
CTTT-----------------------
>MAMOT534-10|Scirpophaga_excerptalis|NIBGE_MOT-00534|HQ991224-SUPPRESSED
GACATTATATTTTATTTTTGGAATTTGAGCTGGTATAGTAGGAACTTCACTTAGTTTACTAATTCGAGCCGAATTAGGTA
CTCCTGGATCACTAATTGGAGATGATCAAATCTATAATACTATTGTAACTGCTCACGCTTTTATTATAATTTTTTTATA
GTTATACCTATTATAATTGGGGGATTCGGAAACTGATTAGTACCTTTAATATTAGGAGCTCCAGATATGGCTTTCCCCCG
AATAAATAACATAAGTTTTTGATTATTACCCCCTTCTTTAACCCTCTTAATCTCAAGAAGAATTGTTGAAAATGGGGCTG
GAACAGGATGAACTGTTTACCCGCCCCTATCCTCCAATATTGCCCACGGTGGGACTTCTGTAGATTTAGCTATTTTTTCA
TTACATTTAGCTGGAATTTCTTCTATTCTAGGGGCTATTAACTTCATTACAACTATTATTAATATGCGAATTAATGGACT
ATCTTTTGATCAAATACCTTTATTCGTGTGAGCAGTTGGTATTACTGCCCTTCTTCTTCTTCTCTCACTACCTGTATTAG
CAGGAGCTATCACTATATTATTAACAGATCGAAACTTAAATACCTCTTTCTTTGACCCAGCAGGAGGTGGGGACCCAATT
CTTTATAAACATTTATTT---------
>MAMOT535-10|Scirpophaga_excerptalis|NIBGE_MOT-00535|HQ991225-SUPPRESSED
GACATTATATTTTATTTTTGGAATTTGAGCTGGTATAGTGGGAACTTCCCTTAGTTTACTAATTCGAGCCGAACTAGGTA
CTCCTGGATCACTAATTGGAGATGATCAAATCTATAATACTATTGTAACTGCTCACGCTTTTATTATAATTTTTTTATA
GTTATACCTATTATAATTGGGGGATTCGGAAACTGATTAGTACCTTTAATATTAGGGGCTCCAGATATAGCTTTTCCCCG
AATAAACAACATAAGTTTTTGATTATTACCCCCTTCTTTAACCCTCTTAATCTCAAGAAGAATTGTTGAAAATGGAGCTG
GAACAGGATGAACTGTTTACCCGCCTCTATCCTCCAATATTGCCCACGGTGGGACTTCTGTAGATTTAGCTATTTTTTCA
TTACATTTAGCTGGAATTTCTTCTATTCTAGGGGCTATTAACTTCATTACAACTATTATTAATATGCGAATTAATGGACT
ATCCTTTGATCAAATACCTTTATTCGTATGAGCAGTTGGTATTACTGCCCTTCTTCTTCTTCTCTCACTACCTGTATTAG
CGGGAGCTATCACTATATTATTAACAGATCGAAACCTAAATACCTCTTTCTTTGACCCAGCAGGAGGGGGGGACCCAATT
CTTTA----------------------
>MAMOT536-10|Scirpophaga_excerptalis|NIBGE_MOT-00536|HQ991226-SUPPRESSED
GACATTATATTTTATTTTTGGAATTTGAGCTGGTATAGTAGGAACTTCCCTTAGTTTACTAATTCGAGCCGAACTAGGTA
CTCCTGGATCACTAATTGGAGATGATCAAATCTATAATACTATTGTAACTGCTCACGCTTTTATTATAATTTTTTTATA
GTTATACCTATTATAATTGGAGGATTCGGAAACTGATTAGTACCTTTAATATTAGGAGCTCCAGATATAGCTTTTCCCCG
AATAAATAACATAAGTTTTTGATTATTGCCCCCTTCTTTAACCCTCTTAATCTCAAGAAGAATTGTTGAAAATGGAGCTG
GAACAGGATGAACTGTTTACCCGCCCCTATCCTCCAATATTGCCCACGGTGGGACTTCTGTAGATTTAGCTATTTTTTCA
TTACATTTAGCTGGAATTTCTTCTATTCTAGGGGCTATTAACTTCATTACAACTATTATTAATATGCGAATTAATGGACT
ATCCTTTGATCAAATACCTTTATTCGTGTGAGCAGTTGGTATTACTGCCCTTCTTCTTCTTCTCTCACTACCTGTATTAG
CGGGAGCTATCACTATATTATTAACAGATCGAAACTTAAATACCTCTTTCTTTGACCCAGCAGGAGGGGGGGACCCAATT
CTTTATCAACATTTATTT---------
>MAMOT546-10|Sesamia_inferens|NIBGE_MOT-00546|HQ991235-SUPPRESSED
AACATTATATTTTATTTTTGGGATTTGAGCTGGTATAGTAGGAACATCATTAAGATTATTAATTCGAGCTGAATTAGGAA
CCCCAGGATCTTTAATTGGAGATGATCAAATTTATAATACTATTGTTACAGCCCATGCTTTTATTATAATTTTTTTTATA
GTTATACCAATTATAATTGGAGGATTTGGAAATTGACTTGTACCTTTAATATTAGGAGCTCCTGATATAGCATTTCCACG
AATAAATAATATAAGATTTTGATTATTACCCCCCTCTTTAACTCTTTTAATTTCAAGTAGAATTGTAGAAAATGGAGCAG
GAACTGGATGAACAGTGTACCCCCCACTTTCATCTAATATTGCTCATGGAGGAAGATCAGTAGATCTAGCTATTTTTTCC
CTTCATTTAGCTGGTATTTCATCTATTTTAGGAGCTATTAATTTTATTACAACAATTATCAATATACGACTAAATAGTTT
ATCTTTTGATCAAATACCTCTATTTATTTGAGCTGTTGGAATTACTGCATTTTTATTATTATTATCTTTACCTGTATTAG
CAGGAGCTATTACAATATTATTGACAGATCGAAATTTAAATACATCATTTTTTGACCCCGCAGGAGGGGGTGATCCTATT
TTATACCAACATTTATTT---------
>MAMOT575-10|Chilo_partellus|NIBGE_MOT-00575|HQ991263-SUPPRESSED
AACTTTATATTTTATTTTTGGAATTTGAGCAGGAATAATTGGAACATCCCTTAGATTATTAATTCGTGCAGAATTAGGAA
CTCCTGGATCTTTAATTGGAGATGATCAAATTTATAATACTATTGTAACAGCACATGCATTTATTATAATTTTTTTTATA
GTTATACCAATTATAATTGGTGGATTTGGAAATTGATTAGTACCTTTAATATTAGGAGCCCCAGATATAGCTTTCCCACG
AATAAATAATATAAGATTTTGATTATTACCACCATCATTAACTTTACTAATTTCTAGAAGAATTGTTGAAAATGGAGCTG

GAACAGGATGAACAGTGTACCCCCCACTATCATCTAATATTGCTCATGCTGGAAGTTCAGTAGATTTAGCAATTTTTTCT
TTACATTTAGCTGGTATTTCATCAATTCTTGGTGCTATTAATTTTATTACAACAATCATTAATATACGAATTAATGGATT
ATTTTTTGATCAAATACCATTATTTGTTTGATCTGTAGGTATTACAGCTTTATTATTATTACTTTCTTTACCTGTTTTAG
CTGGAGCTATTACTATATTATTAACAGATCGAAATTTAAATACATCCTTTTTCGATCCTGCTGGAGGAGGAGATCCTATT
CTTTATCAACACTTATTT---------
>MAMOT576-10|Emmalocera_sp.|NIBGE_MOT-00576|HQ991264-SUPPRESSED
AACTTTATATTTTATTTTTGGAATTTGATCAGGTATAGTAGGTACTTCTTTAAGTCTTCTTATTCGAGCTGAATTAGGAA
CTCCTAGATCTTTAATTGGAGATGACCAAATTTATAATACTATTGTTACTGGGCATGCTTTTATTATAATTTTTTTTATA
GTTATACCTATTATAATTGGAGGATTTGGAAATTGATTAGTTCCTTTAATATTAGGAGCCCCAGATATAGCTTTCCCTCG
AATAAATAATATAAGATTTTGACTTTTACCTCCCTCTCTTAATTTATTAATTTCTAGAAGAATTGTAGAAAATGGAGCTG
GAACAGGTTGAACAGTTTATCCCCCTTTATCTTCTAATATTGCTCATAGTGGAAGTTCTGTTGATCTTGCTATTTTTTCT
TTACATCTAGCAGGAATTTCTTCTATTTTAGGTGCTATTAATTTTATTACTACTATTATTAATATAAAATTAAATGGTTT
ATCTTTTGATCAAATACCTTTATTTGTTTGAGCTGTAGGAATTACAGCTTTATTATTACTTTTATCCTTGCCAGTATTAG
CAGGAGCTATTACTATATTATTAACTGACCGAAATTTAAATACTTCTTTCTTTGACCCTGCAGGAGGAGGAGACCCAATT
TTATACCAACATTTATTT---------
>MAMOT580-10|Chilo_partellus|NIBGE_MOT-00580|HQ991268-SUPPRESSED
AACTTTATATTTTATTTTTGGAATTTGAGCAGGAATAATTGGAACATCCCTTAGATTATTAATTCGTGCAGAATTAGGAA
CTCCTGGATCTTTAATTGGAGATGATCAAATTTATAATACTATTGTAACAGCACATGCATTTATTATAATTTTTTTTATA
GTTATACCAATTATAATTGGTGGATTTGGAAATTGATTAGTACCTTTAATATTAGGAGCCCCAGATATAGCTTTCCCACG
AATAAATAATATAAGATTTTGATTATTACCACCATCATTAACTTTACTAATTTCTAGAAGAATTGTTGAAAATGGAGCTG
GAACAGGATGAACAGTGTACCCCCCACTATCATCTAATATTGCTCATGCTGGAAGTTCAGTAGATTTAGCAATTTTTTCT
TTACATTTAGCTGGTATTTCATCAATTCTTGGTGCTATTAATTTTATTACAACAATCATTAATATACGAATTAATGGATT
ATTTTTTGATCAAATACCATTATTTGTTTGATCTGTAGGTATTACAGCTTTATTATTATTACTTTCTTTACCTGTTTTAG
CTGGAGCTATTACTATATTATTAACAGATCGAAATTTAAATACATCCTTTTTCGATCCTGCTGGAGGAGGAGATCCTATT
CTTTATCAACACTTATTT---------
>MAMOT594-10|Scirpophaga_incertulas|NIBGE_MOT-00594|HQ991281-SUPPRESSED
TACTTTATATTTTATTTTTGGAATTTGAGCTGGTATAGTAGGAACTTCTTTAAGCTTACTTATTCGAGCTGAATTAGGAA
CTTCTGGATCCTTAATTGGAGATGATCAAATCTATAACACTATTGTCACAGCCCATGCCTTTATTATAATTTTTTTTATA
GTTATACCCATTATAATTGGAGGATTTGGAAATTGATTAGTCCCCCTAATATTAGGAGCCCCAGATATAGCTTTCCCCCG
ATTAAATAACATAAGATTCTGATTATTACCCCCCTCTTTAACACTCCTCATTTCTAGAAGAATTGTAGAAAATGGAGCTG
GAACAGGATGAACTGTTTACCCACCCCTATCATCCAATATTGCTCATGGGGGAACATCAGTAGATTTAGCTATTTTTTCA
CTACACCTAGCAGGAATTTCATCTATTTTAGGAGCTATTAATTTTATTACAACCATTATTAATATACGAATTAATGGATT
ATCATTTGACCAAATACCTCTATTTGTGTGAGCTGTTGGTATTACAGCCCTTCTTCTACTCCTCTCTCTCCCAGTTTTAG
CTGGAGCTATTACTATGTTATTAACAGATCGAAATTTAAATACATCTTTTTTGACCCAGCTGGGGGAGGAGATCCAATT
TTATATCAACATTTATTT---------
>MAMOT595-10|Scirpophaga_excerptalis|NIBGE_MOT-00595|HQ991282-SUPPRESSED
GACATTATATTTTATTTTTGGAATTTGAGCTGGTATAGTGGGAACTTCACTTAGTTTACTAATTCGAGCCGAATTAGGTA
CTCCTGGATCACTAATTGGAGATGATCAAATCTATAATACTATTGTAACTGCTCACGCTTTTATTATAATTTTTTTTATA
GTTATACCTATTATAATTGGGGGATTCGGAAACTGATTAGTACCTTTAATATTAGGAGCTCCAGATATGGCTTTCCCCCG
AATAAATAACATAAGTTTTTGATTATTACCCCCTTCTTTAACCCTCTTAATCTCAAGAAGAATTGTTGAAAATGGGGCTG
GAACAGGATGAACTGTTTACCCGCCCCTATCCTCCAATATTGCCCACGGTGGGACTTCTGTAGATTTAGCTATTTTTTCA
TTACATTTAGCTGGAATTTCTTCTATTCTAGGGGCTATTAACTTCATTACAACTATTATTAATATGCGAATTAATGGACT
ATCTTTTGATCAAATACCTTTATTCGTGTGAGCAGTTGGTATTACTGCCCTTCTTCTTCTTCTCTCACTACCTGTATTAG
CAGGAGCTATCACTATATTATTAACAGATCGAAACTTAAATACCTCTTTCTTTGACCCAGCAGGAGGTGGGGACCCAATT
CTTTATCAACATTTATTT---------
>MAMOT596-10|Scirpophaga|NIBGE_MOT-00596|HQ991283-SUPPRESSED
AACATTATATTTTATTTTTGGAATTTGAGCGGGTATAGTGGGAACTTCTTTAAGTTTATTAATTCGAGCTGAATTAGGAA
CTCCAGGATCATTAATTGGAGATGATCAAATTTATAATACTATTGTTACAGCTCATGCTTTTATTATAATTTTTTTCATA
GTTATACCTATTATAATTGGAGGATTTGGTAATTGACTTGTACCTTTAATACTAGGAGCTCCGGATATAGCTTTCCCTCG
AATAAATAATATAAGATTTTGATTATTACCTCCCTCCCTTACTCTTTTAATTTCAAGAAGAATTGTTGAAAATGGAGCTG
GAACAGGTTGAACTGTTTATCCACCTCTATCATCTAATATTGCCCATGGGGGAACCTCAGTAGATTTAGCTATTTTCTCT
TTACATTTAGCAGGTATATCCTCTATTCTTGGAGCTATTAACTTTATTACAACTATTATTAATATAAAAATTAATGGATT
ATCTTTTGACCAAATACCTTTATTTGTTTGAGCTGTAGGGATTACGGCTCTTCTTCTTCTTTTATCTTTACCTGTTTTAG
CTGGAGCTATTACAATATTATTAACAGATCGAAATTTAAATACTTCTTTCTTTGACCCAGCTGGGGGAGGAGATCCTATT
CTTTATCAACATCTCTTT---------
>MAMOT598-10|Chilo_partellus|NIBGE_MOT-00598|HQ991285-SUPPRESSED
AACTTTATATTTTATTTTTGGAATTTGAGCAGGAATAATTGGAACATCCCTTAGATTATTAATTCGTGCAGAATTAGGAA

CTCCTGGATCTTTAATTGGAGATGATCAAATTTATAATACTATTGTAACAGCACATGCATTTATTATAATTTTTTTTATA
GTTATACCAATTATAATTGGTGGATTTGGAAATTGATTAGTACCTTTAATATTAGGAGCCCCAGATATAGCTTTCCCACG
AATAAATAATATAAGATTTTGATTATTACCACCATCATTAACTTTACTAATTTCTAGAAGAATTGTTGAAAATGGAGCTG
GAACAGGATGAACAGTGTACCCCCCACTATCATCTAATATTGCTCATGCTGGAAGTTCAGTAGATTTAGCAATTTTTTCT
TTACATTTAGCTGGTATTTCATCAATTCTTGGTGCTATTAATTTTATTACAACAATCATTAATATACGAATTAATGGATT
ATTTTTTGATCAAATACCATTATTTGTTTGATCTGTAGGTATTACAGCTTTATTATTATTACTTTCTTTACCTGTTTTAG
CTGGAGCTATTACTATATTATTAACAGATCGAAATTTAAATACATCCTTTTTCGATCCTGCTGGAGGAGGAGATCCTATT
CTTTATCAACACTTATTT---------
>MAMOT599-10|Chilo_partellus|NIBGE_MOT-00599|HQ991286-SUPPRESSED
AACTTTATATTTTATTTTTGGAATTTGAGCAGGAATAATTGGAACATCCCTTAGATTATTAATTCGTGCAGAATTAGGAA
CTCCTGGATCTTTAATTGGAGATGATCAAATTTATAATACTATTGTAACAGCACATGCATTTATTATAATTTTTTTTATA
GTTATACCAATTATAATTGGTGGATTTGGAAATTGATTAGTACCTTTAATATTAGGAGCCCCAGATATAGCTTTCCCACG
AATAAATAATATAAGATTTTGATTATTACCACCATCATTAACTTTACTAATTTCTAGAAGAATTGTTGAAAATGGAGCTG
GAACAGGATGAACAGTGTACCCCCCACTATCATCTAATATTGCTCATGCTGGAAGTTCAGTAGATTTAGCAATTTTTTCT
TTACATTTAGCTGGTATTTCATCAATTCTTGGTGCTATTAATTTTATTACAACAATCATTAATATACGAATTAATGGATT
ATTTTTTGATCAAATACCATTATTTGTTTGATCTGTAGGTATTACAGCTTTATTATTATTACTTTCTTTACCTGTTTTAG
CTGGAGCTATTACTATATTATTAACAGATCGAAATTTAAATACATCCTTTTTCGATCCTGCTGGAGGAGGAGATCCTATT
CTTTATCAACACTTATTT---------
>MAMOT648-10|Emmalocera_sp.|NIBGE_MOT-00648|HQ991333-SUPPRESSED
AACTTTATATTTTATTTTTGGAATTTGATCAGGTATAGTAGGTACTTCTTTAAGTCTTCTTATTCGAGCTGAATTAGGAA
CTCCTAGATCTTTAATTGGAGATGATCAAATTTATAATACTATTGTTACTGGACATGCTTTTATTATAATTTTTTTTATA
GTTATACCTATTATAATTGGAGGATTTGGAAATTGATTAGTTCCTTTAATATTAGGAGCCCCAGATATAGCTTTCCCTCG
AATAAATAATATAAGATTTTGACTTTTACCTCCCTCTCTTAATTTATTAATTTCTAGAAGAATTGTAGAAAATGGAGCTG
GAACAGGTTGAACAGTTTATCCCCCTTTATCTTCTAATATTGCTCATAGTGGGAGTTCTGTTGATCTTGCTATTTTTTCT
TTACATCTAGCAGGAATTTCTTCTATTTTAGGTGCTATTAATTTTATTACTACTATTATTAATATAAAATTAAATGGTTT
ATCTTTTGATCAAATACCTTTATTTGTTTGAGCTGTAGGAATTACAGCTTTATTATTACTTTTATCCTTGCCAGTATTAG
CAGGAGCTATTACTATATTATTAACTGACCGAAATTTAAATACTTCTTTCTTTGACCCTGCAGGAGGAGGAGACCCAATT
TTATATCAACATTTATTT---------
>MAMOT654-10|Chilo_partellus|NIBGE_MOT-00654|HQ991339-SUPPRESSED
AACTTTATATTTTATTTTTGGAATTTGAGCAGGAATAATTGGAACATCCCTTAGATTATTAATTCGTGCAGAATTAGGAA
CTCCTGGATCTTTAATTGGAGATGATCAAATTTATAATACTATTGTAACAGCACATGCATTTATTATAATTTTTTTTATA
GTTATACCAATTATAATTGGTGGATTTGGAAATTGATTAGTACCTTTAATATTAGGAGCCCCAGATATAGCTTTCCCACG
AATAAATAATATAAGATTTTGATTATTACCACCATCATTAACTTTACTAATTTCTAGAAGAATTGTTGAAAATGGAGCTG
GAACAGGATGAACAGTGTACCCCCCACTATCATCTAATATTGCTCATGCTGGAAGTTCAGTAGATTTAGCAATTTTTTCT
TTACATTTAGCTGGTATTTCATCAATTCTTGGTGCTATTAATTTTATTACAACAATCATTAATATACGAATTAATGGATT
ATTTTTTGATCAAATACCATTATTTGTTTGATCTGTAGGTATTACAGCTTTATTATTATTACTTTCTTTACCTGTTTTAG
CTGGAGCTATTACTATATTATTAACAGATCGAAATTTAAATACATCCTTTTTCGATCCTGCTGGAGGAGGAGATCCTATT
CTTTATCAACACTTATTT---------
>MAMOT655-10|Emmalocera_sp.|NIBGE_MOT-00655|HQ991340-SUPPRESSED
AACTTTATATTTTATTTTTGGAATTTGATCAGGTATAGTAGGTACTTCTTTAAGTCTTCTTATTCGAGCTGAATTAGGAA
CTCCTAGATCTTTAATTGGAGATGATCAAATTTATAATACTATTGTTACTGGACATGCTTTTATTATAATTTTTTTTATA
GTTATACCTATTATAATTGGAGGATTTGGAAATTGATTAGTTCCTTTAATATTAGGAGCCCCAGATATAGCTTTCCCTCG
AATAAATAATATAAGATTTTGACTTTTACCTCCCTCTCTTAATTTATTAATTTCTAGAAGAATTGTAGAAAATGGAGCTG
GAACAGGTTGAACAGTTTATCCCCCTTTATCTTCTAATATTGCTCATAGTGGAAGTTCTGTTGATCTTGCTATTTTTTCT
TTACATCTAGCAGGAATTTCTTCTATTTTAGGTGCTATTAATTTTATTACTACTATTATTAATATAAAATTAAATGGTTT
ATCTTTTGATCAAATACCTTTATTTGTTTGAGCTGTAGGAATTACAGCTTTATTATTACTTTTATCCTTGCCAGTTTTAG
CAGGAGCTATTACTATATTATTAACTGACCGAAATTTAAATACTTCTTTCTTTGACCCTGCAGGAGGAGGAGACCCAATT
TTATATCAACATTTATTT---------
>MAMOT656-10|Sesamia|NIBGE_MOT-00656|HQ991341-SUPPRESSED
-------------------------TGAGCTGGTATAGTAGGAACTTCATTAAGATTATTAATTCGAGCTGAATTAGGAA
TTCCTGGATCTTTAATTGGGGATGATCAAATTTATAACACTATTGTTACAGCCCATGCTTTTATTATAATTTTTTTTATA
GTTATACCAATTATAATTGGGGGATTTGGTAATTGACTTGTACCTTTAATATTAGGAGCTCCAGATATAGCATTCCCGCG
AATAAATAATATAAGATTTTGATTATTACCCCCCTCCTTAACTTTATTAATTTCAAGTAGAATTGTAGAAAATGGGGCAG
GTACAGGATGAACAGTATATCCACCTCTCTCATCTAATATTGCCCATGGGGGAAGATCAGTAGACTTAGCTATTTTTTCT
CTTCATTTAGCAGGTATTTCATCTATTTTAGGAGCTATTAATTTTATTACAACAATTATTAATATACGATTAAATAGATT
ATCCTTTGATCAAATACCTTTATTTGTTTGAGCTGTTGGAATTACTGCATTTTTATTATTATTATCTTTACCTGTTTTAG
CGGGAGCTATTACAATGTTATTAACAGATCGAAACTTAAATACATCCTTCTTTGACCCTGCGGGAGGAGGTGATCCAATT

CTATATCAACATTTATTT---------
>MAMOT710-10|Scirpophaga_excerptalis|NIBGE_MOT-00710|HQ991394-SUPPRESSED
AACATTATATTTTATTTTTGGAATTTGAGCTGGTATAGTGGGAACTTCCCTTAGTTTACTAATTCGAGCCGAACTAGGTA
CTCCTGGATCACTAATTGGAGATGATCAAATCTATAATACTATTGTAACTGCTCACGCTTTTATTATAATTTTTTTATA
GTTATACCTATTATAATTGGGGGATTCGGAAACTGATTAGTACCTTTAATATTAGGAGCTCCAGATATAGCTTTCCCCG
AATAAACAACATAAGTTTTTGATTATTACCCCCTTCTTTAACCCTCTTAATCTCAAGAAGAATTGTTGAAAATGGAGCTG
GAACAGGATGAACTGTTTACCCGCCCCTATCCTCCAATATTGCCCACGGTGGGACTTCTGTAGATTTAGCTATTTTTTCA
TTACATTTAGCTGGAATTTCTTCTATTCTAGGGGCTATTAACTTCATTACAACTATTATTAATATGCGAATTAATGGACT
ATCCTTTGATCAAATACCTTTATTCGTGTGAGCAGTTGGTATTACTGCCCTTCTTCTTCTTCTCTCACTACCTGTATTAG
CGGGAGCTATCACTATATTATTAACAGATCGAAACCTAAATACCTCTTTCTTTGACCCAGCAGGAGGGGGGGACCCAATT
CTTTATCAACATTTATTT---------
>MAMOT957-10|Sesamia_inferens|NIBGE_MOT-00957|JN988542
AACATTATATTTTATTTTTGGAATTTGAGCTGGTATAGTAGGAACATCATTAAGATTATTAATTCGAGCTGAATTAGGAA
CCCCAGGATCTTTAATTGGAGATGATCAAATTTATAATACTATTGTTACAGCTCATGCTTTTATTATAATTTTTTTTATA
GTTATACCAATTATAATTGGAGGATTTGGAAATTGACTTGTACCTTTAATATTAGGAGCTCCTGATATAGCATTTCCACG
AATAAATAATATAAGATTTTGATTATTACCCCCCTCTTTAACTCTTTTAATTTCAAGTAGAATTGTAGAAAATGGAGCAG
GAACTGGGTGAACAGTGTACCCCCCACTTTCATCTAATATTGCCCATGGAGGAAGATCAGTAGATCTAGCTATTTTTTCC
CTTCATTTAGCTGGTATTTCATCTATTTTAGGAGCTATTAATTTTATTACAACAATTATCAATATACGATTAAATAGTTT
ATCTTTTGATCAAATACCTCTATTTATTTGAGCTGTTGGAATTACTGCATTTTTATTATTATTATCTTTACCTGTATTAG
CAGGAGCTATTACAATATTATTGACAGATCGAAATTTAAATACATCATTCTTTGACCCCGCAGGAGGAGGTGATCCTATT
TTATACCAACATTTATTT---------
>MAMOT1164-11|Scirpophaga|NIBGE_MOT-01164|KX860174
AACTTTATATTTCATTTTTGGTATTTGAGCTGGTATAGTAGGAACTTCCCTTAGTTTATTAATTCGAGCTGAATTAGGAA
CTCCAGGGTCATTAATTGGAGATGATCAAATTTATAATACTATTGTAACTGCTCATGCTTTTATTATAATTTTTTTTATA
GTTATACCTATTATAATTGGAGGATTTGGAAATTGATTAGTACCCCTAATATTAGGAGCTCCTGATATAGCCTTCCCTCG
AATAAATAATATGAGTTTTTGATTATTACCCCCTTCTTTAACTCTCTTAATTTCAAGAAGAATTGTTGAAAATGGTGCTG
GAACAGGATGAACTGTTTACCCCCCCTTTATCTTCTAATATTGCCCATGGCGGAACTTCTGTAGATTTAGCTATTTTCTCT
TTACACCTAGCTGGAATTTCTTCTATTTTAGGAGCTATTAACTTTATTACAACTATTATTAACATACGAATTAATGGGTT
ATCTTTTGATCAAATACCTTTATTCGTTTGAGCAGTTGGTATTACAGCACTTCTTCTTCTTCTTTCATTACCTGTACTTG
CAGGAGCTATTACAATACTTTTAACTGATCGAAATTTAAATACTTCTTTTTTTGACCCAGCGGGAGGAGGAGATCCAATT
CTTTAT---------------------
>MAMOT1609-12|Scirpophaga|NIBGE_MOT-01514|KX863055
AACTTTATATTTCATTTTTGGTATTTGAGCTGGTATAGTAGGAACTTCCCTTAGTTTATTAATTCGAGCTGAATTAGGGA
CTCCAGGGTCATTAATTGGAGATGATCAAATTTATAATACTATTGTAACTGCTCATGCTTTTATTATAATTTTTTTTATA
GTTATACCTATTATAATTGGAGGATTTGGAAATTGACTAGTACCCCTAATATTAGGAGCTCCTGATATAGCCTTCCCTCG
AATAAATAATATAAGTTTTTGATTATTACCCCCTTCTTTAACTCTCTTAATTTCAAGAAGAATTGTTGAAAATGGTGCTG
GAACAGGATGAACTGTTTACCCCCCCTTTATCTTCTAATATTGCCCATGGCGGAACTTCTGTAGATTTAGCTATTTTCTCT
TTACACCTAGCTGGAATTTCTTCTATTTTAGGAGCTATTAACTTTATTACAACTATTATTAACATACGAATTAATGGTTT
ATCTTTTGATCAAATACCTTTATTCGTTTGAGCAGTTGGTATTACAGCACTTCTTCTTCTTCTTTCATTACCTGTACTTG
CAGGAGCTATTACAATACTTTTAACTGATCGAAATTTAAATACTTCTTTTTTTGATCCAGCGGGAGGAGGAGATCCAATT
CTTTATCAACATTTATTT---------
>MAMOT1611-12|Scirpophaga|NIBGE_MOT-01516|KX861379
AACTTTATATTTCATTTTTGGTATCTGAGCTGGTATAGTAGGAACTTCCCTTAGTTTATTAATTCGAGCTGAATTAGGAA
CTCCAGGGTCATTAATTGGAGATGATCAAATTTATAATACTATTGTAACTGCTCATGCTTTTATTATAATTTTTTTTATA
GTTATACCTATTATAATTGGAGGATTTGGAAATTGACTAGTACCCCTAATATTAGGAGCTCCTGATATAGCCTTCCCTCG
AATAAATAATATAAGTTTTTGATTATTACCCCCTTCTTTAACTCTCTTAATTTCAAGAAGAATTGTTGAAAATGGTGCTG
GAACAGGATGAACTGTTTACCCCCCCTTTATCTTCTAATATTGCCCATGGCGGAACTTCTGTAGATTTAGCTATTTTCTCT
TTACACCTAGCTGGAATTTCTTCTATTTTAGGAGCTATTAACTTTATTACAACTATTATTAACATACGAATTAATGGTTT
ATCTTTTGATCAAATACCTTTATTCGTTTGAGCAGTTGGTATTACAGCACTTCTTCTTCTTCTTTCATTACCTGTACTTG
CAGGAGCTATTACAATACTTTTAACTGATCGAAATTTAAATACTTCTTTTTTTGATCCAGCGGGAGGAGGAGATCCAATT
CTTTATCAACATTTATTT---------
>MAMOT1612-12|Scirpophaga|NIBGE_MOT-01517|KX861288
AACTTTATATTTCATTTTTGGTATCTGAGCTGGTATAGTAGGAACTTCCCTTAGTTTATTAATTCGAGCTGAATTAGGAA
CTCCAGGGTCATTAATTGGAGATGATCAAATTTATAATACTATTGTAACTGCTCATGCTTTTATTATAATTTTTTTTATA
GTTATACCTATTATAATTGGAGGATTTGGAAATTGACTAGTACCCCTAATATTAGGAGCTCCTGATATAGCCTTCCCTCG
AATAAATAATATAAGTTTTTGATTATTACCCCCTTCTTTAACTCTCTTAATTTCAAGAAGAATTGTTGAAAATGGTGCTG
GAACAGGATGAACTGTTTACCCCCCCTTTATCTTCTAATATTGCCCATGGCGGAACTTCTGTAGATTTAGCTATTTTCTCT

TTACACCTAGCTGGAATTTCTTCTATTTTAGGAGCTATTAACTTTATTACAACTATTATTAACATACGAATTAATGGTTT
ATCTTTTGATCAAATACCTTTATTCGTTTGAGCAGTTGGTATTACAGCACTTCTTCTTCTTCTTTCATTACCTGTACTTG
CAGGAGCTATTACAATACTTTTAACTGATCGAAATTTAAATACTTCTTTTTTTGATCCAGCGGGAGGAGGAGATCCAATT
CTTTATCAACATTTATTT---------
>MAMOT1613-12|Scirpophaga|NIBGE_MOT-01518|KX862930
AACTTTATATTTCATTTTTGGTATTTGAGCTGGTATAGTAGGAACTTCCCTTAGTTTATTAATTCGAGCTGAATTAGGGA
CTCCAGGGTCATTAATTGGAGATGATCAAATTTATAATACTATTGTAACTGCTCATGCTTTTATTATAATTTTTTTTATA
GTTATACCTATTATAATTGGAGGATTTGGAAATTGACTAGTACCCCTAATATTAGGAGCTCCTGATATAGCCTTCCCTCG
AATAAATAATATAAGTTTTTGATTATTACCCCCTTCTTTAACTCTCTTAATTTCAAGAAGAATTGTTGAAAATGGTGCTG
GAACAGGATGAACTGTTTACCCCCCTTTATCTTCTAATATTGCCCATGGCGGAACTTCTGTAGATTTAGCTATTTTCTCT
TTACACCTAGCTGGAATTTCTTCTATTTTAGGAGCTATTAATTTTATTACAACTATTATTAACATACGAATTAATGGTTT
ATCTTTTGATCAAATACCTTTATTCGTTTGAGCAGTTGGTATTACAGCACTTCTTCTTCTTCTTTCATTACCTGTACTTG
CAGGAGCTATTACAATACTTTTAACTGATCGAAATTTAAATACTTCTTTTTTTGATCCAGCGGGGGGAGGAGATCCAATT
CTTTATCAACATTTATTT---------
>MAMOT1614-12|Scirpophaga|NIBGE_MOT-01519|KX861838
AACTTTATATTTCATTTTTGGTATTTGAGCTGGTATAGTAGGAACTTCCCTTAGTTTATTAATTCGAGCTGAATTAGGAA
CTCCAGGGTCATTAATTGGAGATGATCAAATTTATAATACTATTGTAACTGCTCATGCTTTTATTATAATTTTTTTTATA
GTTATACCTATTATAATTGGAGGATTTGGAAATTGACTAGTACCCCTAATATTAGGAGCTCCTGATATAGCCTTCCCTCG
AATAAATAATATAAGTTTTTGATTATTACCCCCTTCTTTAACTCTCTTAATTTCAAGAAGAATTGTTGAAAATGGTGCTG
GAACAGGATGAACTGTTTACCCCCCTTTATCTTCTAATATTGCCCATGGCGGAACTTCTGTAGATTTAGCTATTTTCTCT
TTACACCTAGCTGGAATTTCTTCTATTTTAGGAGCTATTAACTTTATTACAACTATTATTAACATACGAATTAATGGTTT
ATCTTTTGATCAAATACCTTTATTCGTTTGAGCAGTTGGTATTACGGCACTTCTTCTTCTTCTTTCATTACCTGTACTTG
CAGGAGCTATTACAATACTTTTAACTGATCGAAATTTAAATACTTCTTTTTTTGATCCAGCGGGAGGAGGAGATCCAATT
CTTTATCAACATTTATTT---------
>MAMOT1615-12|Scirpophaga|NIBGE_MOT-01520|KX861444
AACTTTATATTTCATTTTTGGTATTTGAGCTGGTATAGTAGGAACTTCCCTTAGTTTATTAATTCGAGCTGAATTAGGAA
CTCCAGGGTCATTAATTGGAGATGATCAAATTTATAATACTATTGTAACTGCTCATGCTTTTATTATAATTTTTTTTATA
GTTATACCTATTATAATTGGAGGATTTGGAAATTGACTAGTACCCCTAATATTAGGAGCTCCTGATATAGCCTTCCCTCG
AATAAATAATATAAGTTTTTGATTATTACCCCCTTCTTTAACTCTCTTAATTTCAAGAAGAATTGTTGAAAATGGTGCTG
GAACAGGATGAACTGTTTACCCCCCTTTATCTTCTAATATTGCCCATGGCGGAACTTCTGTAGATTTAGCTATTTTCTCT
TTACACCTAGCTGGAATTTCTTCTATTTTAGGAGCTATTAACTTTATTACAACTATTATTAACATACGAATTAATGGTTT
ATCTTTTGATCAAATACCTTTATTCGTTTGAGCAGTTGGTATTACAGCACTTCTTCTTCTTCTTTCATTACCTGTACTTG
CAGGAGCTATTACAATACTTTTAACTGATCGAAATTTAAATACTTCTTTTTTTGATCCAGCAGGAGGAGGAGATCCAATT
CTTTATCAACATTTATTT---------
>MAMOT1616-12|Scirpophaga|NIBGE_MOT-01521|KX860717
-----------------------------------TAGTAGGAACTTCCCTTAGTTTATTAATTCGAGCTGAATTAGGGA
CTCCAGGGTCATTAATTGGAGATGATCAAATTTATAATACTATTGTAACTGCTCATGCTTTTATTATAATTTTTTTTATA
GTTATACCTATTATAATTGGAGGATTTGGAAATTGACTAGTACCCCTAATATTAGGAGCTCCTGATATAGCCTTCCCTCG
AATAAATAATATAAGTTTTTGATTATTACCCCCTTCTTTAACTCTCTTAATTTCAAGAAGAATTGTTGAAAATGGTGCTG
GAACAGGATGAACTGTTTACCCCCCTTTATCTTCTAATATTGCCCATGGCGGAACTTCTGTAGATTTAGCTATTTTCTCT
TTACACCTAGCTGGAATTTCTTCTATTTTAGGAGCTATTAACTTTATTACAACTATTATTAACATACGAATTAATGGTTT
ATCTTTTGATCAAATACCTTTATTCGTTTGAGCAGTTGGTATTACAGCACTTCTTCTTCTTCTTTCATTACCTGTACTTG
CAGGAGCTATTACAATACTTTTAACTGATCGAAATTTAAATACTTCTTTTTTTGATCCAGCGGGAGGAGGAGATCCAATT
CTTTACCAACATTTATTT---------
>MAMOT1617-12|Scirpophaga|NIBGE_MOT-01522|KX861915
AACTTTATATTTCATTTTTGGTATTTGAGCTGGTATAGTAGGAACTTCCCTTAGTTTATTAATTCGAGCTGAATTAGGGA
CTCCAGGGTCATTAATTGGAGATGATCAAATTTATAATACTATTGTAACTGCTCATGCTTTTATTATAATTTTTTTTATA
GTTATACCTATTATAATTGGAGGATTTGGAAATTGACTAGTACCCCTAATATTAGGGGCTCCTGATATAGCCTTCCCTCG
AATAAATAATATAAGTTTTTGATTATTACCCCCTTCTTTAACTCTCTTAATTTCAAGAAGAATTGTTGAAAATGGTGCTG
GAACAGGATGAACTGTTTACCCCCCTTTATCTTCTAATATTGCCCATGGCGGAACTTCTGTAGATTTAGCTATTTTCTCT
TTACACCTAGCTGGAATTTCTTCTATTTTAGGAGCTATTAACTTTATTACAACTATTATTAACATACGAATTAATGGTTT
ATCTTTTGATCAAATACCTTTATTCGTTTGAGCAGTTGGTATTACAGCACTTCTTCTTCTTCTTTCATTACCTGTACTTG
CAGGAGCTATTACAATACTTTTAACTGATCGAAATTTAAATACTTCTTTTTTTGATCCAGCGGGAGGAGGAGATCCAATT
CTTTATCAACATTTATTT---------
>MAMOT3030-12|Scirpophaga|NIBGE_MOT-02935|KX863221
AACATTATATTTTATTTTTGGAATTTGAGCGGGTATAGTGGGAACTTCTTTAAGTTTATTAATTCGAGCTGAATTAGGAA
CCCCAGGATCATTAATTGGAGATGATCAAATTTATAATACTATTGTTACAGCTCATGCTTTTATTATAATTTTTTTCATA

GTTATACCTATTATAATTGGAGGATTTGGTAATTGACTTGTACCTTTAATACTAGGAGCTCCGGATATAGCTTTCCCTCG
AATAAATAATATAAGATTTTGATTATTACCTCCCTCCCTTACTCTTTTAATTTCAAGAAGAATTGTTGAAAATGGAGCTG
GAACAGGTTGAACTGTTTATCCACCTCTATCATCTAATATTGCCCATGGGGGAACCTCAGTAGATTTAGCTATTTTCTCT
TTACATTTAGCAGGTATATCCTCTATTCTTGGAGCTATTAACTTTATTACAACTATTATTAATATAAAAATTAATGGATT
ATCTTTTGACCAAATACCTTTATTTGTTTGAGCTGTAGGGATTACGGCTCTTCTTCTTCTTTTATCTTTACCTGTTTTAG
CTGGAGCTATTACAATATTATTAACAGATCGAAATTTAAATACTTCTTTCTTTGACCCAGCTGGGGGAGGAGATCCTATT
CTTTATCAACATCTCTTT---------
>MILEP011-09|Acrapex_relicta|09-NCCC-296|GU669257-SUPPRESSED
--------------------------------GTATAGTGGGAACTTCTTTGAGATTGCTAATTCGAGCTGAATTAGGAA
ATCCTGGATCTTTAATTGGTGATGATCAAATTTATAATACTATTGTTACAGCCCATGCTTTTATTATAATTTTTTTTATA
GTTATACCTATTATAATTGGAGGATTTGGAAATTGACTTGTACCTCTAATATTAGGAGCCCCAGATATAGCATTCCCACG
AATAAATAATATAAGTTTTTGGTTACTCCCTCCCTCATTAACTTTACTAATTTCAAGAAGAATTGTAGAAAATGGTGCAG
GAACAGGATGAACAGTGTACCCCCCACTTTCATCTAATATTGCTCATGGAGGAAGCTCCGTAGATTTAGCAATTTTTTCC
CTTCATTTAGCAGGTATTTCTTCTATTTTAGGAGCTATTAATTTTATTACCACAATTATTAATATACGATTAAATAATTT
ATCTTTTGATCAAATACCTTTATTTATTTGAGCTGTAGGAATTACTGCATTTTTATTATTATTATCACTACCTGTTTTAG
CAGGAGCCATTACAATATTACTAACAGATCGAAATCTAAATACATCATTTTTTGACCCAGCAGGAGGGGGAGATCCAATT
TTATATCAACATTTATTT---------
>MILEP036-09|Acrapex_relicta|09-NCCC-321|GU669209-SUPPRESSED
---TTCTTTGAGATTACTAATTCGAGCTGAATTAGGAA
ATCCTGGATCTTTAATTGGTGATGATCAAATTTATAATACTATTGTTACAGCCCATGCTTTTATTATAATTTTTTTTATA
GTTATACCTATTATAATTGGAGGATTTGGAAATTGACTTGTACCTCTAATATTAGGAGCCCCAGATATAGCATTCCCACG
AATAAATAATATAAGTTTTTGGTTACTCCCTCCCTCATTAACTTTACTAATTTCAAGAAGAATTGTAGAAAATGGTGCAG
GAACAGGATGAACAGTGTACCCCCCACTTTCATCTAATATTGCTCATGGAGGAAGCTCCGTAGATTTAGCAATTTTTTCC
CTTCATTTAGCAGGTATTTCTTCTATTTTAGGAGCTATTAATTTTATTACCACAATTATTAATATACGATTAAATAATTT
ATCTTTTGATCAAATACCTTTATTTATTTGAGCTGTAGGAATTACTGCATTTTTATTATTATTATCACTACCTGTTTTAG
CAGGAGCCATTACAATATTACTAACAGATCGAAATCTAAATACATCATTTTTTGACCCAGCAGGAGGGGGAGATCCAATT
TTATATCAACATTTATTT---------
>NLLEA1052-12|Chilo_phragmitella|RMNH.INS.539043|KX047866
AACTTTATATTTTATTTTTGGAATTTGAGCTGGAATAATTGGAACATCTCTTAGACTTTTAATTCGAGCTGAATTAGGAA
CTCCAGGATCACTAATTGGAGATGATCAAATTTATAATACTATTGTTACAGCTCATGCATTTATTATAATTTTTTTTATA
GTTATACCTATTATAATTGGTGGTTTTGGAAATTGATTAGTACCTTTAATATTAGGAGCCCCTGATATAGCTTTCCCACG
AATAAATAATATAAGATTTTGATTATTACCACCTTCATTAACCTTATTAATCTCTAGAAGAATTGTTGAAAATGGAGCTG
GAACAGGATGAACAGTGTACCCCCCACTCTCATCTAATATTGCTCATGCTGGAAGTTCAGTAGATTTAGCAATTTTTTCC
TTACATTTAGCTGGAATTTCATCAATTTTAGGTGCTATTAATTTTATTACAACAATTATTAATATACGAATTAATGGATT
ATCATTTGATCAAATACCCCTACTCATTTGAAGAATTGGCATTACAGCATTATTATTATTACTTTCTCTTCCAGTATTAG
CTGGTGCTATTACTATATTATTAACAGATCGAAATTTAAATACATCTTTTTTTGATCCAGCTGGAGGTGGAGATCCTATT
CTCTATCAACATTTATTT---------
>NLLEA1053-12|Chilo_phragmitella|RMNH.INS.539044|KX049438
AACTTTATATTTTATTTTTGGAATTTGAGCTGGAATAATTGGAACATCTCTTAGACTTTTAATTCGAGCTGAATTAGGAA
CTCCAGGATCACTAATTGGAGATGATCAAATTTATAATACTATTGTTACAGCTCATGCATTTATTATAATTTTTTTTATA
GTTATACCTATTATAATTGGTGGTTTTGGAAATTGATTAGTACCTTTAATATTAGGAGCCCCTGATATAGCTTTCCCACG
AATAAATAATATAAGATTTTGATTATTACCACCTTCATTAACCTTATTAATCTCTAGAAGAATTGTTGAAAATGGAGCTG
GAACAGGATGAACAGTGTACCCCCCACTCTCATCTAATATTGCTCATGCTGGAAGTTCAGTAGATTTAGCAATTTTTTCC
TTACATTTAGCTGGAATTTCATCAATTTTAGGTGCTATTAATTTTATTACAACAATTATTAATATACGAATTAATGGATT
ATCATTTGATCAAATACCCCTACTCATTTGAAGAATTGGCATTACAGCATTATTATTATTACTTTCTCTTCCAGTATTAG
CTGGTGCTATTACTATATTATTAACAGATCGAAATTTAAATACATCTTTTTTTGATCCAGCTGGAGGTGGAGATCCTATT
CTCTATCAACATTTATTT---------
>NLLEA1059-12|Chilo_phragmitella|RMNH.INS.539051|KX048950
AACTTTATATTTTATTTTTGGAATTTGAGCTGGAATAATTGGAACATCTCTTAGACTTTTAATTCGAGCTGAATTAGGAA
CTCCAGGATCACTAATTGGAGATGATCAAATTTATAATACTATTGTTACAGCTCATGCATTTATTATAATTTTTTTTATA
GTTATACCTATTATAATTGGTGGTTTTGGAAATTGATTAGTACCTTTAATATTAGGAGCCCCTGATATAGCTTTCCCACG
AATAAATAATATAAGATTTTGATTATTACCACCTTCATTAACCTTATTAATCTCTAGAAGAATTGTTGAAAATGGAGCTG
GAACAGGATGAACAGTGTACCCCCCACTCTCATCTAATATTGCTCATGCTGGAAGTTCAGTAGATTTAGCAATTTTTTCC
TTACATTTAGCTGGAATTTCATCAATTTTAGGTGCTATTAATTTTATTACAACAATTATTAATATACGAATTAATGGATT
ATCATTTGATCAAATACCCCTACTCATTTGAAGAATTGGCATTACAGCATTATTATTATTACTTTCTCTTCCAGTATTAG
CTGGTGCTATTACTATATTATTAACAGATCGAAATTTAAATACATCTTTTTTTGATCCAGCTGGAGGTGGAGATCCTATT
CTCTATCAACATTTATTT---------

>NSWBB073-08|Emmalocera_latilimbella|07-NSWBB-0073|
AACTTTATATTTTATTTTTGGAATTTGATCAGGAATAGTAGGAACATCTATAAGTTTACTTATTCGAGCTGAATTAGGAA
CTCCTGGATCTTTAATTGGAGATGACCAAATTTATAATACTATTGTTACTGGTCATGCTTTTATTATAATTTTTTTTATA
GTTATACCTATTATAATTGGCGGATTTGGAAATTGATTAGTTCCTTTAATATTAGGGGCCCCAGATATAGCTTTTCCTCG
AATAAATAATATAAGATTCTGACTCTTACCCCCTTCCCTTAATTTATTAATTTTTAGAAGAATTGTAGAAAATGGAACAG
GAACAGGATGAACAGTTTATCCCCCTTTATCCTCTAATATTGCCCATAGAGGTAGATCTGTTGATCTTGCTATTTTTTCT
TTACATTTAGCAGGAATTTCTTCTATTTTAGGAGCTATTAACTTTATTACTACTATTATTAATATAAAATTAAATGGATT
ATCATTTGATCAAATACCTTTATTTGTATGAGCTGTAGGAATTACAGCTTTATTATTACTTTTATCTTTACCAGTATTAG
CAGGAGCTATTACTATATTATTAACTGATCGAAATTTAAATACTTCTTTTTTTGACCCTGCTGGAGGAGGAGATCCAATT
TTATATCAACATTTATTT---------
>NSWBB627-08|Emmalocera_latilimbella|07-NSWBB-0627|
AACTTTATATTTTATTTTTGGAATTTGATCAGGAATAGTAGGAACATCTATAAGTTTACTTATTCGAGCTGAATTAGGAA
CTCCTGGATCTTTAATTGGAGATGACCAAATTTATAATACTATTGTTACTGGTCATGCTTTTATTATAATTTTTTTTATA
GTTATACCTATTATAATTGGCGGATTTGGAAATTGATTAGTTCCTTTAATATTAGGGGCCCCAGATATAGCTTTTCCTCG
AATAAATAATATAAGATTCTGACTCTTACCCCCTTCCCTTAATTTATTAATTTTTAGAAGAATTGTAGAAAATGGAGCAG
GAACAGGATGAACAGTTTATCCCCCTTTATCCTCTAATATTGCCCATAGAGGTAGATCTGTTGATCTTGCTATTTTTTCT
TTACATTTAGCAGGAATTTCTTCTATTTTAGGAGCTATTAATTTTATTACTACTATTATTAATATAAAATTAAATGGATT
ATCATTTGATCAAATACCTTTATTTGTATGAGCTGTAGGAATTACAGCTTTATTATTACTTTTATCTTTACCAGTATTAG
CAGGAGCTATTACTATATTATTAACTGATCGAAATTTAAATACTTCTTTTTTTGACCCTGCTGGAGGAGGAGATCCAATT
TTATATCAACATTTATTT---------
>NSWHH098-09|Scirpophaga_imparellus|08-NSWHH-0098|
AACTTTATATTTTATTTTTGGTATTTGAGCTGGAATAGTAGGGACTTCTTTAAGTTTATTAATTCGAGCTGAATTAGGAA
CACCAGGATCATTAATTGGGGATGATCAAATTTATAATACTATTGTTACAGCCCATGCTTTTATTATAATTTTTTTTATA
GTAATACCTATTATAATTGGGGGATTCGGAAATTGACTAGTTCCCCTAATATTAGGAGCCCCTGATATAGCTTTTCCACG
TATAAATAATATAAGTTTTTGATTATTACCTCCCTCTCTTACTCTTTTAATTTCAAGAAGAATTGTTGAAAATGGAGTAG
GAACAGGATGAACTGTTTACCCCCCTTTATCCTCTAATATTGCCCATGGAGGAACATCTGTAGATCTAGCTATTTTTTCC
TTACATTTAGCGGGAATTTCATCTATTTTAGGAGCTATTAATTTTATTACAACTATTATTAATATACGAATTAATGGATT
ATCTTTTGATCAAATACCTTTATTTGTCTGAGCTGTAGGTATTACAGCACTTCTTTTACTTTTATCTTTACCTGTGTTAG
CTGGAGCTATTACTATACTACTAACAGATCGAAATTTAAATACATCTTTTTTCGACCCAGCAGGTGGGGGAGACCCAATT
CTTTATCAACATTTATTT---------
>NSWHH125-09|Acrapex_exsanguis|08-NSWHH-0125|
AACATTATATTTTATTTTTGGAATTTGAGCTGGTATACTAGGAACTTCTTTAAGTTTATTAATCCGAGCTGAATTAGGAA
CTCCAGAATCTTTAATTGGAGATGATCAAATTTATAATACTATTGTTACTGCTCATGCTTTTATTATAATTTTCTTTATA
GTTATACCAATTATAATTGGAGGATTTGGAAATTGACTTGTCCCATTAATACTAGGAGCTCCAGATATAGCATTTCCACG
TATAAATAATATAAGATTTTGATTATTACCTCCCTCTTTAAGTTTATTAATTTCAAGAAGAATTGTAGAAAATGGAGCAG
GAACTGGATGAACAGTATATCCTCCACTCTCATCTAATATTGCTCATAGAGGAAGATCAGTAGATTTAGCTATTTTTTCT
CTTCATTTAGCTGGTATTTCATCTATTTTAGGAGCTATTAATTTTATTACAACAATTATTAATATACGATTAAATAATTT
ATCTTTTGATCAAATACCTTTATTTGTTTGAGCTGTTGGAATTACTGCATTTTTACTATTACTCTCATTACCTGTATTAG
CCGGAGCTATTACAATATTATTAACAGATCGAAATTTAAATACATCATTTTTTGATCCTGCAGGAGGAGGTGATCCAATT
TTATATCAACATTTATTT---------
>NSWHH219-09|Scirpophaga_nivella|08-NSWHH-0219|
-----------------------TTGAGCTGGTATAGTAGGAACTTCTTTAAGATTATTAATTCGAGCTGAATTAGGAA
CTCCAGGATCTTTAATTGGAGATGATCAAATTTATAATACCATTGTTACAGCTCATGCTTTTATTATAATTTTTTTTATA
GTAATGCCAATTATAATTGGAGGGTTTGGAAATTGACTTGTTCCTTTAATATTAGGAGCTCCTGATATAGCTTTCCCTCG
TATAAATAATATAAGATTTTGATTATTACCCCCCTCATTAACTCTCCTAATTTCAAGAAGAATTGTAGAAAATGGTGCAG
GAACAGGGTGAACAGTATACCCCCCCTTATCATCAAATATTGCTCATGGAGGAACTTCTGTAGATTTAGCTATTTTTTCT
TTACATCTTGCAGGAATTTCCTCTATTTTAGGAGCTATTAACTTTATTACCACTATTATTAATATACGAATTAATGGCTT
AACATTTGATCAAATACCTCTCTTTGTTTGAGCTGTTGGAATTACAGCCCTTCTTTTACTCCTCTCATTACCCGTATTAG
CTGGAGCTATTACTATATTATTAACTGATCGAAATTTAAATACCTCTTTTTTTGATCCAGCGGGAGGAGGAGATCCAATC
CTTTATCAACATTTATTT---------
>NSWHH402-09|Scirpophaga_nivella|08-NSWHH-0402|
AACTTTATATTTCATTTTTGGAATTTGAGCTGGTATAGTAGGAACTTCTTTAAGATTATTAATTCGAGCTGAATTAGGAA
CTCCAGGATCTTTAATTGGAGATGATCAAATTTATAATACCATTGTTACAGCTCATGCTTTTATTATAATTTTTTTTATA
GTAATGCCAATTATAATTGGAGGGTTTGGAAATTGACTTGTTCCTTTAATATTAGGAGCTCCTGATATAGCTTTCCCTCG
TATAAATAATATAAGATTTTGATTATTACCCCCCTCATTAACTCTCCTAATTTCAAGAAGAATTGTAGAAAATGGTGCAG
GAACAGGGTGAACAGTATACCCCCCCTTATCATCAAATATTGCTCATGGAGGAACTTCTGTAGATTTAGCTATTTTTTCT
TTACATCTTGCAGGAATTTCCTCTATTTTAGGAGCTATTAACTTTATTACCACTATTATTAATATACGAATTAATGGCTT

AACATTTGATCAAATACCTCTCTTTGTTTGAGCTGTTGGAATTACAGCCCTTCTTTTACTCCTCTCATTACCCGTATTAG
CTGGAGCTATTACTATATTATTAACTGATCGAAATTTAAATACCTCTTTTTTTGATCCAGCGGGAGGAGGAGATCCAATC
CTTTATCAACATTTATTT---------
>NSWHH456-09|Emmalocera_latilimbella|08-NSWHH-0456|
AACTTTATATTTTATTTTTGGAATTTGATCAGGAATAGTAGGAACATCTATAAGTTTACTTATTCGAGCTGAATTAGGAA
CTCCTGGATCTTTAATTGGAGATGACCAAATTTATAATACTATTGTTACTGGTCATGCTTTTATTATAATTTTTTTTATA
GTTATACCTATTATAATTGGCGGATTTGGAAATTGATTAGTTCCTTTAATATTAGGAGCCCCAGATATAGCTTTTCCTCG
AATAAATAATATAAGATTCTGACTCTTACCCCCTTCCCTTAATTTATTAATTTTTAGAAGAATTGTAGAAAATGGAGCAG
GAACAGGATGAACAGTTTATCCCCCTTTATCCTCTAATATTACCCATAGAGGTAGATCTGTTGATCTTGCTATTTTTTCT
TTACATTTAGCAGGAATTTCTTCTATTTTAGGAGCTATTAACTTTATTACTACTATTATTAATATAAAATTAAATGGATT
ATCATTTGATCAAATACCTTTATTTGTATGAGCTGTAGGAATTACAGCTTTATTATTACTTTTATCTTTACCAGTATTAG
CAGGAGCTATTACTATATTATTAACTGATCGAAATTTAAATACTTCTTTTTTTGACCCTGCTGGAGGAGGAGACCCAATT
TTATATCAACATTTATTC---------
>NSWHH468-09|Emmalocera|08-NSWHH-0468|
AACCTTATATTTTATTTTTGGAATTTGATCAGGAATGGTAGGAACATCTTTAAGTTTACTCATTCGAGCTGAATTAGGAA
CTCCTGGGTCTTTAATTGGAAATGATCAAATTTACAATACTATTGTTACTGGTCATGCTTTTATTATAATTTTCTTTATA
GTTATACCTATTATAATTGGTGGATTTGGAAATTGATTAATTCCTTTAATATTAGGTGCTCCAGATATAGCTTTCCCTCG
AATAAATAATATAAGATTTTGACTTTTACCCCCCTCTCTTAATTTATTAATTTCTAGAAGCATCGTAGAAAATGGAGCAG
GAACAGGATGAACTGTATACCCTCCTTTATCTTCTAATATTGCTCACAGTGGAAGATCTGTTGATCTTGCCATCTTTTCT
TTACATTTAGCAGGAATTTCTTCTATTTTAGGAGCTATTAATTTTATTACCACTATTATTAATATAAAATTAAATGGATT
AATATTTGATCAAATACCTTTATTTGTATGAGCTGTAGGTATTACAGCTTTATTATTACTTTTATCATTACCAGTATTAG
CTGGAGCTATTACTATGTTATTAACTGATCGAAATTTAAATACTTCTTTTTTTGACCCTGCTGGAGGAGGAGACCCAATT
TTATACCAACATTTATTT---------
>NSWHH524-09|Emmalocera|08-NSWHH-0524|
AACCTTATATTTTATTTTTGGAATTTGATCAGGAATGGTAGGAACATCTTTAAGTTTACTCATTCGAGCTGAATTAGGAA
CTCCTGGGTCTTTAATTGGAAATGATCAAATTTACAATACTATTGTTACTGGTCATGCTTTTATTATAATTTTCTTTATA
GTTATACCTATTATAATTGGTGGATTTGGAAATTGATTAATTCCTTTAATATTAGGTGCTCCAGATATAGCTTTCCCTCG
AATAAATAATATAAGATTTTGACTTTTACCCCCCTCTCTTAATTTATTAATTTCTAGAAGCATCGTAGAAAATGGAGCAG
GAACAGGATGAACTGTATACCCTCCTTTATCTTCTAATATTGCTCACAGTGGAAGATCTGTTGATCTTGCCATCTTTTCT
TTACATTTAGCAGGAATTTCTTCTATTTTAGGAGCTATTAATTTTATTACCACTATTATTAATATAAAATTAAATGGATT
AATATTTGATCAAATACCTTTATTTGTATGAGCTGTAGGTATTACAGCTTTATTATTACTTTTATCATTACCAGTATTAG
CTGGAGCTATTACTATGTTATTAACTGATCGAAATTTAAATACTTCTTTTTTTGACCCTGCTGGAGGAGGAGACCCAATT
TTATACCAACATTTATTT---------
>NSWHH604-09|Emmalocera_latilimbella|08-NSWHH-0604|
AACTTTATATTTTATTTTTGGAATTTGATCAGGAATAGTAGGAACATCTATAAGTTTACTTATTCGAGCTGAATTAGGAA
CTCCTGGATCTTTAATTGGAGATGACCAAATTTATAATACTATTGTTACTGGTCATGCTTTTATTATAATTTTTTTTATA
GTTATACCTATTATAATTGGCGGATTTGGAAATTGATTAGTTCCTTTAATATTAGGAGCCCCAGATATAGCTTTTCCTCG
AATAAATAATATAAGATTCTGACTCTTACCCCCTTCCCTTAATTTATTAATTTTTAGAAGAATTGTAGAAAATGGAGCAG
GAACAGGATGAACAGTTTATCCCCCTTTATCCTCTAATATTACCCATAGAGGTAGATCTGTTGATCTTGCTATTTTTTCT
TTACATTTAGCAGGAATTTCTTCTATTTTAGGAGCTATTAACTTTATTACTACTATTATTAATATAAAATTAAATGGATT
ATCATTTGATCAAATACCTTTATTTGTATGAGCTGTAGGAATTACAGCTTTATTATTACTTTTATCTTTACCAGTATTAG
CAGGAGCTATTACTATATTATTAACTGATCGAAATTTAAATACTTCTTTTTTTGACCCTGCTGGAGGAGGAGACCCAATT
TTATATCAACATTTATTC---------
>NSWHH708-09|Emmalocera_latilimbella|08-NSWHH-0708|
AACTTTATATTTTATTTTTGGAATTTGATCAGGAATAGTAGGAACATCTATAAGTTTACTTATTCGAGCTGAATTAGGAA
CTCCTGGATCTTTAATTGGAGATGACCAAATTTATAATACTATTGTTACTGGTCATGCTTTTATTATAATTTTTTTTATA
GTTATACCTATTATAATTGGCGGATTTGGAAATTGATTAGTTCCTTTAATATTAGGAGCCCCAGATATAGCTTTTCCTCG
AATAAATAATATAAGATTCTGACTCTTACCCCCTTCCCTTAATTTATTAATTTTTAGAAGAATTGTAGAAAATGGAGCAG
GAACAGGATGAACAGTTTATCCCCCTTTATCCTCTAATATTGCCCATAGAGGTAGATCTGTTGATCTTGCTATTTTTTCT
TTACATTTAGCAGGAATTTCTTCTATTTTAGGAGCTATTAACTTTATTACTACTATTATTAATATAAAATTAAATGGATT
ATCATTTGATCAAATACCTTTATTTGTATGAGCTGTAGGAATTACAGCTTTATTATTACTTTTATCTTTACCAGTATTAG
CAGGAGCTATTACTATATTATTAACTGATCGAAATTTAAATACTTCTTTTTTTGACCCTGCTGGAGGAGGAGACCCAATT
TTATATCAACATTTATTC---------
>NSWHH752-09|Emmalocera|08-NSWHH-0752|
GACCTTATATTTTATTTTTGGAATTTGATCAGGAATAGTAGGAACATCTTTAAGTTTACTTATTCGAGCTGAATTAGGAA
CTCCTGGATCTTTAATTGGAGATGACCAAATTTACAACACTATTGTTACTGGTCATGCTTTTATTATAATTTTCTTTATA
GTTATACCTATTATAATTGGTGGATTTGGAAATTGATTAATTCCTTTAATATTAGGTGCTCCAGATATAGCTTTCCCTCG

AATAAATAATATAAGATTTTGACTTTTACCCCCCTCTCTTAATTTATTAATTTCTAGAAGAATCGTAGAAAATGGAGCAG
GAACAGGATGAACTGTATATCCTCCTTTATCTTCTAATATTGCTCATAGTGGAAGATCTGTTGATCTTGCCATTTTTTCT
TTACATTTAGCAGGAATTTCTTCTATTTTAGGAGCTATTAATTTTATTACCACTATTATTAATATAAAATTAAATGGATT
AATATTTGATCAAATACCTTTATTTGTATGAGCTGTAGGTATTACAGCTTTATTATTACTTTTATCATTACCAGTATTAG
CTGGAGCTATTACTATGTTATTAACTGATCGAAATTTAAATACTTCTTTTTTGATCCTGCTGGAGGGGGAGACCCAATT
TTATACCAACATTTATTT---------
>NSWHH932-09|Acrapex_exsanguis|08-NSWHH-0932|
AACATTATATTTTATTTTTGGAATTTGAGCTGGTATACTAGGAACTTCTTTAAGTTTATTAATCCGAGCTGAATTAGGAA
CTCCAGAATCTTTAATTGGAGATGATCAAATTTACAATACTATTGTTACTGCTCACGCTTTTATTATAATTTTTTTTATA
GTTATACCAATTATAATTGGAGGATTTGGAAATTGACTTGTCCCATTAATATTAGGAGCTCCAGATATAGCATTCCCACG
TATAAATAATATAAGATTTTGATTATTACCTCCCTCTTTAAGTTTATTAATTTCAAGAAGAATTGTAGAAAATGGAGCAG
GAACTGGATGAACAGTATATCCCCCACTCTCATCTAATATTGCTCATAGAGGAAGATCAGTAGACTTAGCTATTTTTTCT
CTTCATTTAGCTGGTATTTCATCTATTTTAGGAGCTATTAATTTTATTACAACAATTATTAATATACGATTAAATAATTT
ATCTTTTGATCAAATACCTTTATTTGTTTGAGCTGTTGGAATTACTGCATTTTTACTATTACTCTCATTACCCGTATTAG
CCGGAGCTATTACAATATTATTAACAGATCGAAATTTAAATACATCATTTTTTGATCCTGCAGGAGGAGGTGATCCAATT
TTATATCAACATTTATTT---------
>NSWHI024-09|Scirpophaga_nivella|08-NSWHH-1024|
AACTTTATATTTCATTTTTGGAATTTGAGCTGGTATAGTAGGAACTTCTTTAAGATTATTAATTCGAGCTGAATTAGGAA
CTCCAGGATCTTTAATTGGAGATGATCAAATTTATAATACCATTGTTACAGCTCATGCTTTTATTATAATTTTTTTTATA
GTAATGCCAATTATAATTGGAGGGTTTGGAAATTGACTTGTTCCTTTAATATTAGGAGCTCCTGATATAGCTTTCCCTCG
TATAAATAATATAAGATTTTGATTATTACCCCCCTCATTAACTCTCCTAATTTCAAGAAGAATTGTAGAAAATGGTGCAG
GAACAGGGTGAACAGTATACCCCCCCTTATCATCAAATATTGCTCATGGAGGAACTTCTGTAGATTTAGCTATTTTTTCT
TTACATCTTGCAGGAATTTCCTCTATTTTAGGAGCTATTAACTTTATTACCACTATTATTAATATACGAATTAATGGCTT
AACATTTGATCAAATACCTCTCTTTGTTTGAGCTGTTGGAATTACAGCCCTTCTTTTACTCCTCTCATTACCCGTATTAG
CTGGAGCTATTACTATATTATTAACTGATCGAAATTTAAATACCTCTTTTTTTGATCCAGCGGGAGGAGGAGATCCAATC
CTTTATCAACATTTATTT---------
>NSWHI101-09|Acrapex_exsanguis|08-NSWHH-1101|
----------------------TTTGAGCTGGTATACTAGGAACTTCTTTAAGTTTATTAATCCGAGCTGAATTAGGAA
CTCCAGAATCTTTAATTGGAGATGATCAAATTTATAATACTATTGTTACTGCTCACGCTTTTATCATAATTTTCTTTATA
GTTATACCAATTATAATTGGAGGATTTGGAAATTGACTTGTCCCATTAATATTAGGAGCTCCAGATATAGCATTTCCACG
TATAAATAATATAAGATTTTGATTATTACCTCCCTCTTTAAGTTTATTAATTTCAAGAAGAATTGTAGAAAATGGAGCAG
GAACTGGGTGAACAGTATATCCTCCACTCTCATCTAATATTGCTCATAGAGGAAGATCAGTAGATTTAGCTATTTTTTCT
CTTCATTTAGCTGGTATTTCATCTATTTTAGGAGCTATTAATTTTATTACAACAATTATTAATATACGATTAAATAATTT
ATCTTTTGATCAAATACCTTTATTTGTTTGAGCTGTTGGAATTACTGCATTTTTACTATTACTCTCATTACCTGTATTAG
CTGGAGCTATTACAATATTATTAACAGATCGAAATTTAAATACATCCTTTTTTGATCCTGCAGGAGGAGGTGATCCAATT
TTATATCAACATTTATTT---------
>NSWHI104-09|Acrapex_exsanguis|08-NSWHH-1104|
AACATTATATTTTATTTTTGGAATTTGAGCTGGTATACTAGGAACTTCTTTAAGTTTATTAATCCGAGCTGAACTAGGAA
CTCCAGAATCTTTAATTGGAGATGATCAAATTTACAATACTATTGTTACTGCTCACGCTTTTATTATAATTTTTTTTATA
GTTATACCAATTATAATTGGAGGATTTGGAAATTGACTTGTCCCATTAATATTAGGAGCTCCAGATATAGCATTCCCACG
TATAAATAATATAAGATTTTGATTATTACCTCCCTCTTTAAGTTTATTAATTTCAAGAAGAATTGTAGAAAATGGAGCAG
GAACTGGATGAACAGTATATCCCCCACTCTCATCTAATATTGCTCATAGAGGAAGATCAGTAGACTTAGCTATTTTTTCT
CTTCATTTAGCTGGTATTTCATCTATTTTAGGAGCTATTAATTTTATTACAACAATTATTAATATACGATTAAATAATTT
ATCTTTTGATCAAATACCTTTATTTGTTTGAGCTGTTGGAATTACTGCATTTTTACTATTACTCTCATTACCCGTATTAG
CCGGAGCTATTACAATATTATTAACAGATCGAAATTTAAATACATCATTTTTTGATCCTGCAGGAGGAGGTGATCCAATT
TTATATCAACATTTATTT---------
>NSWHI106-09|Acrapex_albicostata|08-NSWHH-1106|
TACATTATATTTTATTTTTGGAATTTGGGCAGGTATAGTAGGAACCTCTTTAAGATTATTAATTCGGGCTGAATTAGGAA
CCCCAGGATCTTTAATTGGAGATGATCAAATTTATAATACTATTGTTACAGCTCATGCTTTTATTATAATTTTCTTTATG
GTTATACCTATTATGATTGGGGGATTTGGGAATTGACTTGTCCCTTTAATACTAGGTGCCCCAGATATAGCATTTCCACG
AATAAATAATATAAGTTTTTGATTACTACCCCCCTCTTTAACCCTTCTTATTTCCAGTAGAATTGTAGAAAATGGAGCAG
GAACTGGATGAACTGTATACCCACCTCTTTCATCTAATATTGCCCATGGAGGAAGATCTGTAGATTTAGCTATTTTTTCT
TTACATTTAGCTGGGATTTCTTCTATTTTAGGGGCTATTAACTTTATTACTACAATTATTAATATACGATTAAATAGTTT
ATCCTTTGATCAAATACCTTTATTTATTTGAGCTGTAGGAATTACTGCATTTTTATTATTACTTTCTTTACCTGTATTAG
CTGGAGCTATTACAATACTACTAACAGATCGAAACTTAAATACCTCTTTTTTTGACCCTGCTGGAGGTGGAGATCCAATT
TTATATCAACATTTATTT---------
>NSWHI112-09|Emmalocera|08-NSWHH-1112|

GACCTTATATTTTATTTTTGGAATTTGATCAGGAATAGTAGGAACATCTTTAAGTTTACTTATTCGAGCTGAATTAGGAA
CTCCTGGATCTTTAATTGGAGATGACCAAATTTACAACACTATTGTTACTGGTCATGCTTTTATTATAATTTTCTTTATA
GTTATACCTATTATAATTGGTGGATTTGGAAATTGATTAATTCCTTTAATATTAGGTGCTCCAGATATAGCTTTCCCTCG
AATAAATAATATAAGATTTTGACTTTTACCCCCCTCTCTTAATTTATTAATTTCTAGAAGAATCGTAGAAAATGGAGCAG
GAACAGGATGAACTGTATATCCTCCTTTATCTTCTAATATTGCTCATAGTGGAAGATCTGTTGATCTTGCCATTTTTTCT
TTACATTTAGCAGGAATTTCTTCTATTTTAGGAGCTATTAATTTTATTACCACTATTATTAATATAAAATTAAATGGATT
AATATTTGATCAAATACCTTTATTTGTATGAGCTGTAGGTATTACAGCTTTATTATTACTTTTATCATTACCAGTATTAG
CTGGAGCTATTACTATGTTATTAACTGATCGAAATTTAAATACTTCTTTTTTTGATCCTGCTGGAGGGGGAGACCCAATT
TTATACCAACATTTATTT---------
>NSWHI249-09|Acrapex_exsanguis|08-NSWHH-1249|
AACATTATATTTTATTTTTGGAATTTGAGCTGGTATACTAGGAACTTCTTTAAGTTTATTAATCCGAGCTGAACTAGGAA
CTCCAGAATCTTTAATTGGAGATGATCAAATTTACAATACTATTGTTACTGCTCACGCTTTTATTATAATTTTTTTTATA
GTTATACCAATTATAATTGGAGGATTTGGAAATTGACTTGTCCCATTAATATTAGGAGCTCCAGATATAGCATTCCCACG
TATAAATAATATAAGATTTTGATTATTACCTCCCTCTTTAAGTTTATTAATTTCAAGAAGAATTGTAGAAAATGGAGCAG
GAACTGGATGAACAGTATATCCCCCACTCTCATCTAATATTGCTCATAGAGGAAGATCAGTAGACTTAGCTATTTTTTCT
CTTCATTTAGCTGGTATTTCATCTATTTTAGGAGCTATTAATTTTATTACAACAATTATTAATATACGATTAAATAATTT
ATCTTTTGATCAAATACCTTTATTTATTTGAGCTGTTGGAATTACTGCATTTTTACTATTACTCTCATTACCCGTATTAG
CCGGAGCTATTACAATATTATTAACAGATCGAAATTTAAATACATCATTTTTTGATCCTGCAGGAGGAGGTGATCCAATT
TTATATCAACATTTATTT---------
>NSWHI251-09|Emmalocera_latilimbella|08-NSWHH-1251|
AACTTTATATTTTATTTTTGGAATTTGATCAGGAATAGTAGGAACATCTATAAGTTTACTTATTCGAGCTGAATTAGGAA
CTCCTGGATCTTTAATTGGAGATGACCAAATTTATAATACTATTGTTACTGGTCATGCTTTTATTATAATTTTTTTTATA
GTTATACCTATTATAATTGGCGGATTTGGAAATTGATTAGTTCCTTTAATATTAGGAGCCCCAGATATAGCTTTTCCTCG
AATAAATAATATAAGATTCTGACTCTTACCCCCTTCCCTTAATTTATTAATTTTTAGAAGAATTGTAGAAAATGGAGCAG
GAACAGGATGAACAGTTTATCCCCCTTTATCCTCTAATATTACCCATAGAGGTAGATCTGTTGATCTTGCTATTTTTTCT
TTACATTTAGCAGGAATTTCTTCTATTTTAGGAGCTATTAACTTTATTACTACTATTATTAATATAAAATTAAATGGATT
ATCATTTGATCAAATACCTTTATTTGTATGAGCTGTAGGAATTACAGCTTTATTATTACTTTTATCTTTACCAGTATTAG
CAGGAGCTATTACTATATTATTAACTGATCGAAATTTAAATACTTCTTTTTTTGACCCTGCTGGAGGAGGAGACCCAATT
TTATATCAACATTTATTC---------
>NSWHI390-09|Scirpophaga_nivella|08-NSWHH-1390|
AACTTTATATTTCATTTTTGGAATTTGAGCTGGTATAGTAGGAACTTCTTTAAGATTATTAATTCGAGCTGAATTAGGAA
CTCCAGGATCTTTAATTGGAGATGATCAAATTTATAATACCATTGTTACAGCTCATGCTTTTATTATAATTTTTTTTATA
GTAATGCCAATTATAATTGGAGGGTTTGGAAATTGACTTGTTCCTTTAATATTAGGAGCTCCTGATATAGCTTTCCCTCG
TATAAATAATATAAGATTTTGATTATTACCCCCCTCATTAACTCTCCTAATTTCAAGAAGAATTGTAGAAAATGGTGCAG
GAACAGGGTGAACAGTATACCCCCCCCTTATCATCAAATATTGCTCATGGAGGAACTTCTGTAGATTTAGCTATTTTTTCT
TTACATCTTGCAGGAATTTCCTCTATTTTAGGAGCTATTAACTTTATTACCACTATTATTAATATACGAATTAATGGCTT
AACATTTGATCAAATACCTCTCTTTGTTTGAGCTGTTGGAATTACAGCCCTTCTTTTACTCCTCTCATTACCCGTATTAG
CTGGAGCTATTACTATATTATTAACTGATCGAAATTTAAATACCTCTTTTTTTGATCCAGCGGGAGGAGGAGATCCAATC
CTTTATCAACATTTATTT---------
>NSWHM603-11|Scirpophaga_nivella|BIOUG00951-C10|
AACTTTATATTTCATTTTTGGAATTTGAGCTGGTATAGTAGGAACTTCTTTAAGATTATTAATTCGAGCTGAATTAGGAA
CTCCAGGATCTTTAATTGGAGATGATCAAATTTATAATACCATTGTTACAGCTCATGCTTTTATTATAATTTTTTTTATA
GTAATACCAATTATAATTGGAGGGTTTGGAAATTGACTTGTTCCTTTAATATTAGGAGCTCCTGATATAGCTTTCCCTCG
TATAAATAATATAAGATTTTGATTATTACCCCCCTCATTAACTCTCCTAATTTCAAGAAGAATTGTAGAAAATGGTGCAG
GAACAGGATGAACAGTATACCCCCCCCTTATCATCAAATATTGCTCATGGAGGAACTTCTGTAGATTTAGCTATTTTTTCT
CTACATCTTGCAGGAATTTCCTCTATTTTAGGAGCTATTAACTTTATTACCACTATTATTAATATACGAATTAATGGCTT
AACATTTGATCAAATACCTCTCTTTGTTTGAGCTGTTGGAATTACAGCCCTTCTTTTACTCCTCTCATTACCCGTATTAG
CTGGAGCTATTACTATATTATTAACTGATCGAAATTTAAATACCTCTTTTTTTGATCCAGCGGGAGGAGGAGATCCAATC
CTTTATCAACATTTATTT---------
>NSWHM643-11|Scirpophaga_nivella|BIOUG00951-G02|
AACTTTATATTTCATTTTTGGAATTTGAGCTGGTATAGTAGGAACTTCTTTAAGATTATTAATTCGAGCTGAATTAGGAA
CTCCAGGATCTTTAATTGGAGATGATCAAATTTATAATACCATTGTTACAGCTCATGCTTTTATTATAATTTTTTTTATA
GTAATGCCAATTATAATTGGAGGGTTTGGAAATTGACTTGTTCCTTTAATATTAGGAGCTCCTGATATAGCTTTCCCTCG
TATAAATAATATAAGATTTTGATTATTACCCCCCTCATTAACTCTCCTAATTTCAAGAAGAATTGTAGAAAATGGTGCAG
GAACAGGGTGAACAGTATACCCCCCCCTTATCATCAAATATTGCTCATGGAGGAACTTCTGTAGATTTAGCTATTTTTTCT
TTACATCTTGCAGGAATTTCCTCTATTTTAGGAGCTATTAACTTTATTACCACTATTATTAATATACGAATTAATGGCTT
AACATTTGATCAAATACCTCTCTTTGTTTGAGCTGTTGGAATTACAGCCCTTCTTTTACTCCTCTCATTACCCGTATTAG

CTGGAGCTATTACTATATTATTAACTGATCGAAATTTAAATACCTCTTTTTTTGATCCAGCGGGAGGAGGAGATCCAATC
CTTTATCAACATTTATTT---------
>NSWHM707-11|Scirpophaga_nivella|BIOUG00952-D07|
AACTTTATATTTCATTTTTGGAATTTGAGCTGGTATAGTAGGAACTTCTTTAAGATTATTAATTCGAGCTGAATTAGGAA
CTCCAGGATCTTTAATTGGAGATGATCAAATTTATAATACCATTGTTACAGCTCATGCTTTTATTATAATTTTTTTTATA
GTAATACCAATTATAATTGGAGGGTTTGGAAATTGACTTGTTCCTTTAATATTAGGAGCTCCTGATATAGCTTTCCCTCG
TATAAATAATATAAGATTTTGATTATTACCCCCCTCATTAACTCTCCTAATTTCAAGAAGAATTGTAGAAAATGGTGCAG
GAACAGGATGAACAGTATACCCCCCCTTATCATCAAATATTGCTCATGGAGGAACTTCTGTAGATTTAGCTATTTTTTCT
CTACATCTTGCAGGAATTTCCTCTATTTTAGGAGCTATTAACTTTATTACCACTATTATTAATATACGAATTAATGGCTT
AACATTTGATCAAATACCTCTCTTTGTTTGAGCTGTTGGAATTACAGCCCTTCTTTTACTCCTCTCATTACCCGTATTAG
CTGGAGCTATTACTATATTATTAACTGATCGAAATTTAAATACCTCTTTTTTTGATCCAGCGGGAGGAGGAGATCCAATC
CTTTATCAACATTTATTT---------
>NSWHM715-11|Bathytricha_aethalion|BIOUG00952-E03|
AACATTATATTTTATTTTTGGGATTTGAGCAGGGATGGTAGGAACTTCTTTAAGACTACTAATTCGAGCTGAACTAGGAA
CTCCTGGATCTCTGATTGGGGATGATCAAATTTATAATACTATTGTAACAGCTCATGCTTTTATTATAATTTTTTTTATG
GTTATACCAATCATAATTGGAGGATTTGGAAATTGACTTGTACCTTTAATATTAGGAGCGCCTGATATGGCATTCCCACG
AATAAATAACATAAGTTTTTGATTACTACCACCTTCTTTAACTCTTCTTATTTCAAGTAGAGTTGTAGAAAATGGAGCGG
GAACTGGATGAACAGTTTATCCACCACTCTCATCTAATATTGCTCATGGAGGAAGATCAGTGGACCTAGCTATTTTTTCC
CTCCATTTAGCTGGAATCTCTTCAATTCTAGGAGCTATTAATTTTATTACAACTATTATTAATATACGATTAAATAACTT
ATCTTTTGATCAAATACCTTTATTTATTTGAGCCGTAGGAATTACAGCTTTTTTATTATTATTATCATTACCTGTATTAG
CTGGAGCTATTACTATATTACTAACAGATCGAAATTTAAATACATCATTTTTCGATCCTGCAGGAGGGGGGGATCCAATT
TTATACCAACATTTATTT---------
>NSWHM726-11|Acrapex_albicostata|BIOUG00952-F02|
TACATTATATTTTATTTTTGGAATTTGGGCAGGTATAGTAGGAACCTCTTTAAGATTATTAATTCGGGCTGAATTAGGAA
CCCCAGGATCTTTAATTGGAGATGATCAAATTTATAATACTATTGTTACAGCTCATGCTTTTATTATAATTTTCTTTATG
GTTATACCTATTATGATTGGGGGATTTGGGAATTGACTTGTCCCTTTAATACTAGGTGCCCCAGATATAGCATTTCCACG
AATAAATAATATAAGTTTTTGATTACTACCCCCCTCTTTAACCCTTCTTATTTCCAGTAGAATTGTAGAAAATGGAGCAG
GAACTGGATGAACTGTATACCCACCTCTTTCATCTAATATTGCCCATGGAGGAAGATCTGTAGATTTAGCTATTTTTTCT
TTACATTTAGCTGGGATTTCTTCTATTTTAGGGGCTATTAACTTTATTACTACAATTATTAATATACGATTAAATAGTTT
ATCCTTTGATCAAATACCTTTATTTATTTGAGCTGTAGGAATTACTGCATTTTTATTATTACTTTCTTTACCTGTATTAG
CTGGAGCTATTACAATACTATTAACAGATCGAAACTTAAATACCTCTTTTTTTGACCCTGCTGGAGGTGGAGATCCAATT
TTATATCAACATTTATTT---------
>NSWHM815-11|Emmalocera|BIOUG00953-E08|
GACCTTATATTTTATTTTTGGAATTTGATCAGGAATAGTAGGAACATCTTTAAGTTTACTTATTCGAGCTGAATTAGGAA
CTCCTGGATCTTTAATTGGAGATGACCAAATTTACAACACTATTGTTACTGGTCATGCTTTTATTATAATTTTCTTTATA
GTTATACCTATTATAATTGGTGGATTTGGAAATTGATTAATTCCTTTAATATTAGGTGCTCCAGATATAGCTTTCCCTCG
AATAAATAATATAAGATTTTGACTTTTACCCCCCTCTCTTAATTTATTAATTTCTAGAAGAATCGTAGAAAATGGAGCAG
GAACAGGATGAACTGTATATCCTCCTTTATCTTCTAATATTGCTCATAGTGGAAGATCTGTTGATCTTGCCATTTTTTCT
TTACATTTAGCAGGAATTTCTTCTATTTTAGGAGCTATTAATTTTATTACCACTATTATTAATATAAAATTAAATGGATT
AATATTTGATCAAATACCTTTATTTGTATGAGCTGTAGGTATTACAGCTTTATTATTACTTTTATCATTACCAGTATTAG
CTGGAGCTATTACTATGTTATTAACTGATCGAAATTTAAATACTTCTTTTTTTGATCCTGCTGGAGGAGGAGATCCAATT
TTATACCAACATTTATTT---------
>NSWHM824-11|Emmalocera_latilimbella|BIOUG00953-F05|
AACTTTATATTTTATTTTTGGAATTTGATCAGGAATAGTAGGAACATCTATAAGTTTACTTATTCGAGCTGAATTAGGAA
CTCCTGGATCTTTAATTGGAGATGACCAAATTTATAATACTATTGTTACTGGTCATGCTTTTATTATAATTTTTTTTATA
GTTATACCTATTATAATTGGCGGATTTGGAAATTGATTAGTTCCTTTAATATTAGGAGCCCCAGATATAGCTTTTCCTCG
AATAAATAATATAAGATTCTGACTCTTACCCCCTTCCCTTAATTTATTAATTTTTAGAAGAATTGTAGAAAATGGAGCAG
GAACAGGATGAACAGTTTATCCCCCTTTATCCTCTAATATTACCCATAGAGGTAGATCTGTTGATCTTGCTATTTTTTCT
TTACATTTAGCAGGAATTTCTTCTATTTTAGGAGCTATTAACTTTATTACTACTATTATTAATATAAAATTAAATGGATT
ATCATTTGATCAAATACCTTTATTTGTATGAGCTGTAGGAATTACAGCTTTATTATTACTTTTATCTTTACCAGTATTAG
CAGGAGCTATTACTATATTATTAACTGATCGAAATTTAAATACTTCTTTTTTTGACCCTGCTGGAGGAGGAGACCCAATT
TTATATCAACATTTATTC---------
>NSWHM1083-11|Emmalocera|BIOUG00956-D03|
AACTTTATATTTTATTTTTGGAATTTGGGCCGGAATAGTTGGGACATCTTTAAGTCTTCTCATTCGAGCAGAATTAGGTA
CTCCTGGATCTTTAATTGGAGATGATCAAATTTATAATACTATTGTCACAAGCCATGCTTTTATTATAATTTTTTTTATA
GTTATACCTATTATAATTGGAGGATTCGGAAACTGATTAATCCCTTTAATATTAGGAGCTCCAGATATAGCTTTCCCTCG
AATAAATAATATAAGATTTTGACTTTTACCCCCTTCACTTACTTTATTAATCTCTAGTAGAATTGTAGAAAATGGAGCAG

GAACTGGATGAACTGTTTACCCCCCTTTATCTTCTAATATTGCCCATAGAGGAAGATCTGTAGATCTTACTATTTTTTCC
CTTCATTTAGCAGGAATTTCTTCTATTTTAGGAGCCATTAATTTTATTACTACAATTATTAATATAAAATTAAATGGTTT
ATCATTTGAACAAATATCTTTATTTGTTTGAGCTGTAGGAATTACAGCTTTATTATTACTATTATCTTTACCTGTTCTTG
CAGGAGCTATTACTATATTATTAACAGATCGAAATTTAAATACTTCTTTTTTTGACCCTGCTGGAGGAGGAGATCCAATT
CTTTATCAACATTTATTT---------
>NSWHM1166-11|Emmalocera_latilimbella|BIOUG00944-C03|
AACTTTATATTTTATTTTTGGAATTTGATCAGGAATAGTAGGAACATCTATAAGTTTACTTATTCGAGCTGAATTAGGAA
CTCCTGGATCTTTAATTGGAGATGACCAAATTTATAATACTATTGTTACTGGTCATGCTTTTATTATAATTTTTTTTATA
GTTATACCTATTATAATTGGCGGATTTGGAAATTGATTAGTTCCTTTAATATTAGGAGCCCCAGATATAGCTTTTCCTCG
AATAAATAATATAAGATTCTGACTCTTACCCCCTTCCCTTAATTTATTAATTTTTAGAAGAATTGTAGAAAATGGAGCAG
GAACAGGATGAACAGTTTATCCCCCTTTATCCTCTAATATTACCCATAGAGGTAGATCTGTTGATCTTGCTATTTTTTCT
TTACATTTAGCAGGAATTTCTTCTATTTTAGGAGCTATTAACTTTATTACTACTATTATTAATATAAAATTAAATGGATT
ATCATTTGATCAAATACCTTTATTTGTATGAGCTGTAGGAATTACAGCTTTATTATTACTTTTATCTTTACCAGTATTAG
CAGGAGCTATTACTATATTATTAACTGATCGAAATTTAAATACTTCTTTTTTTGACCCTGCTGGAGGAGGAGACCCAATT
TTATATCAACATTTATTC---------
>NSWHM1209-11|Scirpophaga_nivella|BIOUG00944-F10|
AACTTTATATTTCATTTTTGGAATTTGAGCTGGTATAGTAGGAACTTCTTTAAGATTATTAATTCGAGCTGAATTAGGAA
CTCCAGGATCTTTAATTGGAGATGATCAAATTTATAATACCATTGTTACAGCTCATGCTTTTATTATAATTTTTTTTATA
GTAATGCCAATTATAATTGGAGGGTTTGGAAATTGACTTGTTCCTTTAATATTAGGAGCTCCTGATATAGCTTTCCCTCG
TATAAATAATATAAGATTTTGATTATTACCCCCCTCATTAACTCTCCTAATTTCAAGAAGAATTGTAGAAAATGGTGCAG
GAACAGGGTGAACAGTATACCCCCCCTTATCATCAAATATTGCTCATGGAGGAACTTCTGTAGATTTAGCTATTTTTTCT
TTACATCTTGCAGGAATTTCCTCTATTTTAGGAGCTATTAACTTTATTACCACTATTATTAATATACGAATTAATGGCTT
AACATTTGATCAAATACCTCTCTTTGTTTGAGCTGTTGGAATTACAGCCCTTCTTTTACTCCTCTCATTACCCGTATTAG
CTGGAGCTATTACTATATTATTAACTGATCGAAATTTAAATACCTCTTTTTTTGATCCAGCGGGAGGAGGAGATCCAATC
CTTTATCAACATTTATTT---------
>NSWHM1216-11|Emmalocera|BIOUG00944-G05|
AACCTTATATTTTATTTTTGGAATTTGATCAGGAATGGTAGGAACATCTTTAAGTTTACTCATTCGAGCTGAATTAGGAA
CTCCTGGGTCTTTAATTGGAAATGATCAAATTTACAATACTATTGTTACTGGTCATGCTTTTATTATAATTTTCTTTATA
GTTATACCTATTATAATTGGTGGATTTGGAAATTGATTAATTCCTTTAATATTAGGTGCTCCAGATATAGCTTTCCCTCG
AATAAATAATATAAGATTTTGACTTTTACCCCCCTCTCTTAATTTATTAATTTCTAGAAGCATCGTAGAAAATGGAGCAG
GAACAGGATGAACTGTATACCCTCCTTTATCTTCTAATATTGCTCACAGTGGAAGATCTGTTGATCTTGCCATCTTTTCT
TTACATTTAGCAGGAATTTCTTCTATTTTAGGAGCTATTAATTTTATTACCACTATTATTAATATAAAATTAAATGGATT
AATATTTGATCAAATACCTTTATTTGTATGAGCTGTAGGTATTACAGCTTTATTATTACTTTTATCATTACCAGTATTAG
CTGGAGCTATTACTATGTTATTAACTGATCGAAATTTAAATACTTCTTTTTTTGACCCTGCTGGAGGAGGAGACCCAATT
TTATACCAACATTTATTT---------
>NSWHM1290-11|Scirpophaga_nivella|BIOUG00945-E08|
AACTTTATATTTCATTTTTGGAATTTGAGCTGGTATAGTAGGAACTTCTTTAAGATTATTAATTCGAGCTGAATTAGGAA
CTCCAGGATCTTTAATTGGAGATGATCAAATTTATAATACCATTGTTACAGCTCATGCTTTTATTATAATTTTTTTTATA
GTAATGCCAATTATAATTGGAGGGTTTGGAAATTGACTTGTTCCTTTAATATTAGGAGCTCCTGATATAGCTTTCCCTCG
TATAAATAATATAAGATTTTGATTATTACCCCCCTCATTAACTCTCCTAATTTCAAGAAGAATTGTAGAAAATGGTGCAG
GAACAGGGTGAACAGTATACCCCCCCTTATCATCAAATATTGCTCATGGAGGAACTTCTGTAGATTTAGCTATTTTTTCT
TTACATCTTGCAGGAATTTCCTCTATTTTAGGAGCTATTAACTTTATTACCACTATTATTAATATACGAATTAATGGCTT
AACATTTGATCAAATACCTCTCTTTGTTTGAGCTGTTGGAATTACAGCCCTTCTTTTACTCCTCTCATTACCCGTATTAG
CTGGAGCTATTACTATATTATTAACTGATCGAAATTTAAATACCTCTTTTTTTGATCCAGCGGGAGGAGGAGATCCAATC
CTTTATCAACATTTATTT---------
>NSWHM1296-11|Scirpophaga_nivella|BIOUG00945-F02|
AACTTTATATTTCATTTTTGGAATTTGAGCTGGTATAGTAGGAACTTCTTTAAGATTATTAATTCGAGCTGAATTAGGAA
CTCCAGGATCTTTAATTGGAGATGATCAAATTTATAATACCATTGTTACAGCTCATGCTTTTATTATAATTTTTTTTATA
GTAATGCCAATTATAATTGGAGGGTTTGGAAATTGACTTGTTCCTTTAATATTAGGAGCTCCTGATATAGCTTTCCCTCG
TATAAATAATATAAGATTTTGATTATTACCCCCCTCATTAACTCTCCTAATTTCAAGAAGAATTGTAGAAAATGGTGCAG
GAACAGGGTGAACAGTATACCCCCCCTTATCATCAAATATTGCTCATGGAGGAACTTCTGTAGATTTAGCTATTTTTTCT
TTACATCTTGCAGGAATTTCCTCTATTTTAGGAGCTATTAACTTTATTACCACTATTATTAATATACGAATTAATGGCTT
AACATTTGATCAAATACCTCTCTTTGTTTGAGCTGTTGGAATTACAGCCCTTCTTTTACTCCTCTCATTACCCGTATTAG
CTGGAGCTATTACTATATTATTAACTGATCGAAATTTAAATACCTCTTTTTTTGATCCAGCGGGAGGAGGAGATCCAATC
CTTTATCA-------------------
>NSWHM1328-11|Emmalocera|BIOUG00945-H10|
GACCTTATATTTTATTTTTGGAATTTGATCAGGAATAGTAGGAACATCTTTAAGTTTACTTATTCGAGCTGAATTAGGAA

CTCCTGGATCTTTAATTGGAGATGACCAAATTTACAACACTATTGTTACTGGTCATGCTTTTATTATAATTTTCTTTATA
GTTATACCTATTATAATTGGTGGATTTGGAAATTGATTAATTCCTTTAATATTAGGTGCTCCAGATATAGCTTTCCCTCG
AATAAATAATATAAGATTTTGACTTTTACCCCCCTCTCTTAATTTATTAATTTCTAGAAGAATCGTAGAAAATGGAGCAG
GAACAGGATGAACTGTATATCCTCCTTTATCTTCTAATATTGCTCATAGTGGAAGATCTGTTGATCTTGCCATTTTTTCT
TTACATTTAGCAGGAATTTCTTCTATTTTAGGAGCTATTAATTTTATTACCACTATTATTAATATAAAATTAAATGGATT
AATATTTGATCAAATACCTTTATTTGTATGAGCTGTAGGTATTACAGCTTTATTATTACTTTTATCATTACCAGTATTAG
CTGGAGCTATTACTATGTTATTAACTGATCGAAATTTAAATACTTCTTTTTTTG--------------------------
----
>NSWHM1334-11|Emmalocera_latilimbella|BIOUG00946-A05|
AACTTTATATTTTATTTTTGGAATTTGATCAGGAATAGTAGGAACATCTATAAGTTTACTTATTCGAGCTGAATTAGGAA
CTCCTGGATCTTTAATTGGAGATGACCAAATTTATAATACTATTGTTACTGGTCATGCTTTTATTATAATTTTTTTTATA
GTTATACCTATTATAATTGGCGGATTTGGAAATTGATTAGTTCCTTTAATATTAGGAGCCCCAGATATAGCTTTTCCTCG
AATAAATAATATAAGATTCTGACTCTTACCCCCTTCCCTTAATTTATTAATTTTTAGAAGAATTGTAGAAAATGGAGCAG
GAACAGGATGAACAGTTTATCCCCCTTTATCCTCTAATATTACCCATAGAGGTAGATCTGTTGATCTTGCTATTTTTTCT
TTACATTTAGCAGGAATTTCTTCTATTTTAGGAGCTATTAACTTTATTACTACTATTATTAATATAAAATTAAATGGATT
ATCATTTGATCAAATACCTTTATTTGTATGAGCTGTAGGAATTACAGCTTTATTATTACTTTTATCTTTACCAGTATTAG
CAGGAGCTATTACTATATTATTAACTGATCGAAATTTAAATATTTCTTTTTTTGACCCTGCTGGAGGAGGAGAC------
----
>NSWHM1336-11|Emmalocera_latilimbella|BIOUG00946-A07|
AACTTTATATTTTATTTTTGGAATTTGATCAGGAATAGTAGGAACATCTATAAGTTTACTTATTCGAGCTGAATTAGGAA
CTCCTGGATCTTTAATTGGAGATGACCAAATTTATAATACTATTGTTACTGGTCATGCTTTTATTATAATTTTTTTTATA
GTTATACCTATTATAATTGGCGGATTTGGAAATTGATTAGTTCCTTTAATATTAGGAGCCCCAGATATAGCTTTTCCTCG
AATAAATAATATAAGATTCTGACTCTTACCCCCTTCCCTTAATTTATTAATTTTTAGAAGAATTGTAGAAAATGGAGCAG
GAACAGGATGAACAGTTTATCCCCCTTTATCCTCTAATATTACCCATAGAGGTAGATCTGTTGATCTTGCTATTTTTTCT
TTACATTTAGCAGGAATTTCTTCTATTTTAGGAGCTATTAACTTTATTACTACTATTATTAATATAAAATTAAATGGATT
ATCATTTGATCAAATACCTTTATTTGTATGAGCTGTAGGAATTACAGCTTTATTATTACTTTTATCTTTACCAGTATTAG
CAGGAGCTATTACTATATTATTAACTGATCGAAATTTAAATACTTCTTTTTTTGACCCTGCTGGAGGAGGAGACCCAATT
TTATATCAACATTTATTC---------
>NSWHM1394-11|Bathytricha_truncata|BIOUG00946-F05|
AACATTATATTTCATCTTTGGAATTTGAGCAGGAATAGTGGGAACTTCTTTAAGACTATTAATTCGAGCTGAATTAGGAA
CTCCTGGATCCTTAATTGGAGATGATCAAATTTATAATACTATTGTAACAGCTCATGCCTTTATTATAATTTTTTTTATG
GTTATACCAATTATAATTGGAGGATTTGGAAATTGACTTGTACCTTTAATGTTAGGGGCACCTGATATAGCATTTCCACG
AATAAATAATATAAGTTTTTGATTACTACCCCCTTCTTTAACTCTACTTATTTCGAGAAGAGTTGTAGAAAATGGAGCAG
GAACTGGGTGAACAGTATACCCCCCACTTTCATCTAATGTTGCCCATAGAGGAAGATCTGTAGACTTAGCTATTTTTTCC
CTTCATTTAGCTGGAATTTCTTCTATTTTAGGAGCTATTAATTTTATTACAACTATTATTAACATACGATTAAATAATTT
ATCTTTTGATCAAATACCTTTATTTATTTGAGCTGTAGGAATTACAGCATTTTTATTATTATTATCATTACCTGTATTAG
CTGGAGCTATTACCATATTATTAACAGATCGAAATTTAAACACATCATTTTTTGATCCTGCGGGAGGGGGAGATCCAATC
TTATATCAACATTTATTT---------
>NSWHM1416-11|Emmalocera_latilimbella|BIOUG00946-H03|
AACTTTATATTTTATTTTTGGAATTTGATCAGGAATAGTAGGAACATCTATAAGTTTACTTATTCGAGCTGAATTAGGAA
CTCCTGGATCTTTAATTGGAGATGACCAAATTTATAATACTATTGTTACTGGTCATGCTTTTATTATAATTTTTTTTATA
GTTATACCTATTATAATTGGCGGATTTGGAAATTGATTAGTTCCTTTAATATTAGGAGCCCCAGATATAGCTTTTCCTCG
AATAAATAATATAAGATTCTGACTCTTACCCCCTTCCCTTAATTTATTAATTTTTAGAAGAATTGTAGAAAATGGAGCAG
GAACAGGATGAACAGTTTATCCCCCTTTATCCTCTAATATTACCCATAGAGGTAGATCTGTTGATCTTGCTATTTTTTCT
TTACATTTAGCAGGAATTTCTTCTATTTTAGGAGCTATTAACTTTATTACTACTATTATTAATATAAAATTAAATGGATT
ATCATTTGATCAAATACCTTTATTTGTATGAGCTGTAGGAATTACAGCTTTATTATTACTTTTATCTTTACCAGTATTAG
CAGGAGCTATTACTATATTATTAACTGATCGAAATTTAAATACTTCTTTTTTTGACCCTGCTGGAGGAGGAGACCCAATT
TTATATCAACATTTATTC---------
>NSWHM1425-11|Scirpophaga_nivella|BIOUG00948-A01|
AACTTTATATTTCATTTTTGGAATTTGAGCTGGTATAGTAGGAACTTCTTTAAGATTATTAATTCGAGCTGAATTAGGAA
CTCCAGGATCTTTAATTGGAGATGATCAAATTTATAATACCATTGTTACAGCTCATGCTTTTATTATAATTTTTTTTATA
GTAATGCCAATTATAATTGGAGGGTTTGGAAATTGACTTGTTCCTTTAATATTAGGAGCTCCTGATATAGCTTTCCCTCG
TATAAATAATATAAGATTTTGATTATTACCCCCCTCATTAACTCTCCTAATTTCAAGAAGAATTGTAGAAAATGGTGCAG
GAACAGGGTGAACAGTATACCCCCCCTTATCATCAAATATTGCTCATGGAGGAACTTCTGTAGATTTAGCTATTTTTTCT
TTACATCTTGCAGGAATTTCCTCTATTTTAGGAGCTATTAACTTTATTACCACTATTATTAATATACGAATTAATGGCTT
AACATTTGATCAAATACCTCTCTTTGTTTGAGCTGTTGGAATTACAGCCCTTCTTTTACTCCTCTCATTACCCGTATTAG
CTGGAGCTATTACTATATTATTAACTGATCGAAATTTAAATACCTCTTTTTTTGATCCAGCGGGAGGAGGAGATCCAATC

CTTTATCAACATTTATTT---------
>NSWHM1470-11|Emmalocera|BIOUG00948-D10|
GACCTTATATTTTATTTTTGGAATTTGATCAGGAATAGTAGGAACATCTTTAAGTTTACTTATTCGAGCTGAATTAGGAA
CTCCTGGATCTTTAATTGGAGATGACCAAATTTACAACACTATTGTTACTGGTCATGCTTTTATTATAATTTTCTTTATA
GTTATACCTATTATAATTGGTGGATTTGGAAATTGATTAATTCCTTTAATATTAGGTGCTCCAGATATAGCTTTCCCTCG
AATAAATAATATAAGATTTTGACTTTTACCCCCCTCTCTTAATTTATTAATTTCTAGAAGAATCGTAGAAAATGGAGCAG
GAACAGGATGAACTGTATATCCTCCTTTATCTTCTAATATTGCTCATAGTGGAAGATCTGTTGATCTTGCCATTTTTTCT
TTACATTTAGCAGGAATTTCTTCTATTTTAGGAGCTATTAATTTTATTACCACTATTATTAATATAAAATTAAATGGATT
AATATTTGATCAAATACCTTTATTTGTATGAGCTGTAGGTATTACAGCTTTATTATTACTTTTATCATTACCAGTATTAG
CTGGAGCTATTACTATGTTATTAACTGATCGAAATTTAAATACTTCTTTTTTGATCCTGCTGGAGGAGGAGATCCAATT
TTATACCAACATTTATTT---------
>NSWHM1508-11|Emmalocera_latilimbella|BIOUG00948-G12|
AACTTTATATTTTATTTTTGGAATTTGATCAGGAATAGTAGGAACATCTATAAGTTTACTTATTCGAGCTGAATTAGGAA
CTCCTGGATCTTTAATTGGAGATGACCAAATTTATAATACTATTGTTACTGGTCATGCTTTTATTATAATTTTTTTTATA
GTTATACCTATTATAATTGGCGGATTTGGAAATTGATTAGTTCCTTTAATATTAGGAGTCCCAGATATAGCTTTTCCTCG
AATAAATAATATAAGATTCTGACTCTTACCCCCTTCCCTTAATTTATTAATTTTTAGAAGAATTGTAGAAAATGGAGCAG
GAACAGGATGAACAGTTTATCCCCCTTTATCCTCTAATATTACCCATAGAGGTAGATCTGTTGATCTTGCTATTTTTTCT
TTACATTTAGCAGGAATTTCTTCTATTTTAGGAGCTATTAACTTTATTACTACTATTATTAATATAAAATTAAATGGATT
ATCATTTGATCAAATACCTTTATTTGTATGAGCTGTAGGAATTACAGCTTTATTATTACTTTTATCTTTACCAGTATTAG
CAGGAGCTATTACTATATTATTAACTGATCGAAATTTAAATACTTCTTTTTTGACCCTGCTGGAGGAGGAGACCCAATT
TTATATCAACATTTATTC---------
>NSWHM1523-11|Acrapex_albicostata|BIOUG00949-A04|
TACATTATATTTTATTTTTGGAATTTGGGCAGGTATAGTAGGAACCTCTTTAAGATTATTAATTCGGGCTGAATTAGGAA
CCCCAGGATCTTTAATTGGAGATGATCAAATTTATAATACTATTGTTACAGCTCATGCTTTTATTATAATTTTCTTTATG
GTTATACCTATTATGATTGGGGGATTTGGGAATTGACTTGTCCCTTTAATACTAGGTGCCCCAGATATAGCATTTCCACG
AATAAATAATATAAGTTTTTGATTACTACCCCCCTCTTTAACCCTTCTTATTTCCAGTAGAATTGTAGAAAATGGAGCAG
GAACTGGATGAACTGTATACCCACCTCTTTCATCTAATATTGCCCATGGAGGAAGATCTGTAGATTTAGCTATTTTTTCT
TTACATTTAGCTGGGATTTCTTCTATTTTAGGGGCTATTAACTTTATTACTACAATTATTAATATACGATTAAATAGTTT
ATCCTTTGATCAAATACCTTTATTTATTTGAGCTGTAGGAATTACTGCATTTTTATTATTACTTTCTTTACCTGTATTAG
CTGGAGCTATTACAATACTACTAACAGATCGAAACTTAAATACCTCTTTTTTGACCCTGCTGGAGGTGGAGATCCAATT
TTATATCAACATTTATTT---------
>NSWHM1527-11|Scirpophaga_imparellus|BIOUG00949-A08|
AACTTTATATTTTATTTTTGGTATTTGAGCTGGGATAGTGGGGACTTCTTTAAGTTTATTAATTCGAGCTGAATTAGGAA
CACCAGGATCATTAATTGGGGATGATCAAATTTATAATACTATTGTTACAGCCCATGCTTTTATTATAATTTTTTTTATA
GTAATACCTATTATAATTGGGGGATTCGGAAATTGACTAGTTCCCCTAATATTAGGAGCCCCTGATATAGCTTTTCCACG
TATAAATAATATAAGTTTTTGATTATTACCTCCCTCTCTTACTCTTTTAATTTCAAGAAGAATTGTTGAAAATGGAGCAG
GAACAGGATGAACTGTTTACCCCCCTTTATCCTCTAATATTGCCCATGGGGGAACATCTGTAGATCTAGCTATTTTTTCC
TTACATTTAGCAGGAATTTCATCTATTTTAGGAGCTATTAATTTTATTACAACTATTATTAATATACGAATTAATGGATT
ATCTTTTGATCAAATACCTTTATTTGTCTGAGCTGTAGGTATTACAGCACTTCTTTTACTTTTATCTTTACCTGTATTAG
CTGGAGCTATTACTATACTACTAACAGATCGAAATTTAAATACATCTTTTTTCGACCCAGCAGGTGGGGGAGACCCAATT
CTTTATCAACATTTATTT---------
>NSWHM1558-11|Scirpophaga_nivella|BIOUG00949-D03|
AACTTTATATTTCATTTTTGGAATTTGAGCTGGTATAGTAGGAACTTCTTTAAGATTATTAATTCGAGCTGAATTAGGAA
CTCCAGGATCTTTAATTGGAGATGATCAAATTTATAATACCATTGTTACAGCTCATGCTTTTATTATAATTTTTTTTATA
GTAATACCAATTATAATTGGAGGGTTTGGAAATTGACTTGTTCCTTTAATATTAGGAGCTCCTGATATAGCTTTCCCTCG
TATAAATAATATAAGATTTTGATTATTACCCCCCTCATTAACTCTCCTAATTTCAAGAAGAATTGTAGAAAATGGTGCAG
GAACAGGATGAACAGTATACCCCCCCTTATCATCAAATATTGCTCATGGAGGAACTTCTGTAGATTTAGCTATTTTTTCT
CTACATCTTGCAGGAATTTCCTCTATTTTAGGAGCTATTAACTTTATTACCACTATTATTAATATACGAATTAATGGCTT
AACATTTGATCAAATACCTCTCTTTGTTTGAGCTGTTGGAATTACAGCCCTTCTTTTACTCCTCTCATTACCCGTATTAG
CTGGAGCTATTACTATATTATTAACTGATCGAAATTTAAATACCTCTTTTTTTGATCCAGCGGGAGGAGGAGATCCAATC
CTTTATCAACATTTATTT---------
>NSWHM1931-11|Emmalocera|BIOUG00959-C08|
AACTTTATATTTTATTTTTGGAATTTGAGCAGGAATAGTAGGAACATCTTTAAGTCTTTTAATTCGAGCTGAATTAGGAA
CCCCAGGATCTTTAATTGGTGATGACCAAATTTATAATACTATTGTAACTGGTCATGCTTTTATTATAATTTTTTTTATA
GTGATGCCTATTATAATTGGAGGATTCGGAAATTGATTAGTACCTTTAATACTAGGGGCCCCAGATATAGCTTTCCCACG
AATAAATAATATAAGATTTTGACTTTTACCTCCTTCTCTAACTTTATTAGTCTCTAGAAGAATTGTAGAAAATGGAGCAG
GTACTGGATGAACTGTATACCCCCCTTTATCTTCTAATATTGCCCATGGAGGCAGCTCTGTAGATCTTGCTATTTTTTCT

CTTCATTTAGCAGGAATTTCTTCTATTTTAGGAGCTATTAATTTTATTACCACTATTATTAATATAAAATTAAATGGATT
ATCATTTGACCAAATACCTTTATTTGTTTGAGCTGTAGGTATTACAGCTTTACTTCTTTTATTATCTTTACCTGTATTAG
CAGGAGCTATTACTATACTATTAACTGATCGAAATTTAAATACTTCTTTCTTTGACCCTGCTGGAGGGGGAGACCCAATT
TTATACCAACATTTATTT---------
>NSWHN486-11|Emmalocera_latilimbella|BIOUG00981-B03|
AACTTTATATTTTATTTTTGGAATTTGATCAGGAATAGTAGGAACATCTATAAGTTTACTTATTCGAGCTGAATTAGGAA
CTCCTGGATCTTTAATTGGAGATGACCAAATTTATAATACTATTGTTACTGGTCATGCTTTTATTATAATTTTTTTTATA
GTTATACCTATTATAATTGGCGGATTTGGAAATTGATTAGTTCCTTTAATATTAGGGGCCCCAGATATAGCTTTTCCTCG
AATAAATAATATAAGATTCTGACTCTTACCCCCTTCCCTTAATTTATTAATTTTTAGAAGAATTGTAGAAAATGGAGCAG
GAACAGGATGAACAGTTTATCCCCCTTTATCCTCTAATATTGCTCATAGAGGTAGATCTGTTGATCTTGCTATTTTTTCT
TTACATTTAGCAGGAATTTCTTCTATTTTAGGAGCTATTAACTTTATTACTACTATTATTAATATAAAATTAAATGGATT
ATCATTTGATCAAATACCTTTATTTGTATGAGCTGTAGGAATTACAGCTTTATTATTACTTTTATCTTTACCAGTATTAG
CAGGAGCTATTACTATATTATTAACTGATCGAAATTTAAATACTTCTTTTTTTGACCCTGCTGGAGGAGGAGATCCAATT
TTATATCAACATTTATTT---------
>NSWHN542-11|Emmalocera_latilimbella|BIOUG00981-F11|
AACTTTATATTTTATTTTTGGAATTTGATCAGGAATAGTAGGAACATCTATAAGTTTACTTATTCGAGCTGAATTAGGAA
CTCCTGGATCTTTAATTGGAGATGACCAAATTTATAATACTATTGTTACTGGTCATGCTTTTATTATAATTTTTTTTATA
GTTATACCTATTATAATTGGCGGATTTGGAAATTGATTAGTTCCTTTAATATTAGGGGCCCCAGATATAGCTTTTCCTCG
AATAAATAATATAAGATTCTGACTCTTACCCCCTTCCCTTAATTTATTAATTTTTAGAAGAATTGTAGAAAATGGAGCAG
GAACAGGATGAACAGTTTATCCCCCTTTATCCTCTAATATTGCCCATAGAGGTAGATCTGTTGATCTTGCTATTTTTTCT
TTACATTTAGCAGGAATTTCTTCTATTTTAGGAGCTATTAACTTTATTACTACTATTATTAATATAAAATTAAATGGATT
ATCATTTGATCAAATACCTTTATTTGTATGAGCTGTAGGAATTACAGCTTTATTATTACTTTTATCTTTACCAGTATTAG
CAGGAGCTATTACTATATTATTAACTGATCGAAATTTAAATACTTCTTTTTTTGACCCTGCTGGAGGAGGAGATCCAATT
TTATATCAACATTTATTT---------
>NSWHN553-11|Emmalocera|BIOUG00981-G10|
AACTTTATATTTTATTTTTGGAATTTGAGCAGGAATAGTAGGAACATCTTTAAGTCTTTTAATTCGAGCTGAATTAGGAA
CCCCAGGATCTTTAATTGGTGATGACCAAATTTATAATACTATTGTAACTGGTCATGCTTTTATTATAATTTTTTTTATA
GTGATACCTATTATAATTGGAGGATTCGGAAATTGATTAGTACCTTTAATACTAGGGGCCCCAGATATAGCTTTCCCACG
AATAAATAATATAAGATTTTGACTTTTACCTCCTTCTCTAACTTTATTAGTCTCTAGAAGAATTGTAGAAAATGGAGCAG
GTACTGGATGAACTGTATACCCCCCTTTATCTTCTAATATTGCCCATGGAGGCAGCTCTGTAGATCTTGCTATTTTTTCT
CTTCATTTAGCAGGAATTTCTTCTATTTTAGGAGCTATTAATTTTATTACCACTATTATTAATATAAAATTAAATGGATT
ATCATTTGACCAAATACCTTTATTTGTTTGAGCTGTAGGTATTACAGCTTTACTTCTTTTATTATCTTTACCTGTATTAG
CAGGAGCTATTACTATACTATTAACTGATCGAAATTTAAATACTTCTTTCTTTGACCCTGCTGGAGGGGGAGACCCAATT
TTATACCAACATTTATTT---------
>NSWHN602-11|Emmalocera|BIOUG00982-C12|
AACTTTATATTTTATTTTTGGAATTTGAGCAGGAATAGTAGGAACATCTTTAAGTCTTTTAATTCGAGCTGAATTAGGAA
CCCCAGGATCTTTAATTGGTGATGACCAAATTTATAATACTATTGTAACTGGTCATGCTTTTATTATAATTTTTTTTATA
GTGATGCCTATTATAATTGGAGGATTCGGAAATTGATTAGTACCTTTAATACTAGGGGCCCCAGATATAGCTTTCCCACG
AATAAATAATATAAGATTTTGACTTTTACCTCCTTCTCTAACTTTATTAGTCTCTAGAAGAATTGTAGAAAATGGAGCAG
GTACTGGATGAACTGTATACCCCCCTTTATCTTCTAATATTGCCCATGGAGGCAGCTCTGTAGATCTTGCTATTTTTTCT
CTTCATTTAGCAGGAATTTCTTCTATTTTAGGAGCTATTAATTTTATTACCACTATTATTAATATAAAATTAAATGGATT
ATCATTTGACCAAATACCTTTATTTGTTTGAGCTGTAGGTATTACAGCTTTACTTCTTTTATTATCTTTACCTGTATTAG
CAGGAGCTATTACTATACTATTAACTGATCGAAATTTAAATACTTCTTTCTTTGACCCTGCTGGAGGGGGAGACCCAATT
TTATACCAACATTTATTT---------
>NSWHN820-11|Emmalocera|BIOUG00984-F04|
AACTTTATATTTTATTTTTGGAATTTGAGCAGGAATAGTAGGAACATCTTTAAGTCTTTTAATTCGAGCTGAATTAGGAA
CCCCAGGATCTTTAATTGGTGATGACCAAATTTATAATACTATTGTAACTGGTCATGCTTTTATTATAATTTTTTTTATA
GTGATACCTATTATAATTGGAGGATTCGGAAATTGATTAGTACCTTTAATACTAGGGGCCCCAGATATAGCTTTCCCACG
AATAAATAATATAAGATTTTGACTTTTACCTCCTTCTCTAACTTTATTAGTCTCTAGAAGAATTGTAGAAAATGGAGCAG
GTACTGGATGAACTGTATACCCCCCTTTATCTTCTAATATTGCCCATGGAGGCAGCTCTGTAGATCTTGCTATTTTTTCT
CTTCATTTAGCAGGAATTTCTTCTATTTTAGGAGCTATTAATTTTATTACCACTATTATTAATATAAAATTAAATGGATT
ATCATTTGACCAAATACCTTTATTTGTTTGAGCTGTAGGTATTACAGCTTTACTTCTTTTATTATCTTTACCTGTATTAG
CAGGAGCTATTACTATACTATTAACTGATCGAAATTTAAATACTTCTTTCTTTGACCCTGCTGGAGGGGGAGACCCAATT
TTATACCAACATTTATTT---------
>NSWHN897-11|Emmalocera|BIOUG00985-D10|
AACTTTATATTTTATTTTTGGAATTTGAGCAGGAATAGTAGGAACATCTTTAAGTCTTTTAATTCGAGCTGAATTAGGAA
CCCCAGGATCTTTAATTGGTGATGACCAAATTTATAATACTATTGTAACTGGTCATGCTTTTATTATAATTTTTTTTATA

GTGATACCTATTATAATTGGAGGATTCGGAAATTGATTAGTACCTTTAATACTAGGGGCCCCAGATATAGCTTTCCCACG
AATAAATAATATAAGATTTTGACTTTTACCTCCTTCTCTAACTTTATTAGTCTCTAGAAGAATTGTAGAAAATGGAGCAG
GTACTGGATGAACTGTATACCCCCCTTTATCTTCTAATATTGCCCATGGAGGCAGCTCTGTAGATCTTGCTATTTTTTCT
CTTCATTTAGCAGGAATTTCTTCTATTTTAGGAGCTATTAATTTTATTACCACTATTATTAATATAAAATTAAATGGATT
ATCATTTGACCAAATACCTTTATTTGTTTGAGCTGTAGGTATTACAGCTTTACTTCTTTTATTATCTTTACCTGTATTAG
CAGGAGCTATTACTATACTATTAACTGATCGAAATTTAAATACTTCTTTCTTTGACCCTGCTGGAGGGGGAGACCCAATT
TTATACCAACATTTATTT---------
>NSWHN903-11|Emmalocera|BIOUG00985-E04|
AACTTTATATTTTATTTTTGGAATTTGAGCAGGAATAGTAGGAACATCTTTAAGTCTTTTAATTCGAGCTGAATTAGGAA
CCCCAGGATCTTTAATTGGTGATGACCAAATTTATAATACTATTGTAACTGGTCATGCTTTTATTATAATTTTTTTTATA
GTGATACCTATTATAATTGGAGGATTCGGAAATTGATTAGTACCTTTAATACTAGGGGCCCCAGATATAGCTTTCCCACG
AATAAATAATATAAGATTTTGACTTTTACCTCCTTCTCTAACTTTATTAGTCTCTAGAAGAATTGTAGAAAATGGAGCAG
GTACTGGATGAACTGTATACCCCCCTTTATCTTCTAATATTGCCCATGGAGGCAGCTCTGTAGATCTTGCTATTTTTTCT
CTTCATTTAGCAGGAATTTCTTCTATTTTAGGAGCTATTAATTTTATTACCACTATTATTAATATAAAATTAAATGGATT
ATCATTTGACCAAATACCTTTATTTGTTTGAGCTGTAGGTATTACAGCTTTACTTCTTTTATTATCTTTACCTGTATTAG
CAGGAGCTATTACTATACTATTAACTGATCGAAATTTAAATACTTCTTTCTTTGACCCTGCTGGAGGGGGAGACCCAATT
TTATACCAACATTTATTT---------
>NSWHN941-11|Emmalocera|BIOUG00985-H06|
-----------------------TTTGAGCAGGAATAGTAGGAACATCTTTAAGTCTTTTAATTCGAGCTGAATTAGGAA
CCCCAGGATCTTTAATTGGTGATGACCAAATTTATAATACTATTGTAACTGGTCATGCTTTTATTATAATTTTTTTTATA
GTGATACCTATTATAATTGGAGGATTCGGAAATTGATTAGTACCTTTAATACTAGGGGCCCCAGATATAGCTTTCCCACG
AATAAATAATATAAGATTTTGACTTTTACCTCCTTCTCTAACTTTATTAGTCTCTAGAAGAATTGTAGAAAATGGAGCAG
GTACTGGATGAACTGTATACCCCCCTTTATCTTCTAATATTGCCCATGGAGGCAGCTCTGTAGATCTTGCTATTTTTTCT
CTTCATTTAGCAGGAATTTCTTCTATTTTAGGAGCTATTAATTTTATTACCACTATTATTAATATAAAATTAAATGGATT
ATCATTTGACCAAATACCTTTATTTGTTTGAGCTGTAGGTATTACAGCTTTACTTCTTTTATTATCTTTACCTGTATTAG
CAGGAGCTATTACTATACTATTAACTGATCGAAATTTAAATACTTCTTTCTTTGACCCTGCTGGAGGGGGAGACCCAATT
TTATACCAACATTTATTT---------
>NSWLP283-13|Acrapex_exsanguis|BIOUG04701-H09|
AACATTATATTTTATTTTTGGAATTTGAGCTGGTATACTAGGAACTTCTTTAAGTTTATTAATCCGAGCTGAATTAGGAA
CTCCAGAATCTTTAATTGGAGATGATCAAATTTATAATACTATTGTTACTGCTCACGCTTTTATTATAATTTTCTTTATA
GTTATACCAATTATAATTGGAGGATTTGGAAATTGACTTGTCCCATTAATATTAGGAGCTCCAGATATAGCATTTCCACG
TATAAATAATATAAGATTTTGATTATTACCTCCCTCTTTAAGTTTATTAATTTCAAGAAGAATTGTAGAAAATGGAGCAG
GAACTGGGTGAACAGTATATCCTCCACTCTCATCTAATATTGCTCATAGAGGAAGATCAGTAGATTTAGCTATTTTTTCT
CTTCATTTAGCTGGTATTTCATCTATTTTAGGAGCTATTAATTTTATTACAACAATTATTAATATACGATTAAATAATTT
ATCTTTTGATCAAATACCTTTATTTGTTTGAGCTGTTGGAATTACTGCATTTTTACTATTACTCTCATTACCTGTATTAG
CCGGAGCTATTACAATATTATTAACAGATCGAAATTTAAATACATCATTTTTTGATCCTGCAGGAGGAGGT---------
----
>NSWLP310-13|Acrapex_exsanguis|BIOUG04702-C01|
AACATTATATTTTATTTTTGGAATTTGAGCTGGTATACTAGGAACTTCTTTAAGTTTATTAATCCGAGCTGAACTAGGAA
CTCCAGAATCTTTAATTGGAGATGATCAAATTTACAATACTATTGTTACTGCTCACGCTTTTATTATAATTTTTTTTATA
GTTATACCAATTATAATTGGAGGATTTGGAAATTGACTTGTCCCATTAATATTAGGAGCTCCAGATATAGCATTCCCACG
TATAAATAATATAAGATTTTGATTATTACCTCCCTCTTTAAGTTTATTAATTTCAAGAAGAATTGTAGAAAATGGAGCAG
GAACTGGATGAACAGTATATCCCCCACTCTCATCTAATATTGCTCATAGAGGAAGATCAGTAGACTTAGCTATTTTTTCT
CTTCATTTAGCTGGTATTTCATCTATTTTAGGAGCTATTAATTTTATTACAACAATTATTAATATACGATTAAATAATTT
ATCTTTTGATCAAATACCTTTATTTGTTTGAGCTGTTGGAATTACTGCATTTTTACTATTACTCTCATTACCCGTATTAG
CCGGAGCTATTACAATATTATTAACAGATCGAAATTTAAATAC---------------------------------------
----
>NSWLP316-13|Emmalocera|BIOUG04702-C07|
AACCTTATATTTTATTTTTGGAATTTGATCAGGAATAGTAGGAACATCTTTAAGTTTACTTATTCGAGCTGAATTAGGAA
CTCCTGGATCTTTAATTGGAGATGACCAAATTTACAACACTATTGTTACTGGTCATGCTTTTATTATAATTTTCTTTATA
GTTATACCTATTATAATTGGTGGATTTGGAAATTGATTAATTCCTTTAATATTAGGTGCTCCAGATATAGCTTTCCCTCG
AATAAATAATATAAGATTTTGACTTTTACCCCCCTCTCTTAATTTATTAATTTCTAGAAGAATCGTAGAAAATGGAGCAG
GAACAGGATGAACTGTATATCCTCCTTTATCTTCTAATATTGCTCATAGTGGAAGATCTGTTGATCTTGCCATTTTTTCT
TTACATTTAGCAGGAATTTCTTCTATTTTAGGAGCTATTAATTTTATTACCACTATTATTAATATAAAATTAAATGGATT
AATATTTGATCAAATACCTTTATTTGTATGAGCTGTAGGTATTACAGCTTTATTATTACTTTTATCATTACCAGTATTAG
CTGGAGCTATTACTATGTTATTAACTGATCGAAATTTAAATACTTCTTTTTTTGATCCTGCTGGAGGGGGAGACCC----
----

>NSWLP333-13|Acrapex_exsanguis|BIOUG04702-D12|
AACATTATATTTTATTTTTGGAATTTGAGCTGGTATACTAGGAACTTCTTTAAGTTTATTAATCCGAGCTGAATTAGGAA
CTCCAGAATCTTTAATTGGAGATGATCAAATTTATAATACTATTGTTACTGCTCACGCTTTTATTATAATTTTCTTTATA
GTTATACCAATTATAATTGGAGGATTTGGAAATTGACTTGTCCCATTAATATTAGGAGCTCCAGATATAGCATTTCCACG
TATAAATAATATAAGATTTTGATTATTACCTCCCTCTTTAAGTTTATTAATTTCAAGAAGAATTGTAGAAAATGGAGCAG
GAACTGGGTGAACAGTATATCCTCCACTCTCATCTAATATTGCTCATAGAGGAAGATCAGTAGATTTAGCTATTTTTTCT
CTTCATTTAGCTGGTATTTCATCTATTTTAGGAGCTATTAATTTTATTACAACAATTATTAATATACGATTAAATAATTT
ATCTTTTGATCAAATACCTTTATTTGTTTGAGCTGTTGGAATTACTGCATTTTTACTATTACTCTCATTACCTGTATTAG
CCGGAGCTATTACAATATTATTAACAGATCGAAATTTAAATACATCCTTTTTTGATCCT--------------------
----
>NSWLP349-13|Emmalocera|BIOUG04702-F04|
AACCTTATATTTTATTTTTGGAATTTGATCAGGAATGGTAGGAACATCTTTAAGTTTACTCATTCGAGCTGAATTAGGAA
CTCCTGGGTCTTTAATTGGAAATGATCAAATTTACAATACTATTGTTACTGGTCATGCTTTTATTATAATTTTCTTTATA
GTTATACCTATTATAATTGGTGGATTTGGAAATTGATTAATTCCTTTAATATTAGGTGCTCCAGATATAGCTTTCCCTCG
AATAAATAATATAAGATTTTGACTTTTACCCCCCTCTCTTAATTTATTAATTTCTAGAAGCATCGTAGAAAATGGAGCAG
GAACAGGATGAACTGTATACCCTCCTTTATCTTCTAATATTGCTCACAGTGGAAGATCTGTTGATCTTGCCATCTTTTCT
TTACATTTAGCAGGAATTTCTTCTATTTTAGGAGCTATTAATTTTATTACCACTATTATTAATATAAAATTAAATGGATT
AATATTTGATCAAATACCTTTATTTGTATGAGCTGTAGGTATTACAGCTTTATTATTACTTTTATCATTACCAGTATTAG
CTGGAGCTATTACTATGTTATTAACTGATCGAAATTTAAATACTTCTTTTTTTGACCCTGCT-----------------
----
>NSWLP371-13|Acrapex_exsanguis|BIOUG04702-H02|
AACATTATATTTTATTTTTGGAATTTGAGCTGGTATACTAGGAACTTCTTTAAGTTTATTAATCCGAGCTGAATTAGGAA
CTCCAGAATCTTTAATTGGAGATGATCAAATTTATAATACTATTGTTACTGCTCACGCTTTTATTATAATTTTTTTTATA
GTTATACCAATTATAATTGGAGGATTTGGAAATTGACTTGTCCCATTAATATTAGGAGCTCCAGATATAGCATTCCCACG
TATAAATAATATAAGATTTTGATTATTACCTCCCTCTTTAAGTTTATTAATTTCAAGAAGAATTGTAGAAAATGGAGCAG
GAACTGGATGAACAGTATATCCCCCACTCTCATCTAATATTGCTCATAGAGGAAGATCAGTAGATTTAGCTATTTTTTCT
CTTCATTTAGCTGGTATTTCATCTATTTTAGGAGCTATTAATTTTATTACAACAATTATTAATATACGATTAAATAATTT
ATCTTTTGATCAAATACCTTTATTTGTTTGAGCTGTTGGAATTACTGCATTTTTACTATTACTCTCATTACCCGTATTAG
CCGGGGCTATTACAATATTATTAACAGATCGAAATTTAAATACATCATTTTTTGATCCTGCAGGAGGAGGT---------
----
>NSWLP408-13|Emmalocera_latilimbella|BIOUG04703-C04|
AACTTTATATTTTATTTTTGGAATTTGATCAGGAATAGTAGGAACATCTATAAGTTTACTTATTCGAGCTGAATTAGGAA
CTCCTGGATCTTTAATTGGAGATGACCAAATTTATAATACTATTGTTACTGGTCATGCTTTTATTATAATTTTTTTTATA
GTTATACCTATTATAATTGGCGGATTTGGAAATTGATTAGTTCCTTTAATATTAGGAGCCCCAGATATAGCTTTTCCTCG
AATAAATAATATAAGATTCTGACTCTTACCCCCTTCCCTTAATTTATTAATTTTTAGAAGAATTGTAGAAAATGGAGCAG
GAACAGGATGAACAGTTTATCCCCCTTTATCCTCTAATATTACCCATAGAGGTAGATCTGTTGATCTTGCTATTTTTTCT
TTACATTTAGCAGGAATTTCTTCTATTTTAGGAGCTATTAACTTTATTACTACTATTATTAATATAAAATTAAATGGATT
ATCATTTGATCAAATACCTTTATTTGTATGAGCTGTAGGAATTACAGCTTTATTATTACTTTTATCTTTACCAGTATTAG
CAGGAGCTATTACTATATTATTAACTGATCGAAATTTAAATACTTCTTTTTTTGACCCTGC------------------
----
>NSWLP446-13|Acrapex_exsanguis|BIOUG04703-F06|
AACATTATATTTTATTTTTGGAATTTGAGCTGGTATACTAGGAACTTCTTTAAGTTTATTAATCCGAGCTGAACTAGGAA
CTCCAGAATCTTTAATTGGAGATGATCAAATTTACAATACTATTGTTACTGCTCACGCTTTTATTATAATTTTTTTTATA
GTTATACCAATTATAATTGGAGGATTTGGAAATTGACTTGTCCCATTAATATTAGGAGCTCCAGATATAGCATTTCCACG
TATAAATAATATAAGATTTTGATTATTACCTCCCTCTTTAAGTTTATTAATTTCAAGAAGAATTGTAGAAAATGGAGCAG
GAACTGGATGAACAGTATATCCCCCACTCTCATCTAATATTGCTCATAGAGGAAGATCAGTAGACTTAGCTATTTTTTCT
CTTCATTTAGCTGGTATTTCATCTATTTTAGGAGCTATTAATTTTATTACAACAATTATTAATATACGATTAAATAATTT
ATCTTTTGATCAAATACCTTTATTTGTTTGAGCTGTTGGAATTACTGCATTTTTACTATTACTCTCATTACCCGTATTAG
CCGGAGCTATTACAATATTATTAACAGATCGAAATTTAAATA------------------------------------
----
>NSWLP472-13|Bathytricha_aethalion|BIOUG04703-H08|
AACATTATATTTTATTTTTGGGATTTGAGCAGGGATAGTAGGAACTTCTTTAAGACTACTAATTCGGGCTGAACTAGGAA
CTCCTGGATCTCTAATTGGGGATGATCAAATTTATAATACTATTGTAACAGCTCATGCTTTTATTATAATTTTTTTTATG
GTTATACCAATCATAATTGGAGGATTTGGAAATTGACTTGTACCTTTAATATTAGGAGCGCCTGATATGGCATTCCCACG
AATAAATAACATAAGTTTTTGATTACTACCACCTTCTTTAACTCTTCTTATTTCAAGTAGAGTTGTAGAAAATGGAGCGG
GAACTGGATGAACAGTTTATCCACCACTCTCATCTAATATTGCTCATGGAGGAAGATCAGTGGACCTAGCTATTTTTTCC
CTCCATTTAGCTGGAATCTCTTCAATTCTAGGAGCTATTAATTTTATTACAACTATTATTAATATACGATTAAATAACTT

ATCTTTTGATCAAATACCTTTATTTATTTGAGCCGTAGGAATTACAGCTTTTTTATTATTATTATCATTACCTGTATTAG
CTGGAGCTATTACTATATTACTAACAGATCGAAATTTAAATACATCATTTTTCGATCCTGCAGGAGGGGG----------
----
>NSWLP476-13|Scirpophaga_nivella|BIOUG04704-A01|
AACTTTATATTTCATTTTTGGAATTTGAGCTGGTATAGTAGGAACTTCTTTAAGATTATTAATTCGAGCTGAATTAGGAA
CTCCAGGATCTTTAATTGGAGATGATCAAATTTATAATACCATTGTTACAGCTCATGCTTTTATTATAATTTTTTTTATA
GTAATGCCAATTATAATTGGAGGGTTTGGAAATTGACTTGTTCCTTTAATATTAGGAGCTCCTGATATAGCTTTCCCTCG
TATAAATAATATAAGATTTTGATTATTACCCCCCTCATTAACTCTCCTAATTTCAAGAAGAATTGTAGAAAATGGTGCAG
GAACAGGGTGAACAGTATACCCCCCCTTATCATCAAATATTGCTCATGGAGGAACTTCTGTAGATTTAGCTATTTTTTCT
TTACATCTTGCAGGAATTTCCTCTATTTTAGGAGCTATTAACTTTATTACCACTATTATTAATATACGAATTAATGGCTT
AACATTTGATCAAATACCTCTCTTTGTTTGAGCTGTTGGAATTACAGCCCTTCTTTTACTCCTCTCATTACCCGTATTAG
CTGGAGCTATTACTATATTATTAACTGATCGAAATTTAAATACCTCTTTTTTTGATCCAGCGGGAGGAGGAGA-------
----
>NSWLP478-13|Scirpophaga_nivella|BIOUG04704-A03|
AACTTTATATTTCATTTTTGGAATTTGAGCTGGTATAGTAGGAACTTCTTTAAGATTATTAATTCGAGCTGAATTAGGAA
CTCCAGGATCTTTAATTGGAGATGATCAAATTTATAATACCATTGTTACAGCTCATGCTTTTATTATAATTTTTTTTATA
GTAATGCCAATTATAATTGGAGGGTTTGGAAATTGACTTGTTCCTTTAATATTAGGAGCTCCTGATATAGCTTTCCCTCG
TATAAATAATATAAGATTTTGATTATTACCCCCCTCATTAACTCTCCTAATTTCAAGAAGAATTGTAGAAAATGGTGCAG
GAACAGGGTGAACAGTATACCCCCCCTTATCATCAAATATTGCTCATGGAGGAACTTCTGTAGATTTAGCTATTTTTTCT
TTACATCTTGCAGGAATTTCCTCTATTTTAGGAGCTATTAACTTTATTACCACTATTATTAATATACGAATTAATGGCTT
AACATTTGATCAAATACCTCTCTTTGTTTGAGCTGTTGGAATTACAGCCCTTCTTTTACTCCTCTCATTACCCGTATTAG
CTGGAGCTATTACTATATTATTAACTGATCGAAATTTAAATACCTCTTTTTTTGATCCAGCGGGAGGAGGAG--------
----
>NSWLP494-13|Scirpophaga_nivella|BIOUG04704-B07|
AACTTTATATTTCATTTTTGGAATTTGAGCTGGTATAGTAGGAACTTCTTTAAGATTATTAATTCGAGCTGAATTAGGAA
CTCCAGGATCTTTAATTGGAGATGATCAAATTTATAATACCATTGTTACAGCTCATGCTTTTATTATAATTTTTTTTATA
GTAATGCCAATTATAATTGGAGGGTTTGGAAATTGACTTGTTCCTTTAATATTAGGAGCTCCTGATATAGCTTTCCCTCG
TATAAATAATATAAGATTTTGATTATTACCCCCCTCATTAACTCTCCTAATTTCAAGAAGAATTGTAGAAAATGGTGCAG
GAACAGGGTGAACAGTATACCCCCCCTTATCATCAAATATTGCTCATGGAGGAACTTCTGTAGATTTAGCTATTTTTTCT
TTACATCTTGCAGGAATTTCCTCTATTTTAGGAGCTATTAACTTTATTACCACTATTATTAATATACGAATTAATGGCTT
AACATTTGATCAAATACCTCTCTTTGTTTGAGCTGTTGGAATTACAGCCCTTCTTTTACTCCTCTCATTACCCGTATTAG
CTGGAGCTATTACTATATTATTAACTGATCGAAATTTAAATACCTCTTTTTTTGATCCAGCGGGAGGAGGAGA-------
----
>NSWLP526-13|Scirpophaga_nivella|BIOUG04704-E03|
AACTTTATATTTCATTTTTGGAATTTGAGCTGGTATAGTAGGAACTTCTTTAAGATTATTAATTCGAGCTGAATTAGGAA
CTCCAGGATCTTTAATTGGAGATGATCAAATTTATAATACCATTGTTACAGCTCATGCTTTTATTATAATTTTTTTTATA
GTAATGCCAATTATAATTGGAGGGTTTGGAAATTGACTTGTTCCTTTAATATTAGGAGCTCCTGATATAGCTTTCCCTCG
TATAAATAATATAAGATTTTGATTATTACCCCCCTCATTAACTCTCCTAATTTCAAGAAGAATTGTAGAAAATGGTGCAG
GAACAGGGTGAACAGTATACCCCCCCTTATCATCAAATATTGCTCATGGAGGAACTTCTGTAGATTTAGCTATTTTTTCT
TTACATCTTGCAGGAATTTCCTCTATTTTAGGAGCTATTAACTTTATTACCACTATTATTAATATACGAATTAATGGCTT
AACATTTGATCAAATACCTCTCTTTGTTTGAGCTGTTGGAATTACAGCCCTTCTTTTACTCCTCTCATTACCCGTATTAG
CTGGAGCTATTACTATATTATTAACTGATCGAAATTTAAATACCTCTTTTTTTGATCCAGCGGGAGGAGGAGATCC----
----
>NSWLP577-13|Scirpophaga_nivella|BIOUG04705-A07|
AACTTTATATTTCATTTTTGGAATTTGAGCTGGTATAGTAGGAACTTCTTTAAGATTATTAATTCGAGCTGAATTAGGAA
CTCCAGGATCTTTAATTGGAGATGATCAAATTTATAATACCATTGTTACAGCTCATGCTTTTATTATAATTTTTTTTATA
GTAATGCCAATTATAATTGGAGGGTTTGGAAATTGACTTGTTCCTTTAATATTAGGAGCTCCTGATATAGCTTTCCCTCG
TATAAATAATATAAGATTTTGATTATTACCCCCCTCATTAACTCTCCTAATTTCAAGAAGAATTGTAGAAAATGGTGCAG
GAACAGGGTGAACAGTATACCCCCCCTTATCATCAAATATTGCTCATGGAGGAACTTCTGTAGATTTAGCTATTTTTTCT
TTACATCTTGCAGGAATTTCCTCTATTTTAGGAGCTATTAACTTTATTACCACTATTATTAATATACGAATTAATGGCTT
AACATTTGATCAAATACCTCTCTTTGTTTGAGCTGTTGGAATTACAGCCCTTCTTTTACTCCTCTCATTACCCGTATTAG
CTGGAGCTATTACTATATTATTAACTGATCGAAATTTAAATACCTCTTTTTTTGATCCAGCGGGAGGAGGAGA-------
----
>NSWLP604-13|Emmalocera_latilimbella|BIOUG04705-C10|
AACTTTATATTTTATTTTTGGAATTTGATCAGGAATAGTAGGAACATCTATAAGTTTACTTATTCGAGCTGAATTAGGAA
CTCCTGGATCTTTAATTGGAGATGACCAAATTTATAATACTATTGTTACTGGTCATGCTTTTATTATAATTTTTTTTATA
GTTATACCTATTATAATTGGCGGATTTGGAAATTGATTAGTTCCTTTAATATTAGGAGCCCCAGATATAGCTTTTCCTCG

AATAAATAATATAAGATTCTGACTCTTACCCCCTTCCCTTAATTTATTAATTTTTAGAAGAATTGTAGAAAATGGAGCAG
GAACAGGATGAACAGTTTATCCCCCTTTATCCTCTAATATTGCCCATAGAGGTAGATCTGTTGATCTTGCTATTTTTTCT
TTACATTTAGCAGGAATTTCTTCTATTTTAGGAGCTATTAACTTTATTACTACTATTATTAATATAAAATTAAATGGATT
ATCATTTGATCAAATACCTTTATTTGTATGAGCTGTAGGAATTACAGCTTTATTATTACTTTTATCTTTACCAGTATTAG
CAGGAGCTATTACTATATTATTAACTGATCGAAATTTAAATACTTCTTTTTTTGACCCTGCTGGAGGAGGAGACCC----
----
>NSWLP608-13|Chilo|BIOUG04705-D02|
AACTTTATATTTTATTTTTGGAATTTGAGCAGGAACAGTAGGAACTTCATTAAGTCTTATAATTCGAGCTGAATTAGGAA
ATCCAGGATCTTTAATTGGAGATGATCAAATTTATAACACTATTGTTACAGCACATGCATTTATTATAATTTTTTTTATG
GTAATGCCAATTATAATTGGAGGTTTTGGAAATTGATTAGTACCCCTAATACTAGGAGCCCCAGACATAGCATTTCCACG
AATAAATAACATAAGATTTTGACTTTTACCACCATCATTAACTTTATTAATTTCAAGAAGAATTGTAGAAAATGGAGCAG
GAACAGGATGAACAGTGTATCCCCCCCTCTCATCCAATATTGCTCACGGGGGAAGATCTGTGGATTTAGCTATCTTTTCC
CTACACTTAGCTGGAATTTCATCAATTCTTGGTGCTATTAATTTTATCACAACAATTATTAACATACGAATTAATAACTT
ATCATTTGATCAAATACCTCTATTTGTTTGATCTGTAGGAATTACAGCTCTTTTATTACTCCTCTCTCTACCTGTATTAG
CTGGAGCTATTACTATATTATTAACCGATCGAAACTTAAATACATCCTTCTTTGACCCAGCGGGAGGGGGAGACCC----
----
>NSWLP614-13|Scirpophaga_nivella|BIOUG04705-D08|
AACTTTATATTTCATTTTTGGAATTTGAGCTGGTATAGTAGGAACTTCTTTAAGATTATTAATTCGAGCTGAATTAGGAA
CTCCAGGATCTTTAATTGGAGATGATCAAATTTATAATACCATTGTTACAGCTCATGCTTTTATTATAATTTTTTTTATA
GTAATGCCAATTATAATTGGAGGGTTTGGAAATTGACTTGTTCCTTTAATATTAGGAGCTCCTGATATAGCTTTCCCTCG
TATAAATAATATAAGATTTTGATTATTACCCCCCTCATTAACTCTCCTAATTTCAAGAAGAATTGTAGAAAATGGTGCAG
GAACAGGGTGAACAGTATACCCCCCCTTATCATCAAATATTGCTCATGGAGGAACTTCTGTAGATTTAGCTATTTTTTCT
TTACATCTTGCAGGAATTTCCTCTATTTTAGGAGCTATTAACTTTATTACCACTATTATTAATATACGAATTAATGGCTT
AACATTTGATCAAATACCTCTCTTTGTTTGAGCTGTTGGAATTACAGCCCTTCTTTTACTCCTCTCATTACCCGTATTAG
CTGGAGCTATTACTATATTATTAACTGATCGAAATTTAAATACCTCTTTTTTTGATCCAGCGGGAGGAGGAGATCC----
----
>NSWLP660-13|Scirpophaga_nivella|BIOUG04705-H06|
AACTTTATATTTCATTTTTGGAATTTGAGCTGGTATAGTAGGAACTTCTTTAAGATTATTAATTCGAGCTGAATTAGGAA
CTCCAGGATCTTTAATTGGAGATGATCAAATTTATAATACCATTGTTACAGCTCATGCTTTTATTATAATTTTTTTTATA
GTAATACCAATTATAATTGGAGGGTTTGGAAATTGACTTGTTCCTTTAATATTAGGAGCTCCTGATATAGCTTTCCCTCG
TATAAATAATATAAGATTTTGATTATTACCCCCCTCATTAACTCTCCTAATTTCAAGAAGAATTGTAGAAAATGGTGCAG
GAACAGGATGAACAGTATACCCCCCCTTATCATCAAATATTGCTCATGGAGGAACTTCTGTAGATTTAGCTATTTTTTCT
CTACATCTTGCAGGAATTTCCTCTATTTTAGGAGCTATTAACTTTATTACCACTATTATTAATATACGAATTAATGGCTT
AACATTTGATCAAATACCTCTCTTTGTTTGAGCTGTTGGAATTACAGCCCTTCTTTTACTCCTCTCATTACCCGTATTAG
CTGGAGCTATTACTATATTATTAACTGATCGAAATTTAAATACCTCTTTTTTTGATCCAGCGGGAGGAGGAGATCC----
----
>NSWLP667-13|Emmalocera_latilimbella|BIOUG04706-A02|
AACTTTATATTTTATTTTTGGAATTTGATCAGGAATAGTAGGAACATCTATAAGTTTACTTATTCGAGCTGAATTAGGAA
CTCCTGGATCTTTAATTGGAGATGACCAAATTTATAATACTATTGTTACTGGTCATGCTTTTATTATAATTTTTTTTATA
GTTATACCTATTATAATTGGCGGATTTGGAAATTGATTAGTTCCTTTAATATTAGGAGCCCCAGATATAGCTTTTCCTCG
AATAAATAATATAAGATTCTGACTCTTACCCCCTTCCCTTAATTTATTAATTTTTAGAAGAATTGTAGAAAATGGAGCAG
GAACAGGATGAACAGTTTATCCCCCTTTATCCTCTAATATTACCCATAGAGGTAGATCTGTTGATCTTGCTATTTTTTCT
TTACATTTAGCAGGAATTTCTTCTATTTTAGGAGCTATTAACTTTATTACTACTATTATTAATATAAAATTAAATGGATT
ATCATTTGATCAAATACCTTTATTTGTATGAGCTGTAGGAATTACAGCTTTATTATTACTTTTATCTTTACCAGTATTAG
CAGGAGCTATTACTATATTATTAACTGATCGAAATTTAAATACTTCTTTTTTTGACCCTGCTGGAGGAGGAGA-------
----
>NSWLP682-13|Scirpophaga_nivella|BIOUG04706-B05|
AACTTTATATTTCATTTTTGGAATTTGAGCTGGTATAGTAGGAACTTCTTTAAGATTATTAATTCGAGCTGAATTAGGAA
CTCCAGGATCTTTAATTGGAGATGATCAAATTTATAATACCATTGTTACAGCTCATGCTTTTATTATAATTTTTTTTATA
GTAATGCCAATTATAATTGGAGGGTTTGGAAATTGACTTGTTCCTTTAATATTAGGAGCTCCTGATATAGCTTTCCCTCG
TATAAATAATATAAGATTTTGATTATTACCCCCCTCATTAACTCTCCTAATTTCAAGAAGAATTGTAGAAAATGGTGCAG
GAACAGGGTGAACAGTATACCCCCCCTTATCATCAAATATTGCTCATGGAGGAACTTCTGTAGATTTAGCTATTTTTTCT
TTACATCTTGCAGGAATTTCCTCTATTTTAGGAGCTATTAACTTTATTACCACTATTATTAATATACGAATTAATGGCTT
AACATTTGATCAAATACCTCTCTTTGTTTGAGCTGTTGGAATTACAGCCCTTCTTTTACTCCTCTCATTACCCGTATTAG
CTGGAGCTATTACTATATTATTAACTGATCGAAATTTAAATACCTCTTTTTTTGATCCAGCGGGAGGAGGAGAT------
----
>NSWLP696-13|Scirpophaga_nivella|BIOUG04706-C07|

AACTTTATATTTCATTTTTGGAATTTGAGCTGGTATAGTAGGAACTTCTTTAAGATTATTAATTCGAGCTGAATTAGGAA
CTCCAGGATCTTTAATTGGAGATGATCAAATTTATAATACCATTGTTACAGCTCATGCTTTTATTATAATTTTTTTTATA
GTAATGCCAATTATAATTGGAGGGTTTGGAAATTGACTTGTTCCTTTAATATTAGGAGCTCCTGATATAGCTTTCCCTCG
TATAAATAATATAAGATTTTGATTATTACCCCCCTCATTAACTCTCCTAATTTCAAGAAGAATTGTAGAAAATGGTGCAG
GAACAGGGTGAACAGTATACCCCCCCTTATCATCAAATATTGCTCATGGAGGAACTTCTGTAGATTTAGCTATTTTTTCT
TTACATCTTGCAGGAATTTCCTCTATTTTAGGAGCTATTAACTTTATTACCACTATTATTAATATACGAATTAATGGCTT
AACATTTGATCAAATACCTCTCTTTGTTTGAGCTGTTGGAATTACAGCCCTTCTTTTACTCCTCTCATTACCCGTATTAG
CTGGAGCTATTACTATATTATTAACTGATCGAAATTTAAATACCTCTTTTTTTGATCCAGCGGGAGGAGGAGATCCA---
----
>NSWLP732-13|Scirpophaga_nivella|BIOUG04706-F07|
AACTTTATATTTCATTTTTGGAATTTGAGCTGGTATAGTAGGAACTTCTTTAAGATTATTAATTCGAGCTGAATTAGGAA
CTCCAGGATCTTTAATTGGAGATGATCAAATTTATAATACCATTGTTACAGCTCATGCTTTTATTATAATTTTTTTTATA
GTAATGCCAATTATAATTGGAGGGTTTGGAAATTGACTTGTTCCTTTAATATTAGGAGCTCCTGATATAGCTTTCCCTCG
TATAAATAATATAAGATTTTGATTATTACCCCCCTCATTAACTCTCCTAATTTCAAGAAGAATTGTAGAAAATGGTGCAG
GAACAGGGTGAACAGTATACCCCCCCTTATCATCAAATATTGCTCATGGAGGAACTTCTGTAGATTTAGCTATTTTTTCT
TTACATCTTGCAGGAATTTCCTCTATTTTAGGAGCTATTAACTTTATTACCACTATTATTAATATACGAATTAATGGCTT
AACATTTGATCAAATACCTCTCTTTGTTTGAGCTGTTGGAATTACAGCCCTTCTTTTACTCCTCTCATTACCCGTATTAG
CTGGAGCTATTACTATATTATTAACTGATCGAAATTTAAATACCTCTTTTTTTGATCCAGCGGGAGGAGGAGATCCA---
----
>NSWLP767-13|Emmalocera|BIOUG05361-A07|
GACCTTATATTTTATTTTTGGAATTTGATCAGGAATAGTAGGAACATCTTTAAGTTTACTTATTCGAGCTGAATTAGGAA
CTCCTGGATCTTTAATTGGAGATGATCAAATTTACAACACTATTGTTACTGGTCATGCTTTTATTATAATTTTCTTTATA
GTTATACCTATTATAATTGGTGGATTTGGAAATTGATTAATTCCTTTAATATTAGGTGCTCCAGATATAGCTTTCCCTCG
AATAAATAATATAAGATTTTGACTTTTACCCCCCTCTCTTAATTTATTAATTTCTAGAAGAATCGTAGAAAATGGAGCAG
GAACAGGATGAACTGTATACCCCCCTTTATCTTCTAATATTGCTCATAGTGGAAGATCTGTTGATCTTGCCATTTTTTCT
TTACATTTAGCAGGAATTTCTTCTATTTTAGGAGCTATTAATTTTATTACCACTATTATTAATATAAAATTAAATGGATT
AATATTTGATCAAATACCTTTATTTGTATGAGCTGTAGGTATTACAGCTTTATTATTACTTTTATCATTACCAGTATTAG
CTGGAGCTATTACTATGTTATTAACTGATCGAAATTTAAATACTTCTTTTTTTGA-------------------------
----
>NSWLP785-13|Scirpophaga_nivella|BIOUG05361-C01|
AACTTTATATTTCATTTTTGGAATTTGAGCTGGTATAGTAGGAACTTCTTTAAGATTATTAATTCGAGCTGAATTAGGAA
CTCCAGGATCTTTAATTGGAGATGATCAAATTTATAATACCATTGTTACAGCTCATGCTTTTATTATAATTTTTTTTATA
GTAATACCAATTATAATTGGAGGGTTTGGAAATTGACTTGTTCCTTTAATATTAGGAGCTCCTGATATAGCTTTCCCTCG
TATAAATAATATAAGATTTTGATTATTACCCCCCTCATTAACTCTCCTAATTTCAAGAAGAATTGTAGAAAATGGTGCAG
GAACAGGATGAACAGTATACCCCCCCTTATCATCAAATATTGCTCATGGAGGAACTTCTGTAGATTTAGCTATTTTTTCT
CTACATCTTGCAGGAATTTCCTCTATTTTAGGAGCTATTAACTTTATTACCACTATTATTAATATACGAATTAATGGCTT
AACATTTGATCAAATACCTCTCTTTGTTTGAGCTGTTGGAATTACAGCCCTTCTTTTACTCCTCTCATTACCCGTATTAG
CTGGAGCTATTACTATATTATTAACTGATCGAAATTTAAATACCTCTTTTTTTGATCCAGCGGGAGGAGGAGA-------
----
>NSWLP786-13|Scirpophaga_imparellus|BIOUG05361-C02|
AACTTTATATTTTATTTTTGGTATTTGAGCTGGGATAGTGGGGACTTCTTTAAGTTTATTAATTCGAGCTGAATTAGGAA
CACCAGGATCATTAATTGGGGATGATCAAATTTATAATACTATTGTTACAGCCCATGCTTTTATTATAATTTTTTTTATA
GTAATACCTATTATAATTGGGGGATTCGGAAATTGACTAGTTCCCCTAATATTAGGAGCCCCTGATATAGCTTTTCCACG
TATAAATAATATAAGTTTTTGATTATTACCTCCCTCTCTTACTCTTTTAATTTCAAGAAGAATTGTTGAAAATGGAGCAG
GAACAGGATGAACTGTTTACCCCCCTTTATCCTCTAATATTGCCCATGGAGGAACATCTGTAGATCTAGCTATTTTTTCC
TTACATTTAGCGGGAATTTCATCTATTTTAGGAGCTATTAATTTTATTACAACTATTATTAATATACGAATTAATGGATT
ATCTTTTGATCAAATACCTTTATTTGTCTGAGCTGTAGGTATTACAGCACTTCTTTTACTTTTATCTTTACCTGTATTAG
CTGGAGCTATTACTATACTACTAACAGATCGAAATTTAAATACATCTTTTTTCGACCCAGCAGGTGGGGGAGA-------
----
>NSWLP827-13|Scirpophaga_nivella|BIOUG05361-F07|
AACTTTATATTTCATTTTTGGAATTTGAGCTGGTATAGTAGGAACTTCTTTAAGATTATTAATTCGAGCTGAATTAGGAA
CTCCAGGATCTTTAATTGGAGATGATCAAATTTATAATACCATTGTTACAGCTCATGCTTTTATTATAATTTTTTTTATA
GTAATGCCAATTATAATTGGAGGGTTTGGAAATTGACTTGTTCCTTTAATATTAGGAGCTCCTGATATAGCTTTCCCTCG
TATAAATAATATAAGATTTTGATTATTACCCCCCTCATTAACTCTCCTAATTTCAAGAAGAATTGTAGAAAATGGTGCAG
GAACAGGGTGAACAGTATACCCCCCCTTATCATCAAATATTGCTCATGGAGGAACTTCTGTAGATTTAGCTATTTTTTCT
CTACATCTTGCAGGAATTTCCTCTATTTTAGGAGCTATTAACTTTATTACCACTATTATTAATATACGAATTAATGGCTT
AACATTTGATCAAATACCTCTCTTTGTTTGAGCTGTTGGAATTACAGCCCTTCTTTTACTCCTCTCATTACCCGTATTAG

CTGGAGCTATTACTATATTATTAACTGATCGAAATTTAAATACCTCTTTTTTTGATCCAGCGGGAGGAGGAGA-------
----
>ODOPE480-11|Chilo_phragmitella|BC_ZSM_Lep_52971|KX045788
AACTTTATATTTTATTTTTGGAATTTGAGCTGGAATAATTGGAACATCTCTTAGACTTTTAATTCGAGCTGAATTAGGAA
CTCCAGGATCCCTAATTGGAGATGATCAAATTTATAATACTATTGTTACAGCTCATGCATTTATTATAATTTTTTTTATA
GTTATACCTATTATAATCGGTGGTTTTGGAAATTGATTAGTACCTTTAATATTAGGAGCCCCTGATATAGCTTTCCCACG
AATAAATAATATAAGATTTTGATTATTACCACCTTCATTAACCTTATTAATCTCTAGAAGAATTGTTGAAAATGGAGCTG
GAACAGGATGAACAGTGTACCCCCCACTTTCATCTAATATTGCTCATGCTGGAAGTTCAGTAGATTTAGCAATTTTTTCC
TTACATTTAGCTGGAATTTCATCAATTTTAGGTGCTATTAATTTTATTACAACAATTATTAATATACGAATTAATGGATT
ATCATTTGATCAAATACCCTTACTCATTTGAAGAATTGGTATTACAGCATTATTATTATTACTTTCTCTCCCAGTATTAG
CTGGTGCTATTACTATATTATTAACAGATCGAAATTTAAATACATCTTTTTTTGATCCAGCTGGAGGTGGAGATCCTATT
CTCTATCAACATTTATTT---------
>ODOPE830-11|Chilo_phragmitella|BC_ZSM_Lep_51231|KX040940
AACTTTATATTTTATTTTTGGAATTTGAGCTGGAATAATTGGAACATCTCTTAGACTTTTAATTCGAGCTGAATTAGGAA
CTCCAGGATCCCTAATTGGAGATGATCAAATTTATAATACTATTGTTACAGCTCATGCATTTATTATAATTTTTTTTATA
GTTATACCTATTATAATCGGTGGTTTTGGAAATTGATTAGTACCTTTAATATTAGGAGCCCCTGATATAGCTTTCCCACG
AATAAATAATATAAGATTTTGATTATTACCACCTTCATTAACCTTATTAATCTCTAGAAGAATTGTTGAAAATGGAGCTG
GAACAGGATGAACAGTGTACCCCCCACTTTCATCTAATATTGCTCATGCTGGAAGTTCAGTAGATTTAGCAATTTTTTCC
TTACATTTAGCTGGAATTTCATCAATTTTAGGTGCTATTAATTTTATTACAACAATTATTAATATACGAATTAATGGATT
ATCATTTGATCAAATACCCTTACTCATTTGAAGAATTGGTATTACAGCNTTATTATTATTACTTTCTCTCCCAGTATTAG
CTGGTGCTATTACTATATTATTAACAGATCGAAATTTAAATACATCTTTTTTTGATCCAGCTGGAGGTGGAGATCCTATT
CTCTATCAACATTTATTT---------
>PHLAA247-09|Chilo_phragmitella|TLMF_Lep_00287|HM425787-SUPPRESSED
AACTTTATATTTTATTTTTGGAATTTGAGCTGGAATAATTGGAACATCTCTTAGACTTTTAATTCGAGCTGAATTAGGAA
CTCCAGGATCCCTAATTGGAGATGATCAAATTTATAATACTATTGTTACAGCTCATGCATTTATTATAATTTTTTTTATA
GTTATACCTATTATAATCGGTGGTTTTGGAAATTGATTAGTACCTTTAATATTAGGAGCCCCTGATATAGCTTTCCCACG
AATAAATAATATAAGATTTTGATTATTACCACCTTCATTAACCTTATTAATCTCTAGAAGAATTGTTGAAAATGGAGCTG
GAACAGGATGAACAGTGTACCCCCCACTTTCATCTAATATTGCTCATGCTGGAAGTTCAGTAGATTTAGCAATTTTTTCC
TTACATTTAGCTGGAATTTCATCAATTTTAGGTGCTATTAATTTTATTACAACAATTATTAATATACGAATTAATGGATT
ATCATTTGATCAAATACCCTTACTCATTTGAAGAATTGGTATTACAGCATTATTATTATTACTTTCTCTCCCAGTATTAG
CTGGTGCTATTACTATATTATTAACAGATCGAAATTTAAATACATCTTTTTTTGATCCAGCTGGAGGTGGTG--------
----
>PHLAD520-11|Chilo_phragmitella|TLMF_Lep_03695|JN266141-SUPPRESSED
AACTTTATATTTTATTTTTGGAATTTGAGCTGGAATAATTGGAACATCTCTTAGACTTTTAATTCGAGCTGAATTAGGAA
CTCCAGGATCCCTAATTGGAGATGATCAAATTTATAATACTATTGTTACAGCTCATGCATTTATTATAATTTTTTTTATA
GTTATACCTATTATAATCGGTGGTTTTGGAAATTGATTAGTACCTTTAATATTAGGAGCCCCTGATATAGCTTTCCCACG
AATAAATAATATAAGATTTTGATTATTACCACCTTCATTAACCTTATTAATCTCTAGAAGAATTGTTGAAAATGGAGCTG
GAACAGGATGAACAGTGTACCCCCCACTTTCATCTAATATTGCTCATGCTGGAAGTTCAGTAGATTTAGCAATTTTTTCC
TTACATTTAGCTGGAATTTCATCAATTTTAGGTGCTATTAATTTTATTACAACAATTATTAATATACGAATTAATGGATT
ATCATTTGATCAAATACCCTTACTCATTTGAAGAATTGGTATTACAGCATTATTATTATTACTTTCTCTCCCAGTATTAG
CTGGTGCTATTACTATATTATTAACAGATCGAAATTTAAATACATCTTTTTTTGATCCAGCTGGAGGTGGTGATCCTATT
CTCTATCAACATTTATTT---------
>PHLAD521-11|Chilo_phragmitella|TLMF_Lep_03696|JN266142-SUPPRESSED
AACTTTATATTTTATTTTTGGAATTTGAGCTGGAATAATTGGAACATCTCTTAGACTTTTAATTCGAGCTGAATTAGGAA
CTCCAGGATCCCTAATTGGAGATGATCAAATTTATAATACTATTGTTACAGCTCATGCATTTATTATAATTTTTTTTATA
GTTATACCTATTATAATCGGTGGTTTTGGAAATTGATTAGTACCTTTAATATTAGGAGCCCCTGATATAGCTTTCCCACG
AATAAATAATATAAGATTTTGATTATTACCACCTTCATTAACCTTATTAATCTCTAGAAGAATTGTTGAAAATGGAGCTG
GAACAGGATGAACAGTGTACCCCCCACTTTCATCTAATATTGCTCATGCTGGAAGTTCAGTAGATTTAGCAATTTTTTCC
TTACATTTAGCTGGAATTTCATCAATTTTAGGTGCTATTAATTTTATTACAACAATTATTAATATACGAATTAATGGATT
ATCATTTGATCAAATACCCTTACTCATTTGAAGAATTGGTATTACAGCATTATTATTATTACTTTCTCTCCCAGTATTAG
CTGGTGCTATTACTATATTATTAACAGATCGAAATTTAAATACATCTTTTTTTGATCCAGCTGGAGGTGGTGATCCTATT
CTCTATCAACATTTATTT---------
>PHLAD536-11|Chilo_luteellus|TLMF_Lep_03711|JN266149-SUPPRESSED
AACTTTATATTTTATTTTTGGAATTTGAGCTGGAATAATCGGAACATCTTTGAGTCTTCTTATTCGTGCTGAATTAGGAA
CTCCAGGATCTCTAATTGGTAACGATCAAATTTATAATACTATTGTTACAGCTCATGCATTCATTATAATTTTTTTCATA
GTTATACCAATTATAATTGGTGGATTTGGGAACTGATTAGTACCCCTAATACTGGGGGCCCCTGATATAGCATTCCCACG
AATAAATAATATAAGATTTTGAATATTACCTCCTTCATTAACCCTATTAATTTCTAGAAGAATTGTCGAAAATGGAGCTG

GAACAGGATGAACAGTGTACCCCCCACTGTCATCTAATATCGCTCATGCAGGAAGTTCTGTAGATTTAGCAATTTTTCT
CTTCATTTAGCAGGAATTTCATCTATTTTAGGAGCTATTAATTTTATTACAACAATTATTAATATACGAATTAATGGATT
ATCTTTTGATCAAATATCTTTATTTATCTGATCTGTTGGTATTACAGCATTATTATTACTTCTTTCTTTACCAGTACTAG
CTGGAGCAATTACTATATTATTAACTGATCGAAATTTAAATACATCTTTTTTTGACCCTGCTGGTGGAGGAGATCCAATT
CTTTACCAACATTTATTT---------
>PHLAD537-11|Chilo_luteellus|TLMF_Lep_03712|JN266150-SUPPRESSED
AACTTTATATTTTATTTTTGGAATTTGAGCTGGAATAATCGGAACATCTTTGAGTCTTCTTATTCGTGCTGAATTAGGAA
CTCCAGGATCTCTAATTGGTAACGATCAAATTTATAATACTATTGTTACAGCTCATGCATTCATTATAATTTTTTTCATG
GTTATACCAATTATAATTGGTGGATTTGGAAACTGATTAGTACCCCTAATACTGGGGGCCCCTGATATAGCATTCCCACG
AATAAATAATATAAGATTTTGAATATTACCTCCTTCATTAACCCTATTAATTTCTAGAAGAATTGTCGAAAATGGAGCTG
GAACAGGATGAACAGTGTACCCCCCACTGTCATCTAATATCGCTCATGCAGGAAGTTCTGTAGATTTAGCAATTTTTCT
CTTCATTTAGCAGGAATTTCATCTATTTTAGGAGCTATTAATTTTATTACAACAATTATTAATATACGAATTAATGGATT
ATCTTTTGATCAAATATCTTTATTTATCTGATCTGTTGGTATTACAGCATTATTATTACTTCTTTCTTTACCAGTACTAG
CTGGAGCAATTACTATATTATTAACTGATCGAAATTTAAATACATCTTTTTTTGACCCTGCTGGTGGAGGAGATCCAATT
CTTTATCAACATTTATTT---------
>PHLAE246-11|Chilo_phragmitella|TLMF_Lep_04466|JN266162-SUPPRESSED
AACTTTATATTTTATTTTTGGAATTTGAGCTGGAATAATTGGAACATCTCTTAGACTTTTAATTCGAGCTGAATTAGGAA
CTCCAGGATCCCTAATTGGAGATGATCAAATTTATAATACTATTGTTACAGCTCATGCATTTATTATAATTTTTTTTATA
GTTATACCTATTATAATCGGTGGTTTTGGAAATTGATTAGTACCTTTAATATTAGGAGCCCCTGATATAGCTTTCCCACG
AATAAATAATATAAGATTTTGATTATTACCACCTTCATTAACCTTATTAATCTCTAGAAGAATTGTTGAAAATGGAGCTG
GAACAGGATGAACAGTGTACCCCCCACTTTCATCTAATATTGCTCATGCTGGAAGTTCAGTAGATTTAGCAATTTTTTCC
TTACATTTAGCTGGAATTTCATCAATTTTAGGTGCTATTAATTTTATTACAACAATTATTAATATACGAATTAATGGATT
ATCATTTGATCAAATACCCTTACTCATTTGAAGAATTGGTATTACAGCATTATTATTATTACTTTCTCTCCCAGTATTAG
CTGGTGCTATTACTATATTATTAACAGATCGAAATTTAAATACATCTTTTTTTGATCCAGCTGGAGGTGGAGAT------
----
>PHLAH598-12|Chilo_phragmitella|TLMF_Lep_08417|KM572944
AACTTTATATTTTATTTTTGGAATTTGAGCTGGAATAATTGGAACATCTCTTAGACTTTTAATTCGAGCTGAATTAGGAA
CTCCAGGATCCCTAATTGGAGATGATCAAATTTATAATACTATTGTTACAGCTCATGCATTTATTATAATTTTTTTTATA
GTTATACCTATTATAATCGGTGGTTTTGGAAATTGATTAGTACCTTTAATATTAGGAGCCCCTGATATAGCTTTCCCACG
AATAAATAATATAAGATTTTGATTATTACCACCTTCATTAACCTTATTAATCTCTAGAAGAATTGTTGAAAATGGAGCTG
GAACAGGATGAACAGTGTACCCCCCACTTTCATCTAATATTGCTCATGCTGGAAGTTCAGTAGATTTAGCAATTTTTTCC
TTACATTTAGCTGGAATTTCATCAATTTTAGGTGCTATTAATTTTATTACAACAATTATTAATATACGAATTAATGGATT
ATCATTTGATCAAATACCCTTACTCATTTGAAGAATTGGTATTACAGCATTATTATTATTACTTTCTCTCCCAGTATTAG
CTGGTGCTATTACTATATTATTAACAGATCGAAATTTAAATACATCTTTTTTTGATCCAGCTGGAGGTGGAGATCCTATT
CTCTATCAACATTTATTT---------
>PHLCC341-11|Acrapex_albicostata|BIOUG01160-E12|
TACATTATATTTTATTTTTGGAATTTGGGCAGGTATAGTAGGAACCTCTTTAAGATTATTAATTCGGGCTGAATTAGGAA
CCCCAGGATCTTTAATTGGAGATGATCAAATTTATAATACTATTGTTACAGCTCATGCTTTTATTATAATTTTCTTTATG
GTTATACCTATTATGATTGGGGGATTTGGAAATTGACTTGTCCCTTTAATATTAGGTGCCCCAGATATAGCATTTCCACG
AATAAATAATATAAGTTTTTGATTACTACCCCCCTCTTTAACCCTTCTTATTTCCAGTAGAATTGTAGAAAATGGAGCAG
GAACTGGATGAACTGTATACCCACCCCTTTCATCTAATATTGCCCATGGGGGAAGATCTGTAGATTTAGCTATTTTTTCT
TTACATTTAGCTGGGATTTCTTCTATTTTAGGGGCTATTAACTTTATTACTACAATTATTAATATACGATTAAATAGTTT
ATCCTTTGATCAAATACCTTTATTTATTTGAGCTGTAGGAATTACTGCATTTTTATTATTACTTTCTTTACCTGTATTAG
CTGGAGCTATTACAATACTATTAACAGATCGAAACTTAAATACCTCTTTTTTTGACCCTGCTGGAGGTGGAGATCCAATT
TTATATCAACATTTATTT---------
>PHLCC1027-11|Bathytricha_truncata|BIOUG01198-G09|
AACATTATATTTCATCTTTGGAATTTGAGCAGGAATAGTAGGAACTTCTTTAAGACTATTAATTCGAGCTGAGTTAGGAA
CTCCTGGATCTTTAATTGGGGATGATCAAATTTATAATACTATTGTAACAGCTCATGCCTTTATTATAATTTTTTTTATG
GTTATACCAATTATAATTGGAGGATTTGGAAATTGACTTGTACCTTTAATGTTAGGGGCACCTGATATAGCATTTCCACG
AATAAATAATATAAGTTTTTGATTACTACCCCCTTCTTTAACTCTACTTATTTCGAGAAGAATTGTAGAAAATGGAGCAG
GAACTGGGTGAACAGTATACCCCCCACTCTCATCTAATATTGCCCATAGAGGAAGATCTGTAGACTTAGCTATTTTTTCC
CTTCATTTAGCTGGAATTTCTTCTATTTTAGGGGCTATTAATTTTATCACAACTATTATTAACATACGATTAAATAATTT
ATCTTTTGATCAAATACCTTTATTTATTTGAGCAGTAGGAATTACAGCATTTTTATTATTATTATCATTACCTGTATTAG
CTGGGGCTATTACTATATTATTAACAGATCGAAATTTAAACACATCATTTTTTGATCCTGCGGGAGGAGGAGATCCAATC
TTATATCAACATTTATTT---------
>PHLCD133-12|Bathytricha_truncata|BIOUG01995-H06|
AACATTATATTTCATCTTTGGAATTTGAGCAGGAATAGTAGGAACTTCTTTAAGACTATTAATTCGAGCTGAGTTAGGAA

CTCCTGGATCTTTAATTGGGGATGATCAAATTTATAATACTATTGTAACAGCTCATGCCTTTATTATAATTTTTTTTATG
GTTATACCAATTATAATTGGAGGATTTGGAAATTGACTTGTACCTTTAATGTTAGGGGCACCTGATATAGCATTTCCACG
AATAAATAATATAAGTTTTTGATTACTACCCCCTTCTTTAACTCTACTTATTTCGAGAAGAATTGTAGAAAATGGAGCAG
GAACTGGGTGAACAGTATACCCCCCACTCTCATCTAATATTGCCCATAGAGGAAGATCTGTAGACTTAGCTATTTTTTCC
CTTCATTTAGCTGGAATTTCTTCTATTTTAGGGGCTATTAATTTTATCACAACTATTATTAACATACGATTAAATAATTT
ATCTTTTGATCAAATACCTTTATTTATTTGAGCAGTAGGAATTACAGCATTTTTATTATTATTATCATTACCTGTATTAG
CTGGGGCTATTACTATATTATTAACAGATCGAAATTTAAACACATCATTTTTTGATCCTGCGGGAGGAGGAGATCCAATC
TTATATCAACATTTATTT---------
>PHLCD356-12|Acrapex_exsanguis|BIOUG02114-B12|
AACATTATATTTTATTTTTGGAATTTGAGCTGGTATACTAGGAACTTCTTTAAGTTTATTAATCCGAGCTGAATTAGGAA
CTCCAGAATCTTTAATTGGAGATGATCAAATTTATAATACTATTGTTACTGCTCACGCTTTTATTATAATTTTCTTTATA
GTTATACCAATTATAATTGGAGGATTTGGAAATTGACTTGTCCCATTAATACTAGGAGCTCCAGATATAGCATTTCCACG
TATAAATAATATAAGATTTTGATTATTACCTCCCTCTTTAAGTTTATTAATTTCAAGAAGAATTGTAGAAAATGGAGCAG
GAACTGGATGAACAGTATATCCTCCACTCTCATCTAATATTGCTCATAGAGGAAGATCAGTAGATTTAGCTATTTTTTCT
CTTCATTTAGCTGGTATTTCATCTATTTTAGGAGCTATTAATTTTATTACAACAATTATTAATATACGATTAAATAATTT
ATCTTTTGATCAAATACCTTTATTTGTTTGAGCTGTTGGAATTACTGCATTTTTACTATTACTTTCATTACCTGTATTAG
CCGGAGCTATTACAATATTATTAACAGATCGAAATTTAAATACATCATTTTTTGATCCTGCAGGAGGAGGTGATCCAATT
TTATATCAACATTTATTT---------
>PHLCD545-12|Bathytricha_truncata_PS2|BIOUG02116-B11|
AACATTATATTTCATTTTTGGGATTTGAGCAGGGATAGTAGGAACCTCTCTAAGACTATTAATTCGAGCTGAATTAGGAA
CTCCTGGATCTCTAATTGGAGATGACCAAATCTATAATACTATTGTAACAGCTCATGCCTTTATTATAATTTTTTTTATA
GTTATACCTATTATAATCGGAGGATTTGGGAATTGACTTGTACCTTTAATATTAGGAGCGCCTGATATGGCATTCCCACG
AATAAATAATATGAGTTTTTGATTACTCCCACCCTCTTTAACTCTCCTTATTTCAAGTAGAATTGTAGAAAATGGGGCGG
GAACTGGATGAACAGTTTACCCCCCACTCTCATCTAATATTGCTCACGGGGGGAGATCTGTAGACTTAGCTATTTTTTCC
CTCCACTTAGCTGGGATCTCTTCTATTCTGGGAGCTATTAATTTTATTACAACTATTATTAATATACGATTAAACAGTTT
ATCTTTTGATCAAATACCTTTATTTATTTGAGCTGTAGGAATTACAGCATTTTTATTATTACTATCATTACCTGTATTAG
CTGGGGCTATTACAATATTATTAACAGATCGTAATTTAAATACATCATTTTTTGATCCTGCGGGGGGAGGAGATCCAATT
TTATACCAACATTTATTT---------
>PHLCD647-12|Bathytricha_truncata_PS2|BIOUG02117-C06|
AACATTATATTTCATTTTTGGGATTTGAGCAGGGATAGTAGGAACCTCTCTAAGACTATTAATTCGAGCTGAATTAGGAA
CTCCTGGATCTCTAATTGGAGATGACCAAATCTATAATACTATTGTAACAGCTCATGCCTTTATTATAATTTTTTTTATA
GTTATACCTATTATAATCGGAGGATTTGGGAATTGACTTGTACCTTTAATATTAGGAGCGCCTGATATGGCATTCCCACG
AATAAATAATATGAGTTTTTGGTTACTCCCACCCTCTTTAACTCTCCTTATTTCAAGTAGAATTGTAGAAAATGGGGCGG
GAACTGGATGAACAGTTTACCCCCCACTCTCGTCTAATATTGCTCACGGGGGGAGATCTGTAGACTTAGCTATTTTTTCC
CTCCACTTAGCTGGGATCTCTTCTATTCTGGGAGCTATTAATTTTATTACAACTATTATTAATATACGATTAAACAGTTT
ATCTTTTGATCAAATACCTTTATTTATTTGAGCTGTAGGAATTACAGCATTTTTATTATTACTATCATTACCTGTATTAG
CTGGGGCTATTACAATATTATTAACAGATCGTAATTTAAATACATCATTTTTTGATCCTGCGGGGGGAGGAGATCCAATT
TTATACCAACATTTATTT---------
>PHLCD664-12|Bathytricha_truncata_PS2|BIOUG02117-D11|
AACATTATATTTCATTTTTGGGATTTGAGCAGGGATAGTAGGAACCTCTCTAAGACTATTAATTCGAGCTGAATTAGGAA
CTCCTGGATCTCTAATTGGAGATGACCAAATCTATAATACTATTGTAACAGCTCATGCCTTTATTATAATTTTTTTTATA
GTTATACCTATTATAATCGGAGGATTTGGGAATTGACTTGTACCTTTAATATTAGGAGCGCCTGATATGGCATTCCCACG
AATAAATAATATGAGTTTTTGGTTACTCCCACCCTCTTTAACTCTCCTTATTTCAAGTAGAATTGTAGAAAATGGGGCGG
GAACTGGATGAACAGTTTACCCCCCACTCTCGTCTAATATTGCTCACGGGGGGAGATCTGTAGACTTAGCTATTTTTTCC
CTCCACTTAGCTGGGATCTCTTCTATTCTGGGAGCTATTAATTTTATTACAACTATTATTAATATACGATTAAACAGTTT
ATCTTTTGATCAAATACCTTTATTTATTTGAGCTGTAGGAATTACAGCATTTTTATTATTACTATCATTACCTGTATTAG
CTGGGGCTATTACAATATTATTAACAGATCGTAATTTAAATACATCATTTTTTGATCCTGCGGGGGGGGGAGATCCAATT
TTATACCAACATTTATTT---------
>PHLCD759-12|Bathytricha_truncata_PS2|BIOUG02118-D11|
AACATTATATTTCATTTTTGGGATTTGAGCAGGGATAGTAGGAACCTCTCTAAGACTATTAATTCGAGCTGAATTAGGAA
CTCCTGGATCTCTAATTGGAGATGACCAAATCTATAATACTATTGTAACAGCTCATGCCTTTATTATAATTTTTTTTATA
GTTATACCTATTATAATCGGAGGATTTGGGAATTGACTTGTACCTTTAATATTAGGAGCGCCTGATATGGCATTCCCACG
AATAAATAATATGAGTTTTTGGTTACTCCCACCCTCTTTAACTCTCCTTATTTCAAGTAGAATTGTAGAAAATGGGGCGG
GAACTGGATGAACAGTTTACCCCCCACTCTCGTCTAATATTGCTCACGGGGGGAGATCTGTAGACTTAGCTATTTTTTCC
CTCCACTTAGCTGGGATCTCTTCTATTCTGGGAGCTATTAATTTTATTACAACTATTATTAATATACGATTAAACAGTTT
ATCTTTTGATCAAATACCTTTATTTATTTGAGCTGTAGGAATTACAGCATTTTTATTATTACTATCATTACCTGTATTAG
CTGGGGCTATTACAATATTATTAACAGATCGTAATTTAAATACATCATTTTTTGATCCTGCGGGGGGAGGAGATCCAATT

TTATACCAACATTTATTT---------
>PHLCD761-12|Bathytricha_truncata_PS2|BIOUG02118-E01|
AACATTATATTTCATTTTTGGGATTTGAGCAGGGATAGTAGGAACCTCTCTAAGACTATTAATTCGAGCTGAATTAGGAA
CTCCTGGATCTCTAATTGGAGATGACCAAATCTATAATACTATTGTAACAGCTCATGCCTTTATTATAATTTTTTTATA
GTTATACCTATTATAATCGGAGGATTTGGGAATTGACTTGTACCTTTAATATTAGGAGCGCCTGATATGGCATTCCCACG
AATAAATAATATGAGTTTTTGATTACTCCCACCCTCTTTAACTCTCCTTATTTCAAGTAGAATTGTAGAAAATGGGGCGG
GAACTGGATGAACAGTTTACCCCCCACTCTCATCTAATATTGCTCACGGGGGGAGATCTGTAGACTTAGCTATTTTTTCC
CTCCACTTAGCTGGGATCTCTTCTATTCTGGGAGCTATTAATTTTATTACAACTATTATTAATATACGATTAAACAGTTT
ATCTTTTGATCAAATACCTTTATTTATTTGAGCTGTAGGAATTACAGCATTTTTATTATTACTATCATTACCTGTATTAG
CTGGGGCTATTACAATATTATTAACAGATCGTAATTTAAATACATCATTTTTTGATCCTGCGGGGGGAGGAGATCCAATT
TTATACCAACATTTATTT---------
>PHLCD766-12|Bathytricha_truncata_PS2|BIOUG02118-E06|
AACATTATATTTCATTTTTGGGATTTGAGCAGGGATAGTAGGAACCTCTCTAAGACTATTAATTCGAGCTGAATTAGGAA
CTCCTGGATCTCTAATTGGAGATGACCAAATCTATAATACTATTGTAACAGCTCATGCCTTTATTATAATTTTTTTATA
GTTATACCTATTATAATCGGAGGATTTGGGAATTGACTTGTACCTTTAATATTAGGAGCGCCTGATATGGCATTCCCACG
AATAAATAATATGAGTTTTTGGTTACTCCCACCCTCTTTAACTCTCCTTATTTCAAGTAGAATTGTAGAAAATGGGGCGG
GAACTGGATGAACAGTTTACCCCCCACTCTCGTCTAATATTGCTCACGGGGGGAGATCTGTAGACTTAGCTATTTTTTCC
CTCCACTTAGCTGGGATCTCTTCTATTCTGGGAGCTATTAATTTTATTACAACTATTATTAATATACGATTAAACAGTTT
ATCTTTTGATCAAATACCTTTATTTATTTGAGCTGTAGGAATTACAGCATTTTTATTATTACTATCATTACCTGTATTAG
CTGGGGCTATTACAATATTATTAACAGATCGTAATTTAAATACATCATTTTTTGATCCTGCGGGGGGAGGAGATCCAATT
TTATACCAACATTTATTT---------
>PHLCD1029-12|Bathytricha_truncata|BIOUG02121-E10|
AACATTATATTTCATCTTTGGAATTTGAGCAGGAATAGTAGGAACTTCTTTAAGACTATTAATTCGAGCTGAATTAGGAA
CTCCTGGATCCTTAATTGGAGATGATCAAATTTATAATACTATTGTAACAGCTCATGCCTTTATTATAATTTTTTTATA
GTTATACCAATTATAATCGGGGGATTTGGAAATTGACTTGTACCTTTAATGTTAGGAGCGCCTGATATGGCATTTCCACG
AATAAATAATATAAGTTTTTGATTACTACCCCCTTCTTTAACTCTACTTATTTCGAGAAGAGTTGTAGAAAATGGAGCAG
GAACTGGATGAACAGTATACCCTCCACTTTCATCTAATATTGCCCATAGAGGAAGATCTGTAGACTTAGCTATTTTTTCC
CTTCATTTAGCTGGAATTTCTTCTATTTTAGGAGCTATTAATTTTATTACAACTATTATTAACATACGATTAAATAATTT
ATCTTTTGATCAAATACCTTTATTTATTTGAGCTGTAGGAATTACAGCATTTTTATTATTATTATCATTACCTGTCTTAG
CTGGAGCTATTACTATATTATTAACAGATCGAAATTTAAATACATCATTTTTTGATCCTGCAGGAGGAGGAGATCCAATC
TTATATCAACATTTATTT---------
>PHLCD1041-12|Bathytricha_leonina|BIOUG02121-F10|
AACATTATATTTTATTTTTGGAATTTGAGCAGGAATAGTAGGAACCTCTTTAAGACTATTAATTCGAGCTGAATTAGGAA
CACCTGGATCTTTAATTGGAGATGATCAAATTTATAATACTATTGTAACAGCTCATGCTTTTATTATAATTTTTTTATA
GTTATACCAATTATAATTGGAGGGTTTGGAAATTGACTTGTACCTTTAATATTAGGAGCACCTGATATAGCATTCCCACG
AATAAATAATATAAGTTTTTGATTACTTCCACCCTCTTTAACTCTCCTTATTTCAAGTAGAATTGTAGAAAATGGGGCAG
GAACTGGATGAACAGTTTACCCACCACTCTCATCTAATATTGCTCATGGAGGAAGATCTGTAGACTTAGCTATTTTTTCT
CTCCATTTGGCGGGAATCTCTTCTATTCTAGGAGCTATTAATTTTATTACAACTATTATTAATATACGATTAAATAGCTT
ATCTTTTGATCAAATACCTTTATTTATTTGAGCCGTAGGTATTACAGCATTTTTATTATTATTATCATTACCTGTATTAG
CTGGAGCTATTACTATACTATTAACAGATCGAAATTTAAATACATCATTTTTTGATCCTGCAGGAGGAGGAGATCCAATT
TTATATCAACATTTATTT---------
>PHLCD1301-12|Bathytricha_truncata|BIOUG02107-D09|
AACATTATATTTCATCTTTGGAATTTGAGCAGGAATAGTAGGAACTTCTTTAAGACTATTAATTCGAGCTGAGTTAGGAA
CTCCTGGATCTTTAATTGGGGATGATCAAATTTATAATACTATTGTAACAGCTCATGCCTTTATTATAATTTTTTTATG
GTTATACCAATTATAATTGGAGGATTTGGAAATTGACTTGTACCTTTAATGTTAGGGGCACCTGATATAGCATTTCCACG
AATAAATAATATAAGTTTTTGATTACTACCCCCTTCTTTAACTCTACTTATTTCGAGAAGAATTGTAGAAAATGGAGCAG
GAACTGGGTGAACAGTATACCCCCCACTCTCATCTAATATTGCCCATAGAGGAAGATCTGTAGACTTAGCTATTTTTTCC
CTTCATTTAGCTGGAATTTCTTCTATTTTAGGGGCTATTAATTTTATCACAACTATTATTAACATACGATTAAATAATTT
ATCTTTTGATCAAATACCTTTATTTATTTGAGCAGTAGGAATTACAGCATTTTTATTATTATTATCATTACCTGTATTAG
CTGGGGCTATTACTATATTATTAACAGATCGAAATTTAAACACATCATTTTTTGATCCTGCGGGAGGAGGAGATCCAATC
TTATATCAACATTTATTT---------
>PHLCD1924-12|Acrapex_albicostata|BIOUG02123-G12|
TACATTATATTTTATTTTTGGAATTTGGGCAGGTATAGTAGGAACCTCTTTAAGATTATTAATTCGGGCTGAATTAGGAA
CTCCAGGATCTTTAATTGGAGATGATCAAATTTATAACACTATTGTTACAGCTCATGCTTTTATTATAATTTTCTTTATG
GTTATACCTATTATAATTGGGGGATTTGGAAATTGACTTGTCCCTTTAATACTAGGTGCCCCAGATATAGCATTTCCACG
AATAAATAATATAAGTTTTTGGTTATTACCCCCCTCTTTAACCCTCCTAACTTCCAGTGGAATCGTAGAAAATGGAGCAG
GAACTGGATGAACTGTATACCCACCCCTTTCATCTAATATTGCCCATGGGGGAAGATCTGTAGATTTAGCTATTTTTTCT

TTACATTTAGCTGGGATTTCTTCTATTTTAGGAGCTATTAACTTTATTACTACAATTATTAATATACGATTAAATAGTTT
ATCCTTTGATCAAATACCTTTATTTATTTGAGCTGTAGGAATTACTGCATTTTTATTATTACTTCTTTACCTGTATTAG
CTGGGGCTATTACAATATTATTAACAGATCGAAATTTAAATACCTCTTTTTTGACCCTGCTGGAGGTGGAGATCCAATT
TTATATCAACATTTATTT---------
>PHLCD1945-12|Bathytricha_truncata|BIOUG02124-A10|
AACATTATATTTCATCTTTGGAATTTGAGCAGGAATAGTGGGAACTTCTTTAAGACTATTAATTCGAGCTGAGTTAGGAA
CTCCTGGATCTTTAATTGGGGATGATCAAATTTATAATACTATTGTAACAGCTCATGCCTTTATTATAATTTTTTTTATG
GTTATACCAATTATAATTGGAGGATTTGGAAATTGACTTGTACCTTTAATGTTAGGGGCACCTGATATAGCATTTCCACG
AATAAATAATATAAGTTTTTGATTACTACCCCCTTCTTTAACTCTACTTATTTCGAGAAGAATTGTAGAAAATGGAGCAG
GAACTGGGTGAACAGTATACCCCCCACTCTCATCTAATATTGCCCATAGAGGAAGATCTGTAGACTTAGCTATTTTTTCC
CTTCATTTAGCTGGAATTTCTTCTATTTTAGGGGCTATTAATTTTATCACAACTATTATTAACATACGATTAAATAATTT
ATCTTTTGATCAAATACCTTTATTTATTTGAGCAGTAGGAATTACAGCATTTTTATTATTATTATCATTACCTGTATTAG
CTGGGGCTATTACTATATTATTAACAGATCGAAATTTAAACACATCATTTTTTGATCCTGCGGGAGGAGGAGATCCAATC
TTATATCAACATTTATTT---------
>PHLCD2402-12|Bathytricha_truncata|BIOUG02181-A06|
AACATTATATTTCATTTTTGGAATTTGAGCAGGAATAGTAGGAACTTCTTTAAGACTATTAATTCGAGCTGAATTAGGAA
CTCCTGGATCCTTAATTGGAGATGATCAAATTTATAATACTATTGTAACAGCTCATGCCTTTATTATAATTTTTTTTATA
GTTATACCAATTATAATCGGGGGATTTGGAAATTGACTTGTACCTTTAATGTTAGGAGCGCCTGATATGGCATTTCCACG
AATAAATAATATAAGTTTTTGATTACTACCCCCTTCTTTAACTCTACTTATTTCGAGAAGAGTTGTAGAAAATGGAGCAG
GAACTGGATGAACAGTATACCCTCCACTTTCATCTAATATTGCCCATAGAGGAAGATCTGTAGACTTAGCTATTTTTTCC
CTTCATTTAGCTGGAATTTCTTCTATTTTAGGAGCTATTAATTTTATTACAACTATTATTAACATACGATTAAATAATTT
ATCTTTTGATCAAATACCTTTATTTATTTGAGCTGTAGGAATTACAGCATTTTTATTATTATTATCATTACCTGTCTTAG
CTGGAGCTATTACTATATTATTAACAGATCGAAATTTAAATACATCATTTTTTGATCCTGCAGGAGGAGGAGATCCAATC
TTATATCAACATTTATTT---------
>PHLCD2404-12|Bathytricha_truncata|BIOUG02181-A08|
AACATTATATTTCATCTTTGGAATTTGAGCAGGAATAGTAGGAACTTCTTTAAGACTATTAATTCGAGCTGAGTTAGGAA
CTCCTGGATCTTTAATTGGGGATGATCAAATTTATAATACTATTGTAACAGCTCATGCCTTTATTATAATTTTTTTTATG
GTTATACCAATTATAATTGGAGGATTTGGAAATTGACTTGTACCTTTAATGTTAGGGGCACCTGATATAGCATTTCCACG
AATAAATAATATAAGTTTTTGATTACTACCCCCTTCTTTAACTCTACTTATTTCGAGAAGAATTGTAGAAAATGGAGCAG
GAACTGGGTGAACAGTATACCCCCCACTCTCATCTAATATTGCCCATAGAGGAAGATCTGTAGACTTAGCTATTTTTTCC
CTTCATTTAGCTGGAATTTCTTCTATTTTAGGGGCTATTAATTTTATCACAACTATTATTAACATACGATTAAATAATTT
ATCTTTTGATCAAATACCTTTATTTATTTGAGCAGTAGGAATTACAGCATTTTTATTATTATTATCATTACCTGTATTAG
CTGGGGCTATTACTATATTATTAACAGATCGAAATTTAAACACATCATTTTTTGATCCTGCGGGAGGAGGAGATCCAATC
TTATATCAACATTTATTT---------
>PHLCD2422-12|Bathytricha_truncata|BIOUG02181-C02|
AACATTATATTTCATCTTTGGAATTTGAGCAGGAATAGTAGGAACTTCTTTAAGACTATTAATTCGAGCTGAGTTAGGAA
CTCCTGGATCTTTAATTGGGGATGATCAAATTTATAATACTATTGTAACAGCTCATGCCTTTATTATAATTTTTTTTATG
GTTATACCAATTATAATTGGAGGATTTGGAAATTGACTTGTACCTTTAATGTTAGGGGCACCTGATATAGCATTTCCACG
AATAAATAATATAAGTTTTTGATTACTACCCCCTTCTTTAACTCTACTTATTTCGAGAAGAATTGTAGAAAATGGAGCAG
GAACTGGGTGAACAGTATACCCCCCACTCTCATCTAATATTGCCCATAGAGGAAGATCTGTAGACTTAGCTATTTTTTCC
CTTCATTTAGCTGGAATTTCTTCTATTTTAGGGGCTATTAATTTTATCACAACTATTATTAACATACGATTAAATAATTT
ATCTTTTGATCAAATACCTTTATTTATTTGAGCAGTAGGAATTACAGCATTTTTATTATTATTATCATTACCTGTATTAG
CTGGGGCTATTACTATATTATTAACAGATCGAAATTTAAACACATCATTTTTTGATCCTGCGGGAGGAGGAGATCCAATC
TTATATCAACATTTATTT---------
>PHLCD2598-12|Bathytricha_truncata|BIOUG02183-F07|
AACATTATATTTCATCTTTGGAATTTGAGCAGGAATAGTAGGAACTTCTTTAAGACTATTAATTCGAGCTGAATTAGGAA
CTCCTGGATCCTTAATTGGAGATGATCAAATTTATAATACTATTGTAACAGCTCATGCCTTTATTATAATTTTTTTTATA
GTTATACCAATTATAATCGGGGGATTTGGAAATTGACTTGTACCTTTAATGTTAGGAGCGCCTGATATGGCATTTCCACG
AATAAATAATATAAGTTTTTGATTACTACCCCCTTCTTTAACTCTACTTATTTCGAGAAGAGTTGTAGAAAATGGAGCAG
GAACTGGATGAACAGTATACCCTCCACTTTCATCTAATATTGCCCATAGAGGAAGATCTGTAGACTTAGCTATTTTTTCC
CTTCATTTAGCTGGAATTTCTTCTATTTTAGGAGCTATTAATTTTATTACAACTATTATTAACATACGATTAAATAATTT
ATCTTTTGATCAAATACCTTTATTTATTTGAGCTGTAGGAATTACAGCATTTTTATTATTATTATCATTACCTGTCTTAG
CTGGAGCTATTACTATATTATTAACAGATCGAAATTTAAATACATCATTTTTTGATCCTGCAGGAGGAGGAGATCCAATC
TTATATCAACATTTATTT---------
>PHLCD2752-12|Bathytricha_truncata|BIOUG02185-F07|
AACATTATATTTCATCTTTGGAATTTGAGCAGGAATAGTAGGAACTTCTTTAAGACTATTAATTCGAGCTGAGTTAGGAA
CTCCTGGATCTTTAATTGGGGATGATCAAATTTATAATACTATTGTAACAGCTCATGCCTTTATTATAATTTTTTTTATG

GTTATACCAATTATAATTGGAGGATTTGGAAATTGACTTGTACCTTTAATGTTAGGGGCACCTGATATAGCATTTCCACG
AATAAATAATATAAGTTTTTGATTACTACCCCCTTCTTTAACTCTACTTATTTCGAGAAGAATTGTAGAAAATGGAGCAG
GAACTGGGTGAACAGTATACCCCCCACTCTCATCTAATATTGCCCATAGAGGAAGATCTGTAGACTTAGCTATTTTTTCC
CTTCATTTAGCTGGAATTTCTTCTATTTTAGGGGCTATTAATTTTATCACAACTATTATTAACATACGATTAAATAATTT
ATCTTTTGATCAAATACCTTTATTTATTTGAGCAGTAGGAATTACAGCATTTTTATTATTATTATCATTACCTGTATTAG
CTGGGGCTATTACTATATTATTAACAGATCGAAATTTAAACACATCATTTTTTGATCCTGCGGGAGGAGGAGATCCAATC
TTATATCAACATTTATTT---------
>PHLCD2881-12|Bathytricha_truncata|BIOUG02187-A06|
AACATTATATTTCATCTTTGGAATTTGAGCAGGAATAGTAGGAACTTCTTTAAGACTATTAATTCGAGCTGAATTAGGAA
CTCCTGGATCCTTAATTGGAGATGATCAAATTTATAATACTATTGTAACAGCTCATGCCTTTATTATAATTTTTTTTATA
GTTATACCAATTATAATCGGGGGATTTGGAAATTGACTTGTACCTTTAATGTTAGGAGCGCCTGATATAGCATTTCCACG
AATAAATAATATAAGTTTTTGATTACTACCCCCTTCTTTAACTCTACTTATTTCGAGAAGAGTTGTAGAAAATGGAGCAG
GAACTGGATGAACAGTATACCCTCCACTTTCATCTAATATTGCCCATAGAGGAAGATCTGTAGACTTAGCTATTTTTTCC
CTTCATTTAGCTGGAATTTCTTCTATTTTAGGAGCTATTAATTTTATTACAACTATTATTAACATACGATTAAATAATTT
ATCTTTTGATCAAATACCTTTATTTATTTGAGCTGTAGGAATTACAGCATTTTTATTATTATTATCATTACCTGTCTTAG
CTGGAGCTATTACTATATTATTAACAGATCGAAATTTAAATACATCATTTTTTGATCCTGCAGGAGGAGGAGATCCAATC
TTATATCAACATTTATTT---------
>PHLCD2882-12|Bathytricha_truncata|BIOUG02187-A07|
AACATTATATTTCATCTTTGGAATTTGAGCAGGAATAGTAGGAACTTCTTTAAGACTATTAATTCGAGCTGAATTAGGAA
CTCCTGGATCCTTAATTGGAGATGATCAAATTTATAATACTATTGTAACAGCTCATGCCTTTATTATAATTTTTTTTATA
GTTATACCAATTATAATCGGGGGATTTGGAAATTGACTTGTACCTTTAATGTTAGGAGCGCCTGATATGGCATTTCCACG
AATAAATAATATAAGTTTTTGATTACTACCCCCTTCTTTAACTCTACTTATTTCGAGAAGAGTTGTAGAAAATGGAGCAG
GAACTGGATGAACAGTATACCCTCCACTTTCATCTAATATTGCCCATAGAGGAAGATCTGTAGACTTAGCTATTTTTTCC
CTTCATTTAGCTGGAATTTCTTCTATTTTAGGAGCTATTAATTTTATTACAACTATTATTAACATACGATTAAATAATTT
ATCTTTTGATCAAATACCTTTATTTATTTGAGCTGTAGGAATTACAGCATTTTTATTATTATTATCATTACCTGTCTTAG
CTGGAGCTATTACTATATTATTAACAGATCGAAATTTAAATACATCATTTTTTGATCCTGCAGGAGGAGGAGATCCAATC
TTATATCAACATTTATTT---------
>PHLCD2915-12|Bathytricha_truncata|BIOUG02187-D04|
AACATTATATTTCATCTTTGGAATTTGAGCAGGAATAGTAGGAACTTCTTTAAGACTATTAATTCGAGCTGAGTTAGGAA
CTCCTGGATCTTTAATTGGGGATGATCAAATTTATAATACTATTGTAACAGCTCATGCCTTTATTATAATTTTTTTTATG
GTTATACCAATTATAATTGGAGGATTTGGAAATTGACTTGTACCTTTAATGTTAGGGGCACCTGATATAGCATTTCCACG
AATAAATAATATAAGTTTTTGATTACTACCCCCTTCTTTAACTCTACTTATTTCGAGAAGAATTGTAGAAAATGGAGCAG
GAACTGGGTGAACAGTATACCCCCCACTCTCATCTAATATTGCCCATAGAGGAAGATCTGTAGACTTAGCTATTTTTTCC
CTTCATTTAGCTGGAATTTCTTCTATTTTAGGGGCTATTAATTTTATCACAACTATTATTAACATACGATTAAATAATTT
ATCTTTTGATCAAATACCTTTATTTATTTGAGCAGTAGGAATTACAGCATTTTTATTATTATTATCATTACCTGTATTAG
CTGGGGCTATTACTATATTATTAACAGATCGAAATTTAAACACATCATTTTTTGATCCTGCGGGAGGAGGAGATCCAATC
TTATATCAACATTTATTT---------
>PHSAU245-12|Bathytricha_truncata_PS2|BIOUG02183-G04|
AACATTATATTTCATTTTTGGGATTTGAGCAGGGATAGTAGGAACCTCTCTAAGACTATTAATTCGAGCTGAATTAGGAA
CTCCTGGATCTCTAATTGGAGATGACCAAATCTATAATACTATTGTAACAGCTCATGCCTTTATTATAATTTTTTTTATA
GTTATACCTATTATAATCGGAGGATTTGGGAATTGACTTGTACCTTTAATATTAGGAGCGCCTGATATGGCATTCCCACG
AATAAATAATATGAGTTTTTGGTTACTCCCACCCTCTTTAACTCTCCTTATTTCAAGTAGAATTGTAGAAAATGGGGCGG
GAACTGGATGAACAGTTTACCCCCCACTCTCGTCTAATATTGCTCACGGGGGGAGATCTGTAGACTTAGCTATTTTTTCC
CTCCACTTAGCTGGGATCTCTTCTATTCTGGGAGCTATTAATTTTATTACAACTATTATTAATATACGATTAAACAGTTT
ATCTTTTGATCAAATACCTTTATTTATTTGAGCTGTAGGAATTACAGCATTTTTATTATTACTATCATTACCTGTATTAG
CTGGGGCTATTACAATATTATTAACAGATCGTAATTTAAATACATCATTTTTTGATCCTGCGGGGGGAGGAGATCCAATT
TTATACCAACATTTATTT---------
>PHSAU264-12|Bathytricha|BIOUG02184-A12|
AACATTATATTTCATTTTTGGGATTTGAGCAGGAATAGTGGGAACCTCTCTAAGACTATTAATCCGAGCTGAGTTAGGGA
CTCCCGGGTCTCTAATTGGAGATGACCAAATCTATAACACTATTGTAACAGCCCATGCCTTTATTATAATTTTTTTTATA
GTTATACCTATTATAATCGGAGGATTTGGGAATTGACTTGTACCTTTAATATTAGGGGCACCTGATATGGCGTTCCCGCG
AATAAATAATATGAGTTTTTGGTTACTCCCACCCTCTTTAACTCTCCTCATTTCAAGTAGAATTGTAGAAAATGGGGCGG
GAACTGGGTGAACAGTTTACCCCCCACTCTCGTCTAATATTGCTCATGGGGGAAGATCTGTAGACTTAGCTATTTTTTCC
CTTCACTTAGCTGGAATCTCTTCTATTCTGGGAGCTATTAATTTTATTACAACTATTATTAATATACGATTAAACAGTTT
ATCTTTTGATCAAATACCTTTATTTATTTGAGCTGTAGGAATTACAGCATTTTTATTATTACTGTCATTACCTGTATTAG
CTGGGGCTATTACAATATTATTAACAGATCGTAATTTAAATACATCATTTTTTGATCCTGCGGGGGGAGGAGATCCAATT
TTATATCAACATTTATTT---------

>PHSAU365-12|Bathytricha_truncata_PS2|BIOUG02213-D01|
AACATTATATTTCATTTTTGGGATTTGAGCAGGGATAGTAGGAACCTCTCTAAGACTATTAATTCGAGCTGAATTAGGAA
CTCCTGGATCTCTAATTGGAGATGACCAAATCTATAATACTATTGTAACAGCTCATGCCTTTATTATAATTTTTTTTATA
GTTATACCTATTATAATCGGAGGATTTGGGAATTGACTTGTACCTTTAATATTAGGAGCGCCTGATATGGCATTCCCACG
AATAAATAATATGAGTTTTTGGTTACTCCCACCCTCTTTAACTCTCCTTATTTCAAGTAGAATTGTAGAAAATGGGGCGG
GAACTGGATGAACAGTTTACCCCCCACTCTCGTCTAATATTGCTCACGGGGGGAGATCTGTAGACTTAGCTATTTTTTCC
CTCCACTTAGCTGGGATCTCTTCTATTCTGGGAGCTATTAATTTTATTACAACTATTATTAATATACGATTAAACAGTTT
ATCTTTTGATCAAATACCTTTATTTATTTGAGCTGTAGGAATTACAGCATTTTTATTATTACTATCATTACCTGTATTAG
CTGGGGCTATTACAATATTATTAACAGATCGTAATTTAAATACATCATTTTTTGATCCTGCGGGGGGAGGAGATCCAATT
TTATACCAACATTTATTT---------
>PHSAU706-12|Bathytricha_truncata_PS2|BIOUG02216-H09|
AACATTATATTTCATTTTTGGGATTTGAGCAGGGATAGTAGGAACCTCTCTAAGACTATTAATTCGAGCTGAATTAGGAA
CTCCTGGATCTCTAATTGGAGATGACCAAATCTATAATACTATTGTAACAGCTCATGCCTTTATTATAATTTTTTTTATA
GTTATACCTATTATAATCGGAGGATTTGGGAATTGACTTGTACCTTTAATATTAGGAGCGCCTGATATGGCATTCCCACG
AATAAATAATATGAGTTTTTGATTACTCCCACCCTCTTTAACTCTCCTTATTTCAAGTAGAATTGTAGAAAATGGGGCGG
GAACTGGATGAACAGTTTACCCCCCACTCTCATCTAATATTGCTCACGGGGGGAGATCTGTAGACTTAGCTATTTTTTCC
CTCCACTTAGCTGGGATCTCTTCTATTCTGGGAGCTATTAATTTTATTACAACTATTATTAATATACGATTAAACAGTTT
ATCTTTTGATCAAATACCTTTATTTATTTGAGCTGTAGGAATTACAGCATTTTTATTATTACTATCATTACCTGTATTAG
CTGGGGCTATTACAATATTATTAACAGATCGTAATTTAAATACATCATTTTTTGATCCTGCGGGGGGAGGAGATCCAATT
TTATACCAACATTTATTT---------
>PHSAU719-12|Bathytricha_truncata_PS2|BIOUG02217-A11|
AACATTATATTTCATTTTTGGGATTTGAGCAGGGATAGTAGGAACCTCTCTAAGACTATTAATTCGAGCTGAATTAGGAA
CTCCTGGATCTCTAATTGGAGATGACCAAATCTATAATACTATTGTAACAGCTCATGCCTTTATTATAATTTTTTTTATA
GTTATACCTATTATAATCGGAGGATTTGGGAATTGACTTGTACCTTTAATATTAGGAGCGCCTGATATGGCATTCCCACG
AATAAATAATATGAGTTTTTGATTACTCCCACCCTCTTTAACTCTCCTTATTTCAAGTAGAATTGTAGAAAATGGGGCGG
GAACTGGATGAACAGTTTACCCCCCACTCTCATCTAATATTGCTCACGGGGGGAGATCTGTAGACTTAGCTATTTTTTCC
CTCCACTTAGCTGGGATCTCTTCTATTCTGGGAGCTATTAATTTTATTACAACTATTATTAATATACGATTAAACAGTTT
ATCTTTTGATCAAATACCTTTATTTATTTGAGCTGTAGGAATTACAGCATTTTTATTATTACTATCATTACCTGTATTAG
CTGGGGCTATTACAATATTATTAACAGATCGTAATTTAAATACATCATTTTTTGATCCTGCGGGGGGAGGAGATCCAATT
TTATACCAACATTTATTT---------
>PHSAU720-12|Bathytricha_truncata_PS2|BIOUG02217-A12|
AACATTATATTTCATTTTTGGGATTTGAGCAGGGATAGTAGGAACCTCTCTAAGACTATTAATTCGAGCTGAATTAGGAA
CTCCTGGATCTCTAATTGGAGATGACCAAATCTATAATACTATTGTAACAGCTCATGCCTTTATTATAATTTTTTTTATA
GTTATACCTATTATAATCGGAGGATTTGGGAATTGACTTGTACCTTTAATATTAGGAGCGCCTGATATGGCATTCCCACG
AATAAATAATATGAGTTTTTGGTTACTCCCACCCTCTTTAACTCTCCTTATTTCAAGTAGAATTGTAGAAAATGGGGCGG
GAACTGGATGAACAGTTTACCCCCCACTCTCGTCTAATATTGCTCACGGGGGGAGATCTGTAGACTTAGCTATTTTTTCC
CTCCACTTAGCTGGGATCTCTTCTATTCTGGGAGCTATTAATTTTATTACAACTATTATTAATATACGATTAAACAGTTT
ATCTTTTGATCAAATACCTTTATTTATTTGAGCTGTAGGAATTACAGCATTTTTATTATTACTATCATTACCTGTATTAG
CTGGGGCTATTACAATATTATTAACAGATCGTAATTTAAATACATCATTTTTTGATCCTGCGGGGGGAGGAGATCCAATT
TTATACCAACATTTATTT---------
>PHSAU733-12|Bathytricha_truncata_PS2|BIOUG02217-C01|
AACATTATATTTCATTTTTGGGATTTGAGCAGGGATAGTAGGAACCTCTCTAAGACTATTAATTCGAGCTGAATTAGGAA
CTCCTGGATCTCTAATTGGAGATGACCAAATCTATAATACTATTGTAACAGCTCATGCCTTTATTATAATTTTTTTTATA
GTTATACCTATTATAATCGGAGGATTTGGGAATTGACTTGTACCTTTAATATTAGGAGCGCCTGATATGGCATTCCCACG
AATAAATAATATGAGTTTTTGGTTACTCCCACCCTCTTTAACTCTCCTTATTTCAAGTAGAATTGTAGAAAATGGGGCGG
GAACTGGATGAACAGTTTACCCCCCACTCTCGTCTAATATTGCTCACGGGGGGAGATCTGTAGACTTAGCTATTTTTTCC
CTCCACTTAGCTGGGATCTCTTCTATTCTGGGAGCTATTAATTTTATTACAACTATTATTAATATACGATTAAACAGTTT
ATCTTTTGATCAAATACCTTTATTTATTTGAGCTGTAGGAATTACAGCATTTTTATTATTACTATCATTACCTGTATTAG
CTGGGGCTATTACAATATTATTAACAGATCGTAATTTAAATACATCATTTTTTGATCCTGCGGGGGGAGGAGATCCAATT
TTATACCAACATTTATTT---------
>PHSAU932-12|Bathytricha_truncata_PS2|BIOUG02219-C10|
AACATTATATTTCATTTTTGGGATTTGAGCAGGGATAGTAGGAACCTCTCTAAGACTATTAATTCGAGCTGAATTAGGAA
CTCCTGGATCTCTAATTGGAGATGACCAAATCTATAATACTATTGTAACAGCTCATGCCTTTATTATAATTTTTTTTATA
GTTATACCTATTATAATCGGAGGATTTGGGAATTGACTTGTACCTTTAATATTAGGAGCGCCTGATATGGCATTCCCACG
AATAAATAATATGAGTTTTTGGTTACTCCCACCCTCTTTAACTCTCCTTATTTCAAGTAGAATTGTAGAAAATGGGGCGG
GAACTGGATGAACAGTTTACCCCCCACTCTCGTCTAATATTGCTCACGGGGGGAGATCTGTAGACTTAGCTATTTTTTCC
CTCCACTTAGCTGGGATCTCTTCTATTCTGGGAGCTATTAATTTTATTACAACTATTATTAATATACGATTAAACAGTTT

ATCTTTTGATCAAATACCTTTATTTATTTGAGCTGTAGGAATTACAGCATTTTTATTATTACTATCATTACCTGTATTAG
CTGGGGCTATTACAATATTATTAACAGATCGTAATTTAAATACATCATTTTTTGATCCTGCGGGGGGAGGAGATCCAATT
TTATACCAACATTTATTT---------
>PHSAU1640-12|Bathytricha_truncata_PS2|BIOUG02249-A06|
AACATTATATTTCATTTTTGGGATTTGAGCAGGGATAGTAGGAACCTCTCTAAGACTATTAATTCGAGCTGAATTAGGAA
CTCCTGGATCTCTAATTGGAGATGACCAAATCTATAATACTATTGTAACAGCTCATGCCTTTATTATAATTTTTTTTATA
GTTATACCTATTATAATCGGAGGATTTGGGAATTGACTTGTACCTTTAATATTAGGAGCGCCTGATATGGCATTCCCACG
AATAAATAATATGAGTTTTTGGTTACTCCCACCCTCTTTAACTCTCCTTATTTCAAGTAGAATTGTAGAAAATGGGGCGG
GAACTGGATGAACAGTTTACCCCCCACTCTCGTCTAATATTGCTCACGGGGGGAGATCTGTAGACTTAGCTATTTTTTCC
CTCCACTTAGCTGGGATCTCTTCTATTCTGGGAGCTATTAATTTTATTACAACTATTATTAATATACGATTAAACAGTTT
ATCTTTTGATCAAATACCTTTATTTATTTGAGCTGTAGGAATTACAGCATTTTTATTATTACTATCATTACCTGTATTAG
CTGGGGCTATTACAATATTATTAACAGATCGTAATTTAAATACATCATTTTTTGATCCTGCGGGGGGAGGAGATCCAATT
TTATACCAACATTTATTT---------
>PHSAU1641-12|Bathytricha_truncata_PS2|BIOUG02249-A07|
AACATTATATTTCATTTTTGGGATTTGAGCAGGGATAGTAGGAACCTCTCTAAGACTATTAATTCGAGCTGAATTAGGAA
CTCCTGGATCTCTAATTGGAGATGACCAAATCTATAATACTATTGTAACAGCTCATGCCTTTATTATAATTTTTTTTATA
GTTATACCTATTATAATCGGAGGATTTGGGAATTGACTTGTACCTTTAATATTAGGAGCGCCTGATATGGCATTCCCACG
AATAAATAATATGAGTTTTTGATTACTCCCACCCTCTTTAACTCTCCTTATTTCAAGTAGAATTGTAGAAAATGGGGCGG
GAACTGGATGAACAGTTTACCCCCCACTCTCATCTAATATTGCTCACGGGGGGAGATCTGTAGACTTAGCTATTTTTTCC
CTCCACTTAGCTGGGATCTCTTCTATTCTGGGAGCTATTAATTTTATTACAACTATTATTAATATACGATTAAACAGTTT
ATCTTTTGATCAAATACCTTTATTTATTTGAGCTGTAGGAATTACAGCATTTTTATTATTACTATCATTACCTGTATTAG
CTGGGGCTATTACAATATTATTAACAGATCGTAATTTAAATACATCATTTTTTGATCCTGCGGGGGGAGGAGATCCAATT
TTATACCAACATTTATTT---------
>PHSAU1826-12|Bathytricha_truncata_PS2|BIOUG02251-B11|
AACATTATATTTCATTTTTGGGATTTGAGCAGGGATAGTAGGAACCTCTCTAAGACTATTAATTCGAGCTGAATTAGGAA
CTCCTGGATCTCTAATTGGAGATGACCAAATCTATAATACTATTGTAACAGCTCATGCCTTTATTATAATTTTTTTTATA
GTTATACCTATTATAATCGGAGGATTTGGGAATTGACTTGTACCTTTAATATTAGGAGCGCCTGATATGGCATTCCCACG
AATAAATAATATGAGTTTTTGGTTACTCCCACCCTCTTTAACTCTCCTTATTTCAAGTAGAATTGTAGAAAATGGGGCGG
GAACTGGATGAACAGTTTACCCCCCACTCTCGTCTAATATTGCTCACGGGGGGAGATCTGTAGACTTAGCTATTTTTTCC
CTCCACTTAGCTGGGATCTCTTCTATTCTGGGAGCTATTAATTTTATTACAACTATTATTAATATACGATTAAACAGTTT
ATCTTTTGATCAAATACCTTTATTTATTTGAGCTGTAGGAATTACAGCATTTTTATTATTACTATCATTACCTGTATTAG
CTGGGGCTATTACAATATTATTAACAGATCGTAATTTAAATACATCATTTTTTGATCCTGCGGGGGGAGGAGATCCAATT
TTATACCAACATTTATTT---------
>PHSAU1836-12|Bathytricha_truncata_PS2|BIOUG02251-C09|
AACATTATATTTCATTTTTGGGATTTGAGCAGGGATAGTAGGAACCTCTCTAAGACTATTAATTCGAGCTGAATTAGGAA
CTCCTGGATCTCTAATTGAAGATGACCAAATCTATAATACTATTGTAACAGCTCATGCCTTTATTATAATTTTTTTTATA
GTTATACCTATTATAATCGGAGGATTTGGGAATTGACTTGTACCTTTAATATTAGGAGCGCCTGATATGGCATTCCCACG
AATAAATAATATGAGTTTTTGGTTACTCCCACCCTCTTTAACTCTCCTTATTTCAAGTAGAATTGTAGAAAATGGGGCGG
GAACTGGATGAACAGTTTACCCCCCACTCTCGTCTAATATTGCTCACGGGGGGAGATCTGTAGACTTAGCTATTTTTTCC
CTCCACTTAGCTGGGATCTCTTCTATTCTGGGAGCTATTAATTTTATTACAACTATTATTAATATACGATTAAACAGTTT
ATCTTTTGATCAAATACCTTTATTTATTTGAGCTGTAGGAATTACAGCATTTTTATTATTACTATCATTACCTGTATTAG
CTGGGGCTATTACAATATTATTAACAGATCGTAATTTAAATACATCATTTTTTGATCCTGCGGGGGGAGGAGATCCAATT
TTATACCAACATTTATTT---------
>PHSAU1886-12|Bathytricha_truncata_PS2|BIOUG02251-G11|
AACATTATATTTCATTTTTGGGATTTGAGCAGGGATAGTAGGAACCTCTCTAAGACTATTAATTCGAGCTGAATTAGGAA
CTCCTGGATCTCTAATTGGAGATGACCAAATCTATAATACTATTGTAACAGCTCATGCCTTTATTATAATTTTTTTTATA
GTTATACCTATTATAATCGGAGGATTTGGGAATTGACTTGTACCTTTAATATTAGGAGCGCCTGATATGGCATTCCCACG
AATAAATAATATGAGTTTTTGGTTACTCCCACCCTCTTTAACTCTCCTTATTTCAAGTAGAATTGTAGAAAATGGGGCGG
GAACTGGATGAACAGTTTACCCCCCACTCTCGTCTAATATTGCTCACGGGGGGAGATCTGTAGACTTAGCTATTTTTTCC
CTCCACTTAGCTGGGATCTCTTCTATTCTGGGAGCTATTAATTTTATTACAACTATTATTAATATACGATTAAACAGTTT
ATCTTTTGATCAAATACCTTTATTTATTTGAGCTGTAGGAATTACAGCATTTTTATTATTACTATCATTACCTGTATTAG
CTGGGGCTATTACAATATTATTAACAGATCGTAATTTAAATACATCATTTTTTGATCCTGCGGGGGGAGGAGATCCAATT
TTATACCAACATTTATTT---------
>PHSAU1900-12|Bathytricha_truncata_PS2|BIOUG02252-A02|
AACATTATATTTCATTTTTGGGATTTGAGCAGGGATAGTAGGAACCTCTCTAAGACTATTAATTCGAGCTGAATTAGGAA
CTCCTGGATCTCTAATTGGAGATGACCAAATCTATAATACTATTGTAACAGCTCATGCCTTTATTATAATTTTTTTTATA
GTTATACCTATTATAATCGGAGGATTTGGGAATTGACTTGTACCTTTAATATTAGGAGCGCCTGATATGGCATTCCCACG

AATAAATAATATGAGTTTTTGGTTACTCCCACCCTCTTTAACTCTCCTTATTTCAAGTAGAATTGTAGAAAATGGGGCGG
GAACTGGATGAACAGTTTACCCCCCACTCTCGTCTAATATTGCTCACGGGGGGAGATCTGTAGACTTAGCTATTTTTTCC
CTCCACTTAGCTGGGATCTCTTCTATTCTGGGAGCTATTAATTTTATTACAACTATTATTAATATACGATTAAACAGTTT
ATCTTTTGATCAAATACCTTTATTTATTTGAGCTGTAGGAATTACAGCATTTTTATTATTACTATCATTACCTGTATTAG
CTGGGGCTATTACAATATTATTAACAGATCGTAATTTAAATACATCATTTTTTGATCCTGCGGGGGGAGGAGATCCAATT
TTATACCAACATTTATTT---------
>PHSAU1903-12|Bathytricha_truncata_PS2|BIOUG02252-A05|
AACATTATATTTCATTTTTGGGATTTGAGCAGGGATAGTAGGAACCTCTCTAAGACTATTAATTCGAGCTGAATTAGGAA
CTCCTGGATCTCTAATTGGAGATGACCAAATCTATAATACTATTGTAACAGCTCATGCCTTTATTATAATTTTTTTTATA
GTTATACCTATTATAATCGGAGGATTTGGGAATTGACTTGTACCTTTAATATTAGGAGCGCCTGATATGGCATTCCCACG
AATAAATAATATGAGTTTTTGGTTACTCCCACCCTCTTTAACTCTCCTTATTTCAAGTAGAATTGTAGAAAATGGGGCGG
GAACTGGATGAACAGTTTACCCCCCACTCTCGTCTAATATTGCTCACGGGGGGAGATCTGTAGACTTAGCTATTTTTTCC
CTCCACTTAGCTGGGATCTCTTCTATTCTGGGAGCTATTAATTTTATTACAACTATTATTAATATACGATTAAACAGTTT
ATCTTTTGATCAAATACCTTTATTTATTTGAGCTGTAGGAATTACAGCATTTTTATTATTACTATCATTACCTGTATTAG
CTGGGGCTATTACAATATTATTAACAGATCGTAATTTAAATACATCATTTTTTGATCCTGCGGGGGGAGGAGATCCAATT
TTATACCAACATTTATTT---------
>PMANL3279-14|Eldana_saccharina|USNM_ENT_00980943|
AACATTATACTTTATTTTTGGTATTTGATCAGGAATAGTAGGTACTTCTCTTAGATTACTAATTCGAGCTGAATTAGGAA
ACCCAGGATCTTTAATTGGAGATGACCAAATTTATAATACTATTGTTACAGGCCATGCTTTTATTATAATTTTTTTTATA
GTTATACCTATTATAATTGGAGGATTTGGTAATTGACTTGTACCTCTAATACTCGGGGCTCCCGATATAGCTTTCCCCCG
TATAAATAATATAAGTTTTTGGCTATTACCCCCTTCTCTTTCTCTTCTAATTTTTAGAAGAATTGTTGAAAATGGAGCAG
GAACAGGATGAACAGTTTACCCCCCATTATCTTCAAATATCGCTCATAGAGGAAGTTCTGTAGATTTAGCTATTTTTTCT
CTTCATTTAGCTGGAATCTCATCAATTTTAGGAGCTATTAATTTTATTACAACAGTAATTAATATAAAATTAAATGGTTT
ATCATTTGATCAAATACCTTTATTTGTATGAGCTGTAAGAATTACAGCTCTTCTTTTACTTTTATCTTTACCAGTTTTAG
CAGGTGCAATTACTATACTTTTAACAGATCGTAATCTAAATACATCTTTTTTTGACCCTGCCGGAGGAGGAGACCCTATT
CTTTACCAACATTTATTT---------
>PMANL4009-15|Eldana_saccharina|USNM_ENT_01067633|
AACATTATACTTTATTTTTGGTATTTGATCAGGAATAGTAGGTACTTCTCTTAGATTACTAATTCGAGCTGAATTAGGAA
ACCCAGGATCTTTAATTGGAGATGACCAAATTTATAATACTATTGTTACAGGCCATGCTTTTATTATAATTTTTTTTATA
GTTATACCTATTATAATTGGAGGATTTGGTAATTGACTTGTACCTCTAATACTCGGAGCTCCCGATATAGCTTTCCCCCG
TATAAATAATATAAGTTTTTGGCTATTACCCCCTTCTCTTTCTCTTCTAATTTTTAGAAGAATTGTTGAAAATGGAGCAG
GAACAGGATGAACAGTTTACCCCCCATTATCTTCAAATATCGCTCATAGAGGAAGTTCTGTAGATTTAGCTATTTTTTCT
CTTCATTTAGCTGGAATCTCATCAATTTTAGGGGCTATTAATTTTATTACAACAGTAATTAATATAAAATTAAATGGTTT
ATCATTTGATCAAATACCTTTATTTGTATGAGCTGTAAGAATTACAGCTCTTCTTTTACTTTTATCTTTACCAGTTTTAG
CAGGTGCAATTACTATACTTTTAACAGATCGTAATCTAAATACATCTTTTTTTGACCCTGCCGGAGGAGGAGACCCTATT
CTTTACCAACATTTATTT---------
>PMANL4010-15|Eldana_saccharina|USNM_ENT_01067634|
AACATTATACTTTATTTTTGGTATTTGATCAGGAATAGTAGGTACTTCTCTTAGATTACTAATTCGAGCTGAATTAGGAA
ACCCAGGATCTTTAATTGGAGATGACCAAATTTATAATACTATTGTTACAGGCCATGCTTTTATTATAATTTTTTTTATA
GTTATACCTATTATAATTGGAGGATTTGGTAATTGACTTGTACCTCTAATACTCGGGGCTCCCGATATAGCTTTCCCCCG
TATAAATAATATAAGTTTTTGGCTATTACCCCCTTCTCTTTCTCTTCTAATTTTTAGAAGAATTGTTGAAAATGGAGCAG
GAACAGGATGAACAGTTTACCCCCCATTATCTTCAAATATCGCTCATAGAGGAAGTTCTGTAGATTTAGCTATTTTTTCT
CTTCATTTAGCTGGAATCTCATCAATTTTAGGAGCTATTAATTTTATTACAACAGTAATTAATATAAAATTAAATGGTTT
ATCATTTGATCAAATACCTTTATTTGTATGAGCTGTAAGAATTACAGCTCTTCTTTTACTTTTATCTTTACCAGTTTTAG
CAGGTGCAATTACTATACTTTTAACAGATCGTAATCTAAATACATCTTTTTTTGACCCTGCCGGAGGAGGAGACCCTATT
CTTTACCAACATTTATTT---------
>SCHIN001-18|Chilo_auricilius|W17-CaGu1|
AACTTTATACTTTATTTTTGGAATTTGAAGTGGAATAATTGGGACATCTCTAAGACTTTTAATTCGTGCTGAATTAGGAA
CCCCAGGATCATTAATTGGAGATGATCAAATTTATAATACTATTGTTACAGCTCATGCATTTATTATAATTTTTTTTATA
GTTATACCAATTATAATTGGAGGCTTTGGTAACTGATTAGTACCATTAATGCTAGGAGCTCCTGATATAGCCTTCCCTCG
AATAAATAATATAAGATTTTGATTATTACCCCCATCATTAACATTATTAATTTCTAGAAGAATTGTAGAAAATGGAGCTG
GAACAGGATGAACGGTATACCCCCCCCTTTCATCAAATATTGCCCATGGTGGAAGTTCTGTAGATTTAGCCATTTTTTCT
CTTCATTTAGCTGGTATTTCCTCAATTTTAGGAGCTATTAATTTTATTACAACAATTATTAATATACGAATTAATAAATT
ATCATTTGATCAACTACCATTATTTGTTTGATCTGTTGGTATTACAGCCTTATTATTATTACTTTCATTACCAGTACTAG
CTGGAGCTATTACTATACTTTTAACTGATCGAAACTTAAATACATCTTTTTTTGACCCTGCTGGGGGAGGAGACCCTATT
CTTTACCAACATTTATTTTGATTTTTT
>SCHIN002-18|Chilo_auricilius|W17-CaGu2|

AACTTTATACTTTATTTTTGGAATTTGAAGTGGAATAATTGGGACATCTCTAAGACTTTTAATTCGTGCTGAATTAGGAA
CCCCGGGATCATTAATTGGAGATGATCAAATTTATAATACTATTGTTACAGCTCATGCATTTATTATAATTTTTTTTATA
GTTATACCAATTATAATTGGAGGCTTTGGTAACTGATTAGTACCATTAATGCTAGGAGCTCCTGATATAGCCTTCCCTCG
AATAAATAATATAAGATTTCGATTATTACCCCCATCATTAACATTATTAATTTCTAGAAGAATTGTAGAAAATGGAGCTG
GAACAGGATGAACGGTATACCCCCCCCTTTCATCAAATATTGCCCATGGTGGAAGTTCTGTAGATTTAGCCATTTTTTCT
CTTCATTTAGCTGGTATTTCCTCAATTTTAGGAGCTATTAATTTTATTACAACAATTATTAATATACGAATTAATAAATT
ATCATTTGATCAACTACCATTATTTGTTTGATCTGTTGGTATTACAGCCTTATTATTATTACTTTCATTACCAGTACTAG
CTGGAGCTATTACTATACTTTTAACTGATCGAAACTTAAATACATCTTTTTTTGACCCTGCTGGGGGAGGAGACCCTATT
CTTTACCAACATTTATTTTGATTTTTT
>SCHIN003-18|Chilo_auricilius|W17-CaGu3|
AACTTTATACTTTATTTTTGGAATTTGAAGTGGAATAATTGGGACATCTCTAAGACTTTTAATTCGTGCTGAATTAGGAA
CCCCAGGATCATTAATTGGAGATGATCAAATTTATAATACTATTGTTACAGCTCATGCATTTATTATAATTTTTTTTATA
GTTATACCAATTATAATTGGAGGCTTTGGTAACTGATTAGTACCATTAATGCTAGGAGCTCCTGATATAGCCTTCCCTCG
AATAAATAATATAAGATTTTGATTATTACCCCCATCATTAACATTATTAATTTCTAGAAGAATTGTAGAAAATGGAGCTG
GAACAGGATGAACGGTATACCCCCCCCTTTCATCAAATATTGCCCATGGTGGAAGTTCTGTAGATTTAGCCATTTTTTCT
CTTCATTTAGCTGGTATTTCCTCAATTTTAGGAGCTATTAATTTTATTACAACAATTATTAATATACGAATTAATAAATT
ATCATTTGATCAACTACCATTATTTGTTTGATCTGTTGGTATTACAGCCTTATTATTATTACTTTCATTACCAGTACTAG
CTGGAGCTATTACTATACTTTTAACTGATCGAAACTTAAATACATCTTTTTTTGACCCTGCTGGGGGAGGAGACCCTATT
CTTTACCAACATTTATTTTGATTTTTT
>SCHIN004-18|Chilo_auricilius|W17-CaGu4|
AACTTTATACTTTATTTTTGGAATTTGAAGTGGAATAATTGGGACATCTCTAAGACTTTTAATTCGTGCTGAATTAGGAA
CCCCAGGATCATTAATTGGAGATGATCAAATTTATAATACTATTGTTACAGCTCATGCATTTATTATAATTTTTTTTATA
GTTATACCAATTATAATTGGAGGCTTTGGTAACTGATTAGTACCATTAATGCTAGGAGCTCCTGATATAGCCTTCCCTCG
AATAAATAATATAAGATTTTGATTATTACCCCCATCATTAACATTATTAATTTCTAGAAGAATTGTAGAAAATGGAGCTG
GAACAGGATGAACGGTATACCCCCCCCTTTCATCAAATATTGCCCATGGTGGAAGTTCTGTAGATTTAGCCATTTTTTCT
CTTCATTTAGCTGGTATTTCCTCAATTTTAGGAGCTATTAATTTTATTACAACAATTATTAATATACGAATTAATAAATT
ATCATTTGATCAACTACCATTATTTGTTTGATCTGTTGGTATTACAGCCTTATTATTATTACTTTCATTACCAGTACTAG
CTGGAGCTATTACTATACTTTTAACTGATCGAAACTTAAATACATCTTTTTTTGACCCTGCTGGGGGAGGAGACCCTATT
CTTTACCAACATTTATTTTGATTTTTT
>SCHIN005-18|Chilo_auricilius|W17-CaGu5|
AACTTTATACTTTATTTTTGGAATTTGAAGTGGAATAATTGGGACATCTCTAAGACTTTTAATTCGTGCTGAATTAGGAA
CCCCGGGATCATTAATTGGAGATGATCAAATTTATAATACTATTGTTACAGCTCATGCATTTATTATAATTTTTTTTATA
GTTATACCAATTATAATTGGAGGCTTTGGTAACTGATTAGTACCATTAATGCTAGGAGCTCCTGATATAGCCTTCCCTCG
AATAAATAATATAAGATTTTGATTATTACCCCCATCATTAACATTATTAATTTCTAGAAGAATTGTAGAAAATGGAGCTG
GAACAGGATGAACGGTATACCCCCCCCTTTCATCAAATATTGCCCATGGTGGAAGTTCTGTAGATTTAGCCATTTTTTCT
CTTCATTTAGCTGGTATTTCCTCAATTTTAGGAGCTATTAATTTTATTACAACAATTATTAATATACGAATTAATAAATT
ATCATTTGATCAACTACCATTATTTGTTTGATCTGTTGGTATTACAGCCTTATTATTATTACTTTCATTACCAGTACTAG
CTGGAGCTATTACTATACTTTTAACTGATCGAAACTTAAATACATCTTTTTTTGACCCTGCTGGGGGAGGAGACCCTATT
CTTTACCAACATTTATTTTGATTTTTT
>SCHIN006-18|Chilo_infuscatellus|W17-CiDe1|
AACTTTATATTTTATTTTTGGAATTTGAGCAGGAATAATTGGAACTTCTCTTAGACTTTTAATTCGAGCTGAATTAGGAA
CTCCGGGATCTTTAATTGGGGATGATCAAATTTATAGCACTATTGTTACAGCTCATGCATTTATTATAATTTTTTTTATA
GTAATACCAATTATAATCGGAGGATTTGGAAATTGATTAGTTCCTTTAATACTAGGAGCACCTGATATAGCTTTCCCACG
GATAAATAATATAAGTTTCTGATTATTACCCCCATCATTAACATTATTAATTTCTAGAAGAATTGTTGAAAATGGAGCAG
GAACTGGTTGAACTGTTTATCCCCCTTTATCTTCAAATATTGCTCATGGGGGAAGCTCTGTAGATTTAGCAATTTTTTCC
CTTCATTTAGCAGGTATTTCATCAATTTTAGGAGCTATTAATTTTATTACAACAATTATTAATATACGAGTTAATGGTCC
ATCATTTGATCAAATACCTTTATTTGTTTGATCTGTAGGAATTACAGCACTATTATTATTACTTTCTCTGCCAGTATTAG
CAGGTGCTATTACTATACTACTAACTGATCGAAATCTAAATACATCTTTTTTTGACCCTGCTGGAGGGGGGGATCCAATC
CTCTATCAACATTTATTTTGATTTTCT
>SCHIN007-18|Chilo_infuscatellus|W17-CiDe2|
AACTTTATATTTTATTTTTGGAATTTGAGCAGGAATAATTGGAACTTCTCTTAGACTTTTAATTCGAGCTGAATTAGGAA
CTCCGGGATCTTTAATTGGGGATGATCAAATTTATAACACTATTGTTACAGCTCATGCATTTATTATAATTTTTTTTATA
GTAATACCAATTATAATCGGAGGATTTGGAAATTGATTAGTTCCTTTAATACTAGGAGCACCTGATATAGCTTTCCCACG
GATAAATAATATAAGTTTCTGATTATTACCCCCATCATTAACATTATTAATTTCTAGAAGAATTGTTGAAAATGGAGCAG
GAACTGGTTGAACTGTTTATCCCCCTTTATCTTCAAATATTGCTCATGGGGGAAGCTCTGTAGATTTAGCAATTTTTTCC
CTTCATTTAGCAGGTATTTCATCAATTTTAGGAGCTATTAATTTTATTACAACAATTATTAATATACGAGTTAATGGTCT
ATCATTTGATCAAATACCTTTATTTGTTTGATCTGTAGGAATTACAGCACTATTATTATTACTTTCTCTGCCAGTATTAG

CAGGTGCTATTACTATACTACTAACTGATCGAAATCTAAATACATCTTTTTTTGACCCTGCTGGAGGGGGGGATCCAATC
CTCTATCAACATTTATTTTGATTTTTT
>SCHIN008-18|Chilo_infuscatellus|W17-CiDe3|
AACTTTATATTTTATTTTTGGAATTTGAGCAGGAATAATTGGAACTTCTCTTAGACTTTTAATTCGAGCTGAATTAGGAA
CTCCGGGATCTTTAATTGGGGATGATCAAATTTATAACACTATTGTTACAGCTCATGCATTTATTATAATTTTTTTTATA
GTAATACCAATTATAATCGGAGGATTTGGAAATTGATTAGTTCCTTTAATACTAGGAGCACCTGATATAGCTTTCCCACG
GATAAATAATATAAGTTTCTGATTATTACCCCCATCATTAACATTATTAATTTCTAGAAGAATTGTTGAAAATGGAGCAG
GAACTGGTTGAACTGTTTATCCCCCTTTATCTTCAAATATTGCTCATGGGGGCAGCTCTGTAGATTTAGCAATTTTTTCC
CTTCATTTAGCAGGTATTTCATCAATTTTAGGAGCTATTAATTTTATTACAACAATTATTAATATACGAGTTAATGGTCT
ATCATTTGATCAAATACCTTTATTTGTTTGATCTGTAGGAATTACAGCACTATTATTATTACTTTCTCTGCCAGTATTAG
CAGGTGCTATTACTATACTACTAACTGATCGAAATCTAAATACATCTTTTTTTGACCCTGCTGGAGGGGGGGATCCAATC
CTCTATCAACATTTATTTTGATTTTTT
>SCHIN009-18|Chilo_infuscatellus|W17-CiDe4|
AACTTTATATTTTATTTTTGGAATTTGAGCAGGAATAATTGGAACTTCTCTTAGACTTTTAATTCGAGCTGAATTAGGAA
CTCCGGGATCTTTAATTGGGGATGATCAAATTTATAACACTATTGTTACAGCTCATGCATTTATTATAATTTTTTTTATA
GTAATACCAATTATAATCGGAGGATTTGGAAATTGATTAGTTCCTTTAATACTAGGAGCACCTGATATAGCTTTCCCACG
GATAAATAATATAAGTTTCTGATTATTACCCCCATCATTAACATTATTAATTTCTAGAAGAATTGTTGAAAATGGAGCAG
GAACTGGTTGAACTGTTTATCCCCCTTTATCTTCAAATATTGCTCATGGGGGAAGCTCTGTAGATTTAGCAATTTTTTCC
CTTCATTTAGCAGGTATTTCATCAATTTTAGGAGCTATTAATTTTATTACAACGATTATTAATATACGAGTTAATGGTCT
ATCATTTGATCAAATACCTTTATTTGTTTGATCTGTAGGAATTACAGCACTATTATTATTACTTTCTCTGCCAGTATTAG
CAGGTGCTATTACTATACTACTAACTGATCGAAATCTAAATACATCTTTTTTTGACCCTGCTGGAGGGGGGGATCCAATC
CTCTATCAACATTTATTTTGATTTTTT
>SCHIN010-18|Chilo_infuscatellus|W17-CiDe5|
AACTTTATATTTTATTTTTGGAATTTGAGCAGGAATAATTGGAACTTCTCTTAGACTTTTAATTCGAGCTGAATTAGGAA
CTCCGGGATCTTTAATTGGGGATGATCAAATTTATAACACTATTGTTACAGCTCATGCATTTATTATAATTTTTTTTATA
GTAATACCAATTATAATCGGAGGATTTGGAAATTGATTAGTTCCTTTAATACTAGGAGCACCTGATATAGCTTTCCCACG
GATAAATAATATAAGTTTCTGATTATTACCCCCATCATTAACATTATTAATTTCTAGAAGAATTGTTGAAAATGGAGCAG
GAACTGGTTGAACTGTTTATCCCCCTTTATCTTCAAATATTGCTCATGGGGGAAGCTCTGTAGATTTAGCAATTTTTTCC
CTTCATTTAGCAGGTATTTCATCAATTTTAGGAGCTATTAATTTTATTACAACAATAATTAATATACGAGTTAATGGTCT
ATCATTTGATCAAATACCTTTATTTGTTTGATCTGTAGGAATTACAGCACTATTATTATTACTTTCTCTGCCAGTATTAG
CAGGTGCTATTACTATACTACTAACTGATCGAAATCTAAATACATCTTTTTTTGACCCTGCTGGAGGGGGGGATCCAATC
CTCTATCAACATTTATTTTGATTTTTT
>SCHIN011-18|Chilo_infuscatellus|W17-CiDe6|
AACTTTATATTTTATTTTTGGAATTTGAGCAGGAATAATTGGAACTTCTCTTAGACTTTTAATTCGAGCTGAATTAGGAA
CTCCGGGATCTTTAATTGGGGATGATCAAATTTATAACACTATTGTTACAGCTCATGCATTTATTATAATTTTTTTTATA
GTAATACCAATTATAATCGGAGGATTTGGAAATTGATTAGTTCCTTTAATACTAGGAGCACCTGATATAGCTTTCCCACG
GATAAATAATATAAGTTTCTGATTATTACCCCCATCATTAACATTATTAATTTCTAGAAGAATTGTTGAAAATGGAGCAG
GAACTGGTTGAACTGTTTATCCCCCTTTATCTTCAAATATTGCTCATGGAGGAAGCTCTGTAGATTTAGCAATTTTTTCC
CTTCATTTAGCAGGTATTTCATCAATTTTAGGAGCTATTAATTTTATTACAACAATTATTAATATACGAGTTAATGGTCT
ATCATTTGATCAAATACCTTTATTTGTTTGATCTGTAGGAATTACAGCACTATTATTATTACTTTCTCTGCCAGTATTAG
CAGGTGCTATTACTATACTACTAACTGATCGAAATCTAAATACATCTTTTTTTGACCCTGCTGGAGGGGGGGATCCAATC
CTCTATCAACATTTATTTTGATTTTTT
>SCHIN012-18|Chilo_infuscatellus|W17-CiDe7|
AACTTTATATTTTATTTTTGGAATTTGAGCAGGAATAATTGGAACTTCTCTTAGACTTTTAATTCGAGCTGAATTAGGAA
CTCCGGGATCTTTAATTGGGGATGATCAAATTTATAACACTATTGTTACAGCTCATGCATTTATTATAATTTTTTTTATA
GTAATACCAATTATAATCGGAGGATTTGGAAATTGATTAGTTCCTTTAATACTAGGAGCACCTGATATAGCTTTCCCACG
GATAAATAATATAAGTTTCTGATTATTACCCCCATCATTAACATTATTAATTTCTAGAAGAATTGTTGAAAATGGAGCAG
GAACTGGTTGAACTGTTTATCCCCCTTTATCTTCAAATATTGCTCATGGGGGAAGCTCTGTAGATTTAGCAATTTTTTCC
CTTCATTTAGCAGGTATTTCATCAATTTTAGGAGCTATTAATTTTATTACAACAATTATTAATATACGAGTTAATGGTCT
ATCATTTGATCAAATACCTTTATTTGTTTGATCTGTAGGAATTACAGCACTATTATTATTACTTTCTCTGCCAGTATTAG
CAGGTGCTATTACTATACTACTAACTGATCGAAATCTAAATACATCTTTTTTTGACCCTACTGGAGGGGGGGATCCAATC
CTCTATCAACATTTATTTTGATTTTTT
>SCHIN013-18|Chilo_infuscatellus|W17-CiKa1|
AACTTTATATTTTATTTTTGGAATTTGAGCAGGAATAATTGGAACTTCTCTTAGACTTTTAATTCGAGCTGAATTAGGAA
CTCCGGGATCTTTAATTGGGGATGATCAAATTTATAACACTATTGTTACAGCTCATGCATTTATTATAATTTTTTTTATA
GTAATACCAATTATAATCGGAGGATTTGGAAATTGATTAGTTCCTTTAATACTAGGGGCACCTGATATAGCTTTCCCACG
GATAAATAATATAAGTTTCTGATTATTACCCCCATCATTAACATTATTAATTTCTAGAAGAATTGTTGAAAATGGAGCAG

GAACTGGTTGAACTGTTTATCCCCCTTTATCTTCAAATATTGCTCATGGAGGAAGCTCTGTAGATTTAGCAATTTTTTCC
CTTCATTTAGCAGGTATTTCATCAATTTTAGGAGCTATTAATTTTATTACAACAATTATTAATATACGAGTTAATGGTCT
ATCATTTGATCAAATACCTTTATTTGTTTGATCTGTAGGAATTACAGCACTATTATTATTACTTTCTCTGCCAGTATTAG
CAGGTGCTATTACTATACTACTAACTGATCGAAATCTAAATACATCTTTTTTTGACCCTGCTGGAGGGGGGGATCCAATC
CTCTATCAACATTTATTTTGATTTTTT
>SCHIN014-18|Chilo_infuscatellus|W17-CiKa2|
AACTTTATATTTTATTTTTGGAATTTGAGCAGGAATAATTGGAACTTCTCTTAGACTTTTAATTCGAGCTGAATTAGGAA
CTCCGGGATCTTTAATTGGGGATGATCAAATTTATAACACTATTGTTACAGCTCATGCATTTATTATAATTTTTTTTATA
GTAATACCAATTATAATCGGAGGATTTGGAAATTGATTAGTTCCTTTAATACTAGGAGCACCTGATATAGCTTTCCCACG
GATAAATAATATAAGTTTCTGATTATTACCCCCATCATTAACATTATTAATTTCTAGAAGAATTGTTGAAAATGGAGCAG
GAACTGGTTGAACTGTTTATCCCCCTTTATCTTCAAATATTGCTCATGGGGGAAGCTCTGTAGATTTAGCTATTTTTTCC
CTTCATTTAGCAGGTATTTCATCAATTTTAGGAGCTATTAATTTTATTACAACAATTATTAATATACGAGTTAATGGTCT
ATCATTTGATCAAATACCTTTATTTGTTTGATCTGTAGGAATTACAGCACTATTATTATTACTTTCTCTGCCAGTATTAG
CAGGTGCTATTACTATACTACTAACTGATCGAAATCTAAATACATCTTTTTTTGACCCTGCTGGAGGGGGGGATCCAATC
CTCTATCAACATTTATTTTGATTTTTT
>SCHIN015-18|Chilo_infuscatellus|W17-CiKa3|
AACTTTATATTTTATTTTTGGAATTTGAGCAGGAATAATTGGAACTTCTCTTAGACTTTTAATTCGAGCTGAATTAGGAA
CTCCGGGATCTTTAATTGGGGATGATCAAATTTATAACACCATTGTTACAGCTCATGCATTTATTATAATTTTTTTTATA
GTAATACCAATTATAATCGGAGGATTTGGAAATTGATTAGTTCCTTTAATACTAGGAGCACCTGATATAGCTTTCCCACG
GATAAATAATATAAGTTTCTGATTATTACCCCCATCATTAACATTATTAATTTCTAGAAGAATTGTTGAAAATGGAGCAG
GAACTGGTTGAACTGTTTATCCCCCTTTATCTTCAAATATTGCTCATGGGGGAAGCTCTGTAGATTTAGCAATTTTTTCC
CTTCATTTAGCAGGTATTTCATCAATTTTAGGAGCTATTAATTTTATTACAACAATTATTAATATACGAGTTAATGGTCT
ATCATTTGATCAAATACCTTTATTTGTTTGATCTGTAGGAATTACAGCACTATTATTATTACTTTCTCTGCCAGTATTAG
CAGGTGCTATTACTATACTACTAACTGATCGAAATCTAAATACATCTTTTTTTGACCCTGCTGGAGGAGGGGATCCAATC
CTCTATCAACATTTATTTTGATTTTTT
>SCHIN016-18|Chilo_infuscatellus|W17-CiKa4|
AACTTTATATTTTATTTTTGGAATTTGAGCAGGAATAATTGGAACTTCTCTTAGACTTTTAATTCGAGCTGAATTAGGAA
CTCCGGGATCTTTAATTGGGGATGATCAAATTTATAACACCATTGTTACAGCTCATGCATTTATTATAATTTTTTTTATA
GTAATACCAATTATAATCGGAGGATTTGGAAATTGATTAGTTCCTTTAATACTAGGAGCACCTGATATAGCTTTCCCACG
GATAAATAATATAAGTTTCTGATTATTACCCCCATCATTAACATTATTAATTTCTAGAAGAATTGTTGAAAATGGAGCAG
GAACTGGTTGAACTGTTTATCCCCCTTTATCTTCAAATATTGCTCATGGGGGAAGCTCTGTAGATTTAGCAATTTTTTCC
CTTCATTTAGCAGGTATTTCATCAATTTTAGGAGCTATTAATTTTATTACAACAATTATTAATATACGAGTTAATGGTCT
ATCATTTGATCAAATACCTTTATTTGTTTGATCTGTAGGAATTACAGCACTATTATTATTACTTTCTCTGCCAGTATTAG
CAGGTGCTATTACTATACTACTAACTGATCGAAATCTAAATACATCTTTTTTTGACCCTGCTGGAGGAGGGGATCCAATC
CTCTATCAACATTTATTTTGATTTTTT
>SCHIN017-18|Chilo_infuscatellus|W17-CiLa2|
AACTTTATATTTTATTTTTGGAATTTGAGCAGGAATAATTGGAACTTCTCTTAGACTTTTAATTCGAGCTGAATTAGGAA
CTCCGGGATCTTTAATTGGAGATGATCAAATTTATAATACTATTGTTACAGCTCATGCATTTATTATAATTTTTTTTATA
GTAATACCAATTATAATCGGAGGATTTGGAAATTGATTAGTTCCTTTAATACTAGGGGCACCTGATATAGCTTTCCCACG
GATAAATAATATAAGTTTTTGATTATTGCCACCATCATTAACATTATTGATTTCTAGAAGAATTGTTGAAAACGGAGCAG
GAACTGGTTGAACTGTTTATCCCCCCTTATCTTCAAATATTGCTCATGGGGGAAGCTCTGTAGATTTAGCAATTTTTTCC
CTTCATTTAGCTGGTATCTCATCAATTCTAGGGGCTATTAATTTTATCACAACAATTATTAATATACGAGTTAATGGTCT
ATCATTTGATCAAATACCTTTATTTGTTTGATCTGTAGGAATTACAGCACTACTATTATTACTTTCCTTACCAGTATTAG
CAGGTGCCATTACTATACTACTAACTGATCGAAATCTAAATACATCTTTTTTTGATCCTGCTGGAGGGGGGGATCCAATT
CTATATCAACATTTATTTTGATTTTTT
>SCHIN018-18|Chilo_infuscatellus|W17-CiLa3|
AACTTTATATTTTATTTTTGGAATTTGAGCAGGAATAATTGGAACTTCTCTTAGACTTTTAATTCGAGCTGAATTAGGAA
CTCCGGGATCTTTAATTGGAGATGATCAAATTTATAATACTATTGTTACAGCTCATGCATTTATTATAATTTTTTTTATA
GTAATACCAATTATAATCGGAGGATTTGGAAATTGATTAGTTCCTTTAATACTAGGGGCACCTGATATAGCTTTCCCACG
GATAAATAATATAAGTTTTTGATTATTGCCACCATCATTAACATTATTGATTTCTAGAAGAATTGTTGAAAACGGAGCAG
GAACTGGTTGAACTGTTTATCCCCCCTTATCTTCAAATATTGCTCATGGGGGAAGCTCTGTAGATTTAGCAATTTTTTCC
CTTCATTTAGCTGGTATCTCATCAATTCTAGGGGCTATTAATTTTATCACAACAATTATTAATATACGAGTTAATGGTCT
ATCATTTGATCAAATACCTTTATTTGTTTGATCTGTAGGAATTACAGCACTACTATTATTACTTTCCTTACCAGTATTAG
CAGGTGCCATTACTATACTACTAACTGATCGAAATCTAAATACATCTTTTTTTGATCCTGCTGGAGGGGGGGATCCAATT
CTATATCAACATTTATTTTGATTTTTT
>SCHIN019-18|Chilo_infuscatellus|W17-CiLa4|
AACTTTATATTTTATTTTTGGAATTTGAGCAGGAATAATTGGAACTTCTCTTAGACTTTTAATTCGAGCTGAATTAGGAA

CTCCAGGATCTTTAATTGGGGATGATCAAATTTATAATACCATTGTTACAGCTCATGCATTTATTATAATTTTTTTTATA
GTAATACCAATTATAATCGGAGGATTTGGAAATTGATTAGTTCCTTTAATACTAGGGGCACCTGATATAGCTTTCCCACG
GATAAATAATATAAGTTTTTGATTATTGCCACCATCATTAACATTATTGATTTCTAGAAGAATTGTTGAAAACGGAGCAG
GAACTGGTTGAACTGTTTATCCCCCTTTATCTTCAAATATTGCTCATGGGGGAAGCTCTGTAGATTTAGCAATTTTTTCC
CTTCATTTAGCTGGTATCTCATCAATTTTAGGGGCTATTAATTTTATCACAACAATTATTAATATACGAGTTAATGGTCT
ATCATTTGATCAAATACCTTTATTTGTTTGATCTGTAGGAATTACAGCACTATTATTATTACTTTCCTTACCAGTATTAG
CAGGTGCCATTACTATACTACTAACTGATCGAAATCTAAATACATCTTTTTTTGATCCTGCTGGAGGGGGGGATCCAATC
CTATATCAACATTTATTTTGATTTTTT
>SCHIN020-18|Chilo_infuscatellus|W17-CiLa5|
AACTTTATATTTTATTTTTGGAATTTGAGCAGGAATAATTGGAACTTCTCTTAGACTTTTAATTCGAGCTGAATTAGGAA
CTCCGGGATCTTTAATTGGAGATGATCAAATTTATAATACTATTGTTACAGCTCATGCATTTATTATAATTTTTTTTATA
GTAATACCAATTATAATCGGAGGATTTGGAAATTGATTAGTTCCTTTAATACTAGGGGCACCTGATATAGCTTTCCCACG
GATAAATAATATAAGTTTTTGATTATTGCCACCATCATTAACATTATTGATTTCTAGAAGAATTGTTGAAAACGGAGCAG
GAACTGGTTGAACTGTTTATCCCCCCTTATCTTCAAATATTGCTCATGGGGGAAGCTCTGTAGATTTAGCAATTTTTTCC
CTTCATTTAGCTGGTATCTCATCAATTCTAGGGGCTATTAATTTTATCACAACAATTATTAATATACGAGTTAATGGTCT
ATCATTTGATCAAATACCTTTATTTGTTTGATCTGTAGGAATTACAGCACTACTATTATTACTTTCCTTACCAGTATTAG
CAGGTGCCATTACTATACTACTAACTGATCGAAATCTAAATACATCTTTTTTTGATCCTGCTGGAGGGGGGGATCCAATT
CTATATCAACATTTATTTTGATTTTTT
>SCHIN021-18|Chilo_infuscatellus|W17-CiZj1|
AACTTTATATTTTATTTTTGGAATTTGAGCAGGAATAATTGGAACTTCTCTTAGACTTTTAATTCGAGCTGAATTAGGAA
CTCCGGGATCTTTAATTGGAGATGATCAAATTTATAATACTATTGTTACAGCTCATGCATTTATTATAATTTTTTTTATA
GTAATACCAATTATAATCGGAGGATTTGGAAATTGATTAGTTCCTTTAATACTAGGGGCACCTGATATAGCTTTCCCACG
GATAAATAATATAAGTTTTTGATTATTGCCACCATCATTAACATTATTGATTTCTAGAAGAATTGTTGAAAACGGAGCAG
GAACTGGTTGAACTGTTTATCCCCCCTTATCTTCAAATATTGCTCATGGTGGAAGCTCTGTAGATTTAGCAATTTTTTCC
CTTCATTTAGCTGGTATCTCATCAATTCTAGGGGCTATTAATTTTATCACAACAATTATTAATATACGAGTTAATGGTCT
ATCATTTGATCAAATACCTTTATTTGTTTGATCTGTAGGAATTACAGCACTATTATTATTACTTTCCTTACCAGTATTAG
CAGGTGCCATTACTATACTACTAACTGATCGAAATCTAAATACATCTTTTTTTGATCCTGCTGGAGGGGGGGATCCAATT
CTATATCAACATTTATTTTGATTTTTT
>SCHIN022-18|Chilo_infuscatellus|W17-CiZj2|
AACTTTATATTTTATTTTTGGAATTTGAGCAGGAATAATTGGAACTTCTCTTAGACTTTTAATTCGAGCTGAATTAGGAA
CTCCGGGATCTTTAATTGGAGATGATCAAATTTATAATACTATTGTTACAGCTCATGCATTTATTATAATTTTTTTTATA
GTAATACCAATTATAATCGGAGGATTTGGAAATTGATTAGTTCCTTTAATACTAGGGGCACCTGATATAGCTTTCCCTCG
GATAAATAATATAAGTTTTTGATTATTGCCACCATCATTAACATTATTGATTTCTAGAAGAATTGTTGAAAATGGAGCAG
GAACTGGTTGAACTGTTTATCCCCCCTTATCTTCAAATATTGCTCATGGTGGAAGCTCTGTAGATTTAGCAATTTTTTCC
CTTCATTTAGCTGGTATCTCATCAATTCTAGGGGCTATTAATTTTATCACAACAATTATTAATATACGAGTTAATGGTCT
ATCATTTGATCAAATACCTTTATTTGTTTGATCTGTAGGAATTACAGCACTATTATTATTACTTTCCTTACCAGTATTAG
CAGGTGCCATTACTATACTACTAACTGATCGAAATCTAAATACATCTTTTTTTGATCCTGCTGGGGGGGGGGATCCAATT
CTATATCAACATTTATTTTGATTTTTT
>SCHIN023-18|Chilo_infuscatellus|W17-CiZj3|
AACTTTATATTTTATTTTTGGAATTTGAGCAGGAATAATTGGAACTTCTCTTAGACTTTTAATTCGAGCTGAATTAGGAA
CTCCGGGATCTTTAATTGGAGATGATCAAATTTATAATACTATTGTTACAGCTCATGCATTTATTATAATTTTTTTTATA
GTAATACCAATTATAATCGGAGGATTTGGAAATTGATTAGTTCCTTTAATACTAGGGGCACCTGATATAGCTTTCCCACG
GATAAATAATATAAGTTTTTGATTATTGCCACCATCATTAACATTATTGATTTCTAGAAGAATTGTTGAAAACGGAGCAG
GAACTGGTTGAACTGTTTATCCCCCCTTATCTTCAAATATTGCTCATGGTGGAAGCTCTGTAGATTTAGCAATTTTTTCC
CTTCATTTAGCTGGTATCTCATCAATTCTAGGGGCTATTAATTTTATCACAACAATTATTAATATACGAGTTAATGGTCT
ATCATTTGATCAAATACCTTTATTTGTTTGATCTGTAGGAATTACAGCACTATTATTATTACTTTCCTTACCAGTATTAG
CAGGTGCCATTACTATACTACTAACTGATCGAAATCTAAATACATCTTTTTTTGATCCTGCTGGAGGGGGGGATCCAATT
CTATATCAACATTTATTTTGATTTTTT
>SCHIN024-18|Chilo_infuscatellus|W17-CiZj4|
AACTTTATATTTTATTTTTGGAATTTGAGCAGGAATAATTGGAACTTCTCTTAGACTTTTAATTCGAGCTGAATTAGGAA
CTCCGGGATCTTTAATTGGAGATGATCAAATTTATAATACTATTGTTACAGCTCATGCATTTATTATAATTTTTTTTATA
GTAATACCAATTATAATCGGAGGATTTGGAAATTGATTAGTTCCTTTAATACTAGGGGCACCTGATATAGCTTTCCCACG
GATAAATAATATAAGTTTTTGATTATTGCCACCATCATTAACATTATTGATTTCTAGAAGAATTGTTGAAAATGGAGCAG
GAACTGGTTGAACTGTTTATCCCCCCTTATCTTCAAATATTGCTCATGGTGGAAGCTCTGTAGATTTAGCAATTTTTTCC
CTTCATTTAGCTGGTATCTCATCAATTCTAGGGGCTATTAATTTTATCACAACAATTATTAATATACGAGTTAATGGTCT
ATCATTTGATCAAATACCTTTATTTGTTTGATCTGTAGGAATTACAGCACTATTATTATTACTTTCCTTACCAGTATTAG
CAGGTGCCATTACTATACTACTAACTGATCGAAATCTAAATACATCTTTTTTTGATCCTGCTGGGGGGGGGGATCCAATT

CTATATCAACATTTATTTTGATTTTTT
>SCHIN025-18|Chilo_sacchariphagus|W17-CsFu1|
AACTTTATATTTTATTTTTGGAATTTGAGCTGGAATAGTTGGAACATCCCTTAGACTTTTAATTCGAGCTGAATTAGGAA
ATCCAGGTTCATTAATCGGAGATGATCAAATTTATAATACTATTGTTACAGCCCATGCATTTATTATAATTTTTTTTATA
GTAATACCAATTATAATTGGAGGATTTGGAAATTGATTAGTTCCATTAATATTAGGGGCTCCTGATATAGCCTTCCCTCG
TCTAAATAATATAAGATTTTGATTATTACCCCCTTCTTTAACCCTTCTAATTTCTAGAAGAATCGTTGAAAATGGAGCAG
GAACTGGATGAACAGTCTACCCCCCCCTATCTTCCAATATTTCACATGCTGGAAGTTCAGTAGATTTAGCCATCTTCTCC
CTTCATTTAGCTGGAATTTCTTCAATTTTAGGAGCTATCAATTTCATTACTACAATTATTAATATACGAATTAATGGATT
ATTATTTGATCAAATACCATTATTTGTTTGATCTGTTGGTATCACAGCATTACTTCTCCTCCTTTCTTTACCAGTATTAG
CAGGTGCTATTACTATACTATTAACTGACCGAAATTTAAATACATCTTTTTTTGACCCAGCTGGAGGAGGTGATCCAATT
TTATATCAACATTTATTTTGATTTTTT
>SCHIN026-18|Chilo_sacchariphagus|W17-CsFu2|
AACTTTATATTTTATTTTTGGAATTTGAGCTGGAATAGTTGGAACATCCCTTAGACTTTTAATTCGAGCTGAATTAGGAA
ATCCAGGTTCATTAATCGGAGATGATCAAATTTATAATACTATTGTTACAGCCCATGCATTTATTATAATTTTTTTTATA
GTAATACCAATTATAATTGGAGGATTTGGAAATTGATTAGTTCCATTAATATTAGGGGCTCCTGATATAGCCTTCCCTCG
TCTAAATAATATAAGATTTTGATTATTACCCCCTTCTTTAACCCTTCTAATTTCTAGAAGAATCGTTGAAAATGGAGCAG
GAACTGGATGAACAGTCTACCCCCCCCTATCTTCCAATATTTCACATGCTGGAAGTTCAGTAGATTTAGCCATCTTCTCC
CTTCATTTAGCTGGAATTTCTTCAATTTTAGGAGCTATCAATTTCATTACTACAATTATTAATATACGAATTAATGGATT
ATTATTTGATCAAATACCATTATTTGTTTGATCTGTTGGTATTACAGCATTACTTCTCCTCCTTTCTTTACCAGTATTAG
CAGGTGCTATTACTATACTATTAACTGACCGAAATTTAAATACATCTTTTTTTGACCCAGCTGGAGGAGGTGATCCAATT
TTATATCAACATTTATTTTGATTTTTT
>SCHIN027-18|Chilo_sacchariphagus|W17-CsFu3|
AACTTTATATTTTATTTTTGGAATTTGAGCTGGAATAGTTGGAACATCCCTTAGACTTTTAATTCGAGCTGAATTAGGAA
ATCCAGGTTCATTAATCGGAGATGATCAAATTTATAATACTATTGTTACAGCCCATGCATTTATTATAATTTTTTTTATA
GTAATACCAATTATAATTGGAGGATTTGGAAATTGATTAGTTCCATTAATATTAGGGGCTCCTGATATAGCCTTCCCTCG
TCTAAATAATATAAGATTTTGATTATTACCCCCTTCTTTAACCCTTCTAATTTCTAGAAGAATCGTTGAAAATGGAGCAG
GAACTGGATGAACAGTCTACCCCCCCCTATCTTCCAATATTTCACATGCTGGAAGTTCAGTAGATTTAGCCATCTTCTCC
CTTCATTTAGCTGGAATTTCTTCAATTTTAGGAGCTATCAATTTCATTACTACAATTATTAATATACGAATTAATGGATT
ATTATTTGATCAAATACCATTATTTGTTTGATCTGTTGGTATTACAGCATTACTTCTCCTCCTTTCTTTACCAGTATTAG
CAGGTGCTATTACTATACTATTAACTGACCGAAATTTAAATACATCTTTTTTTGACCCAGCTGGAGGAGGTGATCCAATT
TTATATCAACATTTATTTTGATTTTTT
>SCHIN028-18|Chilo_sacchariphagus|W17-CsFu4|
AACTTTATATTTTATTTTTGGAATTTGAGCTGGAATAGTTGGAACATCCCTTAGACTTTTAATTCGAGCTGAATTAGGAA
ATCCAGGTTCATTAATCGGAGATGATCAAATTTATAATACTATTGTTACAGCCCATGCATTTATTATAATTTTTTTTATA
GTAATACCAATTATAATTGGAGGATTTGGAAATTGATTAGTTCCATTAATATTAGGGGCTCCTGATATAGCCTTCCCTCG
TCTAAATAATATAAGATTTTGATTATTACCCCCTTCTTTAACCCTTCTAATTTCTAGAAGAATCGTTGAAAATGGAGCAG
GAACTGGATGAACAGTCTACCCCCCCCTATCTTCCAATATTTCACATGCTGGAAGTTCAGTAGATTTAGCCATCTTCTCC
CTTCATTTAGCTGGAATTTCTTCAATTTTAGGAGCTATCAATTTCATTACTACAATTATTAATATACGAATTAATGGATT
ATTATTTGATCAAATACCATTATTTGTTTGATCTGTTGGTATTACAGCATTACTTCTCCTCCTTTCTTTACCAGTATTAG
CAGGTGCTATTACTATACTATTAACTGACCGAAATTTAAATACATCTTTTTTTGACCCAGCTGGAGGAGGTGATCCAATT
TTATATCAACATTTATTTTGATTTTTT
>SCHIN029-18|Chilo_sacchariphagus|W17-CsFu5|
AACTTTATATTTTATTTTTGGAATTTGAGCTGGAATAGTTGGAACATCCCTTAGACTTTTAATTCGAGCTGAATTAGGAA
ATCCAGGTTCATTAATCGGAGATGATCAAATTTATAATACTATTGTTACAGCCCATGCATTTATTATAATTTTTTTTATA
GTAATACCAATTATAATTGGAGGATTTGGAAATTGATTAGTTCCATTAATATTAGGGGCTCCTGATATAGCCTTCCCTCG
TCTAAATAATATAAGATTTTGATTATTACCCCCTTCTTTAACCCTTCTAATTTCTAGAAGAATCGTTGAAAATGGAGCAG
GAACTGGATGAACAGTCTACCCCCCCCTATCTTCCAATATTTCACATGCTGGAAGTTCAGTAGATTTAGCCATCTTCTCC
CTTCATTTAGCTGGAATTTCTTCAATTTTAGGAGCTATCAATTTCATTACTACAATTATTAATATACGAATTAATGGATT
ATTATTTGATCAAATACCATTATTTGTTTGATCTGTTGGTATTACAGCATTACTTCTCCTCCTTTCTTTACCAGTATTAG
CAGGTGCTATTACTATACTATTAACTGACCGAAATTTAAATACATCTTTTTTTGACCCAGCTGGAGGAGGTGATCCAATT
TTATATCAACATTTATTTTGATTTTTT
>SCHIN030-18|Chilo_sacchariphagus|W17-CsFu6|
AACTTTATATTTTATTTTTGGAATTTGAGCTGGAATAGTTGGAACATCCCTTAGACTTTTAATTCGAGCTGAATTAGGAA
ATCCAGGTTCATTAATCGGAGATGATCAAATTTATAATACTATTGTTACAGCCCATGCATTTATTATAATTTTTTTTATA
GTAATACCAATTATAATTGGAGGATTTGGAAATTGATTAGTTCCATTAATATTAGGGGCTCCTGATATAGCCTTCCCTCG
TCTAAATAATATAAGATTTTGATTATTACCCCCTTCTTTAACCCTTCTAATTTCTAGAAGAATCGTTGAAAATGGAGCAG
GAACTGGATGAACAGTCTACCCCCCCCTATCTTCCAATATTTCACATGCTGGAAGTTCAGTAGATTTAGCCATCTTCTCC

CTTCATTTAGCTGGAATTTCTTCAATTTTAGGAGCTATCAATTTCATTACTACAATTATTAATATACGAATTAATGGATT
ATTATTTGATCAAATACCATTATTTGTTTGATCTGTTGGTATTACAGCATTACTTCTCCTCCTTTCTTTACCAGTATTAG
CAGGTGCTATTACTATACTATTAACTGACCGAAATTTAAATACATCTTTTTTTGACCCAGCTGGAGGAGGTGATCCAATT
TTATATCAACATTTATTTTGATTTTTT
>SCHIN031-18|Chilo_sacchariphagus|W17-CsFu7|
AACTTTATATTTTATTTTTGGAATTTGAGCTGGAATAGTTGGAACATCCCTTAGACTTTTAATTCGAGCTGAATTAGGAA
ATCCAGGTTCATTAATCGGAGATGATCAAATTTATAATACTATTGTTACAGCCCATGCATTTATTATAATTTTTTTTATA
GTAATACCAATTATAATTGGAGGATTTGGAAATTGATTAGTTCCATTAATATTAGGGGCTCCTGATATAGCCTTCCCTCG
TCTAAATAATATAAGATTTTGATTAATACCCCCTTCTTTAACCCTTCTAATTTCTAGAAGAATCGTTGAAAATGGAGCAG
GAACTGGATGAACAGTCTACCCCCCCCTATCTTCCAATATTTCACATGCTGGAAGTTCAGTAGATTTAGCCATCTTCTCC
CTTCATTTAGCTGGAATTTCTTCAATTTTAGGAGCTATCAATTTCATTACTACAATTATTAATATACGAATTAATGGATT
ATTATTTGATCAAATACCATTATTTGTTTGATCTGTTGGTATTACAGCATTACTTCTCCTCCTTTCTTTACCAGTATTAG
CAGGTGCTATTACTATACTATTAACTGACCGAAATTTAAATACATCTTTTTTTGACCCAGCTGGAGGAGGTGATCCAATT
TTATATCAACATTTATTTTGATTTTTT
>SCHIN032-18|Chilo_sacchariphagus|W17-CsNa1|
AACTTTATATTTTATTTTTGGAATTTGAGCTGGAATAGTTGGAACATCCCTTAGACTTTTAATTCGAGCTGAATTAGGAA
ATCCAGGTTCATTAATCGGAGATGATCAAATTTATAATACTATTGTTACAGCCCATGCATTTATTATAATTTTTTTTATA
GTAATACCAATTATAATTGGAGGATTTGGAAATTGATTAGTTCCATTAATATTAGGGGCTCCTGATATAGCCTTCCCTCG
TCTAAATAATATAAGATTTTGATTATTACCCCCTTCTTTAACCCTTCTAATTTCTAGAAGAATCGTTGAAAATGGAGCAG
GAACTGGATGAACAGTCTACCCCCCCCTATCTTCCAATATTTCACATGCTGGAAGCTCAGTAGATTTAGCCATCTTCTCC
CTTCATTTAGCTGGAATTTCTTCAATTTTAGGAGCTATCAATTTCATTACTACAATTATTAATATACGAATTAATGGATT
ATTATTTGATCAAATACCATTATTTGTTTGATCTGTTGGTATTACGGCATTACTTCTCCTCCTTTCTTTACCAGTATTAG
CAGGTGCTATTACTATACTATTAACTGACCGAAATTTAAATACATCTTTTTTTGACCCAGCTGGAGGAGGTGATCCAATT
TTATATCAACATTTATTTTGATTTTTT
>SCHIN033-18|Chilo_sacchariphagus|W17-CsNa2|
AACTTTATATTTTATTTTTGGAATTTGAGCTGGAATAGTTGGAACATCCCTTAGACTTTTAATTCGAGCTGAATTAGGAA
ATCCAGGTTCATTAATCGGAGATGATCAAATTTATAATACTATTGTTACAGCCCATGCATTTATTATAATTTTTTTTATA
GTAATACCAATTATAATTGGAGGATTTGGAAATTGATTAGTTCCATTAATATTAGGGGCTCCTGATATAGCCTTCCCTCG
TCTAAATAATATAAGATTTTGATTATTACCCCCTTCTTTAACCCTTCTAATTTCTAGAAGAATCGTTGAAAATGGAGCAG
GAACTGGATGAACAGTCTACCCCCCCCTATCTTCCAATATTTCACATGCTGGAAGTTCAGTAGATTTAGCCATCTTCTCC
CTTCATTTAGCTGGAATTTCTTCAATTTTAGGAGCTATCAATTTCATTACTACAATTATTAATATACGAATTAATGGATT
ATTATTTGATCAAATACCATTATTTGTTTGATCTGTTGGTATTACAGCATTACTTCTCCTCCTTTCTTTACCAGTATTAG
CAGGTGCTATTACTATACTATTAACTGACCGAAATTTAAATACATCTTTTTTTGACCCAGCTGGAGGAGGTGATCCAATT
TTATATCAACATTTATTTTGATTTTTT
>SCHIN034-18|Chilo_sacchariphagus|W17-CsNa3|
AACTTTATATTTTATTTTTGGAATTTGAGCTGGAATAGTTGGAACATCCCTTAGACTTTTAATTCGAGCTGAATTAGGAA
ATCCAGGTTCATTAATCGGAGATGATCAAATTTATAATACTATTGTTACAGCCCATGCATTTATTATAATTTTTTTTATA
GTAATACCAATTATAATTGGAGGATTTGGAAATTGATTAGTTCCATTAATATTAGGGGCTCCTGATATAGCCTTCCCTCG
TCTAAATAATATAAGATTTTGATTATTACCCCCTTCTTTAACCCTTCTAATTTCTAGAAGAATCGTTGAAAATGGAGCAG
GAACTGGATGAACAGTCTACCCCCCCCTATCTTCCAATATTTCACATGCTGGAAGTTCAGTAGATTTAGCCATCTTCTCC
CTTCATTTAGCTGGAATTTCTTCAATTTTAGGAGCTATCAATTTCATTACTACAATTATTAATATACGAATTAATGGATT
ATTATTTGATCAAATACCATTATTTGTTTGATCTGTTGGTATTACAGCATTACTTCTCCTCCTTTCTTTACCAGTATTAG
CAGGTGCTATTACTATACTATTAACTGACCGAAATTTAAATACATCTTTTTTTGACCCAGCTGGAGGAGGTGATCCAATT
TTATATCAACATTTATTTTGATTTTTT
>SCHIN035-18|Chilo_sacchariphagus|W17-CsNa4|
AACTTTATATTTTATTTTTGGAATTTGAGCTGGAATAGTTGGAACATCCCTTAGACTTTTAATTCGAGCTGAATTAGGAA
ATCCAGGTTCATTAATCGGAGATGATCAAATTTATAATACTATTGTTACAGCCCATGCATTTATTATAATTTTTTTTATA
GTAATACCAATTATAATTGGAGGATTTGGAAATTGATTAGTTCCATTAATATTAGGGGCTCCTGATATAGCCTTCCCTCG
TCTAAATAATATAAGATTTTGATTATTACCCCCTTCTTTAACCCTTCTAATTTCTAGAAGAATCGTTGAAAATGGAGCAG
GAACTGGATGAACAGTCTACCCCCCCCTATCTTCCAATATTTCACATGCTGGAAGTTCAGTAGATTTAGCCATCTTCTCC
CTTCATTTAGCTGGAATTTCTTCAATTTTAGGAGCTATCAATTTCATTACTACAATTATTAATATACGAATTAATGGATT
ATTATTTGATCAAATACCATTATTTGTTTGATCTGTTGGTATTACAGCATTACTTCTCCTCCTTTCTTTACCAGTATTAG
CAGGTGCTATTACTATACTATTAACTGACCGAAATTTAAATACATCTTTTTTTGACCCAGCTGGAGGAGGTGATCCAATT
TTATATCAACATTTATTTTGATTTTTT
>SCHIN036-18|Chilo_sacchariphagus|W17-CsNa5|
AACTTTATATTTTATTTTTGGAATTTGAGCTGGAATAGTTGGAACATCCCTTAGACTTTTAATTCGAGCTGAATTAGGAA
ATCCAGGTTCATTAATCGGAGATGATCAAATTTATAATACTATTGTTACAGCCCATGCATTTATTATAATTTTTTTTATA

GTAATACCAATTATAATTGGAGGATTTGGAAATTGATTAGTTCCATTAATATTAGGGGCTCCTGATATAGCCTTCCCTCG
TCTAAATAATATAAGATTTTGATTATTACCCCCTTCTTTAACCCTTCTAATTTCTAGAAGAATCGTTGAAAATGGAGCAG
GAACTGGATGAACAGTCTACCCCCCCCTATCTTCCAATATTTCACATGCTGGAAGTTCAGTAGATTTAGCCATCTTCTCC
CTTCATTTAGCTGGAATTTCTTCAATTTTAGGAGCTATCAATTTCATTACTACAATTATTAATATACGAATTAATGGATT
ATTATTTGATCAAATACCATTATTTGTTTGATCTGTTGGTATTACAGCATTACTTCTCCTCCTTTCTTTACCAGTATTAG
CAGGTGCTATTACTATACTATTAACTGACCGAAATTTAAATACATCTTTTTTTGACCCAGCTGGAGGAGGTGATCCAATT
TTATATCAACATTTATTTTGATTTTTT
>SCHIN037-18|Chilo_sacchariphagus|W17-CsNa6|
AACTTTATATTTTATTTTTGGAATTTGAGCTGGAATAGTTGGAACATCCCTTAGACTTTTAATTCGAGCTGAATTAGGAA
ATCCAGGTTCATTAATCGGAGATGATCAAATTTATAATACTATTGTTACAGCCCATGCATTTATTATAATTTTTTTTATA
GTAATACCAATTATAATTGGAGGATTTGGAAATTGATTAGTTCCATTAATATTAGGGGCTCCTGATATAGCCTTCCCTCG
TCTAAATAATATAAGATTTTGATTATTACCCCCTTCTTTAACCCTTCTAATTTCTAGAAGAATCGTTGAAAATGGAGCAG
GAACTGGATGAACAGTCTACCCCCCCCTATCTTCCAATATTTCACATGCTGGAAGCTCAGTAGATTTAGCCATCTTCTCC
CTTCATTTAGCTGGAATTTCTTCAATTTTAGGAGCTATCAATTTCATTACTACAATTATTAATATACGAATTAATGGATT
ATTATTTGATCAAATACCATTATTTGTTTGATCTGTTGGTATTACGGCATTACTTCTCCTCCTTTCTTTACCAGTATTAG
CAGGTGCTATTACTATACTATTAACTGACCGAAATTTAAATACATCTTTTTTTGACCCAGCTGGAGGAGGTGATCCAATT
TTATATCAACATTTATTTTGATTTTTT
>SCHIN038-18|Chilo_sacchariphagus|W17-CsYa1|
AACTTTATATTTTATTTTTGGAATTTGAGCTGGAATAGTTGGAACATCCCTTAGACTTTTAATTCGAGCTGAATTAGGAA
ATCCAGGTTCATTAATCGGAGATGATCAAATTTATAATACTATTGTTACAGCCCATGCATTTATTATAATTTTTTTTATA
GTAATACCAATTATAATTGGAGGATTTGGAAATTGATTAGTTCCATTAATATTAGGGGCTCCTGATATAGCCTTCCCTCG
TCTAAATAATATAAGATTTTGATTATTACCCCCTTCTTTAACCCTTCTAATTTCTAGAAGAATCGTTGAAAATGGAGCAG
GAACTGGATGAACAGTCTACCCCCCCCTATCTTCCAATATTTCACATGCTGGAAGTTCAGTAGATTTAGCCATCTTCTCC
CTTCATTTAGCTGGAATTTCTTCAATTTTAGGAGCTATCAATTTCATTACTACAATTATTAATATACGAATTAATGGATT
ATTATTTGATCAAATACCATTGTTTGTTTGATCTGTTGGTATTACAGCATTACTTCTCCTCCTTTCTTTACCAGTATTAG
CAGGTGCTATTACTATACTATTAACTGACCGAAATTTAAATACATCTTTTTTTGACCCAGCTGGAGGAGGTGATCCAATT
TTATATCAACATTTATTTTGATTTTTT
>SCHIN039-18|Chilo_sacchariphagus|W17-CsYa2|
AACTTTATATTTTATTTTTGGAATTTGAGCTGGAATAGTTGGAACATCCCTTAGACTTTTAATTCGAGCTGAATTAGGAA
ATCCAGGTTCATTAATCGGAGATGATCAAATTTATAATACTATTGTTACAGCCCATGCATTTATTATAATTTTTTTTATA
GTAATACCAATTATAATTGGAGGATTTGGAAATTGATTAGTTCCATTAATATTAGGGGCTCCTGATATAGCCTTCCCTCG
TCTAAATAATATAAGATTTTGATTATTACCCCCTTCTTTAACCCTTCTAATTTCTAGAAGAATCGTTGAAAATGGAGCAG
GAACTGGATGAACAGTCTACCCCCCCCTATCTTCCAATATTTCACATGCTGGAAGTTCAGTAGATTTAGCCATCTTCTCC
CTTCATTTAGCTGGAATTTCTTCAATTTTAGGAGCTATCAATTTCATTACTACAATTATTAATATACGAATTAATGGATT
ATTATTTGATCAAATACCATTATTTGTTTGATCTGTTGGTATTACAGCATTACTTCTCCTCCTTTCTTTACCAGTATTAG
CAGGTGCTATTACTATACTATTAACTGACCGAAATTTAAATACATCTTTTTTTGACCCAGCTGGAGGAGGTGATCCAATT
TTATATCAACATTTATTTTGATTTTTT
>SCHIN040-18|Chilo_sacchariphagus|W17-CsYa3|
AACTTTATATTTTATTTTTGGAATTTGAGCTGGAATAGTTGGAACATCCCTTAGACTTTTAATTCGAGCTGAATTAGGAA
ATCCAGGTTCATTAATCGGAGATGATCAAATTTATAATACTATTGTTACAGCCCATGCATTTATTATAATTTTTTTTATA
GTAATACCAATTATAATTGGAGGATTTGGAAATTGATTAGTTCCATTAATATTAGGGGCTCCTGATATAGCCTTCCCTCG
TCTAAATAATATAAGATTTTGATTATTACCCCCTTCTTTAACCCTTCTAATTTCTAGAAGAATCGTTGAAAATGGAGCAG
GAACTGGATGAACAGTCTACCCCCCCCTATCTTCCAATATTTCACATGCTGGAAGTTCAGTAGATTTAGCCATCTTCTCC
CTTCATTTAGCTGGAATTTCTTCAATTTTAGGAGCTATCAATTTCATTACTACAATTATTAATATACGAATTAATGGATT
ATTATTTGATCAAATACCATTATTTGTTTGATCTGTTGGTATTACAGCATTACTTCTCCTCCTTTCTTTACCAGTATTAG
CAGGTGCTATTACTATACTATTAACTGACCGAAATTTAAATACATCTTTTTTTGACCCAGCTGGAGGAGGTGATCCAATT
TTATATCAACATTTATTTTGATTTTTT
>SCHIN041-18|Chilo_sacchariphagus|W17-CsYa4|
AACTTTATATTTTATTTTTGGAATTTGAGCTGGAATAGTTGGAACATCCCTTAGACTTTTAATTCGAGCTGAATTAGGAA
ATCCAGGTTCATTAATCGGAGATGATCAAATTTATAATACTATTGTTACAGCCCATGCATTTATTATAATTTTTTTTATA
GTAATACCAATTATAATTGGAGGATTTGGAAATTGATTAGTTCCATTAATATTAGGGGCTCCTGATATAGCCTTCCCTCG
TCTAAATAATATAAGATTTTGATTATTACCCCCTTCTTTAACCCTTCTAATTTCTAGAAGAATCGTTGAAAATGGAGCAG
GAACTGGATGAACAGTCTACCCCCCCCTATCTTCCAATATTTCACATGCTGGAAGTTCAGTAGATTTAGCCATCTTCTCC
CTTCATTTAGCTGGAATTTCTTCAATTTTAGGAGCTATCAATTTCATTACTACAATTATTAATATACGAATTAATGGATT
ATTATTTGATCAAATACCATTATTTGTTTGATCTGTTGGTATTACAGCATTACTTCTCCTCCTTTCTTTACCAGTATTAG
CAGGTGCTATTACTATACTATTAACTGACCGAAATTTAAATACATCTTTTTTTGACCCAGCTGGAGGAGGTGATCCAATT
TTATATCAACATTTATTTTGATTTTTT

>SCHIN042-18|Chilo_sacchariphagus|W17-CsYa5|
AACTTTATATTTTATTTTTGGAATTTGAGCTGGAATAGTTGGAACATCCCTTAGACTTTTAATTCGAGCTGAATTAGGAA
ATCCAGGTTCATTAATCGGAGATGATCAAATTTATAATACTATTGTTACAGCCCATGCATTTATTATAATTTTTTTTATA
GTAATACCAATTATAATTGGAGGATTTGGAAATTGATTAGTTCCATTAATATTAGGGGCTCCTGATATAGCCTTCCCTCG
TCTAAATAATATAAGATTTTGATTATTACCCCCTTCTTTAACCCTTCTAATTTCTAGAAGAATCGTTGAAAATGGAGCAG
GAACTGGATGAACAGTCTACCCCCCCCTATCTTCCAATATTTCACATGCTGGAAGTTCAGTAGATTTAGCCATCTTCTCC
CTTCATTTAGCTGGAATTTCTTCAATTTTAGGAGCTATCAATTTCATTACTACAATTATTAATATACGAATTAATGGATT
ATTATTTGATCAAATACCATTATTTGTTTGATCTGTTGGTATTACAGCATTACTTCTCCTCCTTTCTTTACCAGTATTAG
CAGGTGCTATTACTATACTATTAACTGACCGAAATTTAAATACATCTTTTTTTGACCCAGCTGGAGGAGGTGATCCAATT
TTATATCAACATTTATTTTGATTTTTT
>SCHIN043-18|Chilo_sacchariphagus|W17-CsYa6|
AACTTTATATTTTATTTTTGGAATTTGAGCTGGAATAGTTGGAACATCCCTTAGACTTTTAATTCGAGCTGAATTAGGAA
ATCCAGGTTCATTAATCGGAGATGATCAAATTTATAATACTATTGTTACAGCCCATGCATTTATTATAATTTTTTTTATA
GTAATACCAATTATAATTGGAGGATTTGGAAATTGATTAGTTCCATTAATATTAGGGGCTCCTGATATAGCCTTCCCTCG
TCTAAATAATATAAGATTTTGATTATTACCCCCTTCTTTAACCCTTCTAAATTCTAGAAGAATCGTTGAAAATGGAGCAG
GAACTGGATGAACAGTCTACCCCCCCCTATCTTCCAATATTTCACATGCTGGAAGTTCAGTAGATTTAGCCATCTTCTCC
CTTCATTTAGCTGGAATTTCTTCAATTTTAGGAGCTATCAATTTCATTACTACAATTATTAATATACGAATTAATGGATT
ATTATTTGATCAAATACCATTATTTGTTTGATCTGTTGGTATTACAGCATTACTTCTCCTCCTTTCTTTACCAGTATTAG
CAGGTGCTATTACTATACTATTAACTGACCGAAATTTAAATACATCTTTTTTTGACCCAGCTGGAGGAGGTGATCCAATT
TTATATCAACATTTATTTTGATTTTTT
>SCHIN044-18|Chilo_sacchariphagus|W17-CsZj6|
AACTTTATATTTTATTTTTGGAATTTGAGCTGGAATAGTTGGAACATCCCTTAGACTTTTAATTCGAGCTGAATTAGGAA
ATCCAGGTTCATTAATCGGAGATGATCAAATTTATAATACTATTGTTACAGCCCATGCATTTATTATAATTTTTTTTATA
GTAATACCAATTATAATTGGAGGATTTGGAAATTGATTAGTTCCATTAATATTAGGGGCTCCTGATATAGCCTTCCCTCG
TCTAAATAATATAAGATTTTGATTATTACCCCCTTCTTTAACCCTTCTAATTTCTAGAAGAATCGTTGAAAATGGAGCAG
GAACTGGATGAACAGTCTACCCCCCCCTATCTTCCAATATTTCACATGCTGGAAGTTCAGTAGATTTAGCCATCTTCTCC
CTTCATTTAGCTGGAATTTCTTCAATTTTAGGAGCTATCAATTTCATTACTACAATTATTAATATACGAATTAATGGATT
ATTATTTGATCAAATACCATTATTTGTTTGATCTGTTGGTATTACAGCATTACTTCTCCTCCTTTCTTTACCAGTATTAG
CAGGTGCTATTACTATACTATTAACTGACCGAAATTTAAATACATCTTTTTTTGACCCAGCTGGAGGAGGTGATCCAATT
TTATATCAACATTTATTTTGATTTTTT
>SCHIN045-18|Chilo_sacchariphagus|W17-CsZz1|
AACTTTATATTTTATTTTTGGAATTTGAGCTGGAATAGTTGGAACATCCCTTAGACTTTTAATTCGAGCTGAATTAGGAA
ATCCAGGTTCATTAATCGGAGATGATCAAATTTATAATACTATTGTTACAGCCCATGCATTTATTATAATTTTTTTTATG
GTATTACCAATTATAATTGGAGGATTTGGAAATTGATTAGTTCCATTAATATTAGGGGCTCCTGATATAGCCTTCCCTCG
TCTAAATAATATAAGATTTTGATTATTACCCCCTTCTTTAACCCTTCTAATTTCTAGAATAATCGTTGAAAATGGAGCAG
GAAGTGGATGAACAGTCTGCCCCCCCCTATCTTCCACTATTTCACATGCTGGAGGTTCAGTAAAGTTAGCCATCTTCTCC
CTTCATTTAACTGGAATTTCTTCAAGTTTAGGAGCTATCAATTTCATTACTACGATTATTAATATACCAATTAATGGATT
ATTATTTGATCAAATACCATTATTTGTTTGATCTGTTGGTATTACAGCATTACTTCTCCTCCTTTCTTTACCAGTATTAA
CAGGTGCTATTACTATACTATTAACTGACCGAAATTTAAATACATCTTTTTTTGACCCAGCTGGAGGAGGTGATCCAATT
TTATATCAACATTTATTTTGATTTTTT
>SCHIN046-18|Chilo_sacchariphagus|W17-CsZz3|
AACTTTATATTTTATTTTTGGAATTTGAGCTGGAATAGTTGGAACATCCCTTAGACTTTTAATTCGAGCTGAATTAGGAA
ATCCAGGTTCATTAATCGGAGATGATCAAATTTATAATACTATTGTTACAGCCCATGCATTTATTATAATTTTTTTTATA
GTAATACCAATTATAATTGGAGGATTTGGAAATTGATTAGTTCCATTAATATTAGGGGCTCCTGATATAGCCTTCCCTCG
TCTAAATAATATAAGATTTTGATTATTACCCCCTTCTTTAACCCTTCTAATTTCTAGAAGAATCGTTGAAAATGGAGCAG
GAACTGGATGAACAGTCTACCCCCCCCTATCTTCCAATATTTCACATGCTGGAAGTTCAGTAGATTTAGCCATCTTCTCC
CTTCATTTAGCTGGAATTTCTTCAATTTTAGGAGCTATCAATTTCATTACTACAATTATTAATATACGAATTAATGGATT
ATTATTTGATCAAATACCATTATTTGTTTGATCTGTTGGTATTACAGCATTACTTCTCCTCCTTTCTTTACCAGTATTAG
CAGGTGCTATTACTATACTATTAACTGACCGAAATTTAAATACATCTTTTTTTGACCCAGCTGGAGGAGGTGATCCAATT
TTATATCAACATTTATTTTGATTTTTT
>SCHIN047-18|Chilo_sacchariphagus|W17-CsZz4|
AACTTTATATTTTATTTTTGGAATTTGAGCTGGAATAGTTGGAACATCCCTTAGACTTTTAATTCGAGCTGAATTAGGAA
ATCCAGGTTCATTAATCGGAGATGATCAAATTTATAATACTATTGTTACAGCCCATGCATTTATTATAATTTTTTTTATA
GTAATACCAATTATAATTGGAGGATTTGGAAATTGATTAGTTCCATTAATATTAGGGGCTCCTGATATAGCCTTCCCTCG
TCTAAATAATATAAGATTTTGATTATTACCCCCTTCTTTAACCCTTCTAATTTCTAGAAGAATCGTTGAAAATGGAGCAG
GAACTGGATGAACAGTCTACCCCCCCCTATCTTCCAATATTTCACATGCTGGAAGTTCAGTAGATTTAGCCATCTTCTCC
CTTCATTTAGCTGGAATTTCTTCAATTTTAGGAGCTATCAATTTCATTACTACAATTATTAATATACGAATTAATGGATT

ATTATTTGATCAAATACCATTATTTGTTTGATCTGTTGGTATTACAGCATTACTTCTCCTCCTTTCTTTACCAGTATTAG
CAGGTGCTATTACTATACTATTAACTGACCGAAATTTAAATACATCTTTTTTTGACCCAGCTGGAGGAGGTGATCCAATT
TTATATCAACATTTATTTTGATTTTTT
>SCHIN048-18|Chilo_sacchariphagus|W17-CsZz5|
AACTTTATATTTTATTTTTGGAATTTGAGCTGGAATAGTTGGAACATCCCTTAGACTTTTAATTCGAGCTGAATTAGGAA
ATCCAGGTTCATTAATCGGAGATGATCAAATTTATAATACTATTGTTACAGCCCATGCATTTATTATAATTTTTTTTATG
GTATTACCAATTATAATTGGAGGATTTGGAAATTGATTAGTTCCATTAATATTAGGGGCTCCTGATATAGCCTTCCCTCG
TCTAAATAATATAAGATTTTGATTATTACCCCCTTCTTTAACCCTTCTAATTTCTAGAATAATCGTTGAAAATGGAGCAG
GAAGTGGATGAACAGTCTGCCCCCCCCTATCTTCCACTATTTCACATGCTGGAGGTTCAGTAAAGTTAGCCATCTTCTCC
CTTCATTTAACTGGAATTTCTTCAAGTTTAGGAGCTATCAATTTCATTACTACGATTATTAATATACCAATTAATGGATT
ATTATTTGATCAAATACCATTATTTGTTTGATCTGTTGGTATTACAGCATTACTTCTCCTCCTTTCTTTACCAGTATTAA
CAGGTGCTATTACTATACTATTAACTGACCGAAATTTAAATACATCTTTTTTTGACCCAGCTGGAGGAGGTGATCCAATT
TTATATCAACATTTATTTTGATTTTTT
>SCHIN049-18|Chilo_sacchariphagus|W17-CsZz6|
AACTTTATATTTTATTTTTGGAATTTGAGCTGGAATAGTTGGAACATCCCTTAGACTTTTAATTCGAGCTGAATTAGGAA
ATCCAGGTTCATTAATCGGAGATGATCAAATTTATAATACTATTGTTACAGCCCATGCATTTATTATAATTTTTTTTATA
GTAATACCAATTATAATTGGAGGATTTGGAAATTGATTAGTTCCATTAATATTAGGGGCTCCTGATATAGCCTTCCCTCG
TCTAAATAATATAAGATTTTGATTATTACCCCCTTCTTTAACCCTTCTAATTTCTAGAAGAATCGTTGAAAATGGAGCAG
GAACTGGATGAACAGTCTACCCCCCCCTATCTTCCAATATTTCACATGCTGGAAGTTCAGTAGATTTAGCCATCTTCTCC
CTTCATTTAGCTGGAATTTCTTCAATTTTAGGAGCTATCAATTTCATTACTACAATTATTAATATACGAATTAATGGATT
ATTATTTGATCAAATACCATTATTTGTTTGATCTGTTGGTATTACAGCATTACTTCTCCTCCTTTCTTTACCAGTATTAG
CAGGTGCTATTACTATACTATTAACTGACCGAAATTTAAATACATCTTTTTTTGACCCAGCTGGAGGAGGTGATCCAATT
TTATATCAACATTTATTTTGATTTTTT
>SCHIN050-18|Chilo_sacchariphagus|W17-CsZj1|
AACTTTATATTTTATTTTTGGAATTTGAGCTGGAATAGTTGGAACATCCCTTAGACTTTTAATTCGAGCTGAATTAGGAA
ATCCAGGTTCATTAATCGGAGATGATCAAATTTATAATACTATTGTTACAGCCCATGCATTTATTATAATTTTTTTTATA
GTAATACCAATTATAATTGGAGGATTTGGAAATTGATTAGTTCCATTAATATTAGGGGCTCCTGATATAGCCTTCCCTCG
TCTAAATAATATAAGATTTTGATTATTACCCCCTTCTTTAACCCTTCTAATTTCTAGAAGAATCGTTGAAAATGGAGCAG
GAACTGGATGAACAGTCTACCCCCCCCTATCTTCCAATATTTCACATGCTGGAAGTTCAGTAGATTTAGCCATCTTCTCC
CTTCATTTAGCTGGAATTTCTTCAATTTTAGGAGCTATCAATTTCATTACTACAATTATTAATATACGAATTAATGGATT
ATTATTTGATCAAATACCATTATTTGTTTGATCTGTTGGTATTACAGCATTACTTCTCCTCCTTTCTTTACCAGTATTAG
CAGGTGCTATTACTATACTATTAACTGACCGAAATTTAAATACATCTTTTTTTGACCCAGCTGGAGGAGGTGATCCAATT
TTATATCAACATTTATTTTGATTTTTT
>SCHIN051-18|Chilo_sacchariphagus|W17-CsZj2|
AACTTTATATTTTATTTTTGGAATTTGAGCTGGAATAGTTGGAACATCCCTTAGACTTTTAATTCGAGCTGAATTAGGAA
ATCCAGGTTCATTAATCGGAGATGATCAAATTTATAATACTATTGTTACAGCCCATGCATTTATTATAATTTTTTTTTTA
GTAATACCAATTATAATTGGAGGATTTGGAAATTGATTAGTTCCATTAATATTAGGGGCTCCTGATATAGCCTTCCCTCG
TCTAAATAATATAAGATTTTGATTATTACCCCCTTCTTTAACCCTTCTAATTTCTAGAAGAATCGTTGAAAATGGAGCAG
GAACTGGATGAACAGTCTACCCCCCCCTATCTTCCAATATTTCACATGCTGGAAGTTCAGTAGATTTAGCCATCTTCTCC
CTTCATTTAGCTGGAATTTCTTCAATTTTAGGAGCTATCAATTTCATTACTACAATTATTAATATACGAATTAATGGATT
ATTATTTGATCAAATACCATTATTTGTTTGATCTGTTGGTATTACAGCATTACTTCTCCTCCTTTCTTTACCAGTATTAG
CAGGTGCTATTACTATACTATTAACTGACCGAAATTTAAATACATCTTTTTTTGACCCAGCTGGAGGAGGCGATCCAATT
TTATATCAACATTTATCTTGATTTTTT
>SCHIN052-18|Chilo_sacchariphagus|W17-CsZj3|
AACTTTATATTTTATTTTTGGAATTTGAGCTGGAATAGTTGGAACATCCCTTAGACTTTTAATTCGAGCTGAATTAGGAA
ATCCAGGTTCATTAATCGGAGATGATCAAATTTATAATACTATTGTTACAGCCCATGCATTTATTATAATTTTTTTTATA
GTAATACCAATTATAATTGGAGGATTTGGAAATTGATTAGTTCCATTAATATTAGGGGCTCCTGATATAGCCTTCCCTCG
TCTAAATAATATAAGATTTTGATTATTACCCCCTTCTTTAACCCTTCTAATTTCTAGAAGAATCGTTGAAAATGGAGCAG
GAACTGGATGAACAGTCTACCCCCCCCTATCTTCCAATATTTCACATGCTGGAAGTTCAGTAGATTTAGCCATCTTCTCC
CTTCATTTAGCTGGAATTTCTTCAATTTTAGGAGCTATCAATTTCATTACTACAATTATTAATATACGAATTAATGGATT
ATTATTTGATCAAATACCATTATTTGTTTGATCTGTTGGTATTACAGCATTACTTCTCCTCCTTTCTTTACCAGTATTAG
CAGGTGCTATTACTATACTATTAACTGACCGAAATTTAAATACATCTTTTTTTGACCCAGCTGGAGGAGGTGATCCAATT
TTATATCAACATTTATTTTGATTTTTT
>SCHIN053-18|Chilo_sacchariphagus|W17-CsZj4|
AACTTTATATTTTATTTTTGGAATTTGAGCTGGAATAGTTGGAACATCCCTTAGACTTTTAATTCGAGCTGAATTAGGAA
ATCCAGGTTCATTAATCGGAGATGATCAAATTTATAATACTATTGTTACAGCCCATGCATTTATTATAATTTTTTTTATA
GTAATACCAATTATAATTGGAGGATTTGGAAATTGATTAGTTCCATTAATATTAGGGGCTCCTGATATAGCCTTCCCTCG

TCTAAATAATATAAGATTTTGATTATTACCCCCTTCTTTAACCCTTCTAATTTCTAGAAGAATCGTTGAAAATGGAGCAG
GAACTGGATGAACAGTCTACCCCCCCCTATCTTCCAATATTTCACATGCTGGAAGTTCAGTAGATTTAGCCATCTTCTCC
CTTCATTTAGCTGGAATTTCTTCAATTTTAGGAGCTATCAATTTCATTACTACAATTATTAATATACGAATTAATGGATT
ATTATTTGATCAAATACCATTATTTGTTTGATCTGTTGGTATTACAGCATTACTTCTCCTCCTTTCTTTACCAGTATTAG
CAGGTGCTATTACTATACTATTAACTGACCGAAATTTAAATACATCTTTTTTGACCCAGCTGGAGGAGGTGATCCAATT
TTATATCAACATTTATTTTGATTTTTT
>SCHIN054-18|Chilo_sacchariphagus|W17-CsZj5|
AACTTTATATTTTATTTTTGGAATTTGAGCTGGAATAGTTGGAACATCCCTTAGACTTTTAATTCGAGCTGAATTAGGAA
ATCCAGGTTCATTAATCGGAGATGATCAAATTTATAATACTATTGTTACAGCCCATGCATTTATTATAATTTTTTTTATA
GTAATACCAATTATAATTGGAGGATTTGGAAATTGATTAGTTCCATTAATATTAGGGGCTCCTGATATAGCCTTCCCTCG
TCTAAATAATATAAGATTTTGATTATTACCCCCTTCTTTAACCCTTCTAATTTCTAGAAGAATCGTTGAAAATGGAGCAG
GAACTGGATGAACAGTCTACCCCCCCCTATCTTCCAATATTTCACATGCTGGAAGTTCAGTAGATTTAGCCATCTTCTCC
CTTCATTTAGCTGGAATTTCTTCAATTTTAGGAGCTATCAATTTCATTACTACAATTATTAATATACGAATTAATGGATT
ATTATTTGATCAAATACCATTATTTGTTTGATCTGTTGGTATTACAGCATTACTTCTCCTCCTTTCTTTACCAGTATTAG
CAGGTGCTATTACTATACTATTAACTGACCGAAATTTAAATACATCTTTTTTGACCCAGCTGGAGGAGGTGATCCAATT
TTATATCAACATTTATTTTGATTTTTT
>SCHIN056-18|Scirpophaga_excerptalis|W17-SeGu1|
AACATTATATTTTATTTTTGGAATTTGAGCTGGTATAGTAGGGACTTCACTTAGTTTACTAATTCGAGCCGAACTAGGTA
CCCCTGGGTCATTAATTGGAGATGATCAAATCTATAATACTATTGTAACTGCTCACGCTTTTATTATAATTTTTTTTATG
GTTATACCTATTATAATTGGAGGATTCGGAAACTGATTAGTACCTTTAATATTGGGGGCCCCAGATATGGCTTTCCCCCG
AATAAATAATATAAGTTTTTGATTATTACCCCCTTCTTTAACCCTCTTAATCTCAAGAAGAATCGTTGAAAATGGAGCTG
GAACAGGATGAACTGTTTACCCGCCCCTATCCTCCAATATTGCCCACGGTGGGACTTCTGTAGATTTAGCCATTTTTTCA
CTACATTTAGCTGGAATTTCTTCTATTCTAGGGGCTATTAACTTCATTACAACTATTATTAATATGCGAATTAATGGACT
ATCTTTTGATCAAATACCTTTATTCGTGTGAGCAGTTGGTATTACTGCCCTTCTTCTTCTTCTCTCACTACCTGTATTAG
CGGGAGCTATTACTATATTATTAACAGATCGAAACTTAAATACCTCTTTCTTTGACCCAGCAGGAGGTGGAGACCCAATT
CTTTATCAACATTTATTTTGATTTTTT
>SCHIN057-18|Scirpophaga_excerptalis|W17-SeGu2|
AACATTATATTTTATTTTTGGAATTTGAGCTGGTATAGTAGGGACTTCACTTAGTTTACTAATTCGAGCCGAACTAGGTA
CCCCTGGGTCATTAATTGGAGATGATCAAATCTATAATACTATTGTAACTGCTCACGCTTTTATTATAATTTTTTTTATG
GTTATACCTATTATAATTGGAGGATTCGGAAACTGATTAGTACCTTTAATATTAGGGGCCCCAGATATGGCTTTCCCCCG
AATAAATAATATAAGTTTTTGATTATTACCCCCTTCTTTAACCCTCTTAATCTCAAGAAGAATCGTTGAAAATGGAGCTG
GAACAGGATGAACTGTTTACCCGCCCCTATCCTCCAATATTGCCCACGGTGGGACTTCTGTAGATTTAGCCATTTTTTCA
CTACATTTAGCTGGAATTTCTTCTATTCTAGGGGCTATTAACTTCATTACAACTATTATTAATATGCGAATTAATGGACT
ATCTTTTGATCAAATACCTTTATTCGTGTGAGCAGTTGGTATTACTGCCCTTCTTCTTCTTCTCTCACTACCTGTATTAG
CGGGAGCTATTACTATATTATTAACAGATCGAAACTTAAATACCTCTTTCTTTGACCCAGCAGGAGGTGGAGACCCAATT
CTTTATCAACATTTATTTTGATTTTTT
>SCHIN058-18|Scirpophaga_excerptalis|W17-SeGu3|
AACATTATATTTTATTTTTGGAATTTGAGCTGGTATAGTAGGGACTTCACTTAGTTTACTAATTCGAGCCGAACTAGGTA
CTCCTGGGTCATTAATTGGAGATGATCAAATCTATAATACTATTGTAACTGCTCACGCTTTTATTATAATTTTTTTTATA
GTTATACCTATTATAATTGGAGGATTCGGAAACTGATTAGTGCCTTTAATATTAGGGGCTCCAGATATAGCTTTCCCCCG
AATAAATAATATAAGTTTTTGATTATTACCCCCTTCTTTAACCCTCTTAATCTCAAGAAGAATCGTTGAAAATGGAGCTG
GAACAGGATGAACTGTTTACCCGCCCTTATCCTCCAATATTGCCCACAGTGGGACTTCTGTAGATTTAGCCATTTTTTCA
CTACATTTAGCTGGAATTTCTTCTATTCTAGGGGCTATCAACTTCATTACAACTATTATTAATATGCGAATTAATGGACT
ATCTTTTGATCAAATACCTTTATTCGTGTGAGCAGTTGGTATTACTGCTCTTCTTCTTCTTCTCTCACTACCTGTATTAG
CAGGAGTTATCACTATATTATTAACAGATCGAAACTTAAATACCTCTTTCTTTGATCCAGCAGGAGGTGGAGACCCAATT
CTTTATCAACATTTATTCTGATTTTTT
>SCHIN059-18|Scirpophaga_excerptalis|W17-SeGu5|
AACATTATATTTTATTTTTGGAATTTGAGCTGGTATAGTAGGGACTTCACTTAGTTTACTAATTCGAGCCGAATTAGGTA
CCCCTGGATCATTAATTGGAGATAATCAAATCTATAATACTATTGTAACTGCTCACGCTTTTATTATAATTTTTTTTATG
GTTATACCTATTATAATTGGAGGATTCGGAAACTGATTAGTGCCTTTAATATTAGGGGCTCCAGATATAGCTTTCCCCCG
AATAAATAATATAAGTTTTTGATTATTACCCCCTTCTTTAACCCTCTTAATCTCAAGAAGAATCGTTGAAAATGGAGCTG
GAACAGGATGAACTGTTTACCCGCCCTTATCCTCCAATATTGCCCACAGTGGGACTTCTGTAGATTTAGCCATTTTTTCA
CTACATTTAGCTGGAATTTCTTCTATTCTAGGGGCTATTAACTTCATTACAACTATTATTAATATGCGAATTAATGGACT
ATCTTTTGATCAAATACCTTTATTCGTGTGAGCAGTTGGTATTACTGCTCTTCTTCTTCTTCTCTCACTACCTGTATTAG
CAGGAGCTATCACTATATTATTAACAGATCGAAACTTAAATACCTCTTTCTTTGATCCAGCAGGAGGTGGAGACCCAATT
CTTTATCAACATTTATTCTGATTTTTT
>SCHIN060-18|Sesamia_inferens|W17-SiFu1|

AACATTATATTTTATTTTTGGAATTTGAGCTGGTATAGTAGGAACTTCATTAAGATTATTAATTCGAGCTGAATTAGGAA
TTCCTGGATCTTTAATTGGGGATGATCAAATTTATAACACTATTGTTACAGCTCATGCTTTTATTATAATTTTTTTTATA
GTTATACCAATTATAATTGGGGGATTTGGTAATTGACTTGTACCTTTAATATTAGGAGCTCCAGATATAGCATTCCCACG
AATAAATAATATAAGATTTTGATTATTACCCCCCTCTTTAACTTTATTAATTTCAAGTAGAATTGTAGAAAATGGGGCAG
GTACAGGATGAACAGTATATCCACCTCTCTCATCTAATATTGCCCATGGGGGAAGATCAGTAGACTTAGCTATTTTTTCT
CTTCATTTAGCGGGTATTTCATCTATTTTAGGAGCTATTAATTTTATTACAACAATTATTAATATACGATTAAATAGATT
ATCCTTTGATCAAATACCTTTATTTGTTTGAGCTGTTGGGATTACTGCATTTTTATTATTATTATCTTTACCTGTTTTAG
CGGGGGCTATTACAATGTTATTAACAGATCGAAACTTAAATACATCCTTCTTTGACCCTGCGGGAGGGGGTGATCCAATT
TTATATCAACATTTATTTTGATTTTTT
>SCHIN061-18|Sesamia_inferens|W17-SiFu2|
AACATTATATTTTATTTTTGGAATTTGAGCTGGTATAGTAGGAACTTCATTAAGATTATTAATTCGAGCTGAATTAGGAA
TTCCTGGATCTTTAATTGGGGATGATCAAATTTATAACACTATTGTTACAGCTCATGCTTTTATTATAATTTTTTTTATA
GTTATACCAATTATAATTGGGGGATTTGGTAATTGACTTGTACCTTTAATATTAGGAGCTCCAGATATAGCATTCCCACG
AATAAATAATATAAGATTTTGATTATTACCCCCCTCCTTAACTTTATTAATTTCAAGTAGAATTGTAGAAAATGGGGCAG
GTACAGGATGAACAGTATATCCACCTCTCTCATCTAATATTGCCCATGGGGGAAGATCAGTAGACTTAGCTATTTTTTCT
CTTCATTTAGCGGGTATTTCATCTATTTTAGGAGCTATTAATTTTATTACAACAATTATTAATATACGATTAAATAGATT
ATCCTTTGATCAAATACCTTTATTTGTTTGAGCTGTTGGGATTACTGCATTTTTATTATTATTATCTTTACCTGTTTTAG
CGGGGGCTATTACAATGTTATTAACAGATCGAAACTTAAATACATCCTTCTTTGACCCTGCGGGAGGGGGTGATCCAATT
TTATATCAACATTTATTTTGATTTTTT
>SCHIN062-18|Sesamia_inferens|W17-SiFu3|
AACATTATATTTTATTTTTGGAATTTGAGCTGGTATAGTAGGAACTTCATTAAGATTATTAATTCGAGCTGAATTAGGAA
TTCCTGGATCTTTAATTGGGGATGATCAAATTTATAACACTATTGTTACAGCTCATGCTTTTATTATAATTTTTTTTATA
GTTATACCAATTATAATTGGGGGATTTGGTAATTGACTTGTACCTTTAATATTAGGAGCTCCAGATATAGCATTCCCACG
AATAAATAATATAAGATTTTGATTATTACCCCCCTCTTTAACTTTATTAATTTCAAGTAGAATTGTAGAAAATGGGGCAG
GTACAGGATGAACAGTATATCCACCTCTCTCATCTAATATTGCCCATGGGGGAAGATCAGTAGACTTAGCTATTTTTTCT
CTTCATTTAGCGGGTATTTCATCTATTTTAGGAGCTATTAATTTTATTACAACAATTATTAATATACGATTAAATAGATT
ATCCTTTGATCAAATACCTTTATTTGTTTGAGCTGTTGGGATTACTGCATTTTTATTATTATTATCTTTACCTGTTTTAG
CGGGGGCTATTACAATGTTATTAACAGATCGAAACTTAAATACATCCTTCTTTGACCCTGCGGGAGGGGGTGATCCAATT
TTATATCAACATTTATTTTGATTTTTT
>SCHIN063-18|Sesamia_inferens|W17-SiFu4|
AACATTATATTTTATTTTTGGAATTTGAGCTGGTATAGTAGGAACTTCATTAAGATTATTAATTCGAGCTGAATTAGGAA
TTCCTGGATCTTTAATTGGGGATGATCAAATTTATAACACTATTGTTACAGCTCATGCTTTTATTATAATTTTTTTTATA
GTTATACCAATTATAATTGGGGGATTTGGTAATTGACTTGTACCTTTAATATTAGGAGCTCCAGATATAGCATTCCCACG
AATAAATAATATAAGATTTTGATTATTACCCCCCTCCTTAACTTTATTAATTTCAAGTAGAATTGTAGAAAATGGGGCAG
GTACAGGATGAACAGTATATCCACCTCTCTCATCTAATATTGCCCATGGGGGAAGATCAGTAGACTTAGCTATTTTTTCT
CTTCATTTAGCGGGTATTTCATCTATTTTAGGAGCTATTAATTTTATTACAACAATTATTAATATACGATTAAATAGATT
ATCCTTTGATCAAATACCTTTATTTGTTTGAGCTGTTGGGATTACTGCATTTTTATTATTATTATCTTTACCTGTTTTAG
CGGGGGCTATTACAATGTTATTAACAGATCGAAACTTAAATACATCCTTCTTTGACCCTGCGGGAGGGGGTGATCCAATT
TTATATCAACATTTATTTTGATTTTTT
>SCHIN064-18|Sesamia_inferens|W17-SiFu5|
AACATTATATTTTATTTTTGGAATTTGAGCTGGTATAGTAGGAACTTCATTAAGATTATTAATTCGAGCTGAATTAGGAA
TTCCTGGATCTTTAATTGGGGATGATCAAATTTATAACACTATTGTTACAGCTCATGCTTTTATTATAATTTTTTTTATA
GTTATACCAATTATAATTGGGGGATTTGGTAATTGACTTGTACCTTTAATATTAGGAGCTCCAGATATAGCATTCCCACG
AATAAATAATATAAGATTTTGATTATTACCCCCCTCCTTAACTTTATTAATTTCAAGTAGAATTGTAGAAAATGGGGCAG
GTACAGGATGAACAGTATATCCACCTCTCTCATCTAATATTGCCCATGGGGGAAGATCAGTAGACTTAGCTATTTTTTCT
CTACATTTAGCGGGTATTTCATCTATTTTAGGAGCTATTAATTTTATTACAACAATTATTAATATACGATTAAATAGATT
ATCCTTTGATCAAATACCTTTATTTGTTTGAGCTGTTGGGATTACTGCATTTTTATTATTATTATCTTTACCTGTTTTAG
CGGGGGCTATTACAATGTTATTAACAGATCGAAACTTAAATACATCCTTCTTTGACCCTGCGGGAGGGGGTGATCCAATT
TTATATCAACATTTATTTTGATTTTTT
>SCHIN065-18|Sesamia_inferens|W17-SiYa1|
AACATTATATTTTATTTTTGGAATTTGAGCTGGTATAGTAGGAACTTCATTAAGATTATTAATTCGAGCTGAATTAGGAA
TTCCTGGATCTTTAATTGGGGATGATCAAATTTATAACACTATTGTTACAGCTCATGCTTTTATTATAATTTTTTTTATA
GTTATACCAATTATAATTGGGGGATTTGGTAATTGACTTGTACCTTTAATATTAGGAGCTCCAGATATAGCATTCCCACG
AATAAATAATATAAGATTTTGATTATTACCCCCCTCCTTAACTTTATTAATTTCAAGTAGAATCGTAGAAAATGGGGCAG
GTACAGGATGAACAGTATATCCACCTCTCTCATCTAATATTGCCCATGGGGGAAGATCAGTAGACTTAGCTATTTTTTCT
CTTCATTTAGCGGGTATTTCATCTATTTTAGGAGCTATTAATTTTATTACAACAATTATTAATATACGATTAAATAGATT
ATCCTTTGATCAAATACCTTTATTTGTTTGAGCTGTTGGGATTACTGCATTTTTATTATTATTATCTTTACCTGTTTTAG

CGGGGGCTATTACAATGTTATTAACAGATCGAAACTTAAATACATCCTTCTTTGACCCTGCGGGAGGGGGTGATCCAATT
TTATATCAACATTTATTTTGATTTTTT
>SCHIN066-18|Sesamia_inferens|W17-SiYa2|
AACATTATATTTTATTTTTGGAATTTGAGCTGGTATAGTAGGAACTTCATTAAGATTATTAATTCGAGCTGAATTAGGAA
TTCCTGGATCTTTAATTGGGGATGATCAAATTTATAACACTATTGTTACAGCTCATGCTTTTATTATAATTTTTTTTATA
GTTATACCAATTATAATTGGGGGATTTGGTAATTGACTTGTACCTTTAATATTAGGAGCTCCAGATATAGCATTCCCACG
AATAAATAATATAAGATTTTGATTATTACCCCCCTCCTTAACTTTATTAATTTCAAGTAGAATTGTAGAAAATGGGGCAG
GTACAGGATGAACAGTATATCCACCTCTCTCATCTAATATTGCCCATGGGGGAAGATCAGTAGACTTAGCTATTTTTTCT
CTTCATTTAGCGGGTATTTCATCTATTTTAGGAGCTATTAATTTTATTACAACAATTATTAATATACGATTAAATAGATT
ATCCTTTGATCAAATACCTTTATTTGTTTGAGCTGTTGGGATTACTGCATTTTTATTATTATTATCTTTACCTGTTTTAG
CGGGGGCTATTACAATGTTATTAACAGATCGAAACTTAAATACATCCTTCTTTGACCCTGCGGGAGGGGGTGATCCAATT
TTATATCAACATTTATTTTGATTTTTT
>SCHIN067-18|Sesamia_inferens|W17-SiYa3|
AACATTATATTTTATTTTTGGAATTTGAGCTGGTATAGTAGGAACTTCATTAAGATTATTAATTCGAGCTGAATTAGGAA
TTCCTGGATCTTTAATTGGGGATGATCAAATTTATAACACTATTGTTACAGCTCATGCTTTTATTATAATTTTTTTTATA
GTTATACCAATTATAATTGGGGGATTTGGTAATTGACTTGTACCTTTAATATTAGGAGCTCCAGATATAGCATTCCCACG
AATAAATAATATAAGATTTTGATTATTACCCCCCTCCTTAACTTTATTAATTTCAAGTAGAATCGTAGAAAATGGGGCAG
GTACAGGATGAACAGTATATCCACCTCTCTCATCTAATATTGCCCATGGGGGAAGATCAGTAGACTTAGCTATTTTTTCT
CTTCATTTAGCGGGTATTTCATCTATTTTAGGAGCTATTAATTTTATTACAACAATTATTAATATACGATTAAATAGATT
ATCCTTTGATCAAATACCTTTATTTGTTTGAGCTGTTGGGATTACTGCATTTTTATTATTATTATCTTTACCTGTTTTAG
CGGGGGCTATTACAATGTTATTAACAGATCGAAACTTAAATACATCCTTCTTTGACCCTGCGGGAGGGGGTGATCCAATT
TTATATCAACATTTATTTTGATTTTTT
>SCHIN068-18|Sesamia_inferens|W17-SiYa4|
AACATTATATTTTATTTTTGGAATTTGAGCTGGTATAGTAGGAACTTCATTAAGATTATTAATCCGAGCTGAATTAGGAA
TTCCTGGATCTTTAATTGGGGATGATCAAATTTATAACACTATTGTTACAGCTCATGCTTTTATTATAATTTTTTTTATA
GTTATACCAATTATAATTGGGGGATTTGGTAATTGACTTGTACCTTTAATATTAGGAGCTCCAGATATAGCATTCCCACG
AATAAATAATATAAGATTTTGATTATTACCCCCCTCCTTAACTTTATTAATTTCAAGTAGAATTGTAGAAAATGGGGCAG
GTACAGGATGAACAGTATATCCACCTCTCTCATCTAATATTGCCCATGGGGGAAGATCAGTAGACTTAGCTATTTTTTCT
CTTCATTTAGCGGGTATTTCATCTATTTTAGGAGCTATTAATTTTATTACAACAATTATTAATATACGATTAAATAGATT
ATCCTTTGATCAAATACCTTTATTTGTTTGAGCTGTTGGGATTACTGCATTTTTATTATTATTATCTTTACCTGTTTTAG
CGGGGGCTATTACAATGTTATTAACAGATCGAAACTTAAATACATCCTTCTTTGACCCTGCGGGAGGGGGTGATCCAATT
TTATATCAACATTTATTTTGATTTTTT
>SCHIN069-18|Sesamia_inferens|W17-SiYa5|
AACATTATATTTTATTTTTGGAATTTGAGCTGGTATAGTAGGAACTTCATTAAGATTATTAATTCGAGCTGAATTAGGAA
TTCCTGGATCTTTAATTGGGGATGATCAAATTTATAACACTATTGTTACAGCTCATGCTTTTATTATAATTTTTTTTATA
GTTATACCAATTATAATTGGGGGATTTGGTAATTGACTTGTACCTTTAATATTAGGAGCTCCAGATATAGCATTCCCACG
AATAAATAATATAAGATTTTGATTATTACCCCCCTCCTTAACTTTATTAATTTCAAGTAGAATCGTAGAAAATGGGGCAG
GTACAGGATGAACAGTATATCCACCTCTCTCATCTAATATTGCCCATGGGGGAAGATCAGTAGACTTAGCTATTTTTTCT
CTTCATTTAGCGGGTATTTCATCTATTTTAGGAGCTATTAATTTTATTACAACAATTATTAATATACGATTAAATAGATT
ATCCTTTGATCAAATACCTTTATTTGTTTGAGCTGTTGGGATTACTGCATTTTTATTATTATTATCTTTACCTGTTTTAG
CGGGGGCTATTACAATGTTATTAACAGATCGAAACTTAAATACATCCTTCTTTGACCCTGCGGGAGGGGGTGATCCAATT
TTATATCAACATTTATTTTGATTTTTT
>SCHIN070-18|Tetramoera_schistaceana|W17-TsDe1|
AACATTATATTTTATTTTTGGAATTTGAGCCGGAATAATTGGAACATCTCTAAGATTATTAATTCGAGCAGAATTAGGAA
ATCCTGGCTCTTTAATTGGAGATGATCAAATTTATAATACTATTGTAACTGCTCATGCTTTTATTATAATTTTTTTCATA
GTTATACCTATCATAATTGGAGGATTTGGAAATTGATTAGTACCATTAATATTAGGAGCCCCTGATATAGCTTTTCCTCG
TATAAATAATATAAGATTTTGATTACTCCCCCCTTCTATTATATTATTAATTTCAAGAAGAATTGTAGAAAATGGAGCAG
GAACAGGATGAACAGTTTACCCCCCCCTTTCATCTAATATTGCCCATAGAGGTAGATCAGTAGATCTAGCTATTTTTTCT
TTACATTTAGCTGGAATTTCTTCTATTTTAGGAGCTGTAAACTTTATTACAACTATTATTAATATACGACCAAATAATAT
AAGATTAGATCAAATACCCCTATTTGTTTGAGCTGTTGGCATTACAGCTCTTCTTTTATTATTATCTTTACCAGTATTAG
CAGGAGCTATTACTATACTCTTAACAGACCGTAATTTAAATACTTCATTTTTTGATCCTGCTGGTGGAGGAGATCCAATT
TTATACCAACACTTATTTTGATTTTTT
>SCHIN071-18|Tetramoera_schistaceana|W17-TsDe2|
AACATTATATTTTATTTTTGGAATTTGAGCCGGAATAATTGGAACATCTCTAAGATTATTAATTCGAGCAGAATTAGGAA
ATCCTGGCTCTTTAATTGGAGATGATCAAATTTATAATACTATTGTAACTGCTCATGCTTTTATTATAATTTTTTTCATA
GTTATACCTATCATAATTGGAGGATTTGGAAATTGATTAGTACCATTAATATTAGGAGCCCCTGATATAGCTTTTCCTCG
TATAAATAATATAAGATTTTGATTACTCCCCCCTTCTATTATATTATTAATTTCAAGAAGAATTGTAGAAAATGGAGCAG

GAACAGGATGAACAGTTTATCCCCCCCTTTCATCTAATATTGCCCATAGAGGTAGATCAGTAGATCTAGCTATTTTTTCT
TTACATTTAGCTGGAATTTCTTCTATTTTAGGAGCTGTAAACTTTATTACAACTATTATTAATATACGACCAAATAATAT
AAGATTAGATCAAATACCCCTATTTGTTTGAGCTGTTGGCATTACAGCTCTTCTTTTATTATTATCTTTACCAGTATTAG
CAGGAGCTATTACTATACTCTTAACAGACCGTAATTTAAATACTTCATTTTTTGATCCTGCTGGTGGAGGAGATCCAATT
TTATACCAACACTTATTTTGATTTTTT
>SCHIN072-18|Tetramoera_schistaceana|W17-TsDe3|
AACATTATATTTTATTTTTGGAATTTGAGCCGGAATAATTGGAACATCTCTAAGATTATTAATTCGAGCAGAATTAGGAA
ATCCTGGCTCTTTAATTGGAGATGATCAAATTTATAATACTATTGTAACTGCTCATGCTTTTATTATAATTTTTTTCATA
GTTATACCTATCATAATTGGAGGATTTGGAAATTGATTAGTACCATTAATATTAGGAGCCCCTGATATAGCTTTTCCTCG
TATAAATAATATAAGATTTTGATTACTCCCCCCTTCTATTATATTATTAATTTCAAGAAGAATTGTAGAAAATGGAGCAG
GAACAGGATGAACAGTTTACCCCCCCCTTTCATCTAATATTGCCCATAGAGGTAGATCAGTAGATCTAGCTATTTTTTCT
TTACATTTAGCTGGAATTTCTTCTATTTTAGGAGCTGTAAACTTTATTACAACTATTATTAATATACGACCAAATAATAT
AAGATTAGATCAAATACCCCTATTTGTTTGAGCTGTTGGCATTACAGCTCTTCTTTTATTATTATCTTTACCAGTATTAG
CAGGAGCTATTACTATACTCTTAACAGACCGTAATTTAAATACTTCATTTTTTGATCCTGCTGGTGGAGGAGATCCAATT
TTATACCAACACTTATTTTGATTTTTT
>SCHIN073-18|Tetramoera_schistaceana|W17-TsDe4|
AACATTATATTTTATTTTTGGAATTTGAGCCGGAATAATTGGAACATCTCTAAGATTATTAATTCGAGCAGAATTAGGAA
ATCCTGGCTCTTTAATTGGTGATGATCAAATTTATAATACTATTGTAACTGCTCATGCTTTTATTATAATTTTTTTCATA
GTTATACCTATCATAATTGGAGGATTTGGAAATTGATTAGTACCATTAATATTAGGAGCCCCTGATATAGCTTTTCCTCG
TATAAATAATATAAGATTTTGATTACTCCCCCCTTCTATTATATTATTAATTTCAAGAAGAATTGTAGAAAATGGAGCAG
GAACAGGATGAACAGTTTATCCCCCCCTTTCATCTAATATTGCCCATAGAGGTAGATCAGTAGATCTAGCTATTTTTTCT
TTACATTTAGCTGGAATTTCTTCTATTTTAGGAGCTGTAAACTTTATTACAACTATTATTAATATACGACCAAATAATAT
AAGATTAGATCAAATACCCCTATTTGTTTGAGCTGTTGGCATTACAGCTCTTCTTTTATTATTATCTTTACCAGTATTAG
CAGGAGCTATTACTATACTTTTAACAGACCGTAATTTAAATACTTCATTTTTTGATCCTGCTGGTGGAGGAGATCCAATT
TTATACCAACACTTATTTTGATTTTTT
>SCHIN074-18|Tetramoera_schistaceana|W17-TsDe5|
AACATTATATTTTATTTTTGGAATTTGAGCCGGAATAATTGGAACATCTCTAAGATTATTAATTCGAGCAGAATTAGGAA
ATCCTGGCTCTTTAATTGGTGATGATCAAATTTATAATACTATTGTAACTGCTCATGCTTTTATTATAATTTTTTTCATA
GTTATACCTATCATAATTGGAGGATTTGGAAATTGATTAGTACCATTAATATTAGGAGCCCCTGATATAGCTTTTCCTCG
TATAAATAATATAAGATTTTGATTACTCCCCCCTTCTATTATATTATTAATTTCAAGAAGAATTGTAGAAAATGGAGCAG
GAACAGGATGAACAGTTTATCCCCCCCTTTCATCTAATATTGCCCATAGAGGTAGATCAGTAGATCTAGCTATTTTTTCT
TTACATTTAGCTGGAATTTCTTCTATTTTAGGAGCTGTAAACTTTATTACAACTATTATTAATATACGACCAAATAATAT
AAGATTAGATCAAATACCCCTATTTGTTTGAGCTGTTGGCATTACAGCTCTTCTTTTATTATTATCTTTACCAGTATTAG
CAGGAGCTATTACTATACTTTTAACAGACCGTAATTTAAATACTTCATTTTTTGATCCTGCTGGTGGAGGAGATCCAATT
TTATACCAACACTTATTTTGATTTTTT-
>WALPA4489-13|Scirpophaga_nivella|BIOUG05072-B12|
-ACTTTATATTTCATTTTTGGAATTTGAGCTGGTATAGTAGGAACTTCTTTAAGATTATTAATTCGAGCTGAATTAGGAA
CTCCAGGATCTTTAATTGGAAATGATCAAATTTATAATACCATTGTTACAGCTCATGCTTTTATTATAATTTTTTTTATA
GTAATACCAATTATAATTGGAGGATTTGGAAATTGACTTGTTCCTTTAATATTAGGAGCTCCTGATATAGCTTTCCCCCG
TATAAATAATATAAGATTTTGATTATTACCTCCCTCATTAACTCTACTAATTTCAAGAAGAATTGTAGAAAATGGTGCAG
GAACAGGATGAACAGTATACCCTCCCCTATCATCAAATATTGCTCATGGGGGAACTTCTGTAGATTTAGCTATTTTCTCT
TTACATCTTGCAGGAATTTCCTCTATTTTAGGAGCTATTAACTTTATTACCACTATTATTAATATACGAATTAATGGCTT
AACATTTGATCAAATACCCCTCTTTGTTTGAGCTGTTGGAATTACAGCCCTTCTTTTACTCCTCTCATTACCTGTATTAG
CTGGAGCTATTACTATATTATTAACTGATCGAAATTTAAATACCTCTTTTTTTGATCCAGCAGGAGGAGGA---------
----
>WALPB112-13|Acrapex_exsanguis|BIOUG08827-B05|
-ACATTATATTTTATTTTTGGAATTTGAGCTGGTATACTAGGAACTTCTTTAAGTTTATTAATCCGAGCTGAATTAGGAA
CTCCAGAATCTTTAATTGGAGATGATCAAATTTATAATACTATTGTTACTGCTCACGCTTTTATTATAATTTCTTTATA
GTTATACCAATTATAATTGGAGGATTTGGAAATTGACTTGTTCCATTAATACTAGGAGCTCCAGATATAGCATTTCCACG
TATAAATAATATAAGATTTTGATTATTACCTCCCTCTTTAAGTTTATTAATTTCAAGAAGAATTGTAGAAAATGGAGCAG
GAACTGGATGAACAGTATATCCTCCACTCTCATCTAATATTGCTCATAGAGGAAGATCAGTAGATTTAGCTATTTTTTCT
CTTCATTTAGCTGGTATTTCATCTATTTTAGGAGCTATTAATTTTATTACAACAATTATTAATATACGATTAAATAATTT
ATCTTTTGATCAAATACCTTTATTTGTTTGAGCTGTTGGAATTACTGCATTTTTACTATTACTTTCATTACCTGTATTAG
CCGGAGCTATTACAATATTATTAACAGATCGA--

>WALPB396-13|Scirpophaga_percna|BIOUG08996-B04|
AACTTTATATTTTATTTTTGGAATTTGAGCTGGAATAGTAGGAACTTCTTTAAGTTTACTAATTCGAGCTGAATTAGGAA

CCCCAGAATCATTAATTGGAGATGATCAAATTTATAACACTATTGTAACAGCCCATGCTTTTATTATAATTTTTTTTATA
GTTATACCTATTATAATTGGGGGTTTTGGAAATTGACTAGTTCCTCTAATATTAGGAGCTCCTGATATAGCTTTCCCCCG
ATTAAATAATATAAGTTTTTGATTATTACCTCCCTCTCTTACCCTTTTAATTTCAAGAAGAATTGTTGAAAATGGAGCAG
GAACAGGATGAACTGTTTACCCCCCACTATCCTCTAACATCGCCCATGGGGGTACATCAGTAGATTTAGCTATTTTCTCC
CTTCATTTAGCTGGAATCTCATCTATTCTAGGAGCTATTAATTTTATTACTACTATTATCAATATACGAATTAATGGACT
ATCTTTTGATCAAATACCTTTATTTGTTTGAGCTGTAGGAATTACAGCTCTTCTTTTACTTTTATCTTTACCAGTACTTG
CCGGAGCTATTACTATATTACTAACTGATCGAAATTTAAATACTTCTTTCTTTGACCCTGCTGGAGGAGGAGATCCAATT
CTTTATCAACATTTATTC---------
>WALPB441-13|Acrapex_exsanguis|BIOUG08996-F01|
-ACATTATATTTTATTTTTGGAATTTGAGCTGGTATACTAGGAACTTCTTTAAGTTTATTAATCCGAGCTGAATTAGGAA
CTCCAGAATCTTTAATTGGAGATGATCAAATTTATAATACTATTGTTACTGCTCACGCTTTTATTATAATTTTCTTTATA
GTTATACCAATTATAATTGGAGGATTTGGAAATTGACTTGTTCCATTAATACTAGGAGCTCCAGATATAGCATTTCCACG
TATAAATAATATAAGATTTTGATTATTACCTCCCTCTTTAAGTTTATTAATTTCAAGAAGAATTGTAGAAAATGGAGCAG
GAACTGGATGAACAGTATATCCTCCACTCTCATCTAATATTGCTCATAGAGGAAGATCAGTAGATTTAGCTATTTTTTCT
CTTCATTTAGCTGGTATTTCATCTATTTTAGGAGCTATTAATTTTATTACAACAATTATTAATATACGATTAAATAATTT
ATCTTTTGATCAAATACCTTTATTTGTTTGAGCTGTTGGAATTACTGCATTTTTACTATTACTTTCATTACCTGTATTAG
CCGGAGCTATTACAATATTATTAACAGAT--

>WALPB442-13|Acrapex_exsanguis|BIOUG08996-F02|
----------TTTATTTTTGGAATTTGAGCTGGTATACTAGGAACTTCTTTAAGTTTATTAATCCGAGCTGAATTAGGAA
CTCCAGAATCTTTAATTGGAGATGATCAAATTTATAATACTATTGTTACTGCTCACGCTTTTATTATAATTTTCTTTATA
GTTATACCAATTATAATTGGAGGATTTGGAAATTGACTTGTTCCATTAATACTAGGAGCTCCAGATATAGCATTTCCACG
TATAAATAATATAAGATTTTGATTATTACCTCCCTCTTTAAGTTTATTAATTTCAAGAAGAATTGTAGAAAATGGAGCAG
GAACTGGATGAACAGTATATCCTCCACTCTCATCTAATATTGCTCATAGAGGAAGATCAGTAGATTTAGCTATTTTTTCT
CTTCATTTAGCTGGTATTTCATCTATTTTAGGAGCTATTAATTTTATTACAACAATTATTAATATACGATTAAATAATTT
ATCTTTTGATCAAATACCTTTATTTGTTTGAGCTGTTGGAATTACTGCATTTTTACTATTACTTTCATTACCTGTATTAG
CC--

>WALPB443-13|Acrapex_exsanguis|BIOUG08996-F03|
-ACATTATATTTTATTTTTGGAATTTGAGCTGGTATACTAGGAACTTCTTTAAGTTTATTAATCCGAGCTGAATTAGGAA
CTCCAGAATCTTTAATTGGAGATGATCAAATTTATAATACTATTGTTACTGCTCACGCTTTTATTATAATTTTCTTTATA
GTTATACCAATTATAATTGGAGGATTTGGAAATTGACTTGTCCCATTAATACTAGGAGCTCCAGATATAGCATTTCCACG
TATAAATAATATAAGATTTTGATTATTACCTCCCTCTTTAAGTTTATTAATTTCAAGAAGAATTGTAGAAAATGGAGCAG
GAACTGGATGAACAGTATATCCTCCACTCTCATCTAATATTGCTCATAGAGGAAGATCAGTAGATTTAGCTATTTTTTCT
CTTCATTTAGCTGGTATTTCATCTATTTTAGGAGCTATTAATTTTATTACAACAATTATTAATATACGATTAAATAATTT
ATCTTTTGATCAAATACCTTTATTTGTTTGAGCTGTTGGAATTACTGCATTTTTACTATTACTCTCATTACCTGTATTAG
CC--

>WALPC466-14|Scirpophaga_percna|BIOUG17341-H02|
----------TTTATTTTTGGAATTTGAGCTGGAATAGTAGGAACTTCTTTAAGTTTACTAATTCGAGCTGAATTAGGAA
CCCCAGAATCATTAATTGGAGATGATCAAATTTATAACACTATTGTAACAGCCCATGCTTTTATTATAATTTTTTTTATA
GTTATACCTATTATAATTGGGGGTTTTGGAAATTGACTAGTTCCTCTAATATTAGGAGCTCCTGATATAGCTTTCCCCCG
ATTAAATAATATAAGTTTTTGATTATTACCTCCCTCTCTTACCCTTTTAATTTCAAGAAGAATTGTTGAAAATGGAGCAG
GAACAGGATGAACTGTTTACCCCCCACTATCCTCTAACATCGCCCATGGGGGTACATCAGTAGATTTAGCTATTTTCTCC
CTTCATTTAGCTGGAATCTCATCTATTCTAGGAGCTATTAATTTTATTACTACTATTATCAATATACGAATTAATGGACT
ATCTTTTGATCAAATACCTTTATTTGTTTGAGCTGTAGGAATTACAGCTCTTCTTTTACTTTTATCTTTACCAGTACTTG
CCGGAGCTATTACTATATTACTAACTGAT--

>WALPC494-14|Scirpophaga_percna|BIOUG17342-B07|
-ACTTTATATTTTATTTTTGGAATTTGAGCTGGAATAGTAGGAACTTCTTTAAGTTTACTAATTCGAGCTGAATTAGGAA
CCCCAGAATCATTAATTGGAGATGATCAAATTTATAACACTATTGTAACAGCCCATGCTTTTATTATAATTTTTTTTATA
GTTATACCTATTATAATTGGGGGTTTTGGAAATTGACTAGTTCCTCTAATATTAGGAGCTCCTGATATAGCTTTCCCTCG
ATTAAATAATATAAGTTTTTGATTATTACCTCCCTCTCTTACCCTTTTAATTTCAAGAAGAATTGTTGAAAATGGAGCAG
GAACAGGATGAACTGTTTACCCCCCACTATCCTCTAACATCGCCCATGGGGGTACATCAGTAGATTTAGCTATTTTCTCC
CTTCATTTAGCTGGAATCTCATCTATTCTAGGAGCTATTAATTTTATTACTACTATTATCAATATACGAATTAATGGACT
ATCTTTTGATCAAATACCTTTATTTGTTTGAGCTGTAGGAATTACAGCTCTTCTTTTACTTTTATCTTTACCAGTACTTG
CCGGAGCTATTACTATATTACTAACTGATCGAAATTTAAAT---------------------------------------

----
>WALPC497-14|Scirpophaga_percna|BIOUG17342-B10|
-ACTTTATATTTTATTTTTGGAATTTGAGCTGGAATAGTAGGAACTTCTTTAAGTTTACTAATTCGAGCTGAATTAGGAA
CCCCAGAATCATTAATTGGAGATGATCAAATTTATAACACTATTGTAACAGCCCATGCTTTTATTATAATTTTTTTTATA
GTTATACCTATTATAATTGGAGGTTTTGGAAATTGACTAGTTCCTCTAATATTAGGAGCTCCTGATATAGCTTTCCCCCG
ATTAAATAATATAAGTTTTTGATTATTACCTCCCTCTCTTACCCTTTTAATTTCAAGAAGAATTGTTGAAAATGGAGCAG
GAACAGGATGAACTGTTTACCCCCCACTATCCTCTAACATCGCCCATGGGGGTACATCAGTAGATTTAGCTATTTTCTCC
CTTCATTTAGCTGGAATCTCATCTATTCTAGGAGCTATTAATTTTATTACTACTATTATCAATATACGAATTAATGGACT
ATCTTTTGATCAAATACCTTTATTTGTTTGAGCTGTAGGAATTACAGCTCTTCTTTTACTTTTATCTTTACCAGTACTTG
CCGGAGCTATTACTATATTACTAACTGATCGAAATTTAAAT---------------------------------------
----
>WALPC1750-14|Scirpophaga_percna|BIOUG18087-H10|
AACTTTATATTTTATTTTTGGAATTTGAGCTGGAATAGTAGGAACTTCTTTAAGTTTACTAATTCGAGCTGAATTAGGAA
CCCCAGAATCATTAATTGGAGATGATCAAATTTATAACACTATTGTAACAGCCCATGCTTTTATTATAATTTTTTTTATA
GTTATACCTATTATAATTGGAGGTTTTGGAAATTGACTAGTTCCTCTAATATTAGGAGCTCCTGATATAGCTTTCCCCCG
ATTAAATAATATAAGTTTTTGATTATTACCTCCCTCTCTTACCCTTTTAATTTCAAGAAGAATTGTTGAAAATGGAGCAG
GAACAGGATGAACTGTTTACCCCCCACTATCCTCTAACATCGCCCATGGGGGTACATCAGTAGATTTAGCTATTTTCTCC
CTTCATTTAGCTGGAATCTCATCTATTCTAGGAGCTATTAATTTTATTACTACTATTATCAATATACGAATTAATGGACT
ATCTTTTGATCAAATACCTTTATTTGTTTGAGCTGTAGGAATTACAGCTCTTCTTTTACTTTTATCTTTACCAGTACTTG
CCGGAGCTATTACTATATTACTAACTGATCGAAATTTAAATACTTCTTTCTTTGACCCTG--------------------
----
>WALPC5319-15|Acrapex_exsanguis|BIOUG19955-H08|
---TTATTAATCCGAGCTGAATTAGGAA
CTCCAGAATCTTTAATTGGAGATGATCAAATTTATAATACTATTGTTACTGCTCACGCTTTTATTATAATTTTCTTTATA
GTTATACCAATTATAATTGGAGGATTTGGAAATTGACTTGTCCCATTAATACTAGGAGCTCCAGATATAGCATTTCCACG
TATAAATAATATAAGATTTTGATTATTACCTCCCTCTTTAAGTTTATTAATTTCAAGAAGAATTGTAGAAAATGGAGCAG
GAACTGGATGAACAGTATATCCTCCACTCTCATCTAATATTGCTCATAGAGGAAGATCAGTAGATTTAGCTATTTTTTCT
CTTCATTTAGCTGGTATTTCATCTATTTTAGGAGCTATTAATTTTATTACAACAATTATTAATATACGATTAAATAATTT
ATCTTTTGATCAAATACCTTTATTTGTTTGAGCTGTTGGAATTACTGCATTTTTACTATTACTCTCATTACCTGTATTAG
CCGGAGCTATTACAATATTATTAACAGATCGA---

Towards a global DNA barcode reference library for quarantine identifications of lepidopteran stemborers, with an emphasis on sugarcane pests

Timothy R. C. Lee, Stacey J. Anderson, Lucy T. T. Tran-Nguyen, Nader Sallam, Bruno P. Le Ru, Desmond Conlong, Kevin Powell, Andrew Ward, Andrew Mitchell

Supplementary Table 1 - Summary of agreement among the twelve species delimitation methods applied to the Chilo, Sesamia and Scirpophaga subtrees of the haplotypes dataset. MATCH = delimitation agrees with current taxonomy, MERGE = taxon groups with one or more other species, SPLIT = taxon split into multiple specie, COMPLEX = taxon split and at least one partition merged with another species. High congruence lists the delimitation of taxa, where all twelve delimitation methods agreed, or where all but one agreed (these appear in brackets). The seven most economically significant species to Australia are in bold and underlined; medium priority species are underlined but not in bold.

					SUBTREES		
Genus	species	MATCHES	singles	MERGES	SPLITS	COMPLEXES	High congruence
Chilo	***auricilius***	3	0	0	9	0	
Chilo	*crossostichus*	9	0	3	0	0	
Chilo	*crypsimetalla*	1	0	0	11	0	(Split)
Chilo	*demotellus*	0	**12**	0	0	0	SINGLE
Chilo	*diffusilineus*	0	**12**	0	0	0	SINGLE
Chilo	***infuscatellus***	4	0	0	8	0	
Chilo	*luteellus*	**12**	0	0	0	0	MATCH
Chilo	*orichalcociliellus*	2	0	8	2	0	
Chilo	*partellus*	9	0	0	3	0	
Chilo	*phragmitella*	3	0	0	9	0	
Chilo	*plejadellus*	**12**	0	0	0	0	MATCH
Chilo	*polychrysus*	0	**12**	0	0	0	SINGLE
Chilo	*quirimbellus*	3	0	5	4	0	
Chilo	***sacchariphagus***	3	0	0	9	0	
Chilo	*suppressalis*	**12**	0	0	0	0	MATCH
Chilo	***terrenellus***	**12**	0	0	0	0	MATCH
Chilo	*thyrsis*	5	0	7	0	0	
Chilo	*tumidicostalis*	**12**	0	0	0	0	MATCH
Scirpophaga	*excerptalis*	1	0	0	9	0	(Split)
Scirpophaga	*imparellus*	**10**	0	0	0	0	MATCH
Scirpophaga	*incertulas*	8	0	0	2	0	
Scirpophaga	*innotata*	0	0	5	3	2	
Scirpophaga	*nivella*	0	0	7	3	0	
Scirpophaga	*percna*	8	0	0	2	0	
Scirpophaga	*praelata*	**10**	0	0	0	0	MATCH
Sesamia	*calamistis*	**12**	0	0	0	0	MATCH
Sesamia	*cretica*	8	0	0	4	0	
Sesamia	***grisescens***	10	0	0	2	0	
Sesamia	***inferens***	0	0	0	**12**	0	SPLIT
Sesamia	*nonagrioides*	**12**	0	0	0	0	MATCH

Towards a global DNA barcode reference library for quarantine identifications of lepidopteran stemborers, with an emphasis on sugarcane pests

Timothy R. C. Lee, Stacey J. Anderson, Lucy T. T. Tran-Nguyen, Nader Sallam, Bruno P. Le Ru, Desmond Conlong, Kevin Powell, Andrew Ward, Andrew Mitchell

Supplementary Table 2 - Specimens found to be misidentified based on their position in our phylogenetic analyses. These specimen identifications have now been corrected on BOLD.

BOLD Process ID	Original ID	Revised ID	Source
**GBGL18425-15**	*Chilo suppressalis*	*Sesamia inferens*	BOLD, originally from GenBank
**GBGL12188-13**	*Chilo auricilius*	*Sesamia inferens*	BOLD, originally from GenBank
AGIRI003-17	*Chilo auricilius*	*Sesamia inferens*	BOLD
AGIMP003-12	*Chilo auricilius*	*Sesamia inferens*	BOLD
**MAMOT655-10**	*Chilo infuscatellus*	*Emmalocera* sp.	BOLD
**MAIMB232-09**	*Chilo infuscatellus*	*Emmalocera* sp.	BOLD
**MAMOT648-10**	*Chilo infuscatellus*	*Emmalocera* sp.	BOLD
**MAIMB231-09**	*Chilo infuscatellus*	*Emmalocera* sp.	BOLD
**MAMOT576-10**	*Chilo infuscatellus*	*Emmalocera* sp.	BOLD
**GBGL18424-15**	*Chilo suppressalis*	*Chilo*	BOLD, originally from GenBank
**GBGL18410-15**	*Chilo suppressalis*	*Chilo*	BOLD, originally from GenBank
**GBGL18411-15**	*Chilo suppressalis*	*Chilo*	BOLD, originally from GenBank
**GBGL12189-13**	*Scirpophaga excerptalis*	*Chilo sacchariphagus*	BOLD, originally from GenBank
AGIMP002-12	*Scirpophaga excerptalis*	*Chilo sacchariphagus*	BOLD
AGIRI002-17	*Scirpophaga excerptalis*	*Chilo sacchariphagus*	BOLD
**LEPIN013-12**	*Chilo suppressalis*	*Chilo partellus*	BOLD
**BIPR003-13**	*Chilo suppressalis*	*Chilo auricilius*	BOLD
**BIPR004-13**	*Chilo suppressalis*	*Chilo auricilius*	BOLD
**LEPIN045-13**	*Chilo sacchariphagus*	*Chilo auricilius*	BOLD
**AGIRI005-17**	*Chilo sacchariphagus*	*Chilo auricilius*	BOLD
GBGL12186-13	*Chilo sacchariphagus*	*Chilo auricilius*	BOLD, originally from GenBank
AGIMP005-12	*Chilo sacchariphagus*	*Chilo auricilius*	BOLD
**LOQB548-05**	*Chilo polychrysus*	*Chilo*	BOLD
**LOQB529-05**	*Chilo polychrysus*	*Chilo*	BOLD
